# A prototypic small molecule database for bronchoalveolar lavage-based metabolomics

**DOI:** 10.1038/sdata.2018.60

**Published:** 2018-04-17

**Authors:** Scott Walmsley, Charmion Cruickshank-Quinn, Kevin Quinn, Xing Zhang, Irina Petrache, Russell P. Bowler, Richard Reisdorph, Nichole Reisdorph

**Affiliations:** 1 Skaggs School of Pharmacy and Pharmaceutical Sciences, University of Colorado Anschutz Medical Campus, Aurora, CO 80045, USA; 2 Computational Bioscience Program, University of Colorado Anschutz Medical Campus, Aurora, CO 80045, USA; 3 Department of Medicine, National Jewish Health, Denver, CO, 80206, USA

**Keywords:** Mass spectrometry, Data processing, Diagnostic markers, Metabolomics

## Abstract

The analysis of bronchoalveolar lavage fluid (BALF) using mass spectrometry-based metabolomics can provide insight into lung diseases, such as asthma. However, the important step of compound identification is hindered by the lack of a small molecule database that is specific for BALF. Here we describe prototypic, small molecule databases derived from human BALF samples (*n*=117). Human BALF was extracted into lipid and aqueous fractions and analyzed using liquid chromatography mass spectrometry. Following filtering to reduce contaminants and artifacts, the resulting BALF databases (BALF-DBs) contain 11,736 lipid and 658 aqueous compounds. Over 10% of these were found in 100% of samples. Testing the BALF-DBs using nested test sets produced a 99% match rate for lipids and 47% match rate for aqueous molecules. Searching an independent dataset resulted in 45% matching to the lipid BALF-DB compared to<25% when general databases are searched. The BALF-DBs are available for download from MetaboLights. Overall, the BALF-DBs can reduce false positives and improve confidence in compound identification compared to when general databases are used.

## Background and summary

Bronchoalveolar lavage fluid (BALF) can be a valuable biospecimen for the diagnoses and study of lung diseases, including chronic obstructive pulmonary disease (COPD) and asthma. Previous studies have utilized BALF for the purpose of understanding various disease states including asthma pathogenesis^1^, exposure to air pollution^2^, acute toxicity caused by nanoparticle exposure^3^, inflammatory response to cystic fibrosis^4^, and acute respiratory distress syndrome^5^. The use of BALF is often preferable to proximal biofluids, such as plasma, since BALF comprises compounds closest to the site of stress and/or injury in the lung. In diseases such as COPD, understanding how lung-related small molecules may be altered during injury and inflammation can reveal mechanisms of disease^6,7^. Mass spectrometry (MS) based metabolomics is one tool used to measure these compounds and thereby provide biomarkers for improved diagnosis and treatment and to understand disease mechanisms.

A typical untargeted, MS-based clinical metabolomics experiment produces a highly complex dataset^8^. The workflow includes sample preparation whereby small compounds are extracted from biological matrices, followed by liquid chromatography mass spectrometry (LC/MS), where quantitative information on hundreds to thousands of small compounds is acquired. Raw data is often processed using several steps as follows: extraction of unique molecular ‘features’ from raw MS data, collapsing of adducts and dimers to generate compounds, imputation to account for missing values, normalization, differential analysis, and compound annotation. Compound annotation is usually accomplished using mass and isotope ratios to search publicly available metabolomics databases, such as Metlin and/or Human Metabolome Database (HMDB). Annotation assigns names to the compounds; however, confirmation of annotation results, i.e., identification of compounds, is only accomplished following additional steps. This may include tandem mass spectrometry (LC.MS/MS) and/or comparison to authentic standards. Unfortunately, compound identification is challenging for several reasons. For example, databases are generally agnostic to the sample type being studied and can therefore include thousands of irrelevant compounds, several of which can match to the same exact mass. A comprehensive database of compounds that have been specifically observed in a specific sample type, such as BALF, can better streamline compound identification.

This concept is beginning to be realized through efforts at the Human Metabolome Database (HMDB) which supports some biofluid-specific databases, such as for serum^9^, cerebral spinal fluid^10^, and urine^11^. These are largely, or in some cases entirely, populated using platforms other than unbiased LC/MS. For example, NMR, GC/MS, and targeted LC/MS/MS data make up 100% of the HMDB’s human serum metabolome database^9^. This renders it only mildly useful for LC/MS-based metabolomics profiling studies. Others, such as the newly released ‘BinVestigate’ and creDBle^12^ focus on GC/MS and *E. coli*, respectively. Similarly, improved compound identification is being addressed through the Model Organism Metabolomes (MOM) task group of the Metabolomics Society^13^ and through efforts towards a tissue-specific *Drosophila melanogaster* Atlas^14^. While each of these constitutes a step forward in improving confidence in compound identification, to our knowledge, a small molecule database that is specific for use in unbiased LC/MS-based BALF metabolomics studies will be a first-of-its-kind.

The confidence with which a compound is annotated or identified can be described using the following levels, based on guidelines proposed by Sumner *et al*^15^: 1) Highest Confidence- matching to authentic standards confirms structure, 2) High Confidence- putatively identified using MS/MS or other orthogonal technique, 3) Medium Confidence- tentatively identified using mass and isotope ratios against MS database, 4) Low Confidence- tentatively identified using neutral mass without isotope ratios, and 5) unidentified molecular feature^15^. Level 2 (High Confidence) requires MS/MS to generate fragmentation data, which can be matched to MS/MS databases. Other orthogonal techniques that can be used to obtain Level 2 confidence include collisional cross section (CCS) values obtained from ion mobility mass spectrometers^16,17^, optical spectroscopy, and nuclear magnetic resonance (NMR)^18^. These efforts can be cost- and time-prohibitive; therefore, many metabolomics experiments only report Level 2-5 results.

While sample-type specific databases do not completely address this issue, combining the reproducible molecular ‘signatures’ of specific sample types, such as BALF, into a database lends additional confidence to Level 2-5 annotations. Because it is of limited size compared to a general DB, a BALF-DB can be more easily manually curated; for example, MS/MS, CCS values, and descriptors can be added at any time. Finally, any investigators working with BALF can add data/annotations, thereby expanding the DB across disease states. Overall, the assembly of a BALF-specific database has potential to greatly impact the field of lung metabolomics.

To develop a BALF-specific database it is necessary to filter and/or rank results such that only reproducibly detected compounds are included. This dramatically reduces false positives that result from searching non-specific databases. To achieve this, good sample collection and reproducible chromatography and mass spectrometry are required for database building. In addition, database development must include a series of filtering steps to reduce or eliminate entries that result from contaminants, fragmentation, and/or feature extraction artifacts. Ideally, a functional database must be flexible enough to incorporate new compounds as they are detected. Our prototypic BALF-DB includes all of these features and represents a first-step towards a comprehensive, manually curated database that has potential to provide a high level of confidence in compound identification.

**Figure 1:**
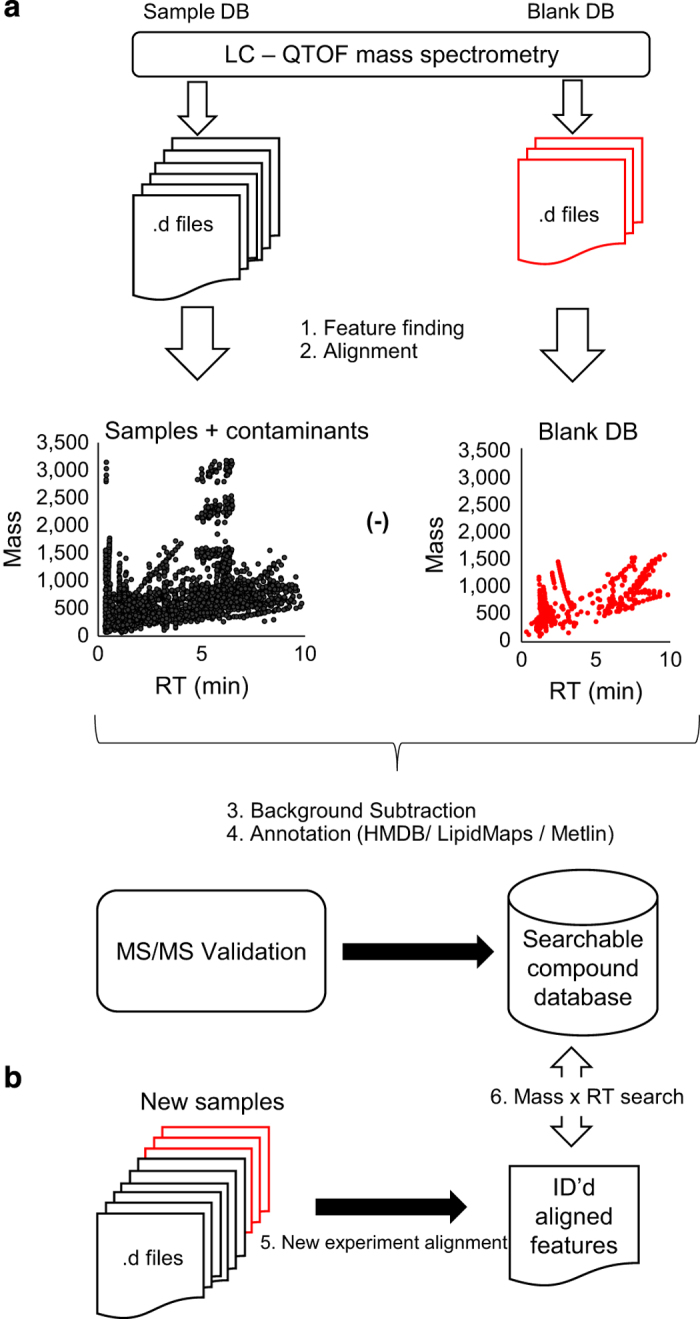
Workflow for database assembly. **(a)** The general workflow for the assembly of searchable compound databases uses raw mass spectrometry files as input (.d files). Data files from instrument /sample blank files were processed separately and compounds in these files were subtracted from the database. Mass spectrometry files (.d) from BALF samples and from blank samples were extracted to generate compounds and data aligned using Profinder. Plots represent typical plots of compounds identified within the samples (black) and blanks (red). After background subtraction, compounds are putatively identified using the HMDB, LipidMaps, and Metlin databases. Tandem mass spectrometry was performed and NIST MS/MS database searched to improve confidence in identifications. **(b)** Datasets from new experiments are searched using mass and retention time. Compounds that were not previously found in the databases can be added. Frequency and reproducibility can be re-assessed after the addition of new datasets.

In this work, we describe the assembly of a prototypic, human BALF-specific small molecule database. Data for the BALF-DB was acquired using consistent sample collection techniques and reproducible LC/MS on 117 human BALF samples. Data processing followed a typical workflow and included additional steps for database assembly (Fig. 1). The usage section demonstrates the BALF-DB’s utility as a searchable database using data from three sources: 1) a subset of the 117 BALF samples (so-called ‘internal’ validation), 2) from an independent experiment of human BALF samples (so-called ‘external’ validation), and 3) from an independent experiments of mouse BALF samples. All data, including raw data and the BALF-DB, is available from the Metabolights metabolomics repository (Data Citation 1). The BALF-DB is also currently available as a downloadable spreadsheets in Microsoft Excel format from MetaboLights (Data Citation 1) as ‘BALFDB_AQUEOUS.xlsx’ and ‘BALFDB_LIPID.xlsx’. In conclusion, we provide a searchable and expandable human BALF small molecule database that can be used to improve confidence in compound identification.

## Methods

### Ethics statement

All methods were performed in accordance with the relevant guidelines and regulations. ‘Subpopulations and intermediate outcome measures in COPD study’ (SPIROMICS) was approved by the institutional review board at each participating center; all subjects provided written informed consent. The SPIROMICS analysis was approved by the National Jewish Health Institutional Review Board (HS-2678) and ClinicalTrials.gov Identifier: NCT01969344. Animal studies were approved by the Animal Care and Use Committee of Indiana University. Human subjects were from the Genetic Epidemiology of COPD (COPDGene) cohort, which is a National Institutes of Health–sponsored multicenter study of the genetic epidemiology of COPD (ref. 19). COPDGene was approved by the institutional review board at each participating center; all subjects were enrolled from January 2008 to April 2011 and provided written informed consent. COPDgene ClinicalTrials.gov Identifier: NCT02445183.

### Sample collection

BALF was collected from subjects from the SPIROMICS cohort (Table 1, *n*=117) as previously described^20^ for use in database development. A table of sample identifiers are described in ‘s_Study id.txt’ (Data Citation 1). BALF used in the external validation test study was collected from subjects (*n*=5) from the COPDGene cohort as previously described and *n*=10 mice as previously described^21^. All samples were frozen at -80 °C prior to sample preparation and analysis.

### BALF sample preparation

For the BALF-DB development, 140 μL of BALF from the SPIROMICS cohort were prepared using protein precipitation with 560 uL of methanol to pellet proteins and extract the metabolites in the supernatant^22,23^. The supernatant was split into two aliquots: 165 μL for HILIC analysis (hydrophilic/aqueous fraction) and 495 μL for C18 analysis (hydrophobic/lipid fraction). The aqueous fraction was dried down in a speedvac at 45 °C and reconstituted in 30 μL of 95:5 water:acetonitrile. The lipid fraction was reconstituted in 90 μL of methanol.

For the BALF-DB external test study using mouse and COPDgene BALF samples, preparation has been previously described^21^. Blank samples were prepared using water as previously described^21^.

### Liquid chromatography

For all samples, including blanks, the BALF lipid fraction was resolved using the same C18 reverse phase chromatography. The separations used an Agilent Zorbax Rapid Resolution HD (RRHD) SB-C18, 1.8 micron (2.1 x 100 mm) analytical column with an Agilent Zorbax SB-C18, 1.8 micron (2.1 x 5 mm) guard column and used an Agilent 1290 series high performance liquid chromatography (HPLC) pump with an 8 μL injection volume. Due to dilution differences, the external study used 4 μL for mouse BALF and 15 μL for human BALF. Flow rate was 0.7 mL/min using the following mobile phases: mobile phase A was water with 0.1% formic acid, and mobile phase B was 60:36:4 isopropyl alcohol:acetonitrile:water with 0.1% formic acid. Gradients were 0-0.5 min 30-70% B, 0.5-7.42 min 70-100% B, 7.42-10.4 min 100% B, 10.4-10.5 min 100-30% B, 10.5-15.1 min 30% B. The auto sampler tray temperature was set to 4 °C and column temperature was 60 °C.

For all samples, analysis of the aqueous fraction was by HILIC chromatography using an Agilent 1290 series pump with a Phenomenex Kinetex HILIC, 2.6 μm, 100 Å, 2.1×50 mm analytical column and an Agilent Zorbax Eclipse Plus-C8 Narrow Bore Guard Column, 2.1×12.5 mm, 5 micron guard column. Sample injection volume was 1 μL. A flow rate of 0.6 ml/min was used with the following mobile phases: mobile phase A was 50% ACN with pH 5.8 ammonium acetate, and mobile phase B was 90% ACN with pH 5.8 ammonium acetate. Gradient elution was as follows: 0-2 min 100% B, 2-2.1 min 100-90% B, 2.1-8.6 min 90-50% B, 8.6-8.7 min 50-0% B, 8.7-14.7 0% B, 14.7-14.8 min 0-100% B, 14.8-24.8 min 100%B. The auto sampler tray temperature was set to 4 °C and column temperature was 20 °C.

### Mass spectrometry

Data acquisition for the lipid fraction used the following methods: For the primary experiment, data were acquired on an Agilent 6545 Time-of-Flight (TOF-MS) with ESI source in positive ionization mode using a scan rate 2.00 spectra/second, mass range 50-1700 m/z, gas temperature 300 ^°^C, gas flow 12.0 L/min, nebulizer 35 psi, skimmer 65 V, capillary voltage 3500 V, fragmentor 120 V, and calibrated with reference m/z’s 121.050873 (Purine) and 922.009798 (Hexakis(1H, 1H, 3H-Tetrafluoropropoxy)Phosphazene). For the external validation test, data were acquired on an Agilent 6210 Time-of-Flight (TOF-MS) with dual ESI source using a scan rate 2.03 (positive mode) spectra/second, mass range 60-1600 m/z, gas temperature 300 ^°^C, gas flow 12.0 L/min, nebulizer 30 psi, skimmer 60 V, capillary voltage 4000 V, fragmentor 120 V, and calibrated with reference m/z’s 121.050873 (Purine) and 922.009798 (Hexakis(1H, 1H, 3H-Tetrafluoropropoxy)Phosphazene).

The aqueous fraction MS conditions were as follows: Agilent 6520 Quadrupole Time-of-Flight (Q-TOF-MS) in positive ionization mode with ESI source, mass range 50-1700 m/z, scan rate 2.00 spectra/second, gas temperature 325 ^°^C, gas flow 12.0 L/min, nebulizer 30 psi, skimmer 60 V, capillary voltage 4000 V, fragmentor 120 V, and calibrated using the reference masses 121.050873 (Purine) and 922.009798 009798 (Hexakis (1H, 1H, 3H-Tetrafluoropropoxy) Phosphazene).

### Quality control (QC)

For BALF-DB development samples from the SPIROMICS cohort, total ion chromatograms (TIC) and extracted ion chromatograms (EIC) for spiked internal standards were evaluated for retention time reproducibility. The largest inter-day retention time variation (5 run days) was 0.45% and 2.02% for the spiked standards in the lipid fraction and aqueous fraction, respectively, corresponding to a 0.067 min and 0.285 min retention time drift. For the external validation test samples, the largest retention time variation (1 run day) was 0.58% and 1.54% (corresponding to<0.05 min retention time drift) for the spiked standards and selected endogenous compounds, respectively. Signal intensities of the TICs were also evaluated for both test and development studies. The largest variation was less than 10% coefficient of variance (CV) in the most abundant range of the TIC. HPLC pressure curve variation was less than 5% CV. Instrument QC samples were injected after every ten samples and analyzed to ensure that peak areas of 9 spiked internal standards were reproducible (<15% CV) throughout the analysis. For BALF-DB development samples, the %CVs for the standards were all<10% for the aqueous fraction and <5% for the lipid fraction; this was also true for the external validation test study.

### Data processing workflow

All data were prepared using a workflow for data processing and compound annotation (Fig. 1a). As described further below, these steps included data extraction and feature finding, chromatogram alignment, compound annotation, and formatting of results. A total of 2 datasets were processed for each sample set (i.e., SPIROMICS, COPDgene, mouse): Lipid positive mode ESI (Lipid), and aqueous positive mode ESI (Aqueous). Contaminant compounds from ‘blank runs’ are removed in a separate step prior to database assembly.

### Data pre-processing and alignment

Data were visually inspected using the MassHunter Qualitative Analysis software suite to determine relative deviation of peak retention times (<0.3 min) and the intensity of noise peaks (~600 counts). Using this information as parameters for the feature finding step, putative compounds denoted as ‘features’, were mined from the raw data and then aligned between samples. A feature was only included if it was observed in a minimum of 2 samples. Data files from ‘blank’ samples were included in this analysis to aid subtraction of contaminant compounds from the final assembled database. To lessen the likelihood of missed peaks during feature extraction^24^, data was processed using the Profinder Software v.8.0.888 (Agilent Technologies) with the following parameters: Batch Recursive Feature Finding, positive mode, single charge, with +H, +Na, +K, and +NH_4_ adducts in positive ionization mode and single charge, with -H, +Cl, +HCOO, +CH_3_COO adducts for negative ionization mode, and absolute height filter of >600. Charge states were limited to ≤2. The resultant dataset comprises multiple adducts/ion species that have been merged into single compounds. Data were aligned during feature finding with Profinder (using 15 ppm mass error and 0.3 min retention time window) and then imported into Mass Profiler Professional (v14.8) for annotation. Compound exchange format (.cef) files were exported and then used for further evaluation. Additionally, exported data tables of detected compound intensities are included in ‘BALFDB_AQUEOUS.xlsx’ and ‘BALFDB_LIPID.xlsx’, within sheet #3 of the spreadsheet documents (Data Citation 1).

### Compound annotation

Molecular formula generation used mass and isotope peaks and included the following elements: C, H, N, O, S, and P and was completed using IDBrowser. Compounds were annotated using formulas to search against the Human Metabolome Database v.3.6 (HMDB)^25^, Lipid Maps and Metlin^26–28^. HMDB was utilized first since the samples were derived from human; we also expected mouse and human metabolites to be highly overlapping. For database searches, an error window of <10 ppm was used and a maximum of 2 charge states were allowed. Additional higher level annotation of compounds was performed by MS/MS using a non-targeted data acquisition approach^29^ followed by a search against the established Lipid Blast and NIST17 MS/MS spectral libraries^30^. Tandem MS improved confidence in several identifications whereby 326 MS/MS spectra produced matches for 235 compounds for the Aqueous experiment and 597 MS/MS spectra produced matches for 236 compounds for the Lipid experiment. Plots of each validated MS/MS spectra are provided in Supplementary File 1 and Supplementary File 2 (Aqueous and Lipid MS/MS spectra, respectively).

### Subtraction of contaminate and background compounds

Following data processing and annotation, the resulting table of intensities was aligned by mass and retention time. This was accomplished for the 2 datasets used to develop the BALF-DB (Lipid and Aqueous fractions of the SPIROMICS samples) and for the external validation test study (COPDgene and mouse samples). In addition, data from the blank samples was used to determine contaminant compounds. Contaminant compounds were subsequently subtracted from the table of intensities for each of the datasets. These aligned, contaminant subtracted lists of compounds representing the BALF-DB and the external validation test study were subsequently used as described in the usage section.

In addition, a dataset (Lipid) was evaluated to determine what percent of data could be accounted for by in-source fragmentation. Following use of an ‘all ions’ experiment to identify possible fragment ions, the Lipid DB was compared to the resulting ‘fragment’ database. Only a small percentage of DB entries, 0.5% (59 compounds), were found to possibly produce a fragmentation and 1.1% (125 compounds) of the DB entries possibly could have arisen from a fragmentation. As this percentage was small and requires additional validation, these DB entries were retained in the Lipid DB and flagged as potential fragments.

### Assembly of BALF database

A custom R (v.3.3) package was used to read the Agilent compound exchange format (.cef) files containing the following values: adduct information, individual adduct *m/z*’s and intensities, compound retention times, and compound masses. The script compiled information using the provided.cef files containing each compound’s contributing adducts from each sample. Compound statistics, including adduct information, were then computed during this process in an automated fashion. An additional.cef file containing the annotation information (formula, compound name, etc) and the averaged values across all the samples was also read. These values were then exported to comma delimited tables. The intensity values were then removed from the tables to produce an annotated mass and retention time database and are in ‘BALFDB_AQUEOUS.xlsx’ and ‘BALFDB_LIPID.xlsx’, within sheet # 2 of each spreadsheet document (Data Citation 1). The entire dataset as described in the Data Records section, is available from MetaboLights (Data Citation 1)^31^ in compliance with reporting guidelines for metabolomics data and provided in ISA-Tab format. Note that the process of annotation results in only the top database match being listed as a final compound name. Therefore, for each compound with either an HMDB or Lipid Maps accession number, both the HMDB and Lipid Maps databases were re-searched to determine if additional compounds could be found with the same molecular formula. Any additional compounds matching to the same molecular formula were added to the database for these entries in separate spreadsheet columns, but in the same row for the compound. This lets the user see other potential matches for these compounds. To create a searchable database, the compound name, molecular formula, retention time, and accession number(s) were written in comma delimited format using Microsoft Excel. HMDB main, sub and super chemical classes were added to the database entries, thereby linking that information to the compounds. Additionally, main lipid categories from Lipid Maps were added to the compound entries.

### Analysis of fragment ions using ‘all ions’ analysis

To determine what DB entries might result from in-source fragmentation, an ‘all ions’ experiment was conducted using the lipid fraction of BAL. For this purpose, a pooled lipid BAL sample was analyzed on an Agilent 6545 Q-TOF. Analysis was performed in MS1 mode using 4 energy channels; the applied collision energy values were 0 V, 10 V, 20 V, and 40 V for channels 1-4 respectively. Data were analyzed in Mass Hunter Qualitative Workflows software using the Find by Formula algorithm with fragment confirmation. Features were first extracted using the Find by Molecular Feature algorithm followed by molecular formula generation for 1,900 of the 2,380 extracted compounds. A database was created which contained the molecular formula, mass, and retention time for each compound. For compound extraction mass and retention time tolerance were limited to +/‒ 10 ppm and +/‒ 0.15 min. For fragment confirmation the minimum number of qualified fragments was set to 1, signal to noise was set to>5.0, and the co-elution score was set to>90. This ‘fragment’ database was then used as a source for fragment confirmation using a Find by Formula algorithm in Mass Hunter.

When this database was compared to the prototypic BAL Lipid-DB, only a small percentage of DB entries, 0.5% (59 compounds), were found to possibly produce a fragmentation and 1.1% (125 compounds) of the DB entries possibly could have arisen from a fragmentation. As this percentage was small and requires additional validation, these DB entries were retained in the Lipid DB and flagged as potential fragments. Future versions of the BAL-DBs can include more extensive evaluation of fragments using additional strategies.

### Code availability

Custom code used for the processing of chemical exchange format files are available for download from a GitHub repository at: https://github.com/swalmsle89/CefTell. Included are code written in the R language for obtaining the ion statistics, computing the isotope ratios from the HMDB and LipidMaps databases and filtering of compounds. Also included are code for searching and processing MS/MS search results, storing the results in a SQL database, and plotting the MS/MS results to pdf files. All code is available under the Apache 2.0 license.

## Data Records

All data from this experiment are in MetaboLights (Data Citation 1). The BALF small molecule database and the search results as described in the usage section are available as two Microsoft Excel spreadsheets: ‘BALFDB_AQUEOUS.xlsx’ and ‘BALFDB_LIPID.xlsx’. These files each contain each compound’s observed retention time and neutral mass, compound annotations, and identifiers from the HMDB or Lipid Maps online repositories. MSI levels of annotation are also included. The BALF databases are each separated into 3 spreadsheet tabs, the 1^st^ containing the database legend, the second for the BALFDB entries, and the 3rd containing the raw detected intensities for each sample.

Raw mass spectrometry data in Agilent.d format from this experiment were also deposited at MetaboLights. These raw data files contain the unprocessed data as produced from the mass spectrometers.

Other ISA-Tab compliant data include sample metadata (‘s_Study id.txt’), information about chromatography (Lipid: ‘a_study_id_metabolite_profiling_mass_spectrometry.txt’ and Aqueous: ‘a_study_id_metabolite_profiling_mass_spectrometry-1.txt’), and metabolite annotations ( Lipid: ‘m_study_id_metabolite_profiling_mass_spectrometry_v2_maf.txt’ and Aqueous: ‘m_study_id_metabolite_profiling_mass_spectrometry_v2_maf-1.txt’).

## Technical Validation

### Composition of the 2 BALF databases

Overall, 2 BALF-DBs of retention time-aligned compounds were assembled from the lipid positive and aqueous positive ionization (see ‘BALFDB_Aqueous.xlsx’ and BALFDB_Lipid.xlsx’) mode SPIROMICS datasets (Data Citation 1). For each of these databases, the total number of BALF-derived and contaminant compounds were computed. For the lipid DB, there were 12559 compounds, of which 246 were determined to be contaminants and after removal there were 12313 compounds. For the aqueous DB, there were 1070 compounds, of which 127 were determined to be contaminants and after removal there were 943 compounds. Contaminant compounds may include compounds derived from plastics used in the sample collection or processing along with other solvents or detergents common to the sample processing workflow^32^. These were subsequently removed from the final BALF databases. After later removal of artifacts arising from data extraction errors, the final lipid DB contained 11737 compounds and the aqueous DB contained 658 compounds.

### Additional DB features to improve confidence: frequency and reproducibility

BALF samples may have inherently high variability due to normal biological variation, disease states, exposures such as cigarette smoke and allergens, and differences in sampling. This may result in false negatives due to compounds only being present in a few samples. To address this, our BALF-DB includes two unique metrics compared to general metabolomics databases: measurements of frequency and reproducibility. For this, frequency is defined as repeated observations of a compound across multiple samples; this measurement is based solely on the appearance of a compound in a sample (yes/no) and is irrespective of compound intensity. Therefore, the frequency for any given compound will increase as it is detected in more samples. Reproducibility describes the repeatability with which a compound’s intensity was detected by the mass spectrometer; this metric is essentially frequency measurements that include %CV. Reproducibility encompasses frequency by its very nature (i.e., a compound has to be observed to be quantitated); however, frequency does not include reproducibility metrics. While we expect reproducibility to vary due to normal biological variation, this metric is useful for evaluating database statistics. Each of these measurements is discussed in the context of the BALF-DB.

### Frequency of observed compounds

**Figure 2:**
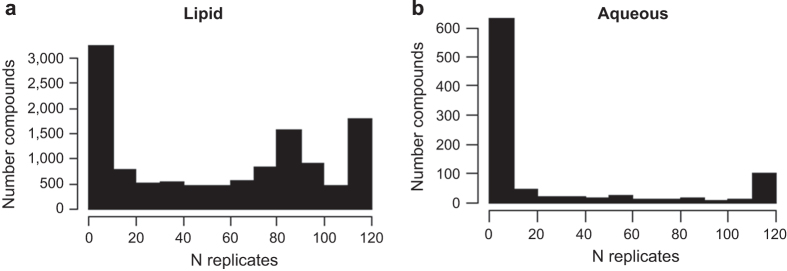
Total compounds observed as a function of the number of replicate samples that the compounds were observed in. The x axis represents the number of mass spectrometry replicates (N) and the y axis represents the number of compounds observed in N replicates. Frequency of observed compounds were generally uniformly observed with the most observed frequency of compounds produced in the largest sample number (N). **(a,b)** Total compounds observed for the Lipid experiment. **(c,d)** Total compounds observed for the Aqueous experiment.

For the Lipid database, there was a high frequency of compounds observed in larger numbers of samples and replicates, as expected (Fig. 2a). This indicated that these compounds could be detected across many samples. This also verified that the data processing steps which aligned the compounds by retention time and neutral mass was successful. A prominent ‘hump’ can be observed at *n*=80 samples in the Lipid DB. This appears to be related to the smoking status of the subjects, with subjects who were current smokers having a higher number of compounds present in their BALF compared to non-smokers or former smokers. This is consistent with our previous findings^21^.

For the aqueous database, the trend was different (Fig. 2b); the data were sparser and far more unique compounds were observed in individual samples. We expect this is due to more natural variation in the range of water soluble compounds to which an individual is exposed; for example, due to inhalation of allergens, pollutants, and even food particles. Additionally, aqueous small compounds are typically of lower concentration and are therefore more subject to feature extraction errors. These discrepancies will likely be alleviated as additional datasets are added to the BALF aqueous DB in future experiments.

### Reproducibility of compounds

**Figure 3:**
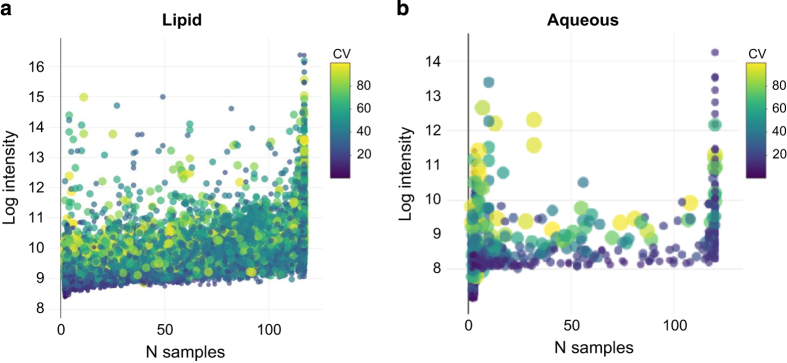
Effect of compound intensity and replicate size on reproducibility. Each compound’s intensity vs. the number of replicates the compound was observed in was plotted and color coded according to the computed percent coefficient of variation (%CV). Each compound is plotted as a point of varying color and size. The x axis indicates the total N replicates that the compound was observed in and the y axis indicates the log intensity. The dots in the scatterplots are color coded according to the color bar on the right of each plot and represent the total %CV. Darker and smaller points indicate compounds with a lower observed %CV. More frequently observed compounds tended to produce higher ranges of intensities for the detected compounds in the Lipid experiment. (**a**) Lipid experiment, (**b**) Aqueous experiment.

Reproducibility was used to determine how the variability of a compound’s abundance affected how often that compound was observed (Fig. 3). This was expressed by plotting trends that indicate the number of replicates in which each compound was observed vs. the log intensity of the compound. While %CV was computed on non-log adjusted data, data were plotted as log transformed to better visually separate the compound intensities. From these plots, the effect on compound intensity was used to ascertain the reproducibility of the compound’s intensity as %CV.

For the Lipid DB (Fig. 3a), there was a slight dependence of the observed frequency of a compound on the compound’s intensity. For those compounds observed in the highest number of replicates, the range of observed intensities was the greatest. This is likely explained by biological variance. Overall, lower abundant compounds are more likely to be missed during feature extraction, thus lower abundant compounds are likely to be less frequently observed. The Aqueous DB reproduced this trend with less distinction (Fig. 3b). Additionally, the Aqueous DB contains a wide range of highly variable compounds of low frequency. This may be explained by the variable extraction efficiency of the soluble compounds together with the biological variance. Nonetheless, the most observed compounds produced lower overall %CV for a higher range of intensity values.

The %CV of the compounds’ intensities across samples was indicated by the size and color of the points in the plots in Fig. 3. For this plot, several compounds with %CV>100 were removed to best display the remaining compounds. The reported %CVs were randomly distributed indicating that the reproducibly detected compounds do not necessarily require high abundances; however, compounds observed with a higher frequency were generally produced from a higher range of intensities for the Lipid DB. As mentioned earlier, the varied %CVs of the Aqueous DB were likely due to biological variance and less reliable feature extraction for lower abundant compounds.

### Annotation of adducts

**Figure 4:**
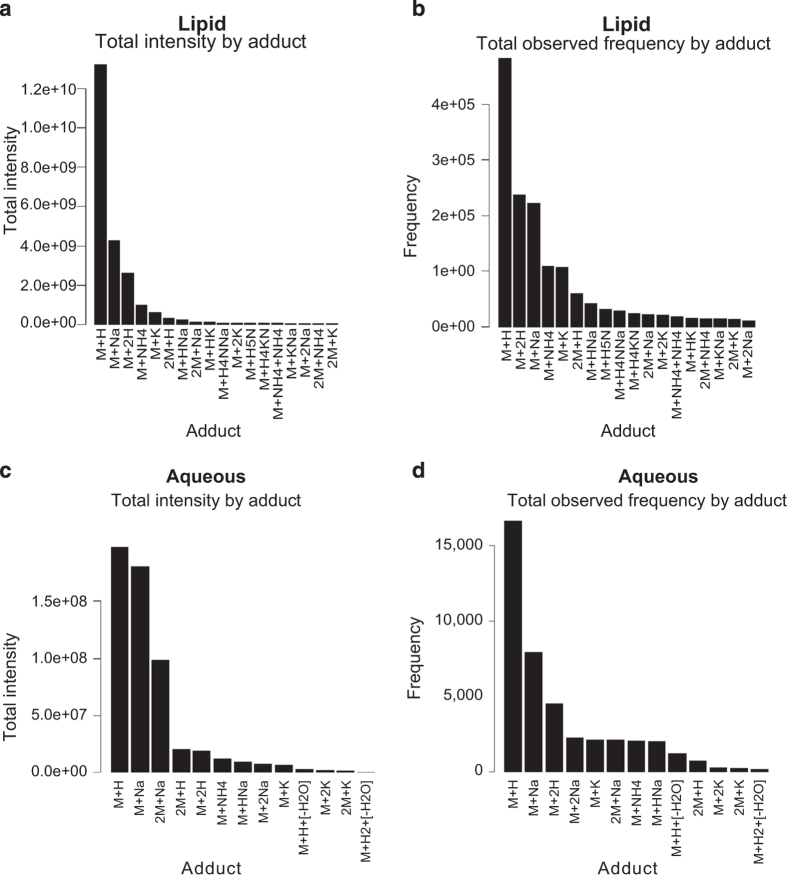
Bar charts of mean observed intensity and max observed frequency by adduct. The amount and total intensity contributed by each adduct indicated how much abundance was detected per adduct and used to ensure the expected predominant species (M+H, M+Na, M+2H) accounted for most of each compound’s detected signal. (**a**,**c**) The mean intensity across all samples was observed for each observable adduct per compound for the Lipid and Aqueous experiments. The y axis indicates the mean intensity observed per sample. The x axis indicates the observed adduct. (**b**,**d**) The total observed adduct count across all samples for the Lipid and Aqueous experiments. The y axis indicates the total observed count by adduct.

Sample specific DBs offer additional advantages over traditional DBs in part because they use empirically-derived data; this enables users to generate DB statistics. An additional advantage of the custom R script utilized here is that individual adducts observed for each compound can be evaluated for their reproducibility of detection when building the BALF-DB. The total mean intensity and frequencies of the contributing adducts were evaluated to determine the amount that each adduct type contributed to the experiment (Fig. 4). In both databases, the M+H adduct was the primary contributing adduct to total abundance and most frequently observed in the databases. The M+2H and M+Na adducts were the next dominant adducts and were typical of compounds observed in metabolomics due to common observed charge state (z=1 or 2) and sample processing (Na+). Other less prevalent adducts include ammoniated (M+NH4), potassium (M+K), and dimers (2M+H). In total, 18 adduct types were observed for the Lipid DB and 16 for the Aqueous DB. The correct assignment of adducts by the data extraction software aided the reduction of potential compound adducts assigned as different compounds in the databases.

### Filtering compounds using isotopic elemental composition

**Figure 5:**
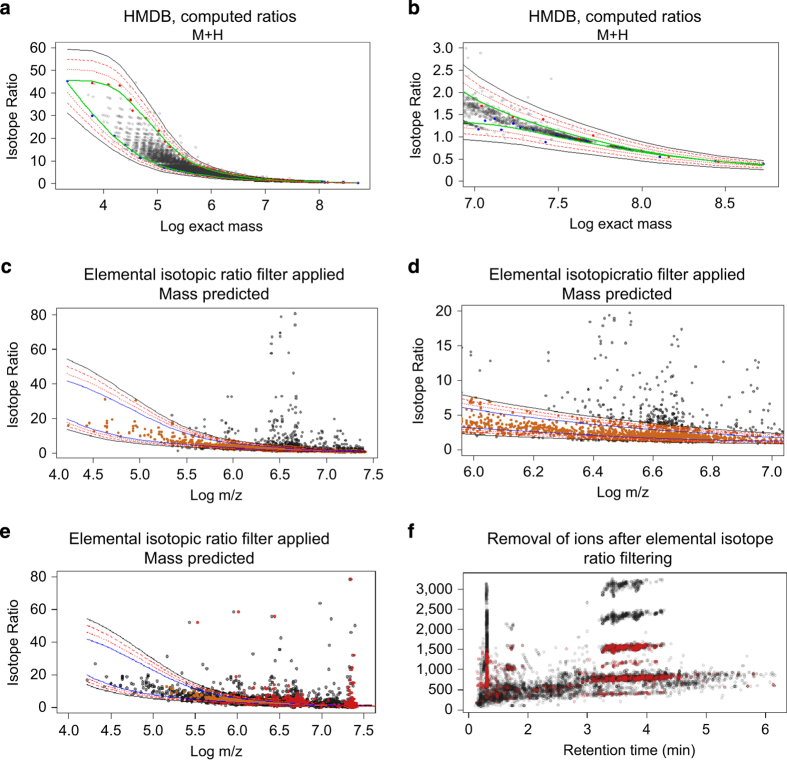
Application of a novel isotope ratio filter to remove database artifacts. Each compound’s detected adducts were scrutinized by comparing their expected range of isotope ratios vs. those theoretically computed using the HMDB and Lipid Maps databases. After developing models depicting the maximum and minimum expected isotope ratios across the range of masses, each detected feature was flagged if found to be outside the range. If flagged outside the range and no other compound-adduct pair existed, the compound was removed from the database. (**a**,**b**) Computed ratios and log mass plotted for unique formulae for the HMDB / LipidMaps databases for [M+H] ions. Green lines indicate the fitted model curves for the upper and lower ratio limits. Dashed red lines through the black line indicate the 10, 20, and 30% variance lines. (**c**,**d**) Log mass and isotope ratios plotted for a single test sample for [M+H]. Orange dots indicate compounds with ions flagged for removal. (**e**) Log mass and isotope ratios were plotted for the aligned dataset using reported mean values. Red dots indicate M+H ions flagged for removal. (**f**) Mass vs. retention time plot of BAL-DB compounds illustrates the location of M+H ions flagged for removal from the analysis.

Data extraction software has several inherent limitations, including an inability to evaluate isotope ratios as part of the feature extraction step. This can result in false matches to general databases due to extraction of artifacts (noise peaks), mis-assigned monoisotopic peaks, or failure to recognize adducts. To reduce the occurrence of erroneous compound matches in the described BALF-DB, the ratio of each compound’s detected isotope clusters, including adducts, were compared to theoretically computed isotope ratios. To achieve this, a model was developed from the atomic formulae contained in the HMDB and LipidMaps (Fig. 5). Using the 9910 unique molecular formulae present in the these databases, theoretical ratios for extracted ^12^C and ^13^C isotopes were computed using Emass^33^. The models developed for the upper and lower ranges of these ratios were dependent on exact mass (Fig. 5a,b), with higher ^12^C/^13^C ratios expected for compounds with lower masses. Using a single BALF sample for testing purposes, each compound and its corresponding adducts was evaluated (Fig. 5c,d) and observed isotope ratios outside of the ranges were flagged for poor quality and removed from subsequent analysis. The method was then applied to the aligned dataset (Fig. 5e) and produced significant numbers of ‘flagged’ adducts. Fig. 5f simulates what flagging of a compound (red dots) for removal will look like if only the M+H adduct was observed for those compounds. Once flagged, compounds with ratios outside of expected ranges and with no other observable adducts passing the model filter were then removed from the database. Using this criteria, 781 (6% of total) compounds were removed from the Lipid DB and 231 (25% of total) compounds removed from the Aqueous DB.

The final prototypic Lipid DB is comprised of 11,737 compounds and the final prototypic aqueous DB is comprised of 658 compounds.

### Molecular classification

**Figure 6:**
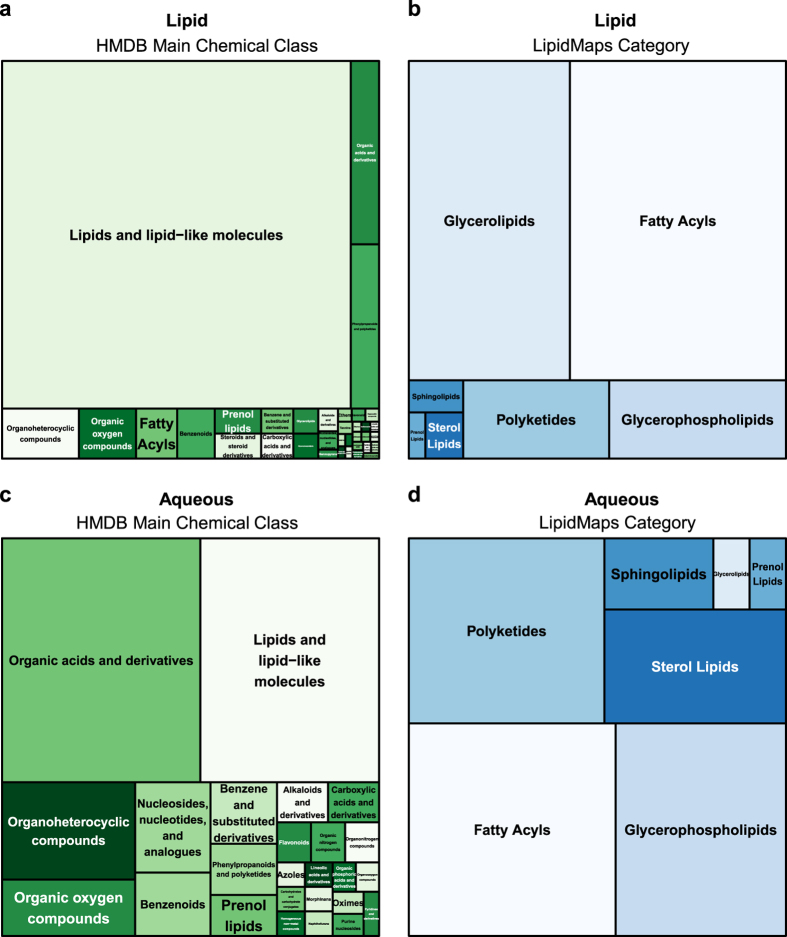
TreeMaps of Human Metabolome Database Chemical Classes and LipidMaps Lipid Categories. Treemaps were plotted to show the relative distribution of main chemical classes for HMDB and Lipid Maps. These relative distributions of molecular identifiers serve as a metric of similarity between bronchoalveolar lavage fluid (BALF) samples and the sample extraction methods (eg lipids vs. aqueous compounds). Text in the plots represent HMDB chemical classes or Lipid Maps categories. Larger squares indicate a larger number of annotated compounds were found in the database. Only compounds with an HMDB accession **(a,c)** or Lipid Maps accession **(b,d)** for the Lipid or Aqueous experiments were mapped to their compound classifiers and the relative quantities of these classifiers were plotted.

To determine the level of consistency in the molecular classes found in the BALF databases, chemical taxonomies were first imported into the DB and then summarized. For this analysis, the HMDB chemical class information and/or Lipid Maps lipid categories were plotted as tree maps (Fig. 6,Table 2, and Table 3).

For this tree map analysis, the main HMDB chemical classes for the Lipid and Aqueous database (Fig. 6a-d) produced differences in the complement of compounds extracted from BALF samples. For example, the general descriptor of *Lipids and lipid-like molecules* comprised the majority of compounds present in the Lipid DB, as expected (Fig. 5a). While *Lipids and lipid-like molecules* are also present in the Aqueous DB, *Organic acids and derivatives*, *Organic oxygen* and *Organoheterocyclic* compounds and other non-lipid categories from the HMDB annotation list were also prevalent (Fig. 5c). The main Lipid Maps lipid categories also produced qualitative differences in the number and type of lipid compounds. For example, the Lipid experiment (Fig. 5b) indicated that *Glycerolipids, Glycerophospholipids, Fatty acyls, Polyketides and Sphingolipid* compounds were the most prevalent lipid classes. In the aqueous experiment (Fig. 5d), however, the *Fatty Acyls, Glycerophospholipids, and Polyketides* had similar representation. This information confirms differences in the molecular classes were concordant with the extraction type (lipid vs. aqueous).

We have presented a sample type specific small molecule database using data acquired from human BALF samples. These BALF samples provide a relatively comprehensive snapshot of compounds likely to be encountered in an experiment utilizing BALF samples. Data were acquired using Lipid and Aqueous fractions following sample preparation. Data were extracted using a common data extraction workflow. Datasets were background subtracted to remove the majority of contaminants from the database. Further, an evaluation of reproducibility demonstrated no dependency of compound abundance on their ability to be identified, with variability (%CV) of the detected compounds appearing random. This suggested that more samples are valuable for detecting additional compounds to add to the BALF repertoire of compounds that might be encountered in the future; this would likely aid future BALF profiling by mass spectrometry if consistency in laboratory protocol were followed. Two novel features were introduced to improve the quality of the database: an isotope ratio validation method and a method to remove in-source fragmentation ions.

## Usage Notes

To demonstrate the utility of the BAL-DBs, we performed an evaluation in two ways: 1) by re-processing and searching a subset of 10 samples used to develop the DBs (internal validation), and 2) searching of samples from separate BALF experiments (external validation), hereafter called ‘Test’ datasets. In both cases, RT and mass aligned compound lists were used for a RT and mass search against the BALF-DB (Table 4). In the Internal Validation Test dataset, 9119 lipid compounds matched the 9197 Lipid DB entries for a 99% match rate. A total of 113 aqueous compounds matched to the 244 Aqueous DB entries for a 47% match rate. When using mass as the sole matching criteria (i.e., no retention time), 9137 lipid and 116 aqueous compounds matched their respective databases. Overall, the Internal Validation Test demonstrated that the small subset (N=10) of samples relative to the overall experiment (N=117) can capture most detected BALF-DB compounds for the Lipid experiment. In the Aqueous experiment, the lower percent matching was likely due to unique and/or low abundant compounds. As indicated earlier, we suspect that this was caused by the inherently variable nature of the BALF.

For the External Test dataset, 10 human and 5 mouse BALF samples from a previously conducted, independent study were used to further demonstrate the utility of the BALF-DB^24^. Sample collection, sample preparation, and LC protocols were the same for the External Test as those described for BALF-DB samples. Data from the External Test were re-extracted and re-processed to be identical to these steps for BALDB development. However, differences were expected due to differences in the specific instrument used. Note that only the Lipid fraction was used for this test.

For the External Test dataset, the total number of detected compounds from the aligned lipid BALF replicates were 2493 (Human) and 2566 (Mouse). This aligned data was then searched using the Lipid BALF-DB using retention time and mass within a 10 ppm mass search and +/- 1.5 min retention time variance. The relatively large retention time window was due to variance in chromatography between the two experiments which were conducted 1 year apart. Searching of the BALF Lipid DB resulted in 874 Human (35%) and 946 Mouse (37%) match rates; this is increased to 45% when only mass is used in the search. This is in contrast to our original results from this dataset, where searching 3164 Human and 2779 Mouse compounds against HMDB resulted in 783 (24.7%) and 624 (22.8%) matches, respectively (Table 5). Match rates were even lower when LipidMaps was searched with 571 (18%) Human and 447 (16.1%) Mouse compounds matching (Table 5). Finally, a total of 83 Human and Mouse compounds had corresponding MS/MS data in the original study compared to 236 when the BALF-DB was used. Together, these results illustrate the improved match rate and improved confidence in compound identifications when the Lipid BALF-DB is used compared to general databases.

These results demonstrate that the complement of compounds identified in the External Test dataset have strong similarity to those in the current BALF-DB experiment. However, several factors contribute to overall lower match rates when an independent, external dataset is used compared to an internal dataset. These include biological and clinical differences in the cohorts, differences in sample collection methods, and, perhaps most significantly, differences in the instrument platform on which data was acquired. The BALF-DB was developed using data from an Agilent 6545 QTOF whereas the External Test samples were analyzed using an Agilent 6520 QTOF. Importantly, searching an independent dataset against the BALF-DB resulted in improved match rates and increased MS/MS compared to searching general databases. In addition, because our BALF-DB includes frequency and reproducibility metrics, one can be more confident that the detected molecules are present in BALF. Overall, these results emphasize the need to continue population of this prototypic BALF-DB with additional samples from a variety of clinical cohorts and instrument platforms. While beyond the scope of the current study, the flexible and highly automated nature of our prototypic BALF-DB make these next steps very achievable.

## Additional information

**How to cite this article:** Walmsley, S. *et al.* A prototypic small molecule database for bronchoalveolar lavage-based metabolomics. *Sci. Data* 5:180060 doi: 10.1038/sdata.2018.60 (2018).

**Publisher’s note:** Springer Nature remains neutral with regard to jurisdictional claims in published maps and institutional affiliations.

## Supplementary Material



Supplementary File 1

Supplementary File 2

## Figures and Tables

**Table 1:** Clinical characteristics of the SPIROMICS cohort (*n*=117).

**Patient Metric**	**Male**	**Female**	**p-value**
Gender	59	58	
Currently a smoker	41	36	0.2844
Never a smoker	5	8	0.5346
Gold1	13	9	0.5058
Gold2	12	8	0.4872
Gold3	4	1	0.3642
**'**Gold’ status refers to the stages of COPD; it is a measure of lung function, with Gold 1 being early stage and Gold 4 being late stage COPD.			

**Table 2:** Total ID’s by HMDB chemical class.

**HMDBChemical Class**	**Lipid**	**Aqueous**
*Alkaloids and derivatives*	5	3
*Azoles*		1
*Azepines*	1	
*Benzene and substituted derivatives*	9	6
*Benzenoids*	21	7
*Benzopyrans*	2	
*Carbohydrates and carbohydrate conjugates*	1	1
*Carbonyl compounds*	1	3
*Carboxylic acids and derivatives*	9	
*Ciguatera toxins*	1	
*Diazines*	1	
*Ethers*	2	
*Fatty Acyls*	23	
*Flavonoids*	2	2
*Glycerolipids*	7	
*Glycerophospholipids*	7	
*Homogeneous non-metal compounds*		1
*Isoflavonoids*	1	
*Lineolic acids and derivatives*	1	1
*Lipids and lipid-like molecules*	1355	64
*Morphinans*		1
*Naphthopyrans*	1	1
*Neoflavonoids*	1	
*Nucleosides, nucleotides, and analogues*	4	10
*Organic acids and derivatives*	58	71
*Organic nitrogen compounds*		2
*Organic oxygen compounds*		11
*Organic phosphoric acids and derivatives*		1
*Organic oxygen compounds*	32	1
*Organoheterocyclic compounds*	43	19
*Organonitrogen compounds*	1	2
*Organosulfur compounds*	2	
*Oximes*		1
*Phenylpropanoic acids*	1	
*Phenylpropanoids and polyketides*	52	5
*Prenol lipids*	13	4
*Protoberberine alkaloids and derivatives*	1	
*Purine nucleosides*		1
*Pyridines and derivatives*	1	1
*Steroids and steroid derivatives*	13	
*Sulfonic acids and derivatives*	1	
*Tannins*	2	
*Tetrahydroisoquinolines*	1	
In general, the chemical classes of compounds were consistent with the fraction in which they were found. For example, the predominant chemical classes of ‘lipids and lipid-like molecules’ were found when compounds from the lipid fraction were searched against HMDB. Conversely, ‘Organic acids and derivatives’ was the predominant class of compounds found in the aqueous fraction when HMDB was searched.		

**Table 3:** Total ID’s by Lipid Maps lipid category.

**Lipid MapsLipid Category**	**Lipid**	**Aqueous**
*Fatty Acyls*	633	17
*Glycerolipids*	472	1
*Glycerophospholipids*	127	14
*Polyketides*	105	14
*Prenol Lipids*	7	1
*Sphingolipids*	16	3
*Sterol Lipids*	16	8
In general, the lipid categories of compounds were consistent with the fraction in which they were found. The predominant lipid categories present in the lipid fraction were of greater number than those for the aqueous fraction and relatively more ‘glycerolipids’ were found in the lipid experiment.		

**Table 4:** Internal and external validation tests and success rates.

		**% Coverage Matched to DB**			**Number of Hits**		
**Dataset**	**Extraction**	**M+RT**	**M**	**RT Tolerance Used**	**M+RT**	**M**	**# Test Compounds**
*COPDGene Human*	*Lipid*	35	45	1.5	874	1131	2493
*Mouse*	*Lipid*	37	45	1.5	946	1165	2566
*Internal Validation*	*Lipid*	99	99	0.5	9119	9137	9197
*Internal Validation*	*Aqueous*	46	47	1.5	113	116	244
Data from a nested subset of 10 samples used to develop the DBs (internal validation) were re-processed and searched against the Lipid and Aqueous BALF-DBs. This resulted in 99% match to the lipid DB and 46 match to the aqueous DB when neutral mass (M) and retention time (RT) were used. When data from two independent experiments, COPDgene Human and Mouse, were searched, the match rates were 35% and 37% respectively. These both increased to 45% when only neutral mass was used.							

**Table 5:** Match rates to HMDB and Lipid Maps using originally processed and searched datasets.

	**Lipid Experiment**			**Aqueous Experiment**		
**Origin**	**# Test Cmpds**	**# HMDB Hits (% of Total)**	**# Lipid Maps Hits (% of Total)**	**# Test Cmpds**	**# HMDB Hits (% of Total)**	**# Lipid Maps Hits (% of Total)**
*Human BALF*	3164	783 (25%)	571 (18%)	275	58 (21%)	17 (6%)
*Mouse BALF*	2779	634 (23%)	447 (16%)	331	77 (23%)	24 (7%)
Data from an independent experiment were processed and searched as previously described [23]. As previously reported, searching 3164 Human and 2779 Mouse compounds against HMDB resulted in 783 (24.7%) and 624 (22.8%) matches, respectively. This is in contrast to the 45% match rate achieved when the BALF-DBs are used for these datasets. In all cases, neutral mass, and not retention time (RT), were used to search the databases. Searching using RT reduces the number of hits but increases confidence in the identification. [Compounds=Cmpds; Human Metabolome Database=HMDB]						
