# Supplementary material for: A prototypic small molecule database for bronchoalveolar lavage-based metabolomics
Source: Sci Data. 2018 Apr 17;5:180060. doi: 10.1038/sdata.2018.60 (PMC5903367; doi:10.1038/sdata.2018.60)

Supplementary File 1

Spectra used in the validation of compounds in the aqueous MS/MS experiment.

Included are plots showing the matches from the NIST 17 library as described in the text. Red lines show peaks produced from the sample, and blue line show peaks from the NIST library entry. Number and text at the top of each spectra show the order number and compound name, the NIST MS Search score, dot product, and database probability as produced by the NIST MS search program. Upper left text in each plot are the referenced experimental file name and experiment tag (AQ), the observed precursor mz and the retention time in minutes. The collision energy (cev), polarity (pos) and adducted ion observed are noted. A distance metric (the square root of the sum of squares for the difference between the matched fragment peaks) is included (dist). The lower left panel includes the nist library index number (nist_msms) as stored in our SQL database, the CAS number, a molecular formula and the library MZ. Precursor mass error (err) is noted in ppm and Dalton (Da).

1 .   .beta.−Methylphenethylamine
Score=560 Dot=999 prob=19.4

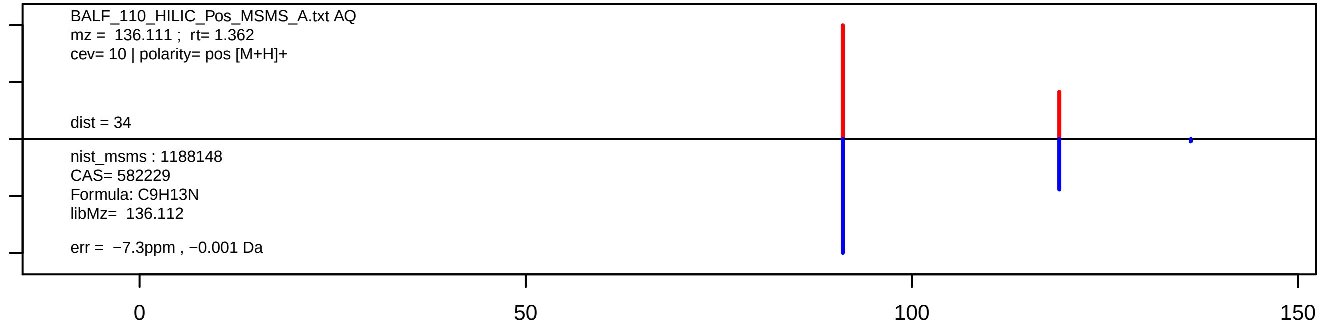

2 .   (−)−Methamphetamine
Score=559 Dot=998 prob=48.8

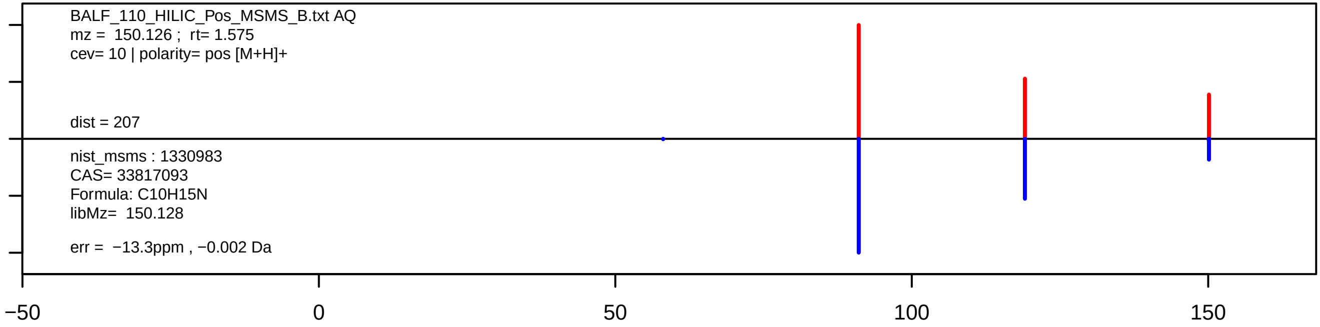

3 .   (+)−Methamphetamine
Score=555 Dot=994 prob=51.2

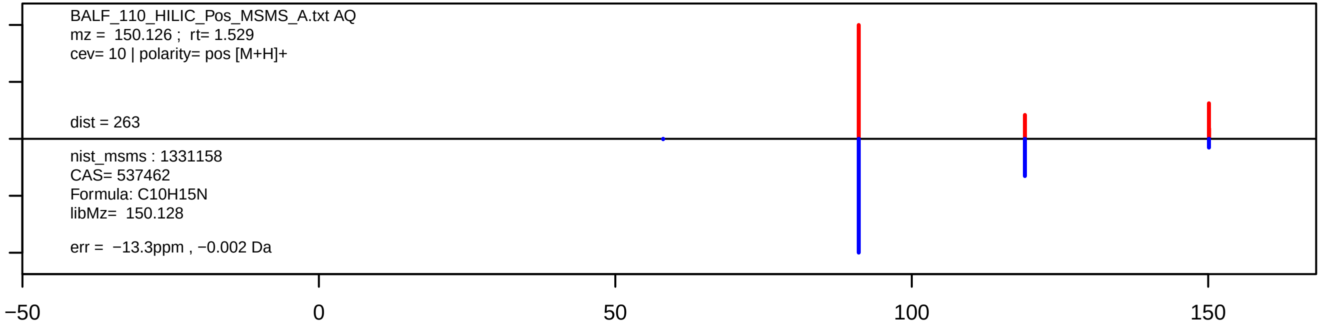

4 .   (3−Carboxypropyl)trimethylammonium cation
Score=757 Dot=929 prob=99

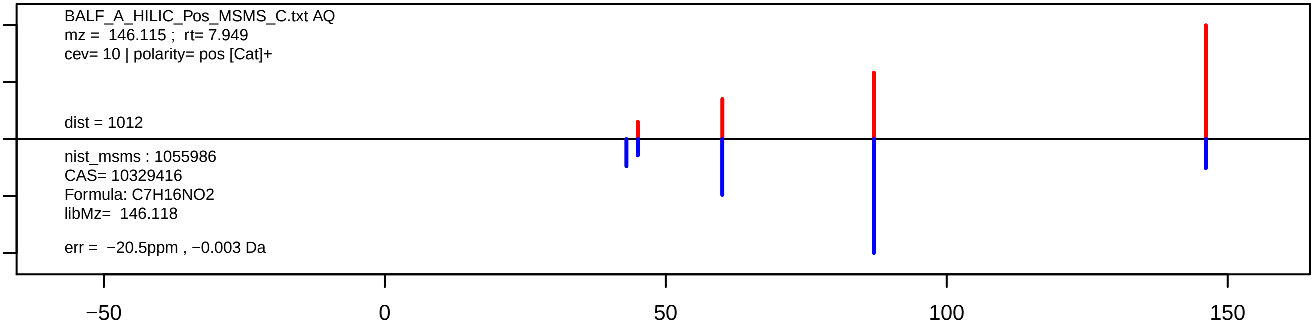

5 .   (5E)−4−Methyl−5−{[(phenylsulfonyl)oxy]imino}−1,1'−bi(cyclohexane)−3,6−dien−2−one
Score=159 Dot=941 prob=88.9

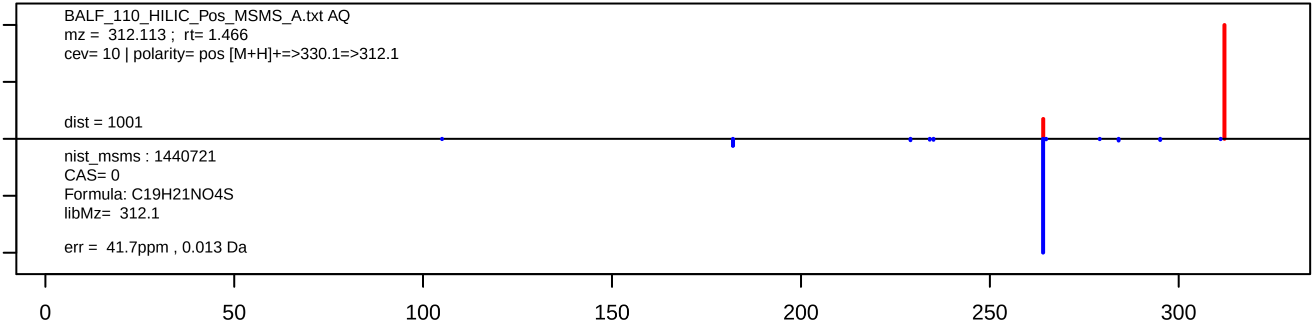

6 .   1−Arachidoyl−2−hydroxy−sn−glycero−3−phosphocholine
Score=399 Dot=998 prob=21.8

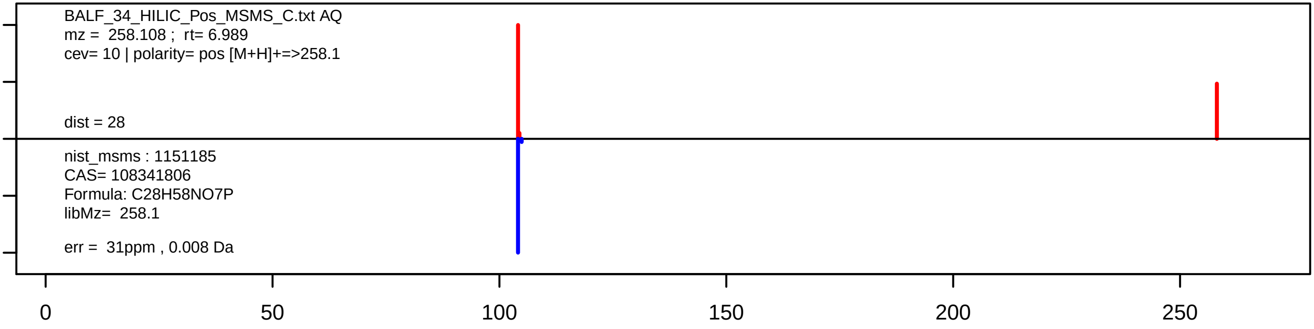

7 . 1−Methyl−3−phenylpropylamine
Score=560 Dot=999 prob=35.7

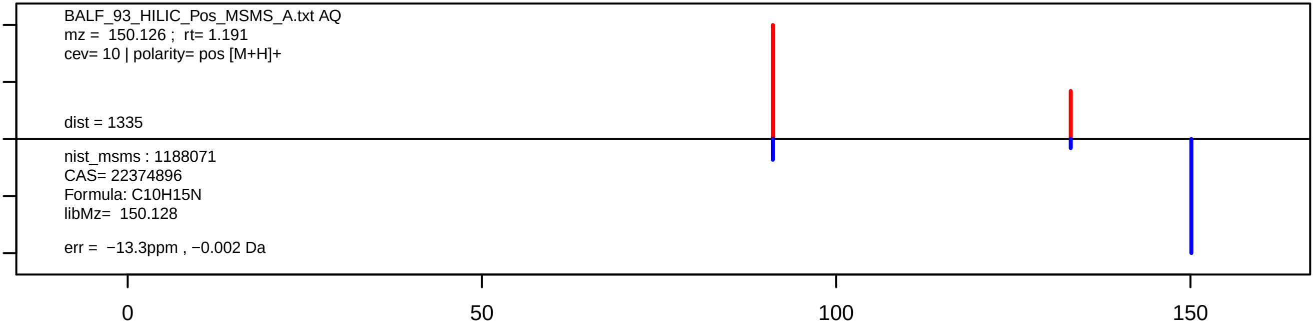

8 . 1−Oleoyl−2−palmitoyl−sn−glycero−3−phosphocholine
Score=365 Dot=833 prob=70.2

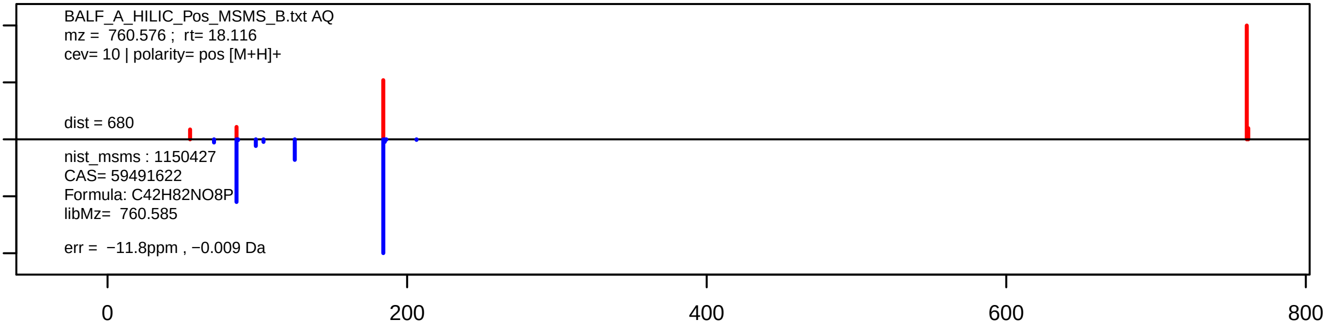

9 . 1−Pentadecanoyl−sn−glycero−3−phosphocholine
Score=400 Dot=999 prob=11.3

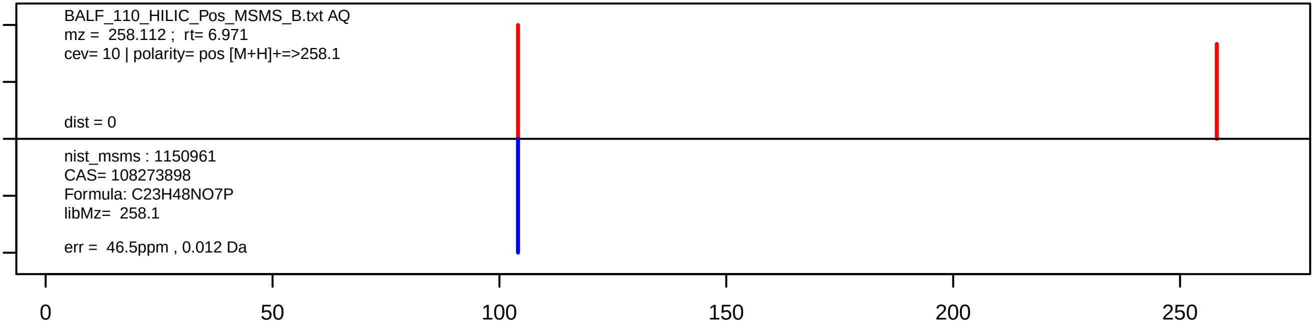

10 .   1−Propanone, 1−(1,3−benzodioxol−5−yl)−2−(dimethylamino)−
Score=928 Dot=953 prob=99

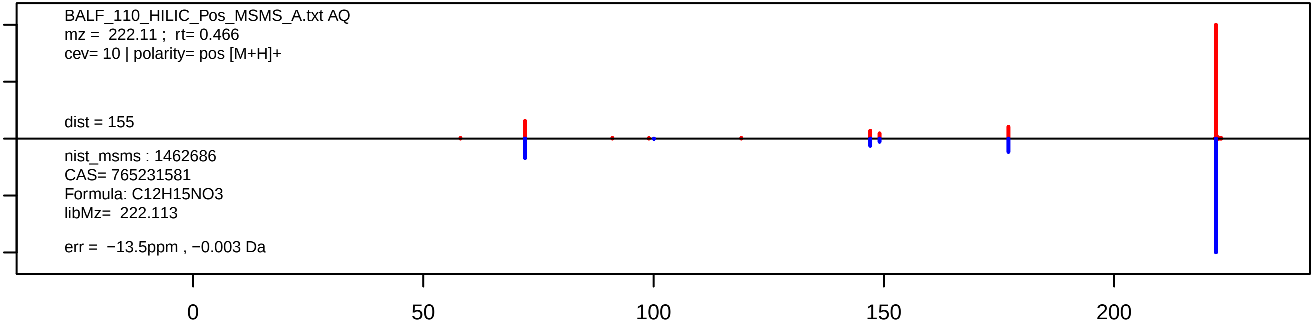

11 .   1,2−Dihexadecanoyl−sn−glycero−3−phosphocholine
Score=320 Dot=987 prob=90

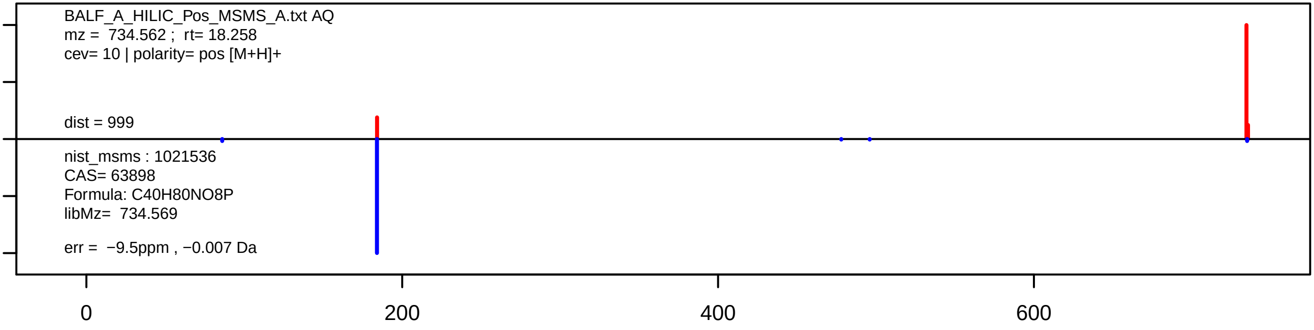

12 .   1,2−dioleoyl−sn−glycero−3−phosphatidylcholine
Score=400 Dot=999 prob=50

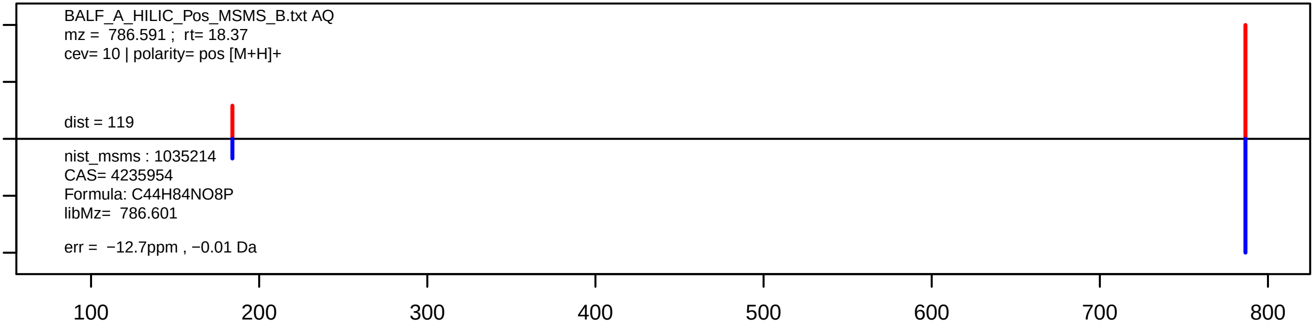

13 . 1,2−Dipalmitoyl−sn−glycero−O−ethyl−3−phosphatidylcholine cation
Score=502 Dot=960 prob=82.2

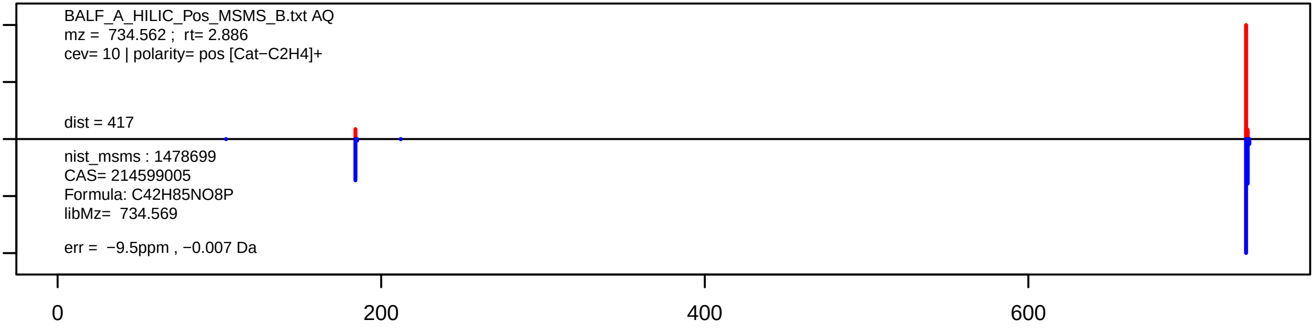

14 . 2−(Ethylsulfonyl)ethanol
Score=412 Dot=841 prob=83.4

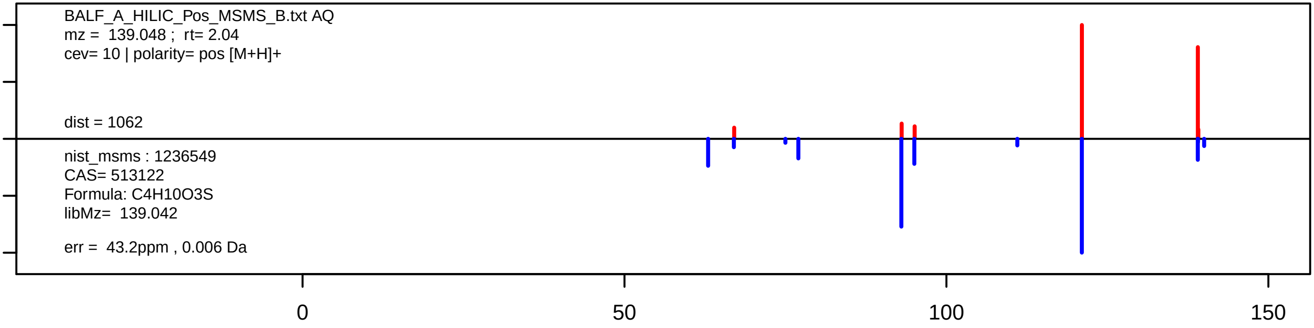

15 . 2−[(2,6−Dimethylphenyl)amino]−N,N,N−triethyl−2−oxoethanaminium cation
Score=382 Dot=959 prob=78.8

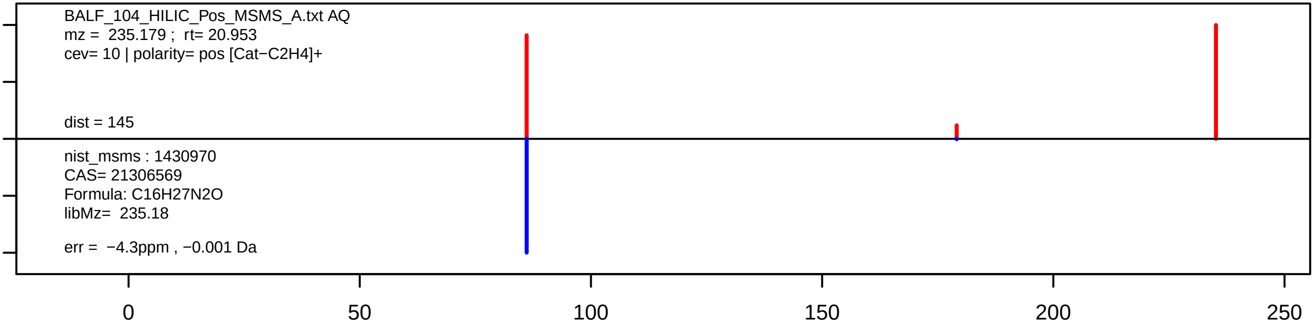

16 . 2−Methylamino−1−(3,4−methylenedioxyphenyl)propan−1−one
Score=867 Dot=957 prob=59.9

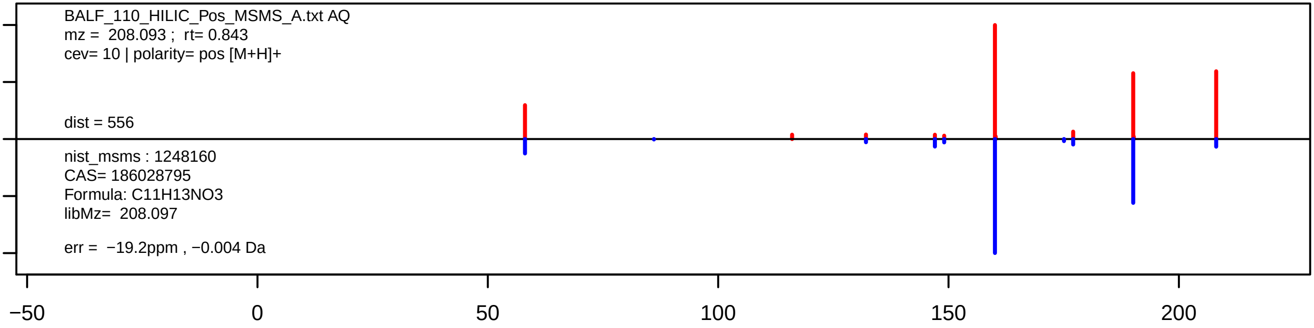

17 . 2−Methylamino−1−phenylbutane
Score=411 Dot=966 prob=8.8

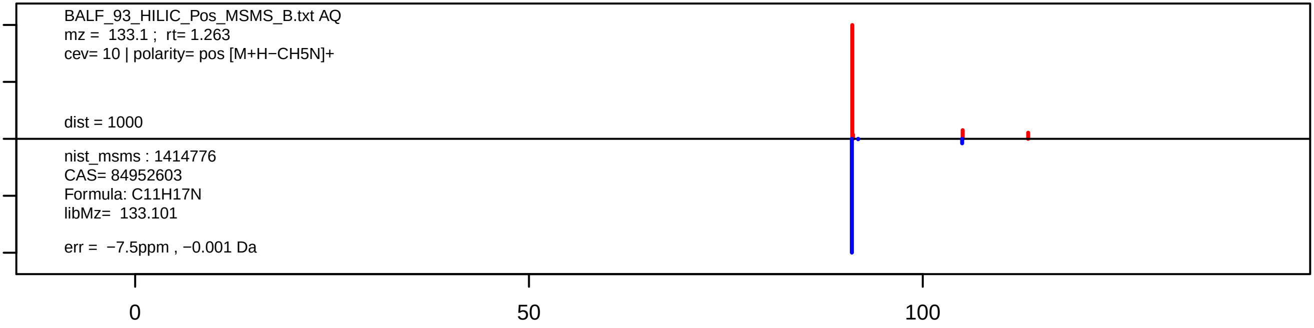

18 . 2−Oleoyl−1−palmitoyl−sn−glycero−3−phosphocholine
Score=369 Dot=932 prob=83.8

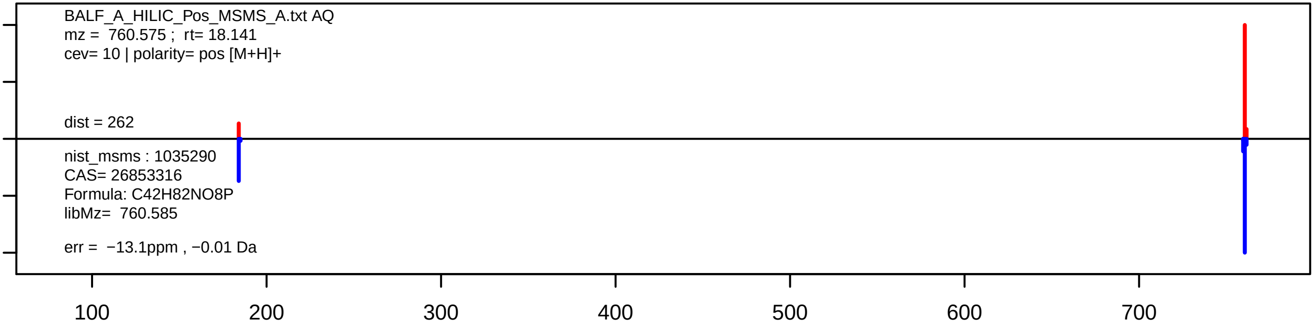

19 . 2,3−Methylenedioxymethcathinone
Score=754 Dot=918 prob=90.4

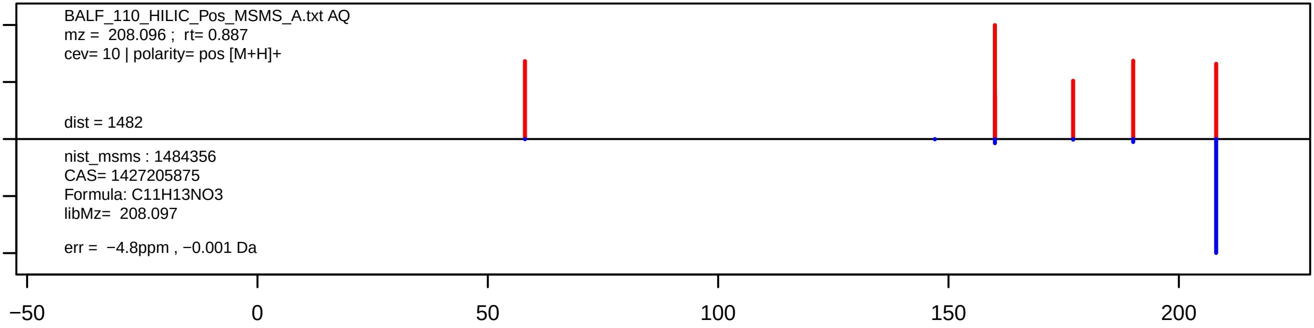

20 . 2,6−Pyridinediamine, N6−[2−[[4−(2,4−dichlorophenyl)−5−(1H−imidazol−2−yl)−2−pyrimidinyl]amino]ethyl]−3−nitro−
Score=427 Dot=824 prob=93.2

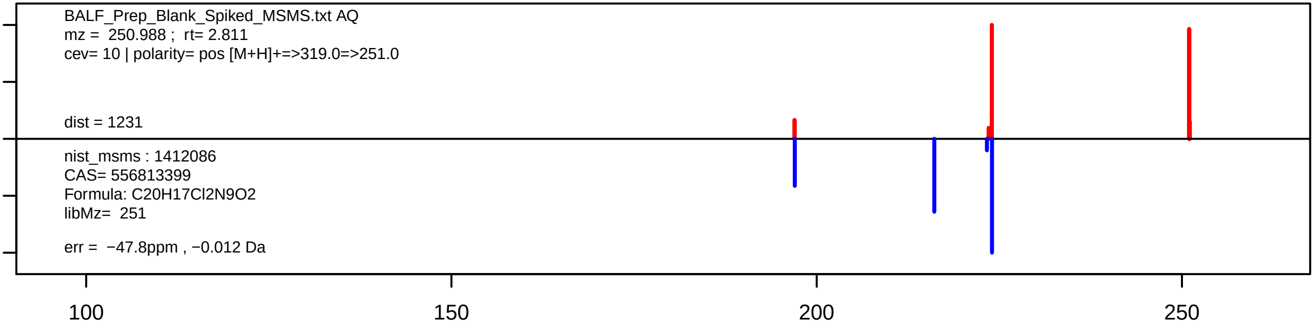

21 . 2'−Deoxyinosine
Score=540 Dot=982 prob=97.7

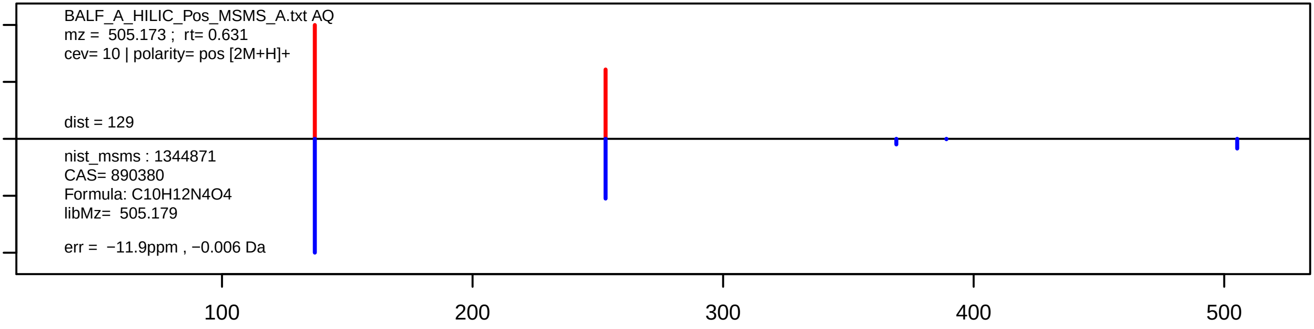

22 .   3−[N,N−Bis(2−hydroxyethyl)amino]−2−hydroxypropanesulfonic acid
Score=400 Dot=999 prob=25.6

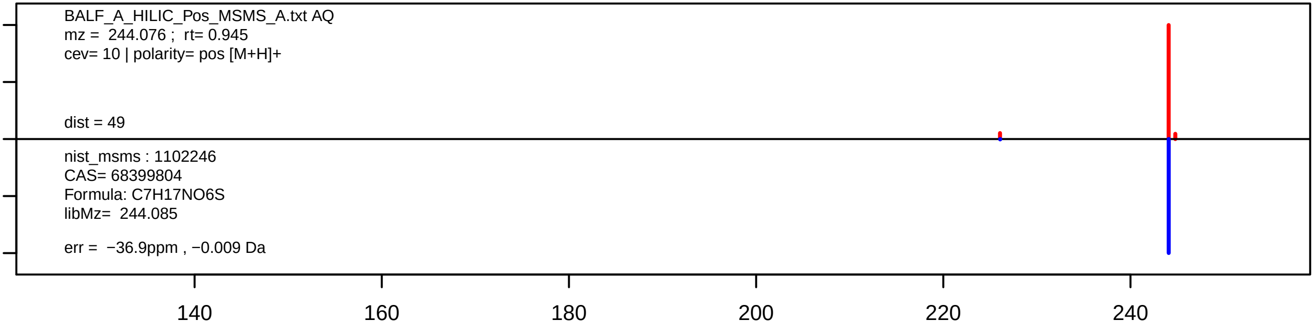

23 .   3−Aminopentanoic acid
Score=474 Dot=844 prob=78.5

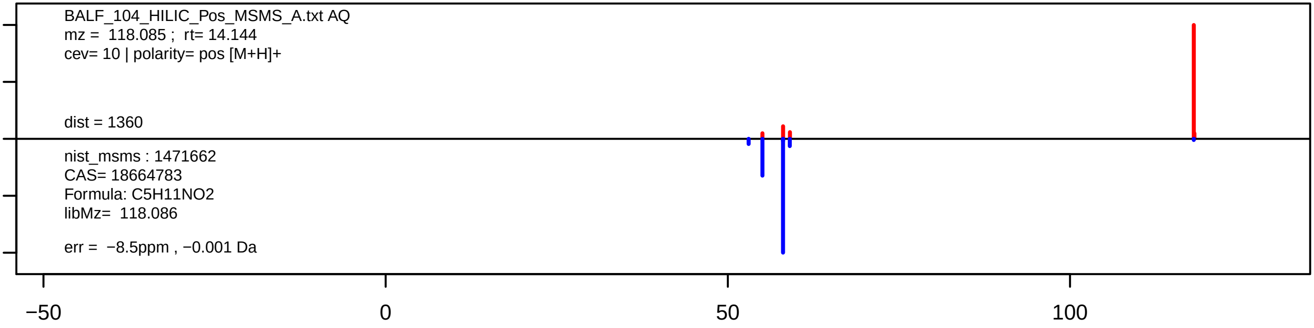

24 .   3−Hydroxy−2',4',5'−trimethoxyflavone
Score=200 Dot=924 prob=79.2

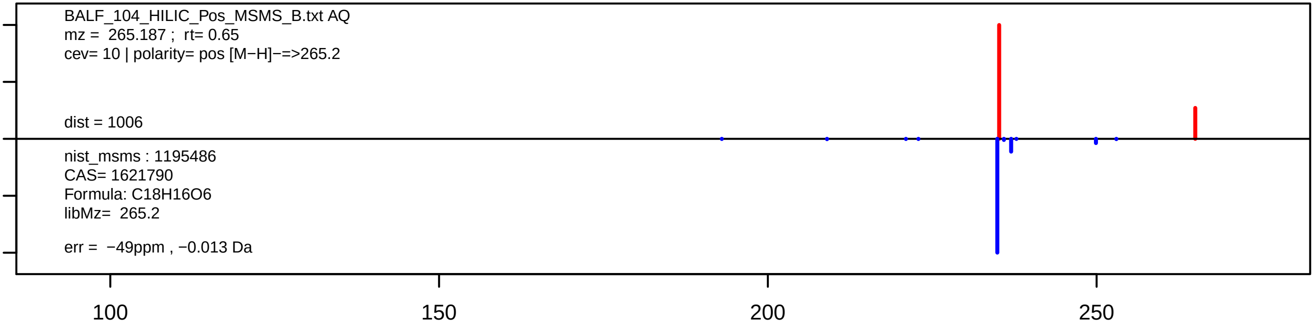

25 . 3−Isoquinolinecarboxamide, 1−(2−chlorophenyl)−N−methyl−N−(1−methylpropyl)−
Score=326 Dot=959 prob=49.6

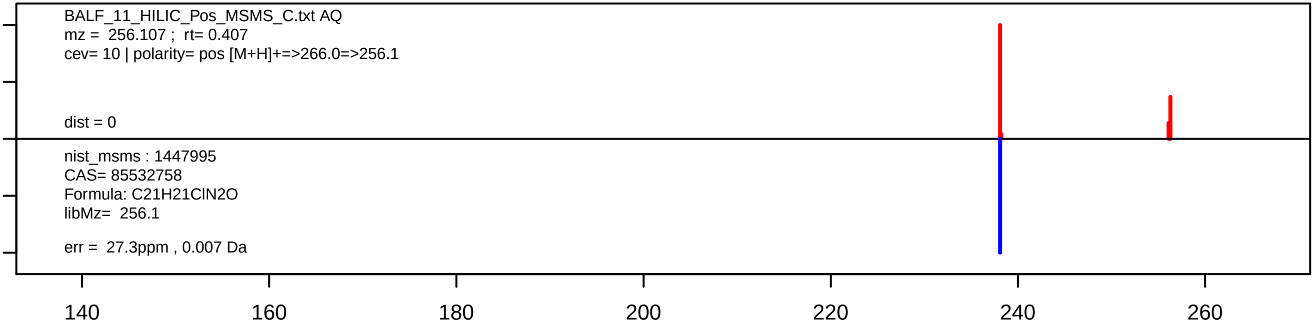

26 . 3,4−Dimethoxymethcathinone
Score=131 Dot=892 prob=45.6

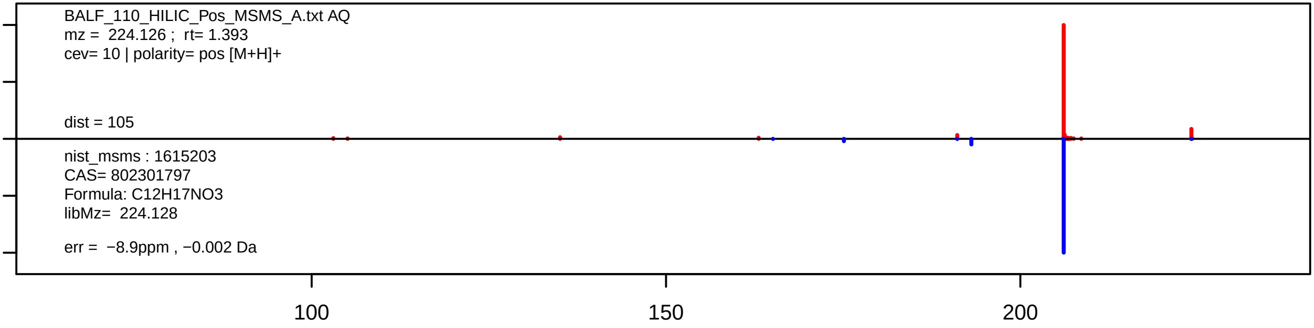

27 . 4−Imidazoleacrylic acid
Score=885 Dot=959 prob=98.5

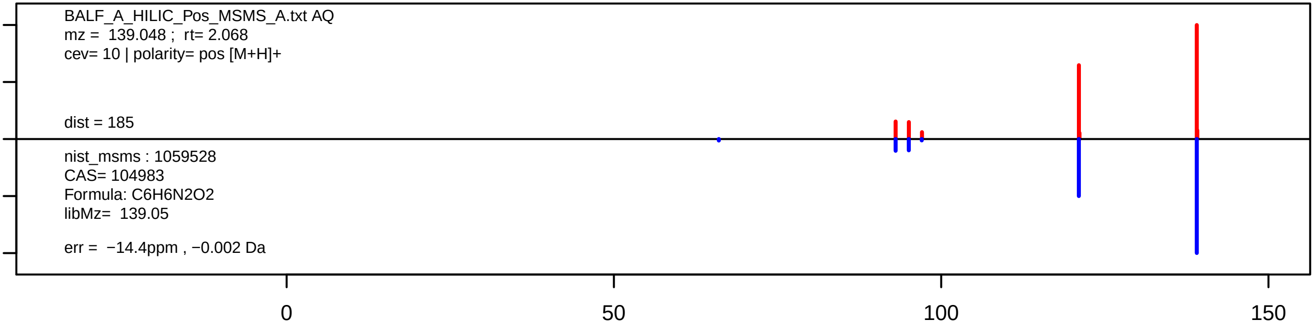

28 . 4−Phenylbutylamine
Score=637 Dot=968 prob=57.4

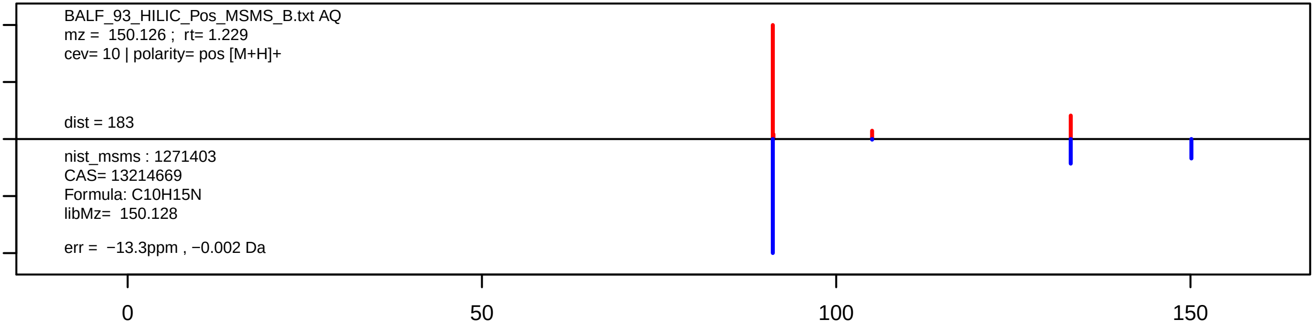

29 . 4'−Chloro−.alpha.−pyrrolidinopropiophenone
Score=257 Dot=881 prob=92.3

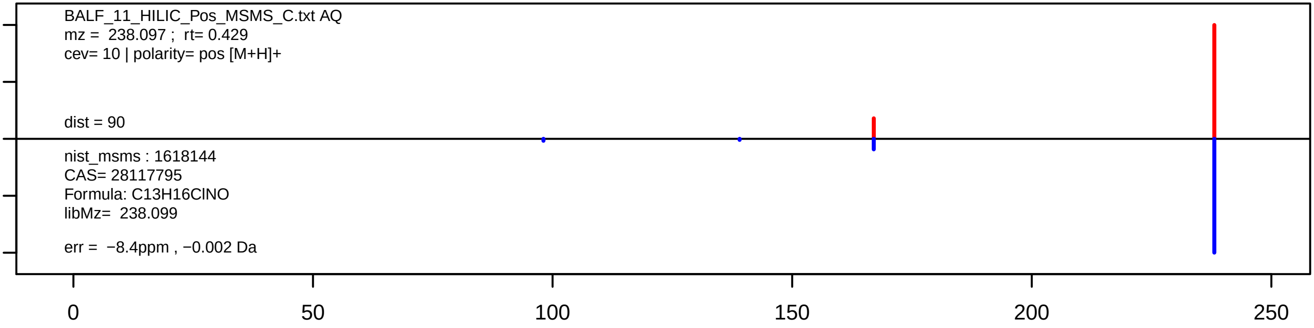

30 . 5−Aminovaleric acid
Score=404 Dot=958 prob=95.4

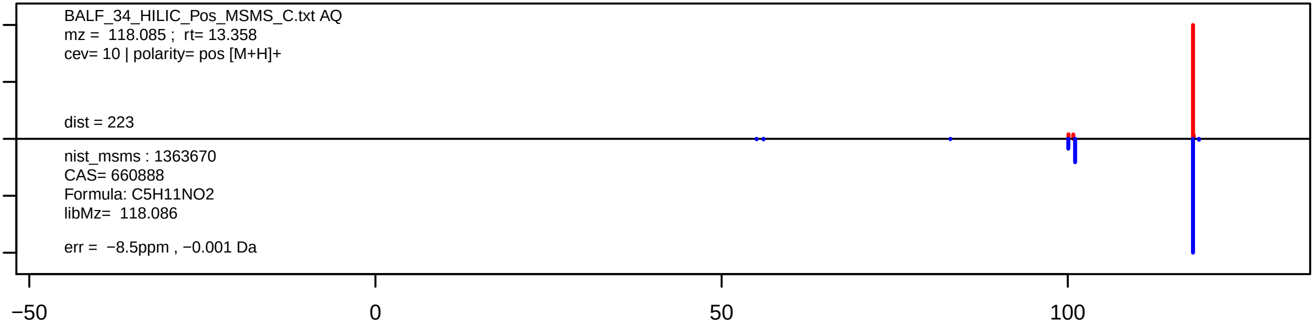

31 .   5,5−Dimethylimidazolidine−2,4−dione
Score=400 Dot=999 prob=3.5

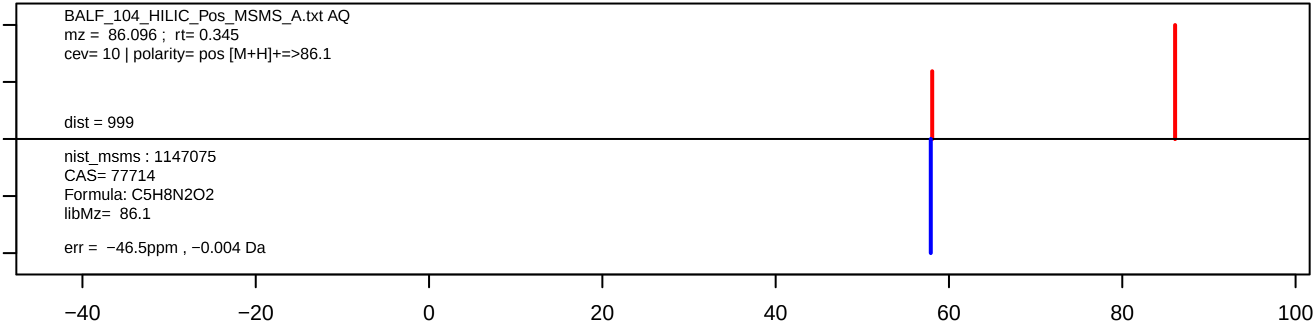

32 .   5.alpha.−Androstan−17.beta.−ol−3−one
Score=400 Dot=999 prob=32.9

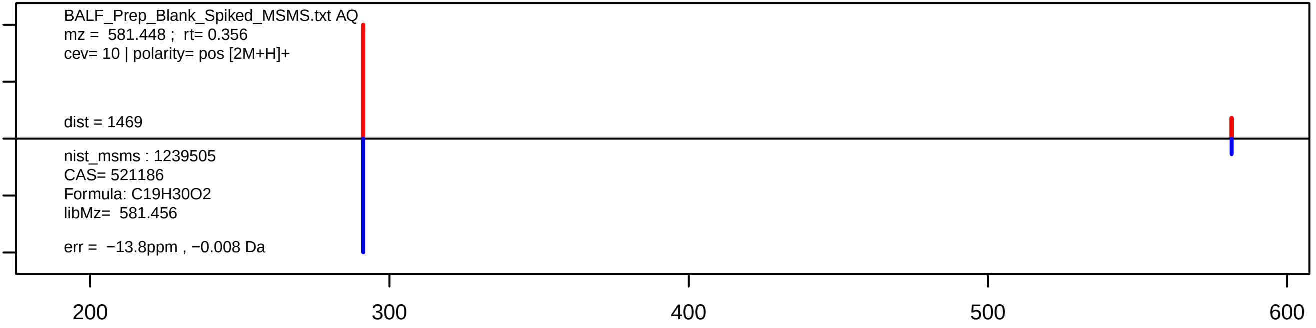

33 .   7H−Pyrrolo[2,3−g]benzothiazol−7−one, 6,8−dihydro−8−(1H−imidazol−5−ylmethylene)−
Score=342 Dot=964 prob=63.5

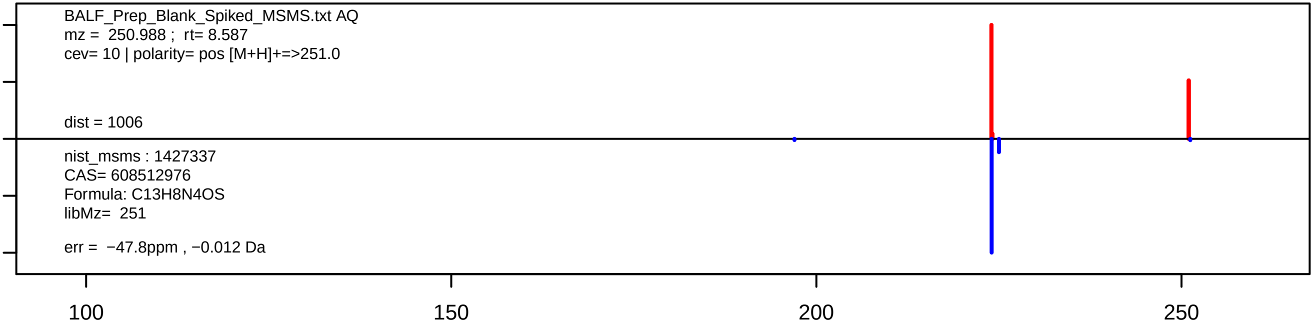

34 .   Acetic acid
Score=132 Dot=946 prob=52.5

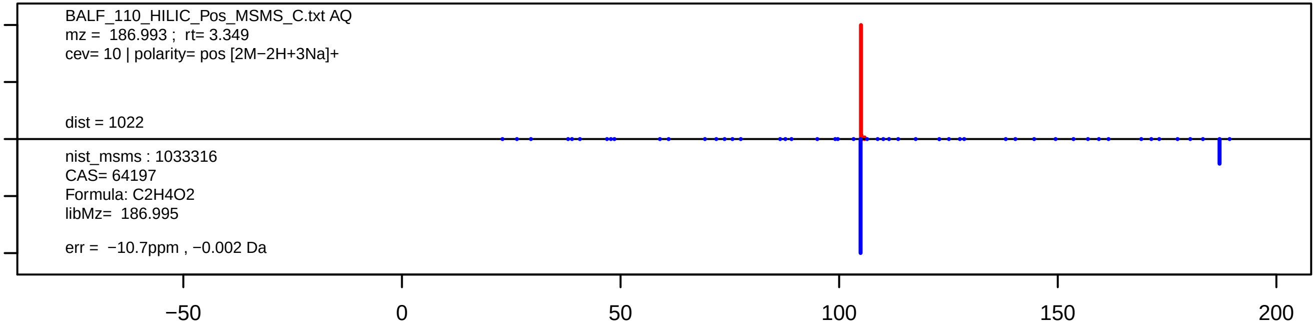

35 .   Acetyl−DL−carnitine
Score=244 Dot=907 prob=59.5

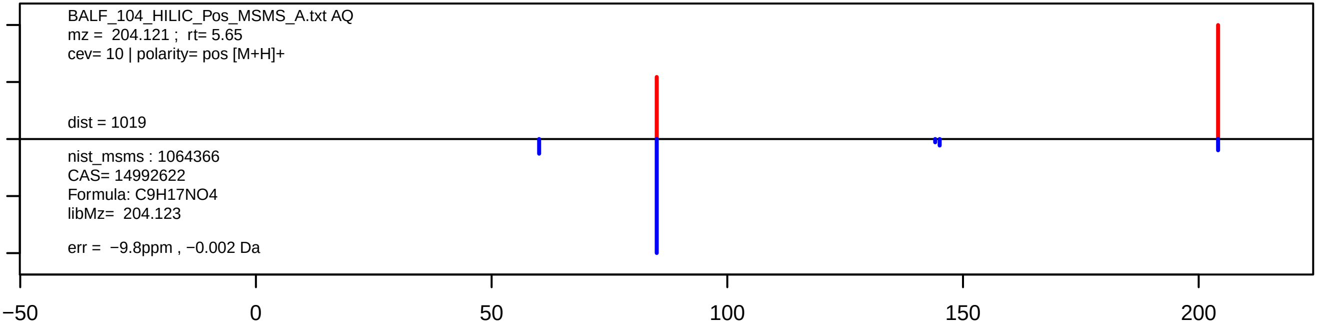

36 .   Acetyl−L−carnitine
Score=992 Dot=992 prob=51.2

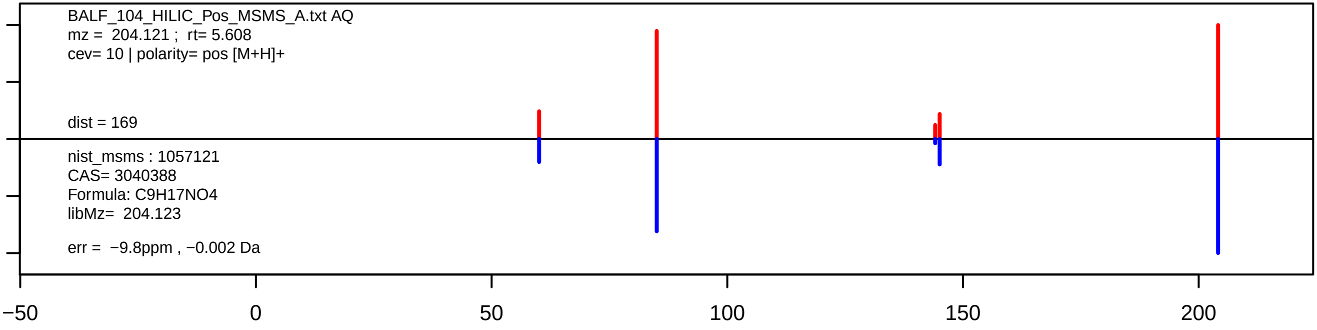

37 .   Adenosine
Score=318 Dot=827 prob=89

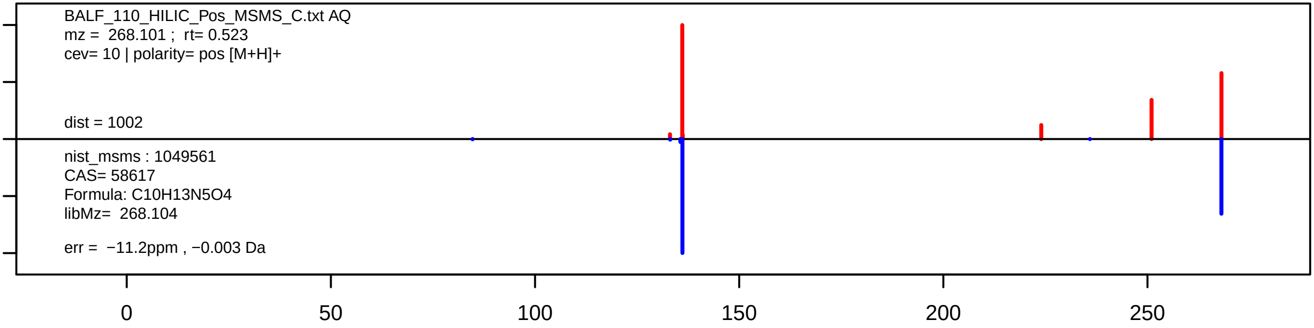

38 .   Adenosine 2'−monophosphate
Score=288 Dot=820 prob=42.8

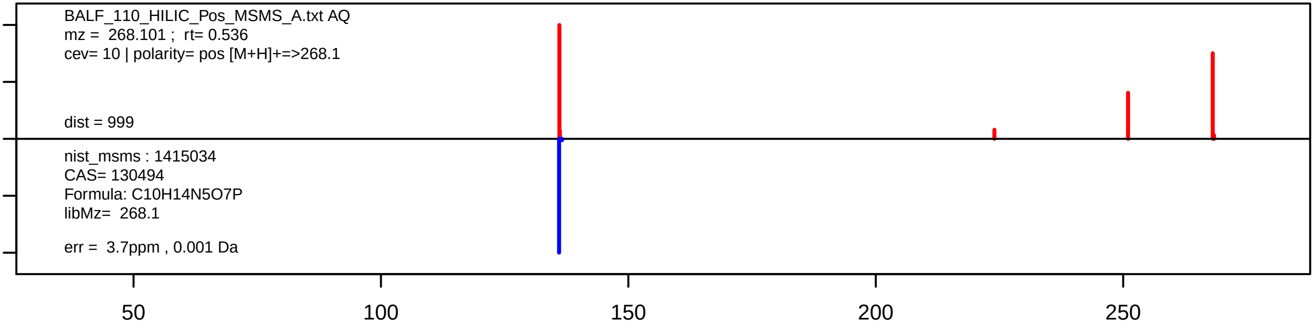

39 .   Ala−His
Score=841 Dot=935 prob=97.9

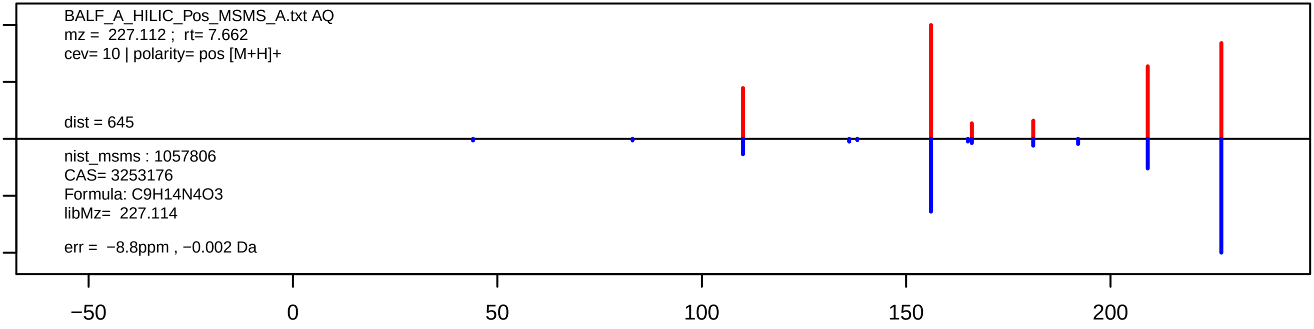

40 . Ala−Lys
Score=740 Dot=918 prob=46.2

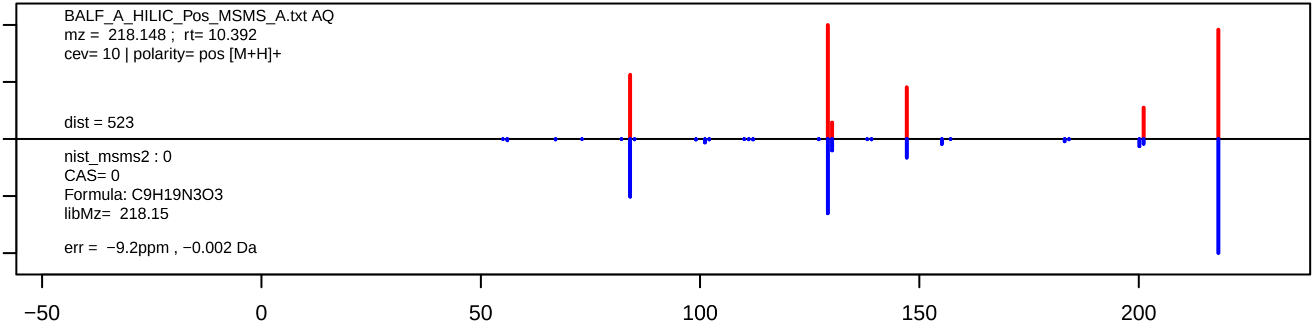

41 . Ala−Phe
Score=386 Dot=877 prob=98

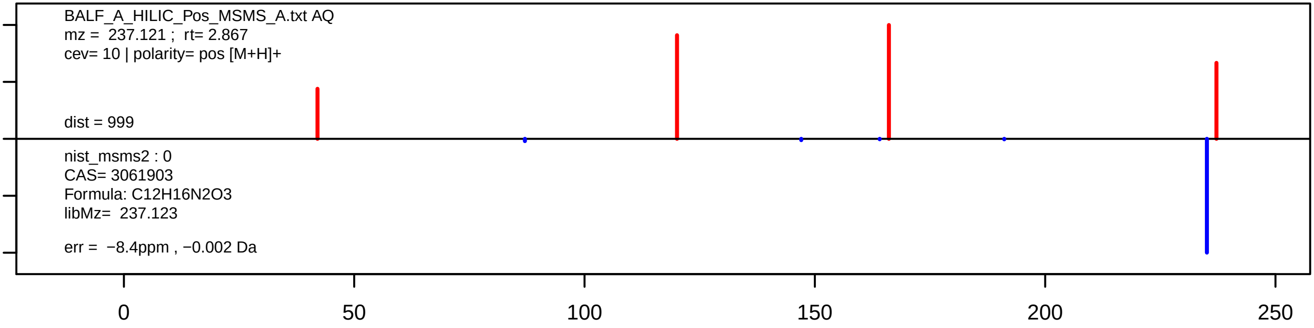

42 . Albuterol
Score=915 Dot=960 prob=98.7

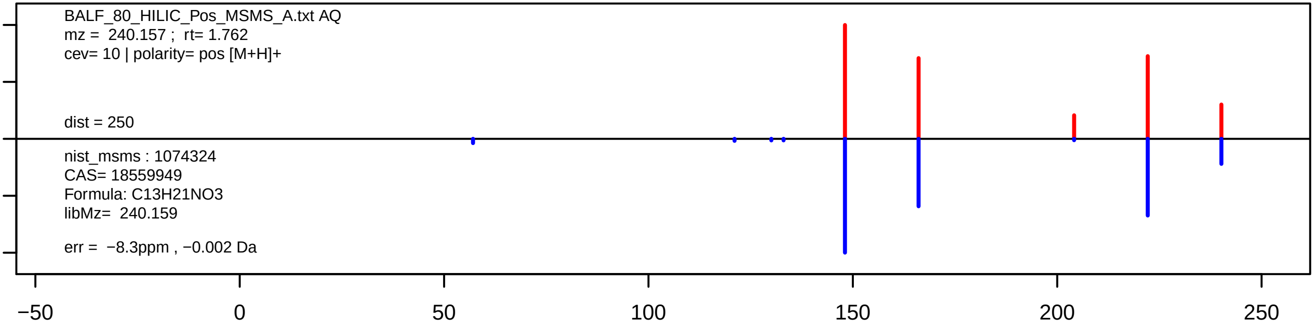

43 . Allopurinol
Score=400 Dot=999 prob=6

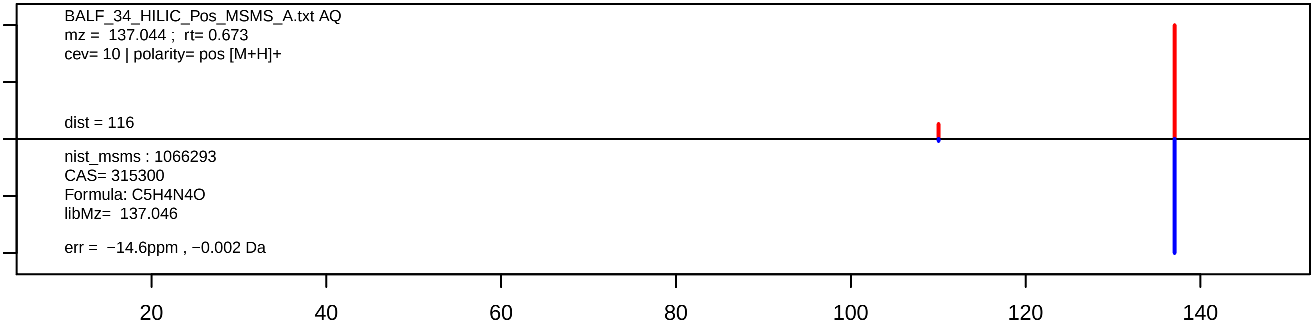

44 . Allopurinol riboside
Score=430 Dot=973 prob=87.5

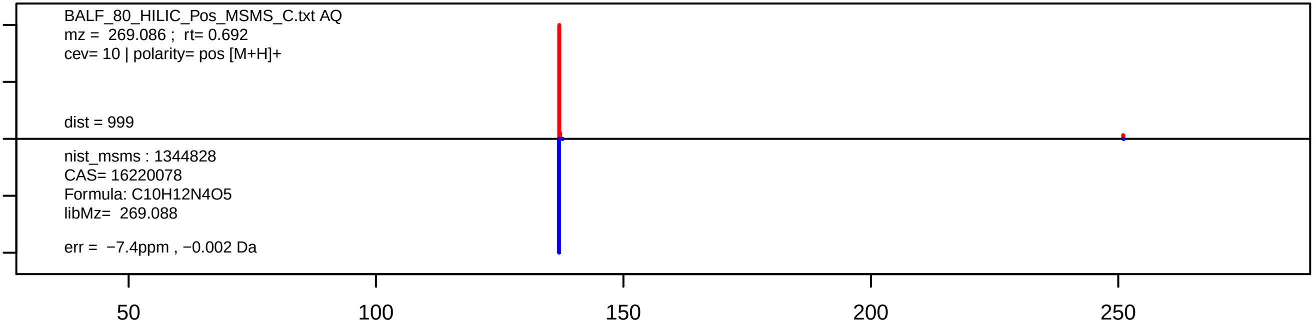

45 . Aminodiphenylmethane
Score=823 Dot=991 prob=48.9

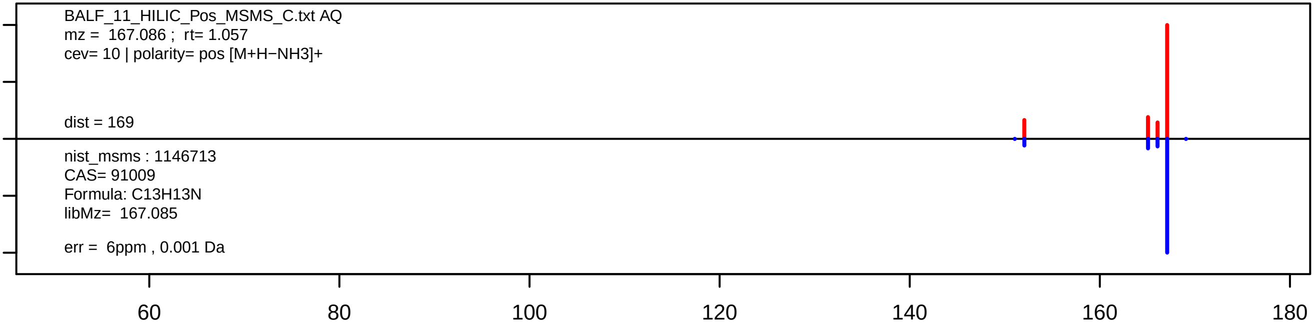

46 . Amphetamine
Score=560 Dot=999 prob=14.8

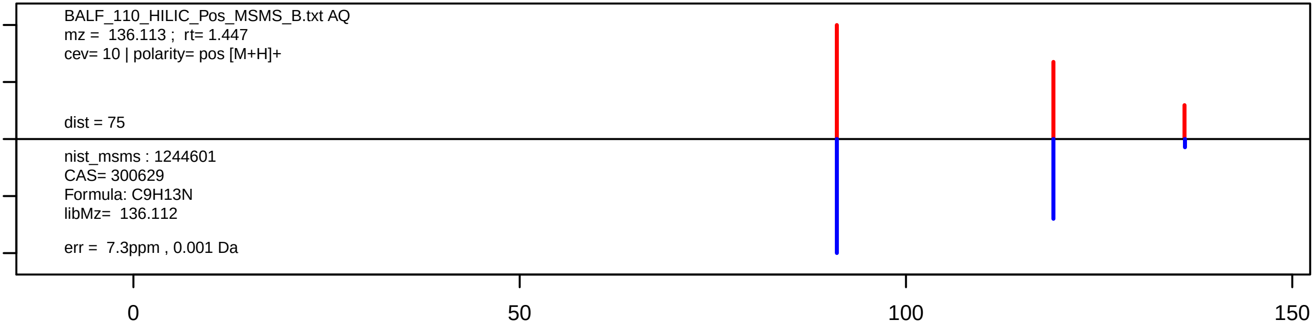

47 . Arg−Phe
Score=49 Dot=865 prob=17.9

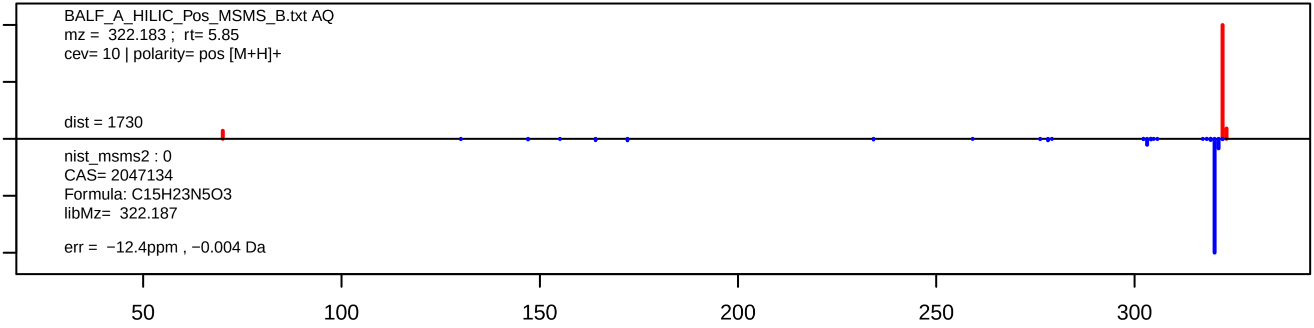

48 . Arg−Val
Score=467 Dot=826 prob=48.9

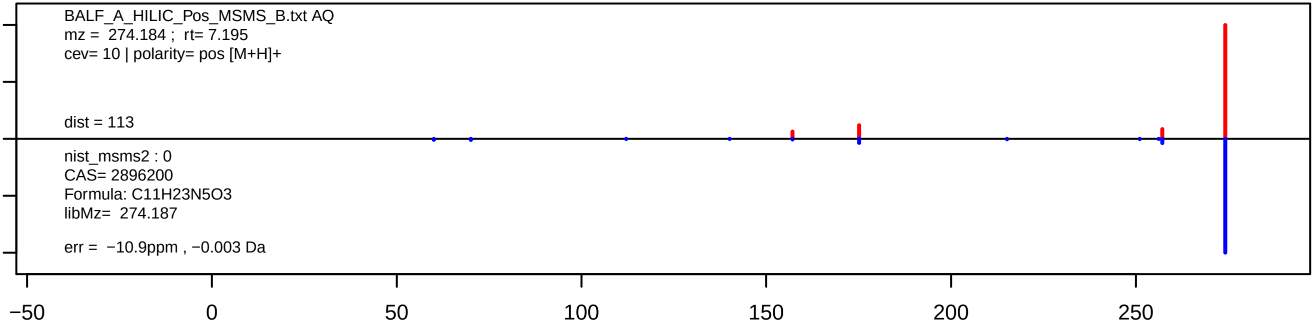

49 . Benzhydrol
Score=815 Dot=983 prob=44.1

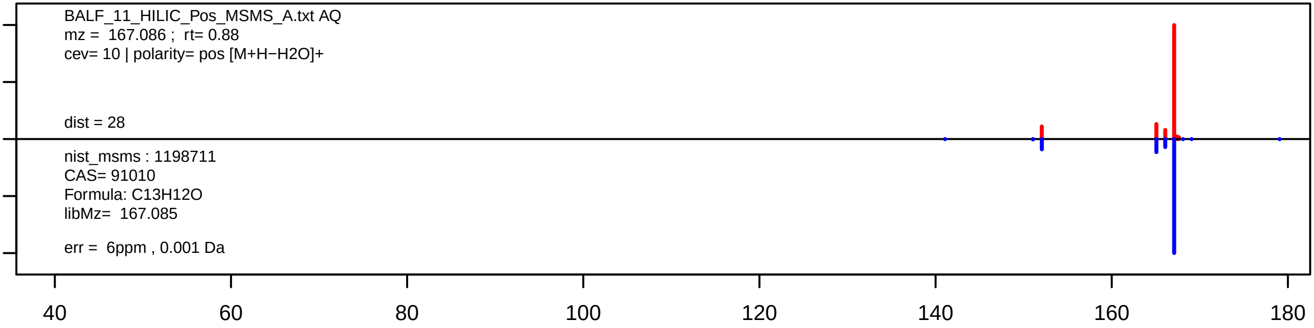

50 . Benzoylnorecgonine
Score=400 Dot=999 prob=100

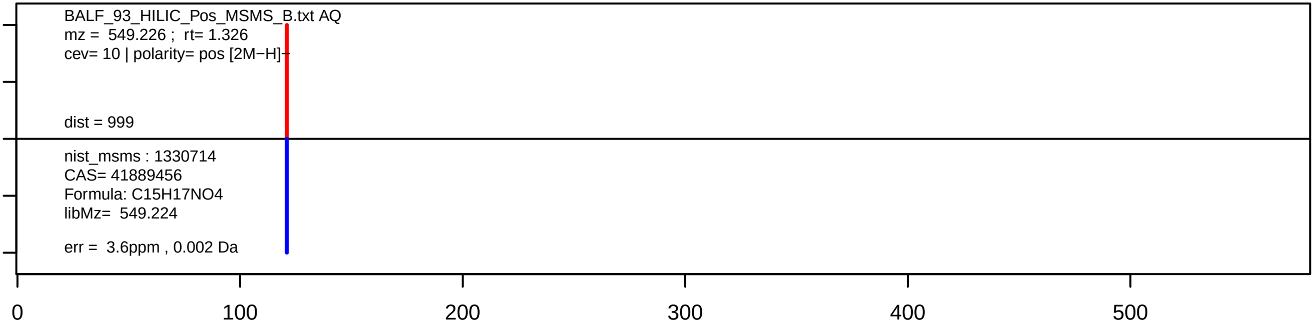

51 . Benzyl alcohol
Score=400 Dot=999 prob=1.5

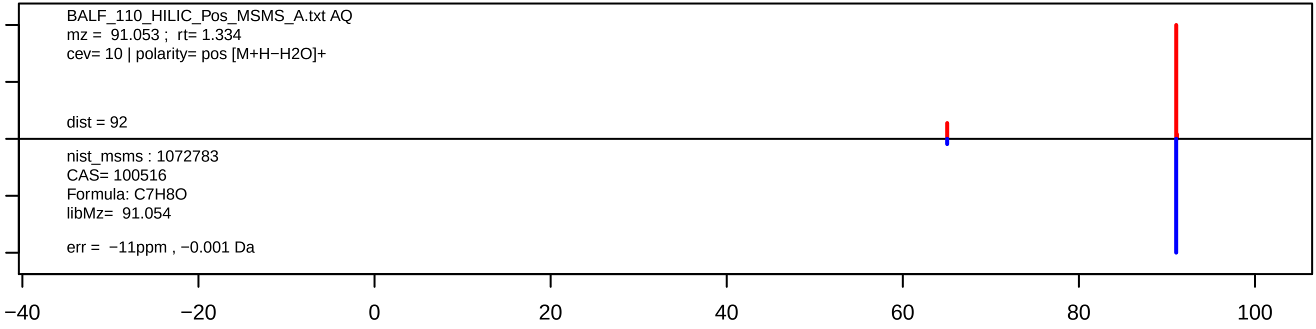

52 . Betaine
Score=560 Dot=999 prob=98.7

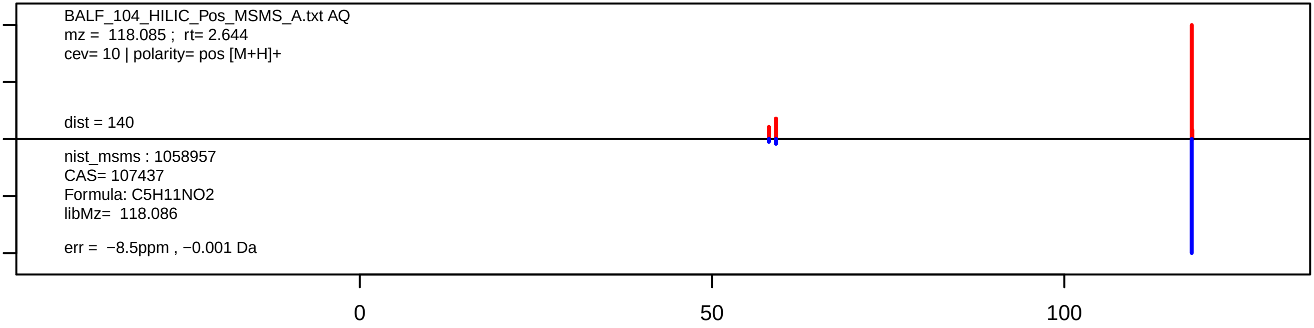

53 . Bupropion
Score=637 Dot=887 prob=98.1

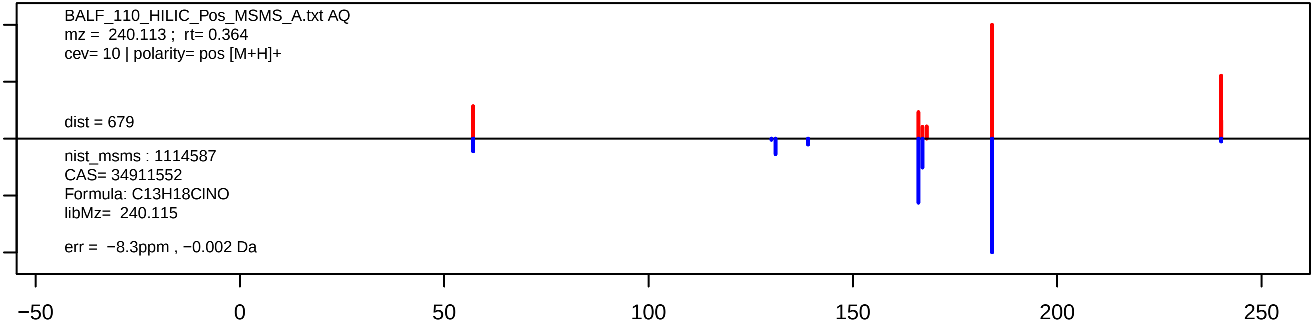

54 . Carbamic acid, N−(2,2−diphenylacetyl)−, ethyl ester
Score=435 Dot=840 prob=49.3

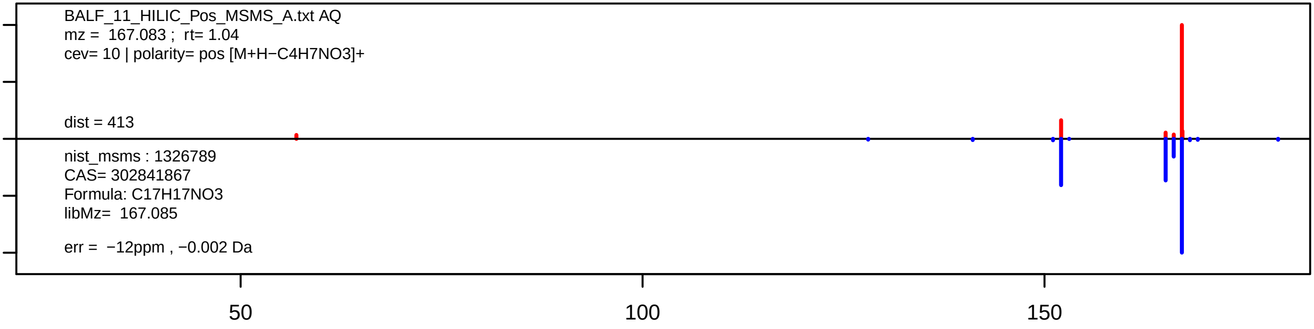

55 . Choline cation
Score=555 Dot=874 prob=97.2

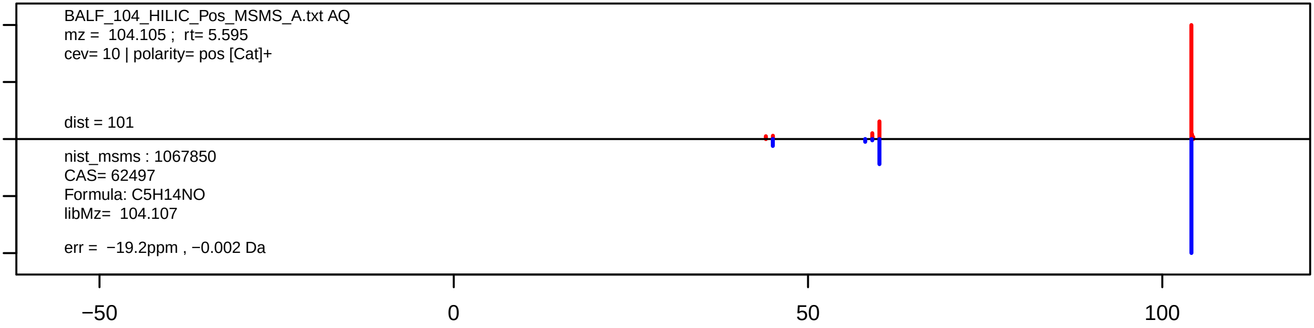

56 . Citalopram
Score=395 Dot=855 prob=96.3

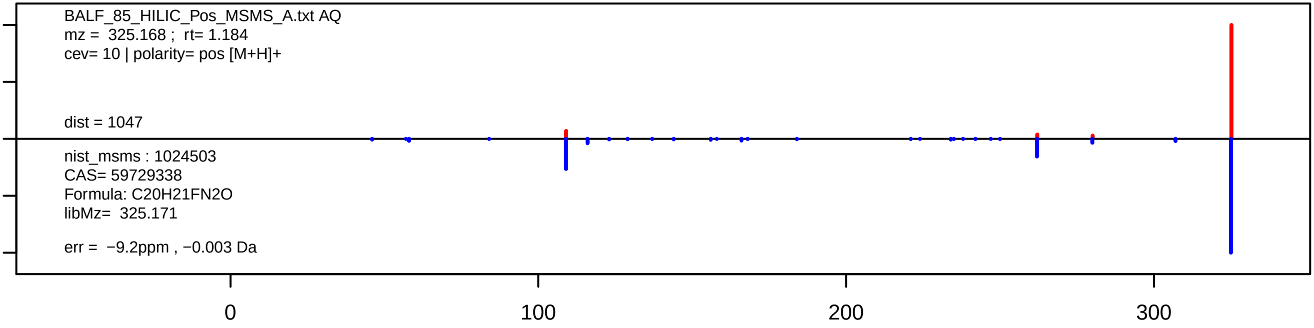

57 . Creatinine
Score=465 Dot=998 prob=98.7

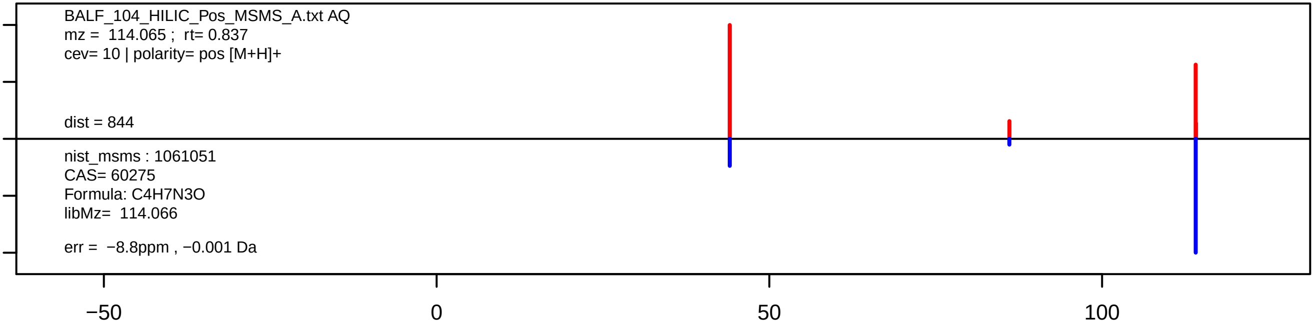

58 . Cyclizine
Score=877 Dot=963 prob=51.4

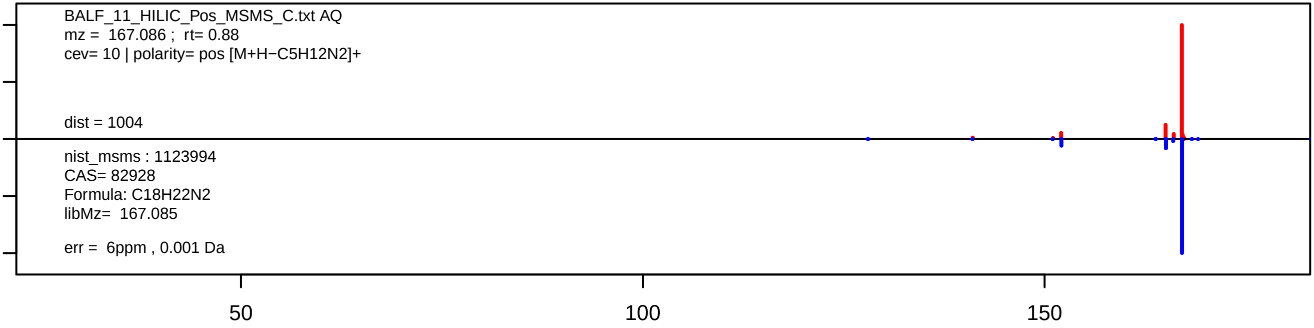

59 . Cyclohexylamine
Score=629 Dot=964 prob=46.4

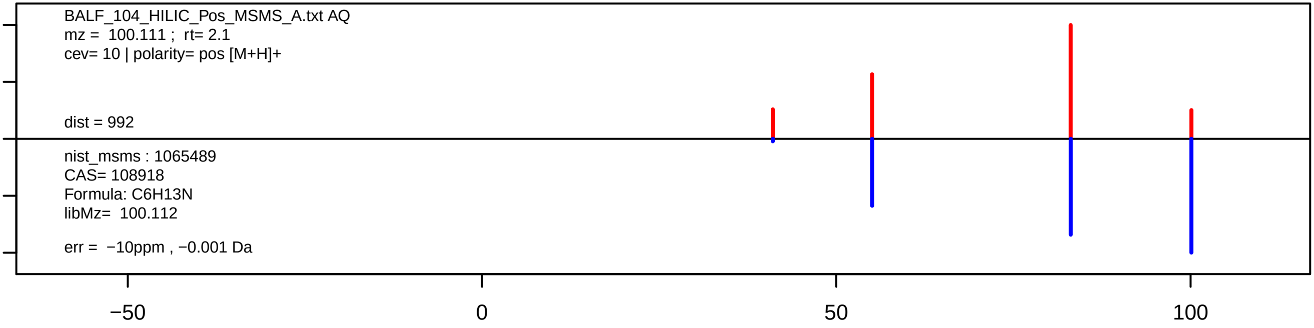

60 . D−(+)−Galactose
Score=407 Dot=811 prob=83.5

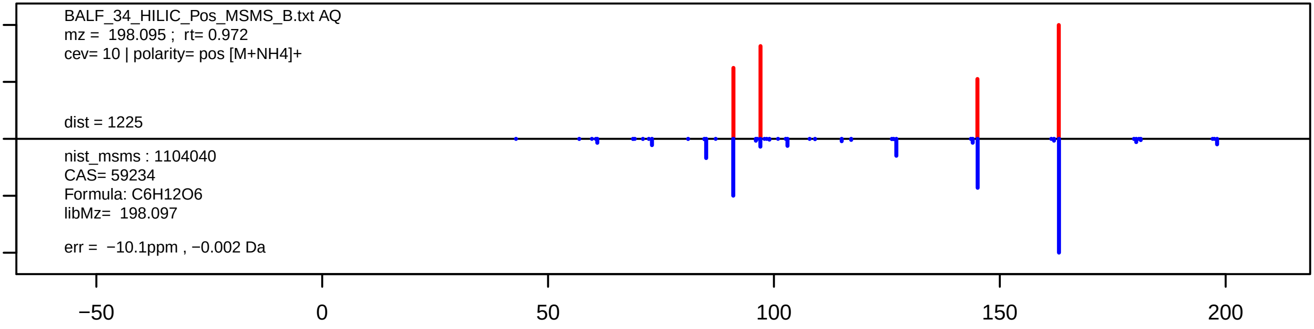

61 . D−Fructose
Score=667 Dot=864 prob=66.3

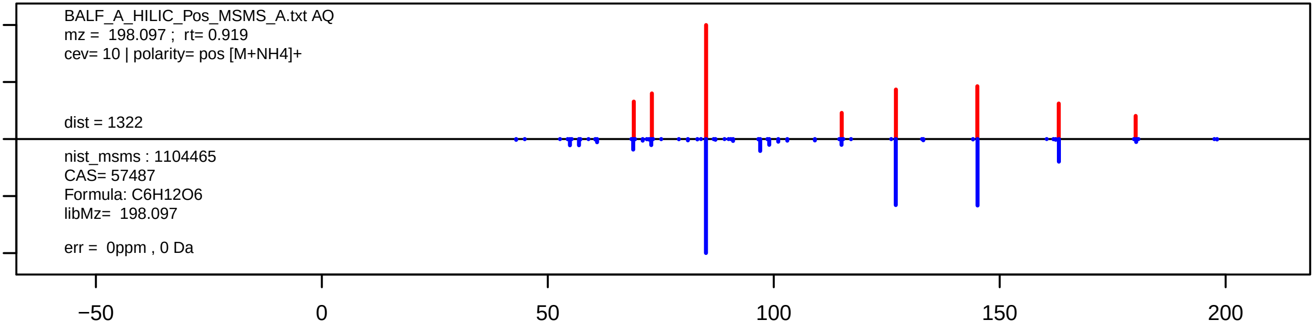

62 . D−Ornithine
Score=845 Dot=999 prob=58.8

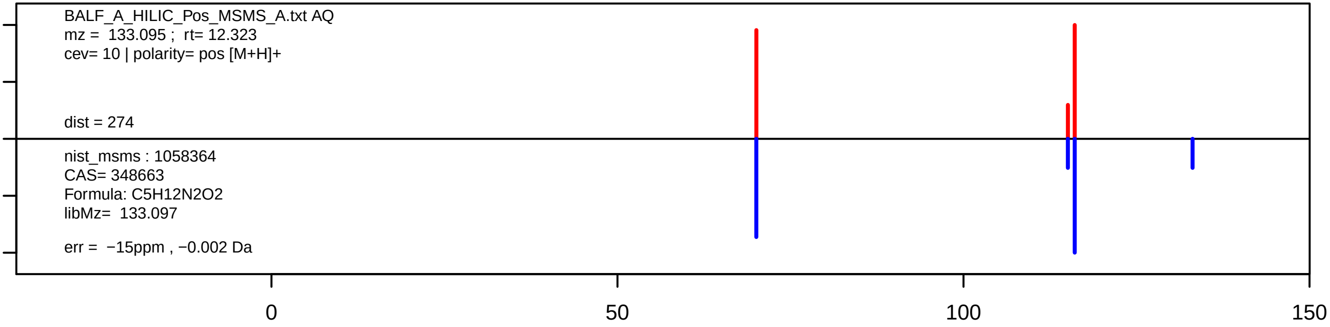

63 . D−Pipecolinic acid
Score=370 Dot=876 prob=30.9

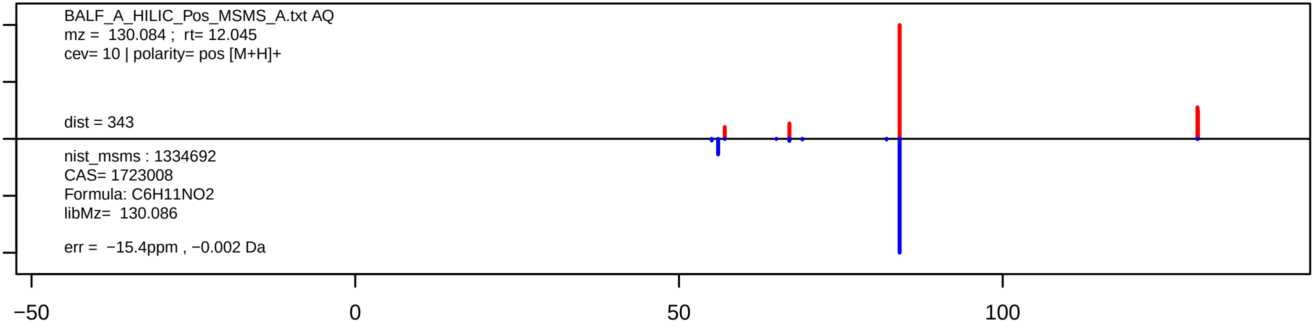

64 . Desalkylebastine
Score=549 Dot=997 prob=38.8

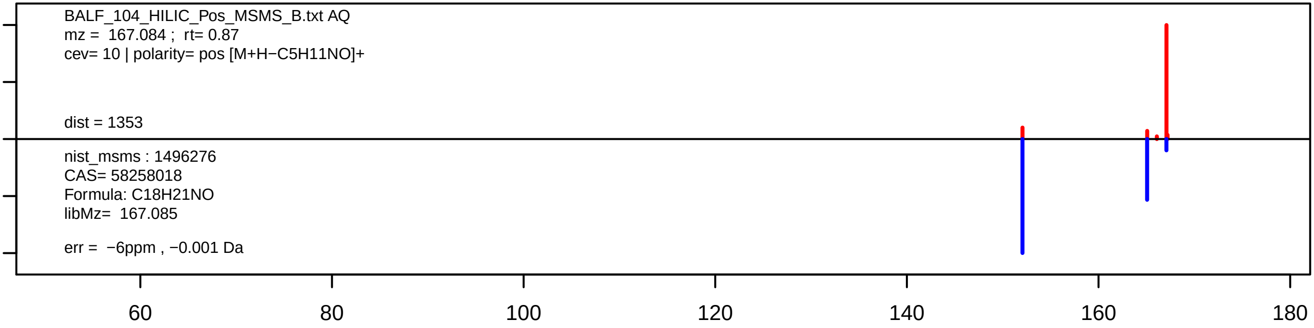

65 . Dextromethorphan
Score=249 Dot=863 prob=69.3

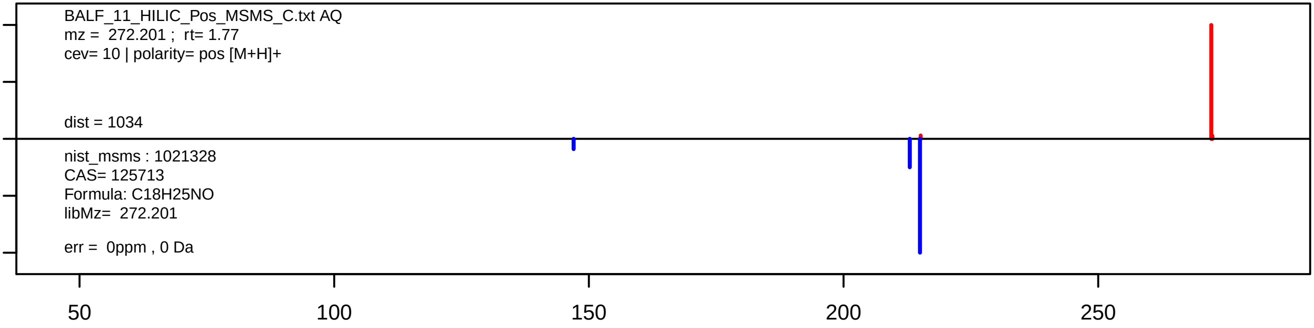

66 . Di(3,7−dimethyl−1−octyl) phthalate
Score=792 Dot=897 prob=91

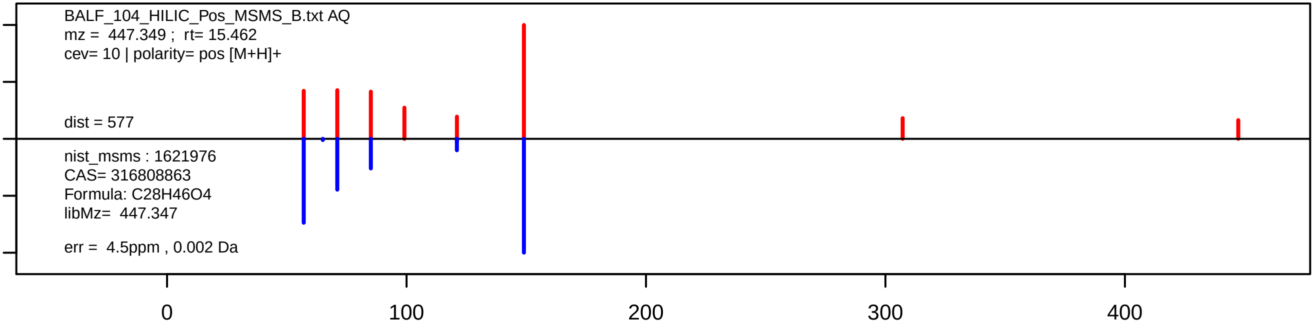

67 .   Dibenzylamine
Score=361 Dot=946 prob=98.5

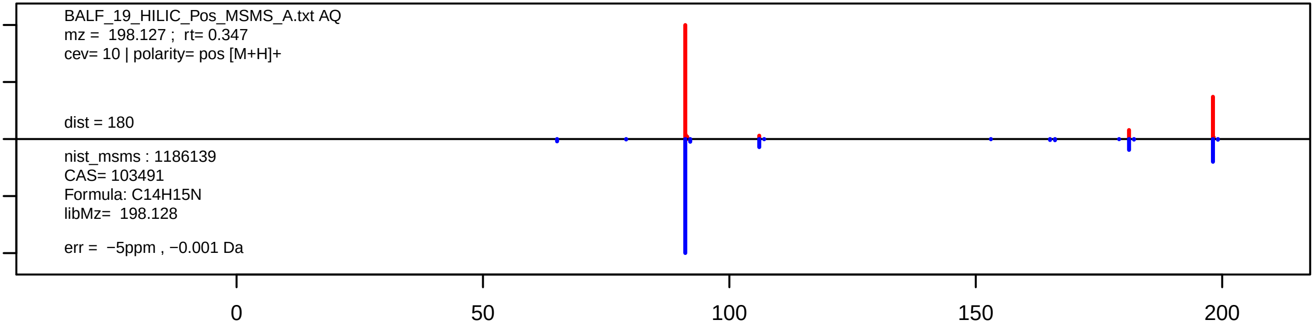

68 .   Dicyclohexylamine
Score=570 Dot=920 prob=51.5

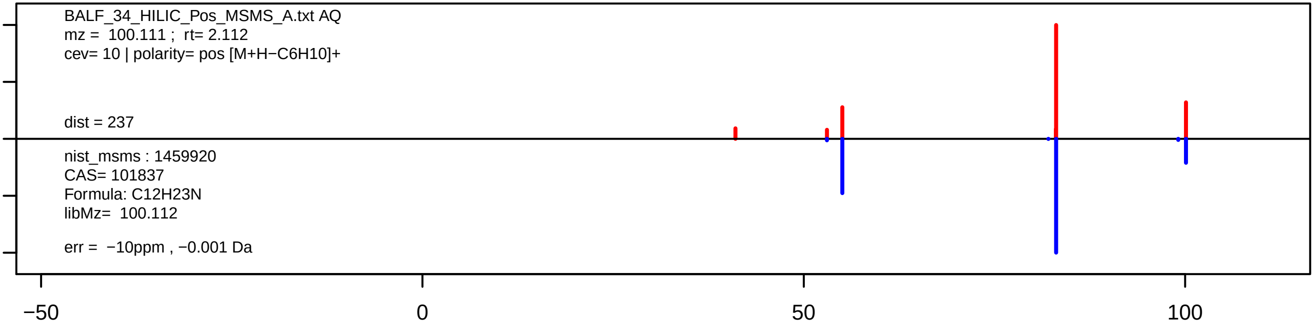

69 .   Diethanolamine
Score=761 Dot=941 prob=99

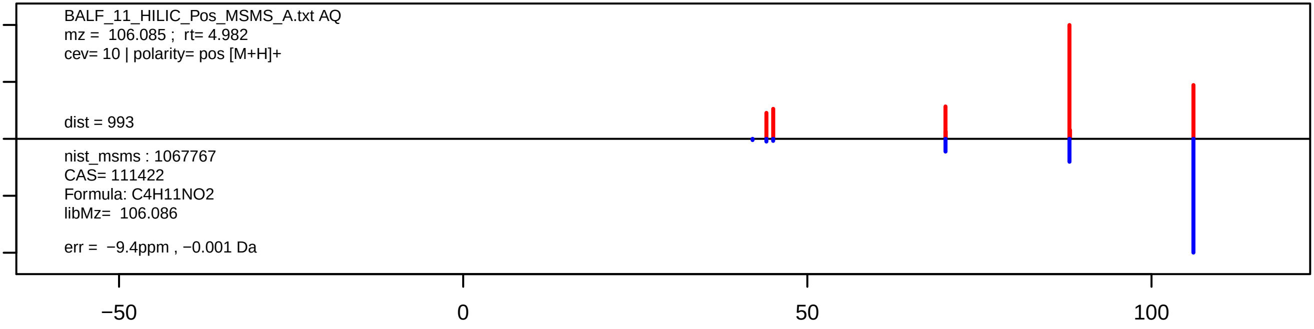

70 . Diisooctyl phthalate
Score=865 Dot=940 prob=74.1

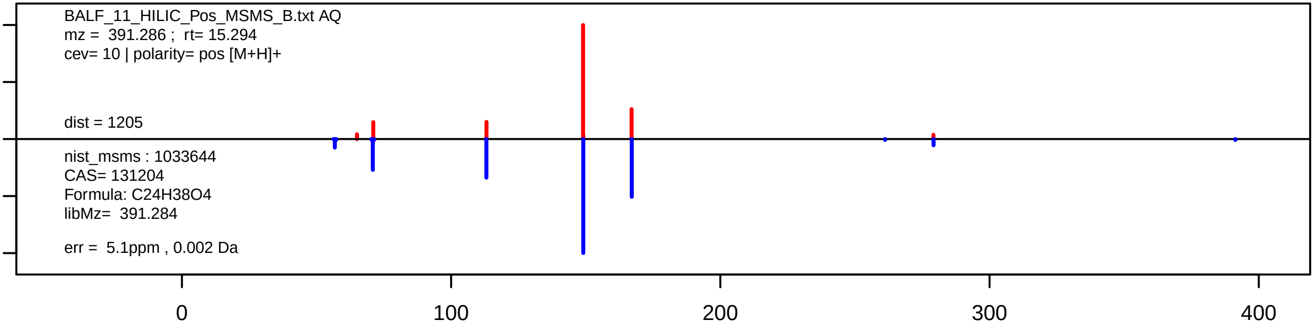

71 . Dioctyl phthalate
Score=927 Dot=970 prob=91.7

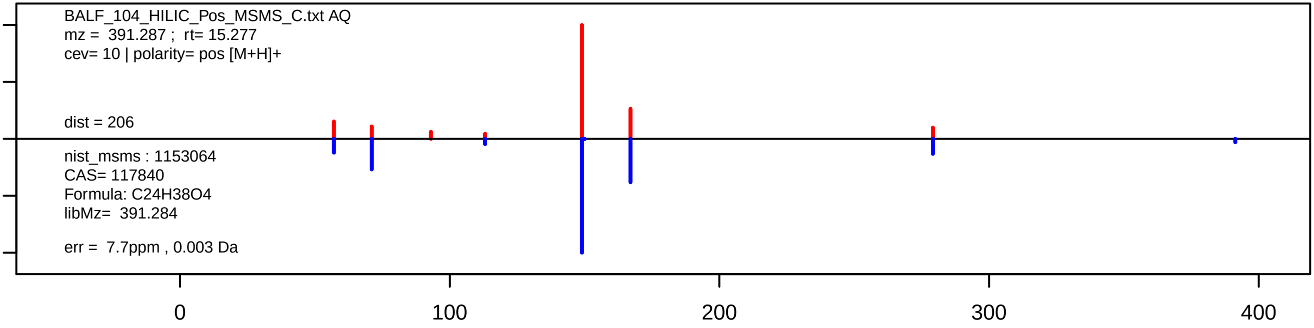

72 . Diphenhydramine
Score=371 Dot=963 prob=98.6

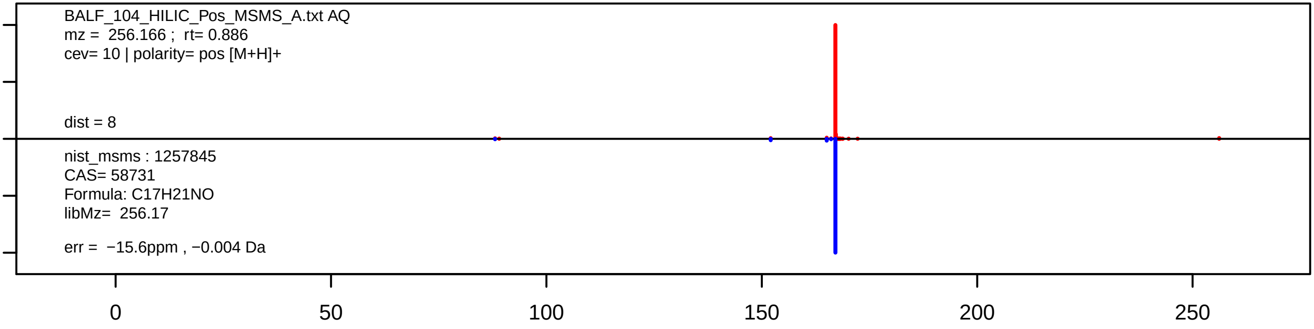

73 . DL−Arginine
Score=388 Dot=978 prob=25.1

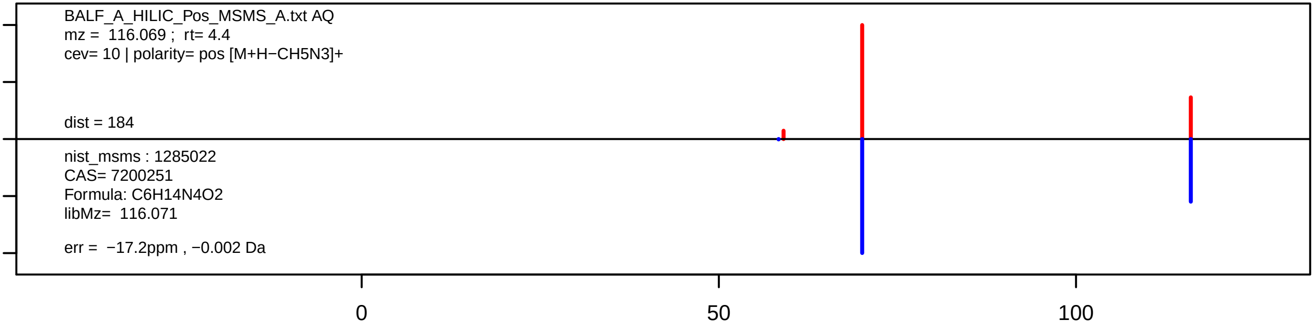

74 . DL−Carnitine
Score=592 Dot=897 prob=86.9

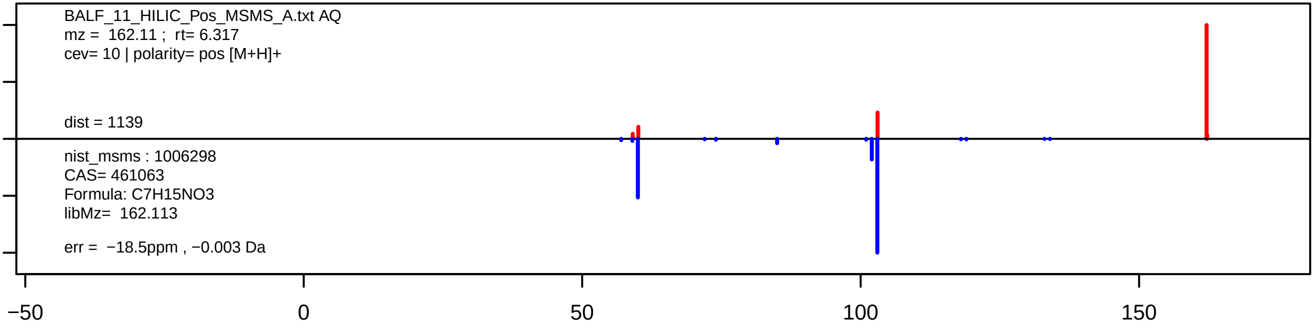

75 . DL−Leu−DL−Val
Score=680 Dot=984 prob=54.6

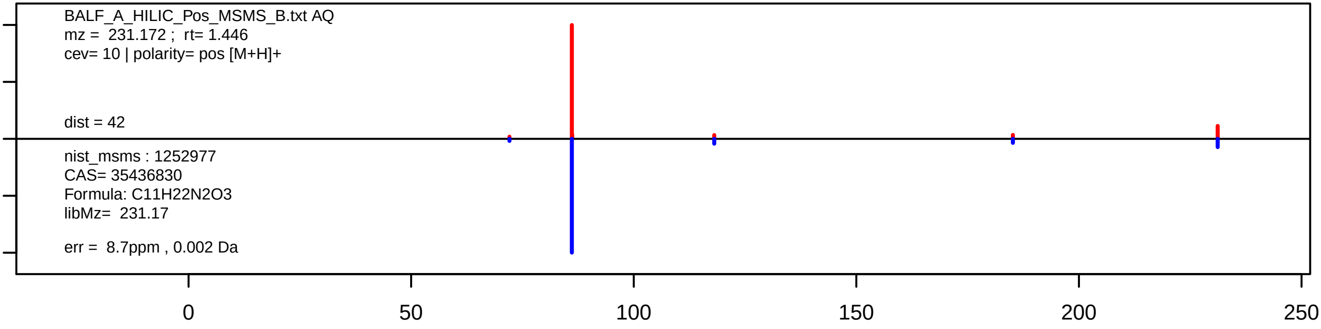

76 . DL−Ornithine
Score=658 Dot=999 prob=56.3

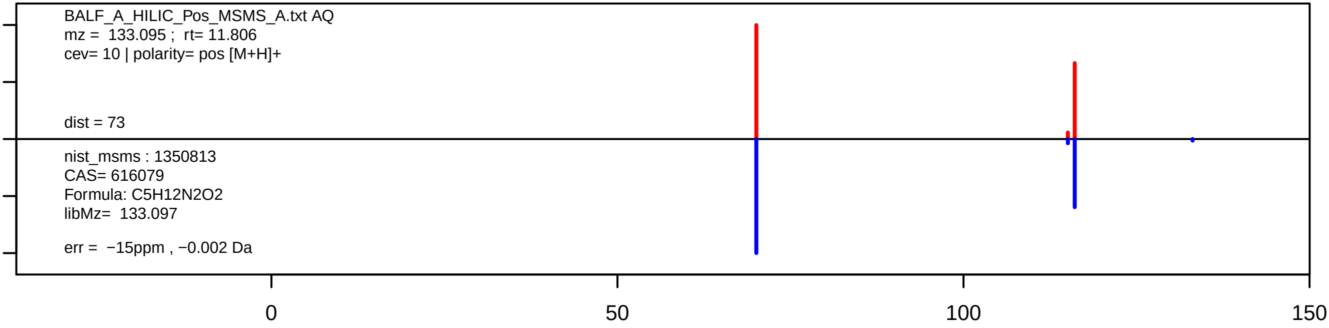

77 . DL−Phenylalanine
Score=377 Dot=955 prob=74

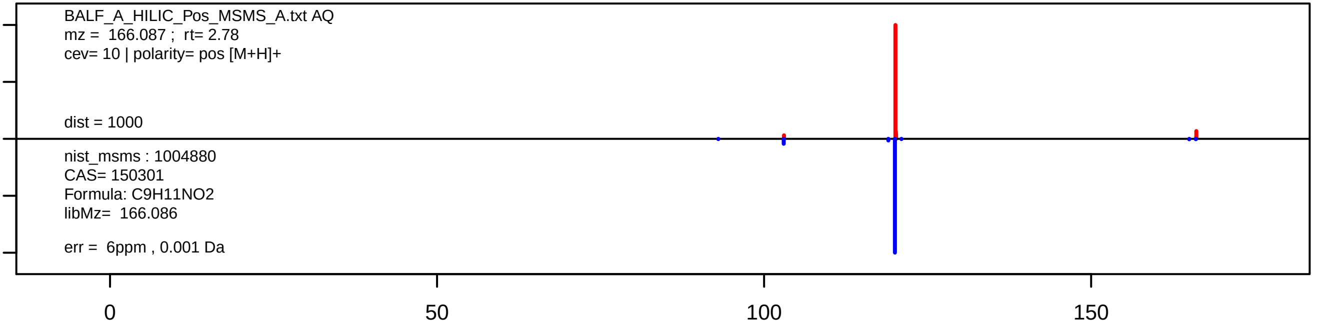

78 . Dyphylline
Score=301 Dot=953 prob=84.7

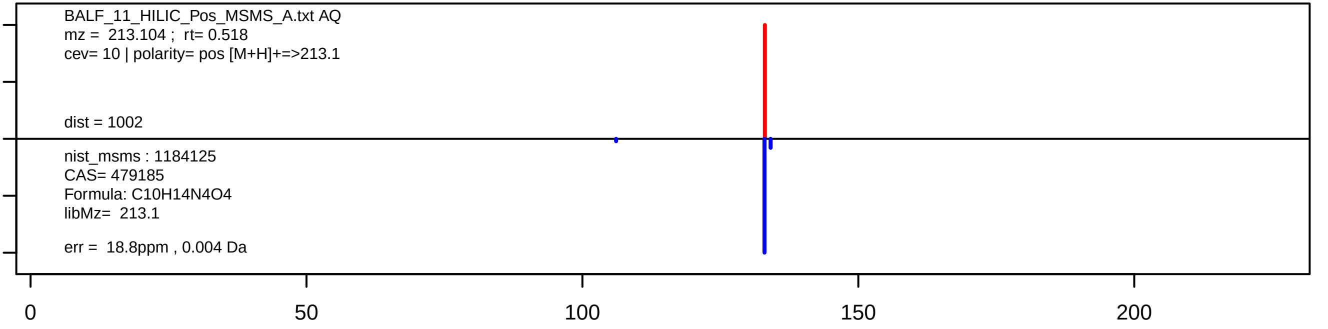

79 . Epoxiconazole
Score=176 Dot=803 prob=82.7

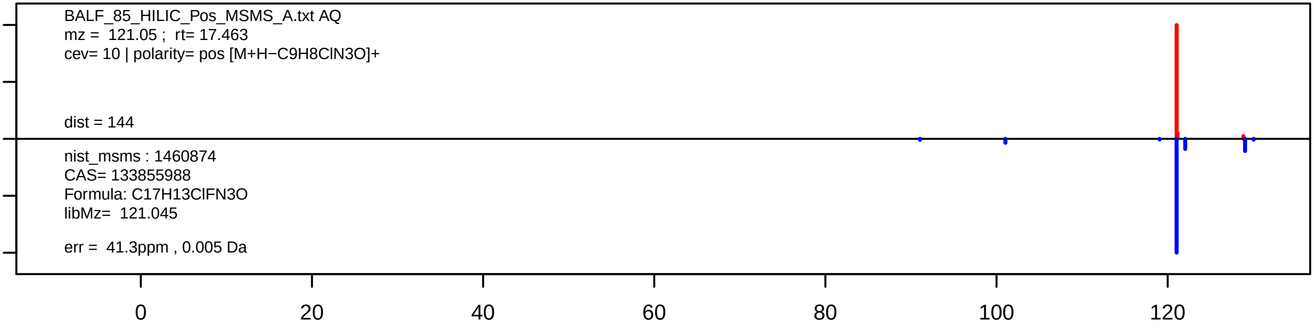

80 . Fluoxetine
Score=355 Dot=973 prob=98.6

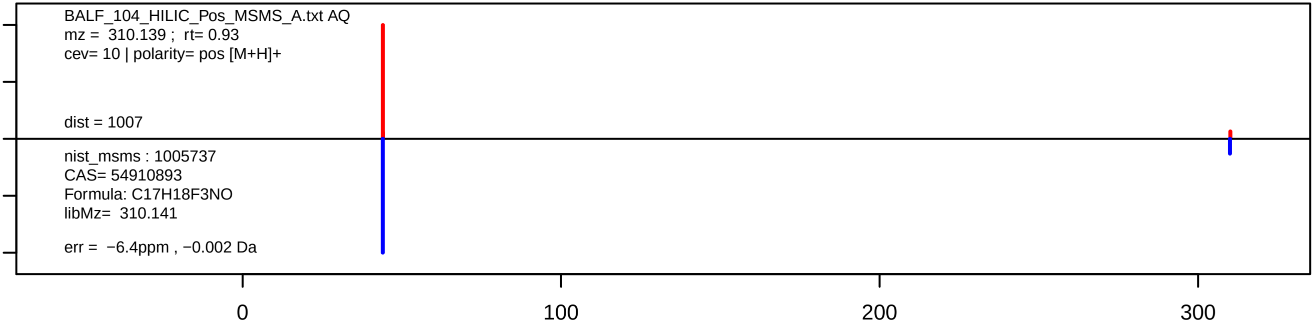

81 . Gabapentin
Score=627 Dot=930 prob=94

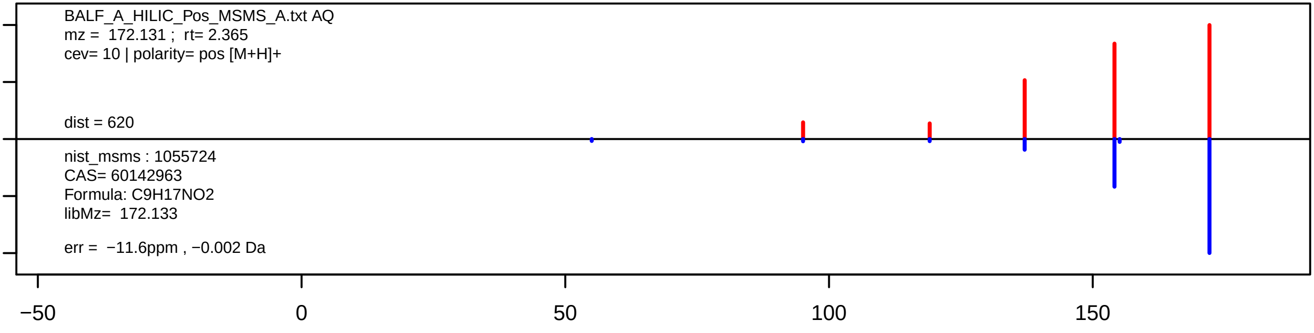

82 . Geranyl pyrophosphate
Score=251 Dot=968 prob=95.8

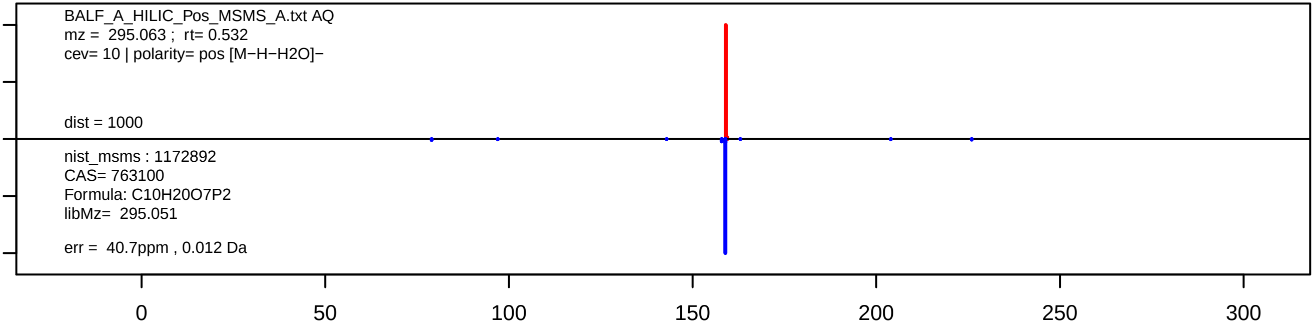

83 . Glycerophosphocholine
Score=354 Dot=975 prob=16.7

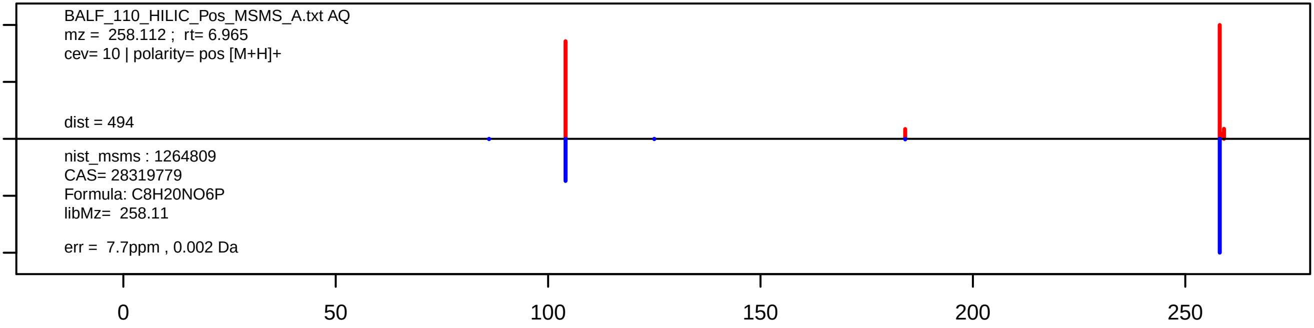

84 . Glycyl−L−norleucine
Score=354 Dot=960 prob=16.3

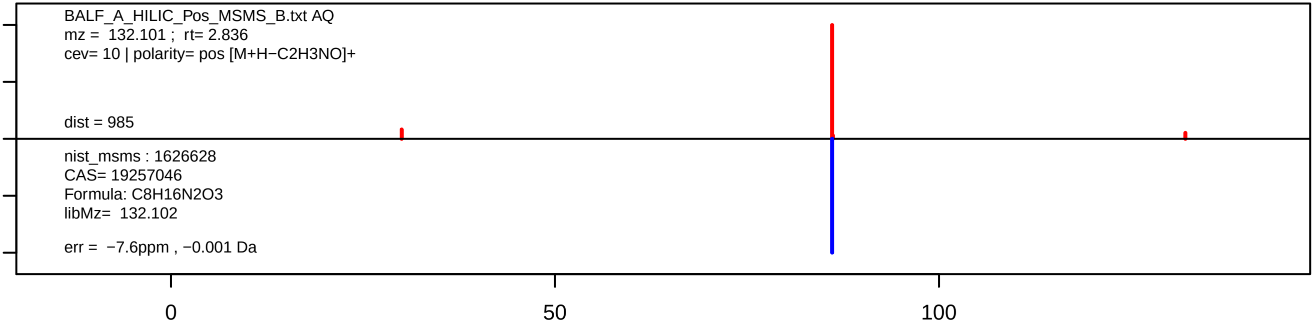

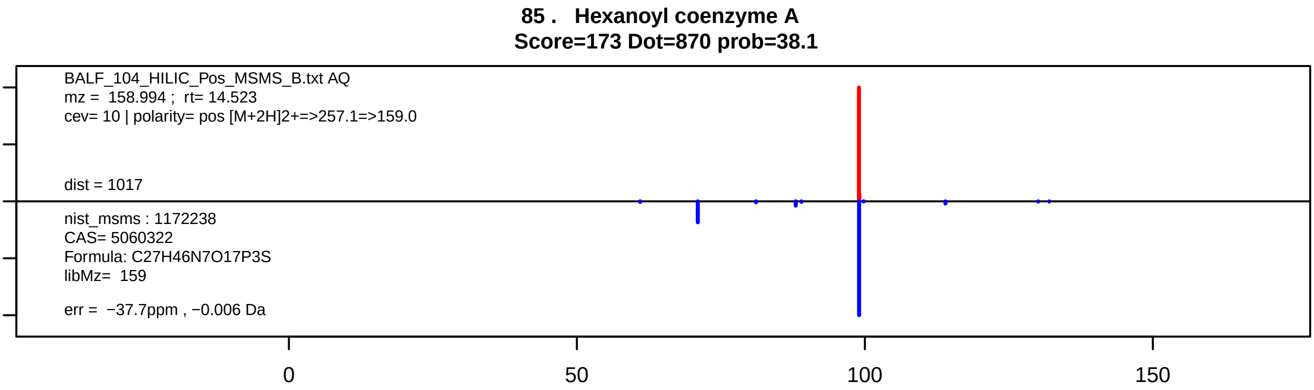
85 . Hexanoyl coenzyme A
Score=173 Dot=870 prob=38.1
BALF_104_HILIC_Pos_MSMS_B.txt AQ
mz = 158.994 ; rt= 14.523
cev= 10 | polarity= pos [M+2H]2+=>257.1=>159.0
dist = 1017
nist_msms : 1172238
CAS= 5060322
Formula: C27H46N7O17P3S
libMz= 159
err = −37.7ppm , −0.006 Da
0
50
100
150

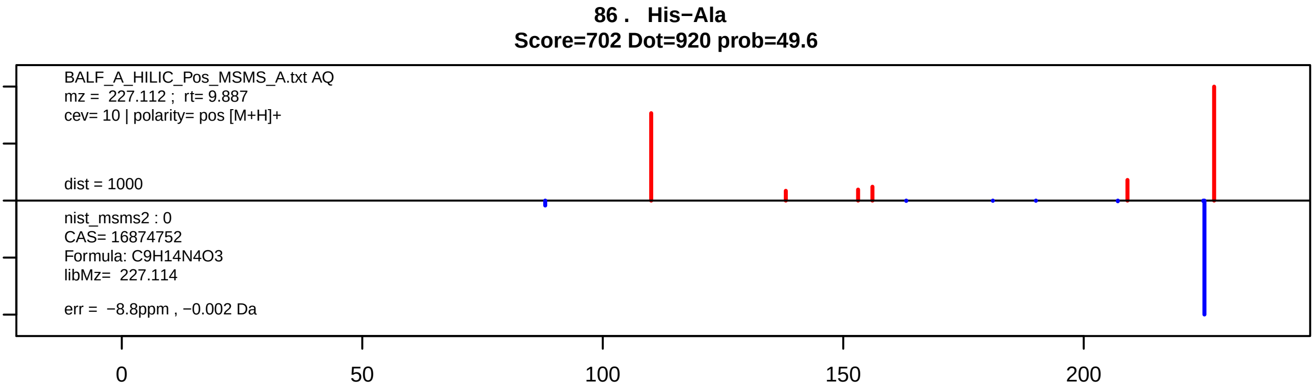
86 . His−Ala
Score=702 Dot=920 prob=49.6
BALF_A_HILIC_Pos_MSMS_A.txt AQ
mz = 227.112 ; rt= 9.887
cev= 10 | polarity= pos [M+H]+
dist = 1000
nist_msms2 : 0
CAS= 16874752
Formula: C9H14N4O3
libMz= 227.114
err = −8.8ppm , −0.002 Da
0
50
100
150
200

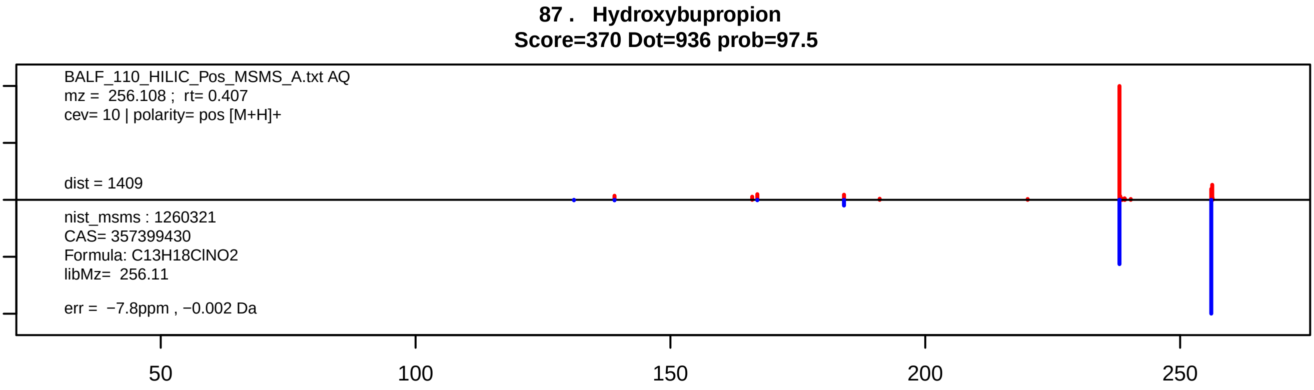
87 . Hydroxybupropion
Score=370 Dot=936 prob=97.5
BALF_110_HILIC_Pos_MSMS_A.txt AQ
mz = 256.108 ; rt= 0.407
cev= 10 | polarity= pos [M+H]+
dist = 1409
nist_msms : 1260321
CAS= 357399430
Formula: C13H18ClNO2
libMz= 256.11
err = −7.8ppm , −0.002 Da
50
100
150
200
250

88 . Hydroxyzine
Score=275 Dot=965 prob=95.8

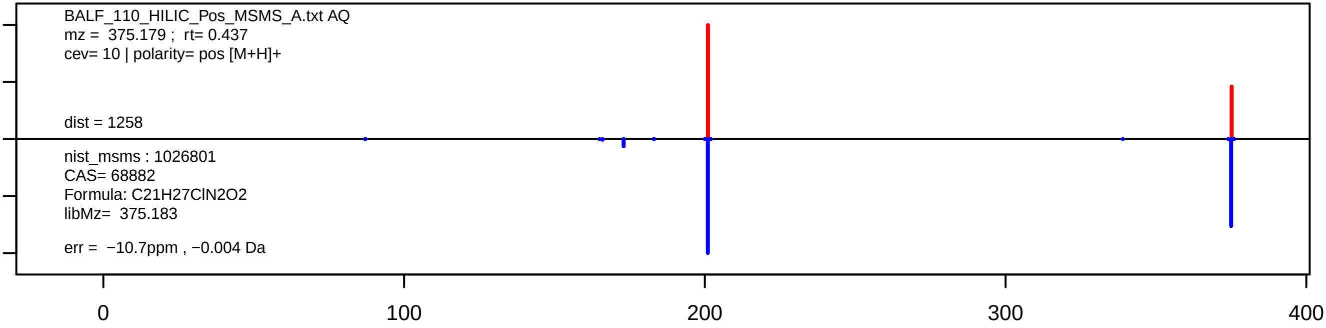

89 . Hypoxanthine
Score=810 Dot=926 prob=76.3

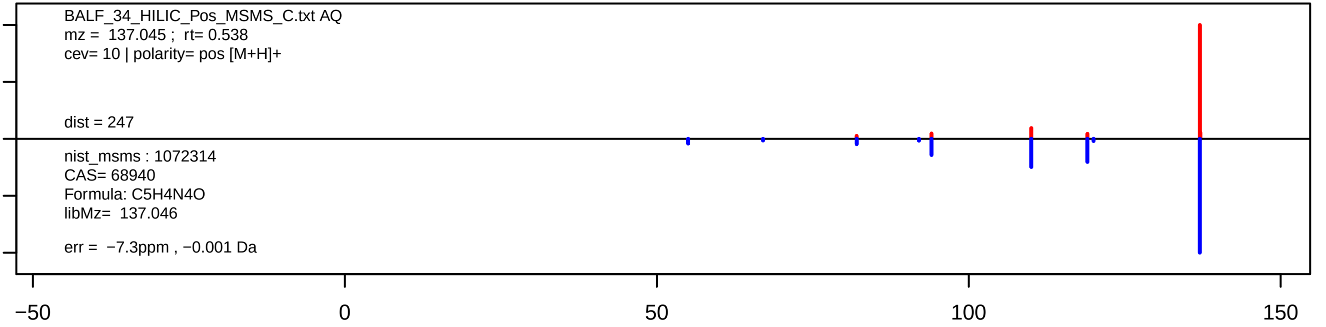

90 . Ile−Ala
Score=400 Dot=999 prob=23.1

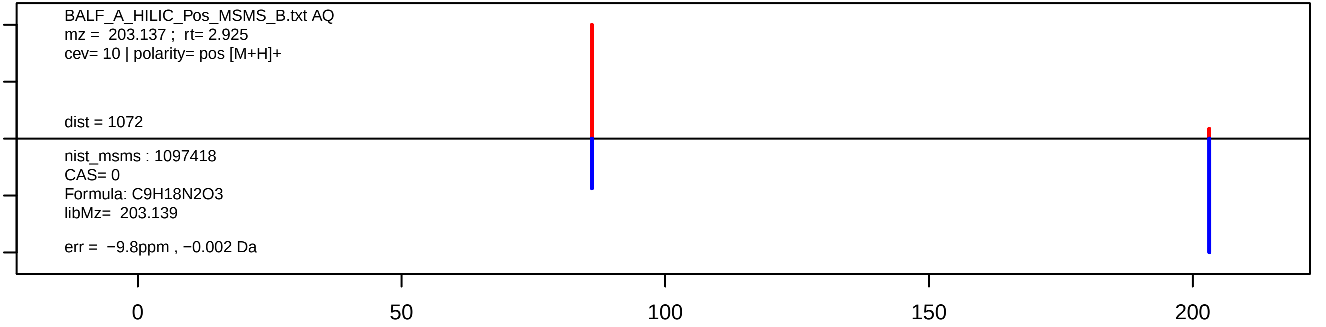

91 . Ile−Arg
Score=854 Dot=880 prob=98.8

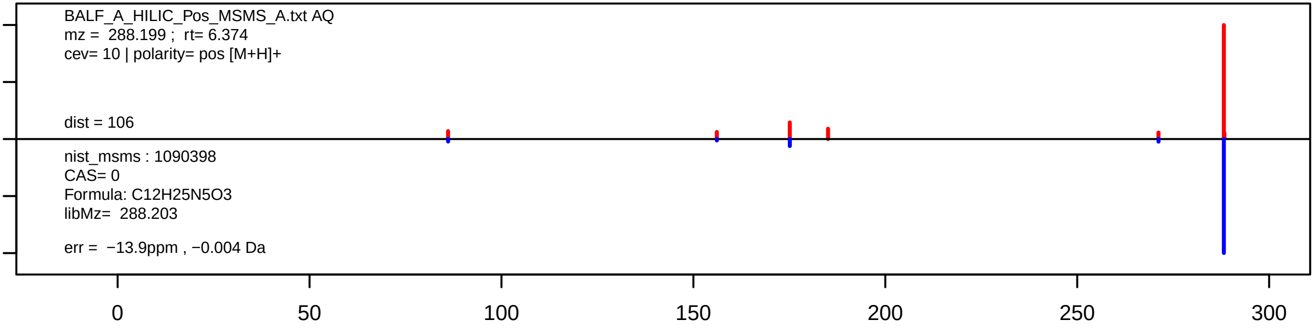

92 . Ile−Ile
Score=619 Dot=980 prob=79.8

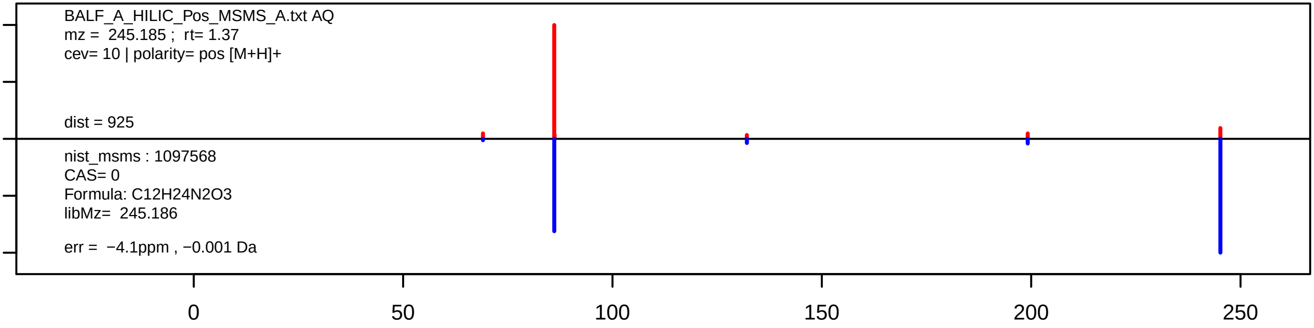

93 . Ile−Leu
Score=378 Dot=954 prob=25.9

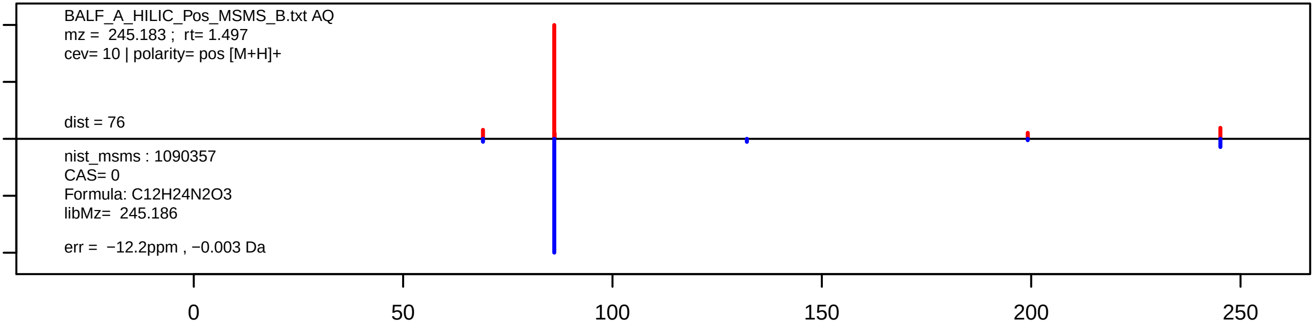

94 . Ile−Thr
Score=433 Dot=905 prob=91.9

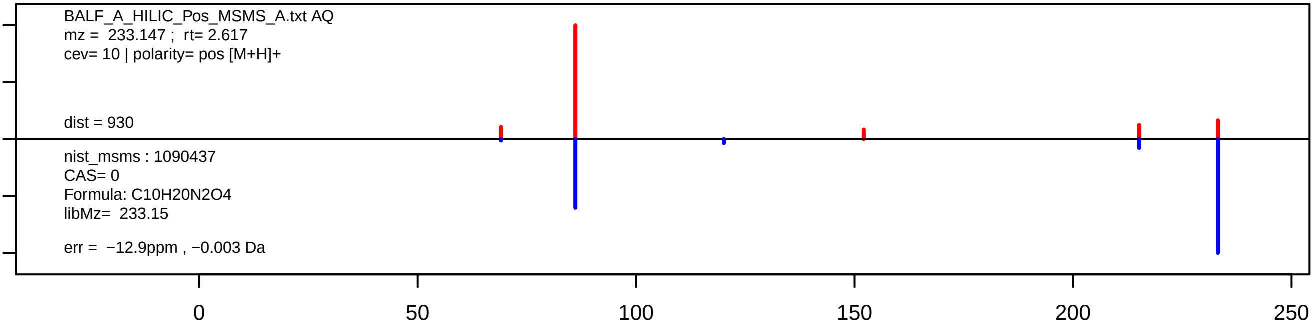

95 . Ile−Val
Score=361 Dot=960 prob=79.6

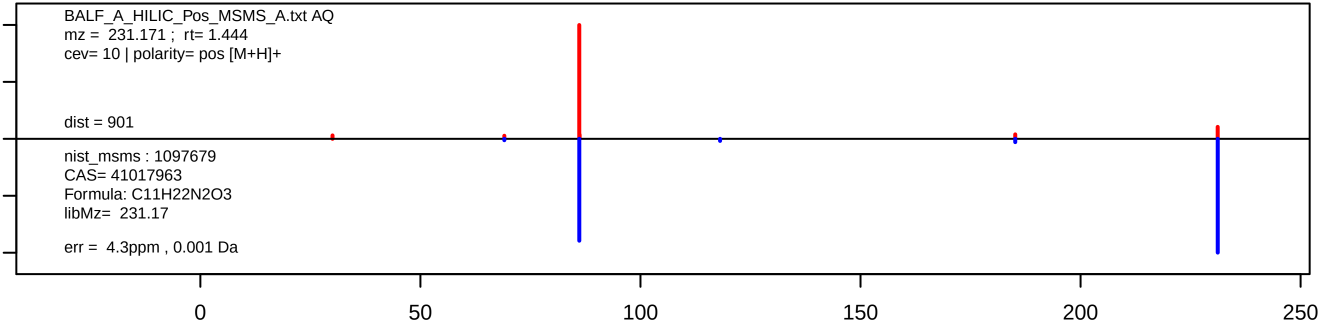

96 . Imipramine
Score=317 Dot=934 prob=8.8

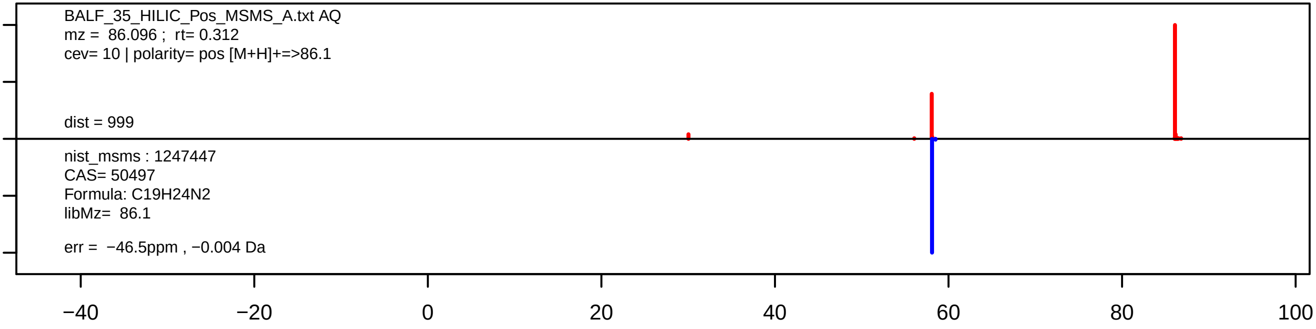

97 . Inosine
Score=341 Dot=947 prob=48.8

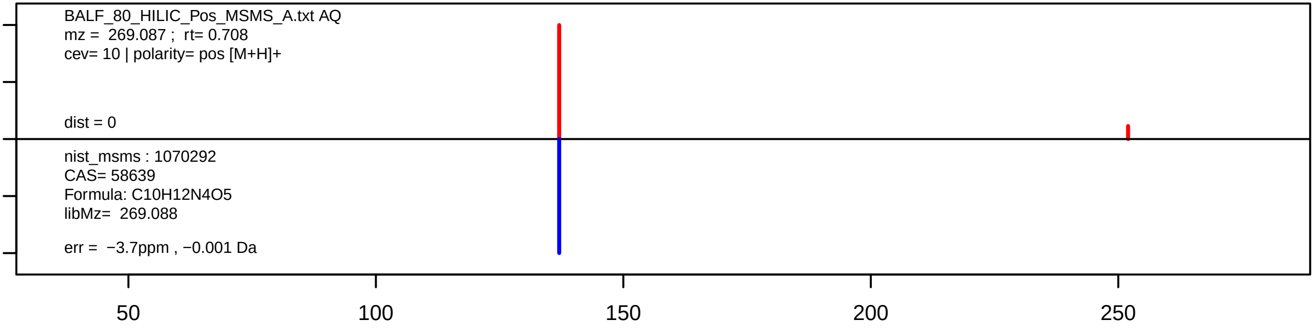

98 . L−(+)−Gulose
Score=346 Dot=817 prob=67.2

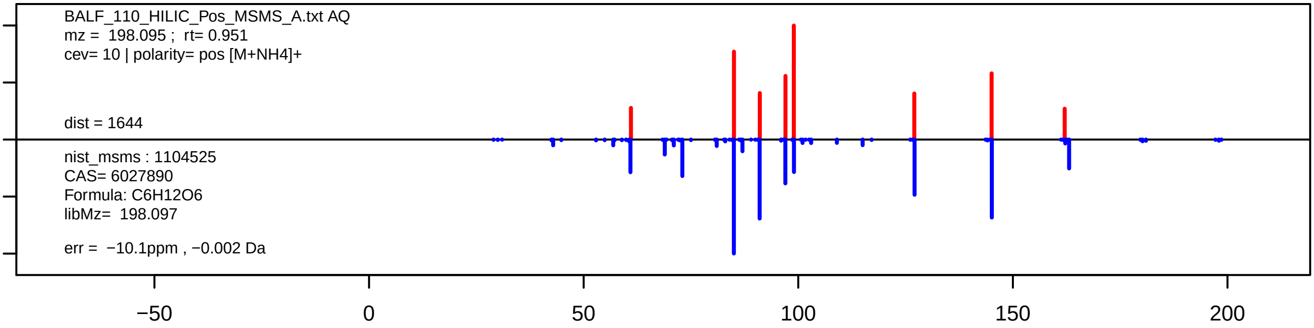

99 . L−Alanyl−L−norleucine
Score=432 Dot=936 prob=72.1

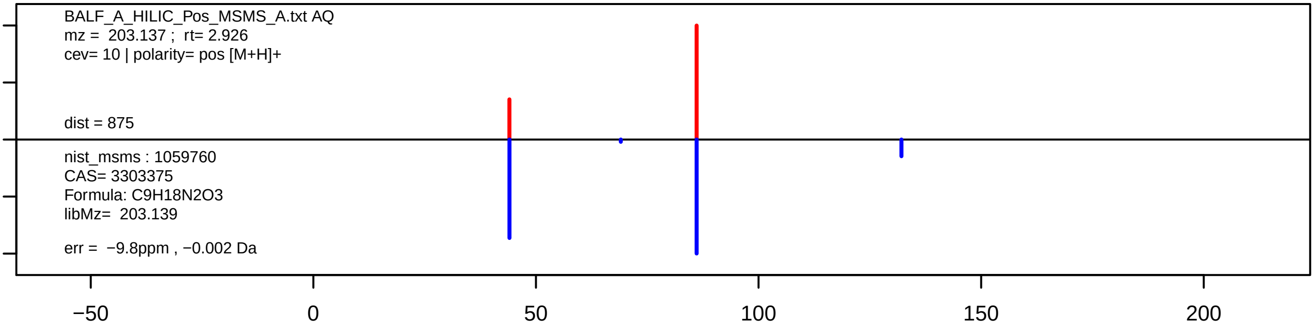

100 . L−Arginine
Score=921 Dot=963 prob=42.1

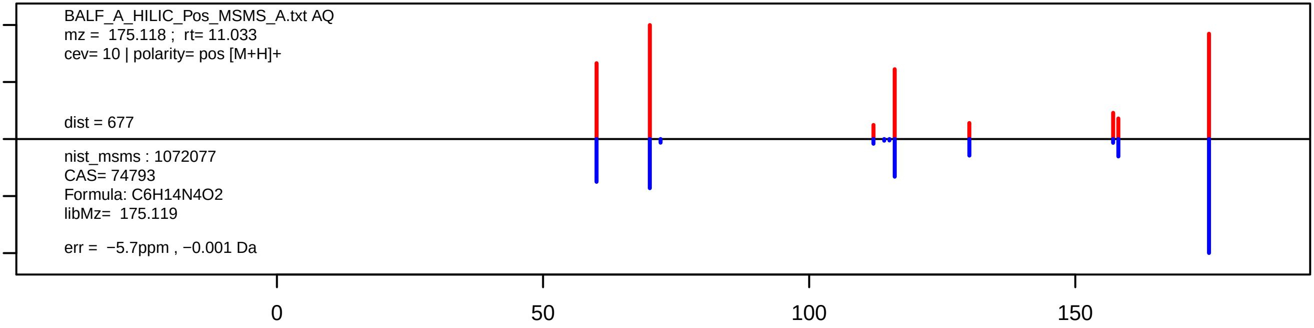

101 . L−Carnitine
Score=844 Dot=945 prob=91.9

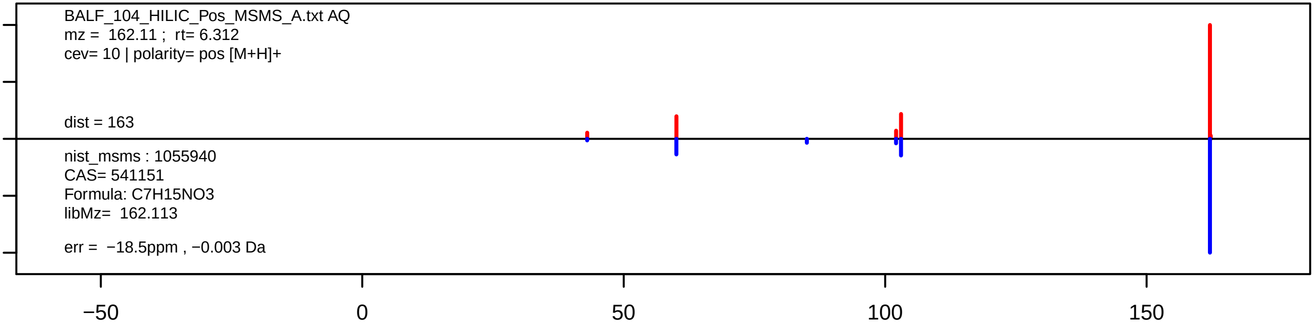

102 . L−Citrulline
Score=708 Dot=921 prob=98.8

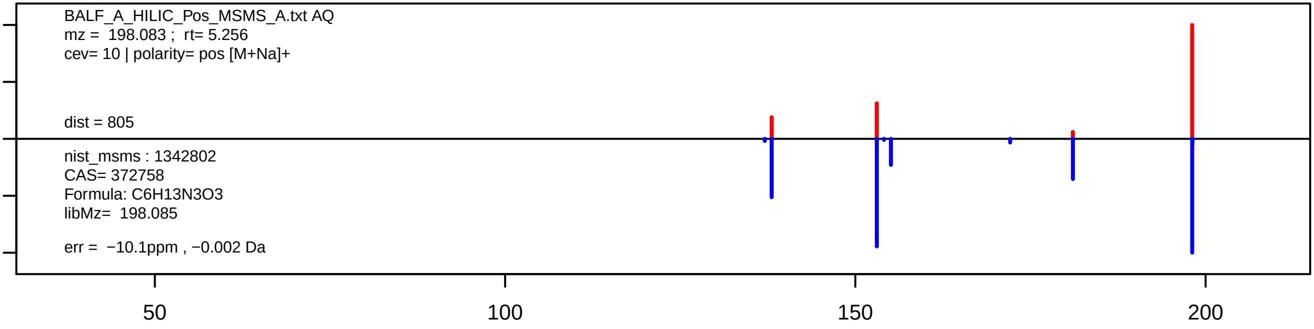

103 . L−Deprenyl
Score=377 Dot=981 prob=44.1

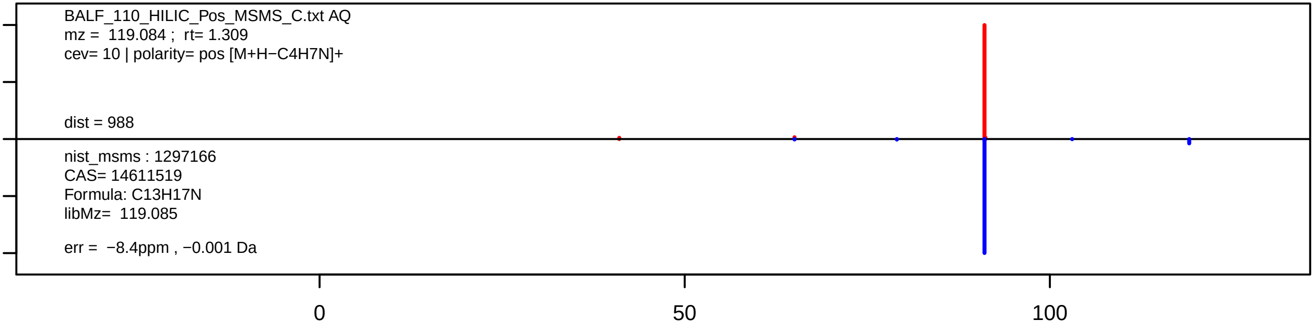

104 . L−Glutamic acid
Score=982 Dot=987 prob=58.2

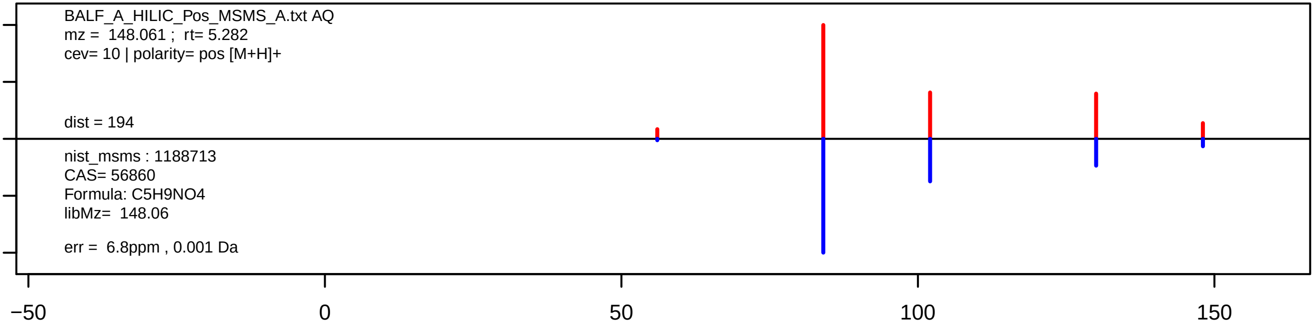

105 . L−Histidine
Score=400 Dot=999 prob=72.3

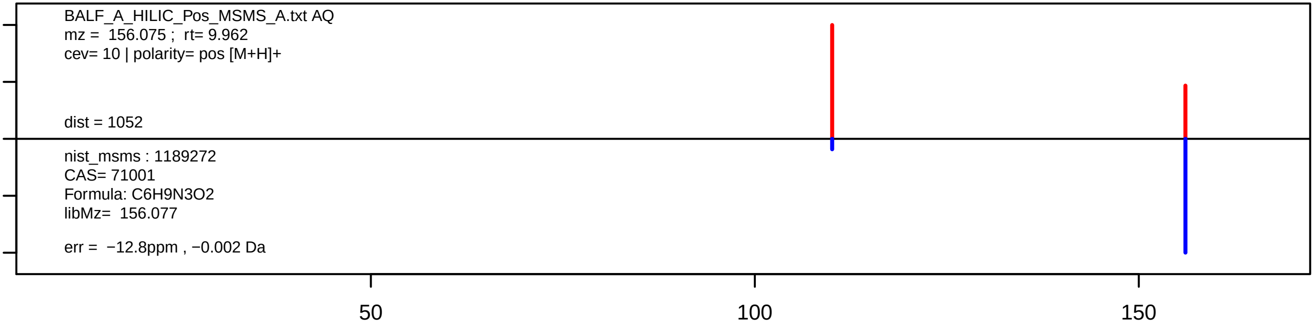

106 . L−Hydroxyarginine
Score=371 Dot=985 prob=11.4

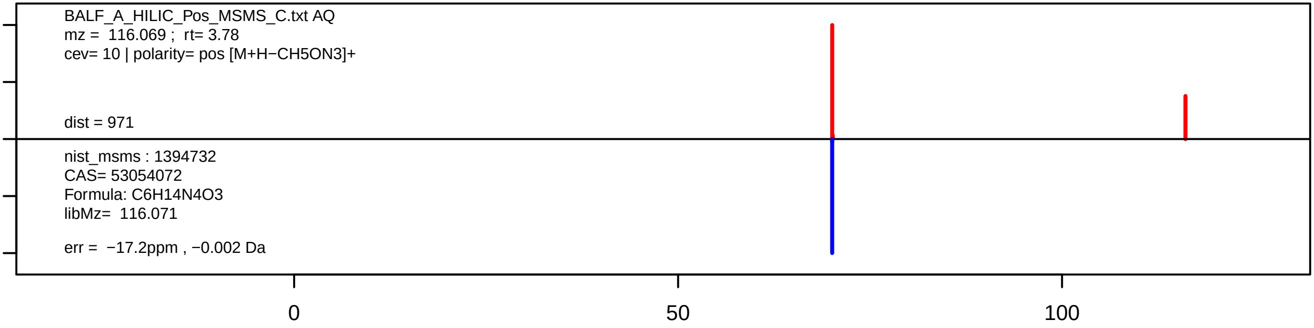

107 . L−Isoleucine
Score=388 Dot=957 prob=44.1

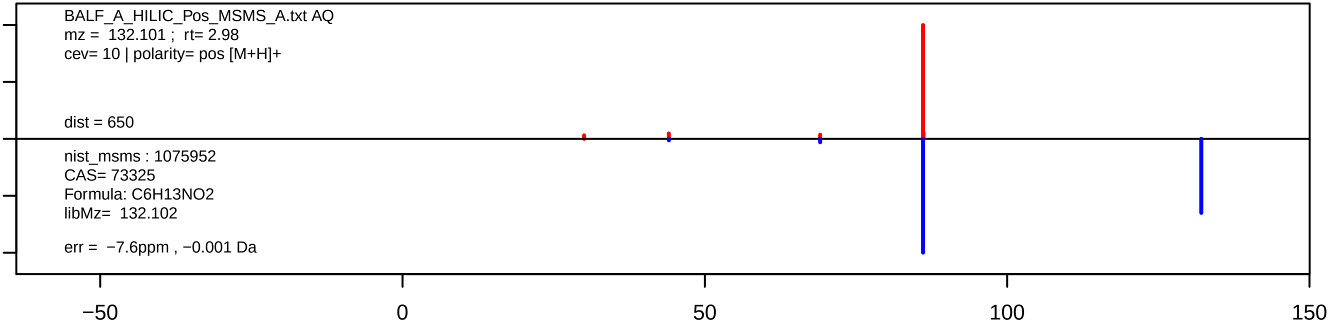

108 . L−Leucine
Score=507 Dot=970 prob=98.3

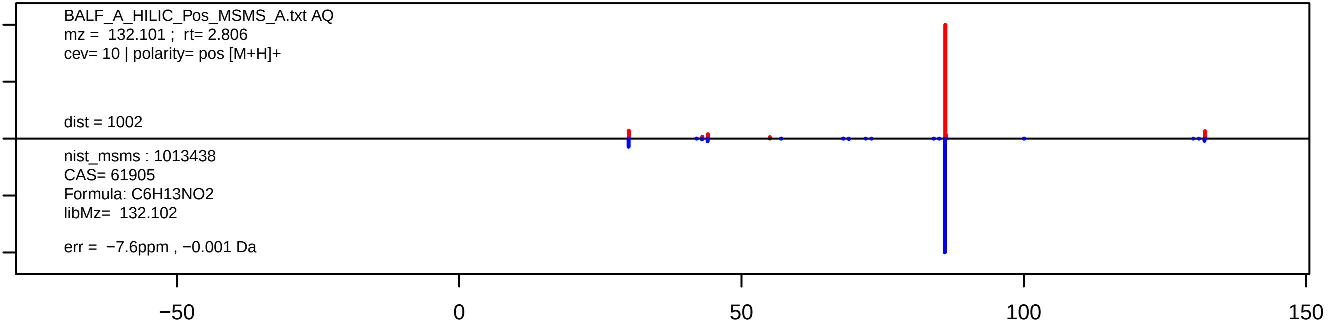

109 . L−Lysine
Score=532 Dot=890 prob=53.6

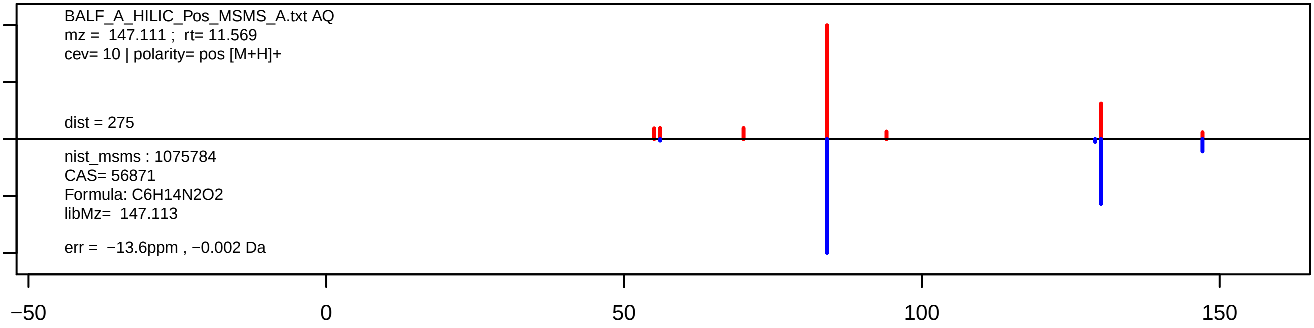

110 . L−Norvaline
Score=558 Dot=997 prob=47

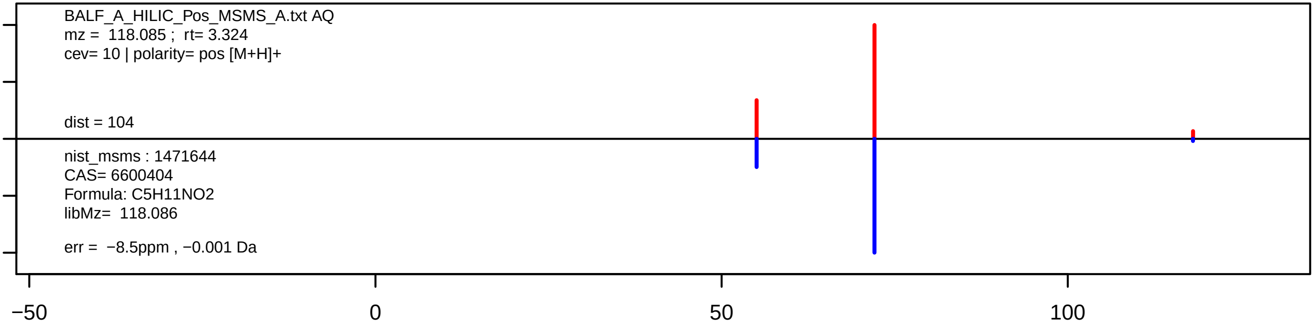

111 . L−Phenylalanine
Score=362 Dot=973 prob=57.9

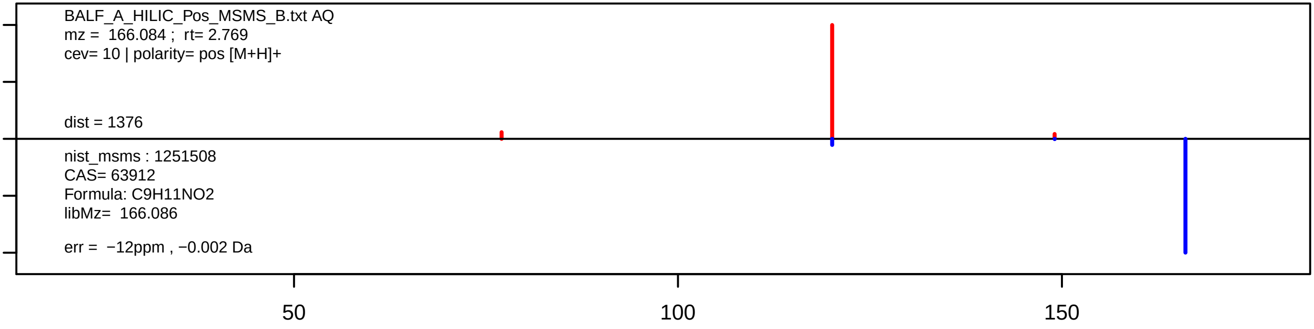

112 . L−Pipecolic acid
Score=400 Dot=999 prob=5.7

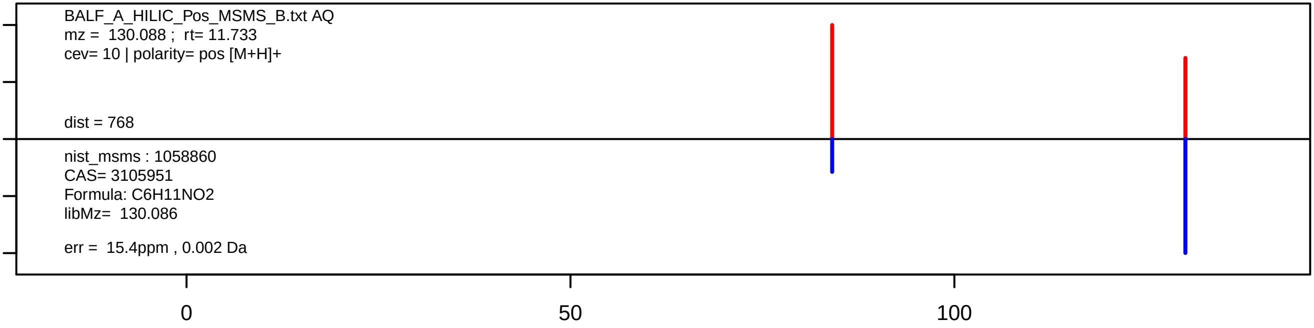

113 . L−Proline
Score=400 Dot=999 prob=7.2

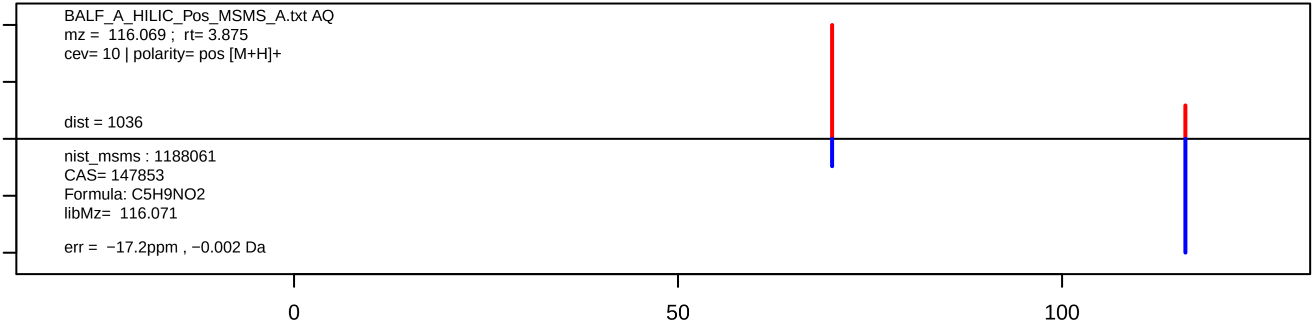

114 . L−Propionylcarnitine
Score=744 Dot=942 prob=99

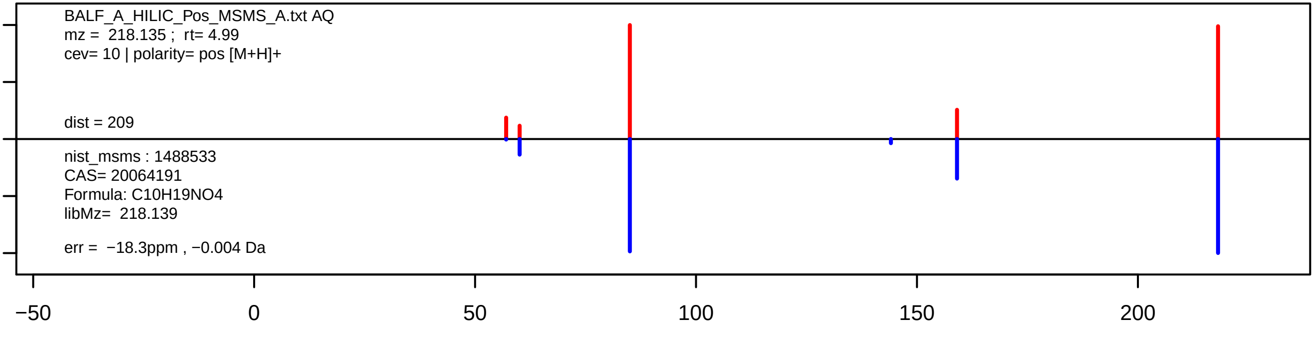

115 . L−Tyrosine
Score=609 Dot=824 prob=73.9

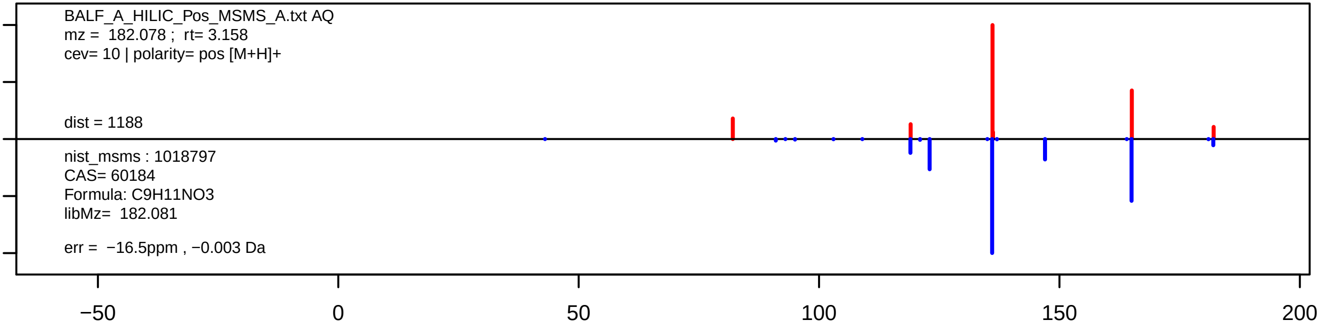

116 . L−Valine
Score=335 Dot=935 prob=15.5

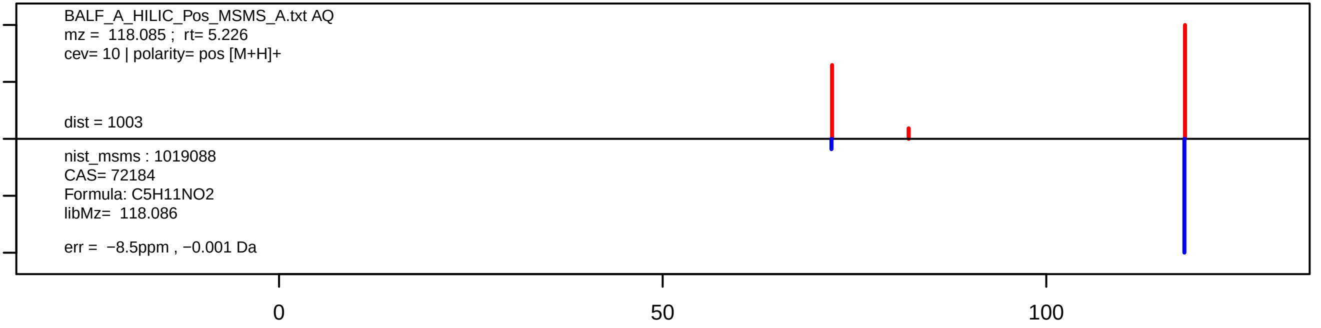

117 . Lauric acid diethanolamide
Score=547 Dot=991 prob=90.3

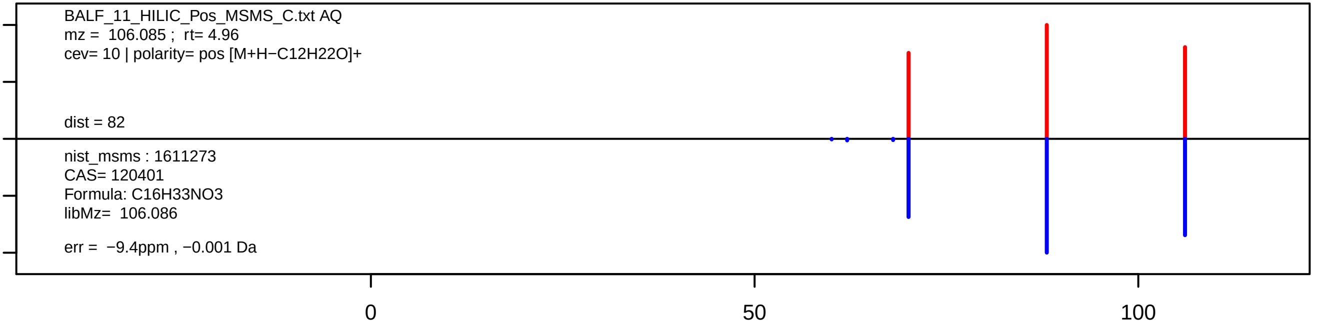

118 . Leu−Ala
Score=337 Dot=981 prob=22.3

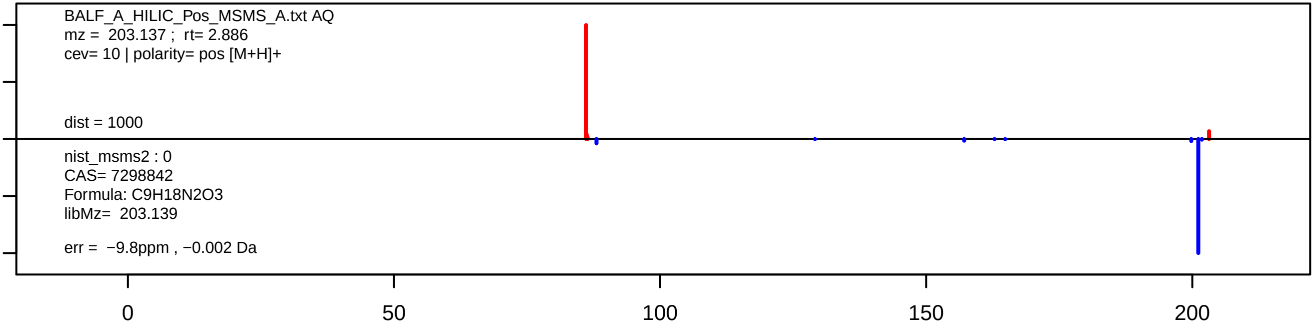

119 . Leu−Leu
Score=357 Dot=927 prob=40.5

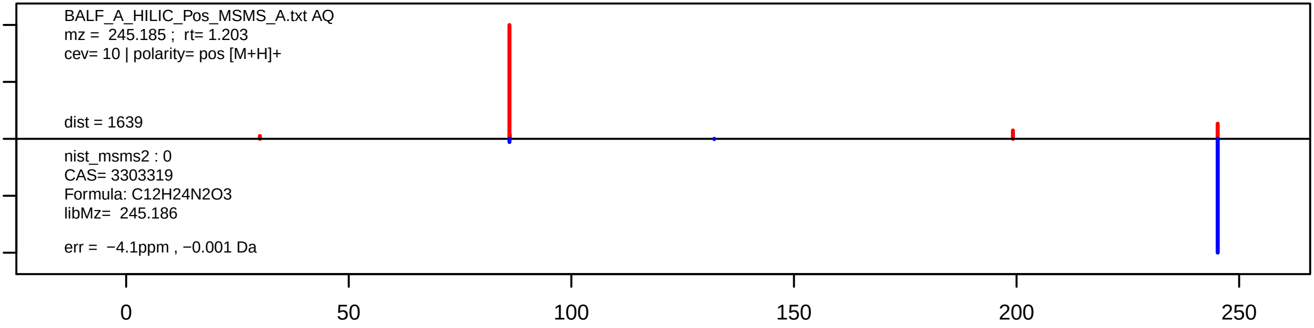

120 . Leu−Phe
Score=58 Dot=853 prob=34.6

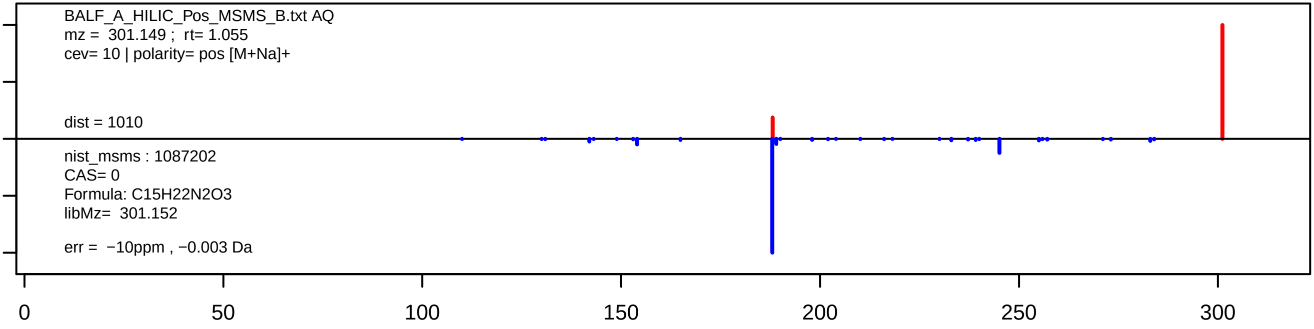

121 . Leu−Pro
Score=417 Dot=922 prob=39.2

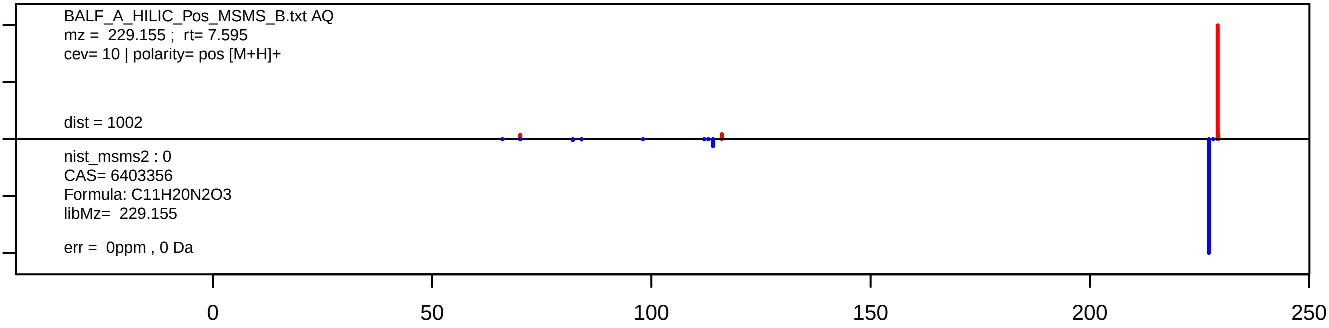

122 . Leu−Tyr
Score=392 Dot=808 prob=44.4

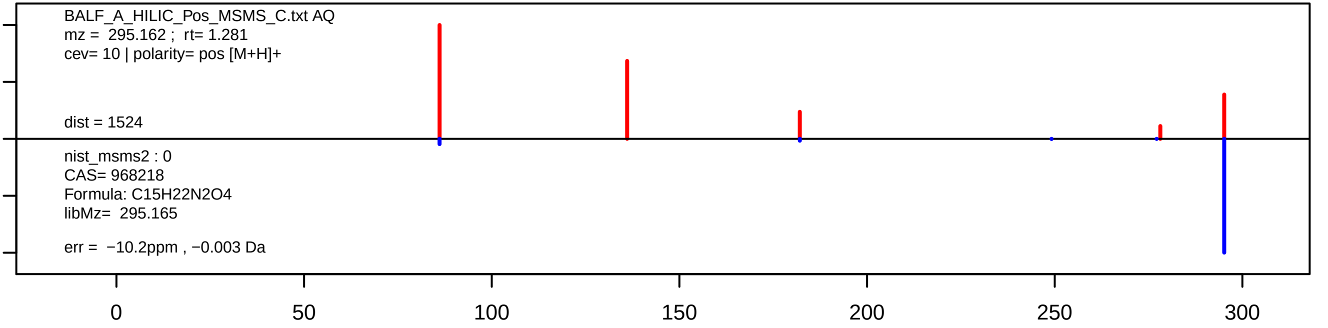

123 . Lidocaine
Score=400 Dot=999 prob=58.1

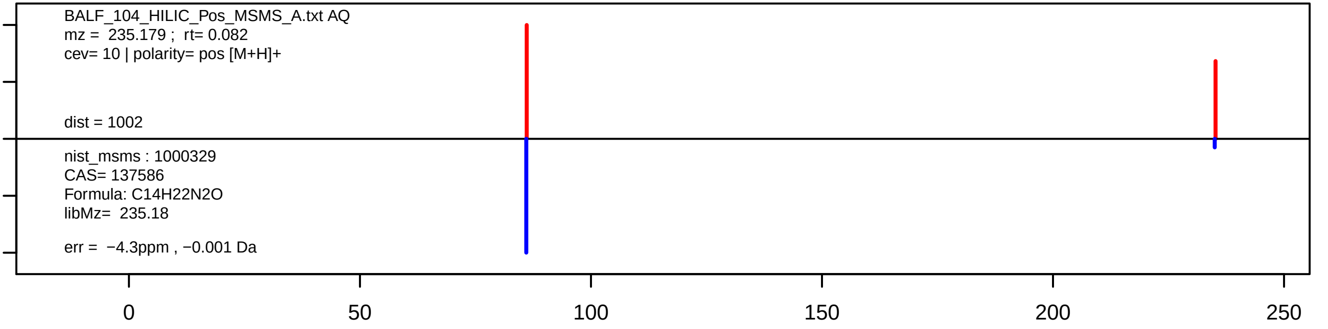

124 . Lys−Ala
Score=848 Dot=953 prob=49.5

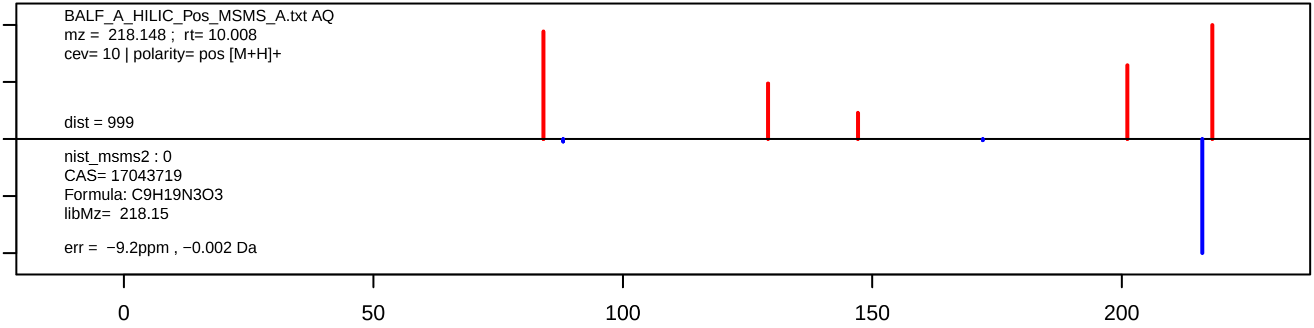

125 . Lys−Glu
Score=458 Dot=833 prob=41.5

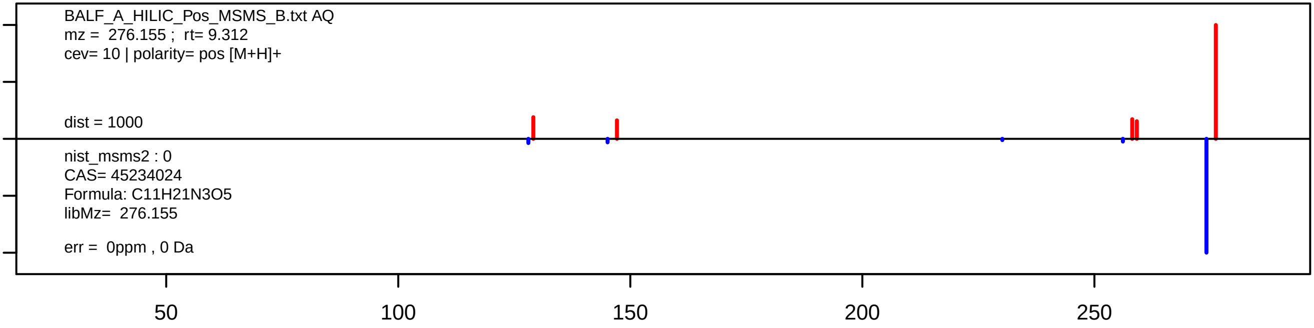

126 . Lys−Ile
Score=921 Dot=957 prob=28.4

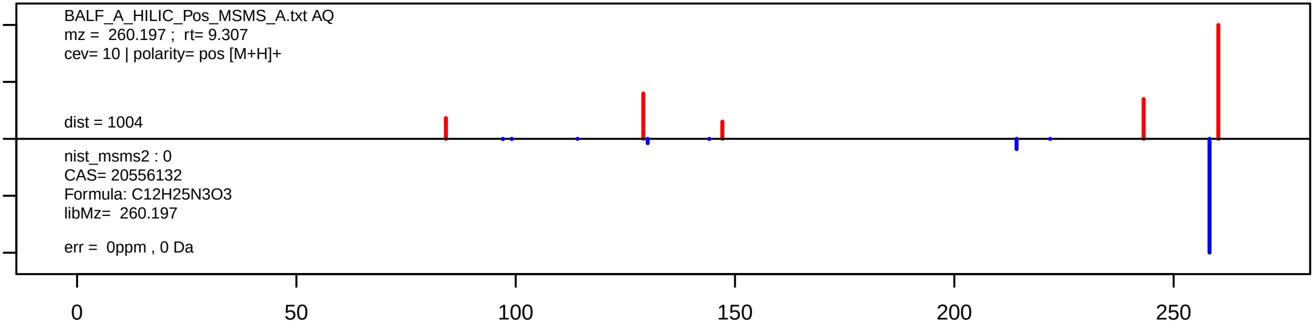

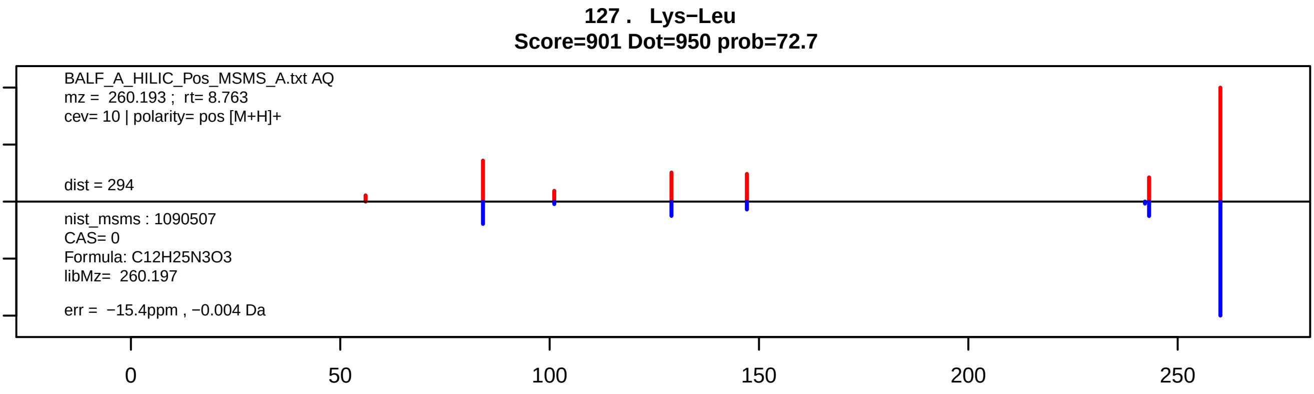
127 . Lys−Leu
Score=901 Dot=950 prob=72.7
BALF_A_HILIC_Pos_MSMS_A.txt AQ
mz = 260.193 ; rt= 8.763
cev= 10 | polarity= pos [M+H]+
dist = 294
nist_msms : 1090507
CAS= 0
Formula: C12H25N3O3
libMz= 260.197
err = −15.4ppm , −0.004 Da
0
50
100
150
200
250

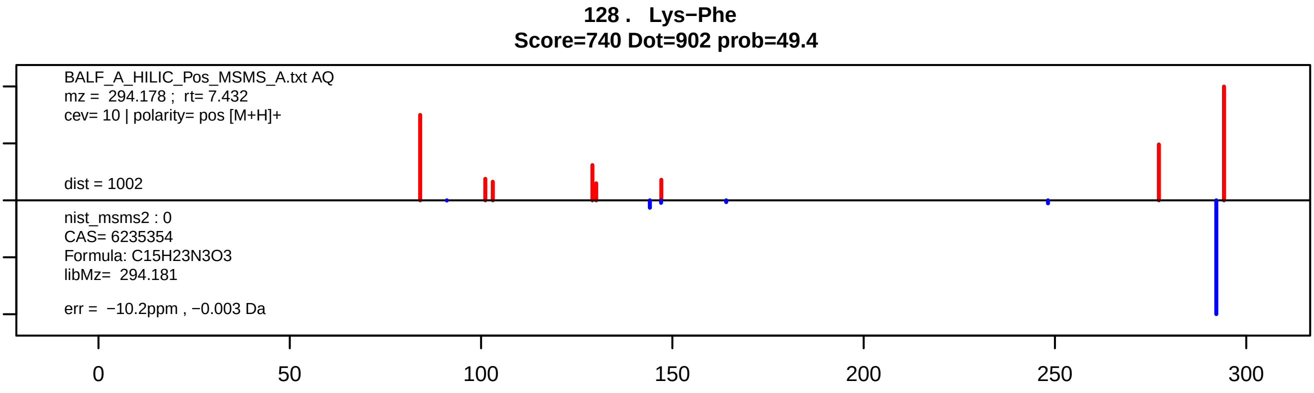
128 . Lys−Phe
Score=740 Dot=902 prob=49.4
BALF_A_HILIC_Pos_MSMS_A.txt AQ
mz = 294.178 ; rt= 7.432
cev= 10 | polarity= pos [M+H]+
dist = 1002
nist_msms2 : 0
CAS= 6235354
Formula: C15H23N3O3
libMz= 294.181
err = −10.2ppm , −0.003 Da
0
50
100
150
200
250
300

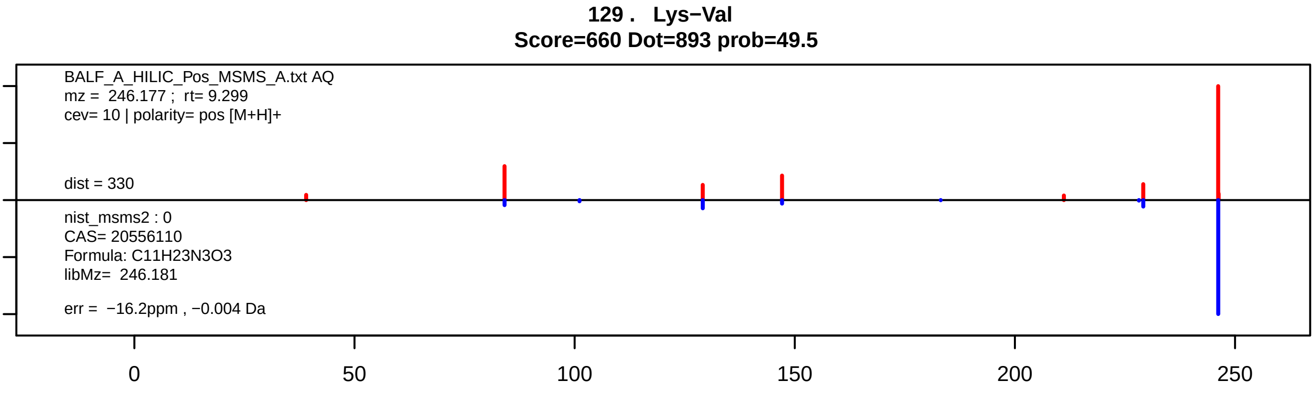
129 . Lys−Val
Score=660 Dot=893 prob=49.5
BALF_A_HILIC_Pos_MSMS_A.txt AQ
mz = 246.177 ; rt= 9.299
cev= 10 | polarity= pos [M+H]+
dist = 330
nist_msms2 : 0
CAS= 20556110
Formula: C11H23N3O3
libMz= 246.181
err = −16.2ppm , −0.004 Da
0
50
100
150
200
250

130 .   Met−Ile
Score=329 Dot=876 prob=47.5

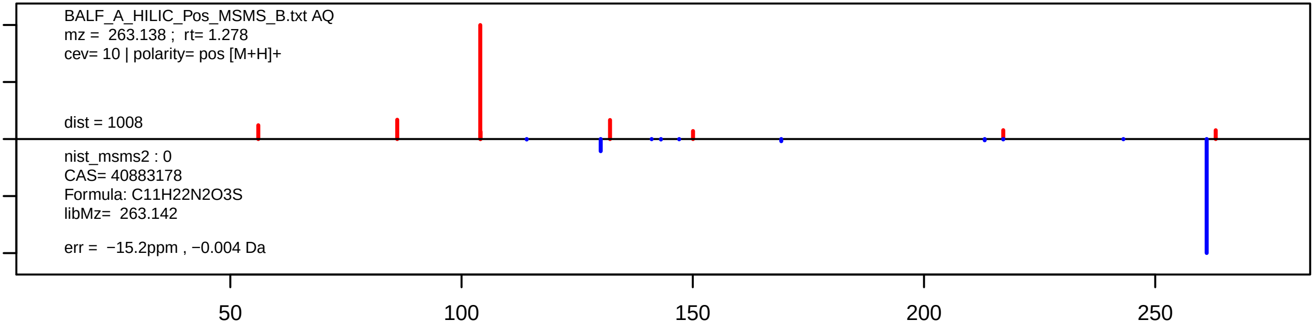

131 .   Met−Leu
Score=233 Dot=804 prob=59.9

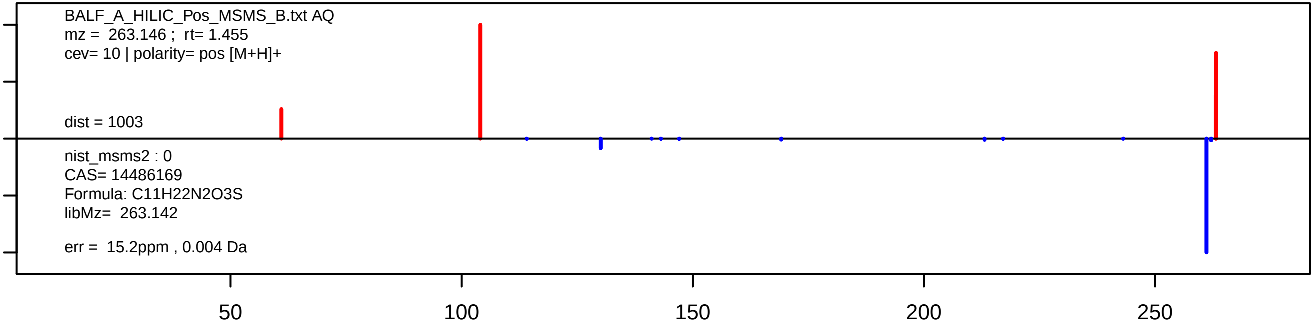

132 .   Met−Val
Score=413 Dot=916 prob=49.5

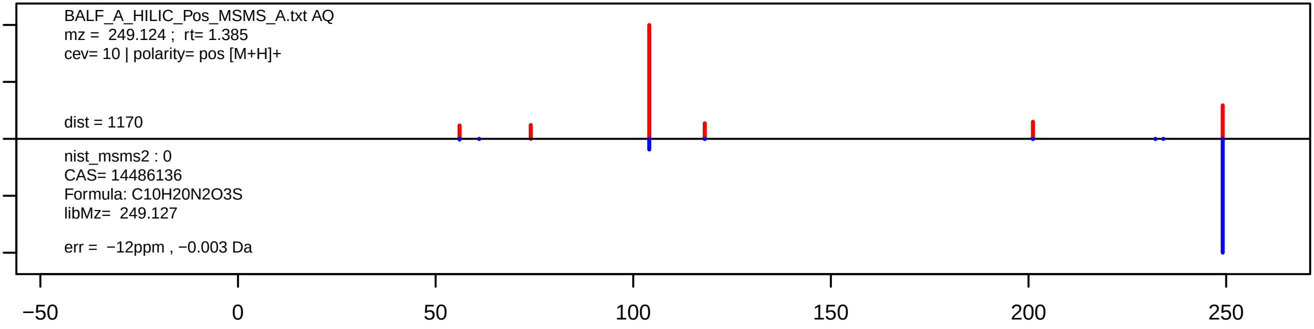

133 . Metformin
Score=884 Dot=953 prob=99

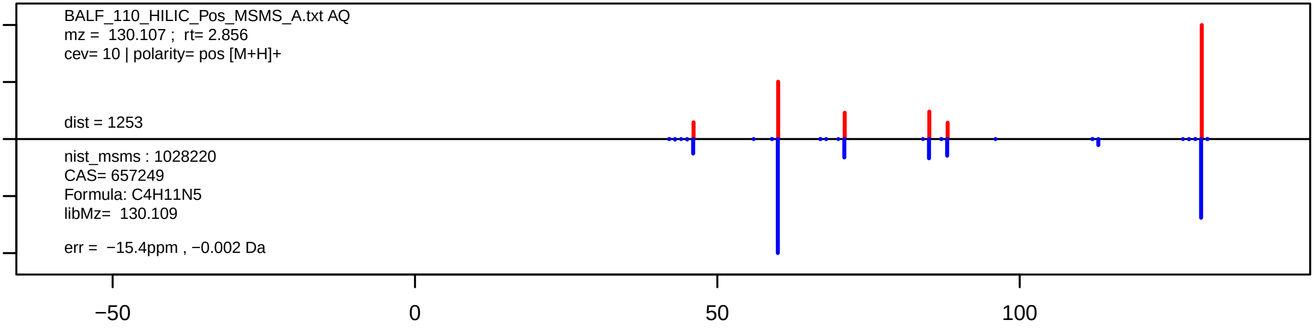

134 . Methadone
Score=275 Dot=909 prob=80.2

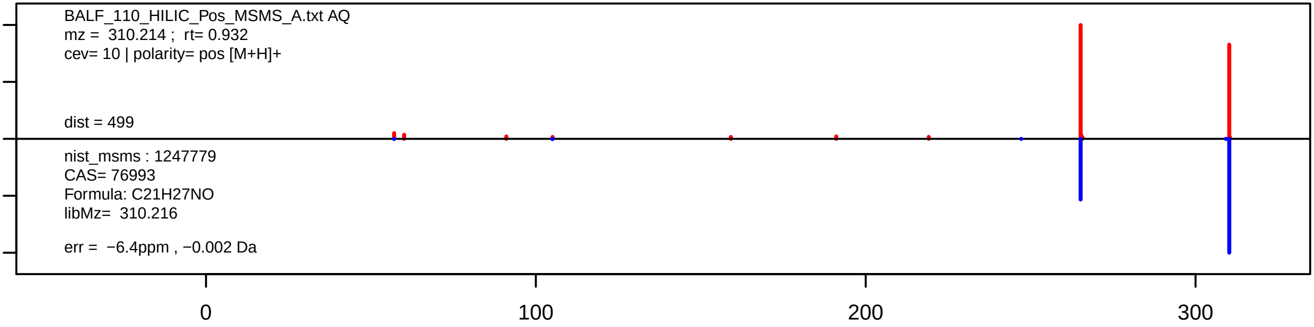

135 . Methyl 4−hydroxybenzoate
Score=882 Dot=920 prob=99

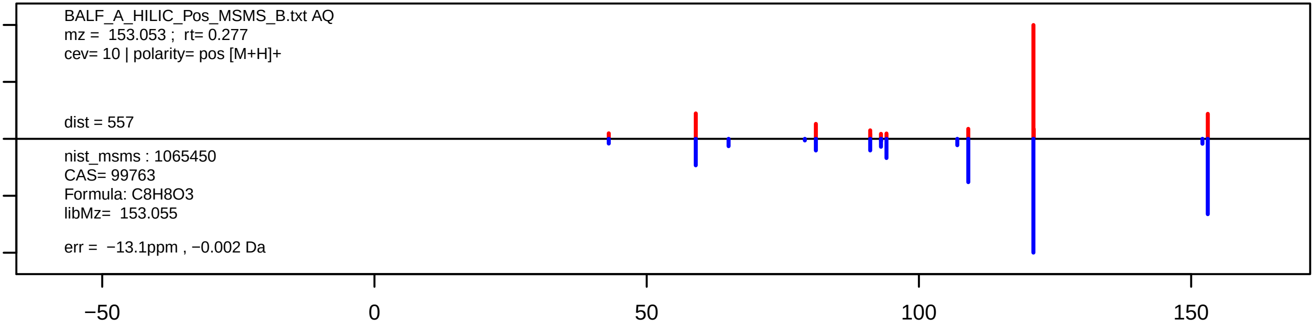

136 . N−.alpha.−(tert−Butoxycarbonyl)−L−histidine
Score=358 Dot=973 prob=72.1

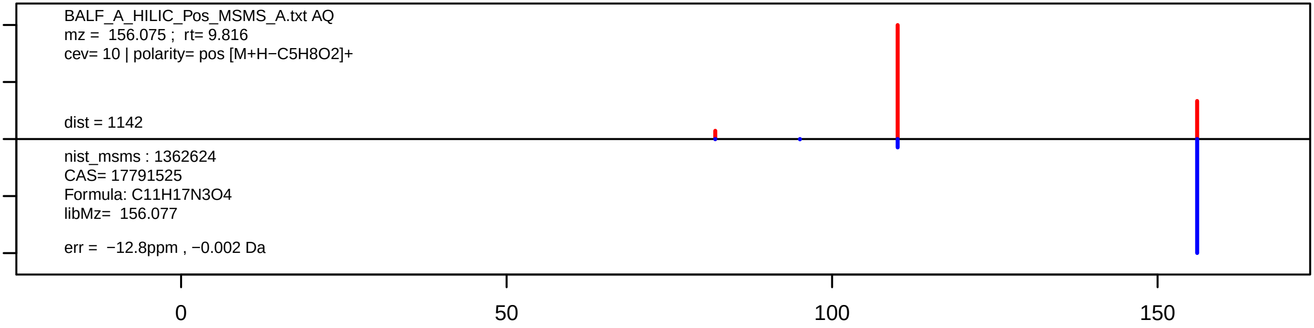

137 . N−Acetyl−L−carnosine
Score=396 Dot=992 prob=37

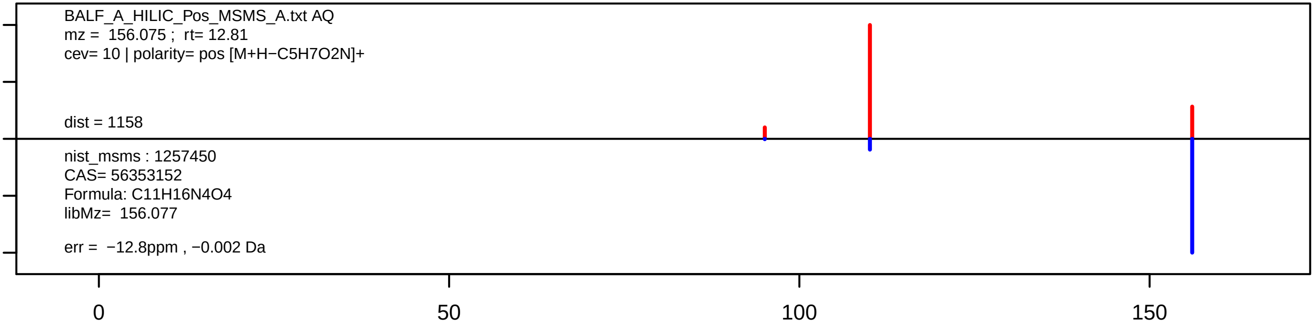

138 . N−Acetyl−L−phenylalanine
Score=627 Dot=944 prob=35

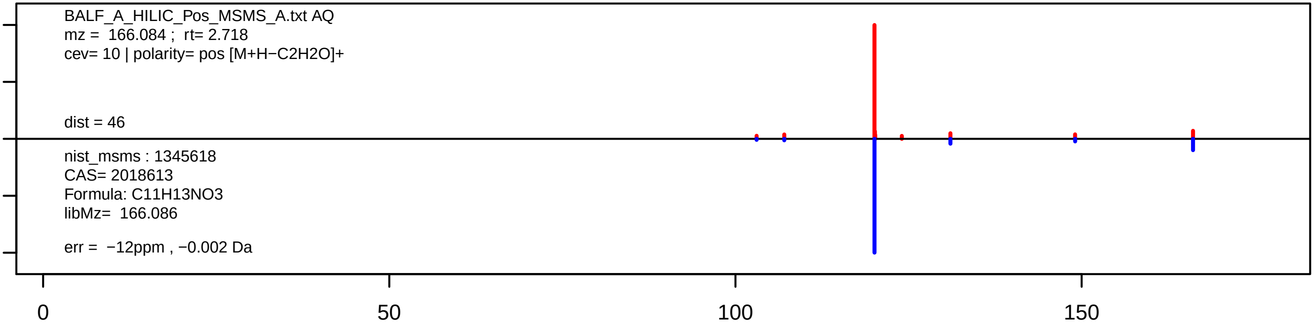

139 . N−Acetyl−L−tyrosine
Score=914 Dot=953 prob=51.7

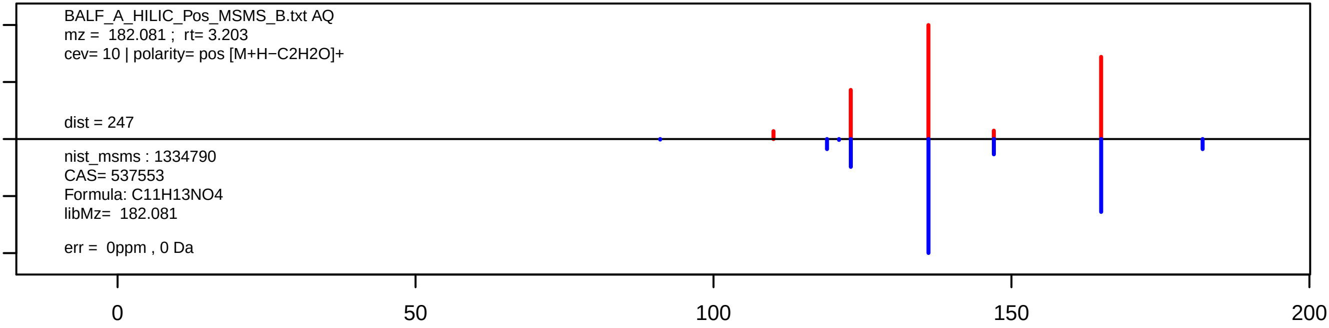

140 . N−Desmethyltramadol
Score=208 Dot=903 prob=96.4

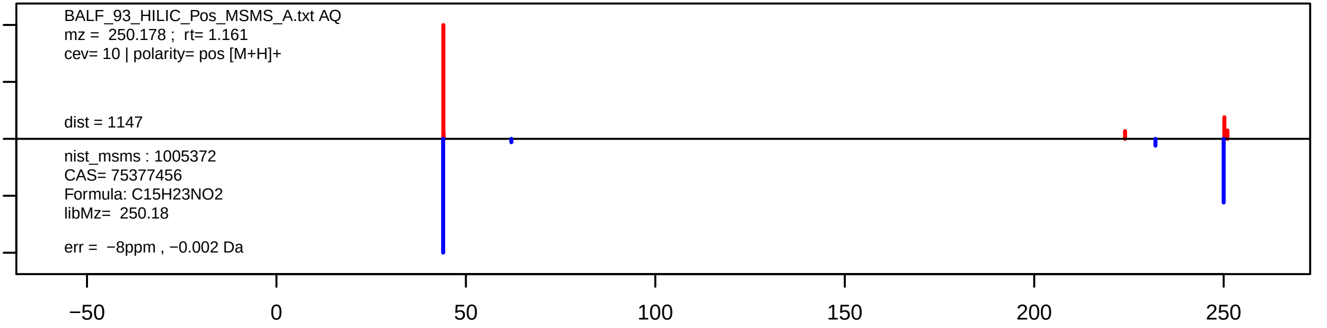

141 . N−Methyl−.alpha.−aminoisobutyric acid
Score=218 Dot=856 prob=85

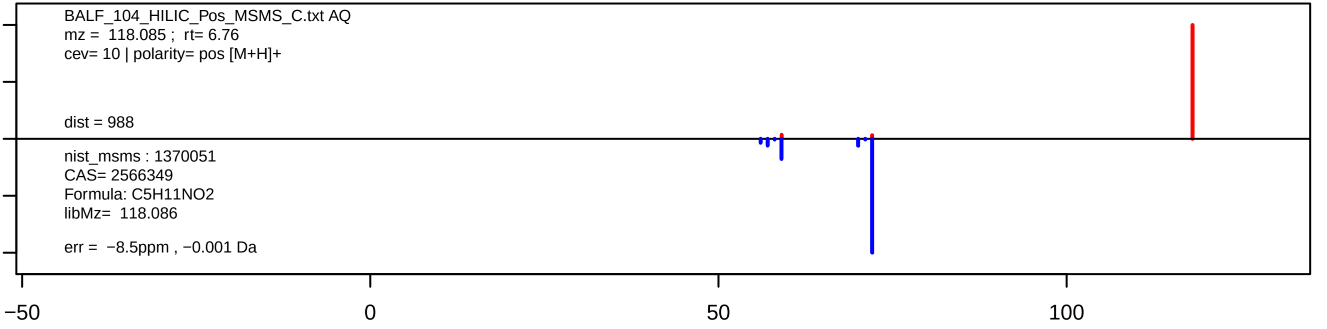

142 . N5−(1−Iminoethyl)−L−ornithine
Score=364 Dot=974 prob=12.2

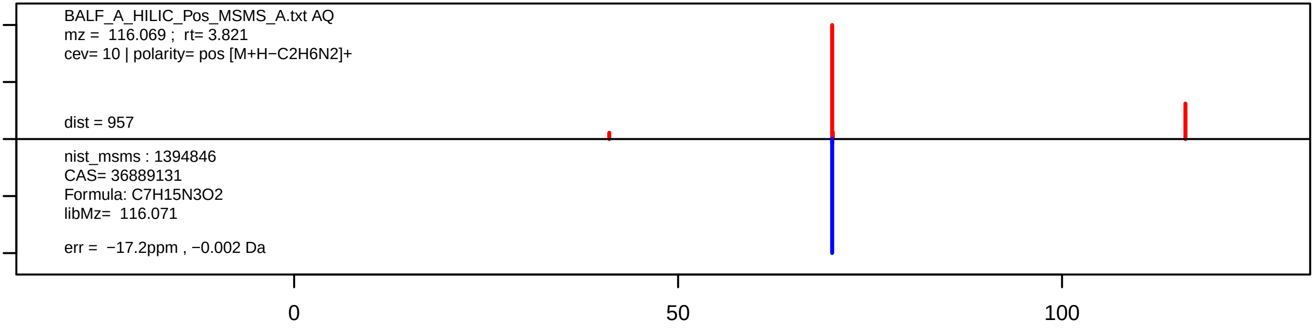

143 . Norquetiapine
Score=314 Dot=817 prob=89.6

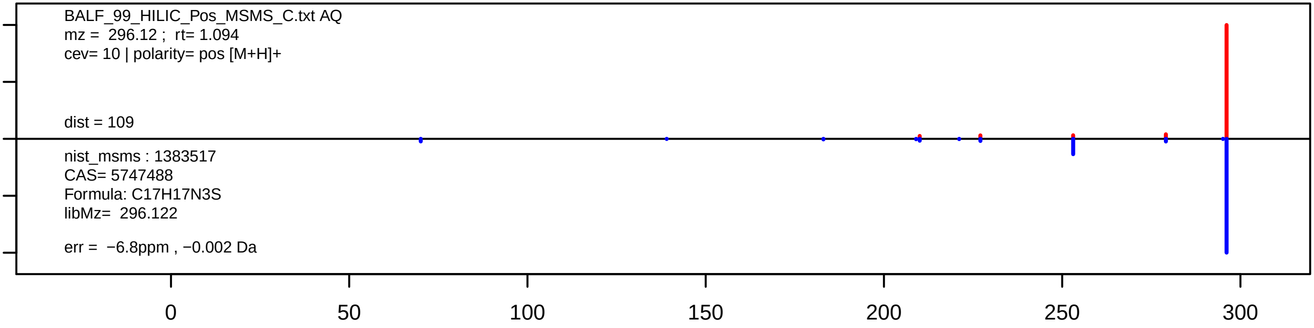

144 . O−Desmethylvenlafaxine
Score=484 Dot=913 prob=83.4

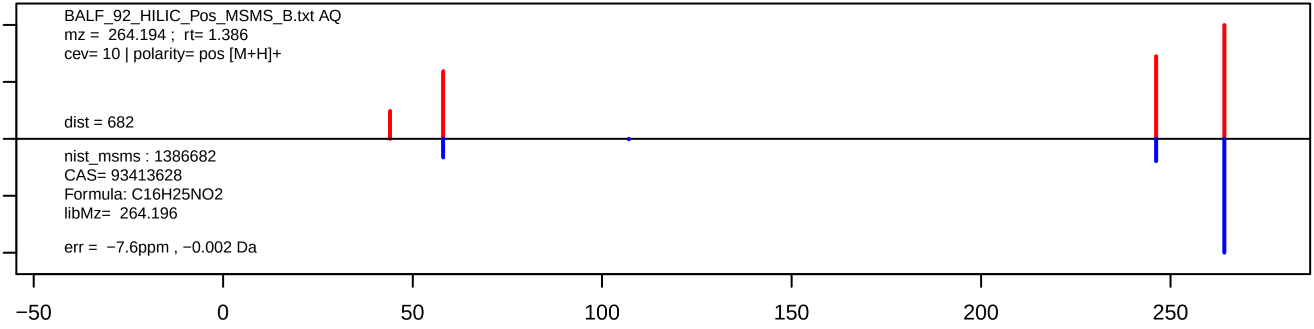

145 . Ondansetron
Score=426 Dot=943 prob=98.6

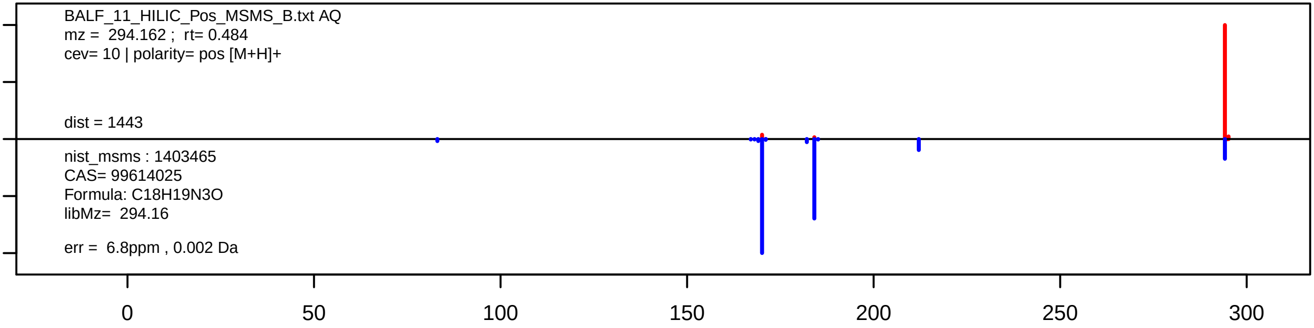

146 . Phe−Ile
Score=491 Dot=961 prob=43.3

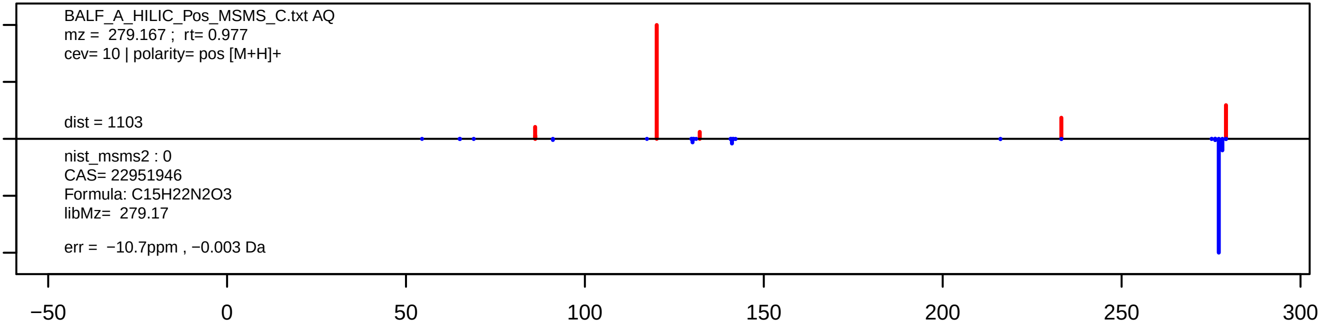

147 . Phe−Leu
Score=279 Dot=899 prob=30.1

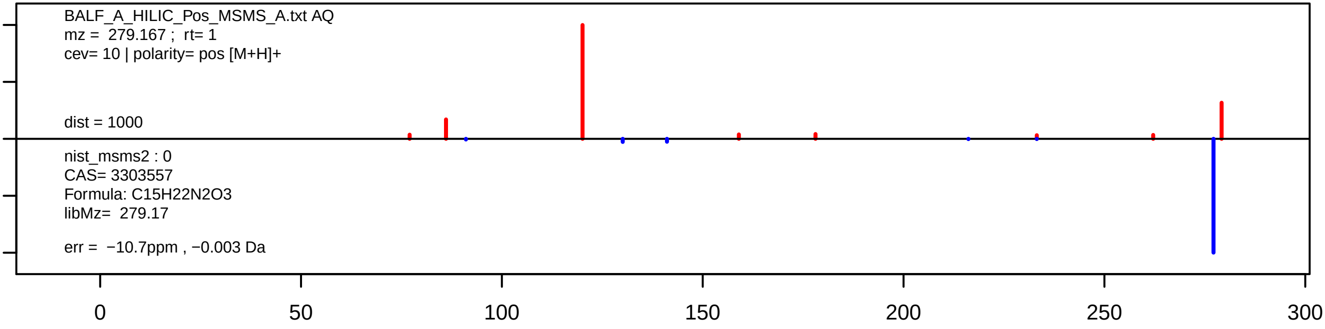

148 . Phe−Phe
Score=269 Dot=958 prob=95.6

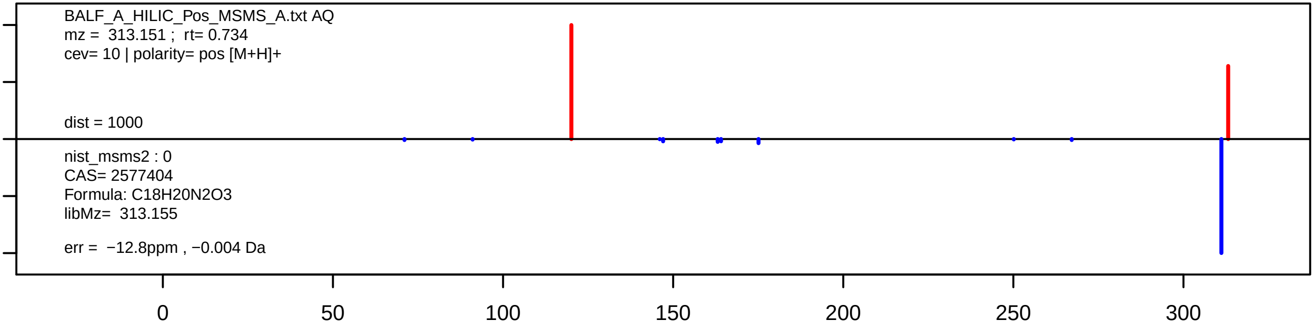

149 . Phe−Val
Score=379 Dot=927 prob=49.7

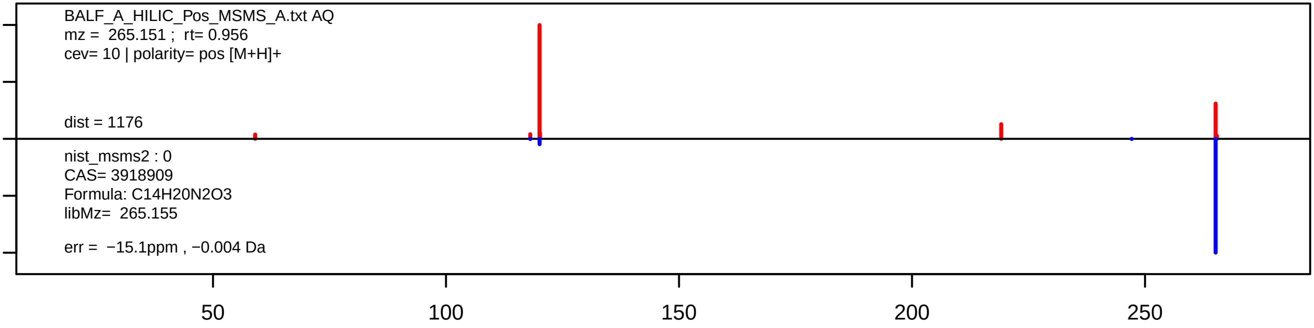

150 . Phenylacetyl−L−glutamine
Score=501 Dot=837 prob=97.9

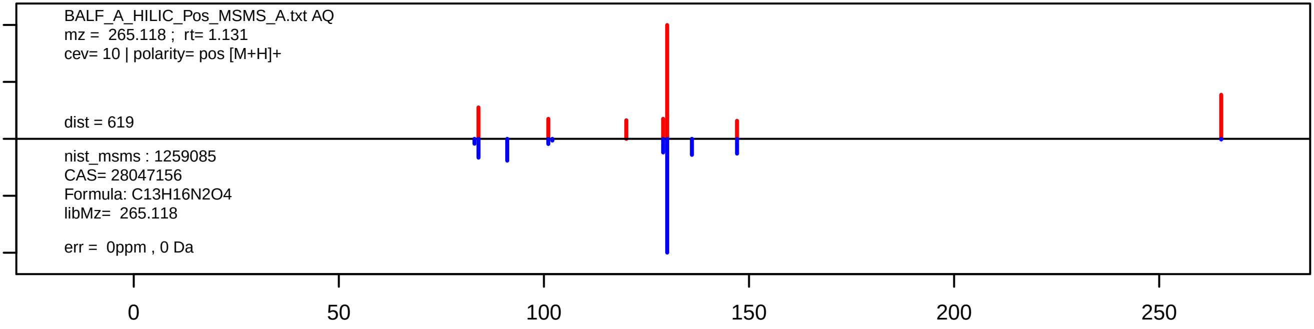

151 . Pro−Ala
Score=400 Dot=999 prob=35.8

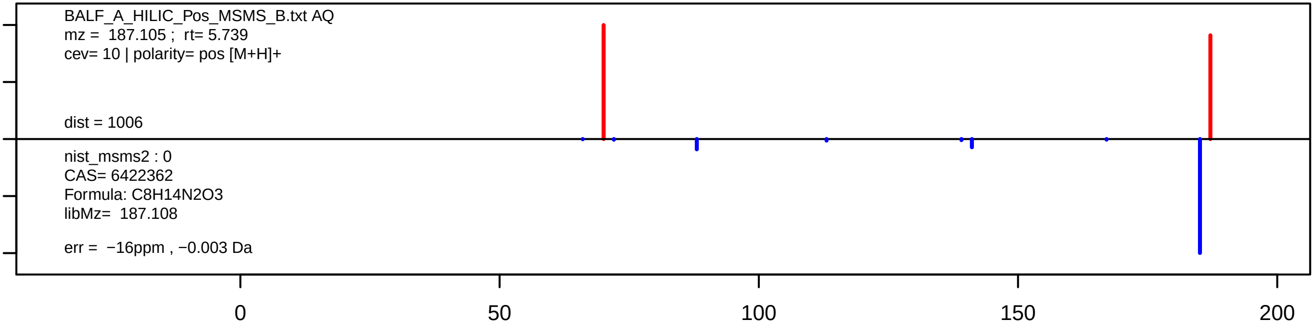

152 . Propionylcarnitine
Score=517 Dot=990 prob=78.6

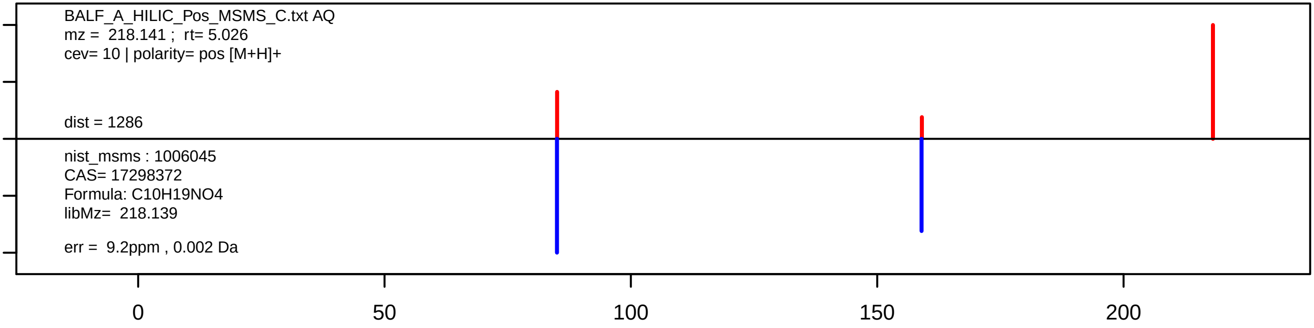

153 . Purine
Score=428 Dot=900 prob=73.2

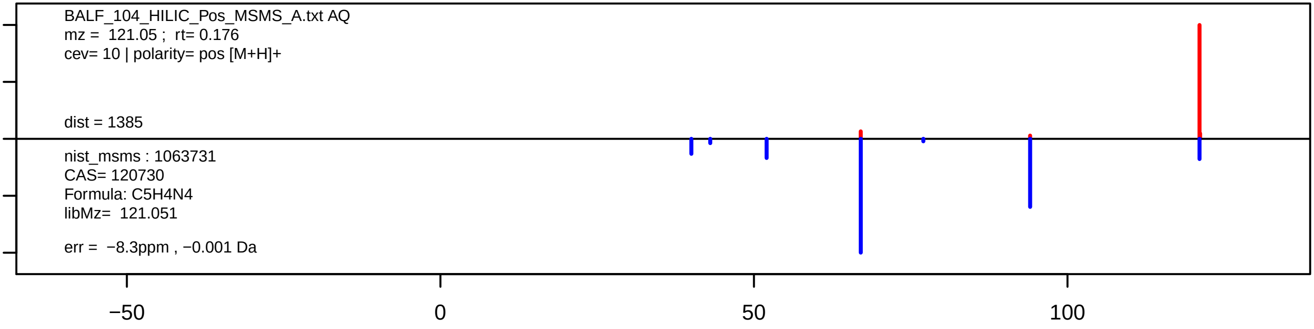

154 . R−(−)−O−Desmethylvenlafaxine
Score=538 Dot=906 prob=84.5

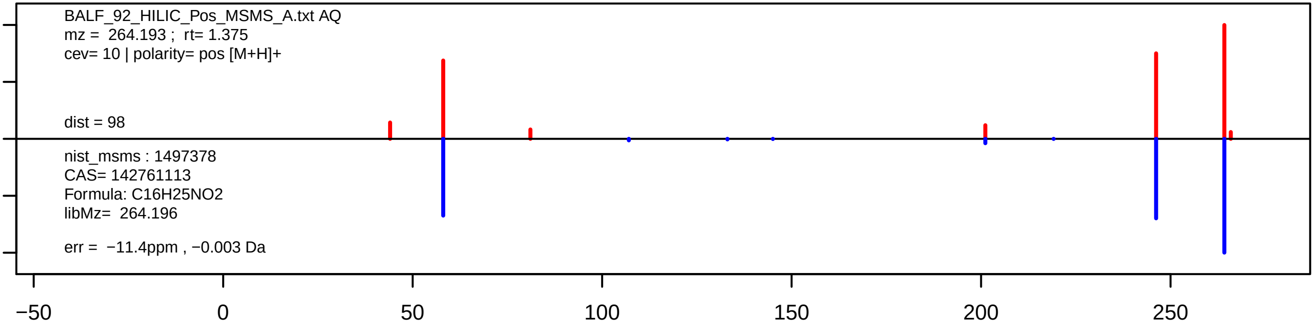

155 . rac erythro−Dihydrobupropion
Score=603 Dot=951 prob=58.8

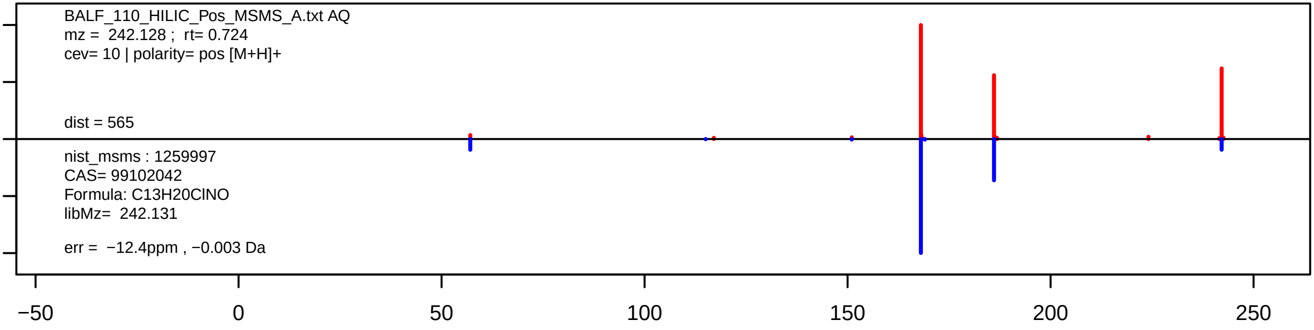

156 . Risperidone
Score=400 Dot=999 prob=96.4

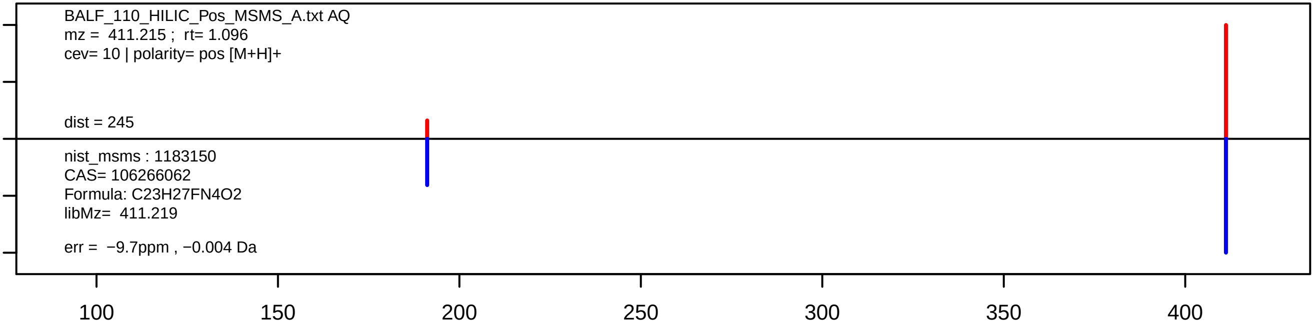

157 . Ropinirole
Score=329 Dot=889 prob=10.2

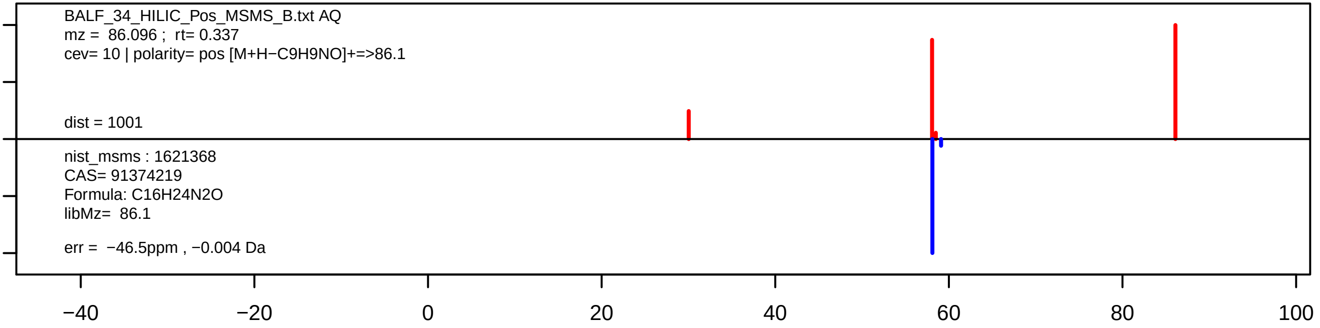

158 . Sarcosine
Score=400 Dot=999 prob=55.4

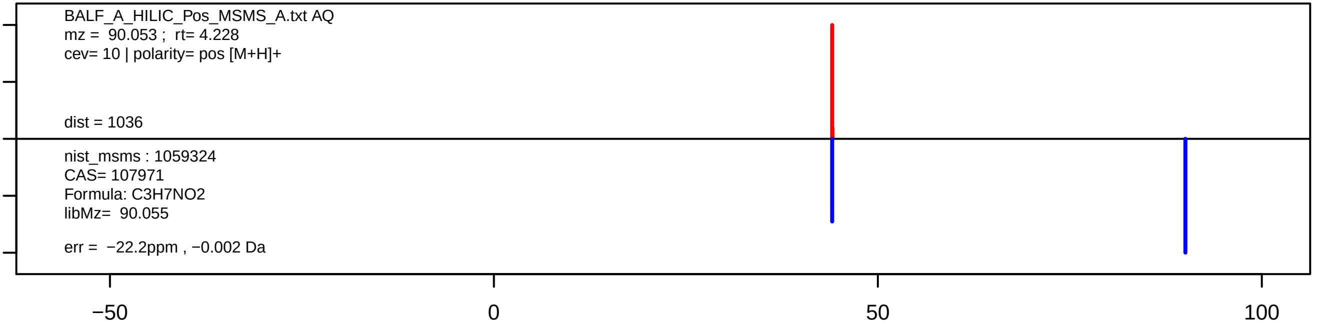

159 . Ser−Ile
Score=569 Dot=837 prob=77.6

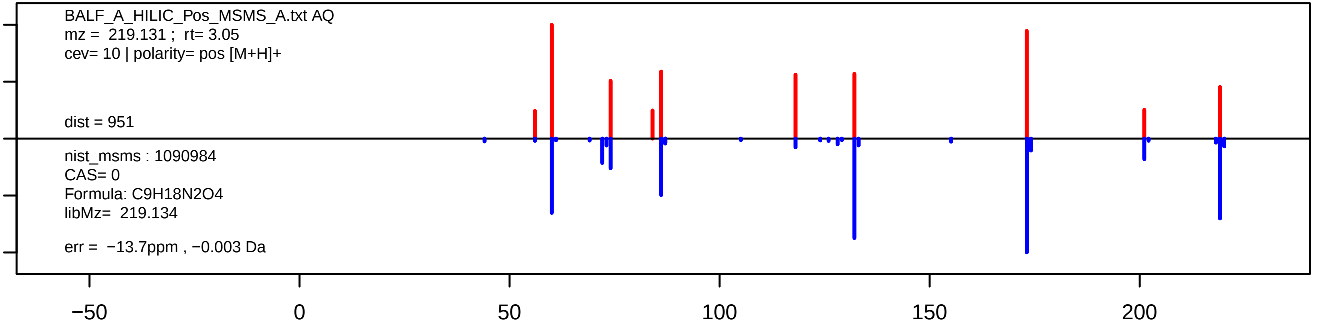

160 . Ser−Leu
Score=733 Dot=827 prob=95.4

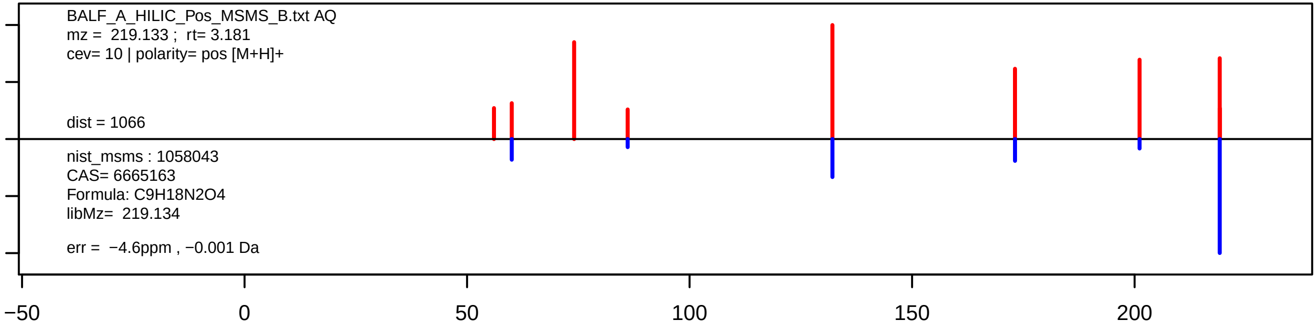

161 . Taurine
Score=489 Dot=954 prob=95.1

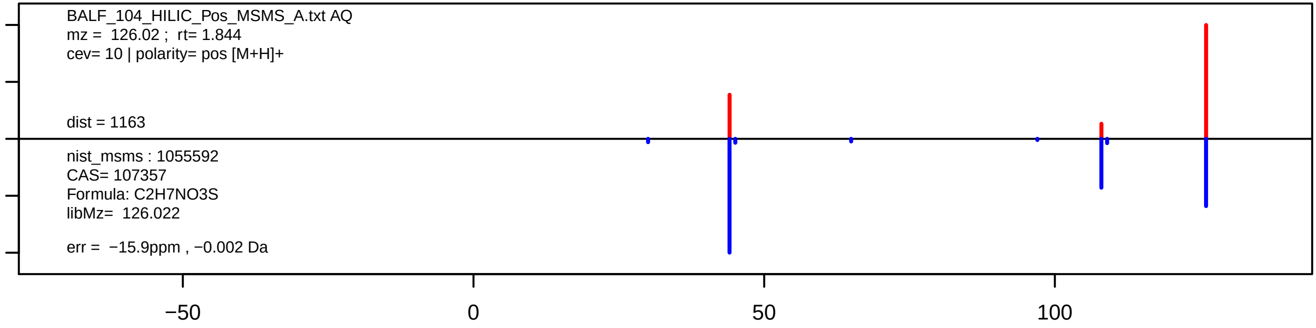

162 . Tetradonium cation
Score=204 Dot=849 prob=96.1

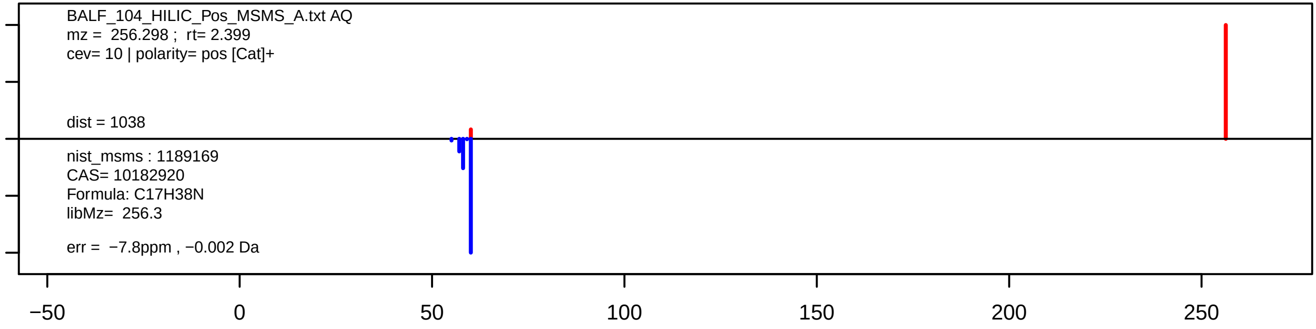

163 . Tetraethylammonium cation
Score=313 Dot=833 prob=11.8

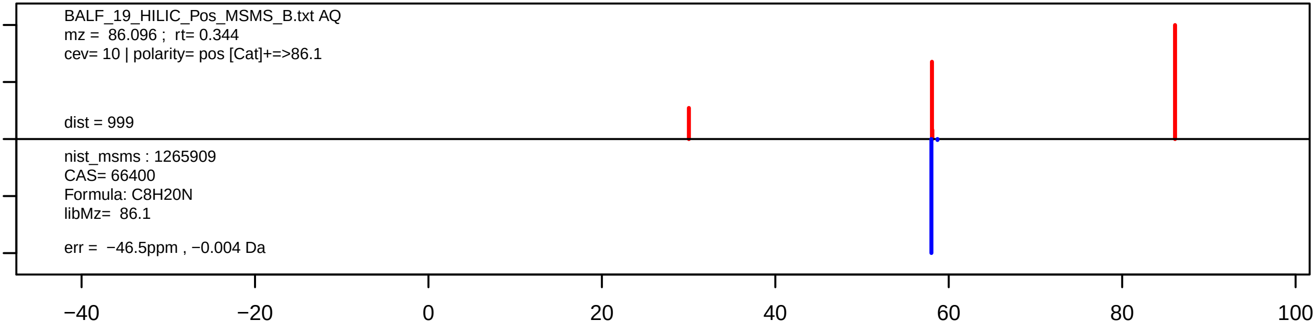

164 . Tetraethylene glycol
Score=456 Dot=921 prob=98.2

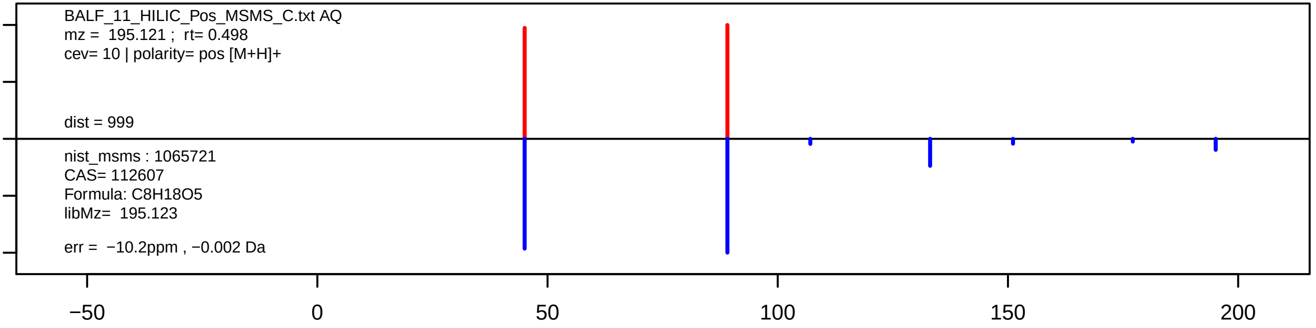

165 . Thr−Leu
Score=802 Dot=877 prob=99

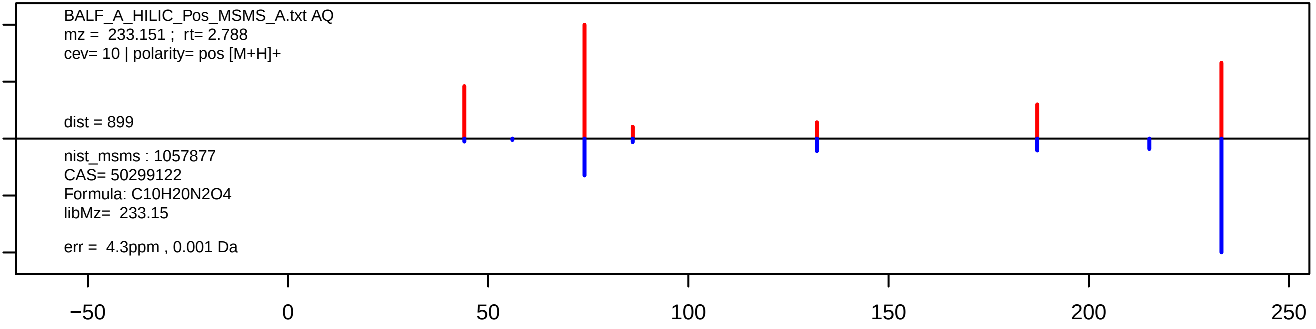

166 . Thr−Val−Leu
Score=443 Dot=887 prob=49.6

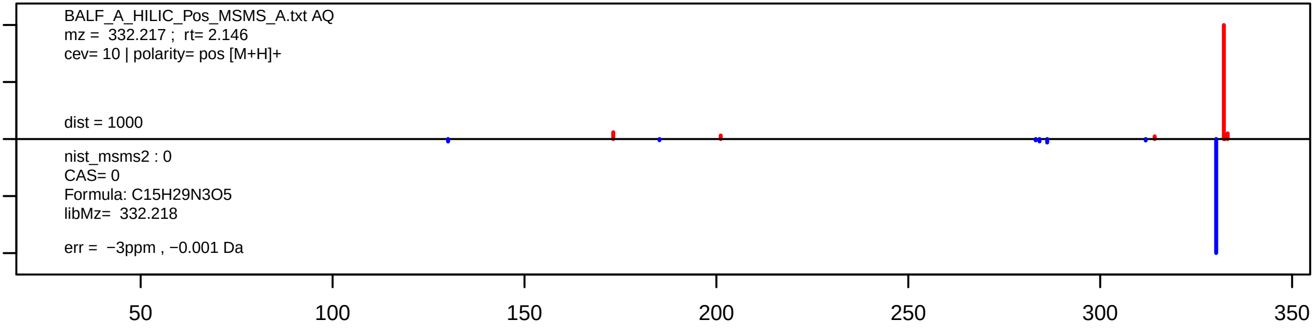

167 . threo−Dihydrobupropion
Score=624 Dot=959 prob=58.9

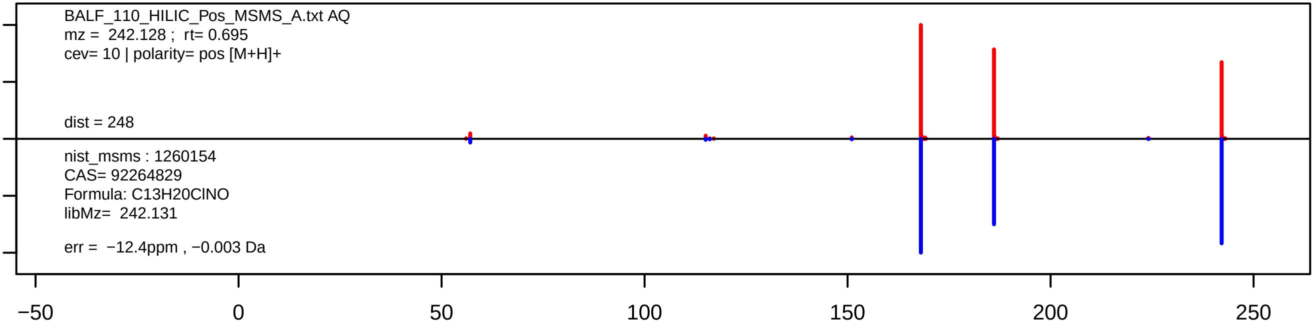

168 . Tramadol
Score=341 Dot=972 prob=97

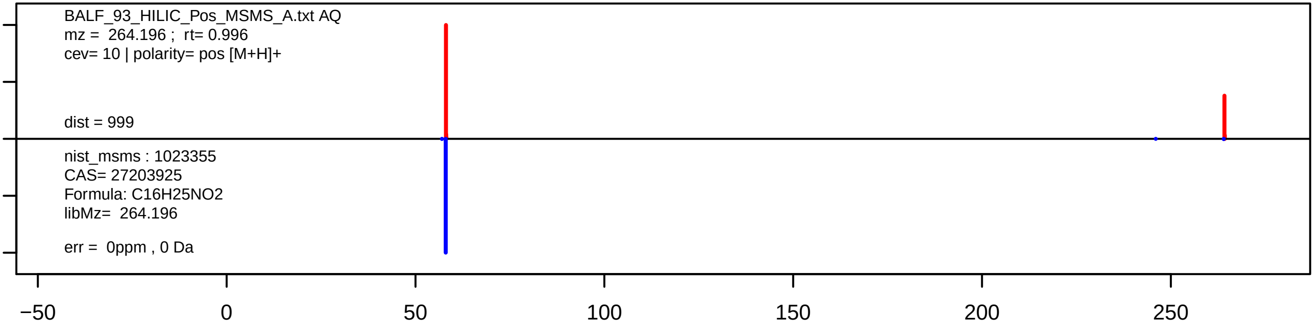

169 .   Tyr−Leu
Score=155 Dot=810 prob=20.3

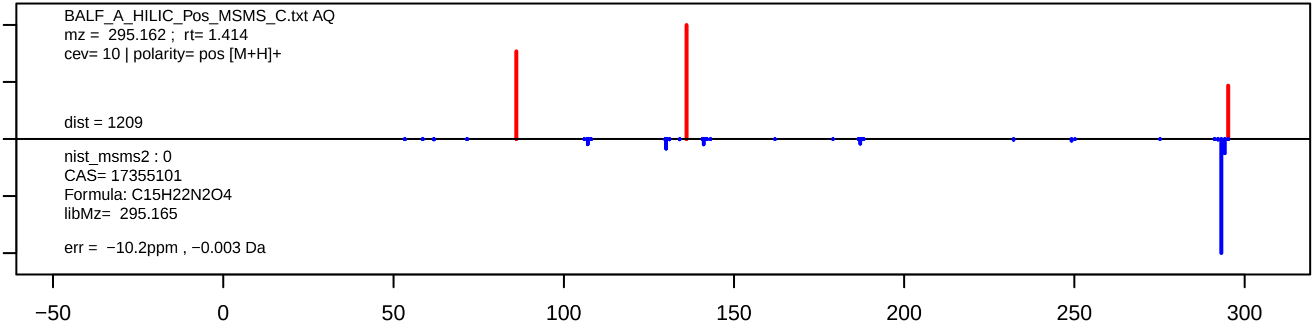

170 .   Val−Ala
Score=314 Dot=883 prob=46.4

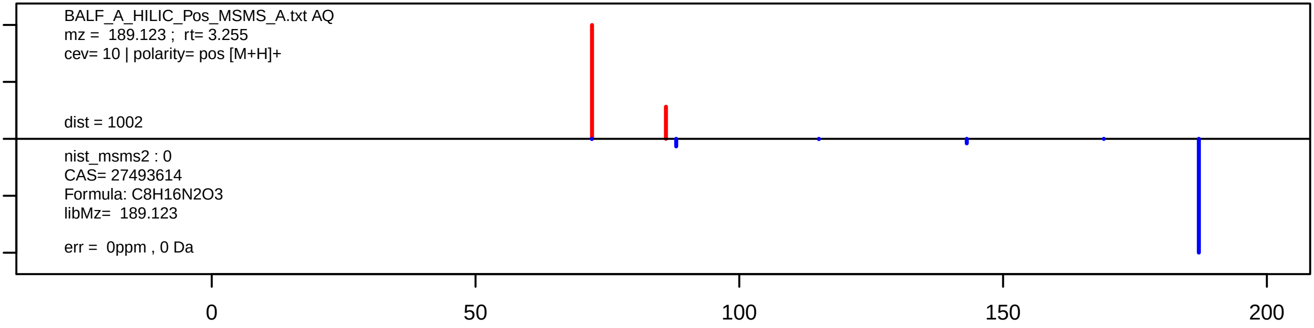

171 .   Val−Ile
Score=386 Dot=940 prob=37.5

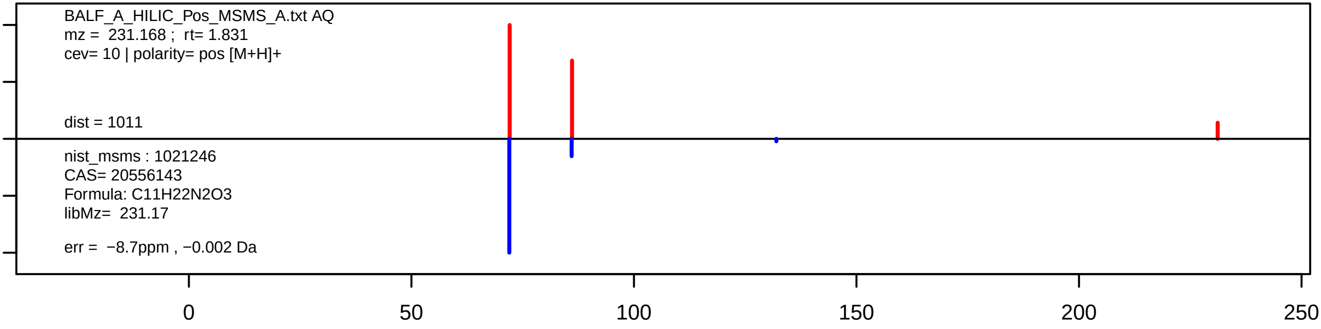

172 . Val−Leu
Score=565 Dot=824 prob=81

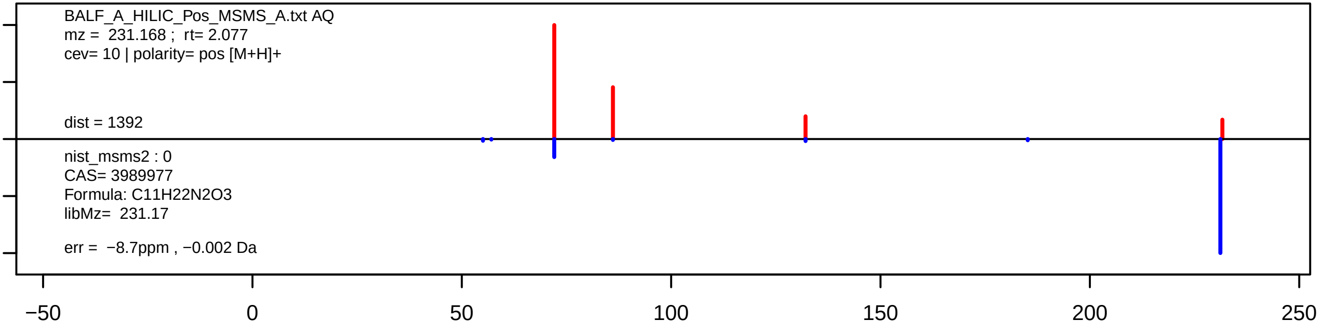

173 . Val−Trp
Score=348 Dot=868 prob=97.8

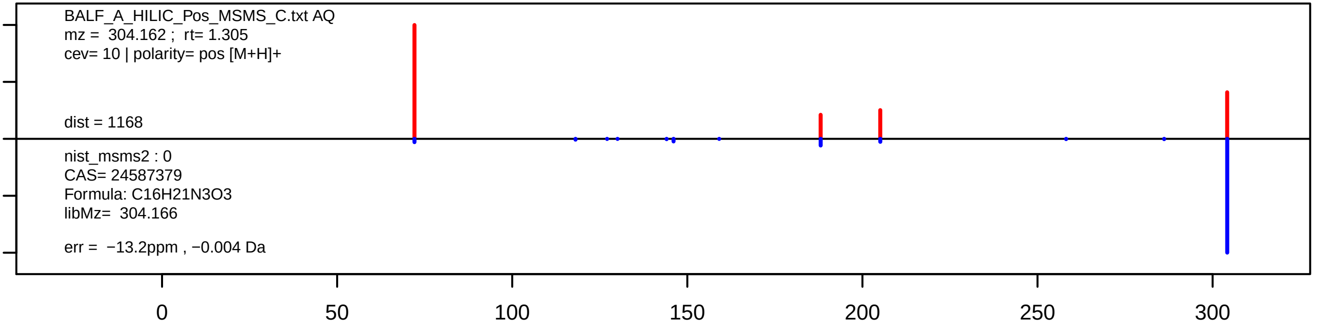

174 . Val−Tyr
Score=490 Dot=923 prob=49.5

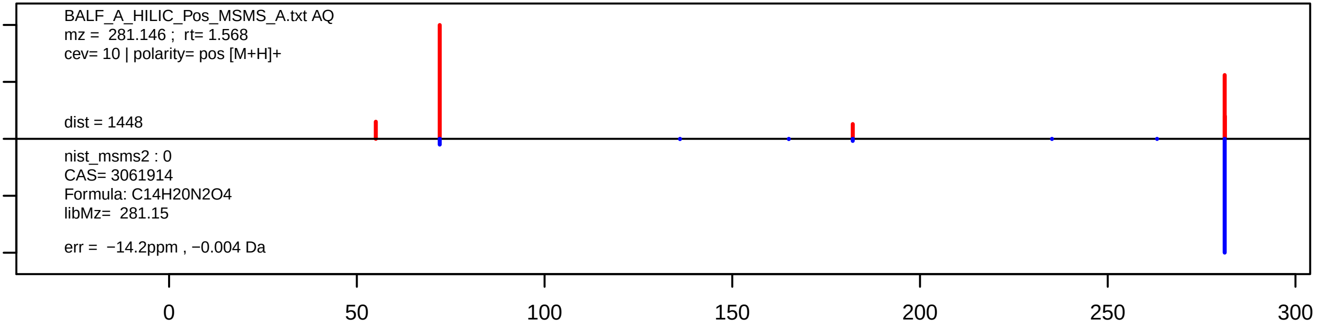

175 . Val−Val
Score=456 Dot=940 prob=99

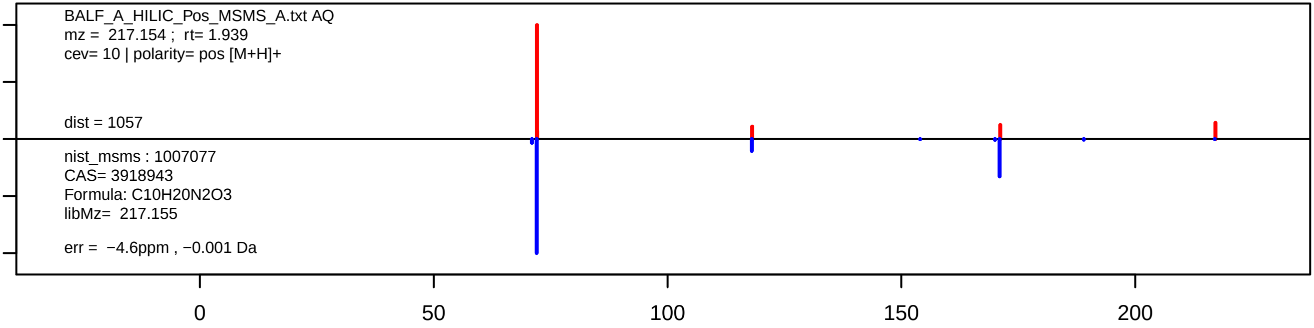

176 . Venlafaxine
Score=606 Dot=958 prob=98.3

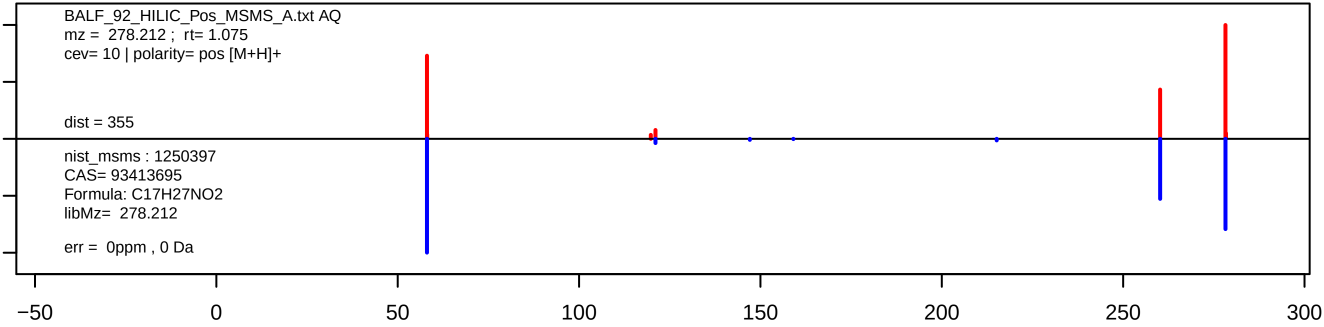

177 . Xanthine
Score=409 Dot=850 prob=55

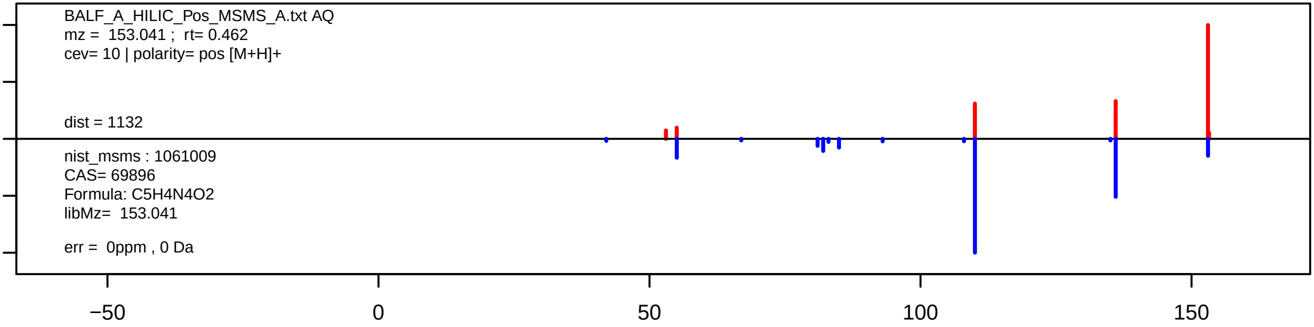

178 . .alpha.−Guanidinoglutaric acid
Score=629 Dot=976 prob=69.1

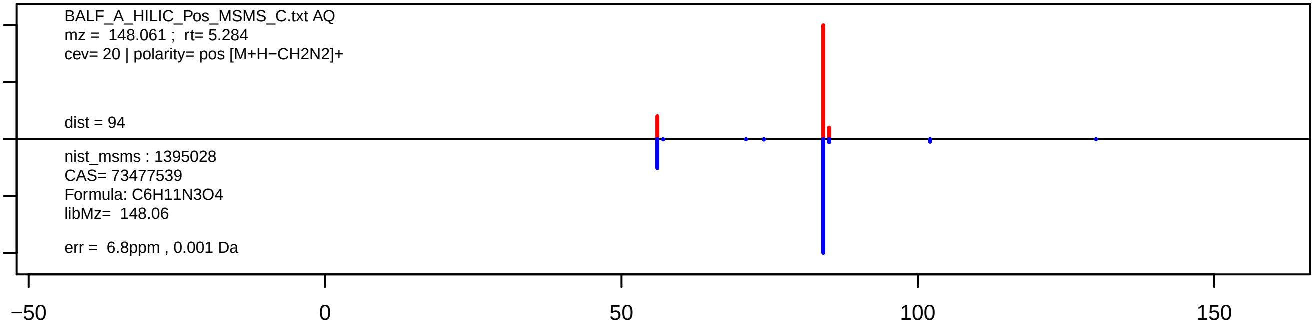

179 . .beta.−Methylphenethylamine
Score=400 Dot=999 prob=1.3

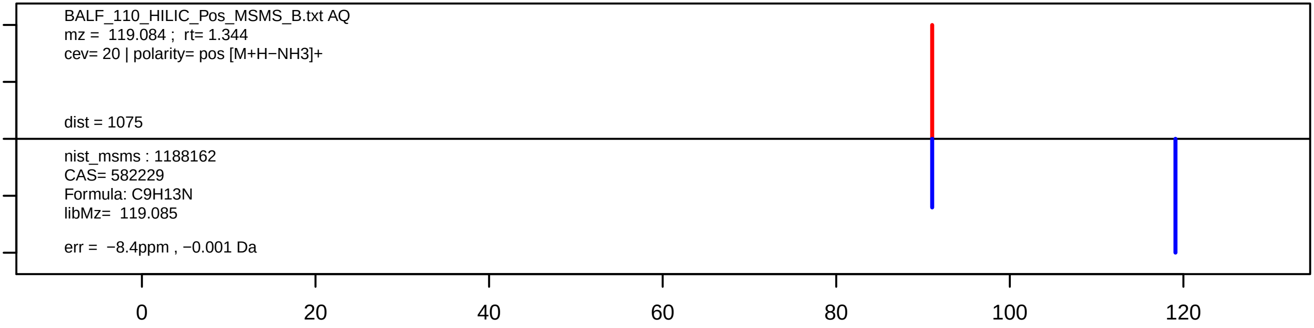

180 . (−)−Cotinine
Score=397 Dot=920 prob=77.9

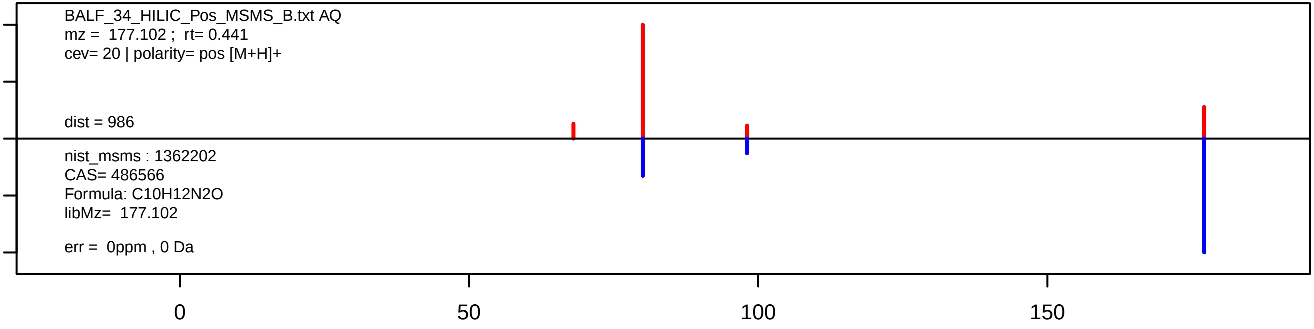

181 . (−)−Methamphetamine
Score=201 Dot=941 prob=74.2

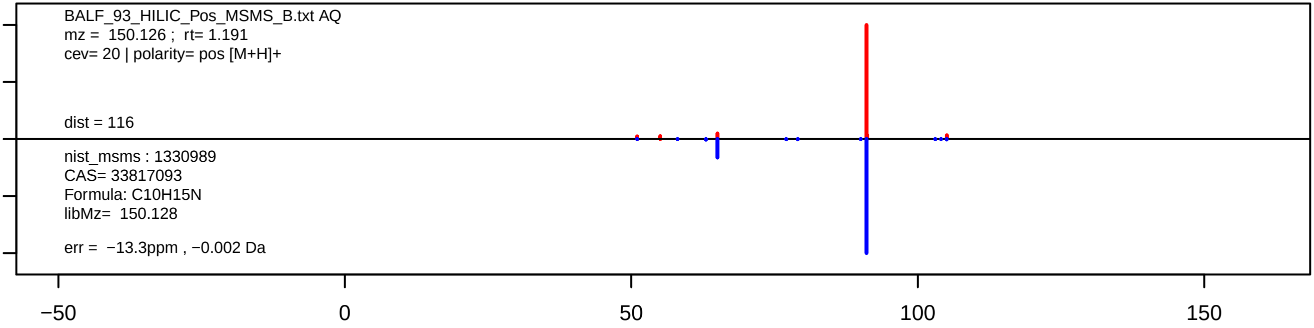

182 . (.+/−.)−N−Ethyl−1−phenyl−2−butylamine
Score=278 Dot=936 prob=51.8

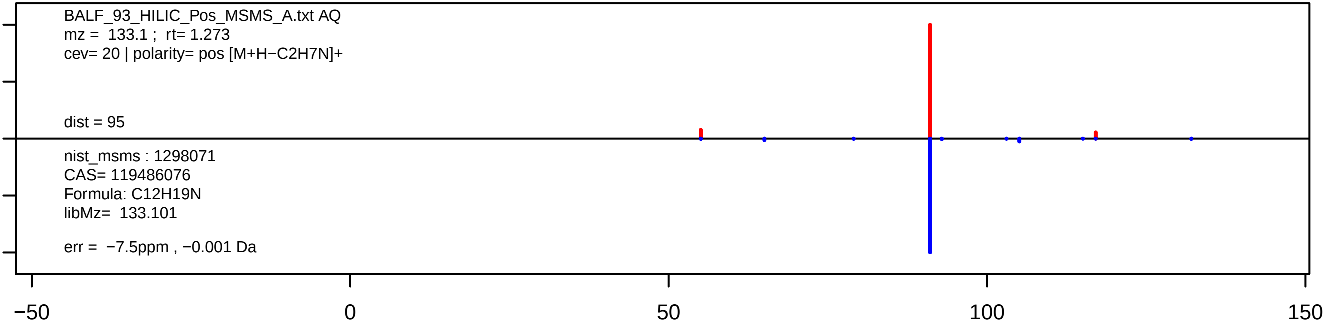

183 . (+)−Methamphetamine
Score=399 Dot=999 prob=20.9

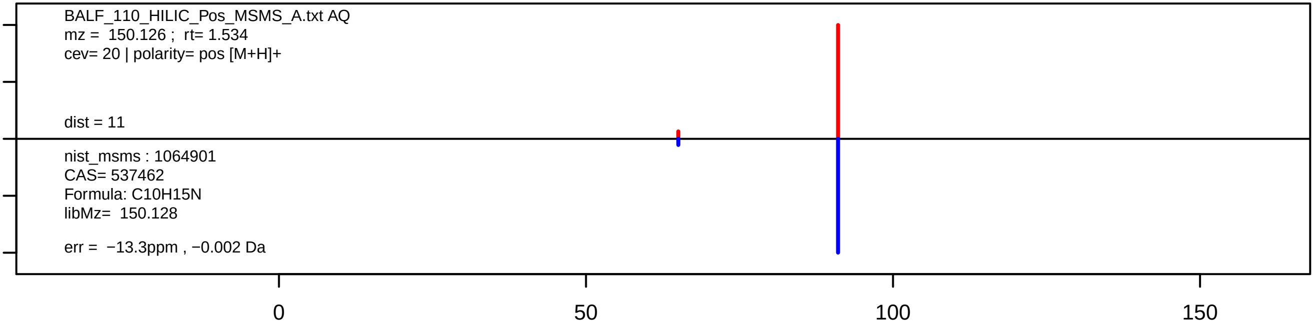

184 . (3−Carboxypropyl)trimethylammonium cation
Score=705 Dot=942 prob=97.5

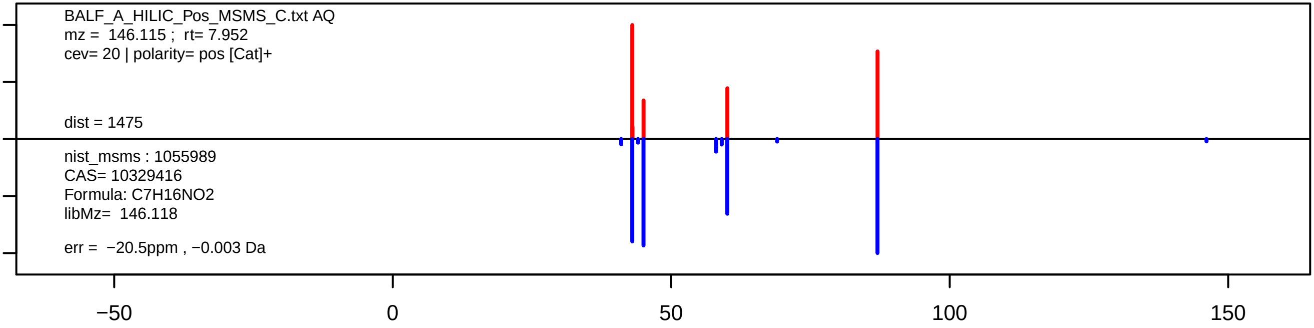

185 . (5E)−4−Methyl−5−{[(phenylsulfonyl)oxy]imino}−1,1'−bi(cyclohexane)−3,6−dien−2−one
Score=159 Dot=941 prob=88.9

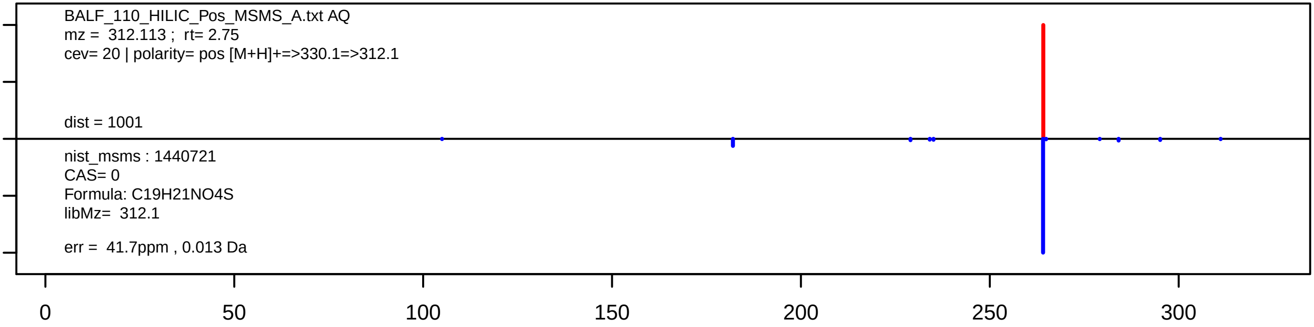

186 . 1−(1Z−Octadecenyl)−2−(5Z,8Z,11Z,14Z−eicosatetraenoyl)−sn−glycero−3−phosphoethanolamine
Score=375 Dot=896 prob=96.6

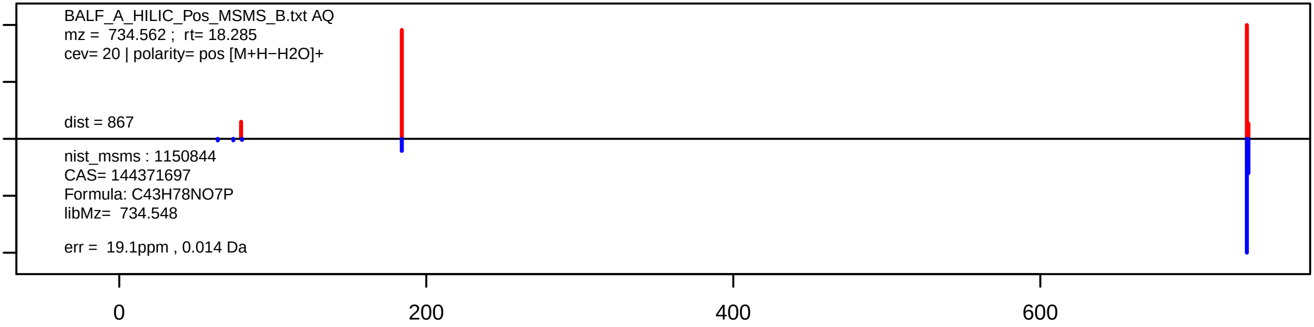

187 .   1−Methyl−3−phenylpropylamine
Score=326 Dot=916 prob=37.1

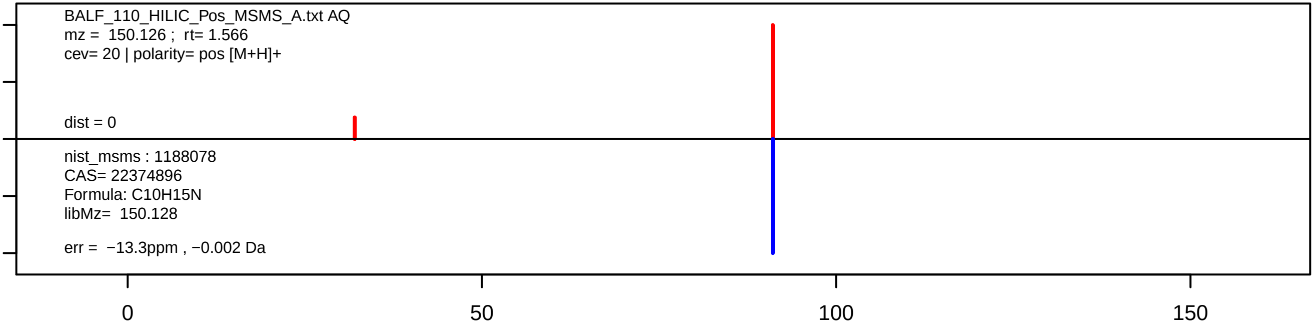

188 .   1−Oleoyl−2−palmitoyl−sn−glycero−3−phosphocholine
Score=458 Dot=943 prob=88.3

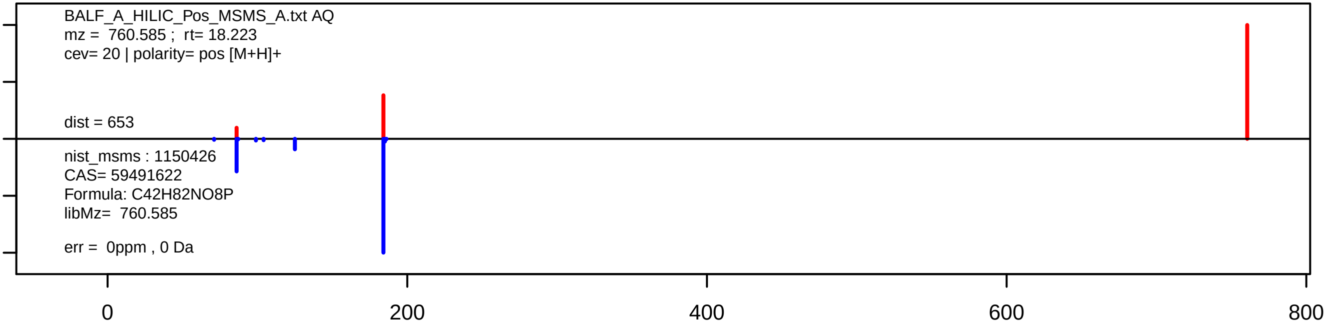

189 .   1−Palmitoyl−2−linoleoyl−sn−glycero−3−phosphocholine
Score=554 Dot=846 prob=97.7

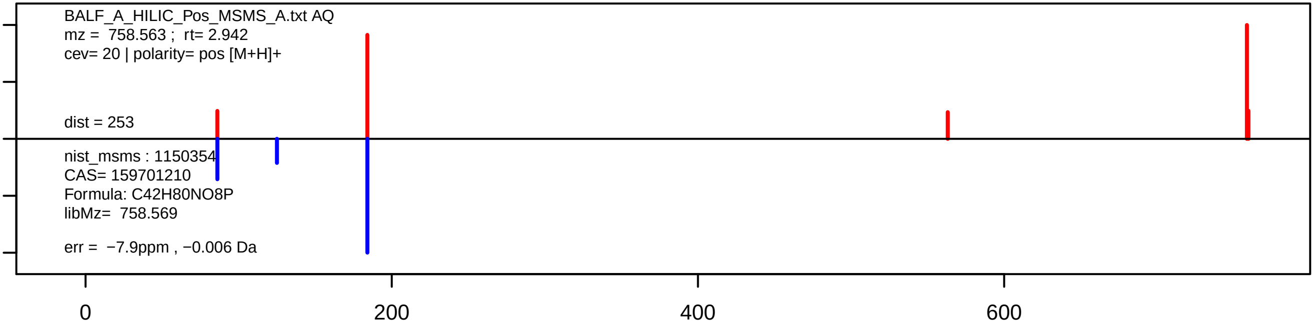

190 . 1−Propanone, 1−(1,3−benzodioxol−5−yl)−2−(dimethylamino)−
Score=921 Dot=955 prob=98.4

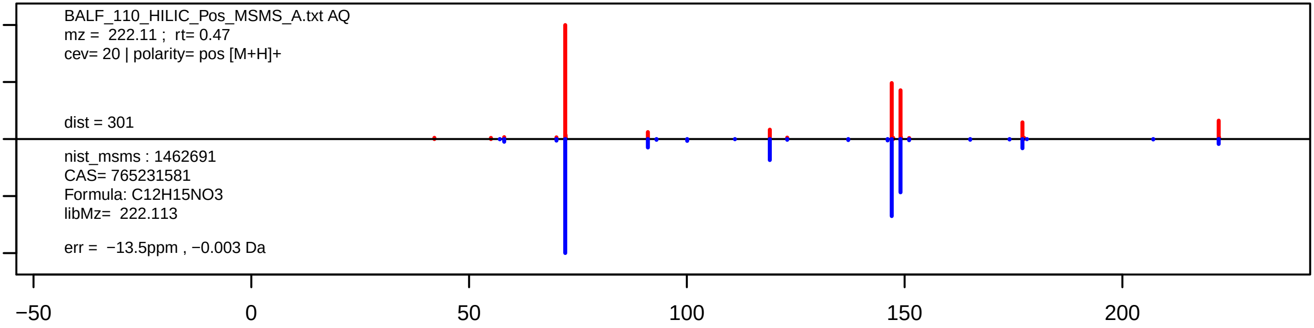

191 . 1−Stearoyl−2−myristoyl−sn−glycero−3−phosphocholine
Score=347 Dot=912 prob=73.3

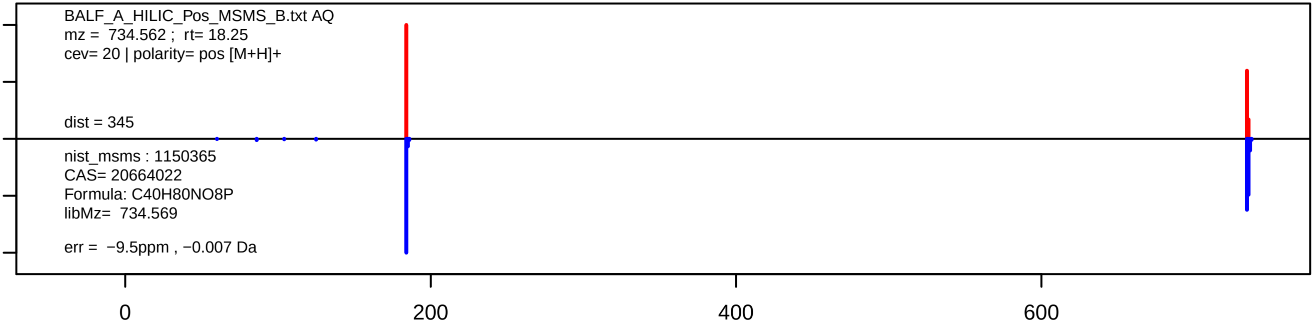

192 . 1,2−Dihexadecanoyl−sn−glycero−3−phosphocholine
Score=216 Dot=886 prob=72.8

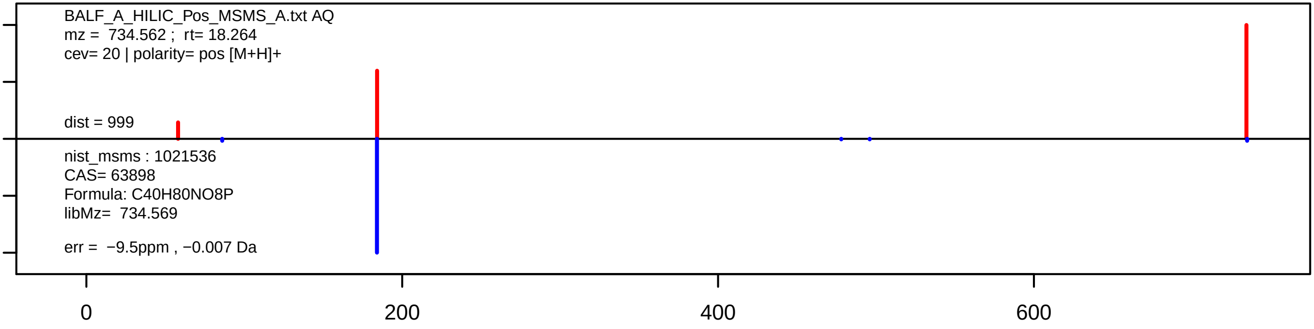

193 . 1,2−dioleoyl−sn−glycero−3−phosphatidylcholine
Score=400 Dot=999 prob=50

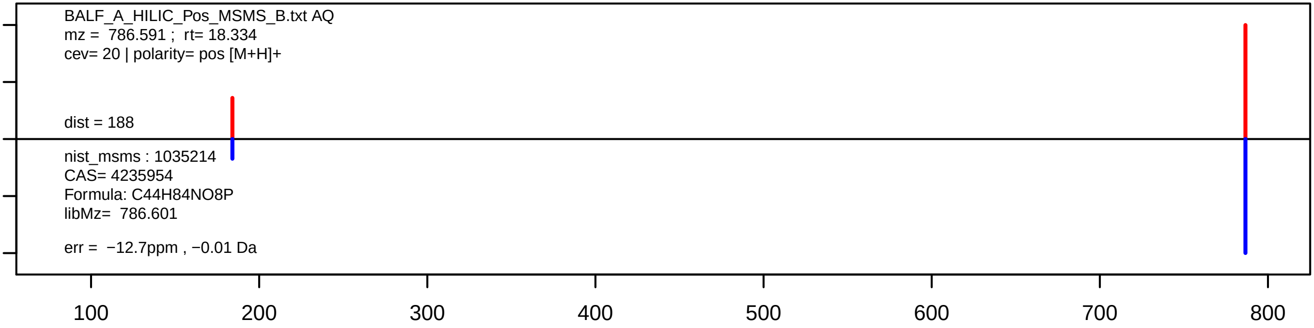

194 . 1,2−Dipalmitoyl−sn−glycero−O−ethyl−3−phosphatidylcholine cation
Score=239 Dot=868 prob=44

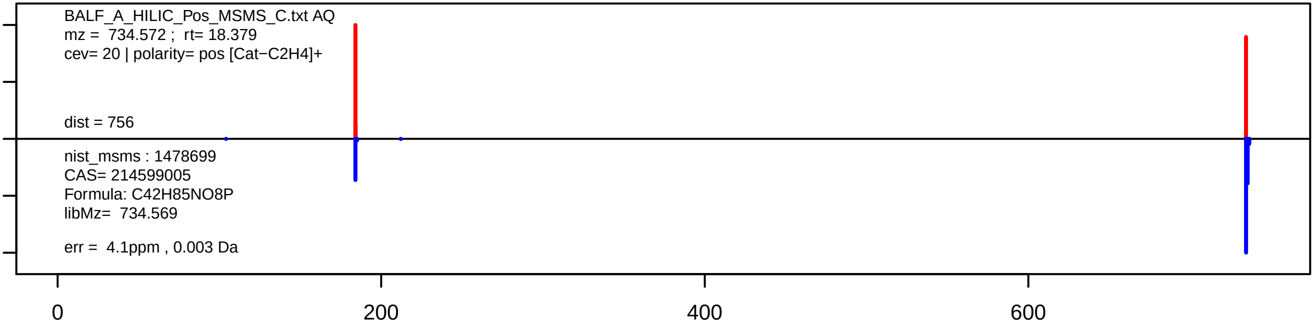

195 . 2−[(2,6−Dimethylphenyl)amino]−N,N,N−triethyl−2−oxoethanaminium cation
Score=537 Dot=999 prob=61.5

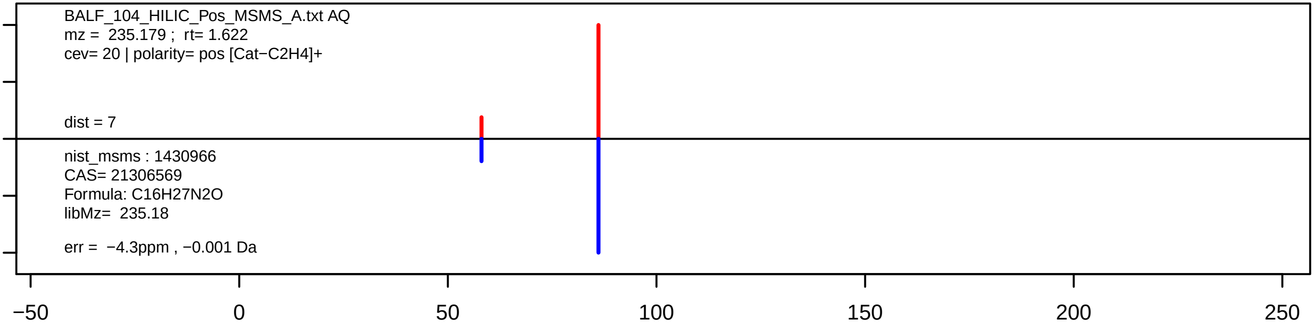

196 . 2−Amino−1−phenylbutane
Score=380 Dot=943 prob=48

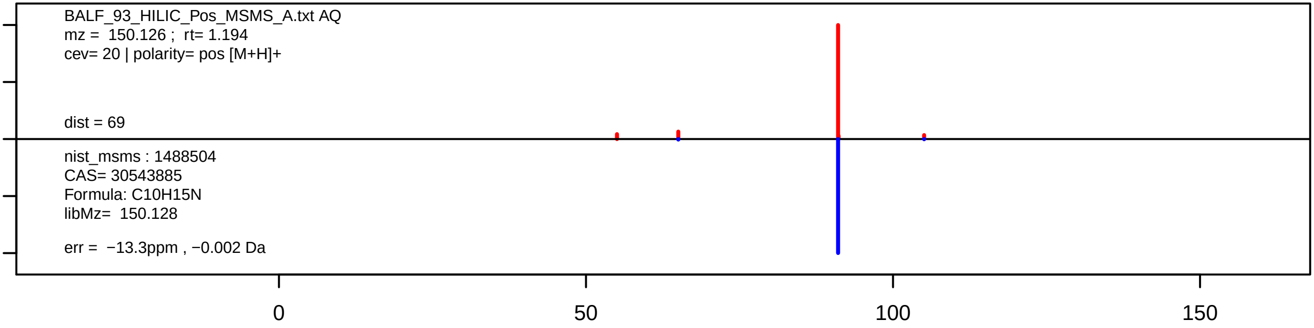

197 . 2−Methylamino−1−(3,4−methylenedioxyphenyl)propan−1−one
Score=894 Dot=911 prob=54.2

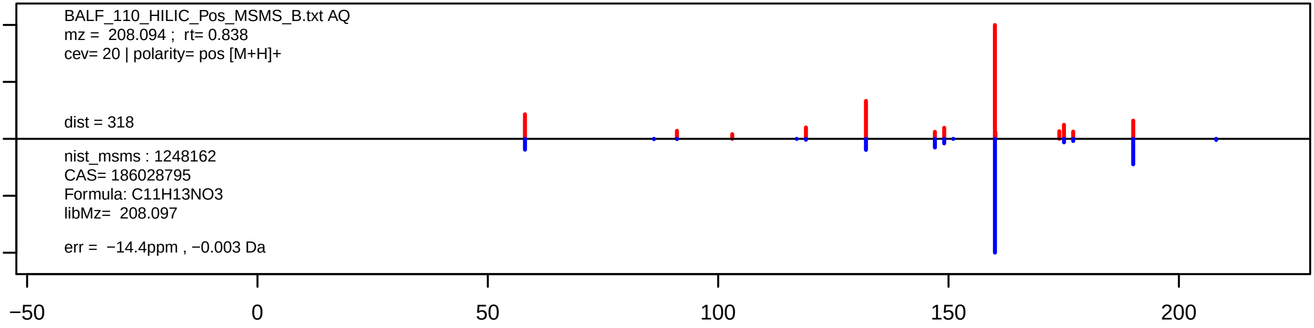

198 . 2−Methylamino−1−phenylbutane
Score=442 Dot=989 prob=8.8

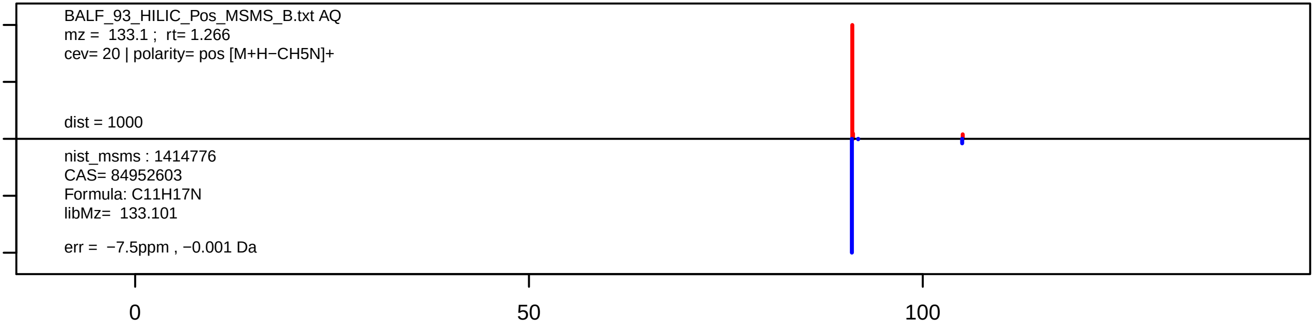

199 . 2−Oleoyl−1−palmitoyl−sn−glycero−3−phosphocholine
Score=400 Dot=999 prob=96.6

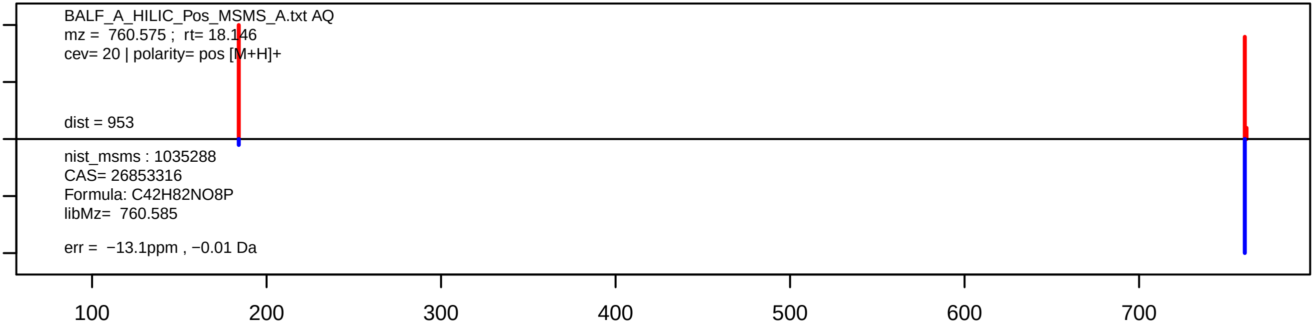

200 . 2,3−Methylenedioxymethcathinone
Score=352 Dot=900 prob=75.6

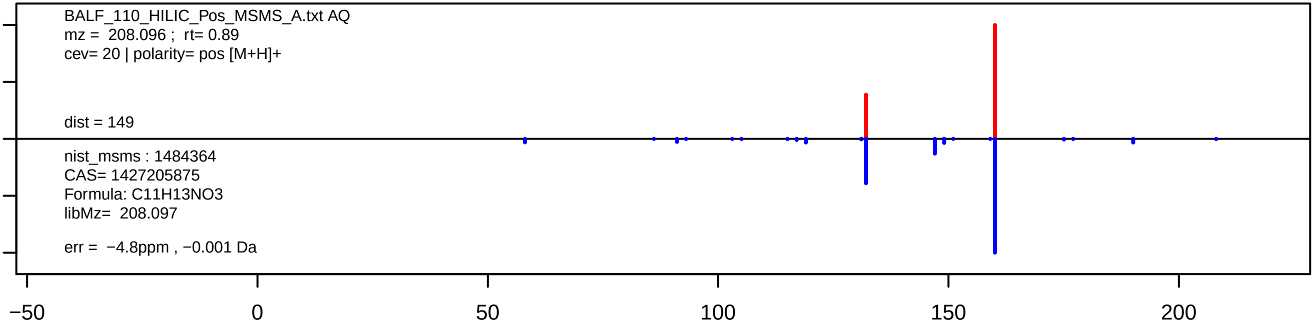

201 . 2,6−Pyridinediamine, N6−[2−[[4−(2,4−dichlorophenyl)−5−(1H−imidazol−2−yl)−2−pyrimidinyl]amino]ethyl]−3−nitro−
Score=360 Dot=804 prob=69

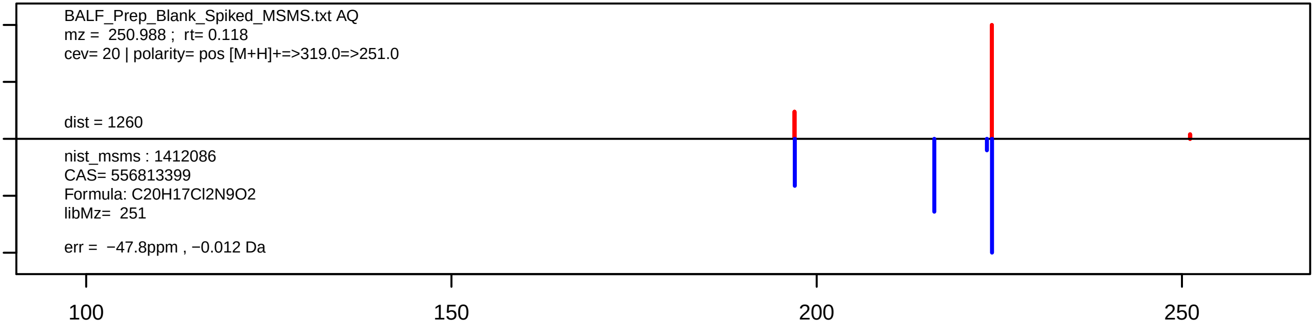

202 . 2'−Deoxyinosine
Score=294 Dot=817 prob=96.3

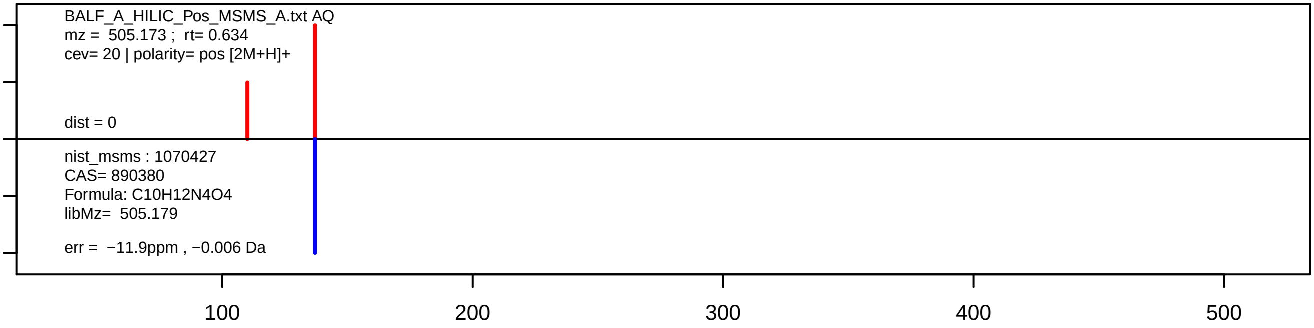

203 . 3−[N,N−Bis(2−hydroxyethyl)amino]−2−hydroxypropanesulfonic acid
Score=400 Dot=999 prob=24.9

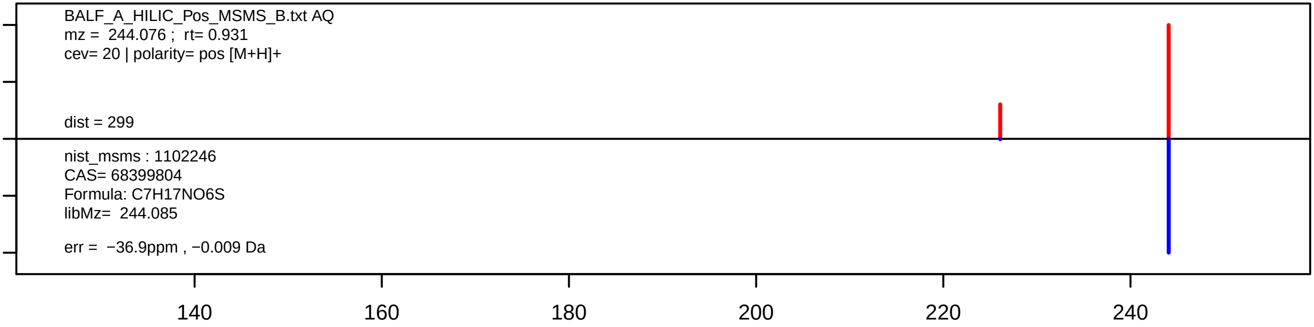

204 . 3−Aminopentanoic acid
Score=473 Dot=876 prob=75

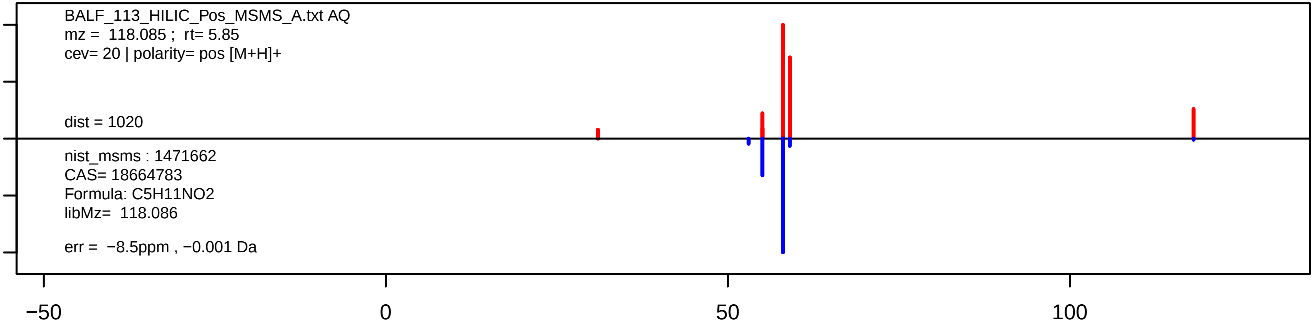

205 .   4−(Methylamino)−4−(3−pyridyl)butyric acid
Score=647 Dot=879 prob=86.2

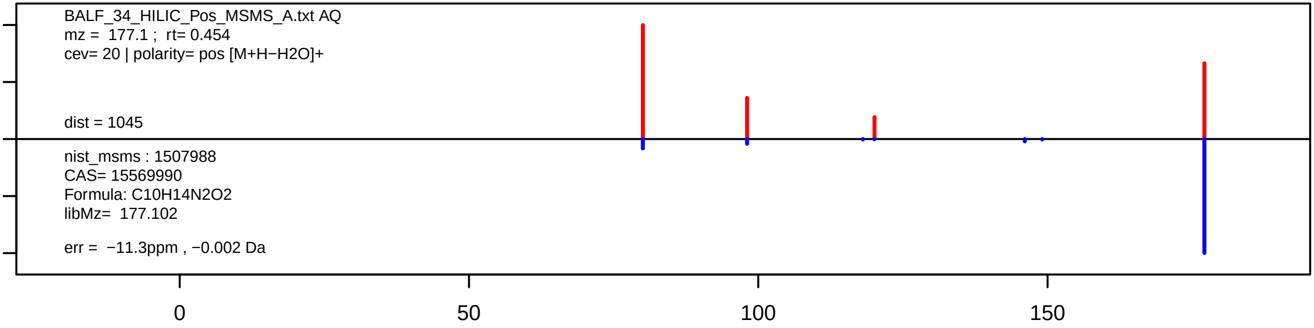

206 .   4−Imidazoleacrylic acid
Score=891 Dot=945 prob=98.3

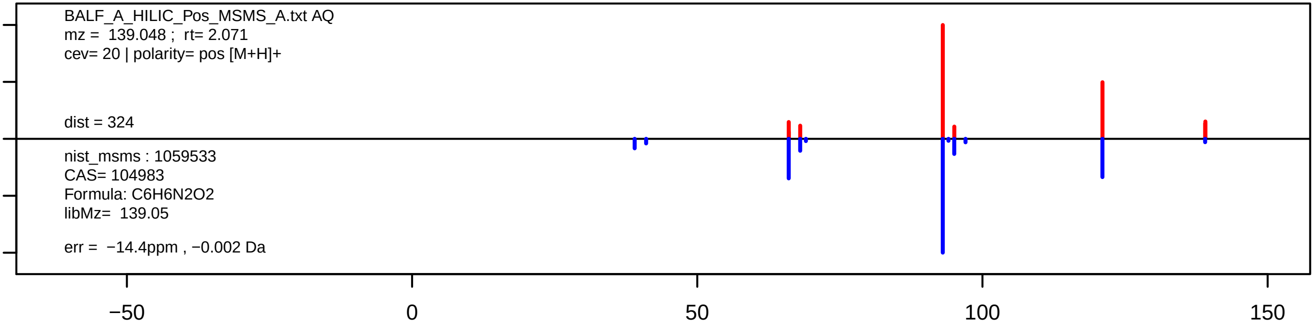

207 .   4−Phenylbutylamine
Score=291 Dot=837 prob=92

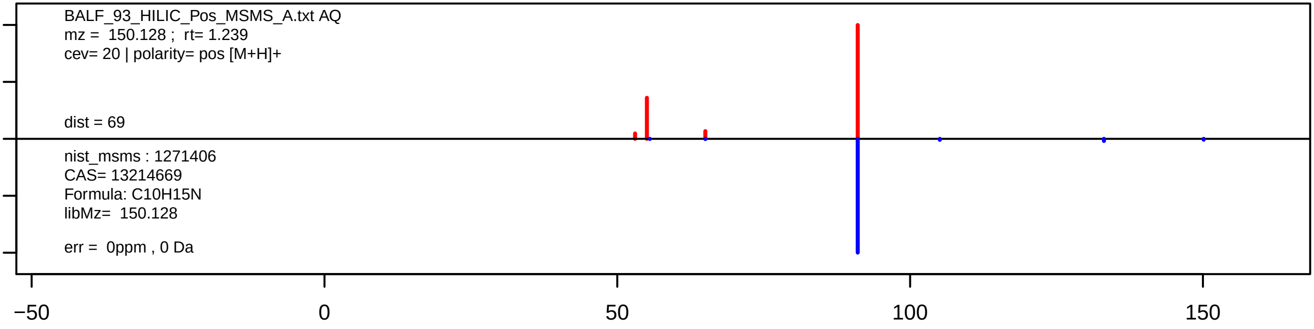

208 . 4.beta.−Galactobiose
Score=400 Dot=999 prob=28

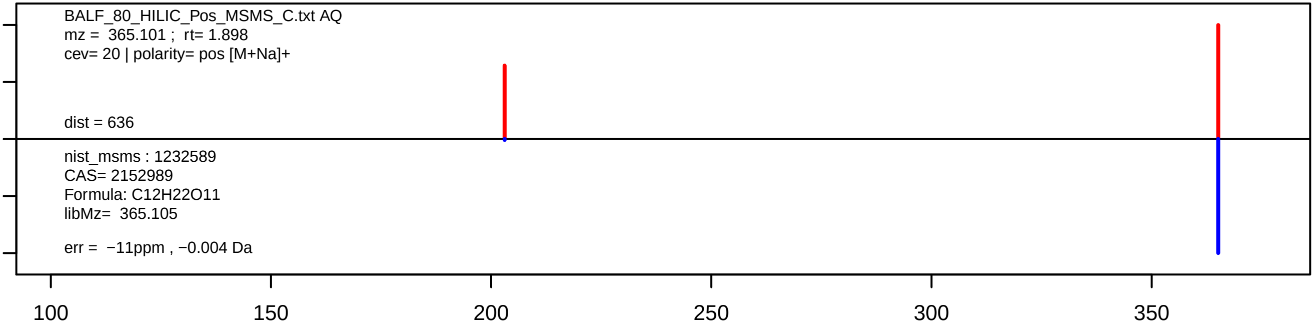

209 . 5−Aminovaleric acid
Score=746 Dot=887 prob=98.4

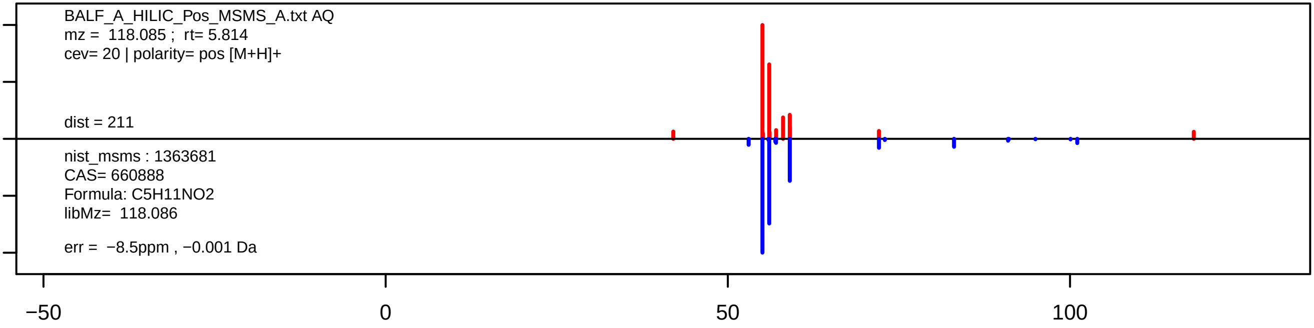

210 . 5,5−Dimethylimidazolidine−2,4−dione
Score=400 Dot=999 prob=3.5

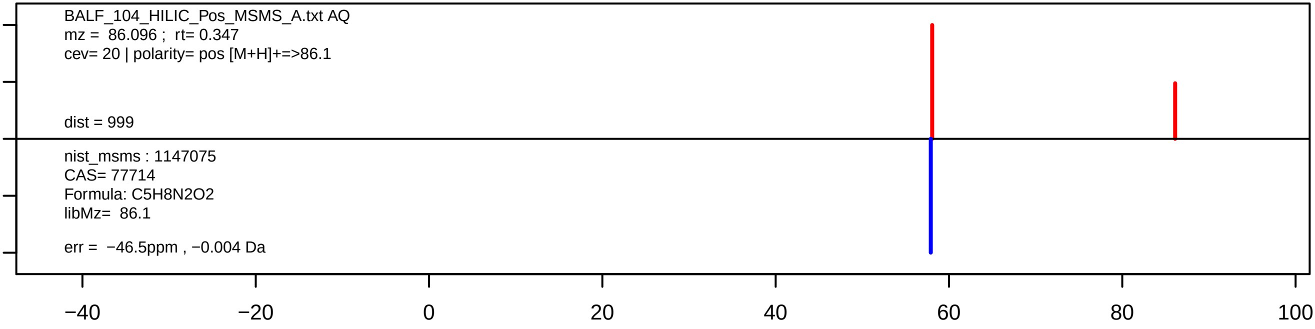

211 . 5.alpha.−Androstan−17.beta.−ol−3−one
Score=400 Dot=999 prob=32.9

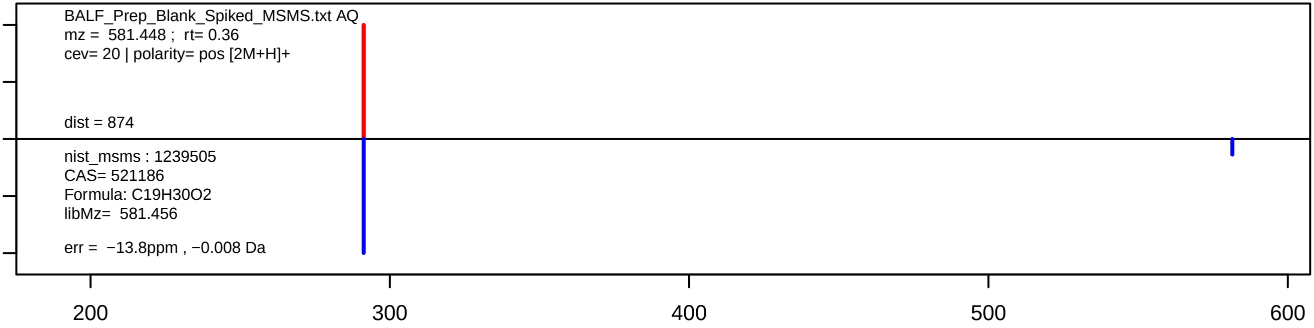

212 . 5'−S−Methyl−5'−thioadenosine
Score=159 Dot=803 prob=70.3

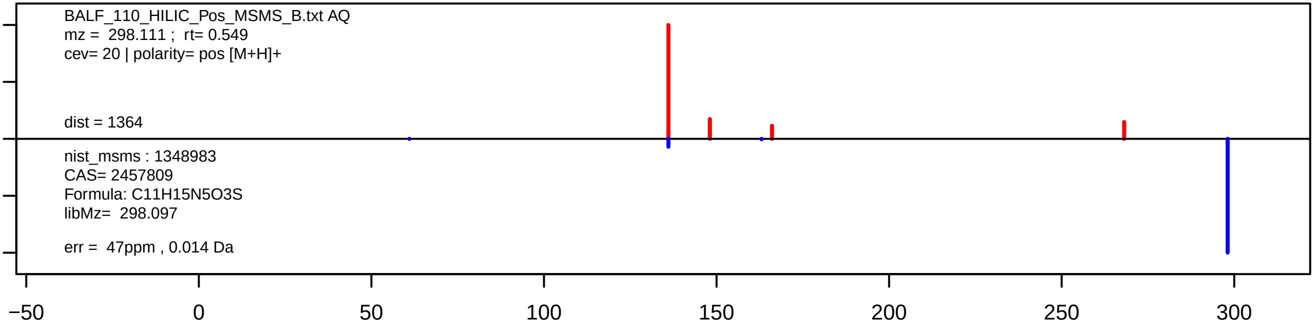

213 . 7H−Pyrrolo[2,3−g]benzothiazol−7−one, 6,8−dihydro−8−(1H−imidazol−5−ylmethylene)−
Score=415 Dot=969 prob=95.2

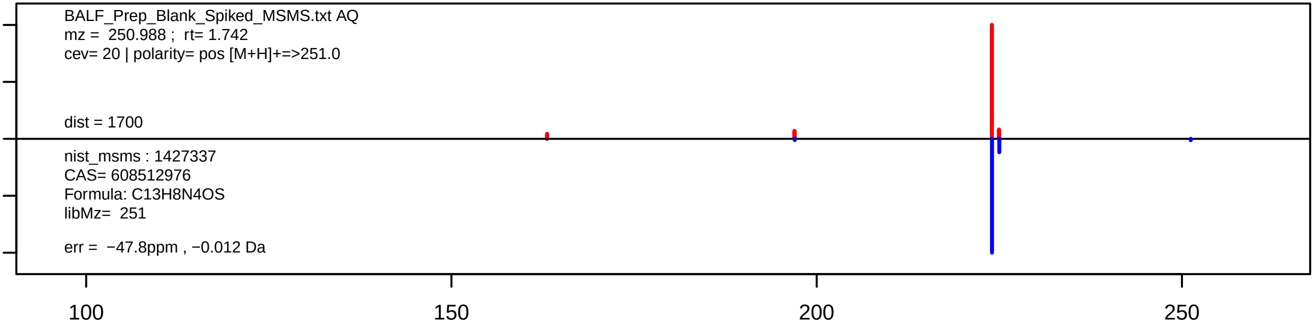

214 . Acetyl−DL−carnitine
Score=828 Dot=989 prob=76.6

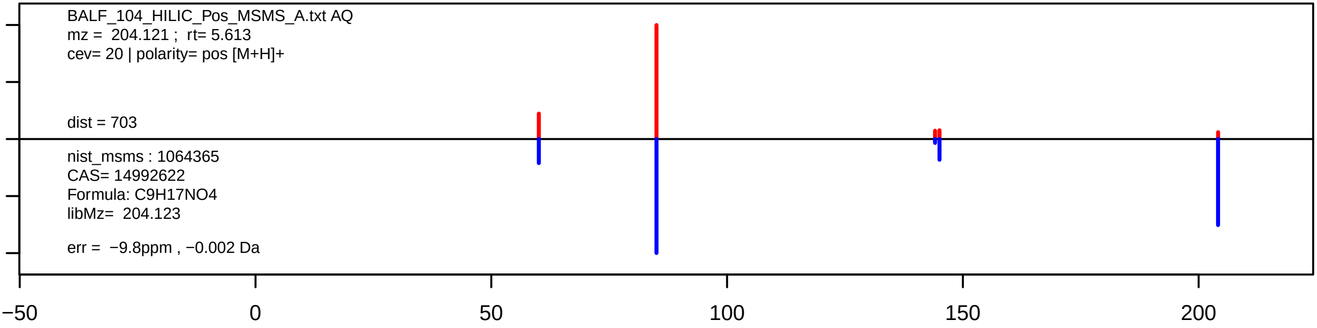

215 . Acetyl−L−carnitine
Score=842 Dot=998 prob=65.1

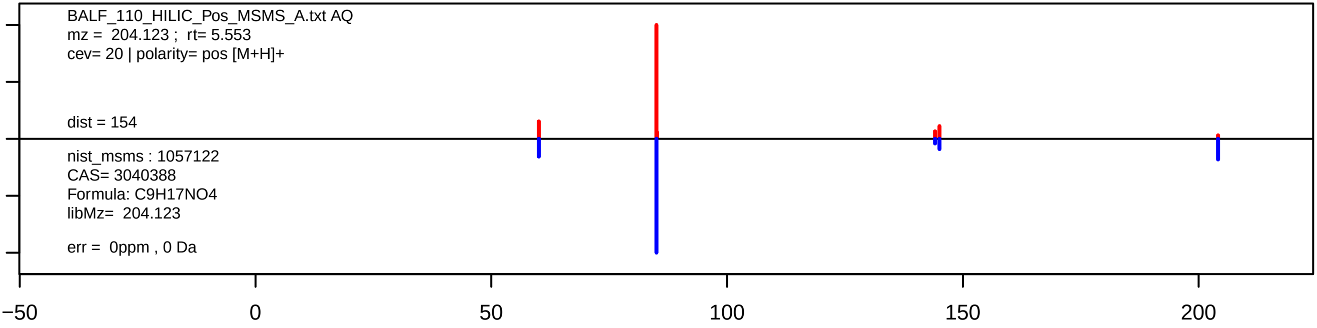

216 . Adenosine
Score=312 Dot=834 prob=78.3

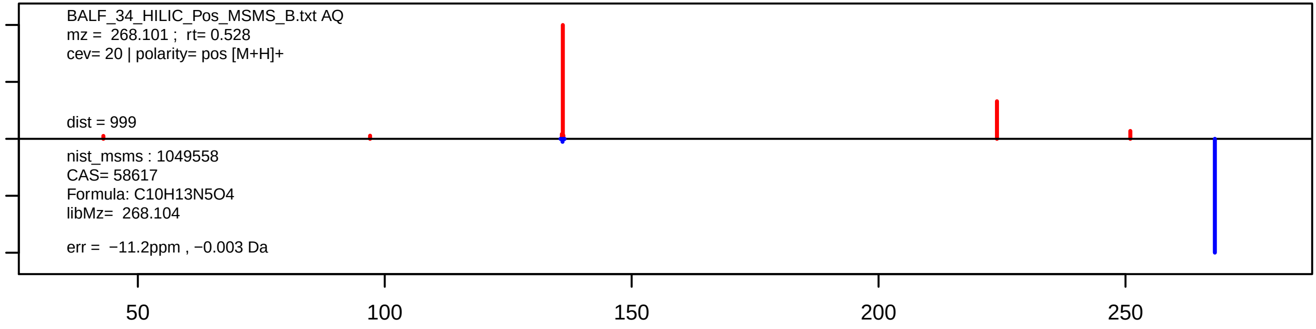

217 . Ala−His
Score=381 Dot=892 prob=94.5

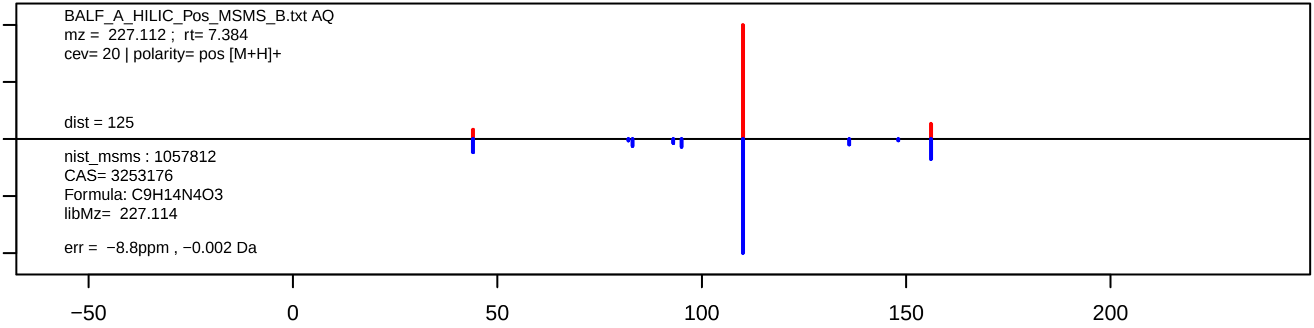

218 . Ala−Lys
Score=563 Dot=908 prob=49

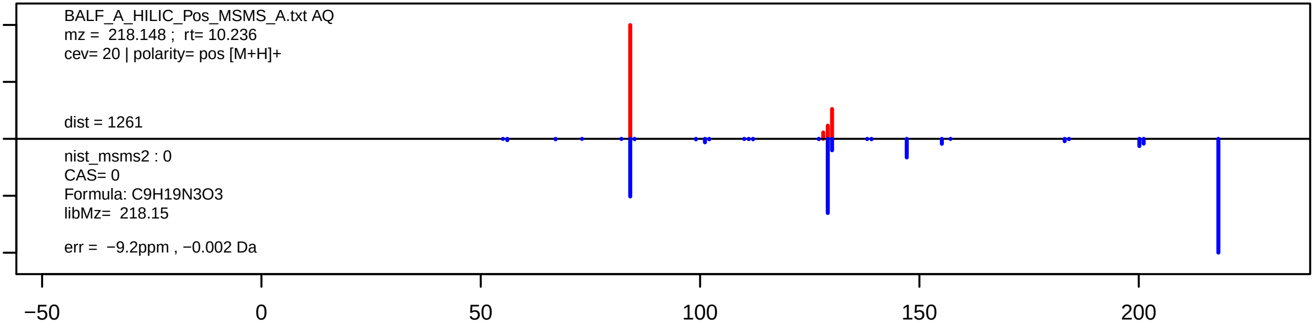

219 . Ala−Phe
Score=299 Dot=801 prob=88.3

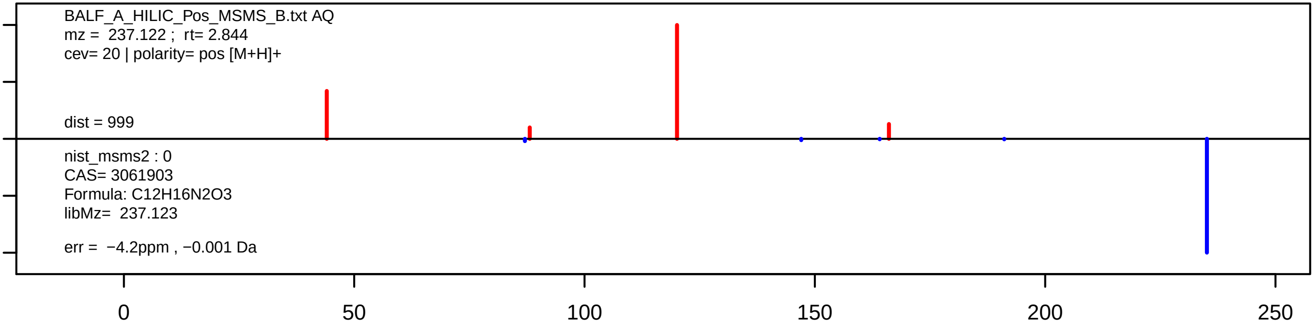

220 .   Albuterol
Score=344 Dot=905 prob=98.3

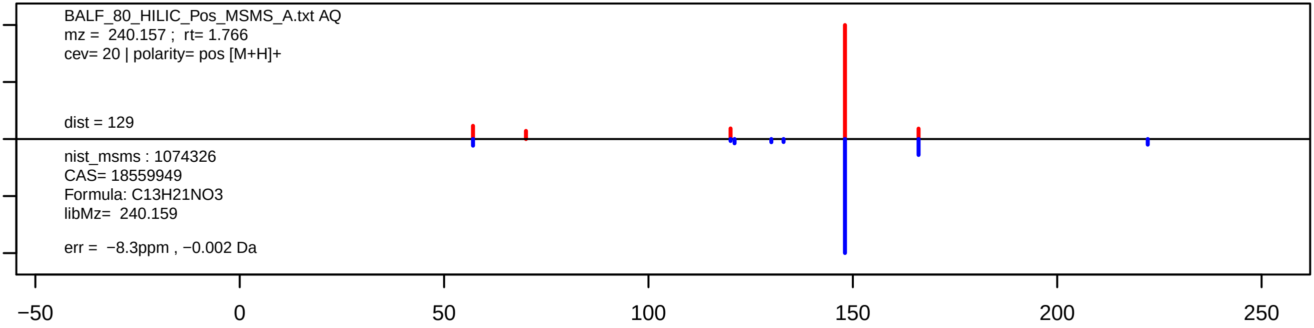

221 .   Allopurinol
Score=512 Dot=815 prob=72.1

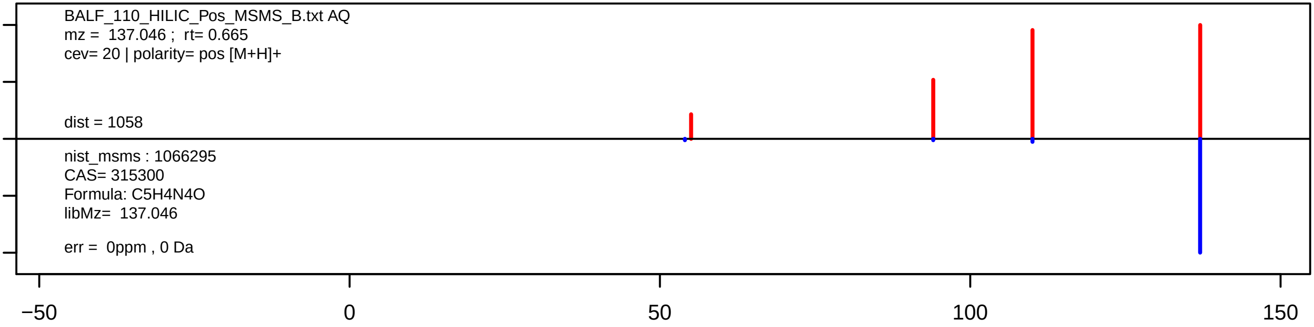

222 .   Allopurinol riboside
Score=400 Dot=999 prob=49.1

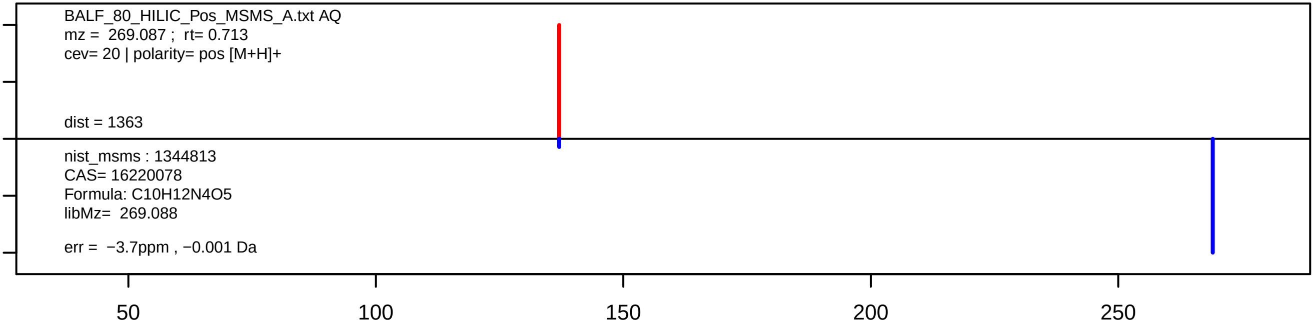

223 . Aminodiphenylmethane
Score=851 Dot=965 prob=52.9

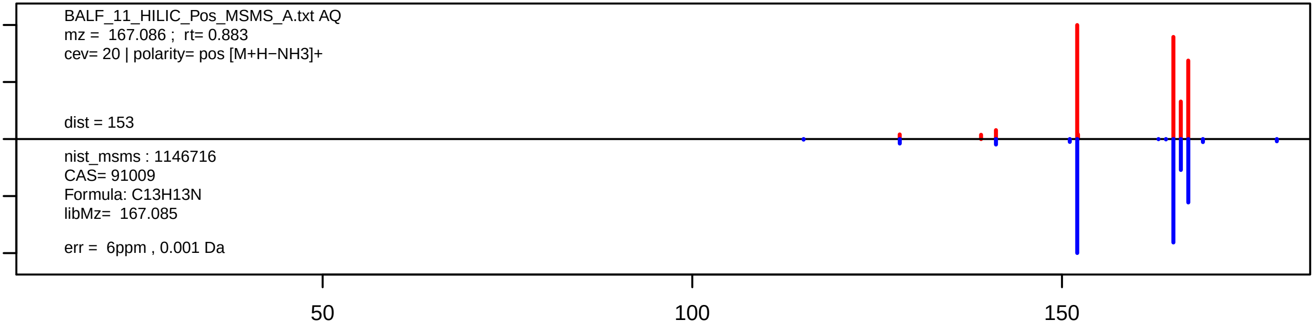

224 . Arg−Leu
Score=553 Dot=818 prob=48.7

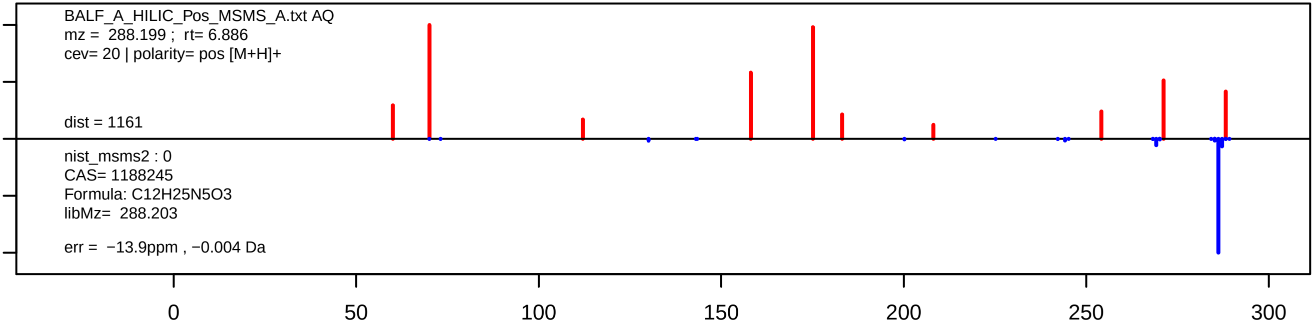

225 . Arg−Val
Score=551 Dot=810 prob=49.7

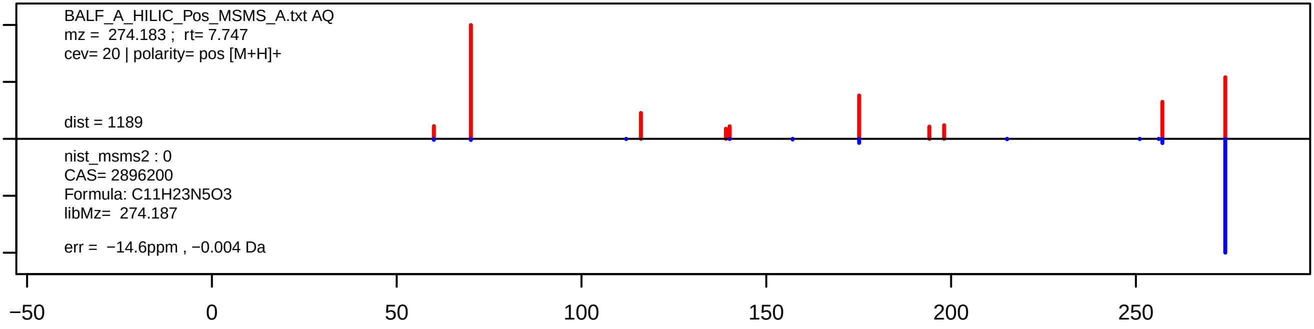

226 . Benzhydrol
Score=914 Dot=970 prob=39.9

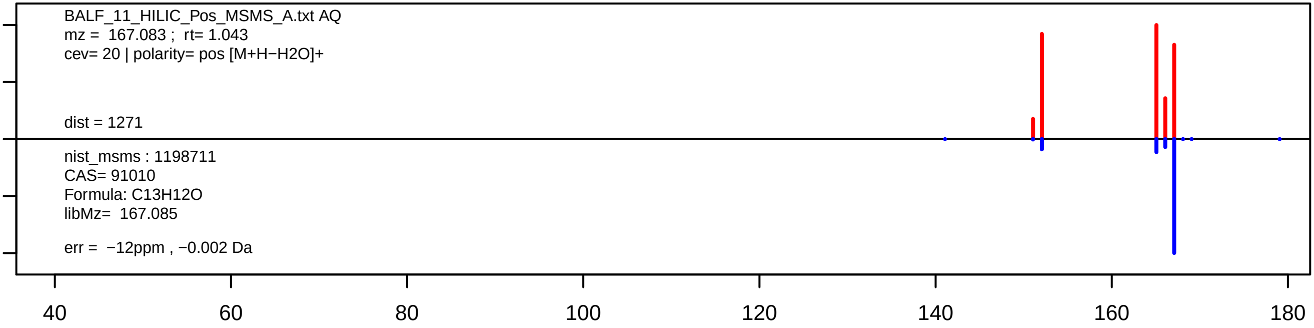

227 . Benzyl alcohol
Score=668 Dot=972 prob=92.1

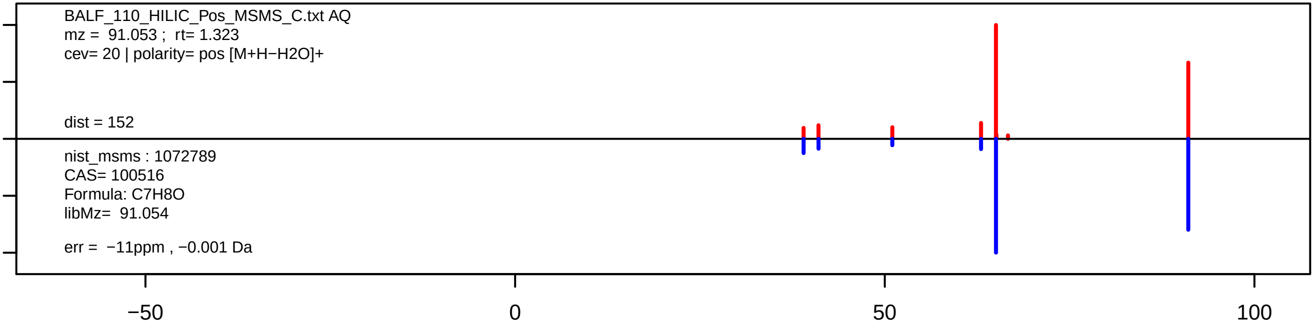

228 . Betaine
Score=557 Dot=999 prob=98.5

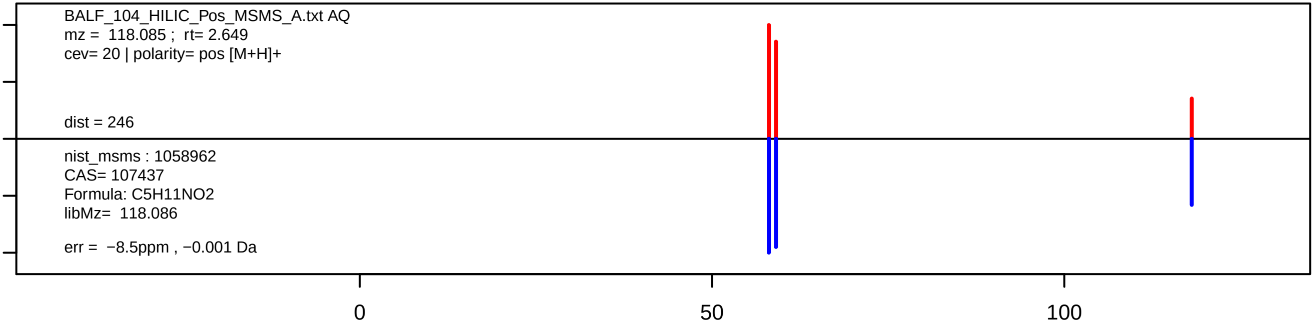

229 . Bis(2−ethylhexyl) phthalate
Score=766 Dot=947 prob=68.2

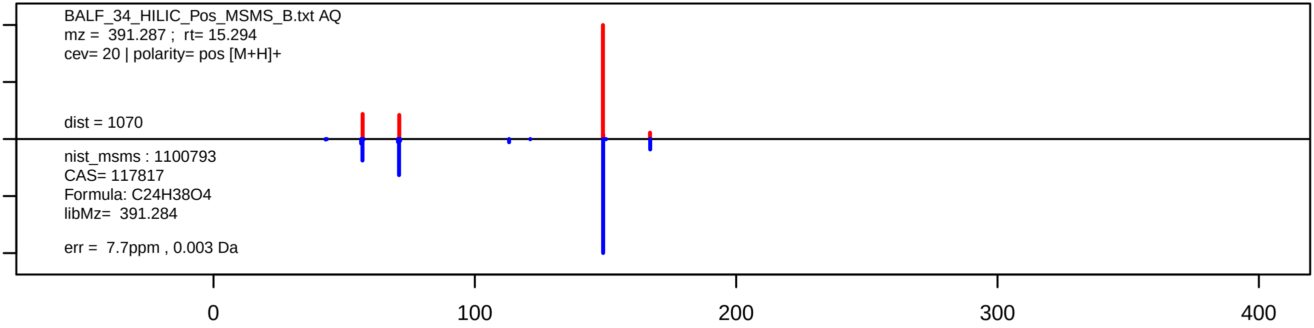

230 . Bupropion
Score=692 Dot=861 prob=97.9

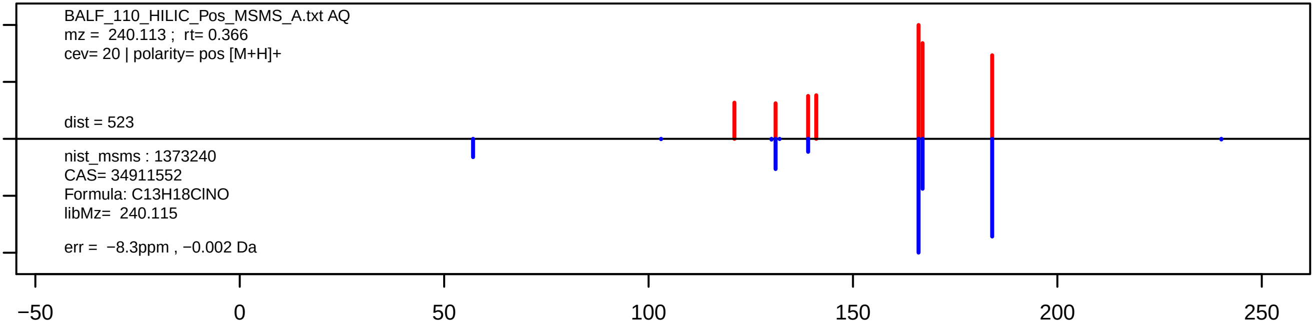

231 . Carbamic acid, N−(2,2−diphenylacetyl)−, ethyl ester
Score=720 Dot=961 prob=75

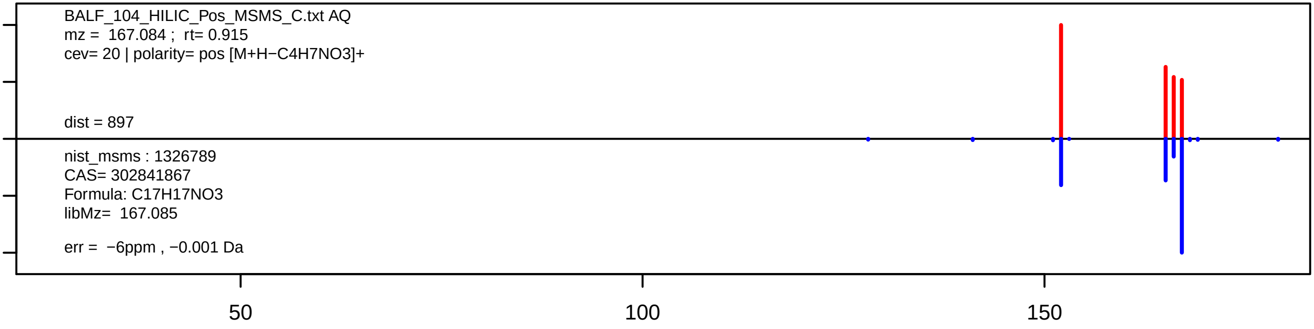

232 . Choline cation
Score=890 Dot=932 prob=93.7

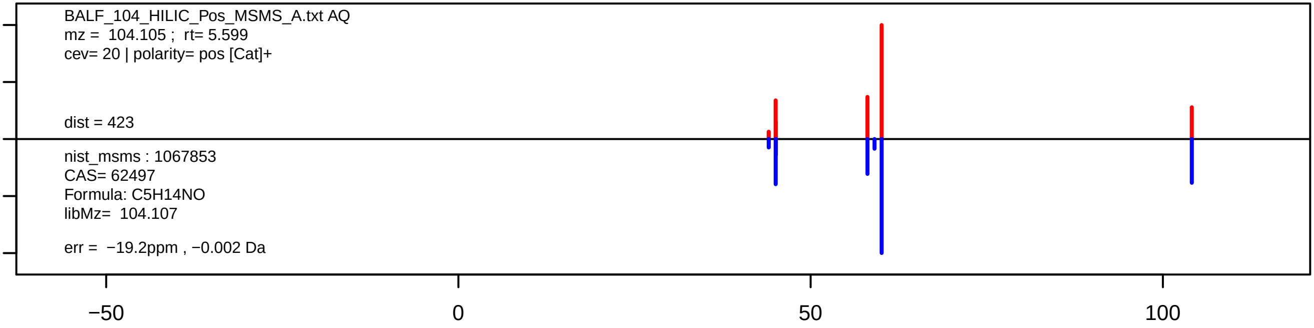

233 . Citalopram
Score=508 Dot=855 prob=94.5

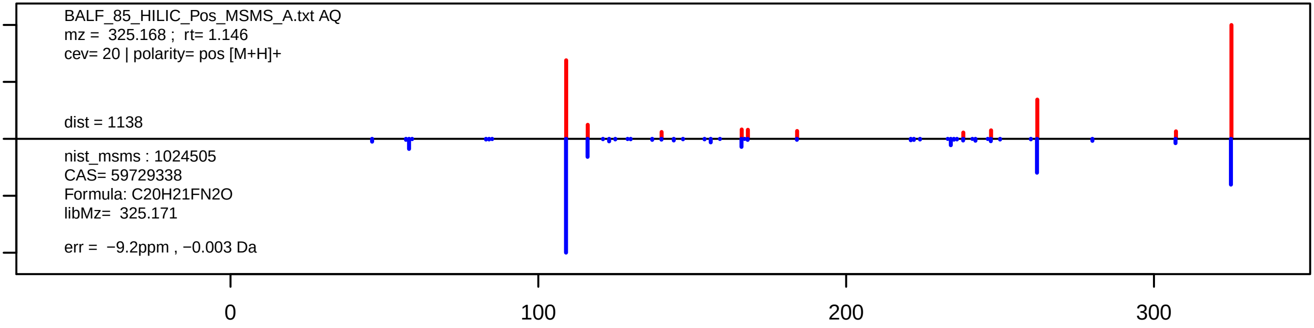

234 . Creatinine
Score=324 Dot=937 prob=94.3

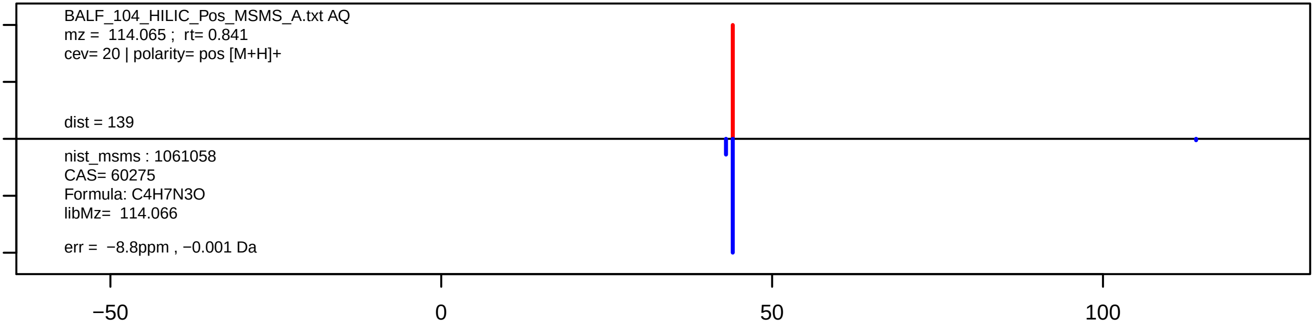

235 . Cumylamine
Score=321 Dot=933 prob=17.8

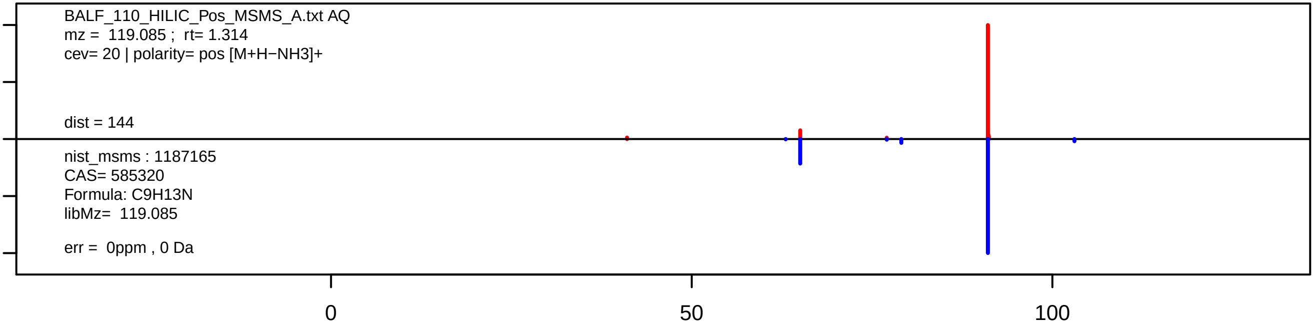

236 . Cyclizine
Score=526 Dot=980 prob=86.9

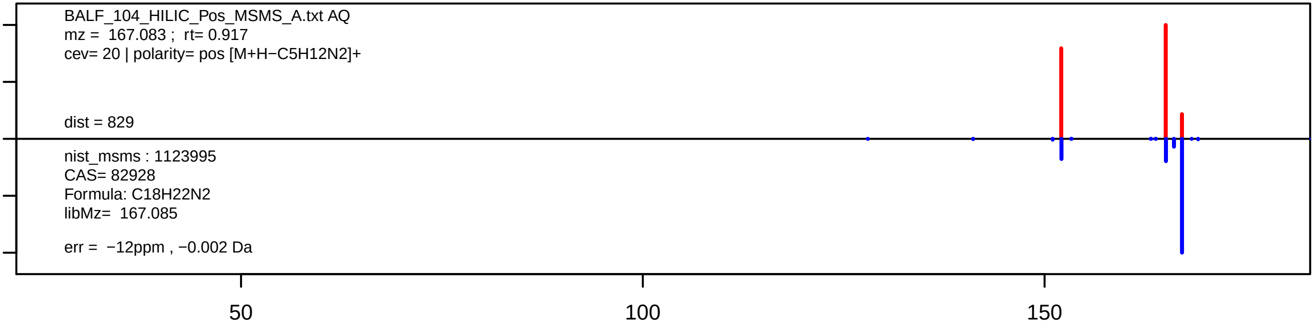

237 . Cyclohexylamine
Score=641 Dot=947 prob=91.1

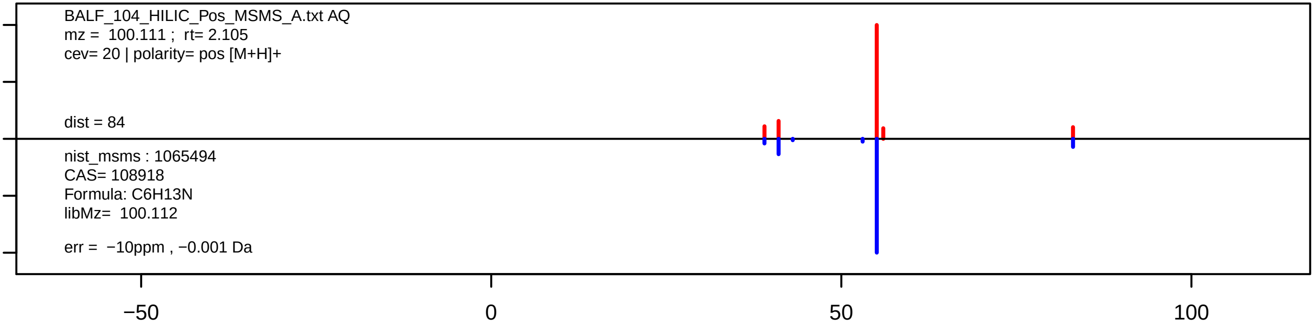

238 .   D−(+)−Amphetamine
Score=399 Dot=999 prob=19.7

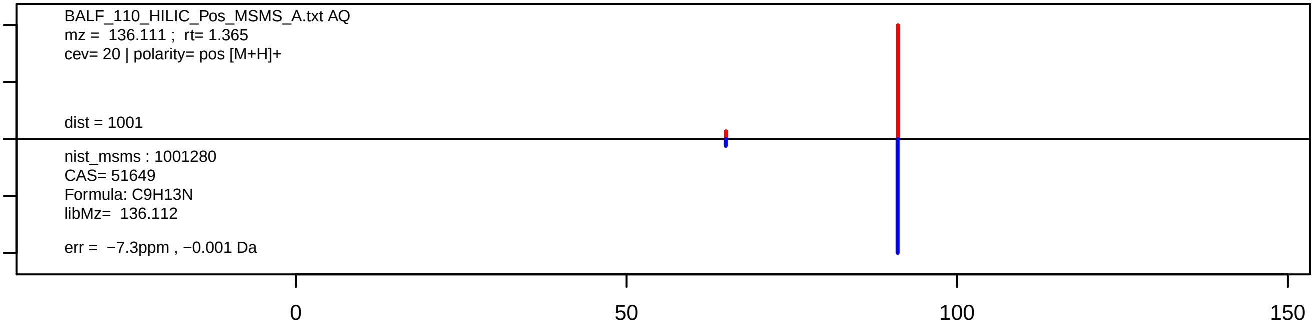

239 .   D−Pipecolinic acid
Score=469 Dot=957 prob=82.8

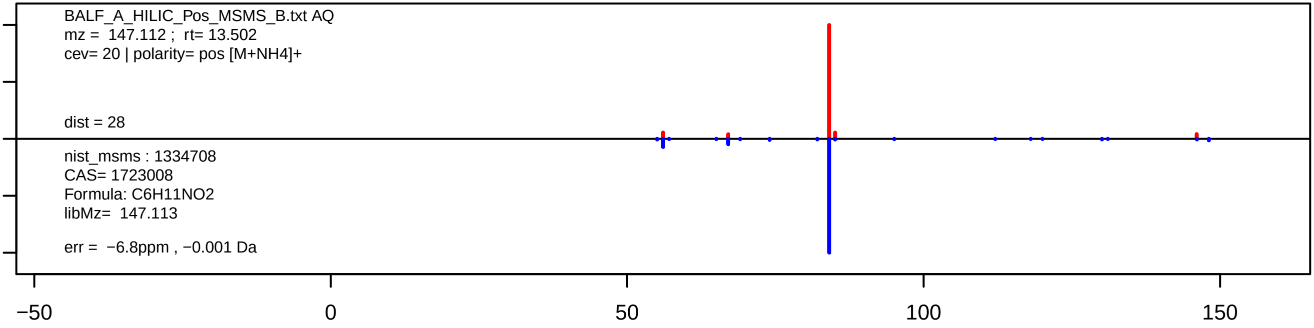

240 .   Dextromethorphan
Score=628 Dot=858 prob=96

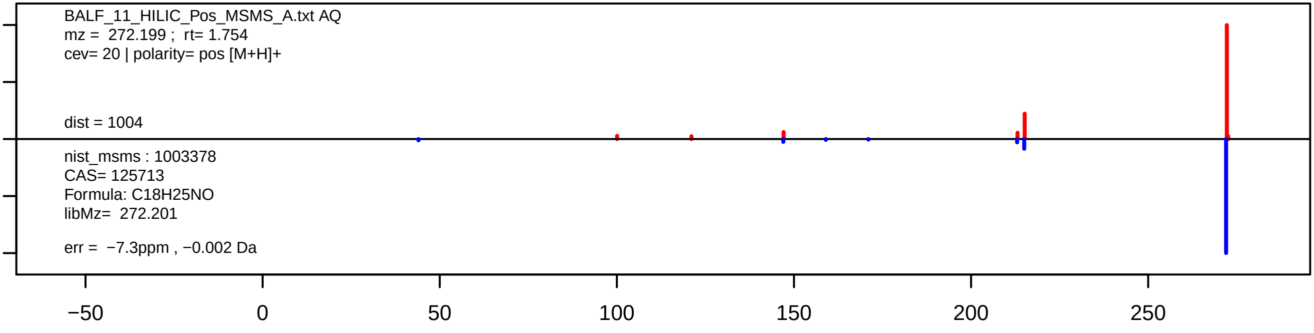

241 . Di(2−nonyl) phthalate
Score=373 Dot=994 prob=34.9

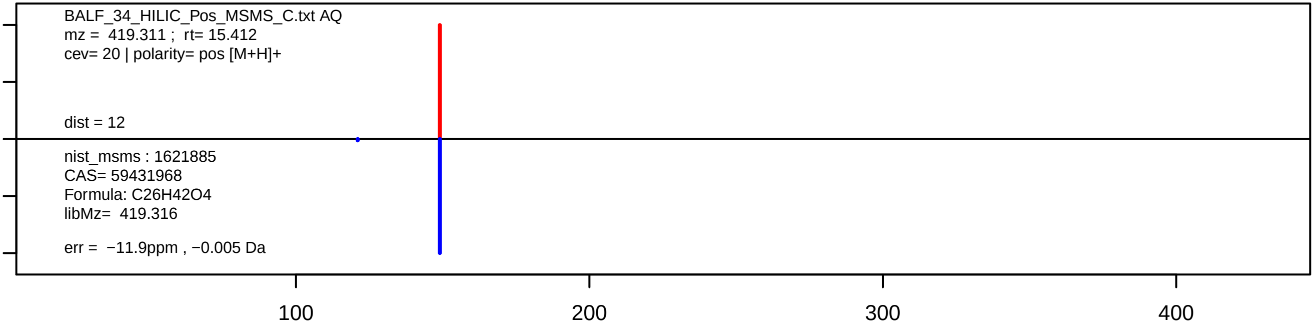

242 . Di(3,7−dimethyl−1−octyl) phthalate
Score=634 Dot=849 prob=92

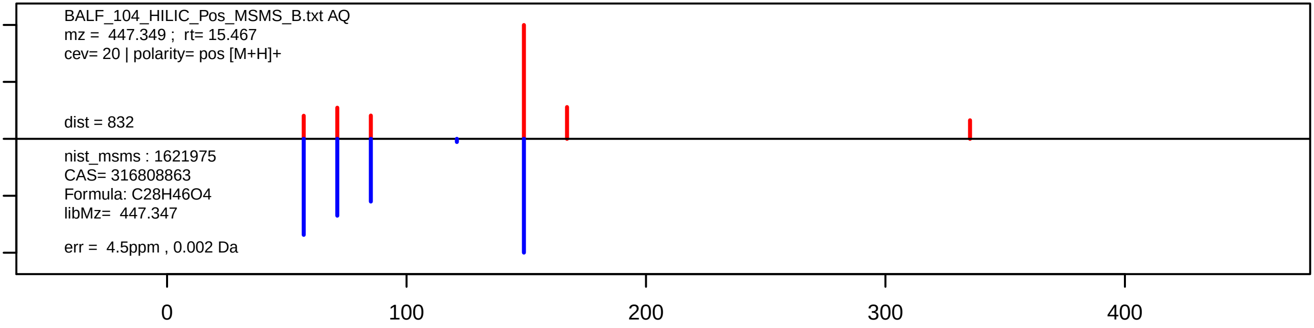

243 . Dibenzylamine
Score=186 Dot=929 prob=81.8

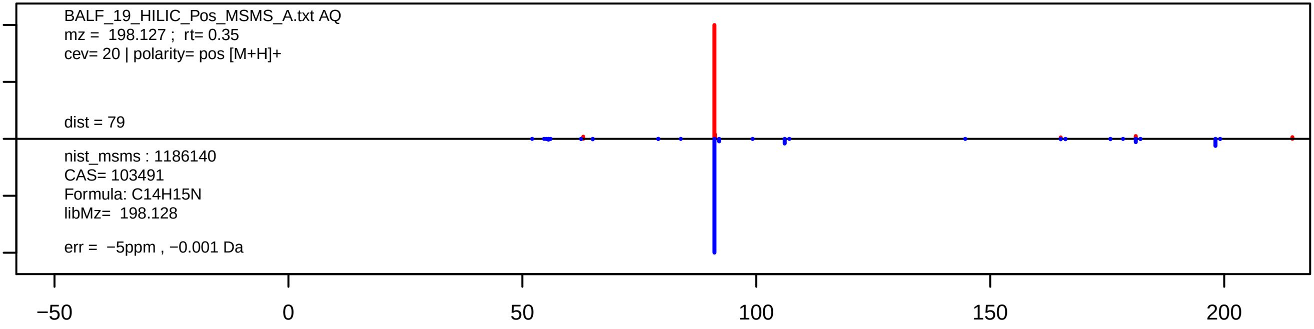

244 . Dicyclohexylamine
Score=508 Dot=991 prob=69.4

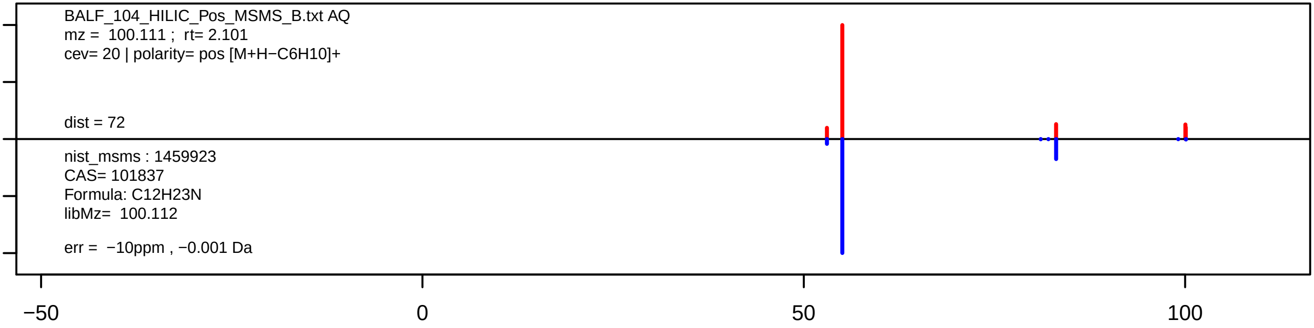

245 . Diethanolamine
Score=795 Dot=889 prob=99

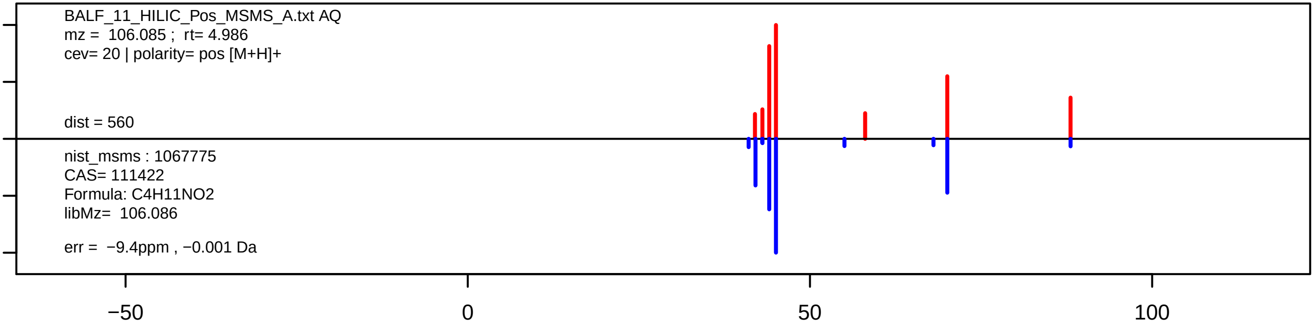

246 . Diisodecyl phthalate
Score=296 Dot=826 prob=59.6

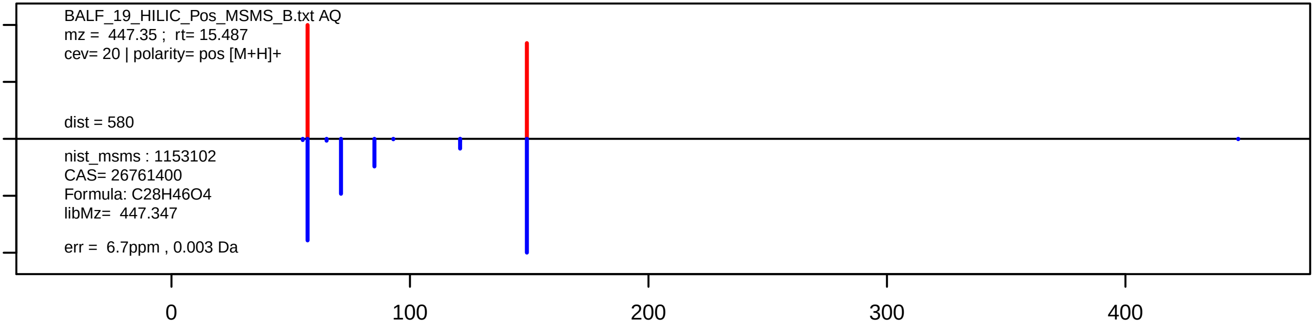

247 . Diisooctyl phthalate
Score=860 Dot=975 prob=41

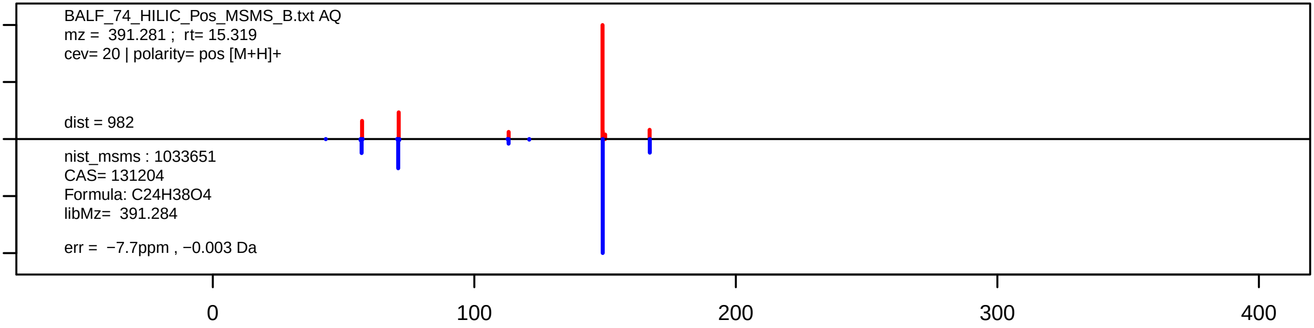

248 . Dioctyl phthalate
Score=734 Dot=976 prob=98.9

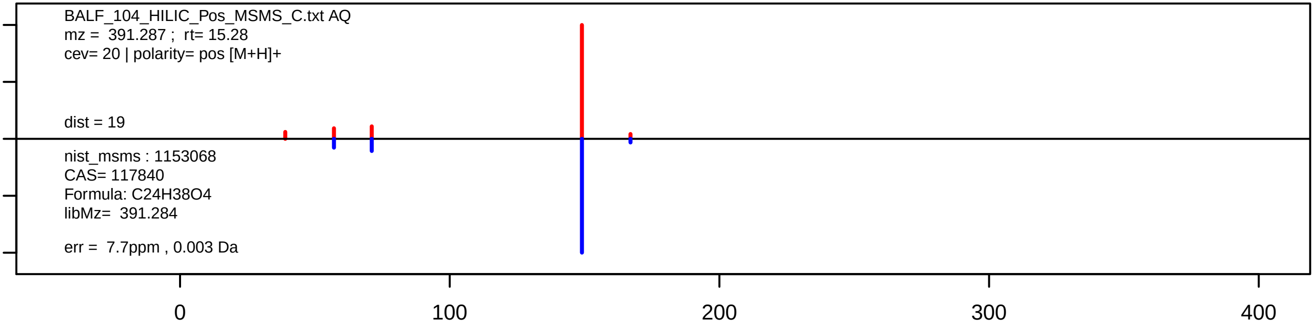

249 . Diphenhydramine
Score=523 Dot=965 prob=98.4

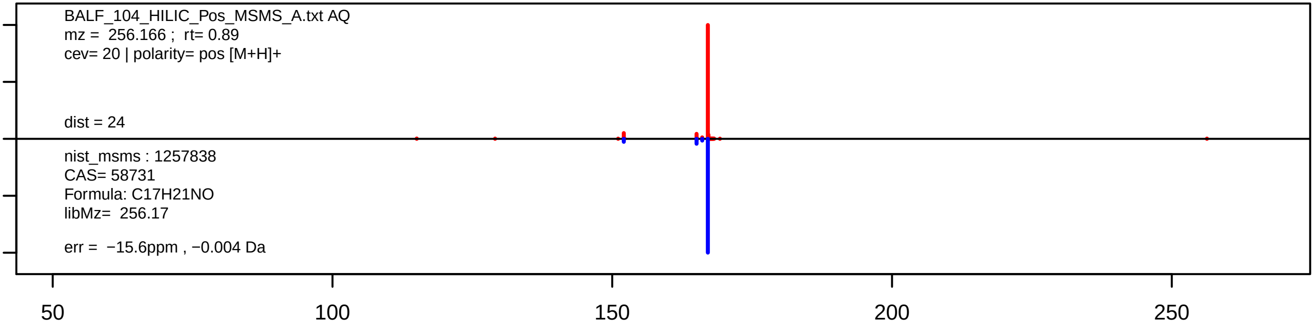

250 . DL−Arginine
Score=687 Dot=913 prob=78.2

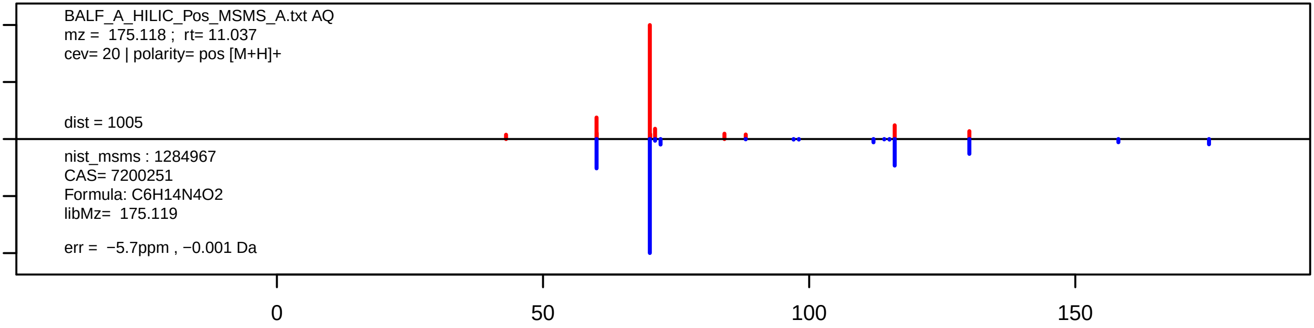

251 . DL−o−Tyrosine
Score=780 Dot=887 prob=98.3

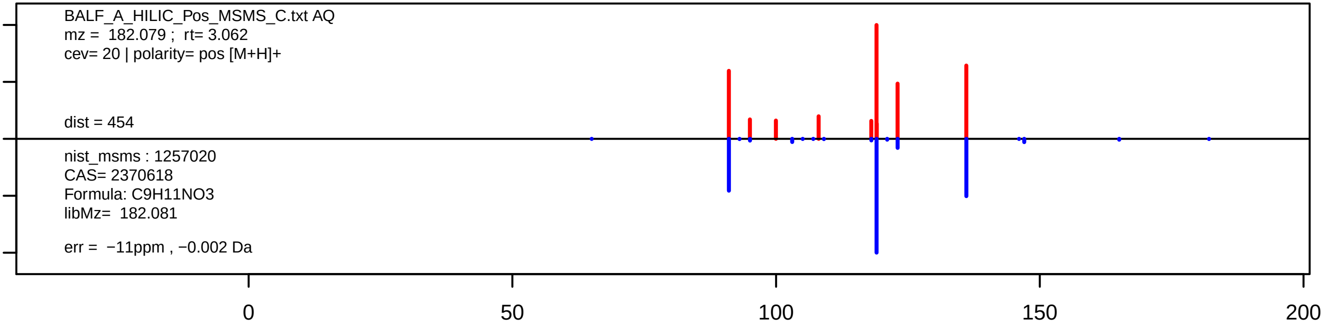

252 . DL−Ornithine
Score=369 Dot=992 prob=49.5

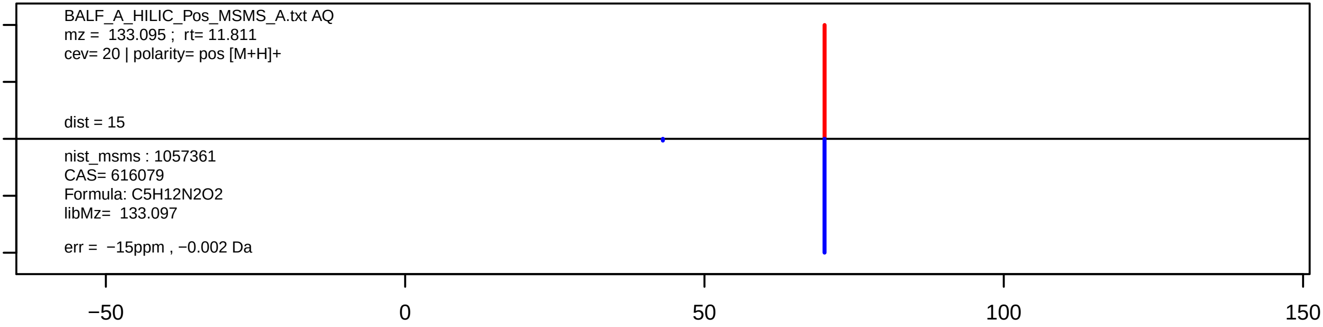

253 .   DL−Phenylalanine
Score=520 Dot=922 prob=96.3

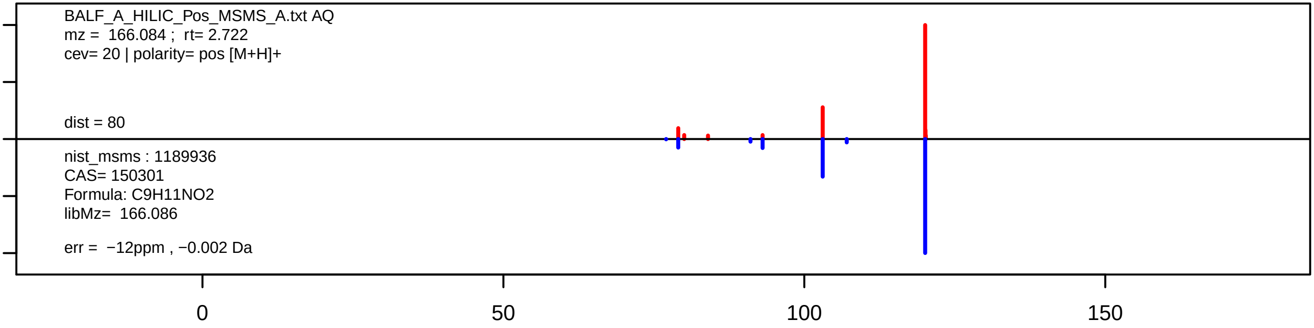

254 .   Fluoxetine
Score=300 Dot=943 prob=98.5

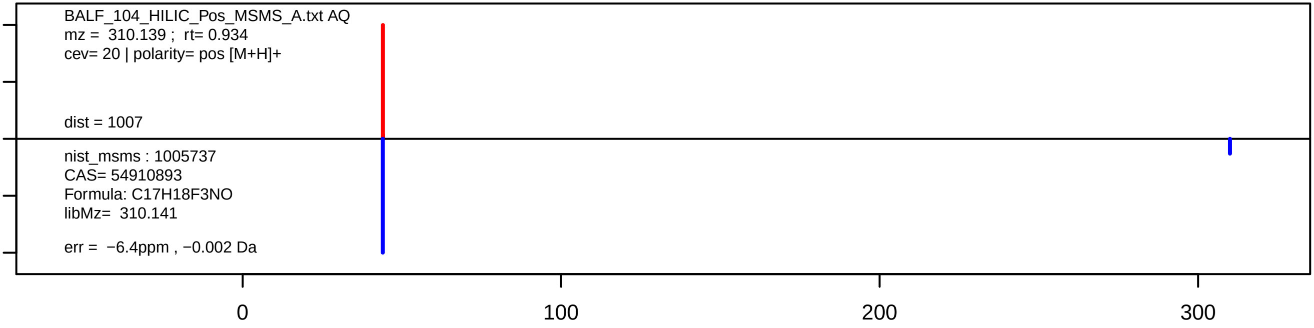

255 .   Gabapentin
Score=780 Dot=914 prob=98.8

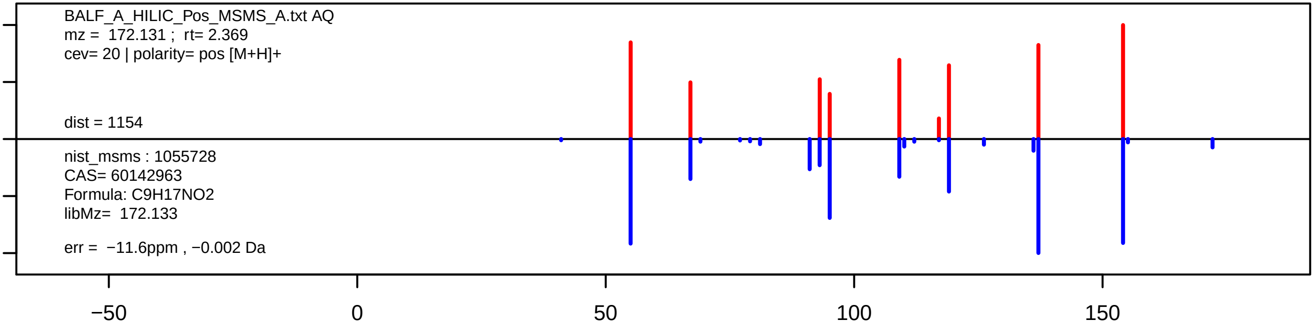

256 . Geranyl pyrophosphate
Score=298 Dot=979 prob=97.9

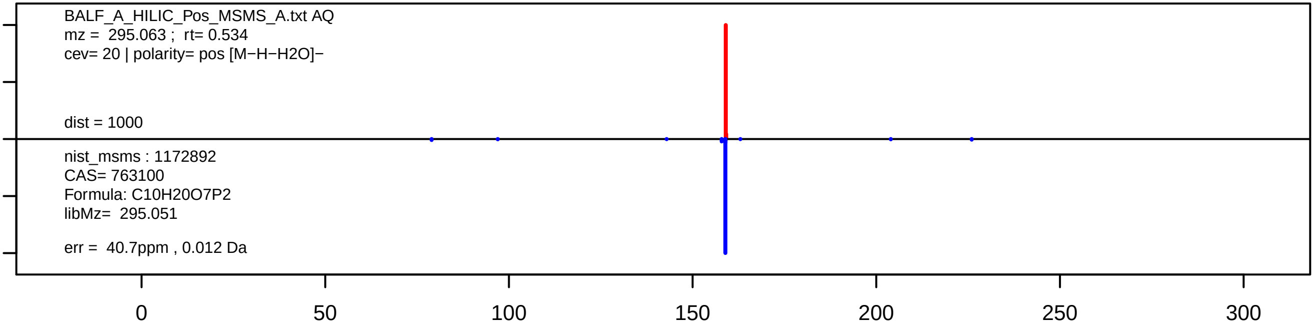

257 . Glycerophosphocholine
Score=803 Dot=926 prob=93.2

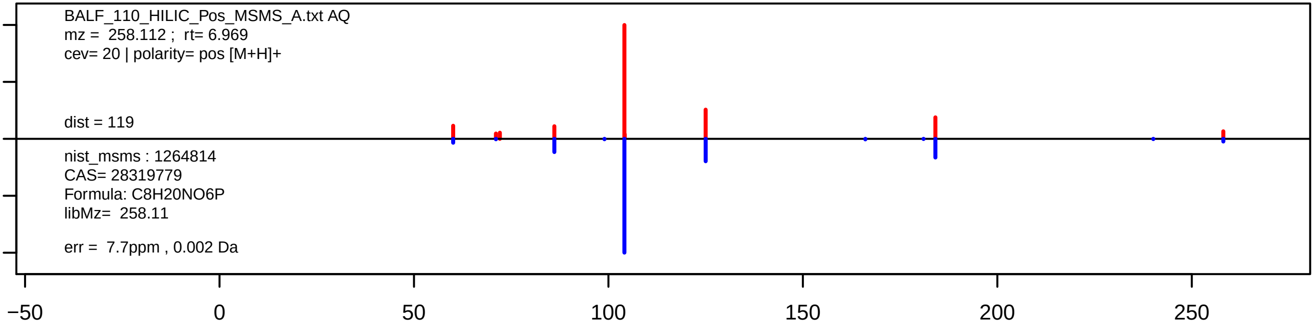

258 . Guanine
Score=508 Dot=935 prob=73.8

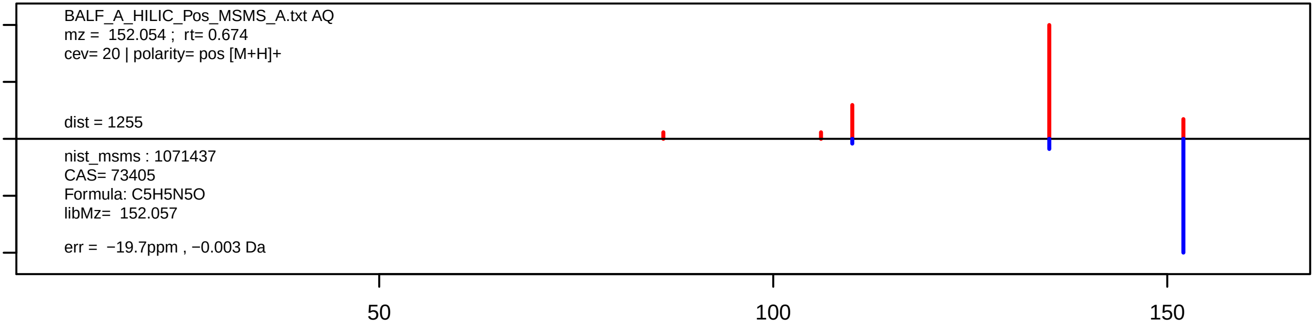

259 . Hexanoyl coenzyme A
Score=189 Dot=807 prob=63.6

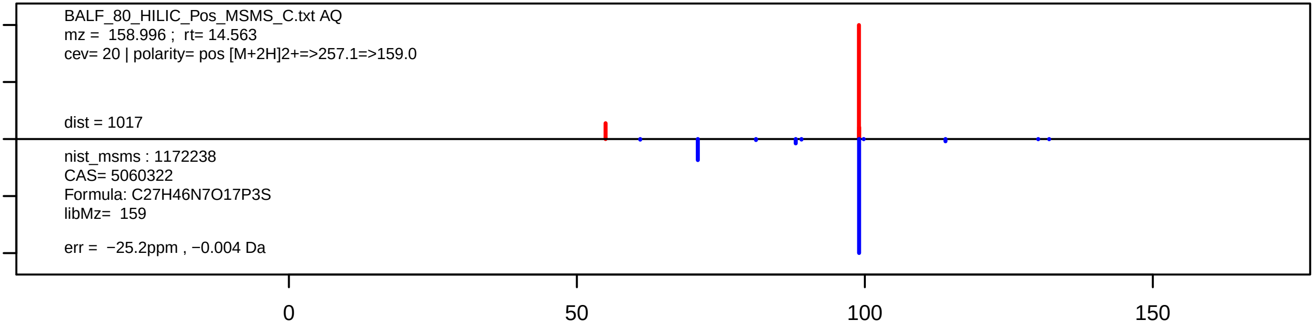

260 . His−.beta.−Ala
Score=123 Dot=803 prob=31.4

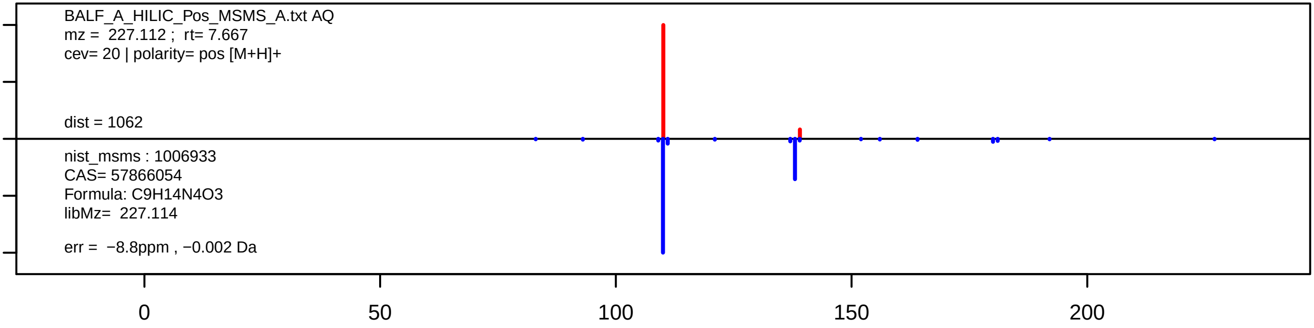

261 . His−Ala
Score=361 Dot=923 prob=31.6

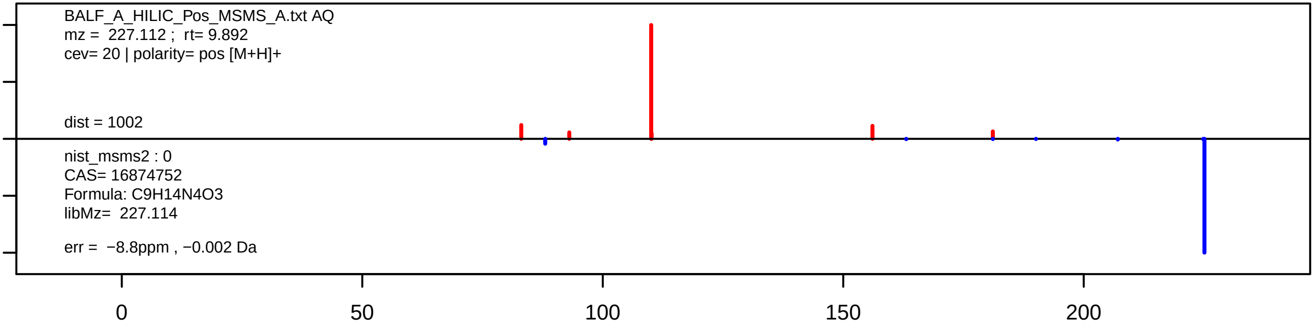

262 . Hydroxybupropion
Score=861 Dot=868 prob=98.3

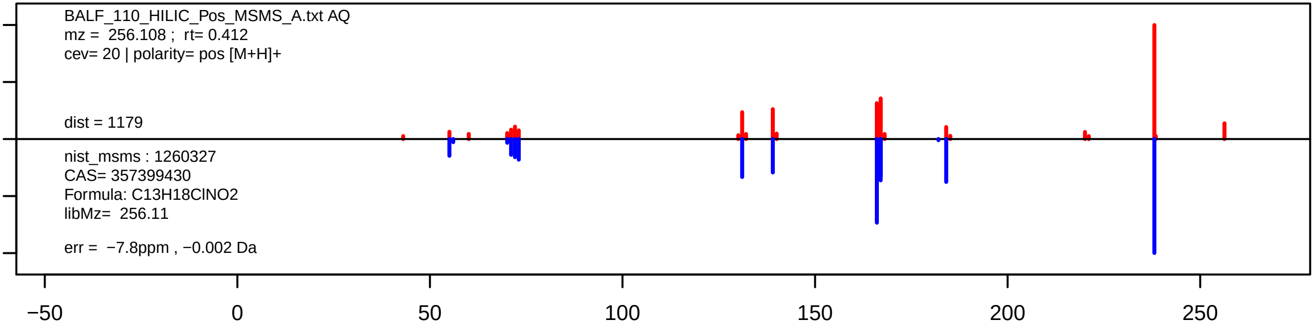

263 . Hydroxyebastine
Score=889 Dot=937 prob=30.9

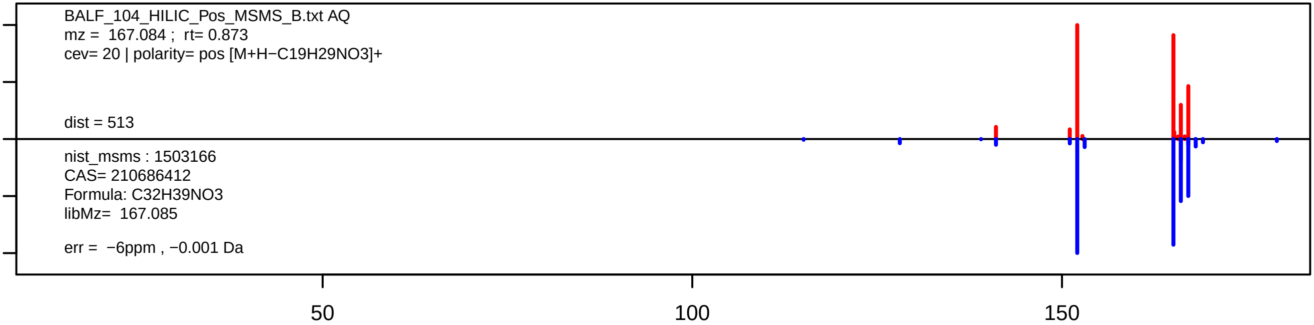

264 . Hydroxyzine
Score=275 Dot=965 prob=95.8

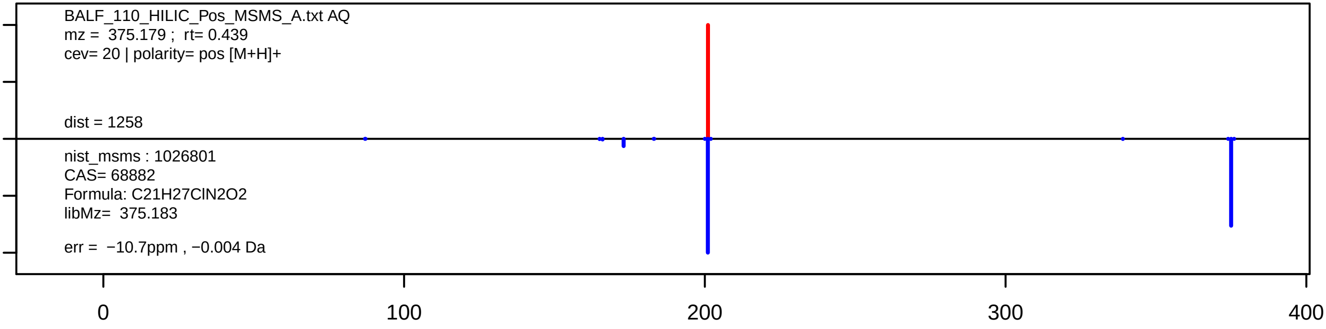

265 . Hypoxanthine
Score=689 Dot=833 prob=90.7

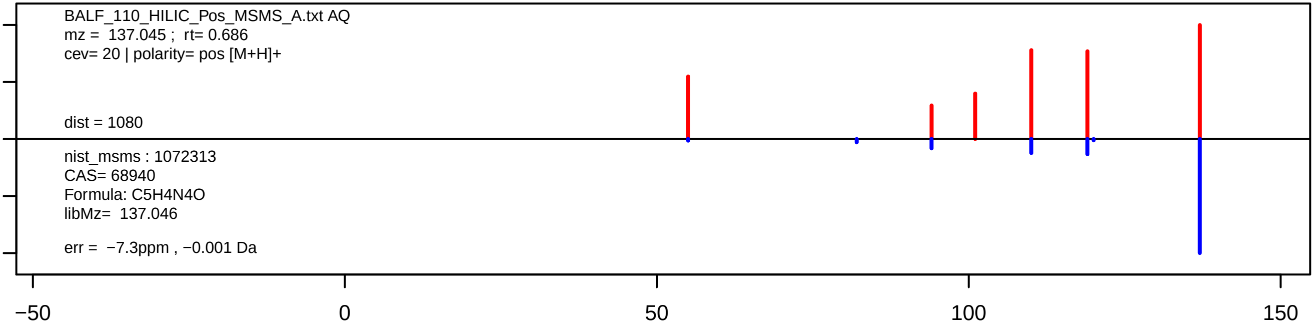

266 . Ile−Ala
Score=418 Dot=806 prob=68.2

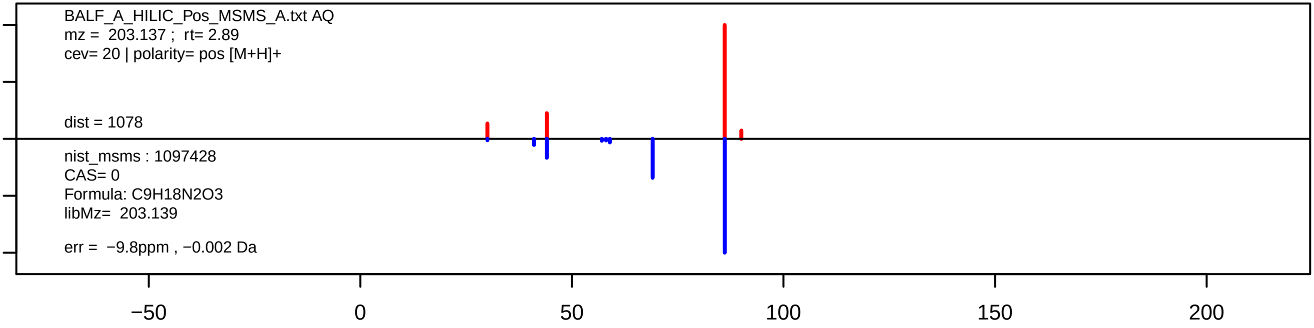

267 . Ile−Arg
Score=524 Dot=886 prob=98.6

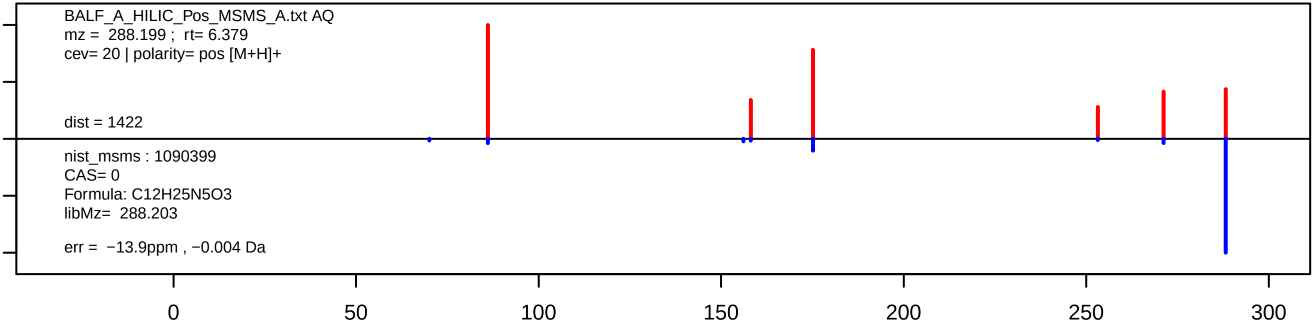

268 . Ile−Ile
Score=398 Dot=972 prob=25.7

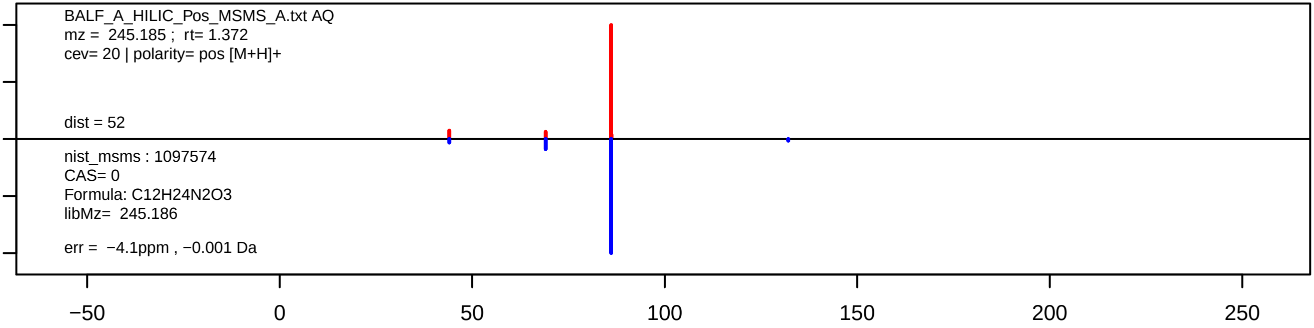

269 . Ile−Leu
Score=429 Dot=987 prob=48.5

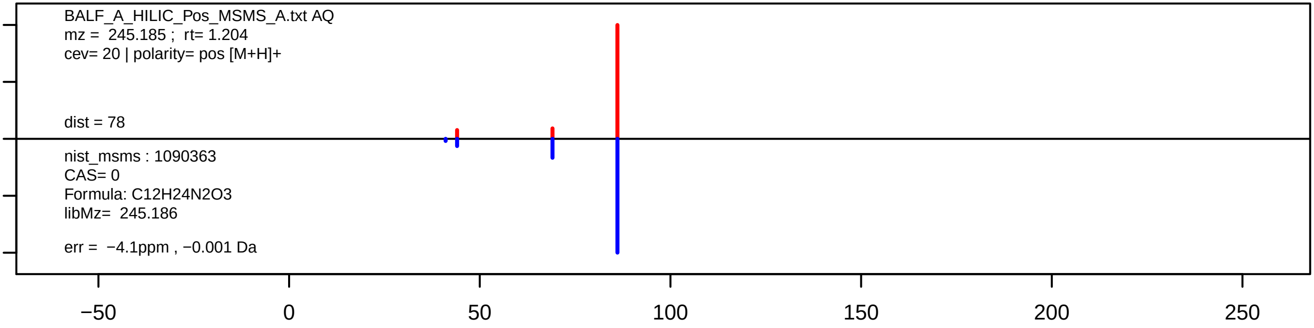

270 . Ile−Thr
Score=514 Dot=920 prob=49.4

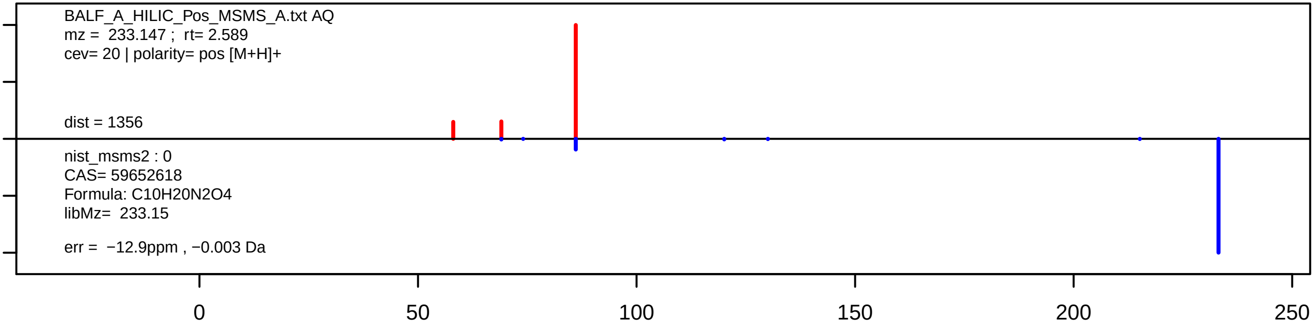

271 . Ile−Val
Score=511 Dot=952 prob=91.8

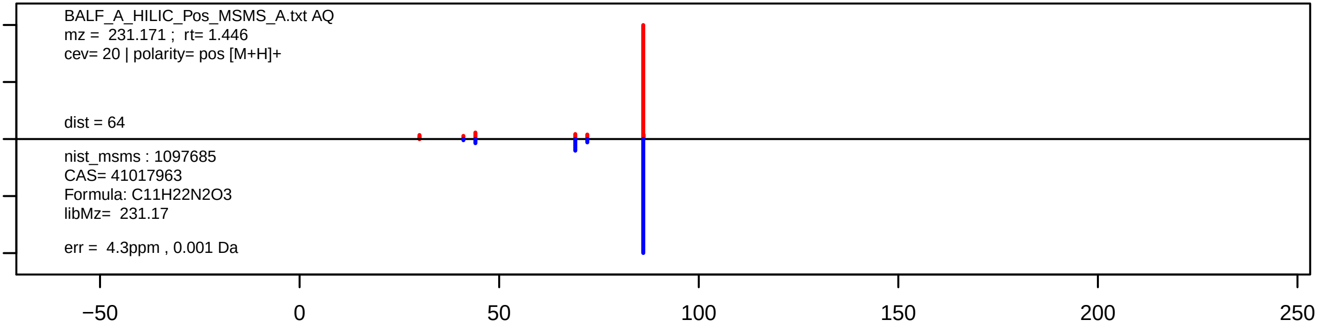

272 . Inosine
Score=334 Dot=956 prob=60.8

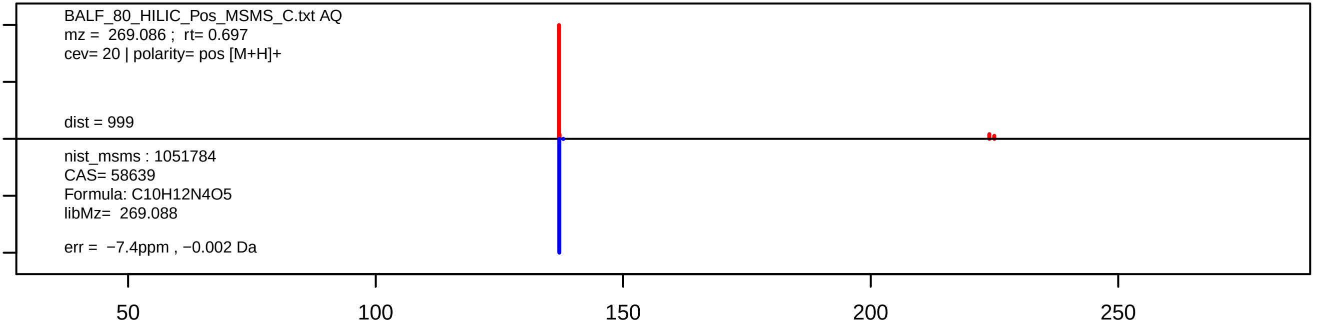

273 . Ipratropium cation
Score=847 Dot=907 prob=99

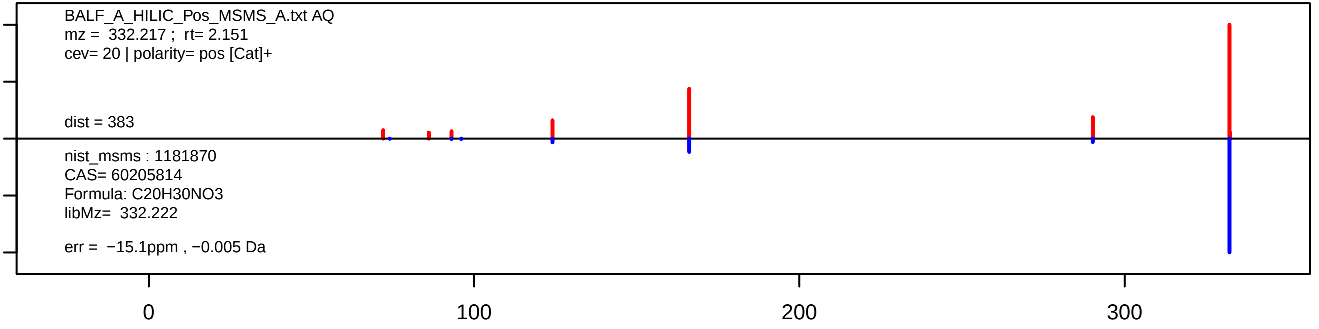

274 . L−Alanyl−L−norleucine
Score=560 Dot=999 prob=38.9

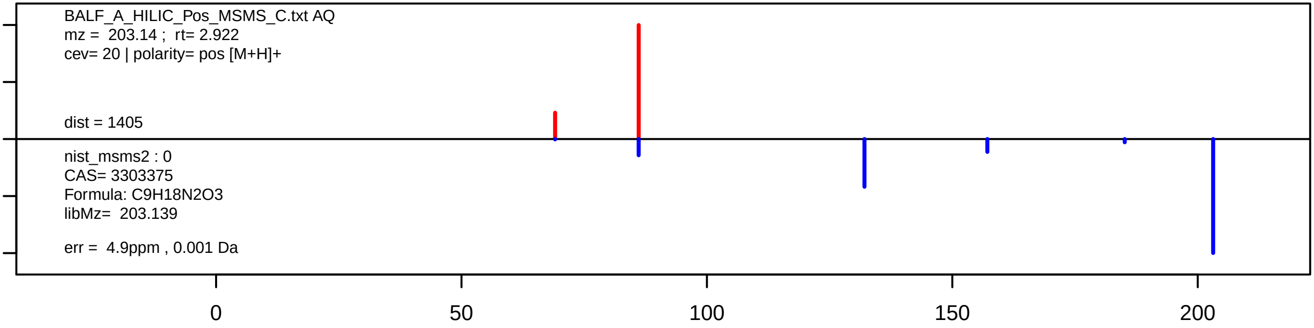

275 . L−Arginine
Score=568 Dot=954 prob=72.7

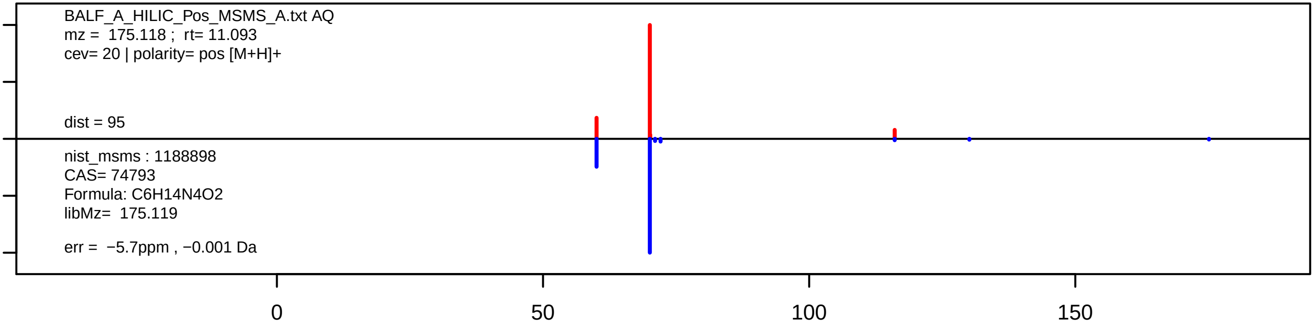

276 . L−Carnitine
Score=820 Dot=924 prob=98.9

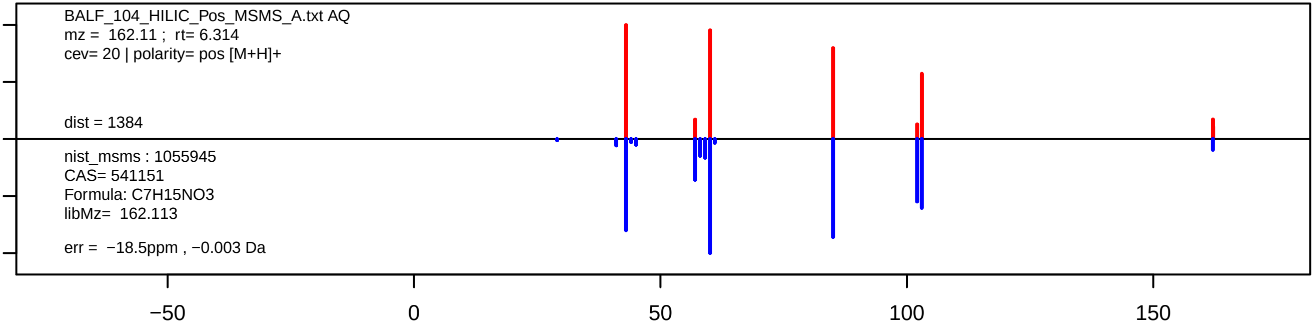

277 .   L−Carnosine
Score=758 Dot=927 prob=37.8

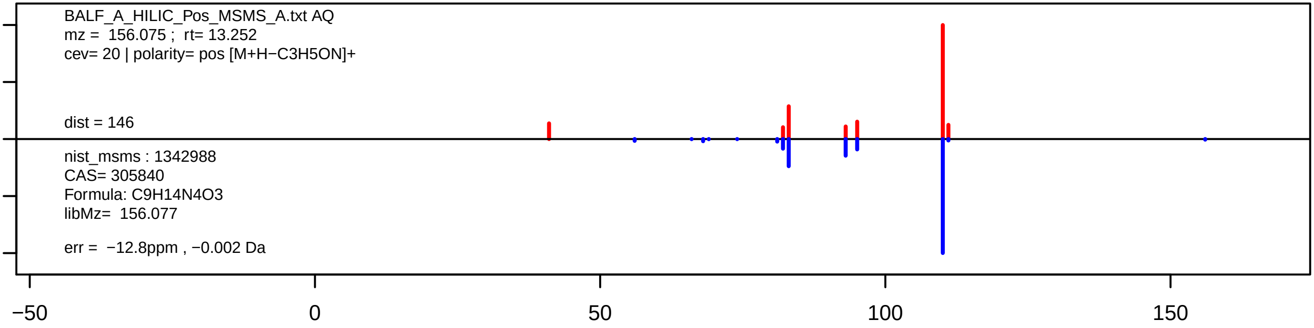

278 .   L−Citrulline
Score=393 Dot=909 prob=97.5

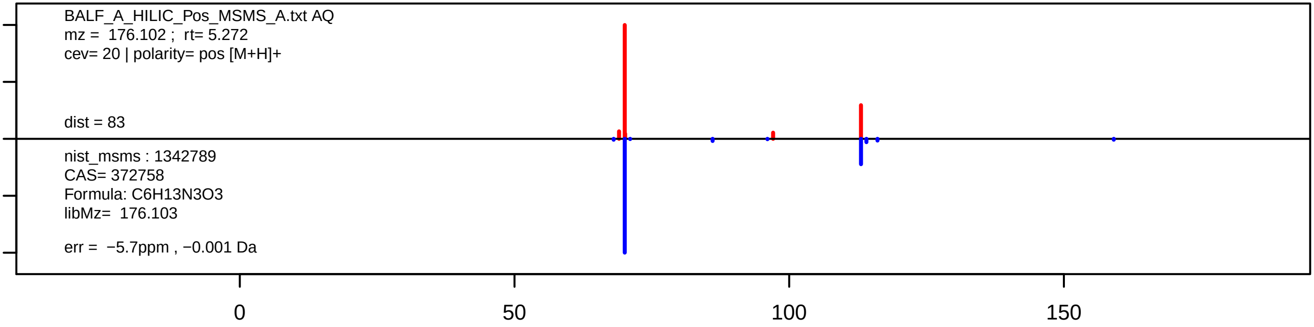

279 .   L−Glutamic acid
Score=745 Dot=942 prob=89.3

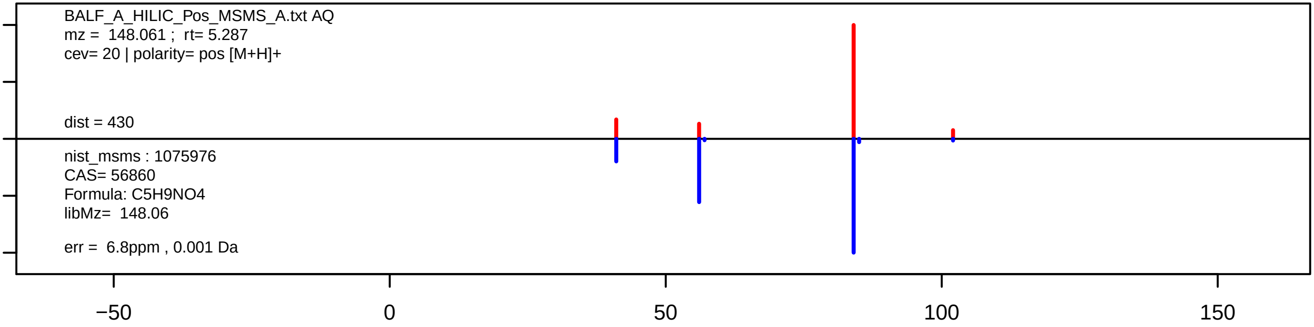

280 .   L−Histidine
Score=768 Dot=902 prob=79.2

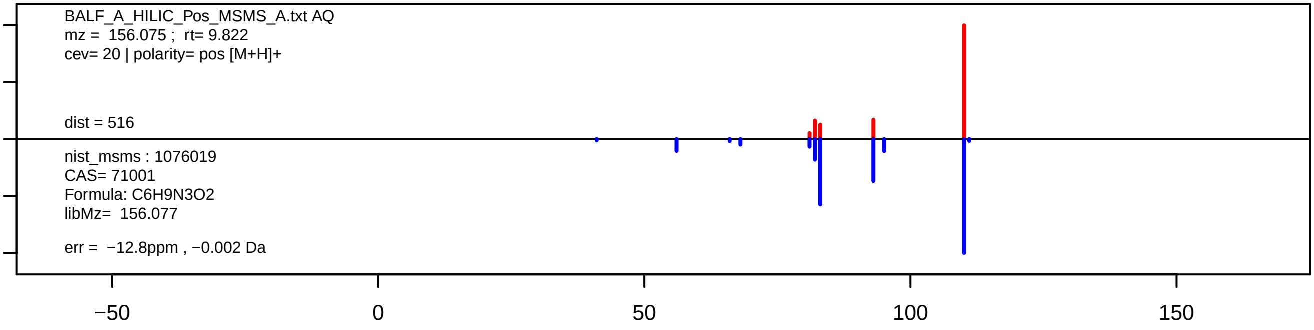

281 .   L−Hydroxyarginine
Score=399 Dot=999 prob=10.5

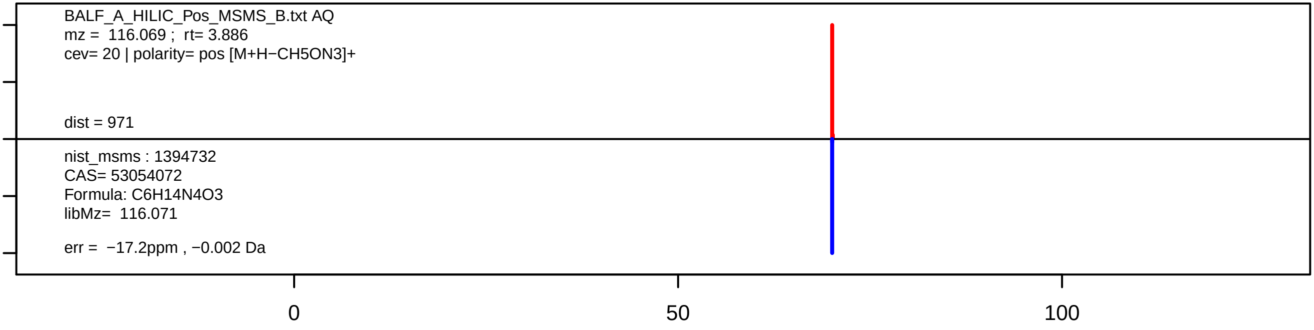

282 .   L−Isoleucine
Score=642 Dot=869 prob=93.4

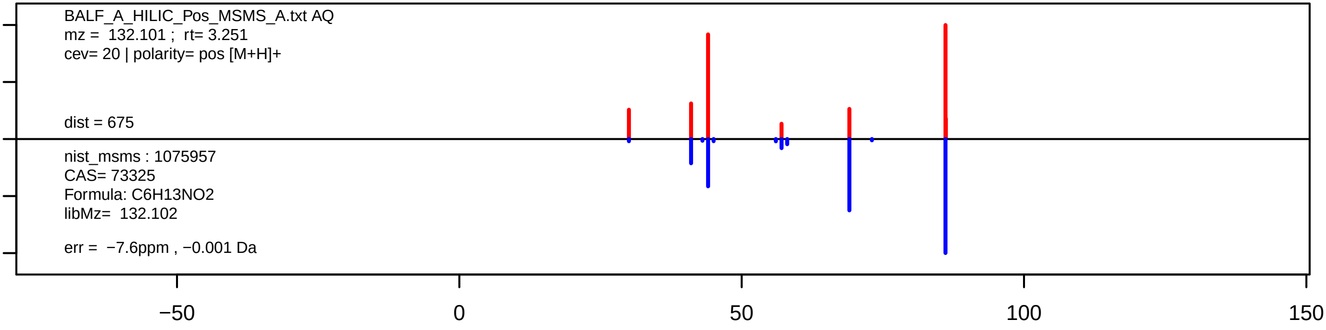

283 . L−Leucine
Score=941 Dot=965 prob=98.8

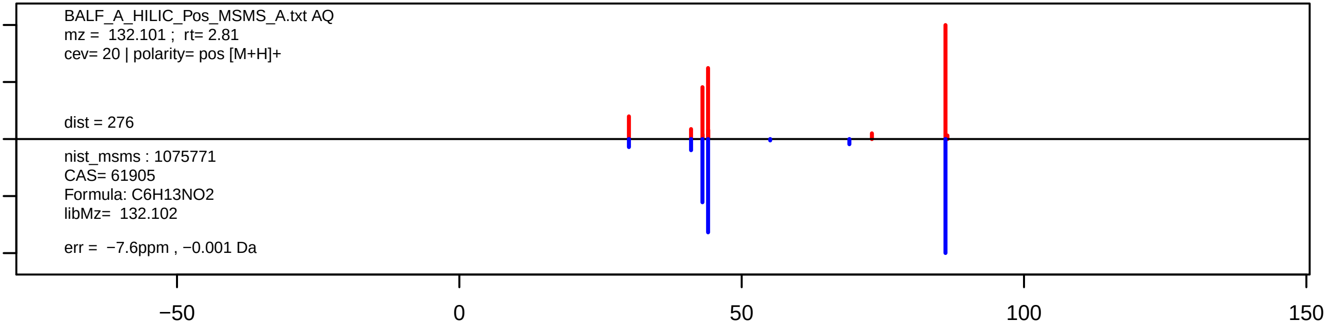

284 . L−Lysine
Score=252 Dot=901 prob=89.5

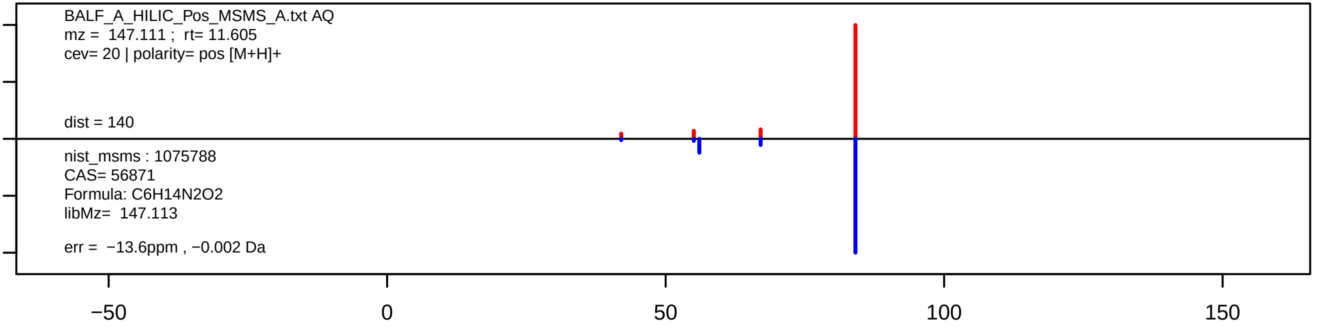

285 . L−Pipecolic acid
Score=400 Dot=999 prob=5.7

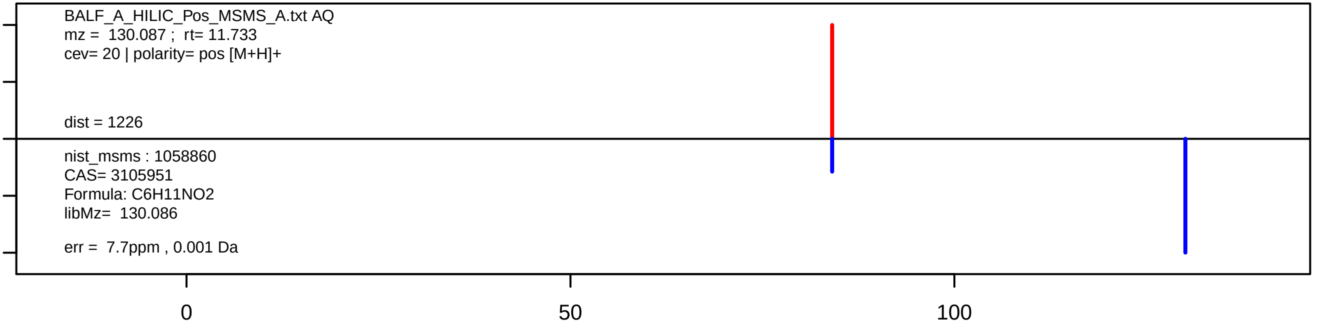

286 . L−Proline
Score=400 Dot=999 prob=7.2

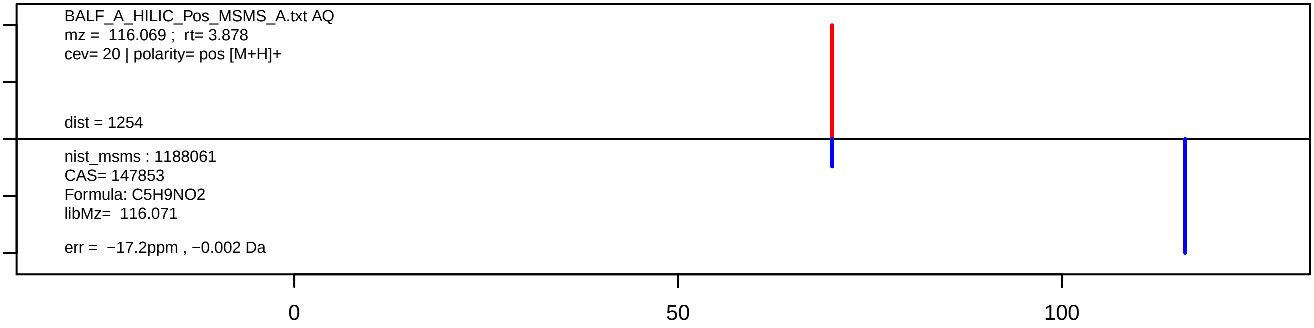

287 . L−Propionylcarnitine
Score=226 Dot=932 prob=57.7

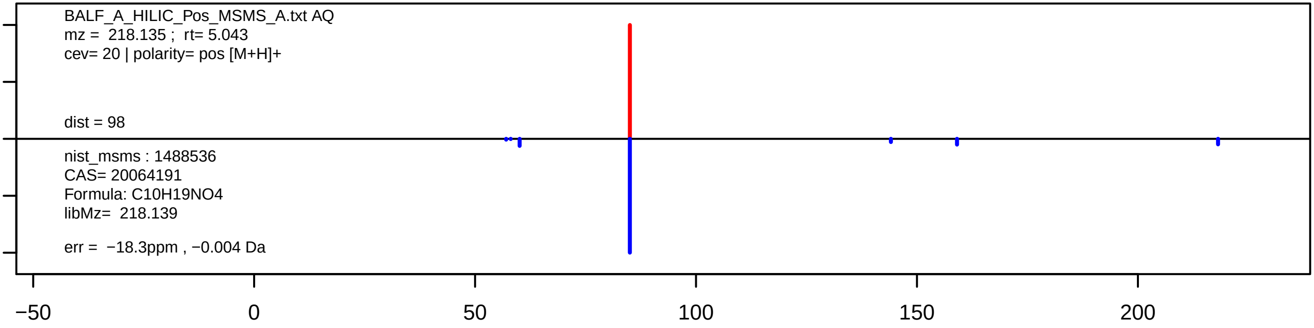

288 . L−Tyrosine
Score=890 Dot=951 prob=72.8

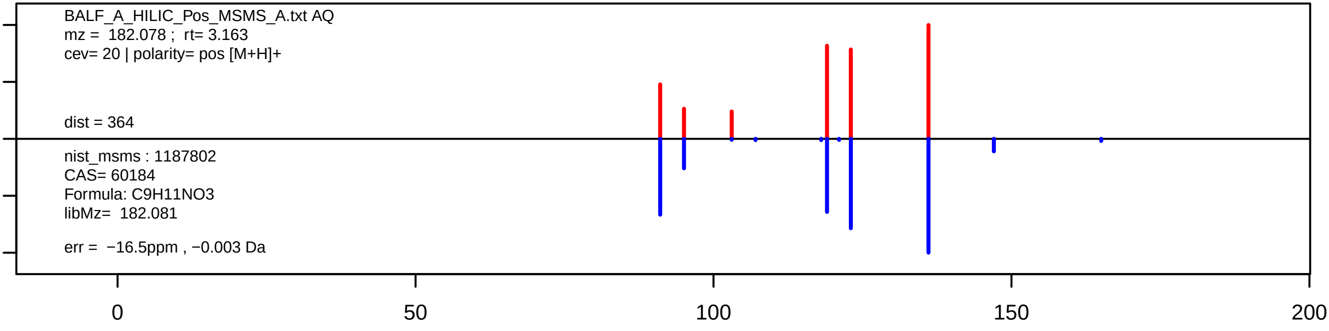

289 . L−Valine
Score=721 Dot=964 prob=98.1

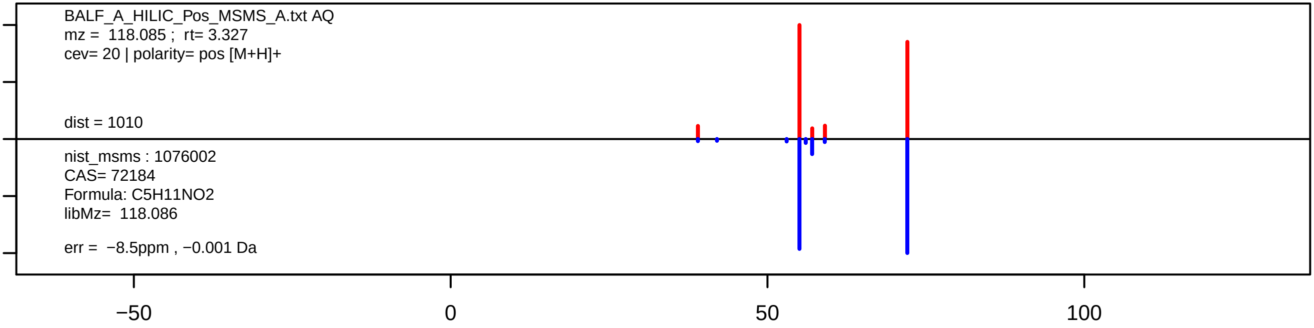

290 . Leu−Leu
Score=458 Dot=922 prob=44

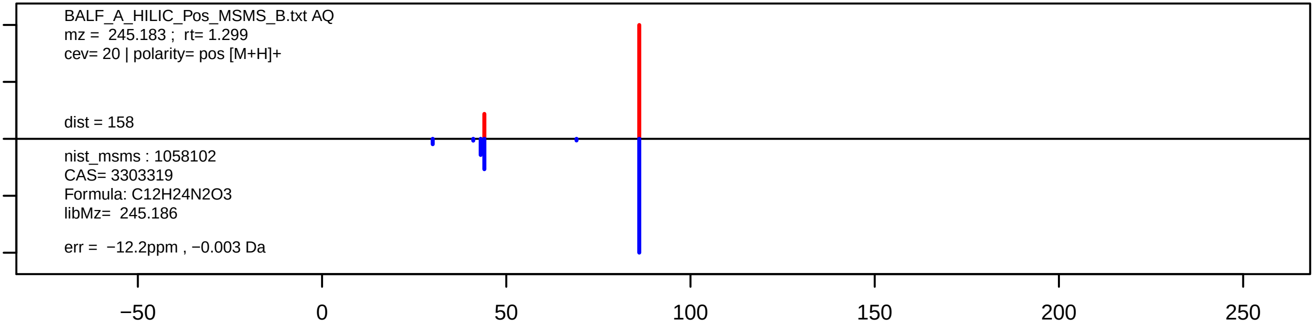

291 . Leu−Met
Score=480 Dot=907 prob=47.1

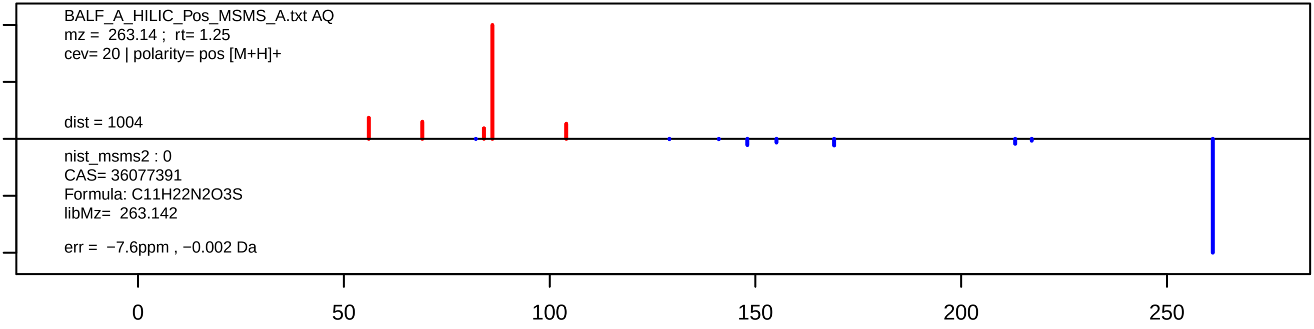

292 . Leu−Phe
Score=58 Dot=853 prob=34.6

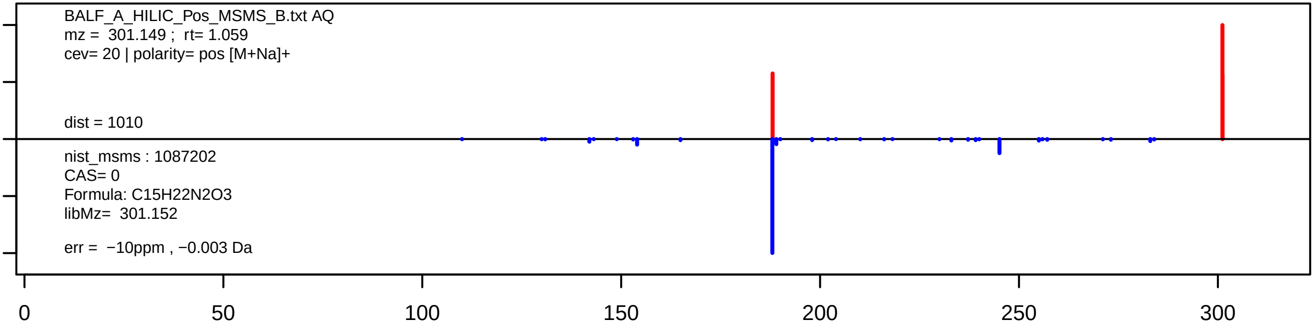

293 . Leu−Thr
Score=471 Dot=906 prob=48.9

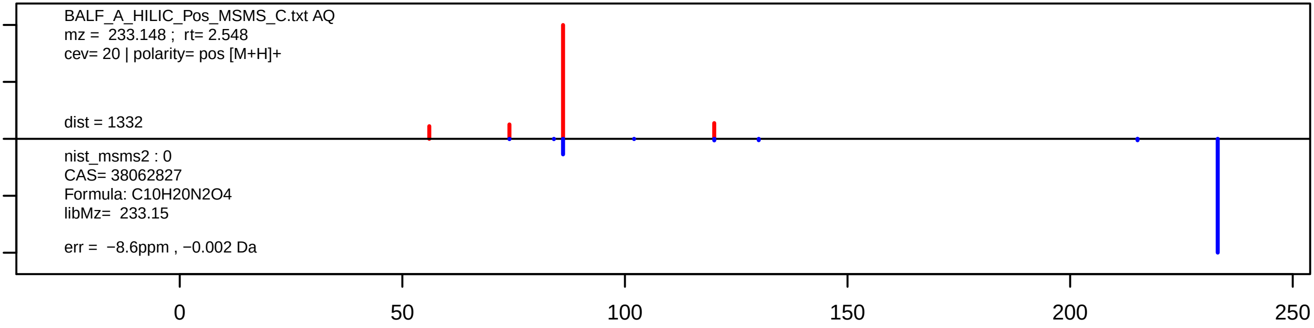

294 . Leu−Val
Score=396 Dot=878 prob=51.2

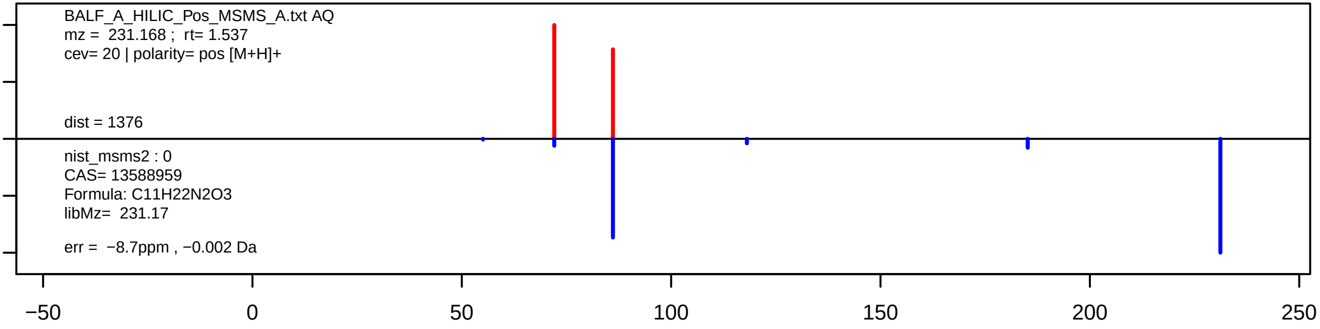

295 . Lidocaine
Score=444 Dot=988 prob=90.4

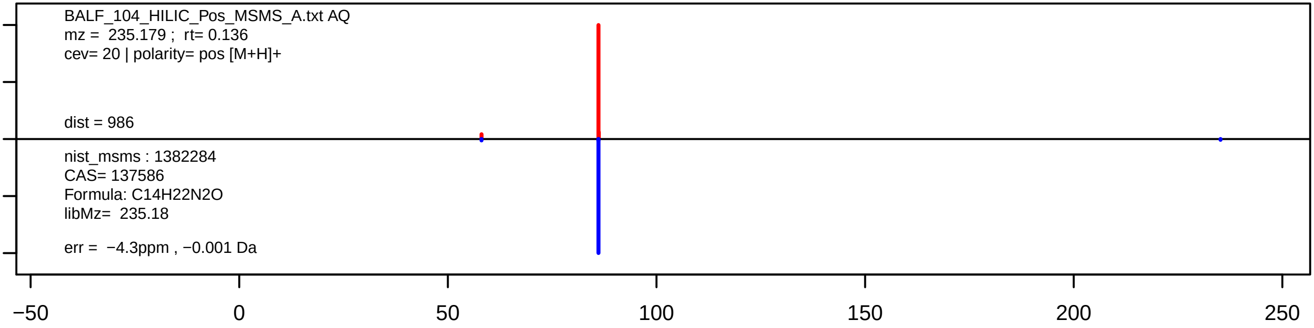

296 . Lys−Ala
Score=167 Dot=907 prob=39.3

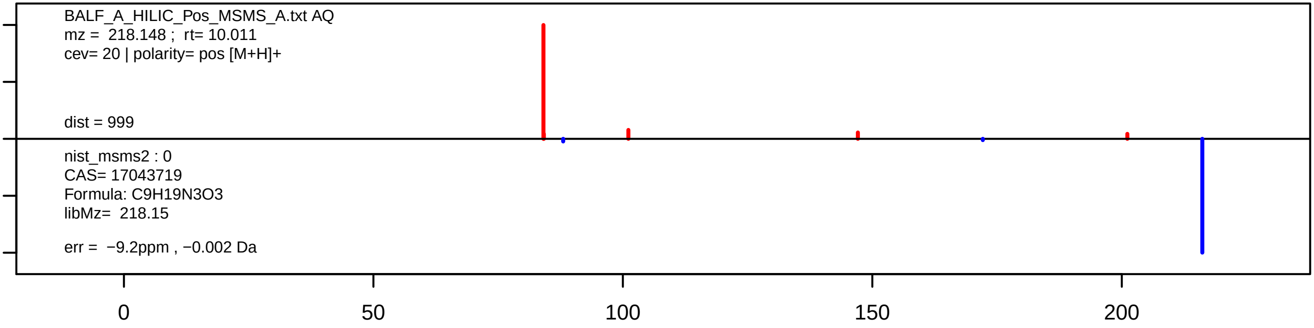

297 . Lys−Ile
Score=715 Dot=856 prob=90.5

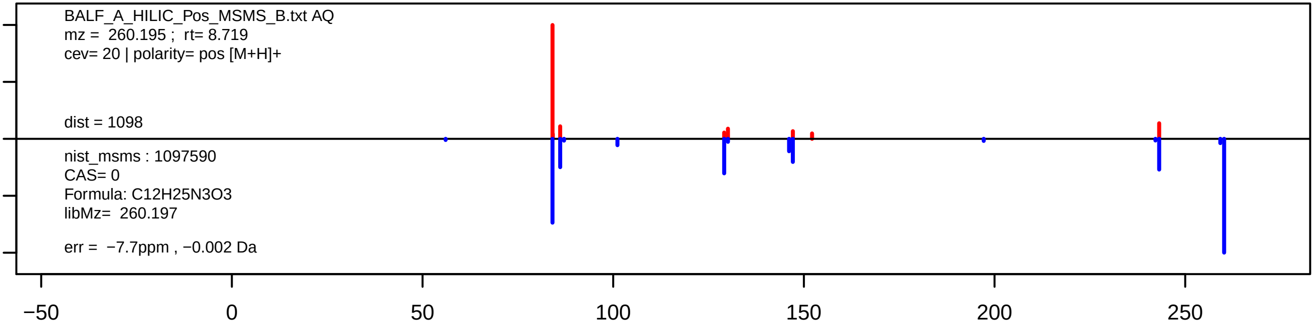

298 . Lys−Leu
Score=263 Dot=870 prob=63.4

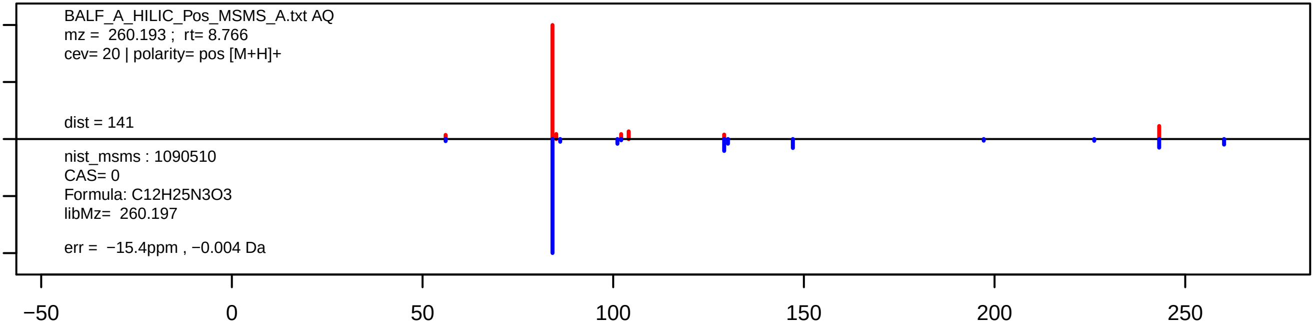

299 . Lys−Phe
Score=227 Dot=903 prob=45.8

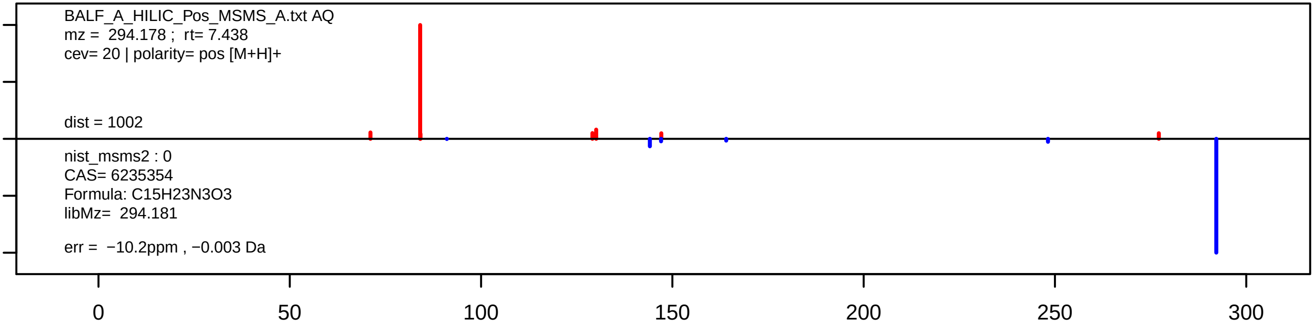

300 . Lys−Val
Score=372 Dot=844 prob=48.8

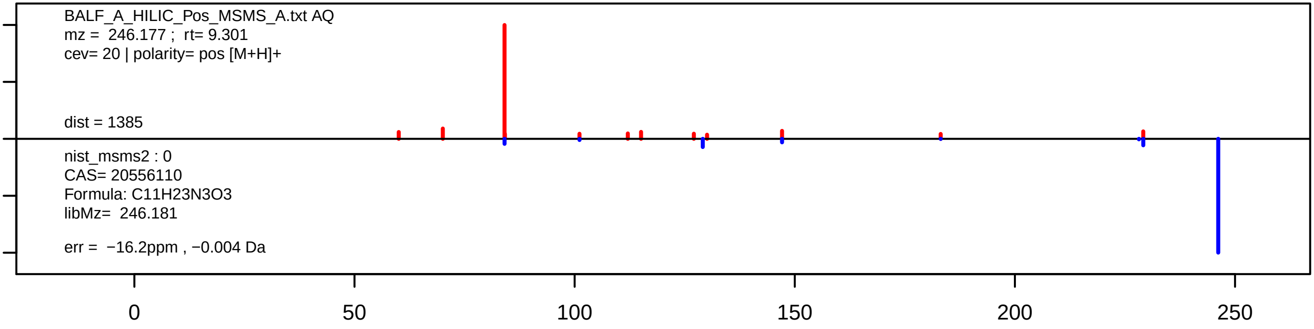

301 . Met−Leu
Score=411 Dot=834 prob=63

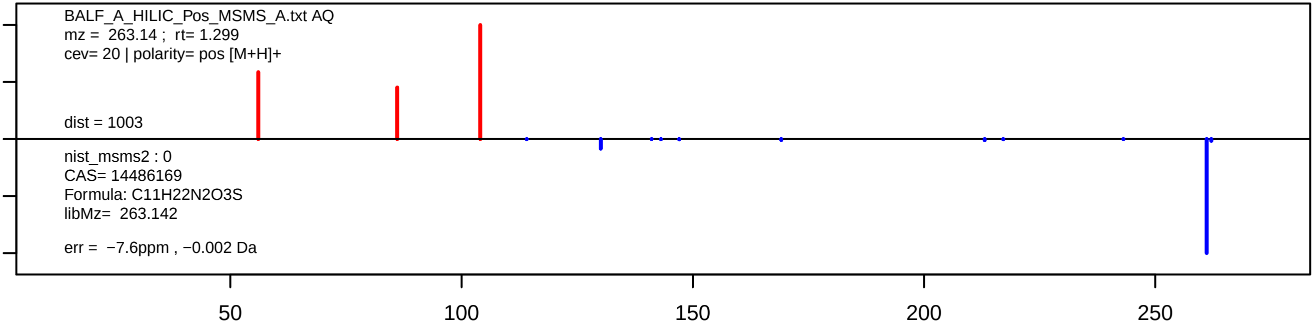

302 . Met−Val
Score=627 Dot=865 prob=49.4

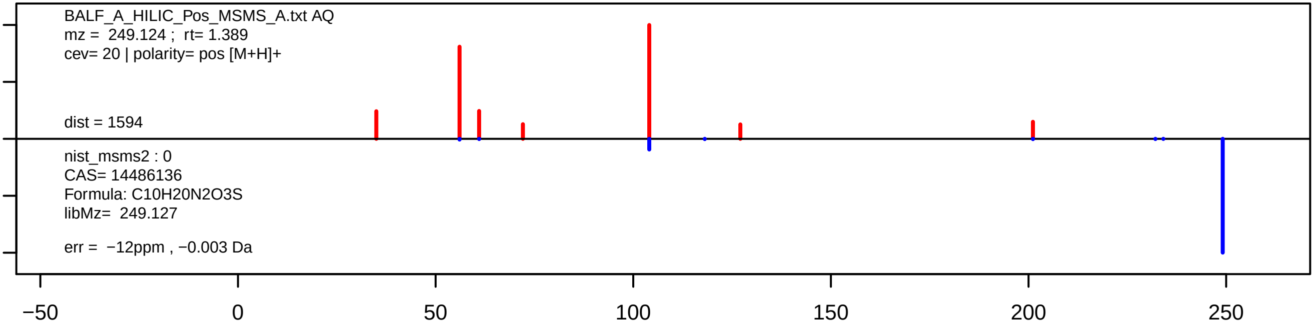

303 . Metformin
Score=849 Dot=940 prob=99

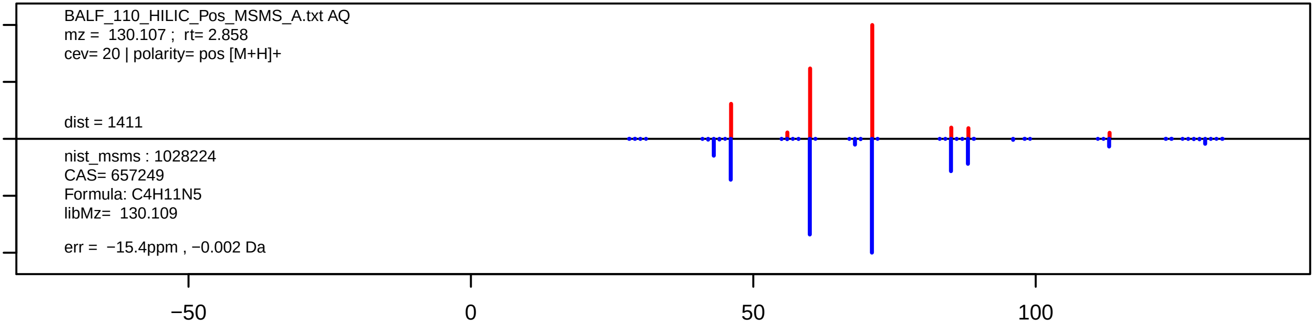

304 .   Methadone
Score=758 Dot=902 prob=97.6

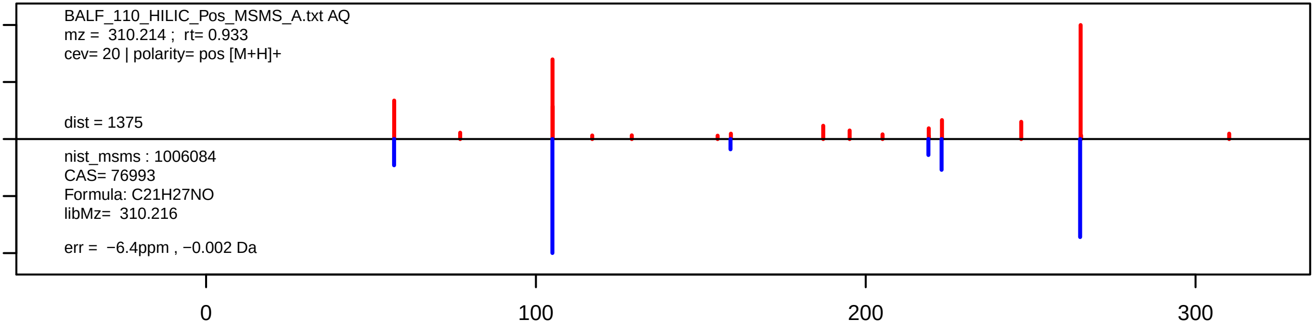

305 .   Methyl 4−hydroxybenzoate
Score=789 Dot=903 prob=98.8

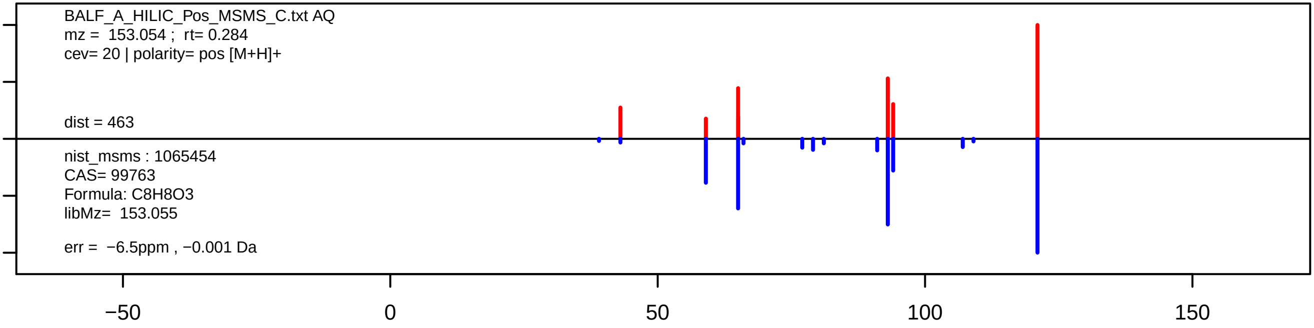

306 .   Methyl salicylate
Score=405 Dot=885 prob=92.6

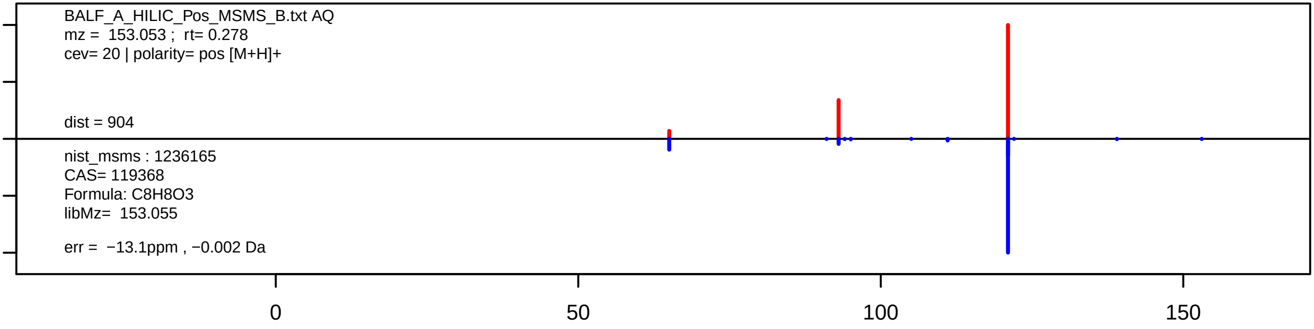

307 . Monoisobutyl phthalate
Score=274 Dot=980 prob=39.1

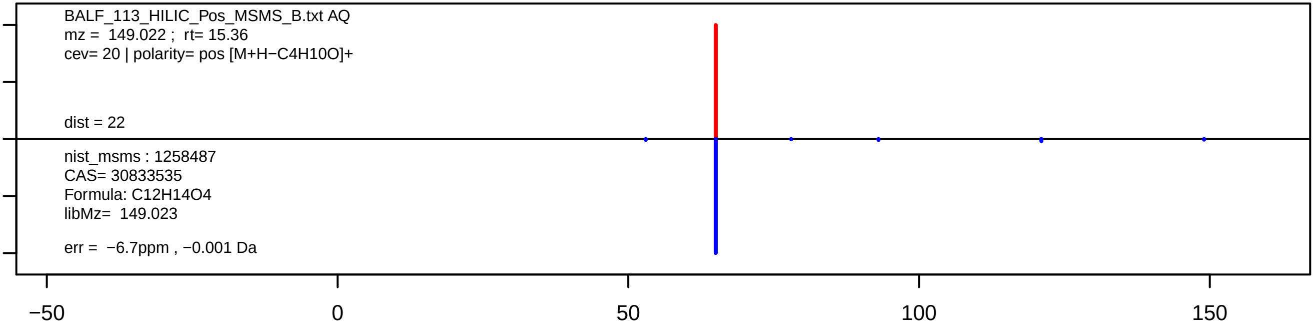

308 . N−.alpha.−(tert−Butoxycarbonyl)−L−histidine
Score=954 Dot=966 prob=26.7

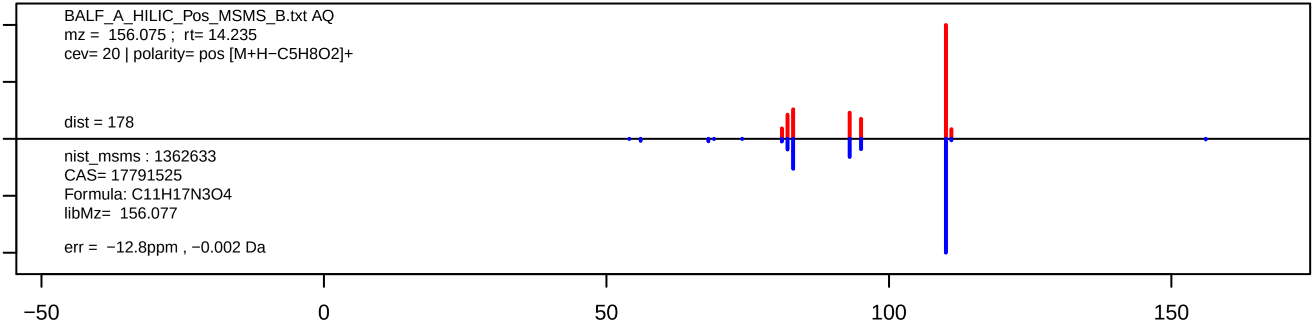

309 . N−Acetyl−3,4−methylenedioxymethcathinone
Score=787 Dot=952 prob=71

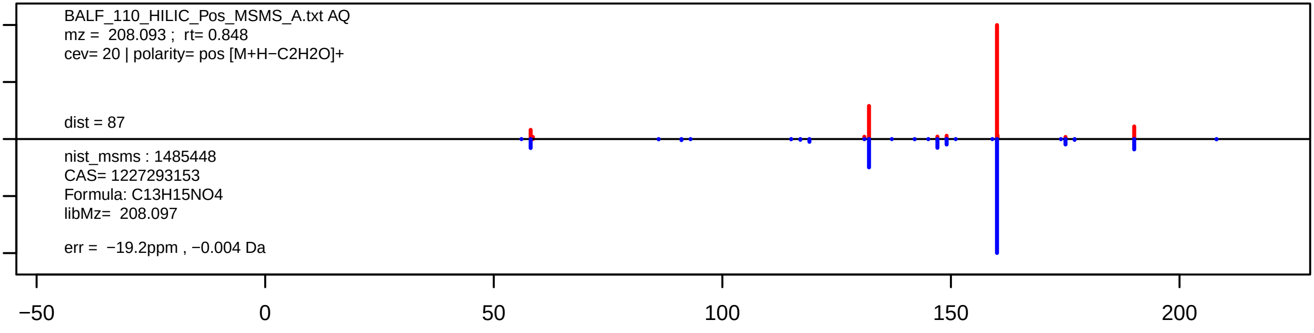

310 . N−Acetyl−L−carnosine
Score=816 Dot=922 prob=34.5

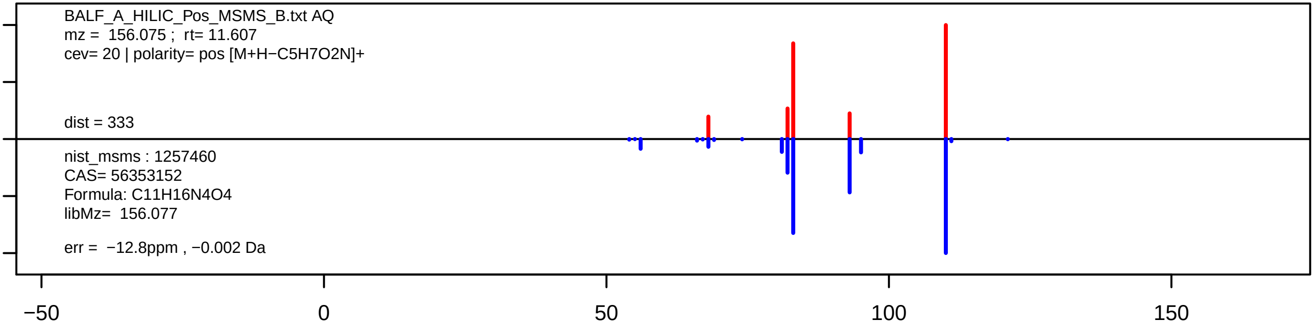

311 . N−Acetylamphetamine
Score=405 Dot=961 prob=50.2

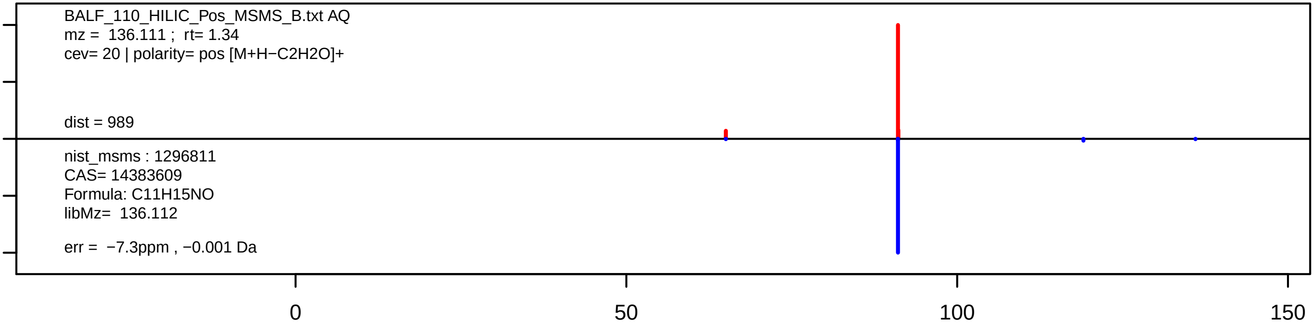

312 . N−Desmethyltramadol
Score=218 Dot=872 prob=89.5

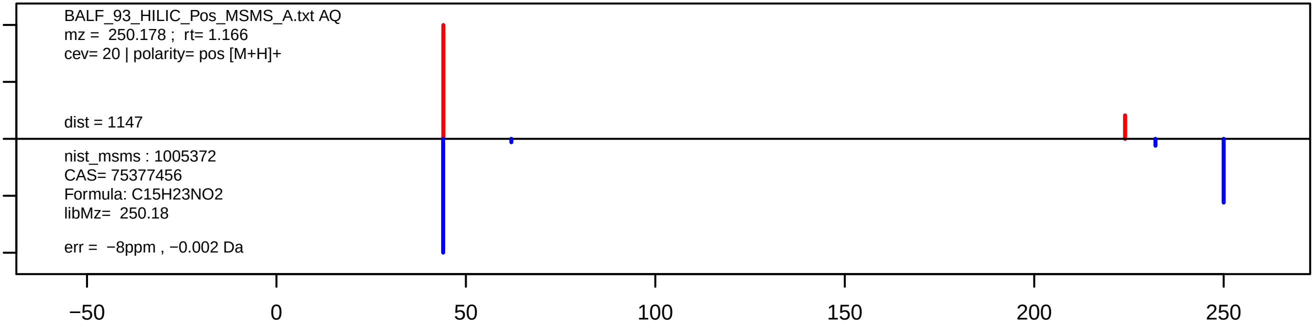

313 . N−Methyl−L−proline
Score=261 Dot=859 prob=78.3

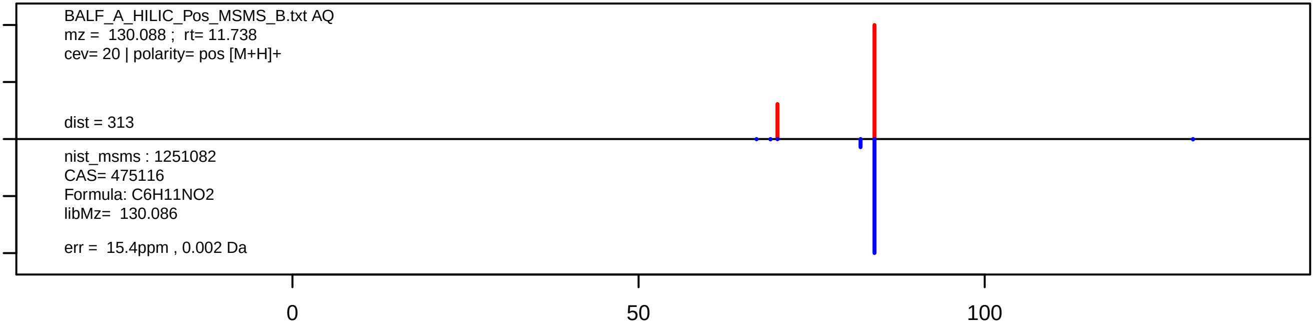

314 . N,N−Dimethylbenzylamine
Score=400 Dot=999 prob=20.8

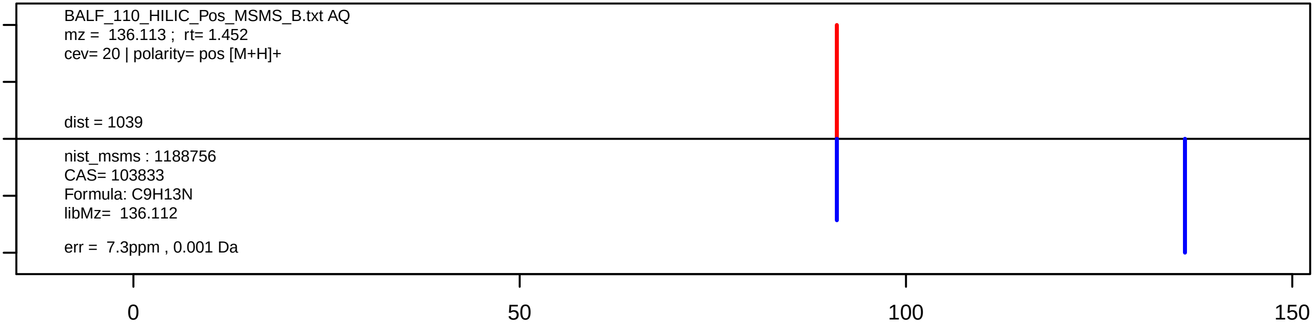

315 . N5−(1−Iminoethyl)−L−ornithine
Score=399 Dot=999 prob=11.1

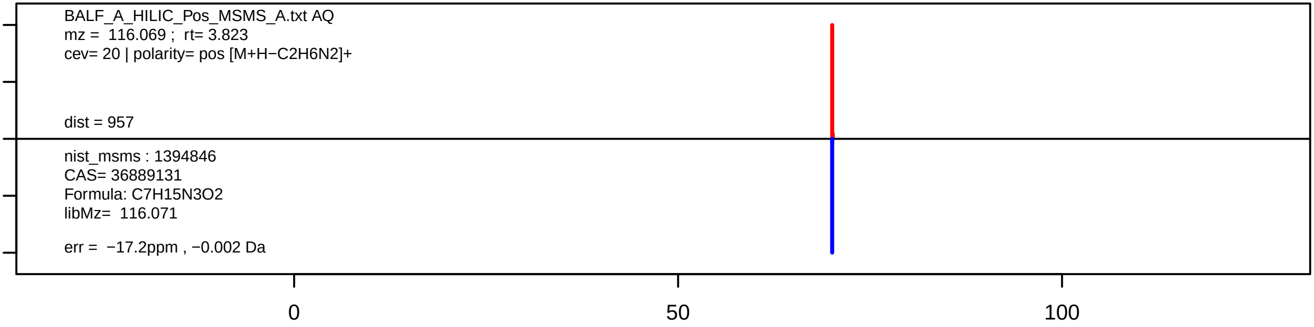

316 . Norquetiapine
Score=875 Dot=927 prob=99

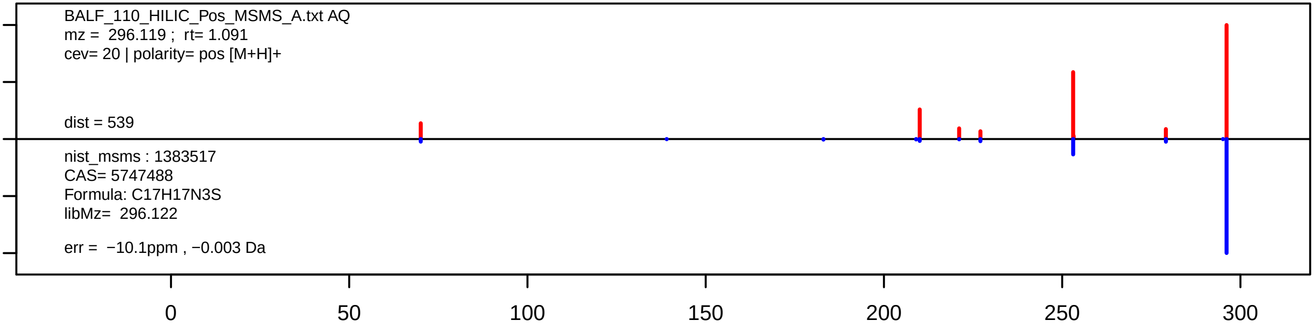

317 . O−Desmethylvenlafaxine
Score=341 Dot=828 prob=76.3

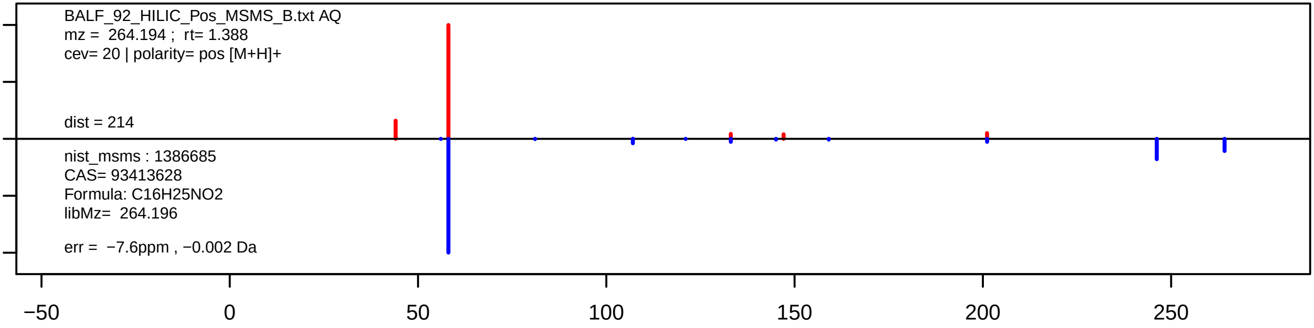

318 . Ondansetron
Score=782 Dot=988 prob=97.5

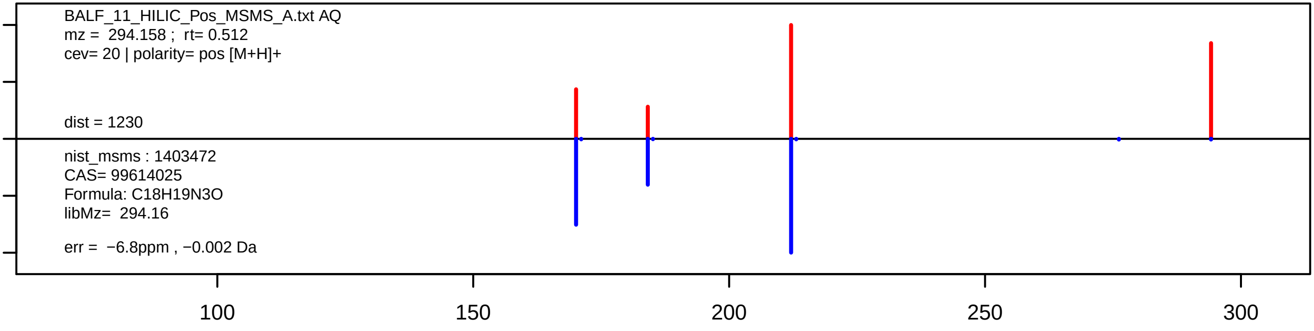

319 . Oxycodone
Score=353 Dot=964 prob=80

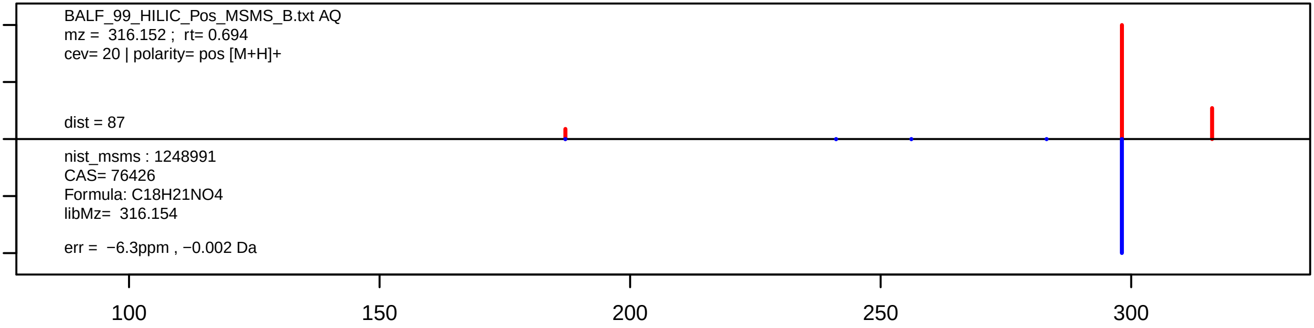

320 . Phe−Ile
Score=444 Dot=972 prob=45.9

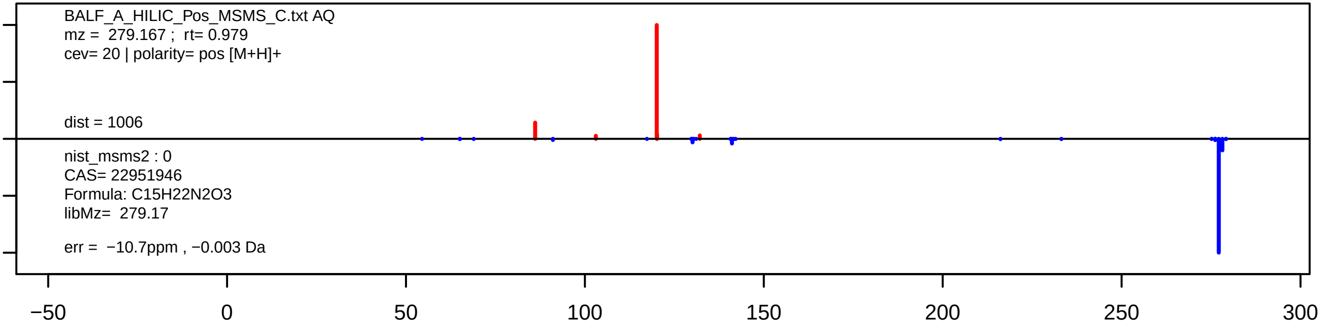

321 . Phe−Leu
Score=279 Dot=929 prob=26.8

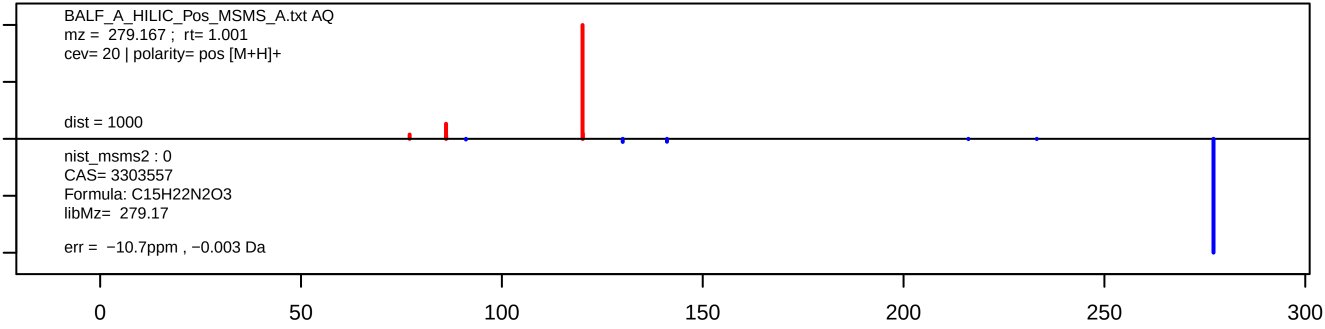

322 . Phe−Phe
Score=440 Dot=938 prob=99

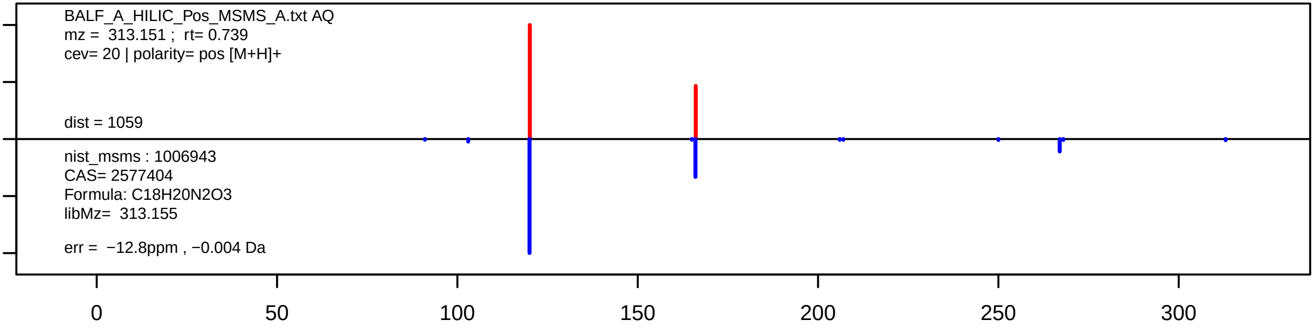

323 . Phe−Val
Score=278 Dot=963 prob=49.4

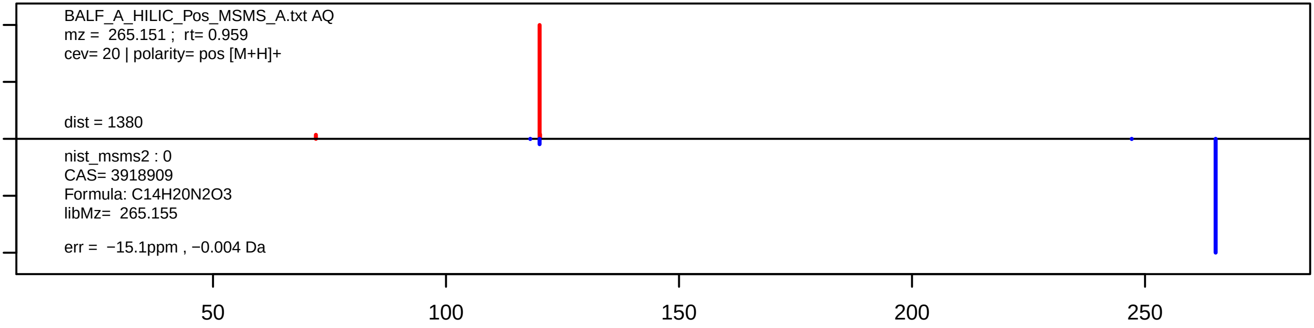

324 . Phenylacetyl−L−glutamine
Score=726 Dot=861 prob=97.9

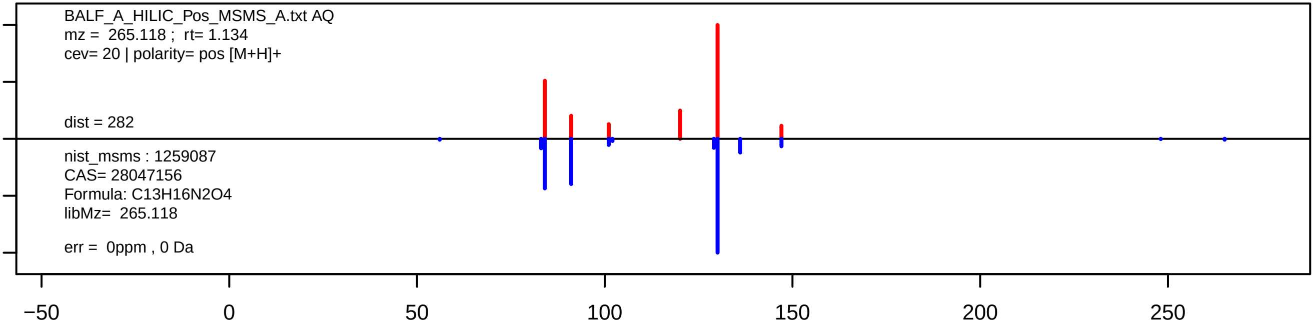

325 . Pro−Ala
Score=382 Dot=955 prob=45.1

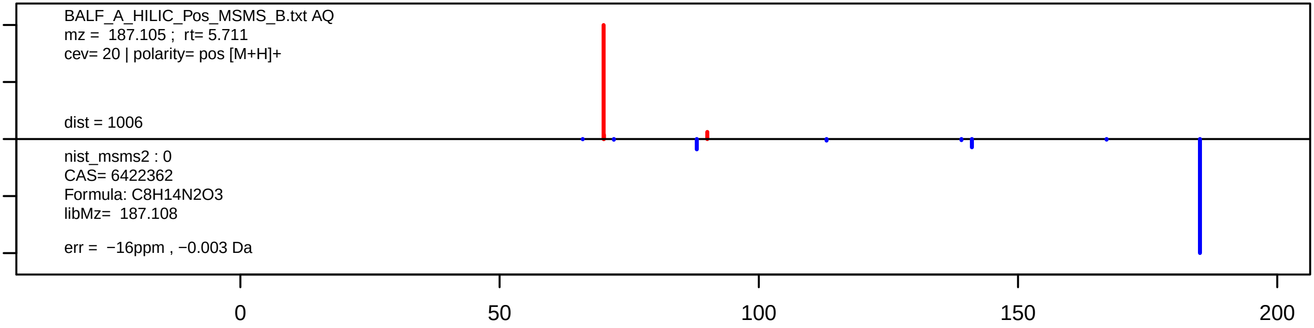

326 . Purine
Score=644 Dot=977 prob=98.1

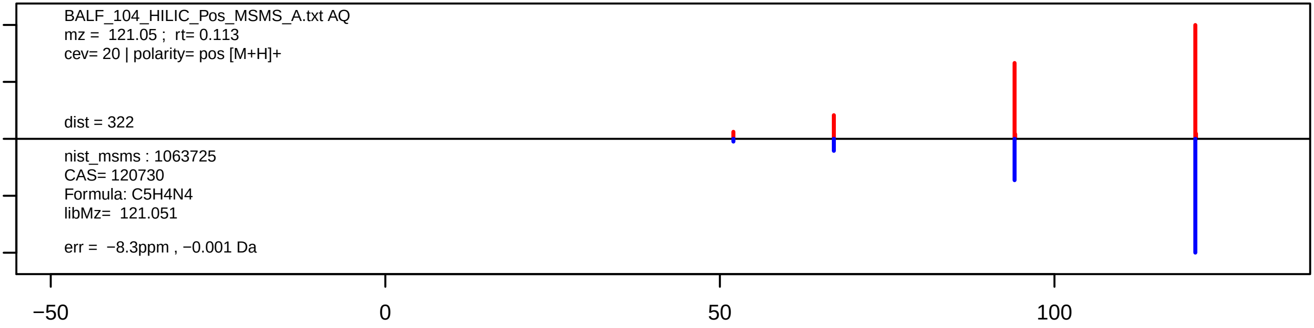

327 . Quetiapine sulfoxide
Score=330 Dot=861 prob=98.9

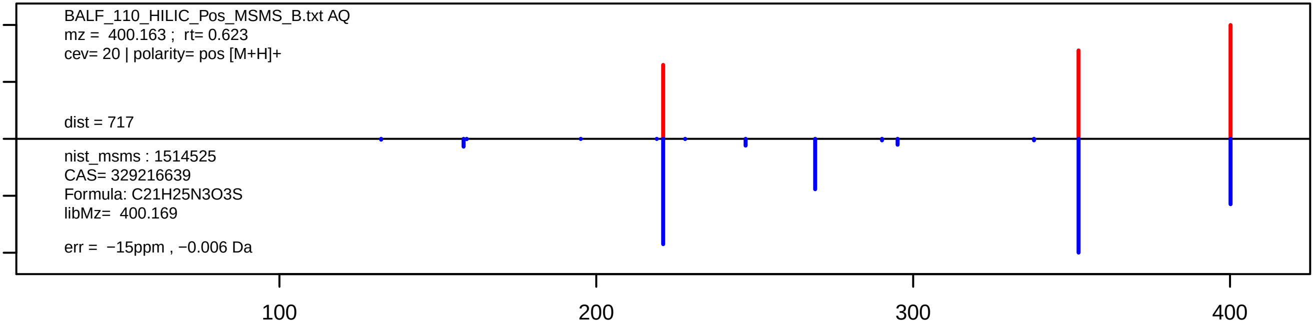

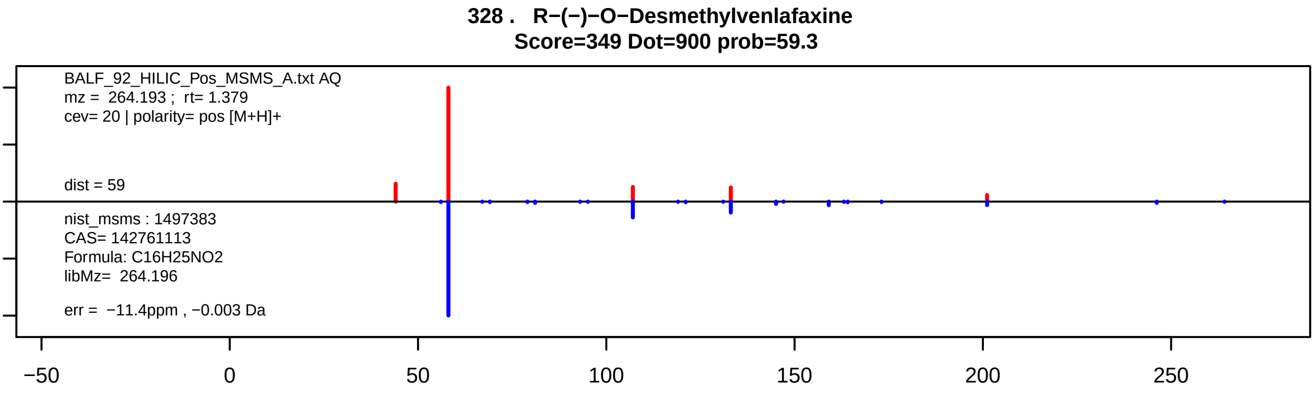
328 . R−(−)−O−Desmethylvenlafaxine
Score=349 Dot=900 prob=59.3
BALF_92_HILIC_Pos_MSMS_A.txt AQ
mz = 264.193 ; rt= 1.379
cev= 20 | polarity= pos [M+H]+
dist = 59
nist_msms : 1497383
CAS= 142761113
Formula: C16H25NO2
libMz= 264.196
err = −11.4ppm , −0.003 Da
−50
0
50
100
150
200
250

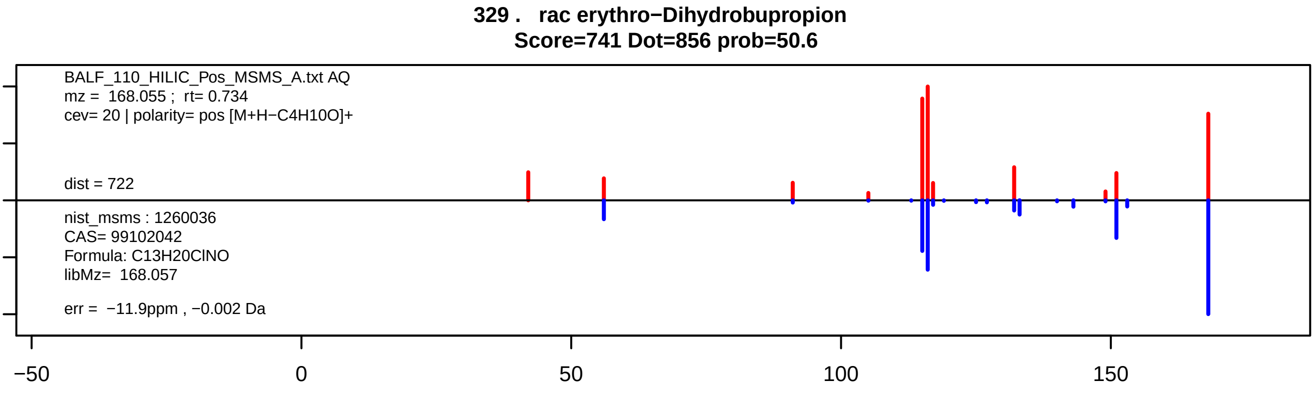
329 . rac erythro−Dihydrobupropion
Score=741 Dot=856 prob=50.6
BALF_110_HILIC_Pos_MSMS_A.txt AQ
mz = 168.055 ; rt= 0.734
cev= 20 | polarity= pos [M+H−C4H10O]+
dist = 722
nist_msms : 1260036
CAS= 99102042
Formula: C13H20ClNO
libMz= 168.057
err = −11.9ppm , −0.002 Da
−50
0
50
100
150

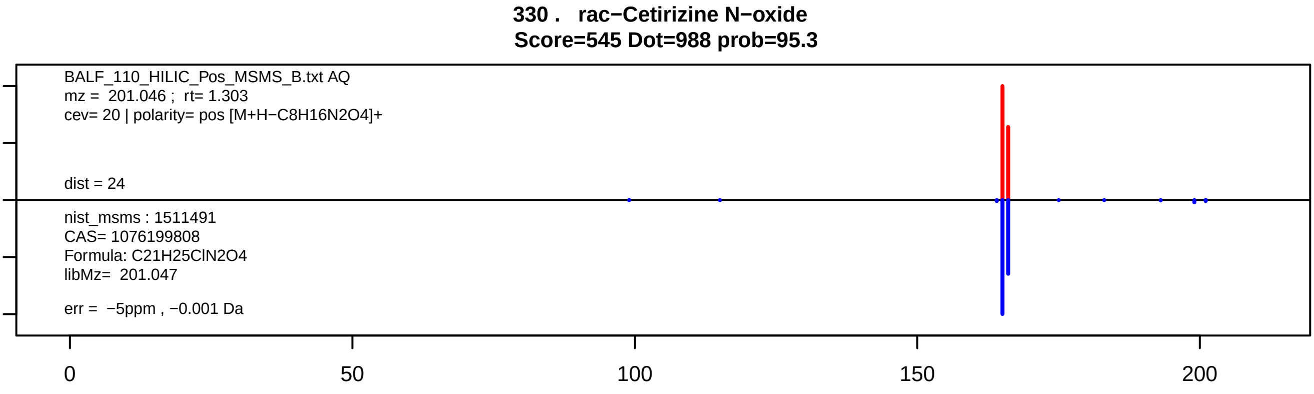
330 . rac−Cetirizine N−oxide
Score=545 Dot=988 prob=95.3
BALF_110_HILIC_Pos_MSMS_B.txt AQ
mz = 201.046 ; rt= 1.303
cev= 20 | polarity= pos [M+H−C8H16N2O4]+
dist = 24
nist_msms : 1511491
CAS= 1076199808
Formula: C21H25ClN2O4
libMz= 201.047
err = −5ppm , −0.001 Da
0
50
100
150
200

331 . Risperidone
Score=400 Dot=999 prob=96.4

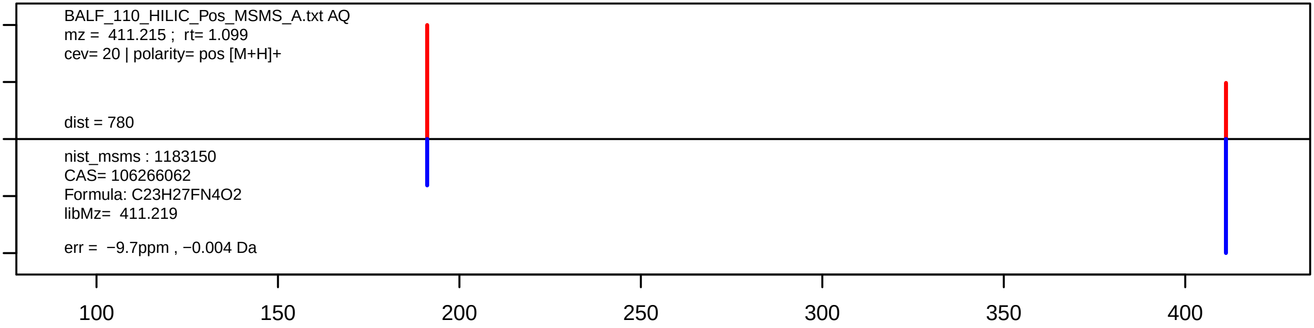

332 . Sarcosine
Score=400 Dot=999 prob=55.4

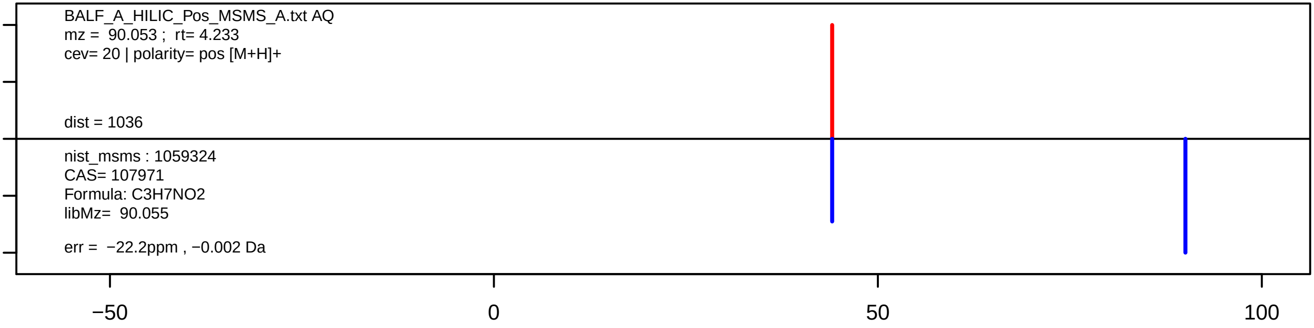

333 . Ser−Ile
Score=609 Dot=857 prob=73.6

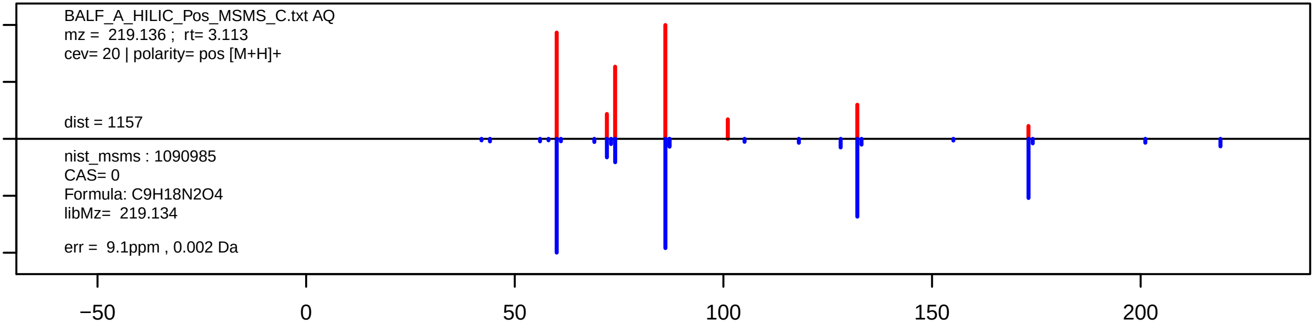

334 . Sucrose
Score=560 Dot=999 prob=76.8

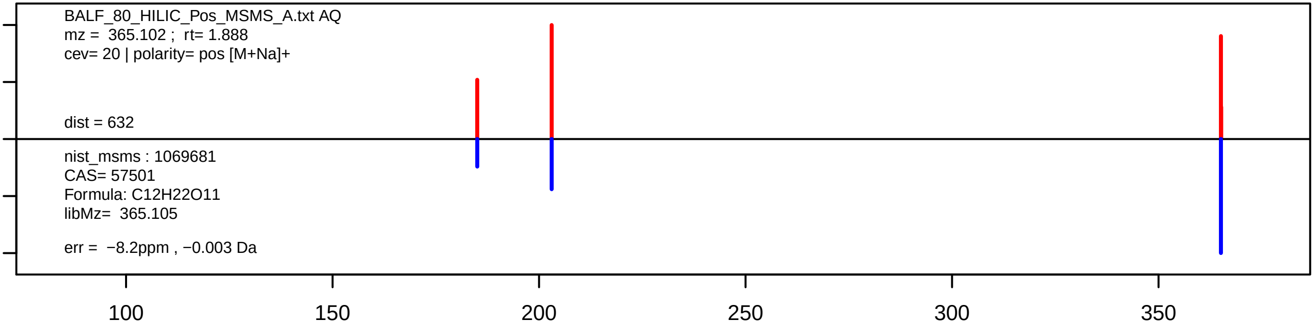

335 . Taurine
Score=179 Dot=911 prob=100

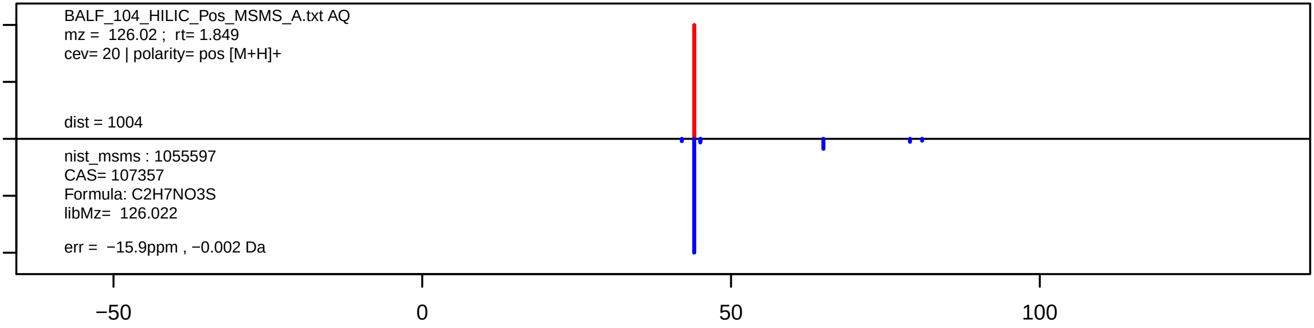

336 . Tetradonium cation
Score=456 Dot=920 prob=97.8

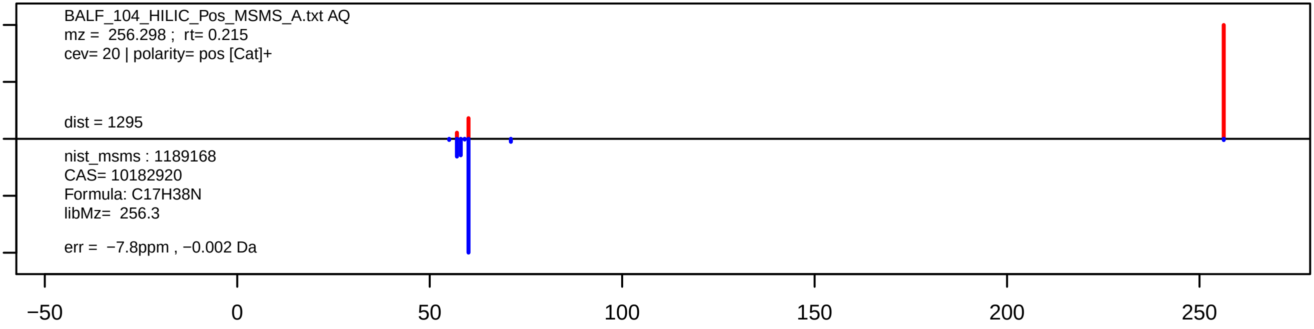

337 . Tetraethylene glycol
Score=368 Dot=991 prob=99

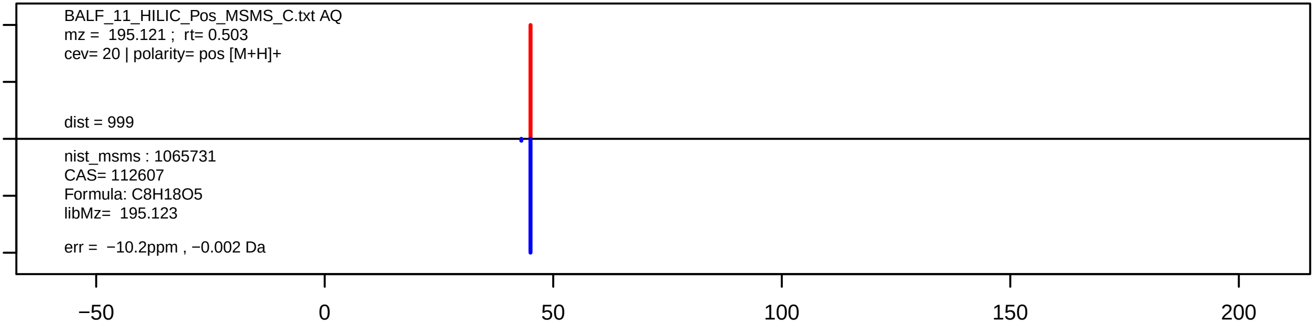

338 . Thr−Leu
Score=879 Dot=946 prob=99

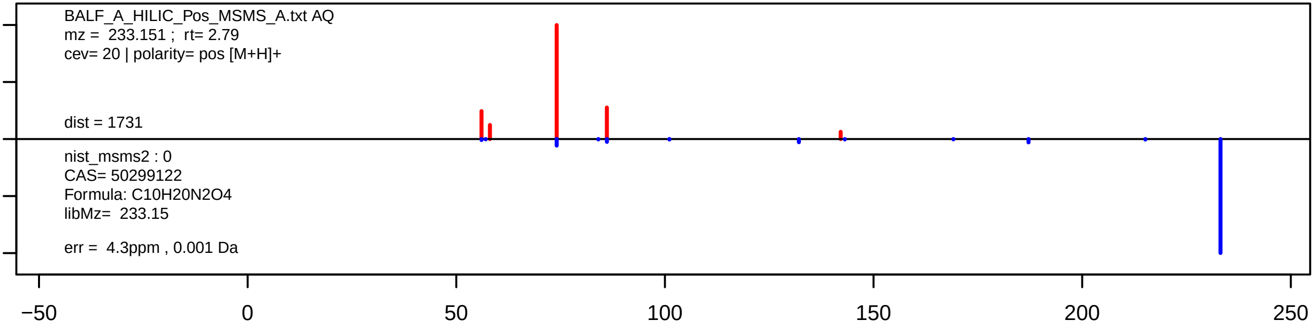

339 . threo−Dihydrobupropion
Score=854 Dot=962 prob=92.9

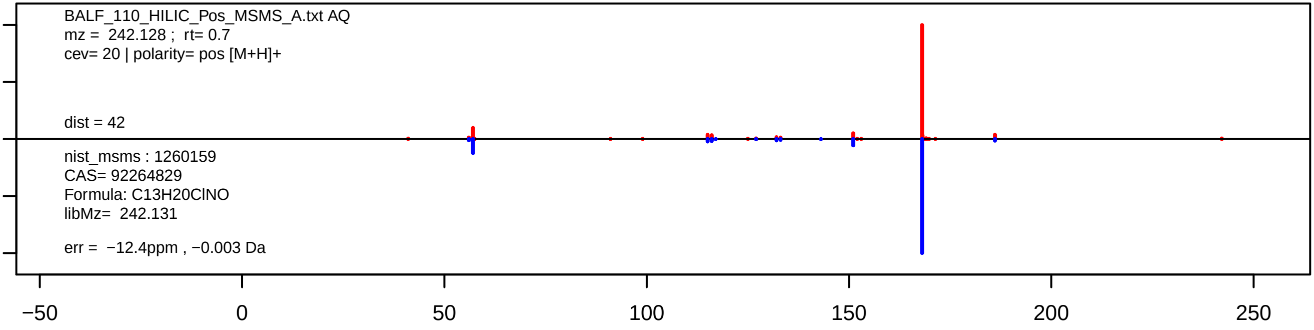

340 . Tramadol
Score=340 Dot=971 prob=96.9

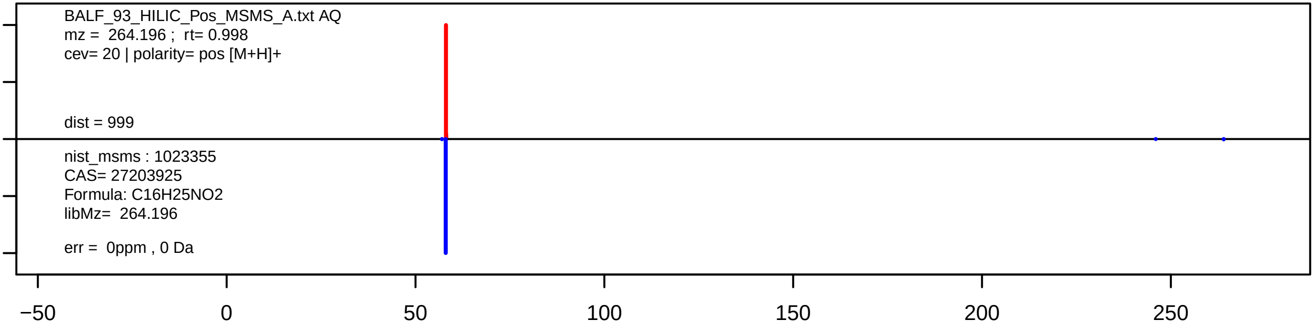

341 . Tyr−Arg
Score=502 Dot=806 prob=48.9

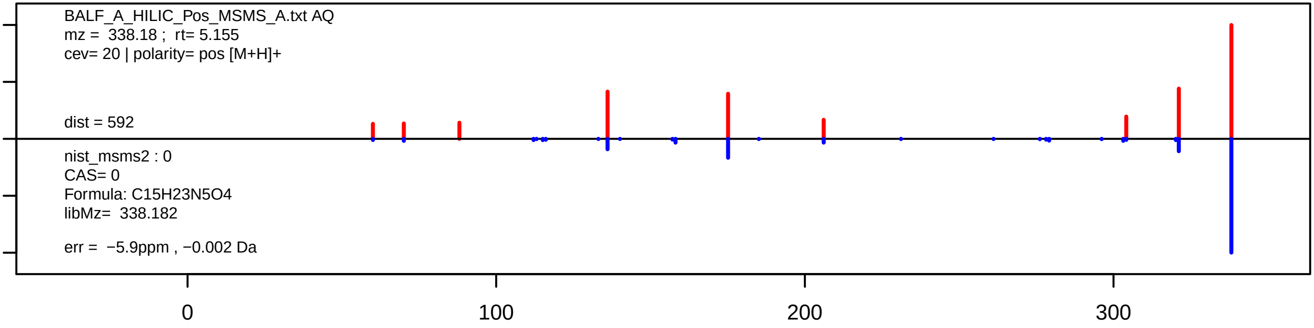

342 . Tyr−Ile
Score=255 Dot=845 prob=24.3

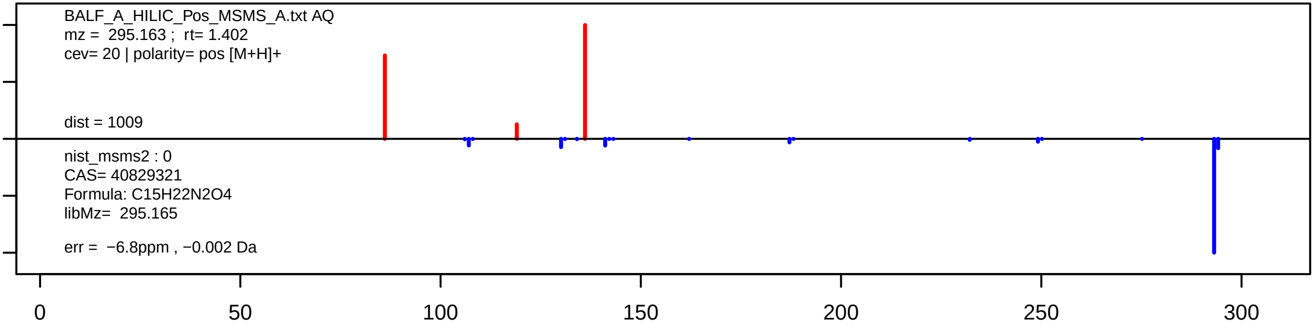

343 .   Tyr−Leu
Score=176 Dot=847 prob=25.6

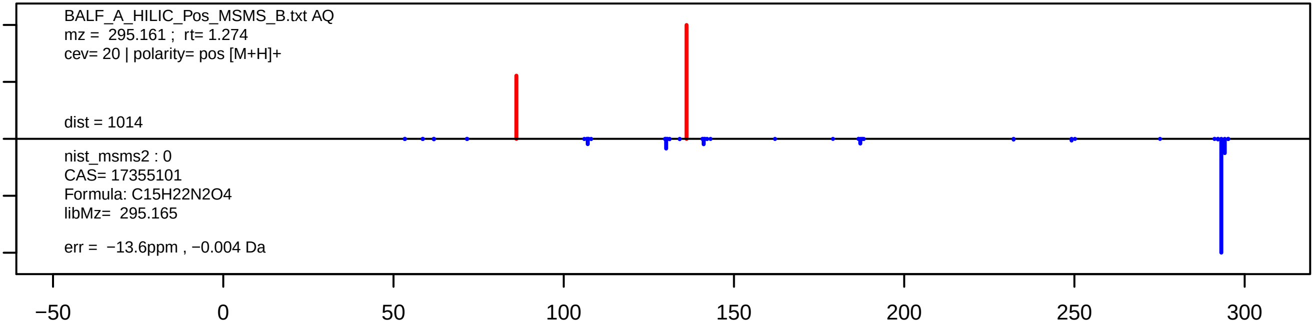

344 .   Tyramine
Score=656 Dot=896 prob=63.1

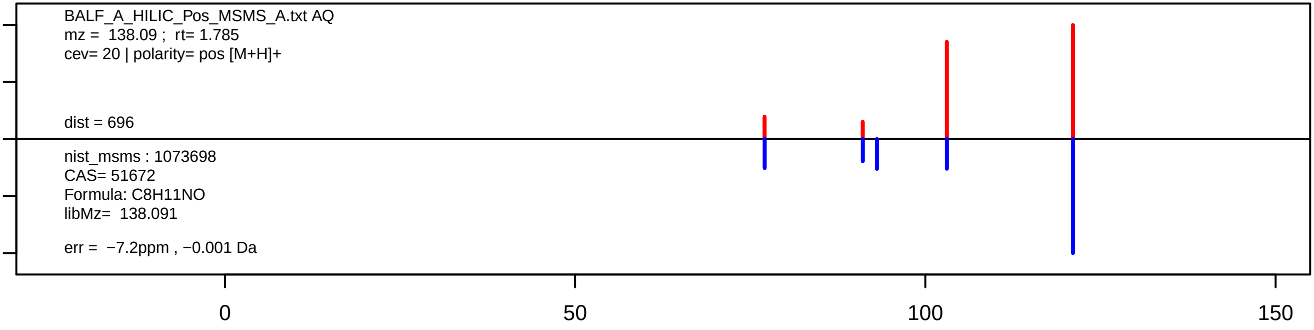

345 .   Val−Ala
Score=378 Dot=865 prob=37.3

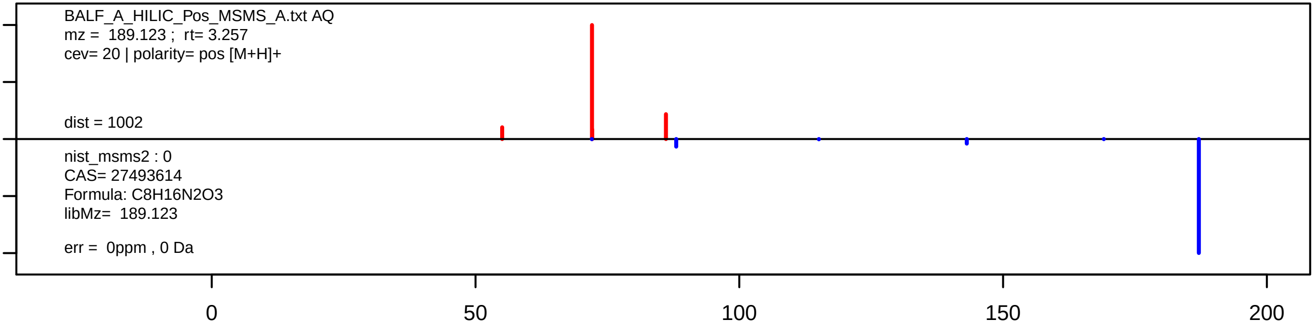

346 . Val−Arg
Score=722 Dot=827 prob=98.7

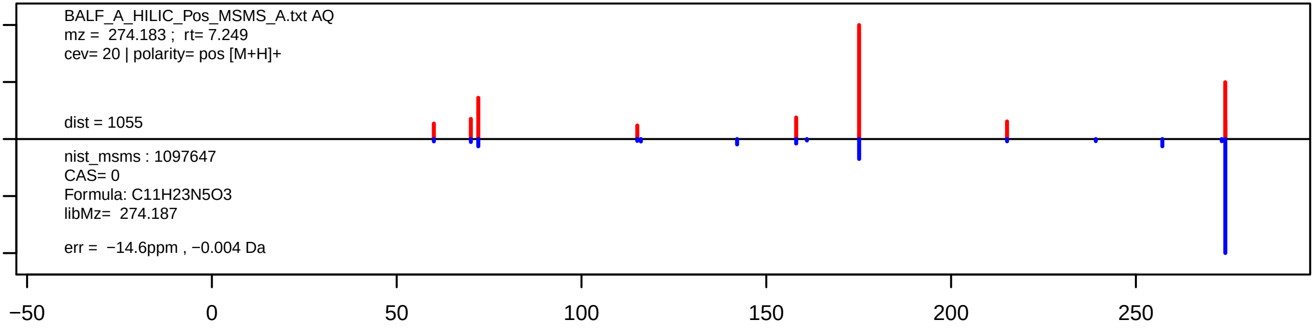

347 . Val−Ile
Score=351 Dot=966 prob=92.9

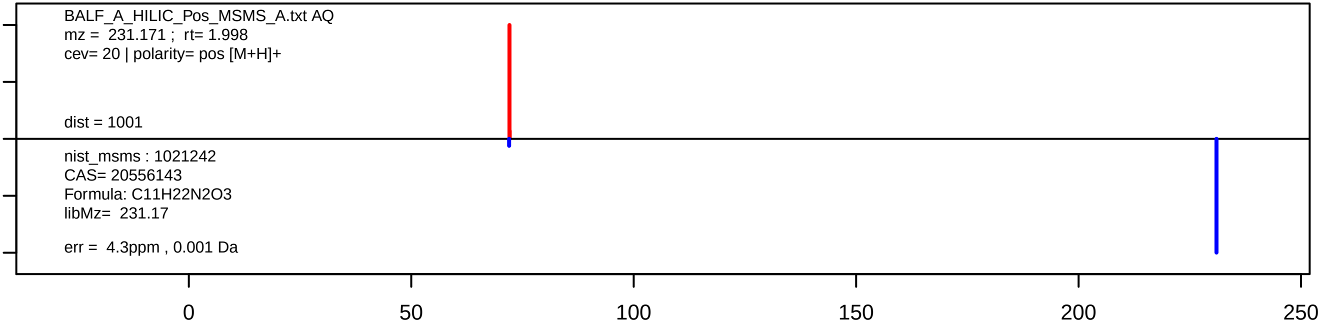

348 . Val−Leu
Score=618 Dot=955 prob=69.5

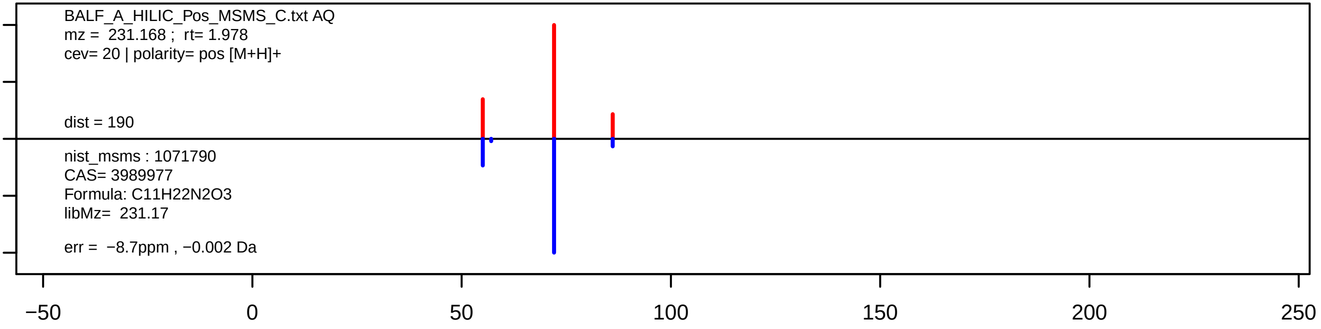

349 .   Val−Tyr
Score=468 Dot=860 prob=48.5

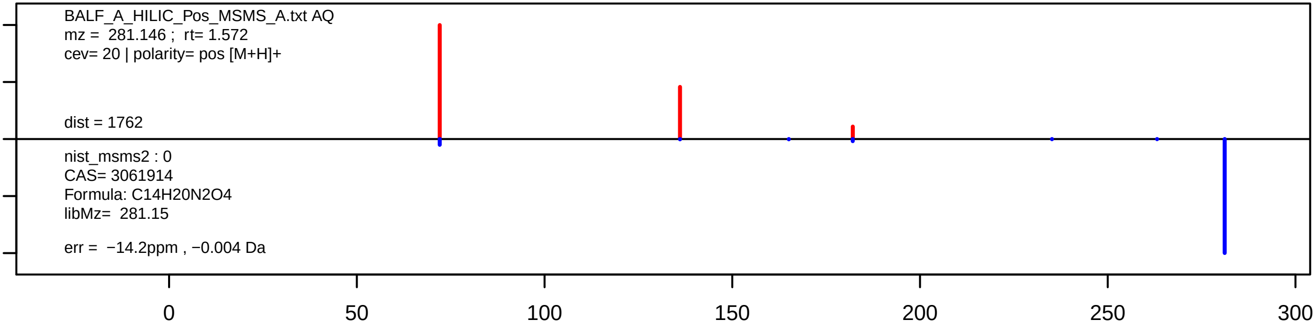

350 .   Val−Val
Score=425 Dot=955 prob=97.4

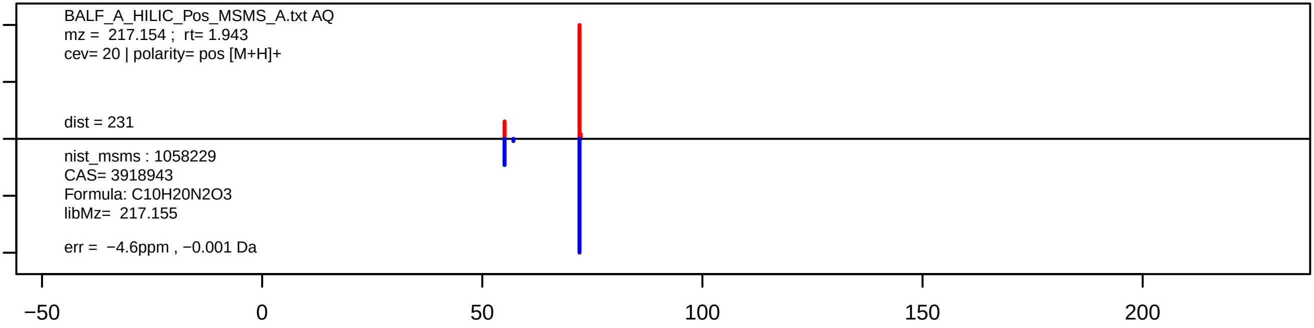

351 .   Venlafaxine
Score=562 Dot=943 prob=98.5

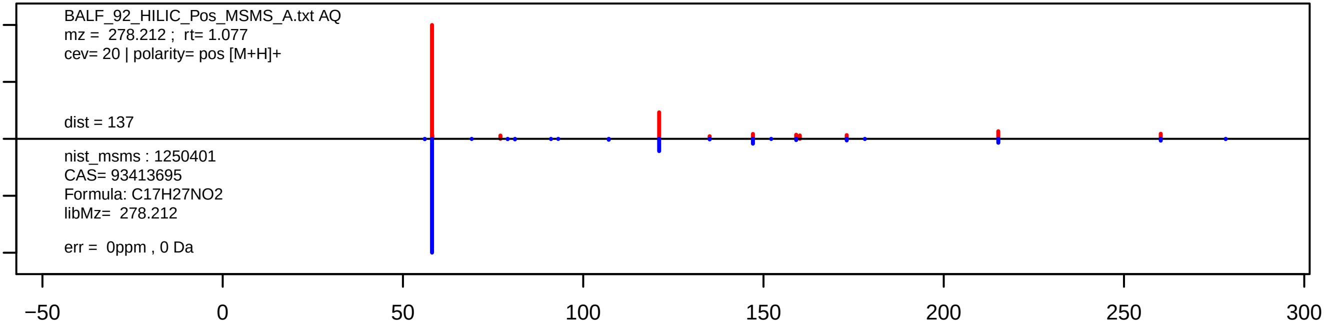

352 . Xanthine
Score=682 Dot=858 prob=98.9

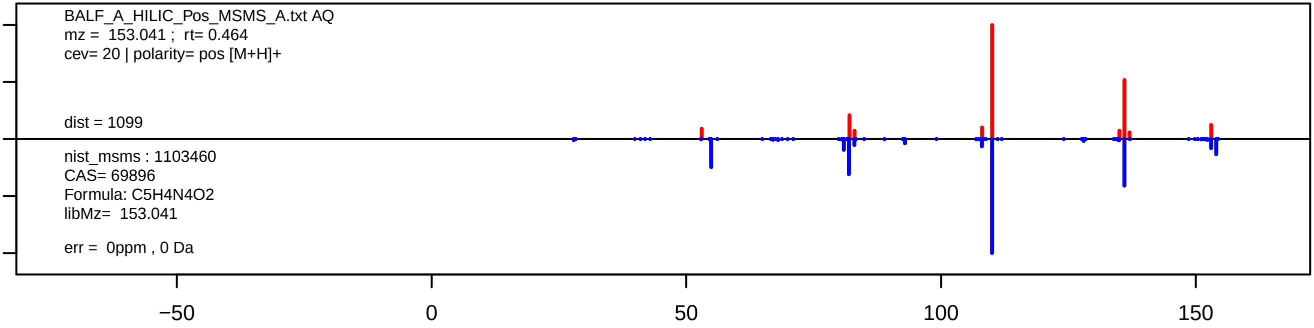

353 . .beta.−Methylphenethylamine
Score=490 Dot=894 prob=73.2

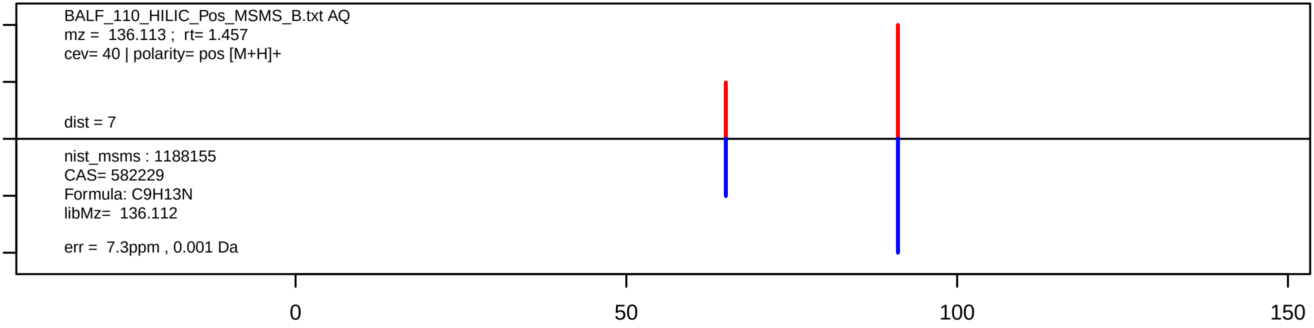

354 . (−)−Cotinine
Score=283 Dot=823 prob=49.3

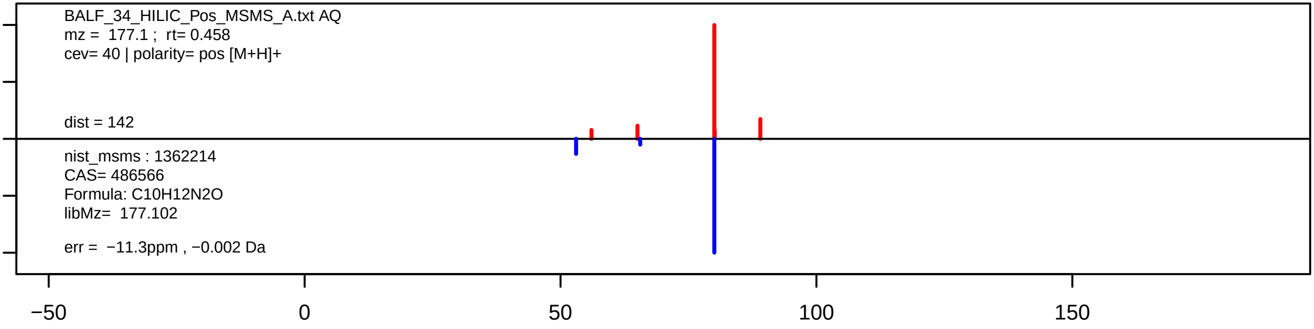

355 . (−)−Methamphetamine
Score=618 Dot=921 prob=91.4

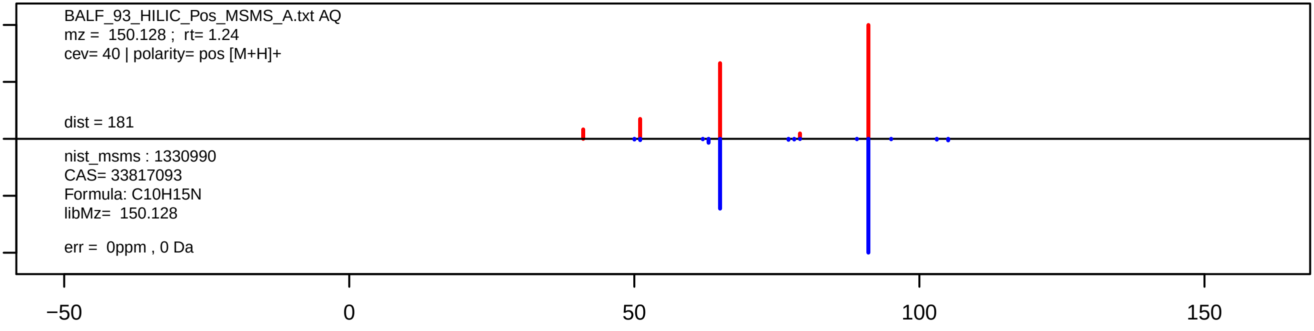

356 . (+)−Methamphetamine
Score=646 Dot=979 prob=91.8

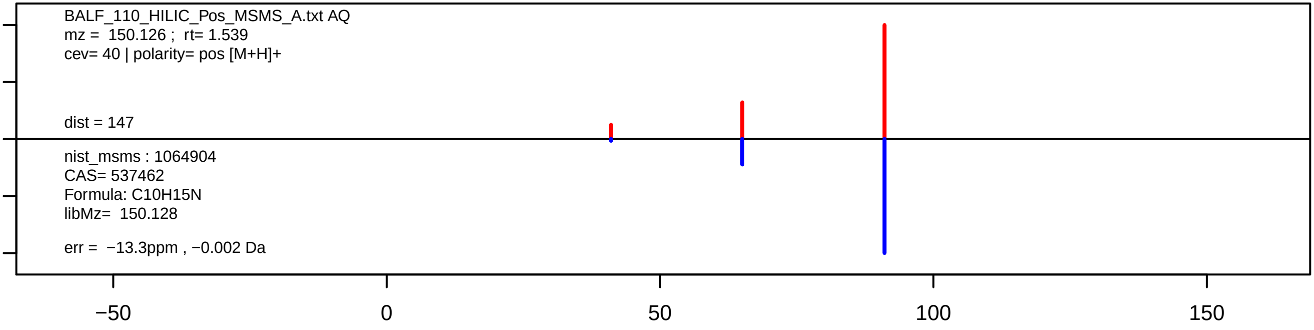

357 . (3−Carboxypropyl)trimethylammonium cation
Score=573 Dot=859 prob=98.2

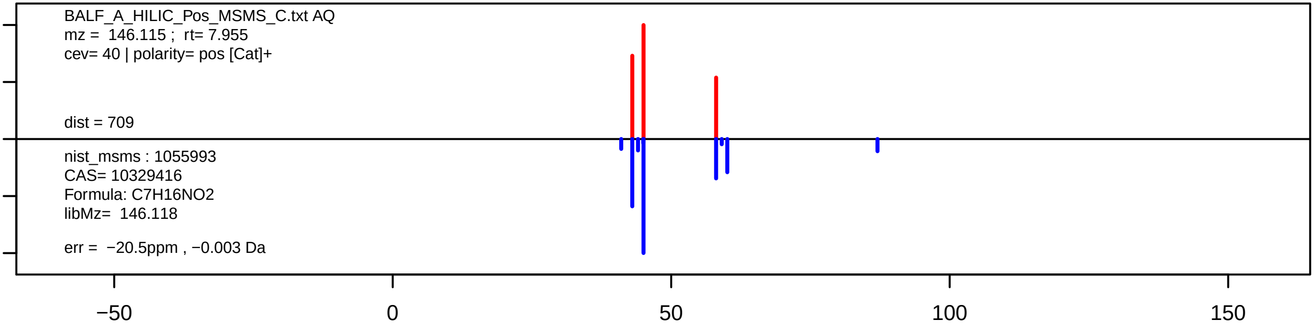

358 .   1−Methyl−3−phenylpropylamine
Score=604 Dot=953 prob=47.5

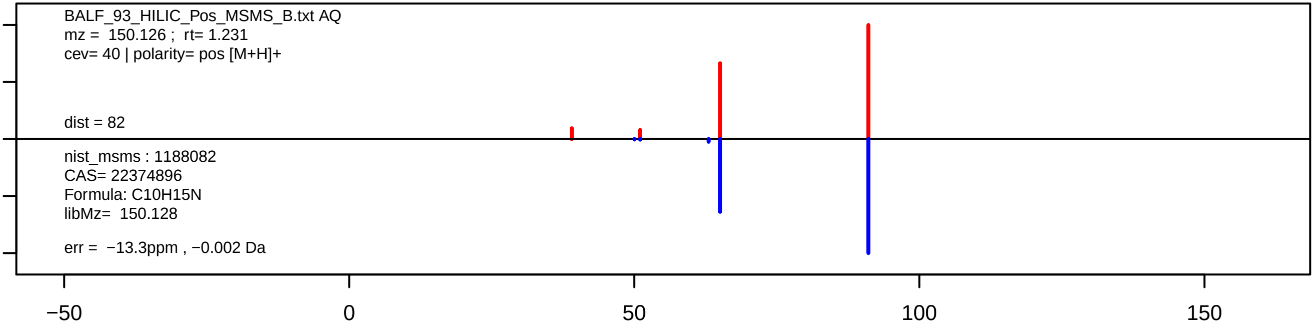

359 .   1−Oleoyl−2−palmitoyl−sn−glycero−3−phosphocholine
Score=359 Dot=873 prob=89.1

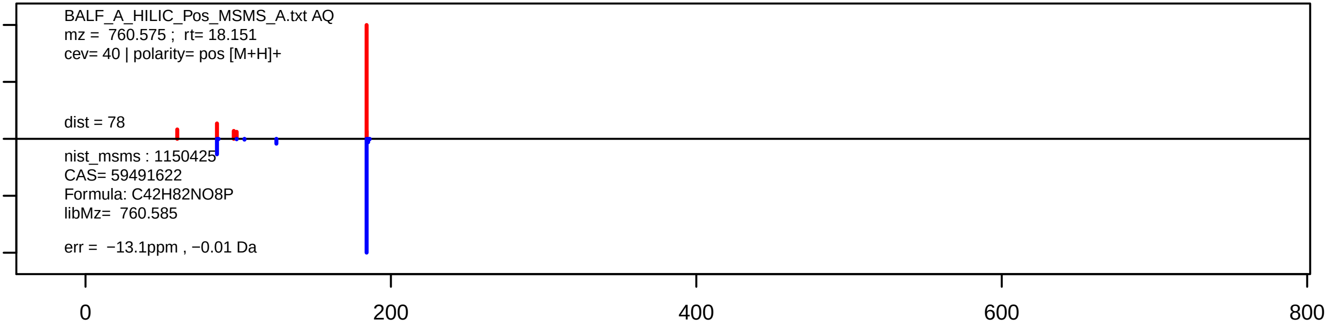

360 .   1−Palmitoyl−2−linoleoyl−sn−glycero−3−phosphocholine
Score=404 Dot=918 prob=80.2

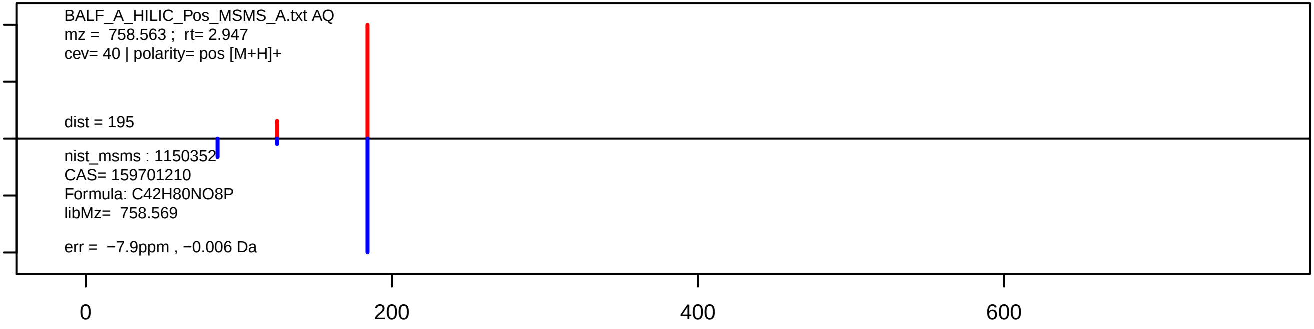

361 . 1−Propanone, 1−(1,3−benzodioxol−5−yl)−2−(dimethylamino)−
Score=846 Dot=918 prob=98.5

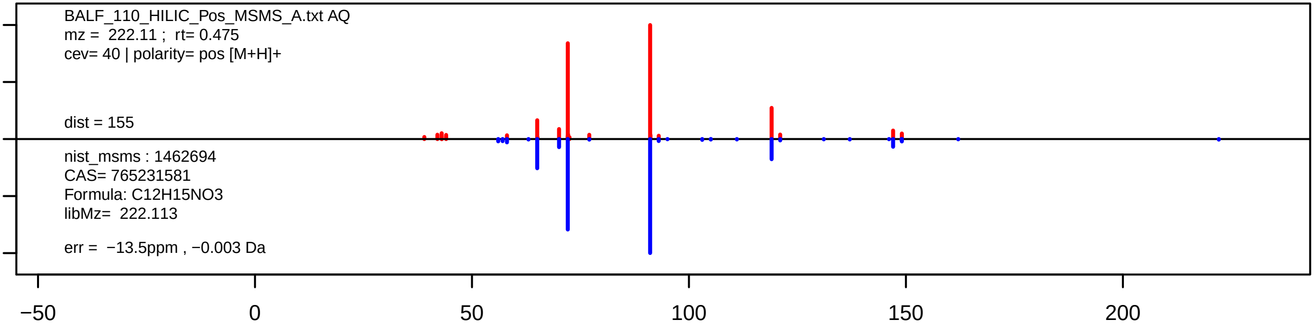

362 . 1,2−Dihexadecanoyl−sn−glycero−3−phosphocholine
Score=318 Dot=952 prob=42.9

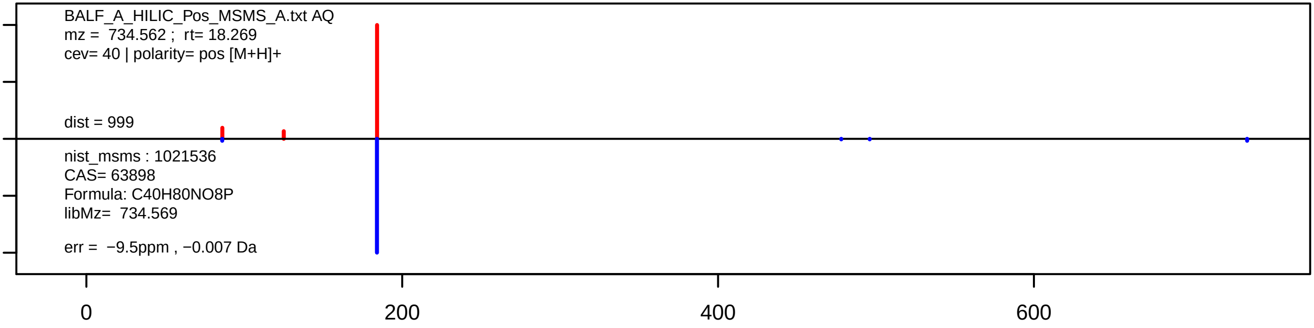

363 . 1,2−dioleoyl−sn−glycero−3−phosphatidylcholine
Score=400 Dot=999 prob=50

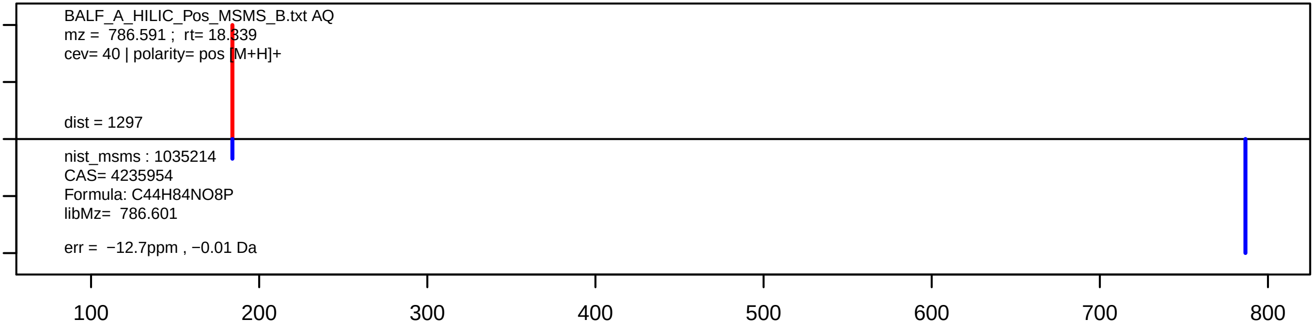

364 . 1,2−Dipalmitoyl−sn−glycero−O−ethyl−3−phosphatidylcholine cation
Score=665 Dot=965 prob=91.8

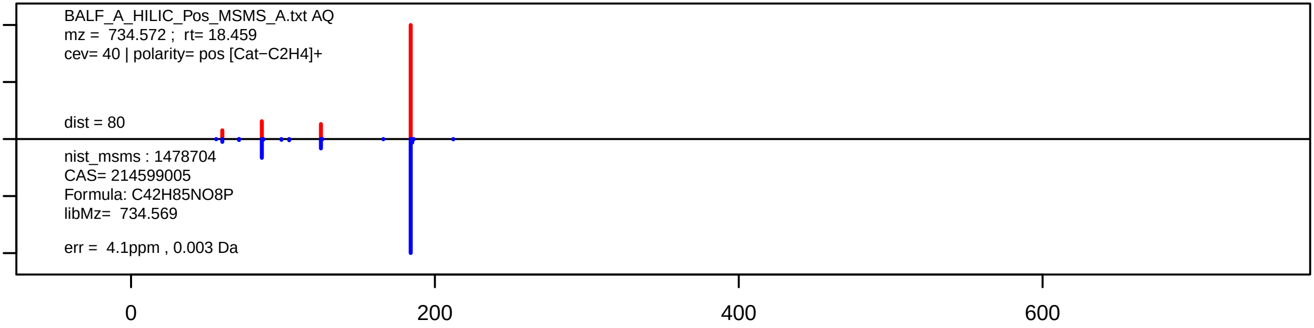

365 . 1,2,4−Triazin−5−amine, N−([1,1'−biphenyl]−4−ylmethyl)−6−phenyl−3−(2−pyridinyl)−
Score=868 Dot=906 prob=47.4

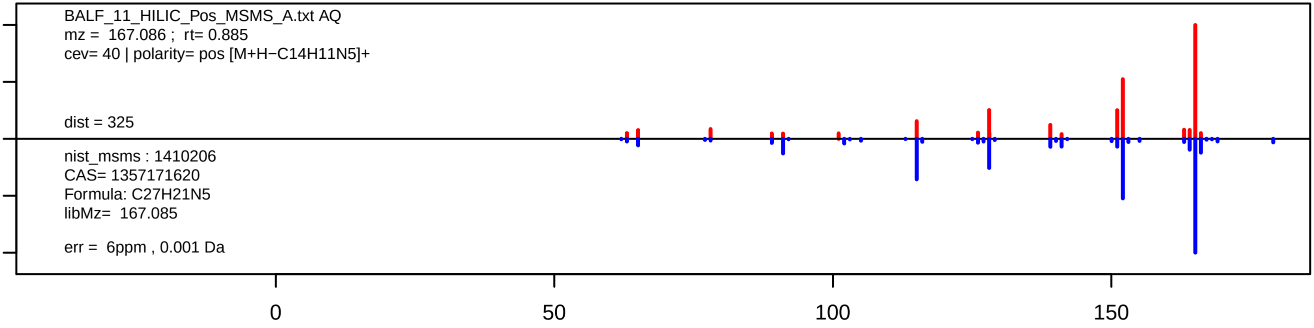

366 . 2−[(2,6−Dimethylphenyl)amino]−N,N,N−triethyl−2−oxoethanaminium cation
Score=559 Dot=999 prob=52.6

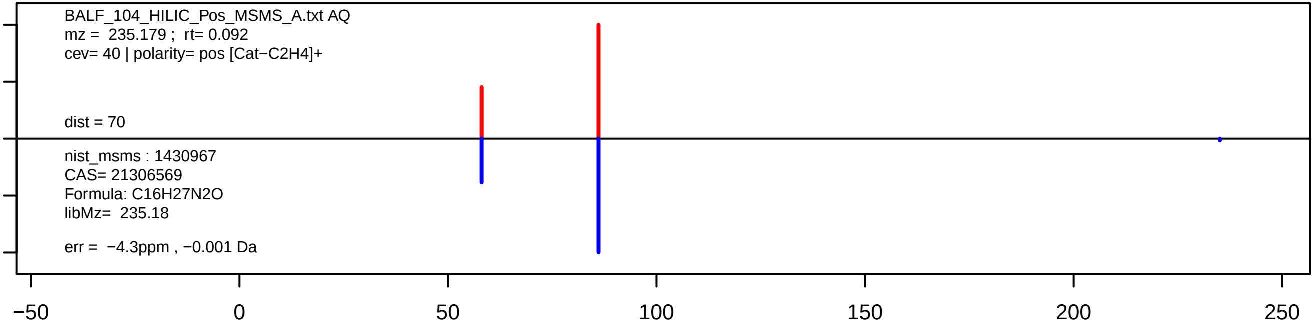

367 . 2−Amino−1−phenylbutane
Score=552 Dot=991 prob=31.7

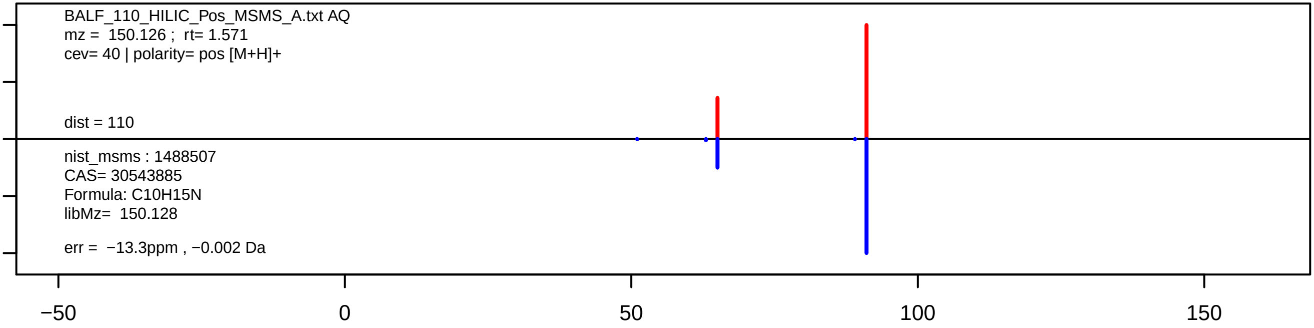

368 . 2−Methylamino−1−(3,4−methylenedioxyphenyl)propan−1−one
Score=847 Dot=853 prob=93.1

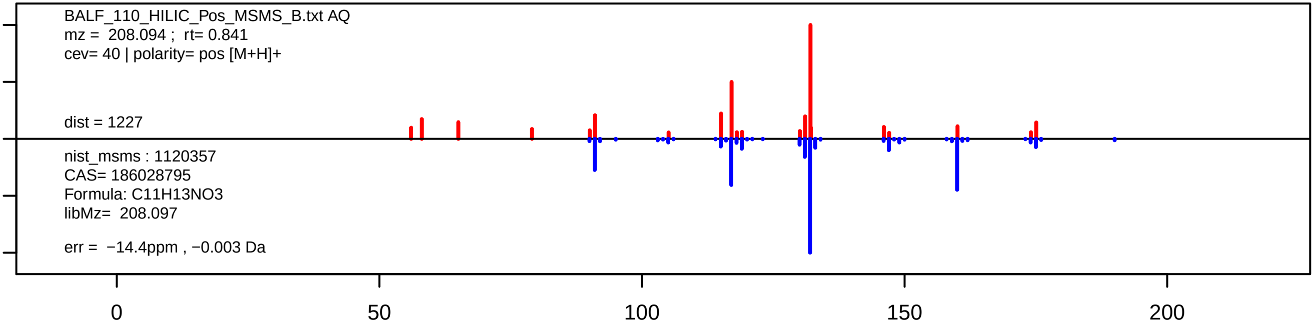

369 . 2−Oleoyl−1−palmitoyl−sn−glycero−3−phosphocholine
Score=364 Dot=972 prob=92.3

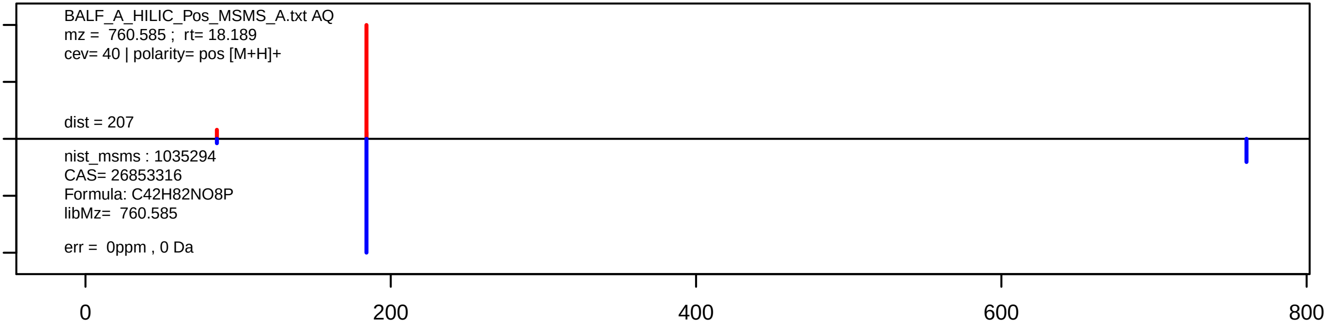

370 . 2'−Deoxyinosine
Score=400 Dot=999 prob=97.2

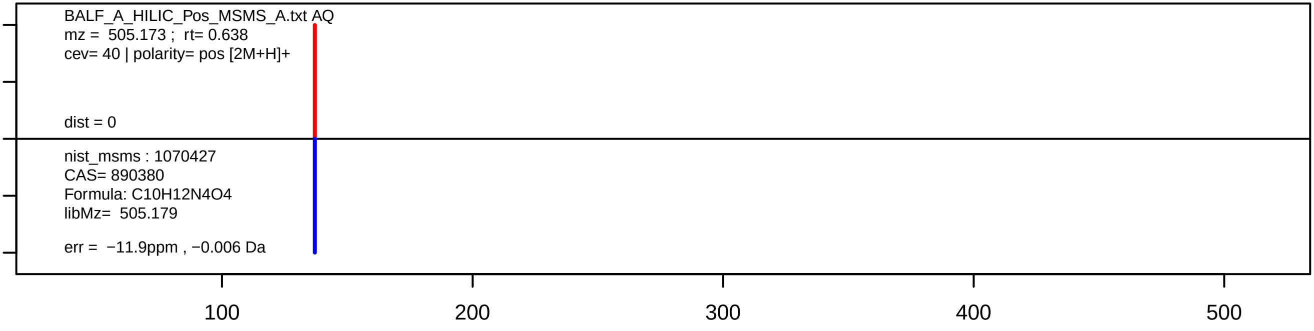

371 . 3−Aminopentanoic acid
Score=306 Dot=926 prob=47.7

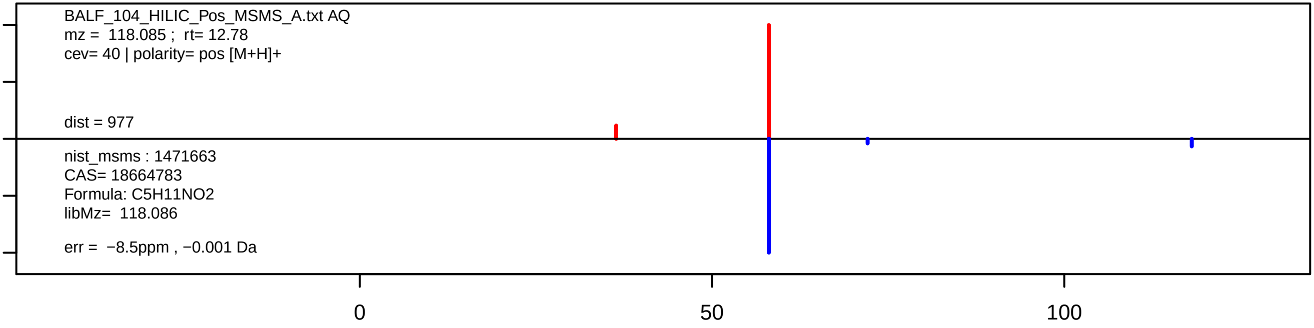

372 . 4−Imidazoleacrylic acid
Score=689 Dot=888 prob=97.6

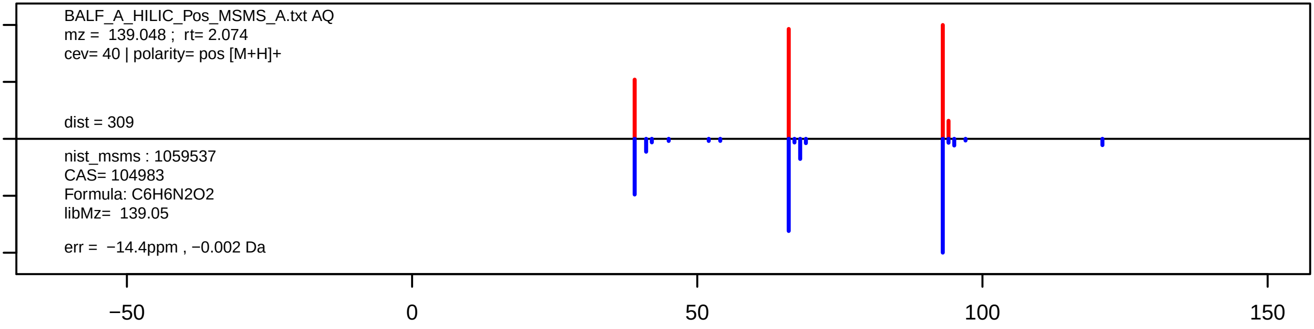

373 .   4−Phenylbutylamine
Score=594 Dot=968 prob=62.1

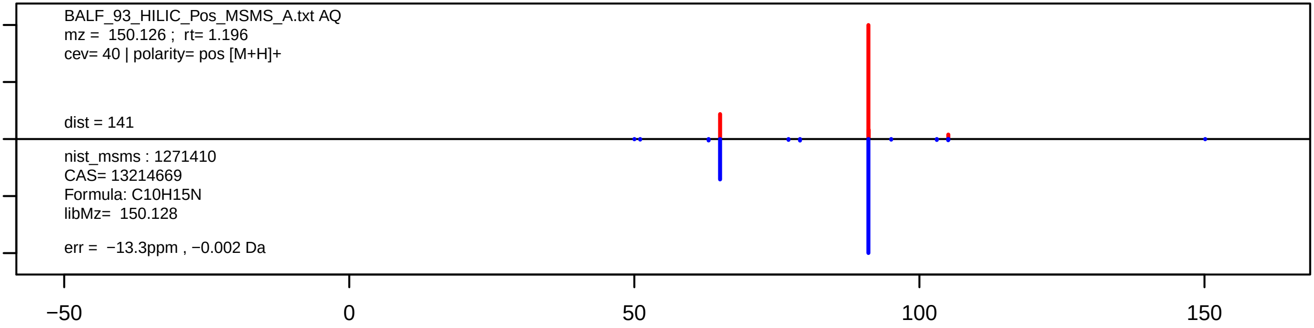

374 .   5−Acetylamino−6−formylamino−3−methyluracil
Score=181 Dot=890 prob=74.7

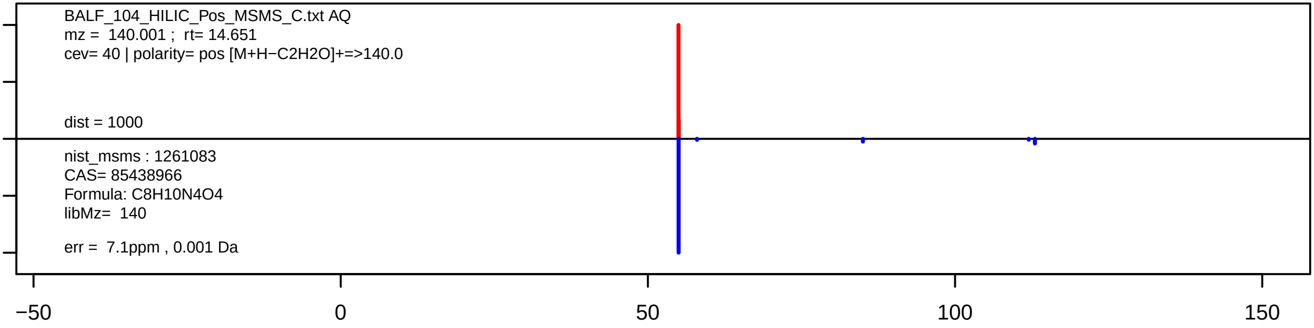

375 .   5−Aminovaleric acid
Score=620 Dot=817 prob=91

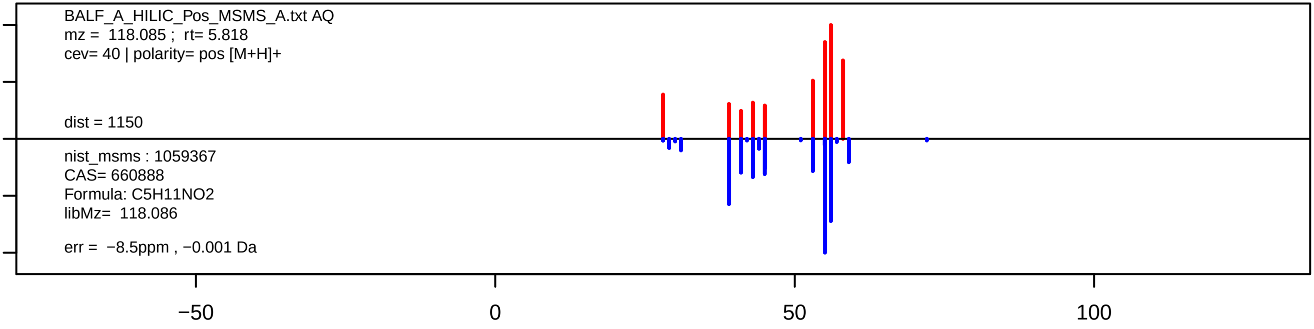

376 .   5,5−Dimethylimidazolidine−2,4−dione
Score=400 Dot=999 prob=3.5

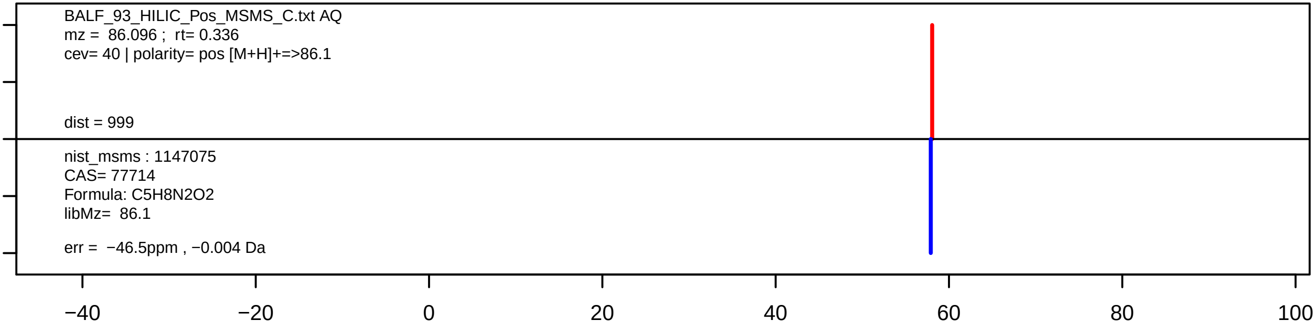

377 .   5.alpha.−Androstan−17.beta.−ol−3−one
Score=400 Dot=999 prob=32.9

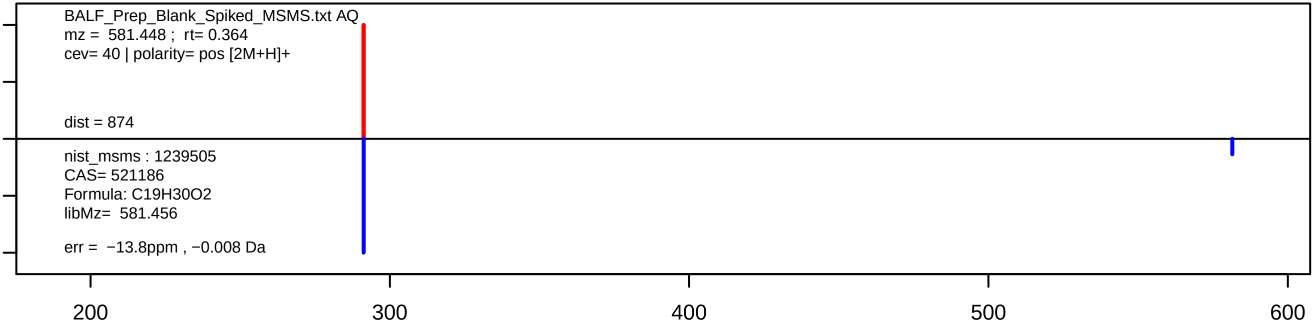

378 .   5'−S−Methyl−5'−thioadenosine
Score=179 Dot=845 prob=75

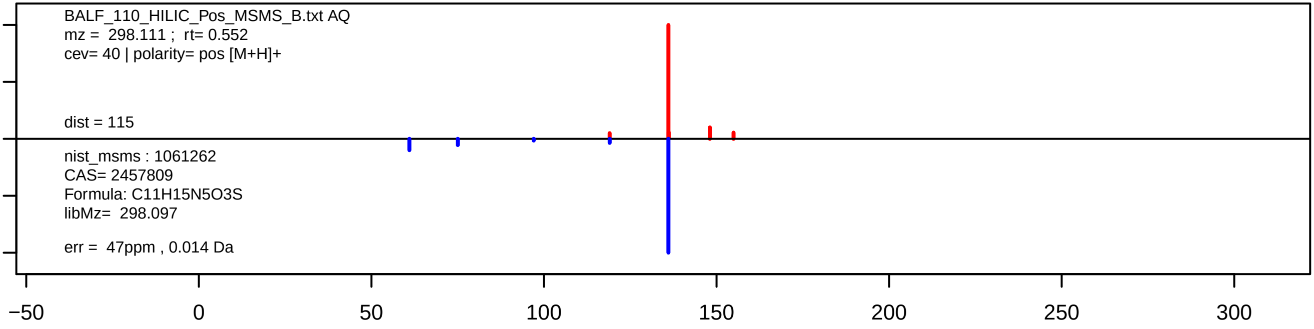

379 . Acetyl−DL−carnitine
Score=816 Dot=906 prob=86.9

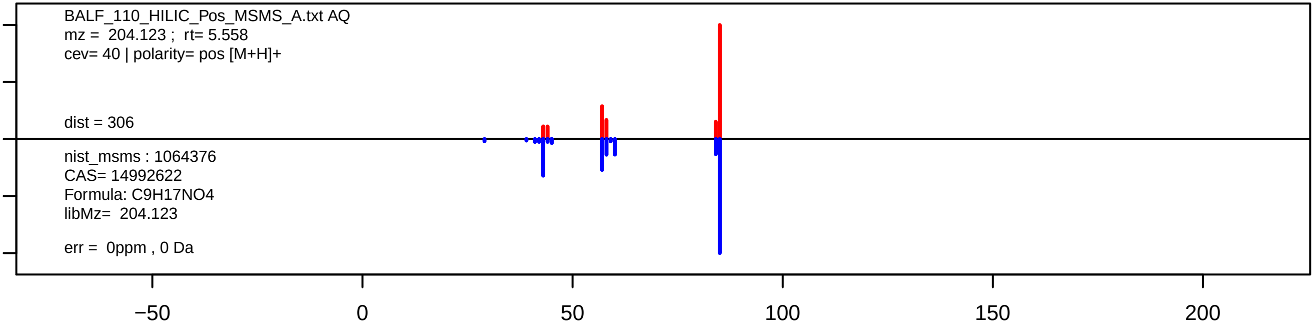

380 . Acetyl−L−carnitine
Score=606 Dot=901 prob=64.3

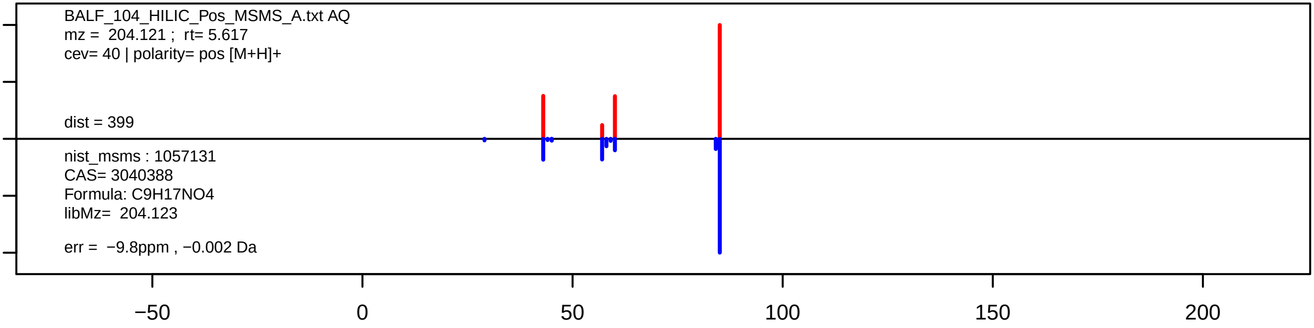

381 . Adenosine
Score=300 Dot=802 prob=91.3

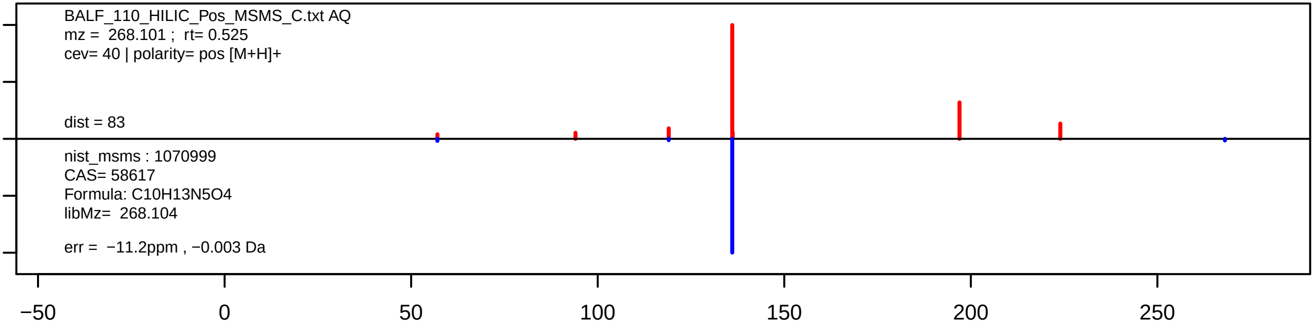

382 . Ala−Ala−Lys
Score=403 Dot=909 prob=98.5

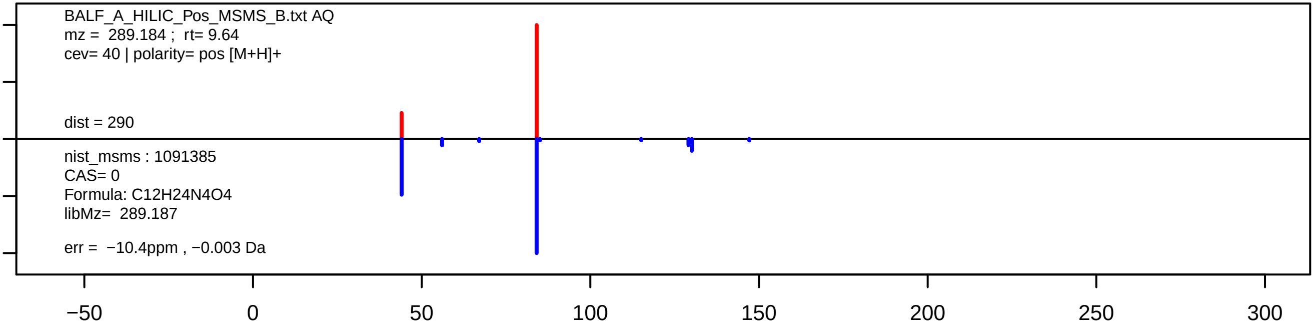

383 . Ala−His
Score=751 Dot=901 prob=75.3

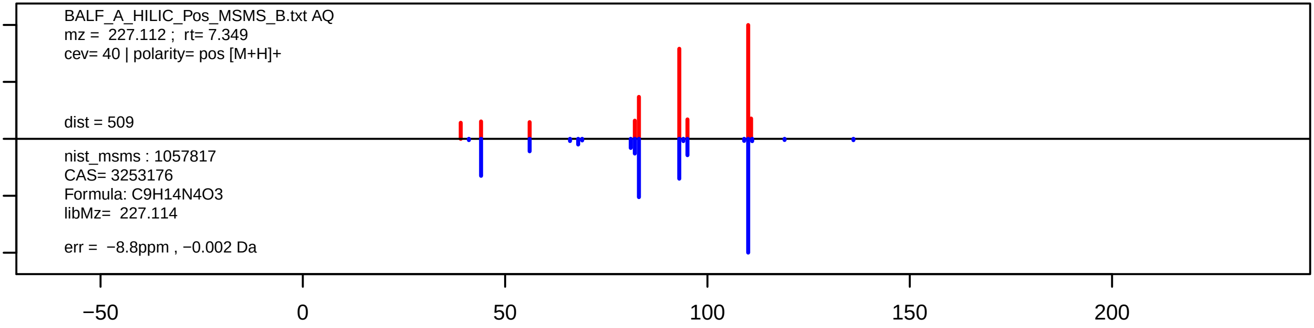

384 . Ala−Phe
Score=329 Dot=861 prob=91.8

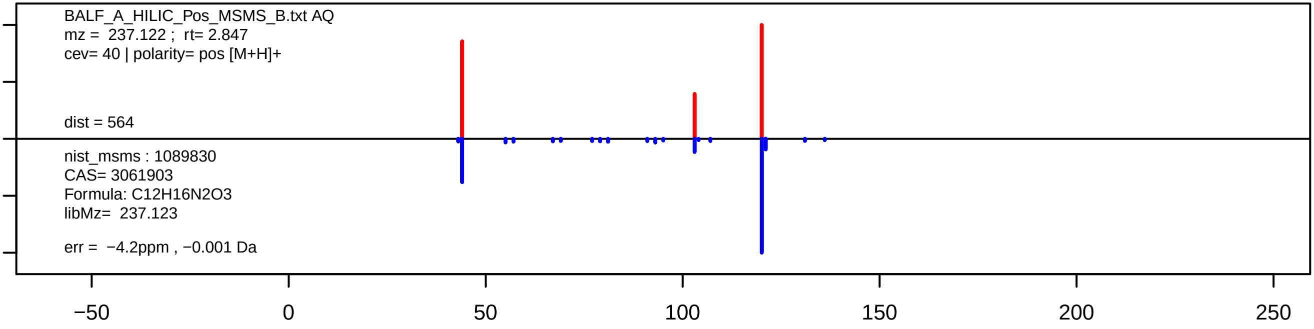

385 . Ala−Pro
Score=359 Dot=944 prob=41.8

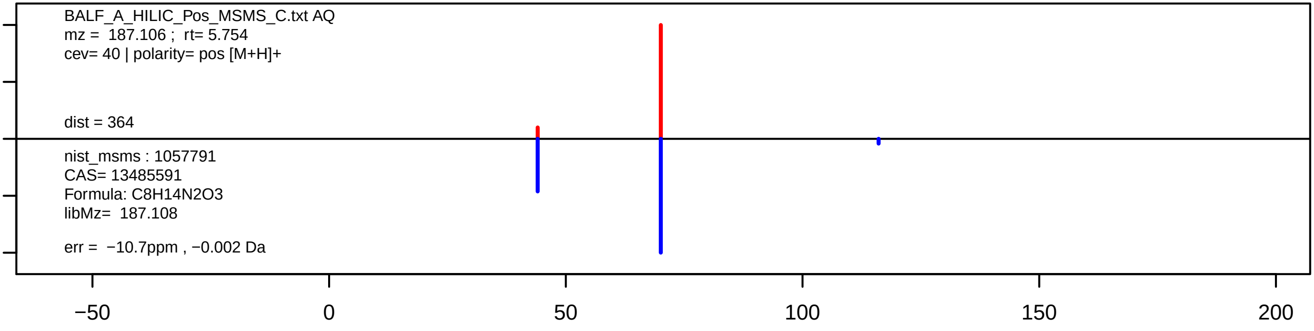

386 . Albuterol
Score=706 Dot=832 prob=97.9

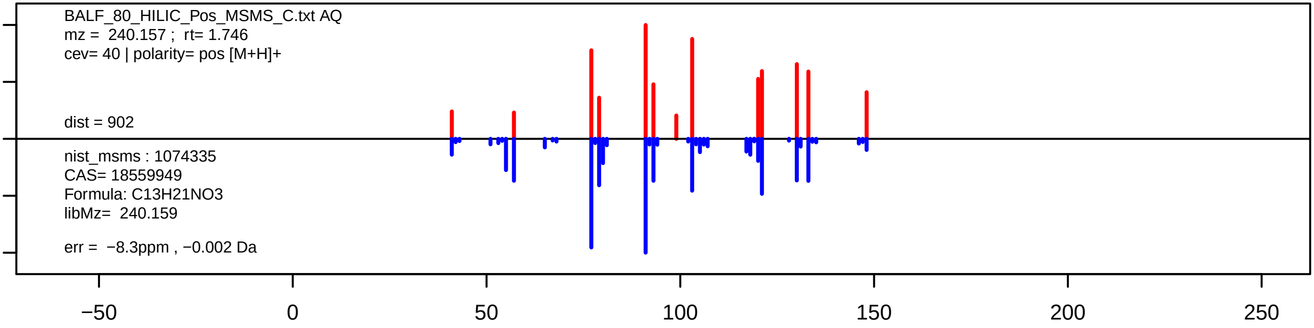

387 . Aminodiphenylmethane
Score=541 Dot=835 prob=59.2

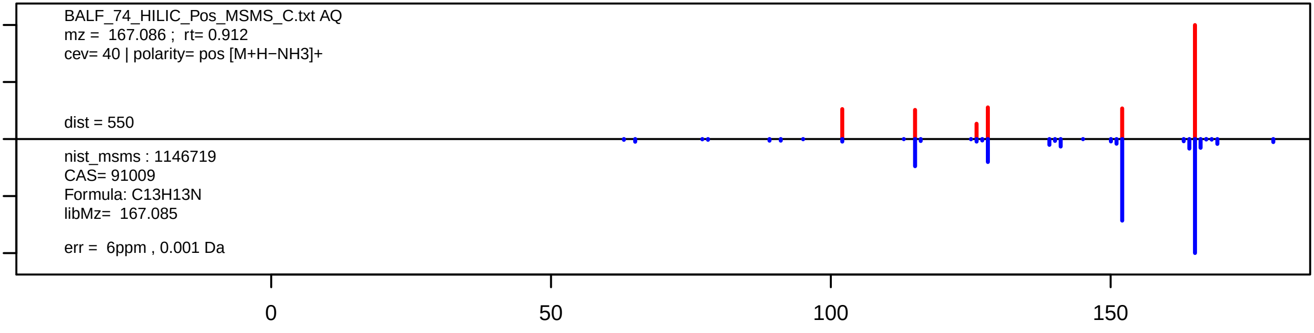

388 . Amphetamine
Score=593 Dot=886 prob=33.9

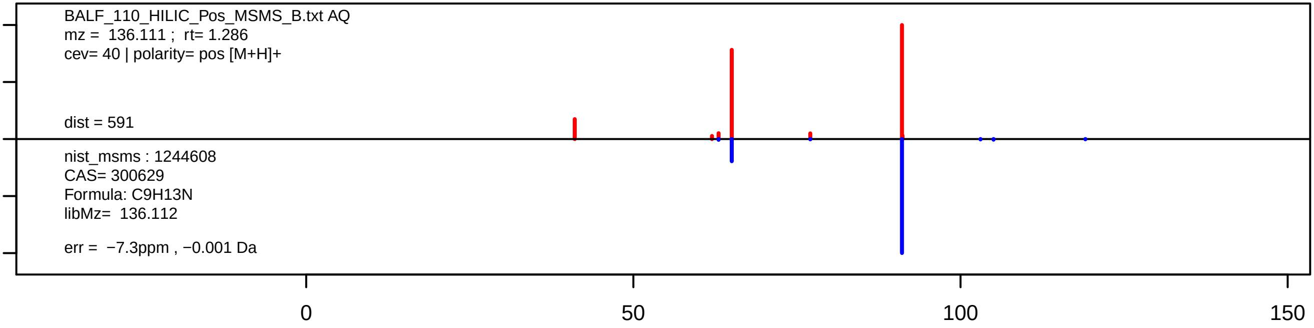

389 . Arg−Leu
Score=119 Dot=857 prob=27

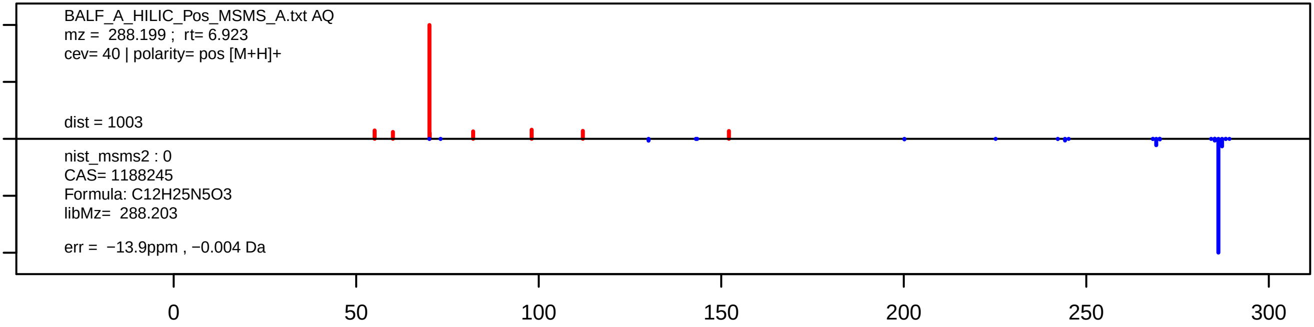

390 . Arg−Ser
Score=308 Dot=836 prob=34

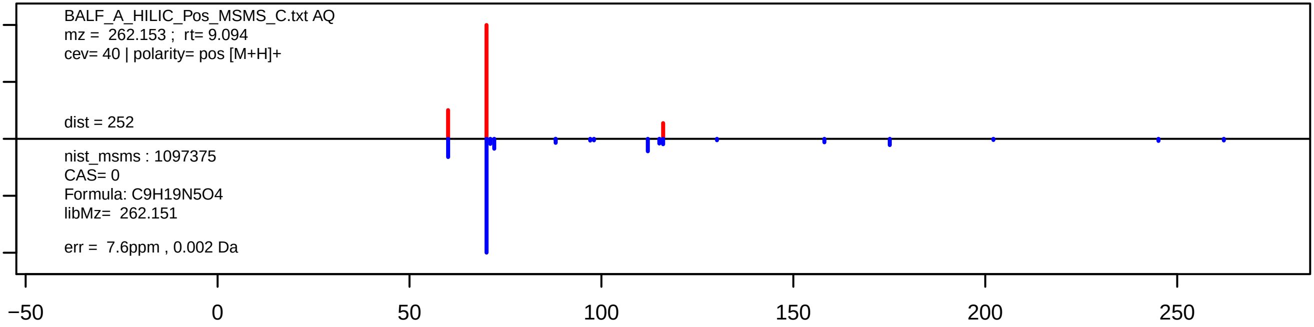

391 . Arg−Thr
Score=66 Dot=901 prob=29.2

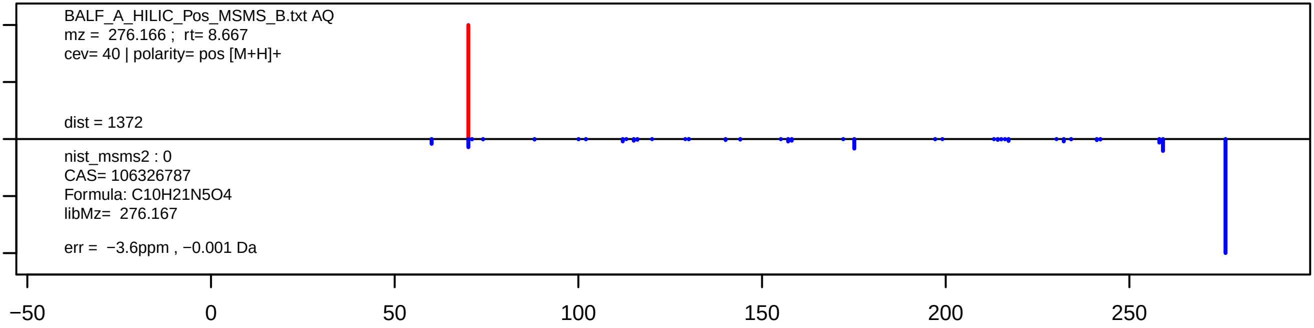

392 . Arg−Val
Score=162 Dot=814 prob=47.1

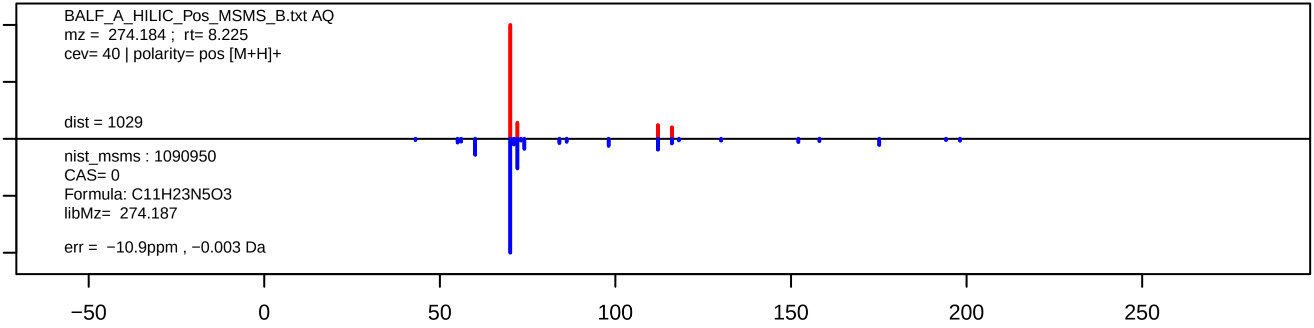

393 . Benzhydrol
Score=646 Dot=865 prob=56.4

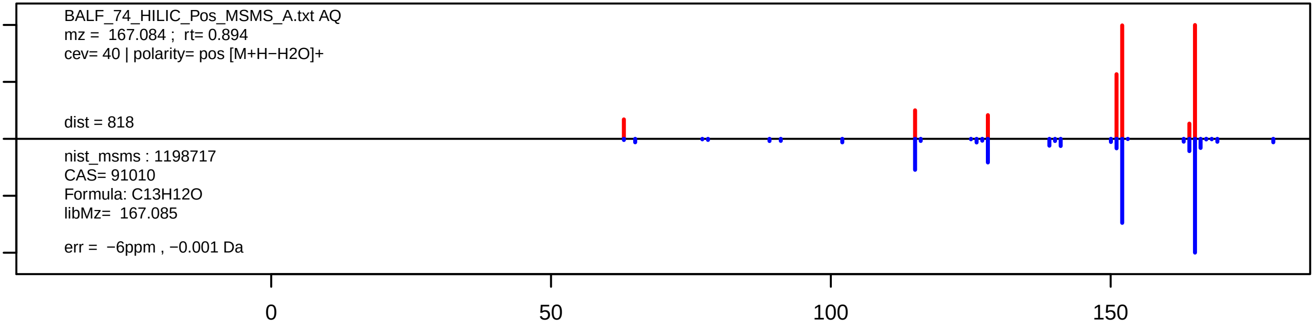

394 . Benzyl alcohol
Score=826 Dot=909 prob=97.8

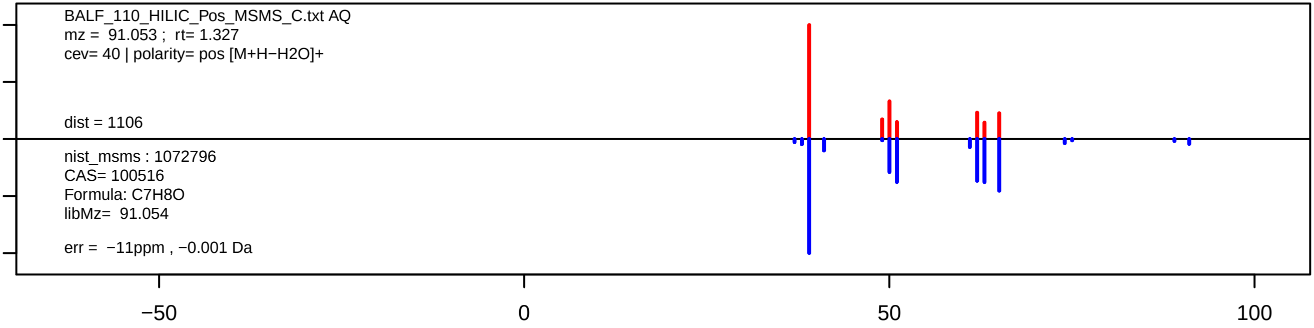

395 . Betaine
Score=358 Dot=959 prob=81.8

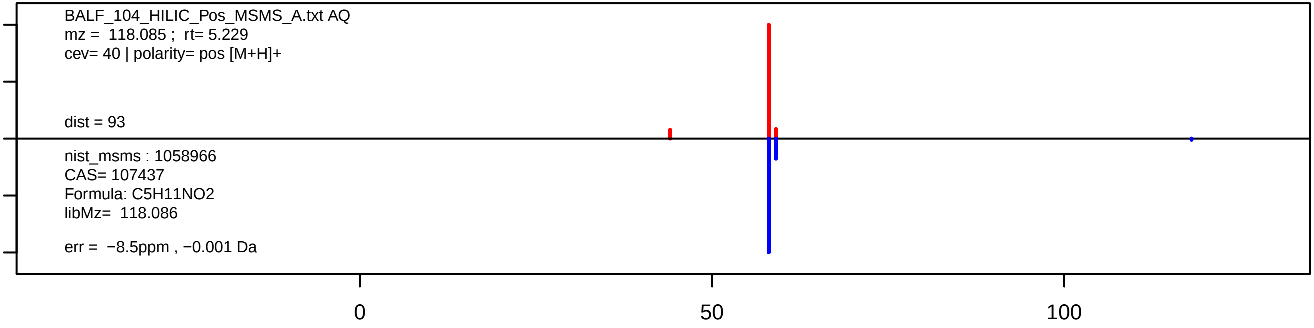

396 . Bis(2,2,6,6−tetramethyl−4−piperidyl) sebacate
Score=310 Dot=979 prob=96.5

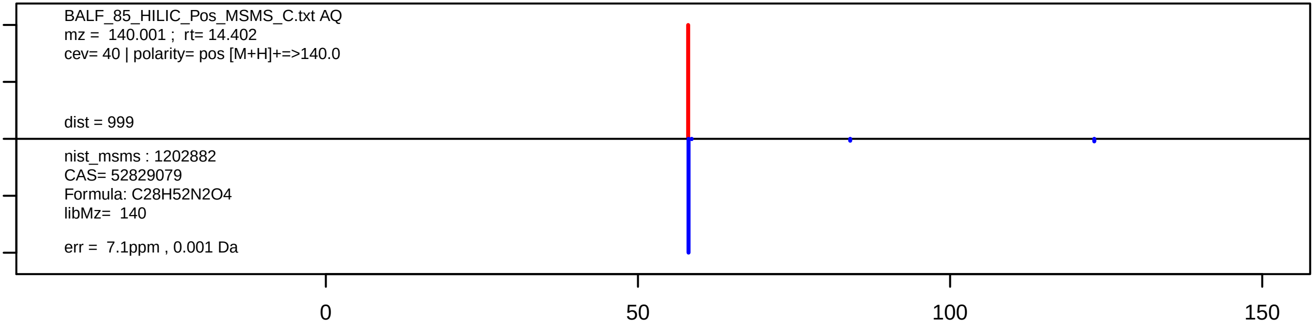

397 . Blood Group A Trisaccharide
Score=584 Dot=907 prob=91.3

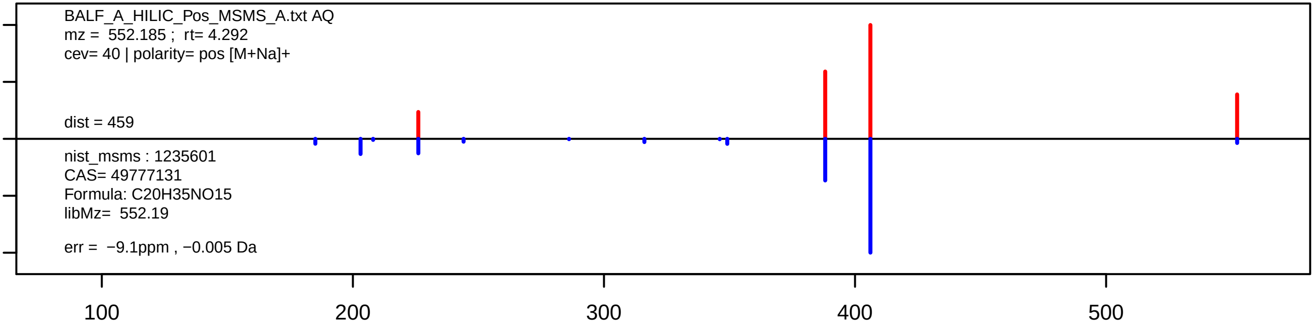

398 . Bupropion
Score=820 Dot=905 prob=97.8

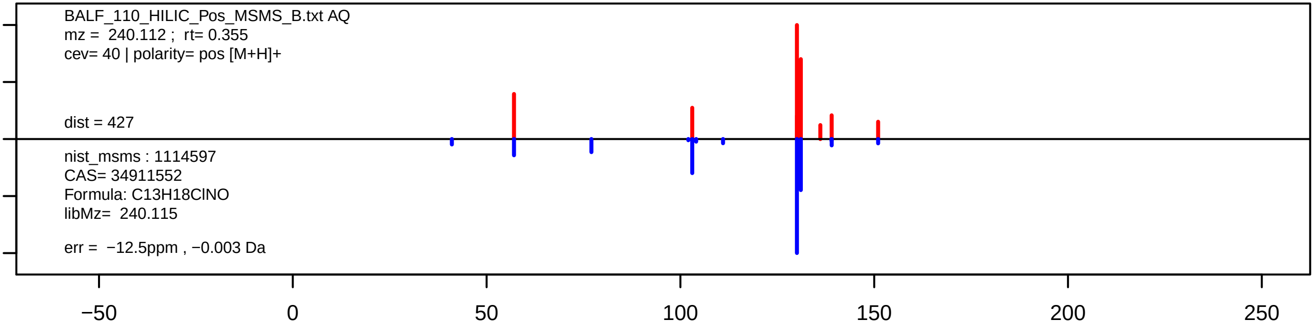

399 . Choline cation
Score=525 Dot=873 prob=90.5

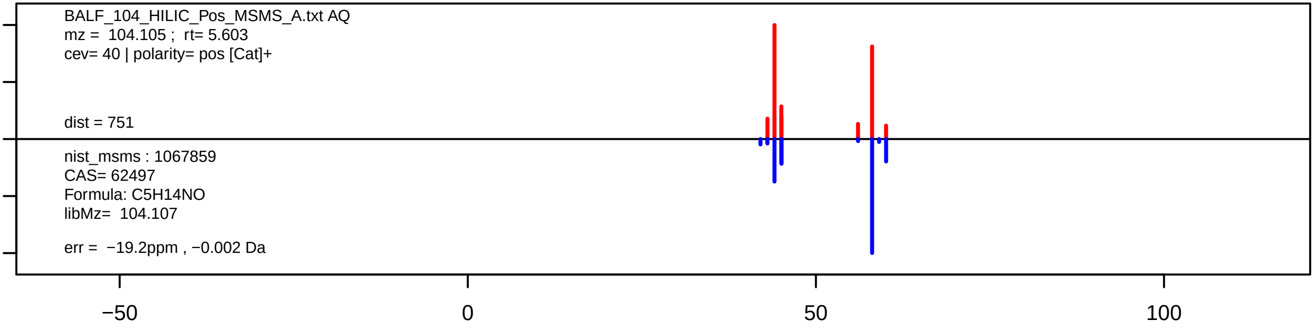

400 . Citalopram
Score=505 Dot=832 prob=96.5

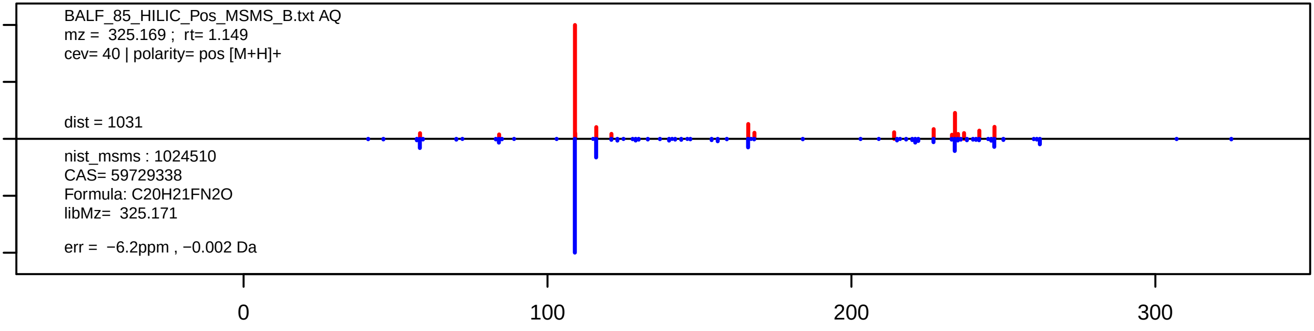

401 . Creatinine
Score=534 Dot=931 prob=99

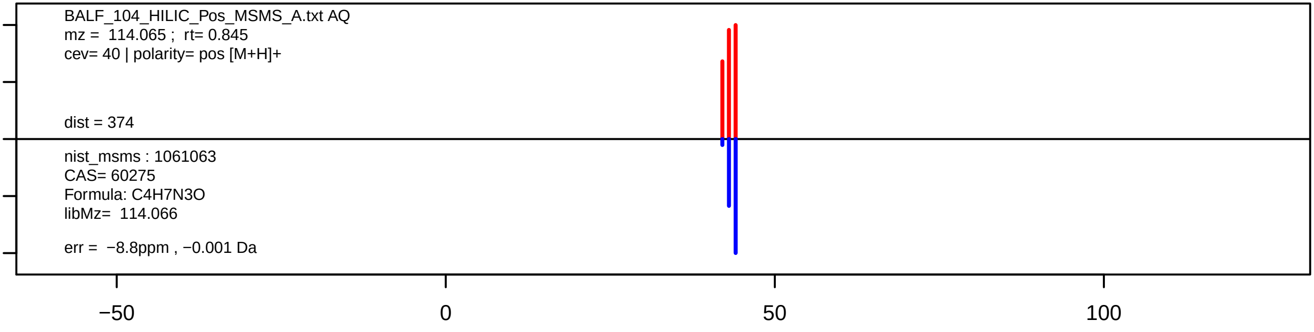

402 . Cyclizine
Score=435 Dot=863 prob=95.9

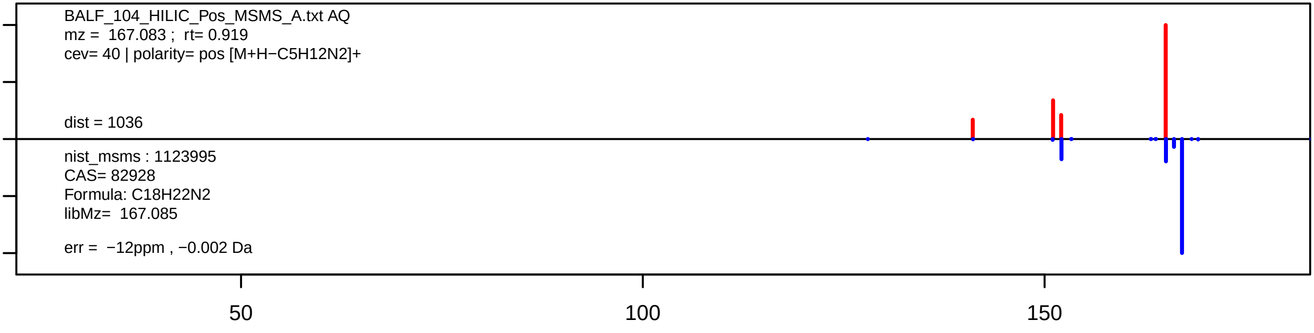

403 .   Cyclohexylamine
Score=735 Dot=947 prob=82.7

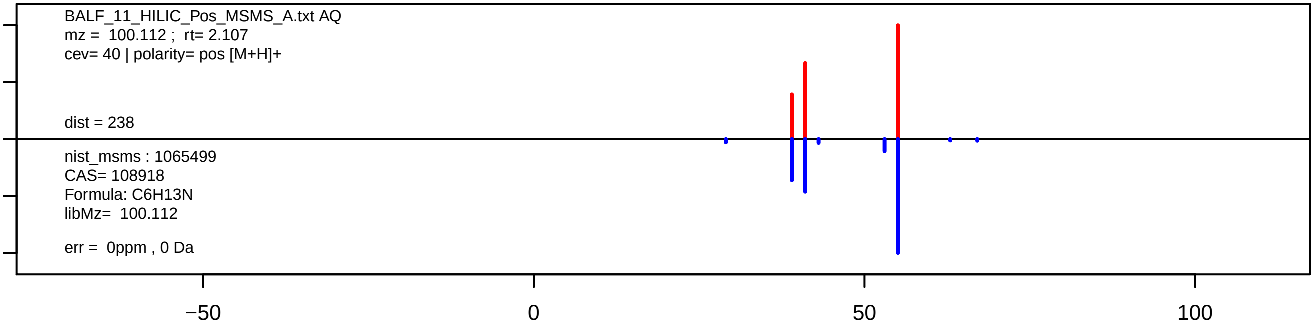

404 .   D−Ornithine
Score=561 Dot=891 prob=81.2

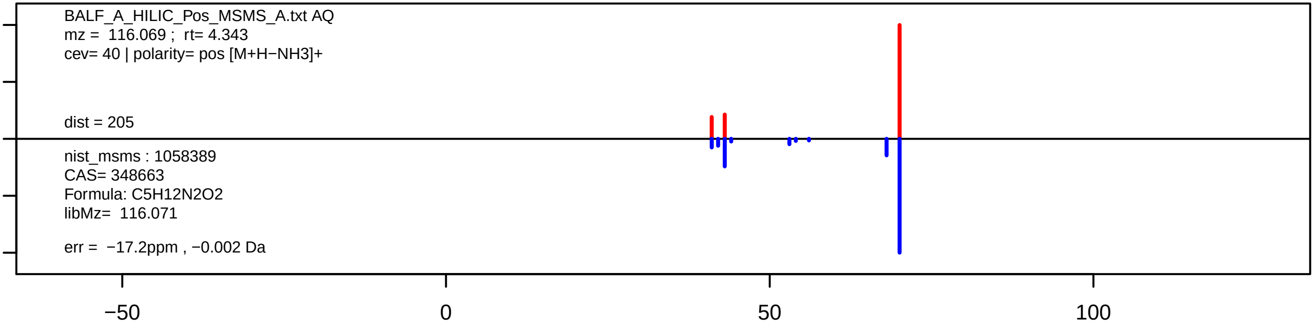

405 .   Dextromethorphan
Score=462 Dot=831 prob=91.9

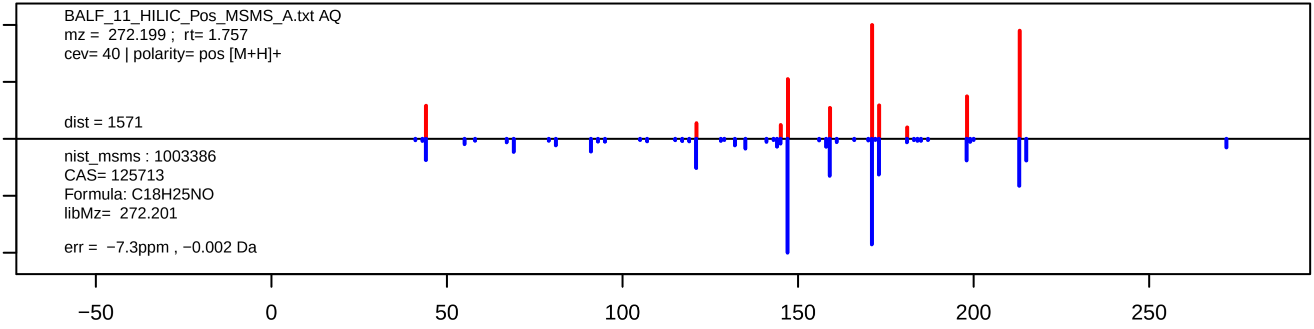

406 . Di(2−nonyl) phthalate
Score=373 Dot=994 prob=34.9

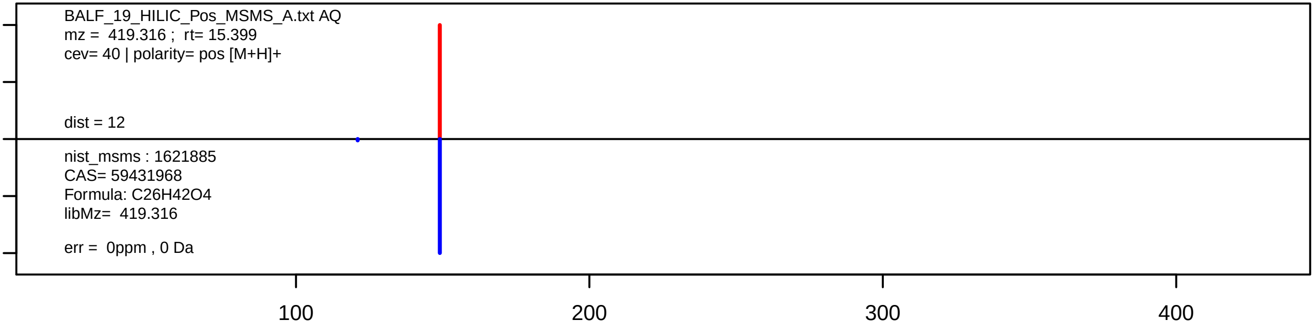

407 . Di(3,7−dimethyl−1−octyl) phthalate
Score=706 Dot=901 prob=57

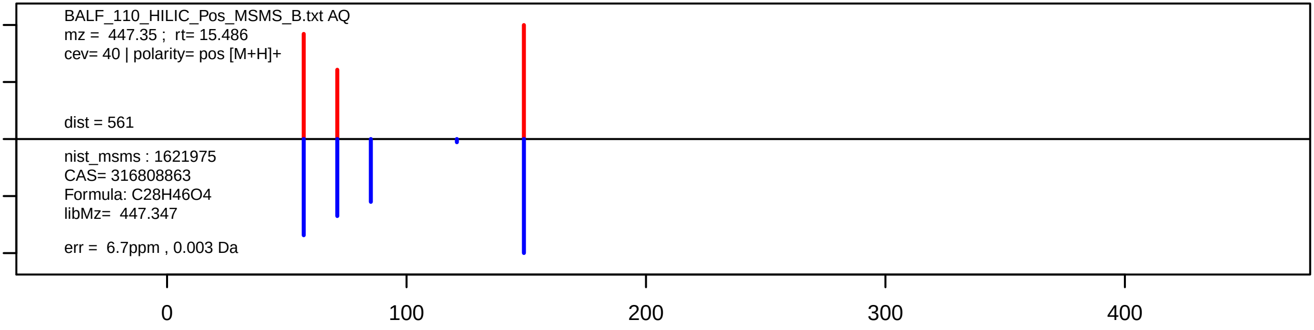

408 . Dibenzylamine
Score=388 Dot=923 prob=96.4

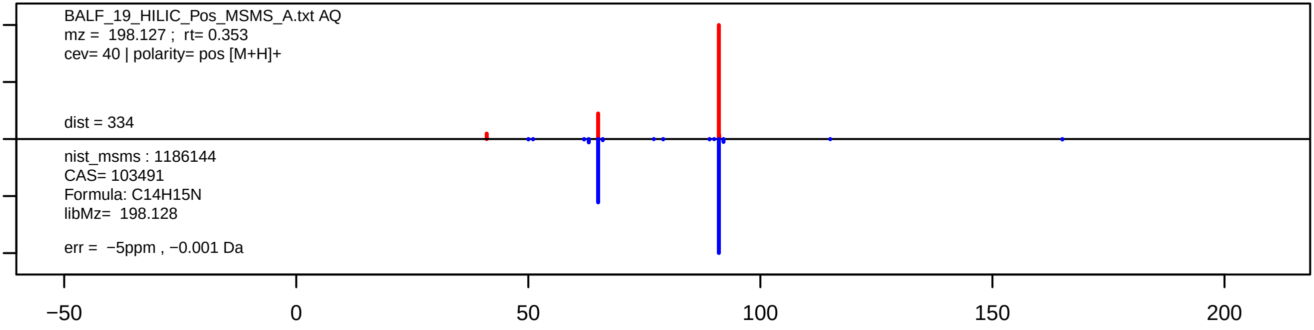

409 . Diisodecyl phthalate
Score=305 Dot=828 prob=57.5

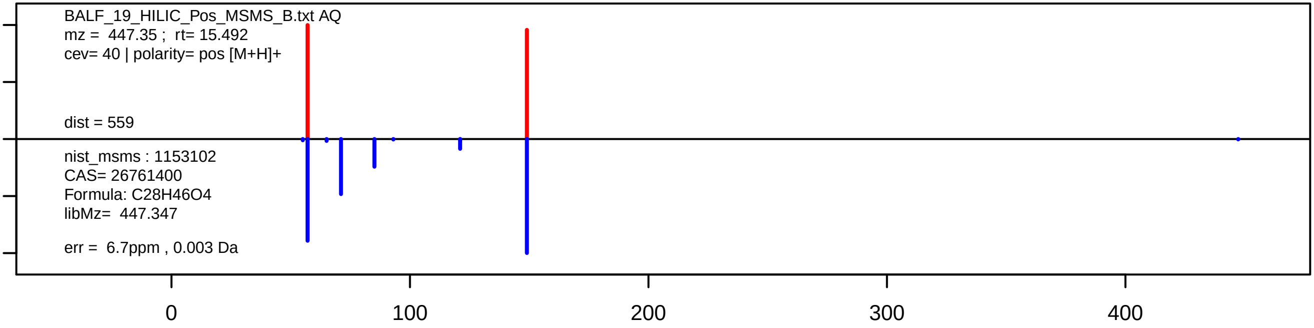

410 . Diisooctyl phthalate
Score=687 Dot=907 prob=96.2

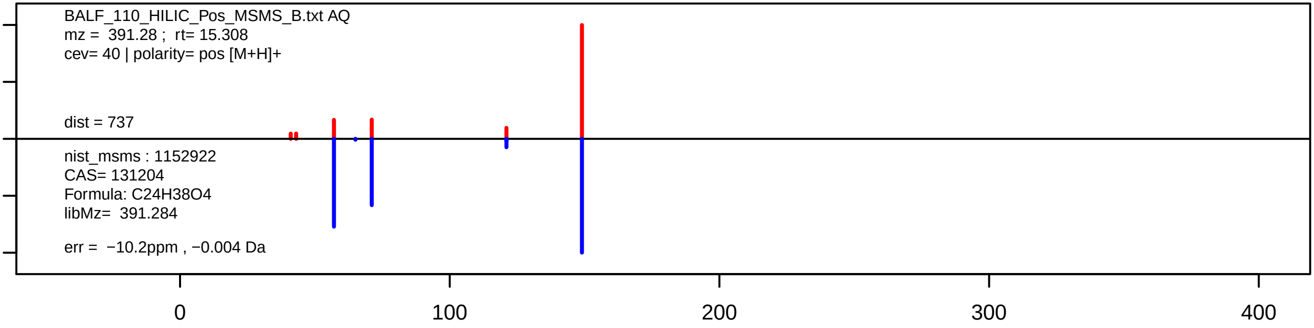

411 . Dioctyl phthalate
Score=307 Dot=879 prob=84.1

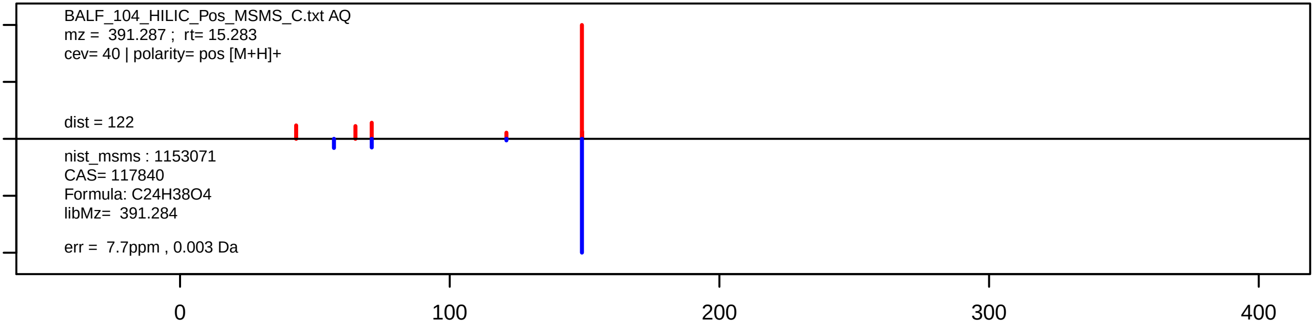

412 . Diphenhydramine
Score=921 Dot=952 prob=98.8

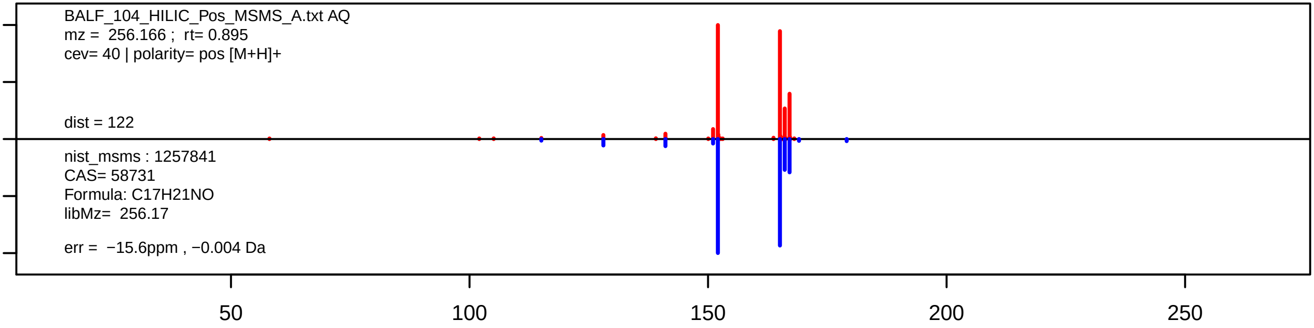

413 . DL−Arginine
Score=400 Dot=891 prob=71.6

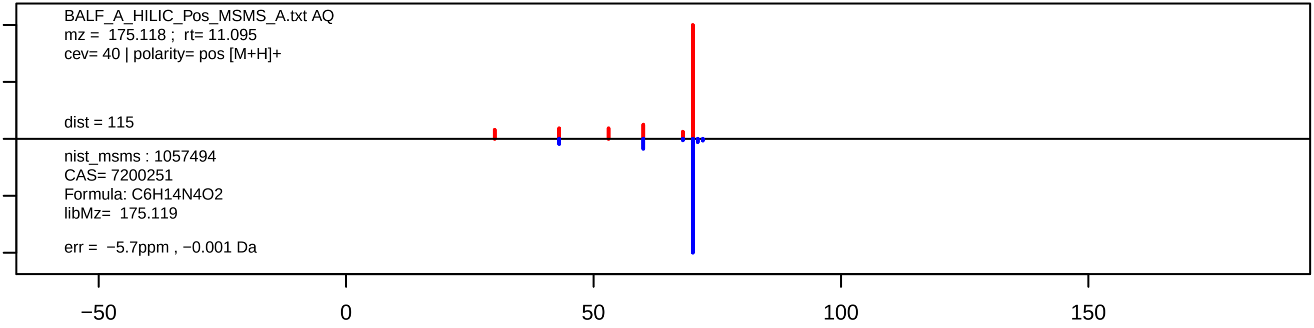

414 . DL−Ornithine
Score=393 Dot=982 prob=49.5

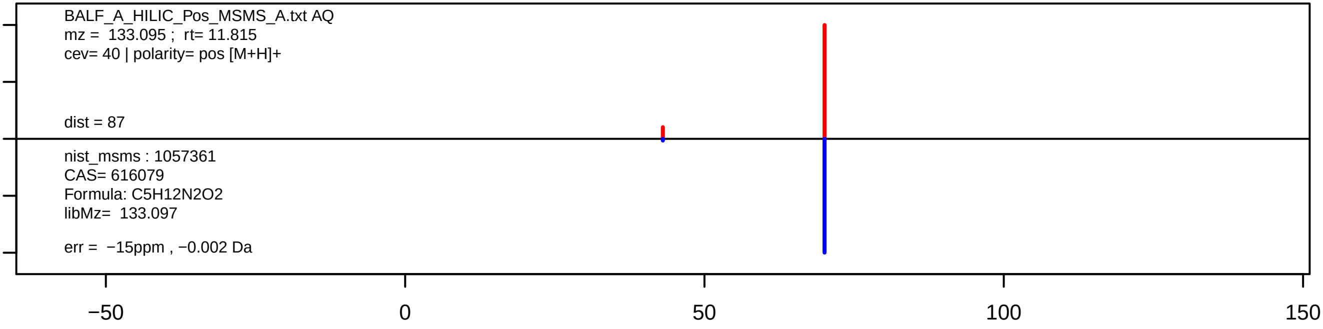

415 . DL−Phenylalanine
Score=882 Dot=949 prob=48.4

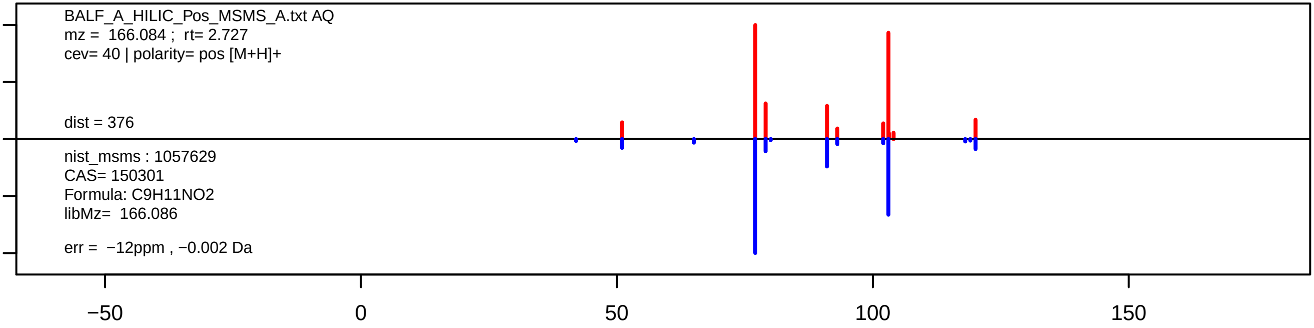

416 . Fluoxetine
Score=373 Dot=991 prob=98.6

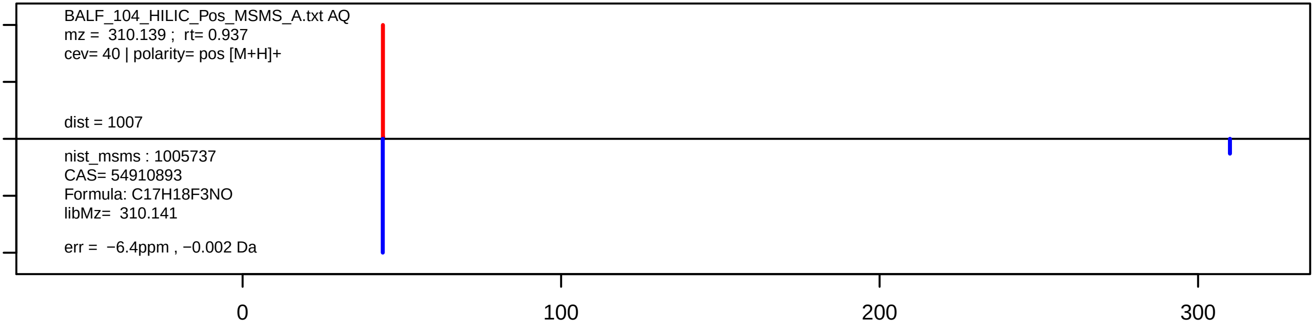

417 . Glycerophosphocholine
Score=600 Dot=885 prob=98.5

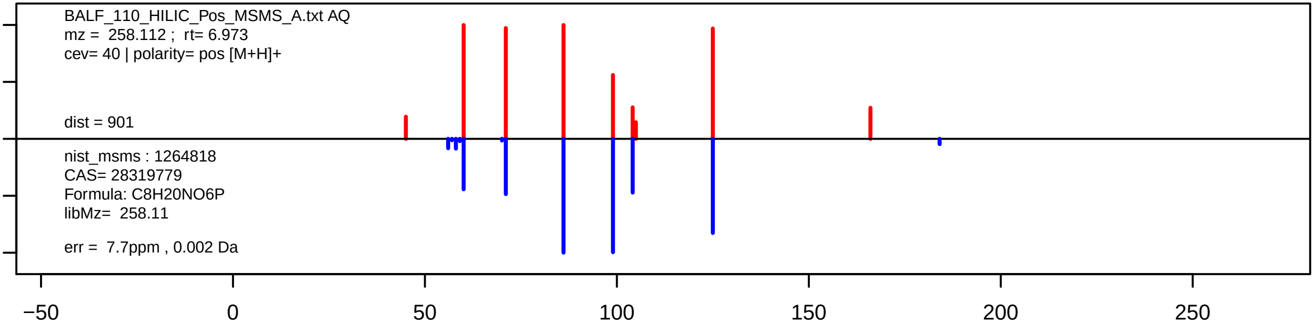

418 . His−Ala
Score=469 Dot=905 prob=45.5

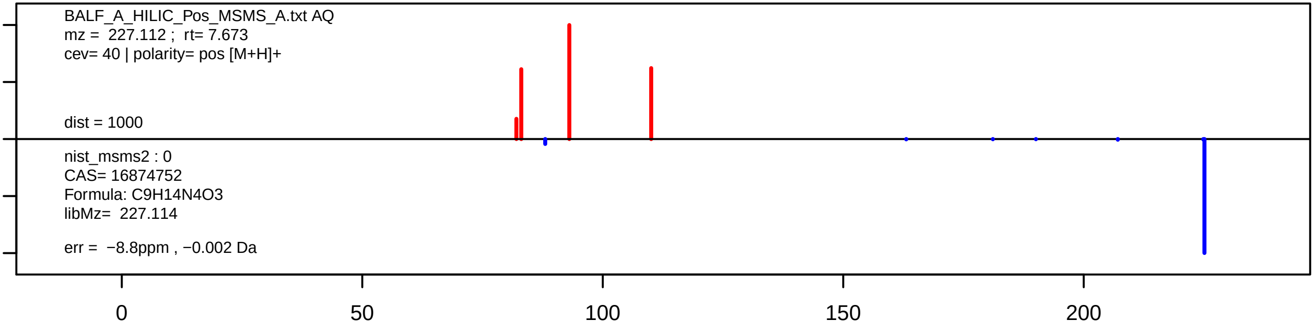

419 . Hydroxybupropion
Score=878 Dot=900 prob=98.4

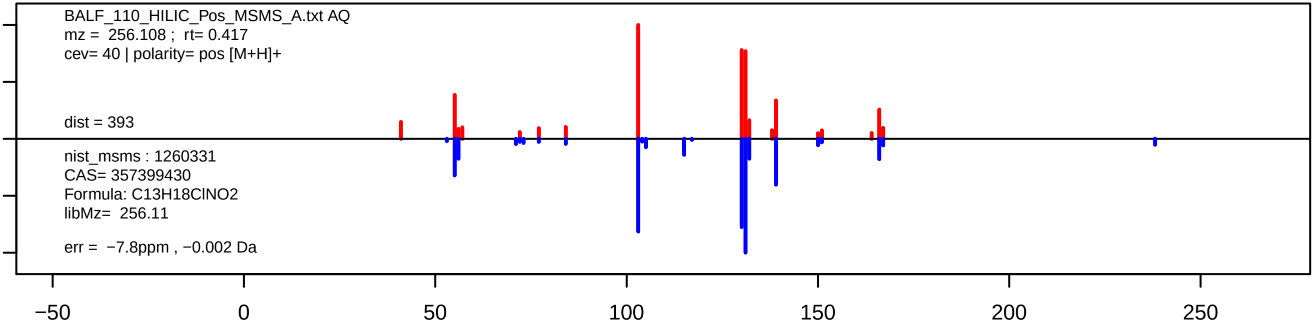

420 . Hydroxyzine
Score=697 Dot=933 prob=98.8

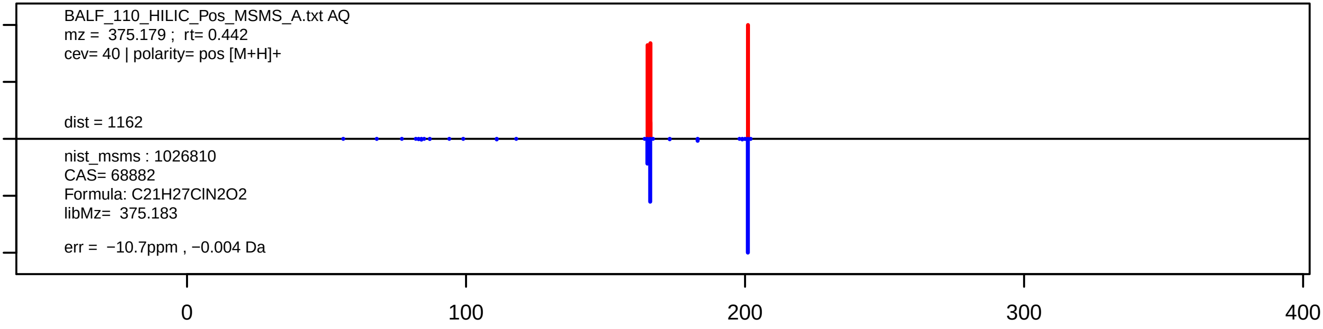

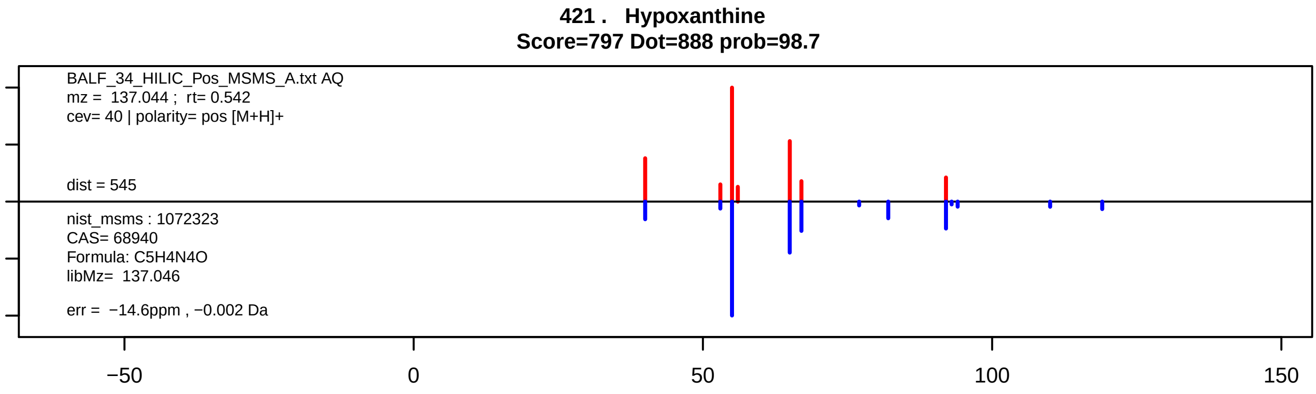
421 . Hypoxanthine
Score=797 Dot=888 prob=98.7
BALF_34_HILIC_Pos_MSMS_A.txt AQ
mz = 137.044 ; rt= 0.542
cev= 40 | polarity= pos [M+H]+
dist = 545
nist_msms : 1072323
CAS= 68940
Formula: C5H4N4O
libMz= 137.046
err = −14.6ppm , −0.002 Da
−50
0
50
100
150

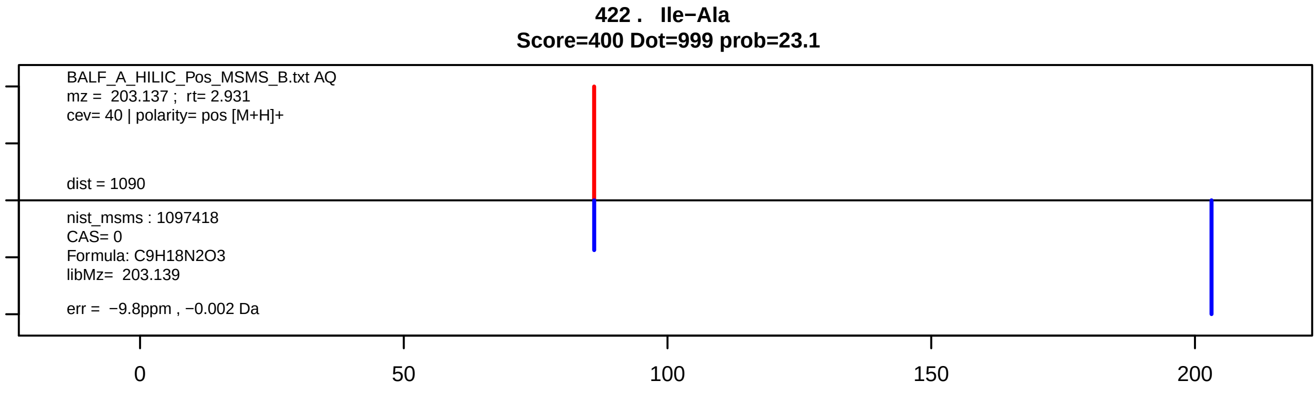
422 . Ile−Ala
Score=400 Dot=999 prob=23.1
BALF_A_HILIC_Pos_MSMS_B.txt AQ
mz = 203.137 ; rt= 2.931
cev= 40 | polarity= pos [M+H]+
dist = 1090
nist_msms : 1097418
CAS= 0
Formula: C9H18N2O3
libMz= 203.139
err = −9.8ppm , −0.002 Da
0
50
100
150
200

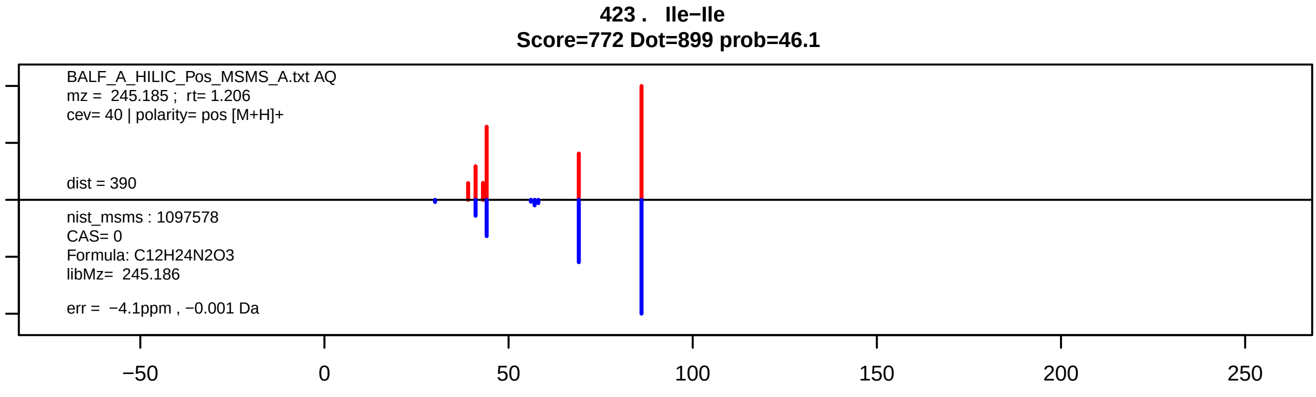
423 . Ile−Ile
Score=772 Dot=899 prob=46.1
BALF_A_HILIC_Pos_MSMS_A.txt AQ
mz = 245.185 ; rt= 1.206
cev= 40 | polarity= pos [M+H]+
dist = 390
nist_msms : 1097578
CAS= 0
Formula: C12H24N2O3
libMz= 245.186
err = −4.1ppm , −0.001 Da
−50
0
50
100
150
200
250

424 . Ile−Leu
Score=596 Dot=838 prob=79.5

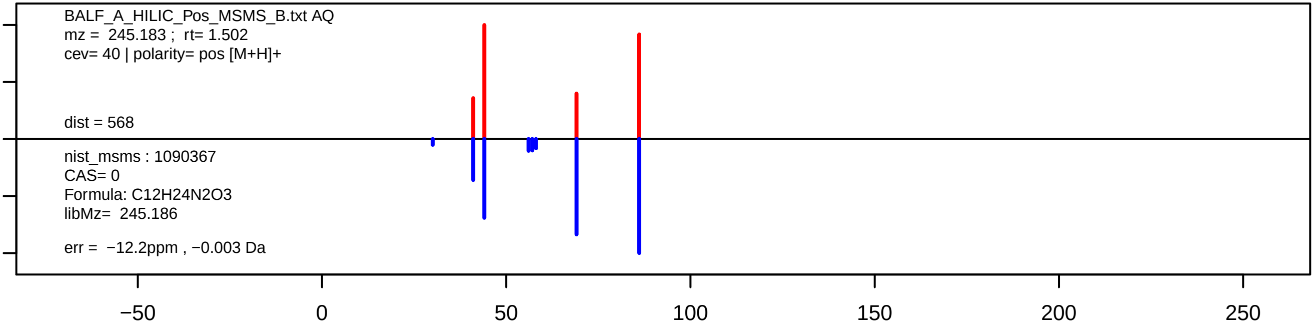

425 . Ile−Thr
Score=248 Dot=888 prob=48.4

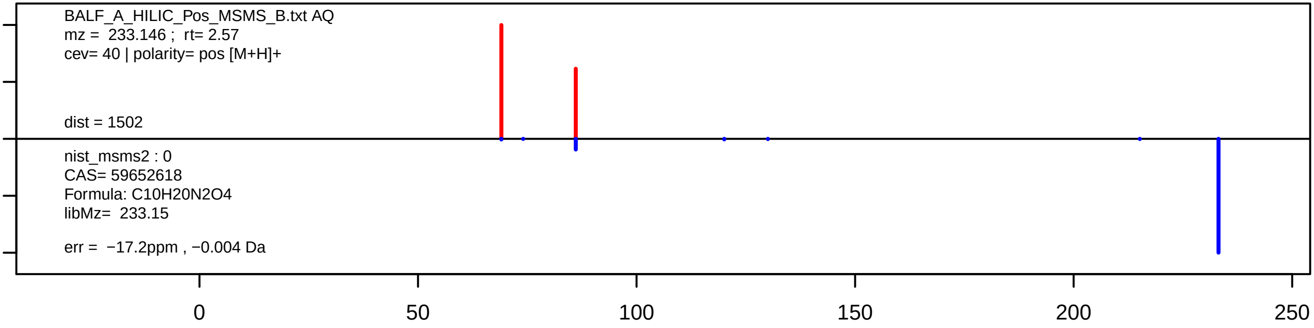

426 . Ile−Val
Score=392 Dot=843 prob=93.9

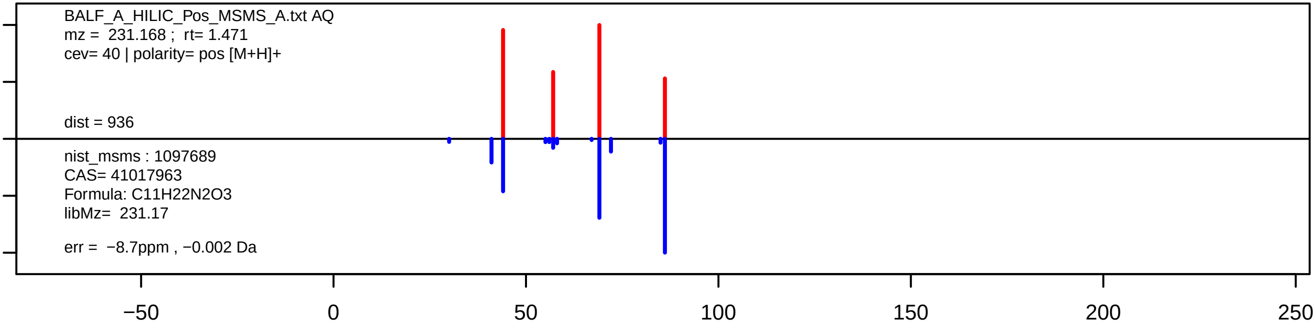

427 . Inosine
Score=397 Dot=878 prob=84.8

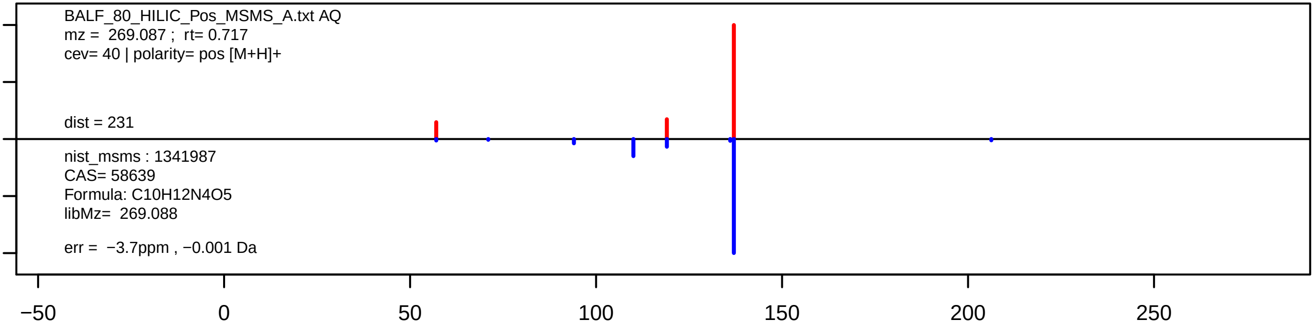

428 . Ipratropium cation
Score=663 Dot=836 prob=85.4

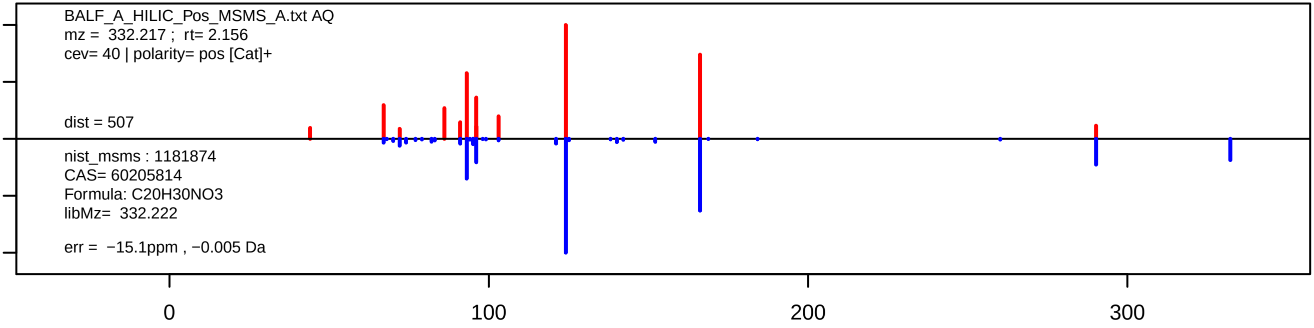

429 . L−Alanyl−L−norleucine
Score=197 Dot=909 prob=81

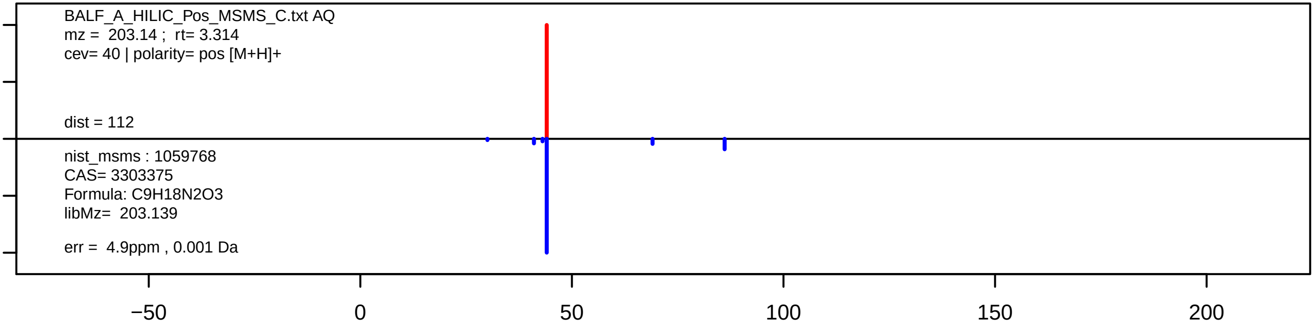

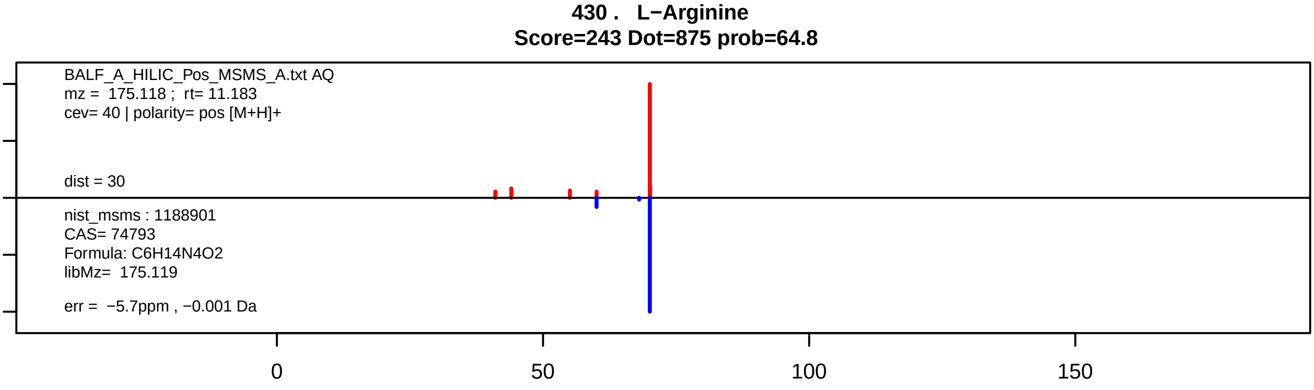
430 . L−Arginine
Score=243 Dot=875 prob=64.8
BALF_A_HILIC_Pos_MSMS_A.txt AQ
mz = 175.118 ; rt= 11.183
cev= 40 | polarity= pos [M+H]+
dist = 30
nist_msms : 1188901
CAS= 74793
Formula: C6H14N4O2
libMz= 175.119
err = −5.7ppm , −0.001 Da
0
50
100
150

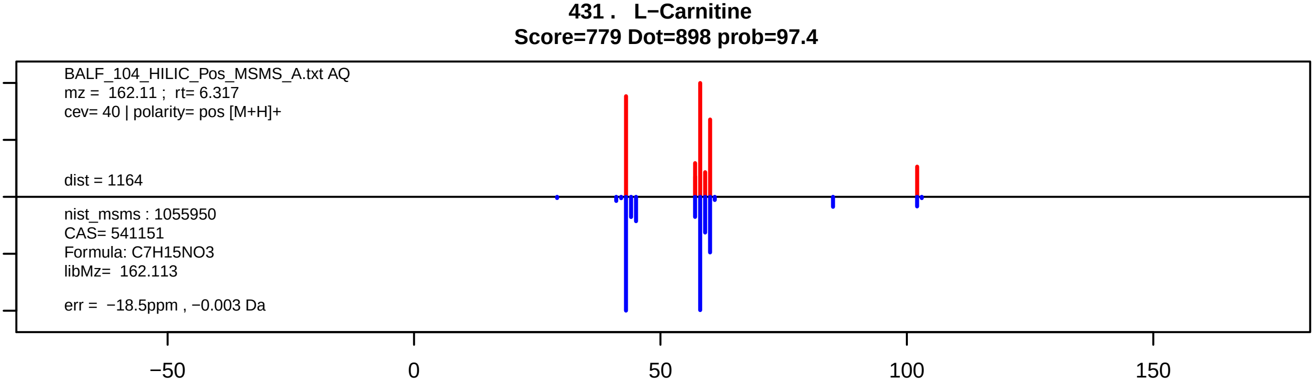
431 . L−Carnitine
Score=779 Dot=898 prob=97.4
BALF_104_HILIC_Pos_MSMS_A.txt AQ
mz = 162.11 ; rt= 6.317
cev= 40 | polarity= pos [M+H]+
dist = 1164
nist_msms : 1055950
CAS= 541151
Formula: C7H15NO3
libMz= 162.113
err = −18.5ppm , −0.003 Da
−50
0
50
100
150

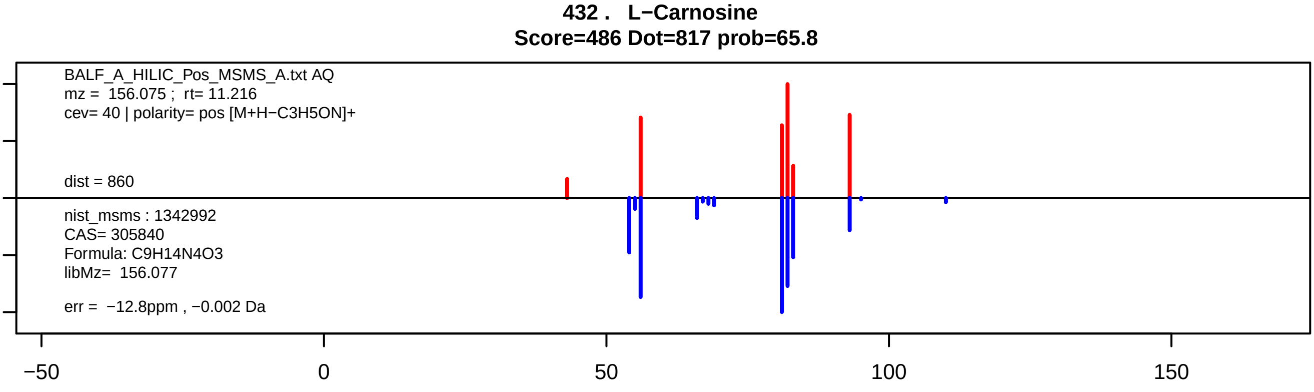
432 . L−Carnosine
Score=486 Dot=817 prob=65.8
BALF_A_HILIC_Pos_MSMS_A.txt AQ
mz = 156.075 ; rt= 11.216
cev= 40 | polarity= pos [M+H−C3H5ON]+
dist = 860
nist_msms : 1342992
CAS= 305840
Formula: C9H14N4O3
libMz= 156.077
err = −12.8ppm , −0.002 Da
−50
0
50
100
150

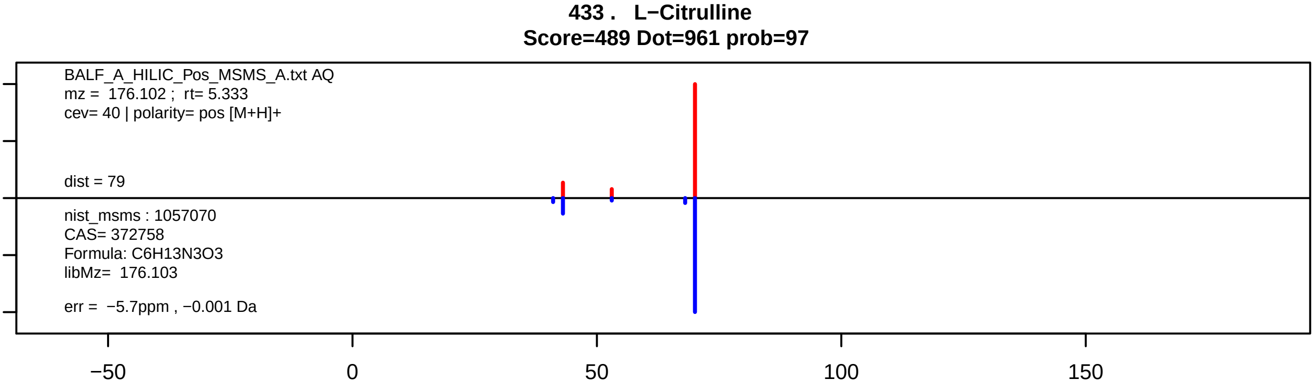
433 . L−Citrulline
Score=489 Dot=961 prob=97
BALF_A_HILIC_Pos_MSMS_A.txt AQ
mz = 176.102 ; rt= 5.333
cev= 40 | polarity= pos [M+H]+
dist = 79
nist_msms : 1057070
CAS= 372758
Formula: C6H13N3O3
libMz= 176.103
err = −5.7ppm , −0.001 Da
−50
0
50
100
150

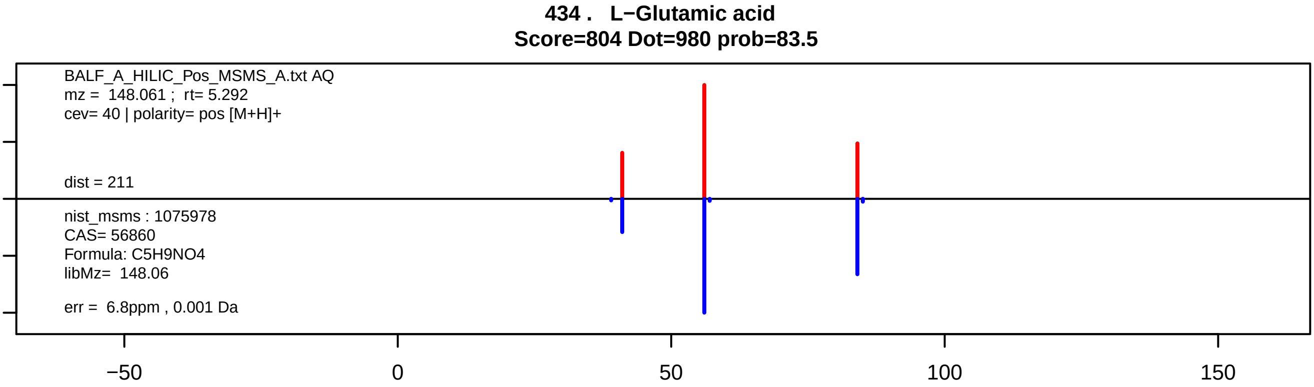
434 . L−Glutamic acid
Score=804 Dot=980 prob=83.5
BALF_A_HILIC_Pos_MSMS_A.txt AQ
mz = 148.061 ; rt= 5.292
cev= 40 | polarity= pos [M+H]+
dist = 211
nist_msms : 1075978
CAS= 56860
Formula: C5H9NO4
libMz= 148.06
err = 6.8ppm , 0.001 Da
−50
0
50
100
150

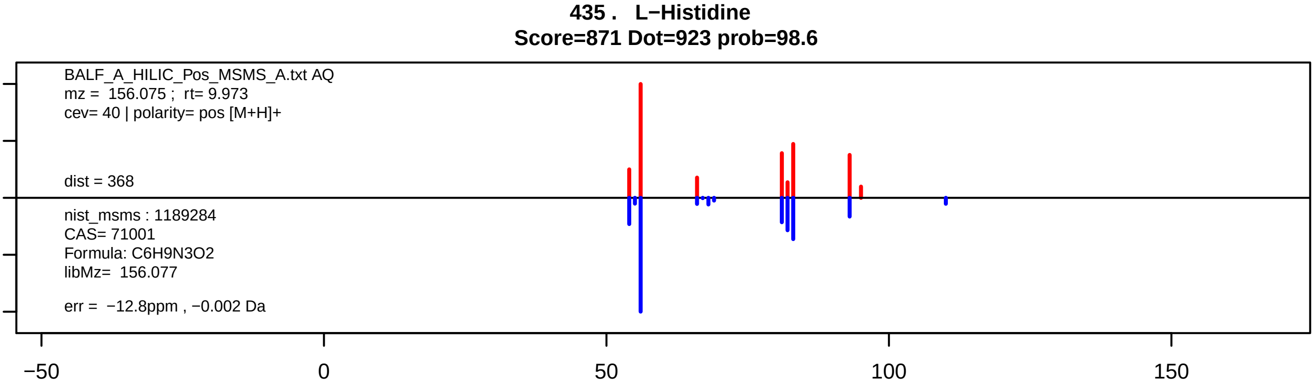
435 . L−Histidine
Score=871 Dot=923 prob=98.6
BALF_A_HILIC_Pos_MSMS_A.txt AQ
mz = 156.075 ; rt= 9.973
cev= 40 | polarity= pos [M+H]+
dist = 368
nist_msms : 1189284
CAS= 71001
Formula: C6H9N3O2
libMz= 156.077
err = −12.8ppm , −0.002 Da
−50
0
50
100
150

436 . L−Leucine
Score=795 Dot=936 prob=98.7

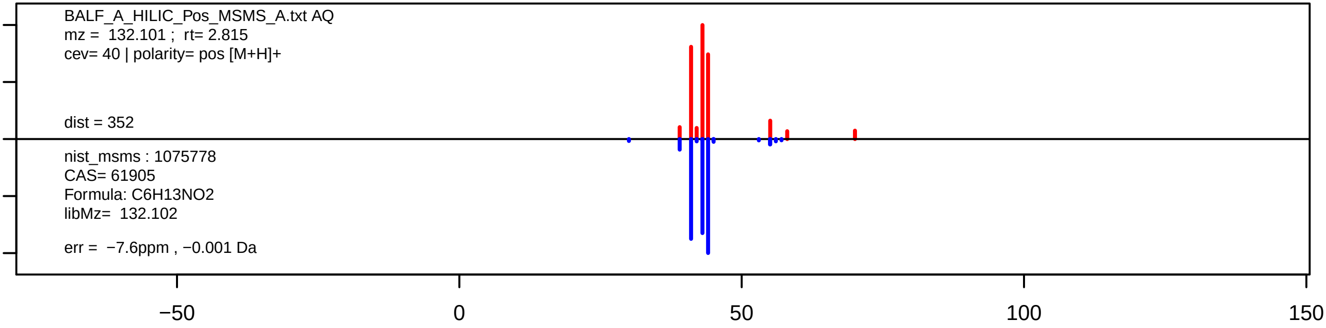

437 . L−Lysine
Score=643 Dot=864 prob=74.2

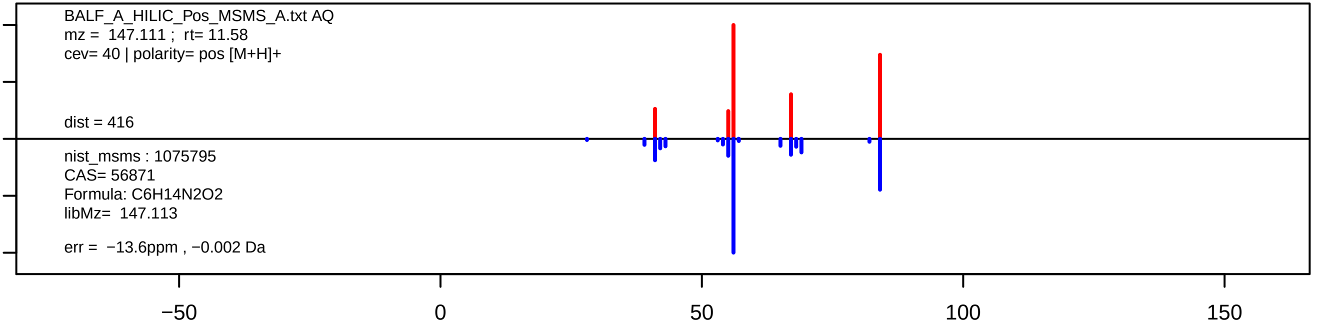

438 . L−Proline
Score=337 Dot=939 prob=7.1

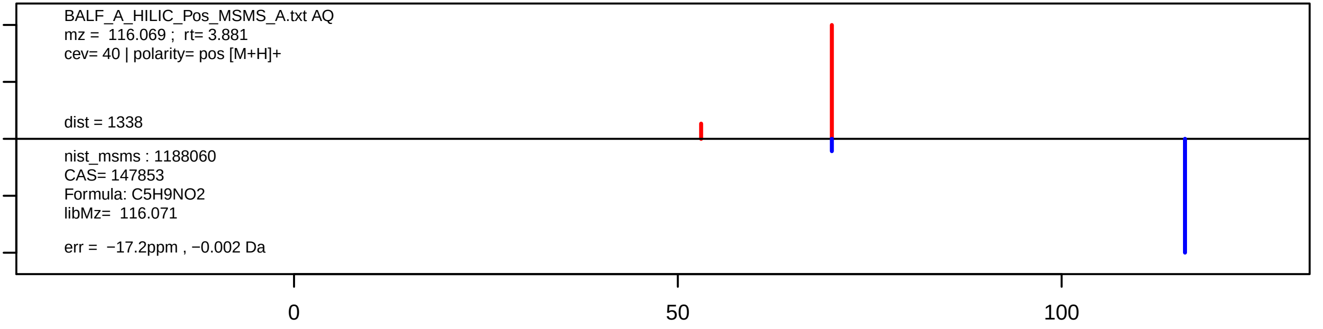

439 . L−Propionylcarnitine
Score=630 Dot=876 prob=98.5

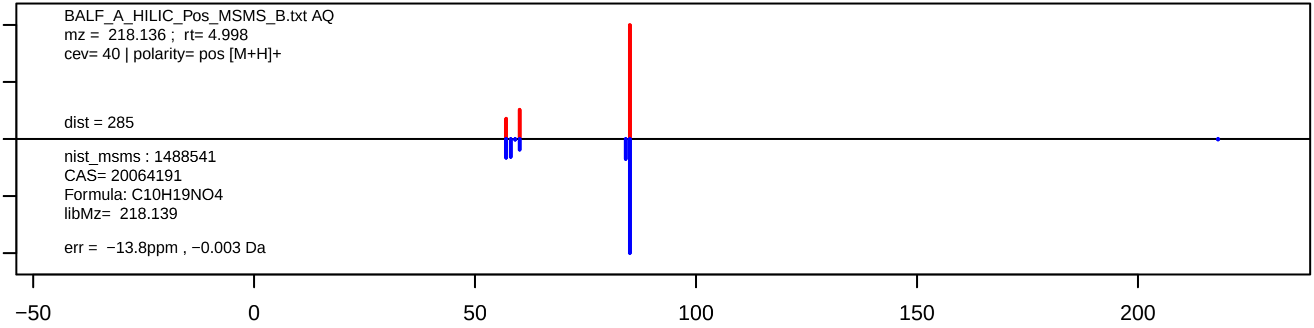

440 . L−Tyrosine
Score=690 Dot=871 prob=98.1

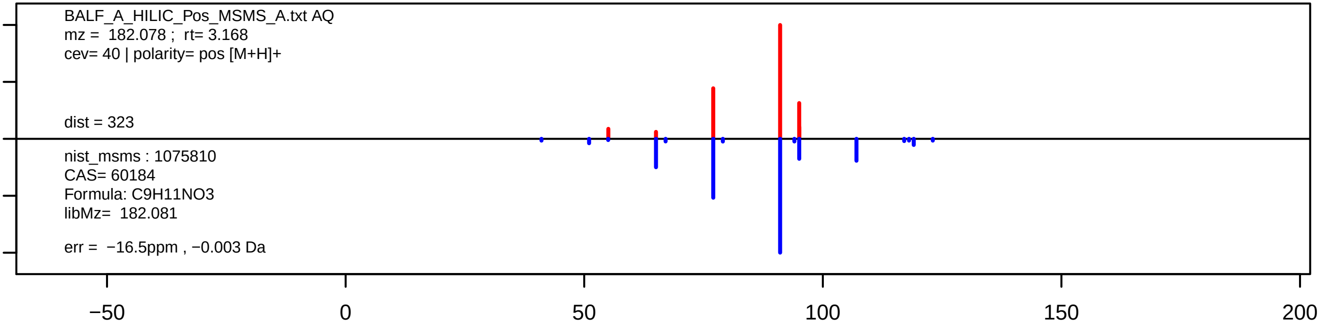

441 . Leu−Arg
Score=114 Dot=803 prob=18.1

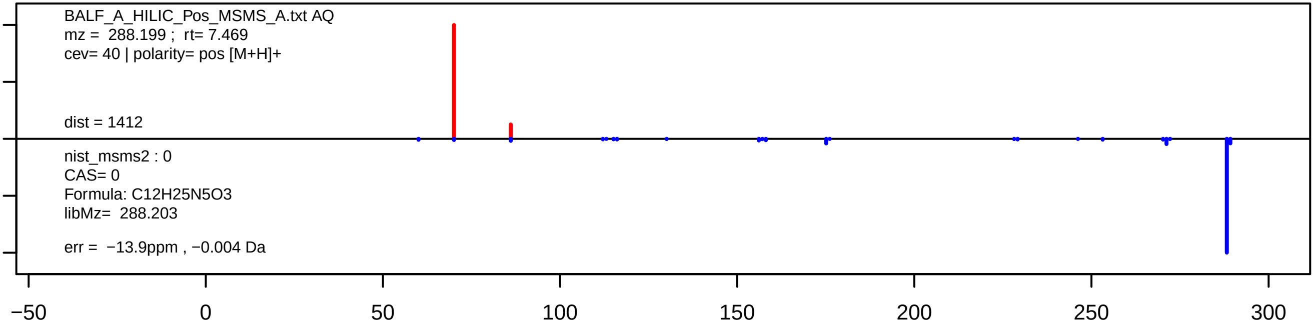

442 . Leu−Leu
Score=326 Dot=858 prob=87

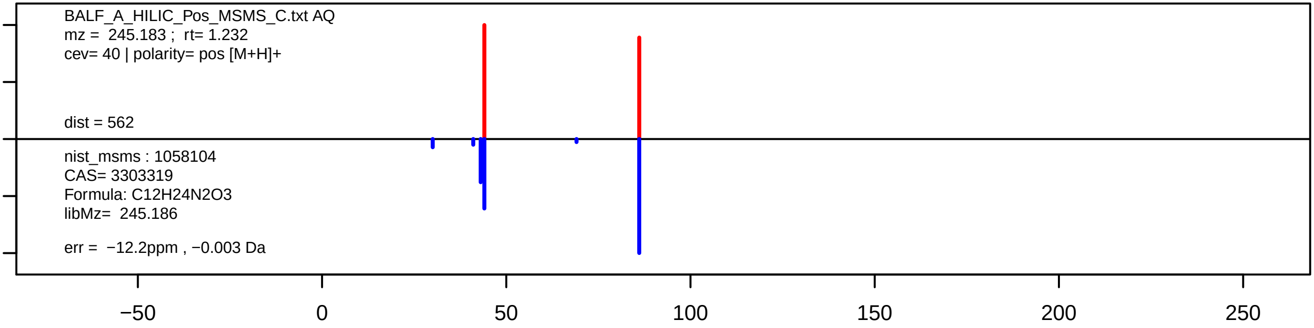

443 . Leu−Met
Score=691 Dot=869 prob=49.3

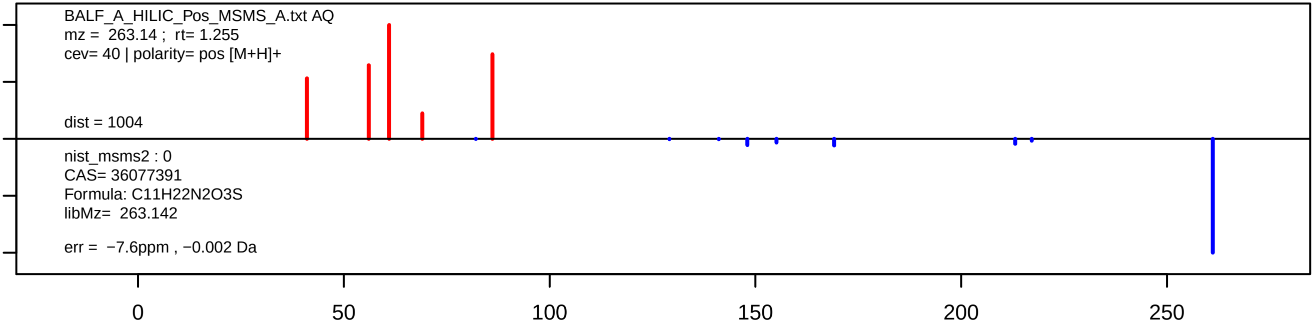

444 . Leu−Pro
Score=174 Dot=816 prob=23.8

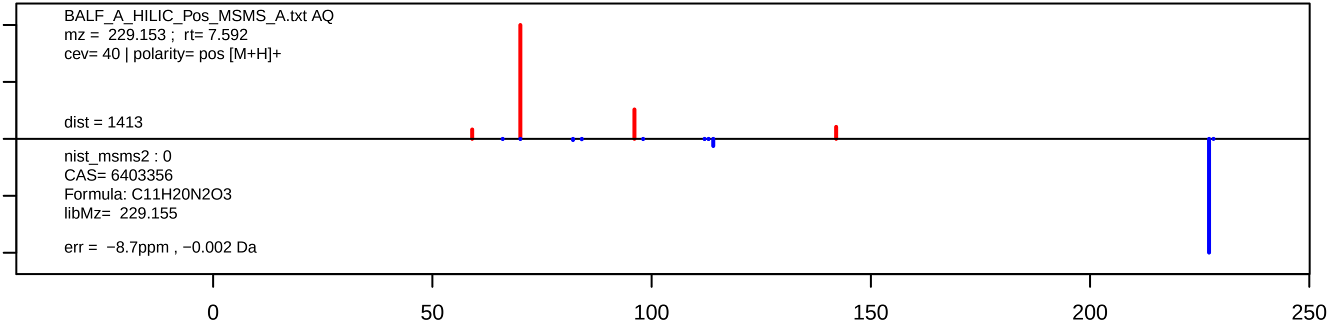

445 . Leu−Val
Score=636 Dot=898 prob=73.5

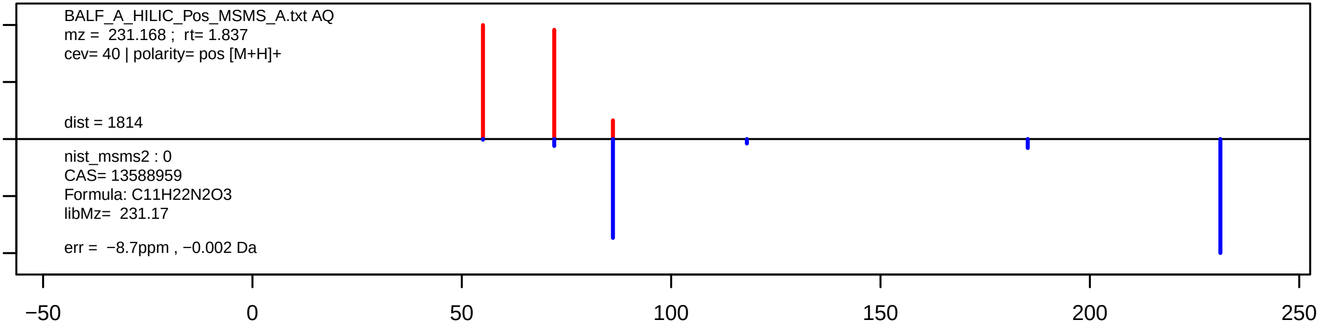

446 . Lidocaine
Score=614 Dot=963 prob=91.9

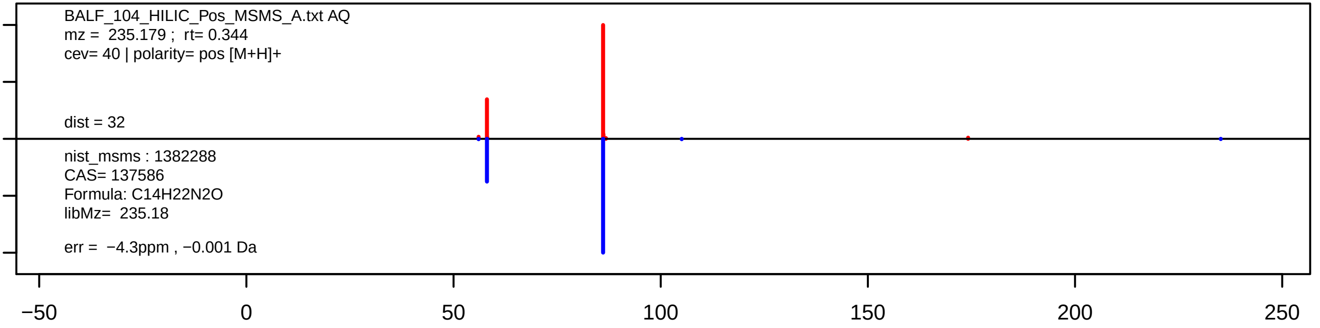

447 . Lys−Ala
Score=694 Dot=965 prob=44.4

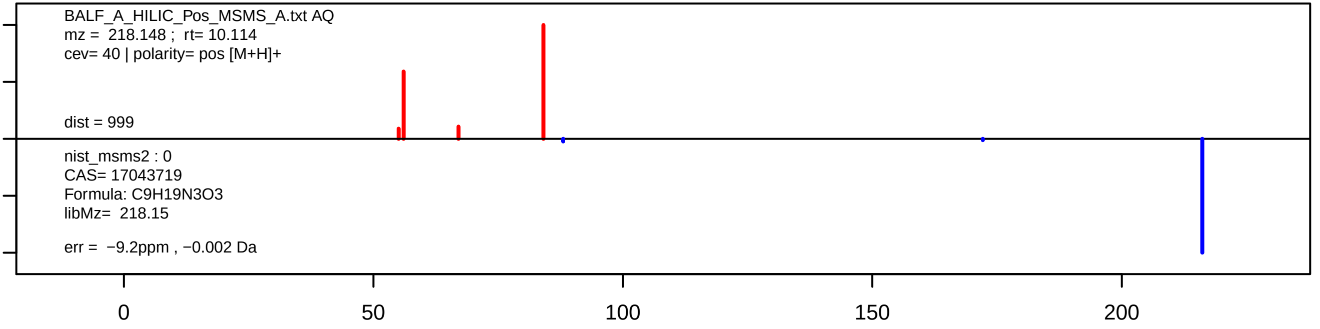

448 . Lys−Ile
Score=582 Dot=962 prob=31.7

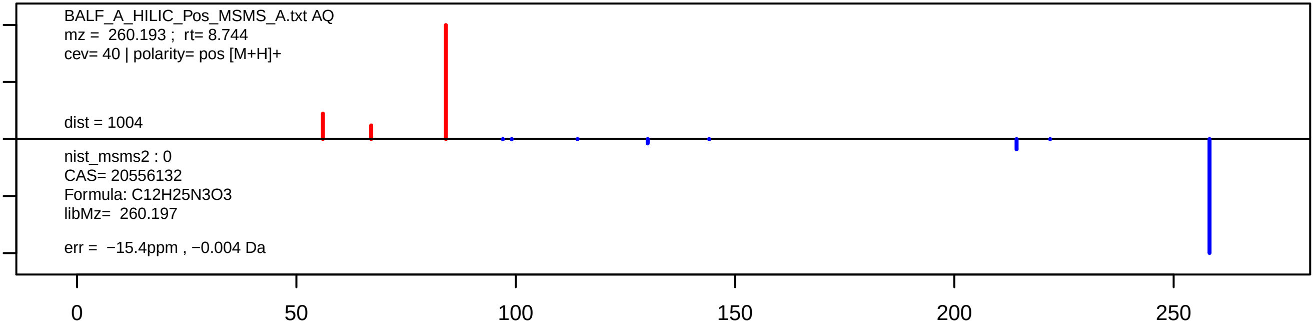

449 . Lys−Leu
Score=369 Dot=957 prob=19.5

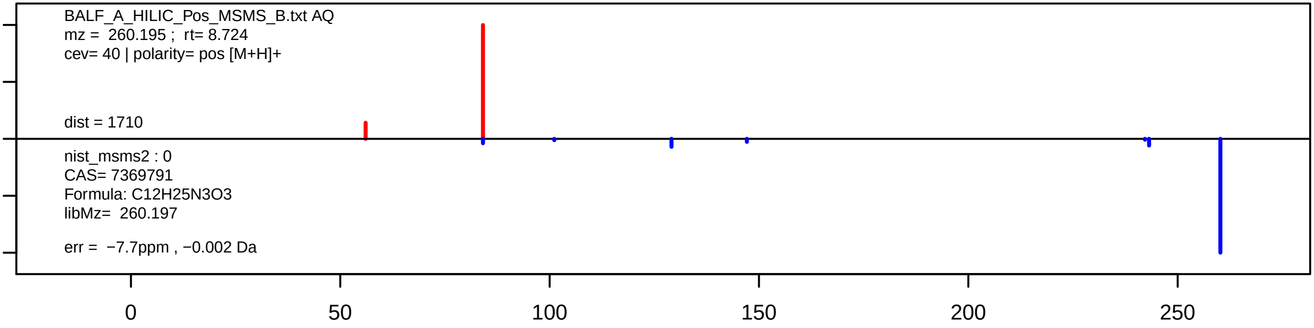

450 . Lys−Phe
Score=374 Dot=901 prob=46.5

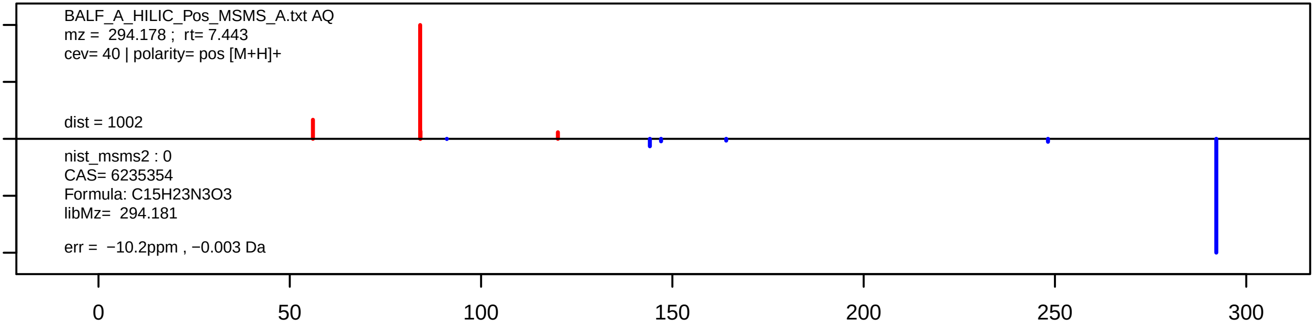

451 . Lys−Val
Score=562 Dot=883 prob=46.3

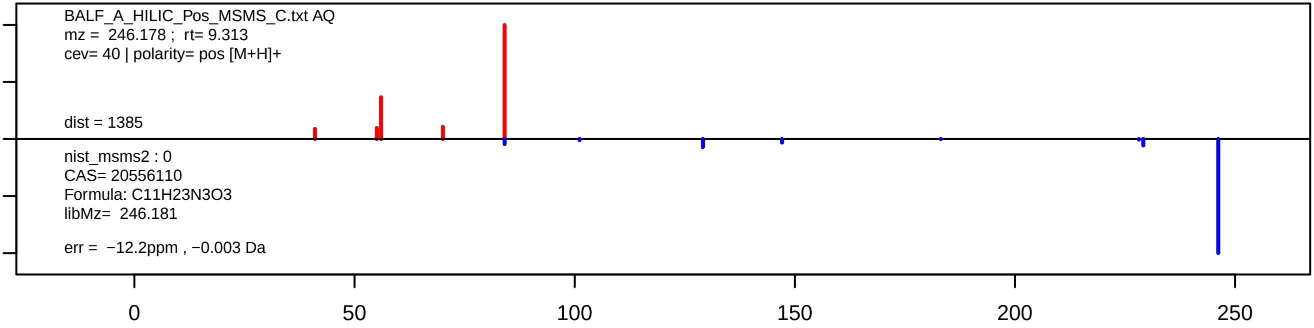

452 . Met−Val
Score=316 Dot=903 prob=49.1

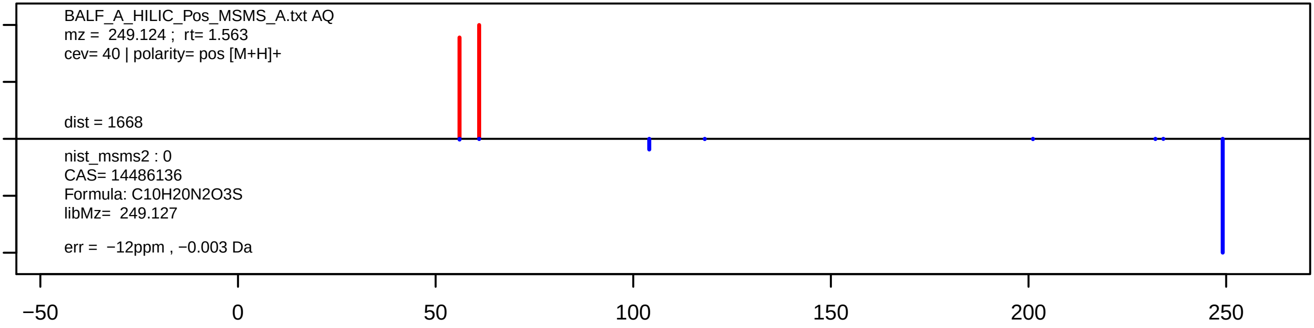

453 . Metformin
Score=576 Dot=873 prob=96.7

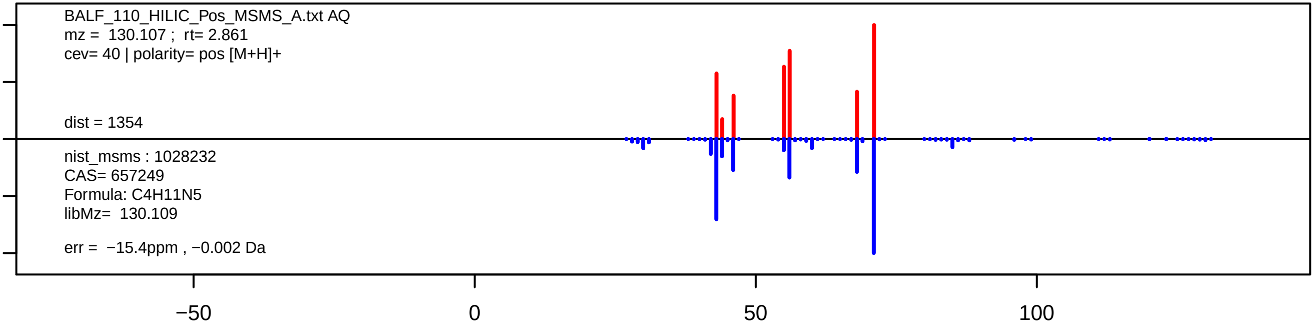

454 .   Methadone
Score=636 Dot=835 prob=98.8

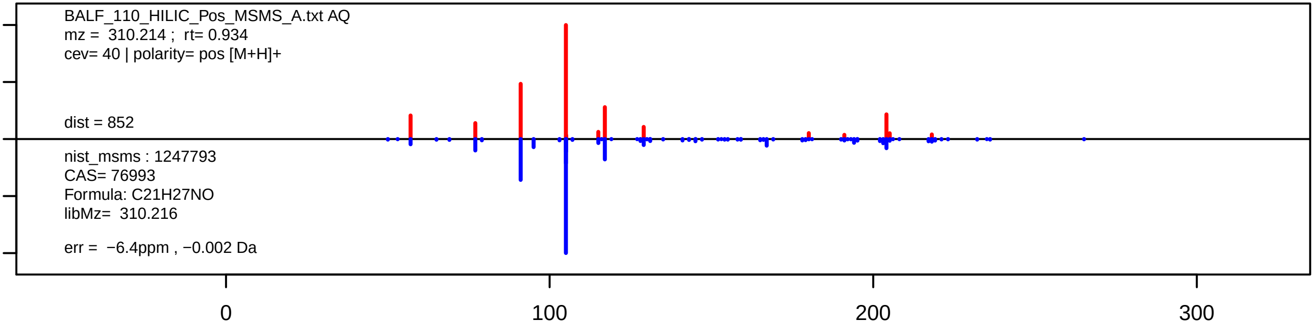

455 .   Methyl 4−hydroxybenzoate
Score=515 Dot=846 prob=97.7

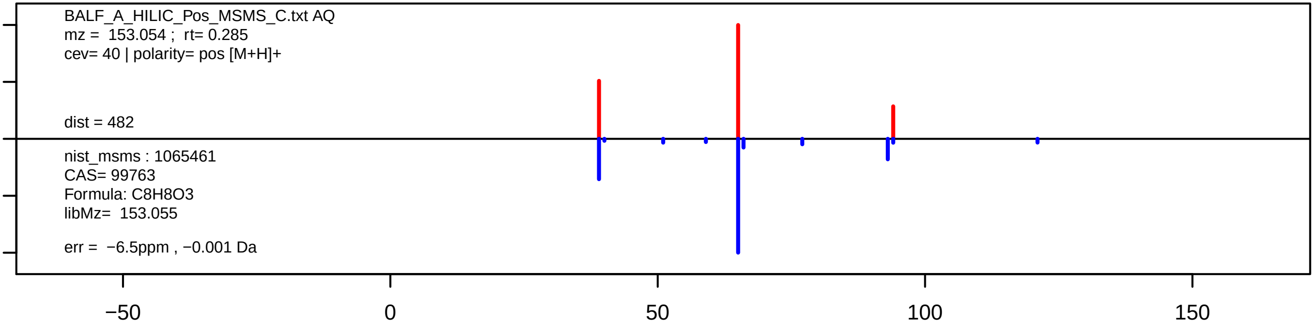

456 .   N−.alpha.−(tert−Butoxycarbonyl)−L−histidine
Score=756 Dot=918 prob=43.9

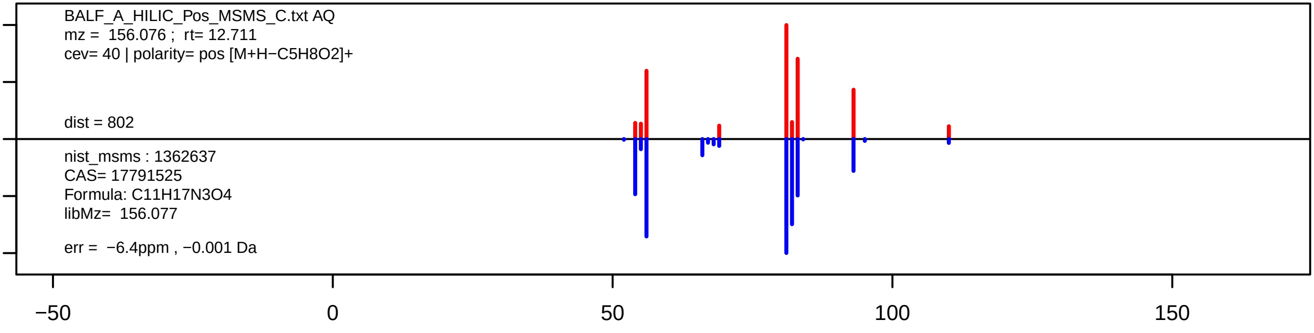

457 . N−Acetyl−L−carnosine
Score=655 Dot=858 prob=33.9

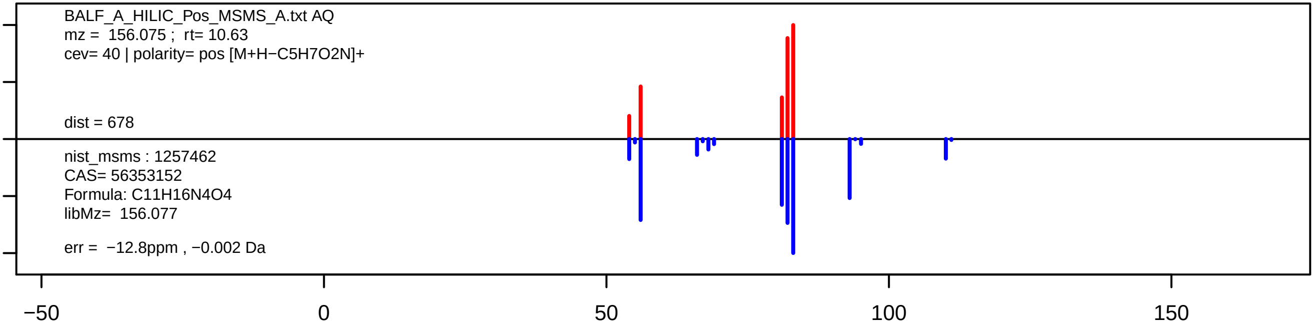

458 . N−Desmethyltramadol
Score=237 Dot=911 prob=97.3

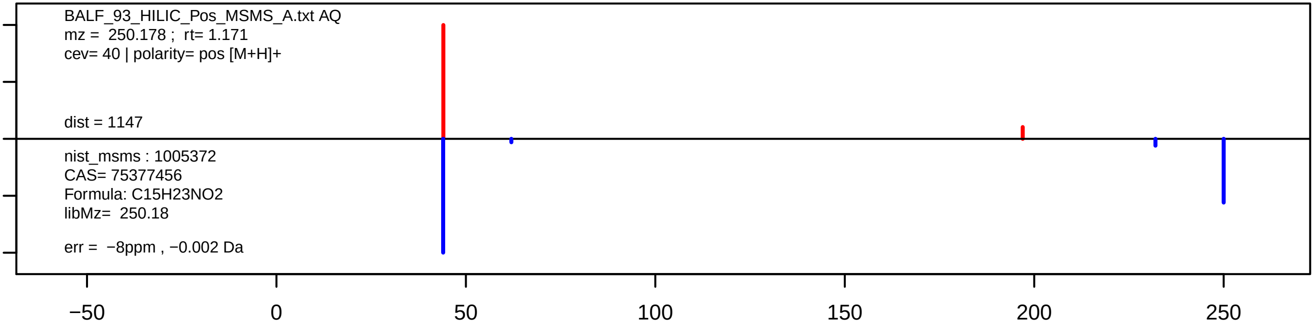

459 . N−Palmitoyl−D−sphingosine
Score=407 Dot=873 prob=99

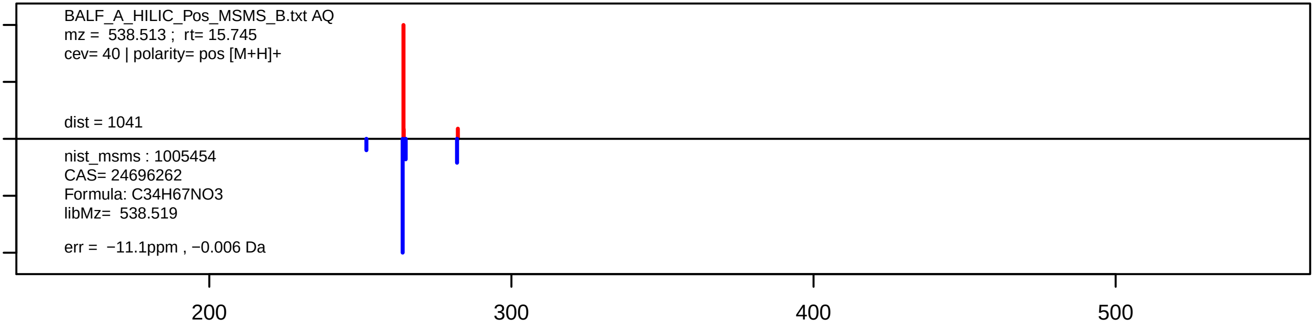

460 . N,N−Dimethylbenzylamine
Score=490 Dot=862 prob=49.8

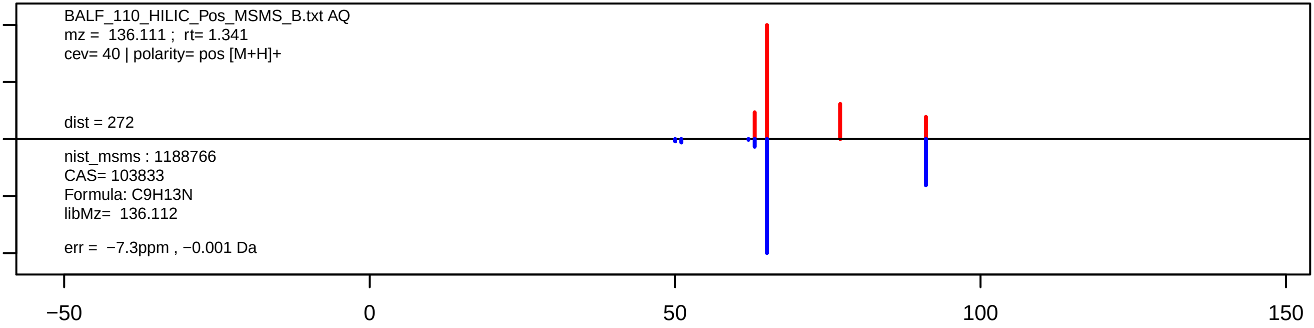

461 . Nialamide
Score=400 Dot=999 prob=97.5

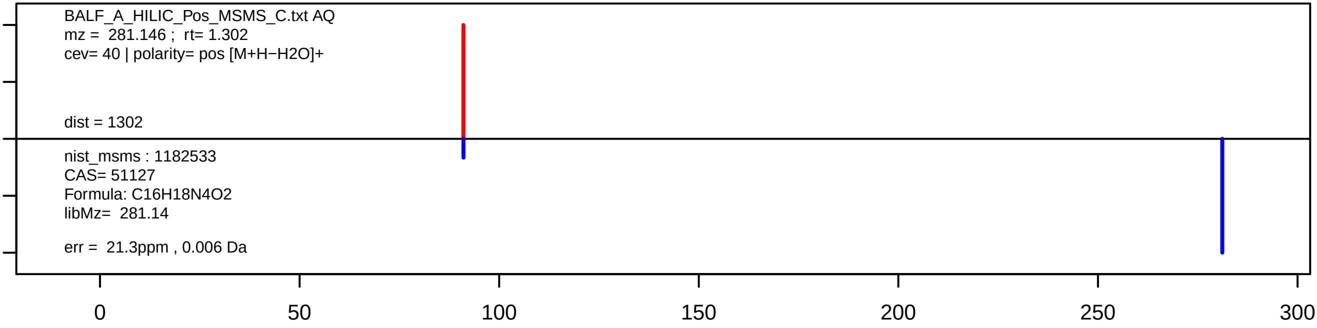

462 . Norquetiapine
Score=765 Dot=893 prob=98.7

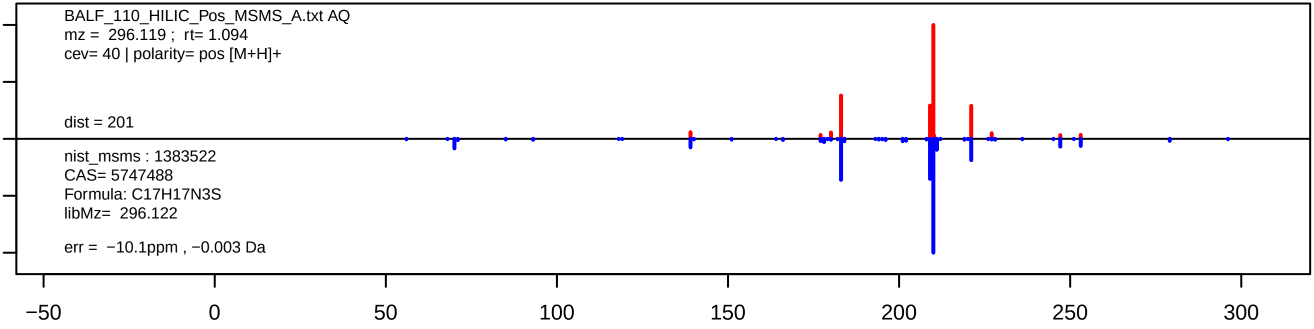

463 . O−Desmethyl−cis−tramadol
Score=387 Dot=998 prob=98.7

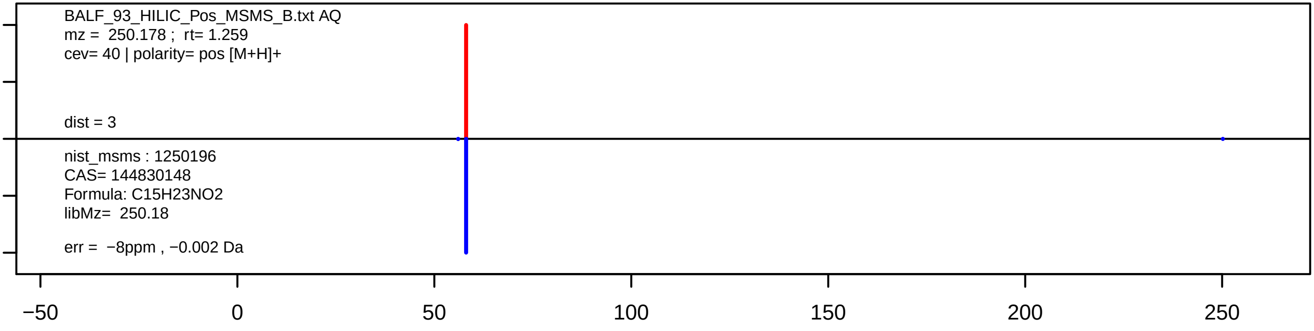

464 . O−Desmethylvenlafaxine
Score=358 Dot=878 prob=67.9

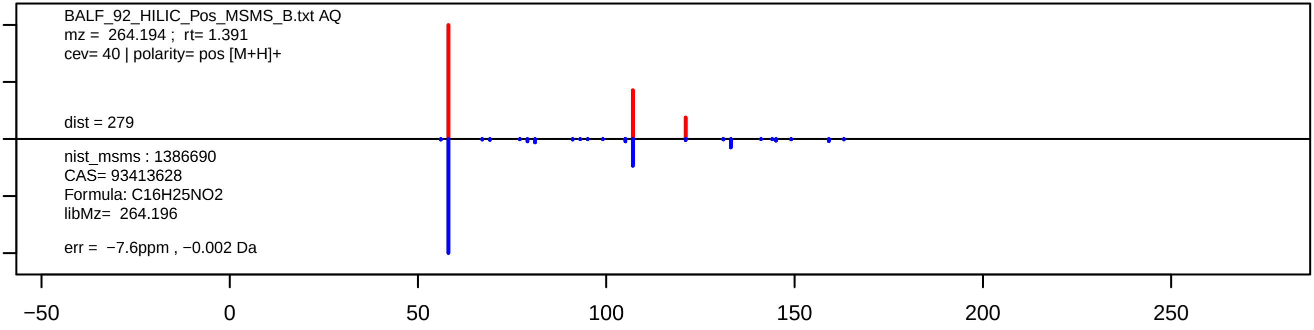

465 . Ondansetron
Score=780 Dot=876 prob=97.5

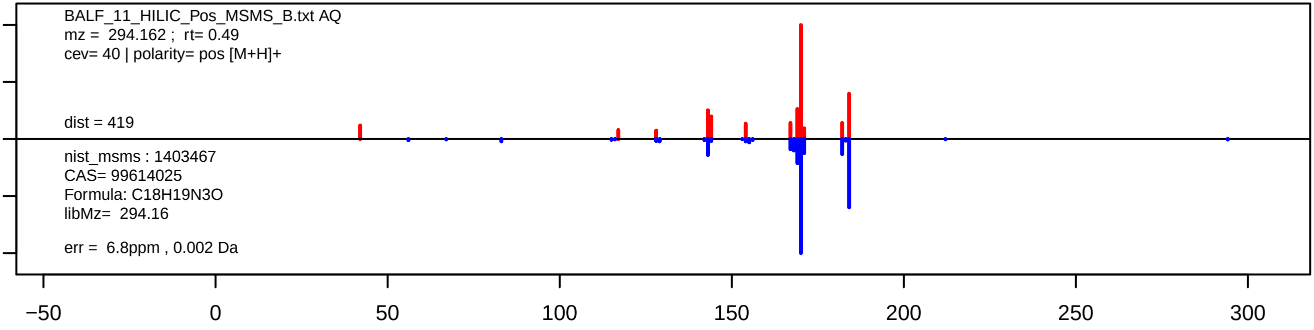

466 . Phe−Ala
Score=332 Dot=994 prob=93.8

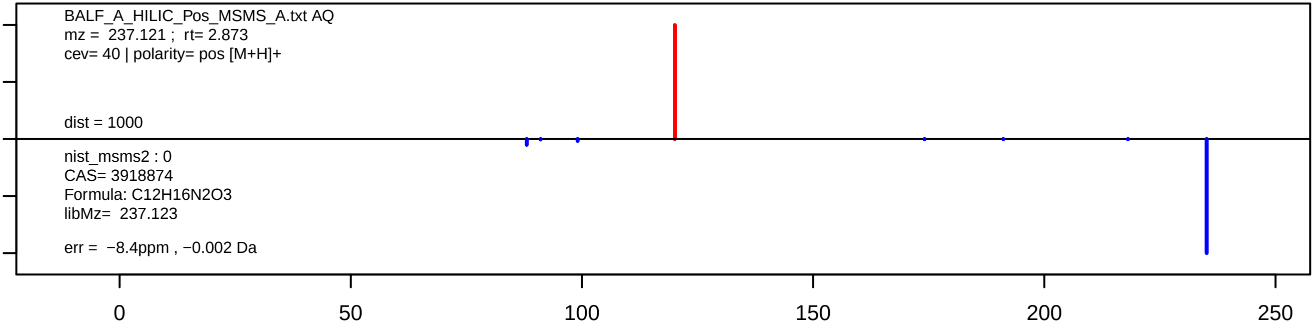

467 . Phe−Ile
Score=449 Dot=897 prob=45.6

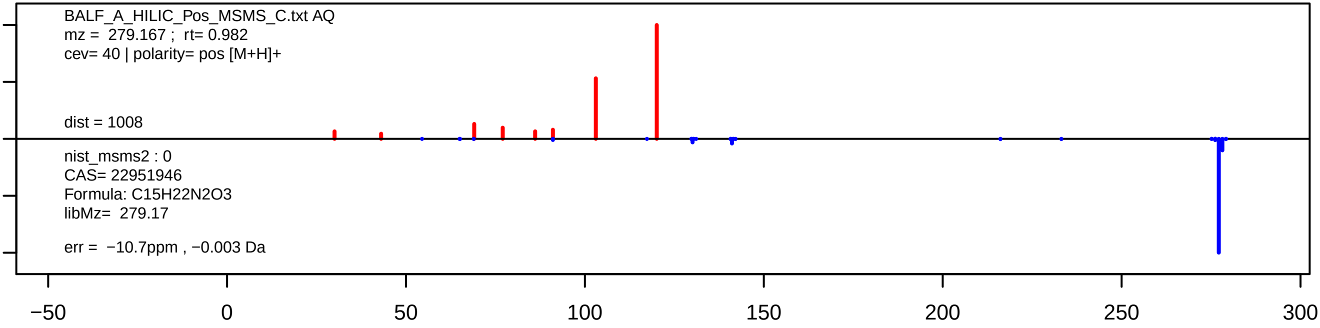

468 . Phe−Leu
Score=536 Dot=931 prob=36.5

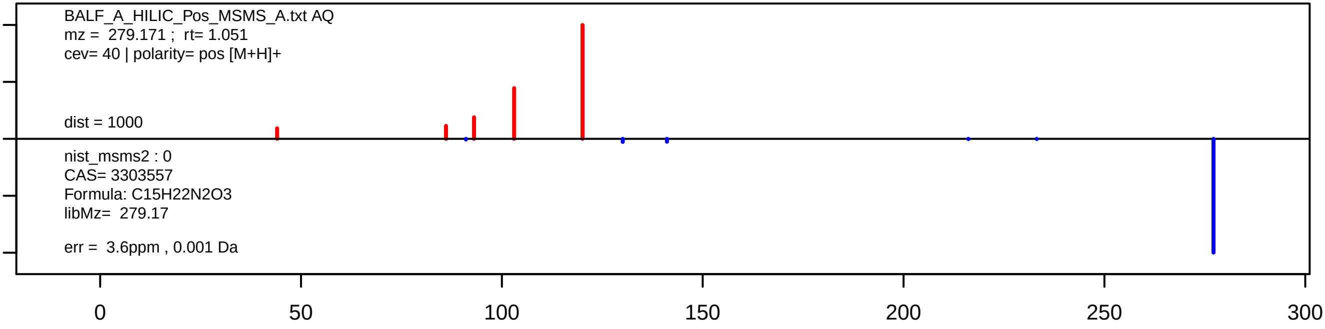

469 . Phe−Phe
Score=269 Dot=958 prob=95.6

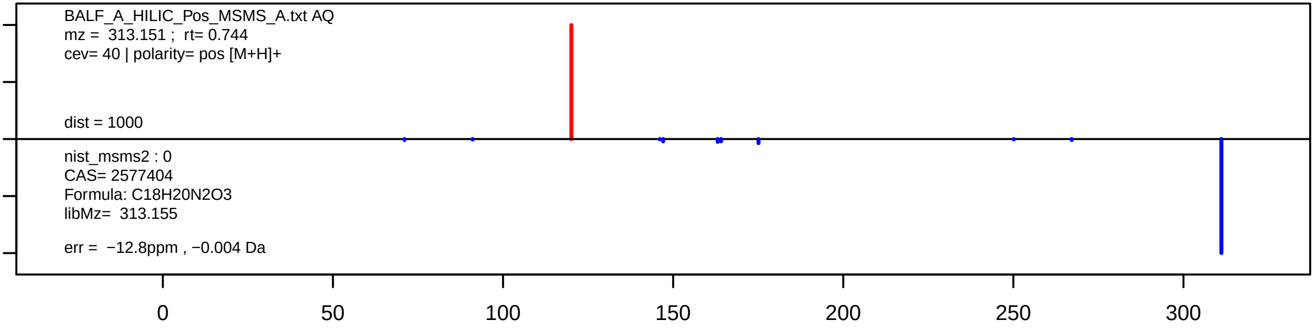

470 . Phe−Val
Score=714 Dot=939 prob=35.2

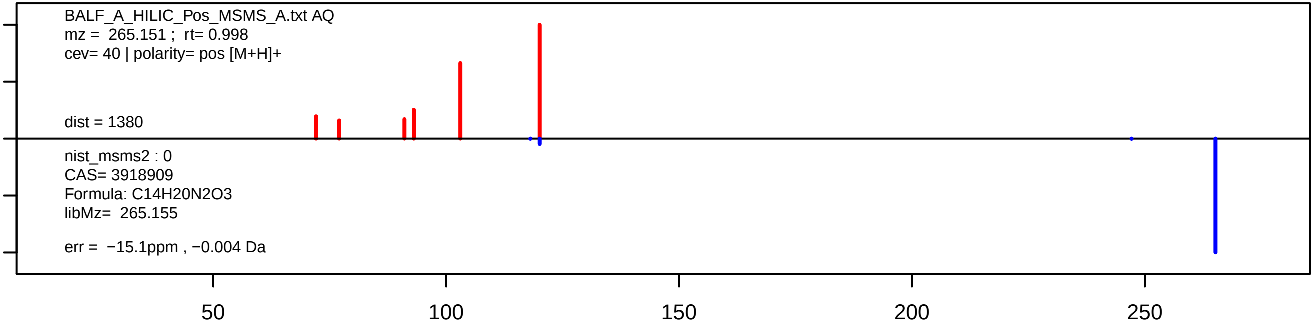

471 . Phenylacetyl−L−glutamine
Score=564 Dot=884 prob=99

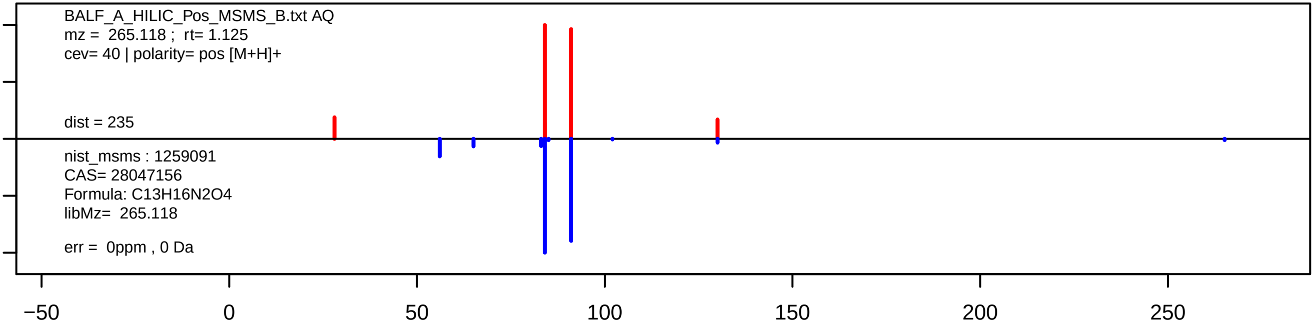

472 . Phenylglyoxylic acid
Score=322 Dot=977 prob=92.6

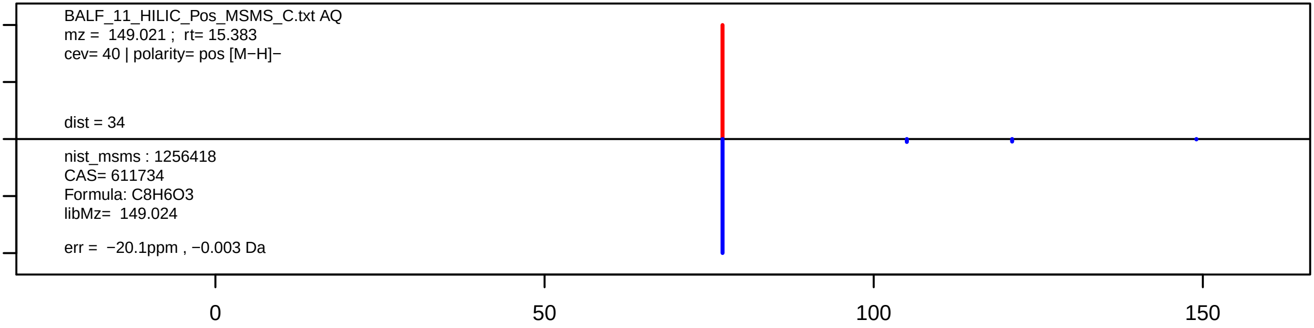

473 . Phosphoric acid
Score=327 Dot=991 prob=56.5

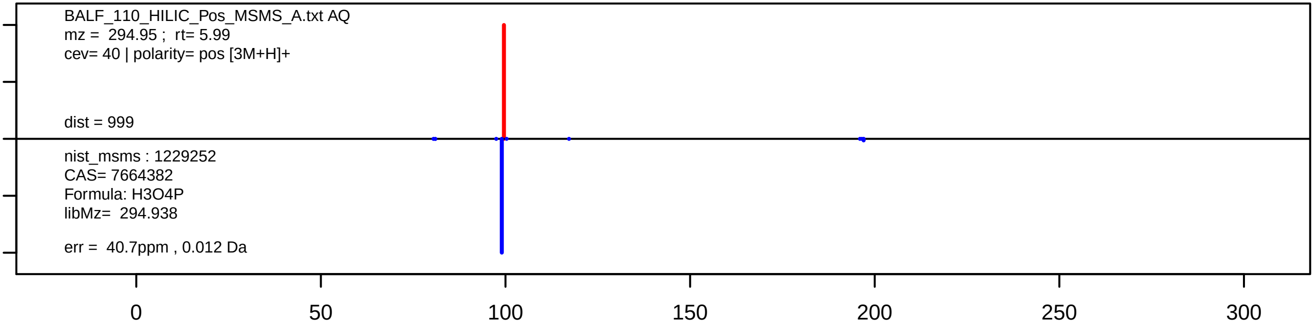

474 . Pro−Ala
Score=304 Dot=919 prob=41.6

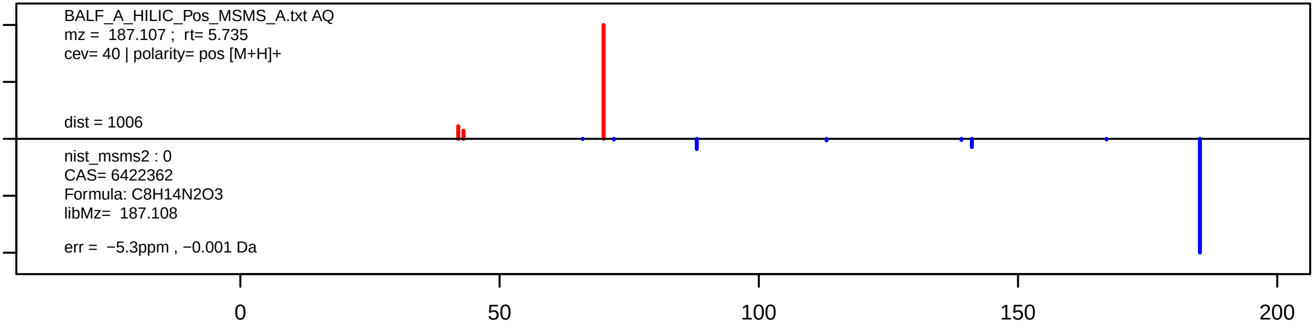

475 . Pro−Leu
Score=206 Dot=887 prob=29.4

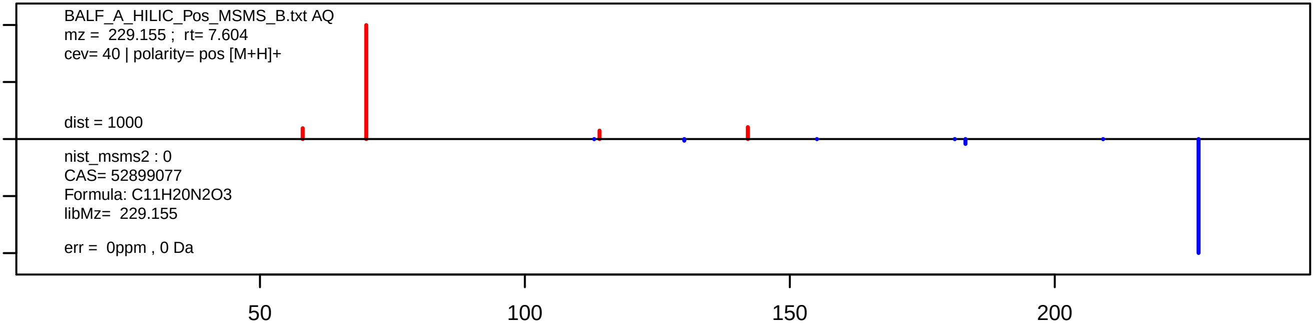

476 . Purine
Score=861 Dot=953 prob=98.3

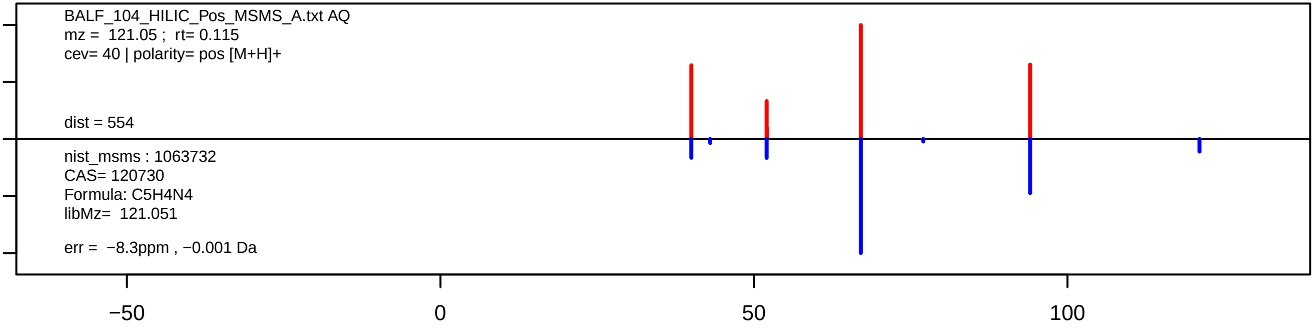

477 . rac erythro−Dihydrobupropion
Score=973 Dot=919 prob=80.3

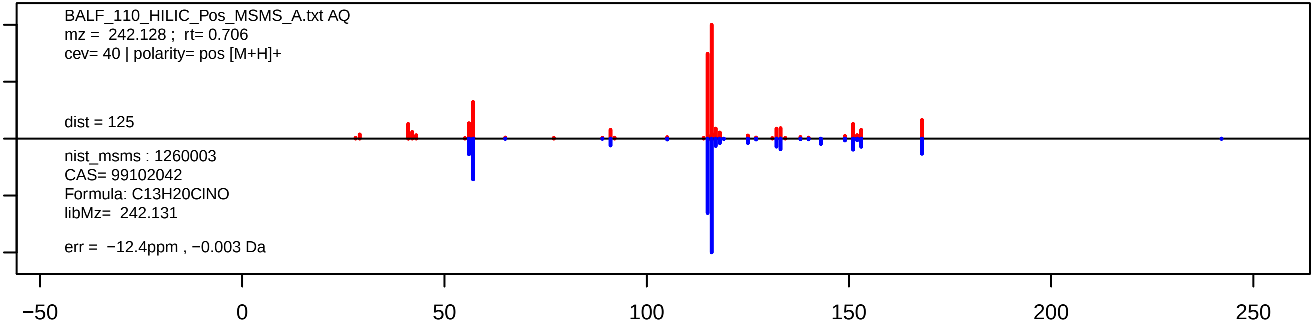

478 . Risperidone
Score=319 Dot=908 prob=95.6

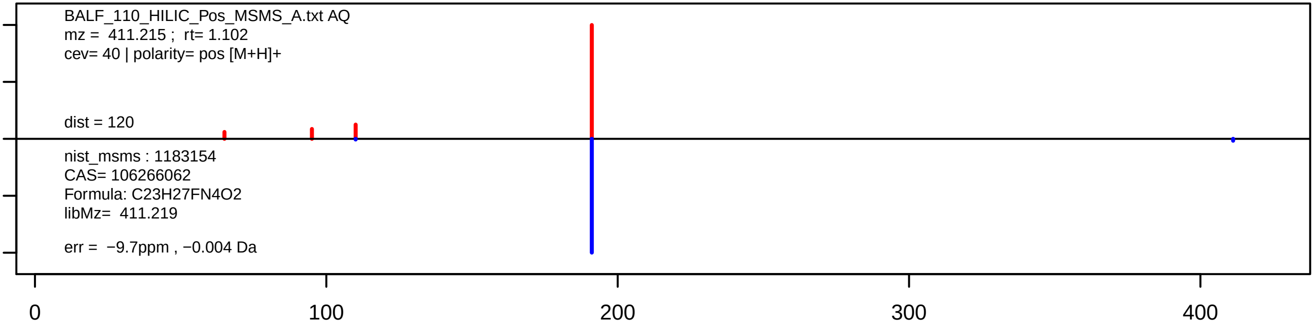

479 . Sarcosine
Score=336 Dot=891 prob=82.3

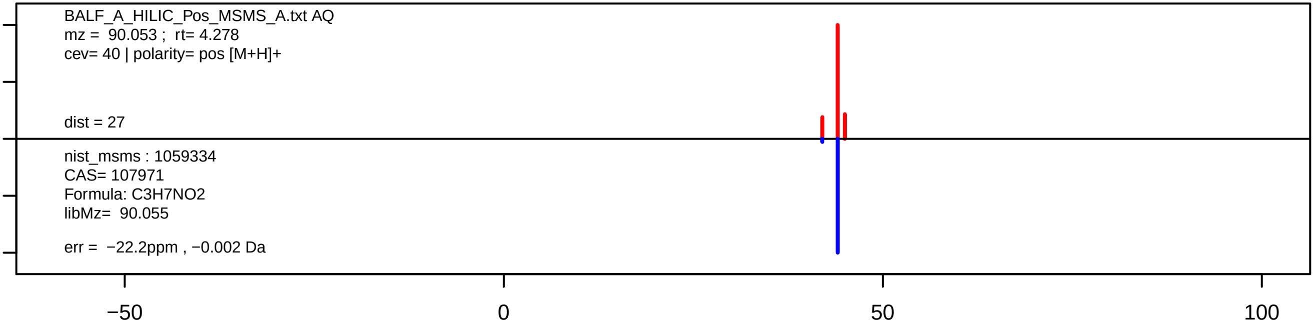

480 . Ser−Arg
Score=289 Dot=850 prob=47.5

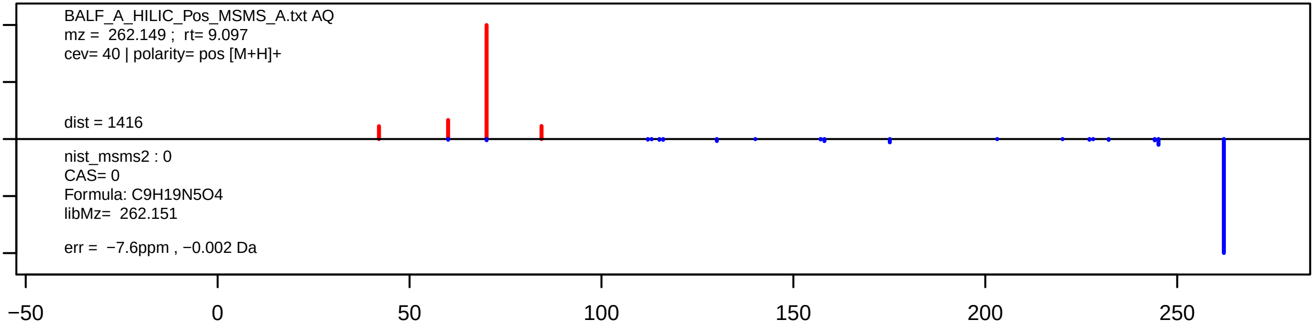

481 . Ser−Ile
Score=678 Dot=876 prob=43.7

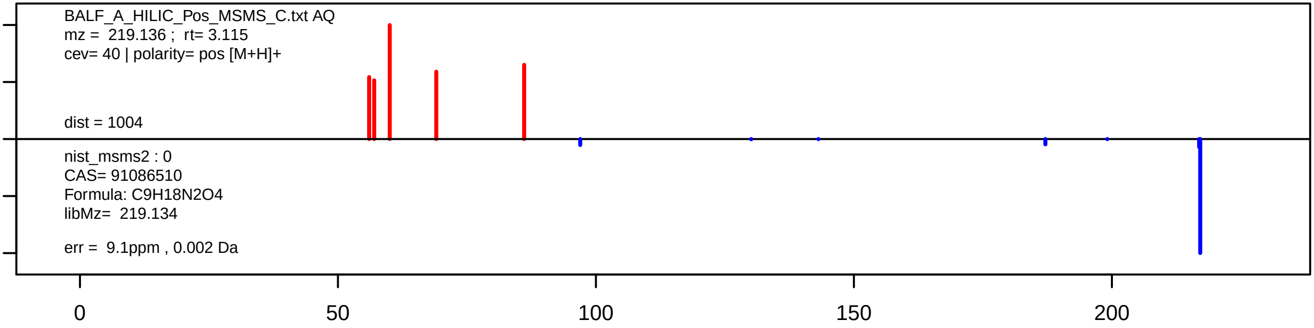

482 . Sialyl Lewis X
Score=473 Dot=964 prob=78.1

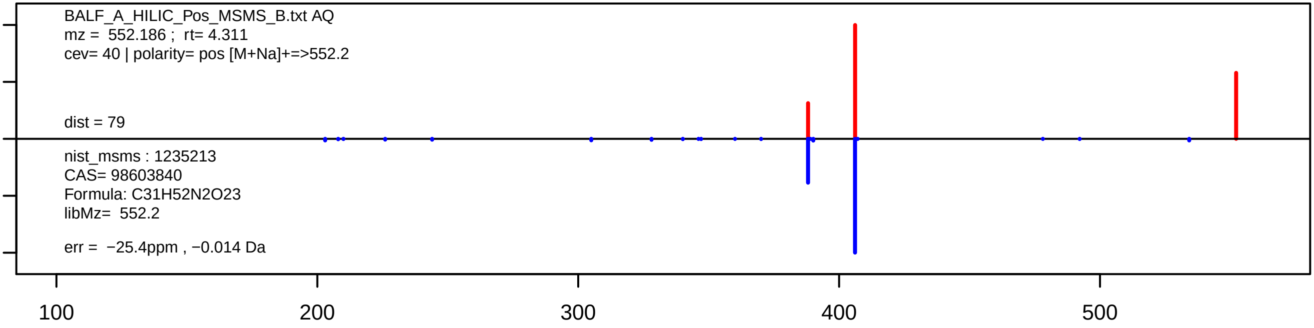

483 . Taurine
Score=179 Dot=911 prob=100

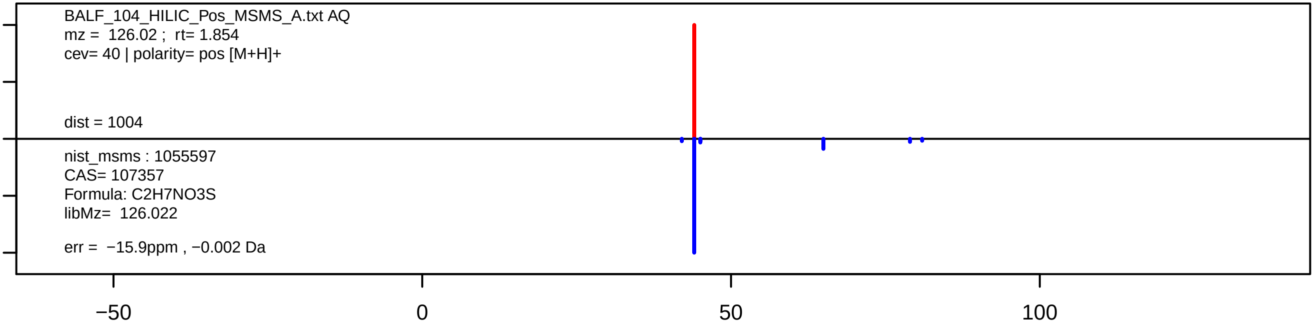

484 . Tetradonium cation
Score=384 Dot=835 prob=97.7

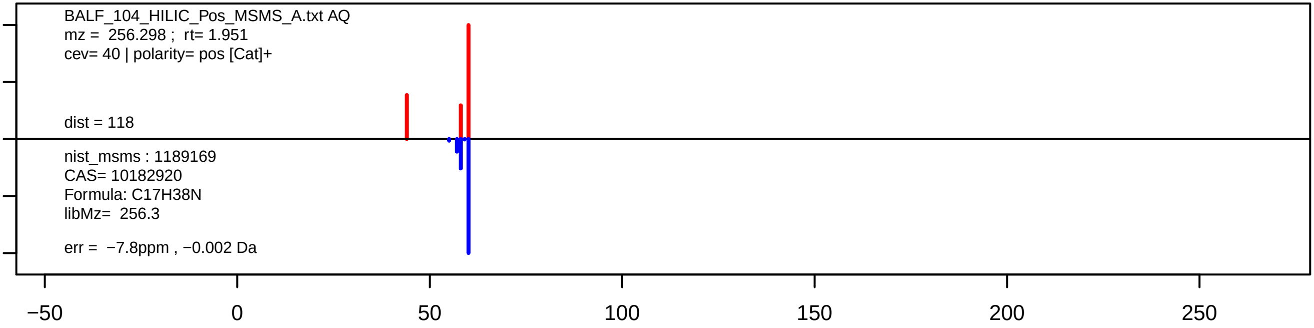

485 . Thr−Arg
Score=190 Dot=814 prob=42.1

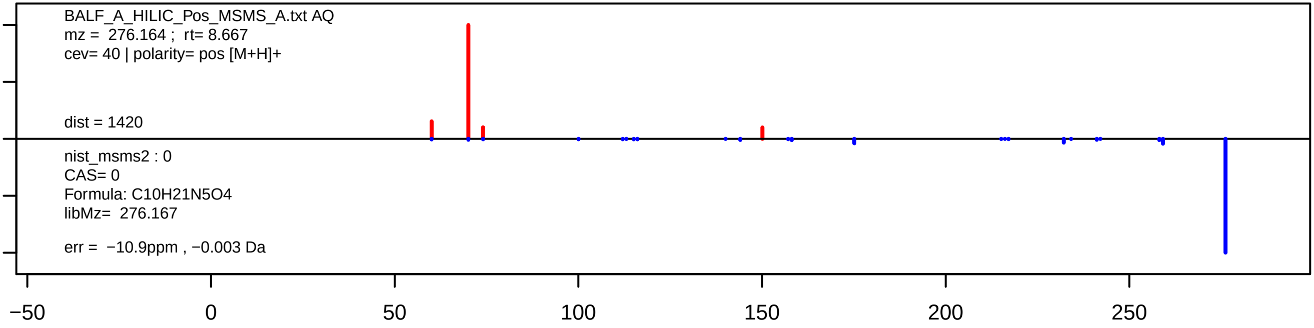

486 . Thr−Leu
Score=915 Dot=953 prob=95.2

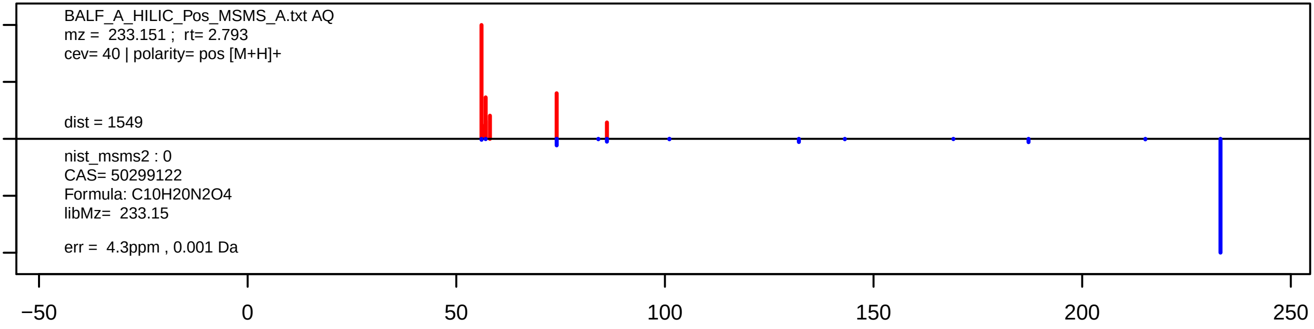

487 .   threo−Dihydrobupropion
Score=961 Dot=932 prob=53.2

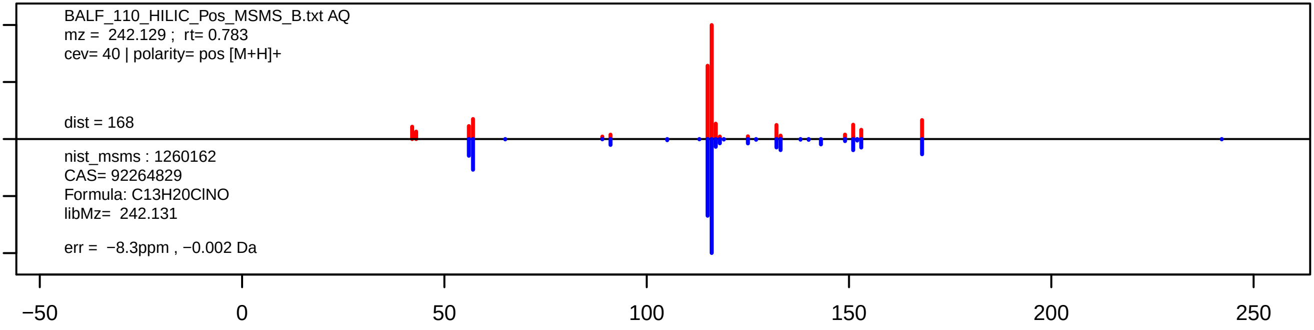

488 .   Tramadol
Score=305 Dot=970 prob=96.9

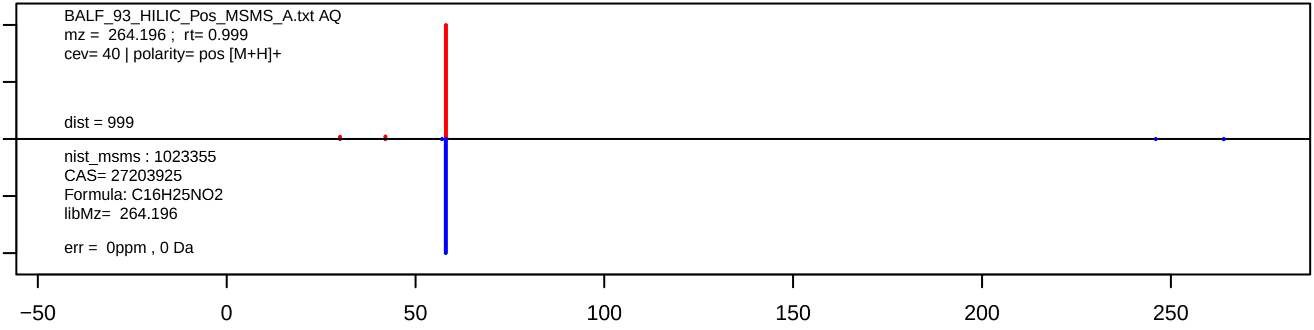

489 .   Tyr−Arg
Score=496 Dot=844 prob=49.7

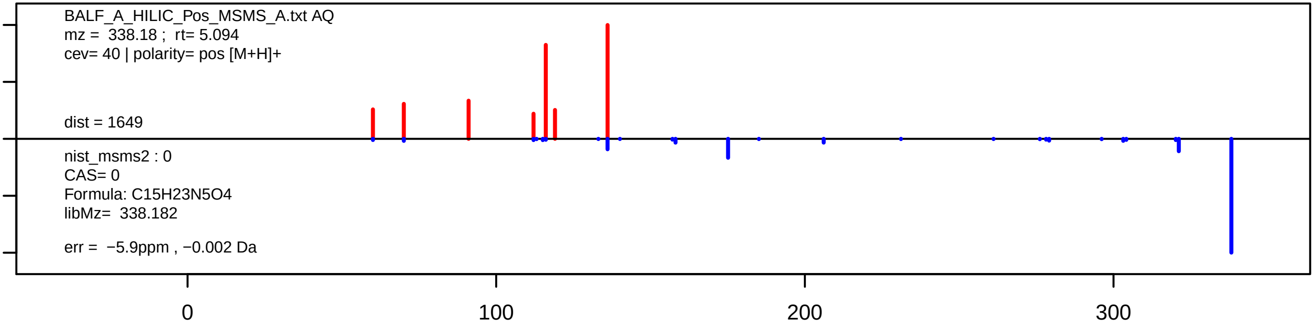

490 . Tyr−Leu
Score=372 Dot=823 prob=31.8

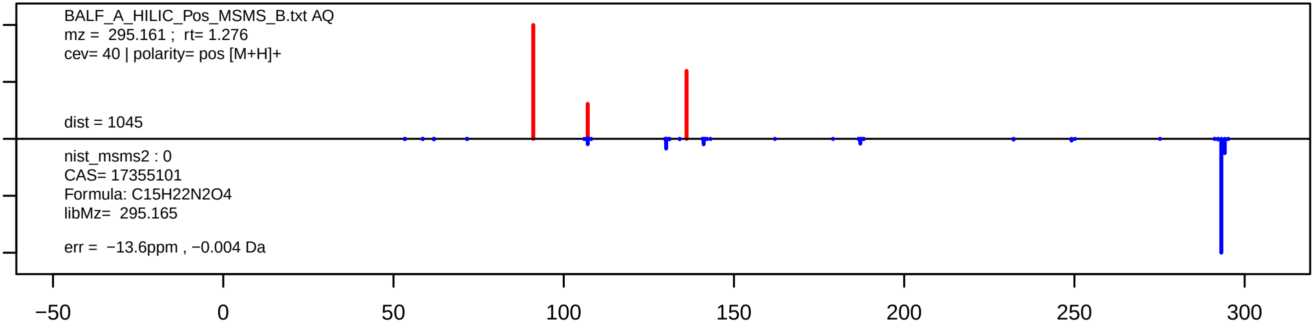

491 . Tyramine
Score=585 Dot=817 prob=84.3

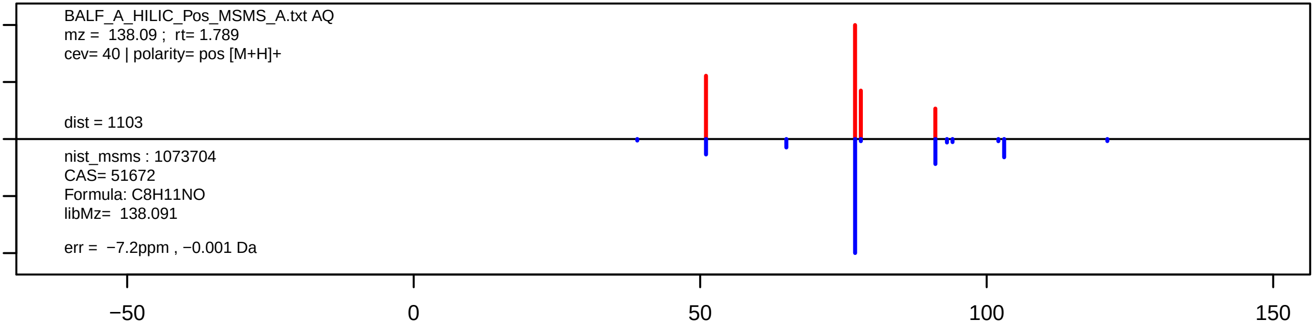

492 . Val−Ala
Score=422 Dot=895 prob=45.9

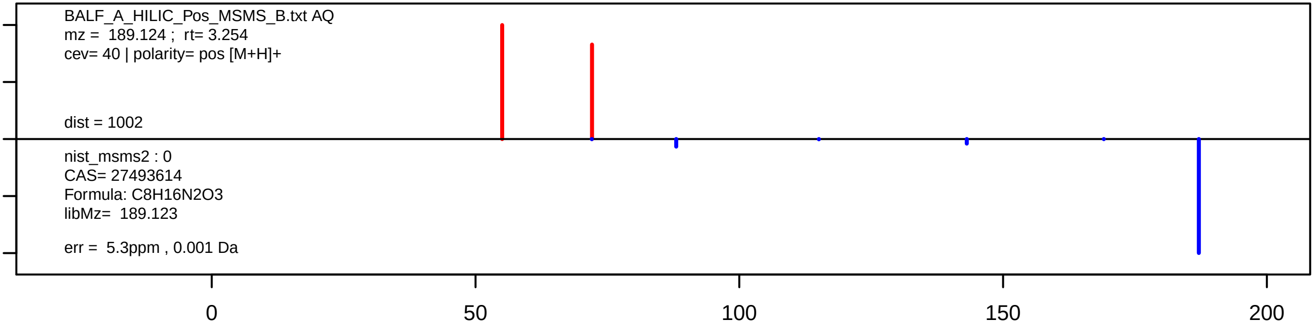

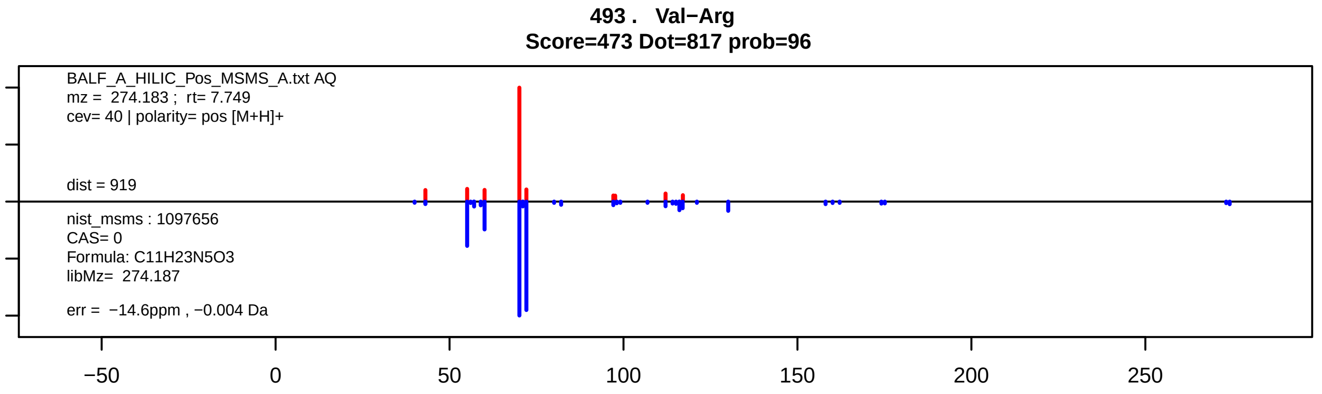
493 . Val−Arg
Score=473 Dot=817 prob=96
BALF_A_HILIC_Pos_MSMS_A.txt AQ
mz = 274.183 ; rt= 7.749
cev= 40 | polarity= pos [M+H]+
dist = 919
nist_msms : 1097656
CAS= 0
Formula: C11H23N5O3
libMz= 274.187
err = −14.6ppm , −0.004 Da
−50
0
50
100
150
200
250

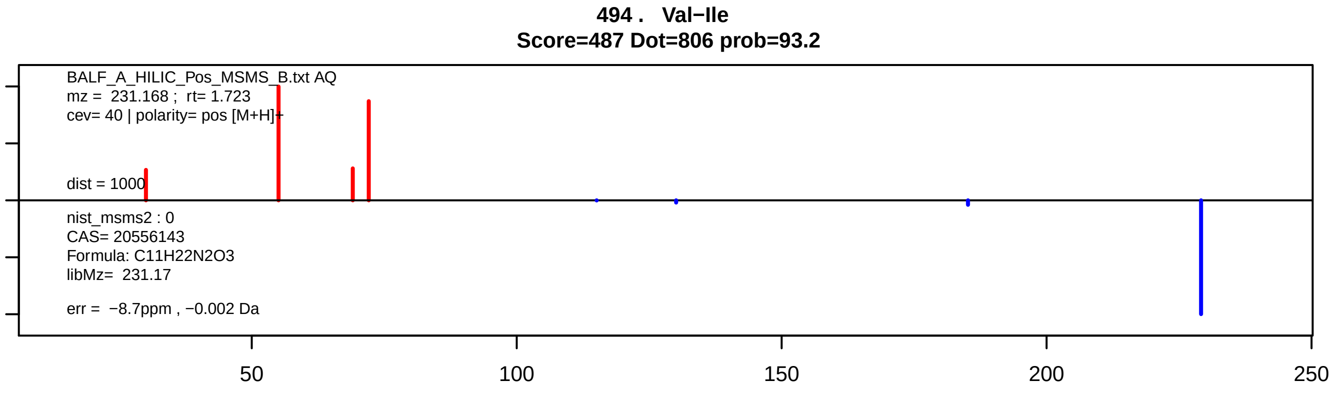
494 . Val−Ile
Score=487 Dot=806 prob=93.2
BALF_A_HILIC_Pos_MSMS_B.txt AQ
mz = 231.168 ; rt= 1.723
cev= 40 | polarity= pos [M+H]+
dist = 1000
nist_msms2 : 0
CAS= 20556143
Formula: C11H22N2O3
libMz= 231.17
err = −8.7ppm , −0.002 Da
50
100
150
200
250

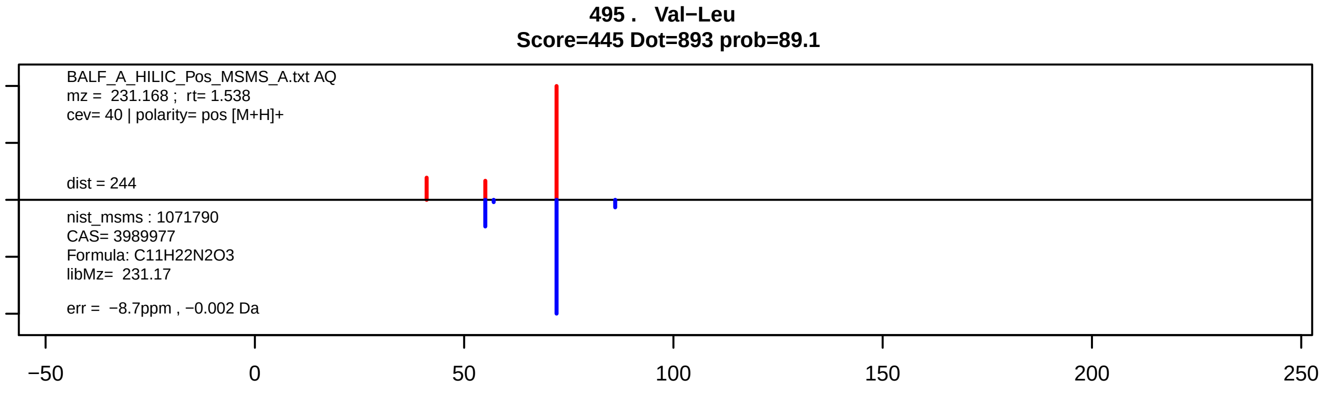
495 . Val−Leu
Score=445 Dot=893 prob=89.1
BALF_A_HILIC_Pos_MSMS_A.txt AQ
mz = 231.168 ; rt= 1.538
cev= 40 | polarity= pos [M+H]+
dist = 244
nist_msms : 1071790
CAS= 3989977
Formula: C11H22N2O3
libMz= 231.17
err = −8.7ppm , −0.002 Da
−50
0
50
100
150
200
250

496 . Val−Tyr
Score=242 Dot=838 prob=47

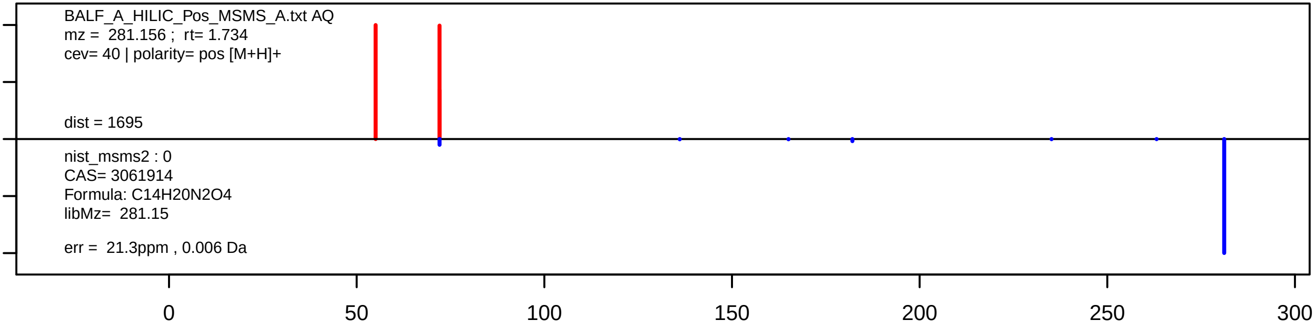

497 . Val−Val
Score=717 Dot=909 prob=97.4

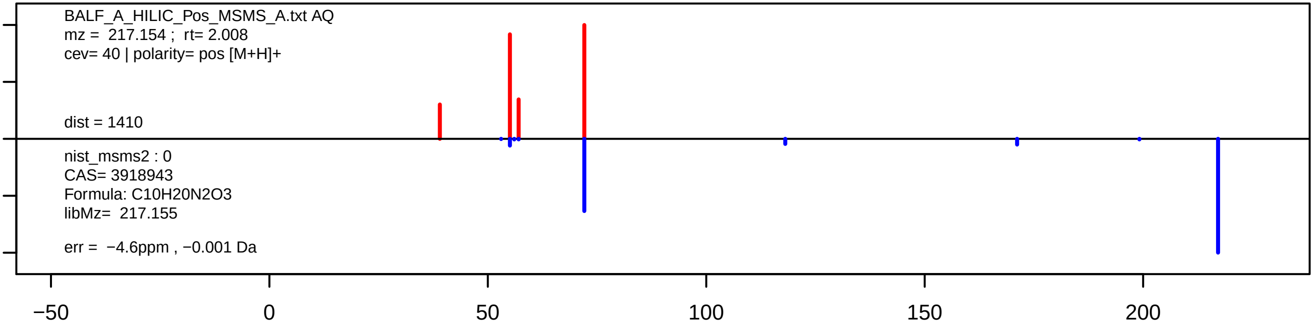

498 . Venlafaxine
Score=686 Dot=925 prob=94.9

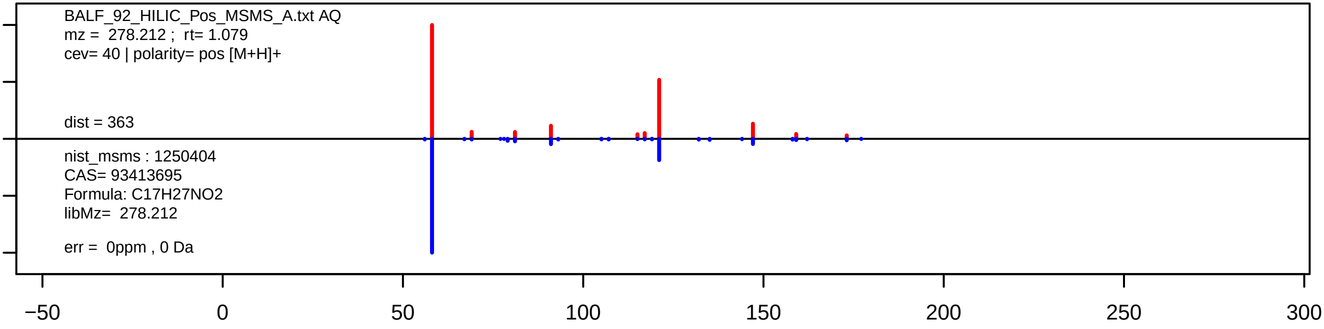

499 .   Xanthine
Score=842 Dot=910 prob=97

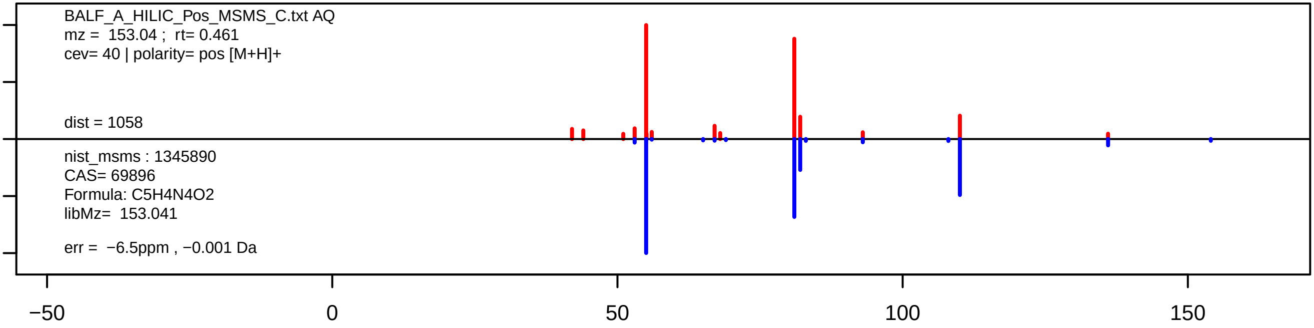

Supplement: Supplementary File 1 [file sdata201860-s2.pdf]
