# Supplementary material for: A prototypic small molecule database for bronchoalveolar lavage-based metabolomics
Source: Sci Data. 2018 Apr 17;5:180060. doi: 10.1038/sdata.2018.60 (PMC5903367; doi:10.1038/sdata.2018.60)

Supplementary File 2

Spectra used in the validation of compounds in the lipid MS/MS experiment.

Included are plots showing the matches from the NIST 17 library as described in the text. Red lines show peaks produced from the sample, and blue line show peaks from the NIST library entry. Number and text at the top of each spectra show the order number and compound name, the NIST MS Search score, dot product, and database probability as produced by the NIST MS search program. Upper left text in each plot are the referenced experimental file name and experiment tag (LIPID_MTBE), the observed precursor mz and the retention time in minutes. The collision energy (cev), polarity (pos) and adducted ion observed are noted. A distance metric (the square root of the sum of squares for the difference between the matched fragment peaks) is included (dist). The lower left panel includes the nist library index number (nist_msms) as stored in our SQL database, the CAS number, a molecular formula and the library MZ. Precursor mass error (err) is noted in ppm and Dalton (Da).

1 .   .beta.−Methylphenethylamine
Score=400 Dot=999 prob=1.3

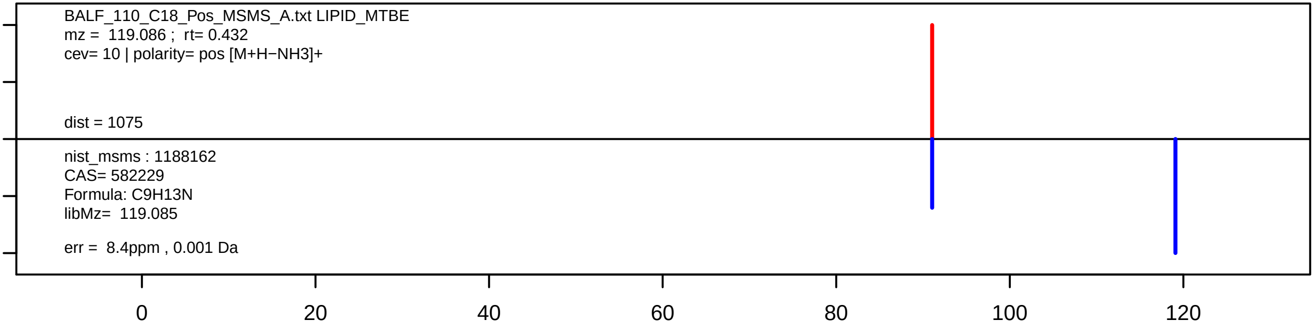

2 .   .epsilon.−Caprolactam
Score=575 Dot=820 prob=80.8

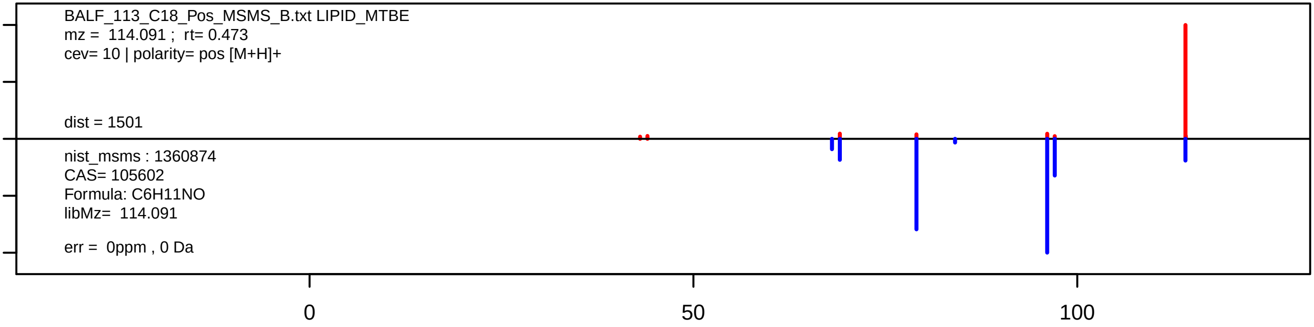

3 .   1−(1Z−Octadecenyl)−2−(4Z,7Z,10Z,13Z,16Z,19Z−docosahexaenoyl)−sn−glycero−3−phosphoethanolamine
Score=467 Dot=825 prob=91

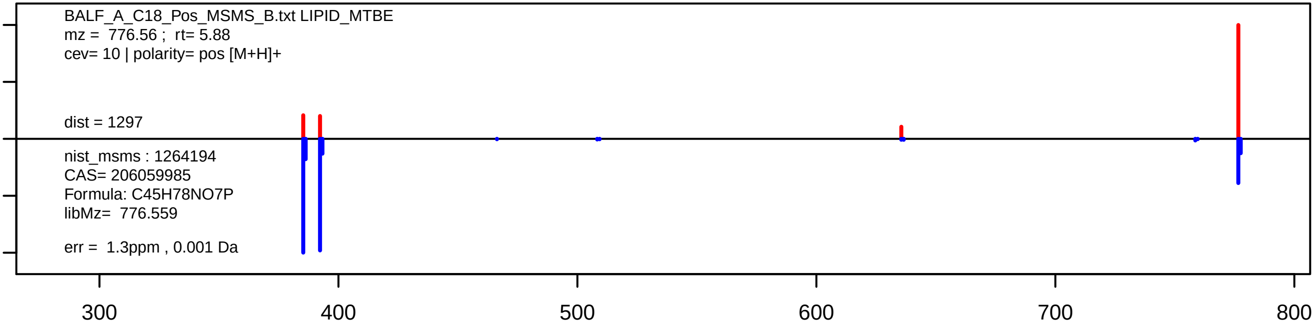

4 . 1−(1Z−Octadecenyl)−2−(5Z,8Z,11Z,14Z−eicosatetraenoyl)−sn−glycero−3−phosphocholine
Score=317 Dot=891 prob=99

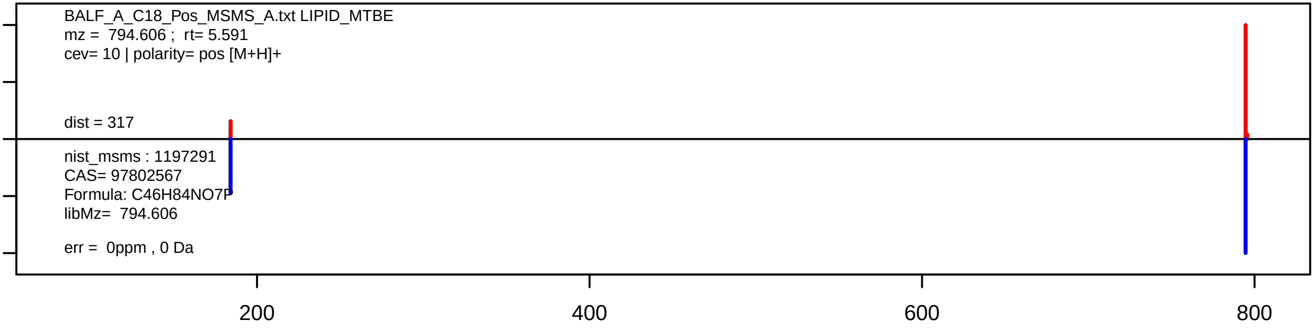

5 . 1−(1Z−Octadecenyl)−2−(5Z,8Z,11Z,14Z−eicosatetraenoyl)−sn−glycero−3−phosphoethanolamine
Score=554 Dot=929 prob=96.9

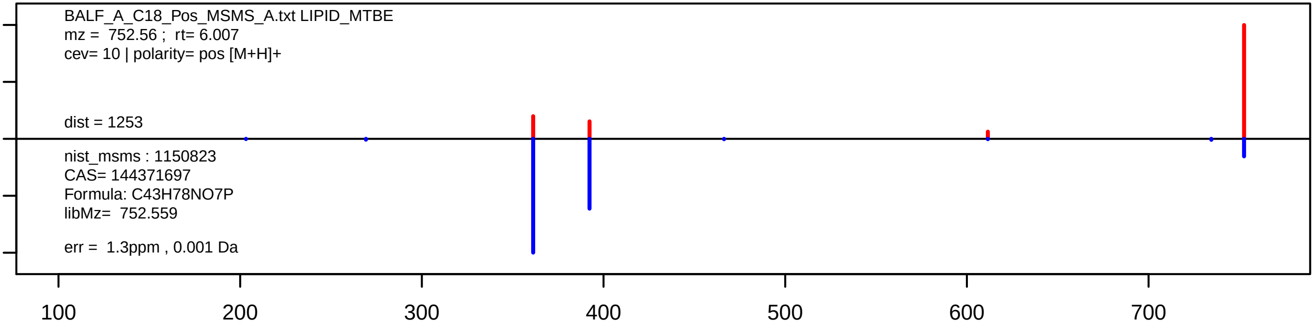

6 . 1−(1Z−Octadecenyl)−2−(9Z−octadecenoyl)−sn−glycero−3−phosphocholine
Score=400 Dot=999 prob=99

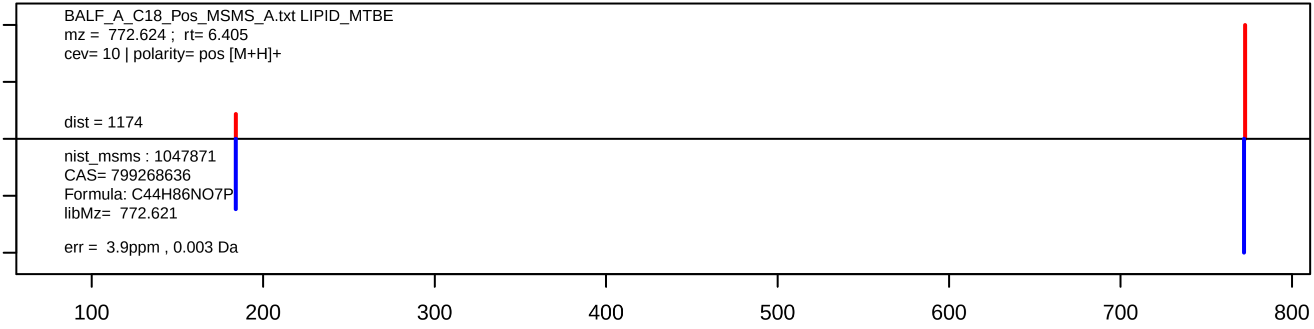

7 . 1−(1Z−Octadecenyl)−sn−glycero−3−phosphocholine
Score=220 Dot=859 prob=95.1

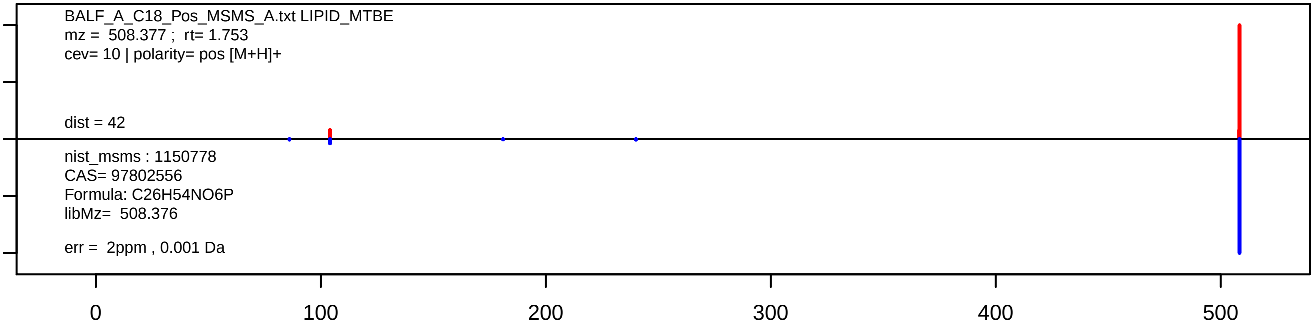

8 . 1−Cyclohexylethanol
Score=810 Dot=979 prob=43

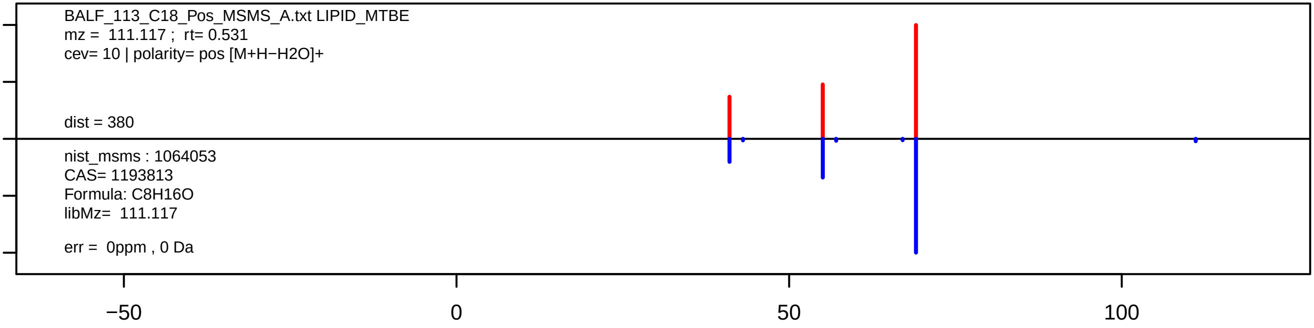

9 . 1−Eicosatrienoyl−sn−glycero−3−phosphoethanolamine
Score=285 Dot=862 prob=20.2

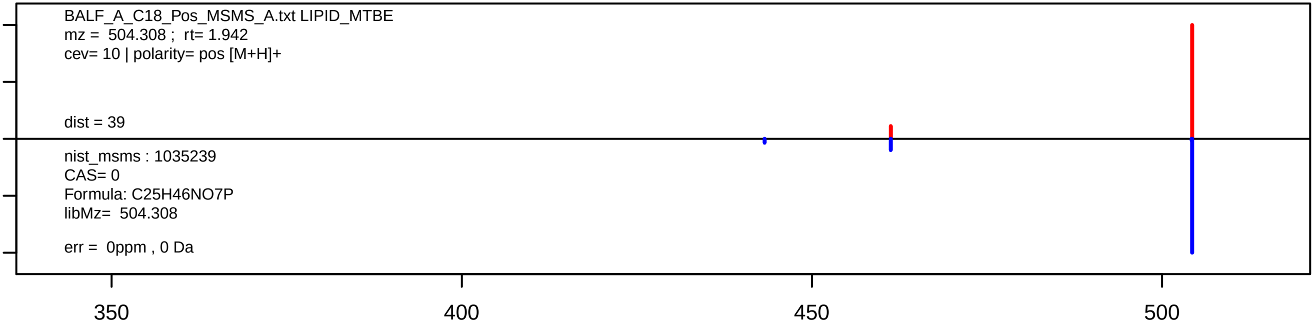

10 . 1−Heneicosanoyl−2−(4Z,7Z,10Z,13Z,16Z,19Z−docosahexaenoyl)−sn−glycero−3−phosphocholine
Score=281 Dot=987 prob=99

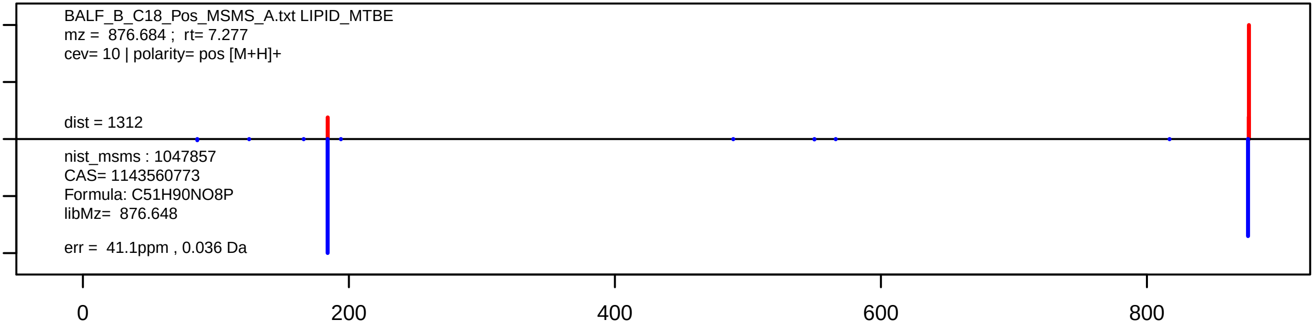

11 . 1−Heptadecanoyl−2−(5Z,8Z,11Z,14Z−eicosatetraenoyl)−sn−glycero−3−phosphocholine
Score=239 Dot=983 prob=100

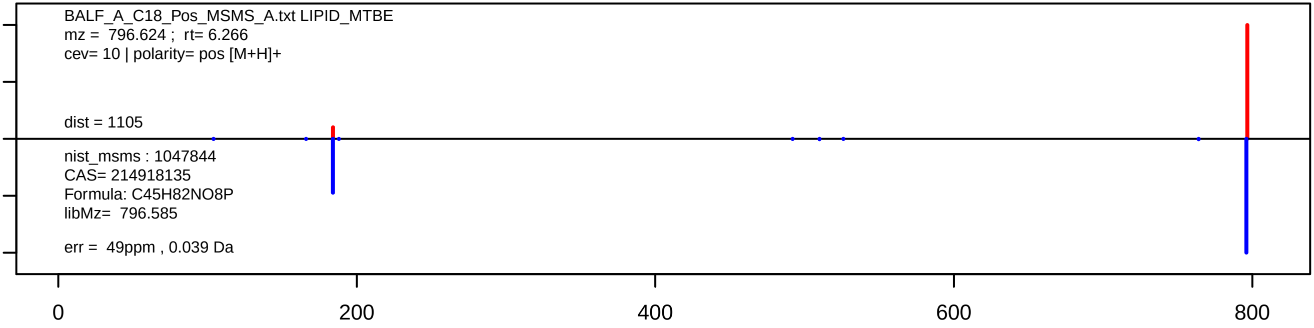

12 . 1−Heptadecanoyl−sn−glycero−3−phosphocholine
Score=479 Dot=945 prob=91.9

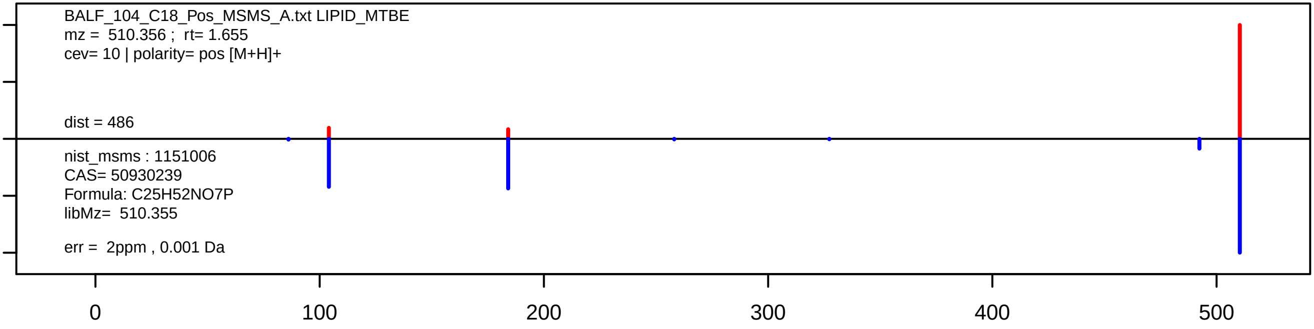

13 .   1−Hexadecanoyl−2−(14−pentadecenoyl)−sn−glycero−3−phosphocholine
Score=295 Dot=991 prob=94

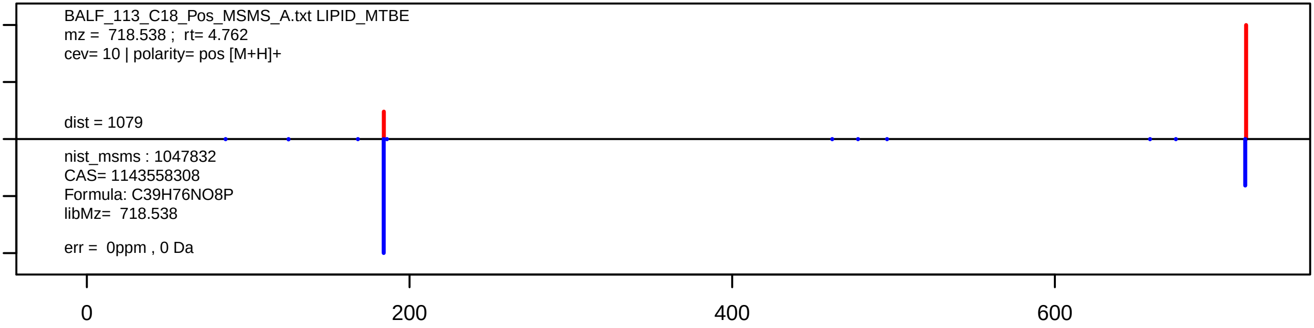

14 .   1−Hexadecanoyl−2−(5Z,8Z,11Z,14Z−eicosatetraenoyl)−sn−glycero−3−phosphocholine
Score=400 Dot=999 prob=97.8

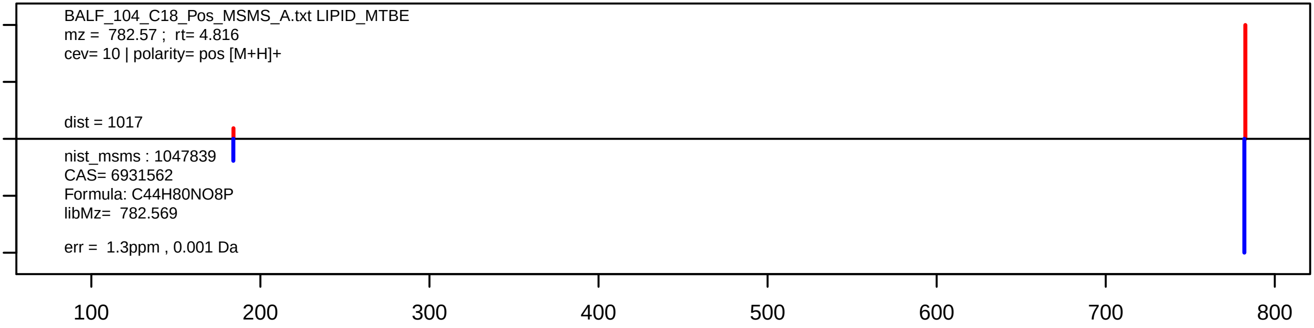

15 .   1−Hexadecanoyl−2−octadecadienoyl−sn−glycero−3−phosphocholine
Score=329 Dot=921 prob=50

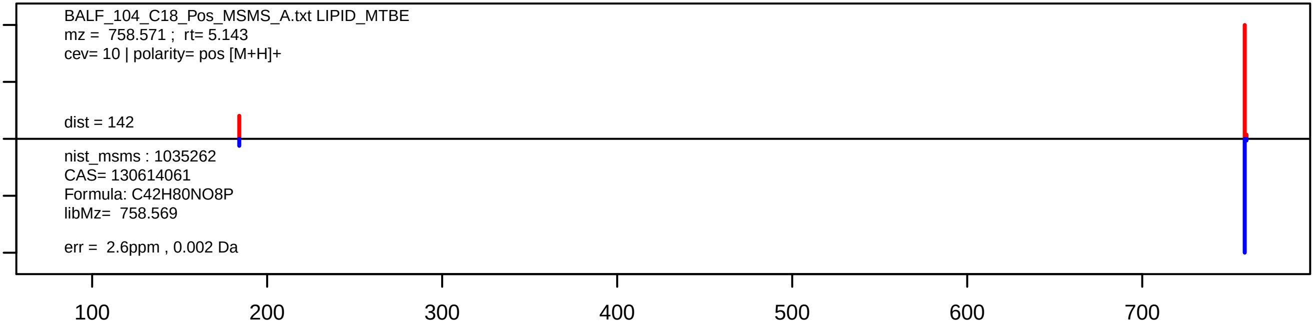

16 . 1−Hexadecyl−2−(5Z,8Z,11Z,14Z−eicosatetraenoyl)−sn−glycero−3−phosphocholine
Score=332 Dot=929 prob=100

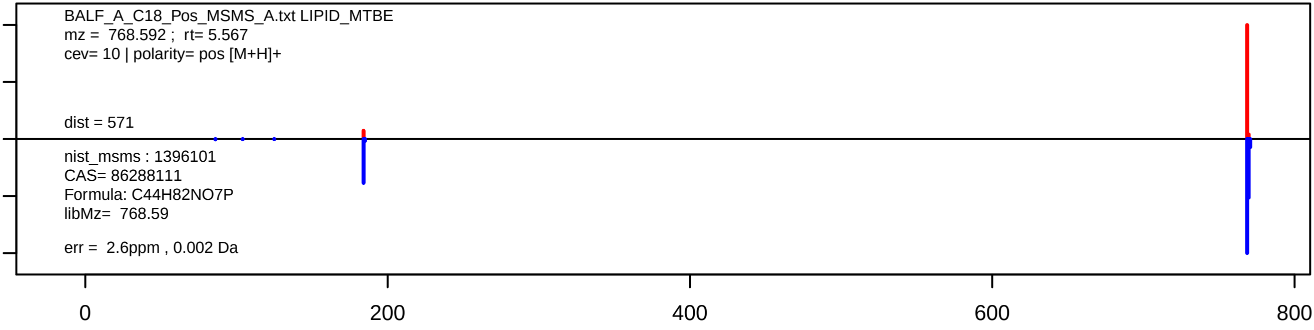

17 . 1−Hexadecyl−2−(8Z,11Z,14Z−eicosatrienoyl)−sn−glycero−3−phosphocholine
Score=346 Dot=954 prob=100

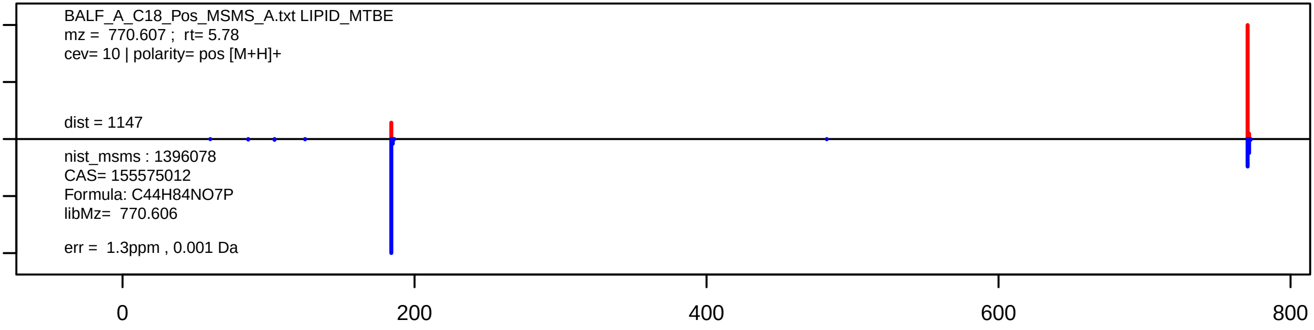

18 . 1−Hexadecyl−2−(9Z−octadecenoyl)−sn−glycero−3−phosphocholine
Score=400 Dot=999 prob=100

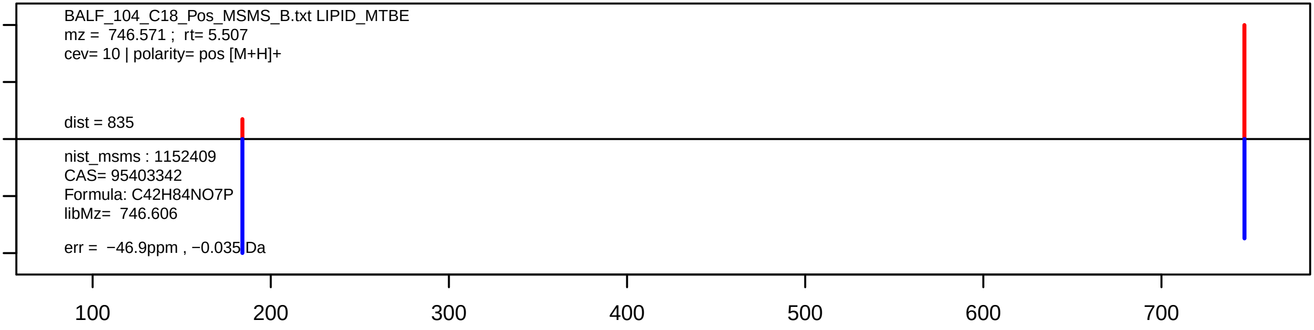

19 . 1−Hexadecyl−sn−glycero−3−phosphocholine
Score=400 Dot=999 prob=99

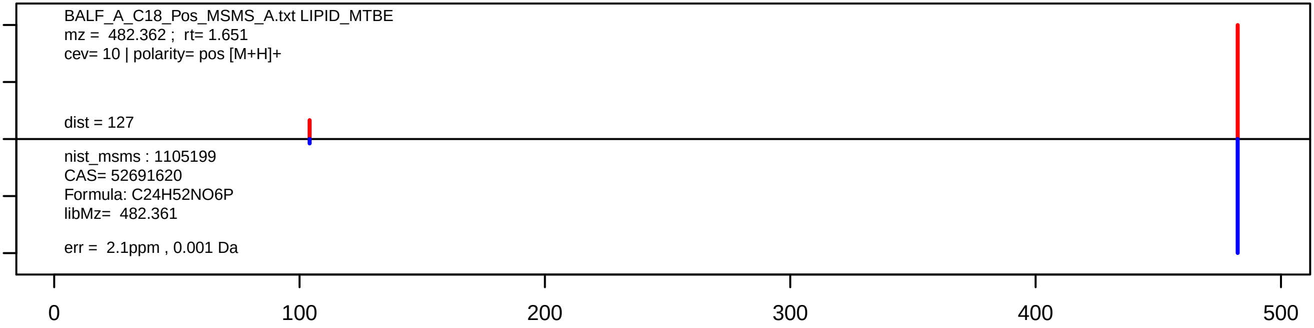

20 . 1−Hexadecylamine
Score=511 Dot=946 prob=98.5

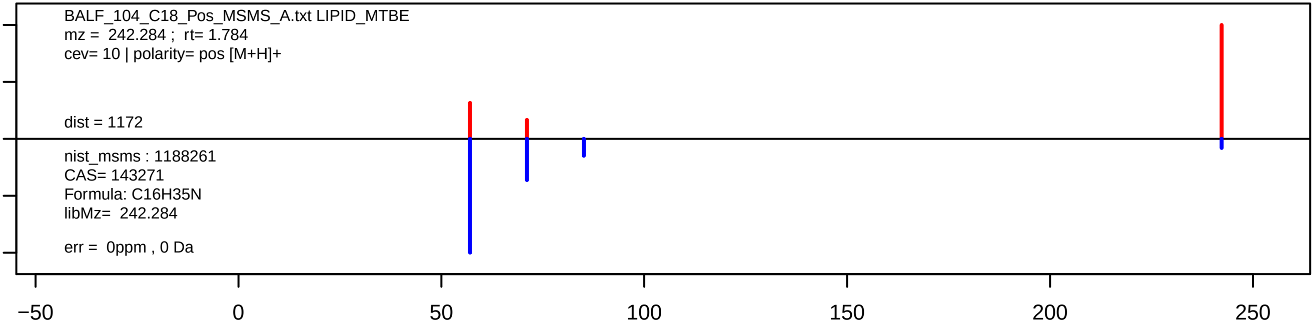

21 . 1−Linoleoyl−2−stearoyl−sn−glycero−3−phospho−(1'−sn−glycerol)
Score=282 Dot=927 prob=48.1

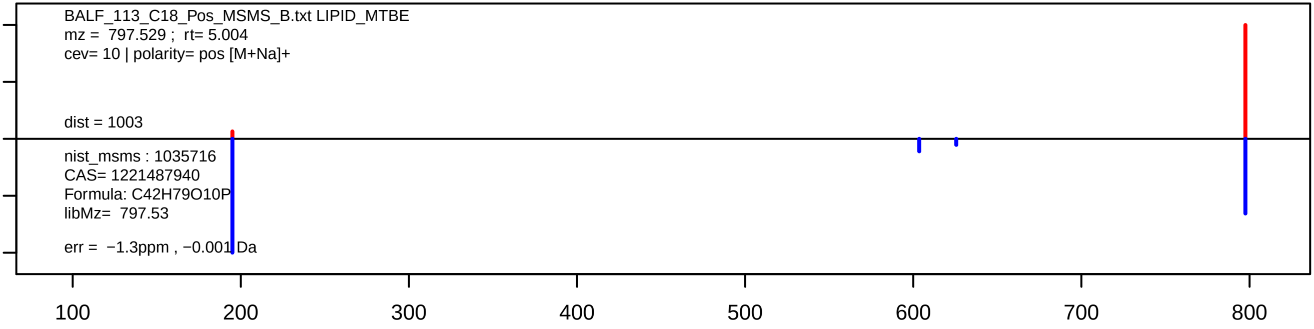

22 . 1−Methyl−3−phenylpropylamine
Score=400 Dot=999 prob=35.7

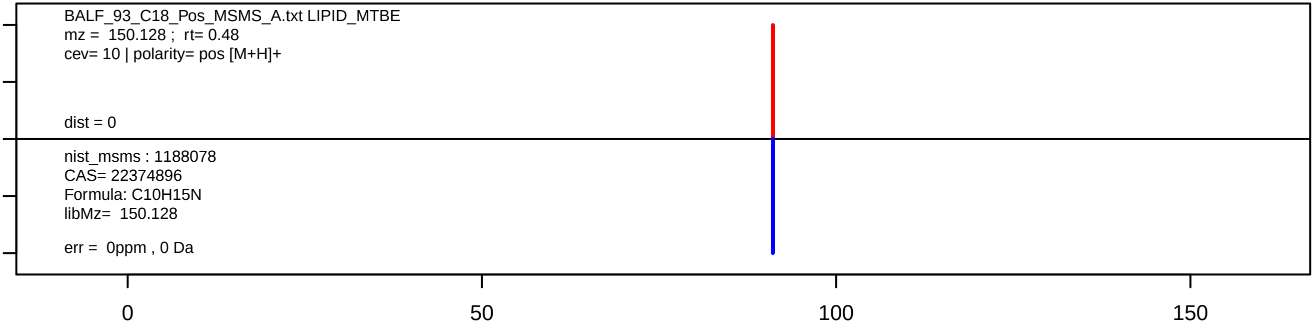

23 . 1−Myristoyl−2−palmitoyl−sn−glycero−3−phosphocholine
Score=336 Dot=874 prob=64.2

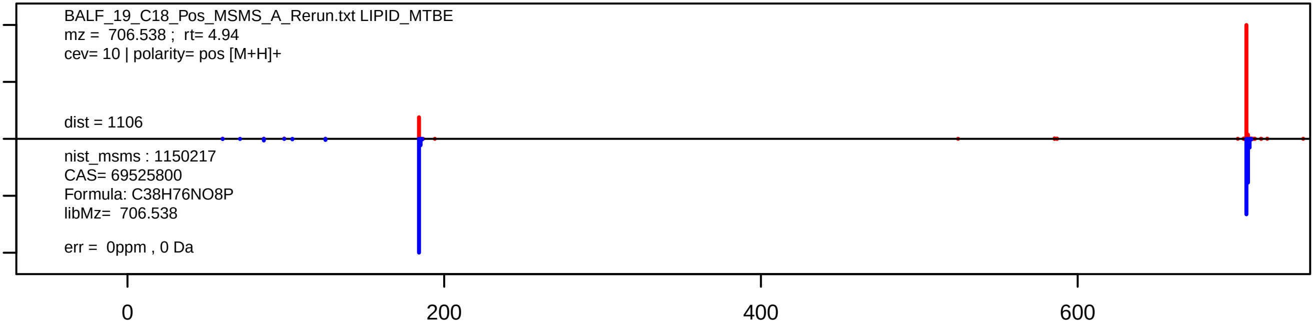

24 . 1−Myristoyl−2−stearoyl−sn−glycero−3−phosphocholine
Score=435 Dot=934 prob=36.9

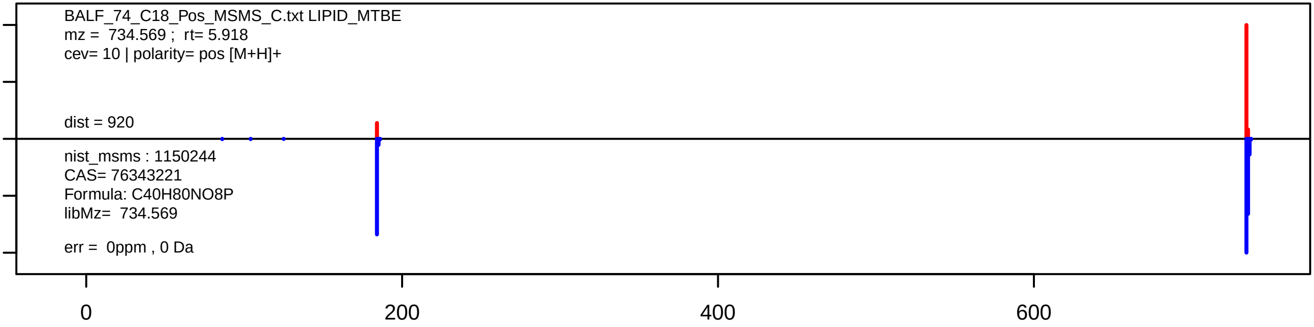

25 . 1−O−Hexadecyl−2−O−(4Z,7Z,10Z,13Z,16Z,19Z−docosahexaenoyl)−sn−glyceryl−3−phosphorylcholine
Score=109 Dot=880 prob=92.5

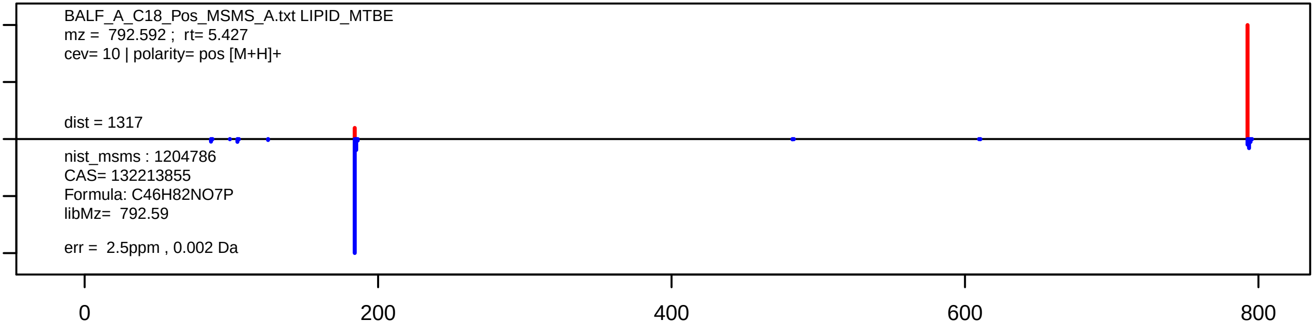

26 . 1−O−Hexadecyl−2−O−(5Z,8Z,11Z,14Z,17Z−eicosapentaenoyl)−sn−glyceryl−3−phosphorylcholine
Score=144 Dot=917 prob=96.8

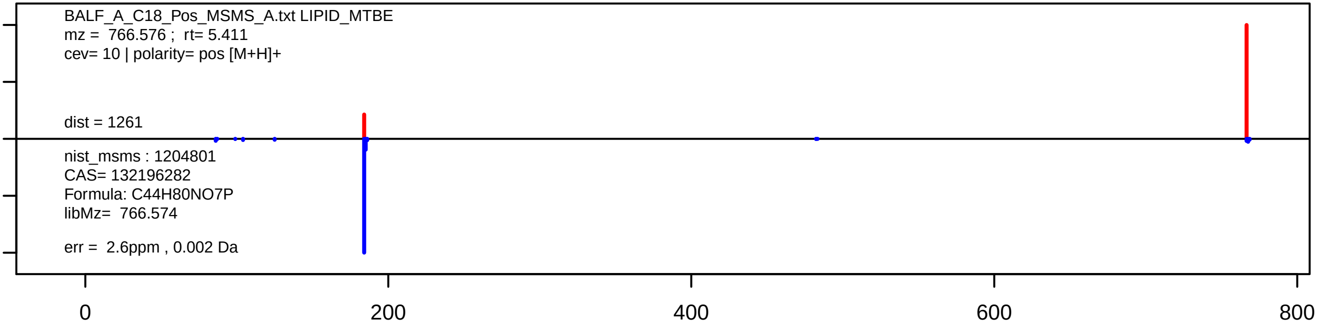

27 . 1−O−Hexadecyl−2−O−acetyl−sn−glyceryl−3−phosphorylcholine
Score=400 Dot=999 prob=98.6

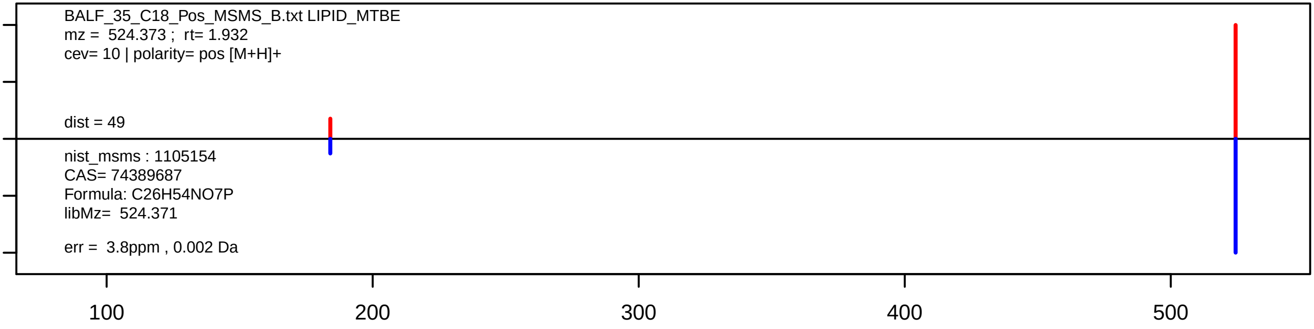

28 . 1−O−Octadecyl−sn−glyceryl−3−phosphorylcholine
Score=631 Dot=920 prob=92.8

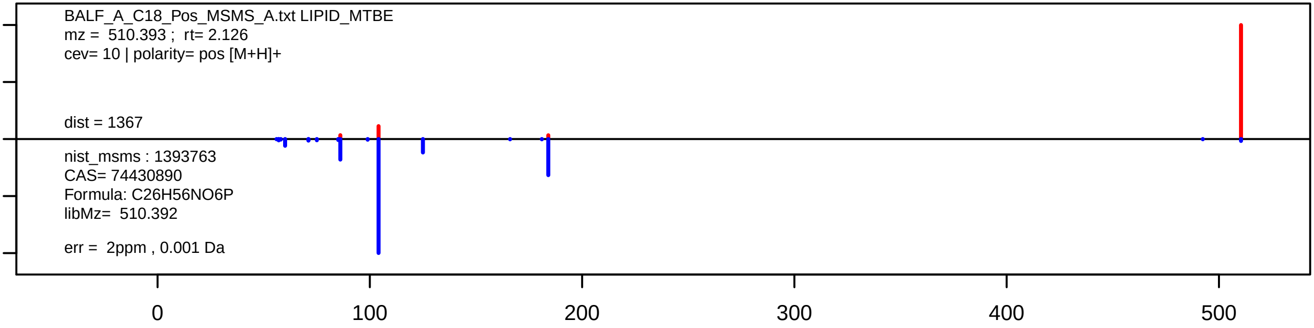

29 . 1−Octen−3−ol
Score=697 Dot=989 prob=41.4

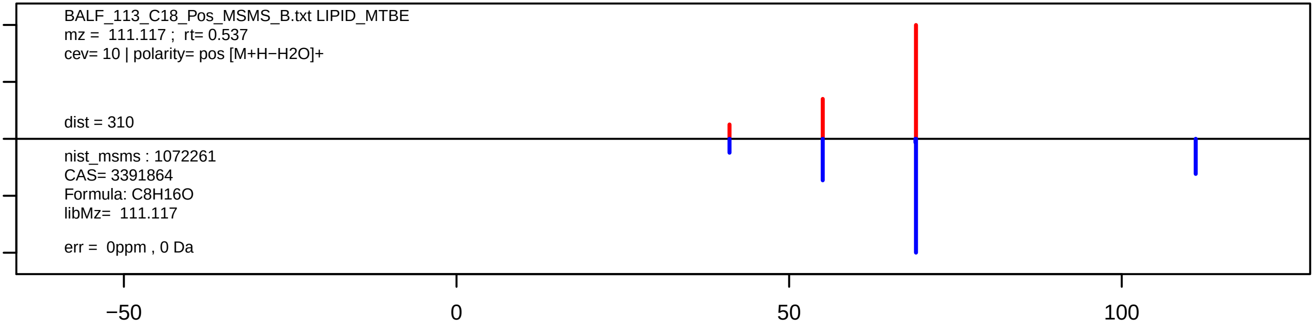

30 . 1−Oleoyl−2−myristoyl−sn−glycero−3−phosphocholine
Score=435 Dot=931 prob=95.4

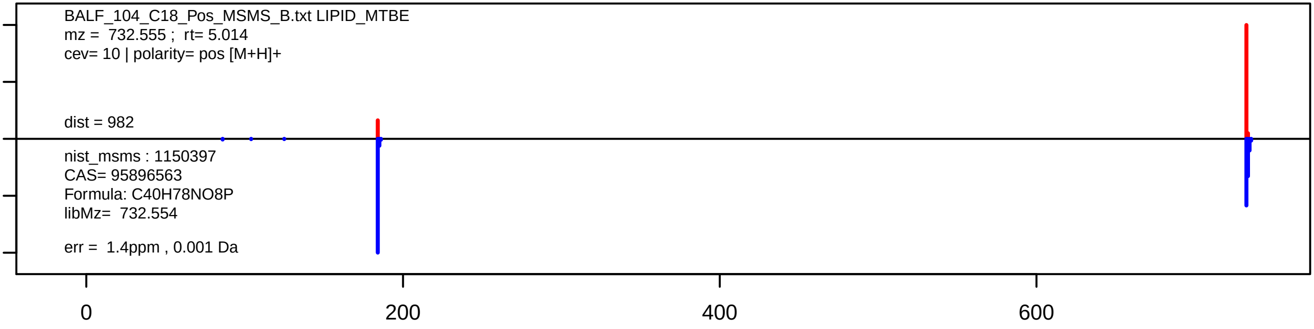

31 . 1−Oleoyl−sn−glycero−3−phosphocholine

Score=401 Dot=930 prob=90.2

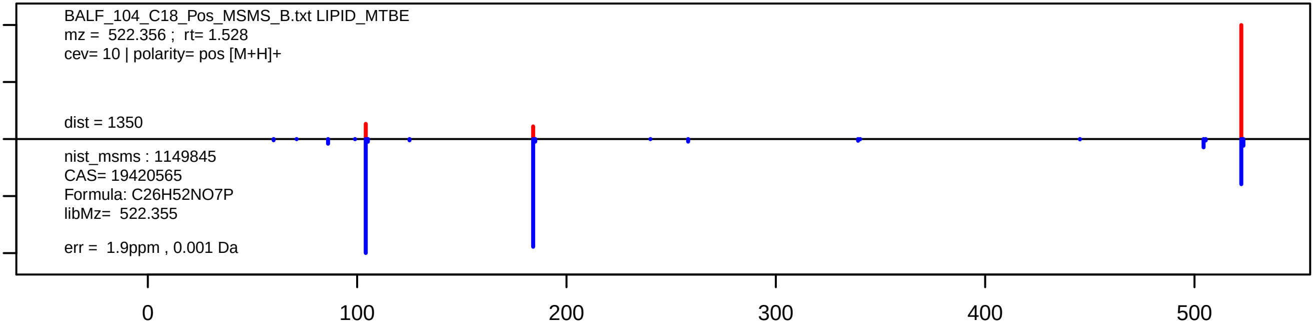

32 . 1−Oleoyl−sn−glycero−3−phosphoethanolamine

Score=391 Dot=824 prob=34.9

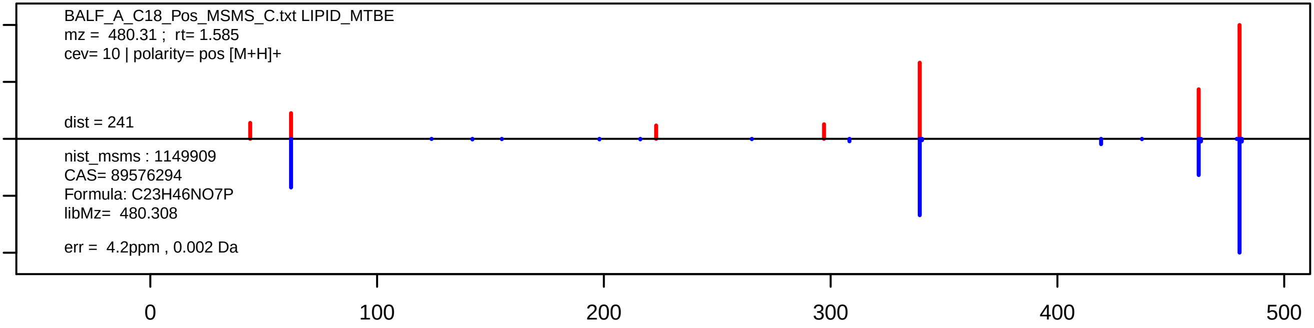

33 . 1−Palmitoyl−2−arachidonoyl−sn−glycero−3−phospho−(1'−sn−glycerol)

Score=96 Dot=895 prob=55.6

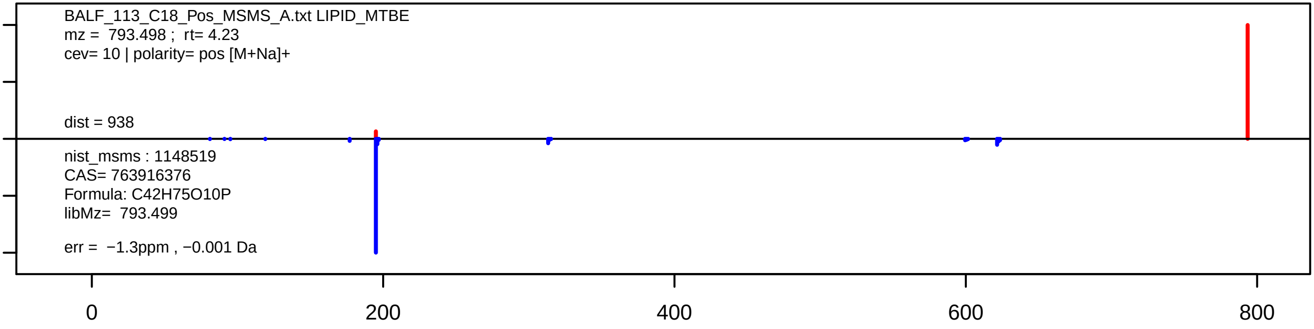

34 . 1−Palmitoyl−2−docosahexaenoyl−sn−glycero−3−phosphocholine
Score=400 Dot=999 prob=100

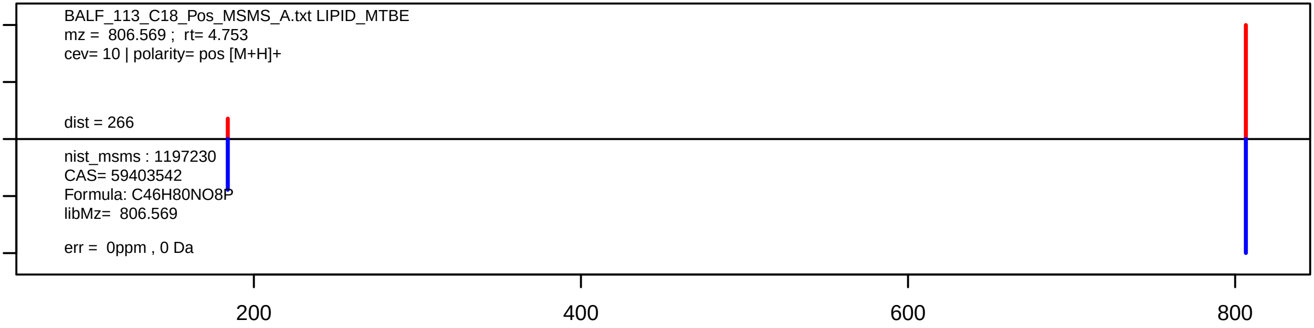

35 . 1−Palmitoyl−2−hydroxy−sn−glycero−3−phosphoethanolamine
Score=882 Dot=942 prob=97.4

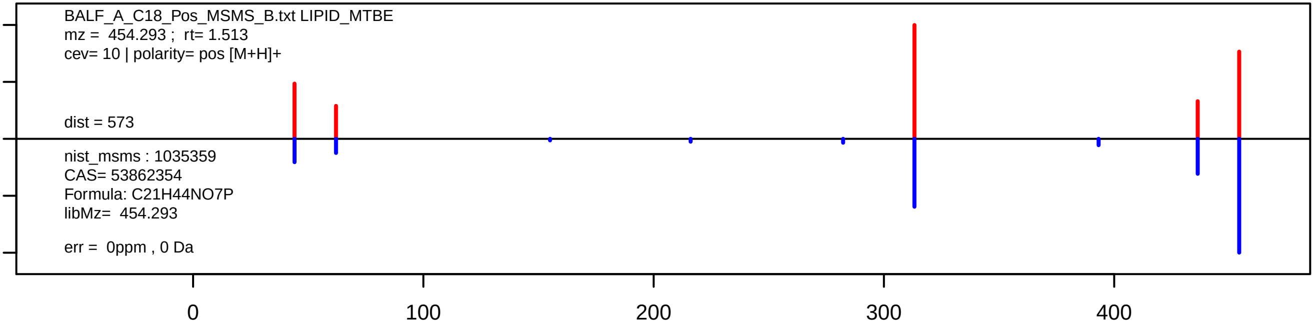

36 . 1−Palmitoyl−2−lauroyl−sn−glycero−3−phosphorylcholine
Score=346 Dot=925 prob=83

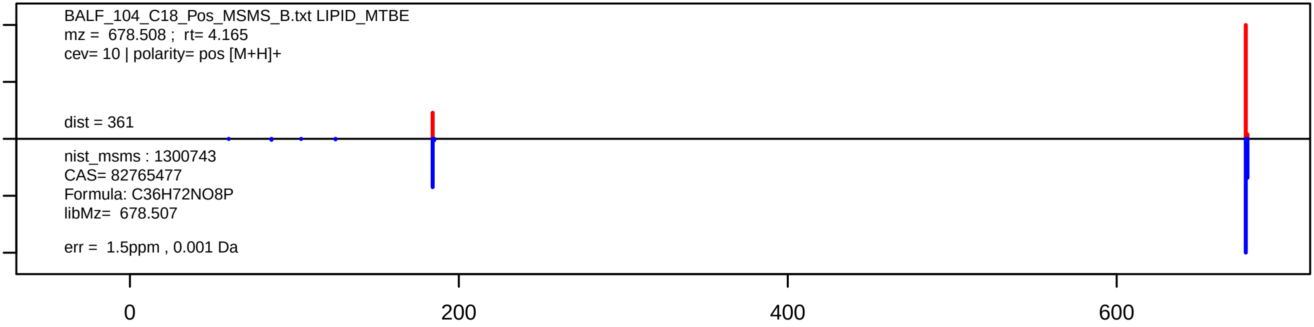

37 . 1−palmitoyl−2−linoleoyl−sn−glycero−3−phospho−(1'−rac−glycerol)
Score=296 Dot=948 prob=98.5

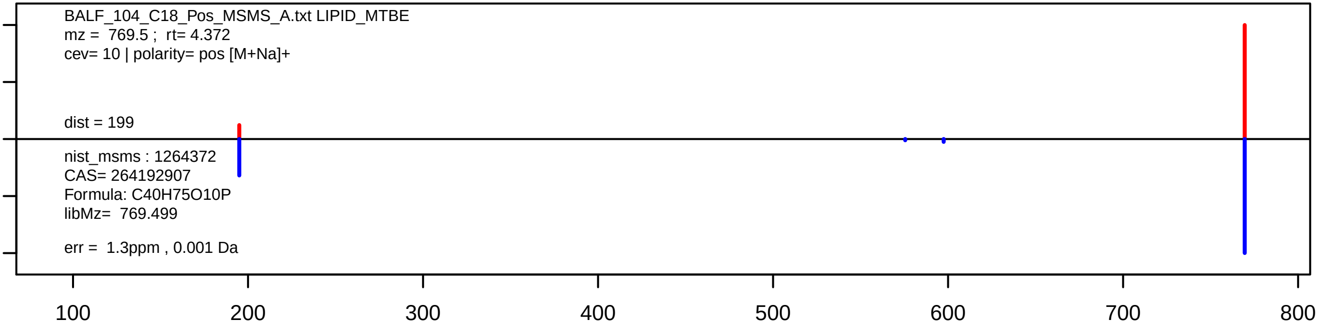

38 . 1−Palmitoyl−2−linoleoyl−sn−glycero−3−phosphocholine
Score=400 Dot=999 prob=93

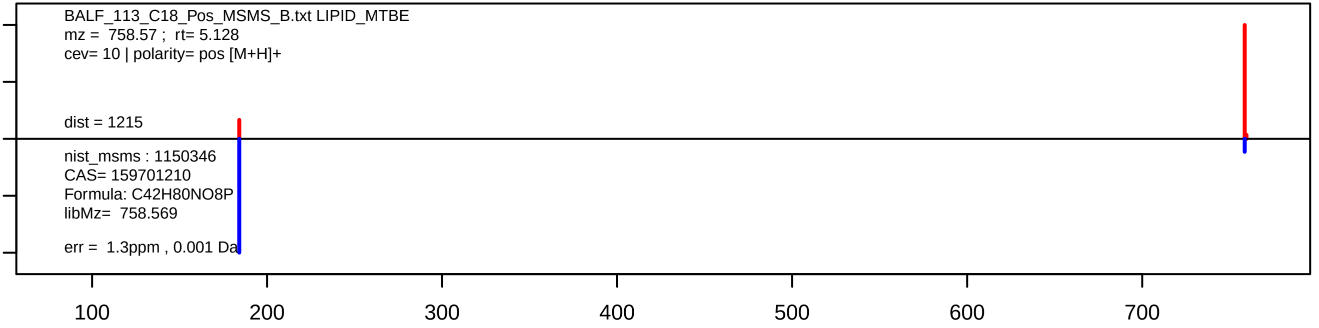

39 . 1−Palmitoyl−2−myristoyl−sn−glycero−3−phosphocholine
Score=557 Dot=960 prob=97.4

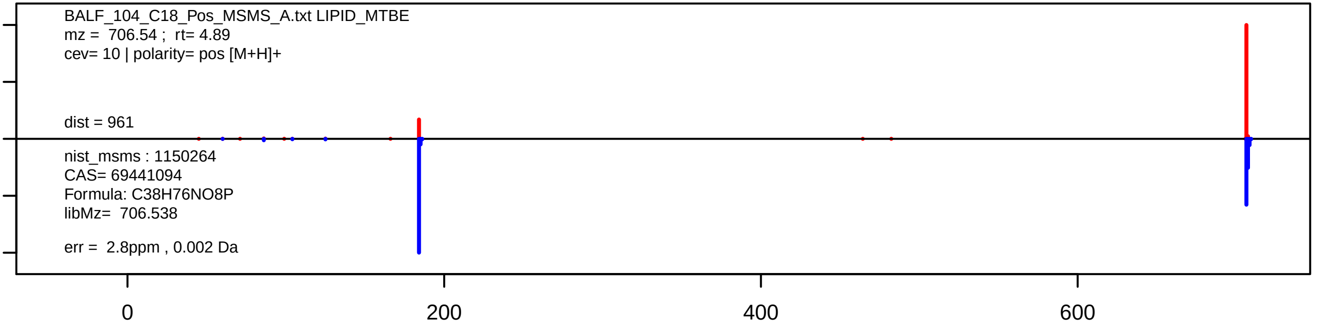

40 . 1−Palmitoyl−2−oleoyl−phosphatidylglycerol
Score=400 Dot=999 prob=100

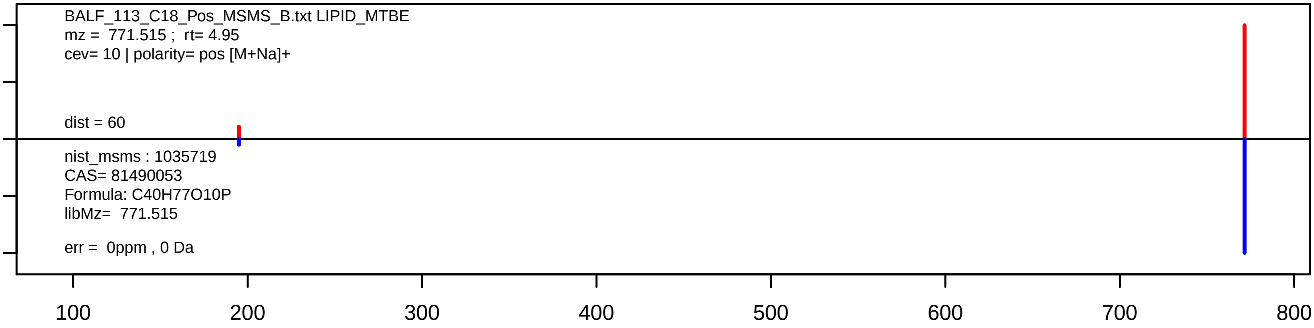

41 . 1−Palmitoyl−2−oleoyl−sn−glycero−3−phosphoethanolamine
Score=326 Dot=915 prob=10.1

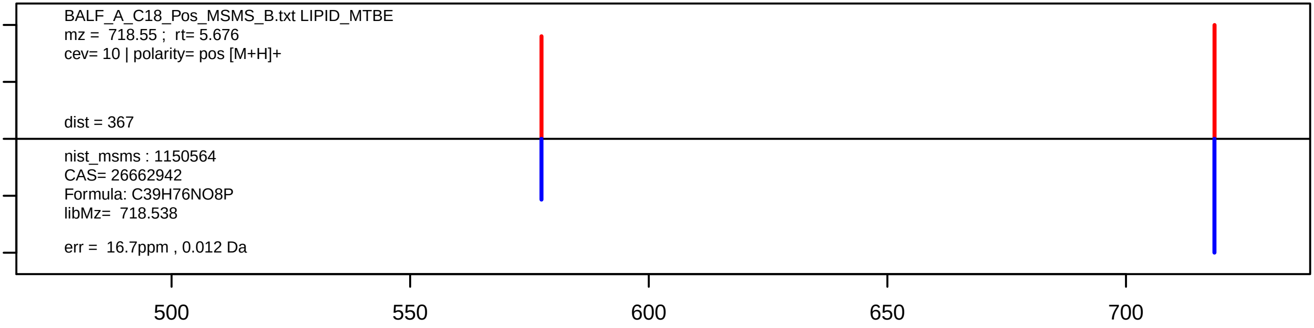

42 . 1−Palmitoyl−3−oleoyl−sn−glycero−2−phosphoethanolamine
Score=400 Dot=999 prob=9.4

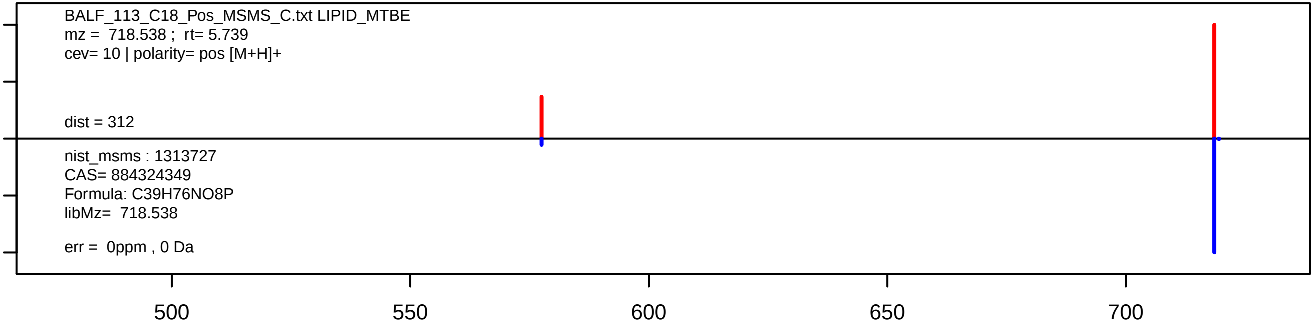

43 .   1−Palmitoyl−sn−glycero−3−phosphocholine
Score=238 Dot=819 prob=98.1

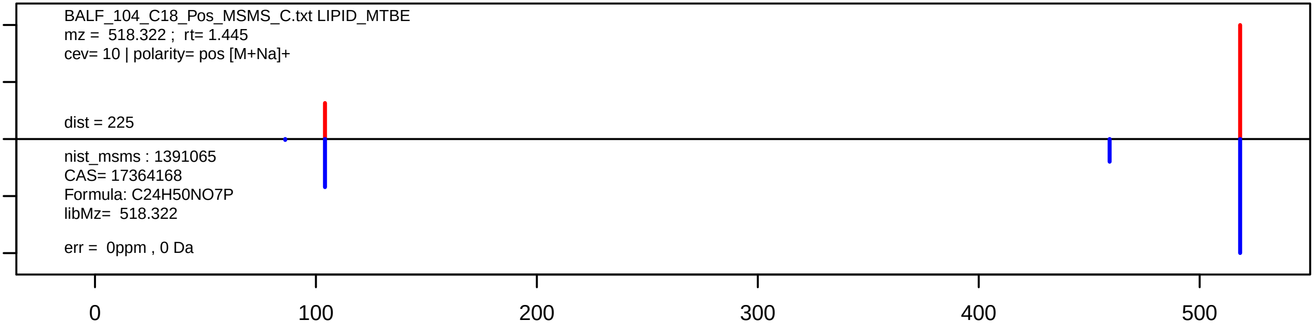

44 .   1−Pentadecanoyl−sn−glycero−3−phosphocholine
Score=453 Dot=887 prob=82.3

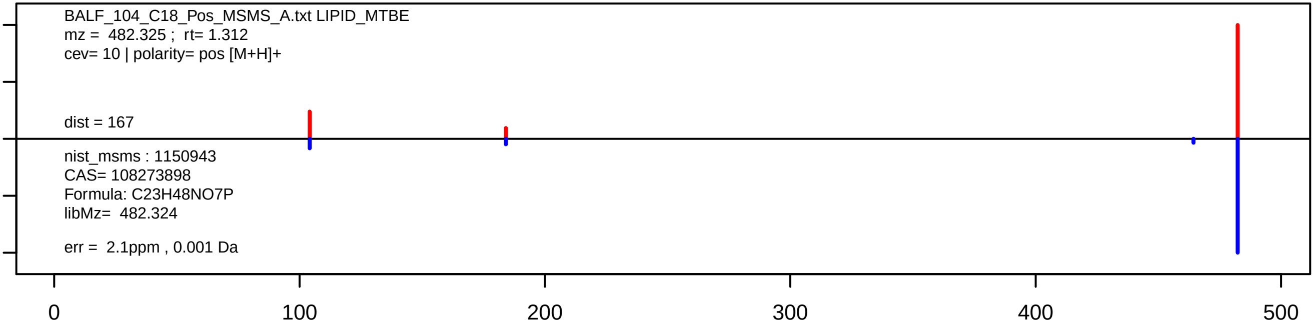

45 .   1−Propanol
Score=374 Dot=965 prob=97.6

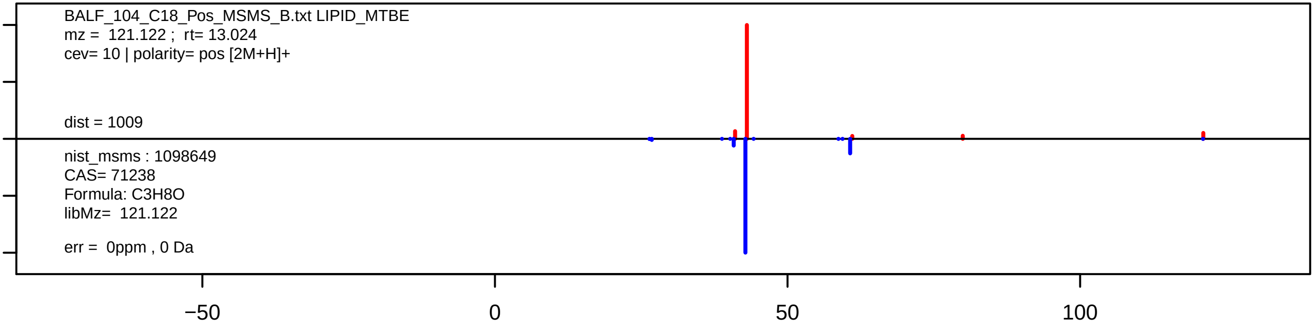

46 . 1−Stearoyl−2−arachidonyl−sn−glycero−3−phosphocholine
Score=166 Dot=931 prob=61.5

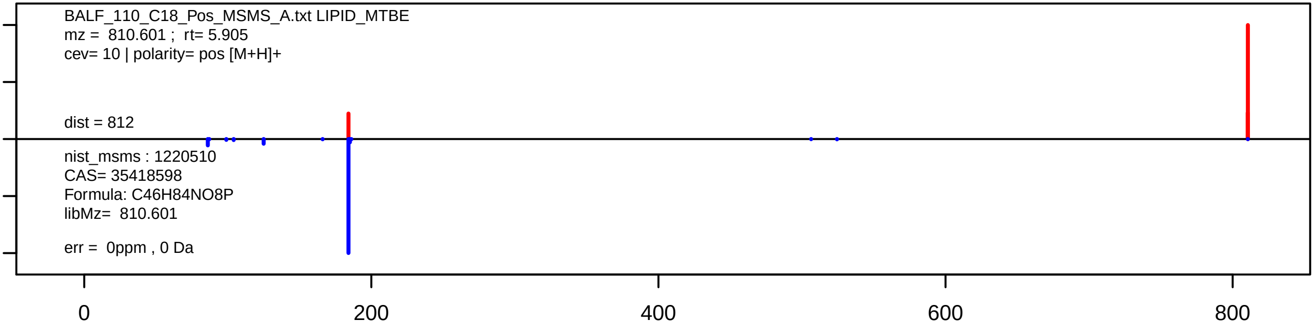

47 . 1−Stearoyl−2−hydroxy−sn−glycero−3−phosphocholine
Score=459 Dot=945 prob=98.1

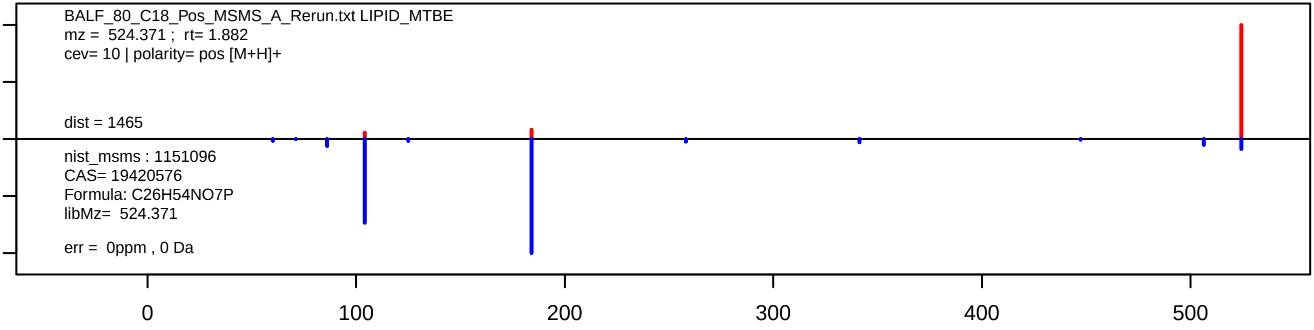

48 . 1−Stearoyl−2−hydroxy−sn−glycero−3−phosphoethanolamine
Score=894 Dot=978 prob=98.8

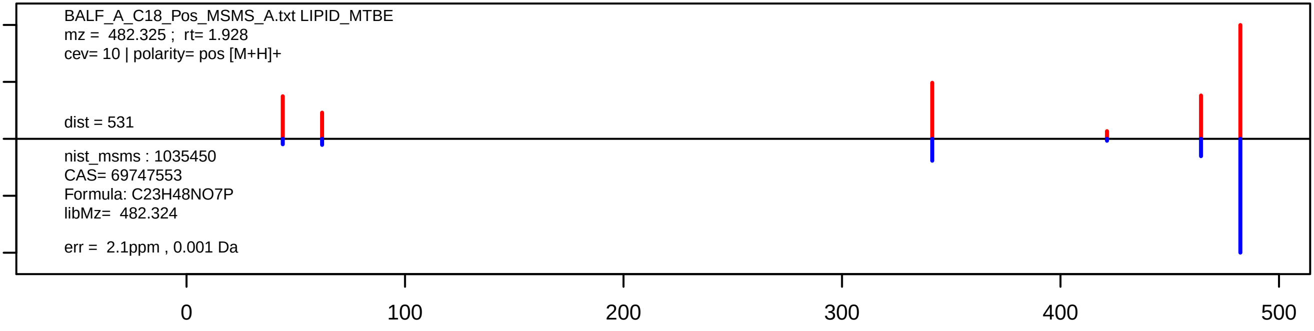

49 . 1−Stearoyl−2−linoleoyl−sn−glycero−3−phosphocholine
Score=400 Dot=999 prob=50

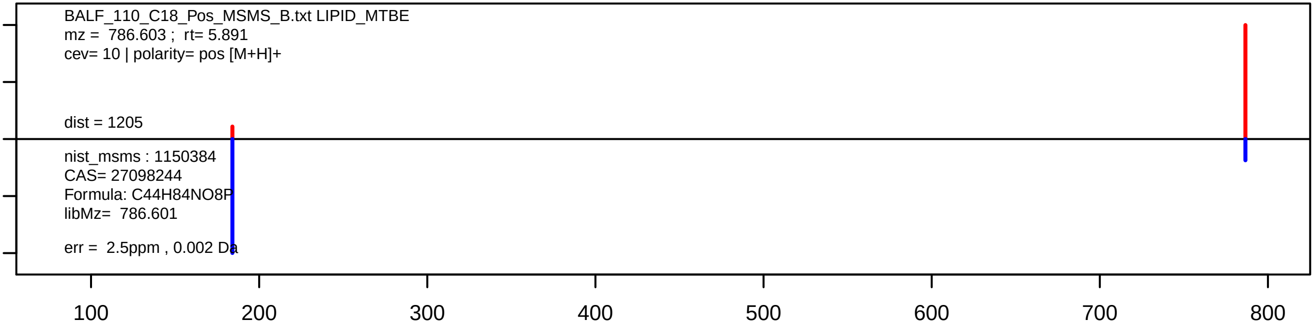

50 . 1−Stearoyl−2−myristoyl−sn−glycero−3−phosphocholine
Score=397 Dot=918 prob=80.7

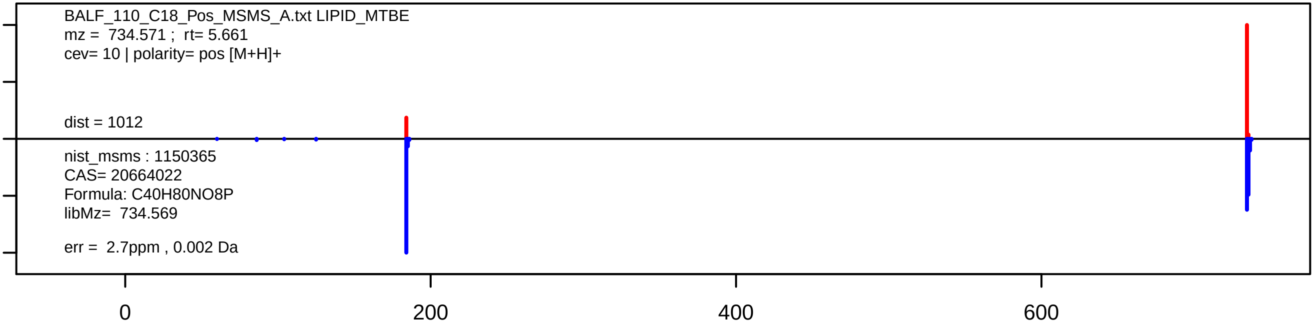

51 . 1−Stearoyl−2−oleoyl−sn−glycero−3−phosphoethanolamine
Score=400 Dot=999 prob=10.4

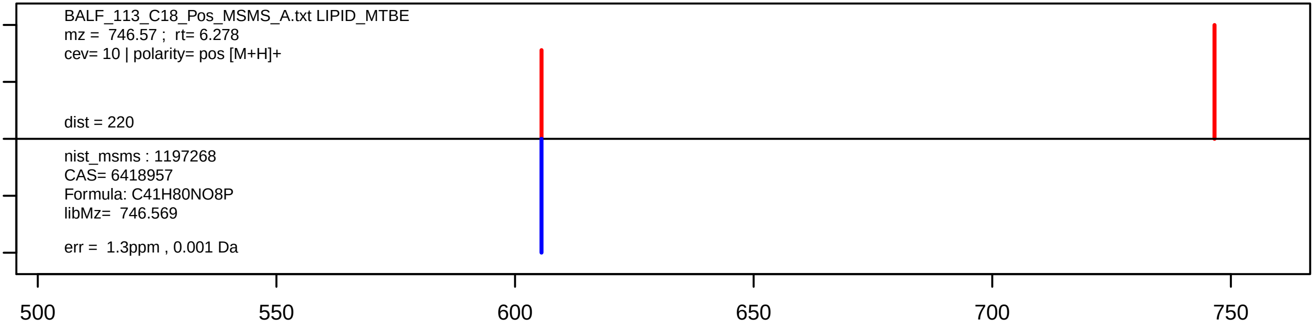

52 . 1,2−Diheptadecanoyl−sn−glycero−3−phosphocholine
Score=186 Dot=942 prob=91.8

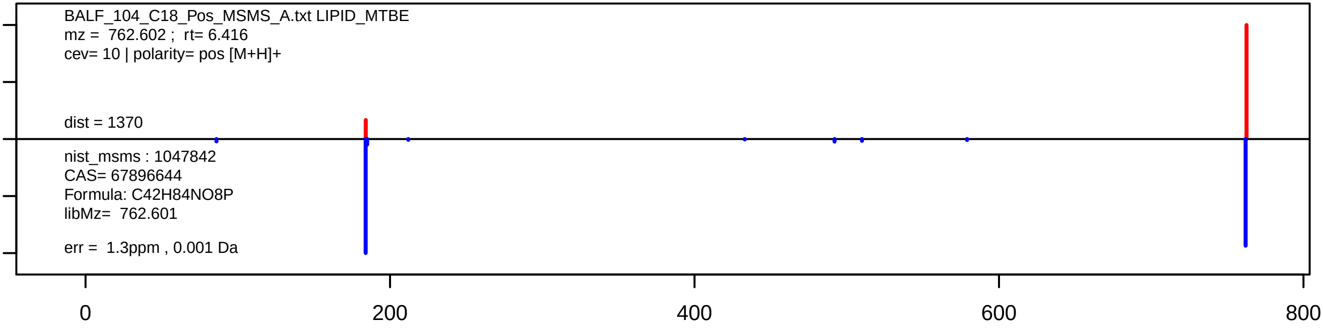

53 . 1,2−Dihexadecanoyl−sn−glycero−3−phosphocholine
Score=297 Dot=826 prob=81.8

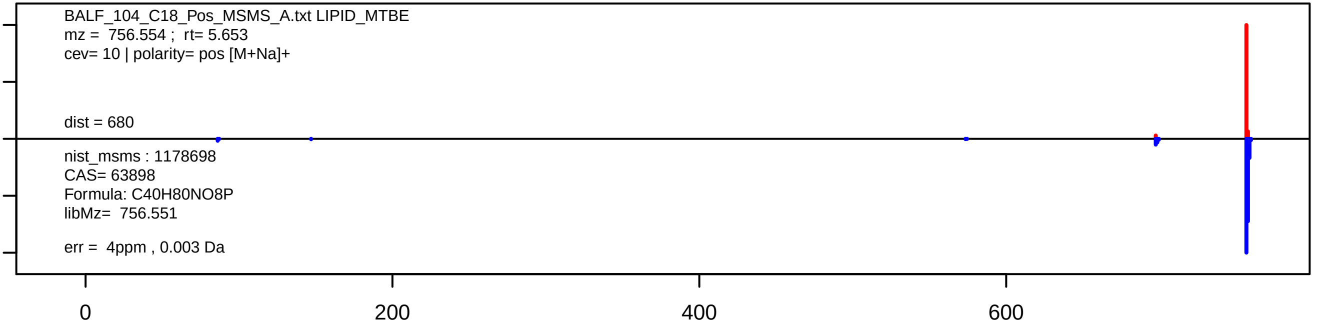

54 . 1,2−Dilinoleoyl−sn−glycero−3−phosphocholine
Score=362 Dot=889 prob=87.2

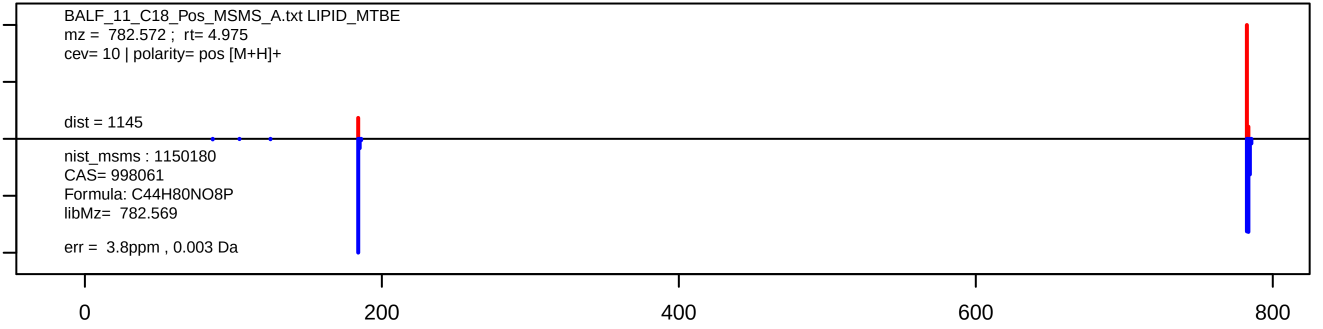

55 . 1,2−Dilinoleoyl−sn−glycero−3−phosphoethanolamine
Score=399 Dot=999 prob=5.3

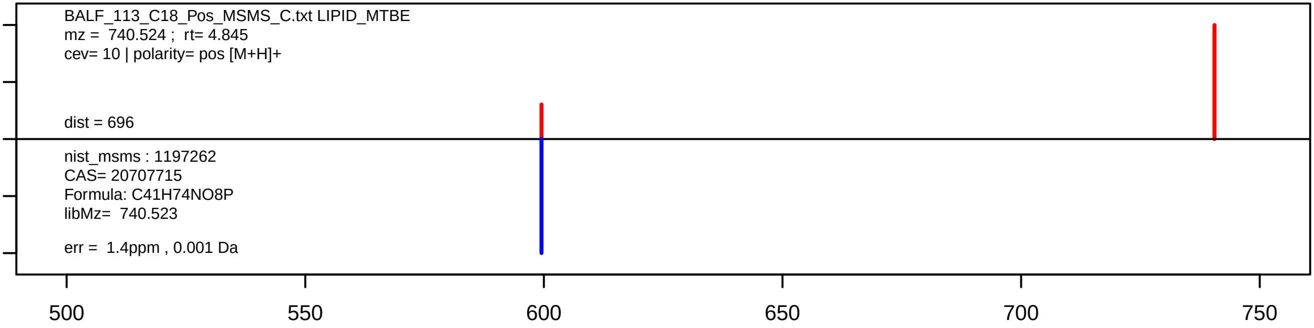

56 . 1,2−dioleoyl−sn−glycero−3−phosphatidylcholine
Score=400 Dot=999 prob=50

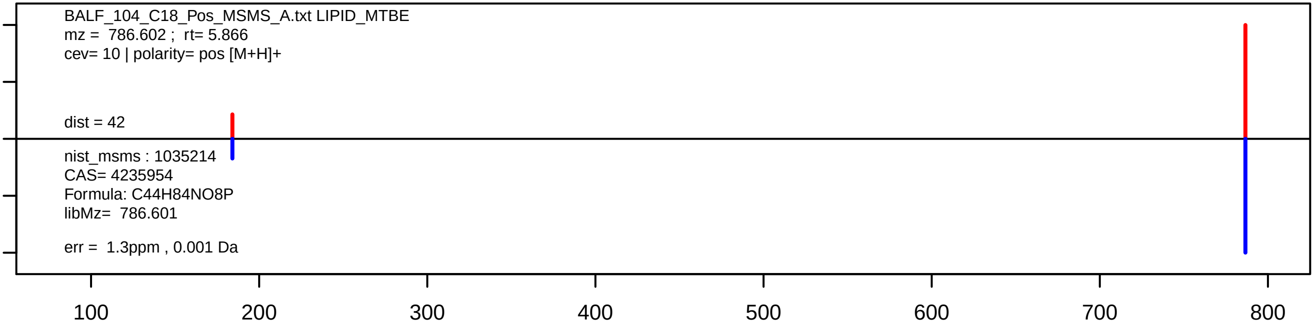

57 . 1,2−Dioleoyl−sn−glycero−3−phosphoethanolamine
Score=399 Dot=999 prob=3.6

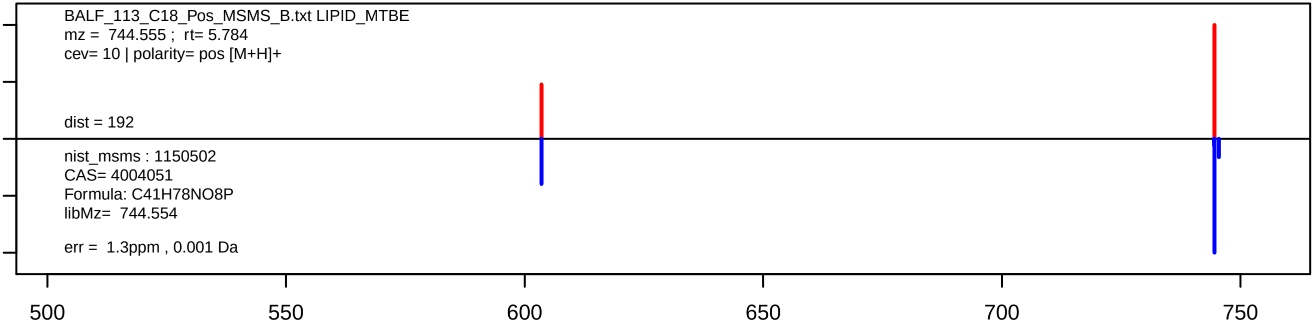

58 . 1,2−Dioleoyl−sn−glycerol
Score=284 Dot=872 prob=64.2

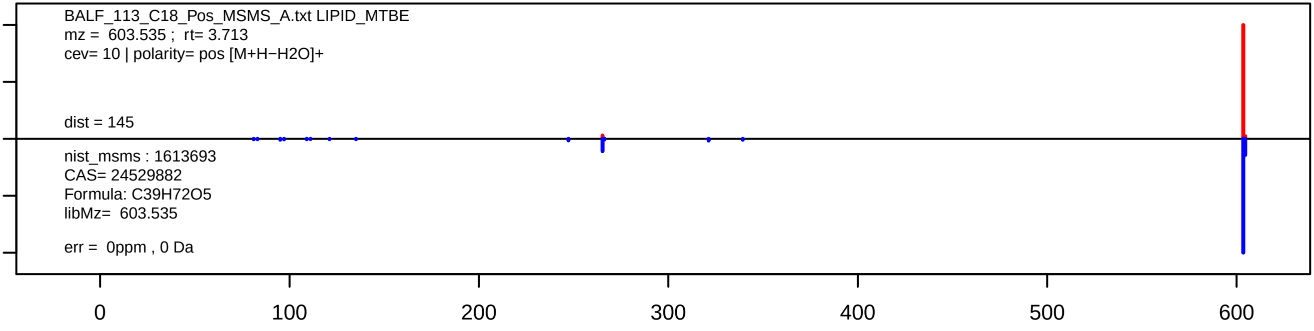

59 . 1,2−Dipalmitoleoyl−sn−glycero−3−phosphocholine
Score=324 Dot=889 prob=57.9

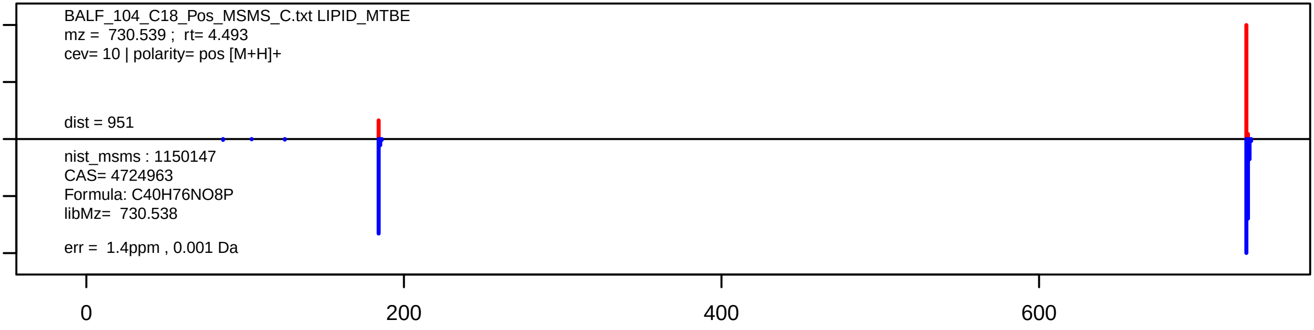

60 . 1,2−Dipalmitoyl−sn−glycero−3−phosphoethanolamine−N−methyl
Score=172 Dot=884 prob=40.4

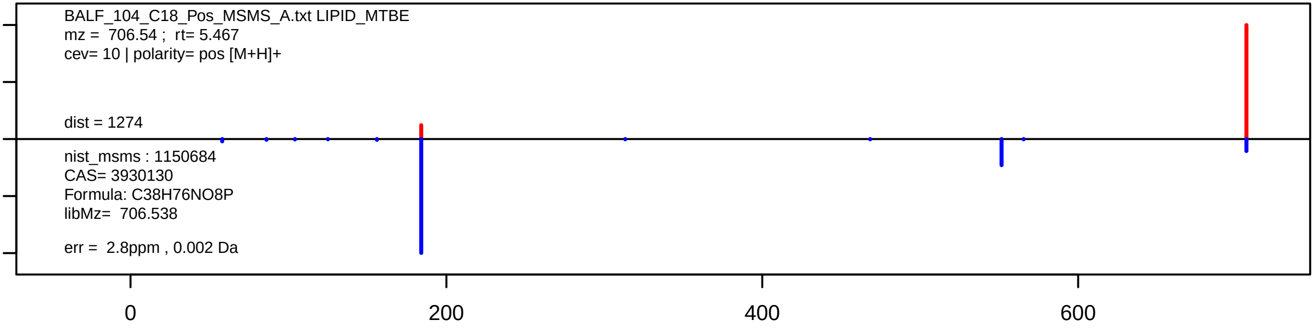

61 . 1,2−Dipalmitoyl−sn−glycero−3−phosphoethanolamine−N,N−dimethyl
Score=237 Dot=843 prob=100

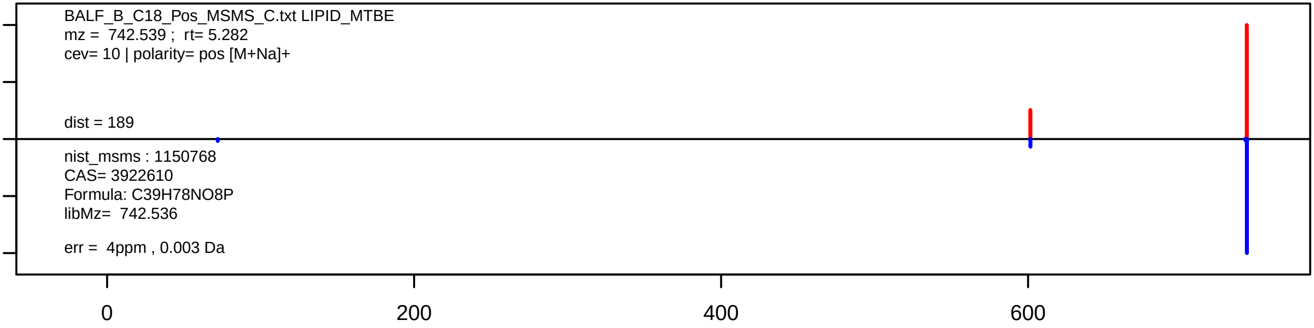

62 . 1,2−Dipalmitoyl−sn−glycero−O−ethyl−3−phosphatidylcholine cation
Score=281 Dot=970 prob=66.4

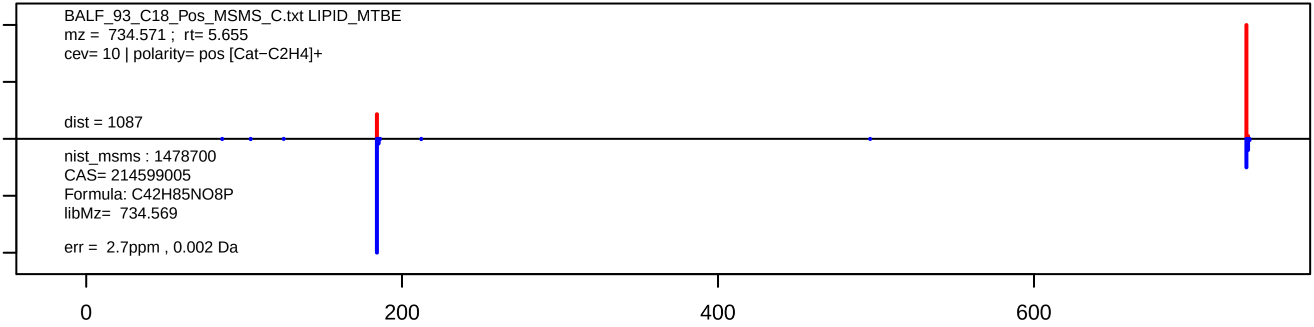

63 . 1,2−Dipentadecanoyl−sn−glycero−3−phosphocholine
Score=475 Dot=955 prob=77.4

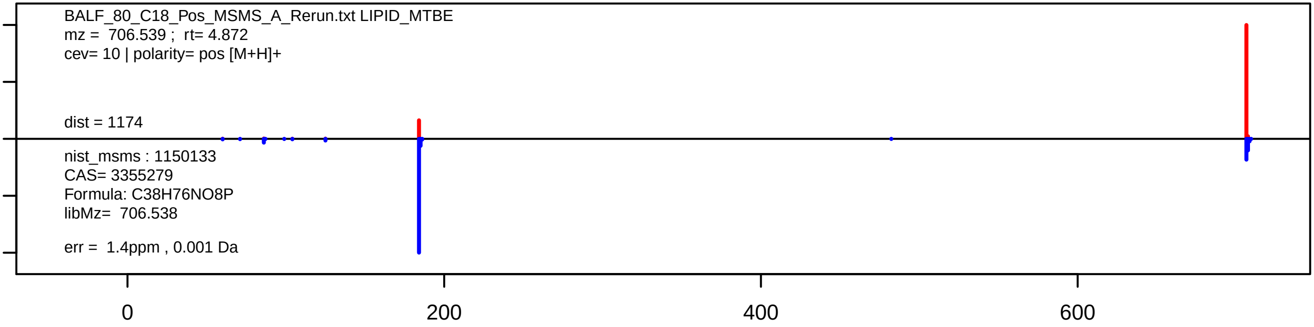

64 . 1,2−Ditetradecanoyl−sn−glycero−3−phosphocholine
Score=191 Dot=954 prob=60

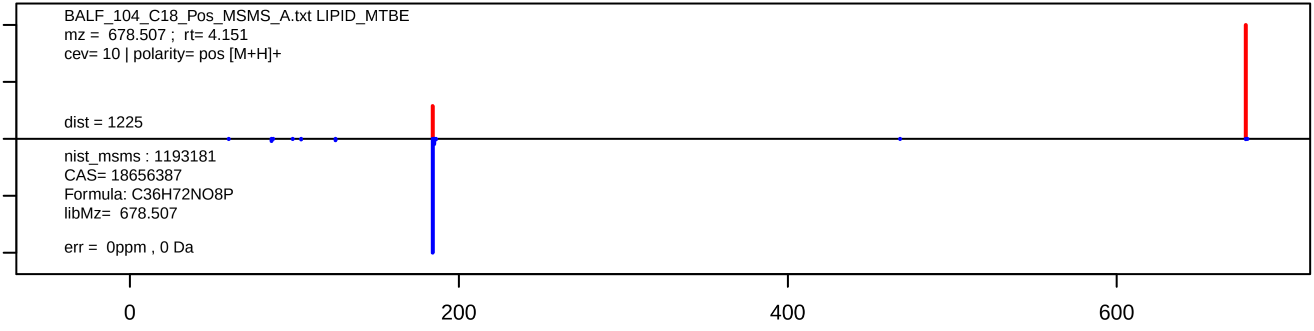

65 . 13−Keto−9Z,11E−octadecadienoic acid
Score=317 Dot=927 prob=91.6

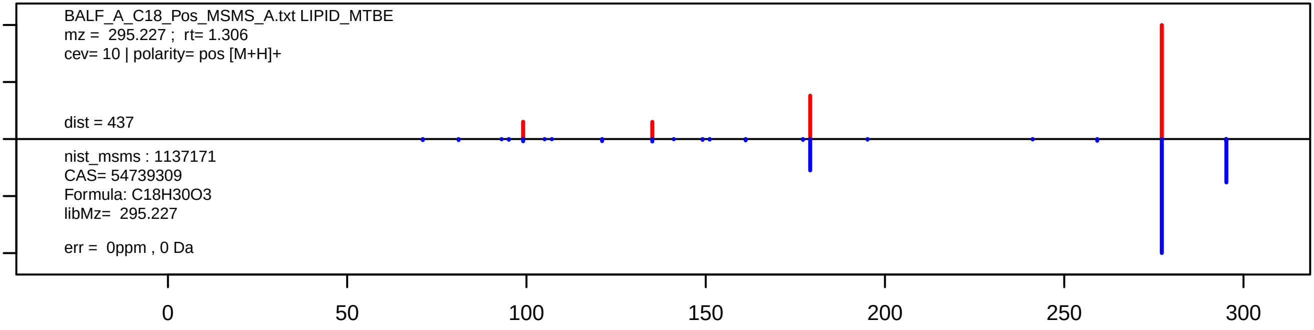

66 . 17−Phenyltrinorprostaglandin A2
Score=254 Dot=849 prob=46.9

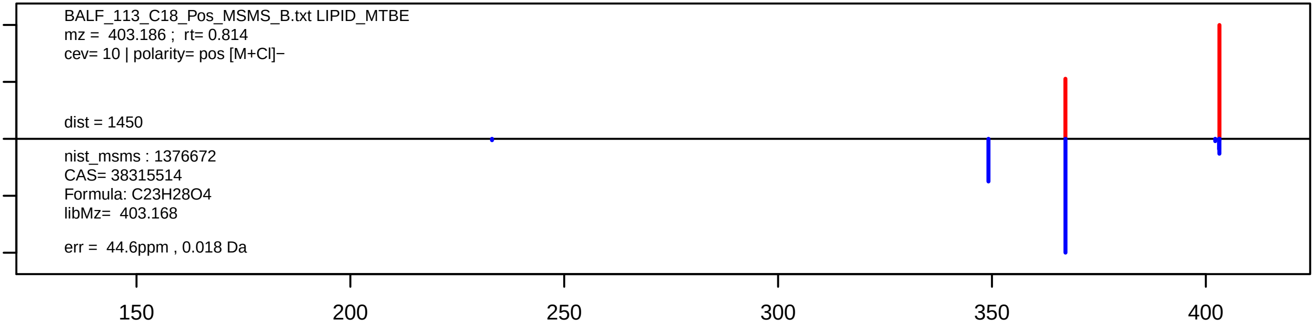

67 . 2−(2H−Benzotriazol−2−yl)−4,6−bis(1−methyl−1−phenylethyl)phenol
Score=355 Dot=996 prob=99

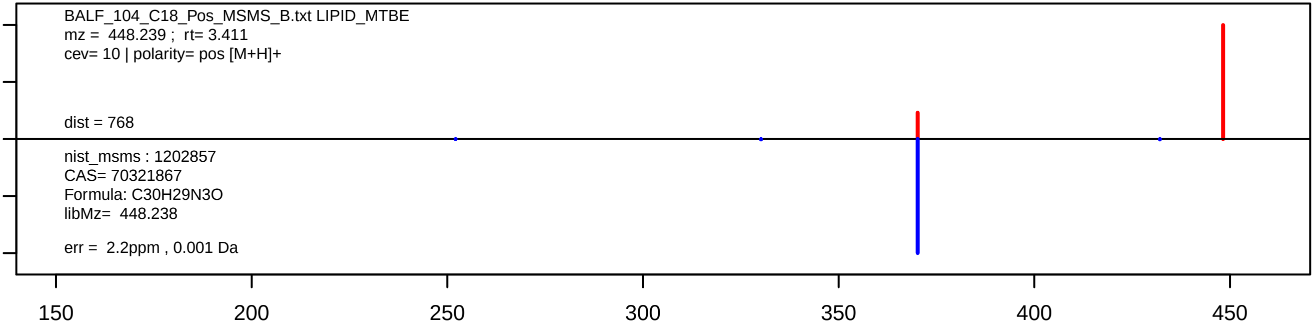

68 . 2−[(2,6−Dimethylphenyl)amino]−N,N,N−triethyl−2−oxoethanaminium cation
Score=530 Dot=999 prob=61.5

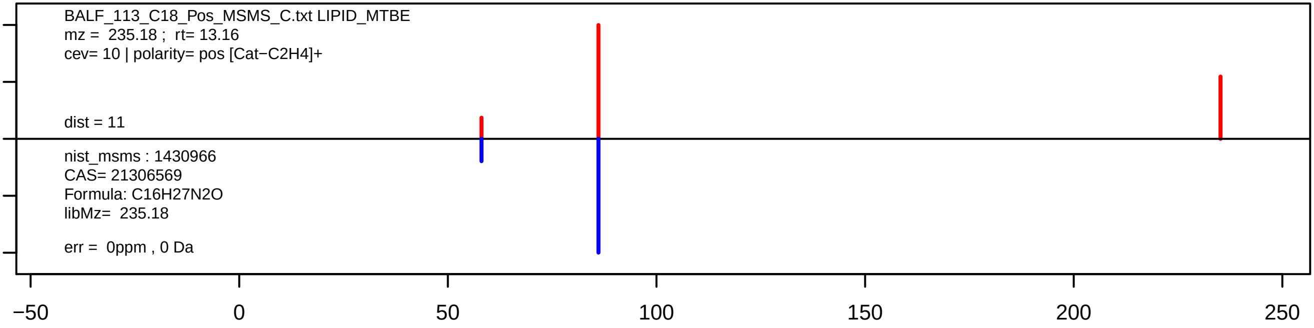

69 . 2−Docosahexaenoyl−1−palmitoyl−sn−glycero−3−phosphoethanolamine
Score=358 Dot=996 prob=1.6

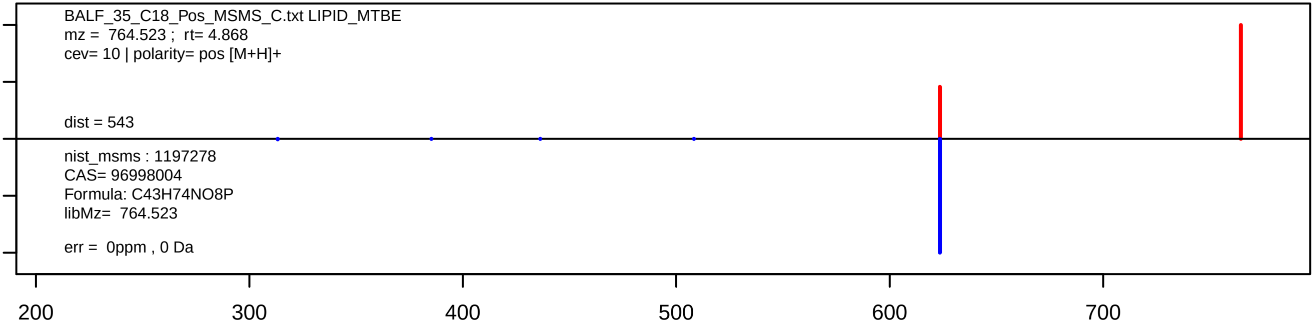

70 . 2−Ethylcyclohexanol
Score=682 Dot=993 prob=40.8

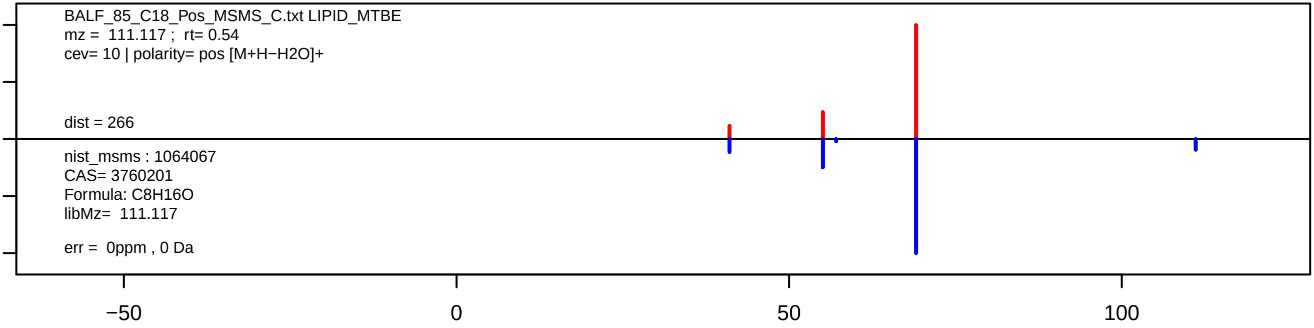

71 . 2−Hexadecanoylthio−1−ethylphosphorylcholine
Score=400 Dot=999 prob=97

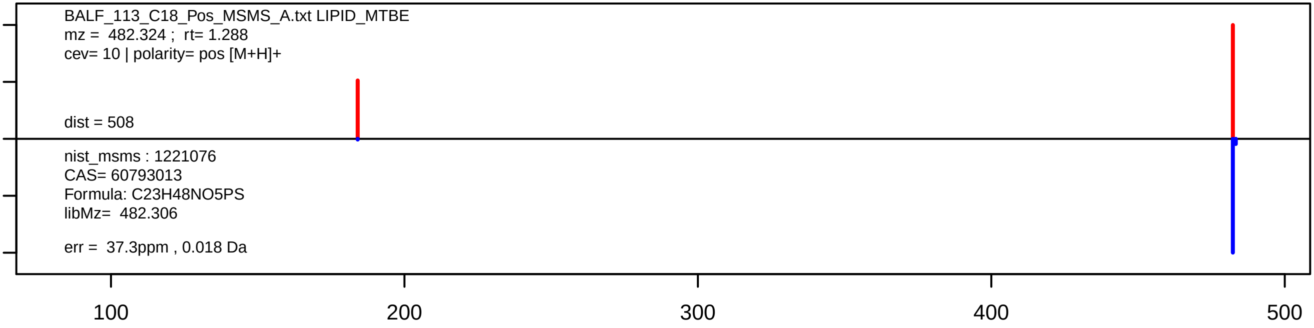

72 . 2−Linoleoyl−1−palmitoyl−sn−glycero−3−phosphoethanolamine
Score=369 Dot=992 prob=98

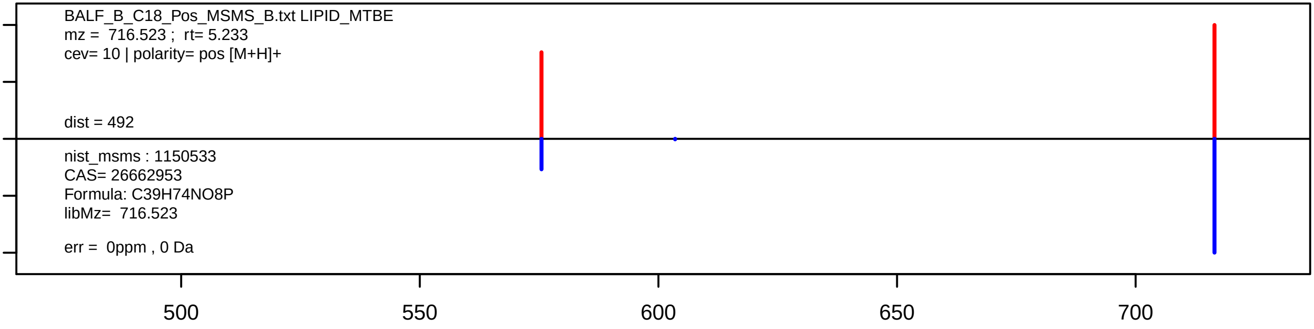

73 . 2−Oleoyl−1−palmitoyl−sn−glycero−3−phosphocholine
Score=400 Dot=999 prob=96.6

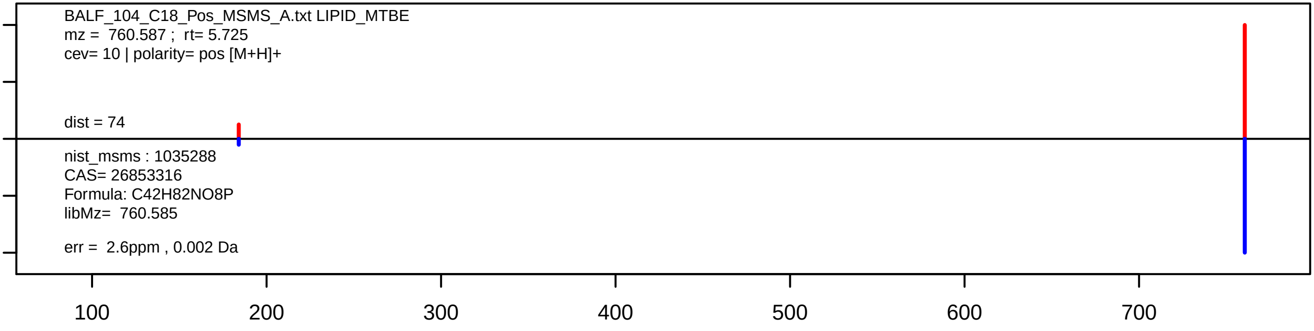

74 . 2−Oleoyl−1−stearoyl−sn−glycero−3−phosphoserine
Score=400 Dot=999 prob=70.9

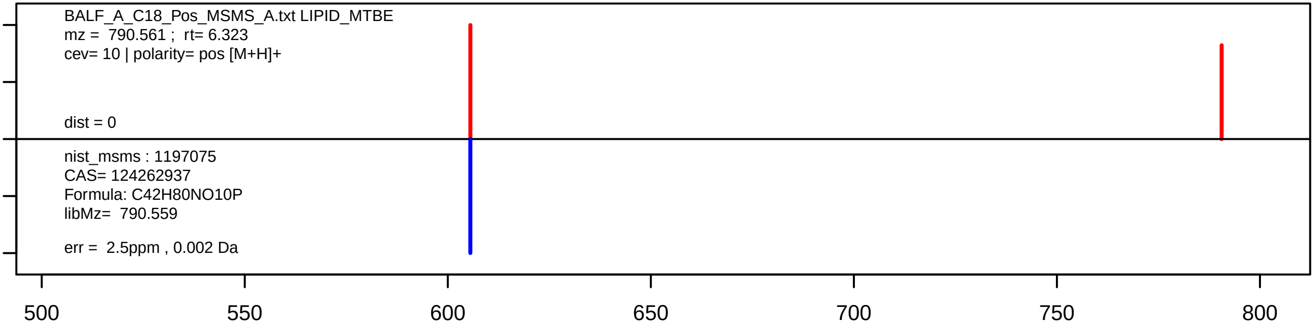

75 . 2−Penten−1−ol, (Z)−
Score=428 Dot=986 prob=24.7

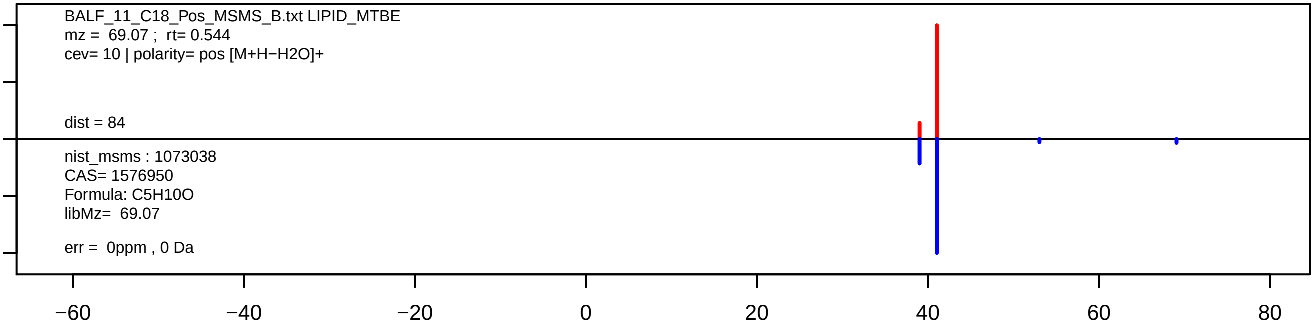

76 . 20−Hydroxy−4Z,7Z,10Z,13Z,16Z,18E−docosahexaenoic acid
Score=388 Dot=998 prob=78.9

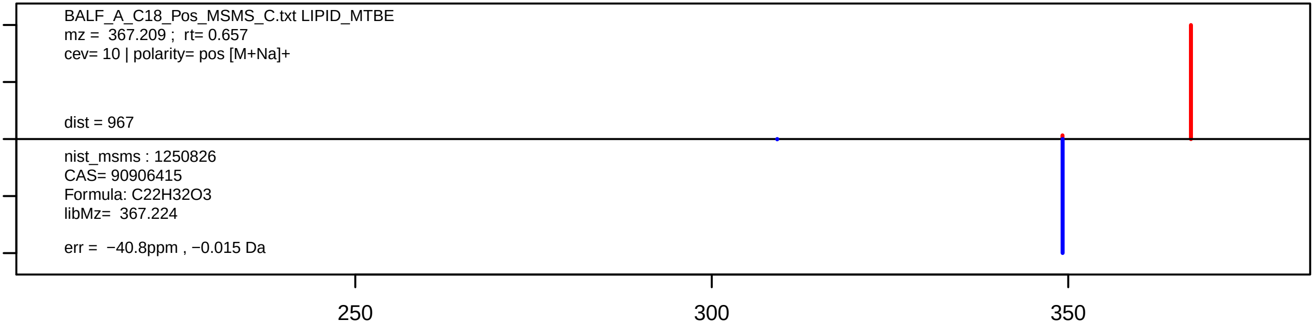

77 . 2S−Amino−4E−octadecene−1,3S−diol
Score=363 Dot=972 prob=82.3

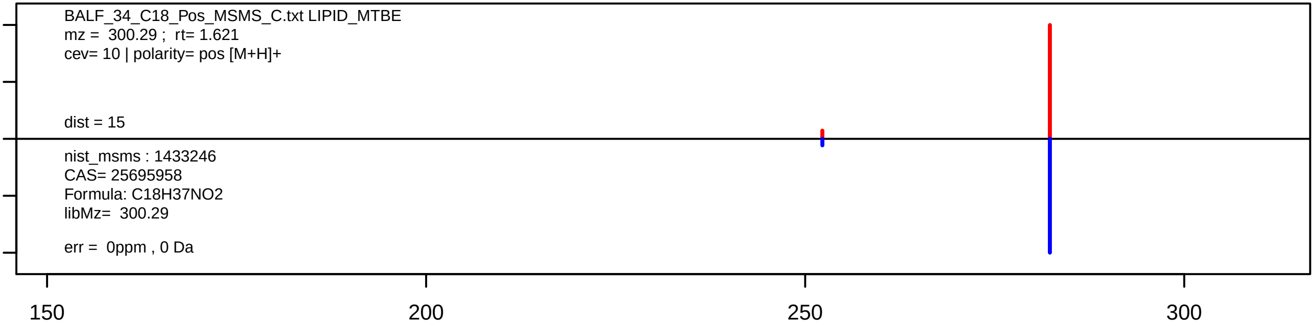

78 . 3−Hydroxy−2',4',5'−trimethoxyflavone
Score=200 Dot=924 prob=79.2

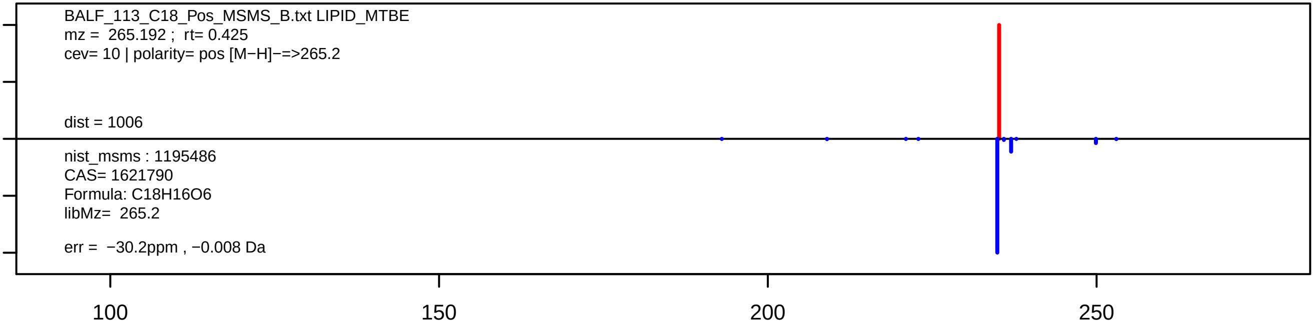

79 . 3,4−Dimethoxymethcathinone
Score=291 Dot=946 prob=63.8

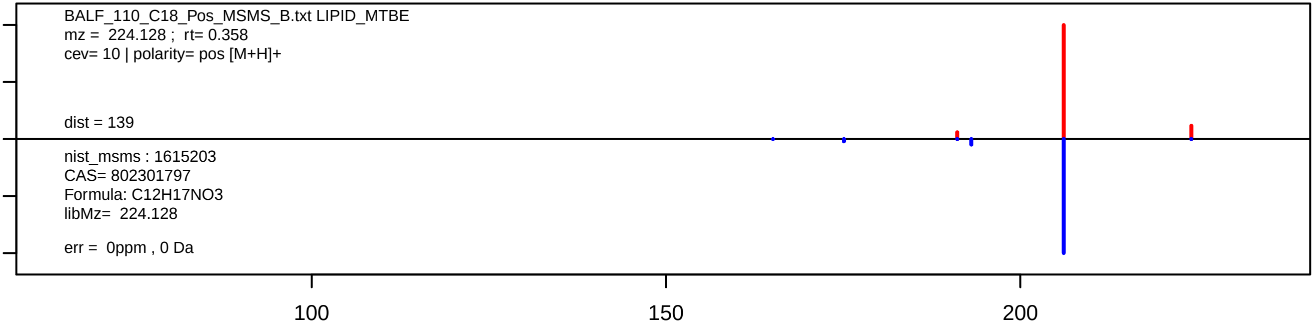

80 . 4−(Butylamino)benzoic acid
Score=368 Dot=836 prob=82.2

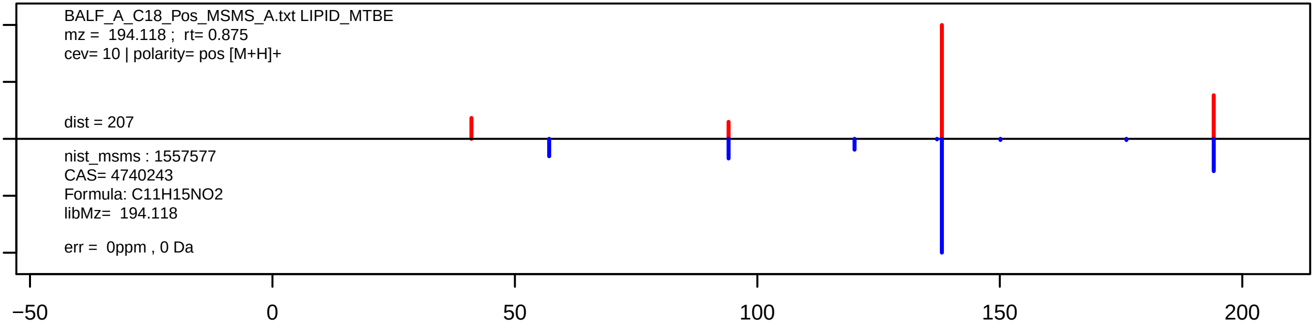

81 . 4−Cholestenone
Score=400 Dot=999 prob=66.6

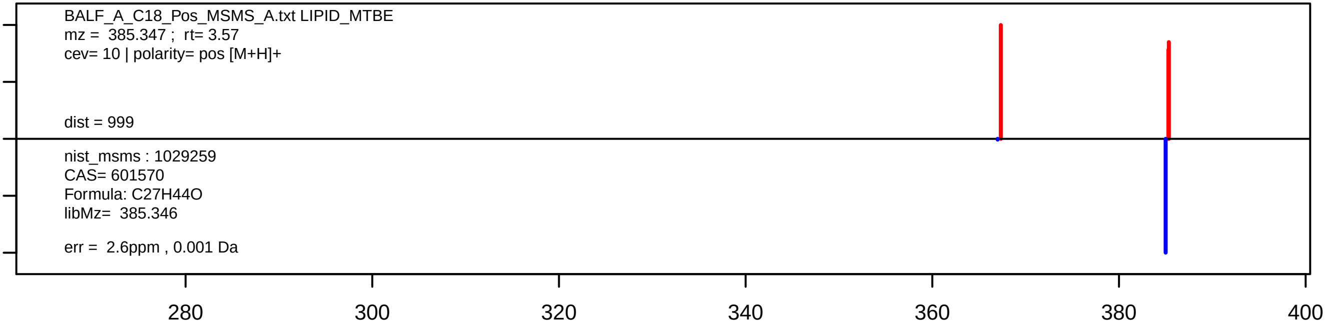

82 . 4−Methoxy−N,N−dimethyltryptamine
Score=280 Dot=847 prob=89.8

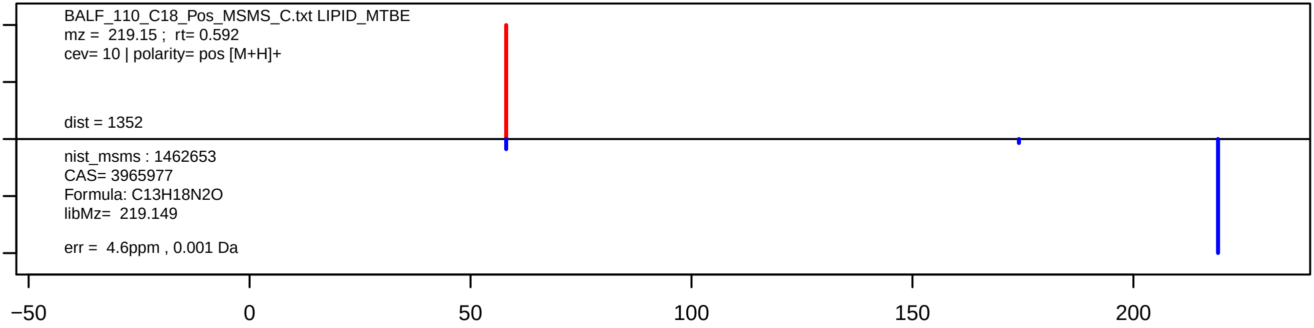

83 . 4−Penten−1−ol
Score=554 Dot=993 prob=27.9

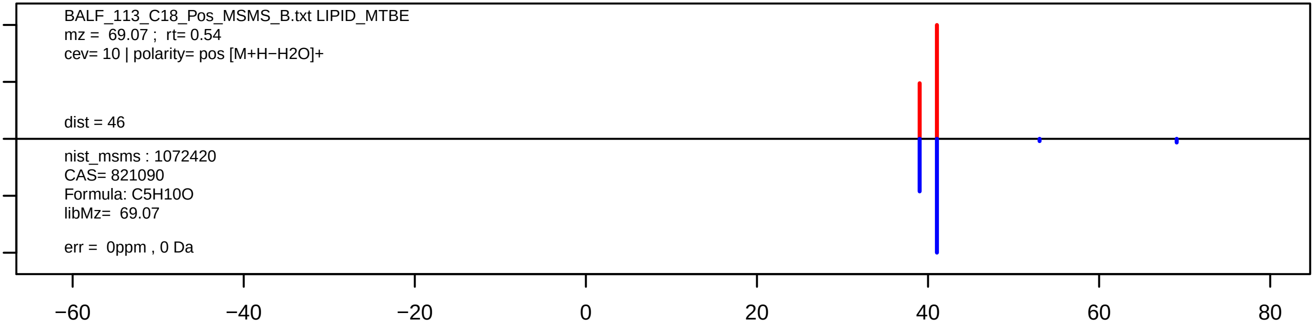

84 . 4−Penten−1−one, 3,3,4−trimethyl−1−[1−[(tetrahydro−2H−pyran−4−yl)methyl]−1H−indol−3−yl]−
Score=524 Dot=856 prob=94.7

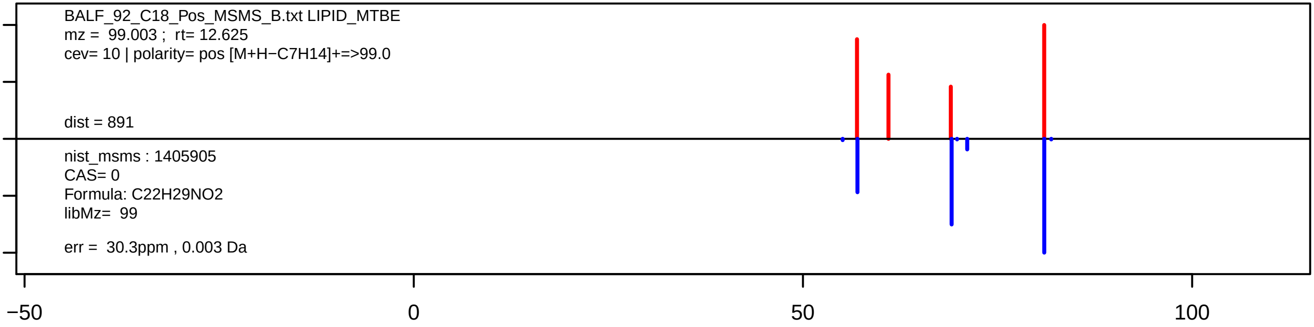

85 . 4−Penten−2−ol
Score=335 Dot=956 prob=37.1

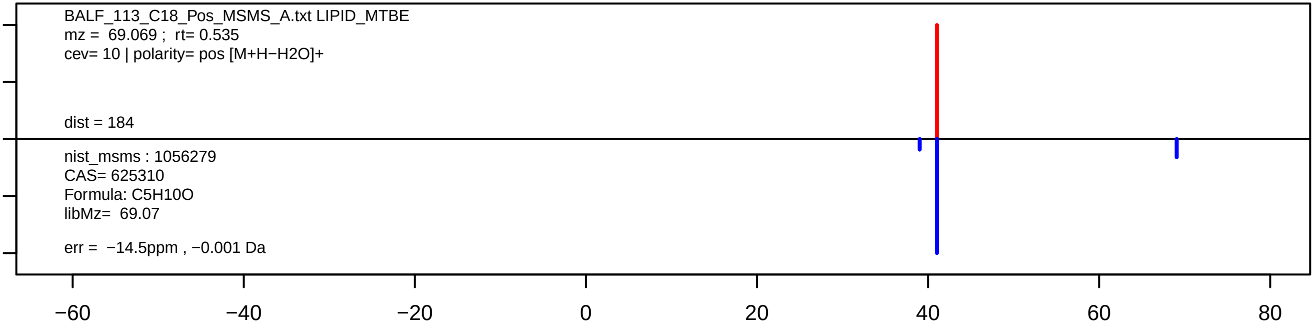

86 . 5,5−Dimethylimidazolidine−2,4−dione
Score=400 Dot=999 prob=3.5

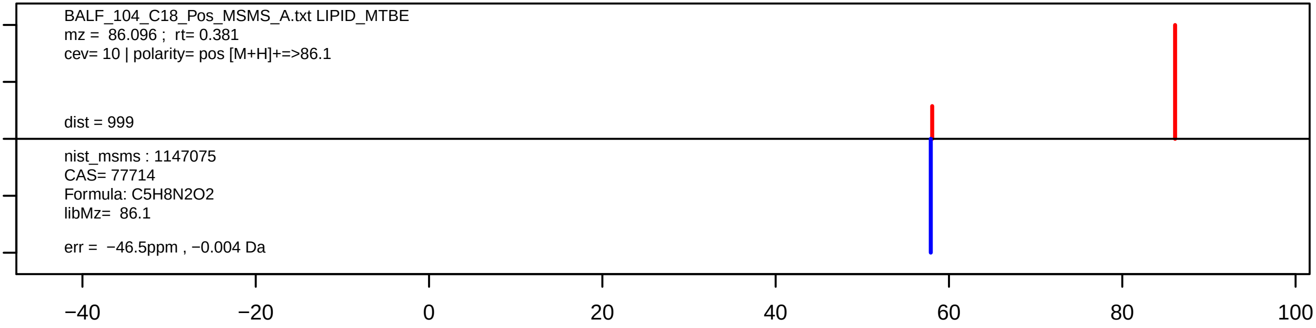

87 . 7.alpha.,12.alpha.−Dihydroxy−5.beta.−cholestan−3−one
Score=327 Dot=942 prob=65.6

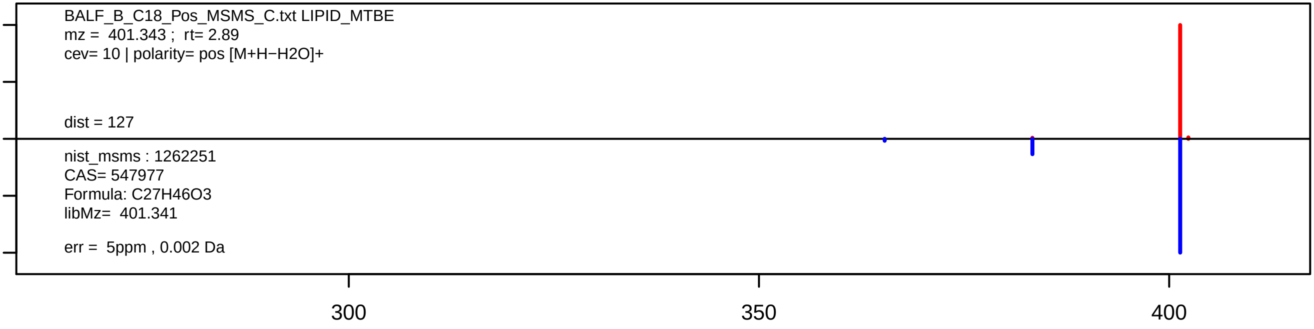

88 . 9−Oxo−10(E),12(E)−octadecadienoic acid
Score=282 Dot=880 prob=29.6

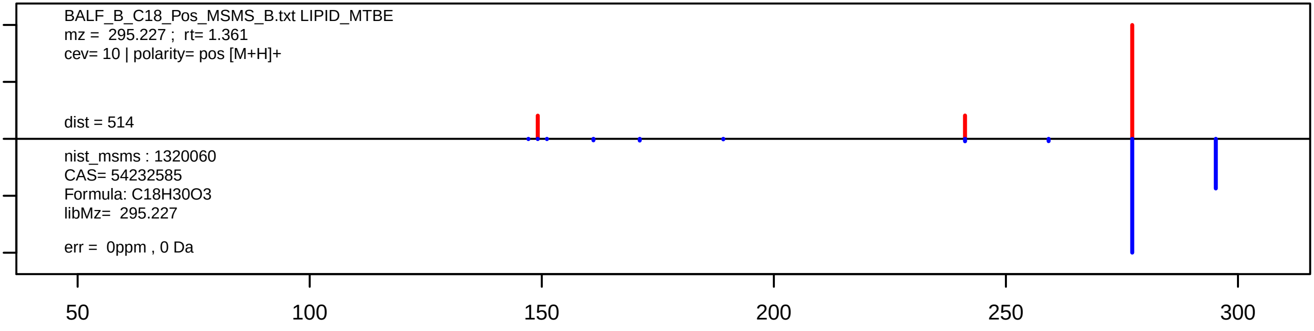

89 . Aloin
Score=357 Dot=992 prob=100

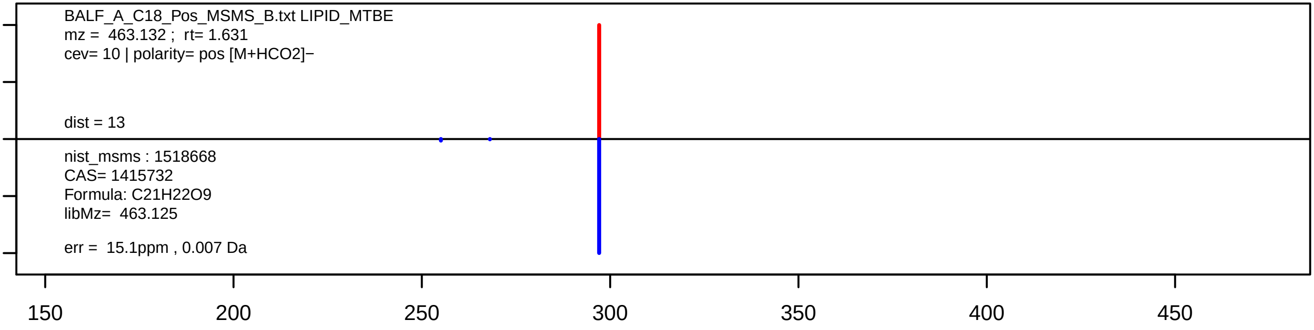

90 . Amphetamine
Score=560 Dot=999 prob=19.7

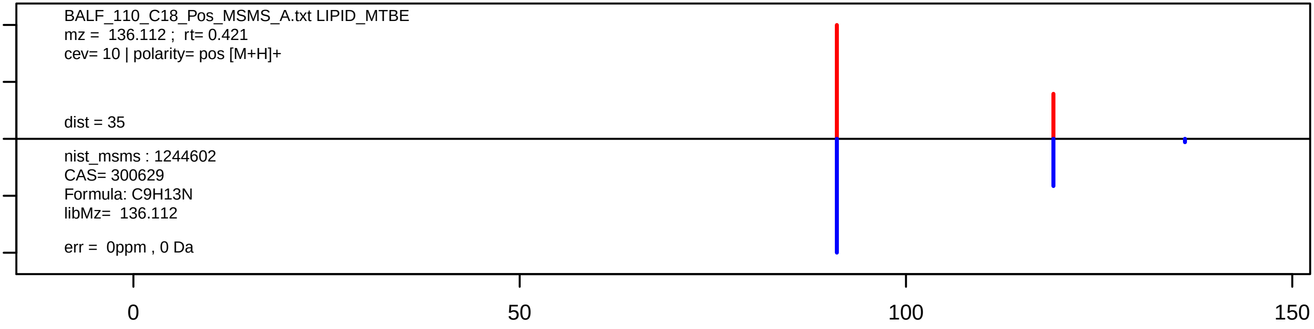

91 . Arachidonoylthiophosphorylcholine
Score=268 Dot=899 prob=83.3

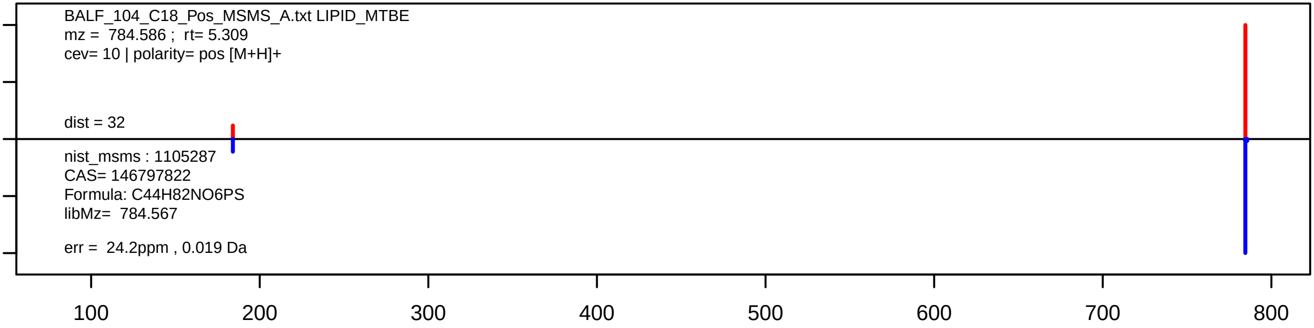

92 . Benzhydrol
Score=792 Dot=982 prob=43.2

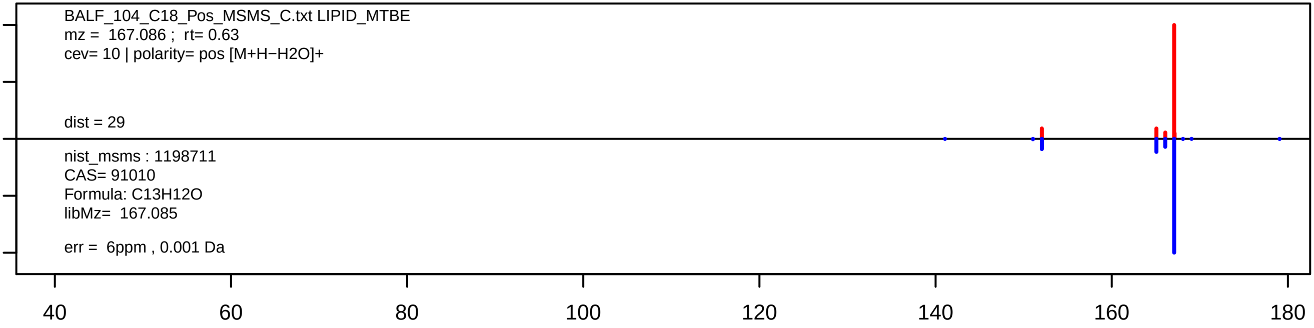

93 . Benzocaine
Score=628 Dot=935 prob=94

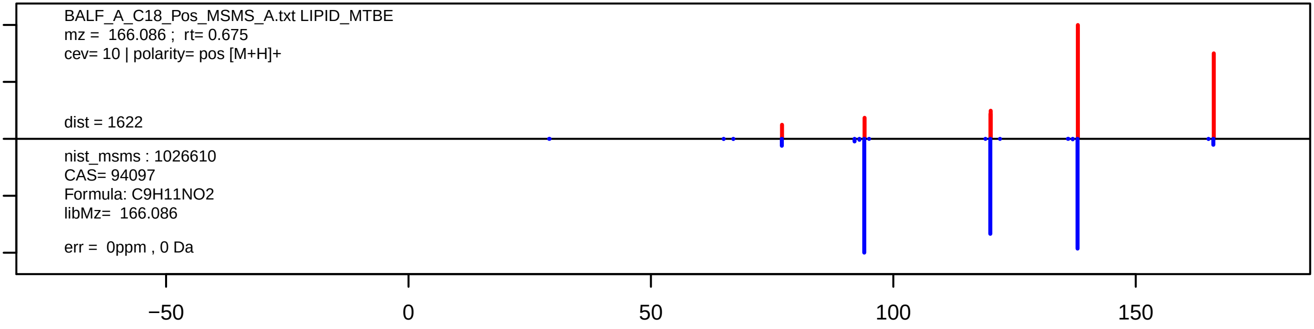

94 . Benzyl alcohol
Score=400 Dot=999 prob=1.5

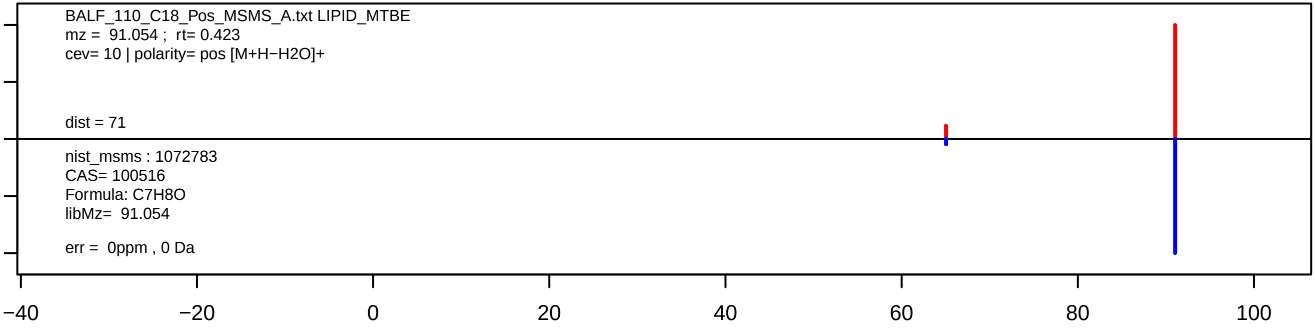

95 . Benzyldimethyltetradecylammonium cation
Score=381 Dot=998 prob=98.3

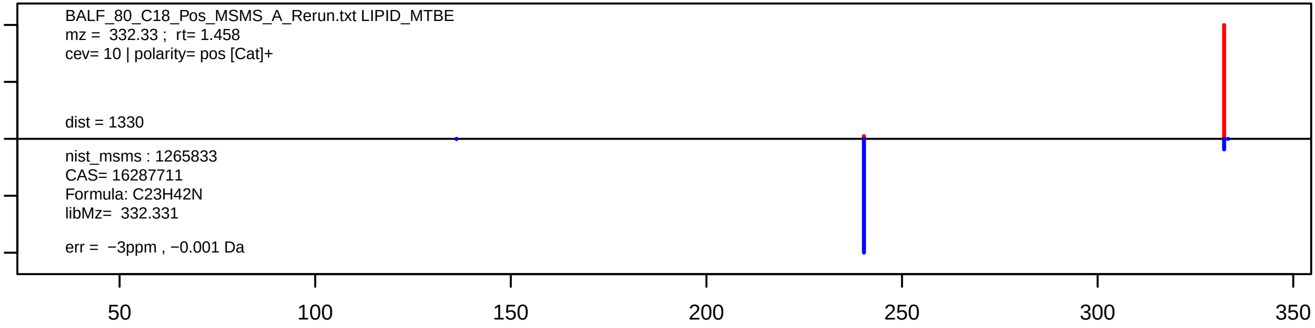

96 . Benzyldodecyldimethylammonium cation
Score=349 Dot=881 prob=86.5

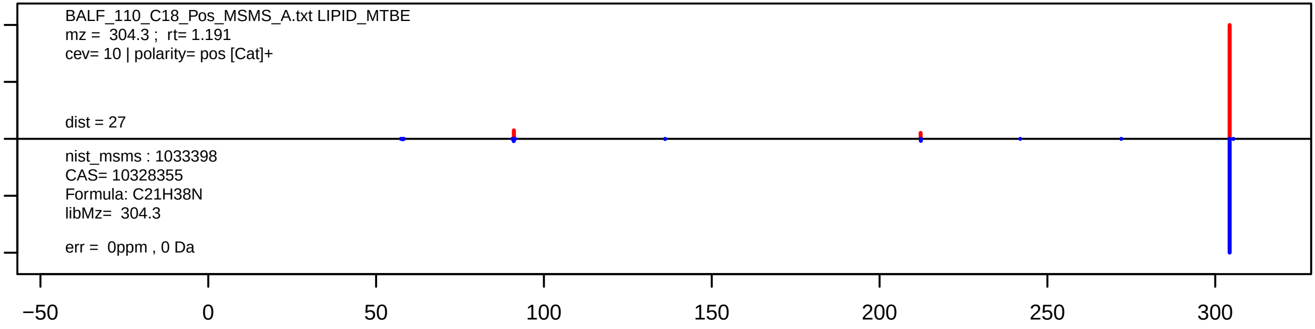

97 . Betaine
Score=552 Dot=998 prob=98.1

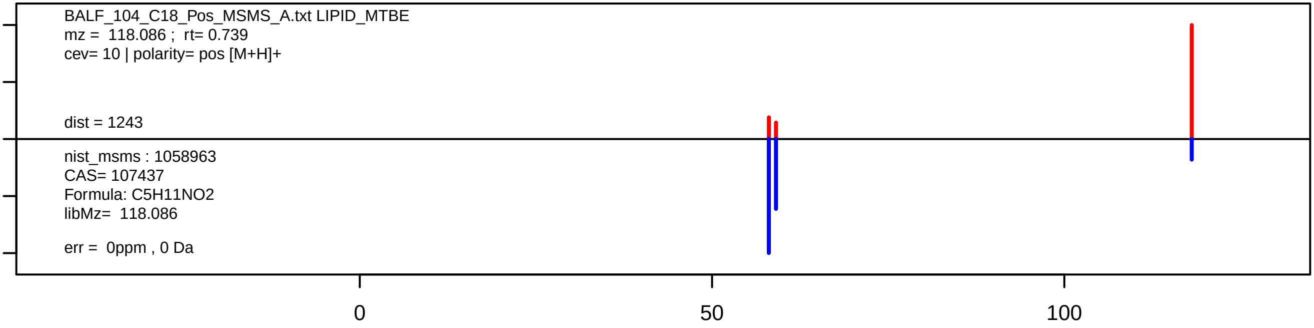

98 . Betaine aldehyde cation
Score=314 Dot=883 prob=45

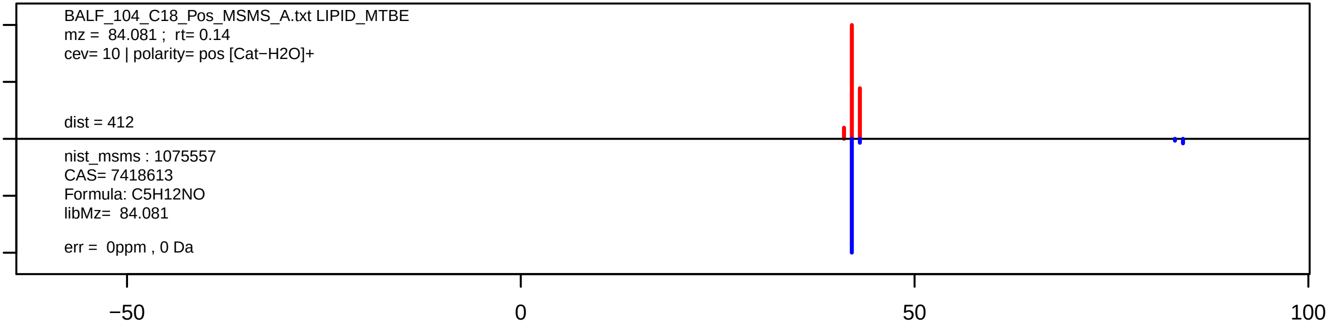

99 . Bis(2−ethylhexyl) adipate
Score=851 Dot=932 prob=97.6

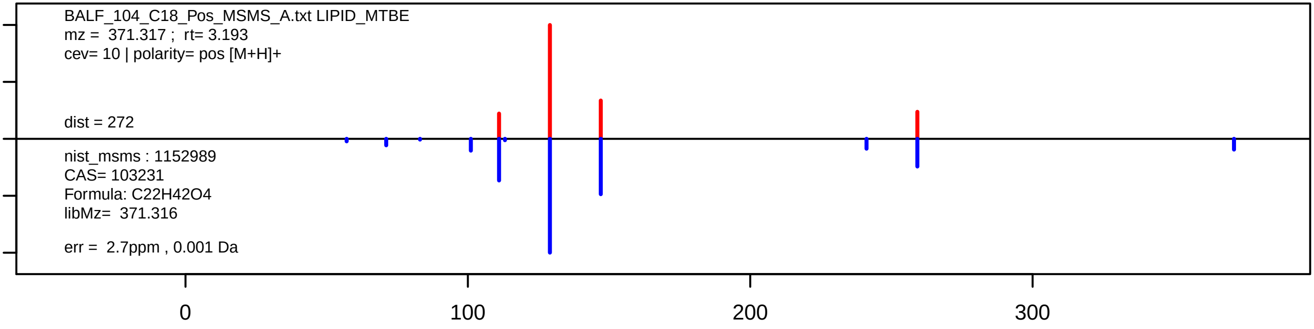

100 .   Bitertanol
Score=400 Dot=999 prob=32.3

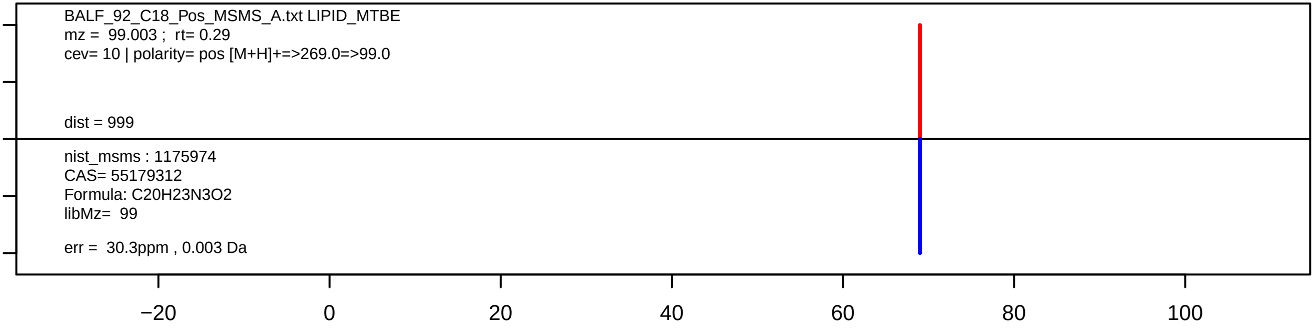

101 .   Bufexamac
Score=400 Dot=999 prob=19.6

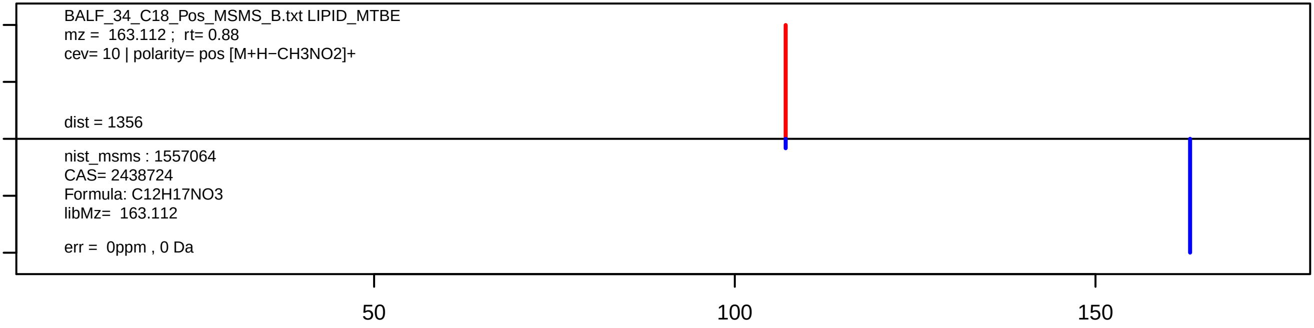

102 .   Bupropion
Score=905 Dot=991 prob=98.6

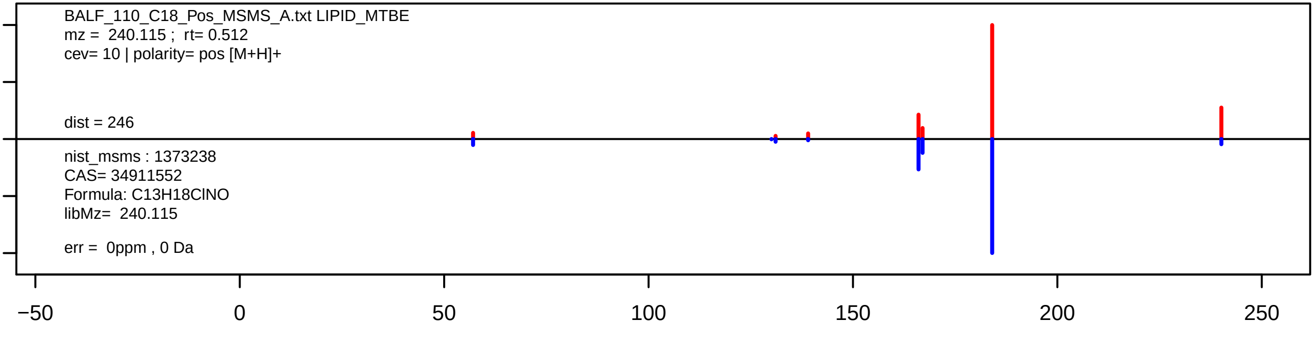

103 . Choline cation
Score=542 Dot=986 prob=96.9

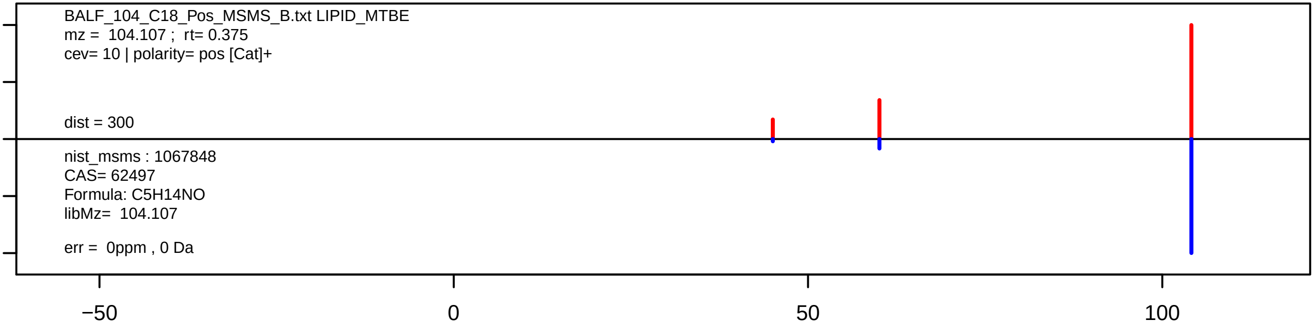

104 . Citalopram
Score=244 Dot=854 prob=98.5

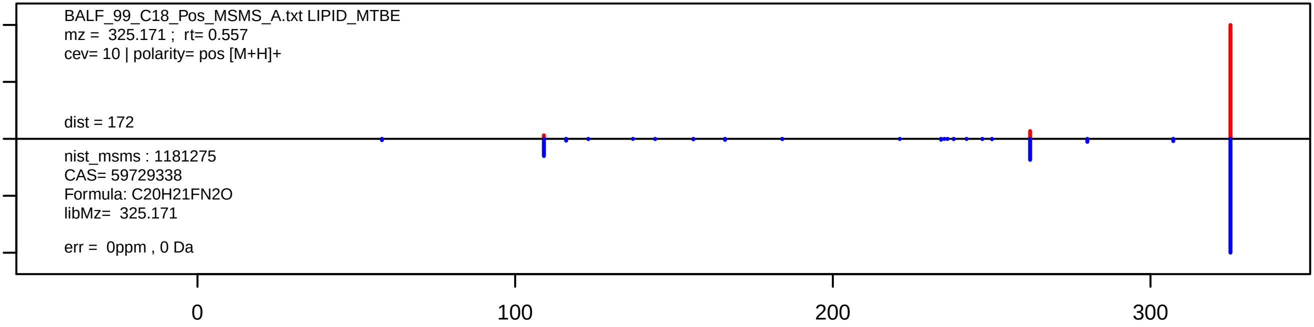

105 . Cyclizine
Score=825 Dot=985 prob=79.2

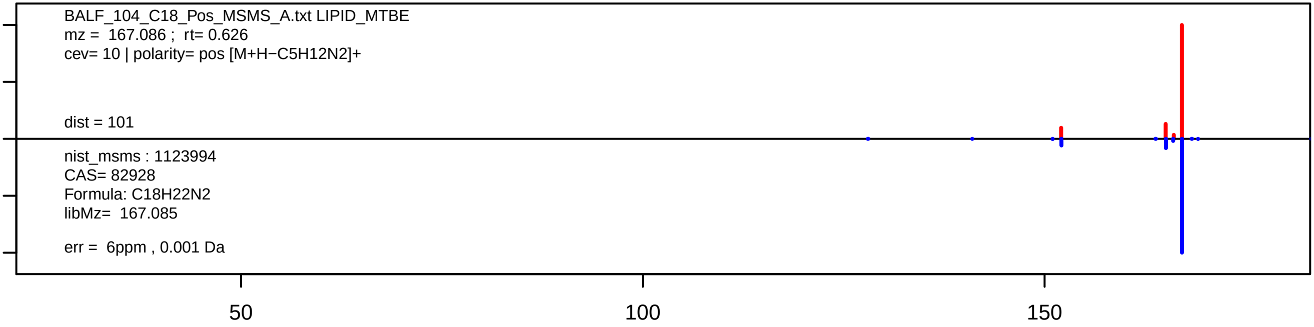

106 . D−erythro−N−stearoylsphingosine
Score=233 Dot=881 prob=89.3

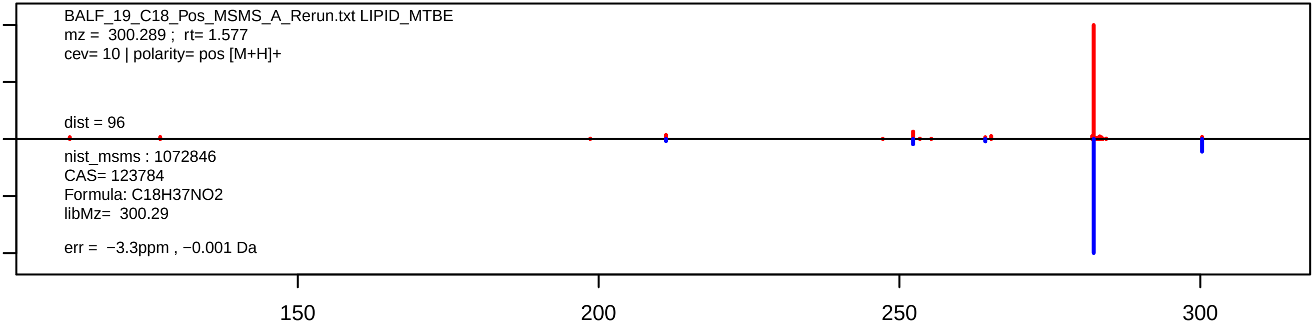

107 . D−erythro−Sphinganine
Score=593 Dot=955 prob=98.7

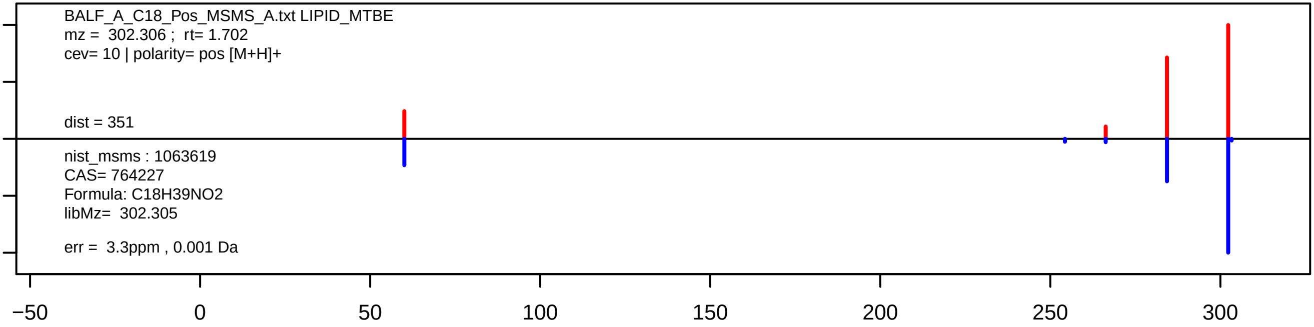

108 . Decamethylcyclopentasiloxane
Score=742 Dot=940 prob=98.7

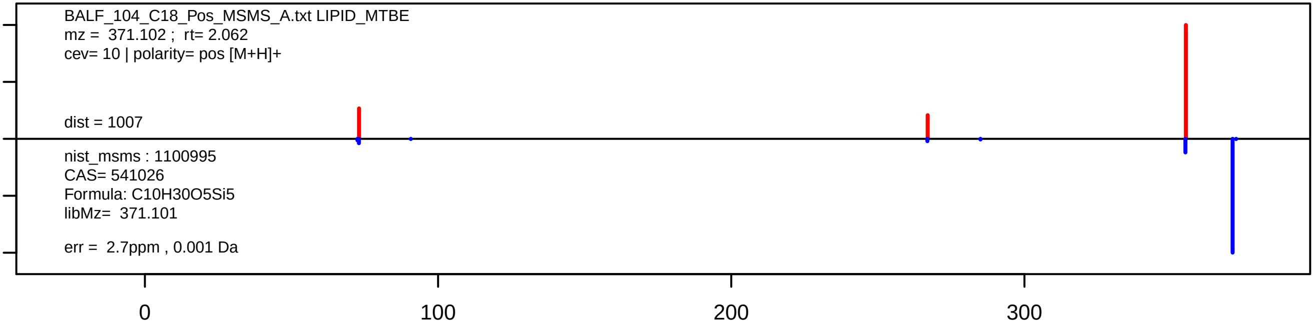

109 . Desethylamiodarone
Score=342 Dot=994 prob=100

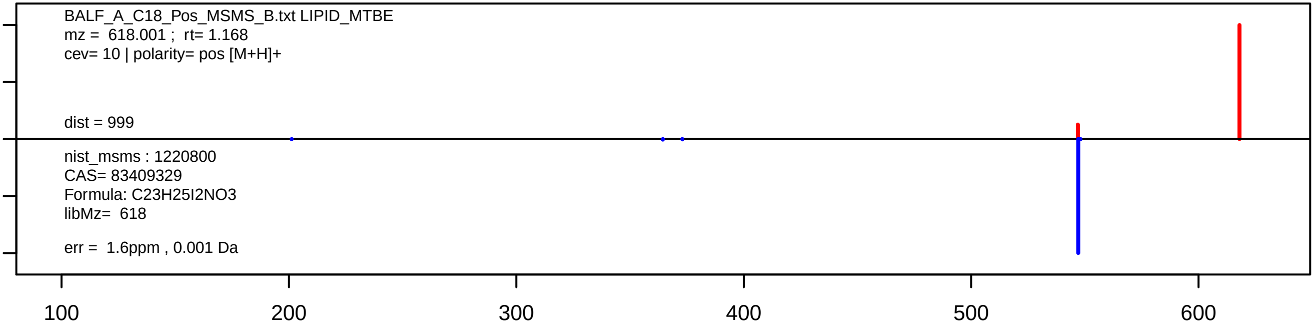

110 . DG 36:2; [M+NH4]+; DG(18:0/18:2/0:0)
Score=658 Dot=878 prob=49.6

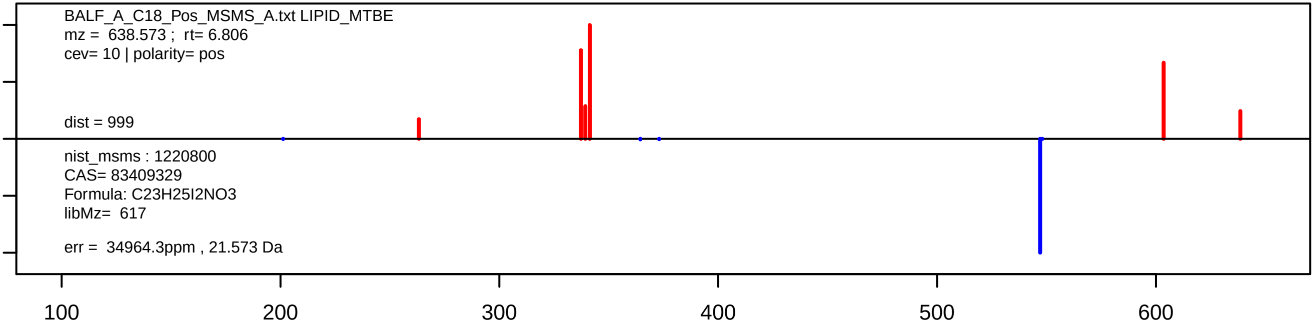

111 . Dibenzylamine
Score=184 Dot=823 prob=75.6

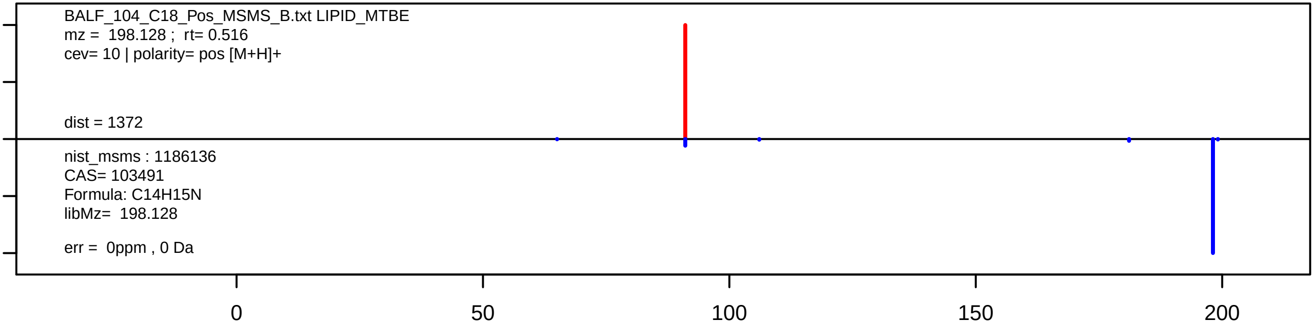

112 . Dibutyl phthalate
Score=243 Dot=859 prob=85.2

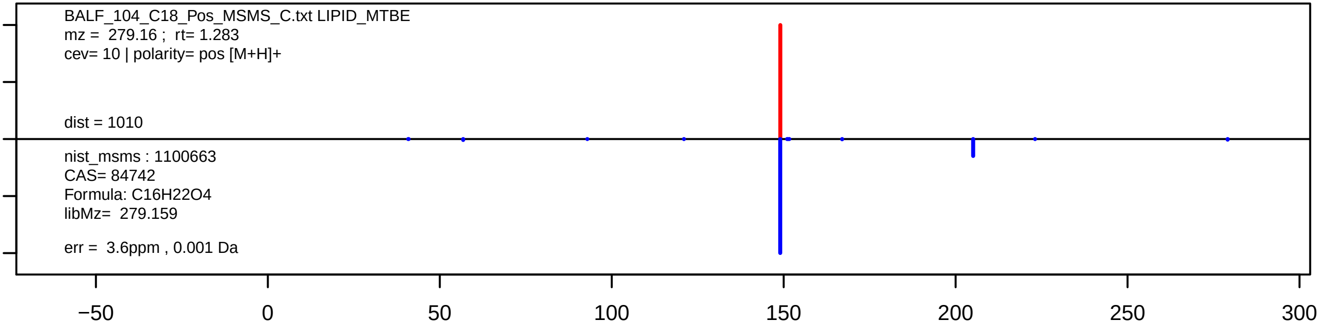

113 . Didodecyl 3,3'−thiodipropionate oxide
Score=352 Dot=985 prob=99

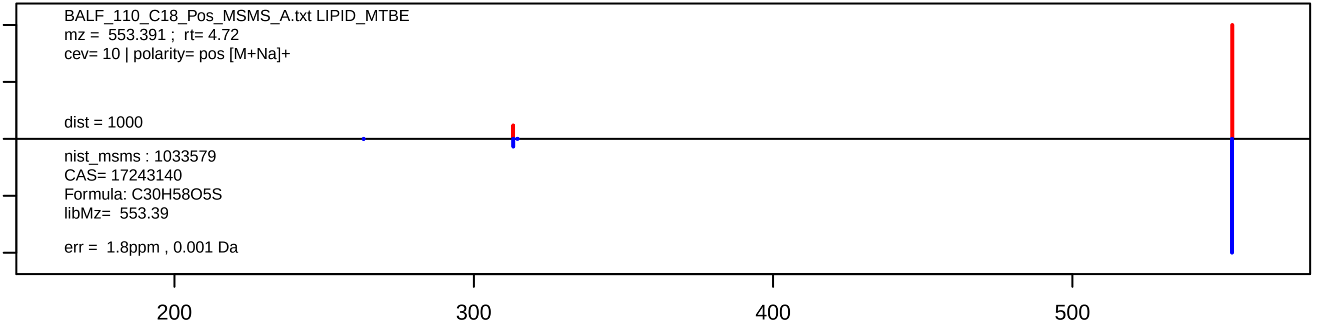

114 . Diisooctyl phthalate
Score=259 Dot=885 prob=46.1

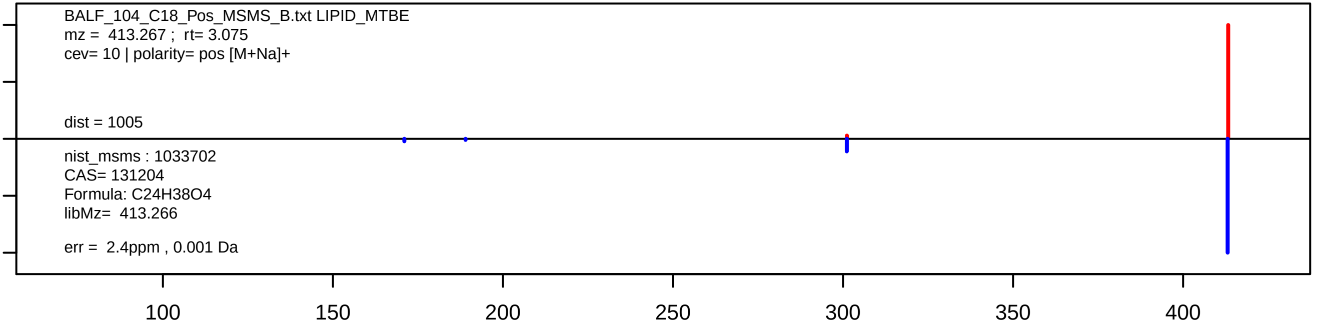

115 . Dimethyldioctadecylammonium cation
Score=127 Dot=805 prob=69.6

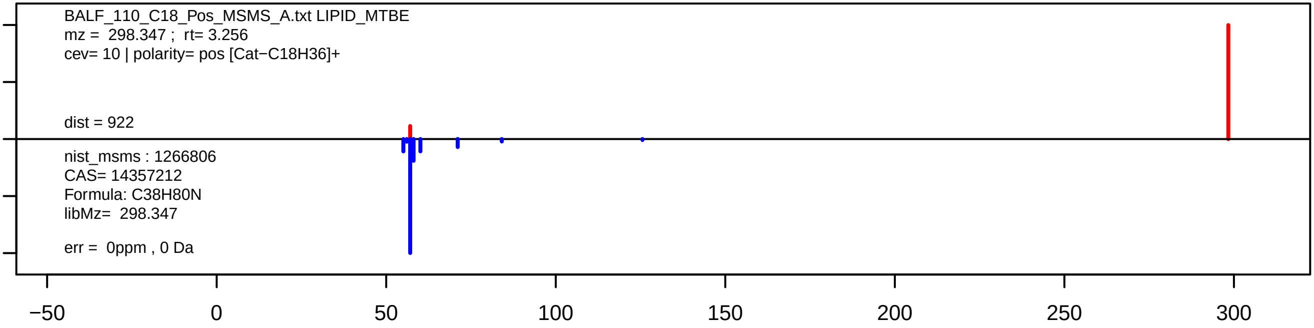

116 . Dioctyl phthalate
Score=853 Dot=879 prob=80.8

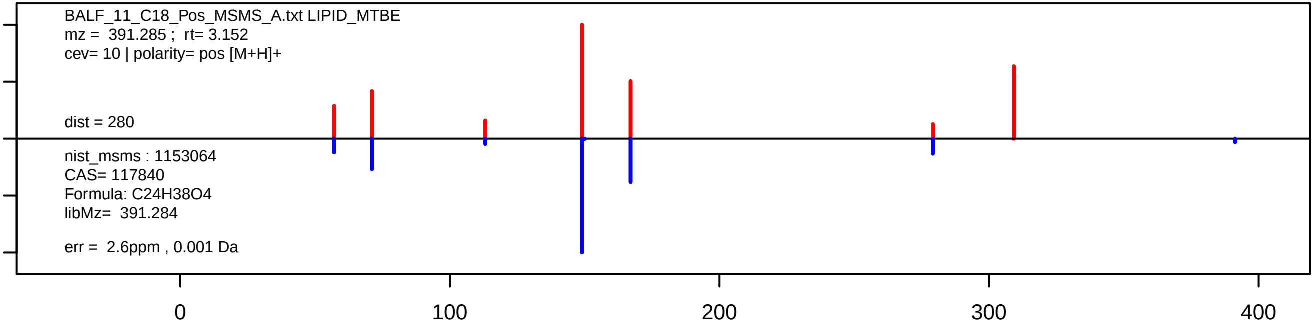

117 . Diphenhydramine
Score=379 Dot=995 prob=97.8

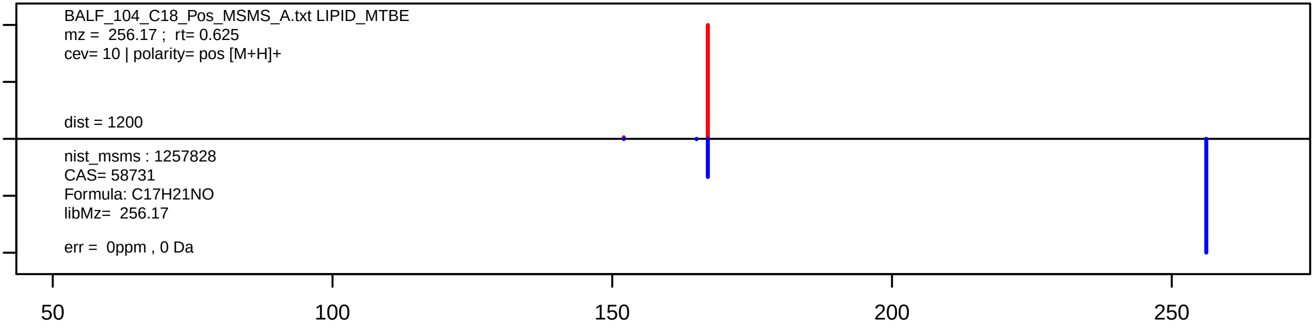

118 . Dodecamethylcyclohexasiloxane
Score=225 Dot=906 prob=90.9

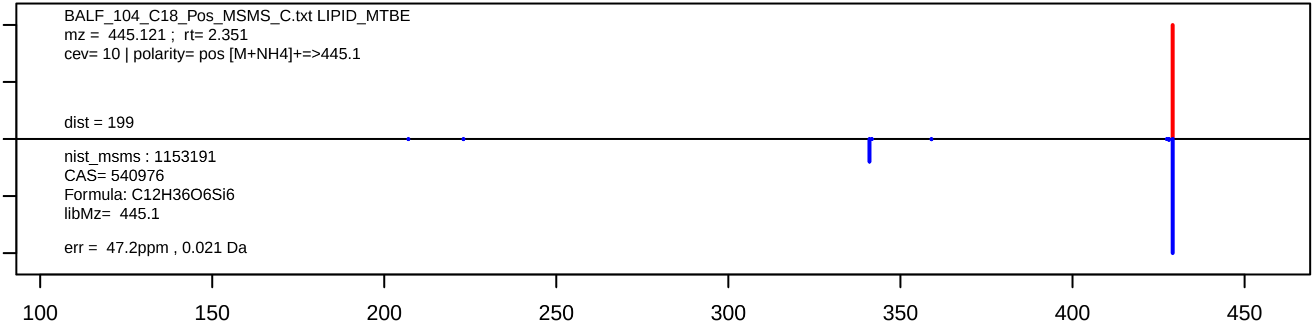

119 . Epitestosterone
Score=579 Dot=806 prob=85.4

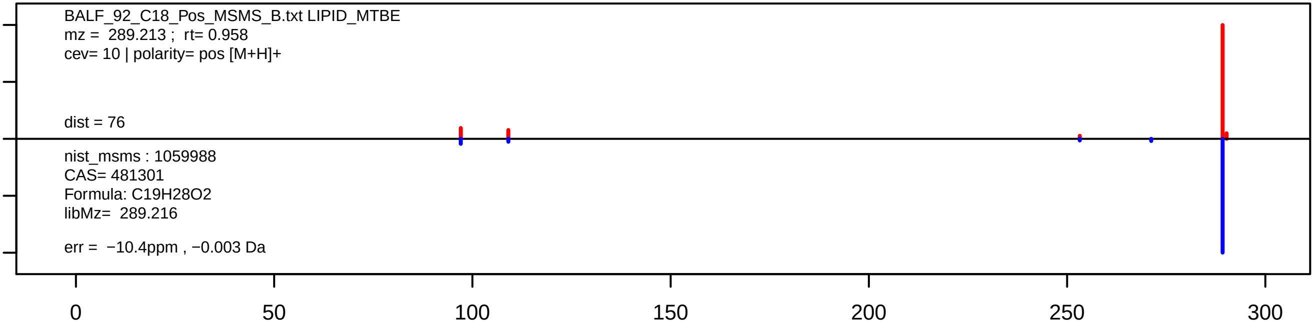

120 . Erucamide
Score=400 Dot=999 prob=96.2

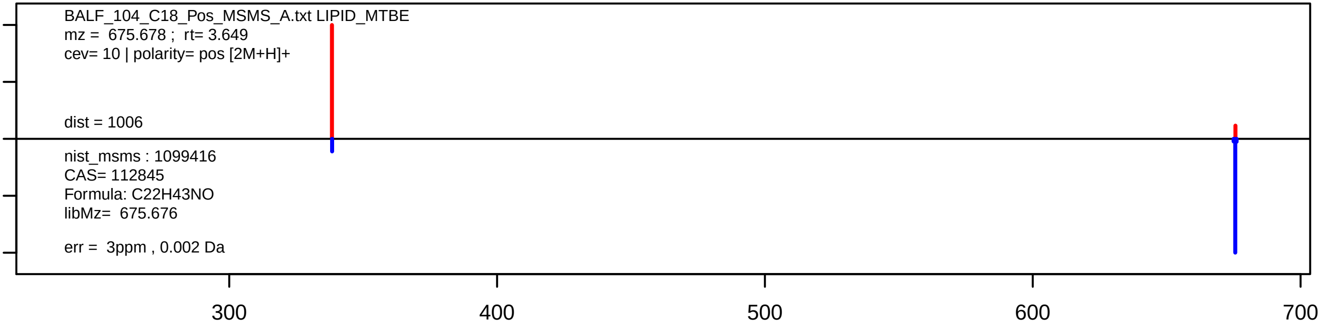

121 . Ethyl tramadol
Score=359 Dot=985 prob=98

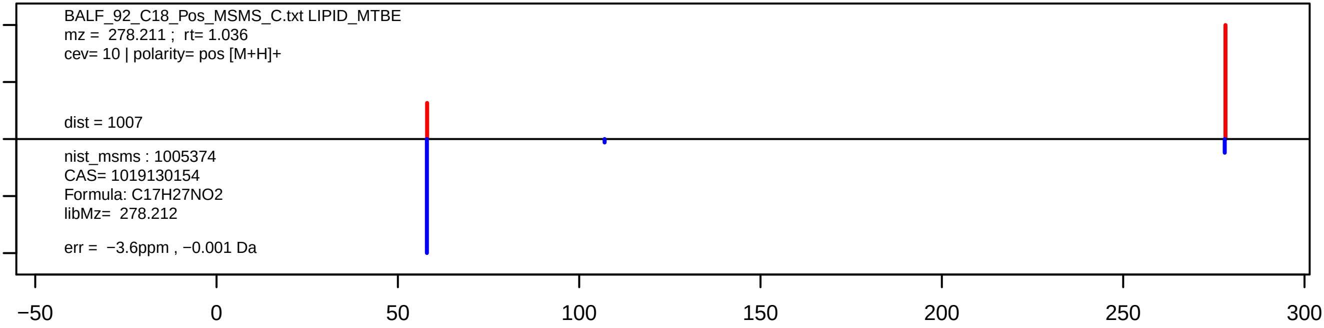

122 . Fluoxetine
Score=327 Dot=917 prob=97.9

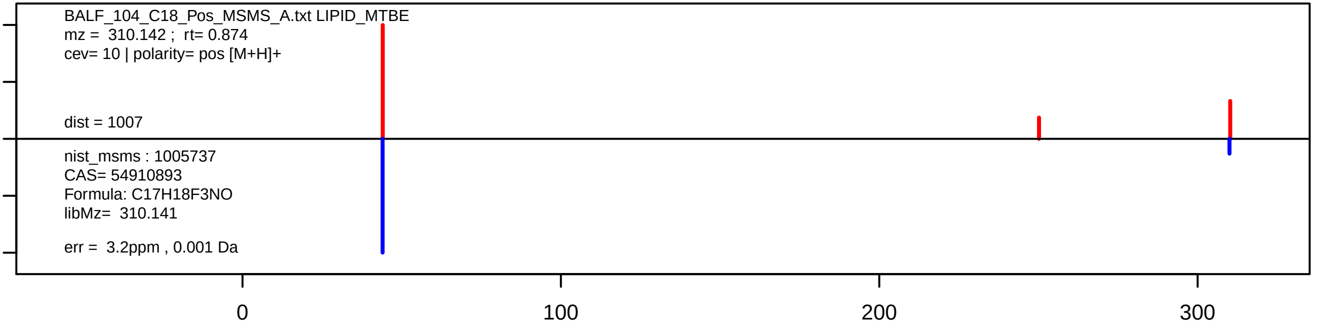

123 . Hemin cation
Score=446 Dot=953 prob=96.5

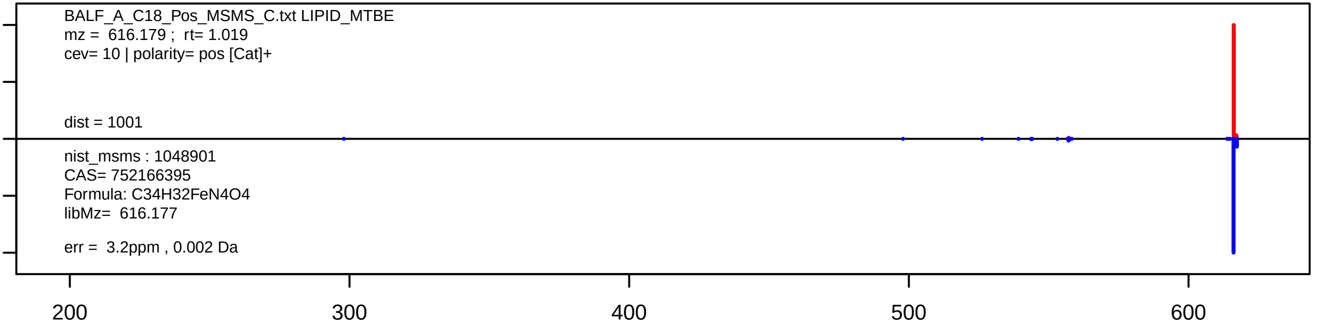

124 . Hexadecyltrimethylammonium cation
Score=232 Dot=883 prob=94.6

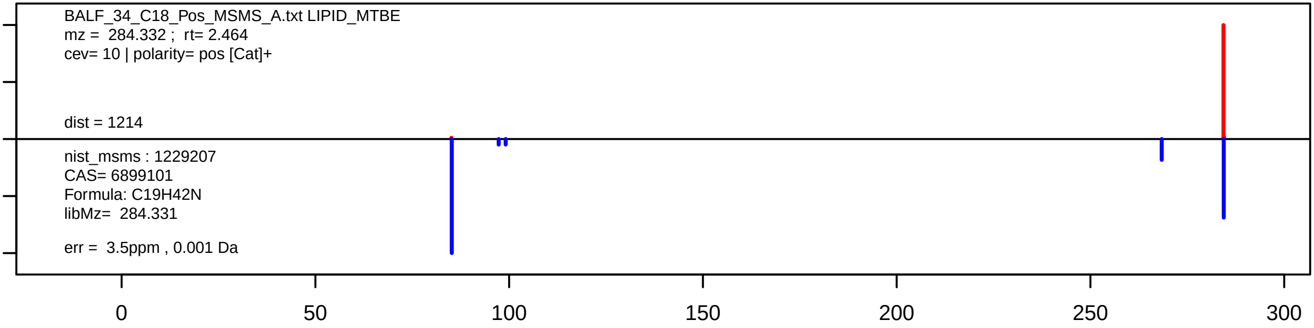

125 . Hydroxybupropion
Score=346 Dot=946 prob=61

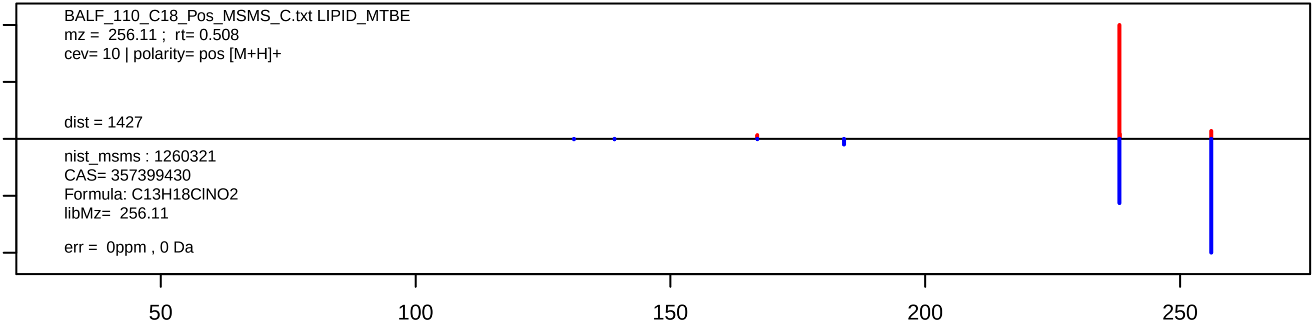

126 . Hydroxyzine
Score=275 Dot=965 prob=95.8

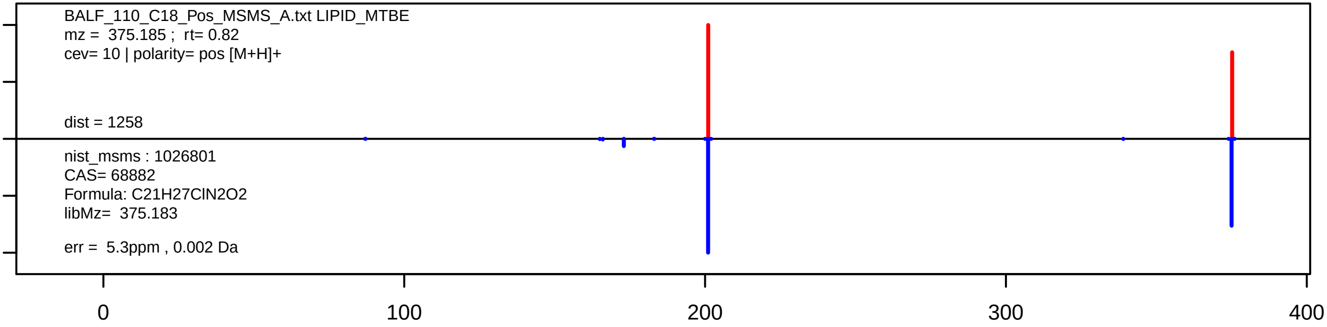

127 .   Imipramine
Score=316 Dot=840 prob=15.4

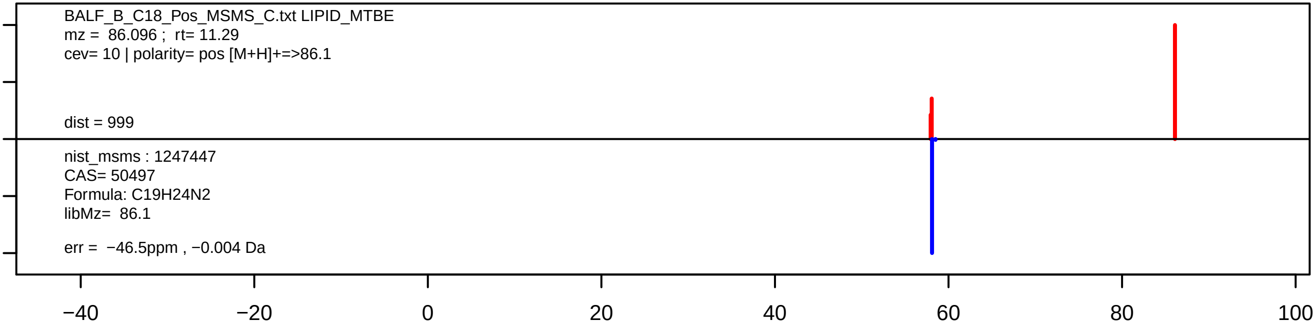

128 .   L−Isoleucine
Score=400 Dot=999 prob=12.5

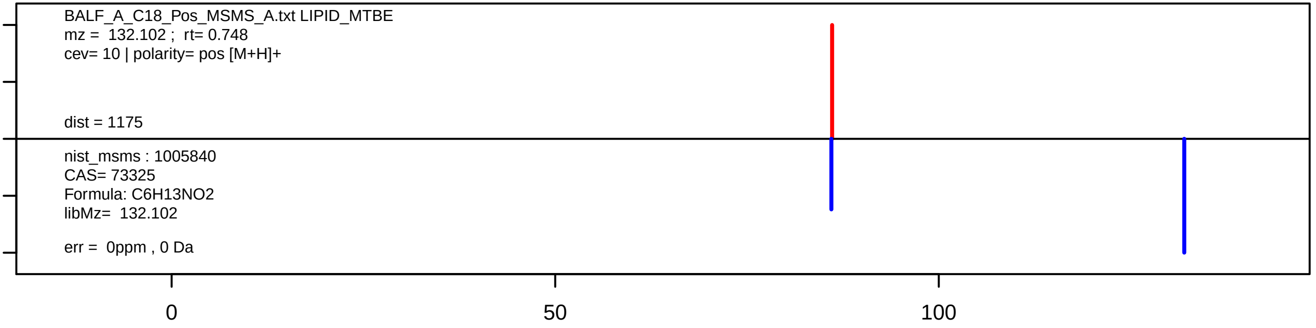

129 .   L−Leucine
Score=431 Dot=902 prob=79

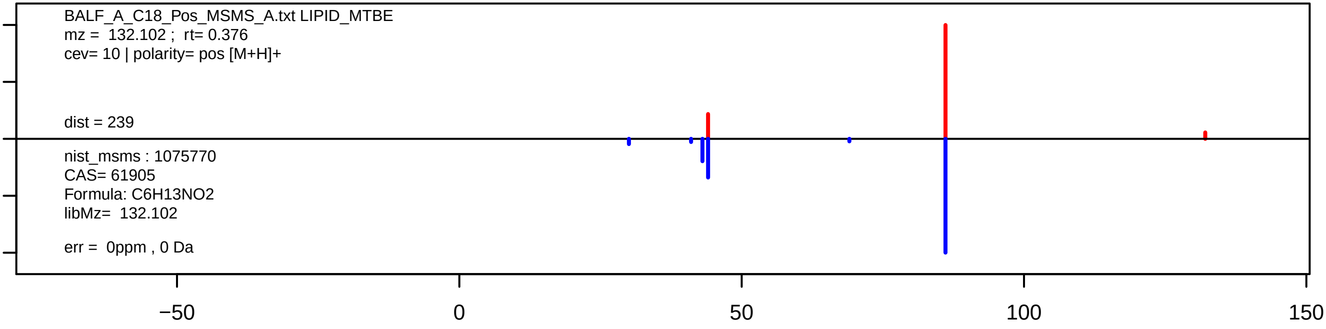

130 . L−Norvaline
Score=302 Dot=863 prob=17.1

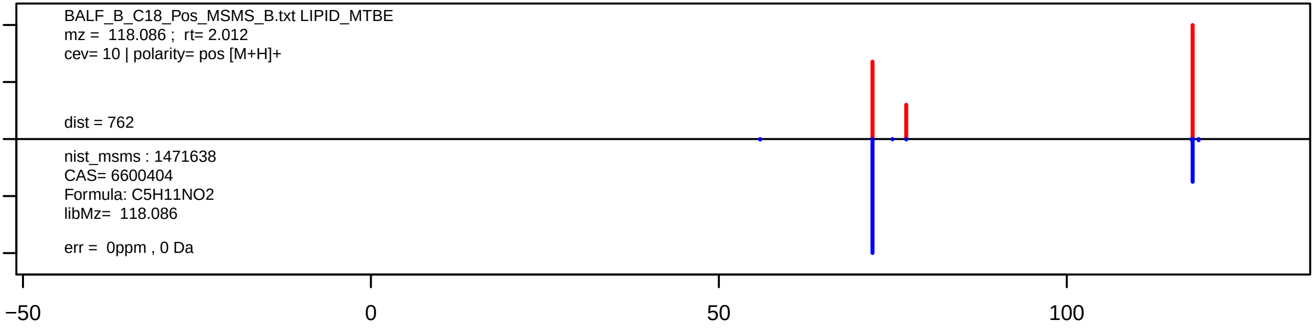

131 . L−Valine
Score=400 Dot=999 prob=14.4

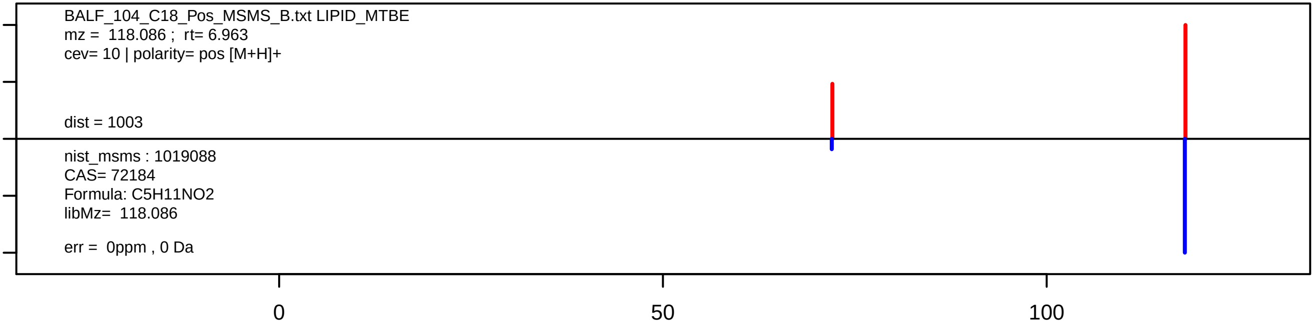

132 . Lidocaine
Score=399 Dot=999 prob=47.7

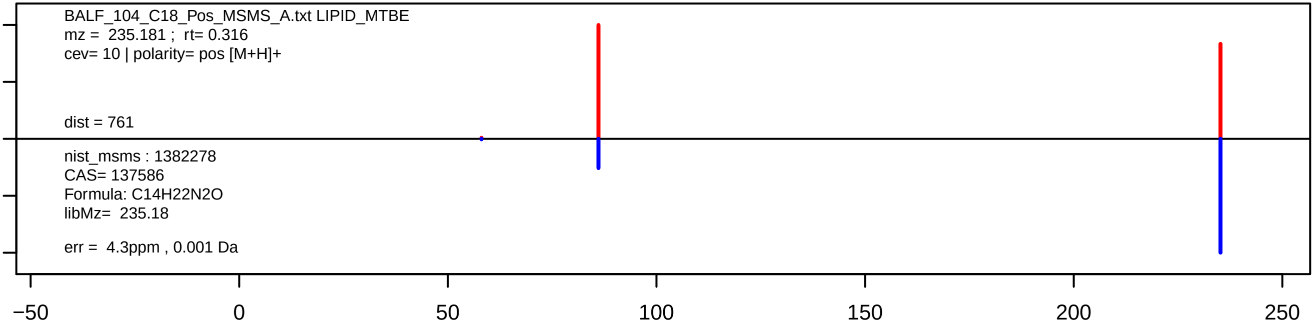

133 . lysoPC 18:0; [M+Na]+; PC(18:0/0:0)
Score=375 Dot=995 prob=53.1

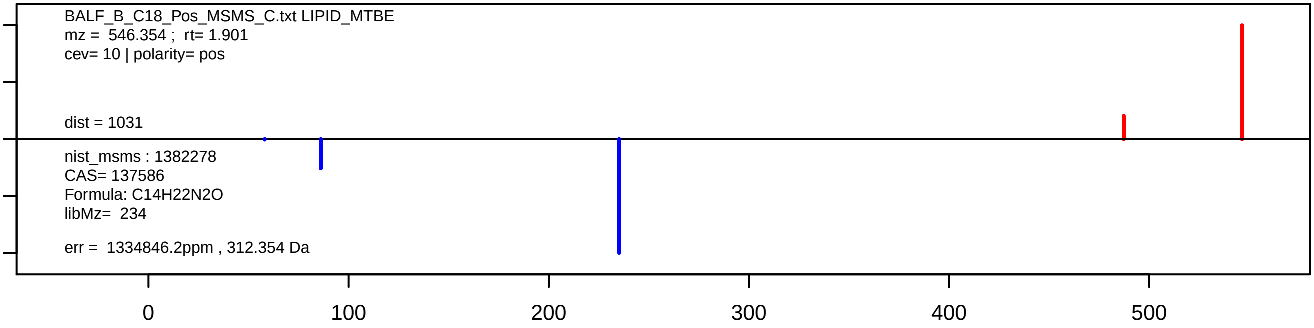

134 . lysoPC 18:2; [M+H]+; PC(18:2(2E,4E)/0:0)
Score=240 Dot=870 prob=15.9

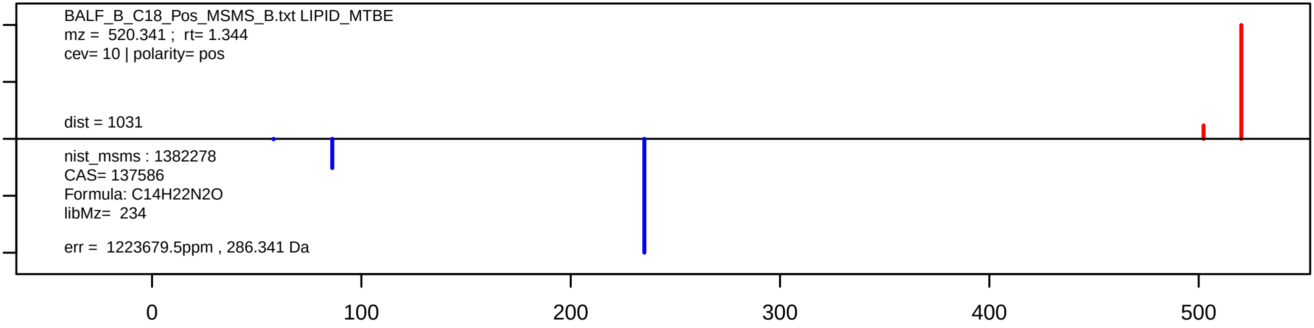

135 . lysoPC 18:2; [M+Na]+; PC(18:2(2E,4E)/0:0)
Score=375 Dot=995 prob=16.6

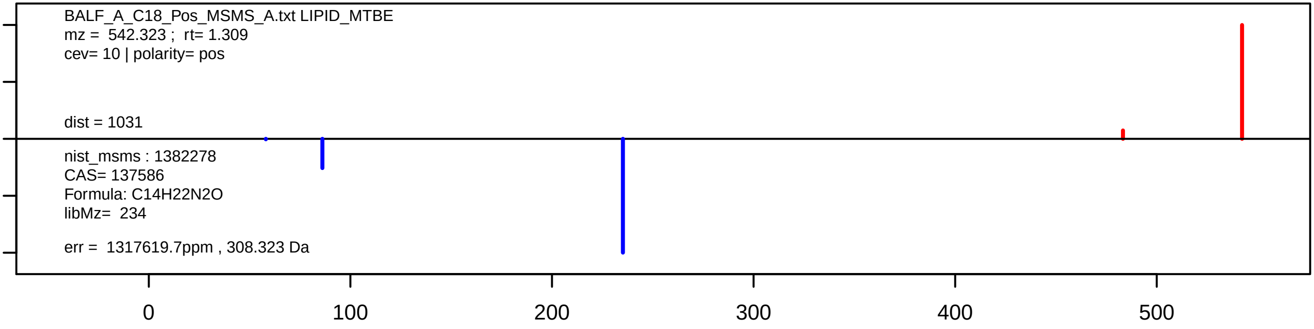

136 . lysoPE 18:1; [M+H]+; PE(18:1(11E)/0:0)
Score=482 Dot=947 prob=10.9

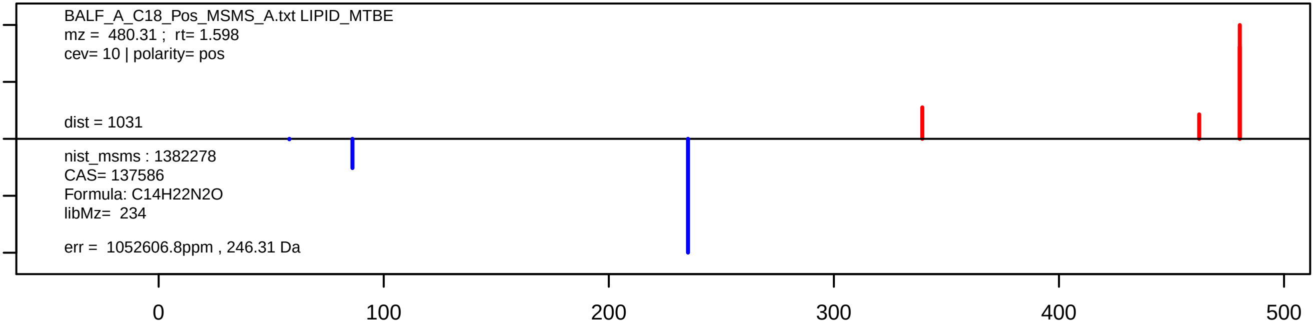

137 . lysoPE 18:2; [M+H]+; PE(18:2(2E,4E)/0:0)
Score=332 Dot=898 prob=16.5

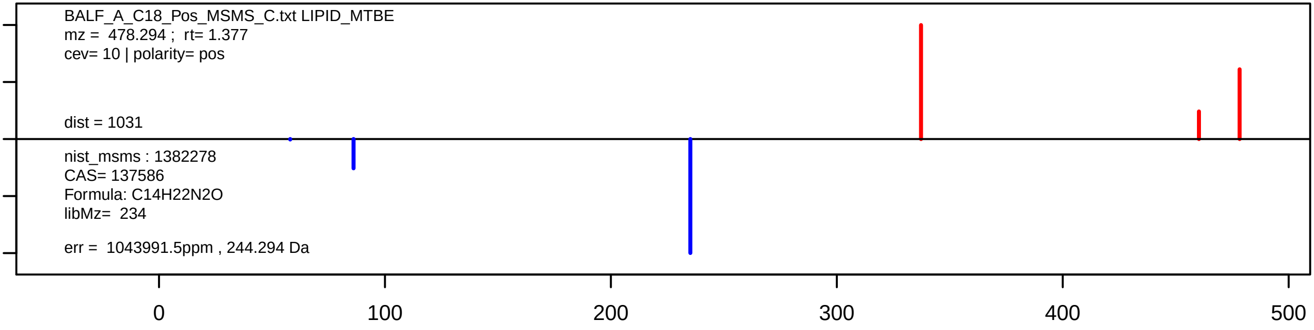

138 . lysoPE 20:1; [M+H]+; PE(20:1(11E)/0:0)
Score=452 Dot=942 prob=24.1

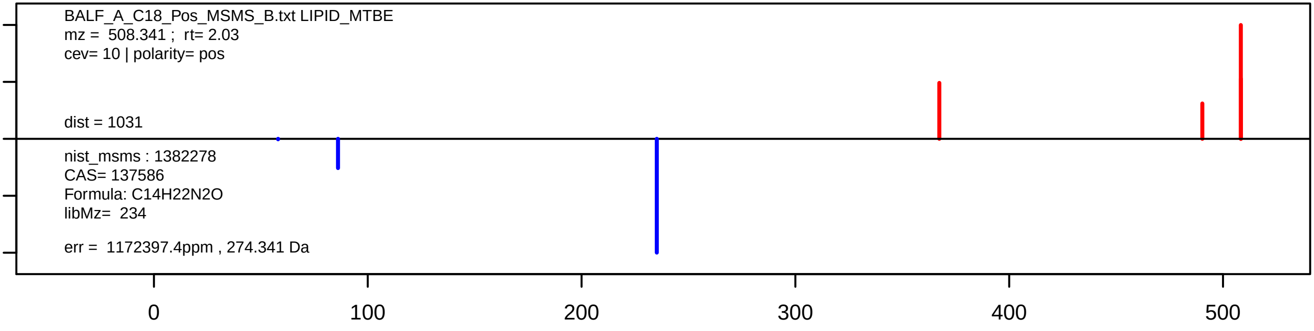

139 . Metformin
Score=181 Dot=807 prob=80.3

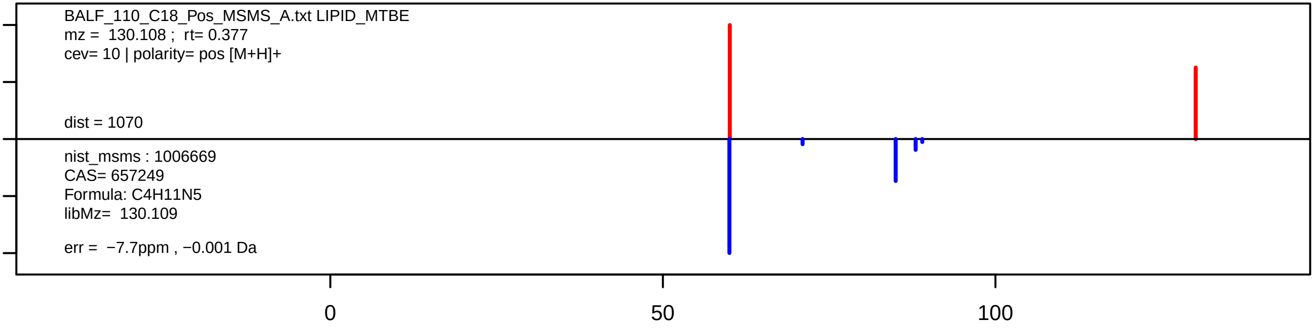

140 . Methadone
Score=400 Dot=999 prob=43.4

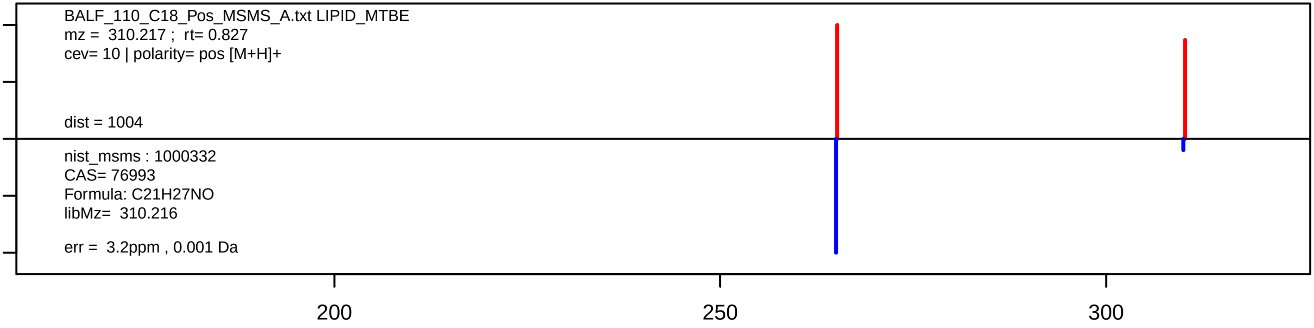

141 . Misoprostol (free acid)
Score=253 Dot=911 prob=94.5

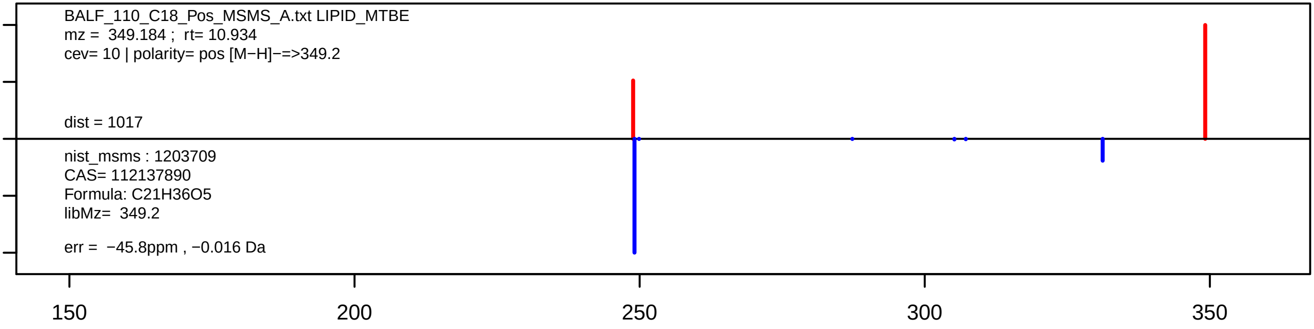

142 . Mono−2−ethylhexyl phthalate
Score=354 Dot=829 prob=68.3

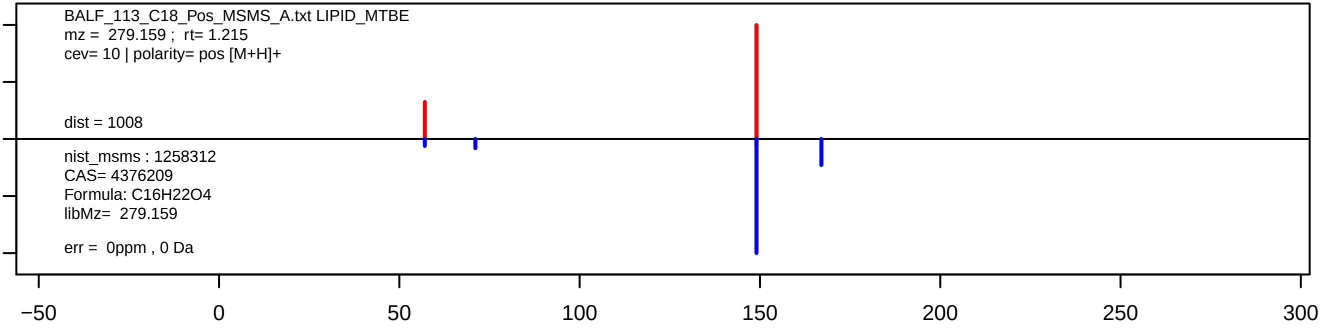

143 . Monoisobutyl phthalate
Score=274 Dot=980 prob=39.1

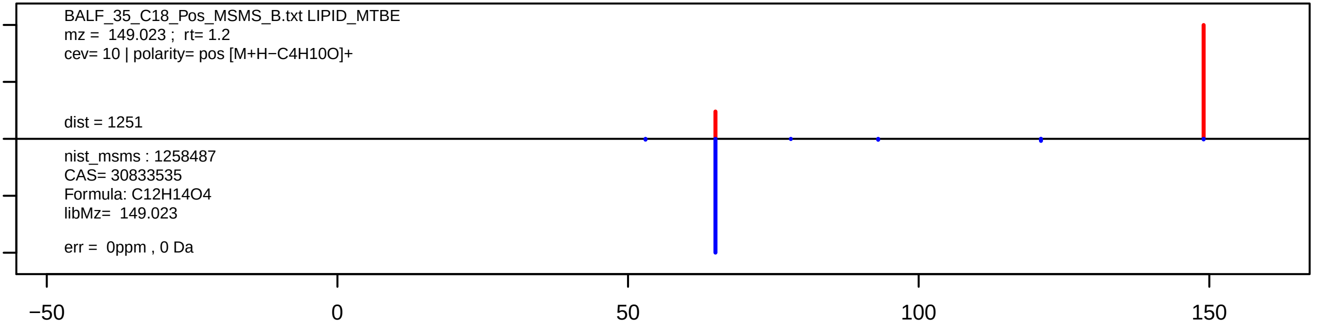

144 . N−(Octadecanoyl)sphing−4−enine−1−phosphocholine
Score=400 Dot=999 prob=99

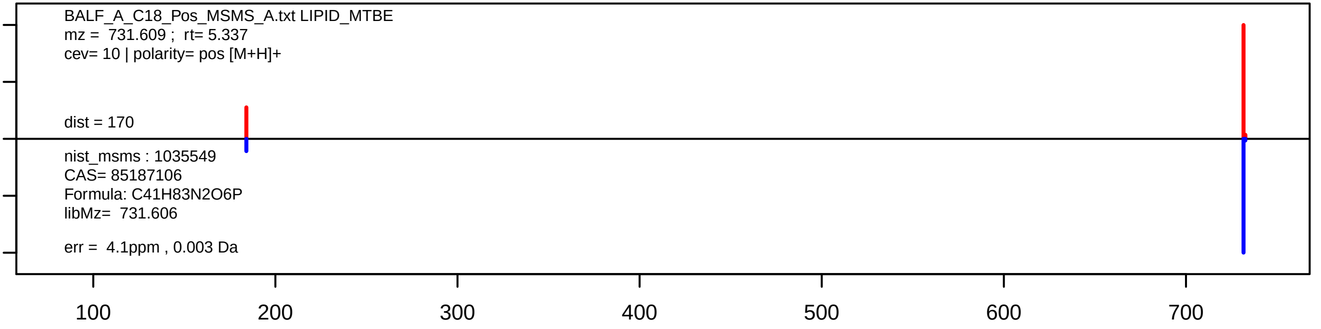

145 . N−Acetyl−D−lactosamine
Score=954 Dot=985 prob=23.2

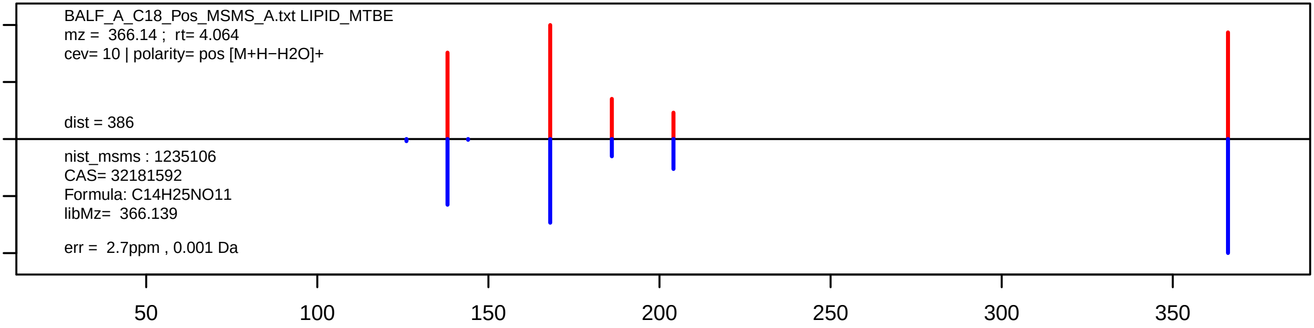

146 . N−Acetyl−D−norleucine
Score=381 Dot=862 prob=41.5

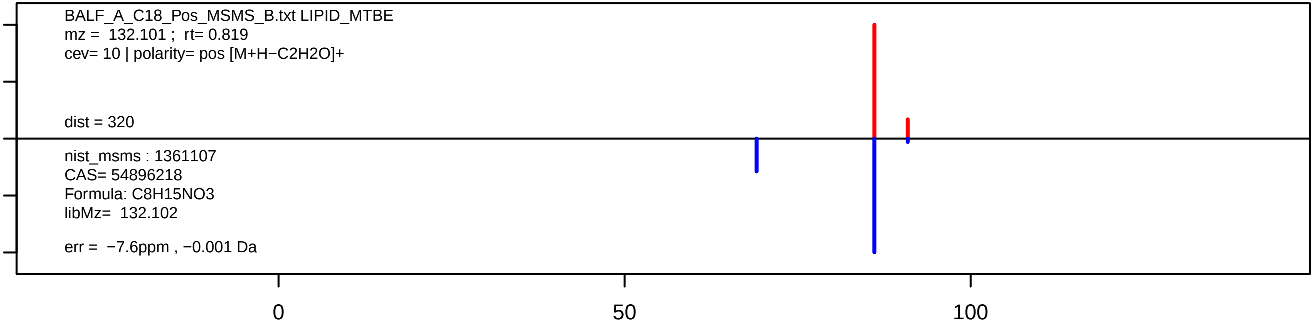

147 . N−Acetyl−DL−leucine
Score=361 Dot=910 prob=38.9

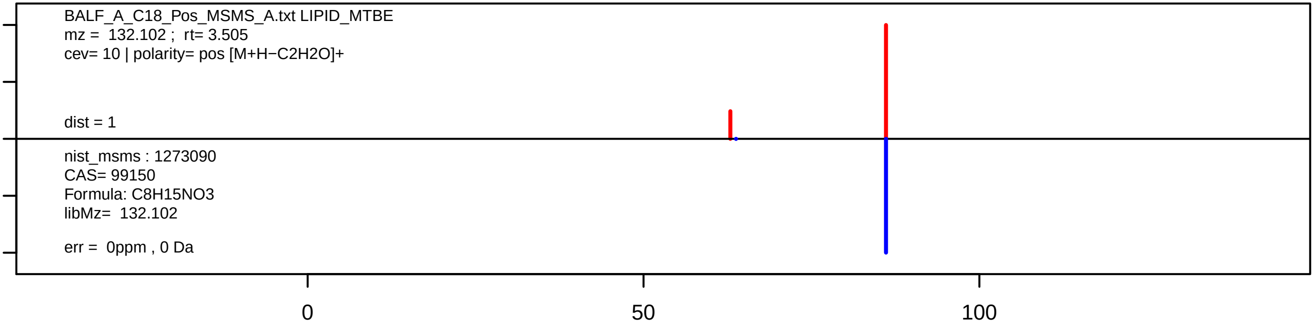

148 . N−Desmethyltramadol
Score=302 Dot=957 prob=98.5

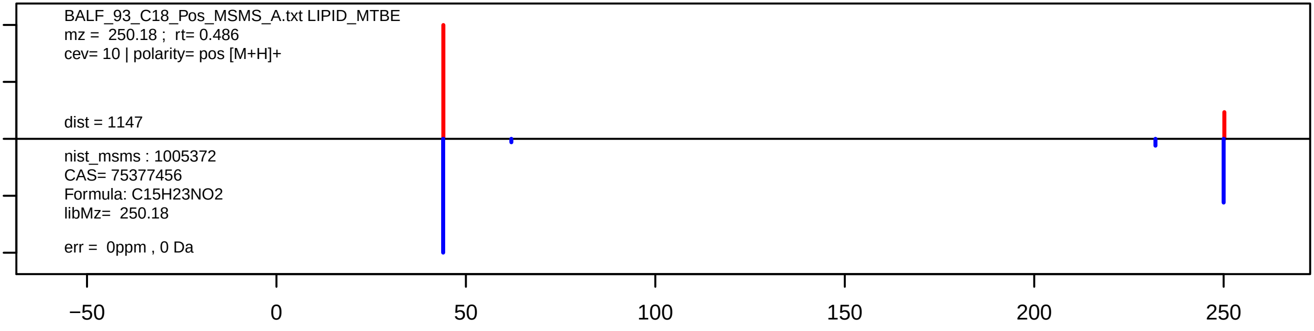

149 . N−Docosanoyl−4−sphingenyl−1−O−phosphorylcholine
Score=355 Dot=982 prob=91.7

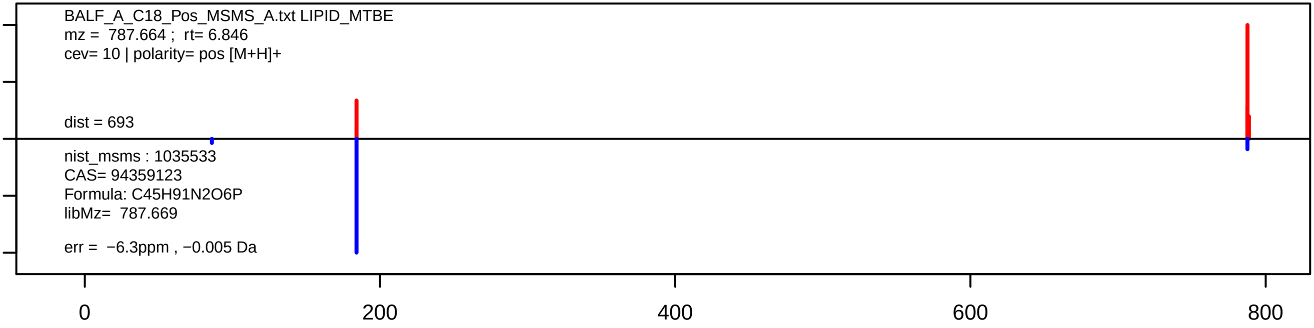

150 . N−Methyl−.alpha.−aminoisobutyric acid
Score=256 Dot=816 prob=22.7

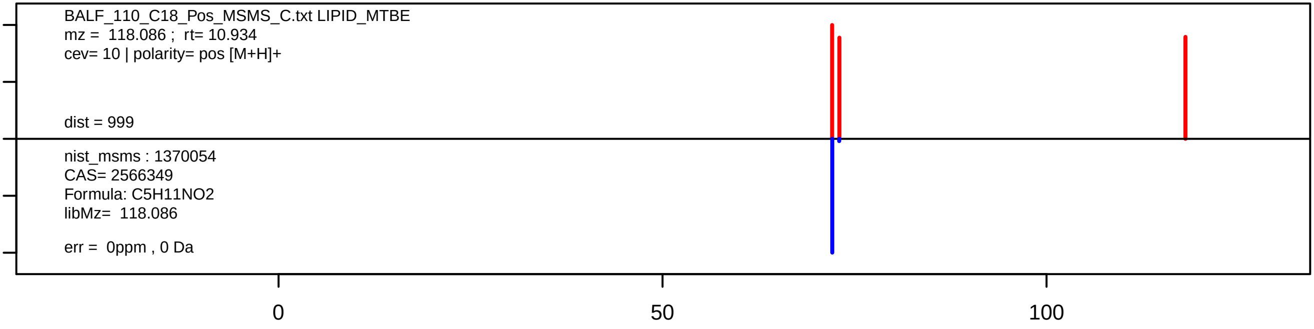

151 . N−Nervonoyl−D−erythro−sphingosylphosphorylcholine
Score=400 Dot=999 prob=100

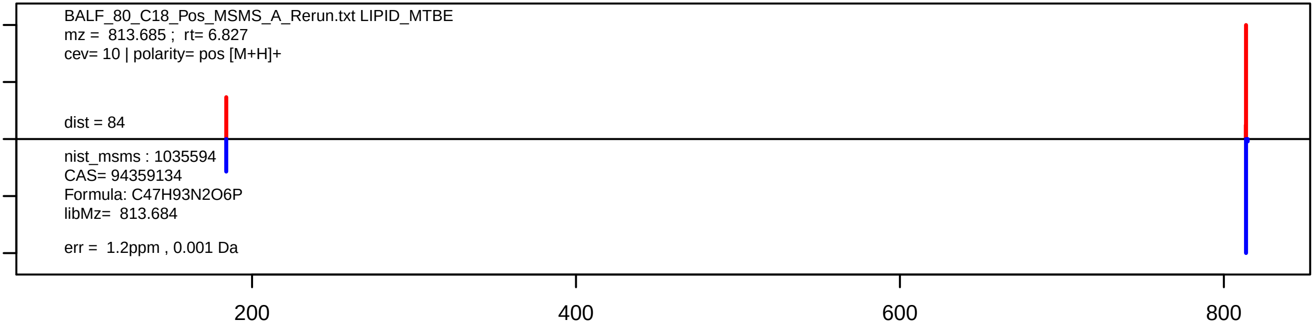

152 . n−Octadecylamine
Score=303 Dot=953 prob=96.7

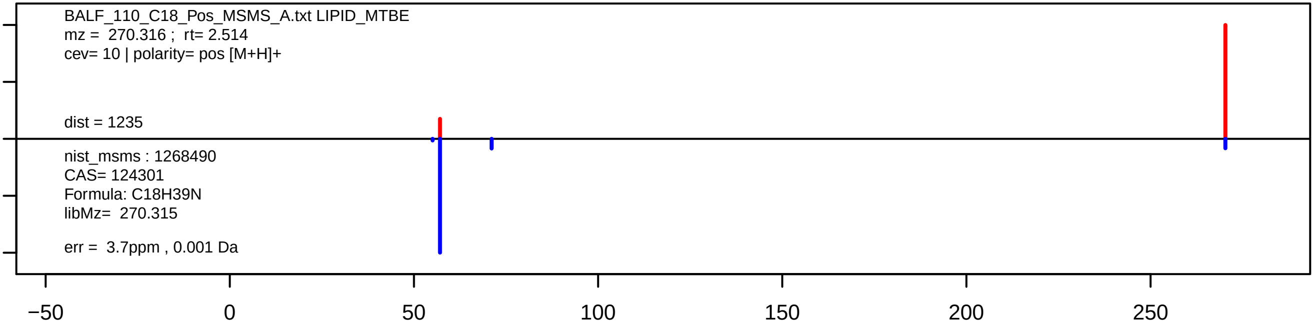

153 . N−Oleoyl−D−erythro−sphingosylphosphorylcholine
Score=400 Dot=999 prob=99

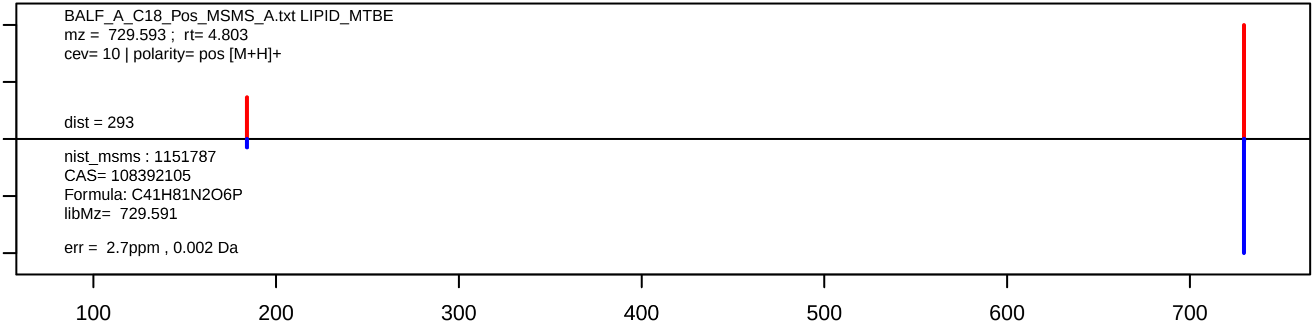

154 . N−Tetracosanoyl−4−sphingenyl−1−O−phosphorylcholine
Score=194 Dot=906 prob=98

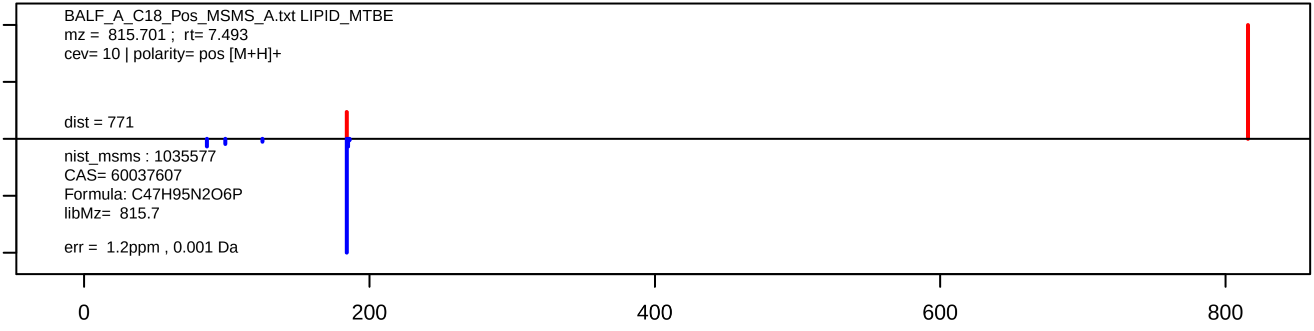

155 . N−Tetracosenoyl−4−sphingenine
Score=120 Dot=833 prob=65.6

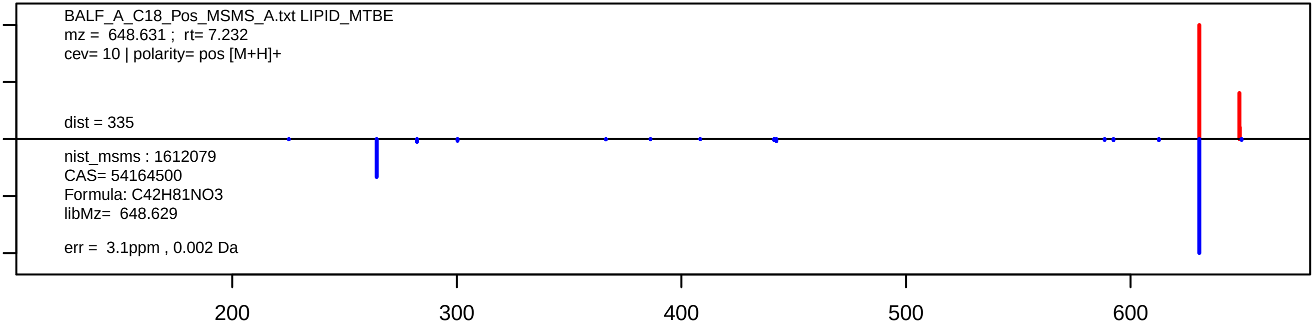

156 . Norquetiapine
Score=756 Dot=890 prob=98.9

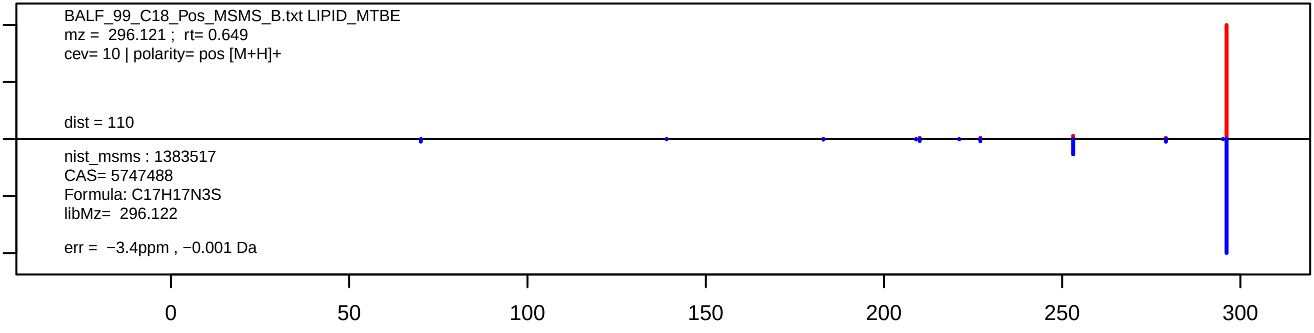

157 . Oleamide
Score=450 Dot=872 prob=67

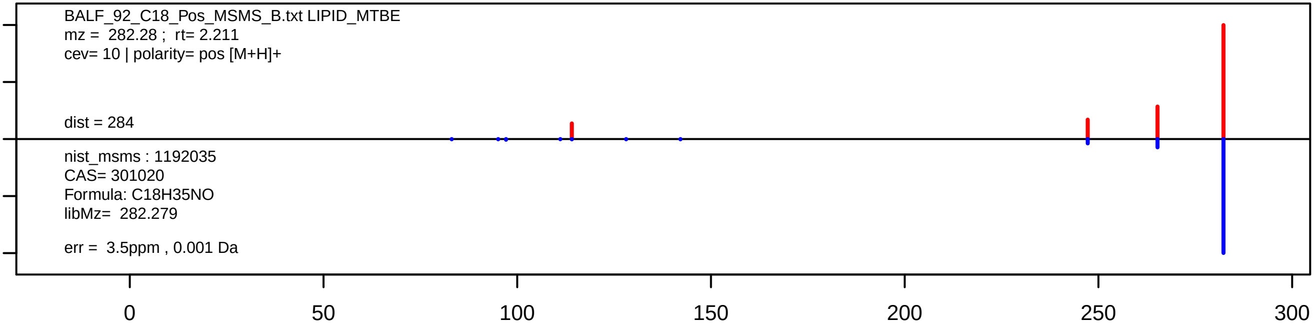

158 . Palmitoyl sphingomyelin
Score=400 Dot=999 prob=100

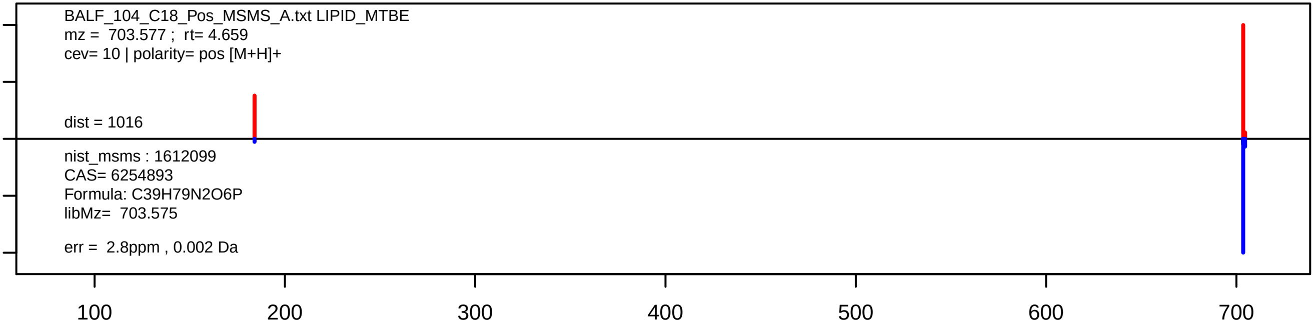

159 . Palmitoyl−L−carnitine
Score=232 Dot=831 prob=84.8

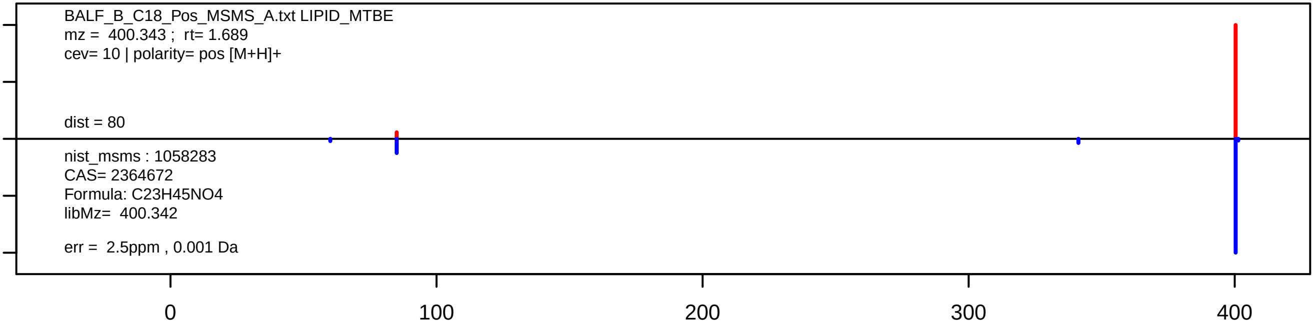

160 .   Palmitoyleicosapentaenoyl phosphatidylcholine
Score=181 Dot=953 prob=100

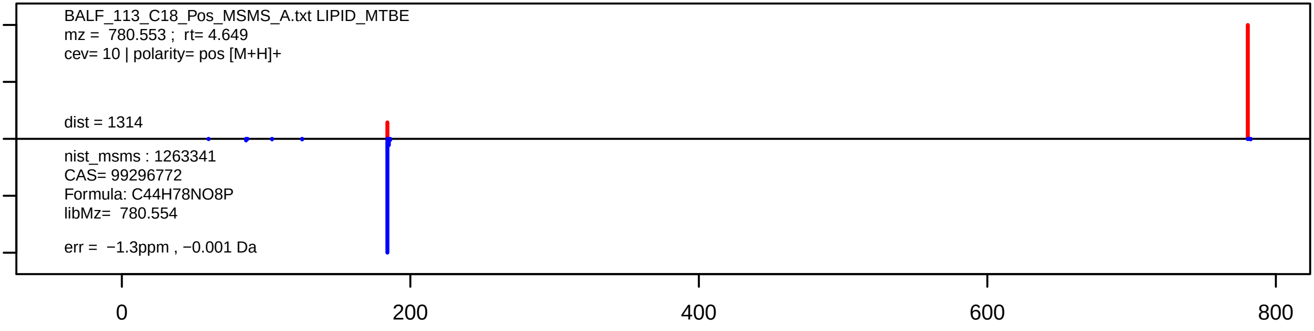

161 .   PC 30:0; [M+Na]+; GPCho(15:0/15:0)
Score=435 Dot=963 prob=17.5

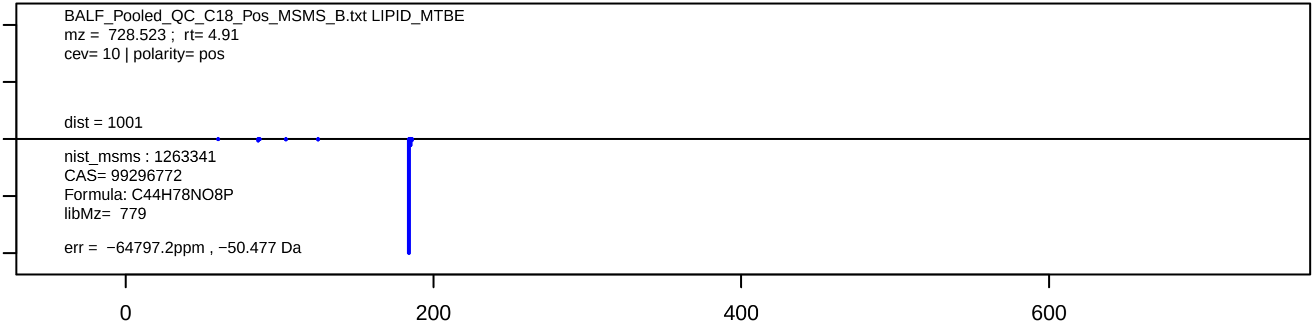

162 .   PC 32:0; [M+Na]+; GPCho(16:0/16:0)
Score=519 Dot=974 prob=19.7

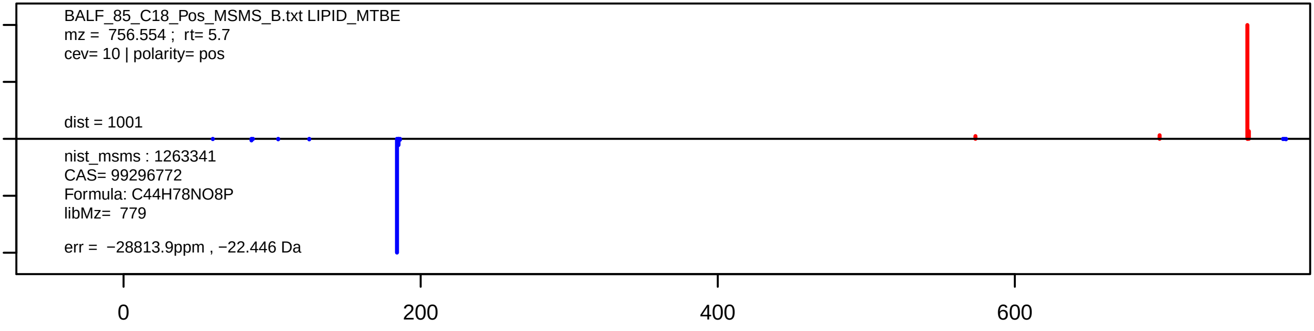

163 .   PC 36:4; [M+Na]+; GPCho(18:2(2E,4E)/18:2(2E,4E))
Score=528 Dot=975 prob=2.1

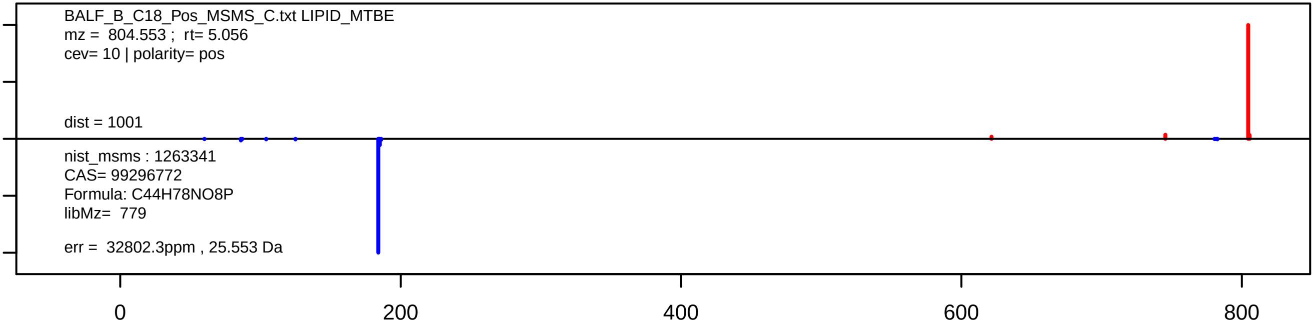

164 .   PC 39:8; [M+Na]+; GPCho(17:2(9Z,12Z)/22:6(4Z,7Z,10Z,13Z,16Z,19Z))
Score=481 Dot=953 prob=49.6

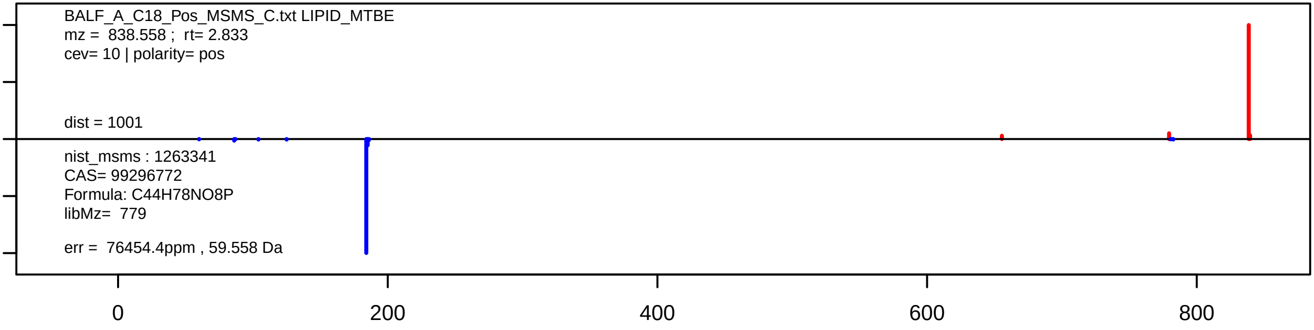

165 .   PE 19:1; [M+H]+; GPEtn(2:0/17:1(9Z))
Score=351 Dot=996 prob=10

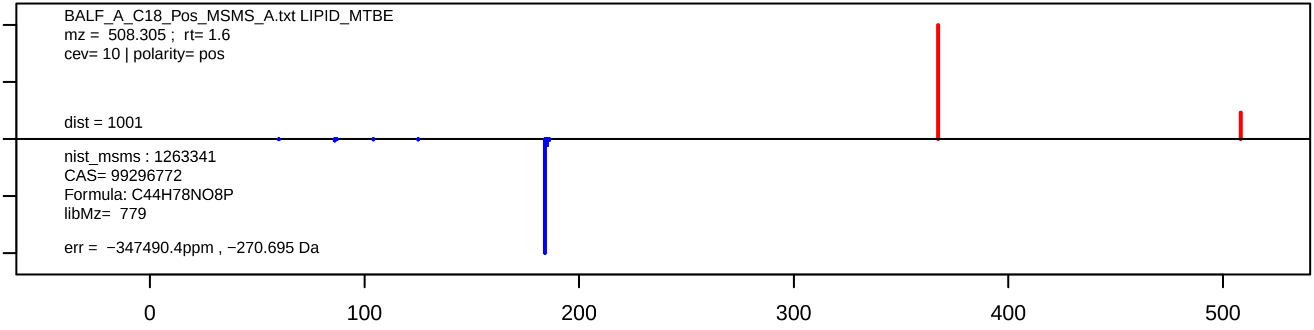

166 . PE 19:1; [M+Na]+; GPEtn(2:0/17:1(9Z))
Score=370 Dot=833 prob=9.9

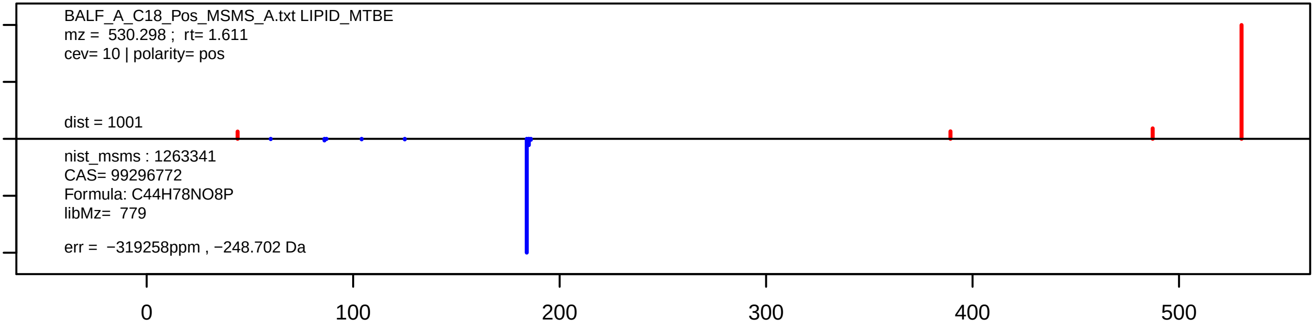

167 . PE 19:2; [M+H]+; GPEtn(2:0/17:2(9Z,12Z))
Score=351 Dot=996 prob=49.5

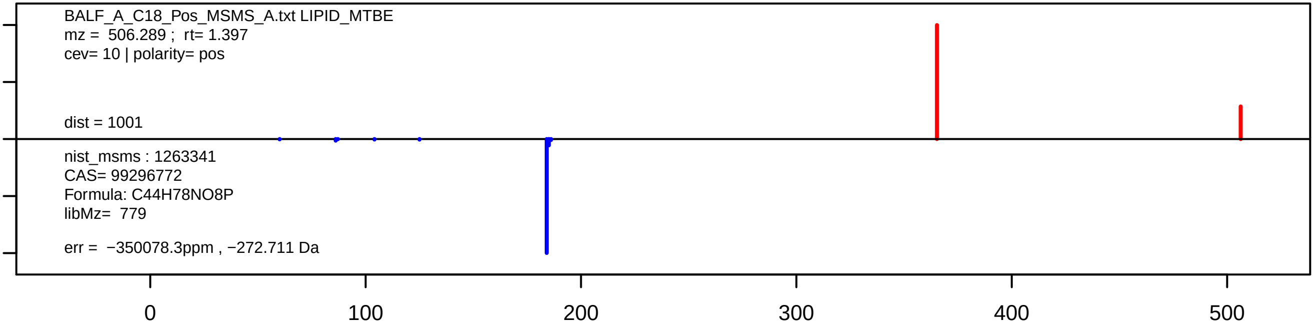

168 . PE 23:4; [M+H]+; GPEtn(3:0/20:4(5E,8E,11E,14E))
Score=351 Dot=996 prob=9.9

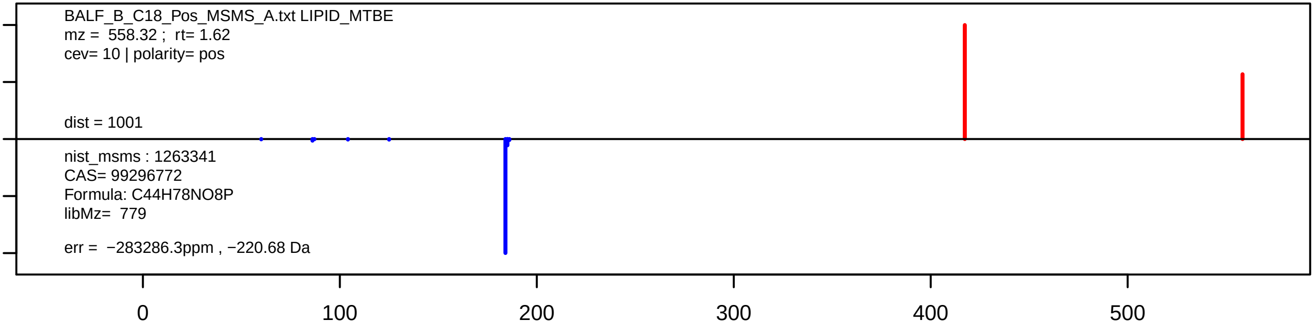

169 . PE 30:0; [M+H]+; GPEtn(15:0/15:0)
Score=372 Dot=998 prob=8.8

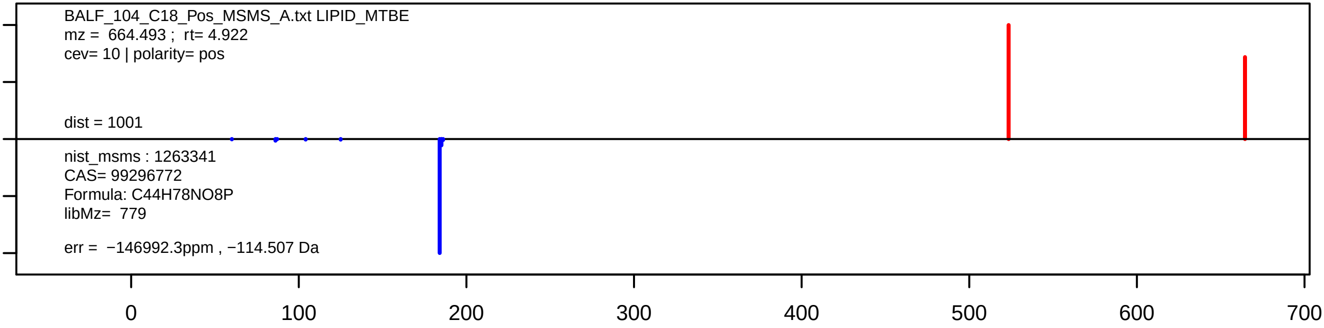

170 . PE 30:0; [M+H]+; GPEtn(4:0/26:0)
Score=152 Dot=873 prob=43.6

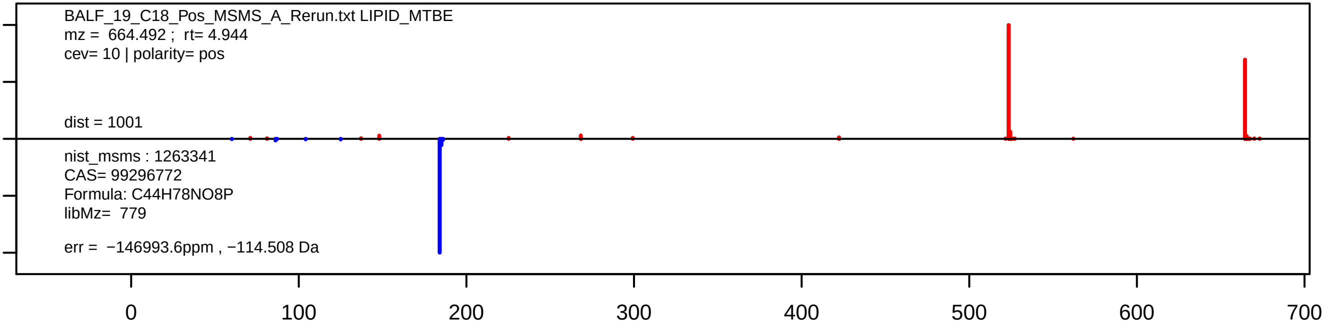

171 . PE 30:0; [M+H]+; GPEtn(9:0/21:0)
Score=324 Dot=978 prob=45.1

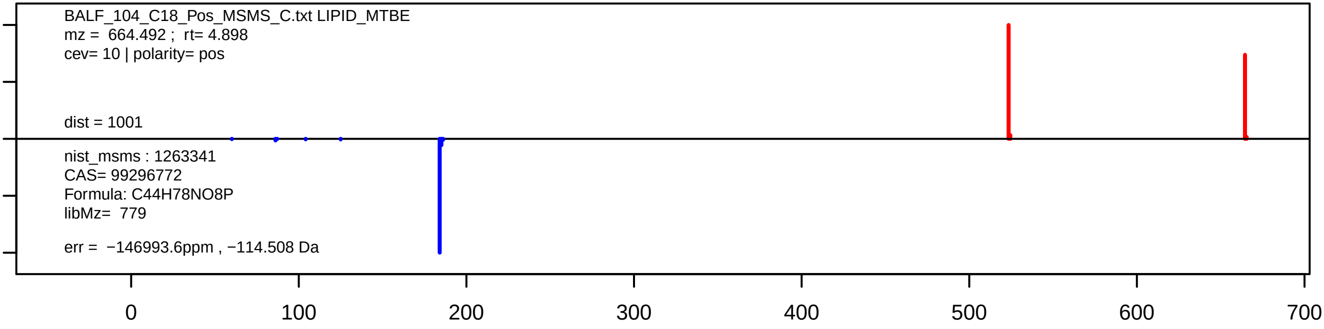

172 . PE 34:0; [M+H]+; GPEtn(17:0/17:0)
Score=372 Dot=998 prob=11.6

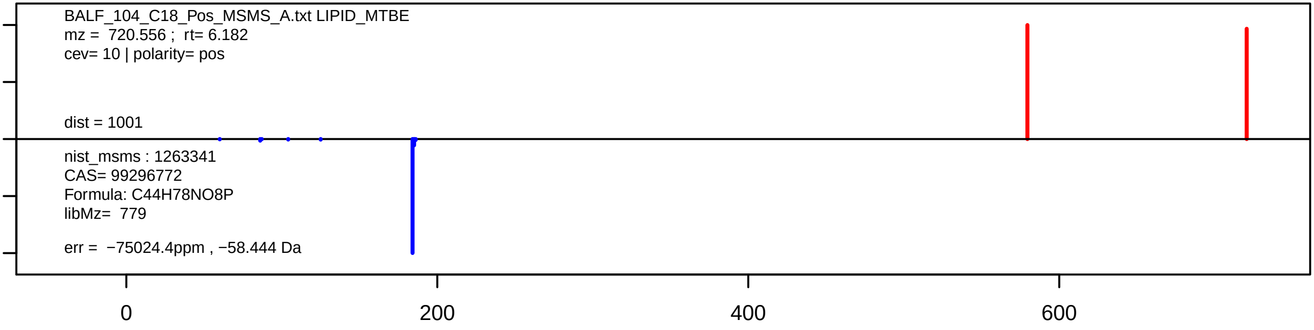

173 . PE 34:0; [M+H]+; GPEtn(9:0/25:0)
Score=312 Dot=963 prob=19.6

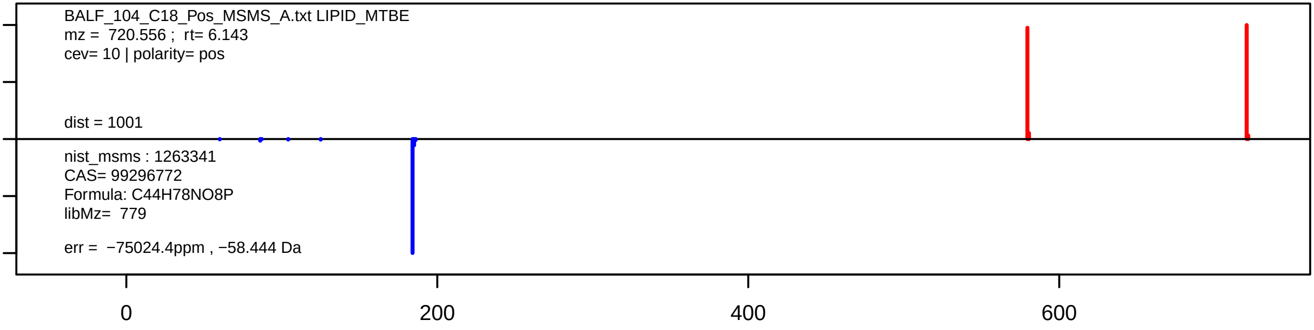

174 . PE 34:2; [M+H]+; GPEtn(17:1(9Z)/17:1(9Z))
Score=372 Dot=998 prob=3

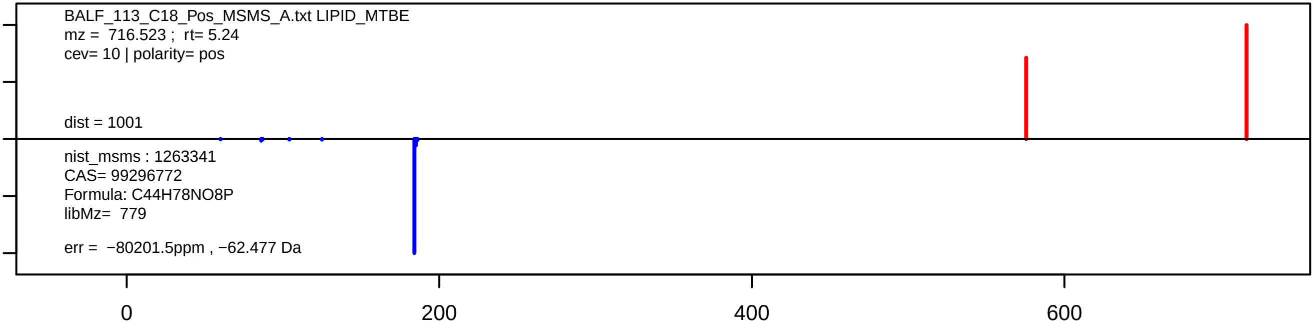

175 . PE 35:1; [M+H]+; GPEtn(9:0/26:1(5Z))
Score=351 Dot=996 prob=2.4

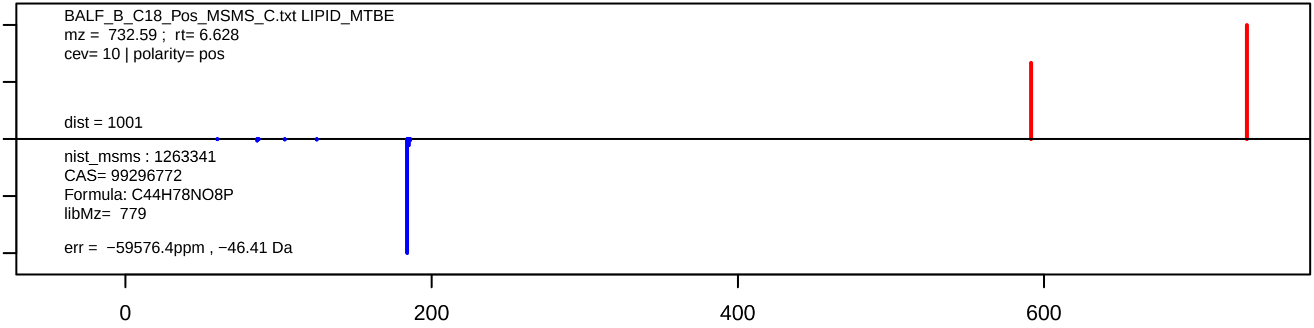

176 . PE 36:3; [M+H]+; GPEtn(14:1(9Z)/22:2(13Z,16Z))
Score=351 Dot=996 prob=1

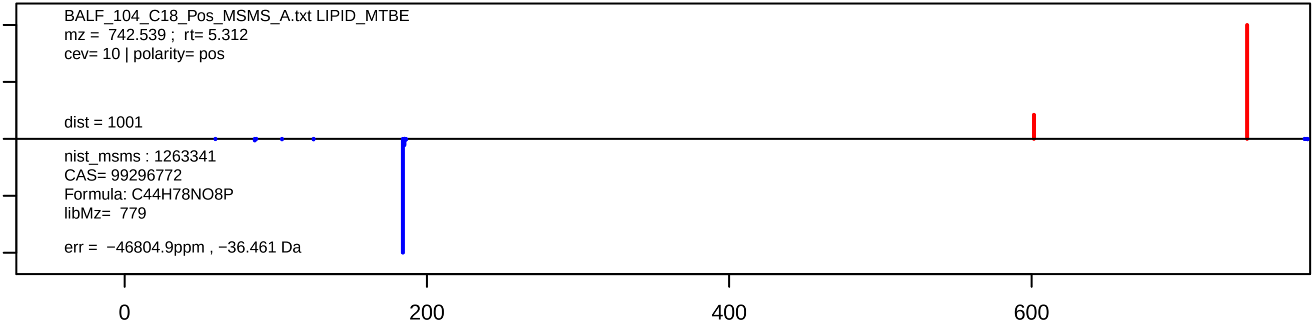

177 . PE 37:4; [M+H]+; GPEtn(13:0/24:4(5Z,8Z,11Z,14Z))
Score=351 Dot=996 prob=4.5

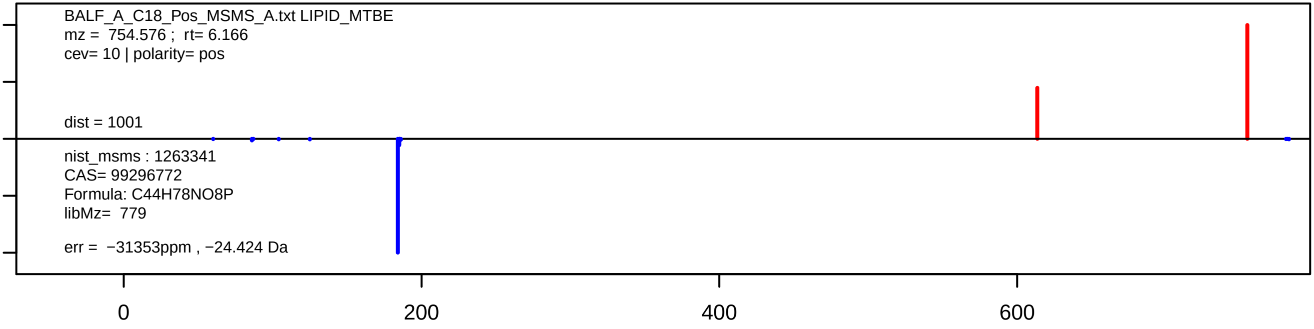

178 . PE 38:4; [M+H]+; GPEtn(14:0/24:4(5Z,8Z,11Z,14Z))
Score=351 Dot=996 prob=1.1

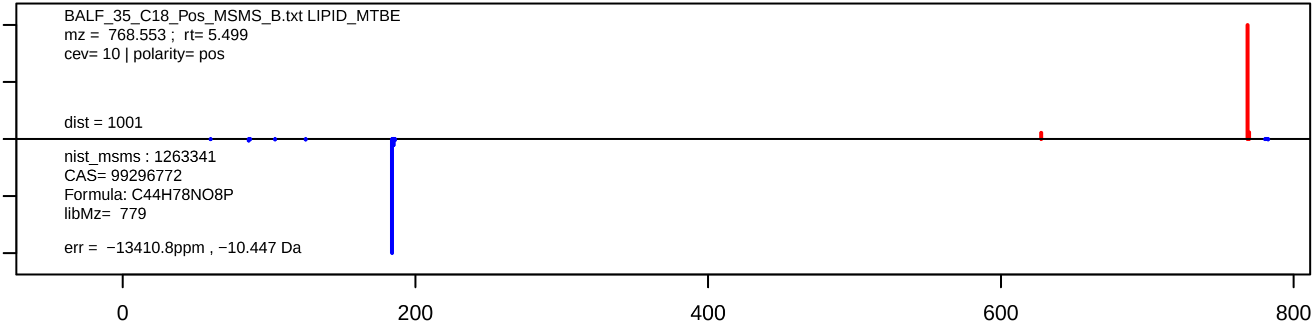

179 . PE 39:3; [M+H]+; GPEtn(17:1(9Z)/22:2(13Z,16Z))
Score=351 Dot=996 prob=8.2

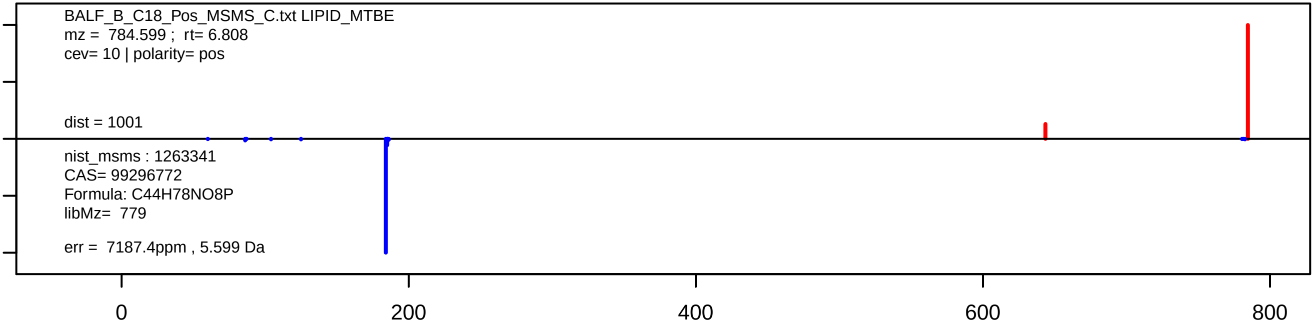

180 . PE 39:4; [M+H]+; GPEtn(15:0/24:4(5Z,8Z,11Z,14Z))
Score=351 Dot=996 prob=6.1

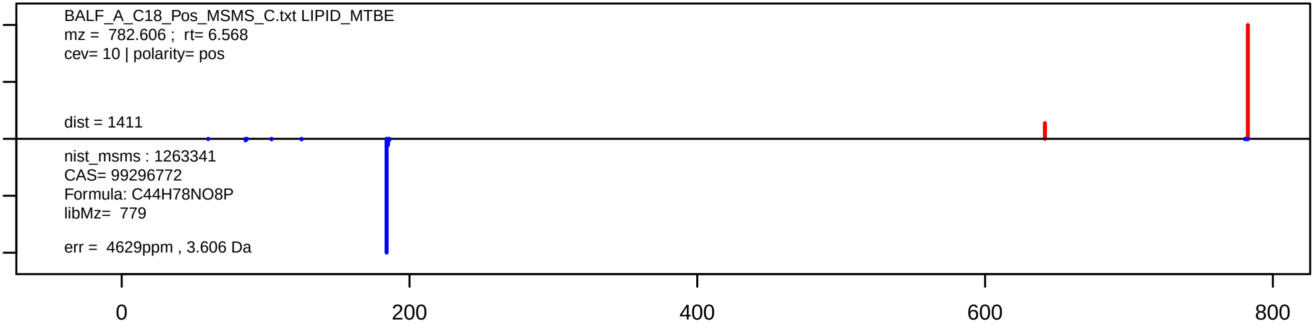

181 . PE 39:4; [M+H]+; GPEtn(17:2(9Z,12Z)/22:2(13Z,16Z))
Score=301 Dot=851 prob=21.3

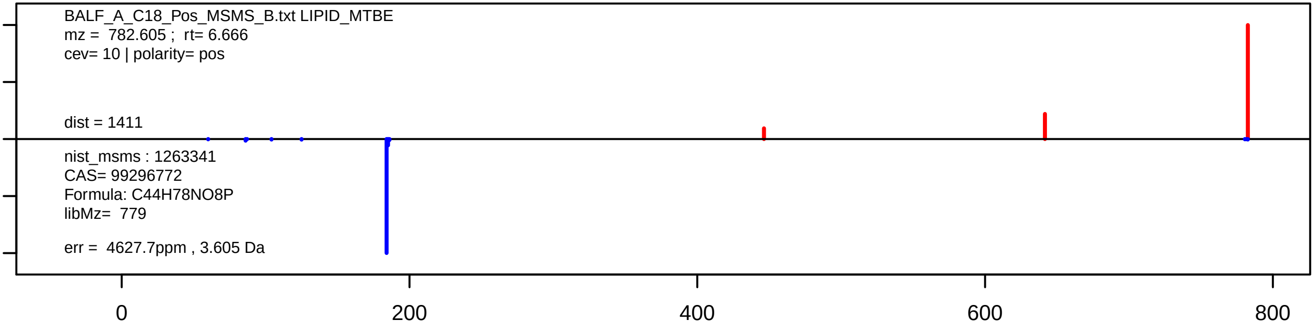

182 . PE 41:3; [M+H]+; GPEtn(15:1(9Z)/26:2(5E,9Z))
Score=351 Dot=996 prob=3.7

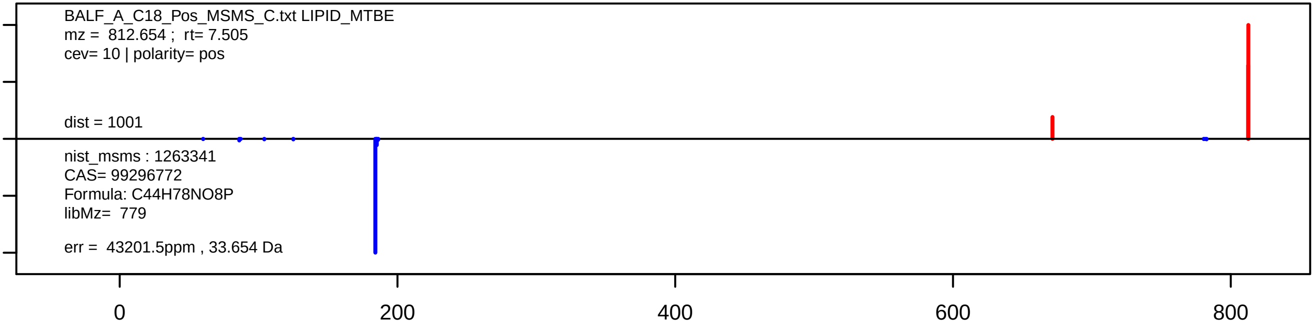

183 . PE 41:4; [M+H]+; GPEtn(17:0/24:4(5Z,8Z,11Z,14Z))
Score=351 Dot=996 prob=6.9

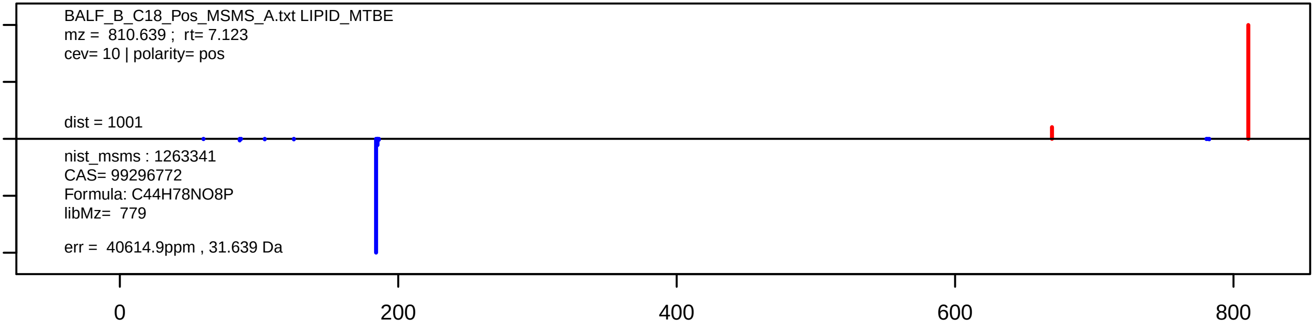

184 . plasmenyl−PC 32:0; [M+Na]+; PC(P−16:0/16:0)
Score=276 Dot=957 prob=26.6

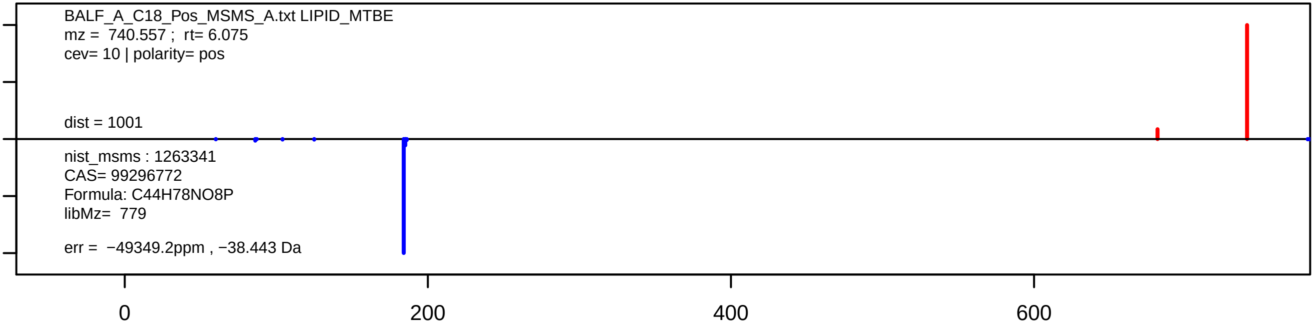

185 . plasmenyl−PC 33:0; [M+Na]+; PC(P−16:0/17:0)
Score=276 Dot=957 prob=23.4

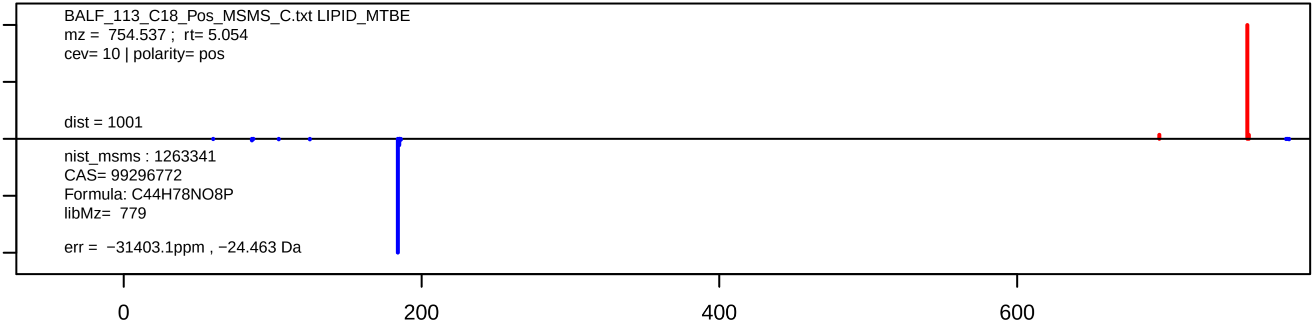

186 . plasmenyl−PC 34:0; [M+Na]+; PC(P−16:0/18:0)
Score=276 Dot=957 prob=30

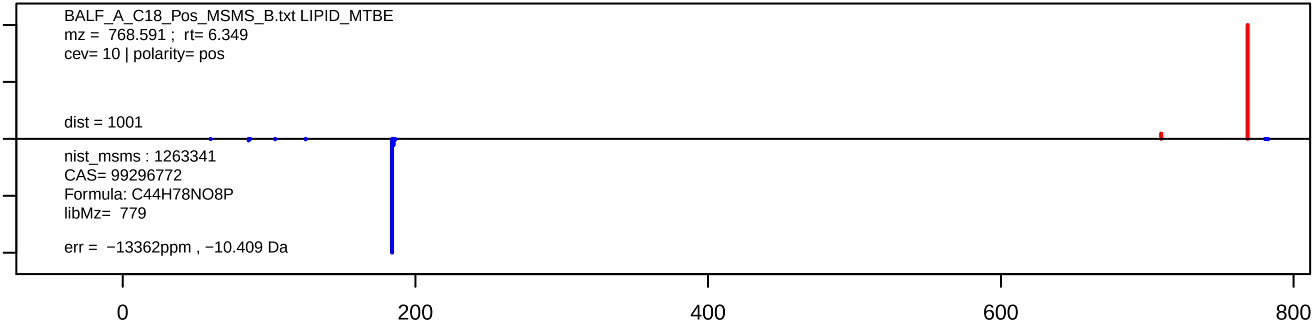

187 . plasmenyl−PC 35:2; [M+Na]+; PC(P−18:0/17:2(9Z,12Z))
Score=276 Dot=957 prob=45.7

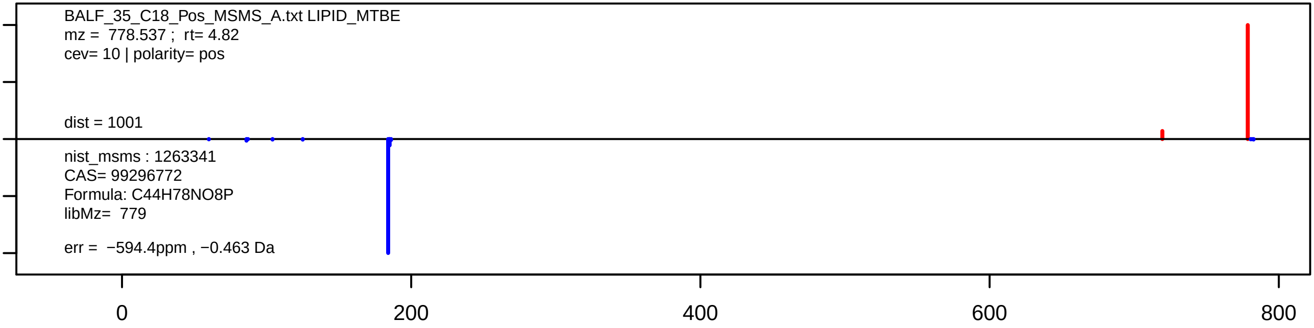

188 . plasmenyl−PC 36:1; [M+Na]+; PC(P−16:0/20:1(11E))
Score=276 Dot=957 prob=6.7

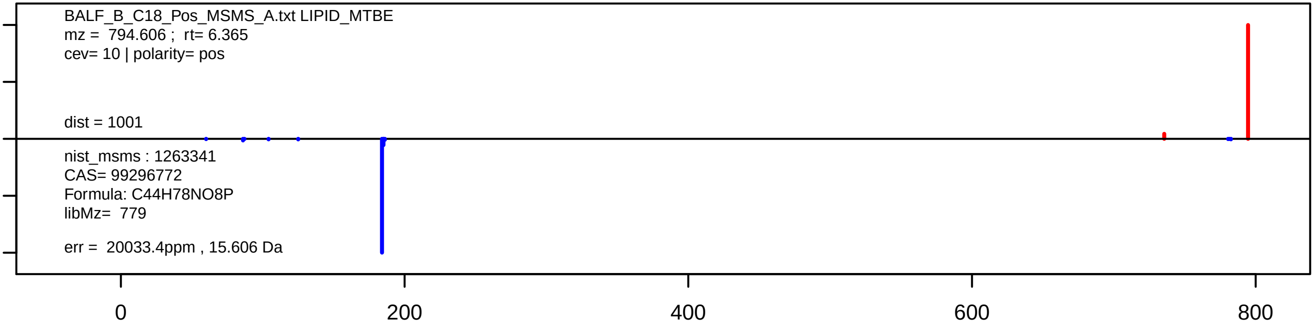

189 . plasmenyl−PC 36:3; [M+Na]+; PC(P−16:0/20:3(5Z,8Z,11Z))
Score=276 Dot=957 prob=20.9

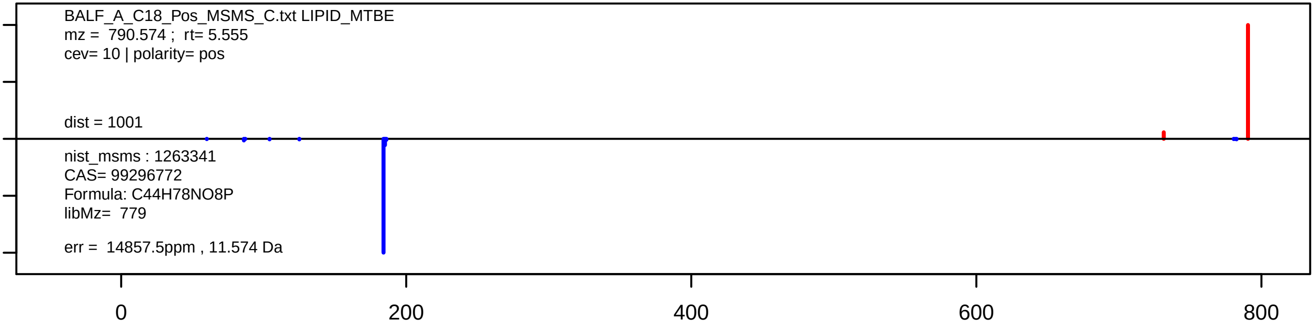

190 . plasmenyl−PC 37:0; [M+Na]+; PC(P−16:0/21:0)
Score=276 Dot=957 prob=22.9

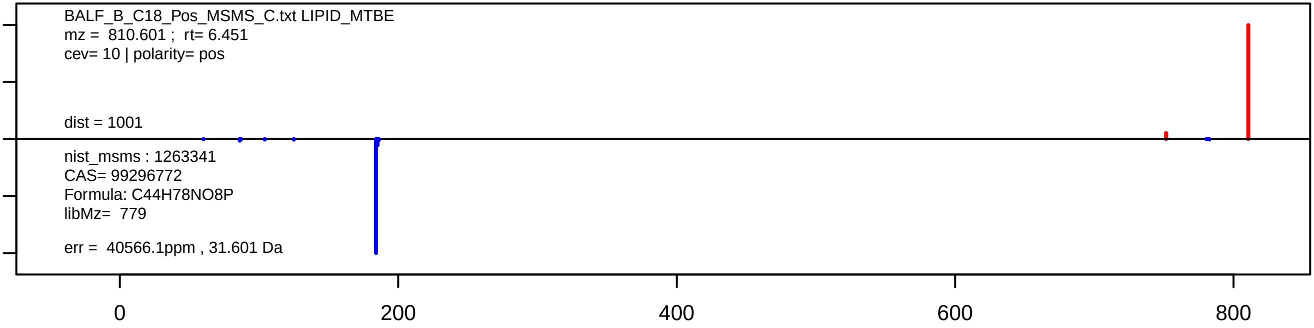

191 . plasmenyl−PC 37:1; [M+Na]+; PC(P−20:0/17:1(9Z))
Score=276 Dot=957 prob=14.3

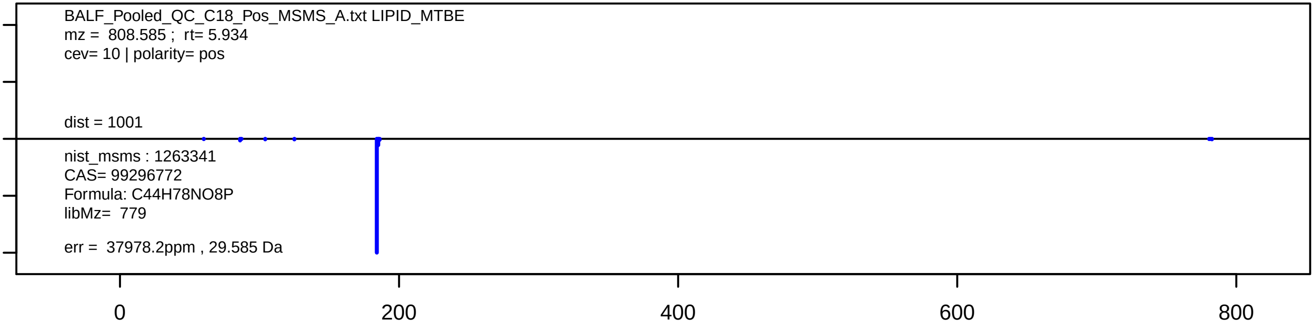

192 . plasmenyl−PC 38:1; [M+Na]+; PC(P−16:0/22:1(13Z))
Score=276 Dot=957 prob=6.6

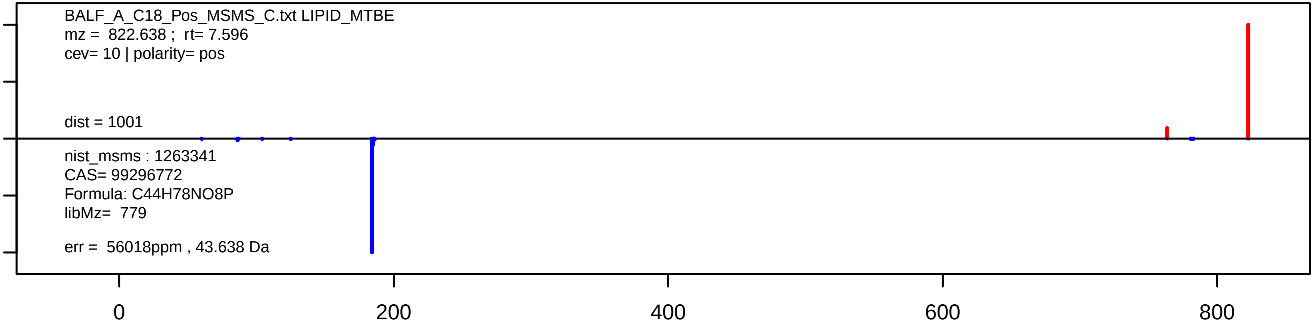

193 . plasmenyl−PC 38:2; [M+Na]+; PC(P−16:0/22:2(13Z,16Z))
Score=276 Dot=957 prob=10.3

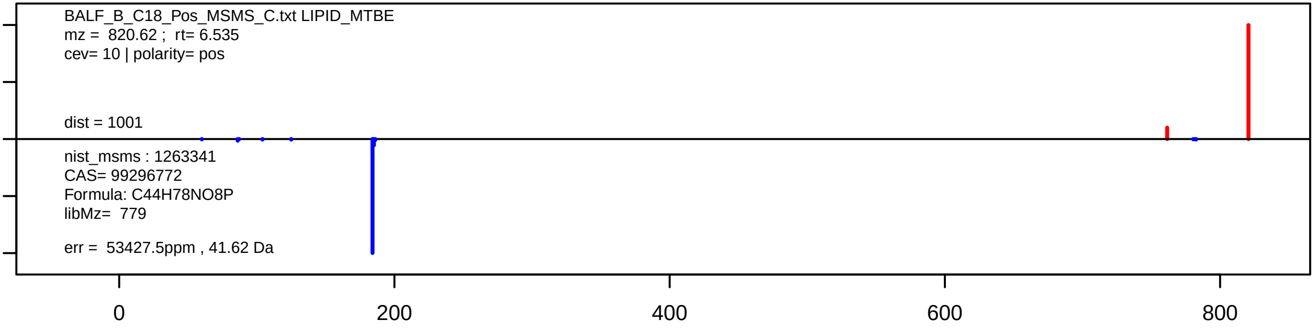

194 . plasmenyl−PE 34:1; [M+H]+; PE(P−16:0/18:1(11E))
Score=434 Dot=819 prob=11.1

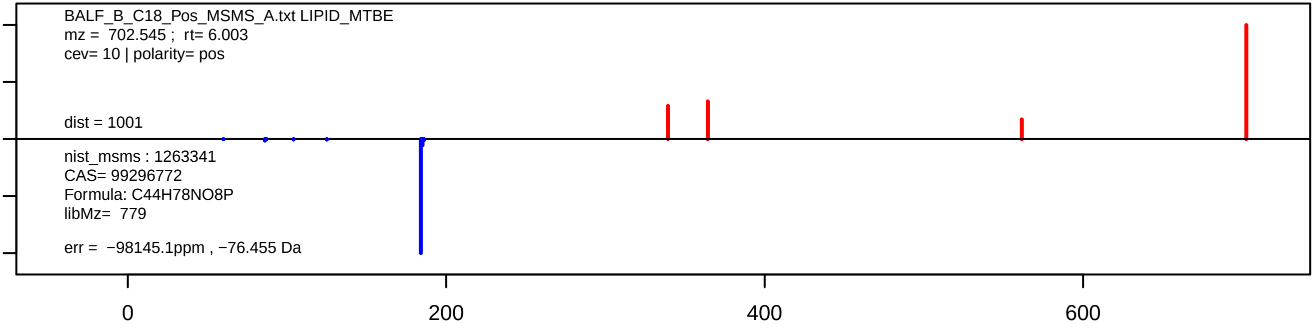

195 . plasmenyl−PE 34:2; [M+H]+; PE(P−16:0/18:2(2E,4E))
Score=336 Dot=853 prob=16.6

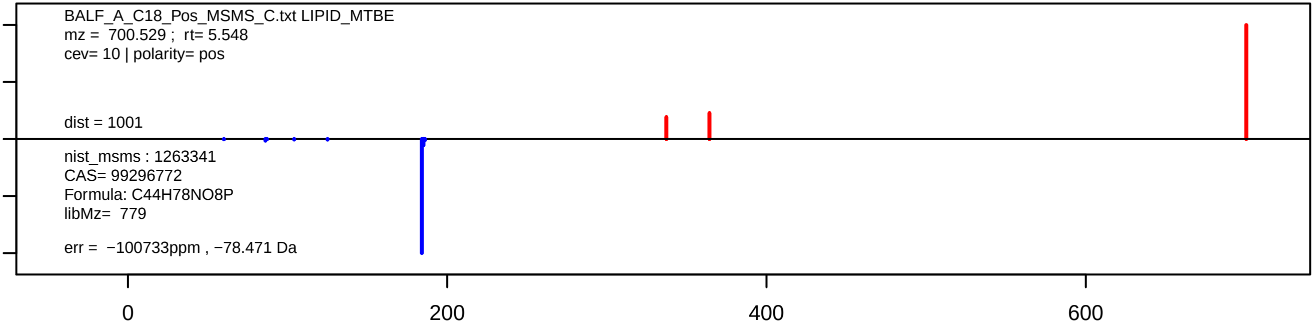

196 . plasmenyl−PE 36:1; [M+H]+; PE(P−18:0/18:1(11E))
Score=361 Dot=861 prob=11

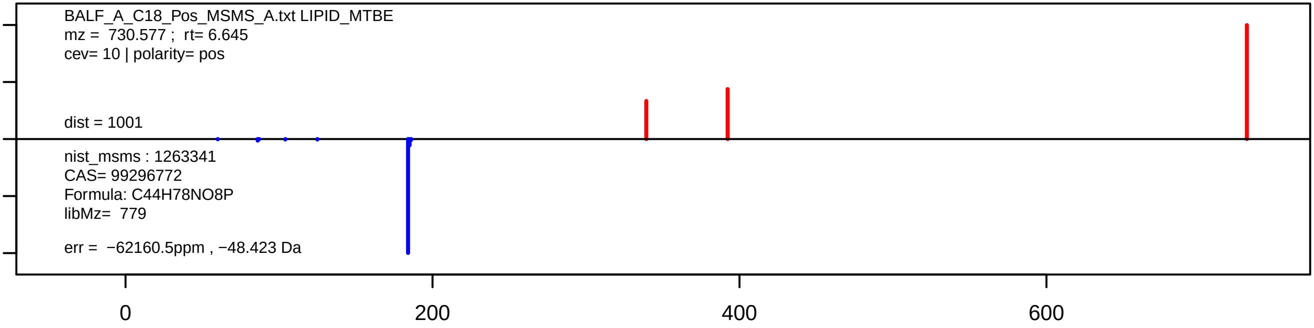

197 . plasmenyl−PE 36:2; [M+H]+; PE(P−18:0/18:2(2E,4E))
Score=547 Dot=859 prob=15.6

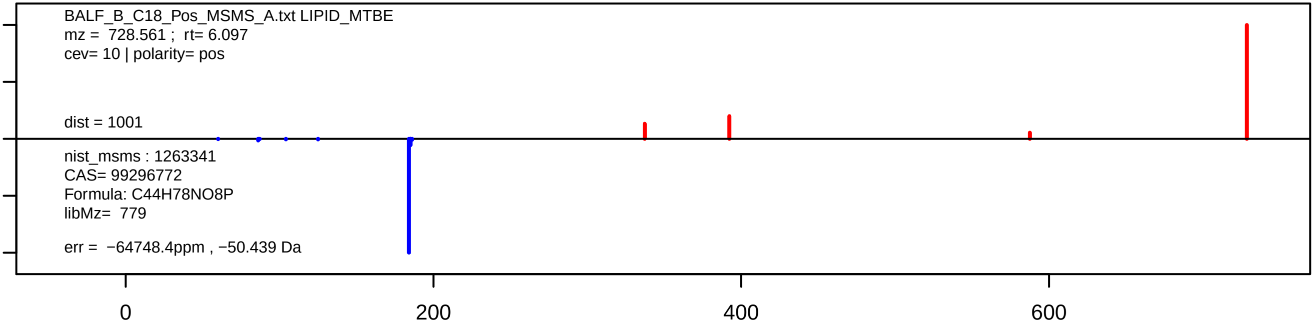

198 . plasmenyl−PE 36:4; [M+H]+; PE(P−16:0/20:4(5E,8E,11E,14E))
Score=418 Dot=826 prob=33.2

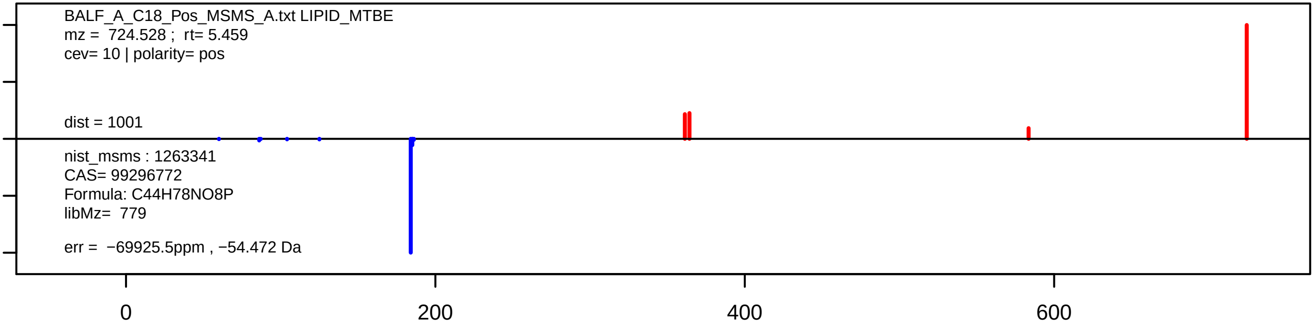

199 . plasmenyl−PE 38:1; [M+H]+; PE(P−20:0/18:1(11E))
Score=524 Dot=834 prob=10.7

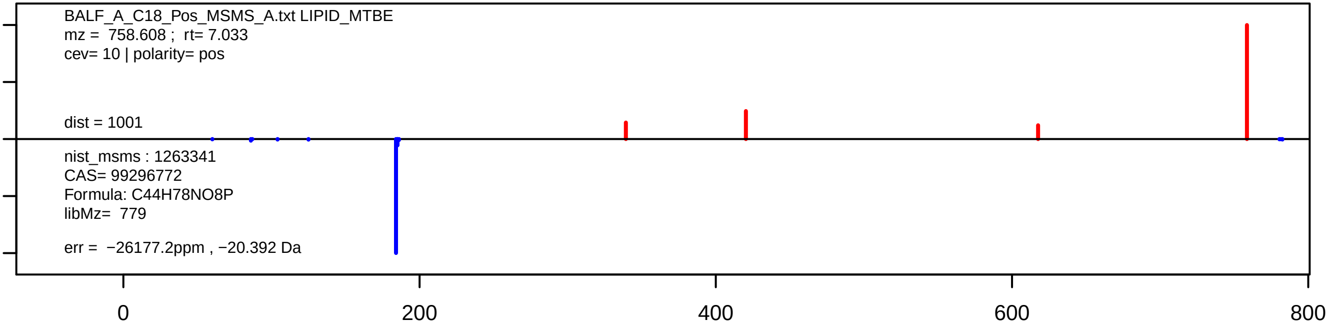

200 . plasmenyl−PE 38:2; [M+H]+; PE(P−20:0/18:2(2E,4E))
Score=182 Dot=821 prob=16.7

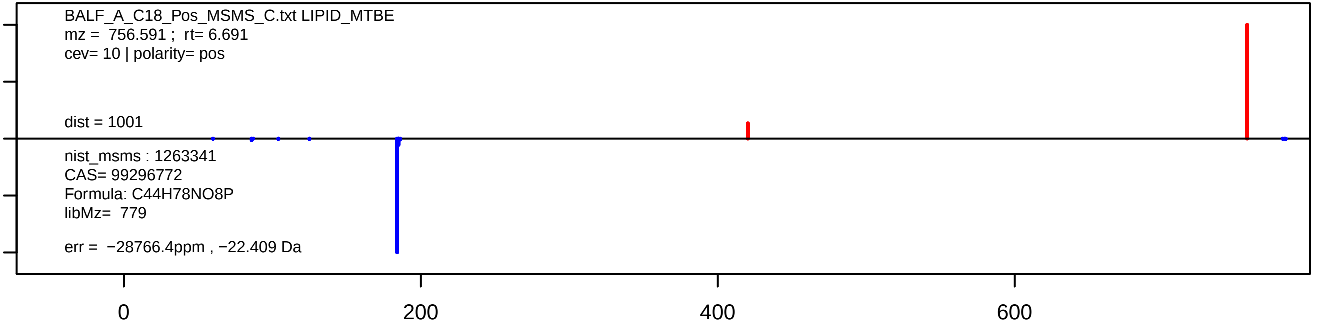

201 . plasmenyl−PE 38:4; [M+H]+; PE(P−16:0/22:4(7Z,10Z,13Z,16Z))
Score=274 Dot=837 prob=97.2

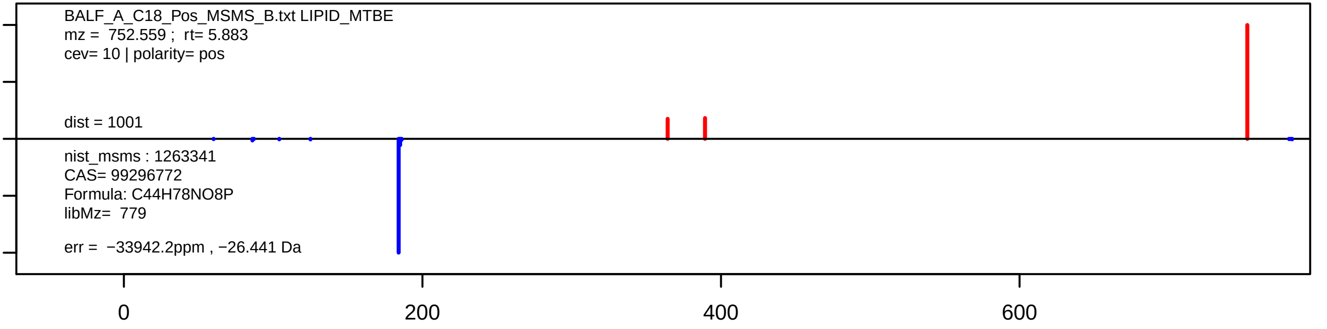

202 . plasmenyl−PE 38:6; [M+H]+; PE(P−16:0/22:6(4Z,7Z,10Z,13Z,16Z,19Z))
Score=176 Dot=810 prob=100

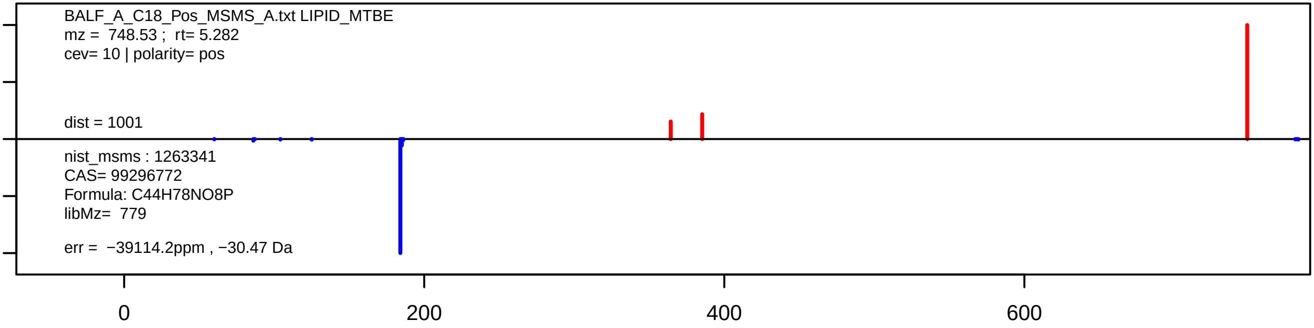

203 . plasmenyl−PE 40:4; [M+H]+; PE(P−18:0/22:4(7Z,10Z,13Z,16Z))
Score=240 Dot=829 prob=97.2

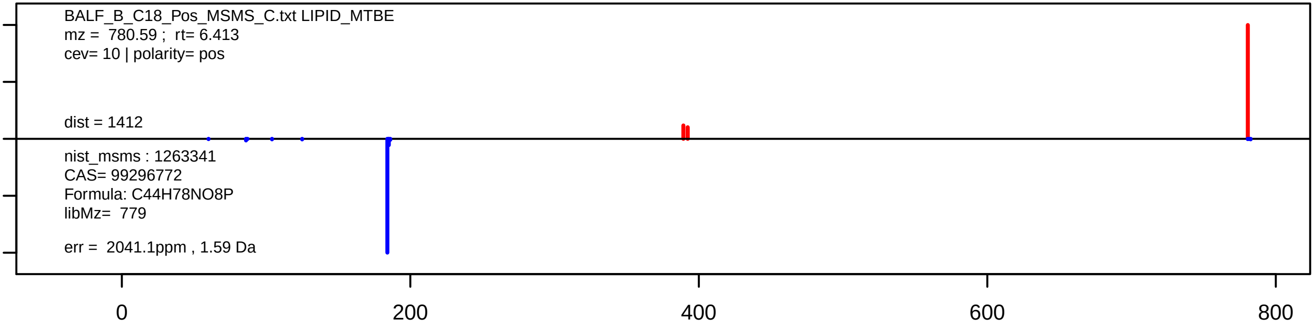

204 . plasmenyl−PE 40:4; [M+H]+; PE(P−20:0/20:4(5E,8E,11E,14E))
Score=395 Dot=808 prob=32.4

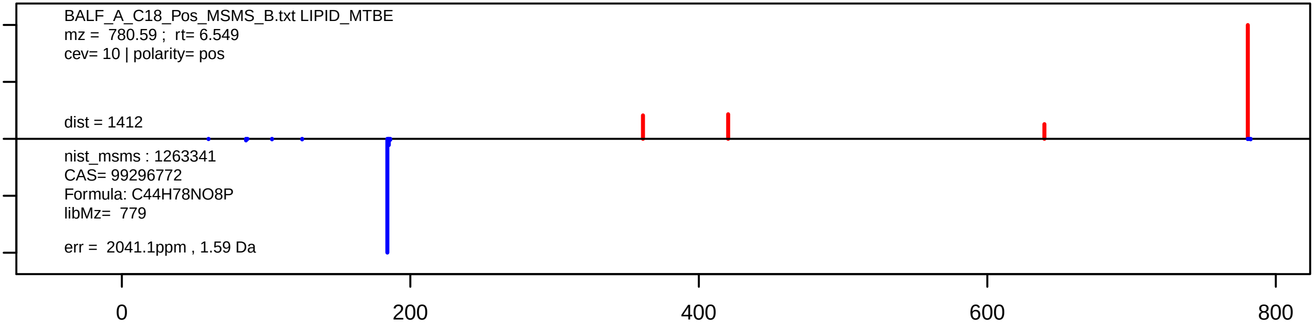

205 . plasmenyl−PE 40:5; [M+H]+; PE(P−18:0/22:5(4Z,7Z,10Z,13Z,16Z))
Score=498 Dot=821 prob=48.1

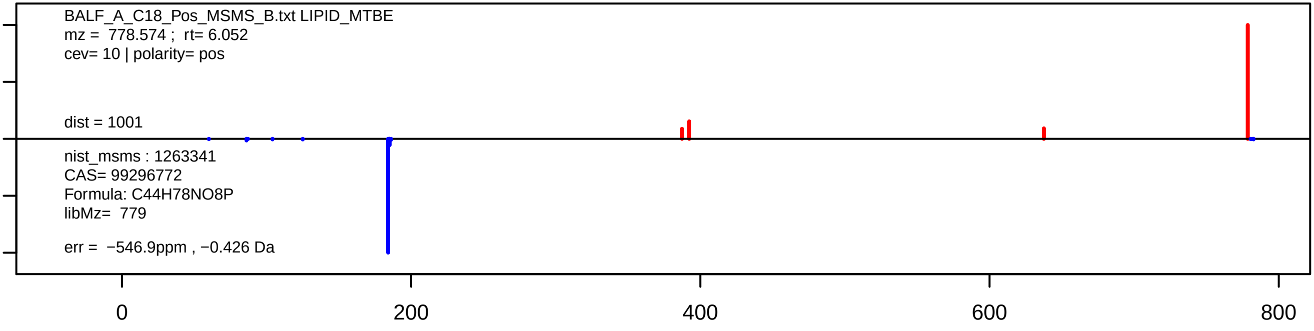

206 . Ramipril
Score=353 Dot=979 prob=98.7

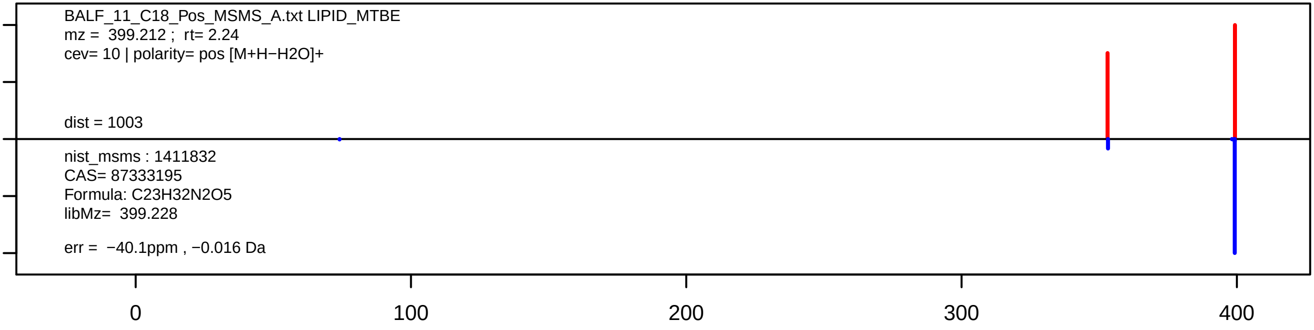

207 . Risperidone
Score=400 Dot=999 prob=96.4

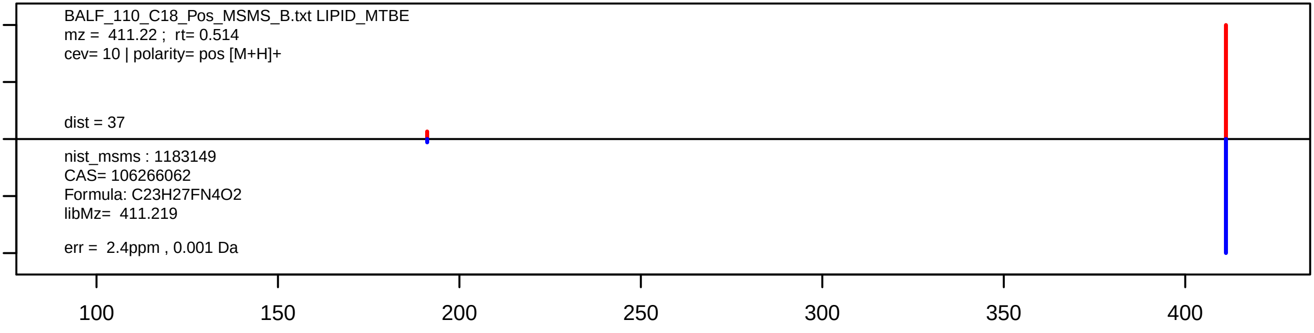

208 . SM 32:1; [M+Na]+; SM(d14:0/18:1(9Z))
Score=366 Dot=990 prob=24.9

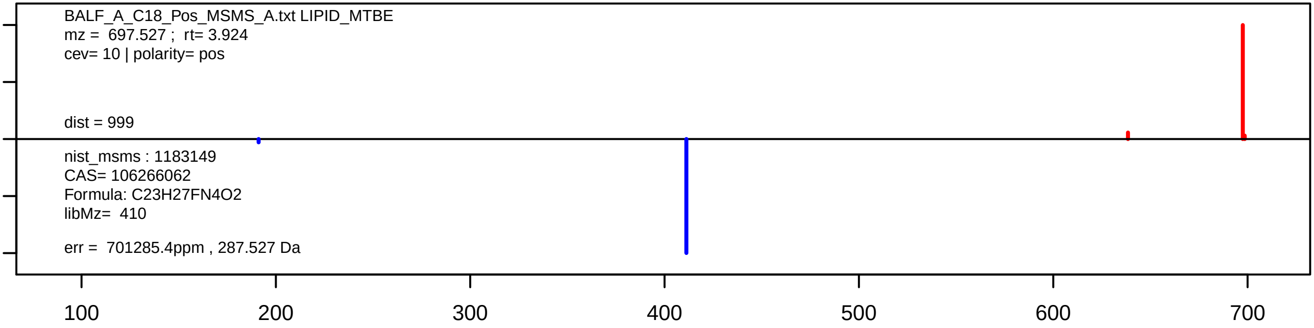

209 . SM 33:1; [M+Na]+; SM(d15:0/18:1(9Z))
Score=366 Dot=990 prob=24.9

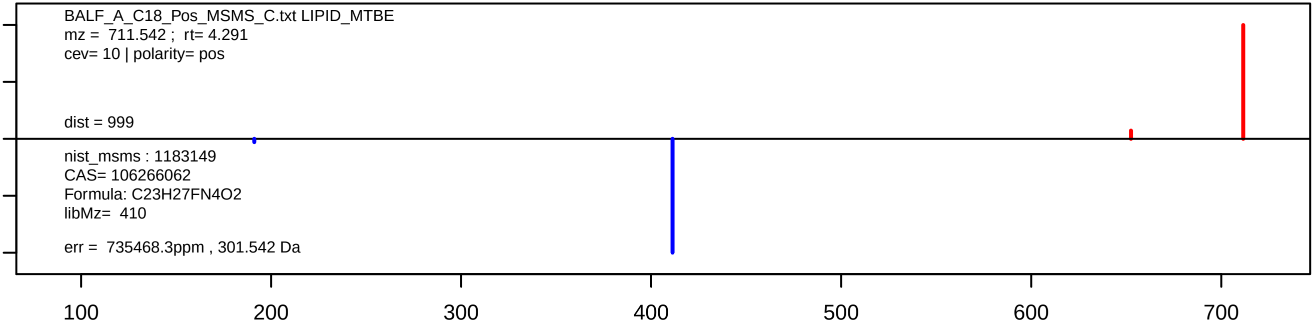

210 . SM 34:1; [M+Na]+; SM(d14:1(4E)/20:0)
Score=366 Dot=990 prob=24.8

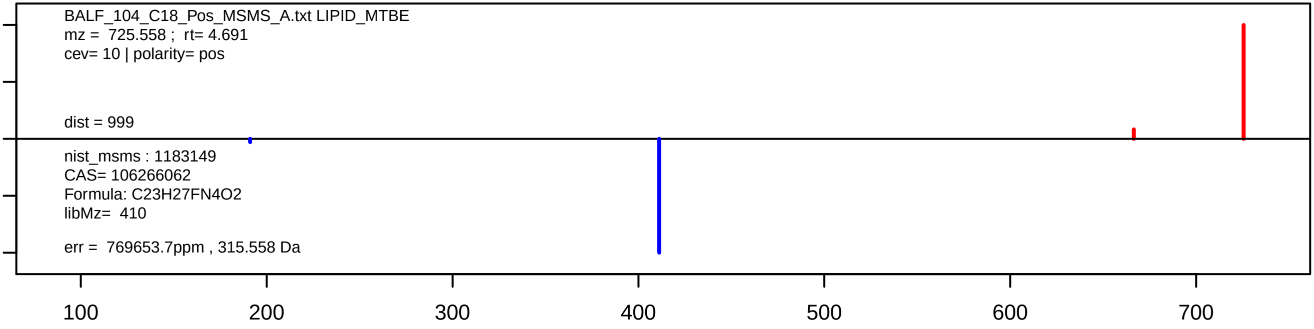

211 .   SM 34:2; [M+Na]+; SM(d16:1(4E)/18:1(9Z))
Score=366 Dot=990 prob=100

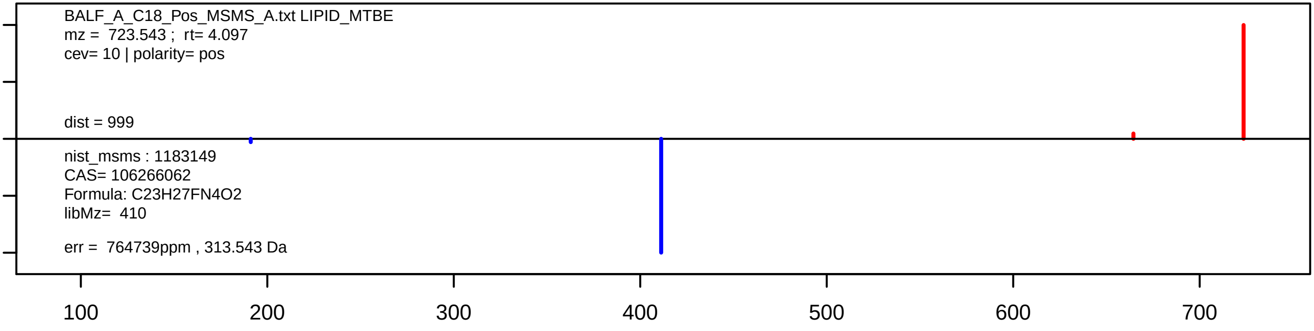

212 .   SM 36:1; [M+Na]+; SM(d14:1(4E)/22:0)
Score=366 Dot=990 prob=24.9

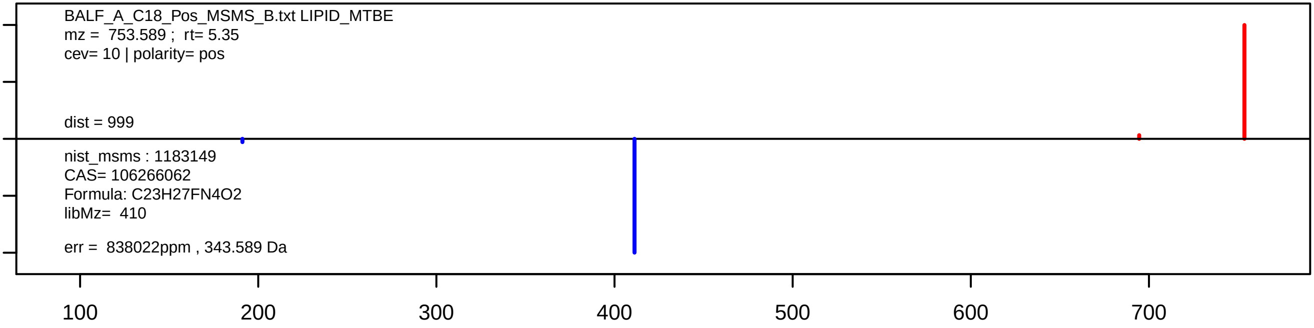

213 .   SM 42:2; [M+Na]+; SM(d16:1(4E)/26:1(17Z))
Score=366 Dot=990 prob=49.8

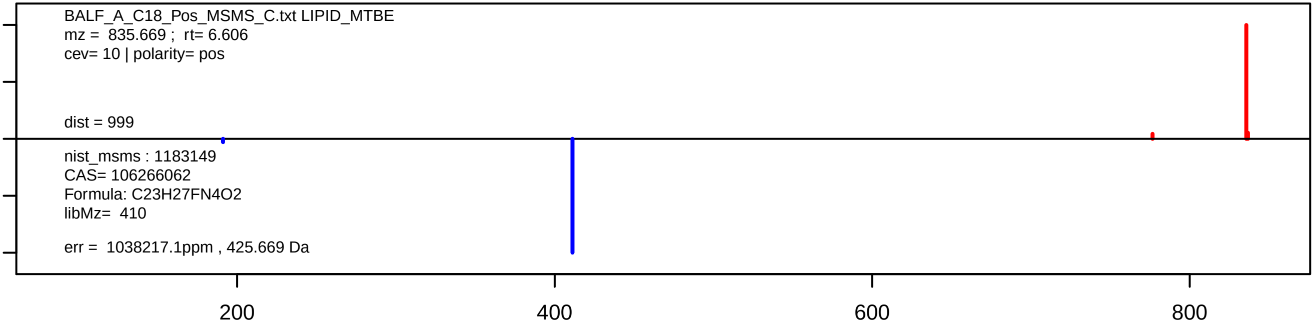

214 . Stearoyl−L−carnitine
Score=435 Dot=819 prob=97.5

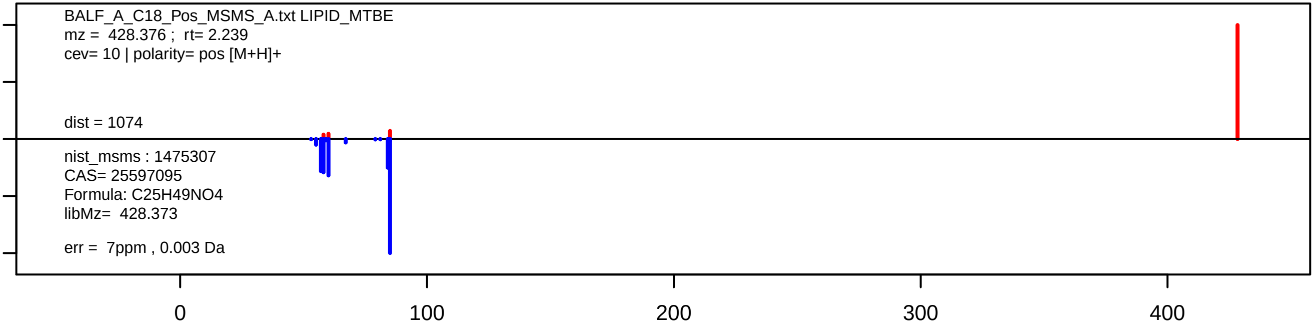

215 . Tetraethylammonium cation
Score=270 Dot=892 prob=7.8

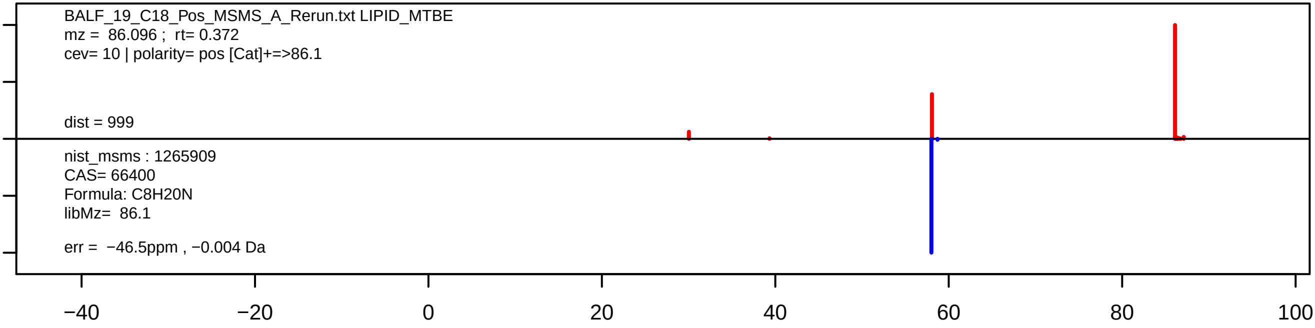

216 . Tetraheptylammonium cation
Score=156 Dot=888 prob=73.7

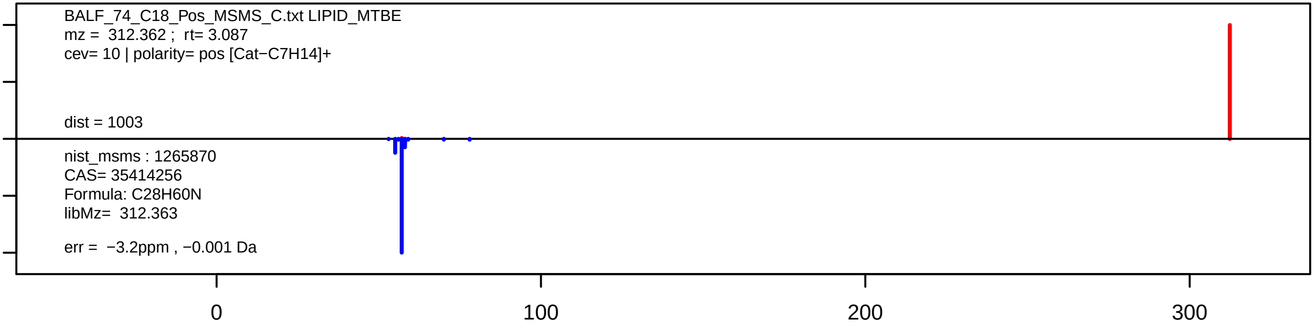

217 . Thioetheramidephosphatidylcholine
Score=394 Dot=862 prob=99

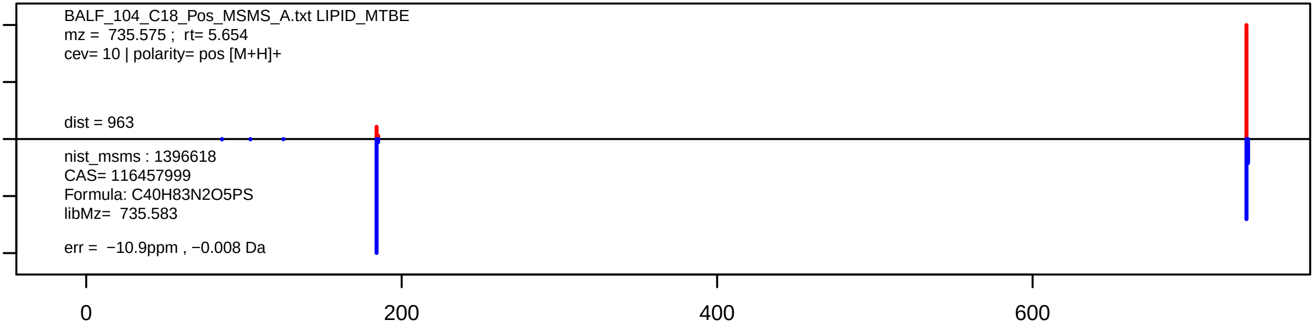

218 . Thr−Val−Leu
Score=459 Dot=968 prob=54.8

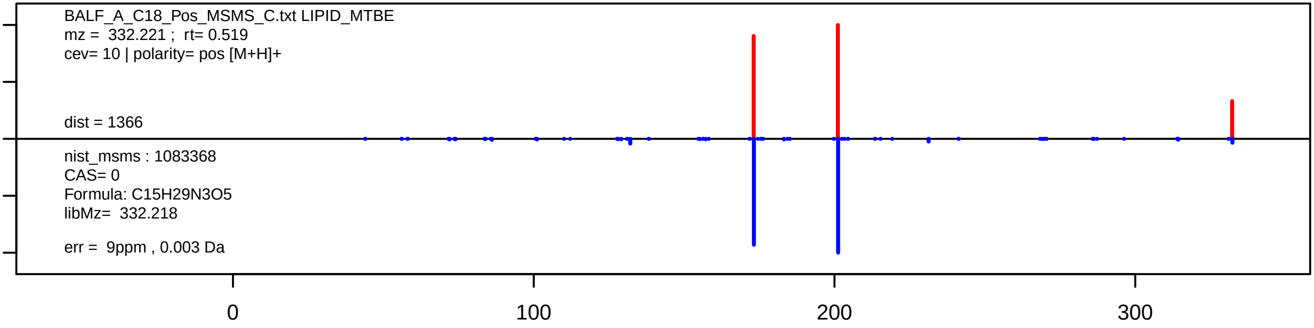

219 . threo−Dihydrobupropion
Score=815 Dot=984 prob=98.4

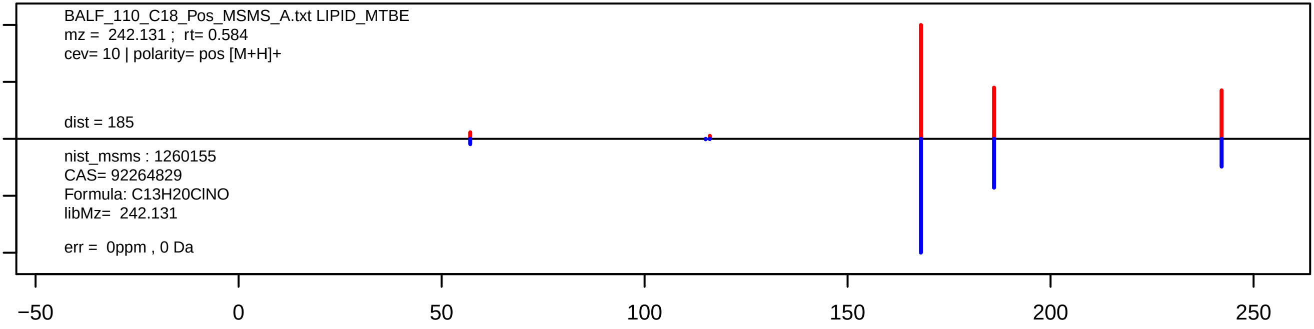

220 . Tramadol
Score=294 Dot=868 prob=81.3

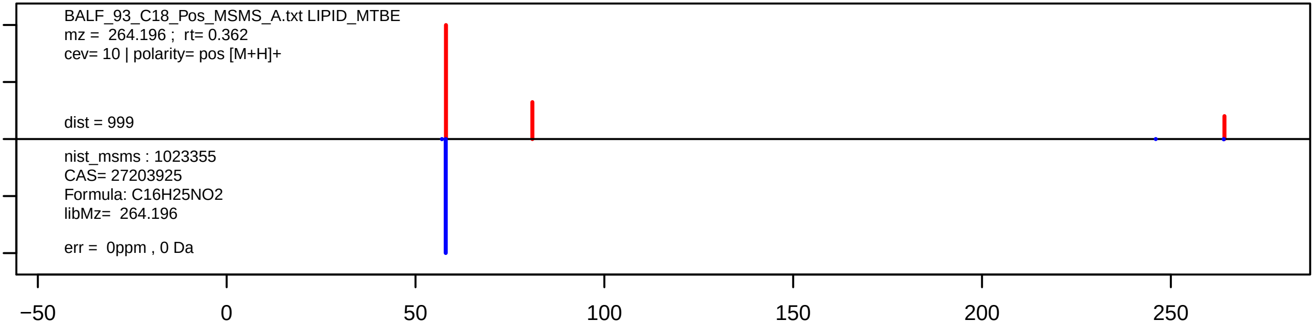

221 . Tributyl phosphate
Score=167 Dot=913 prob=40

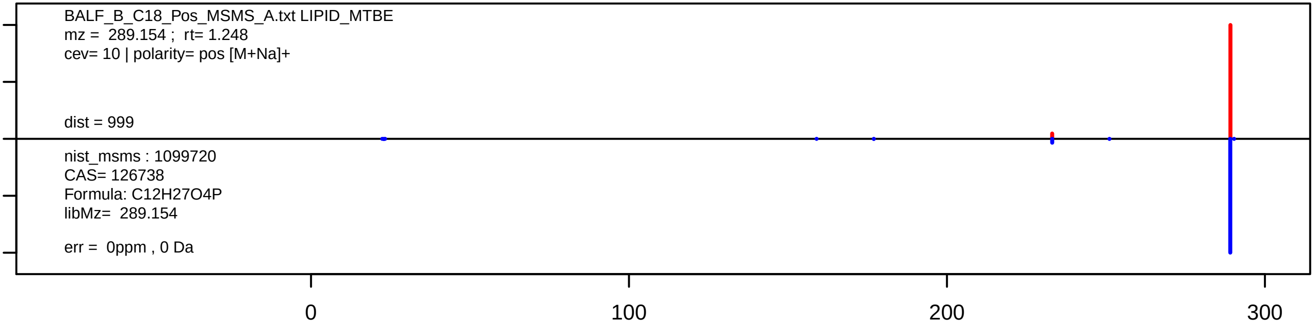

222 . Unoprostone isopropyl ester
Score=335 Dot=815 prob=87.9

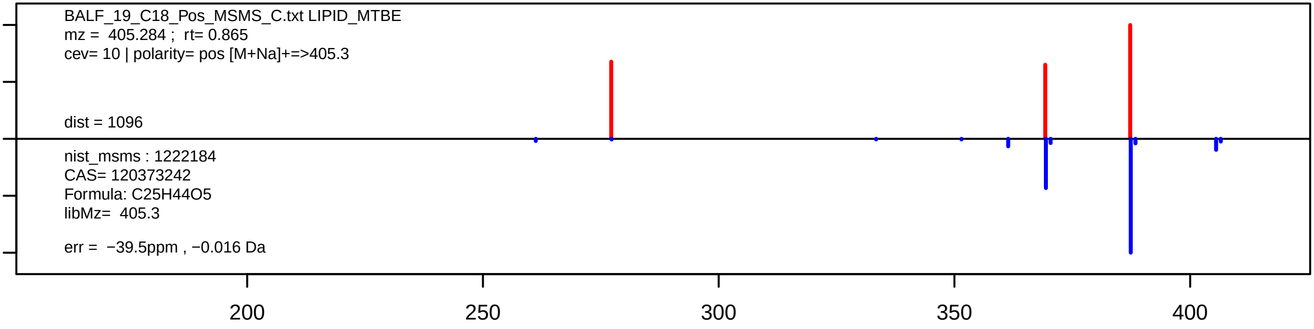

223 .   Venlafaxine
Score=532 Dot=979 prob=98.5

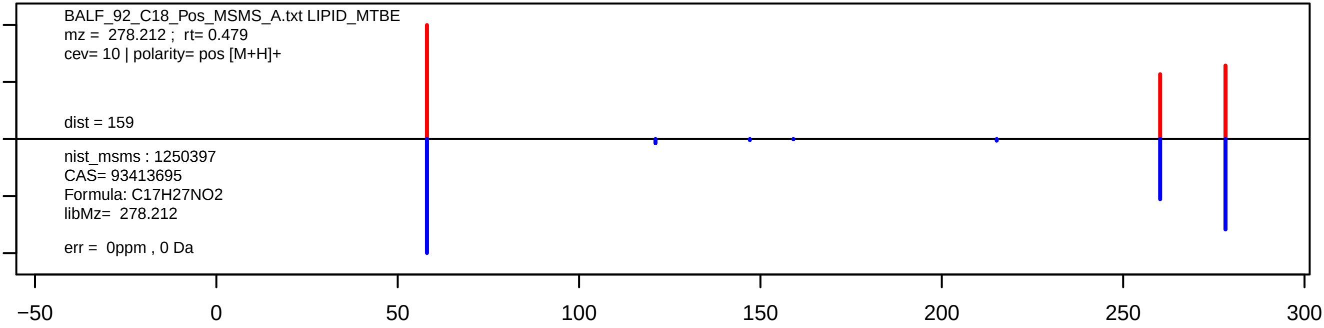

224 .   Zolmitriptan N−oxide
Score=400 Dot=999 prob=96.6

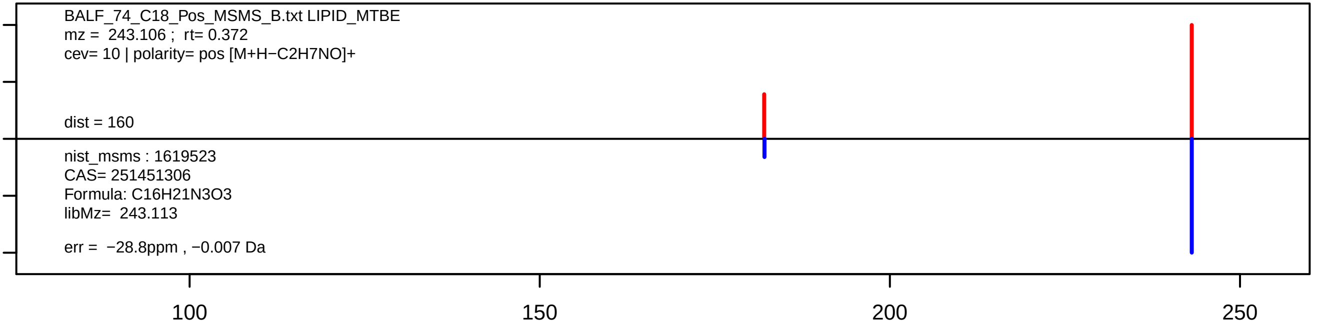

225 .   .beta.−Methylphenethylamine
Score=400 Dot=999 prob=1.3

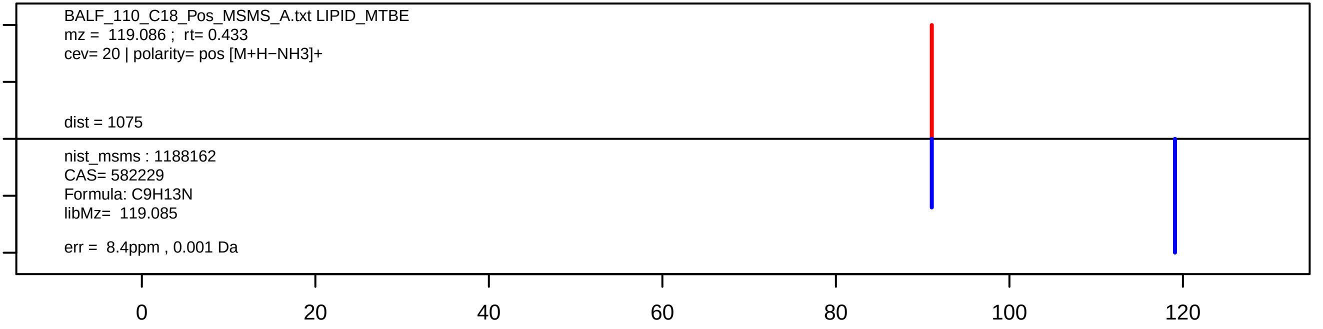

226 . 1−(1Z−Octadecenyl)−2−(4Z,7Z,10Z,13Z,16Z,19Z−docosahexaenoyl)−sn−glycero−3−phosphoethanolamine
Score=483 Dot=873 prob=90.9

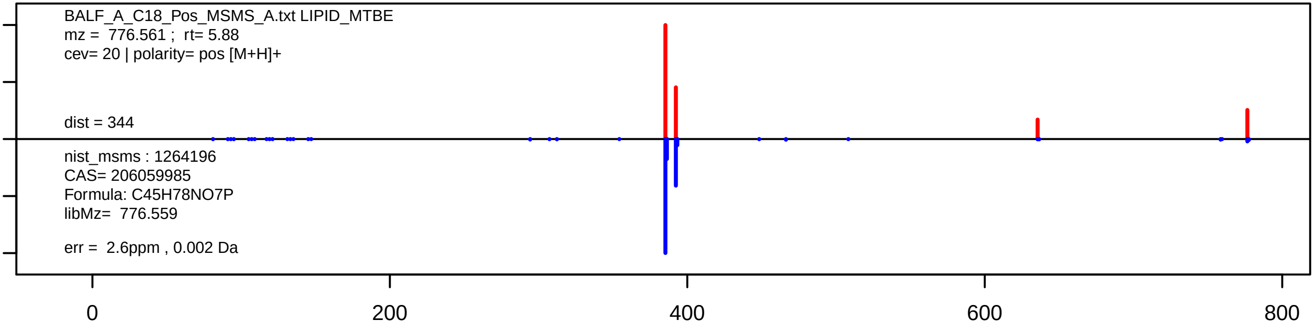

227 . 1−(1Z−Octadecenyl)−2−(5Z,8Z,11Z,14Z−eicosatetraenoyl)−sn−glycero−3−phosphocholine
Score=400 Dot=999 prob=99

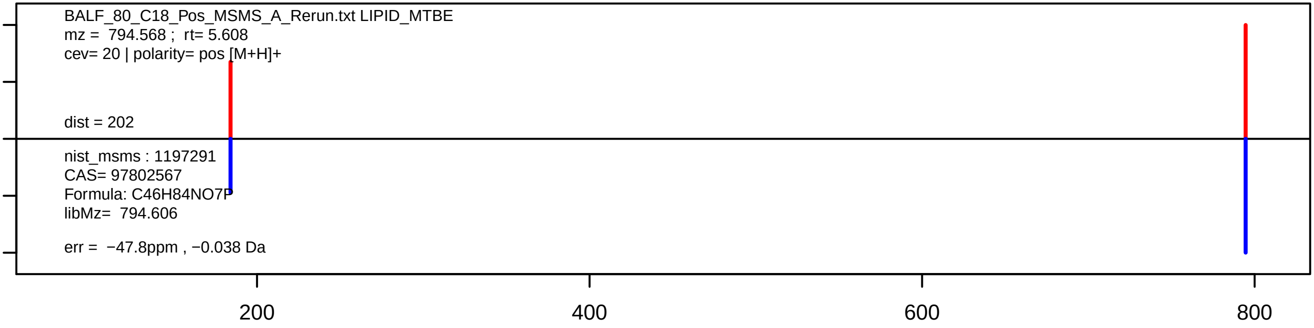

228 . 1−(1Z−Octadecenyl)−2−(5Z,8Z,11Z,14Z−eicosatetraenoyl)−sn−glycero−3−phosphoethanolamine
Score=543 Dot=991 prob=97.1

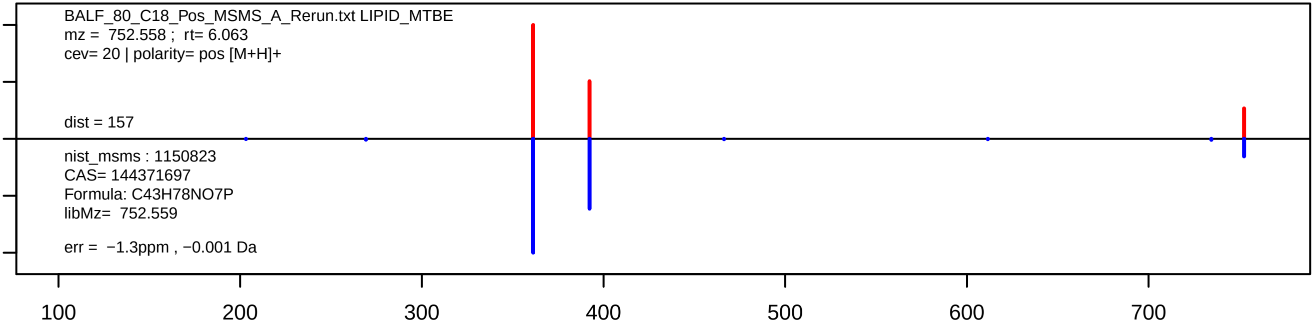

229 . 1−(1Z−Octadecenyl)−2−(9Z−octadecenoyl)−sn−glycero−3−phosphocholine
Score=400 Dot=999 prob=99

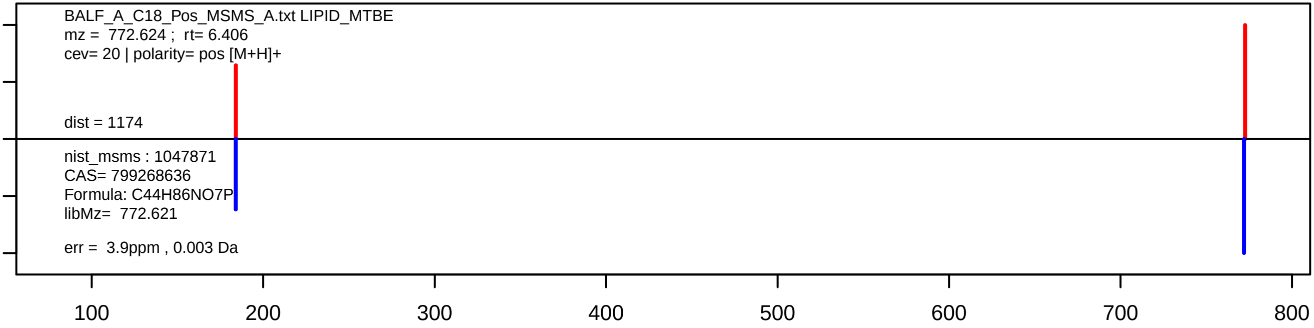

230 . 1−(1Z−Octadecenyl)−sn−glycero−3−phosphocholine
Score=417 Dot=912 prob=98.9

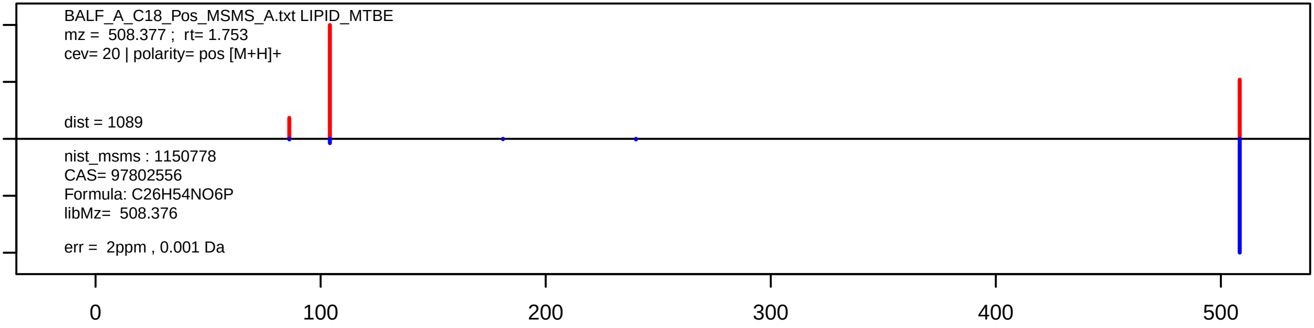

231 . 1−Cyclohexylethanol
Score=701 Dot=943 prob=41.5

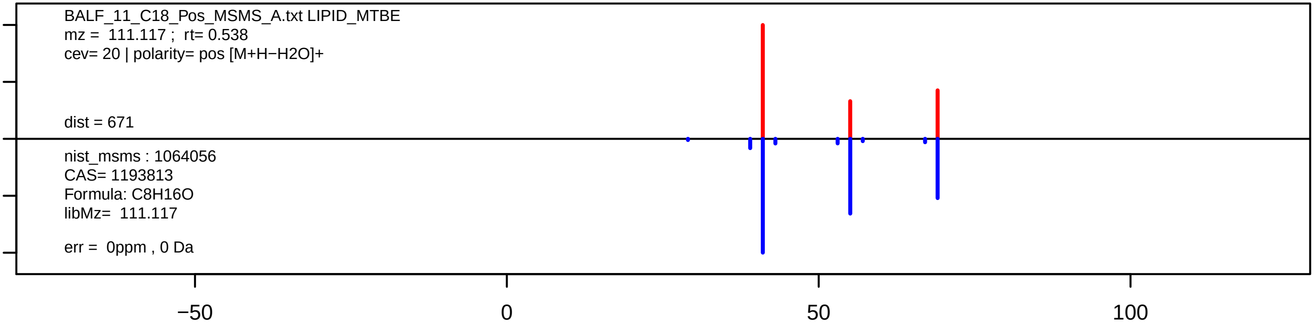

232 .   1−Eicosatrienoyl−sn−glycero−3−phosphoethanolamine
Score=464 Dot=988 prob=48.6

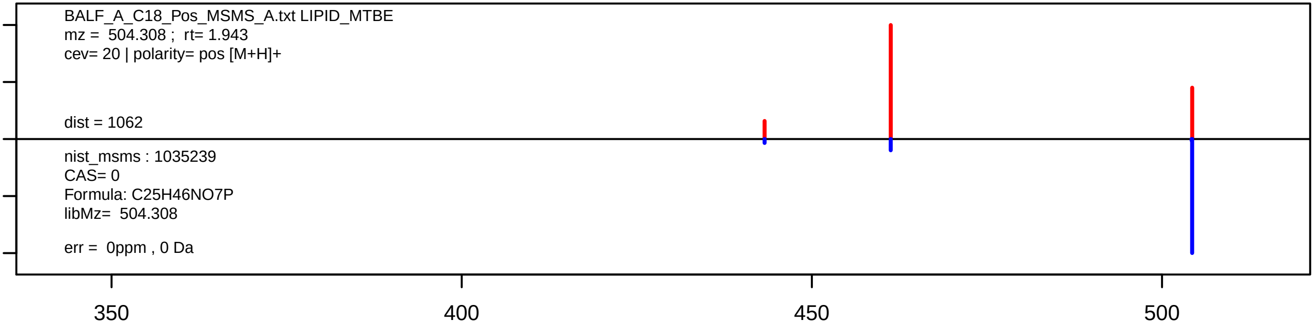

233 .   1−Heneicosanoyl−2−(4Z,7Z,10Z,13Z,16Z,19Z−docosahexaenoyl)−sn−glycero−3−phosphocholine
Score=281 Dot=987 prob=99

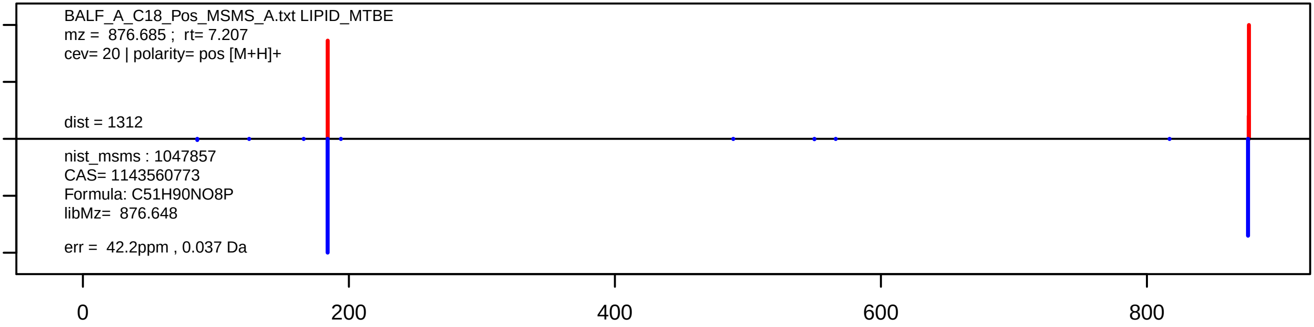

234 .   1−Heptadecanoyl−2−(5Z,8Z,11Z,14Z−eicosatetraenoyl)−sn−glycero−3−phosphocholine
Score=239 Dot=983 prob=100

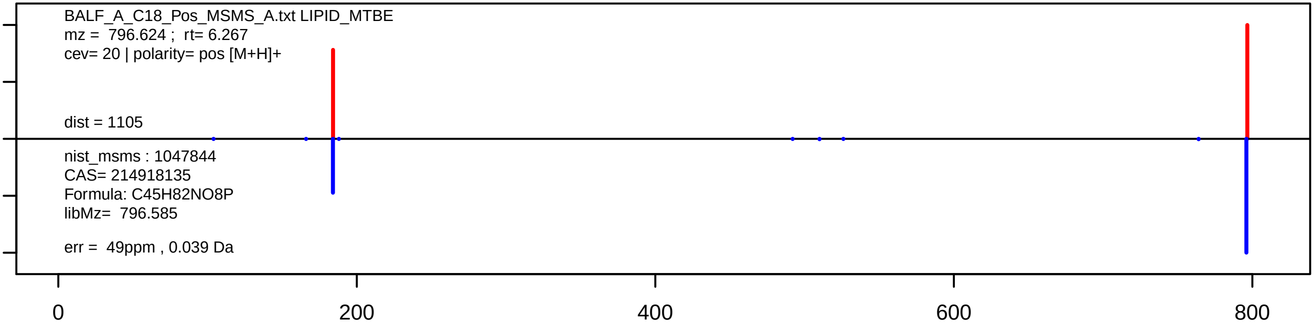

235 . 1−Heptadecanoyl−sn−glycero−3−phosphocholine
Score=486 Dot=946 prob=94.8

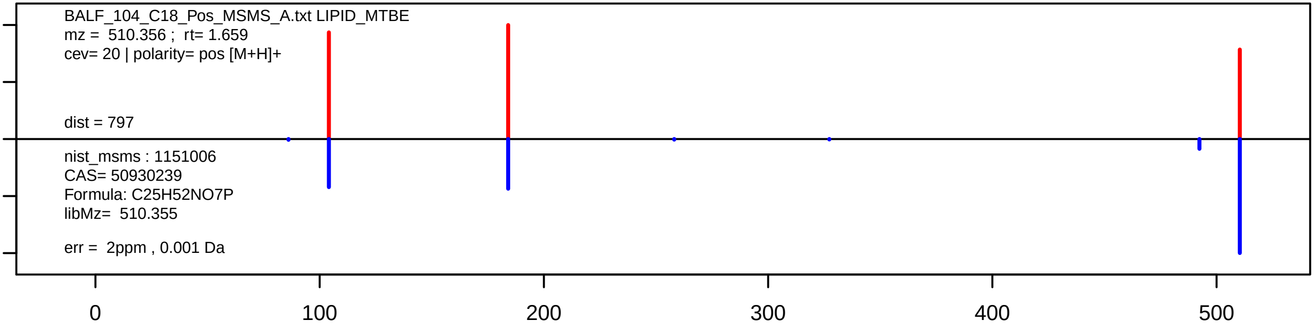

236 . 1−Hexadecanoyl−2−(14−pentadecenoyl)−sn−glycero−3−phosphocholine
Score=295 Dot=991 prob=94

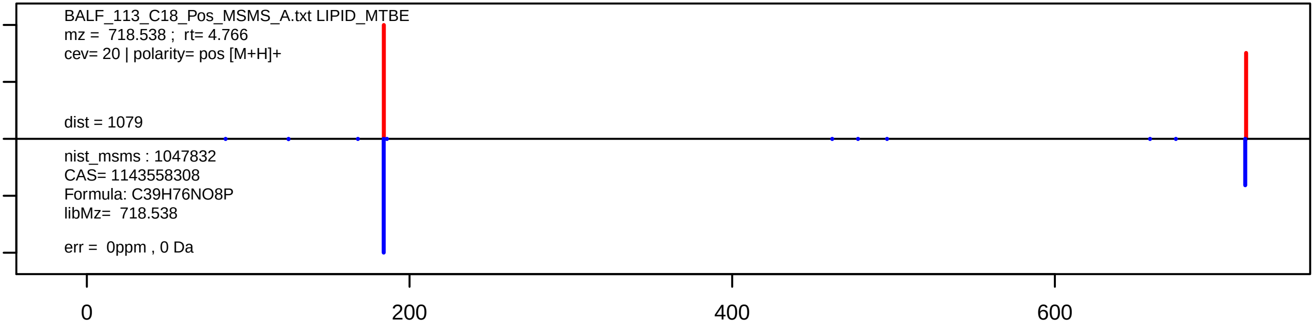

237 . 1−Hexadecanoyl−2−(5Z,8Z,11Z,14Z−eicosatetraenoyl)−sn−glycero−3−phosphocholine
Score=400 Dot=999 prob=97.8

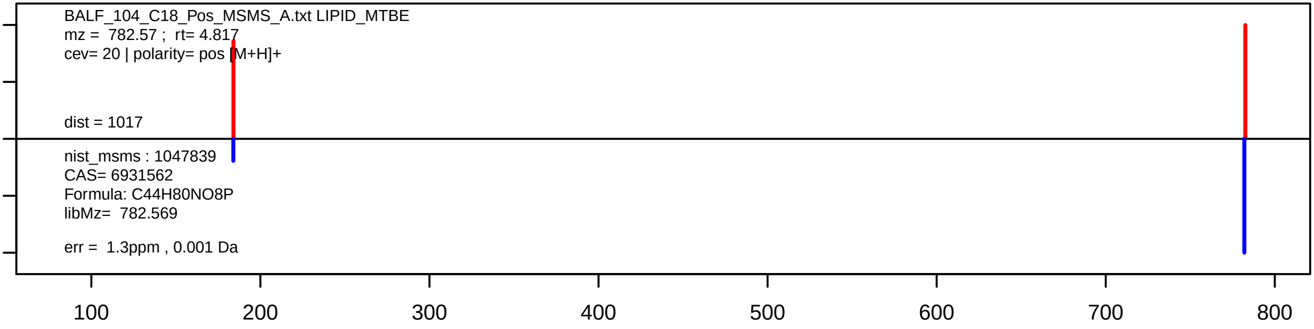

238 . 1−Hexadecanoyl−2−octadecadienoyl−sn−glycero−3−phosphocholine
Score=422 Dot=988 prob=83.1

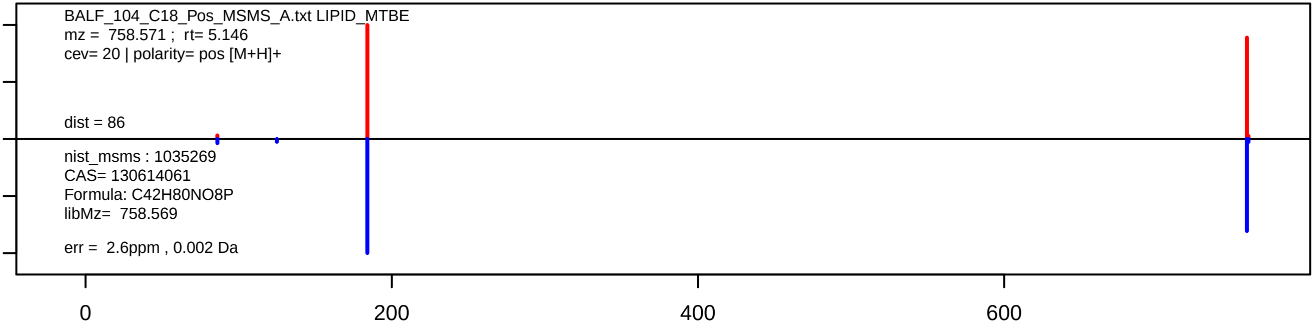

239 . 1−Hexadecyl−2−(5Z,8Z,11Z,14Z−eicosatetraenoyl)−sn−glycero−3−phosphocholine
Score=235 Dot=958 prob=100

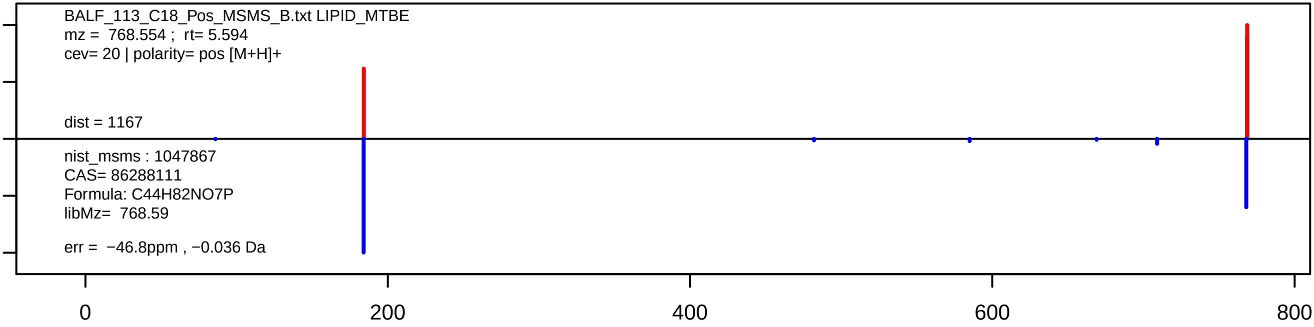

240 . 1−Hexadecyl−2−(8Z,11Z,14Z−eicosatrienoyl)−sn−glycero−3−phosphocholine
Score=145 Dot=856 prob=100

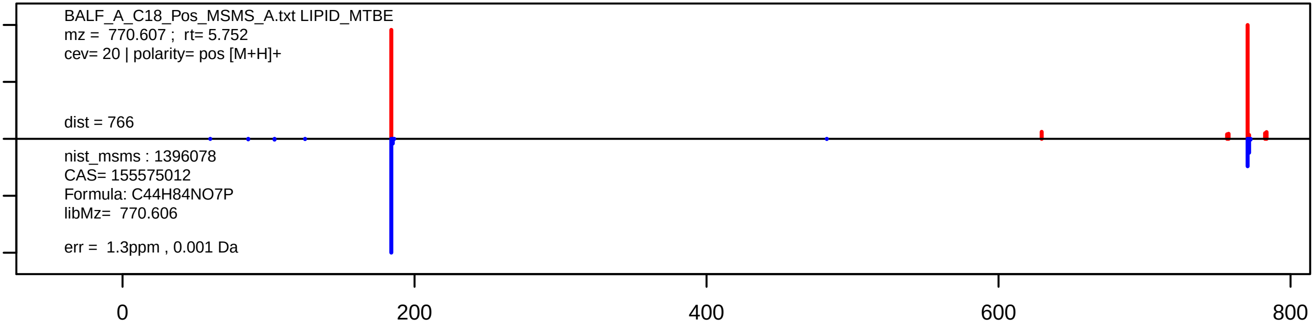

241 . 1−Hexadecyl−2−(9Z−octadecenoyl)−sn−glycero−3−phosphocholine
Score=400 Dot=999 prob=100

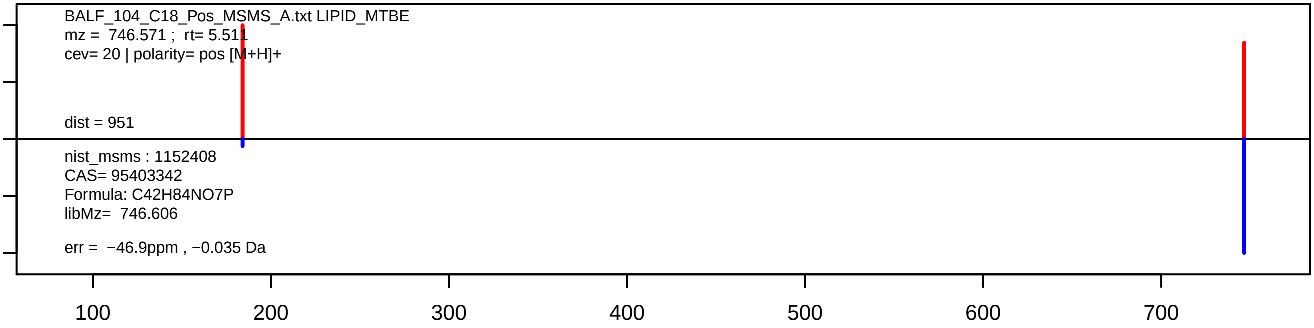

242 . 1−Hexadecyl−sn−glycero−3−phosphocholine
Score=400 Dot=999 prob=99

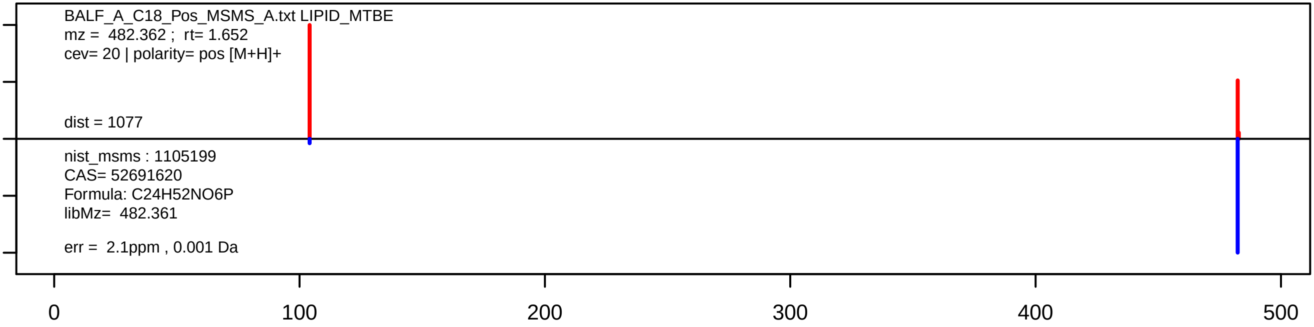

243 . 1−Hexadecylamine
Score=641 Dot=837 prob=98.2

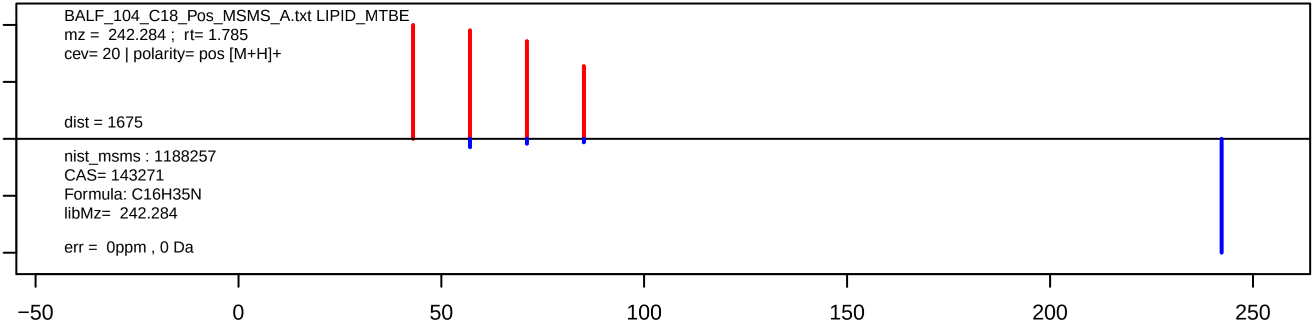

244 . 1−Hexadecylpyridinium cation
Score=232 Dot=837 prob=96.7

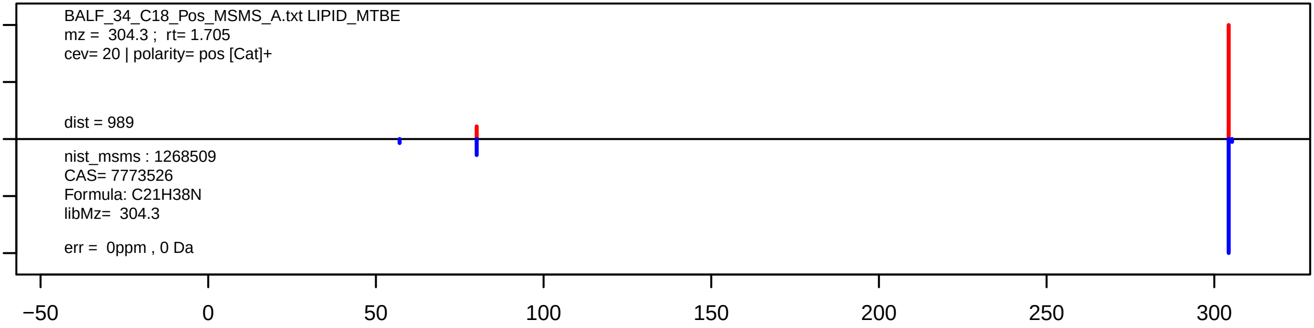

245 . 1−Linoleoyl−2−stearoyl−sn−glycero−3−phospho−(1'−sn−glycerol)
Score=282 Dot=927 prob=48.1

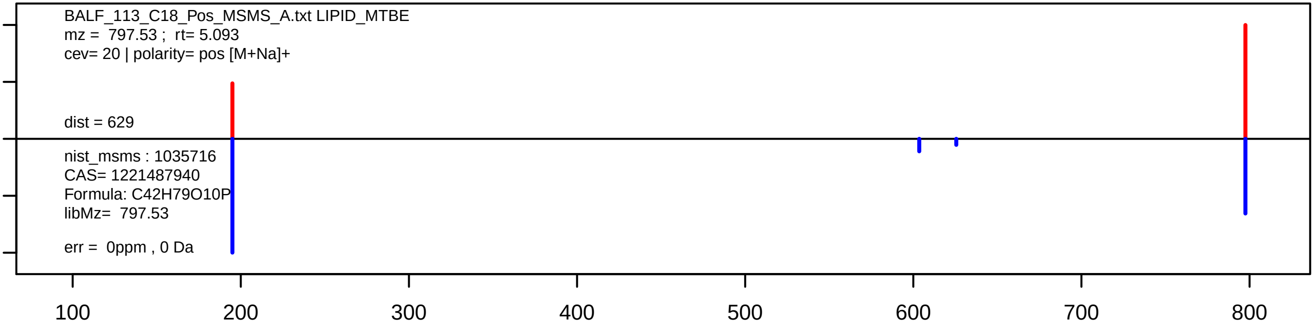

246 . 1−Methyl−3−phenylpropylamine
Score=400 Dot=999 prob=35.7

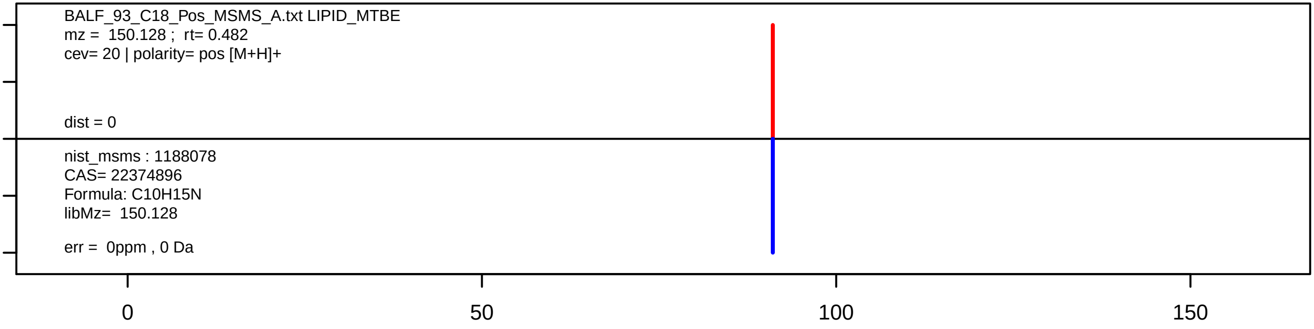

247 .   1−Myristoyl−2−palmitoyl−sn−glycero−3−phosphocholine
Score=200 Dot=927 prob=49.6

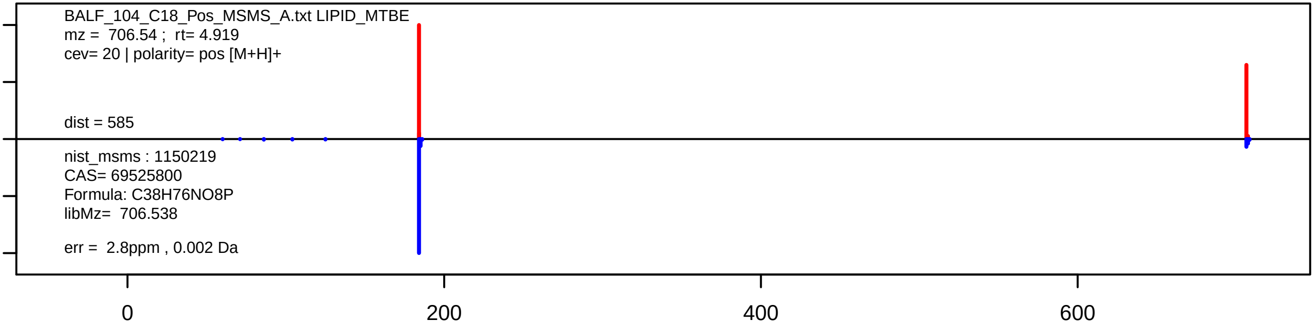

248 .   1−O−Hexadecyl−2−O−(4Z,7Z,10Z,13Z,16Z,19Z−docosahexaenoyl)−sn−glyceryl−3−phosphorylcholine
Score=109 Dot=880 prob=92.5

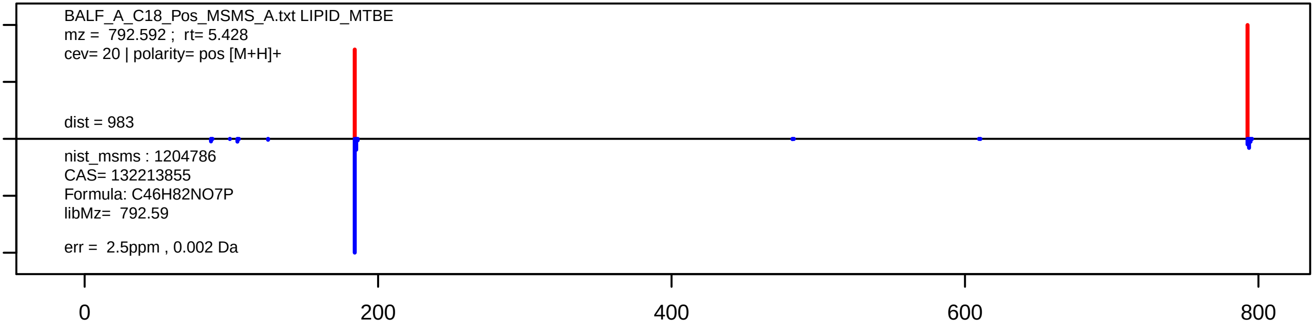

249 .   1−O−Hexadecyl−2−O−(5Z,8Z,11Z,14Z,17Z−eicosapentaenoyl)−sn−glyceryl−3−phosphorylcholine
Score=144 Dot=917 prob=96.8

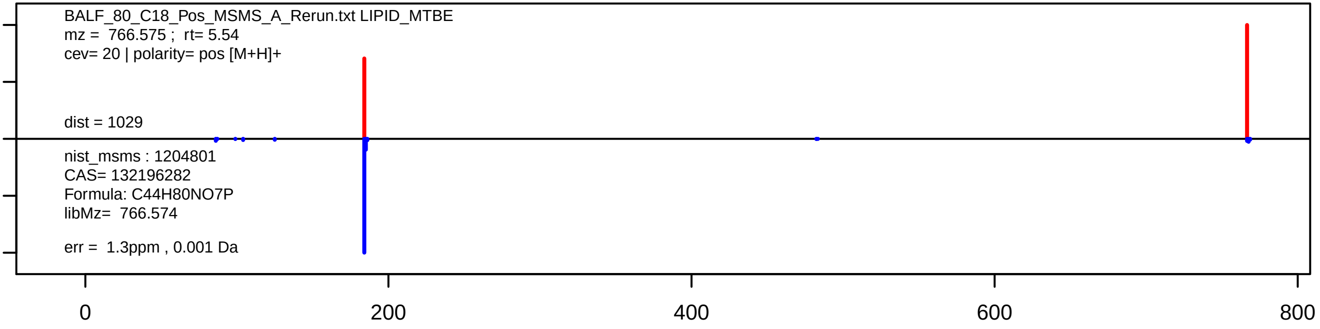

250 . 1−O−Hexadecyl−2−O−acetyl−sn−glyceryl−3−phosphorylcholine
Score=400 Dot=999 prob=98.6

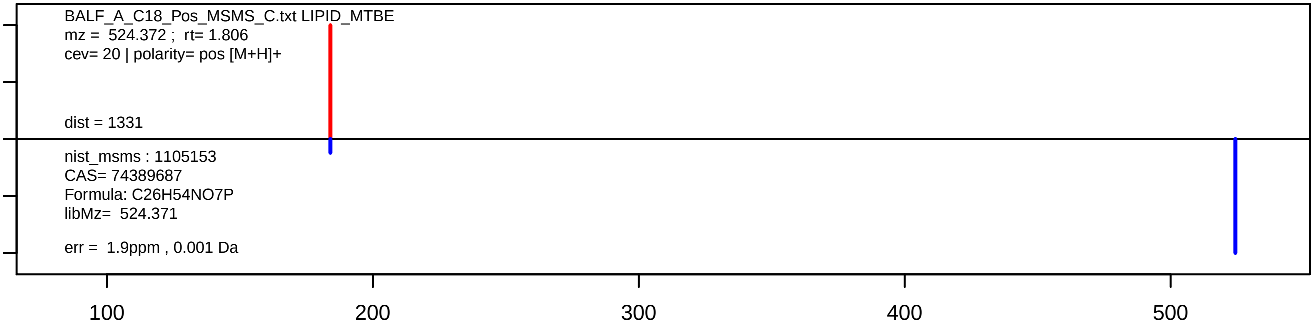

251 . 1−O−Octadecyl−sn−glyceryl−3−phosphorylcholine
Score=399 Dot=999 prob=97.4

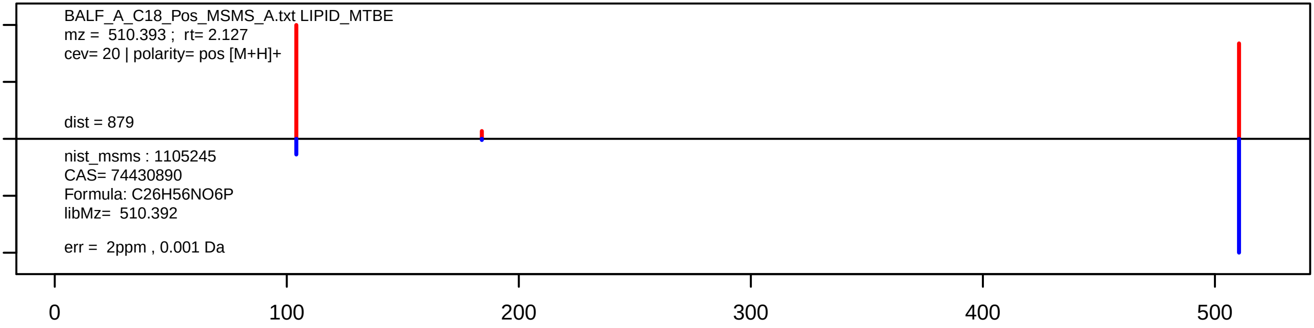

252 . 1−Octen−3−ol
Score=754 Dot=949 prob=70.5

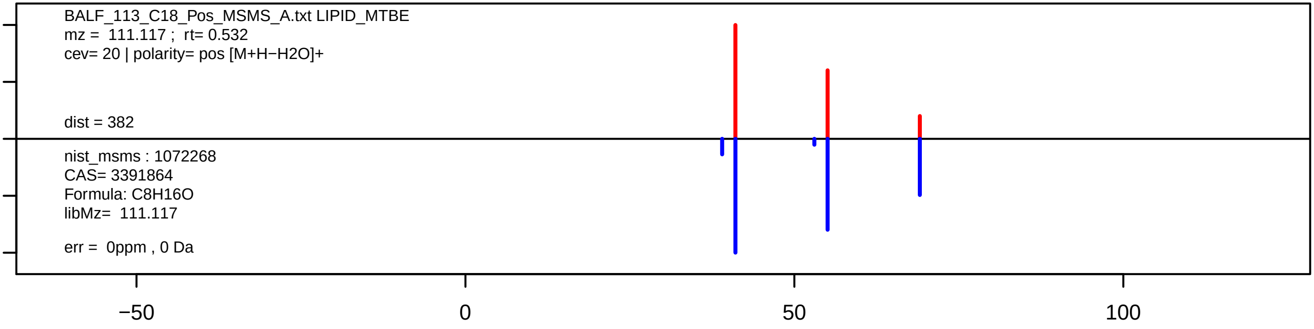

253 . 1−Oleoyl−2−myristoyl−sn−glycero−3−phosphocholine
Score=185 Dot=914 prob=77.6

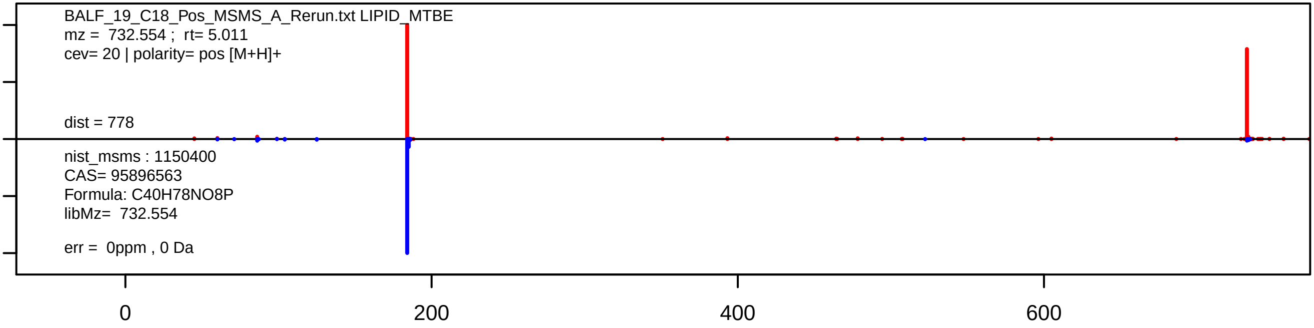

254 . 1−Oleoyl−2−palmitoyl−sn−glycero−3−phosphocholine
Score=356 Dot=970 prob=60.8

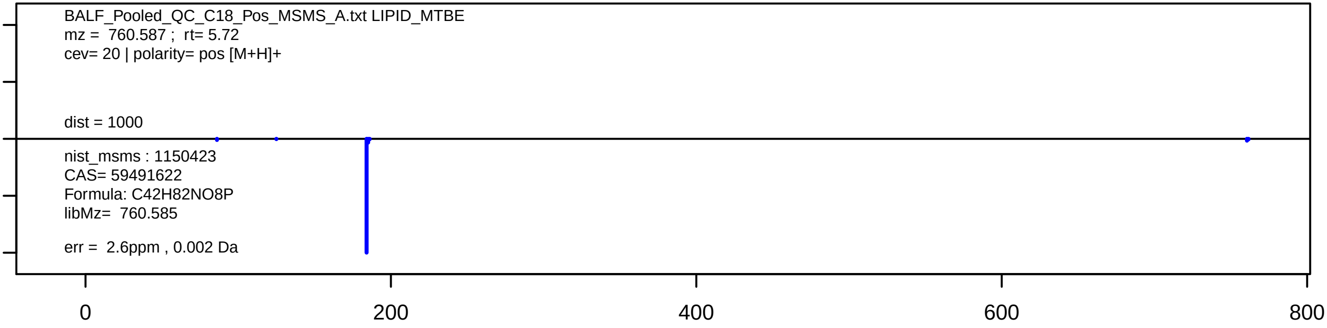

255 . 1−Oleoyl−sn−glycero−3−phosphocholine
Score=615 Dot=906 prob=96.6

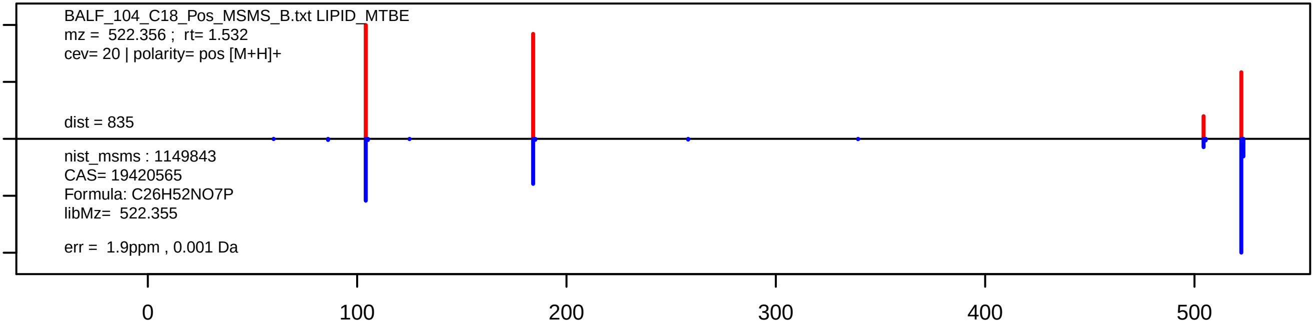

256 . 1−Oleoyl−sn−glycero−3−phosphoethanolamine
Score=488 Dot=816 prob=96.6

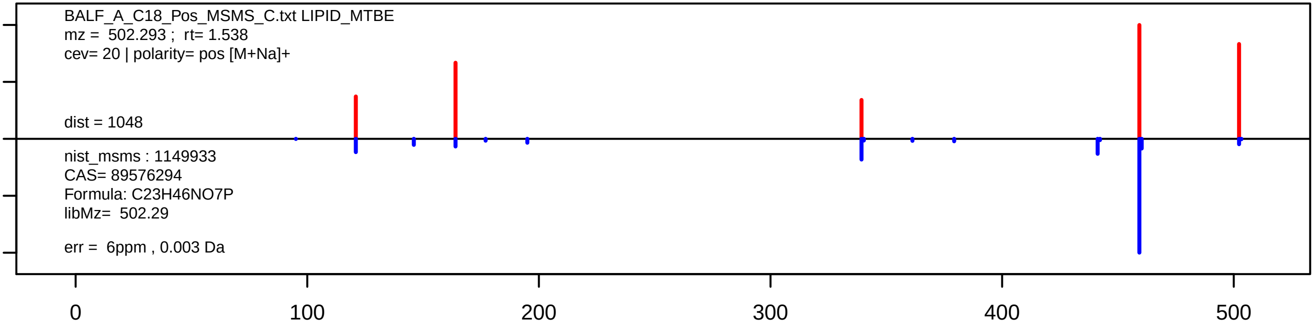

257 . 1−Palmitoyl−2−arachidonoyl−sn−glycero−3−phospho−(1'−sn−glycerol)
Score=96 Dot=895 prob=55.6

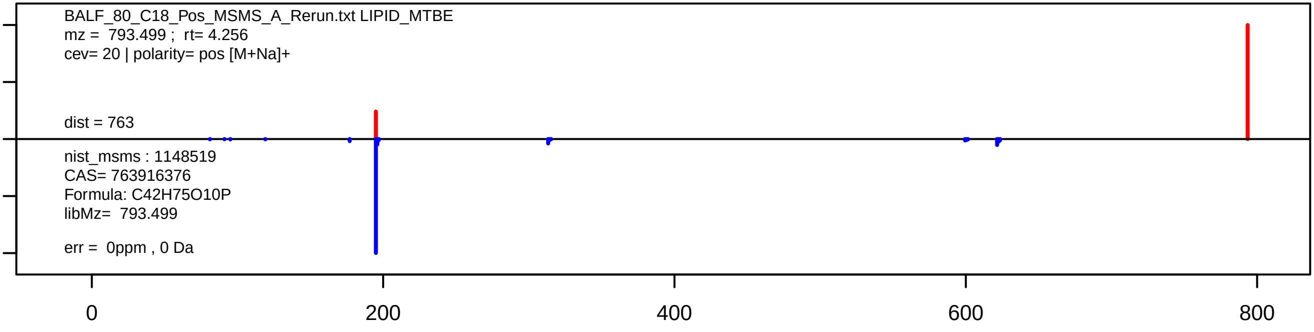

258 . 1−Palmitoyl−2−docosahexaenoyl−sn−glycero−3−phosphocholine
Score=400 Dot=999 prob=100

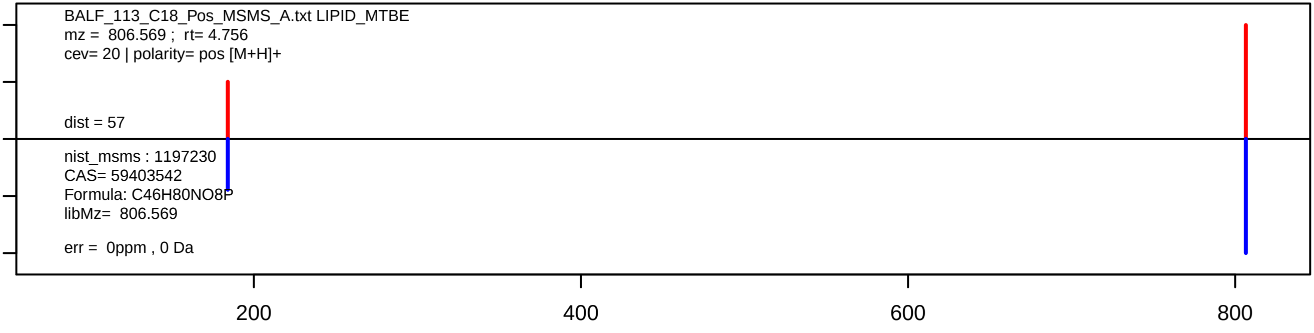

259 . 1−Palmitoyl−2−hydroxy−sn−glycero−3−phosphoethanolamine
Score=381 Dot=841 prob=89.2

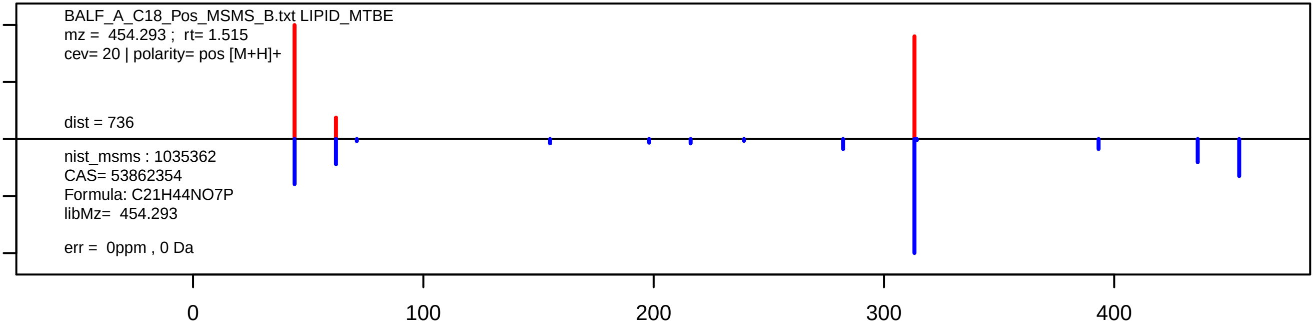

260 . 1−palmitoyl−2−linoleoyl−sn−glycero−3−phospho−(1'−rac−glycerol)
Score=296 Dot=948 prob=98.5

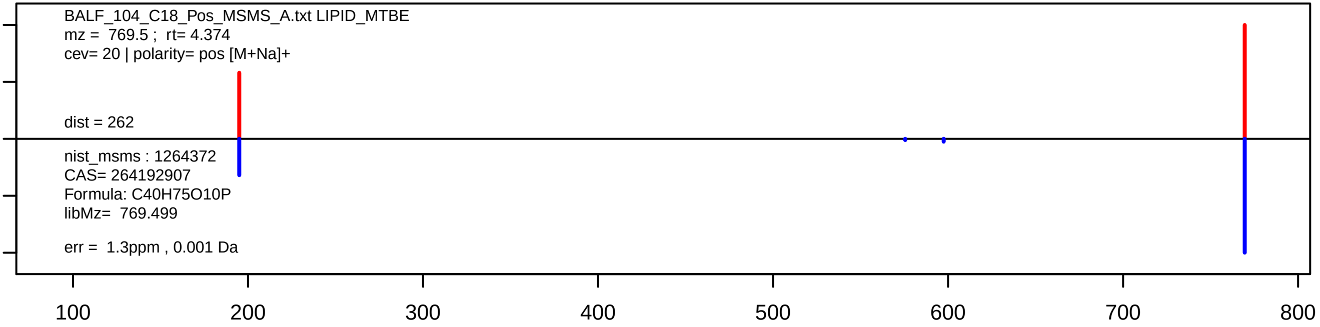

261 . 1−Palmitoyl−2−linoleoyl−sn−glycero−3−phosphocholine
Score=399 Dot=999 prob=82.7

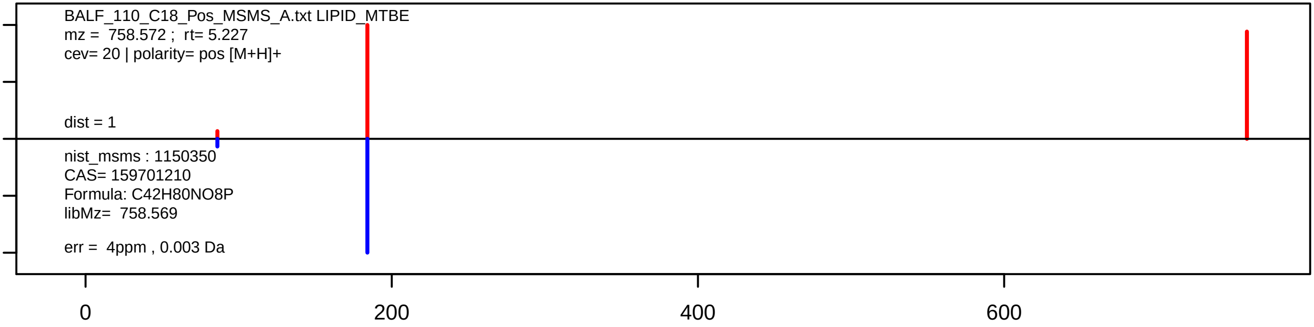

262 . 1−Palmitoyl−2−myristoyl−sn−glycero−3−phosphocholine
Score=241 Dot=949 prob=49

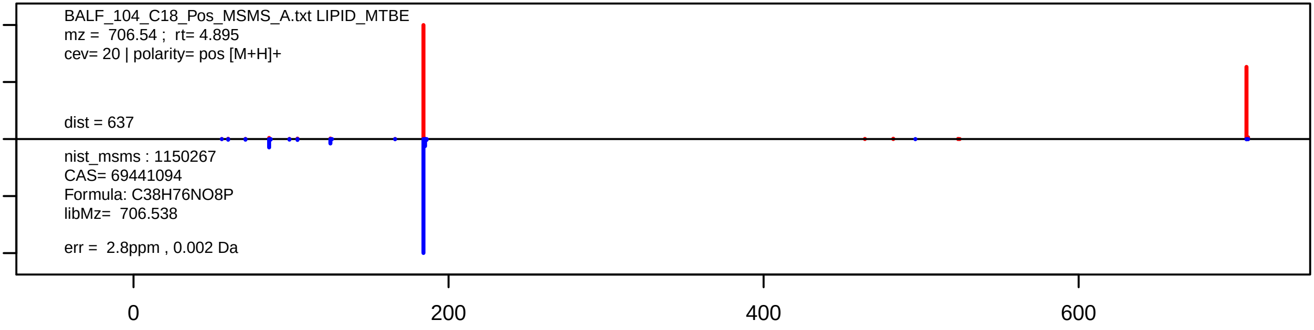

263 . 1−Palmitoyl−2−oleoyl−phosphatidylglycerol
Score=400 Dot=999 prob=100

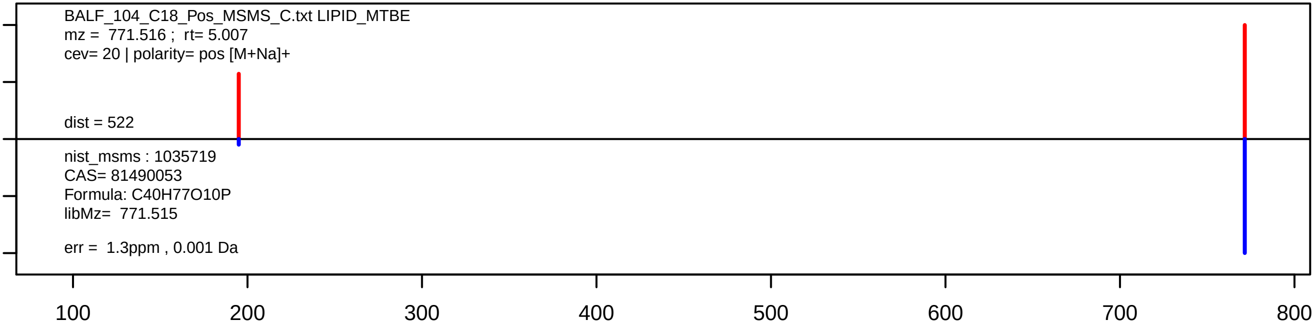

264 . 1−Palmitoyl−2−oleoyl−sn−glycero−3−phosphoethanolamine
Score=295 Dot=818 prob=10.1

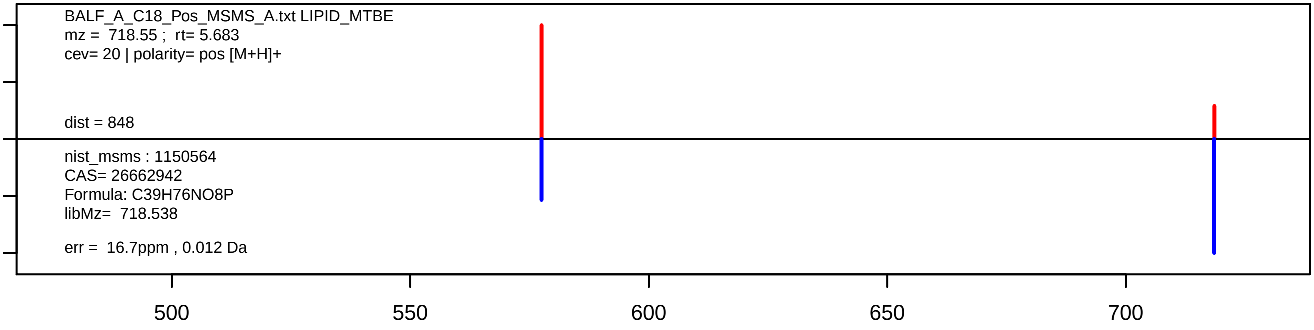

265 . 1−Palmitoyl−3−oleoyl−sn−glycero−2−phosphoethanolamine
Score=400 Dot=999 prob=9.4

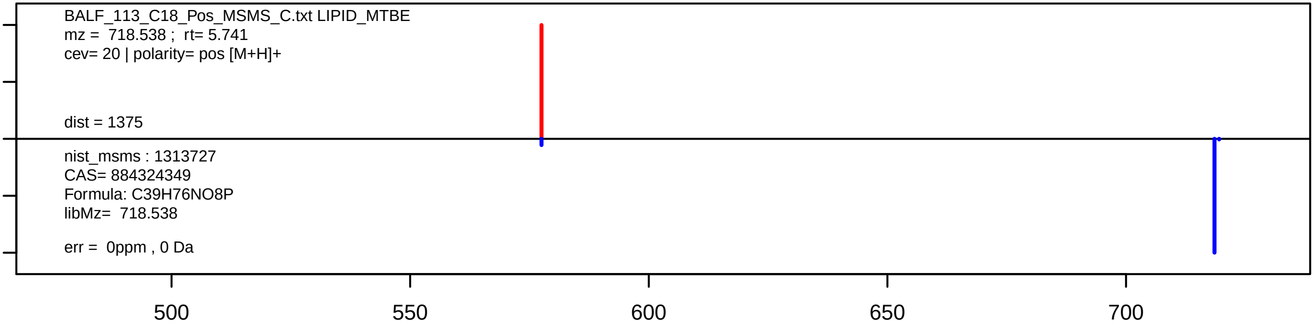

266 . 1−Palmitoyl−sn−glycero−3−phosphocholine
Score=531 Dot=981 prob=98.7

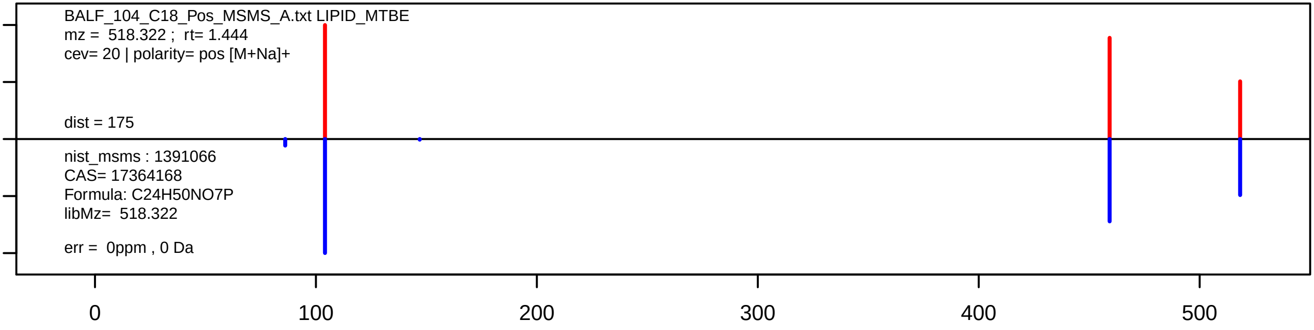

267 . 1−Pentadecanoyl−sn−glycero−3−phosphocholine
Score=598 Dot=974 prob=98.6

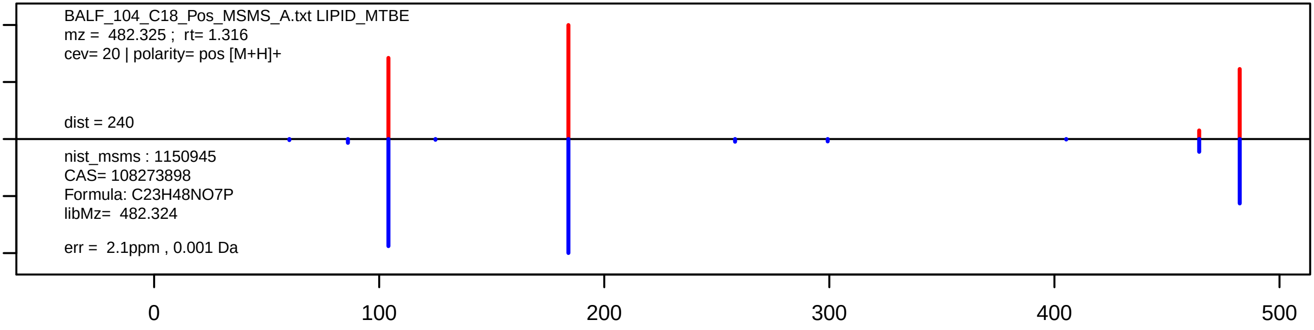

268 . 1−Propanol
Score=486 Dot=912 prob=97.8

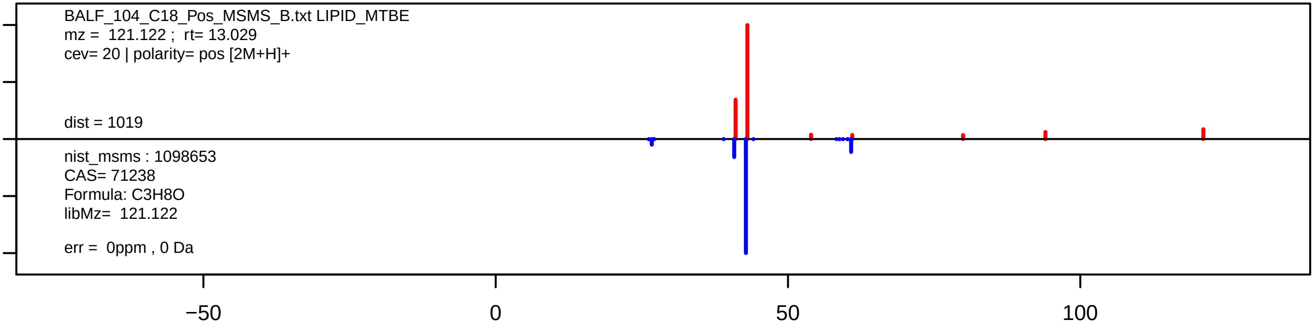

269 . 1−Propanone, 1−(1,3−benzodioxol−5−yl)−2−(dimethylamino)−
Score=268 Dot=810 prob=96.4

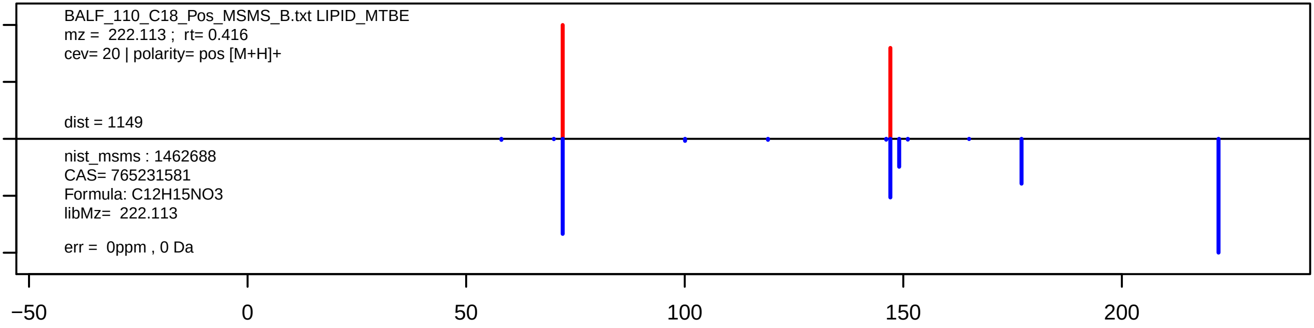

270 . 1−Stearoyl−2−arachidonyl−sn−glycero−3−phosphocholine
Score=166 Dot=931 prob=61.5

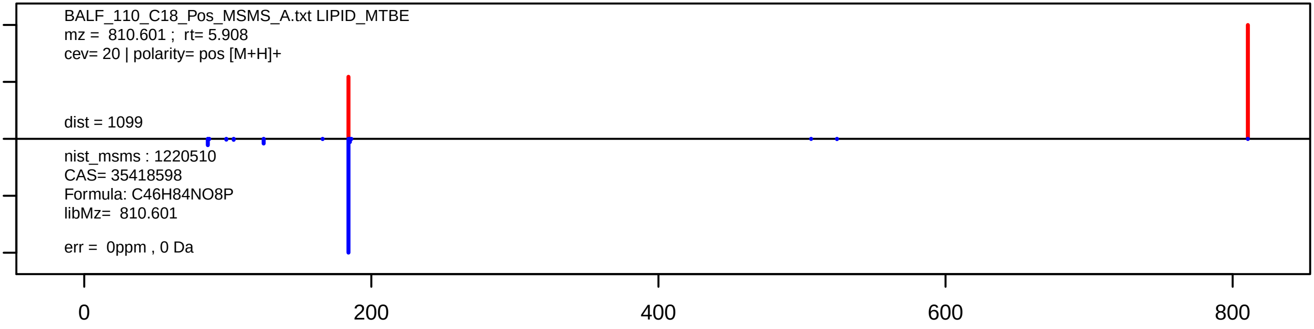

271 . 1−Stearoyl−2−hydroxy−sn−glycero−3−phosphocholine
Score=458 Dot=945 prob=97.9

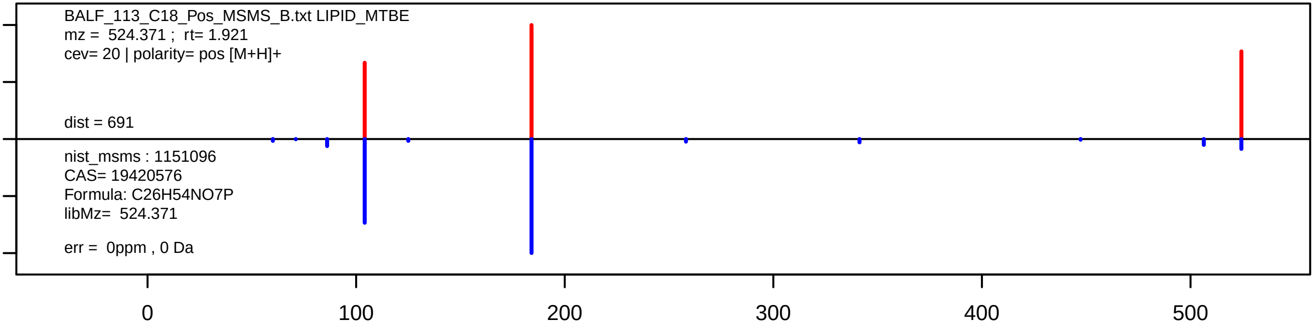

272 . 1−Stearoyl−2−hydroxy−sn−glycero−3−phosphoethanolamine
Score=282 Dot=855 prob=25.8

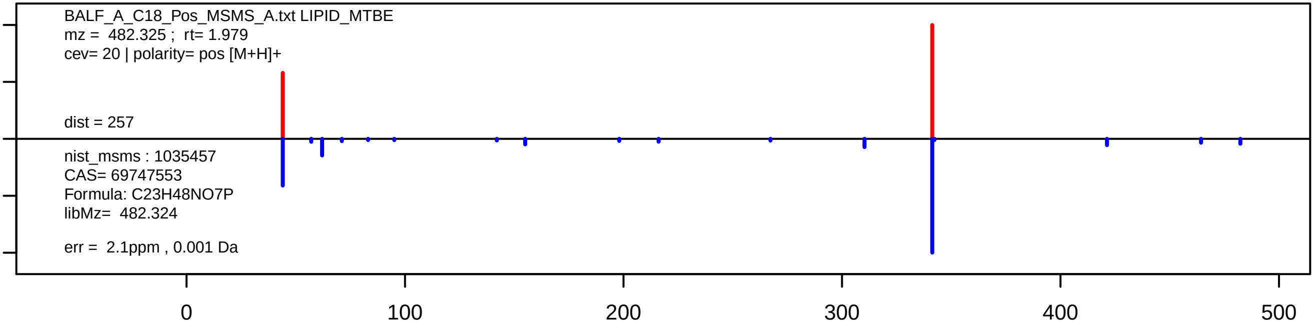

273 . 1−Stearoyl−2−linoleoyl−sn−glycero−3−phosphoethanolamine
Score=321 Dot=976 prob=89.8

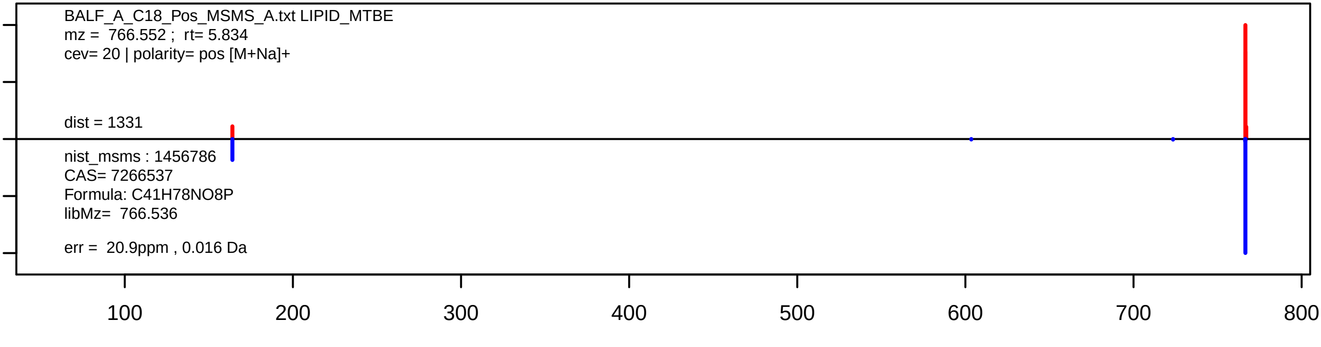

274 .   1−Stearoyl−2−myristoyl−sn−glycero−3−phosphocholine
Score=356 Dot=970 prob=73.7

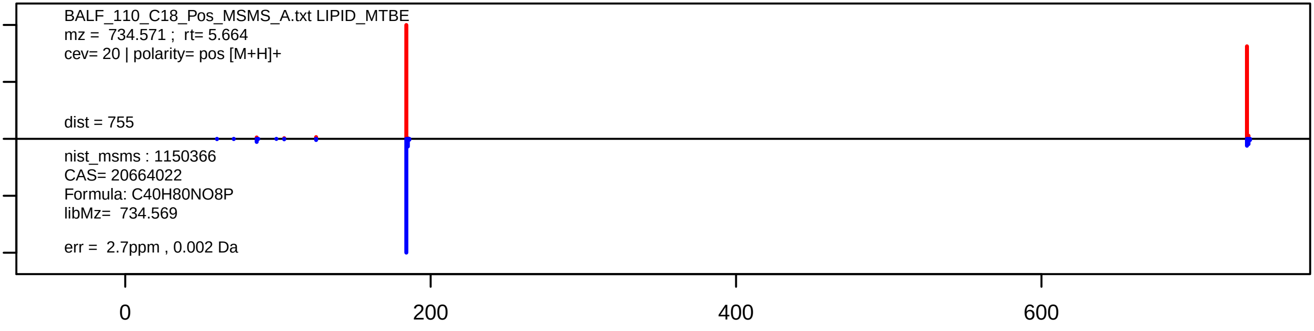

275 .   1−Stearoyl−2−oleoyl−sn−glycero−3−phosphoethanolamine
Score=400 Dot=999 prob=10.4

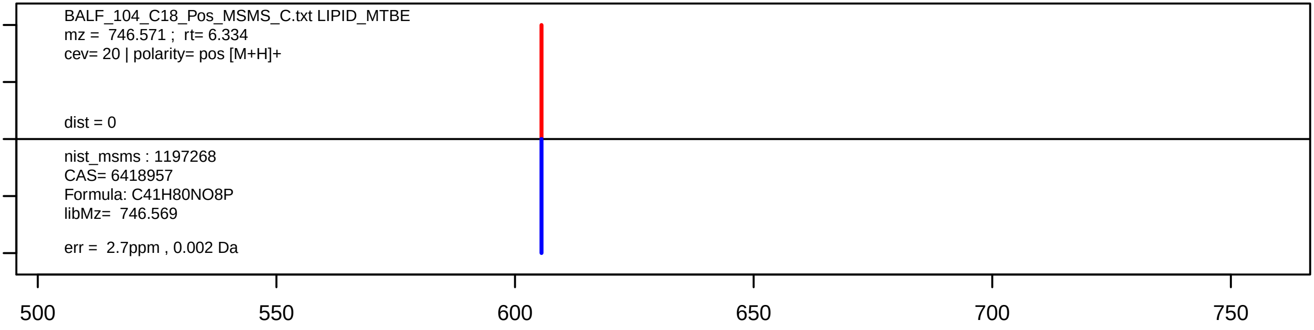

276 .   1,2−Benzenedicarboxylic acid
Score=382 Dot=890 prob=68.6

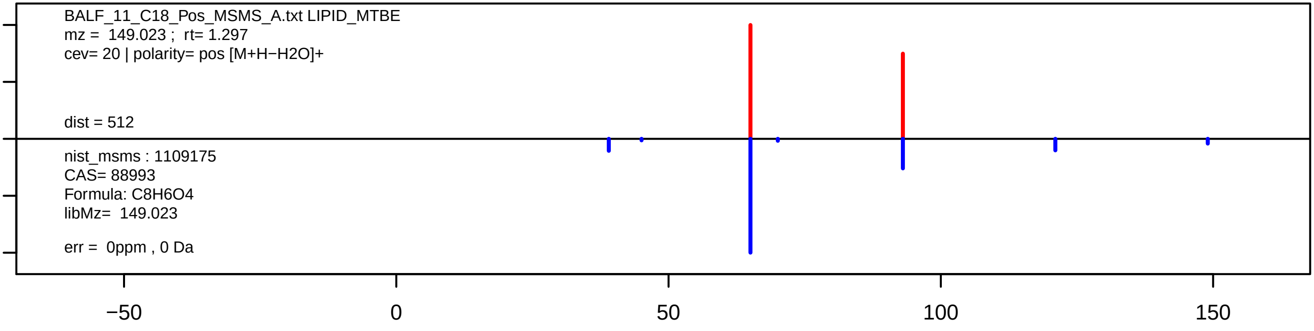

277 . 1,2−Diheptadecanoyl−sn−glycero−3−phosphocholine
Score=186 Dot=942 prob=91.8

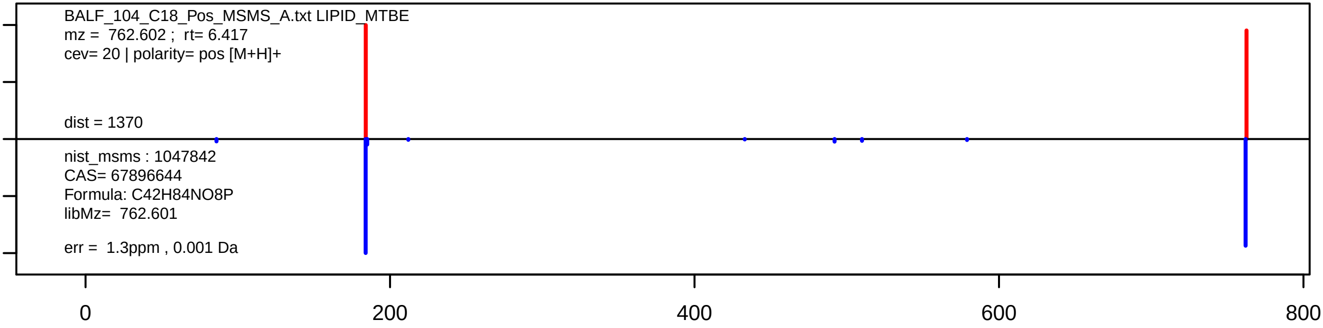

278 . 1,2−Dihexadecanoyl−sn−glycero−3−phosphocholine
Score=320 Dot=987 prob=84.7

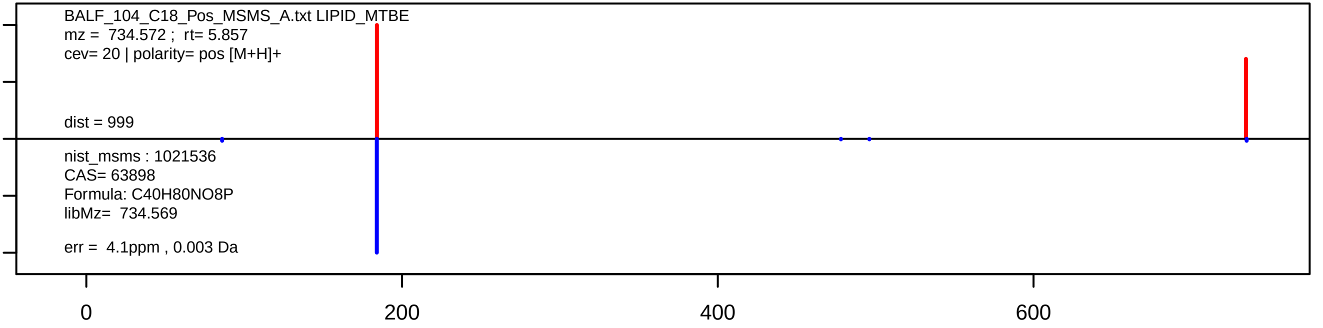

279 . 1,2−Dilinoleoyl−sn−glycero−3−phosphoethanolamine
Score=400 Dot=999 prob=5.4

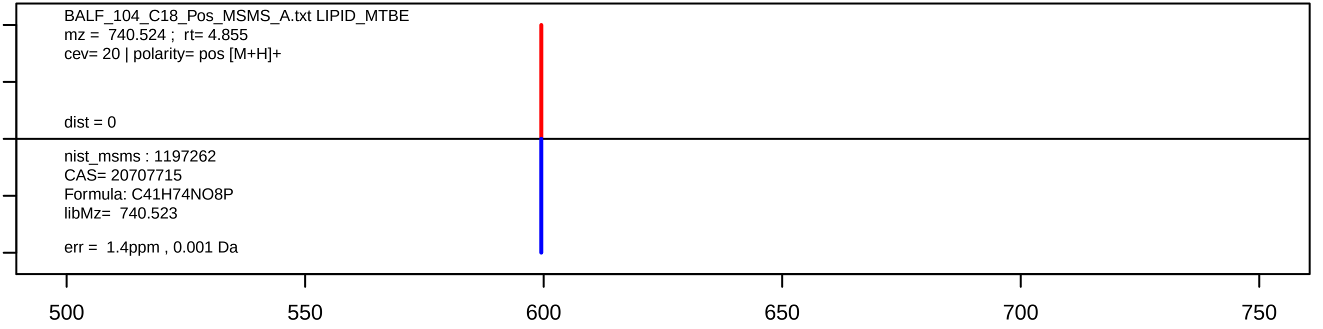

280 .   1,2−dioleoyl−sn−glycero−3−phosphatidylcholine
Score=400 Dot=999 prob=50

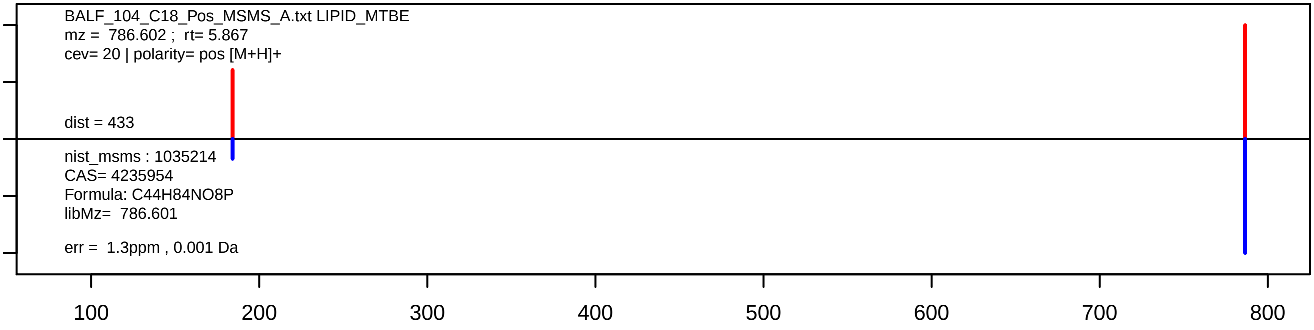

281 .   1,2−Dioleoyl−sn−glycero−3−phosphoethanolamine
Score=346 Dot=958 prob=4

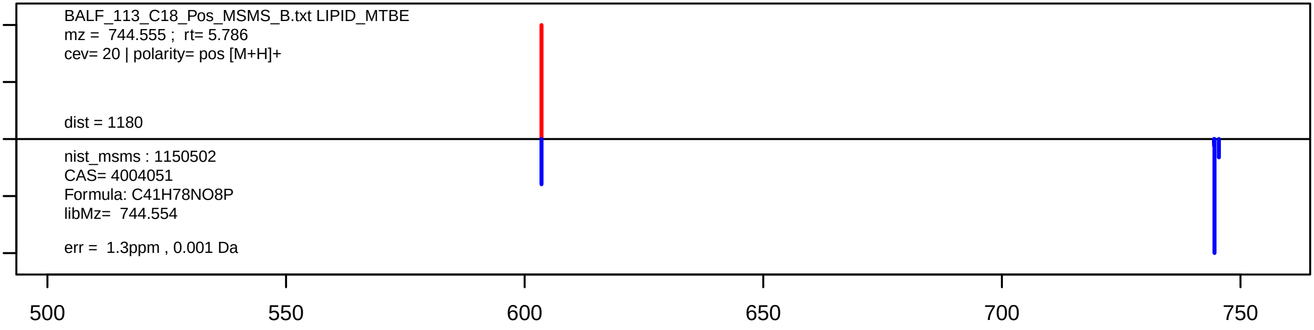

282 .   1,2−Dipalmitoleoyl−sn−glycero−3−phosphocholine
Score=601 Dot=861 prob=89.9

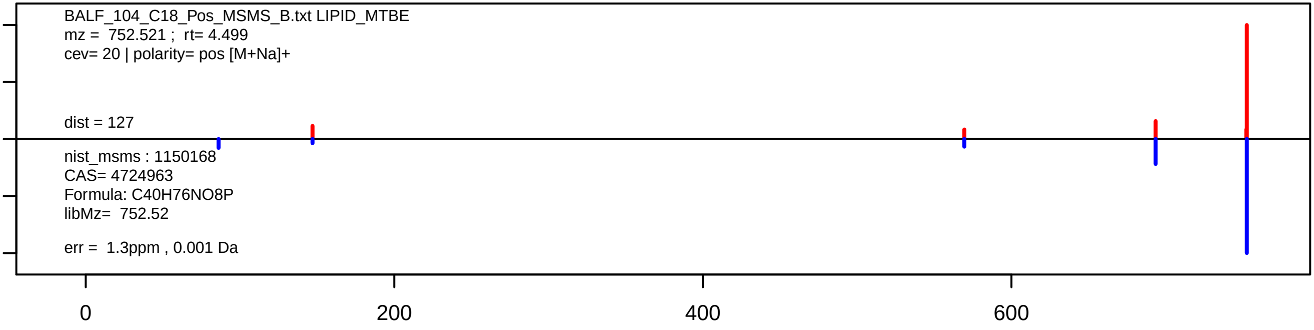

283 .   1,2−Dipalmitoyl−sn−glycero−3−phosphoethanolamine−N−methyl
Score=172 Dot=884 prob=40.4

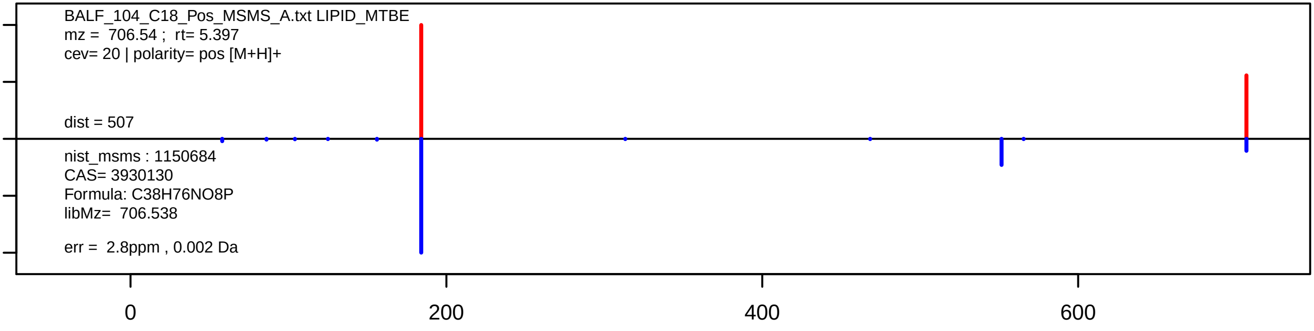

284 .   1,2−Dipalmitoyl−sn−glycero−O−ethyl−3−phosphatidylcholine cation
Score=235 Dot=940 prob=49

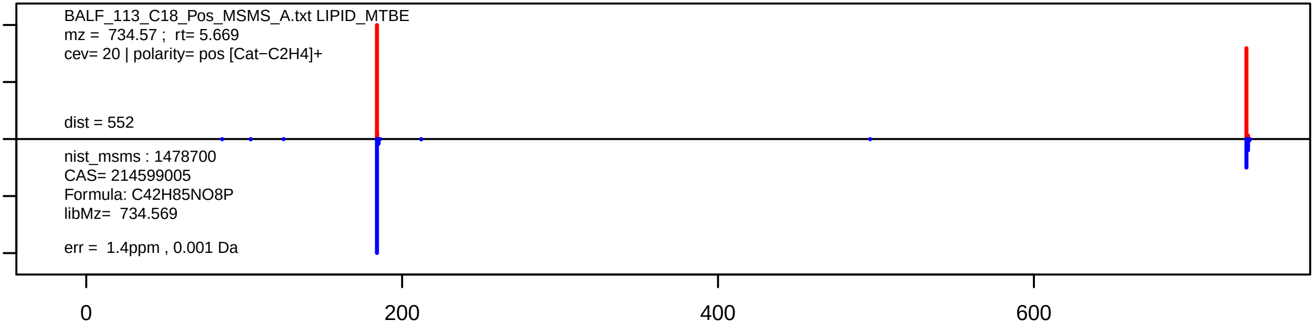

285 .   1,2−Dipentadecanoyl−sn−glycero−3−phosphocholine
Score=266 Dot=925 prob=32.2

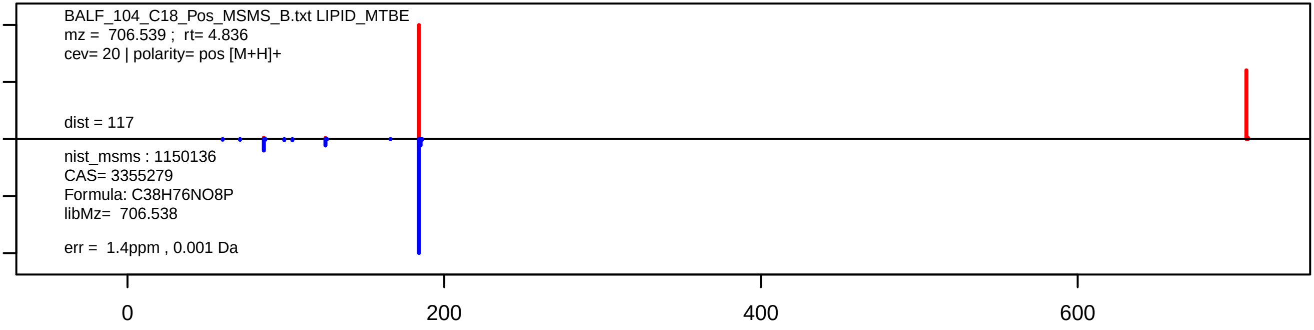

286 . 1,2−Distearoyl−sn−glycero−3−phosphoethanolamine
Score=391 Dot=999 prob=20.9

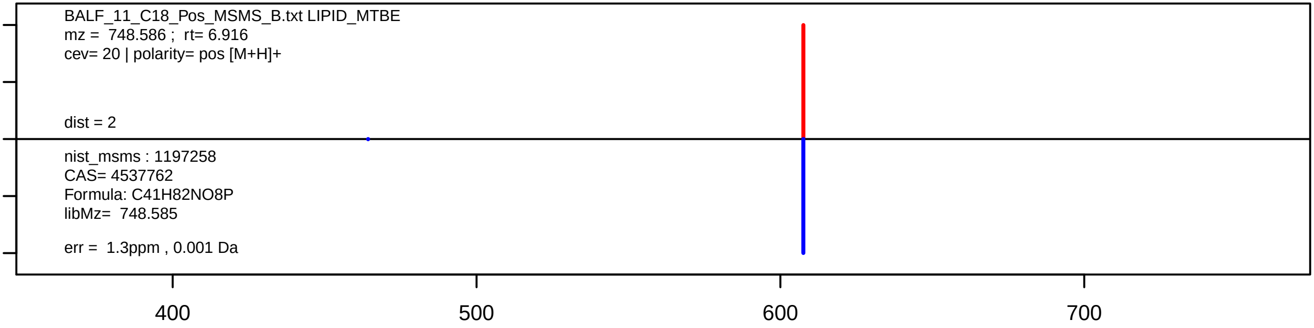

287 . 1,2−Ditetradecanoyl−sn−glycero−3−phosphocholine
Score=250 Dot=961 prob=55.4

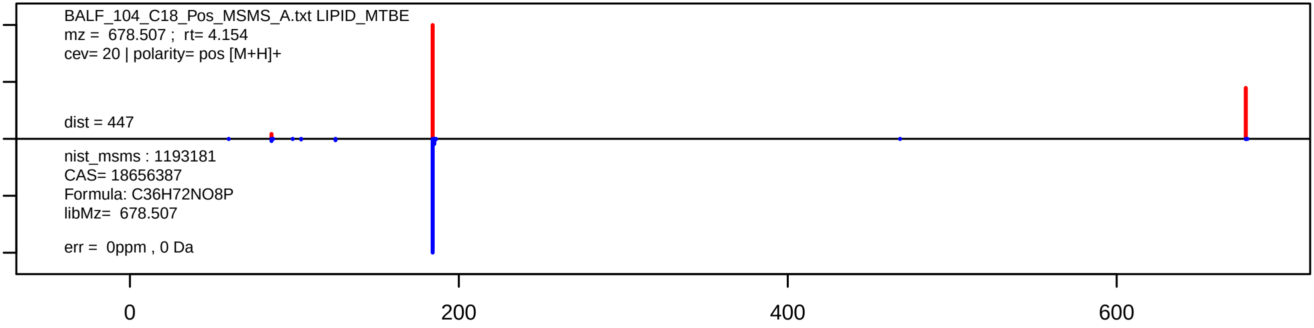

288 . 15(R),19(R)−Hydroxyprostaglandin E2
Score=500 Dot=951 prob=71.2

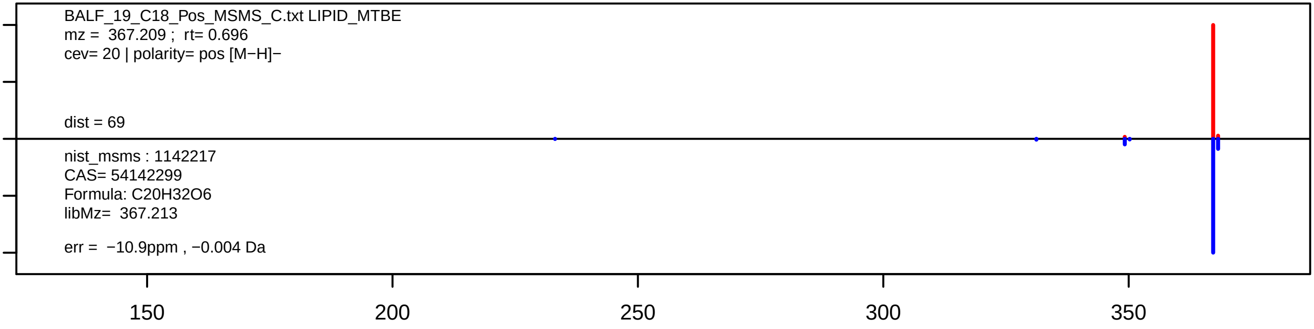

289 . 17−Phenyltrinorprostaglandin A2
Score=254 Dot=849 prob=46.9

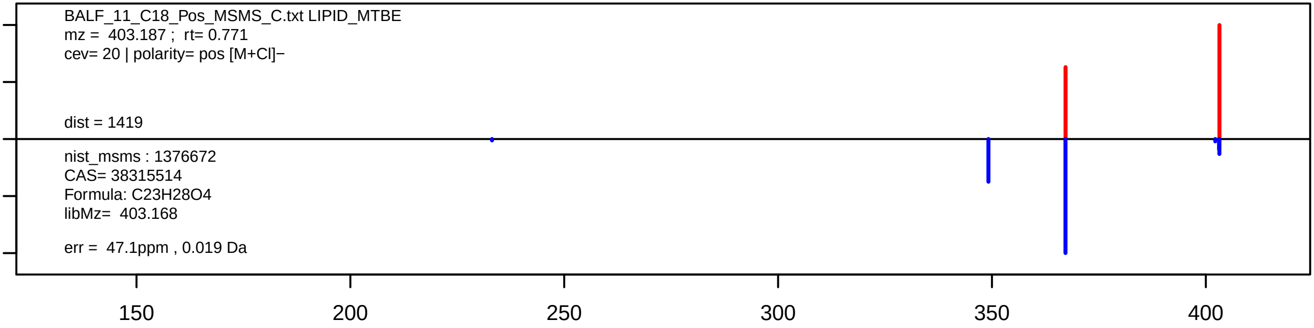

290 . 2−(2H−Benzotriazol−2−yl)−4,6−bis(1−methyl−1−phenylethyl)phenol
Score=355 Dot=996 prob=99

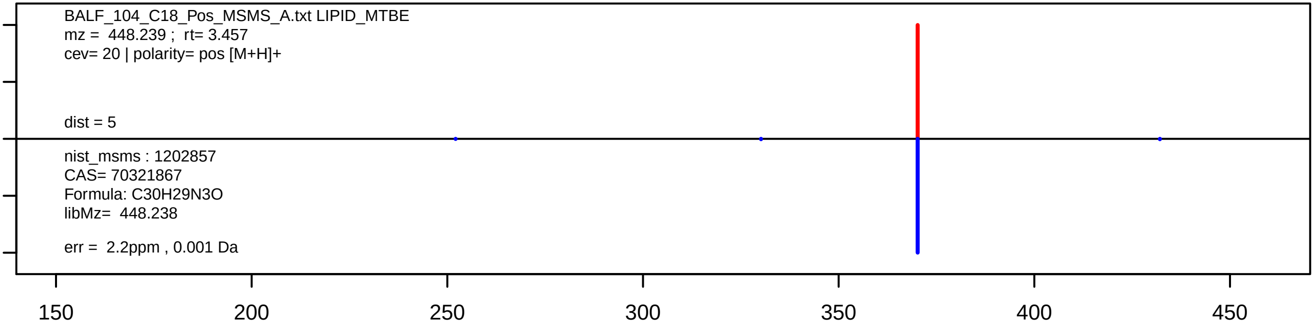

291 . 2−[(2,6−Dimethylphenyl)amino]−N,N,N−triethyl−2−oxoethanaminium cation
Score=557 Dot=997 prob=52.6

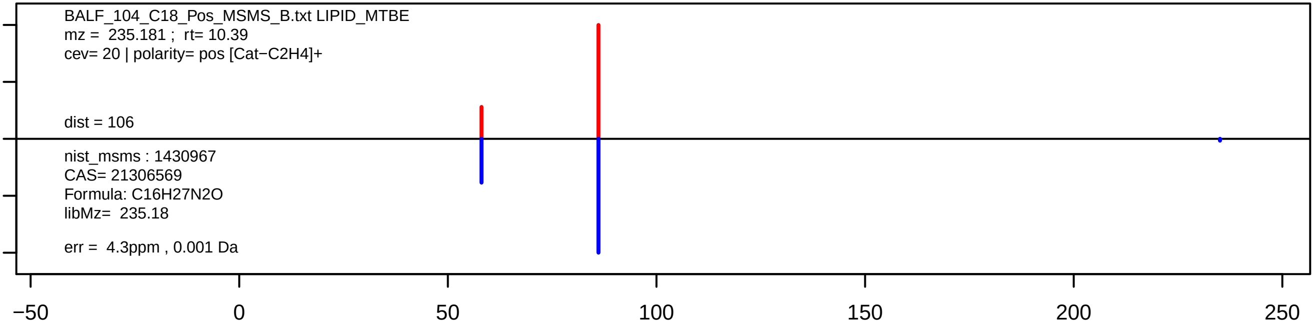

292 . 2−Docosahexaenoyl−1−palmitoyl−sn−glycero−3−phosphoethanolamine
Score=358 Dot=996 prob=1.6

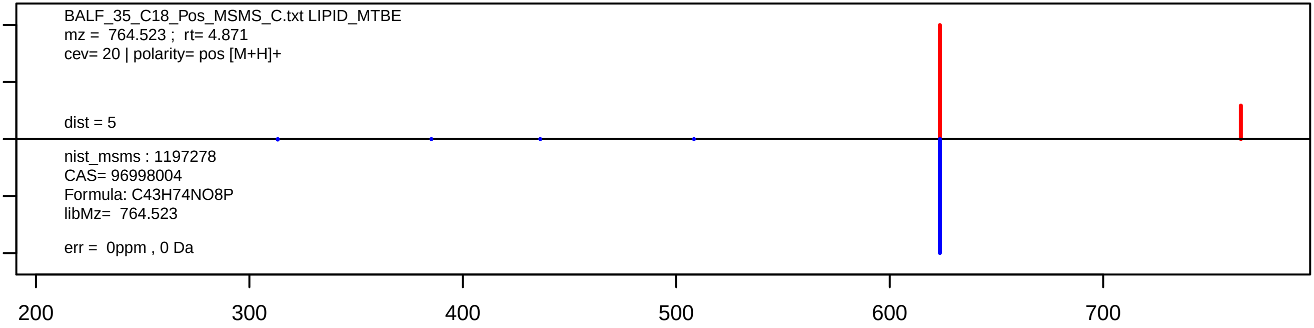

293 . 2−Ethylcyclohexanol
Score=814 Dot=910 prob=54.8

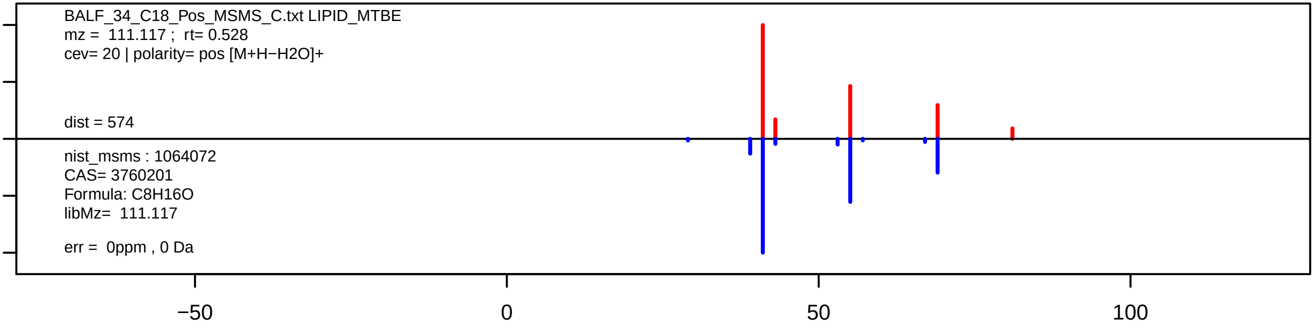

294 . 2−Hexadecanoylthio−1−ethylphosphorylcholine
Score=399 Dot=999 prob=97

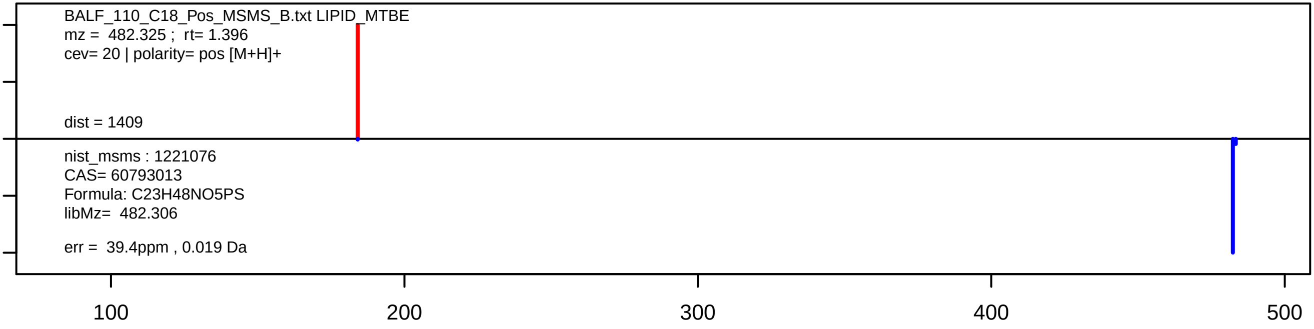

295 . 2−Linoleoyl−1−palmitoyl−sn−glycero−3−phosphoethanolamine
Score=369 Dot=992 prob=98

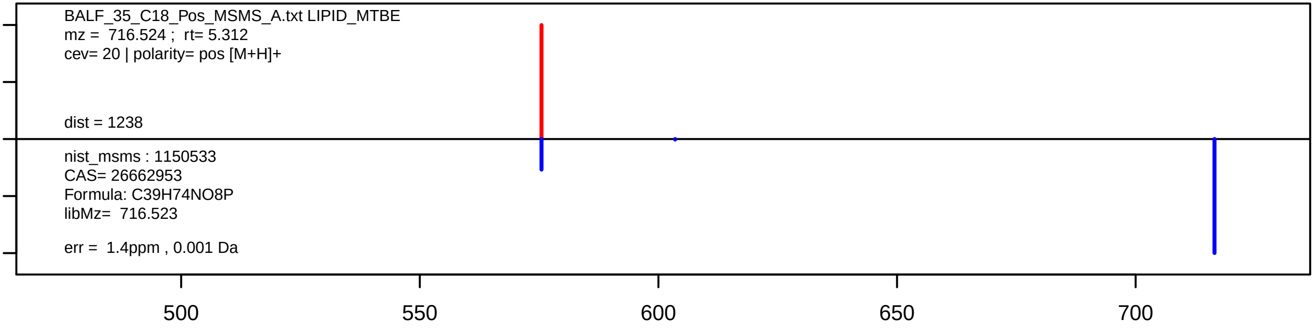

296 . 2−Oleoyl−1−palmitoyl−sn−glycero−3−phosphocholine
Score=400 Dot=999 prob=96.6

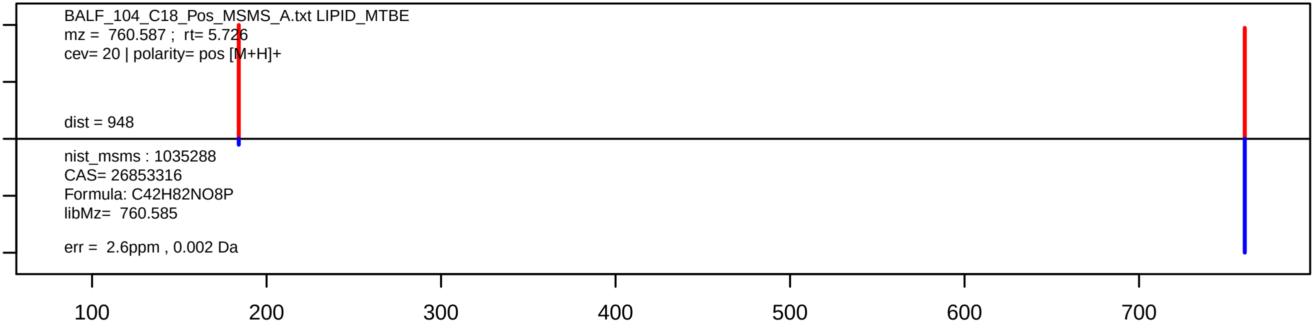

297 . 2−Oleoyl−1−stearoyl−sn−glycero−3−phosphoserine
Score=398 Dot=996 prob=70.9

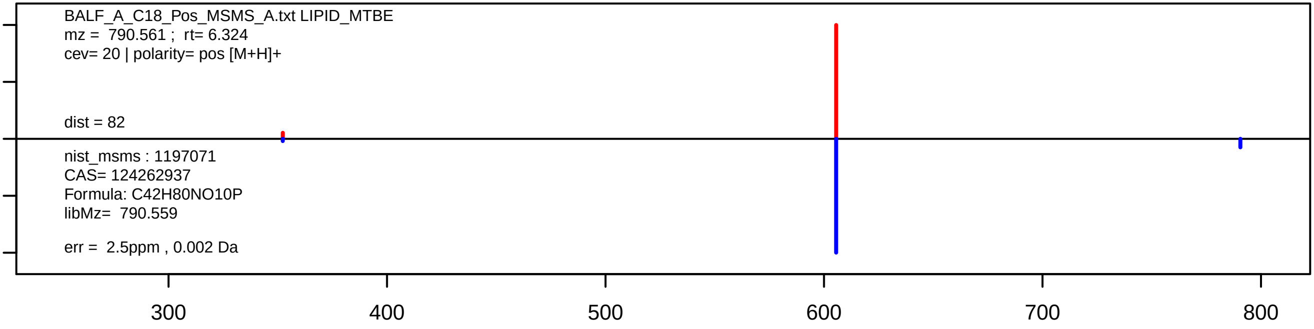

298 . 2−Pyrrolidinone, 1−methyl−
Score=341 Dot=930 prob=58.1

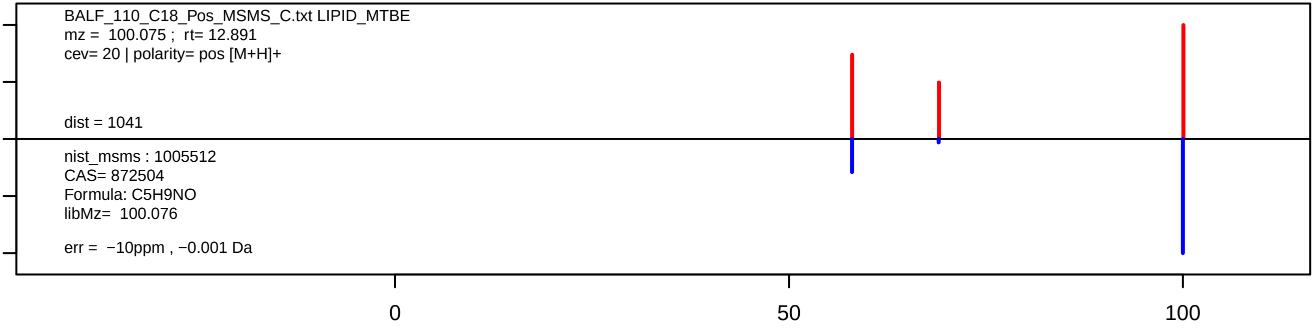

299 . 20−Hydroxy−4Z,7Z,10Z,13Z,16Z,18E−docosahexaenoic acid
Score=388 Dot=998 prob=78.9

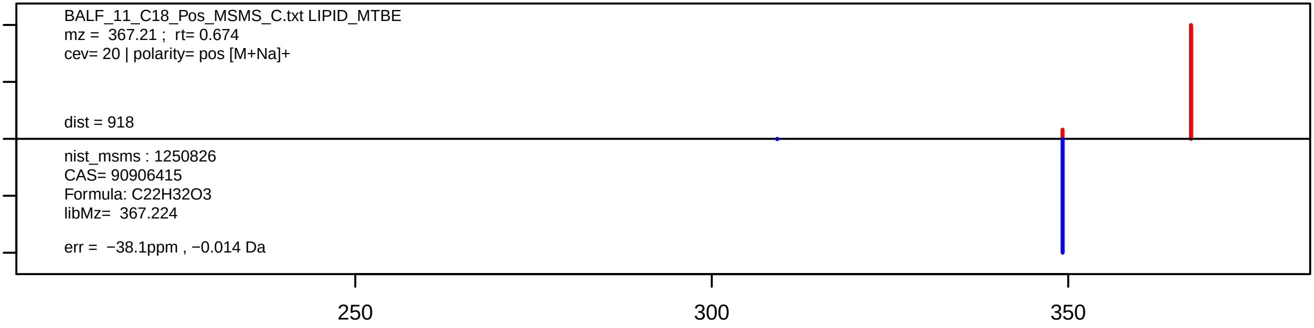

300 . 2S−Amino−4E−octadecene−1,3S−diol
Score=334 Dot=873 prob=82.8

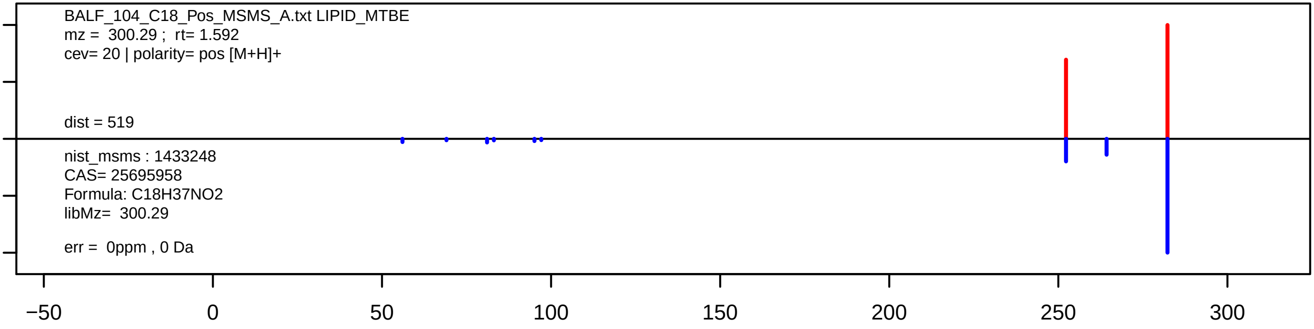

301 .   3−Aminopentanoic acid
Score=559 Dot=934 prob=91.8

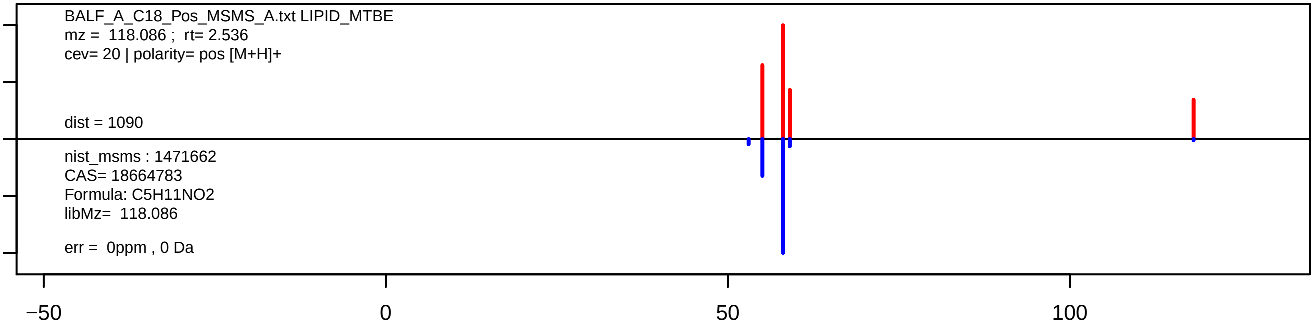

302 .   3,4−Dimethoxymethcathinone
Score=259 Dot=853 prob=66.3

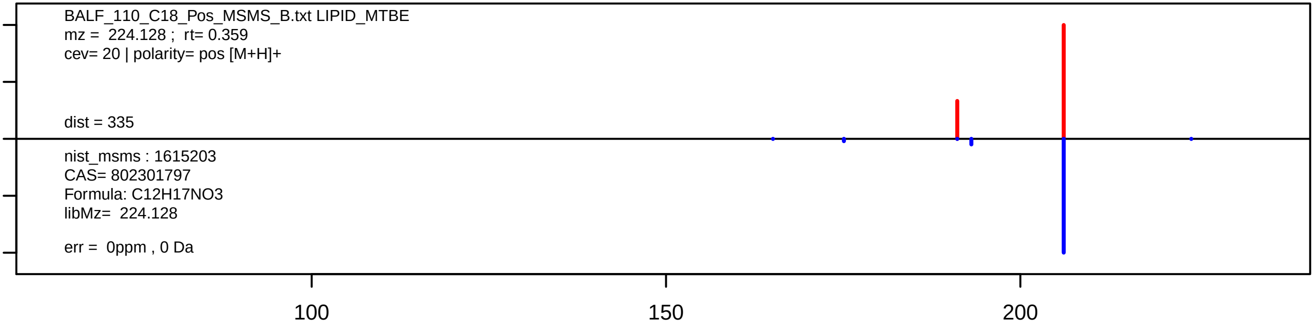

303 .   4−(Butylamino)benzoic acid
Score=368 Dot=840 prob=96.5

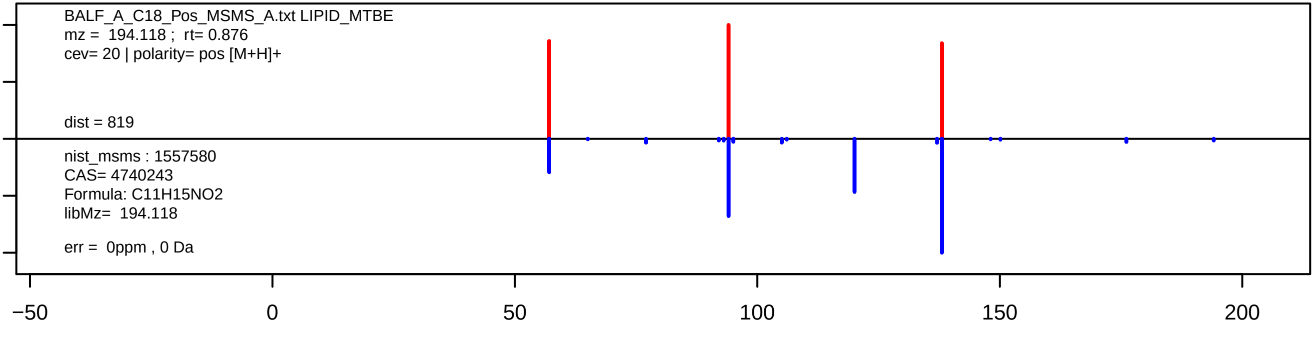

304 . 4−Imidazolidineheptanoic acid, 3−[(3R)−3−cyclohexyl−3−hydroxypropyl]−2,5−dioxo−, (4R)−rel−
Score=359 Dot=985 prob=62.6

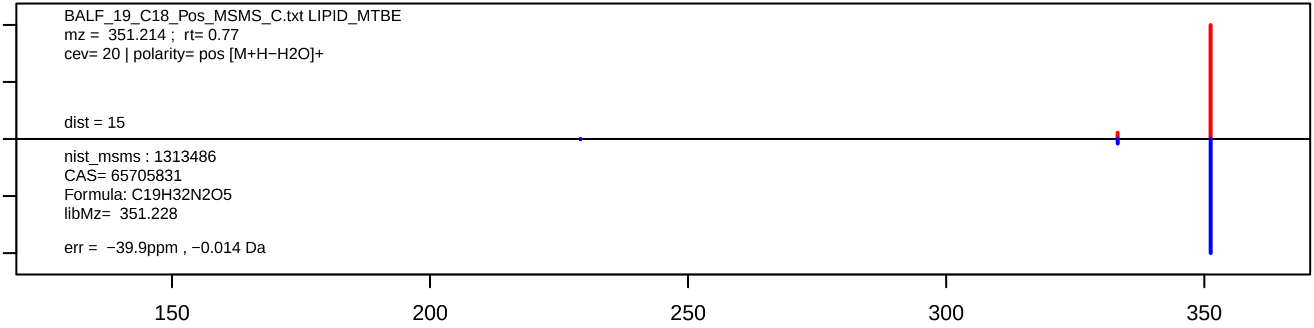

305 . 4−Methoxy−N,N−dimethyltryptamine
Score=280 Dot=847 prob=89.8

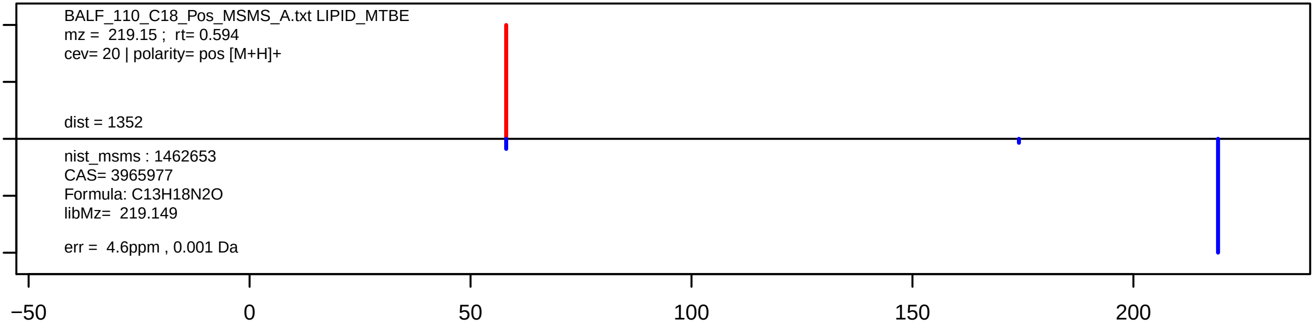

306 . 4−Penten−1−ol
Score=235 Dot=889 prob=63.8

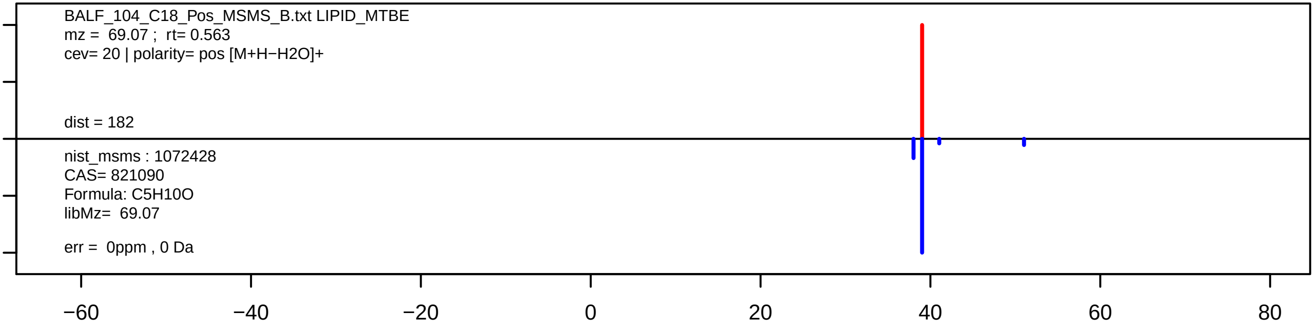

307 .   4−Penten−2−ol
Score=335 Dot=956 prob=37.1

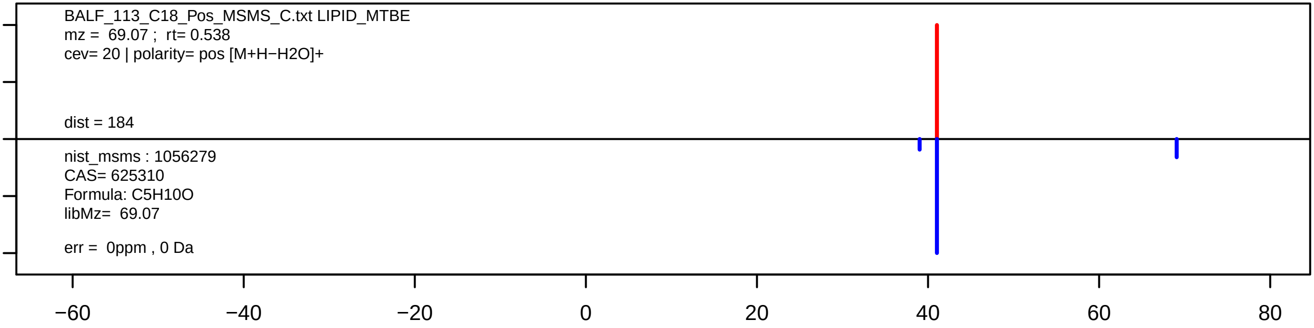

308 .   4−Pregnen−17.alpha., 20.alpha.−diol−3−one
Score=332 Dot=858 prob=67.3

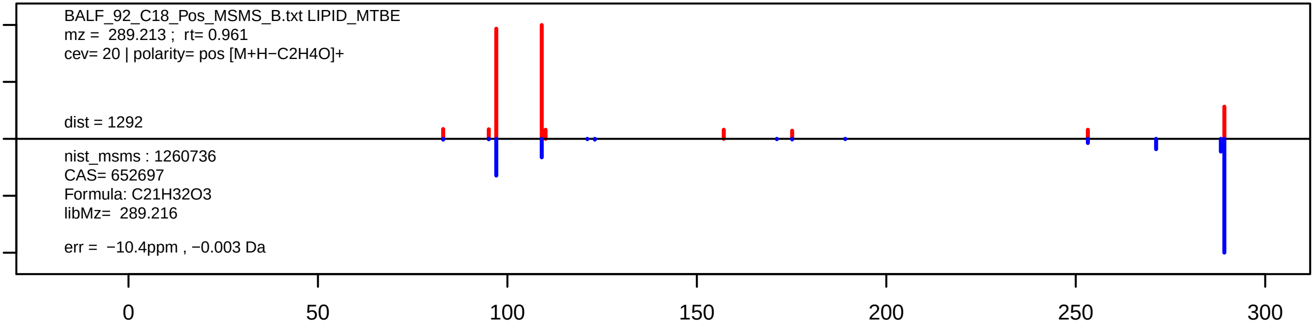

309 .   5,5−Dimethylimidazolidine−2,4−dione
Score=400 Dot=999 prob=3.5

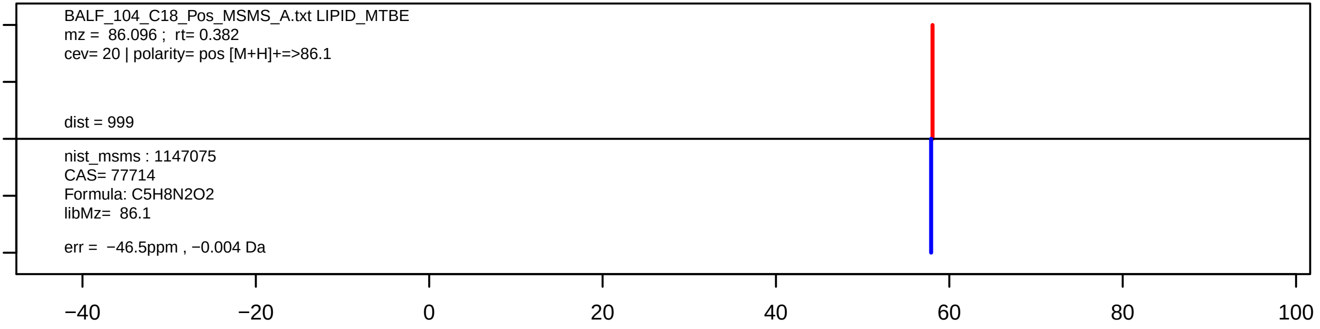

310 . 5.alpha.−Androstan−17.beta.−ol−3−one
Score=400 Dot=999 prob=32.9

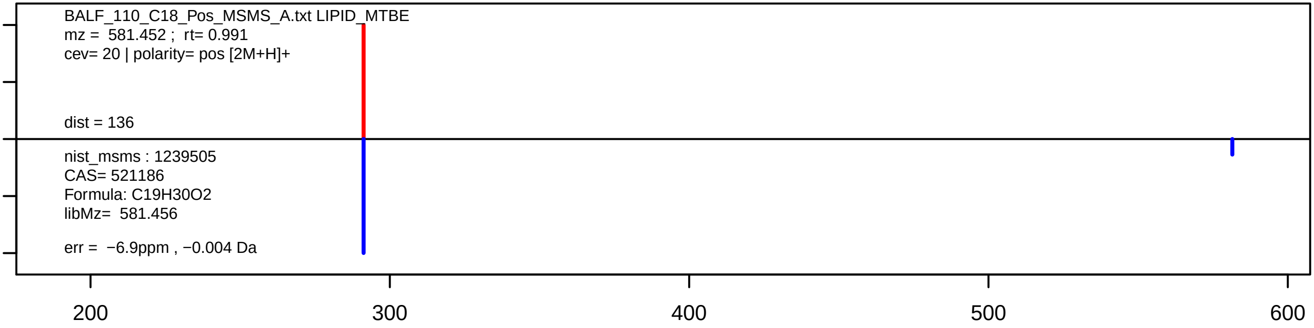

311 . 6'−Sialyl−N−acetyllactosamine
Score=626 Dot=851 prob=93

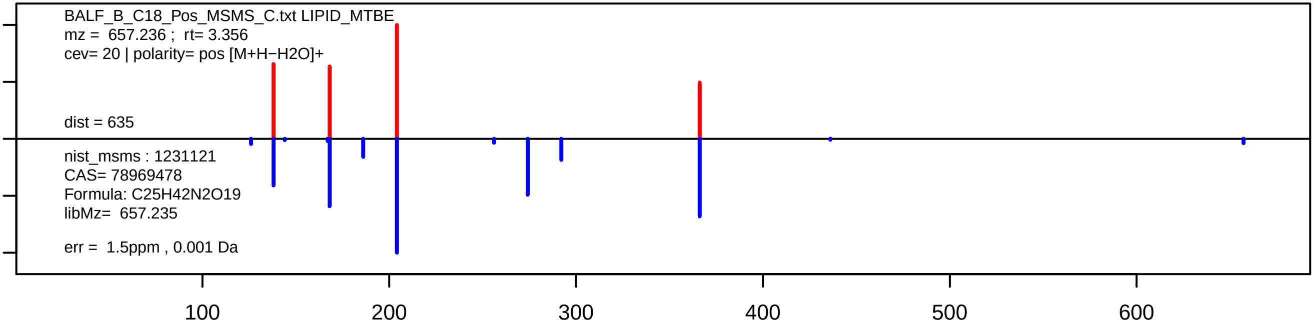

312 . 7.alpha.,12.alpha.−Dihydroxy−5.beta.−cholestan−3−one
Score=327 Dot=942 prob=65.6

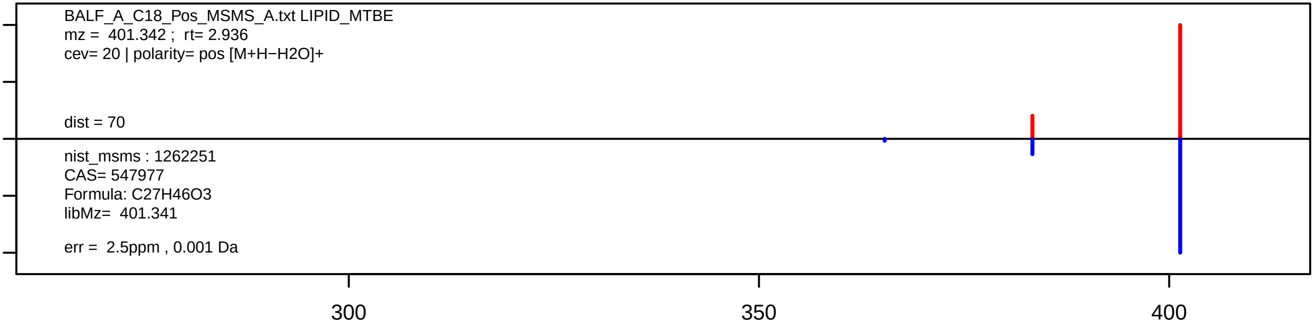

313 .   9−Hydroxy−10E,12Z−octadecadienoic acid, cholesteryl ester
Score=259 Dot=860 prob=93.7

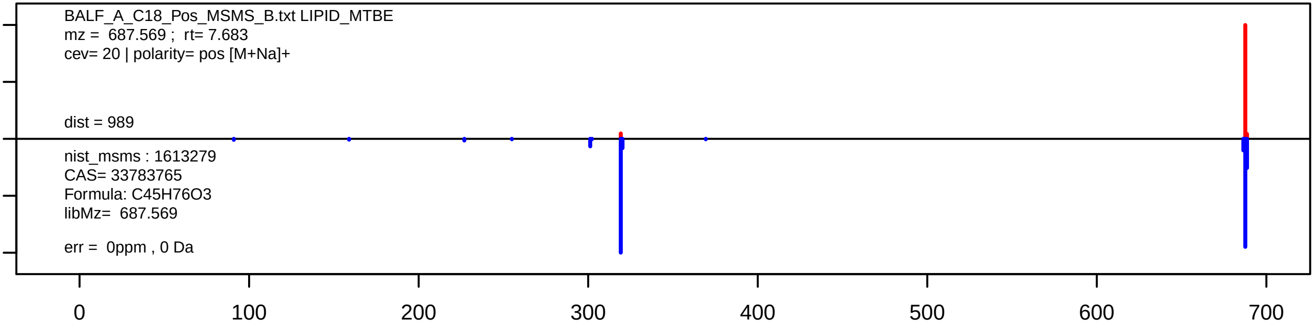

314 .   Acetyl−DL−carnitine
Score=244 Dot=907 prob=59.5

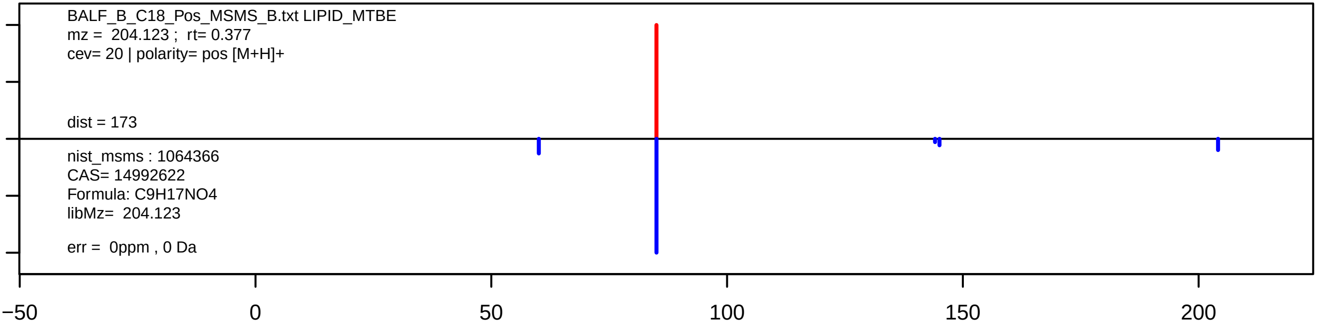

315 .   Aminodiphenylmethane
Score=940 Dot=979 prob=39.8

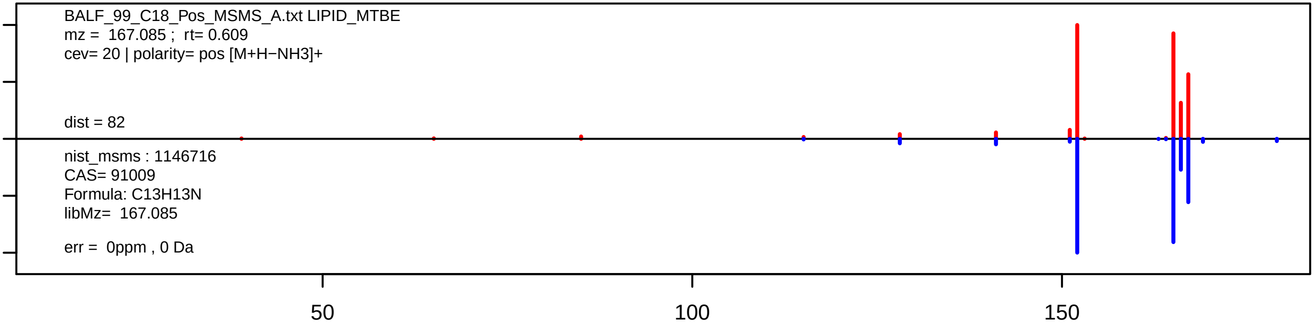

316 . Amiodarone
Score=190 Dot=842 prob=94

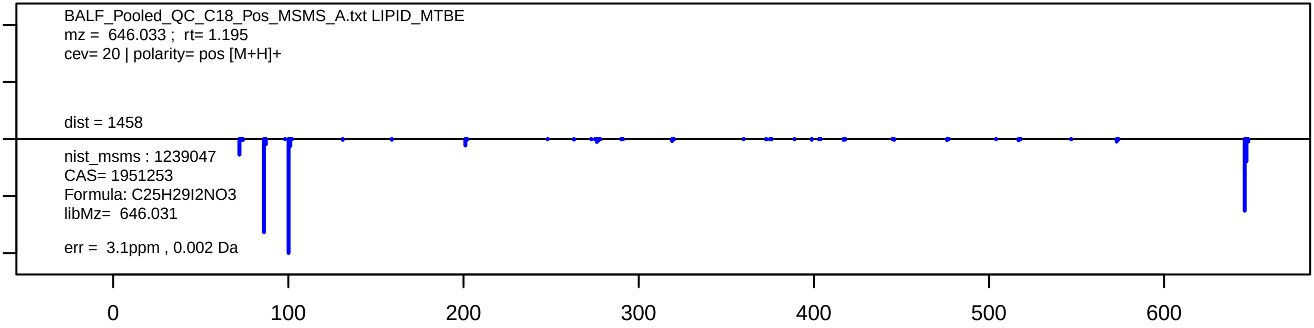

317 . Anthranilic acid
Score=400 Dot=999 prob=20.1

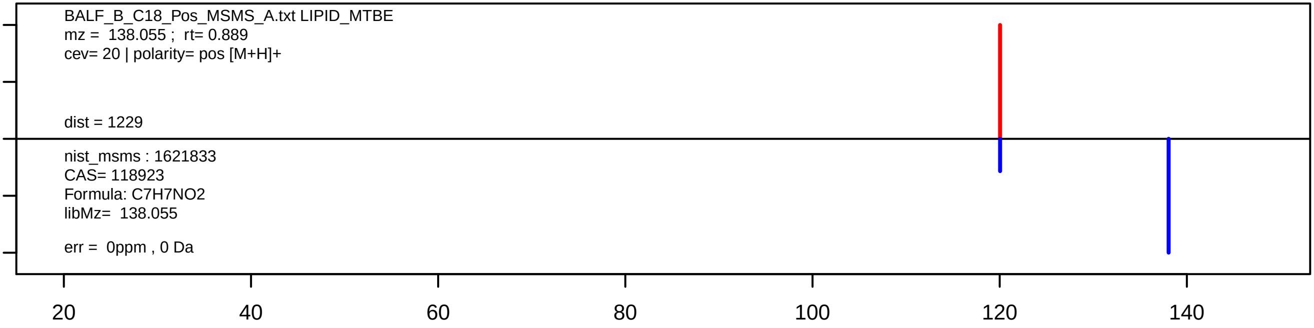

318 . Arachidonoylthiophosphorylcholine
Score=268 Dot=899 prob=83.3

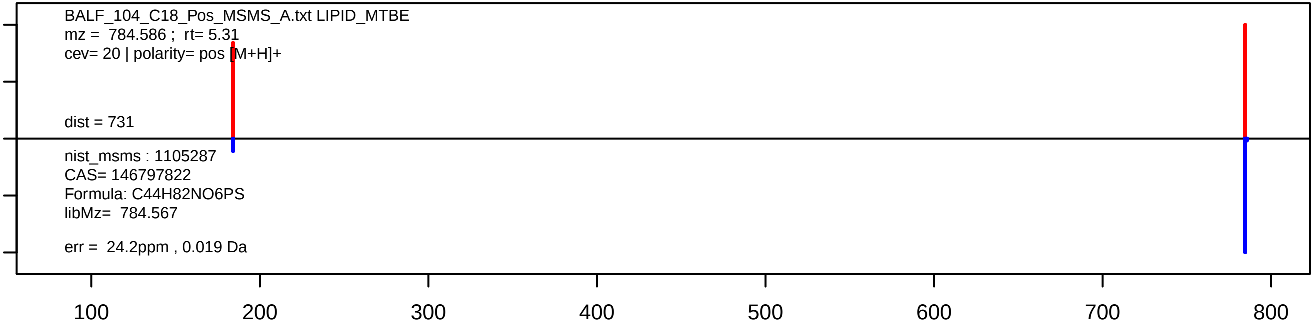

319 . Azithromycin
Score=383 Dot=837 prob=99

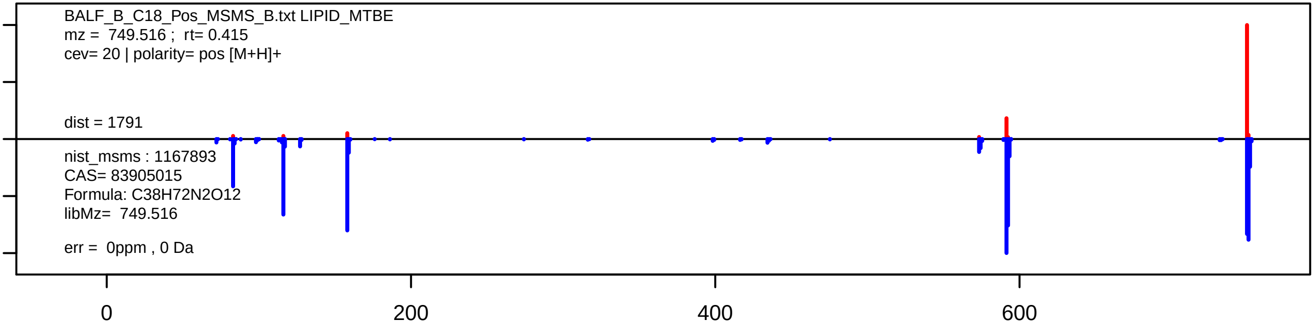

320 . Benzhydrol
Score=895 Dot=977 prob=35.3

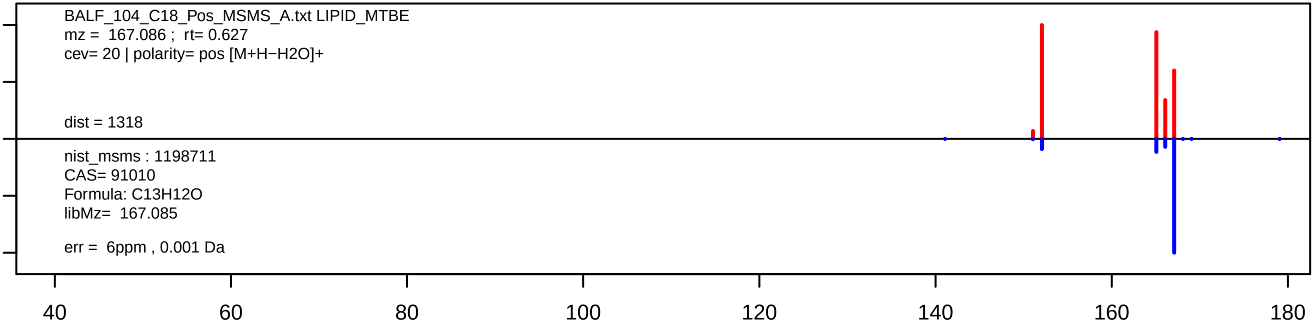

321 . Benzocaine
Score=763 Dot=948 prob=82

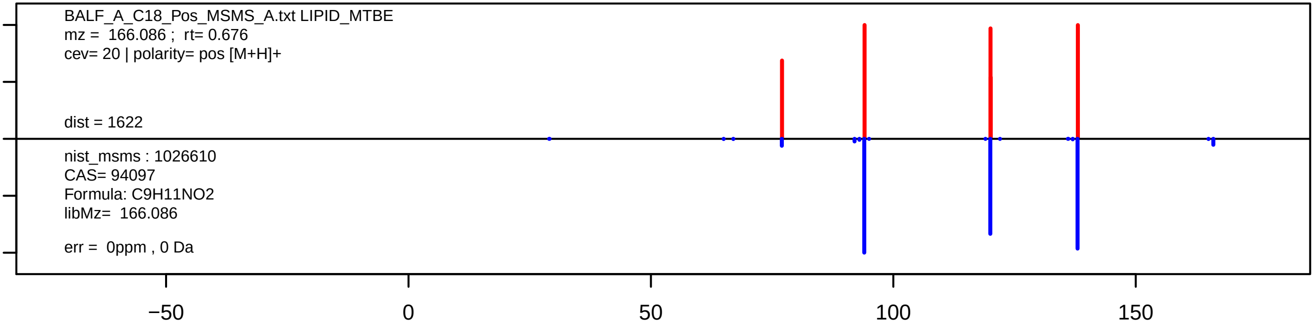

322 . Benzyl alcohol
Score=400 Dot=999 prob=1.5

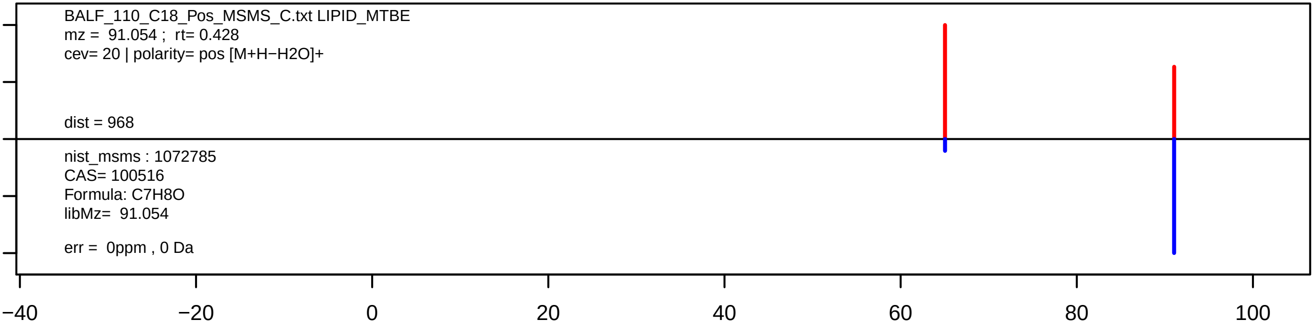

323 . Benzyl hexadecyl dimethylammonium cation
Score=754 Dot=975 prob=99

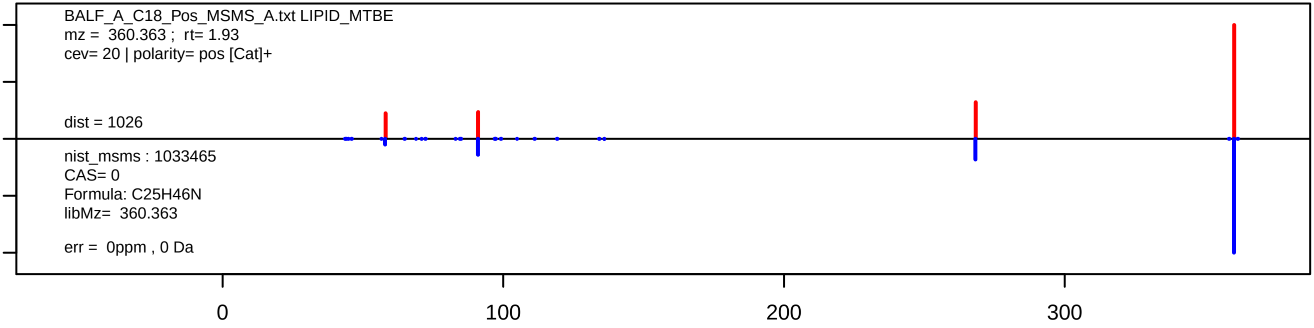

324 . Benzylamine
Score=407 Dot=903 prob=62.5

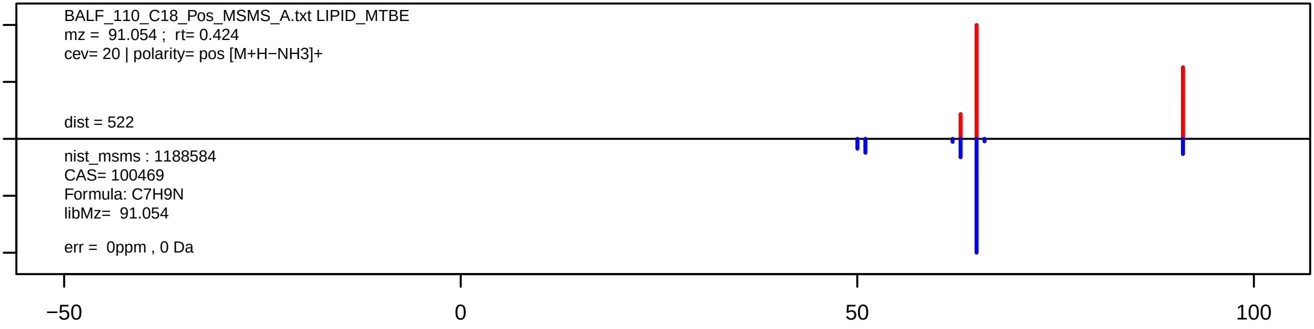

325 . Benzyldimethylstearylammonium cation
Score=398 Dot=873 prob=81

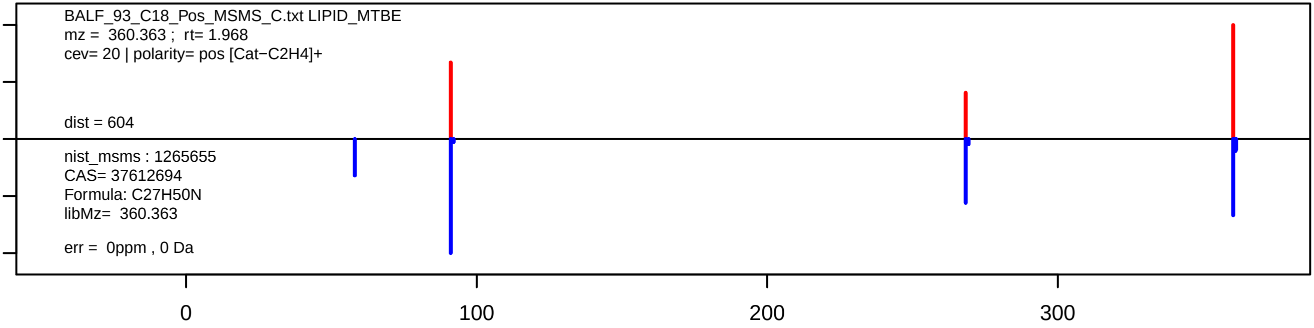

326 . Benzyldimethyltetradecylammonium cation
Score=843 Dot=996 prob=98.7

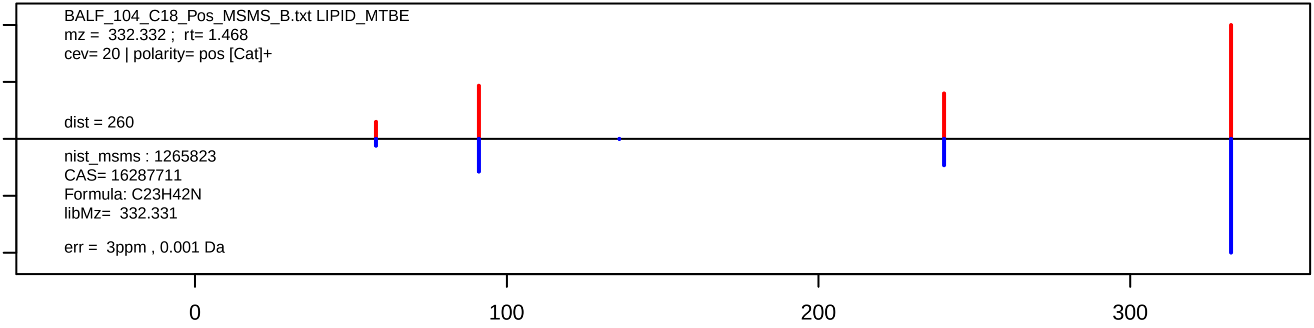

327 . Benzyldodecyldimethylammonium cation
Score=689 Dot=955 prob=98.8

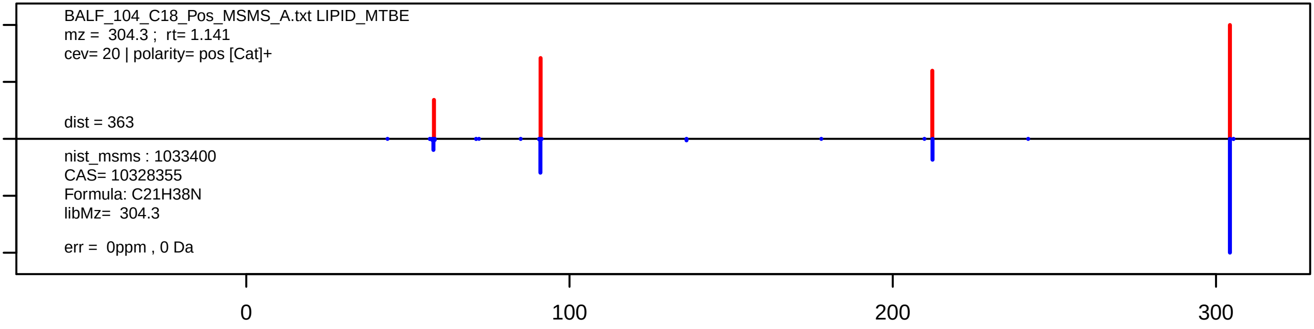

328 . Betaine
Score=552 Dot=999 prob=98.4

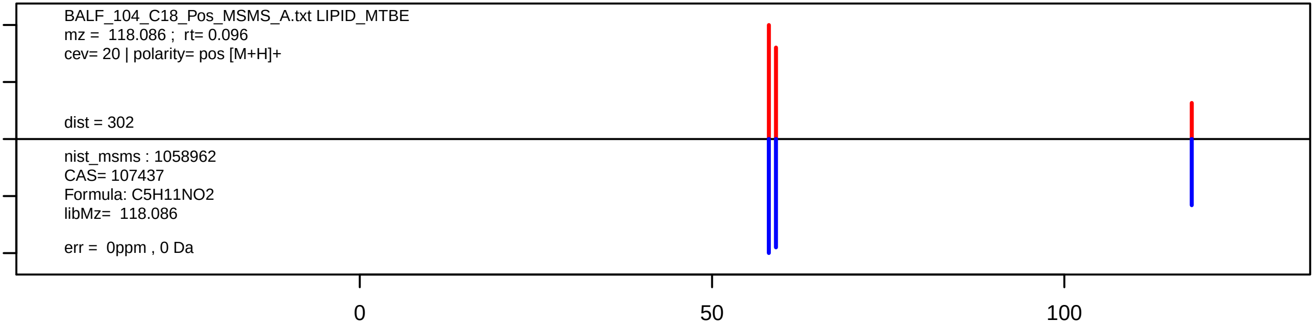

329 . Betaine aldehyde cation
Score=358 Dot=936 prob=98.7

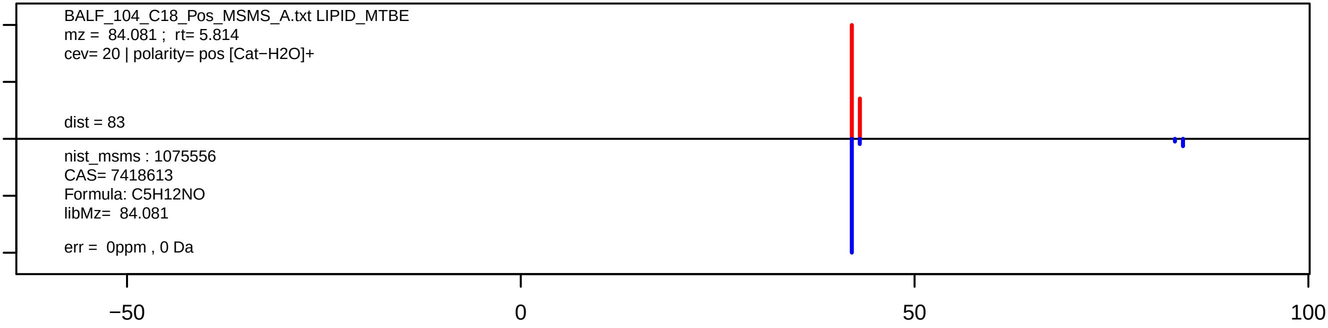

330 . Bis(2−ethylhexyl) adipate
Score=827 Dot=947 prob=97.1

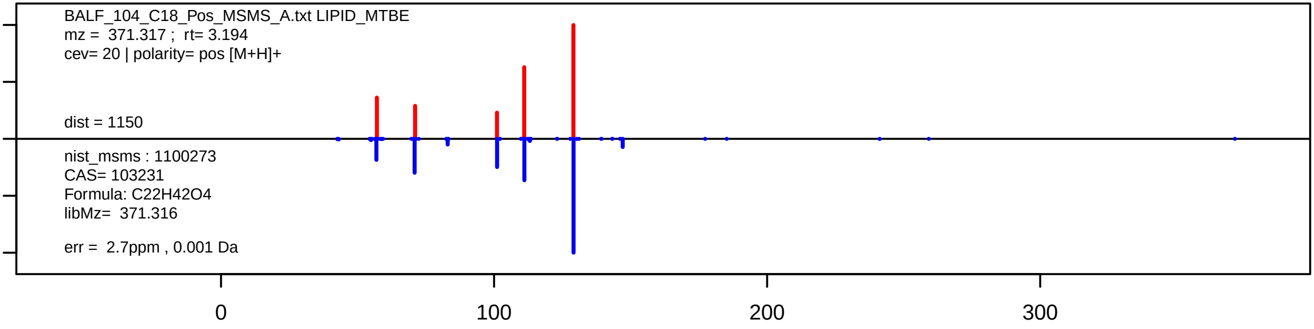

331 . Bis(2−ethylhexyl) phthalate
Score=637 Dot=908 prob=56.5

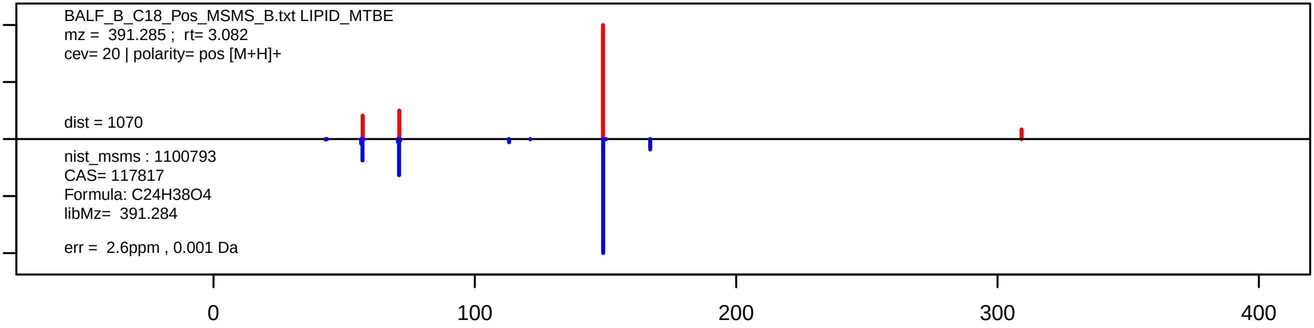

332 . Bufexamac
Score=400 Dot=999 prob=19.6

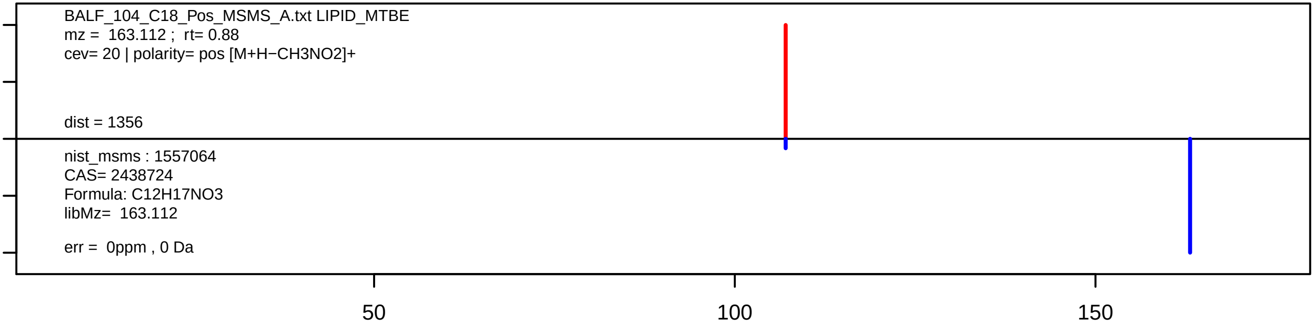

333 . Bupropion
Score=922 Dot=961 prob=94.7

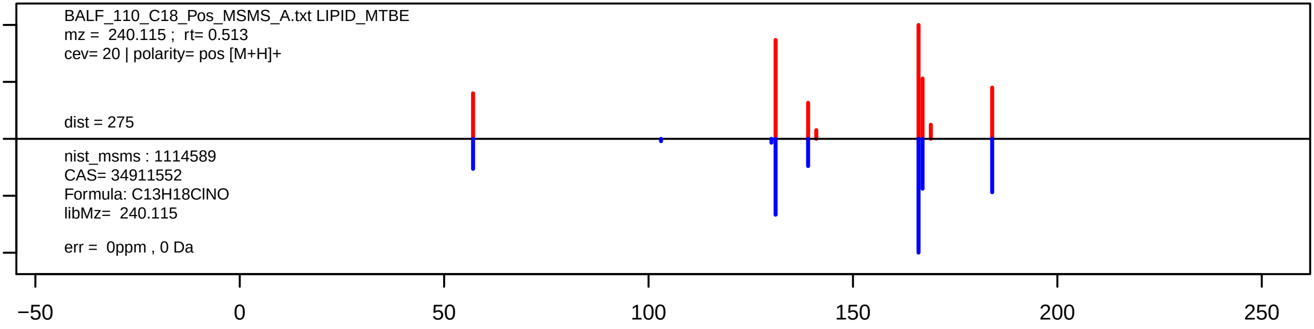

334 . Carbamic acid, N−(2,2−diphenylacetyl)−, ethyl ester
Score=955 Dot=972 prob=65.9

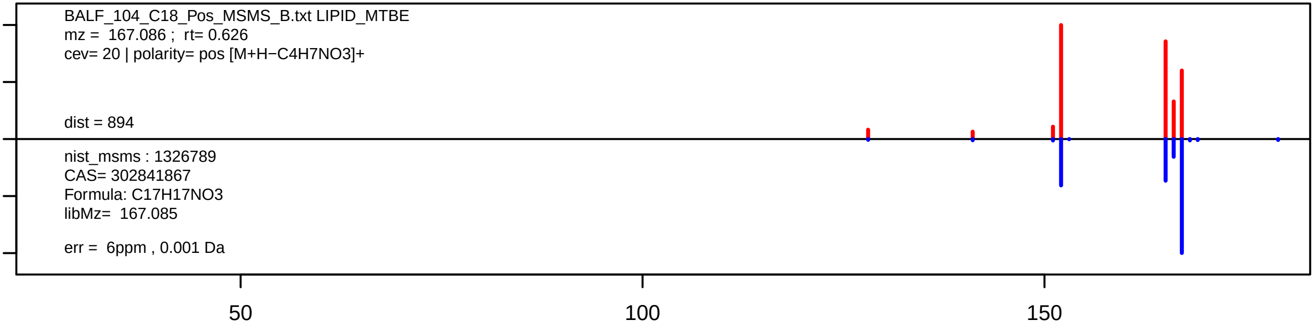

335 . Choline cation
Score=553 Dot=993 prob=96.9

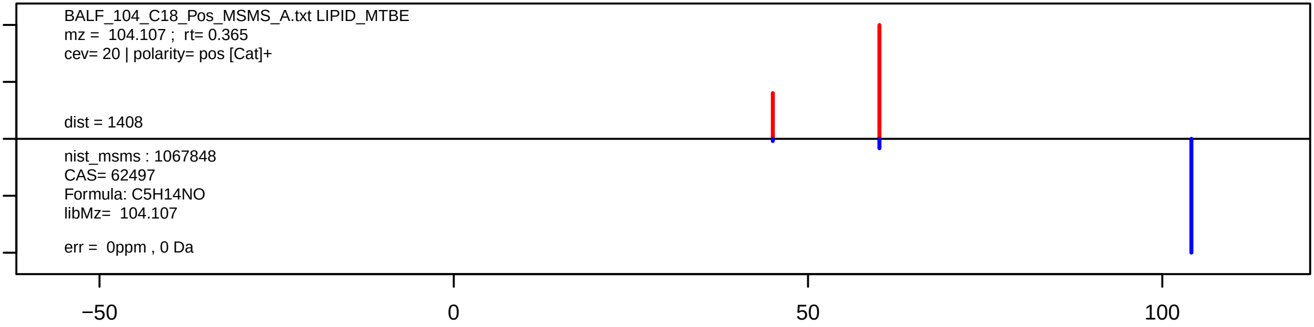

336 . Citalopram
Score=241 Dot=853 prob=98.5

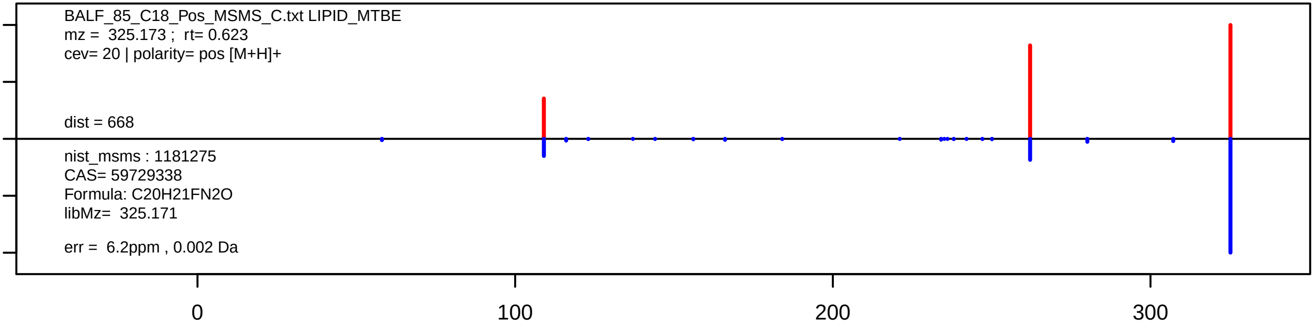

337 . Cyclizine
Score=800 Dot=984 prob=61

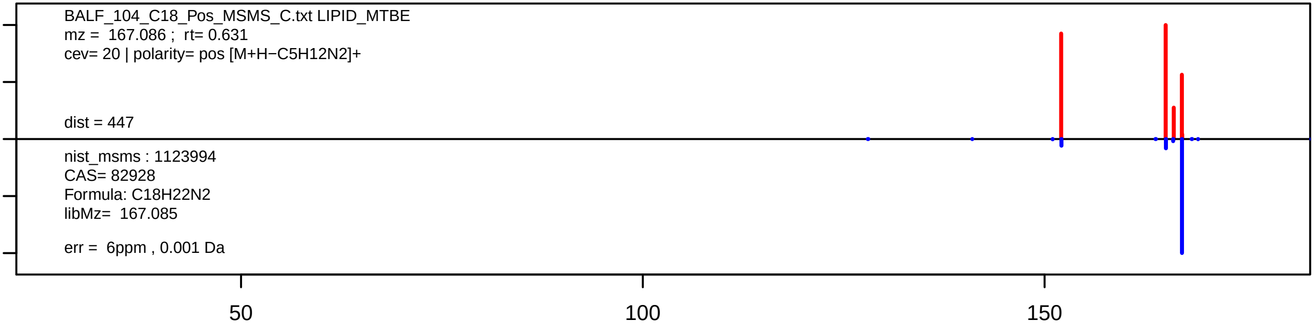

338 . Cycloxydime
Score=193 Dot=882 prob=84.7

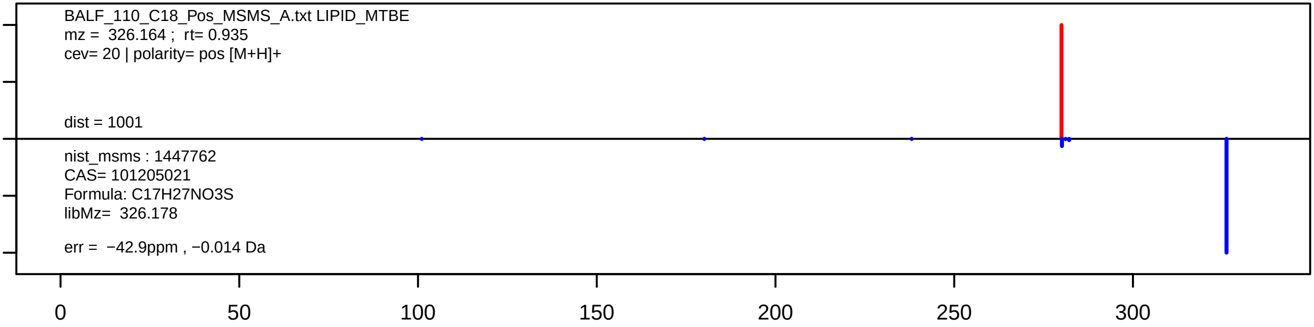

339 . D−(+)−Amphetamine
Score=399 Dot=998 prob=18.9

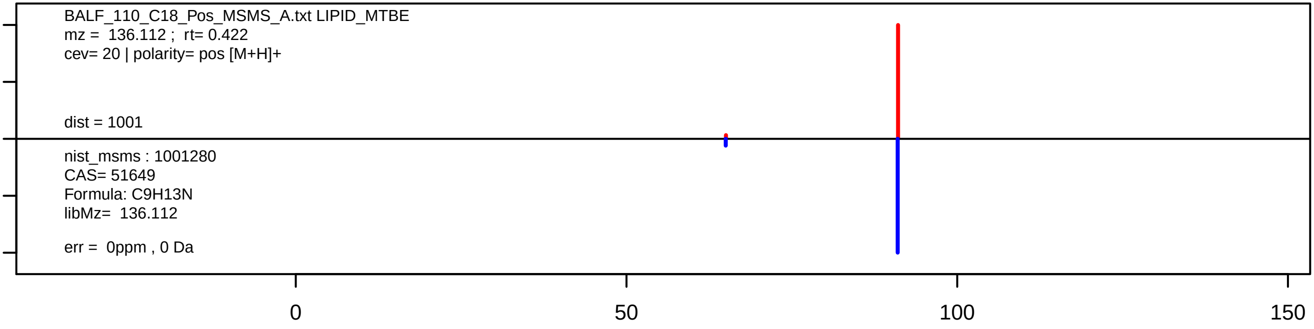

340 . D−erythro−N−stearoylsphingosine
Score=926 Dot=930 prob=51.8

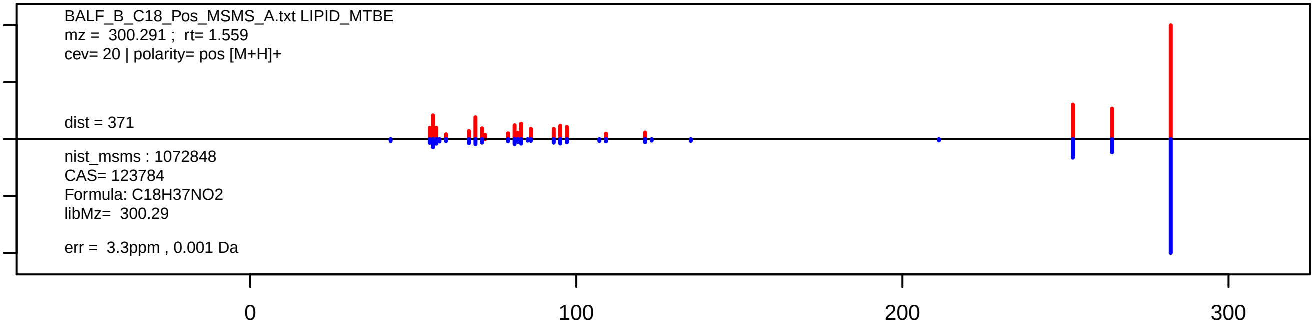

341 . D−erythro−Sphinganine
Score=622 Dot=855 prob=95.8

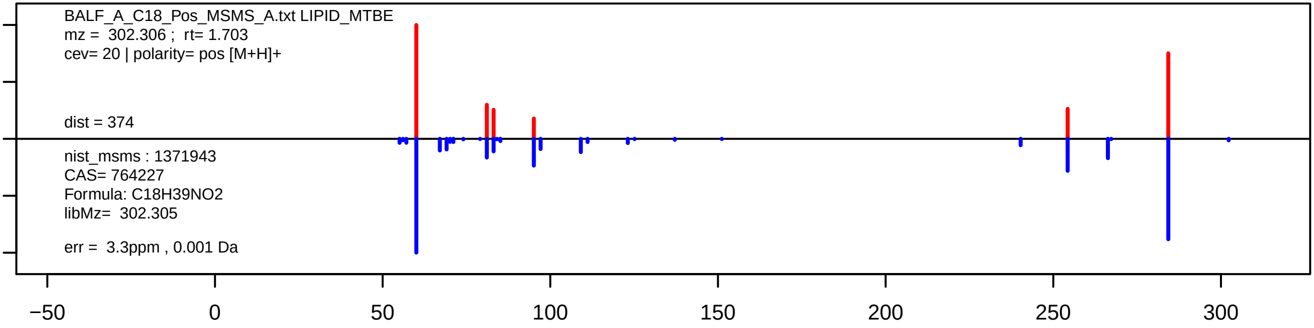

342 . Decamethylcyclopentasiloxane
Score=338 Dot=902 prob=99

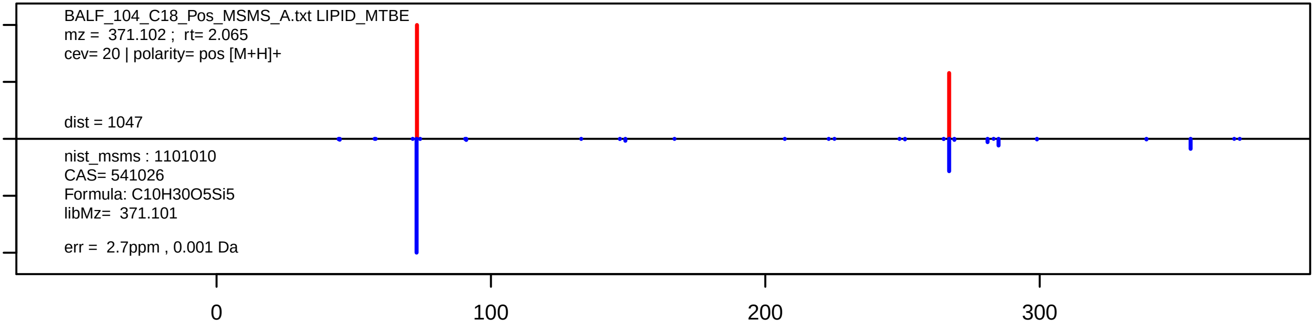

343 . Decanoyl−L−carnitine
Score=125 Dot=933 prob=75.9

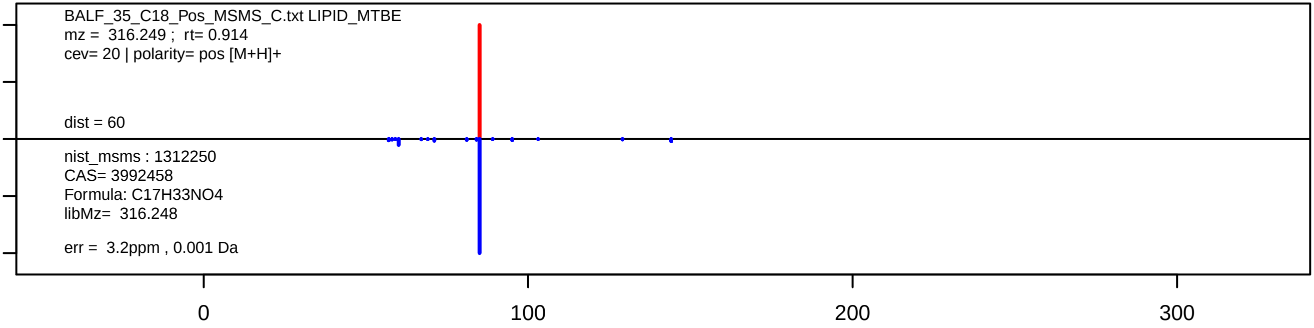

344 . Desalkylebastine
Score=559 Dot=999 prob=36.3

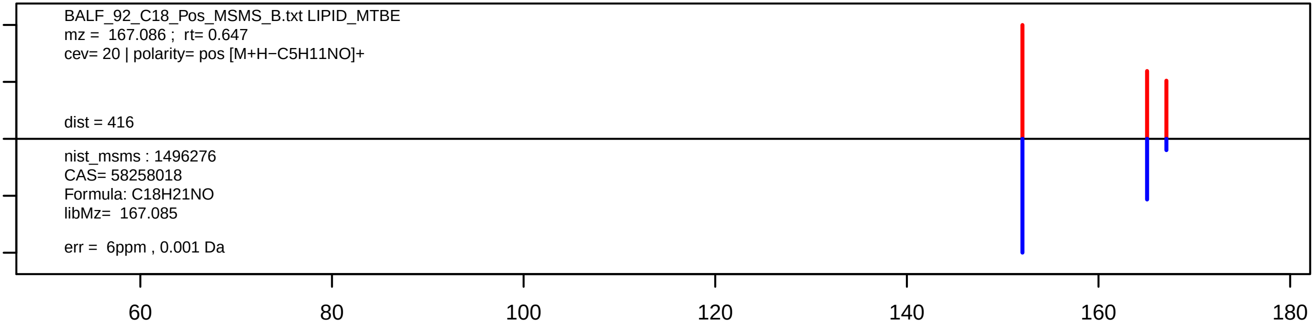

345 . Desethylamiodarone
Score=256 Dot=903 prob=100

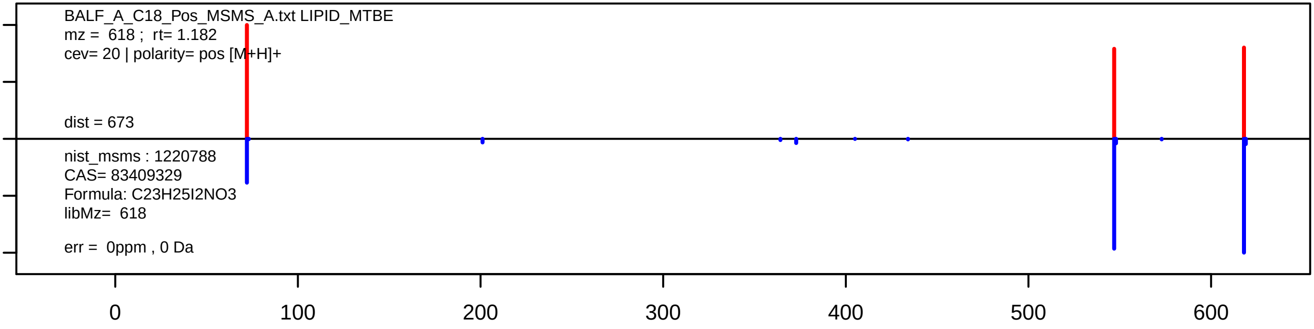

346 .   DG 36:2; [M+NH4]+; DG(18:0/18:2/0:0)
Score=642 Dot=895 prob=49.7

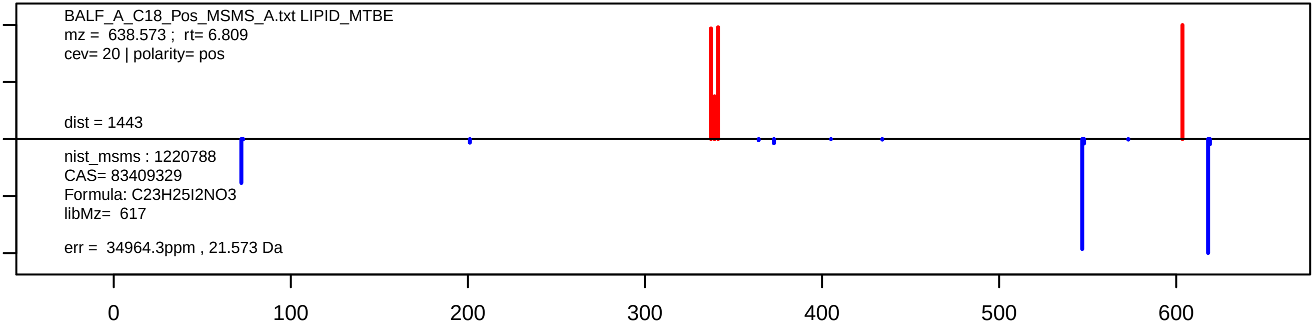

347 .   Di(2−nonyl) phthalate
Score=338 Dot=961 prob=73.3

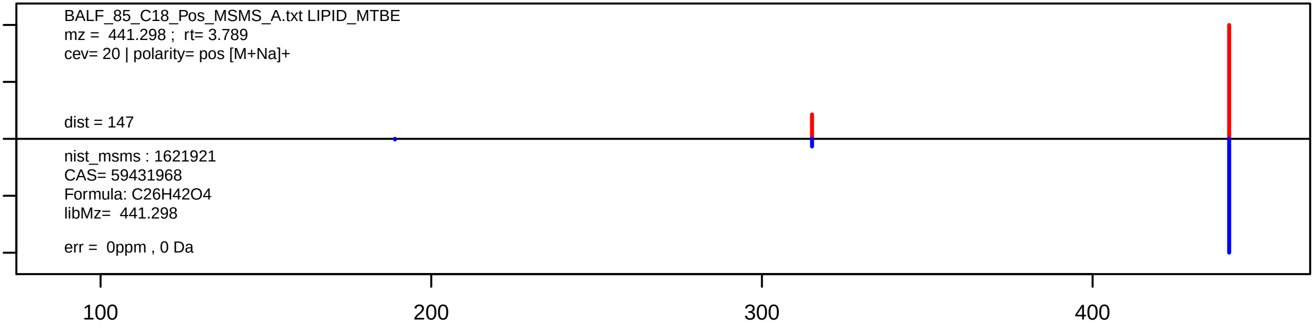

348 .   Dibenzylamine
Score=184 Dot=823 prob=75.6

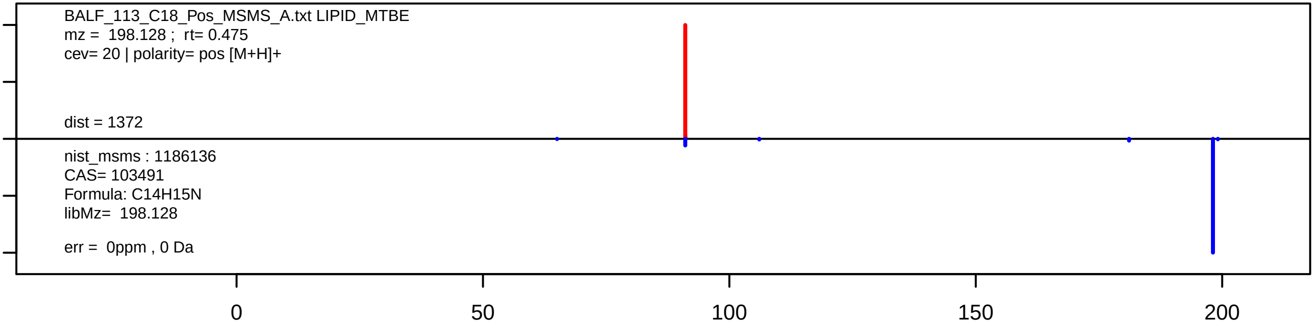

349 . Dibutyl phthalate
Score=282 Dot=979 prob=81

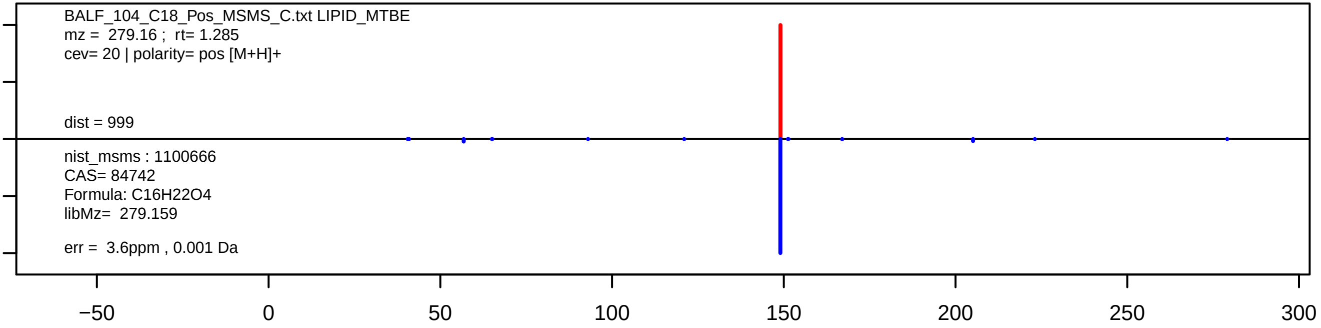

350 . Dicyclohexylamine
Score=254 Dot=927 prob=92

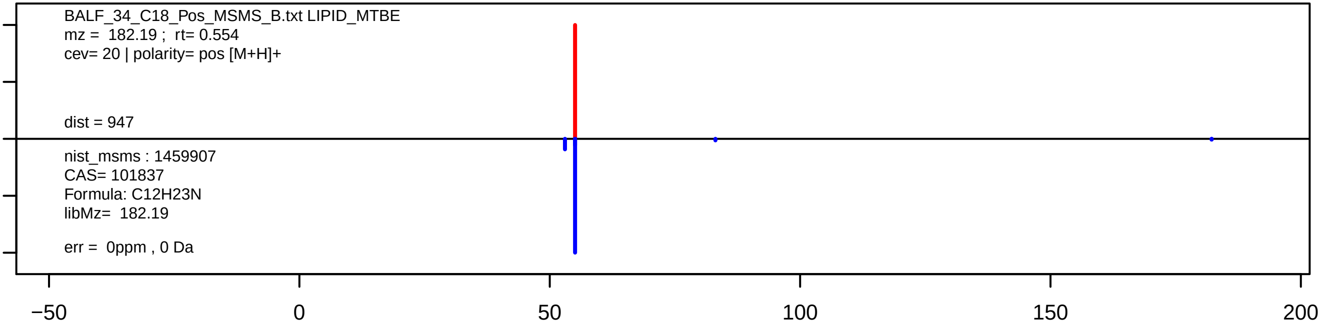

351 . Didodecyl 3,3'−thiodipropionate oxide
Score=352 Dot=985 prob=99

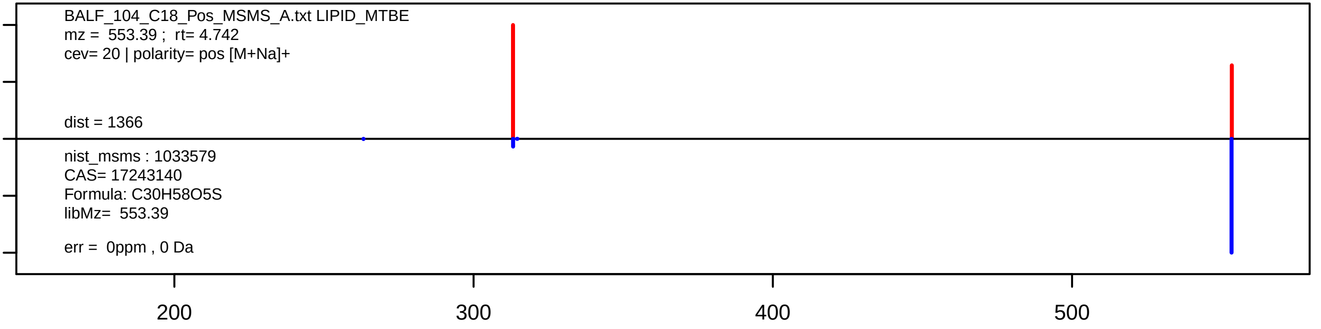

352 . Diisooctyl phthalate
Score=259 Dot=885 prob=46.1

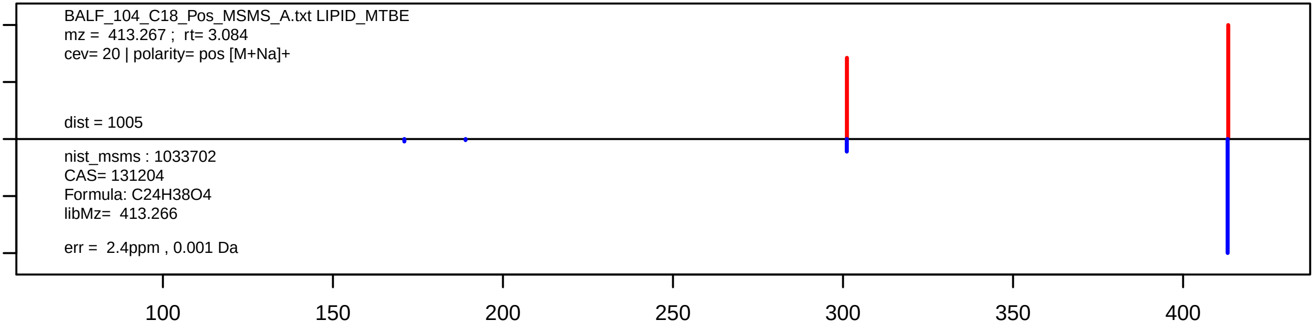

353 . Diltiazem
Score=400 Dot=999 prob=97.3

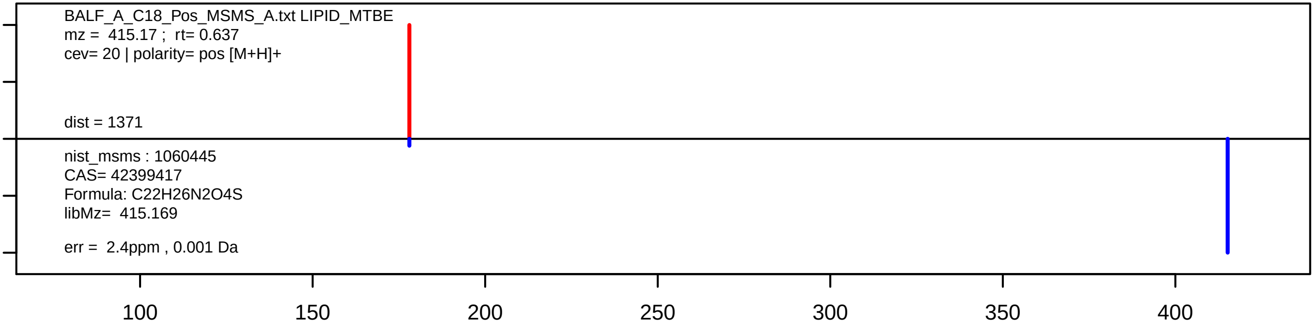

354 . Dimethocaine
Score=317 Dot=804 prob=51.6

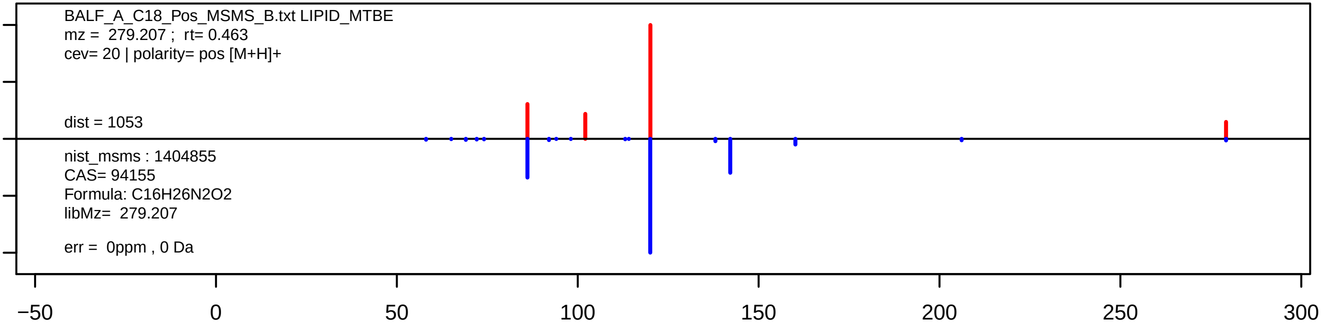

355 . Dimethyldioctadecylammonium cation
Score=127 Dot=805 prob=69.6

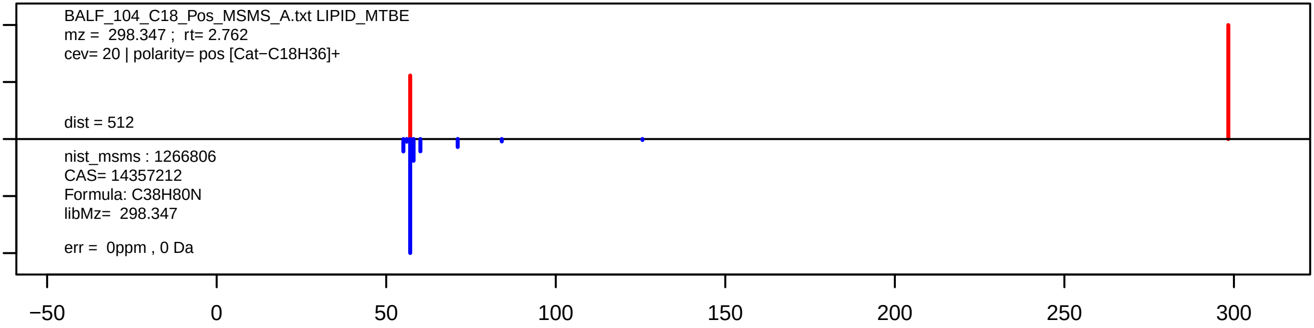

356 . Dioctyl phthalate
Score=241 Dot=906 prob=81.3

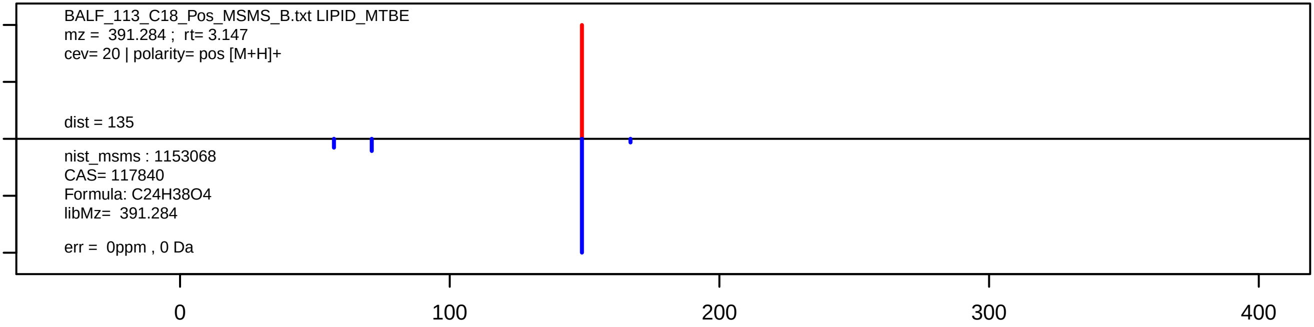

357 . Diphenhydramine
Score=626 Dot=975 prob=98.9

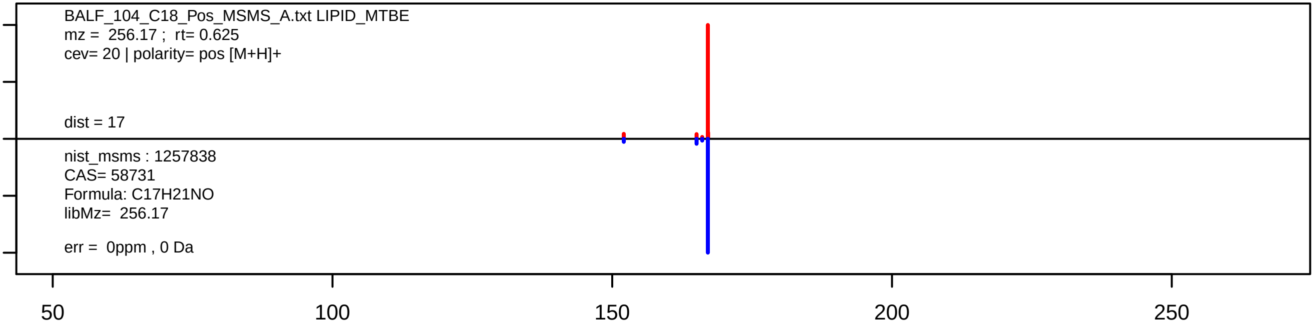

358 . Dipropyl phthalate
Score=373 Dot=994 prob=71.3

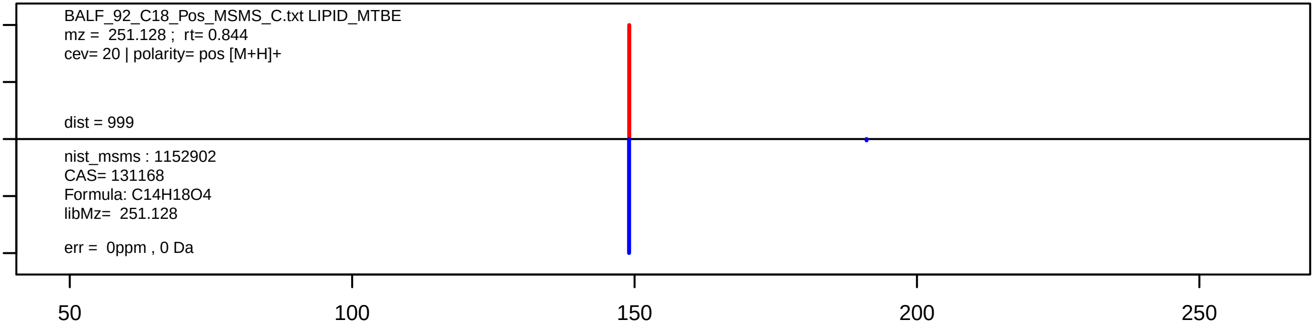

359 . Docosanamide
Score=604 Dot=841 prob=98.9

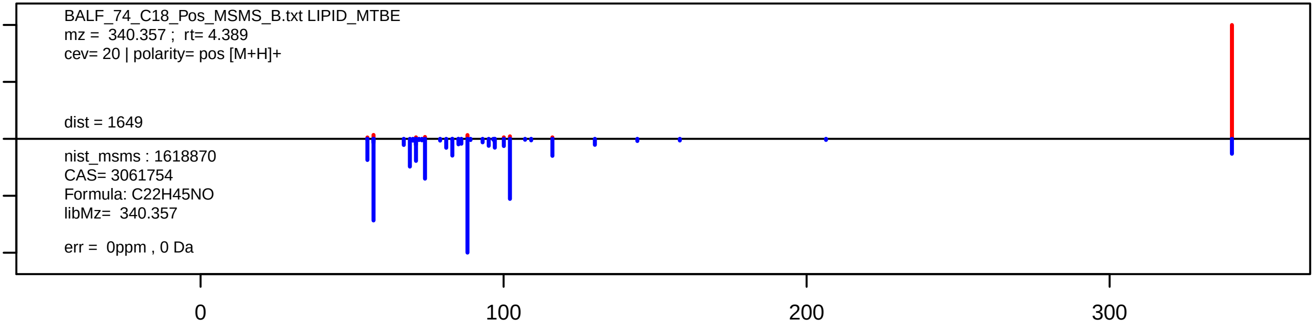

360 . Dodecamethylcyclohexasiloxane
Score=254 Dot=830 prob=96.5

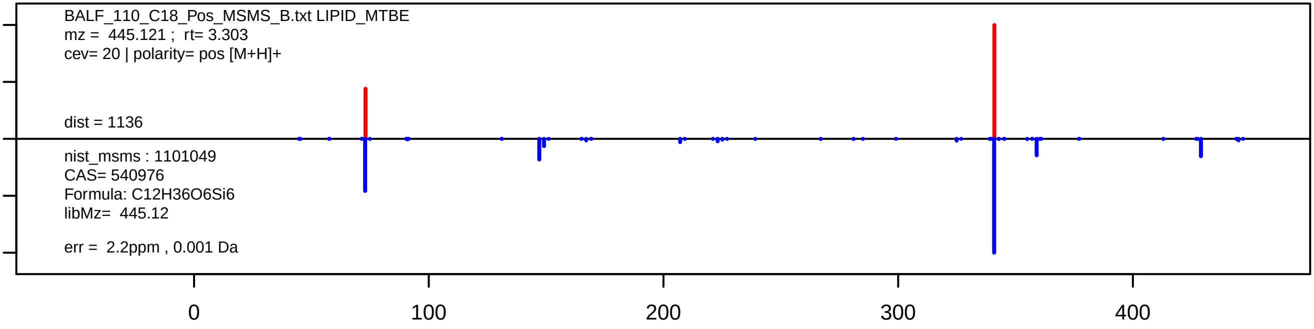

361 . Epitestosterone
Score=529 Dot=963 prob=97.2

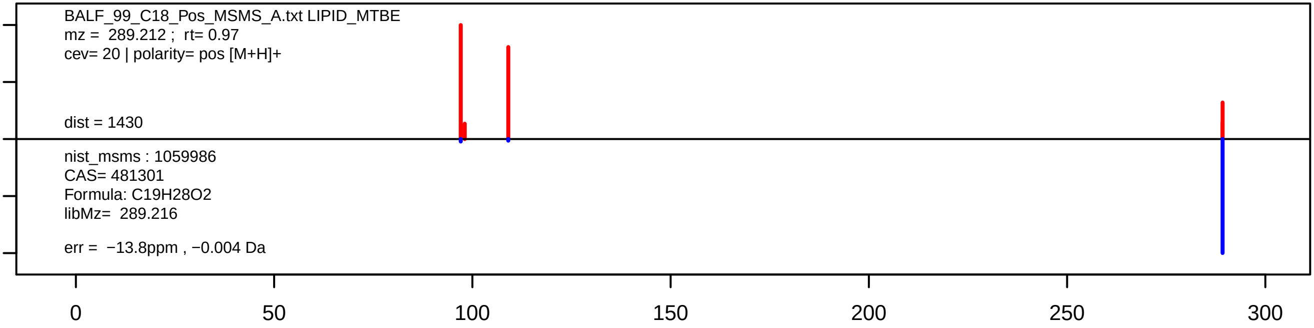

362 . Erucamide
Score=400 Dot=999 prob=96.2

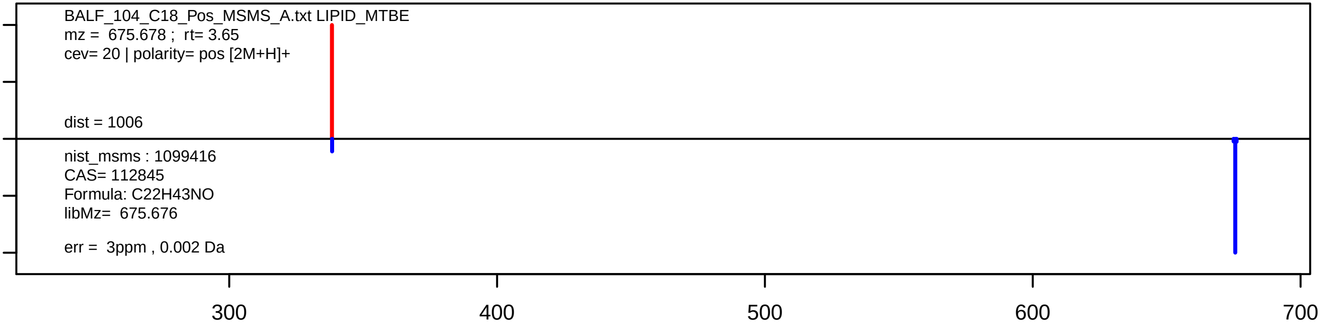

363 . Ethyl tramadol
Score=359 Dot=985 prob=98

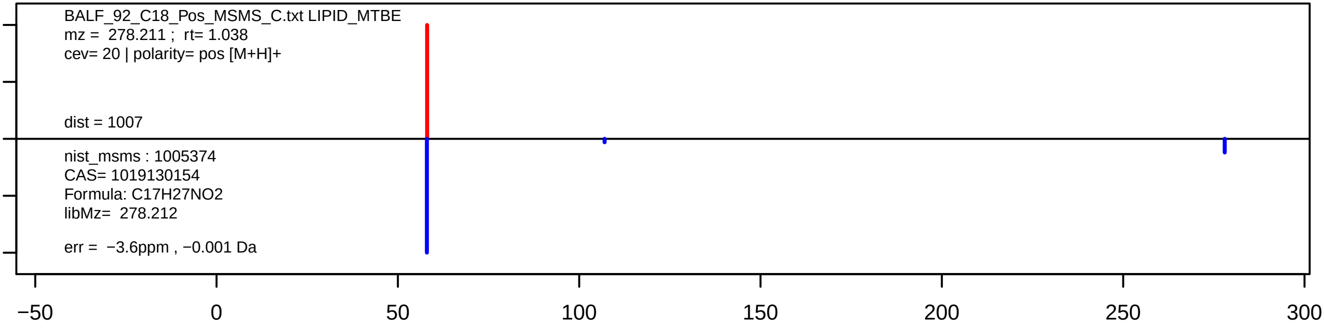

364 . Ethylhexadecyldimethylammonium cation
Score=184 Dot=808 prob=81.8

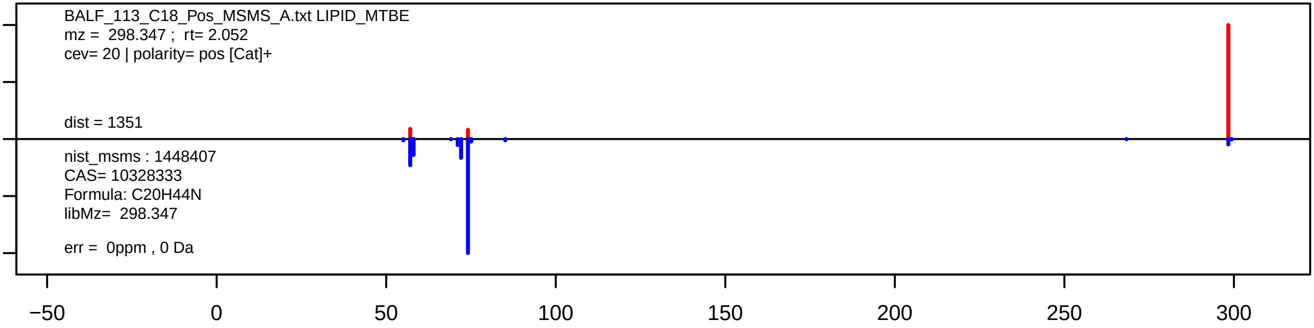

365 . Fluazifop−P
Score=400 Dot=999 prob=84.2

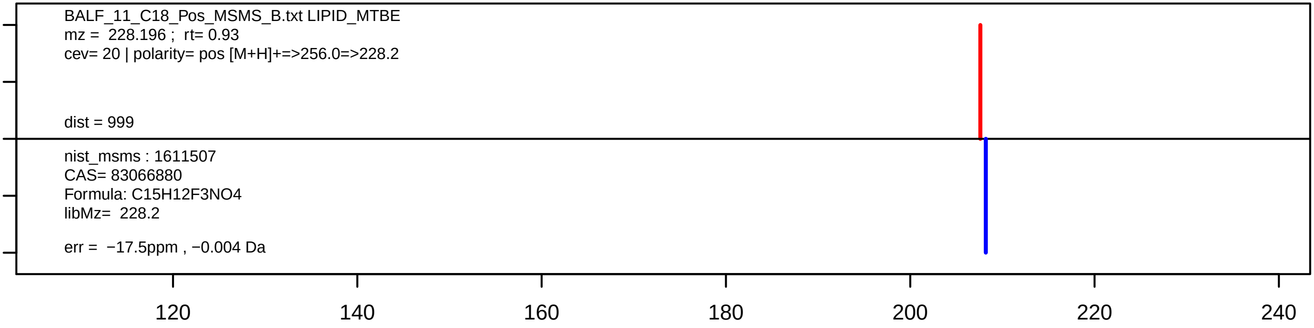

366 . Fluoxetine
Score=321 Dot=903 prob=97.3

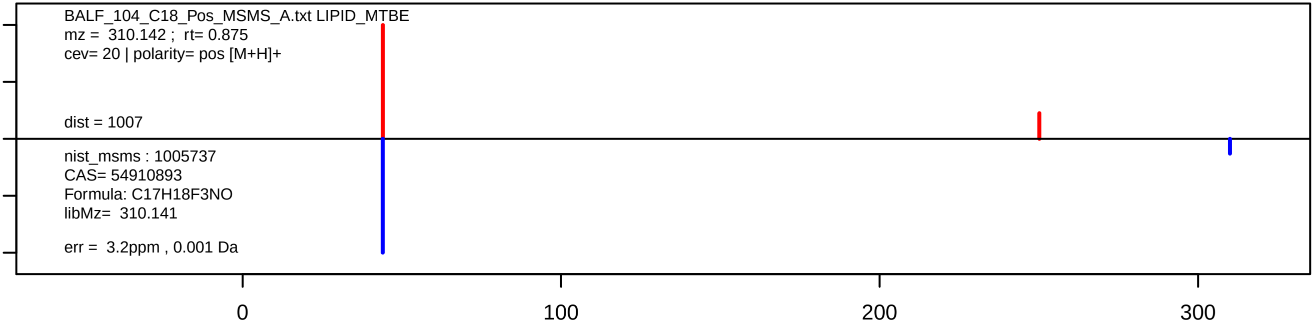

367 . Hemin cation
Score=346 Dot=910 prob=92.5

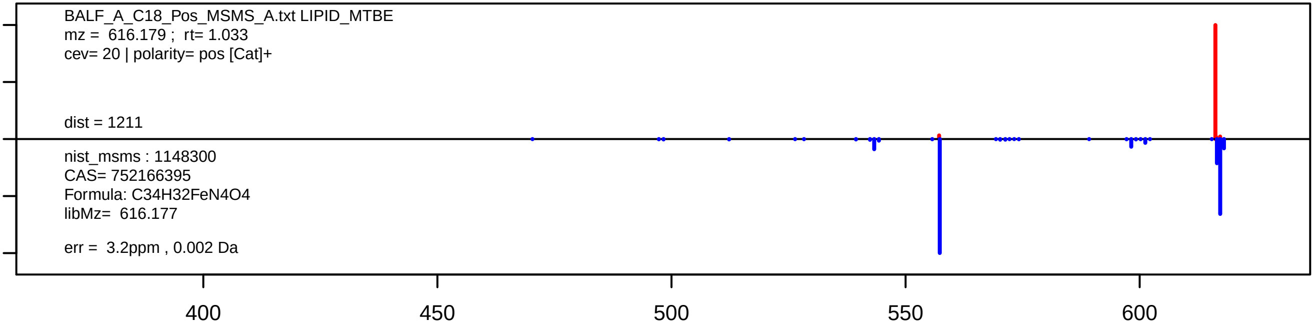

368 . Hexadecyltrimethylammonium cation
Score=232 Dot=883 prob=94.6

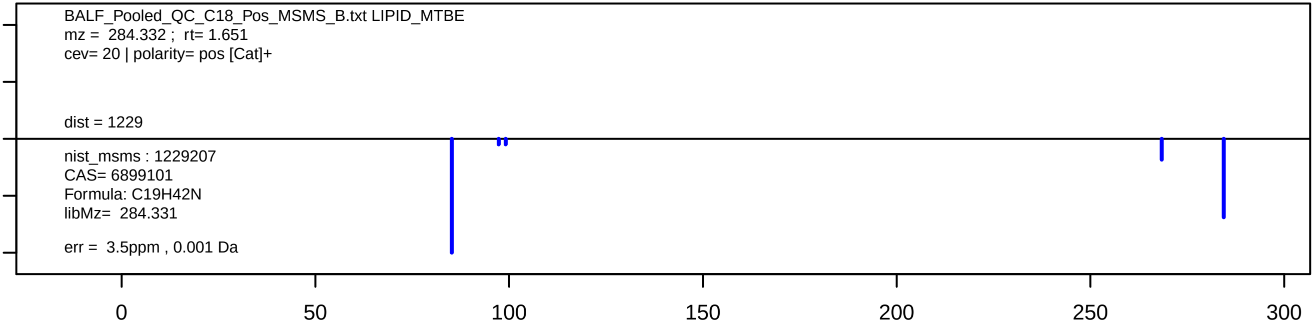

369 . Hexamethylcyclotrisiloxane
Score=351 Dot=847 prob=86.9

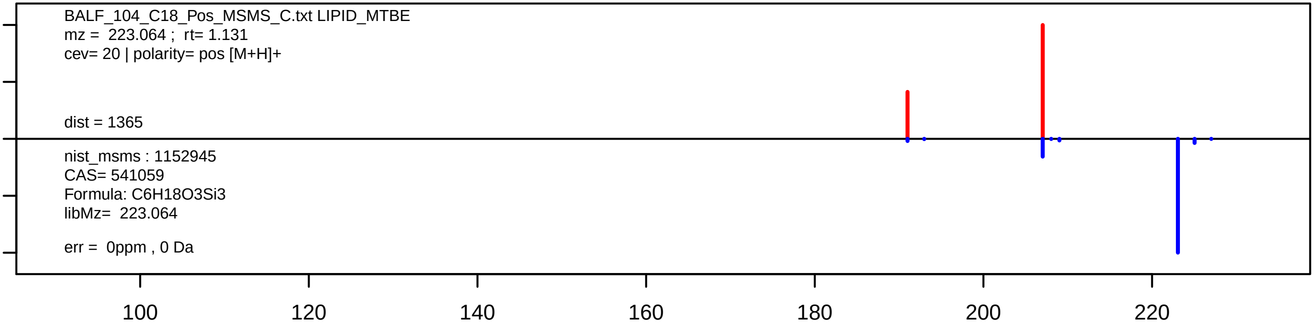

370 . Hydroxybupropion
Score=784 Dot=879 prob=96.6

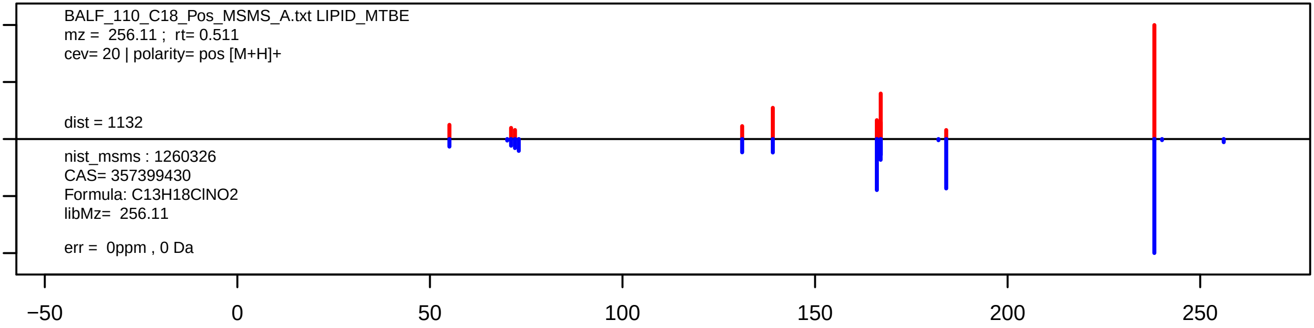

371 . Hydroxyzine
Score=275 Dot=965 prob=95.8

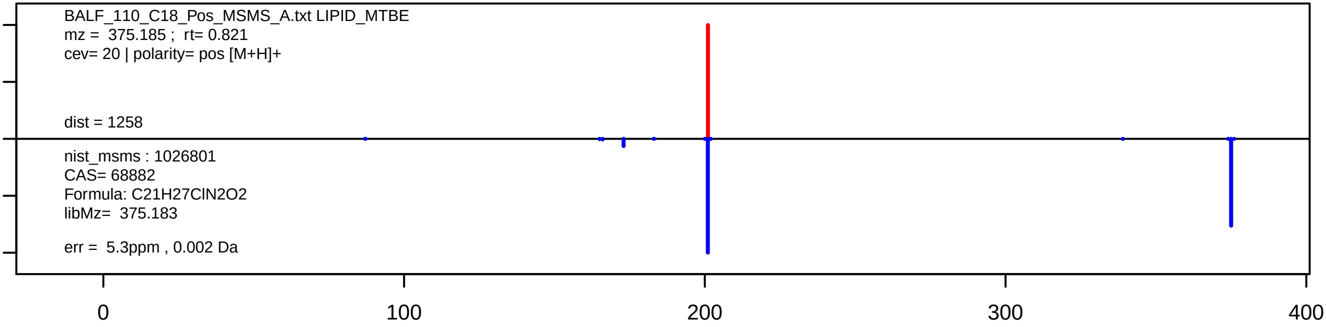

372 . L−Isoleucine
Score=400 Dot=999 prob=12.5

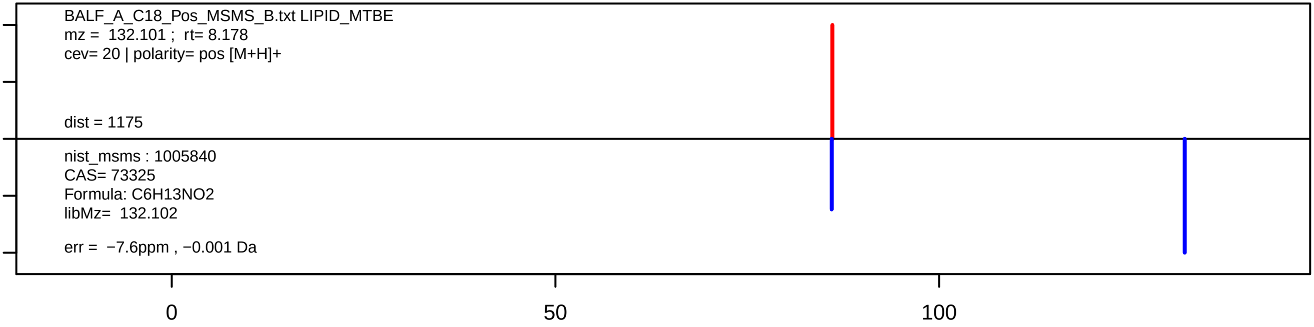

373 . L−Leucine
Score=797 Dot=930 prob=98.9

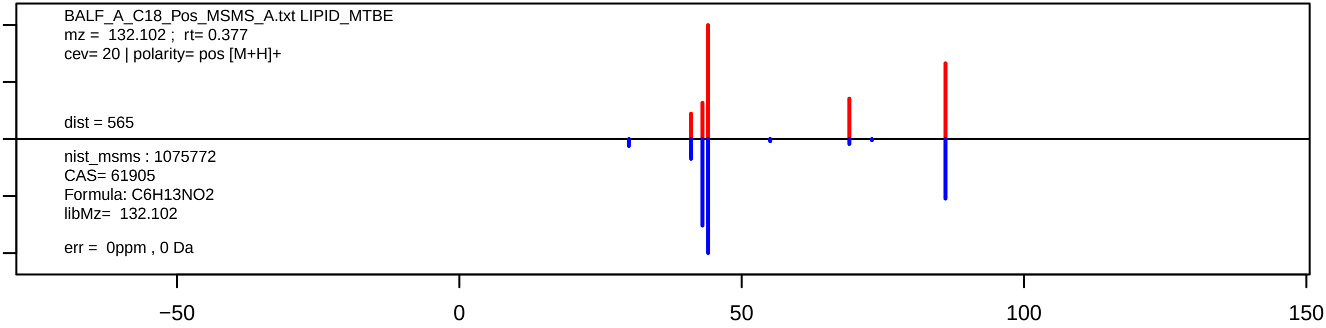

374 . L−Valine
Score=475 Dot=853 prob=79.6

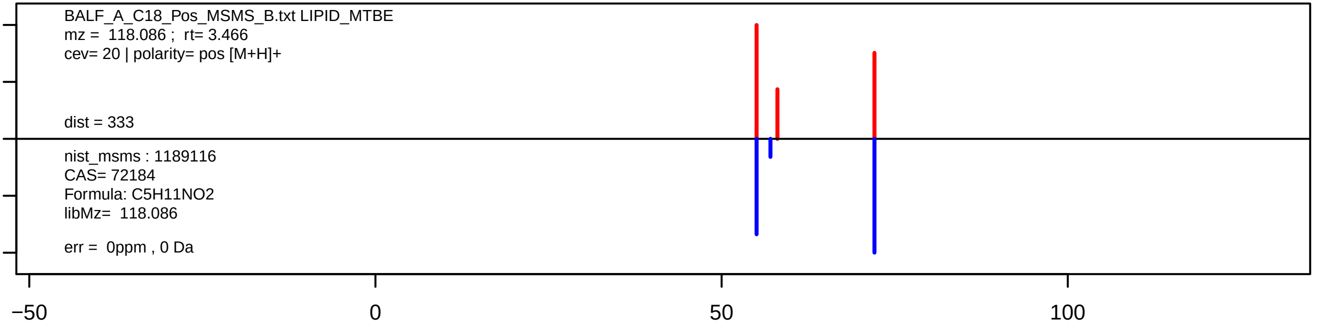

375 . Leu−Leu
Score=354 Dot=916 prob=64

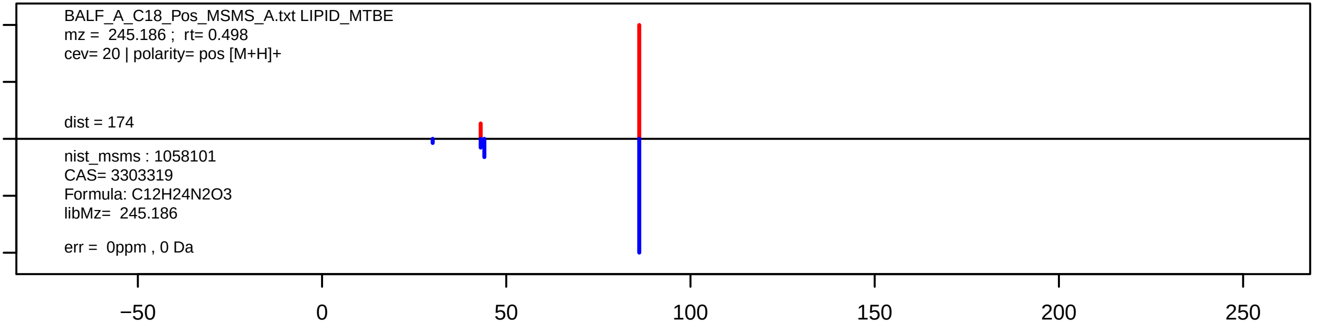

376 . Lidocaine
Score=399 Dot=999 prob=48.9

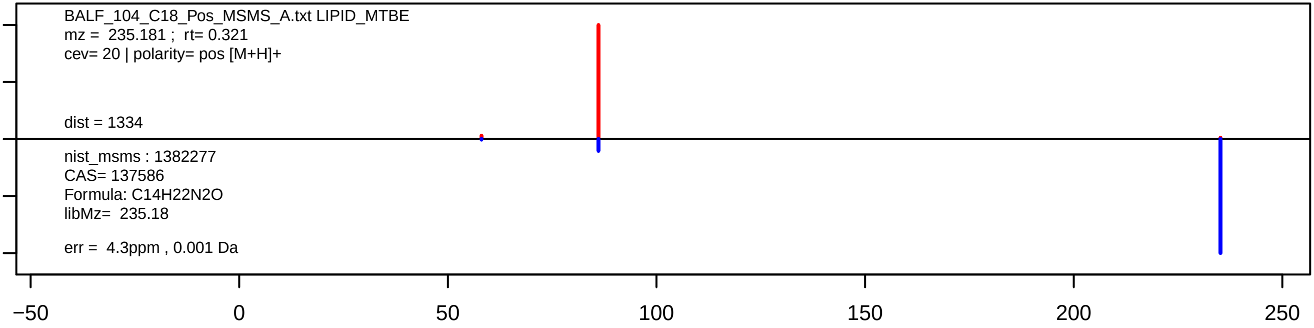

377 . lysoPC 18:0; [M+Na]+; PC(18:0/0:0)
Score=375 Dot=995 prob=53.1

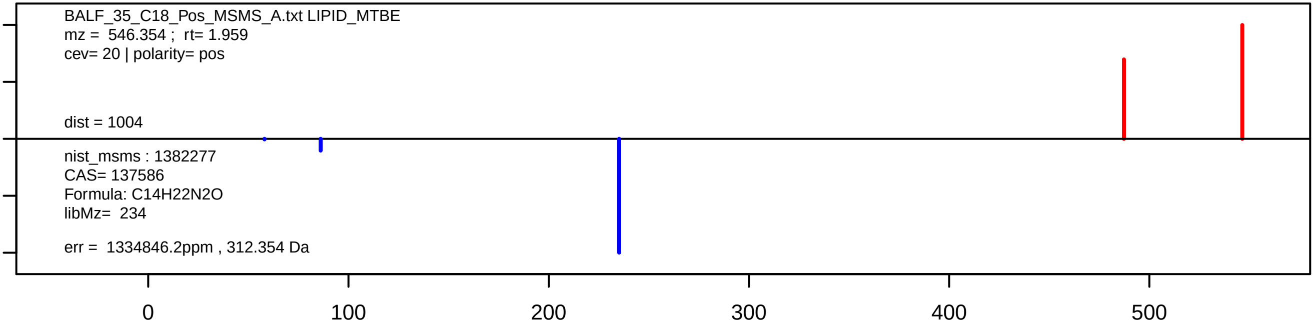

378 . lysoPC 18:2; [M+Na]+; PC(18:2(2E,4E)/0:0)
Score=251 Dot=808 prob=16.3

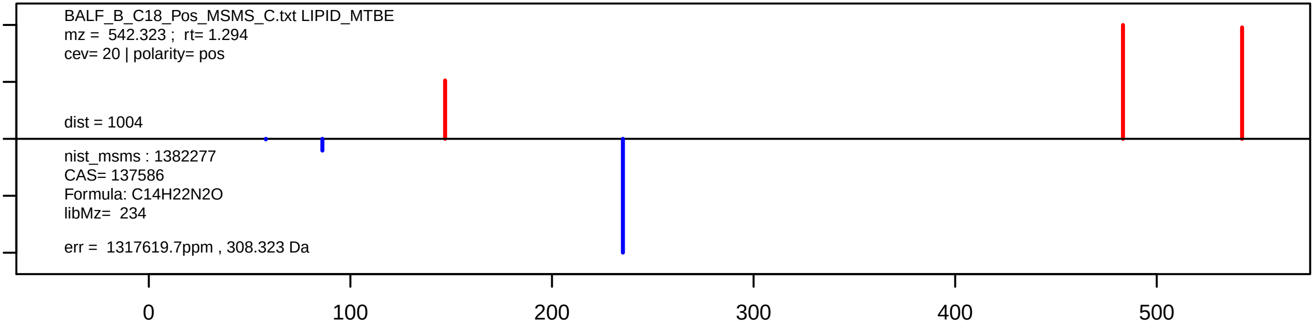

379 .   Mefenorex
Score=340 Dot=802 prob=97.1

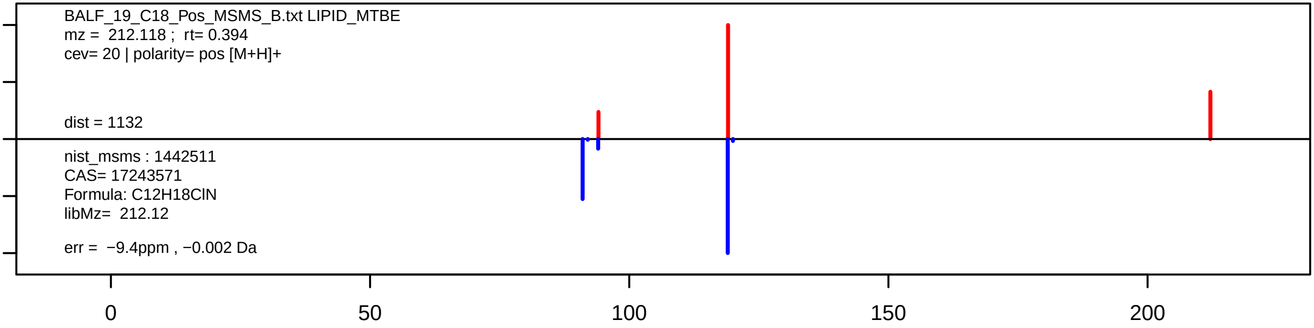

380 .   Metformin
Score=588 Dot=877 prob=95.8

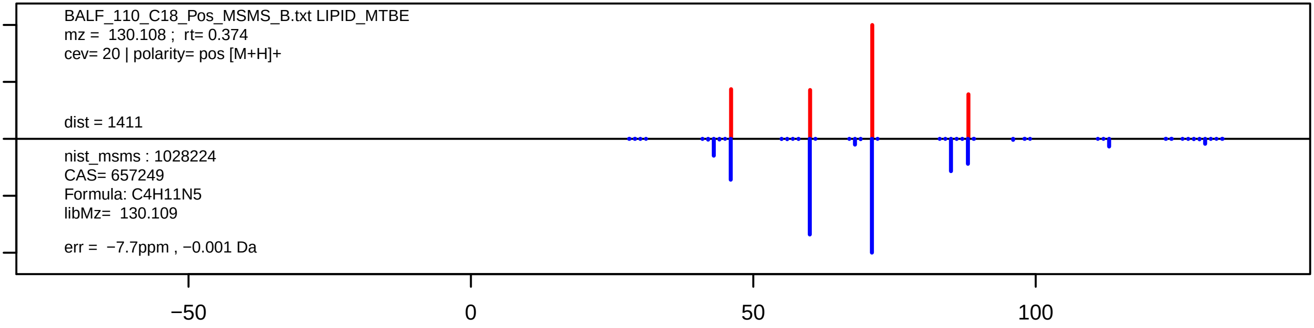

381 .   Methadone
Score=328 Dot=842 prob=80.1

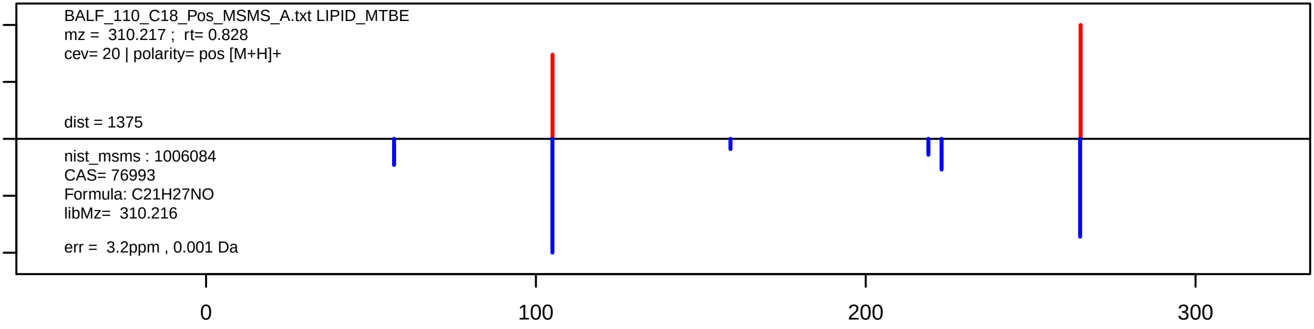

382 .   Mono−2−ethylhexyl phthalate
Score=389 Dot=861 prob=95.2

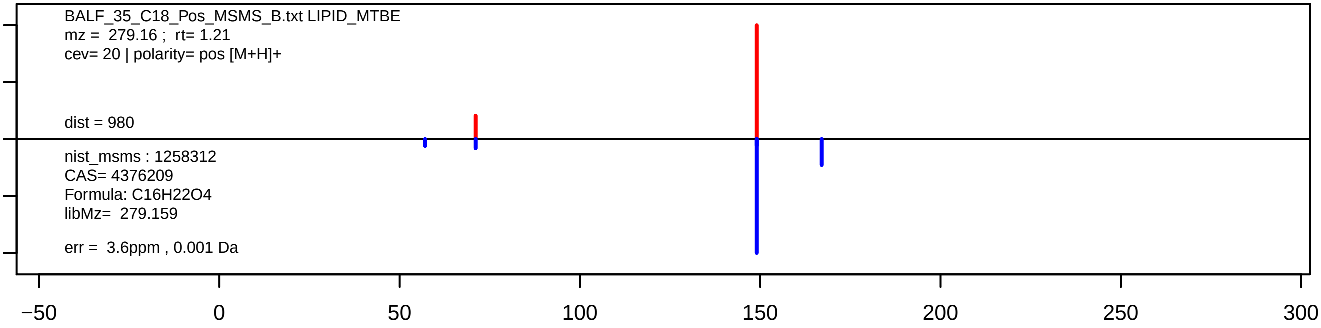

383 .   Monoisobutyl phthalate
Score=274 Dot=980 prob=39.1

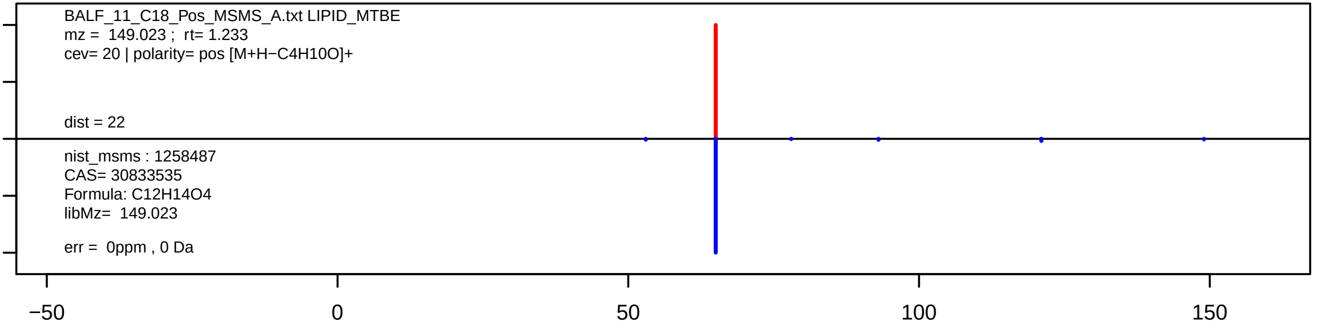

384 .   N−(Octadecanoyl)sphing−4−enine−1−phosphocholine
Score=400 Dot=999 prob=99

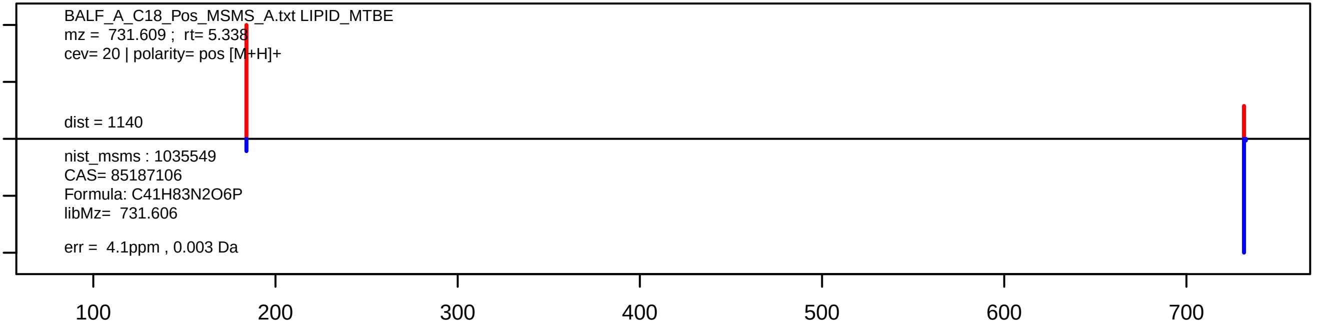

385 . N−Desmethyltramadol
Score=302 Dot=957 prob=98.5

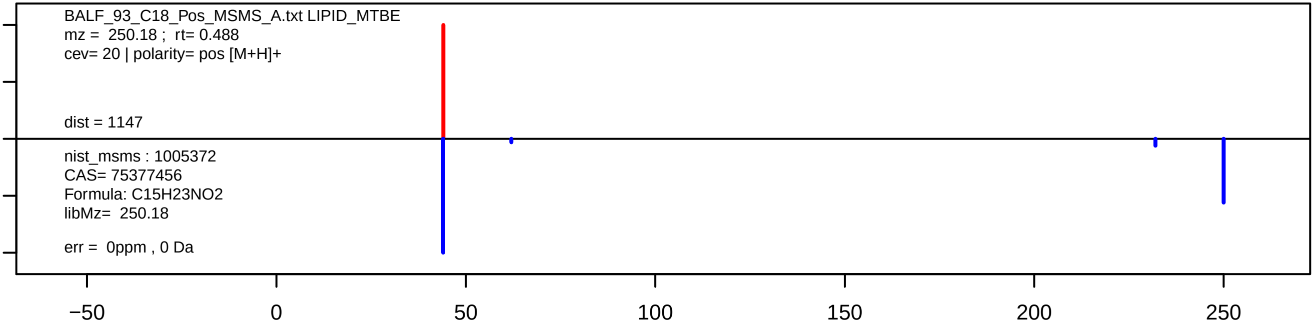

386 . N−Docosanoyl−4−sphingenyl−1−O−phosphorylcholine
Score=355 Dot=982 prob=91.7

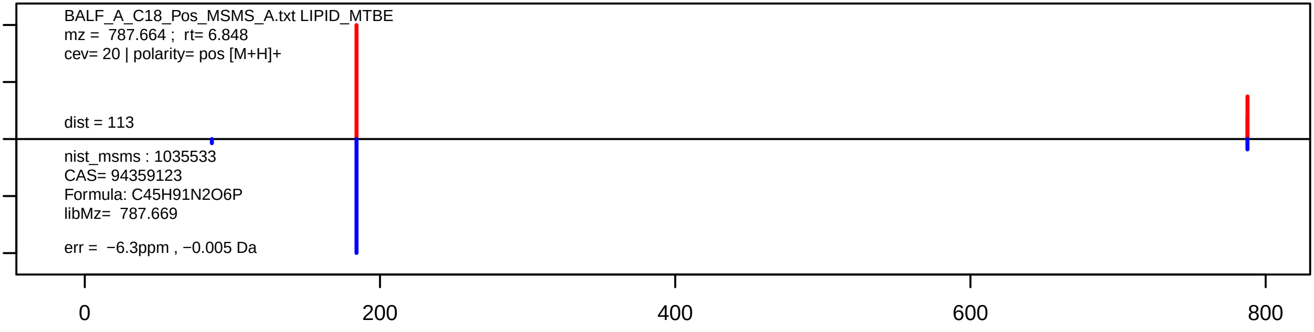

387 . N−Nervonoyl−D−erythro−sphinganine
Score=167 Dot=950 prob=84.2

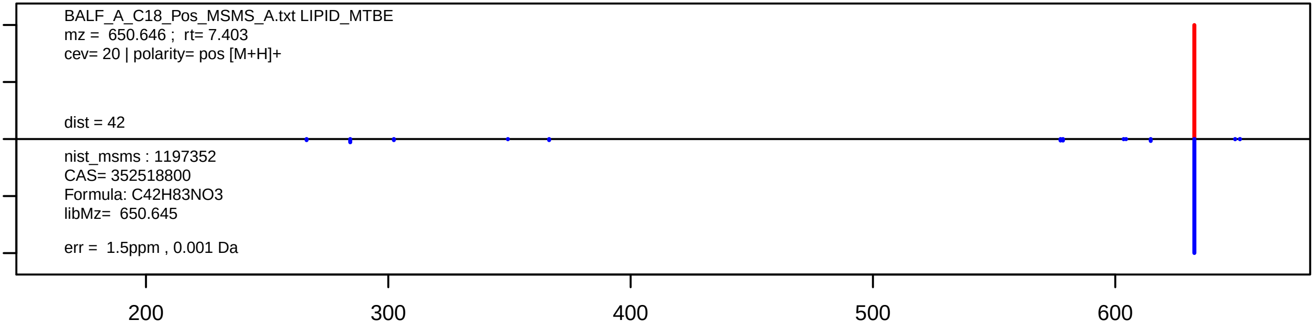

388 . N−Nervonoyl−D−erythro−sphingosylphosphorylcholine
Score=400 Dot=999 prob=100

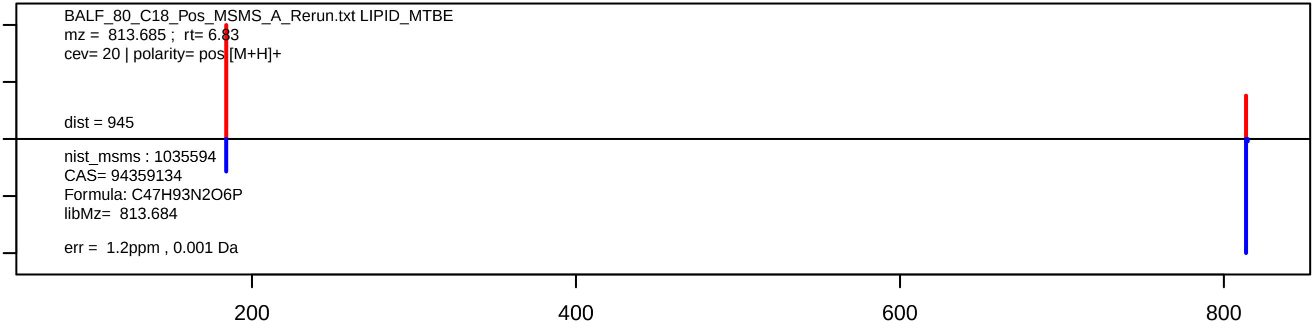

389 . n−Octadecylamine
Score=303 Dot=953 prob=96.7

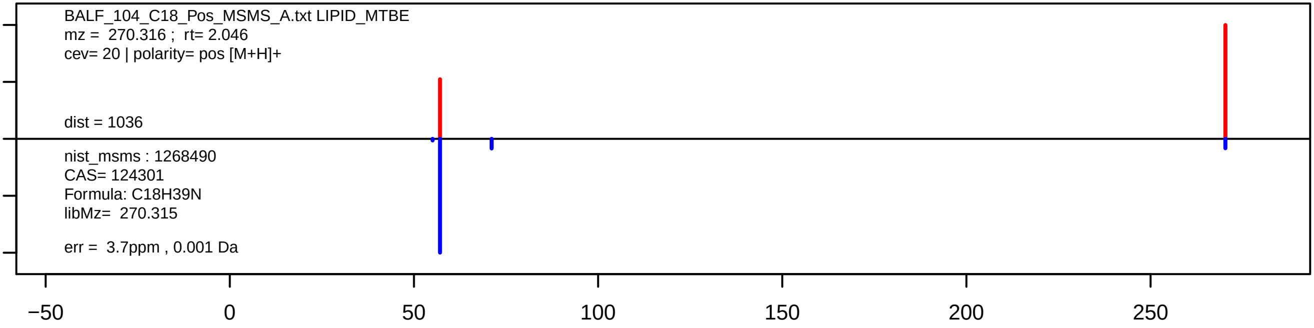

390 . N−Oleoyl−D−erythro−sphingosylphosphorylcholine
Score=400 Dot=999 prob=99

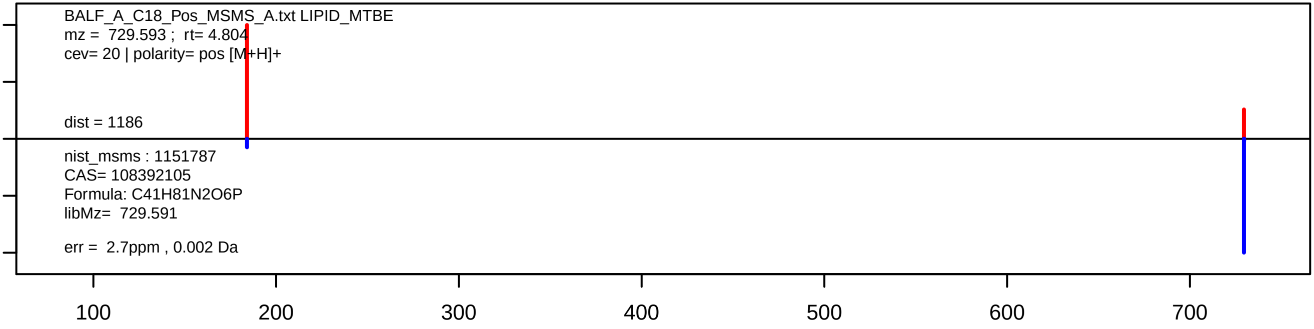

391 . N−Palmitoyl−D−sphingosine
Score=299 Dot=812 prob=98.8

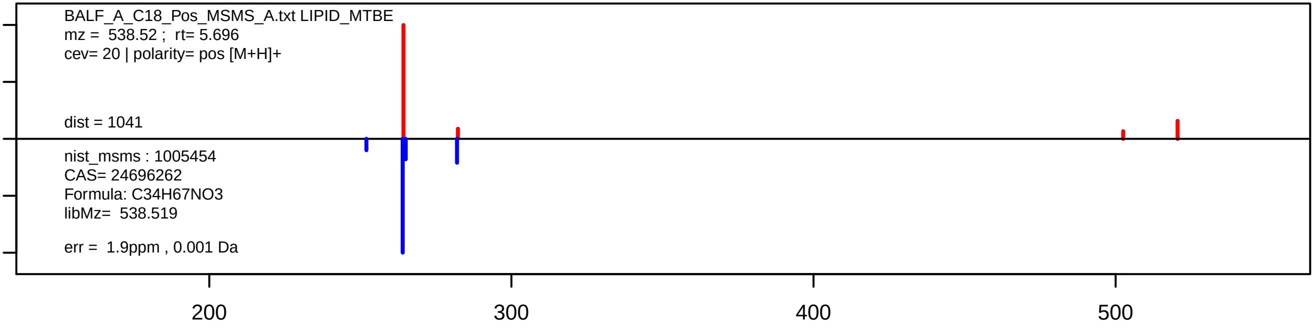

392 . N−Tetracosanoyl−4−sphingenyl−1−O−phosphorylcholine
Score=194 Dot=906 prob=98

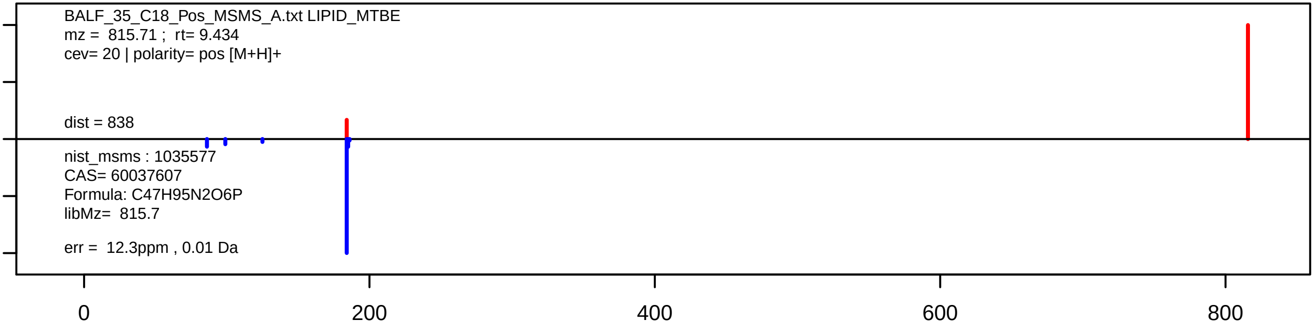

393 . N−Tetracosenoyl−4−sphingenine
Score=335 Dot=895 prob=97.4

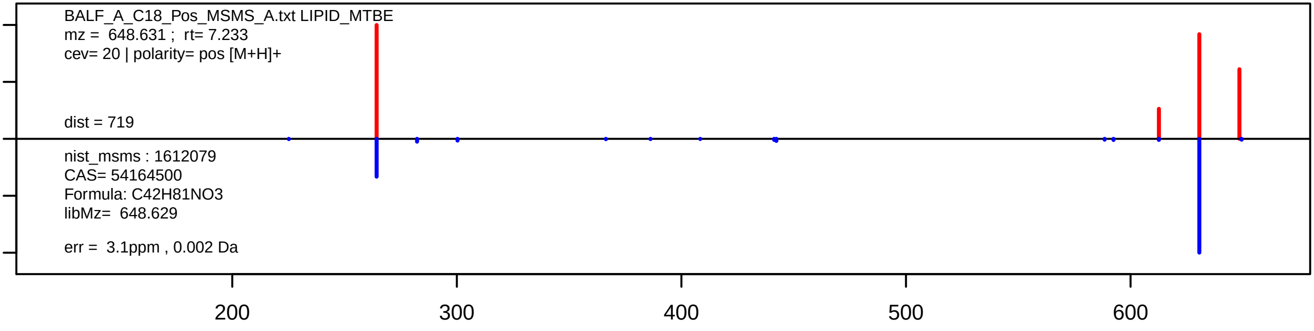

394 . N−Tigloylglycine
Score=486 Dot=992 prob=98.7

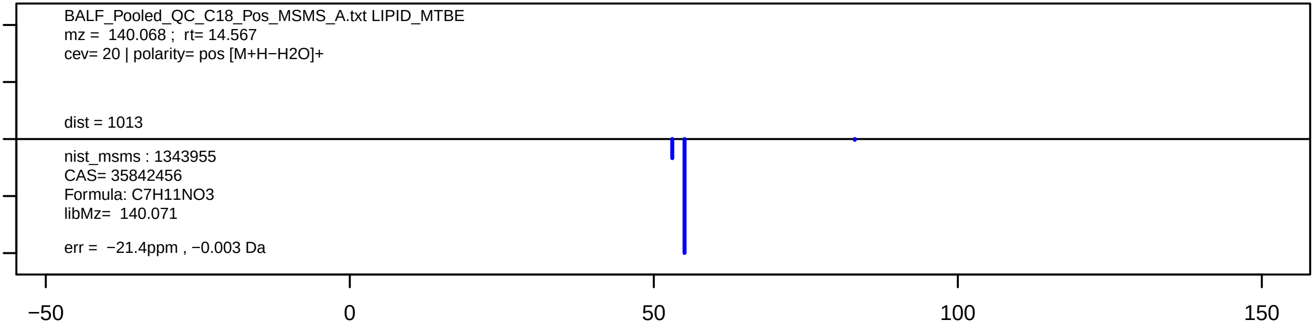

395 . N,N−Dimethylbenzylamine
Score=400 Dot=999 prob=20.8

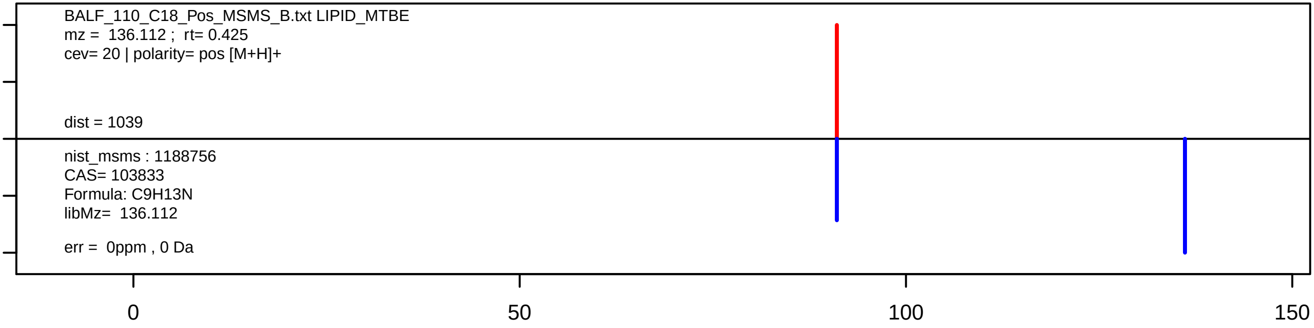

396 . Norquetiapine
Score=610 Dot=878 prob=99

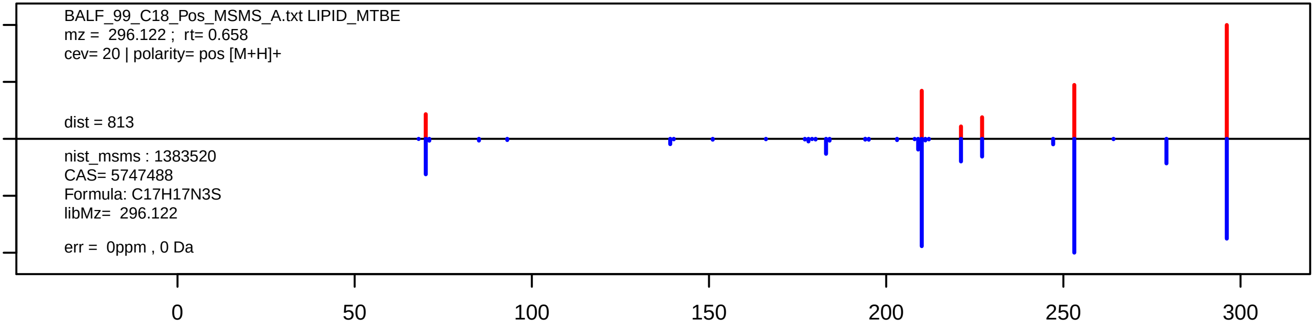

397 . Octamethylcyclotetrasiloxane
Score=569 Dot=947 prob=98.7

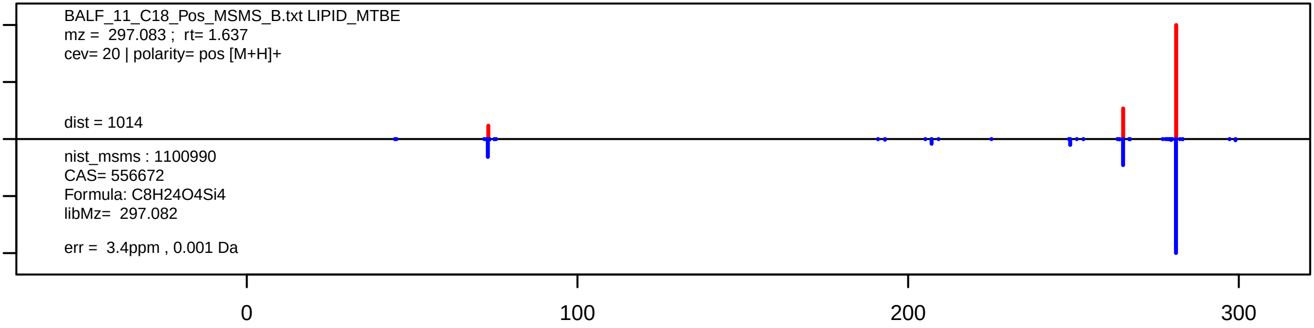

398 . Oleamide
Score=421 Dot=829 prob=89.6

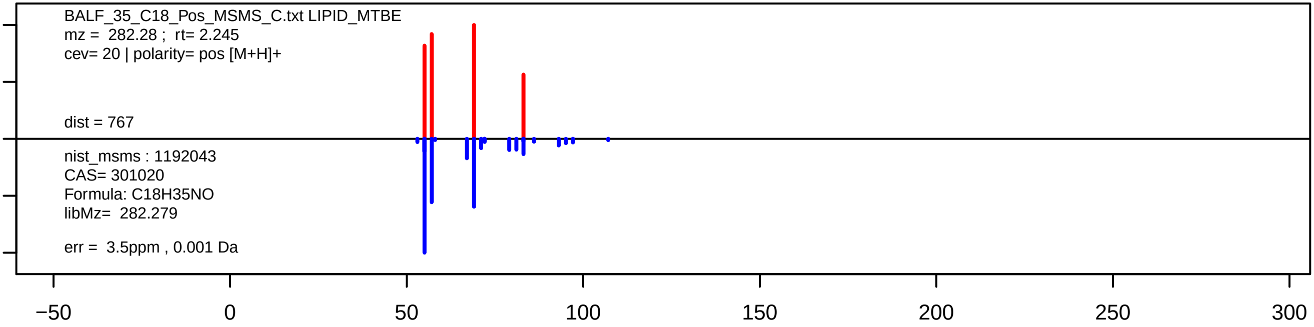

399 . Palmitoyl ethanolamide
Score=259 Dot=885 prob=19.1

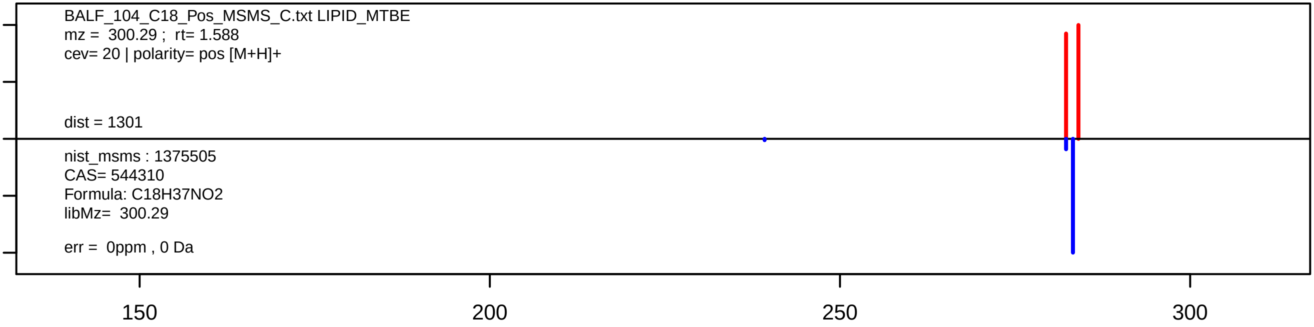

400 . Palmitoyl sphingomyelin
Score=400 Dot=999 prob=100

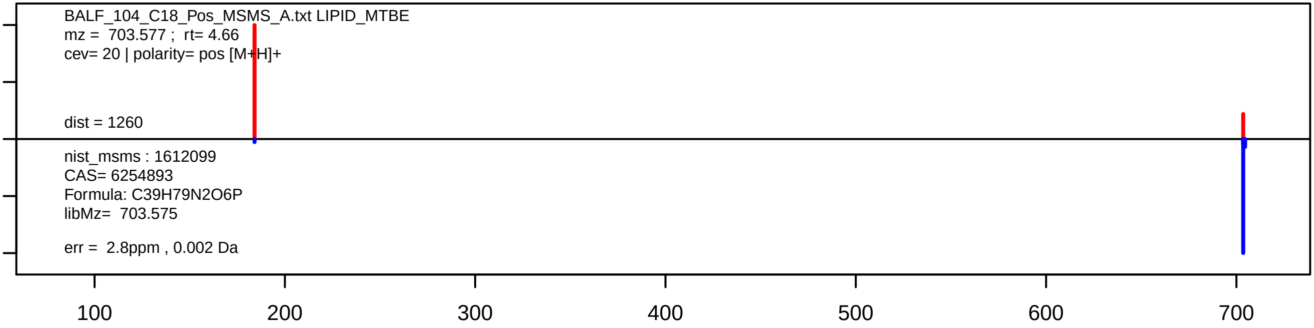

401 . Palmitoyl−L−carnitine
Score=232 Dot=831 prob=84.8

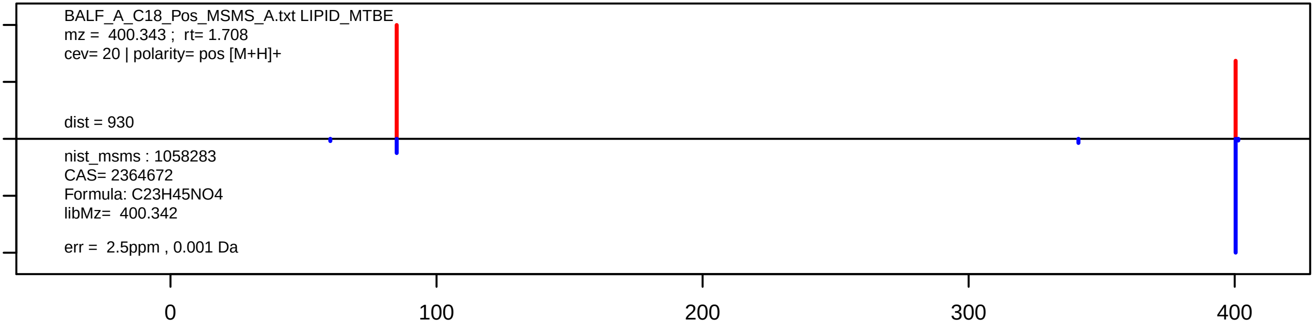

402 . Palmitoylcarnitine
Score=796 Dot=954 prob=58.4

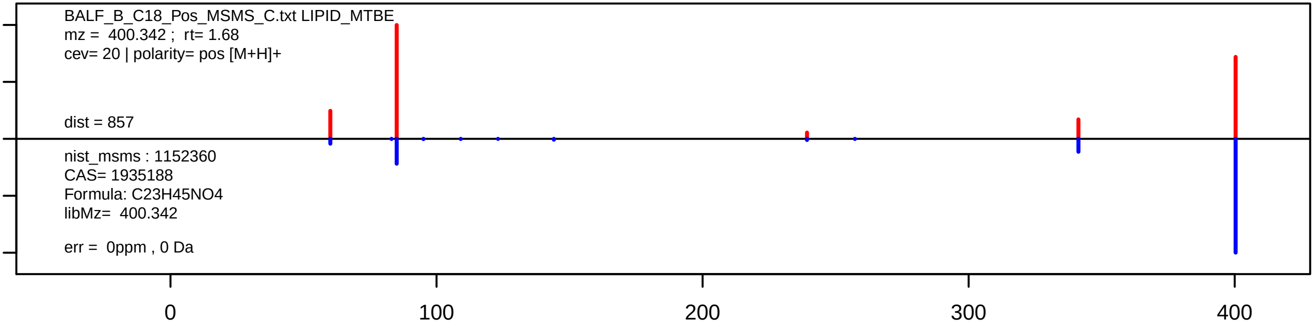

403 . Palmitoyleicosapentaenoyl phosphatidylcholine
Score=181 Dot=953 prob=100

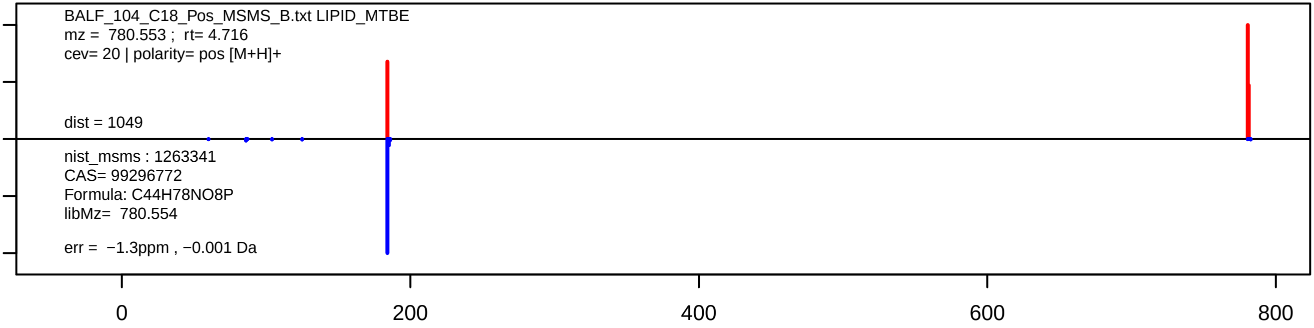

404 . PC 28:0; [M+Na]+; GPCho(14:0/14:0)
Score=530 Dot=975 prob=19.4

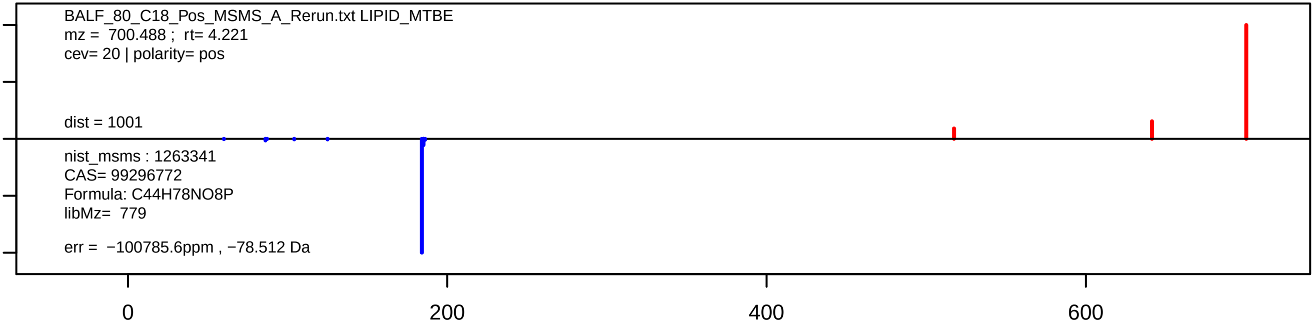

405 . PC 30:0; [M+Na]+; GPCho(15:0/15:0)
Score=529 Dot=975 prob=19.6

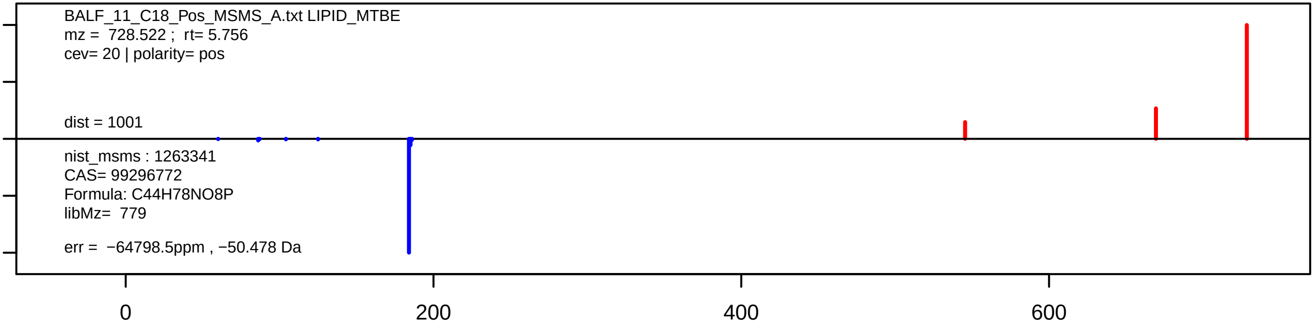

406 . PC 30:1; [M+Na]+; GPCho(4:0/26:1(5Z))
Score=478 Dot=952 prob=2.4

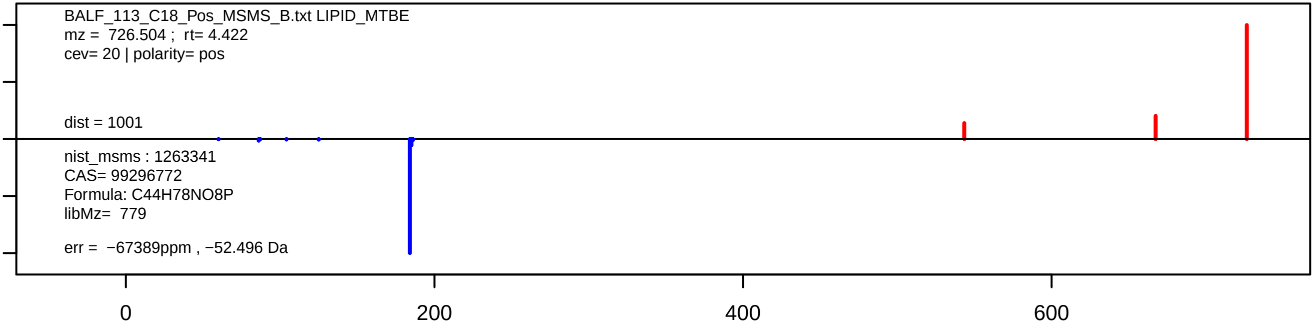

407 . PC 32:0; [M+Na]+; GPCho(16:0/16:0)
Score=370 Dot=842 prob=24.1

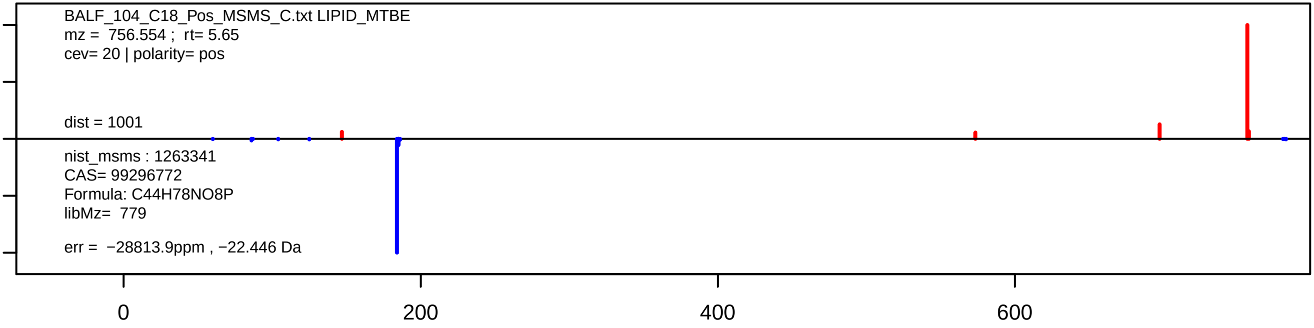

408 . PC 32:1; [M+Na]+; GPCho(6:0/26:1(5Z))
Score=329 Dot=852 prob=2.4

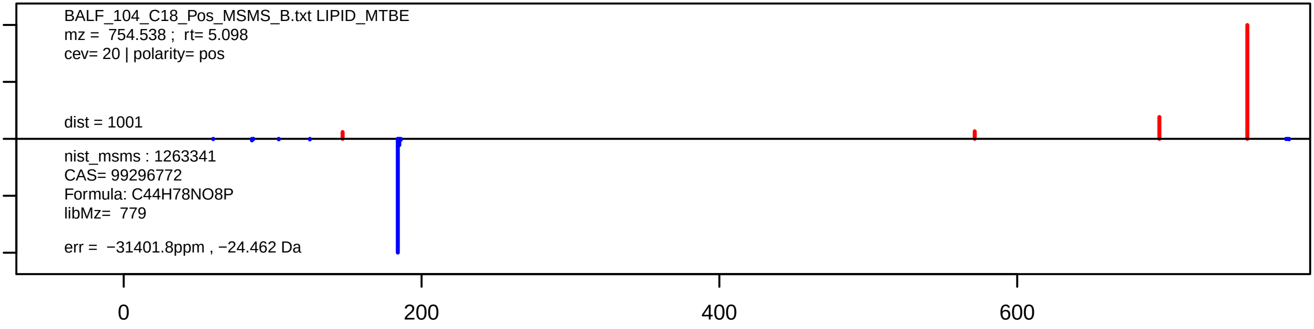

409 . PC 32:2; [M+Na]+; GPCho(16:1(7Z)/16:1(7Z))
Score=467 Dot=967 prob=7.3

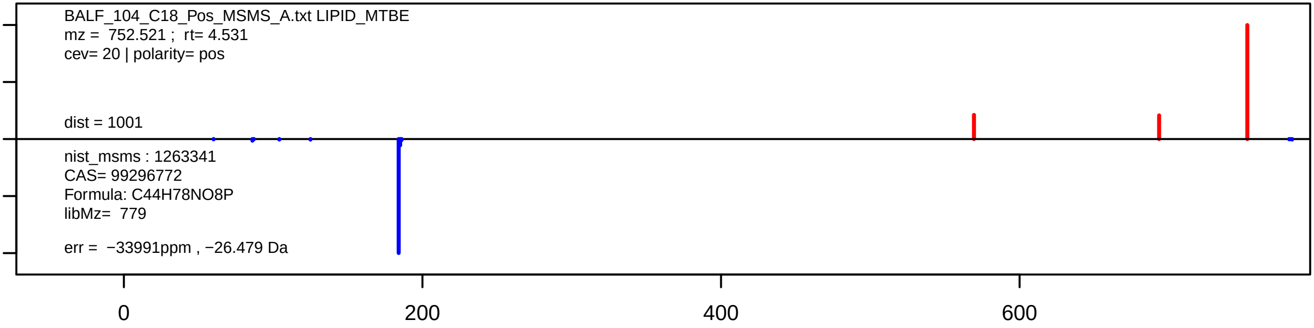

410 . PC 33:0; [M+Na]+; GPCho(7:0/26:0)
Score=427 Dot=946 prob=4.9

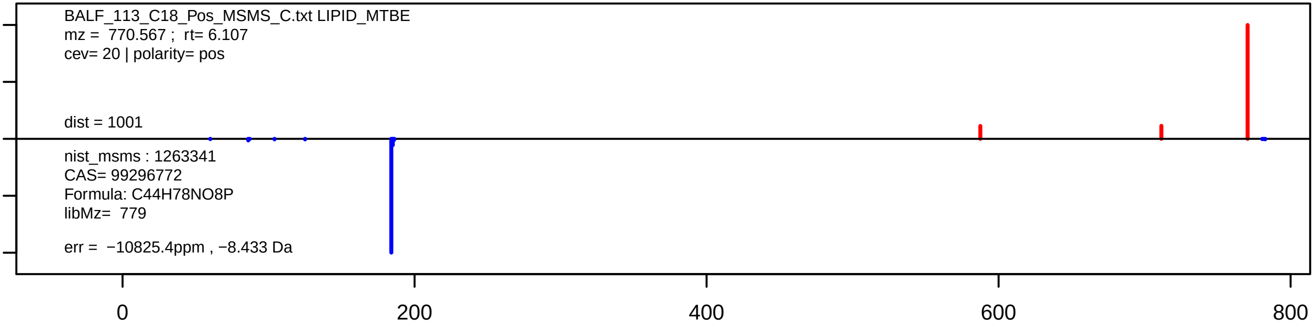

411 . PC 33:1; [M+Na]+; GPCho(7:0/26:1(5Z))
Score=291 Dot=807 prob=2.4

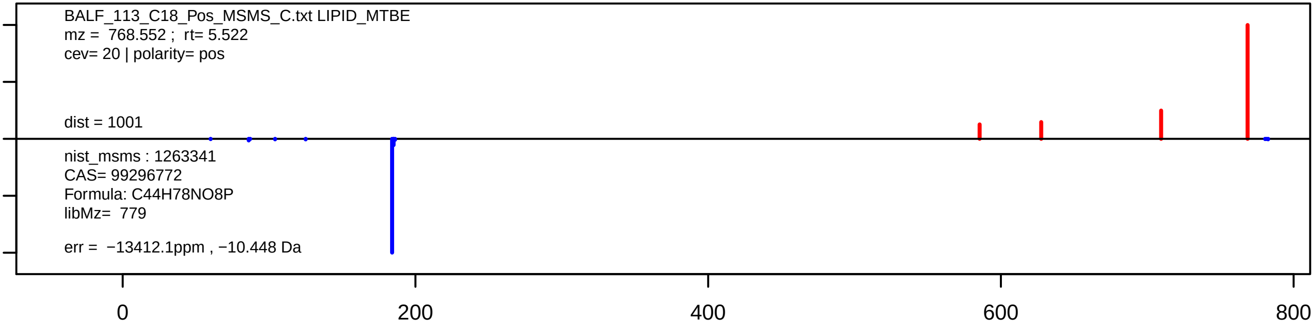

412 . PC 34:0; [M+Na]+; GPCho(17:0/17:0)
Score=372 Dot=851 prob=24.9

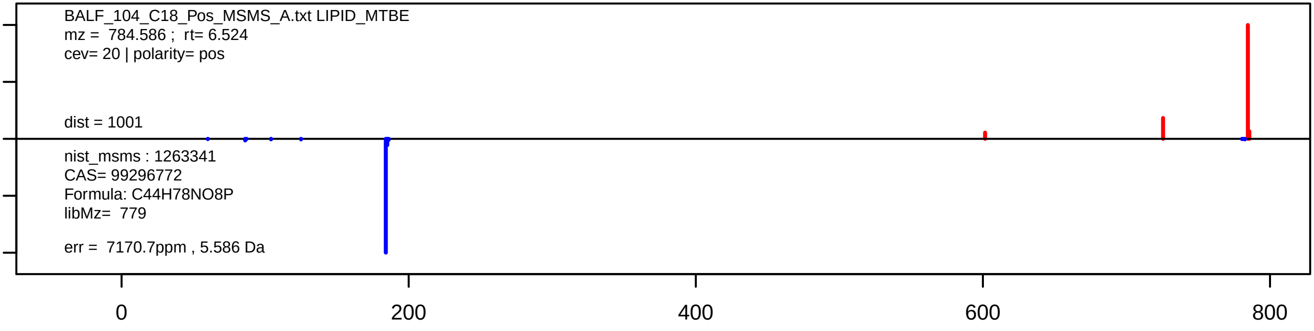

413 . PC 34:1; [M+Na]+; GPCho(8:0/26:1(5Z))
Score=477 Dot=952 prob=2.4

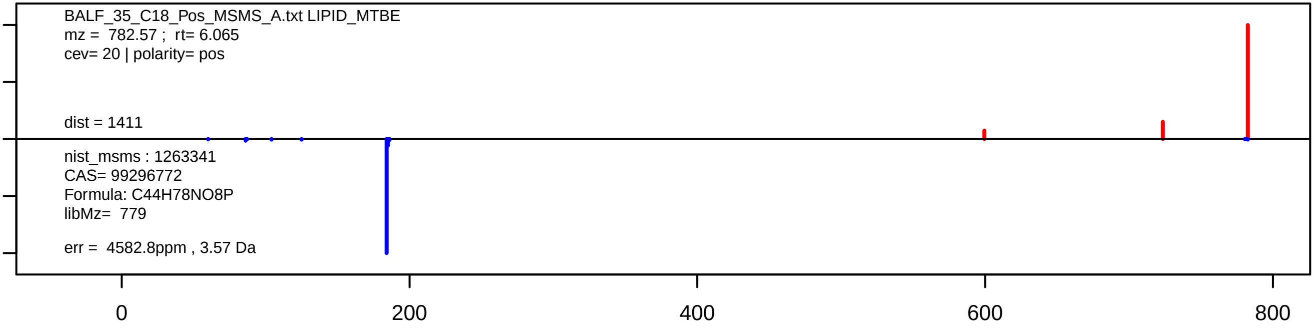

414 . PC 34:2; [M+Na]+; GPCho(17:1(9Z)/17:1(9Z))
Score=348 Dot=825 prob=9.1

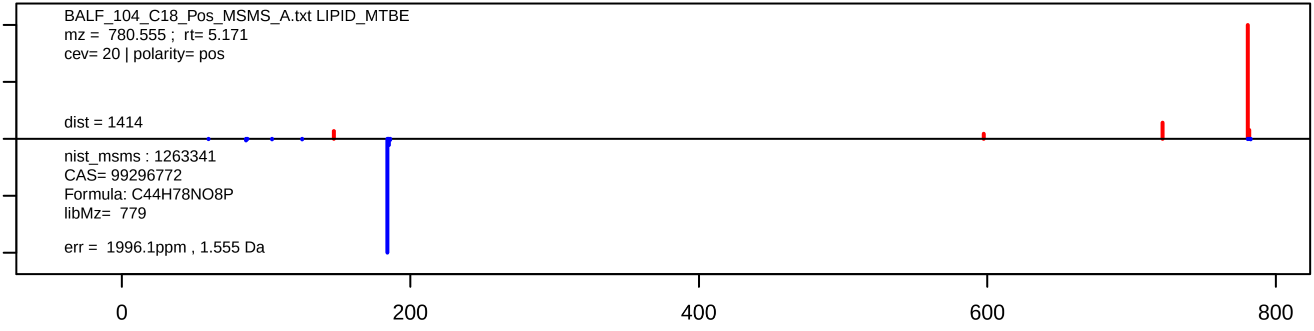

415 . PC 34:3; [M+Na]+; GPCho(14:0/20:3(5Z,8Z,11Z))
Score=465 Dot=948 prob=2.6

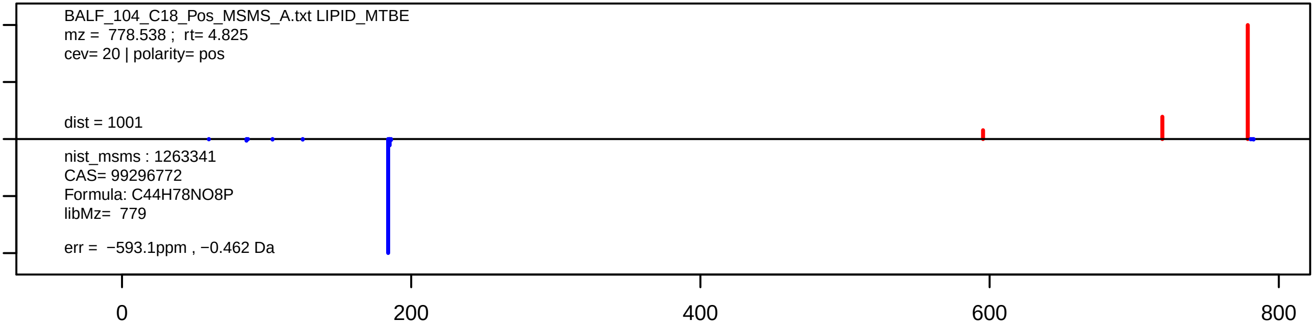

416 . PC 35:2; [M+Na]+; GPCho(9:0/26:2(5E,9Z))
Score=445 Dot=940 prob=1.9

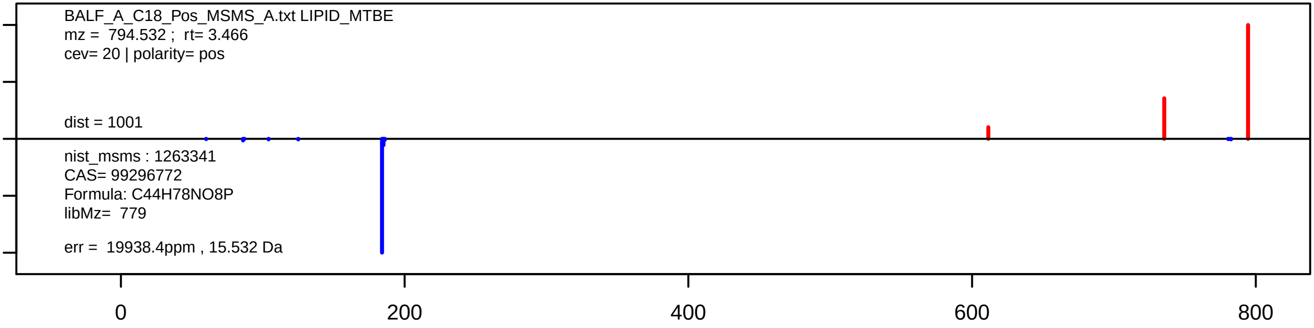

417 . PC 36:1; [M+Na]+; GPCho(10:0/26:1(5Z))
Score=470 Dot=950 prob=2.4

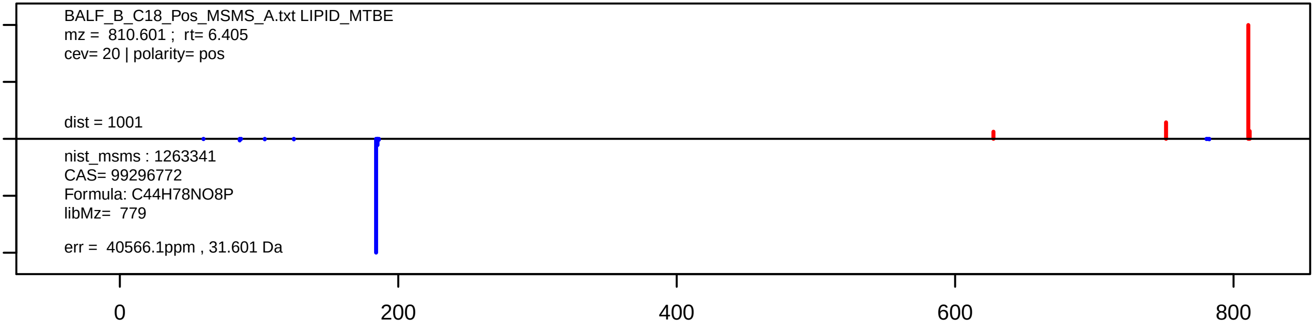

418 . PC 36:2; [M+Na]+; GPCho(18:1(11E)/18:1(11E))
Score=461 Dot=966 prob=1.2

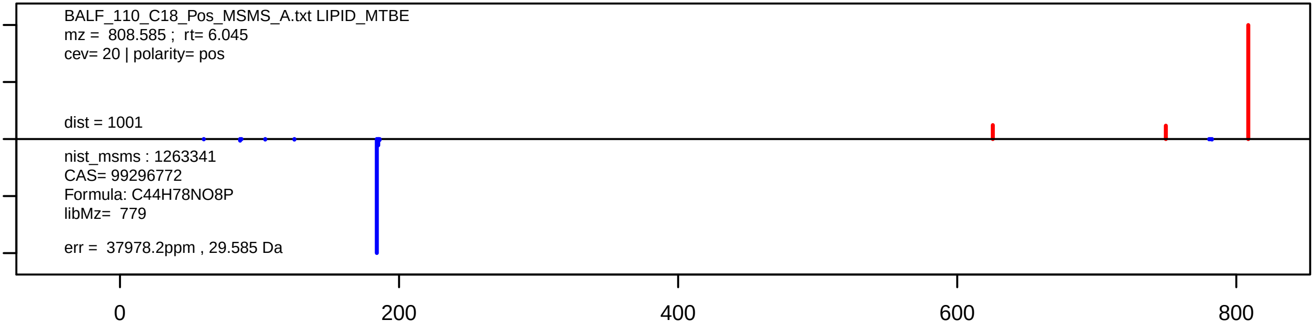

419 . PC 36:3; [M+Na]+; GPCho(14:1(9Z)/22:2(13Z,16Z))
Score=474 Dot=951 prob=1

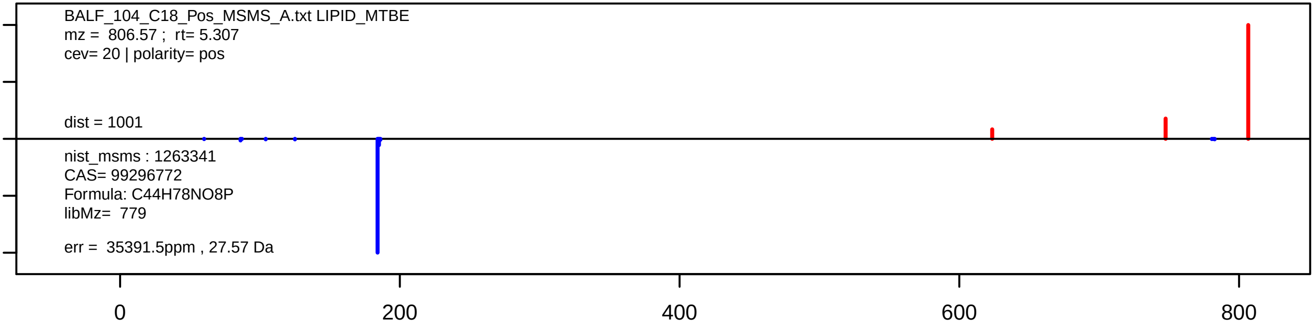

420 . PC 36:4; [M+Na]+; GPCho(18:2(2E,4E)/18:2(2E,4E))
Score=326 Dot=811 prob=2.2

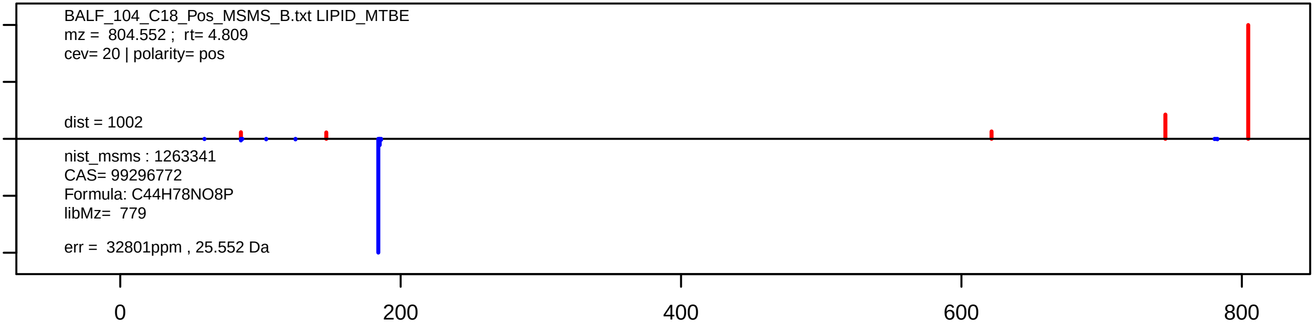

421 . PC 36:5; [M+Na]+; GPCho(14:0/22:5(4Z,7Z,10Z,13Z,16Z))
Score=427 Dot=946 prob=1.2

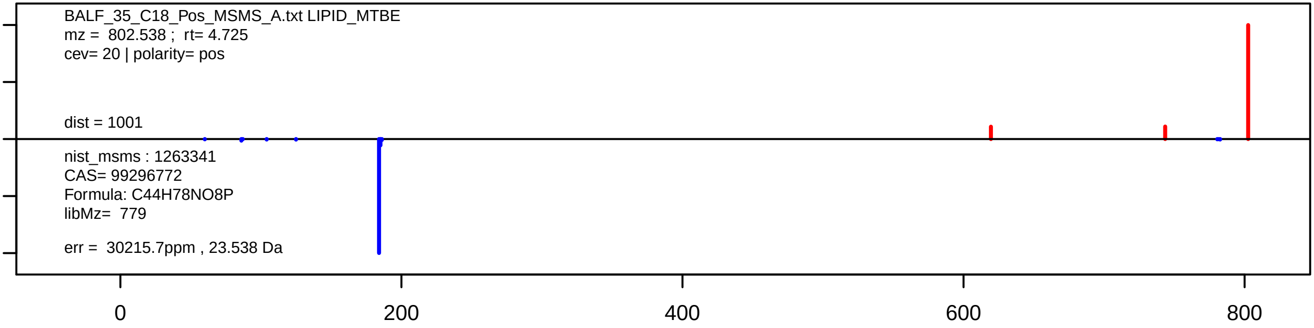

422 . PC 37:7; [M+Na]+; GPCho(15:1(9Z)/22:6(4Z,7Z,10Z,13Z,16Z,19Z))
Score=480 Dot=953 prob=24.9

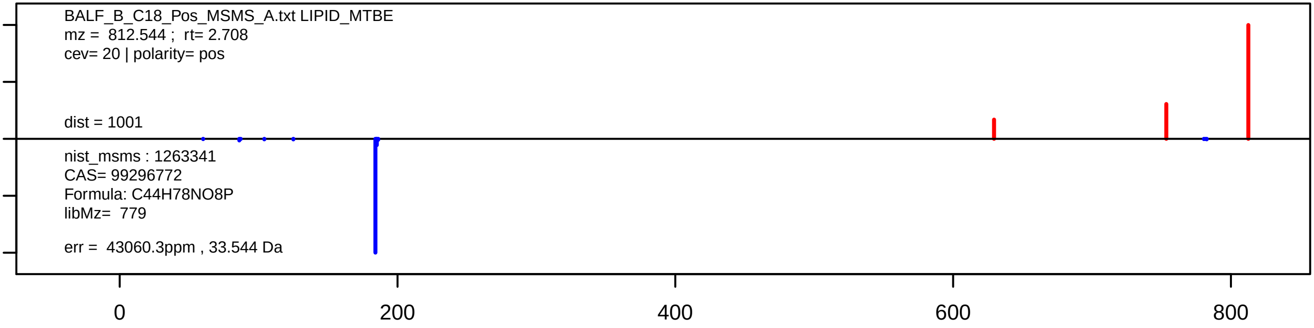

423 . PC 38:2; [M+Na]+; GPCho(12:0/26:2(5E,9Z))
Score=278 Dot=805 prob=1

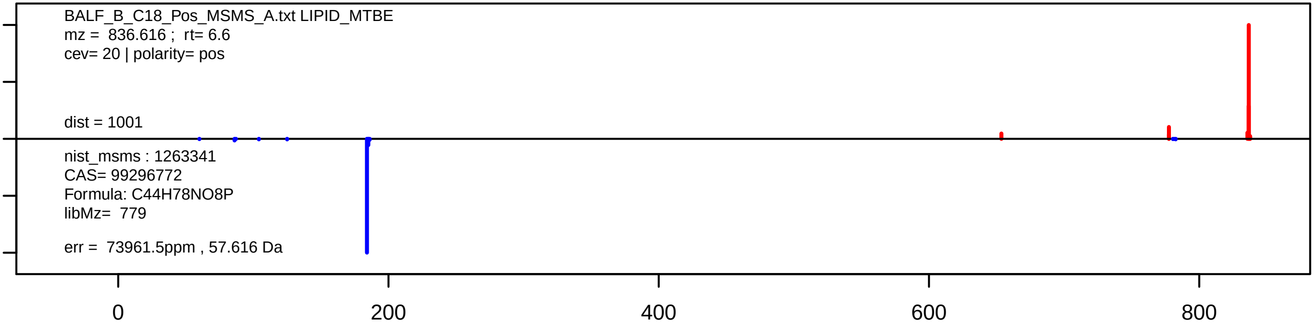

424 .   PC 38:5; [M+Na]+; GPCho(14:1(9Z)/24:4(5Z,8Z,11Z,14Z))
Score=440 Dot=947 prob=1

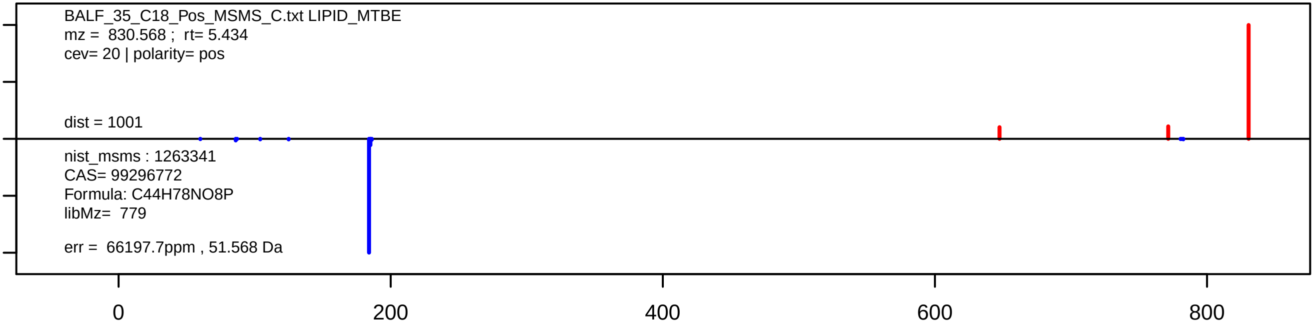

425 .   PC 38:6; [M+Na]+; GPCho(16:0/22:6(4Z,7Z,10Z,13Z,16Z,19Z))
Score=474 Dot=951 prob=1.2

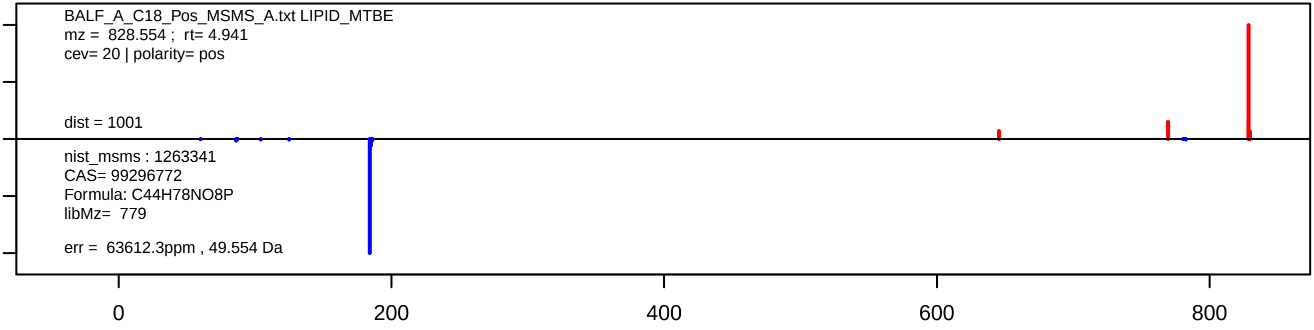

426 .   PE 19:1; [M+H]+; GPEtn(2:0/17:1(9Z))
Score=262 Dot=946 prob=9.9

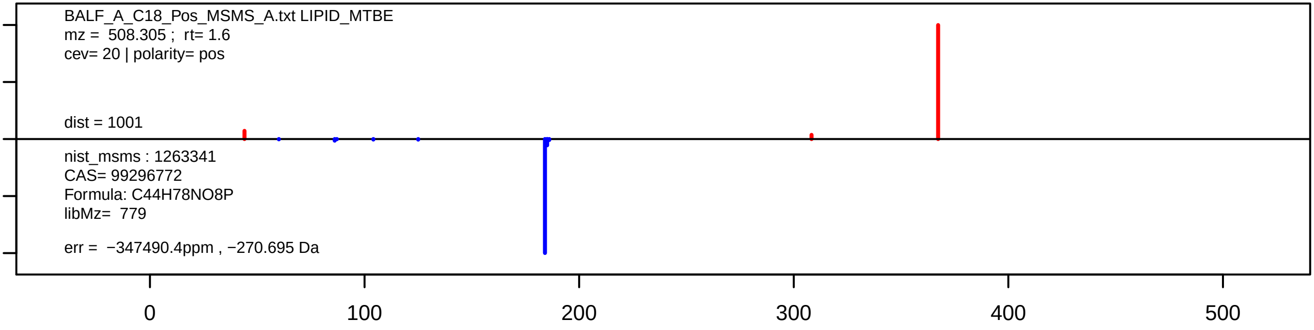

427 . PE 19:2; [M+H]+; GPEtn(2:0/17:2(9Z,12Z))
Score=351 Dot=996 prob=49.5

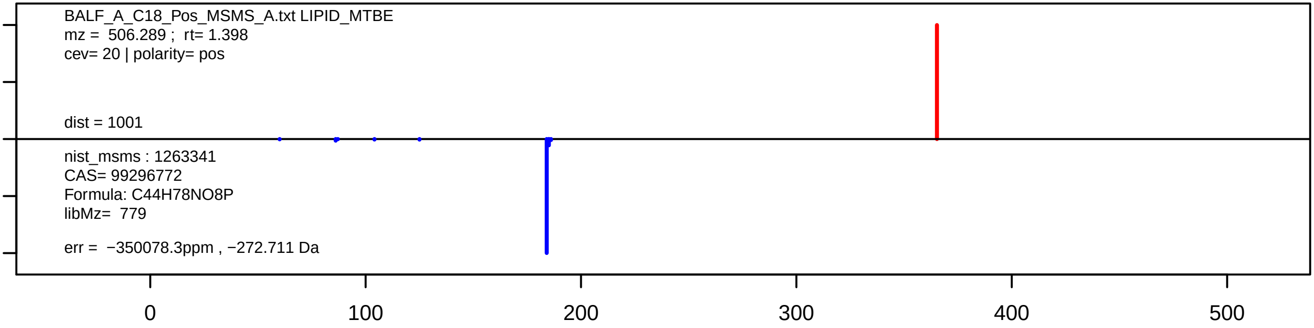

428 . PE 23:4; [M+H]+; GPEtn(3:0/20:4(5E,8E,11E,14E))
Score=351 Dot=996 prob=9.9

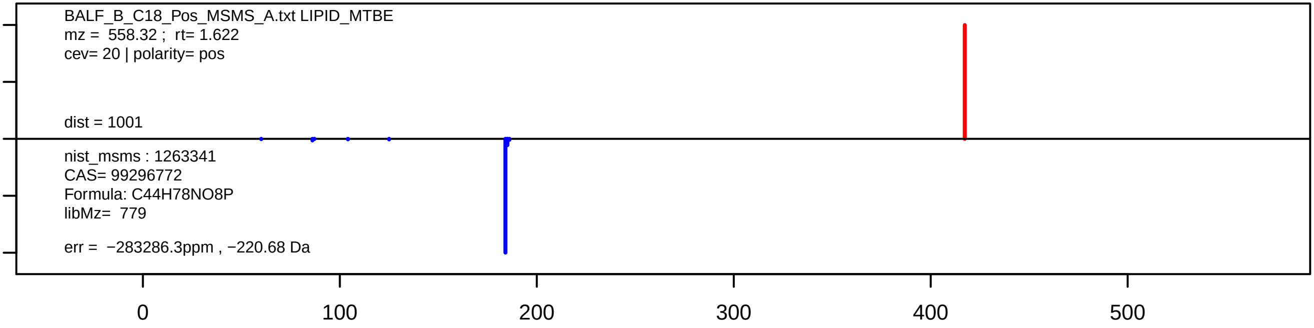

429 . PE 30:0; [M+H]+; GPEtn(15:0/15:0)
Score=372 Dot=998 prob=8.8

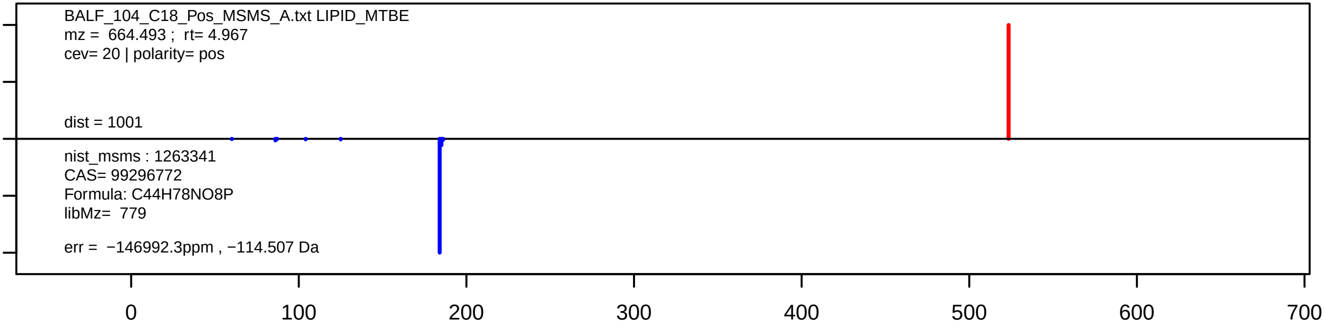

430 . PE 30:0; [M+H]+; GPEtn(4:0/26:0)
Score=261 Dot=940 prob=35.6

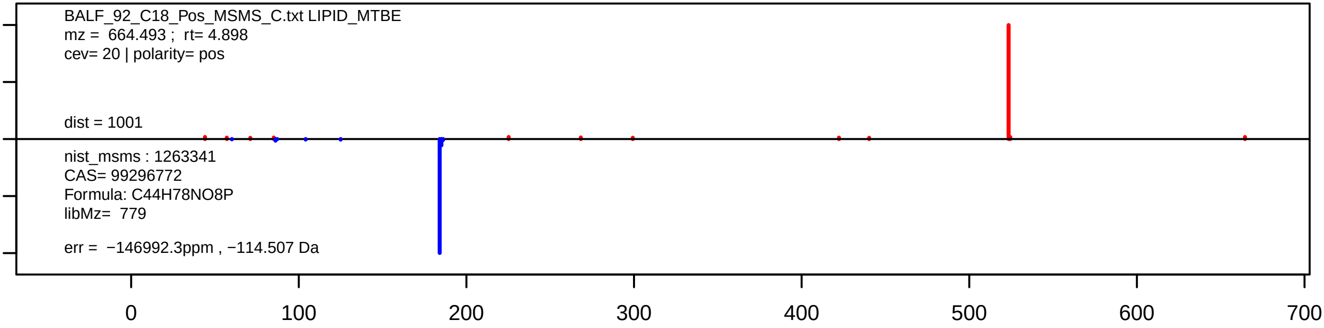

431 . PE 30:0; [M+H]+; GPEtn(5:0/25:0)
Score=309 Dot=961 prob=23.7

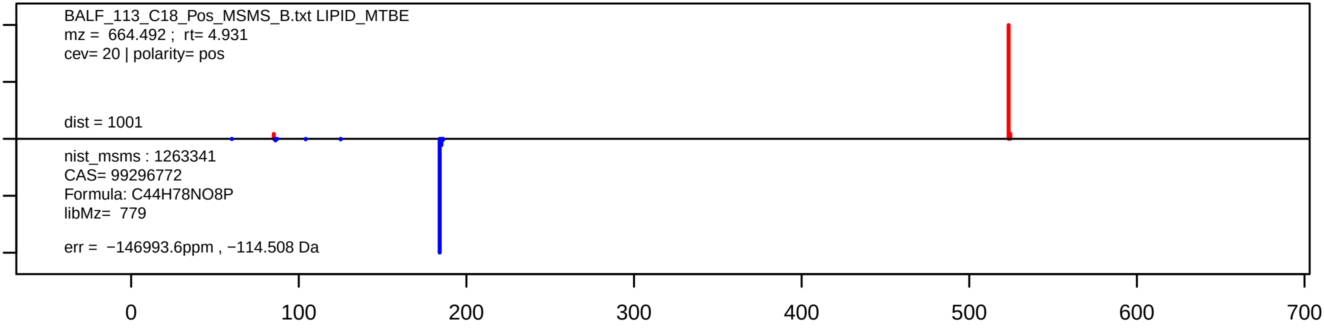

432 . PE 30:0; [M+H]+; GPEtn(9:0/21:0)
Score=347 Dot=984 prob=12.6

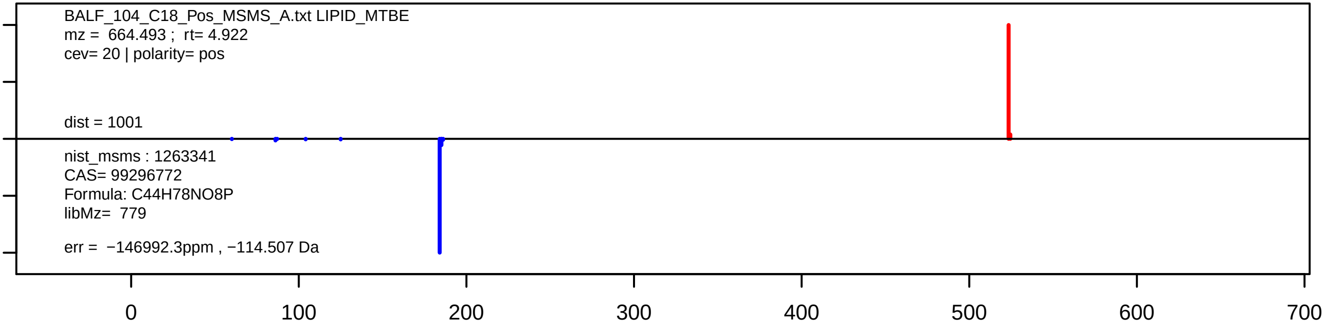

433 . PE 30:0; [M+Na]+; GPEtn(15:0/15:0)
Score=257 Dot=844 prob=8.6

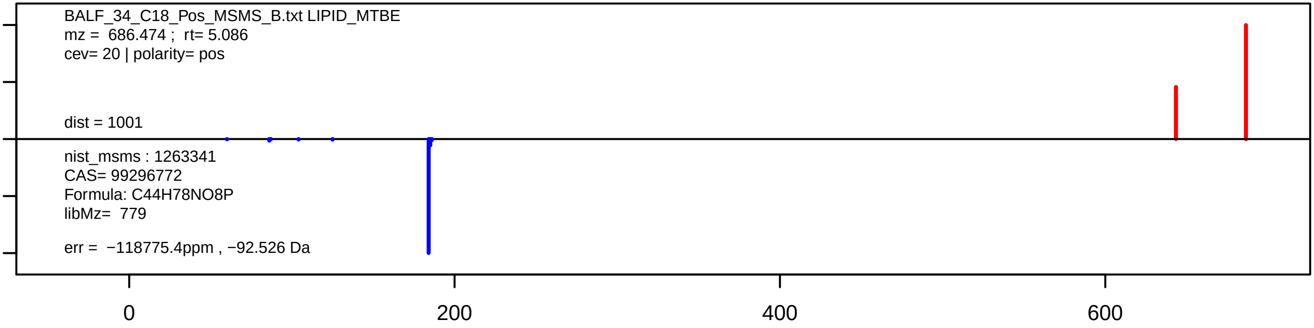

434 . PE 34:0; [M+H]+; GPEtn(17:0/17:0)
Score=372 Dot=998 prob=11.6

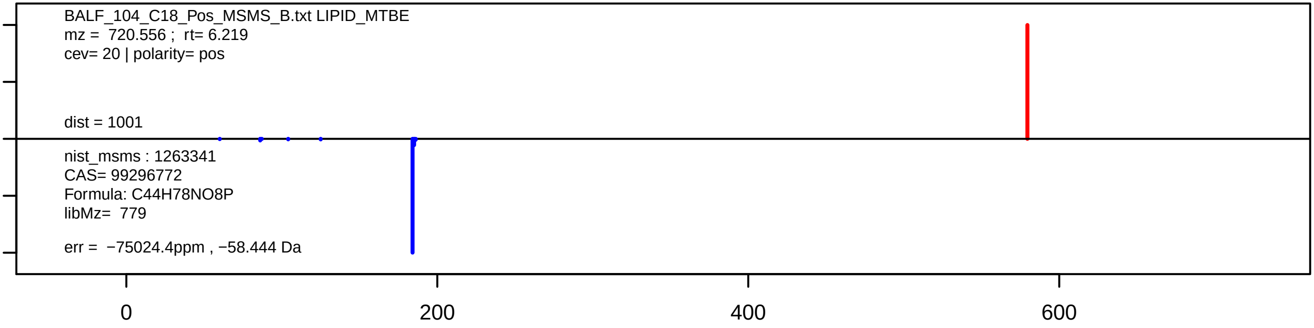

435 . PE 34:0; [M+H]+; GPEtn(9:0/25:0)
Score=326 Dot=980 prob=18.6

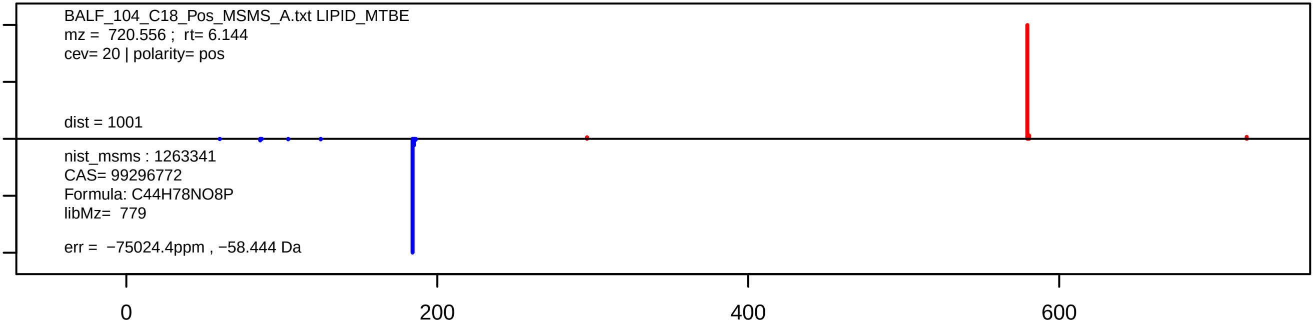

436 . PE 34:2; [M+H]+; GPEtn(17:1(9Z)/17:1(9Z))
Score=372 Dot=998 prob=3

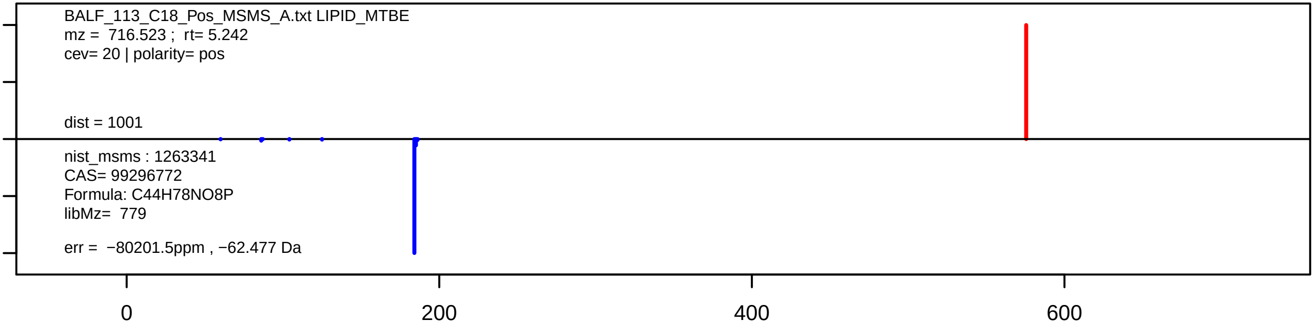

437 . PE 35:1; [M+H]+; GPEtn(9:0/26:1(5Z))
Score=265 Dot=951 prob=2.4

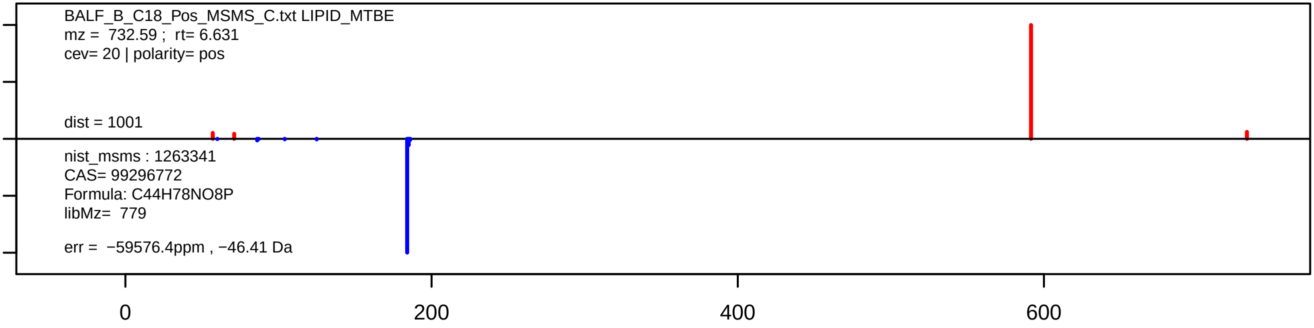

438 . PE 36:3; [M+H]+; GPEtn(14:1(9Z)/22:2(13Z,16Z))
Score=264 Dot=899 prob=1

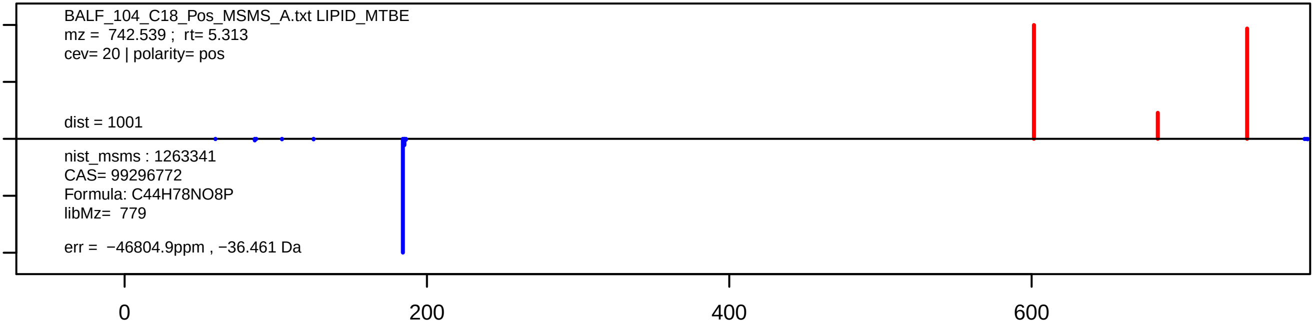

439 . PE 37:4; [M+H]+; GPEtn(13:0/24:4(5Z,8Z,11Z,14Z))
Score=256 Dot=876 prob=4.5

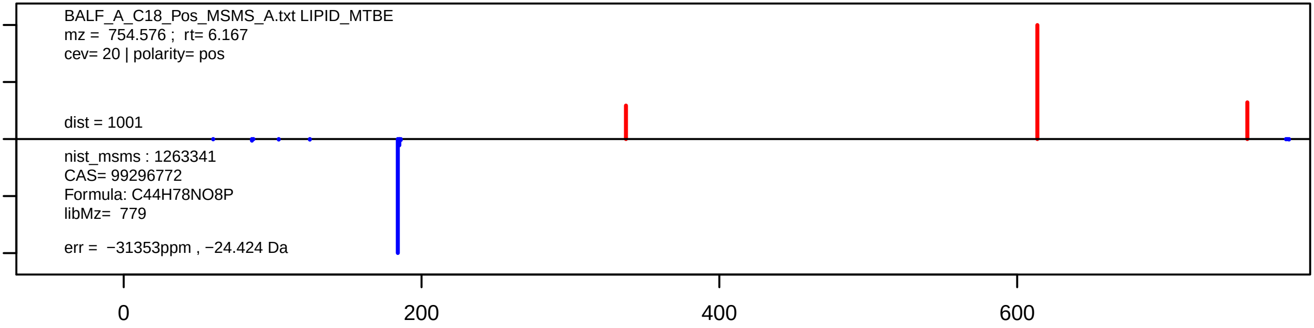

440 . PE 37:7; [M+Na]+; GPEtn(15:1(9Z)/22:6(4Z,7Z,10Z,13Z,16Z,19Z))
Score=238 Dot=843 prob=23.8

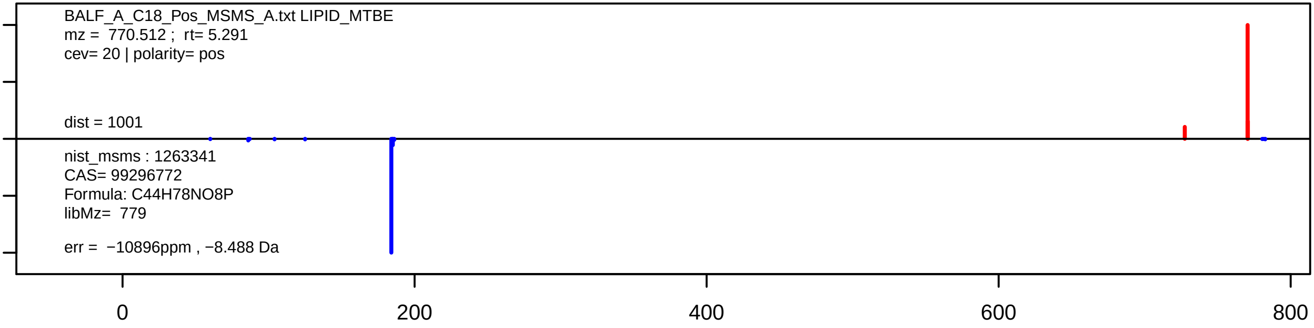

441 . PE 38:4; [M+H]+; GPEtn(14:0/24:4(5Z,8Z,11Z,14Z))
Score=351 Dot=996 prob=1.1

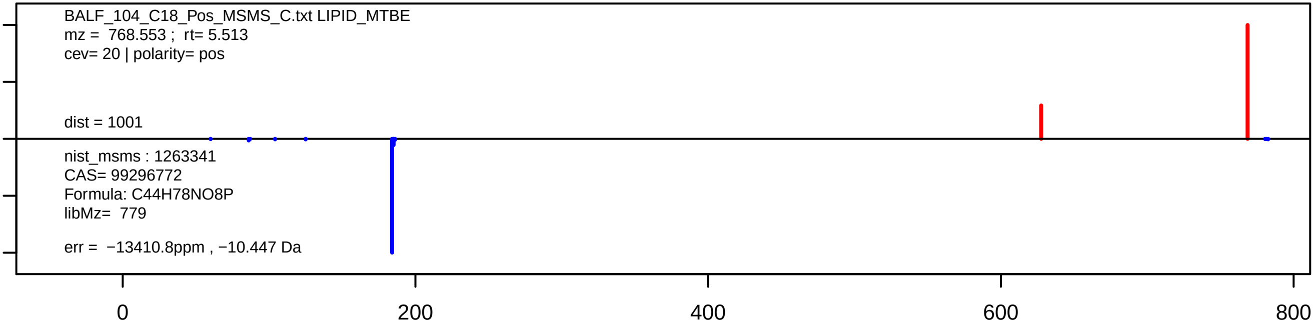

442 . PE 39:3; [M+H]+; GPEtn(17:1(9Z)/22:2(13Z,16Z))
Score=255 Dot=874 prob=8.2

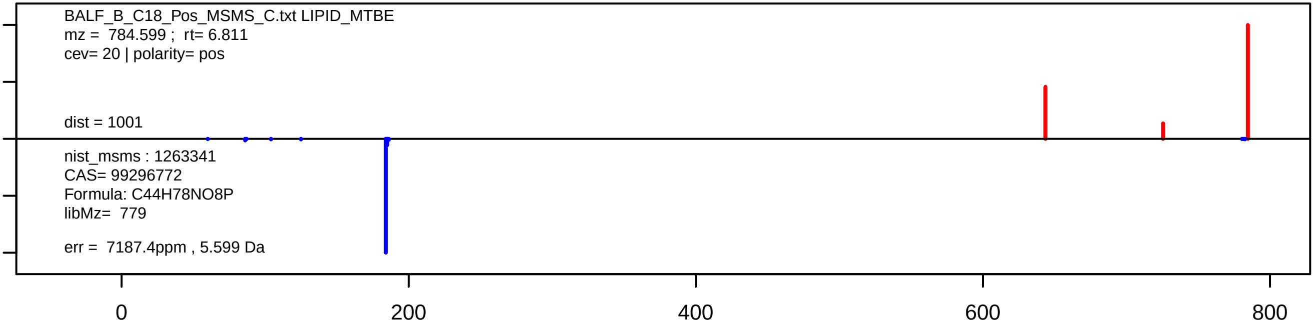

443 . PE 39:4; [M+H]+; GPEtn(15:0/24:4(5Z,8Z,11Z,14Z))
Score=351 Dot=996 prob=6.1

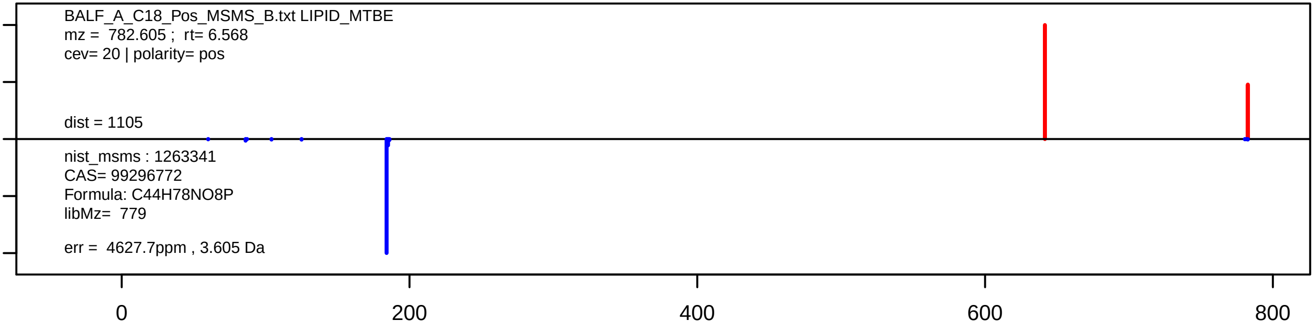

444 . PE 41:4; [M+H]+; GPEtn(17:0/24:4(5Z,8Z,11Z,14Z))
Score=351 Dot=996 prob=6.9

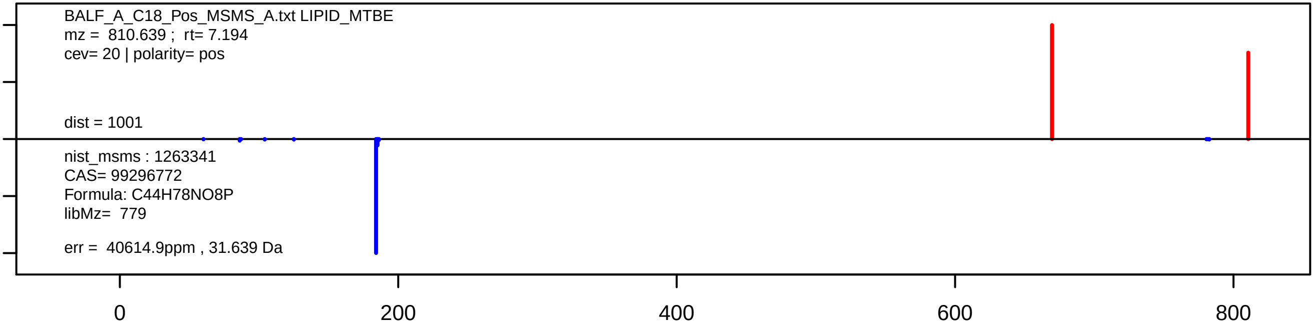

445 .   Phthalic anhydride
Score=616 Dot=959 prob=81.3

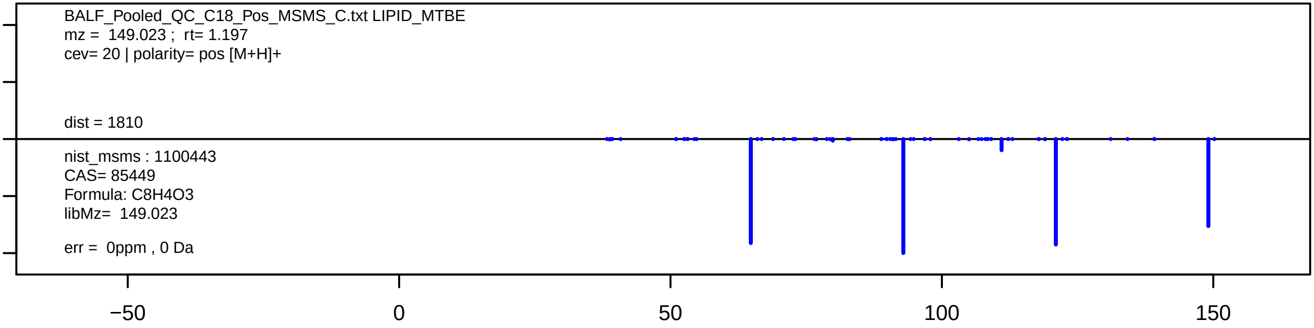

446 .   plasmenyl−PC 31:0; [M+Na]+; PC(P−16:0/15:0)
Score=276 Dot=957 prob=23.4

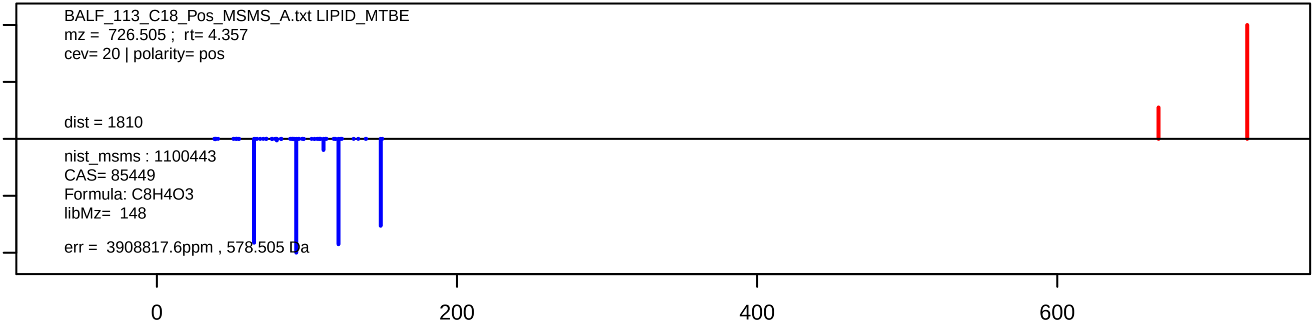

447 .   plasmenyl−PC 31:1; [M+Na]+; PC(P−16:0/15:1(9Z))
Score=276 Dot=957 prob=64.8

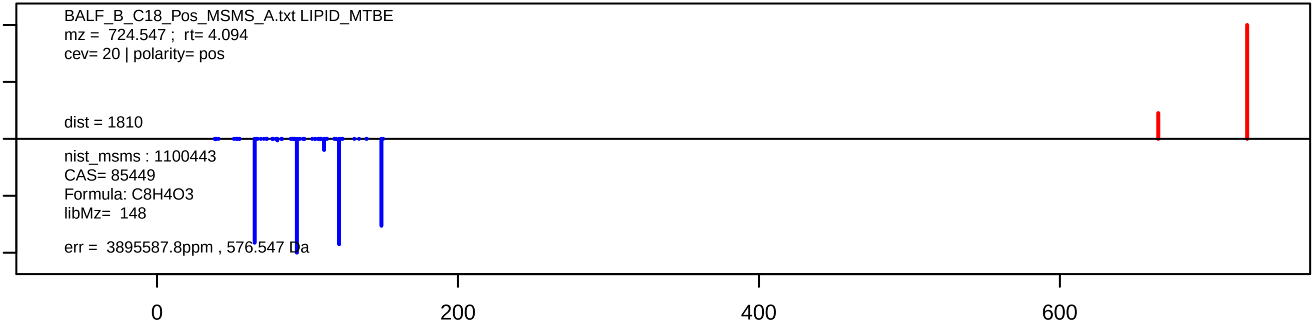

448 . plasmenyl−PC 32:0; [M+Na]+; PC(P−16:0/16:0)
Score=276 Dot=957 prob=26.6

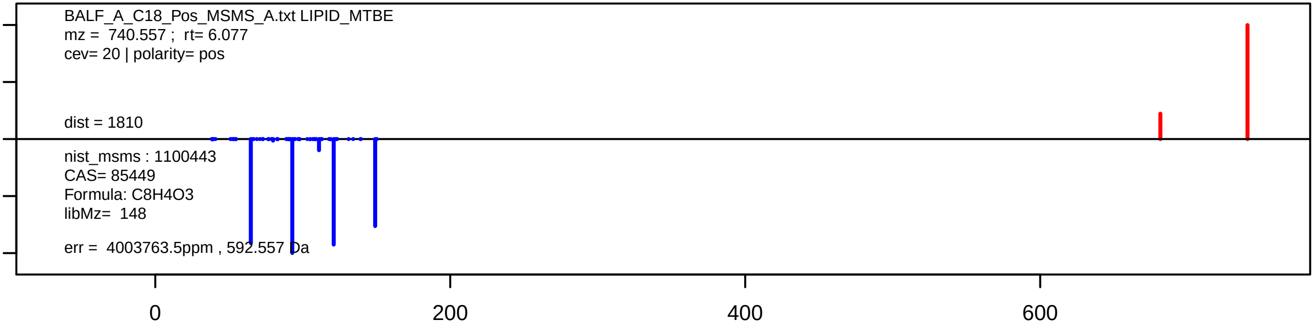

449 . plasmenyl−PC 33:0; [M+Na]+; PC(P−16:0/17:0)
Score=179 Dot=831 prob=13.3

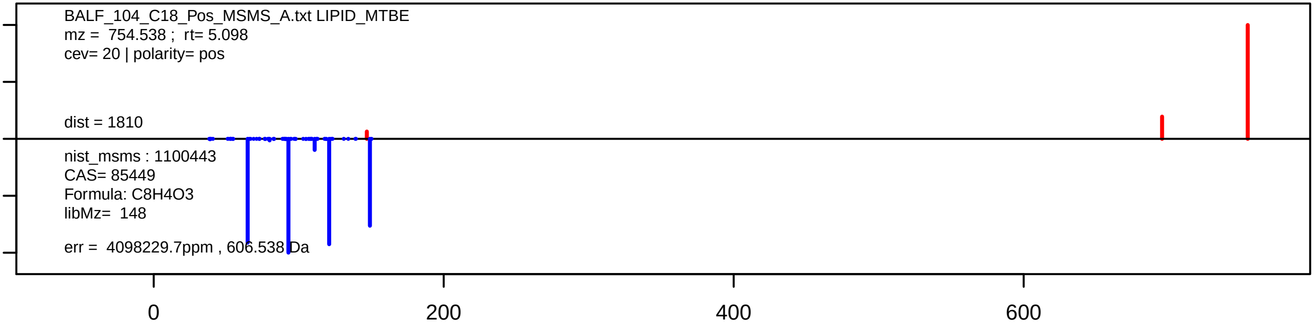

450 . plasmenyl−PC 33:1; [M+Na]+; PC(P−16:0/17:1(9Z))
Score=276 Dot=957 prob=33.6

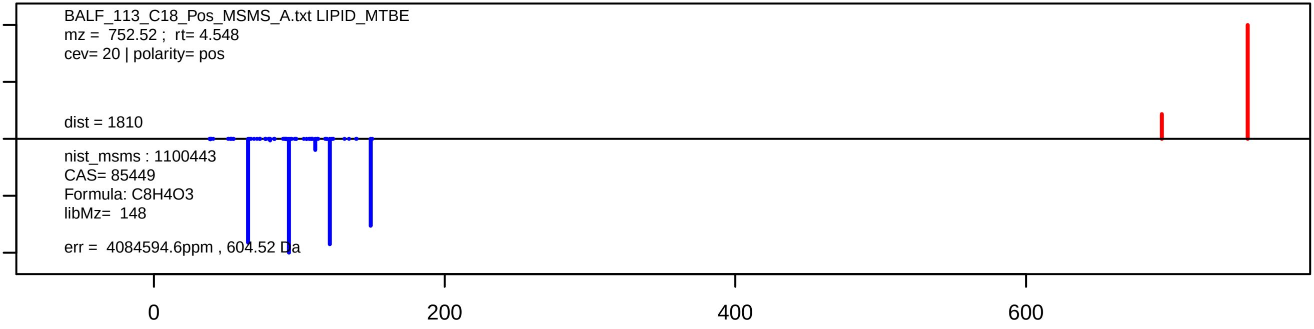

451 . plasmenyl−PC 34:0; [M+Na]+; PC(P−16:0/18:0)
Score=276 Dot=957 prob=30

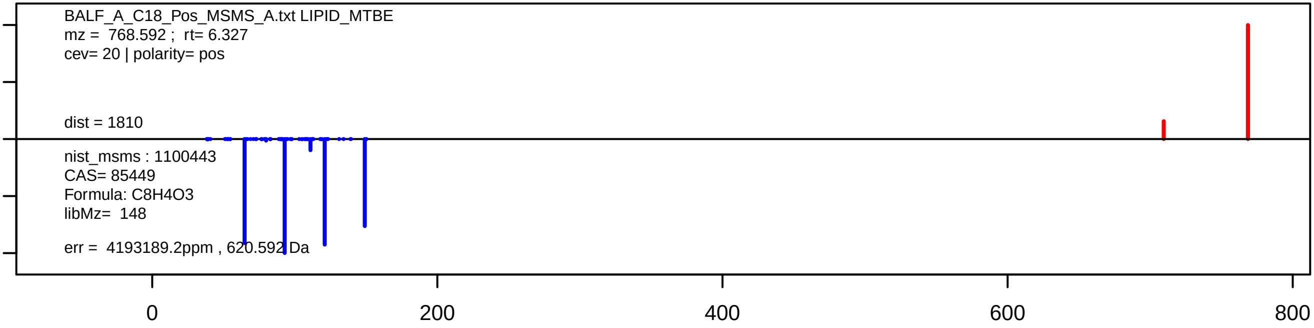

452 . plasmenyl−PC 34:1; [M+Na]+; PC(P−16:0/18:1(11E))
Score=276 Dot=957 prob=7.4

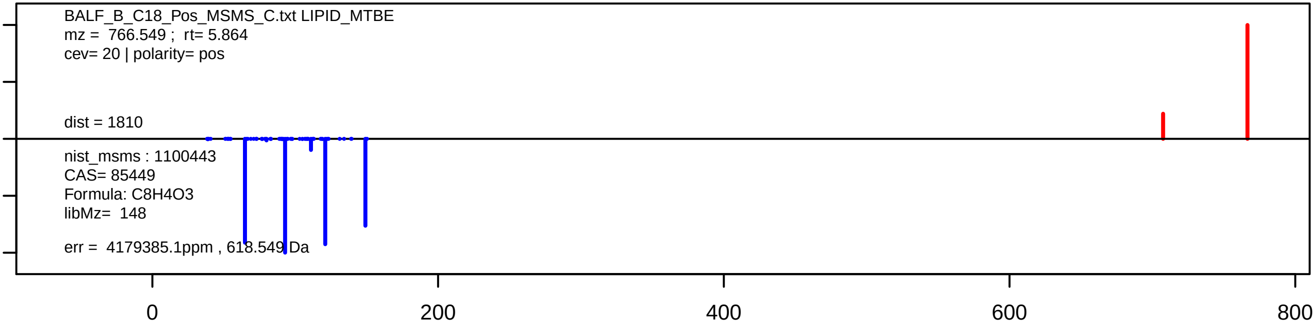

453 . plasmenyl−PC 35:0; [M+Na]+; PC(P−16:0/19:0)
Score=276 Dot=957 prob=33

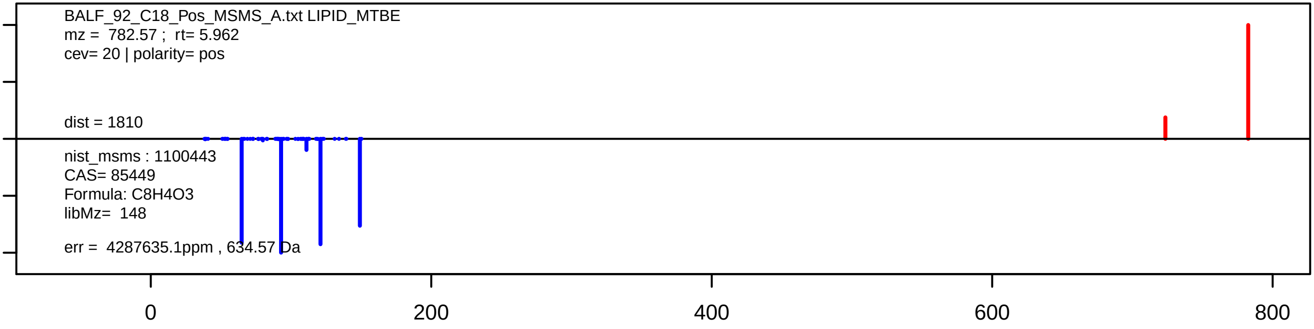

454 . plasmenyl−PC 35:2; [M+Na]+; PC(P−18:0/17:2(9Z,12Z))
Score=276 Dot=957 prob=45.7

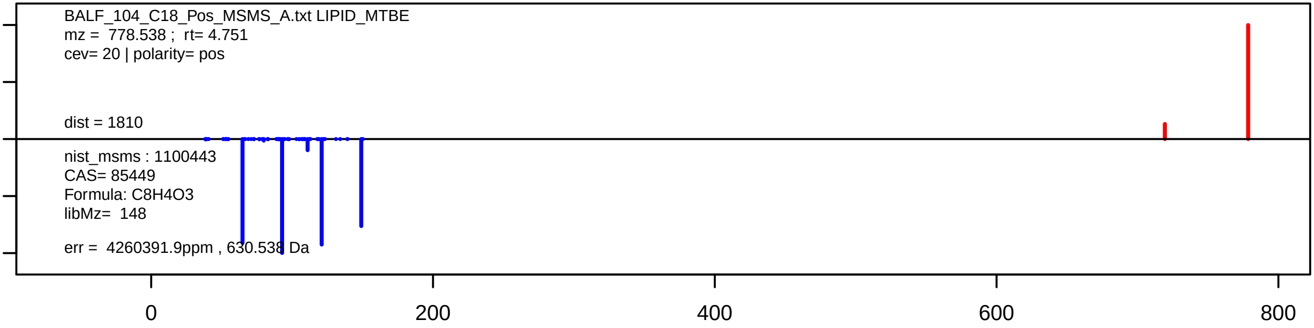

455 . plasmenyl−PC 36:0; [M+Na]+; PC(P−16:0/20:0)
Score=276 Dot=957 prob=23.3

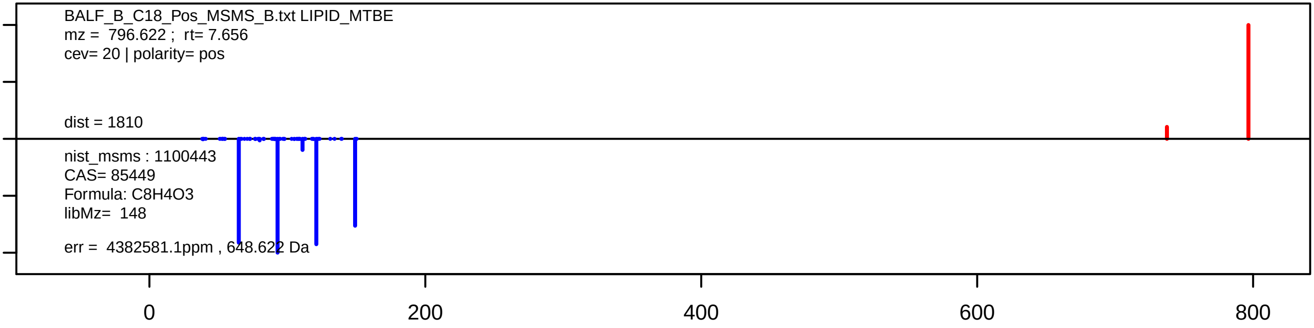

456 . plasmenyl−PC 36:1; [M+Na]+; PC(P−16:0/20:1(11E))
Score=276 Dot=957 prob=6.7

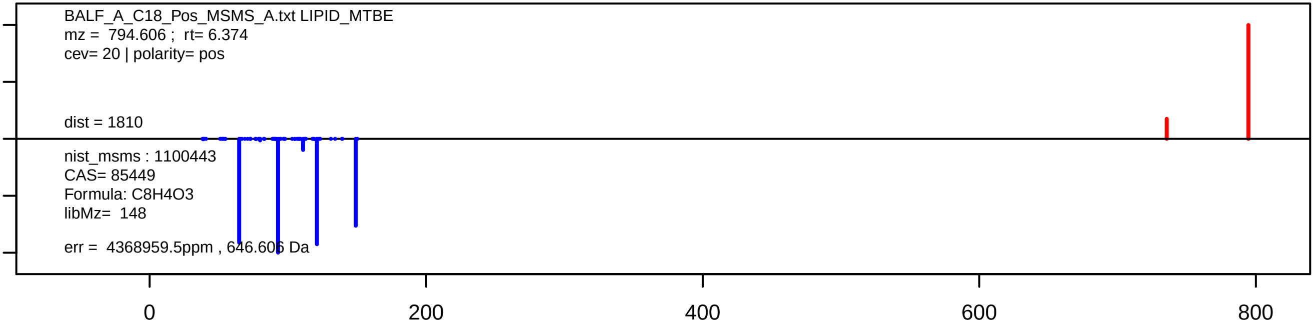

457 . plasmenyl−PC 36:2; [M+Na]+; PC(P−16:0/20:2(11Z,14Z))
Score=276 Dot=957 prob=12.4

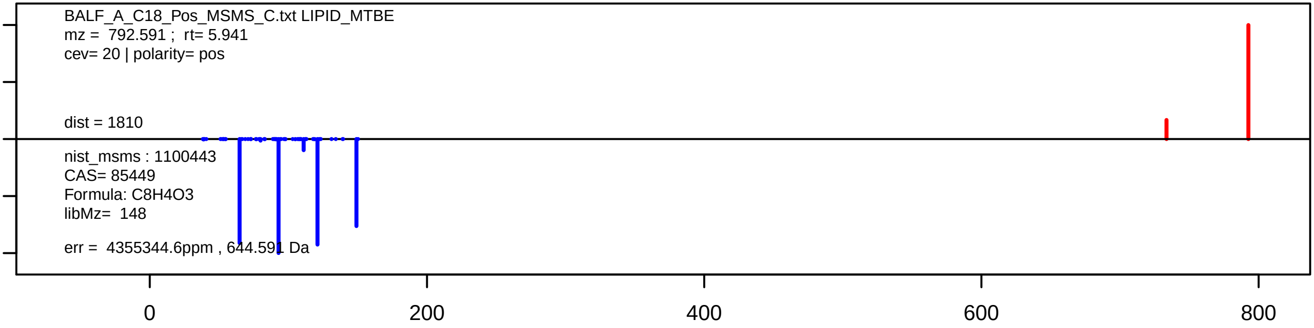

458 . plasmenyl−PC 36:3; [M+Na]+; PC(P−16:0/20:3(5Z,8Z,11Z))
Score=276 Dot=957 prob=20.9

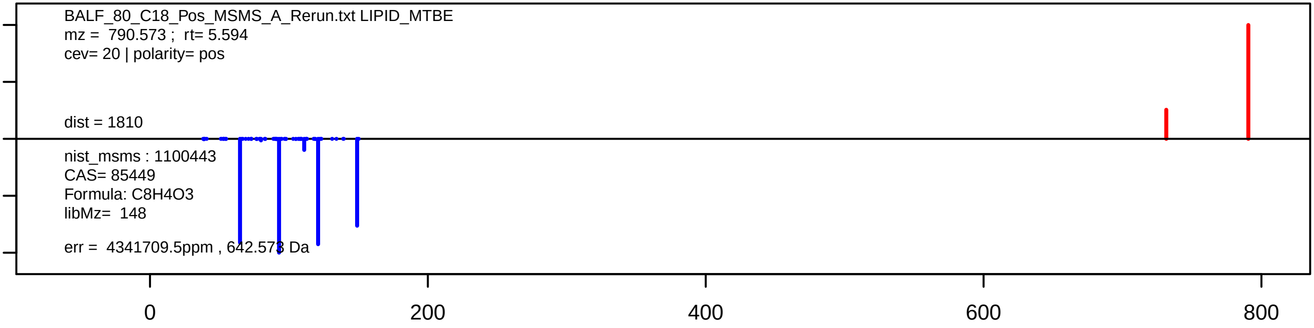

459 . plasmenyl−PC 36:4; [M+Na]+; PC(P−16:0/20:4(5E,8E,11E,14E))
Score=276 Dot=957 prob=17.9

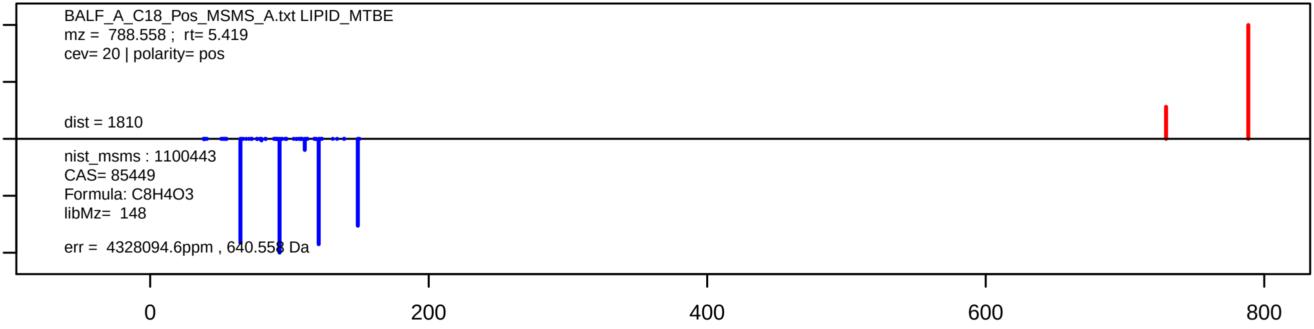

460 . plasmenyl−PC 37:0; [M+Na]+; PC(P−16:0/21:0)
Score=276 Dot=957 prob=22.9

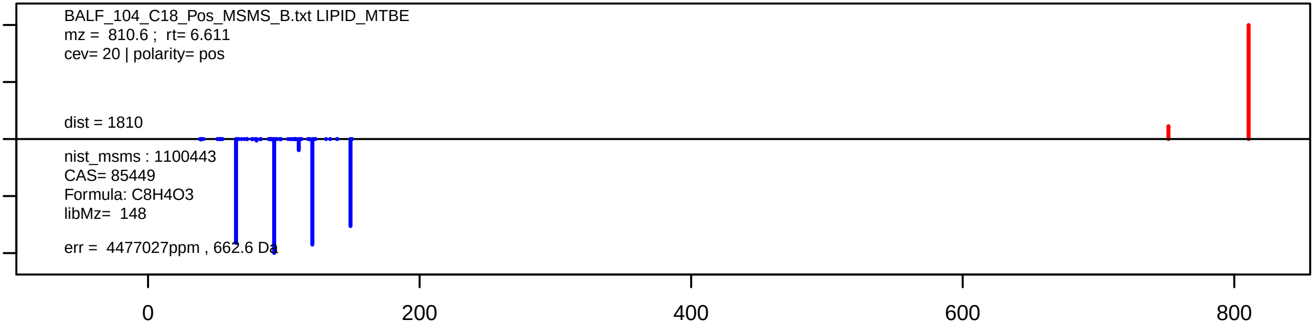

461 . plasmenyl−PC 37:1; [M+Na]+; PC(P−20:0/17:1(9Z))
Score=276 Dot=957 prob=14.3

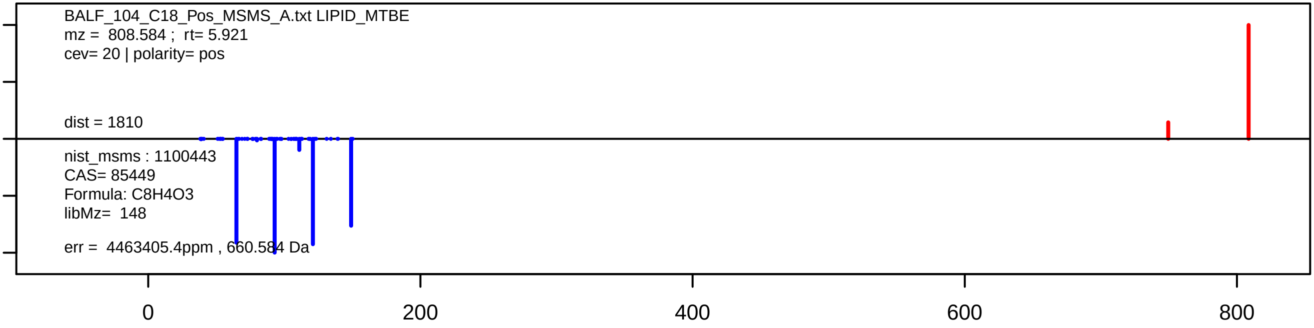

462 . plasmenyl−PC 38:0; [M+Na]+; PC(P−16:0/22:0)
Score=276 Dot=957 prob=23.4

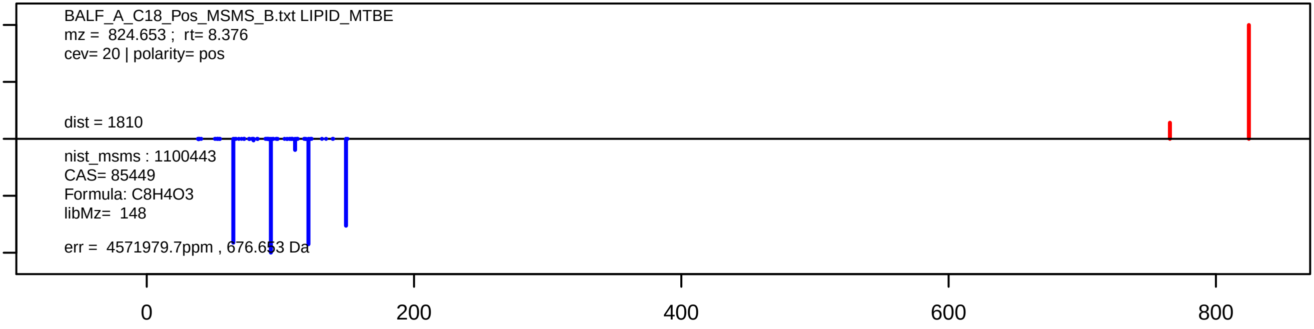

463 . plasmenyl−PC 38:1; [M+Na]+; PC(P−16:0/22:1(13Z))
Score=276 Dot=957 prob=6.6

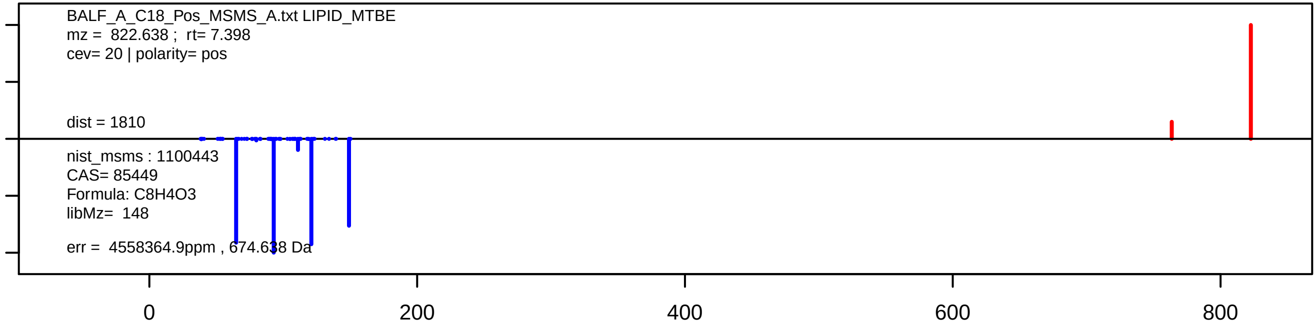

464 . plasmenyl−PC 38:2; [M+Na]+; PC(P−16:0/22:2(13Z,16Z))
Score=276 Dot=957 prob=10.3

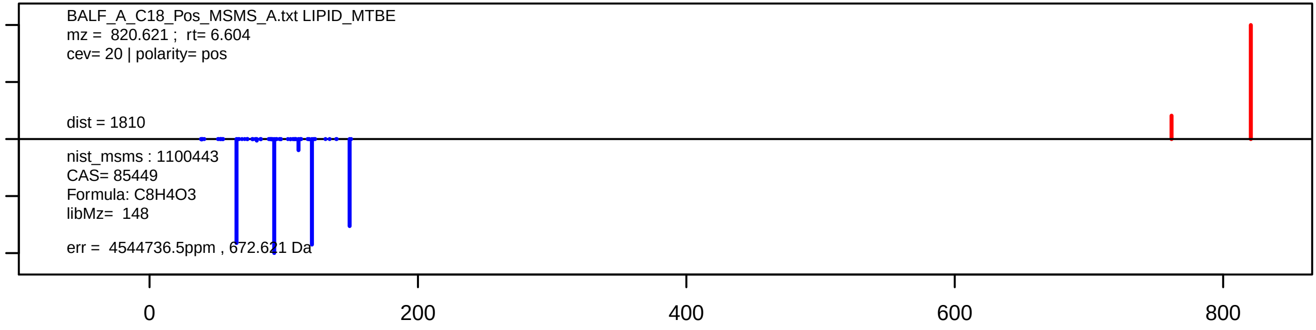

465 . plasmenyl−PC 40:0; [M+Na]+; PC(P−16:0/24:0)
Score=276 Dot=957 prob=23.4

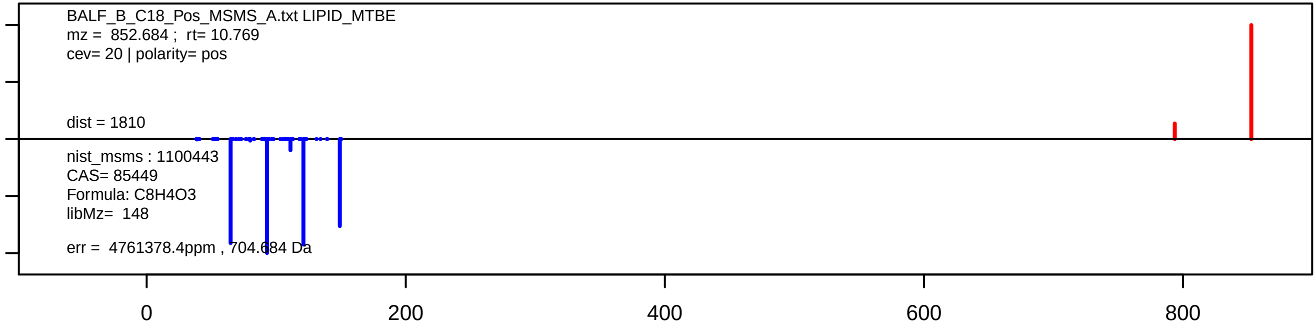

466 . plasmenyl−PC 40:1; [M+Na]+; PC(P−16:0/24:1(15Z))
Score=276 Dot=957 prob=14.5

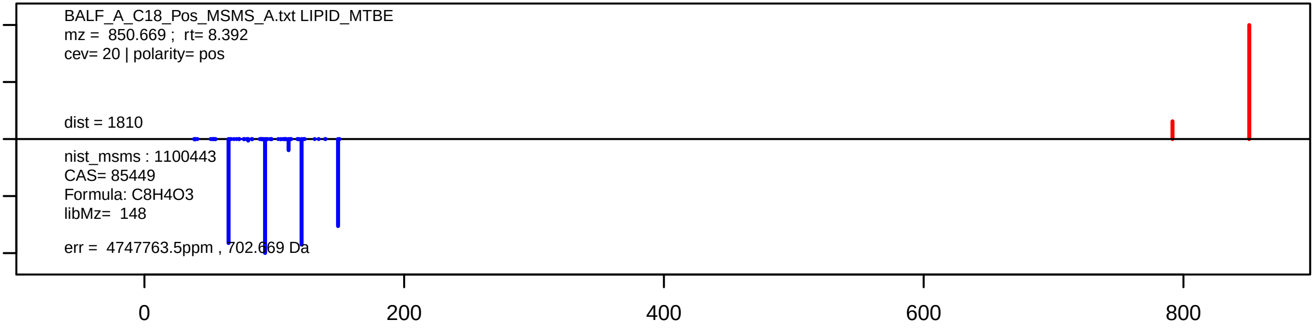

467 . plasmenyl−PC 40:2; [M+Na]+; PC(P−18:0/22:2(13Z,16Z))
Score=276 Dot=957 prob=29.7

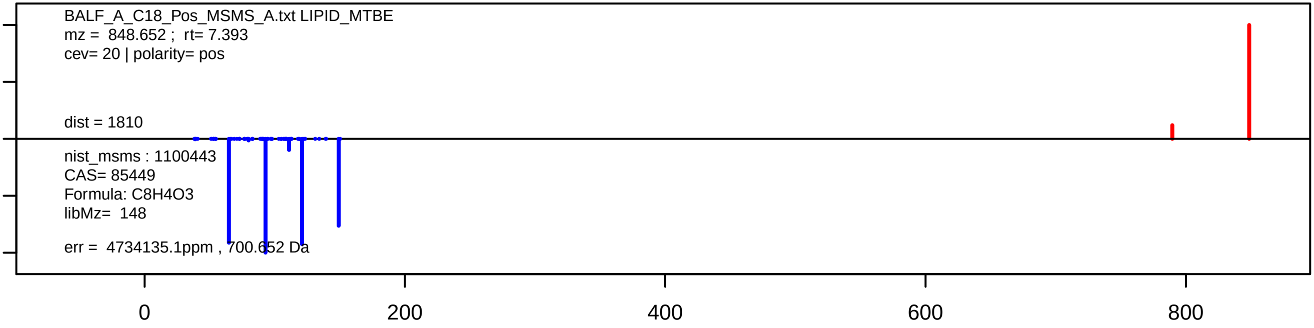

468 . plasmenyl−PC 40:3; [M+Na]+; PC(P−20:0/20:3(5Z,8Z,11Z))
Score=276 Dot=957 prob=40.1

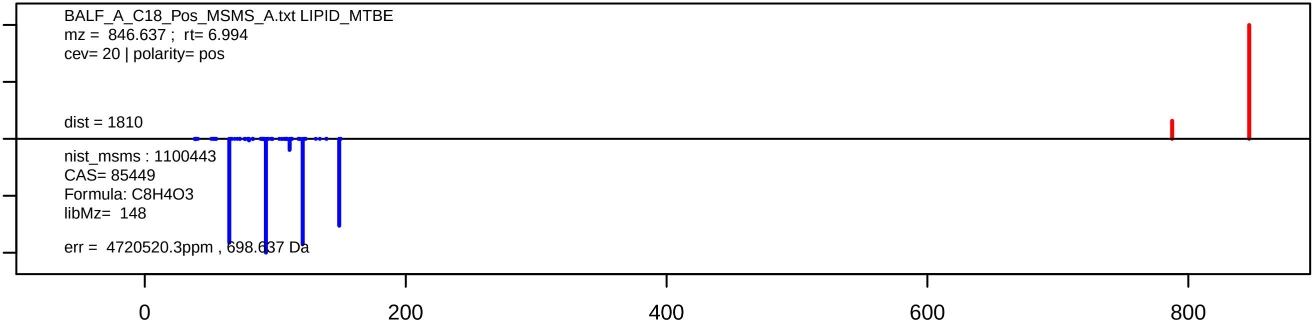

469 . plasmenyl−PC 42:1; [M+Na]+; PC(P−16:0/26:1(5Z))
Score=276 Dot=957 prob=25.7

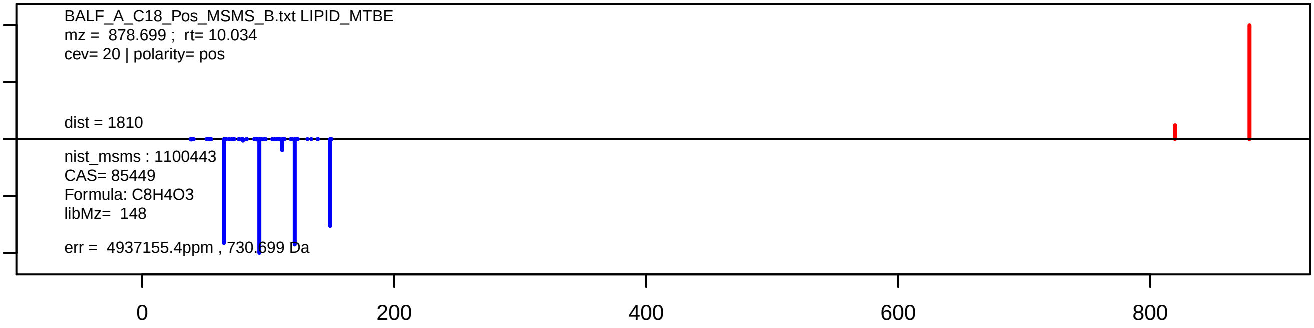

470 . plasmenyl−PC 42:2; [M+Na]+; PC(P−16:0/26:2(5E,9Z))
Score=276 Dot=957 prob=22.4

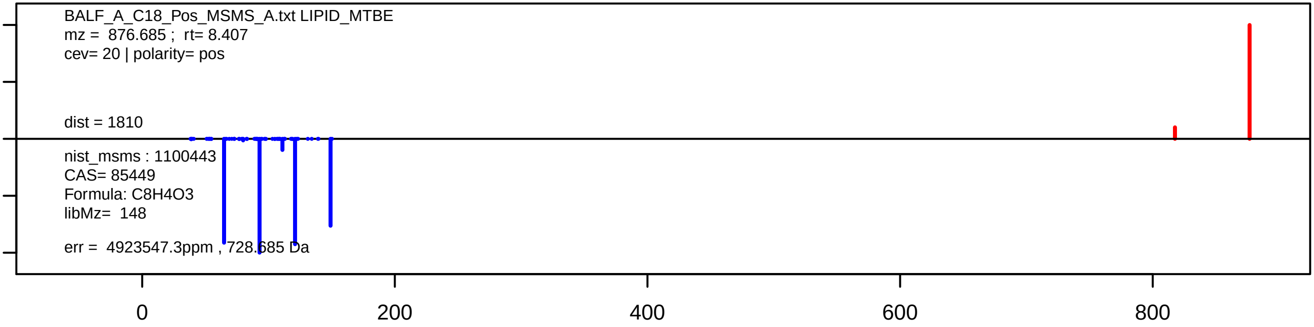

471 . plasmenyl−PC 42:4; [M+Na]+; PC(P−18:0/24:4(5Z,8Z,11Z,14Z))
Score=276 Dot=957 prob=43

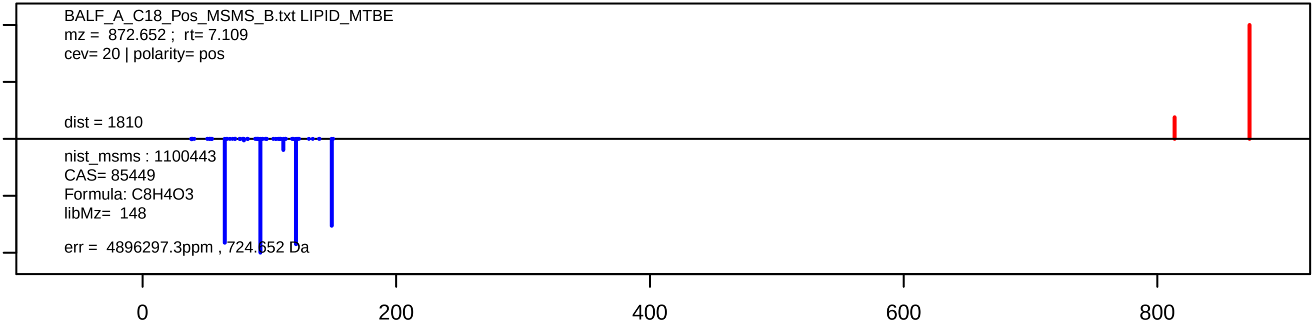

472 . plasmenyl−PC 44:4; [M+Na]+; PC(P−20:0/24:4(5Z,8Z,11Z,14Z))
Score=276 Dot=957 prob=79.4

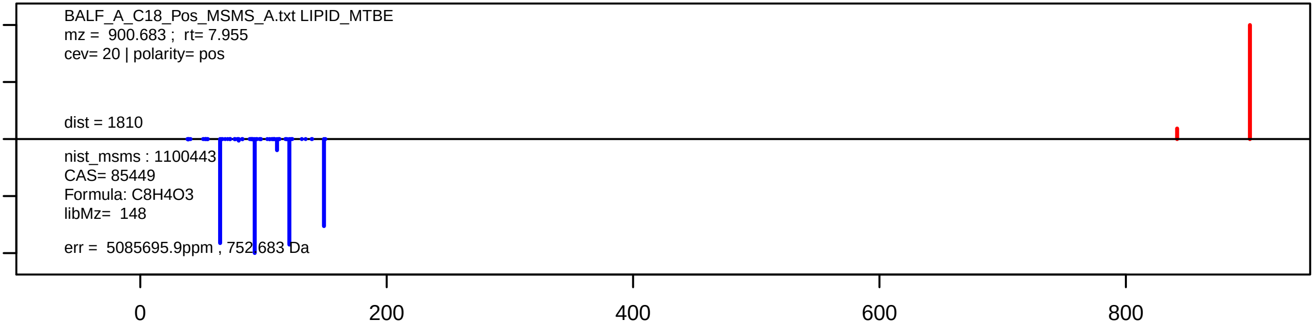

473 . plasmenyl−PE 34:1; [M+H]+; PE(P−16:0/18:1(11E))
Score=161 Dot=805 prob=10.9

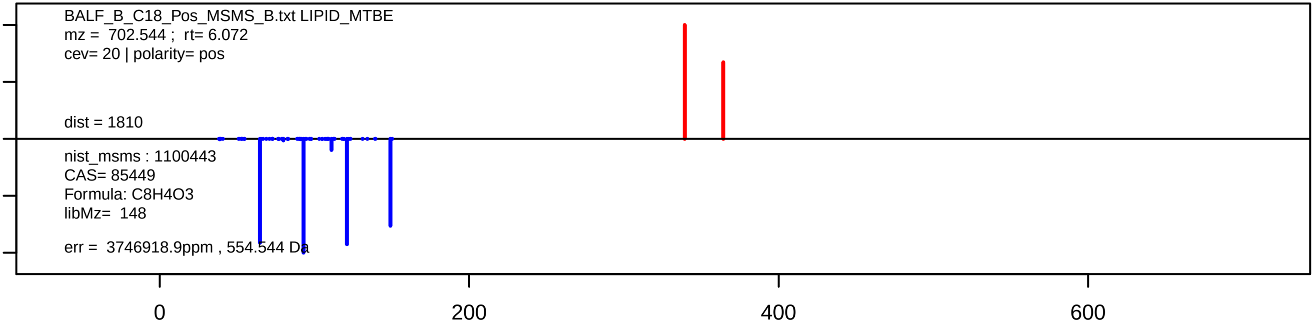

474 . plasmenyl−PE 34:2; [M+H]+; PE(P−16:0/18:2(2E,4E))
Score=197 Dot=817 prob=16.4

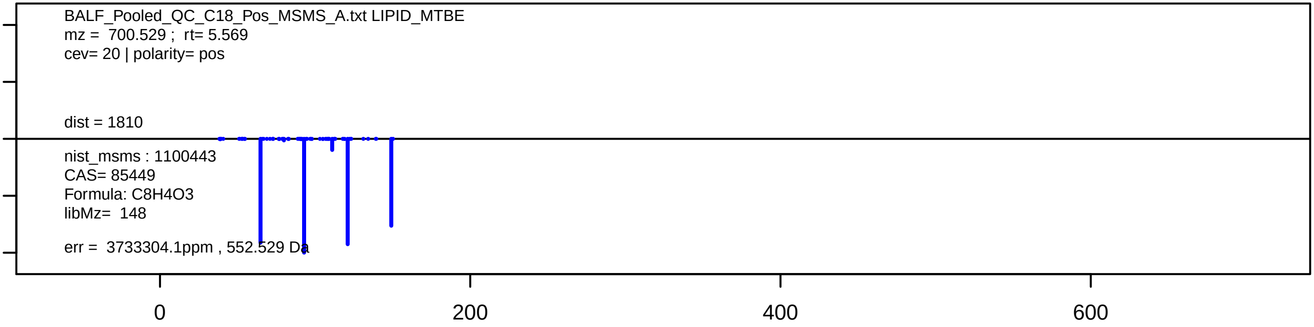

475 . plasmenyl−PE 36:1; [M+H]+; PE(P−18:0/18:1(11E))
Score=174 Dot=809 prob=10.7

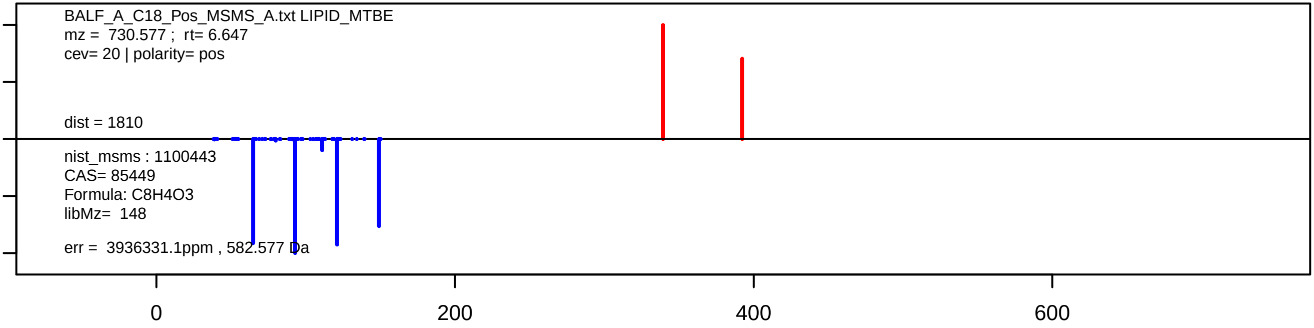

476 . plasmenyl−PE 36:1; [M+Na]+; PE(P−16:0/20:1(11E))
Score=187 Dot=894 prob=6.7

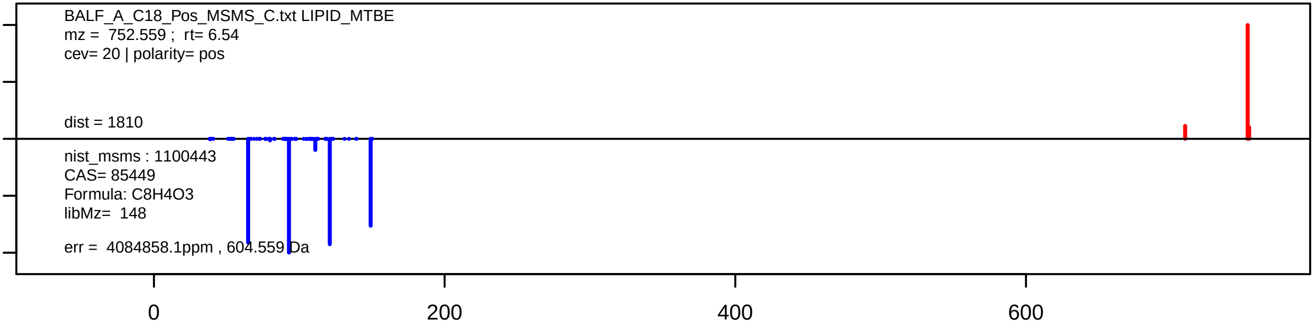

477 . plasmenyl−PE 36:4; [M+H]+; PE(P−16:0/20:4(5E,8E,11E,14E))
Score=196 Dot=807 prob=30.1

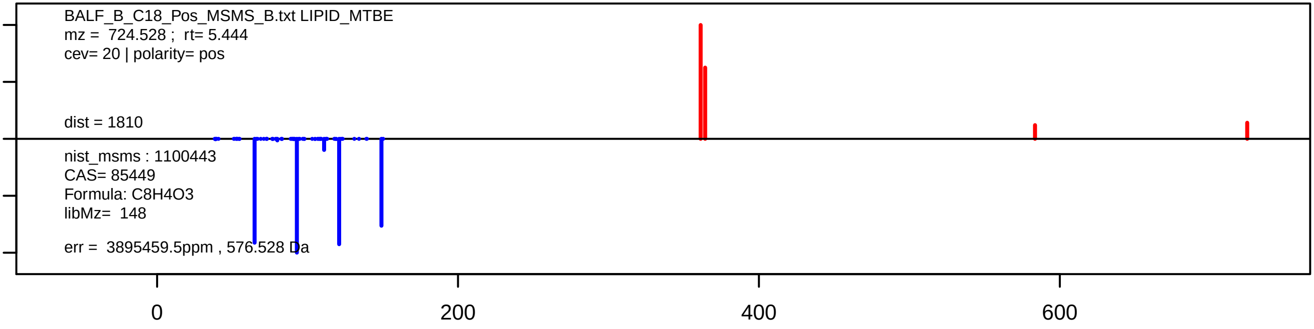

478 . plasmenyl−PE 38:2; [M+H]+; PE(P−20:0/18:2(2E,4E))
Score=273 Dot=837 prob=16.7

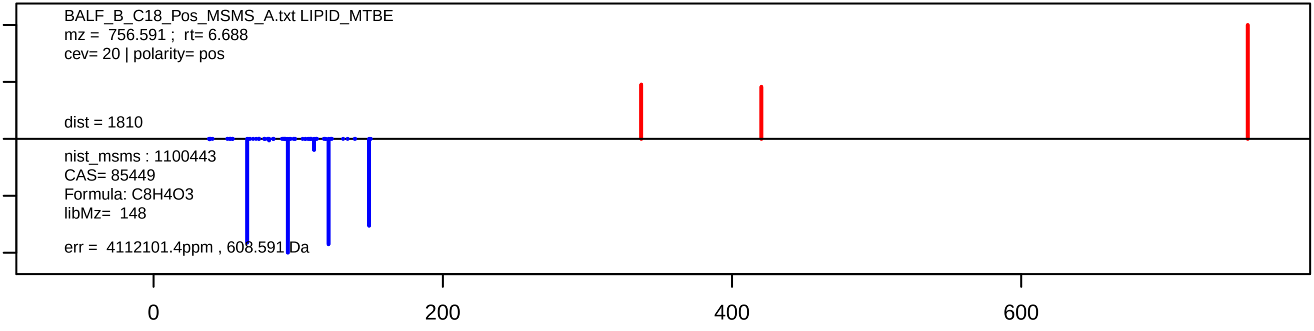

479 . plasmenyl−PE 38:2; [M+Na]+; PE(P−16:0/22:2(13Z,16Z))
Score=187 Dot=894 prob=11.1

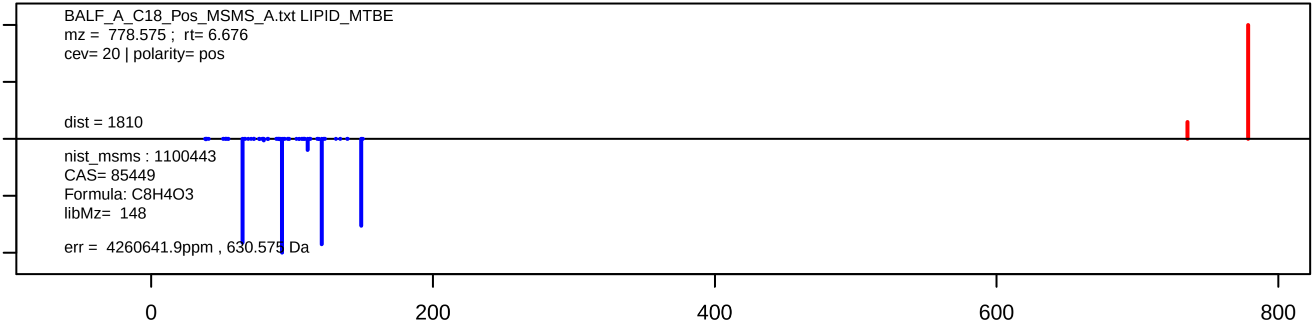

480 . plasmenyl−PE 40:5; [M+Na]+; PE(P−18:0/22:5(4Z,7Z,10Z,13Z,16Z))
Score=187 Dot=894 prob=33.3

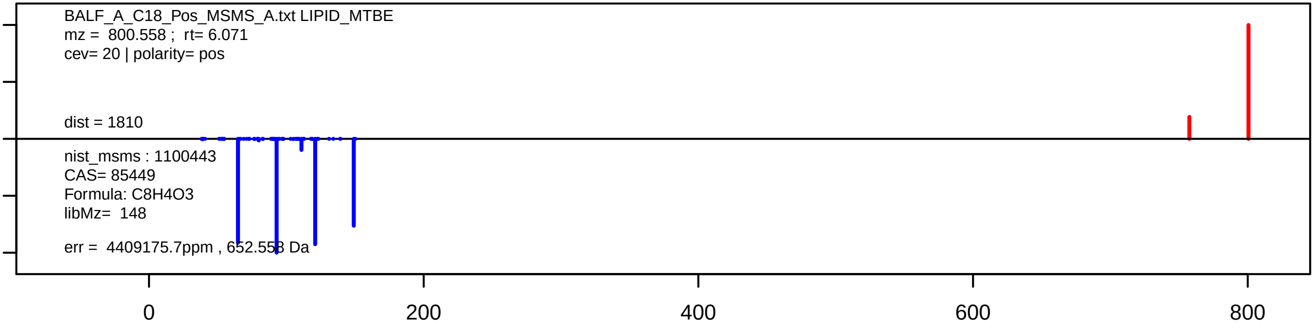

481 . Quetiapine
Score=378 Dot=921 prob=92.2

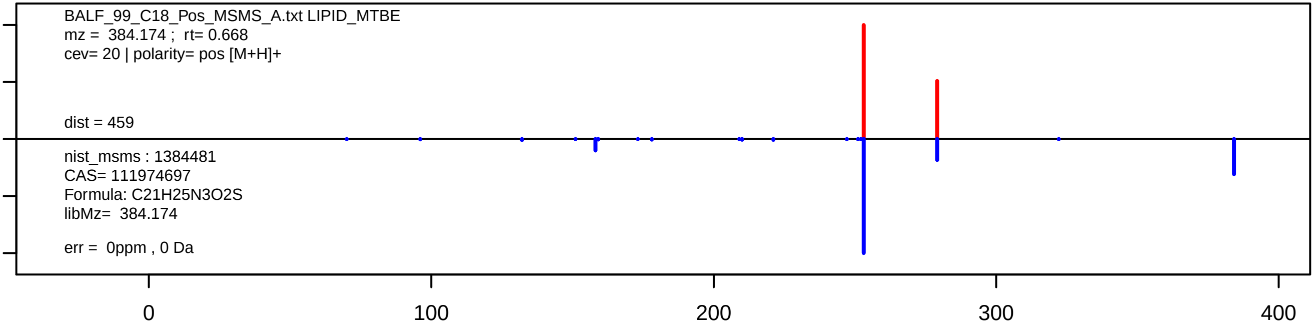

482 . rac erythro−Dihydrobupropion
Score=574 Dot=861 prob=51.8

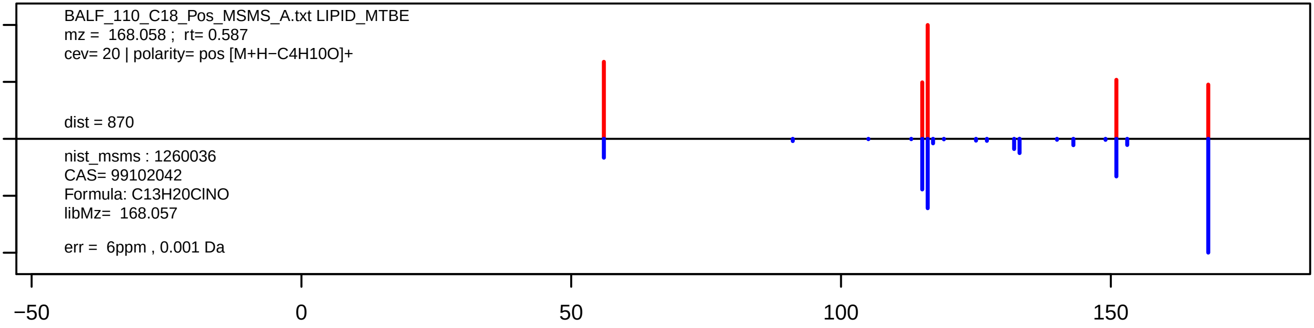

483 . Ramipril
Score=353 Dot=979 prob=98.7

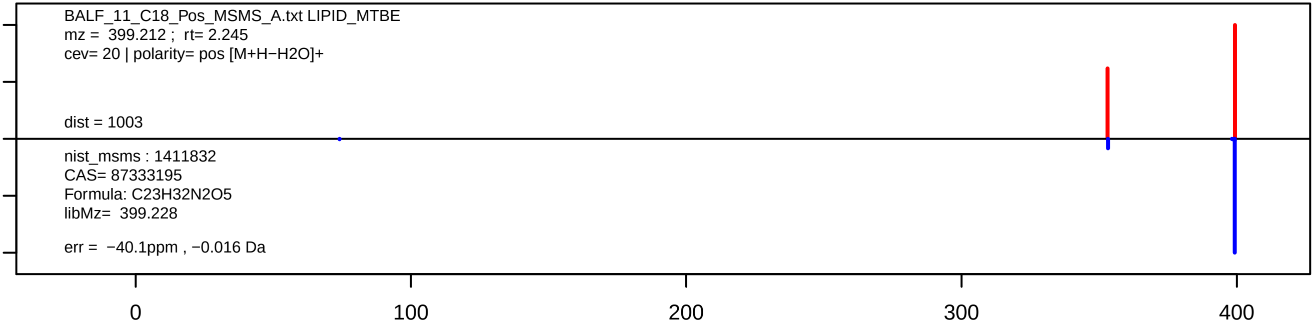

484 . Risperidone
Score=400 Dot=999 prob=96.4

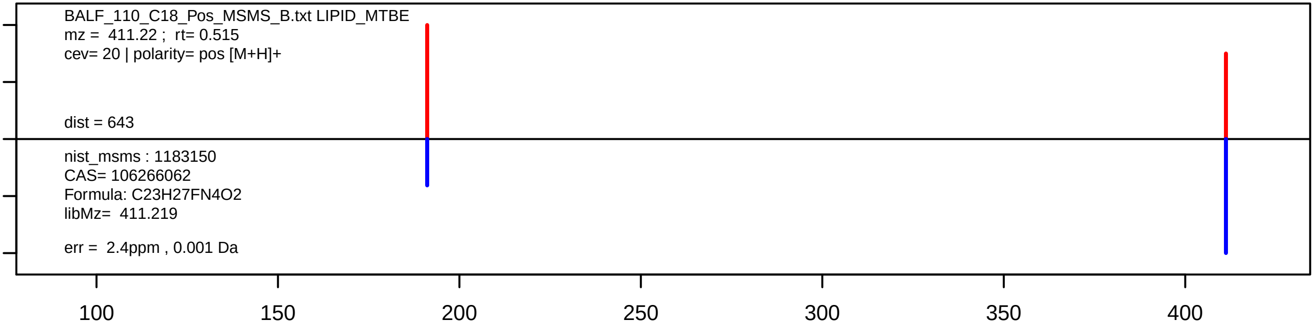

485 . SM 32:1; [M+Na]+; SM(d14:0/18:1(9Z))
Score=294 Dot=807 prob=24.9

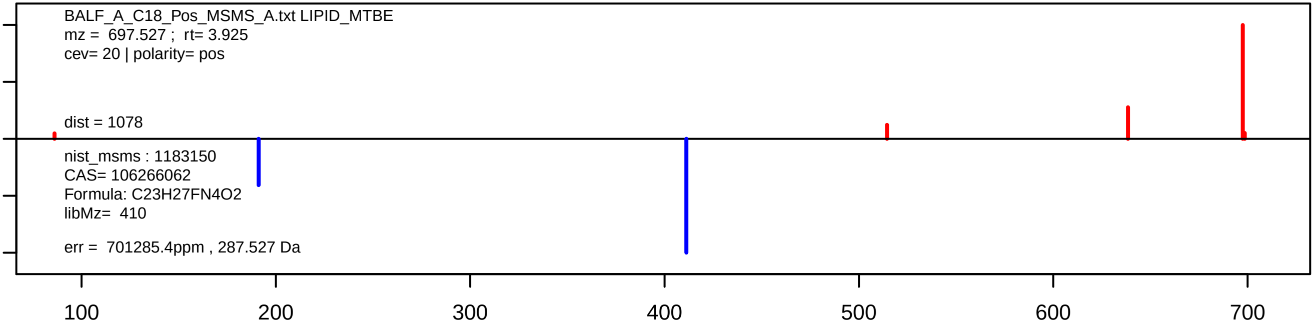

486 . SM 33:1; [M+Na]+; SM(d15:0/18:1(9Z))
Score=366 Dot=990 prob=24.9

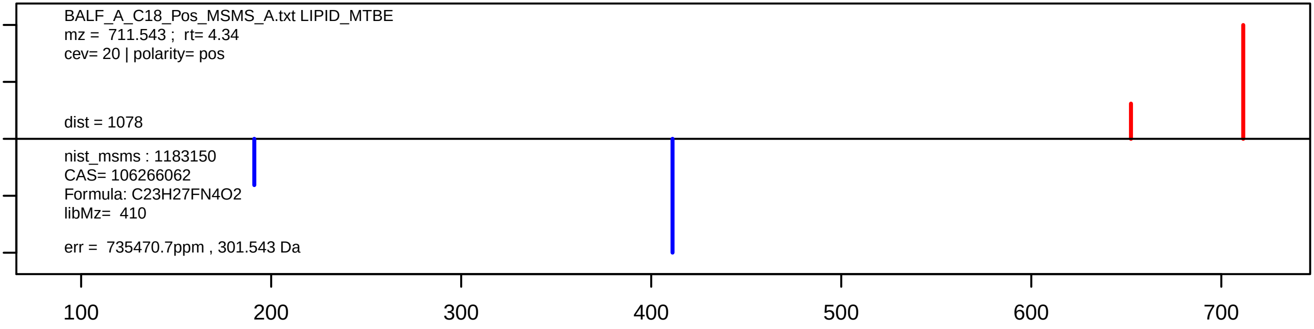

487 . SM 34:1; [M+Na]+; SM(d14:1(4E)/20:0)
Score=366 Dot=990 prob=24.8

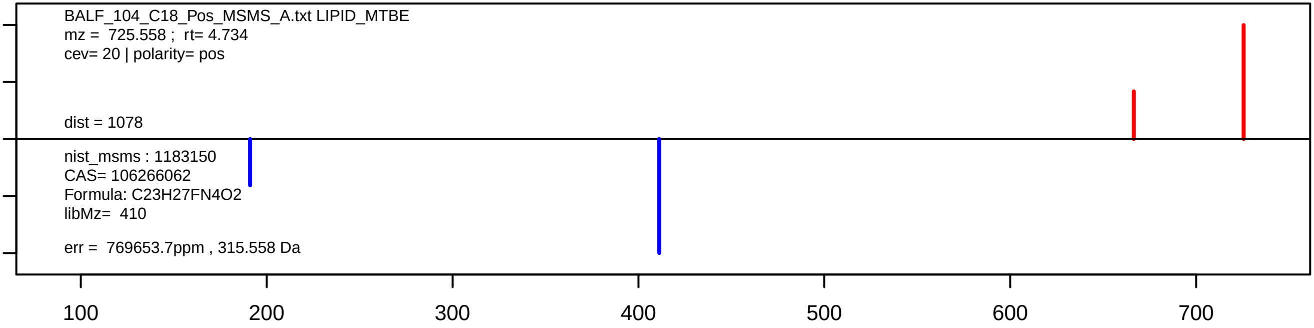

488 . SM 34:2; [M+Na]+; SM(d16:1(4E)/18:1(9Z))
Score=374 Dot=940 prob=100

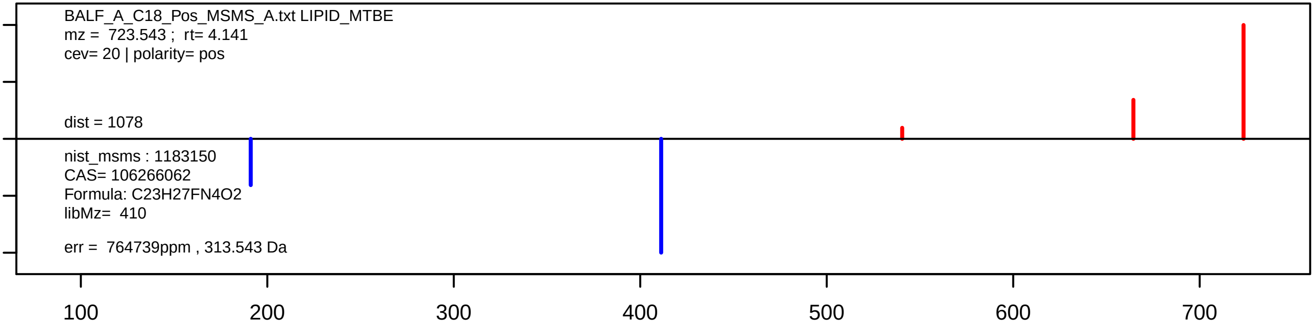

489 . SM 36:1; [M+Na]+; SM(d14:1(4E)/22:0)
Score=366 Dot=990 prob=24.9

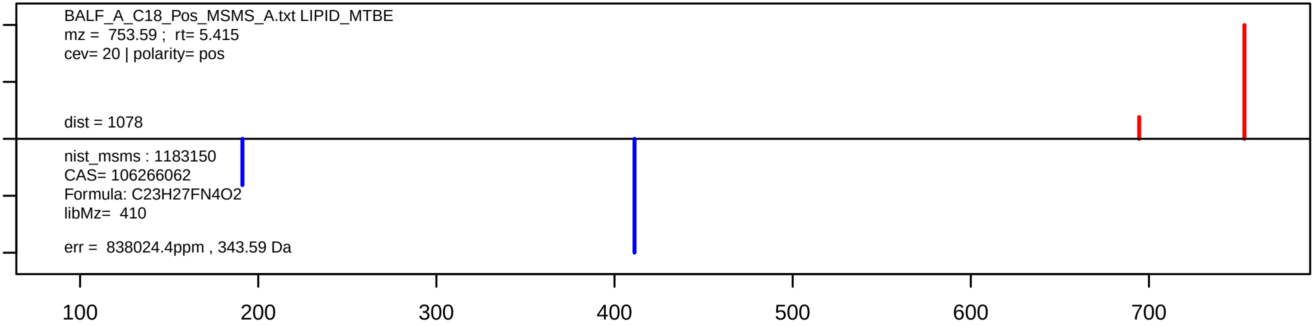

490 . SM 36:2; [M+Na]+; SM(d18:1(4E)/18:1(9Z))
Score=366 Dot=990 prob=86

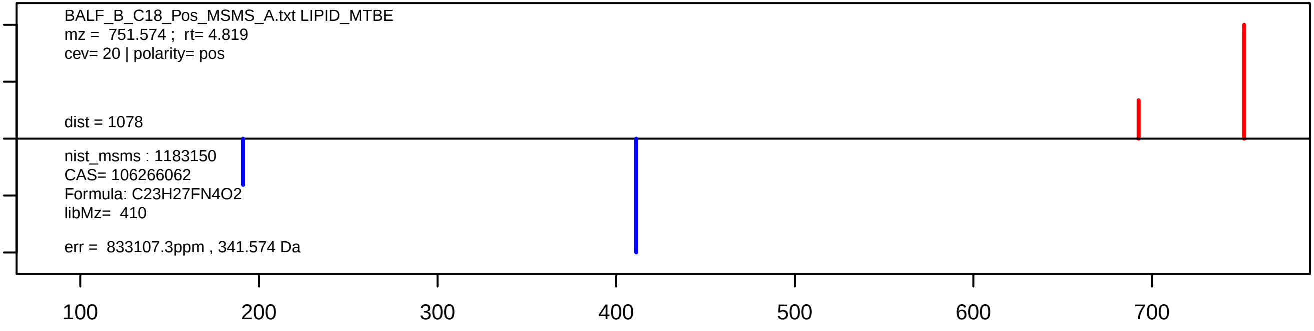

491 . SM 37:0; [M+Na]+; SM(d15:0/22:0)
Score=366 Dot=990 prob=33.2

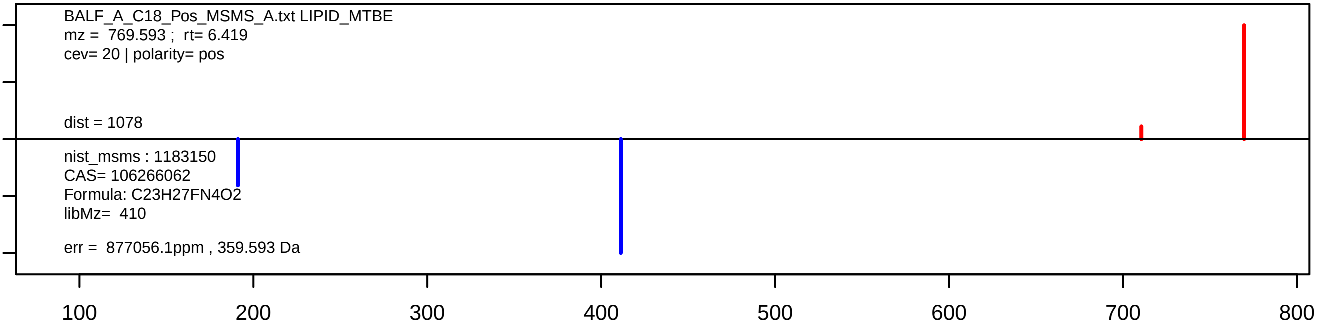

492 . SM 37:1; [M+Na]+; SM(d15:1(4E)/22:0)
Score=366 Dot=990 prob=24.9

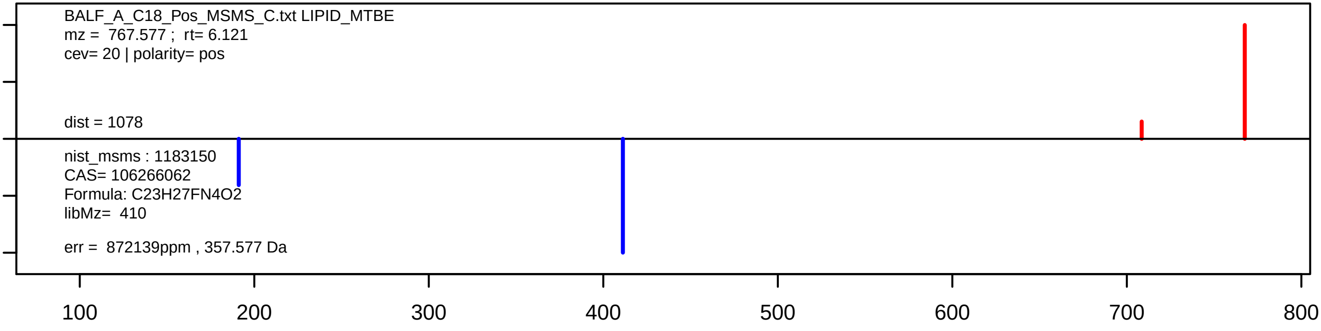

493 . SM 39:0; [M+Na]+; SM(d15:0/24:0)
Score=366 Dot=990 prob=33.3

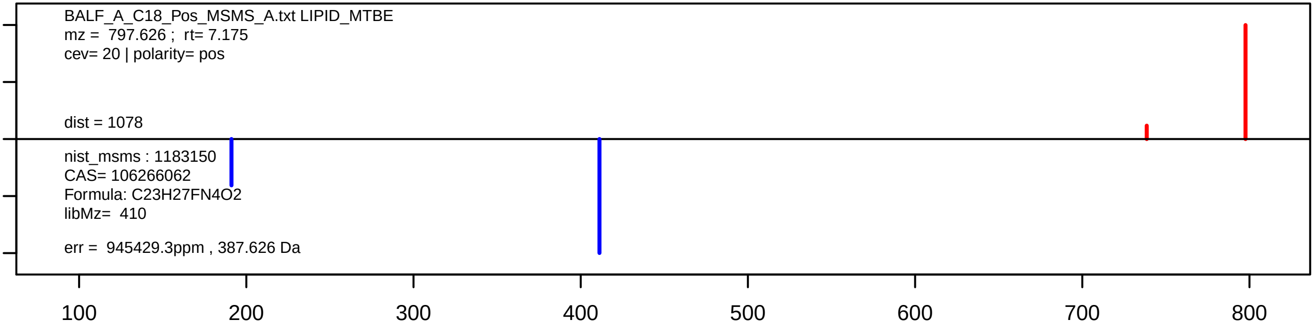

494 . SM 41:1; [M+Na]+; SM(d15:0/26:1(17Z))
Score=366 Dot=990 prob=20

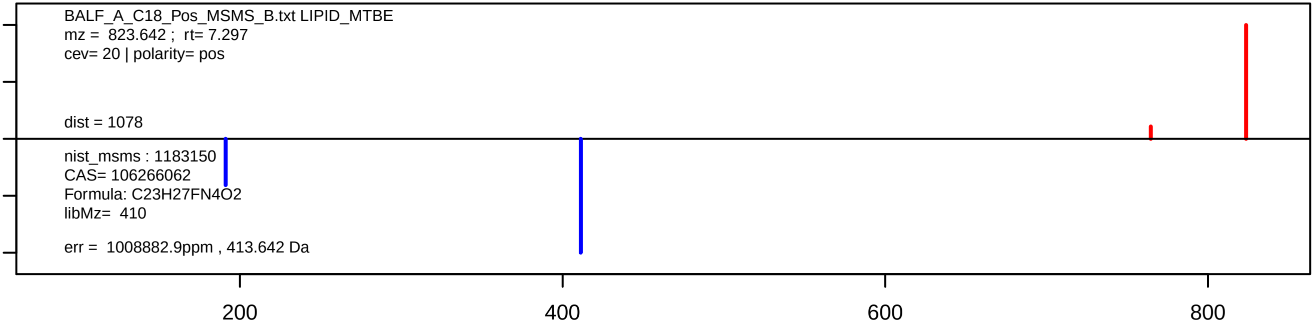

495 . SM 42:2; [M+Na]+; SM(d16:1(4E)/26:1(17Z))
Score=366 Dot=990 prob=49.8

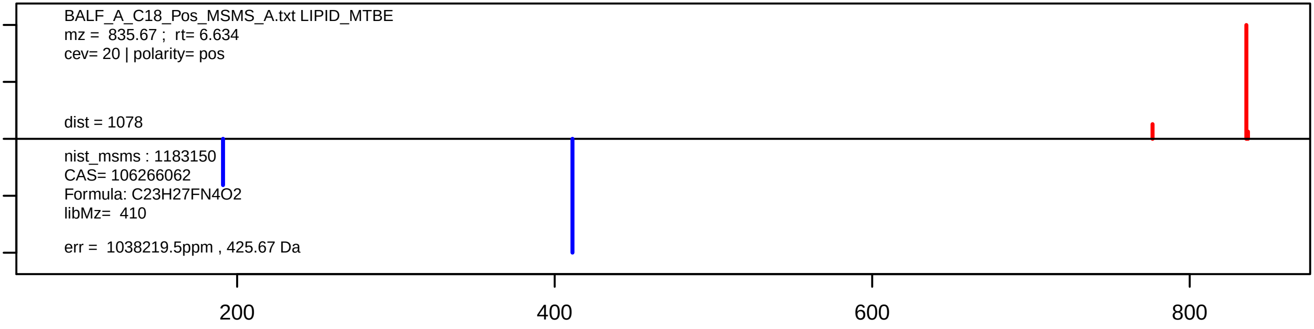

496 . SM 43:1; [M+Na]+; SM(d17:0/26:1(17Z))
Score=366 Dot=990 prob=25

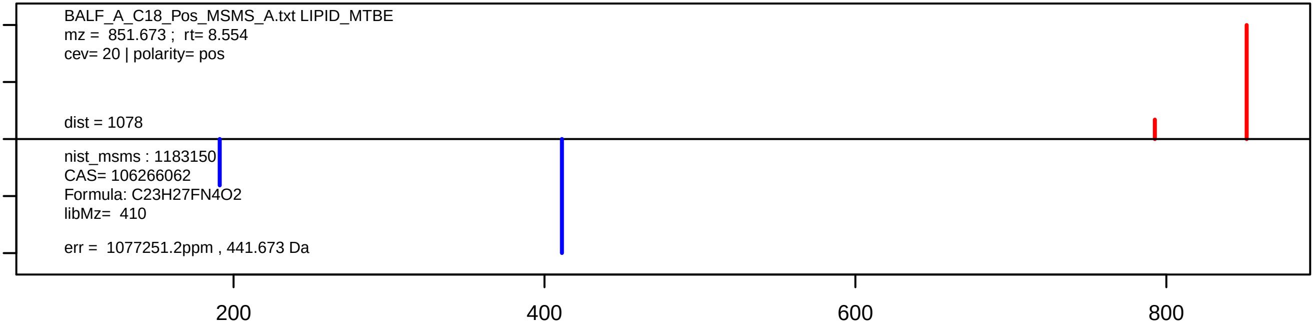

497 . SM 45:1; [M+Na]+; SM(d19:0/26:1(17Z))
Score=366 Dot=990 prob=50

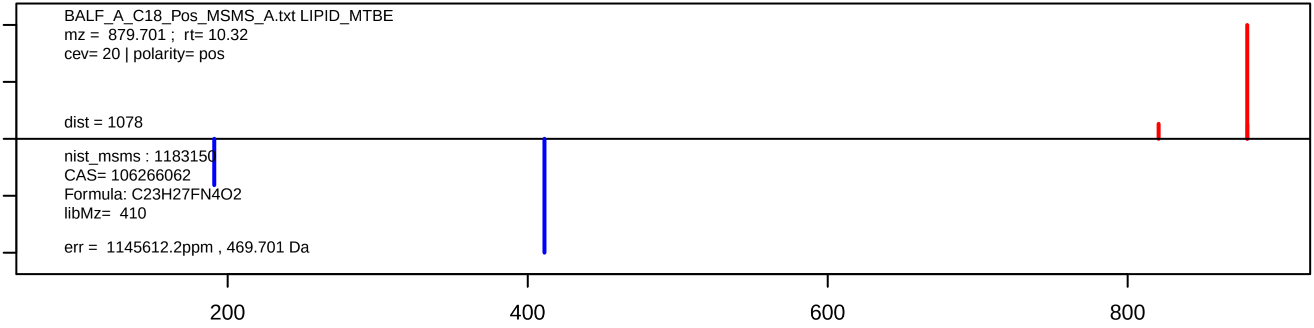

498 . Stearamide
Score=541 Dot=812 prob=98.7

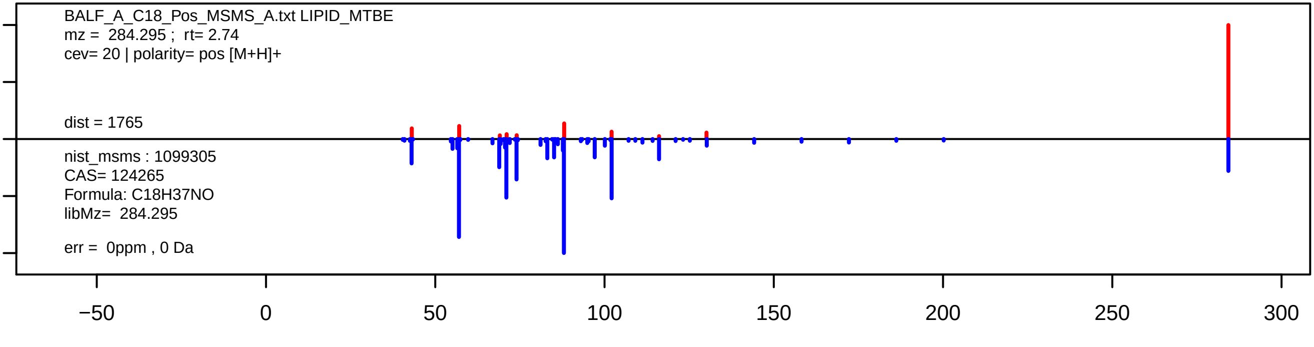

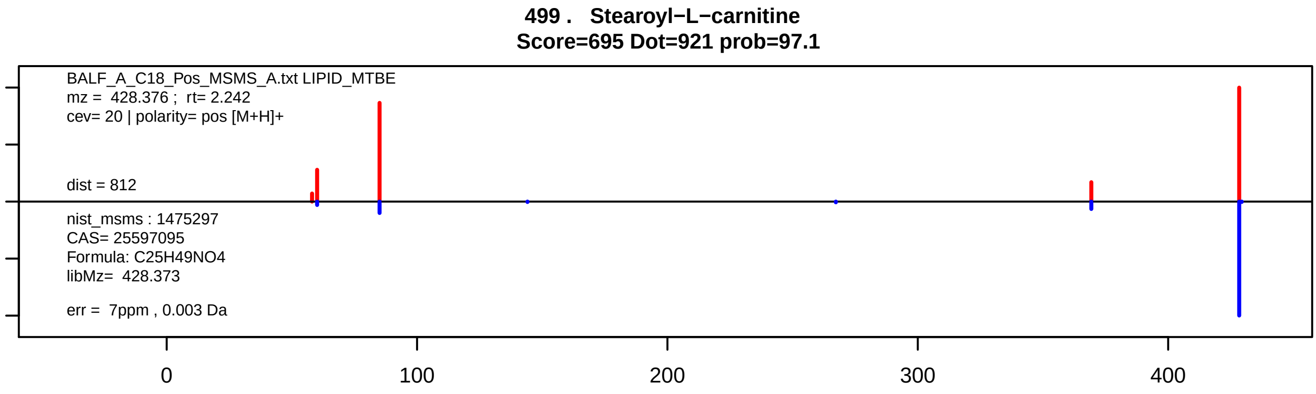
499 . Stearoyl−L−carnitine
Score=695 Dot=921 prob=97.1
BALF_A_C18_Pos_MSMS_A.txt LIPID_MTBE
mz = 428.376 ; rt= 2.242
cev= 20 | polarity= pos [M+H]+
dist = 812
nist_msms : 1475297
CAS= 25597095
Formula: C25H49NO4
libMz= 428.373
err = 7ppm , 0.003 Da
0
100
200
300
400

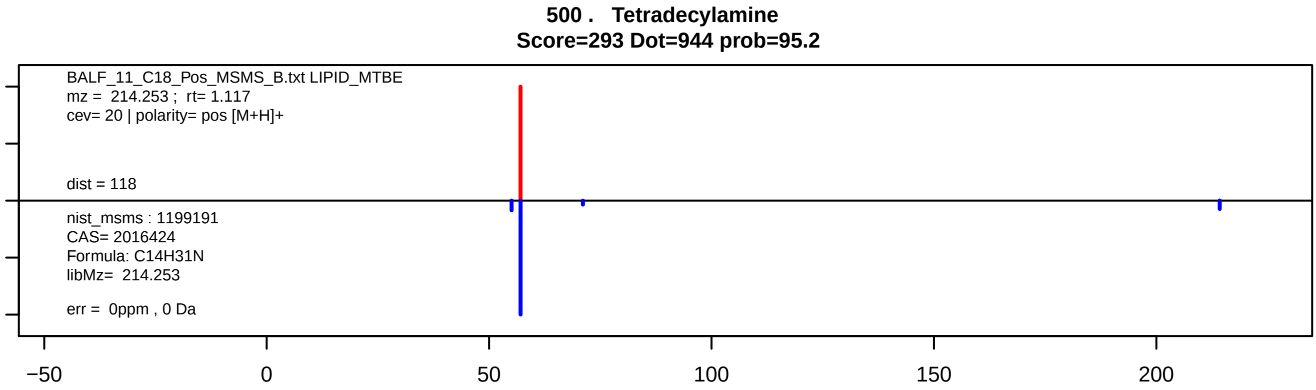
500 . Tetradecylamine
Score=293 Dot=944 prob=95.2
BALF_11_C18_Pos_MSMS_B.txt LIPID_MTBE
mz = 214.253 ; rt= 1.117
cev= 20 | polarity= pos [M+H]+
dist = 118
nist_msms : 1199191
CAS= 2016424
Formula: C14H31N
libMz= 214.253
err = 0ppm , 0 Da
−50
0
50
100
150
200

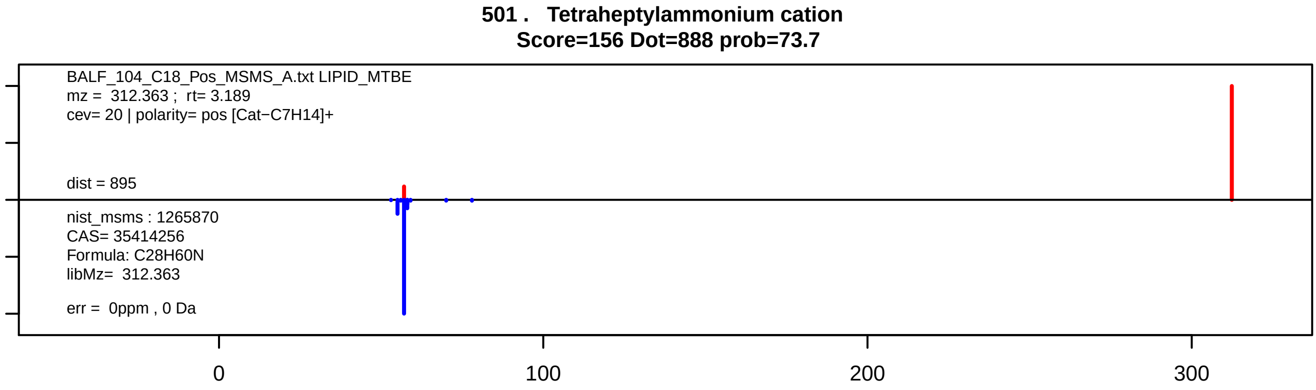
501 . Tetraheptylammonium cation
Score=156 Dot=888 prob=73.7
BALF_104_C18_Pos_MSMS_A.txt LIPID_MTBE
mz = 312.363 ; rt= 3.189
cev= 20 | polarity= pos [Cat−C7H14]+
dist = 895
nist_msms : 1265870
CAS= 35414256
Formula: C28H60N
libMz= 312.363
err = 0ppm , 0 Da
0
100
200
300

502 . TG 52:3; [M+NH4]+; TG(16:0/18:1/18:2)
Score=806 Dot=967 prob=96.2

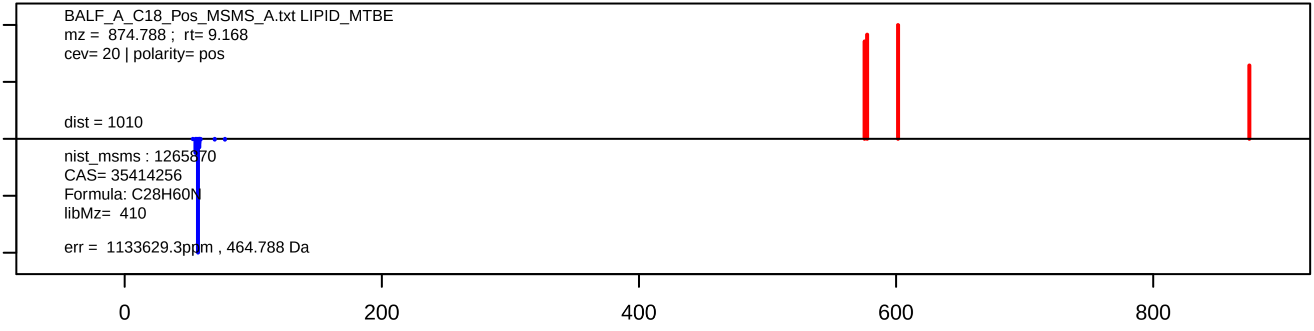

503 . Thioetheramidephosphatidylcholine
Score=410 Dot=878 prob=99

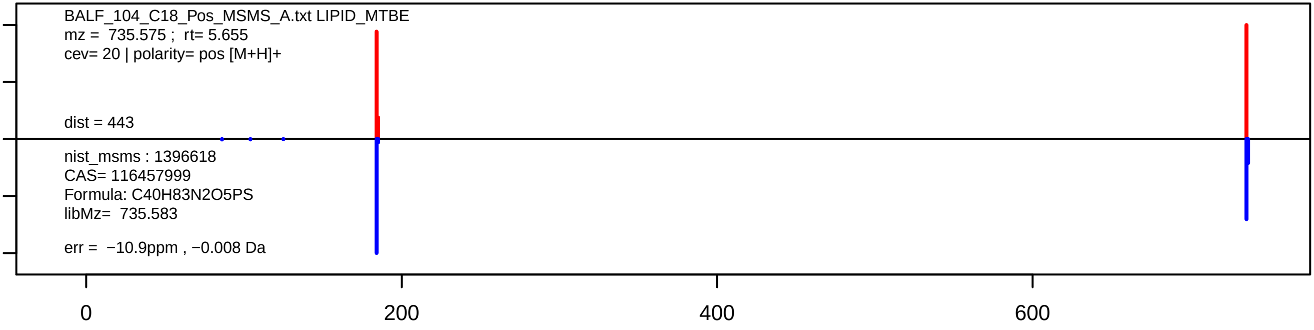

504 . threo−Dihydrobupropion
Score=706 Dot=973 prob=96.7

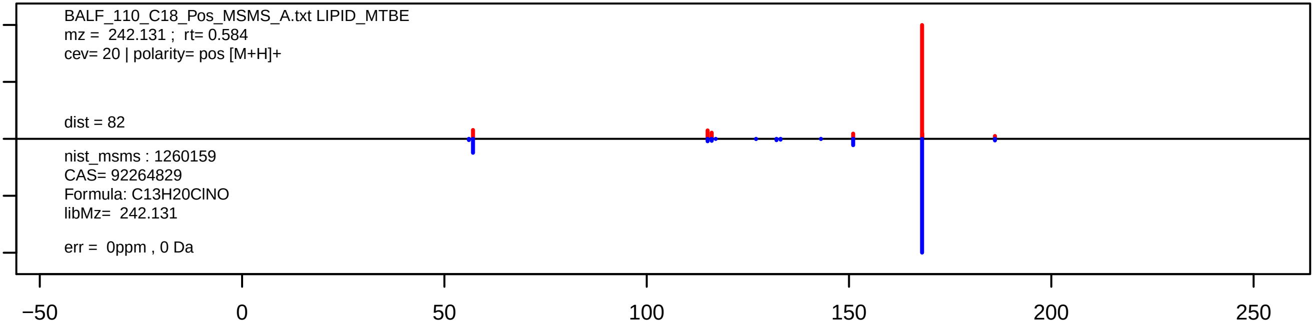

505 .   Tramadol
Score=332 Dot=957 prob=96.6

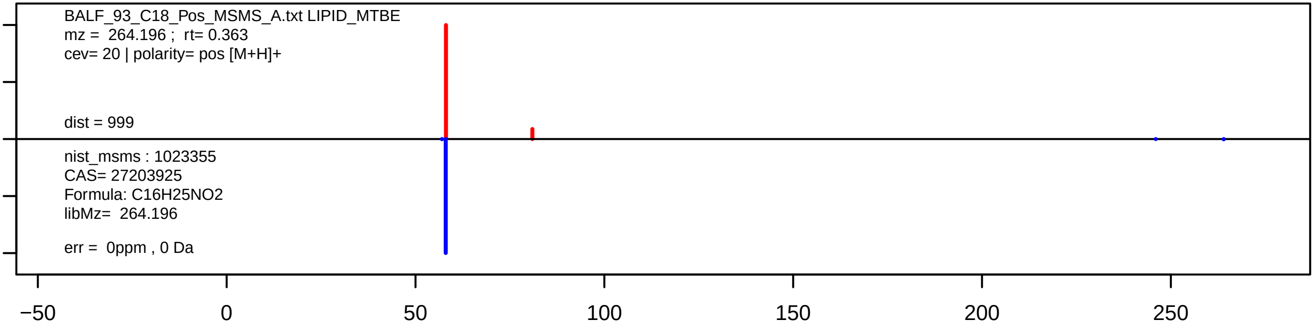

506 .   Tributyl phosphate
Score=400 Dot=999 prob=97.5

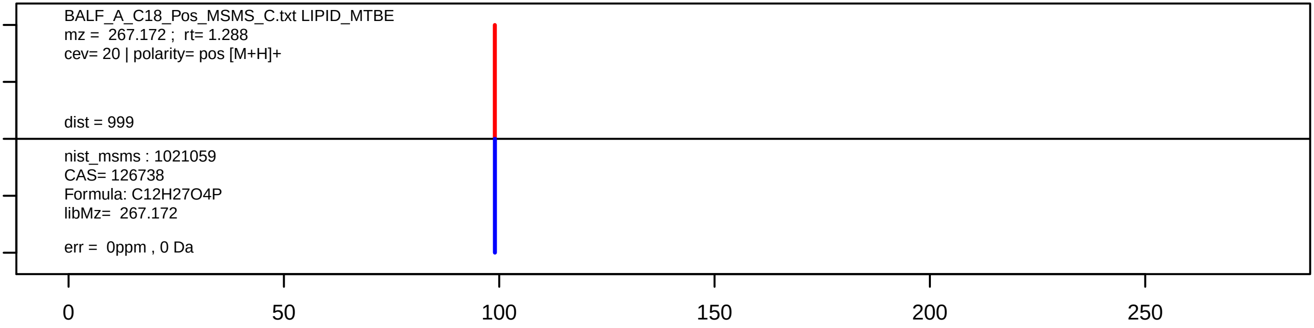

507 .   Triethylhexyammonium cation
Score=495 Dot=943 prob=42.4

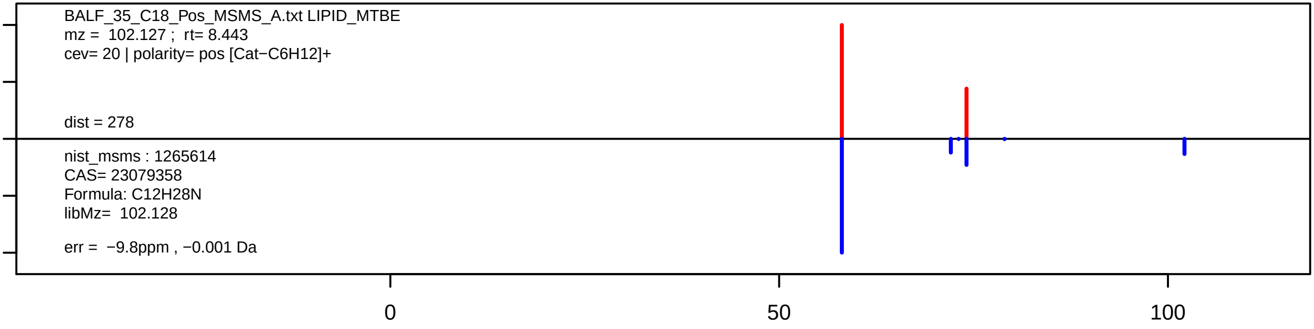

508 .   Venlafaxine
Score=609 Dot=966 prob=97.6

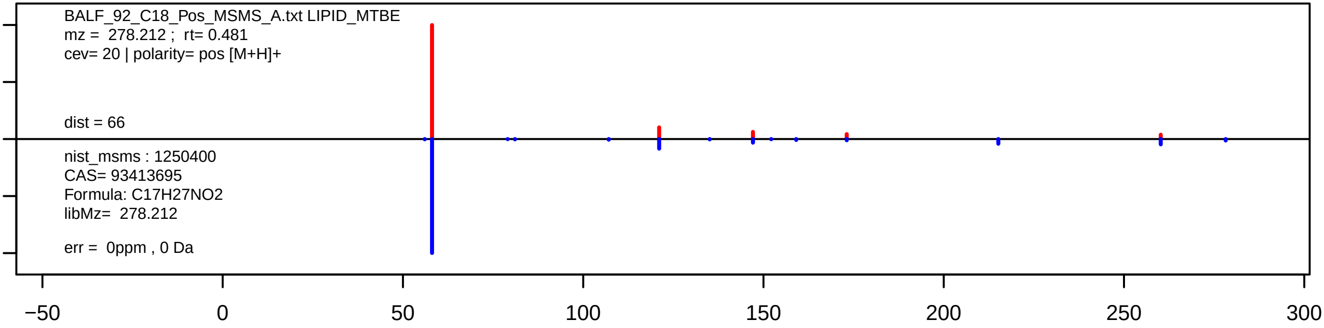

509 .   (+)−Methamphetamine
Score=609 Dot=936 prob=93.5

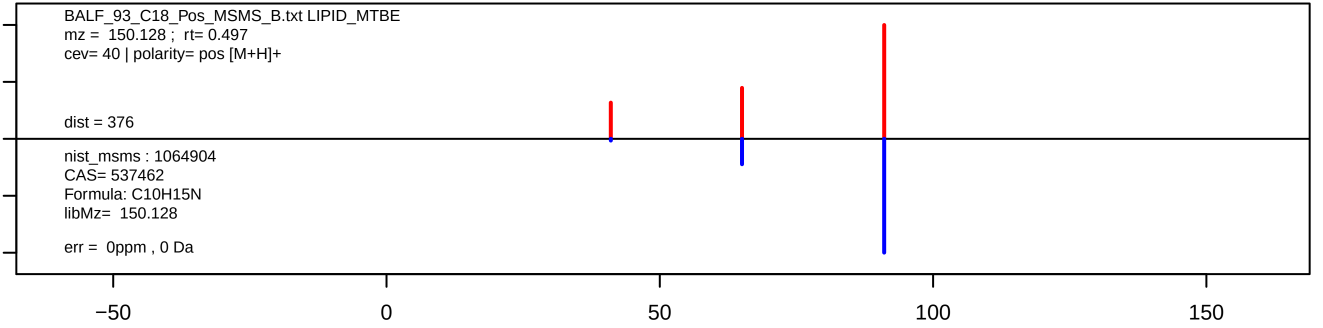

510 .   1−(1Z−Hexadecenyl)−sn−glycero−3−phosphocholine
Score=302 Dot=814 prob=97.7

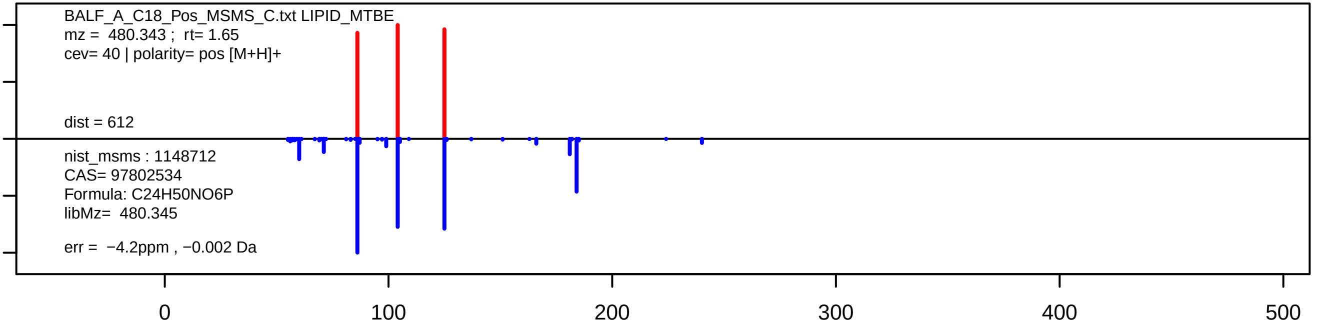

511 . 1−(1Z−Octadecenyl)−2−(5Z,8Z,11Z,14Z−eicosatetraenoyl)−sn−glycero−3−phosphocholine
Score=400 Dot=999 prob=99

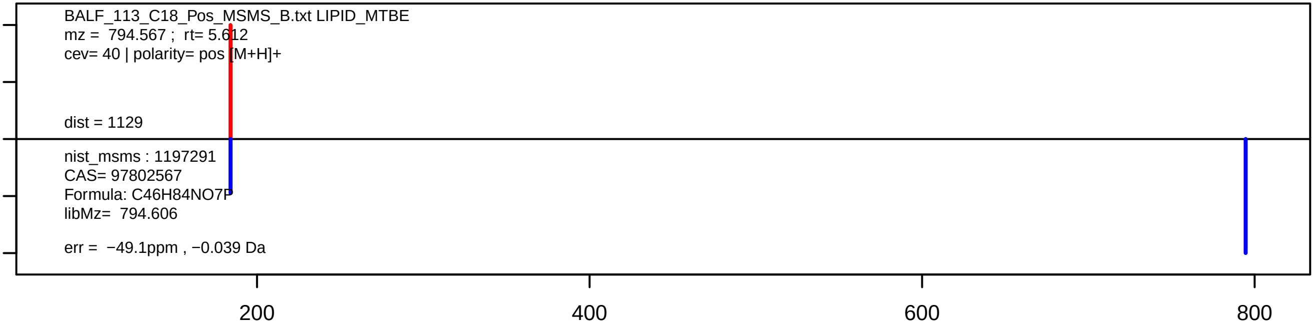

512 . 1−(1Z−Octadecenyl)−2−(9Z−octadecenoyl)−sn−glycero−3−phosphocholine
Score=365 Dot=984 prob=98.9

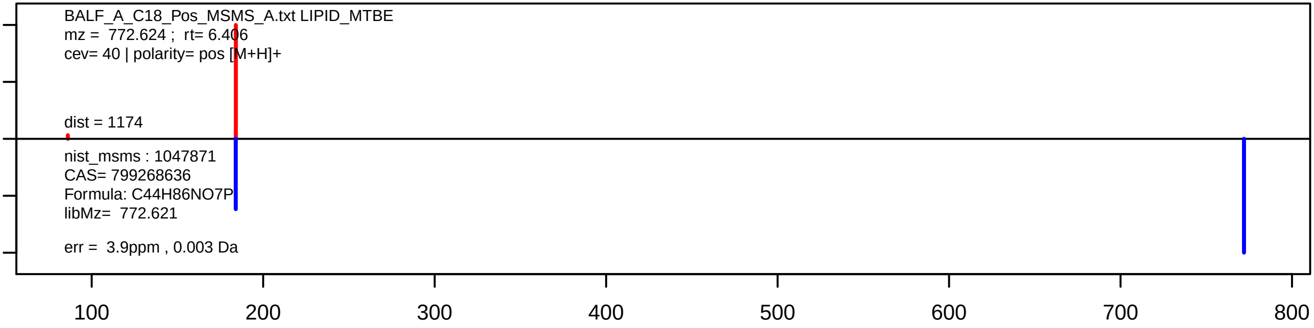

513 . 1−(1Z−Octadecenyl)−sn−glycero−3−phosphocholine
Score=618 Dot=866 prob=98.9

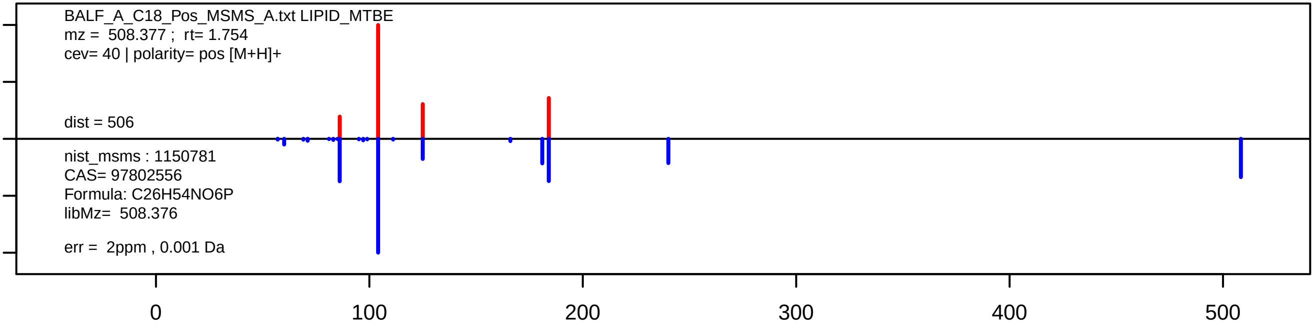

514 . 1−Cyclohexylethanol
Score=380 Dot=886 prob=66.4

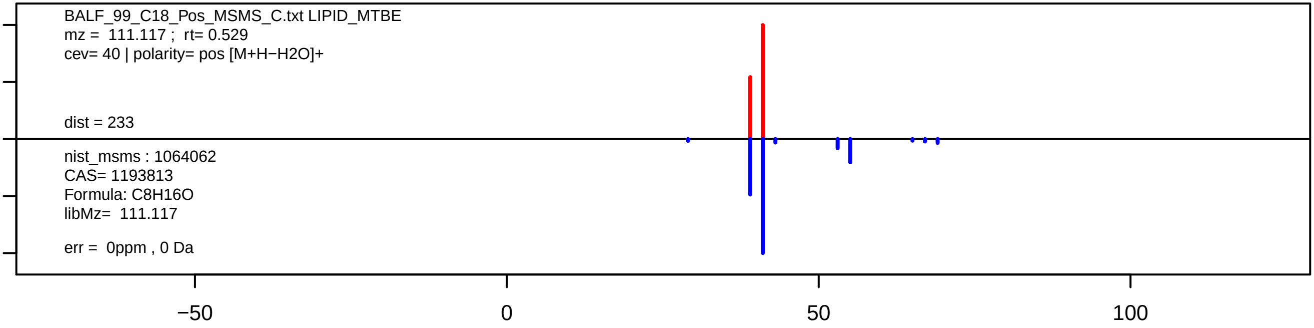

515 . 1−Heneicosanoyl−2−(4Z,7Z,10Z,13Z,16Z,19Z−docosahexaenoyl)−sn−glycero−3−phosphocholine
Score=281 Dot=987 prob=99

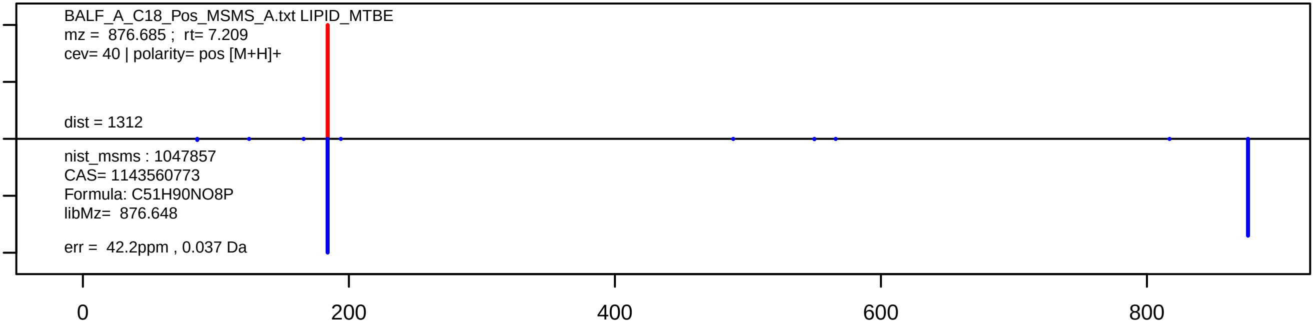

516 . 1−Heptadecanoyl−2−(5Z,8Z,11Z,14Z−eicosatetraenoyl)−sn−glycero−3−phosphocholine
Score=145 Dot=932 prob=100

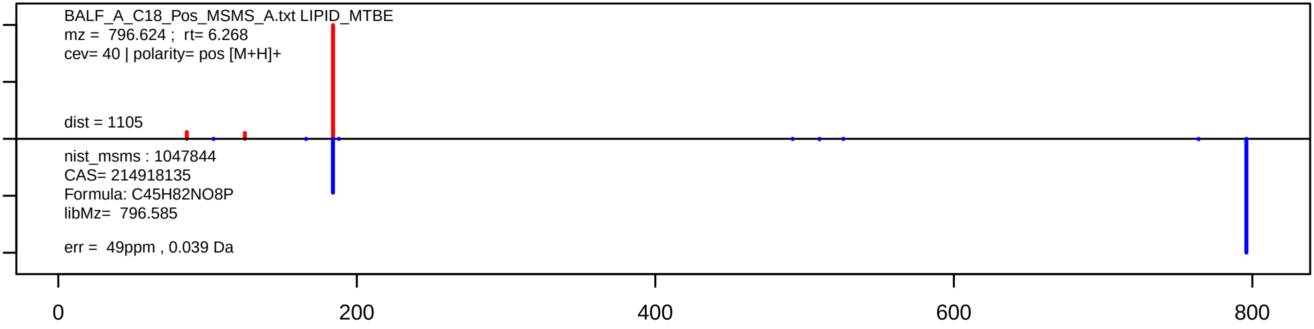

517 . 1−Heptadecanoyl−2−(9Z−tetradecenoyl)−sn−glycero−3−phosphocholine
Score=286 Dot=829 prob=56.6

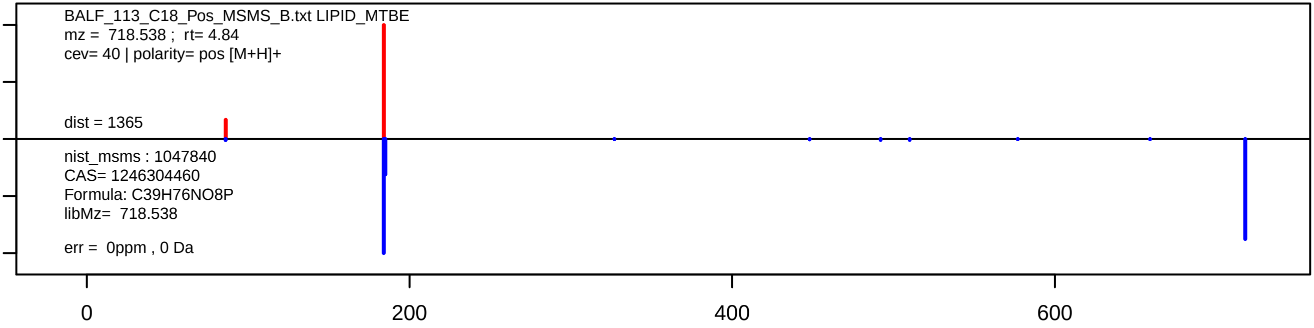

518 . 1−Heptadecanoyl−sn−glycero−3−phosphocholine
Score=702 Dot=915 prob=98.5

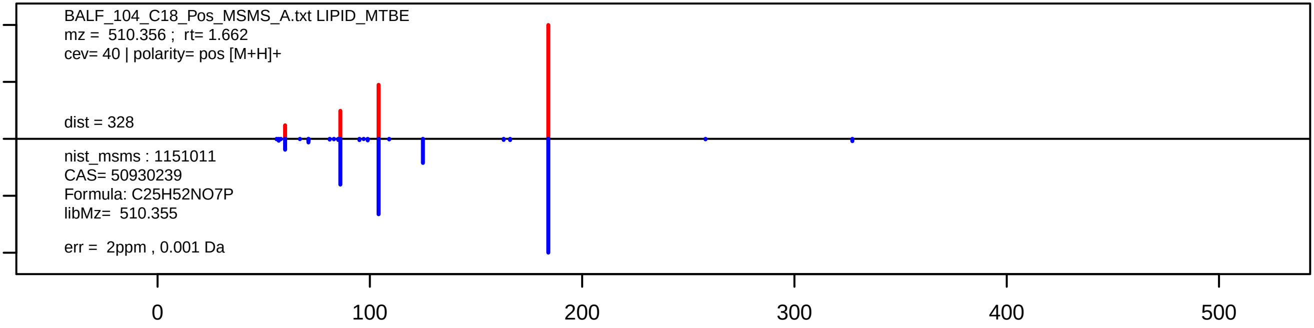

519 . 1−Hexadecanoyl−2−(14−pentadecenoyl)−sn−glycero−3−phosphocholine
Score=295 Dot=991 prob=94

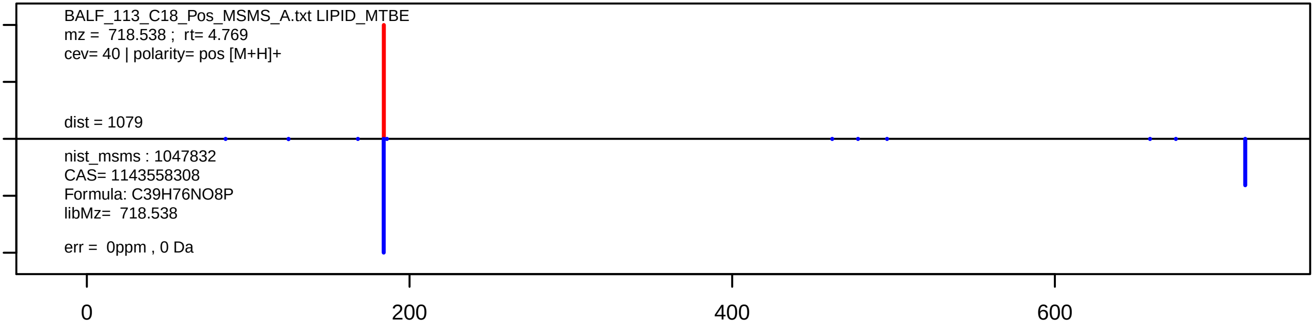

520 . 1−Hexadecanoyl−2−(5Z,8Z,11Z,14Z−eicosatetraenoyl)−sn−glycero−3−phosphocholine
Score=400 Dot=999 prob=97.8

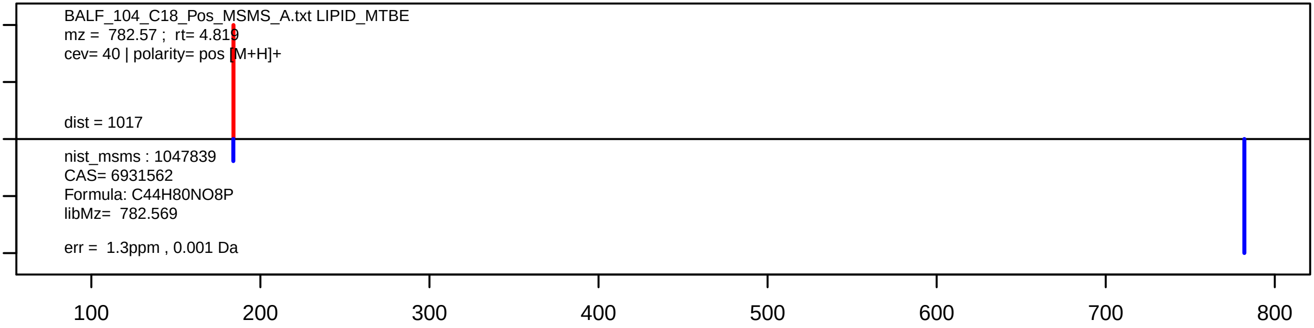

521 . 1−Hexadecanoyl−2−octadecadienoyl−sn−glycero−3−phosphocholine
Score=575 Dot=976 prob=97.3

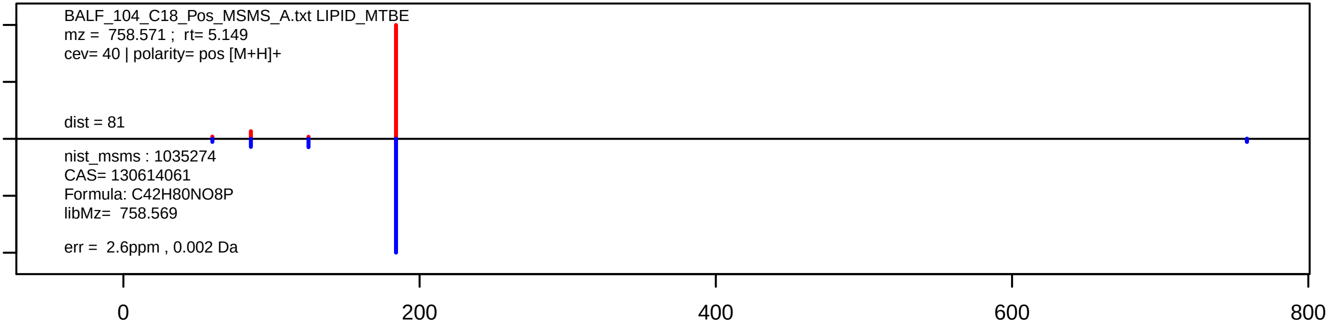

522 . 1−Hexadecyl−2−(5Z,8Z,11Z,14Z−eicosatetraenoyl)−sn−glycero−3−phosphocholine
Score=273 Dot=819 prob=99

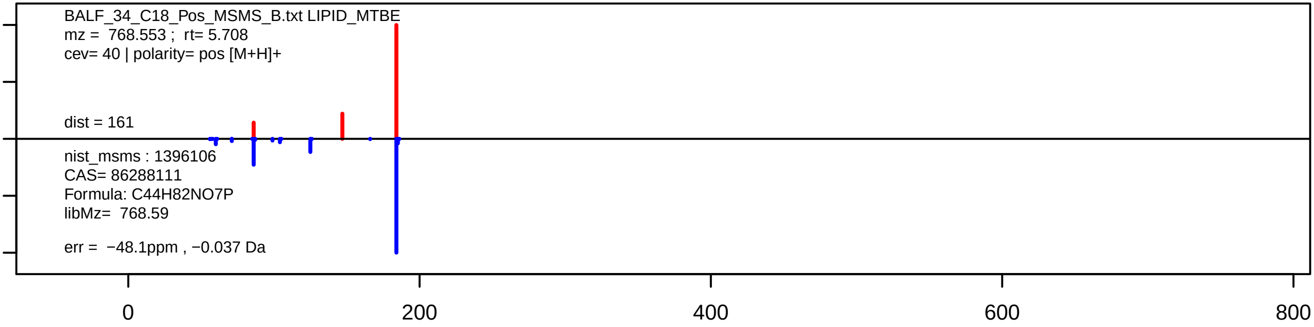

523 . 1−Hexadecyl−2−(8Z,11Z,14Z−eicosatrienoyl)−sn−glycero−3−phosphocholine
Score=243 Dot=931 prob=100

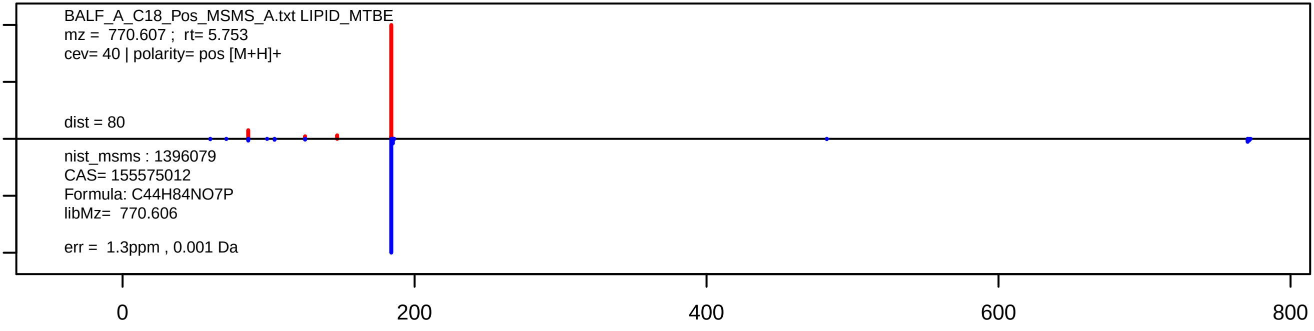

524 . 1−Hexadecyl−2−(9Z−octadecenoyl)−sn−glycero−3−phosphocholine
Score=400 Dot=999 prob=100

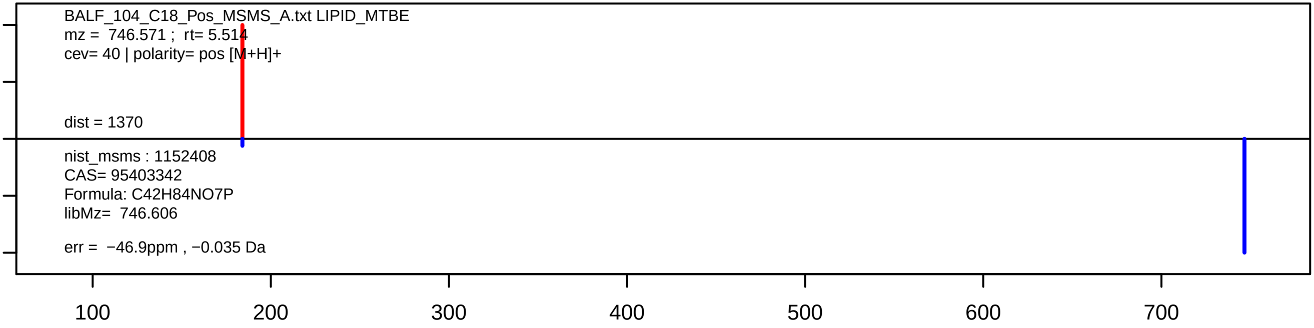

525 . 1−Hexadecyl−sn−glycero−3−phosphocholine
Score=891 Dot=955 prob=99

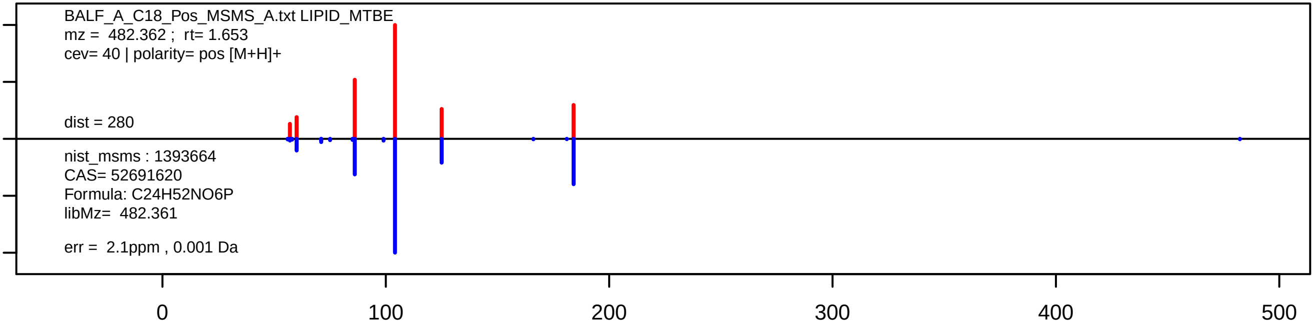

526 . 1−Hexadecylamine
Score=199 Dot=805 prob=88.5

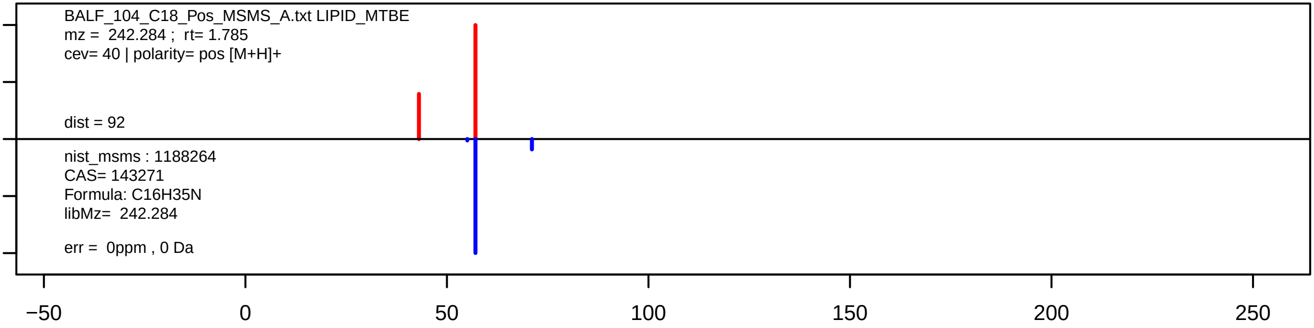

527 . 1−Hexadecylpyridinium cation
Score=560 Dot=914 prob=95.4

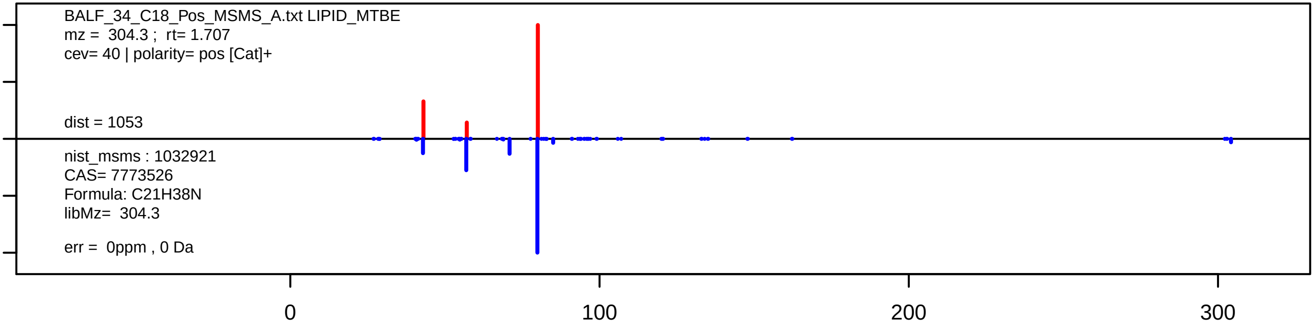

528 . 1−Linoleoyl−2−stearoyl−sn−glycero−3−phospho−(1'−sn−glycerol)
Score=370 Dot=883 prob=55.1

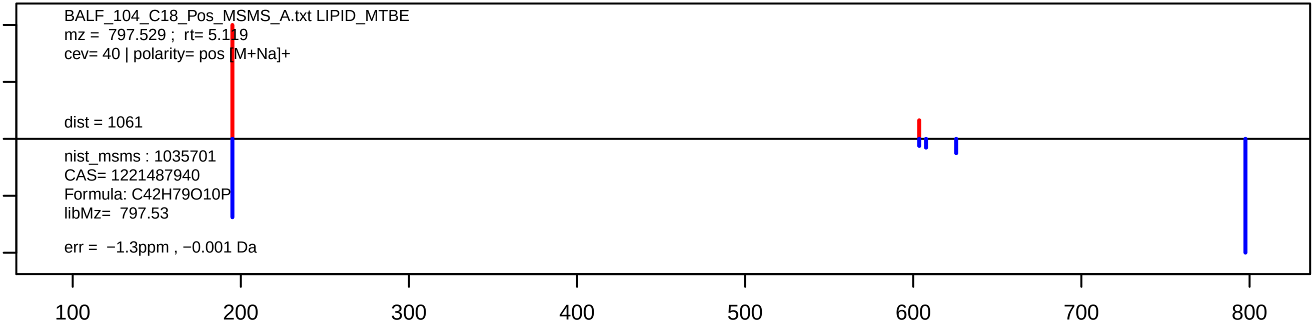

529 .   1−Methyl−3−phenylpropylamine
Score=400 Dot=999 prob=35.7

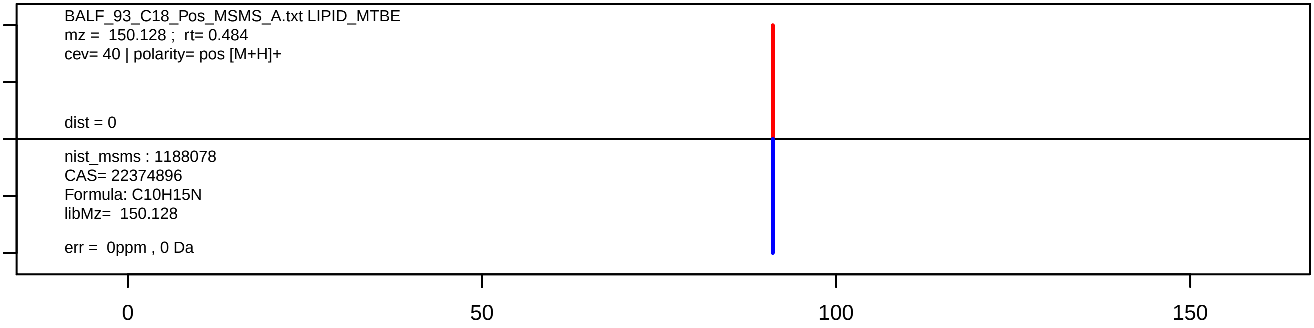

530 .   1−Myristoyl−2−palmitoyl−sn−glycero−3−phosphocholine
Score=290 Dot=921 prob=44.3

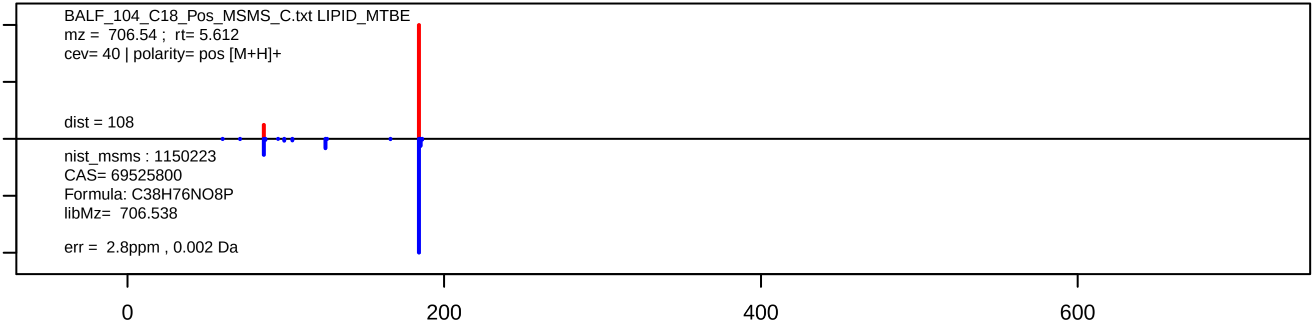

531 .   1−O−Hexadecyl−2−O−(2E−butenoyl)−sn−glyceryl−3−phosphocholine
Score=473 Dot=830 prob=98.9

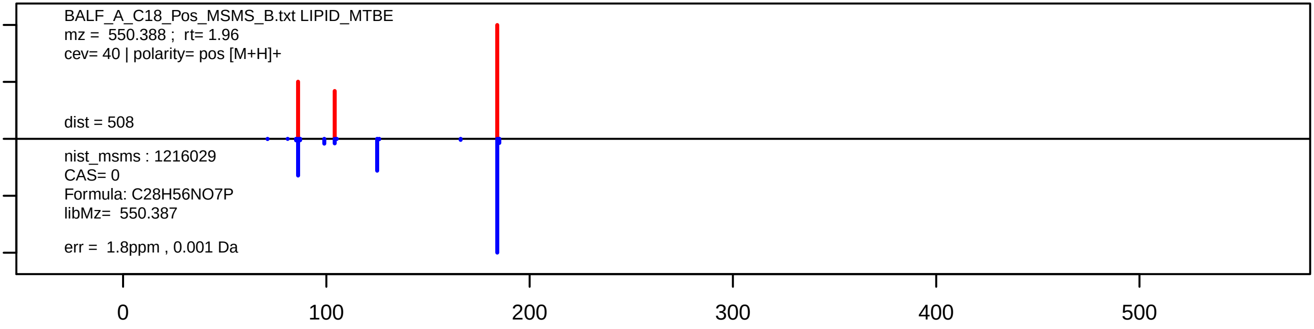

532 .   1−O−Hexadecyl−2−O−(4Z,7Z,10Z,13Z,16Z,19Z−docosahexaenoyl)−sn−glyceryl−3−phosphorylcholine
Score=182 Dot=905 prob=96.5

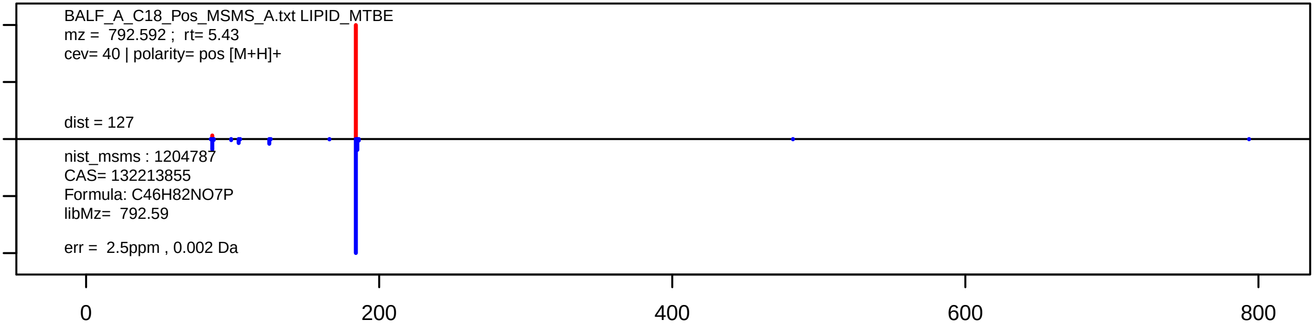

533 .   1−O−Hexadecyl−2−O−(5Z,8Z,11Z,14Z,17Z−eicosapentaenoyl)−sn−glyceryl−3−phosphorylcholine
Score=144 Dot=917 prob=96.8

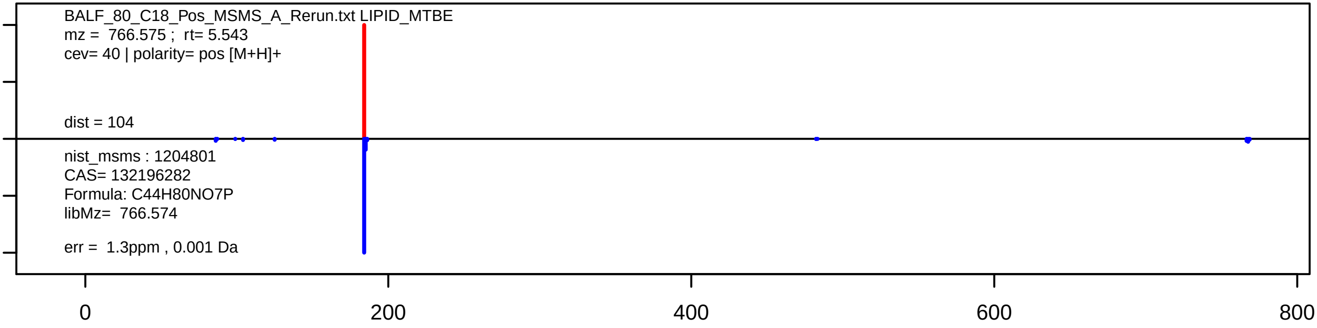

534 .   1−O−Hexadecyl−2−O−acetyl−sn−glyceryl−3−phosphorylcholine
Score=421 Dot=907 prob=98.3

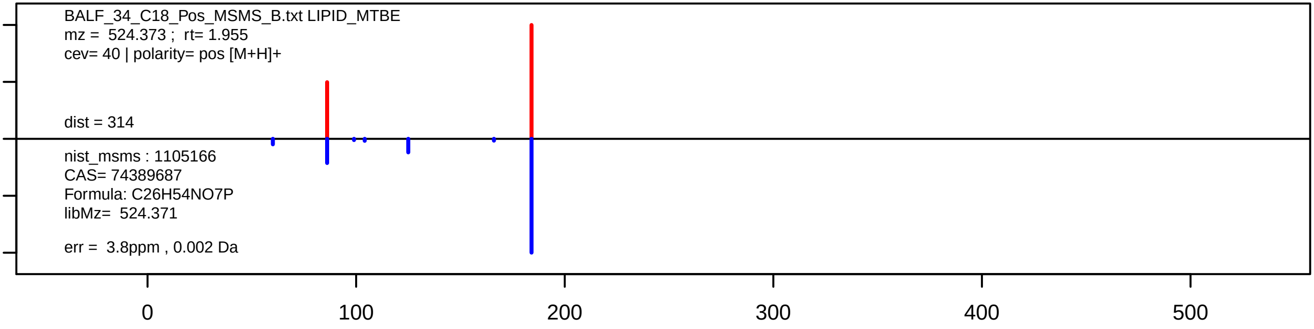

535 . 1−O−Octadecyl−sn−glyceryl−3−phosphorylcholine
Score=942 Dot=965 prob=98.4

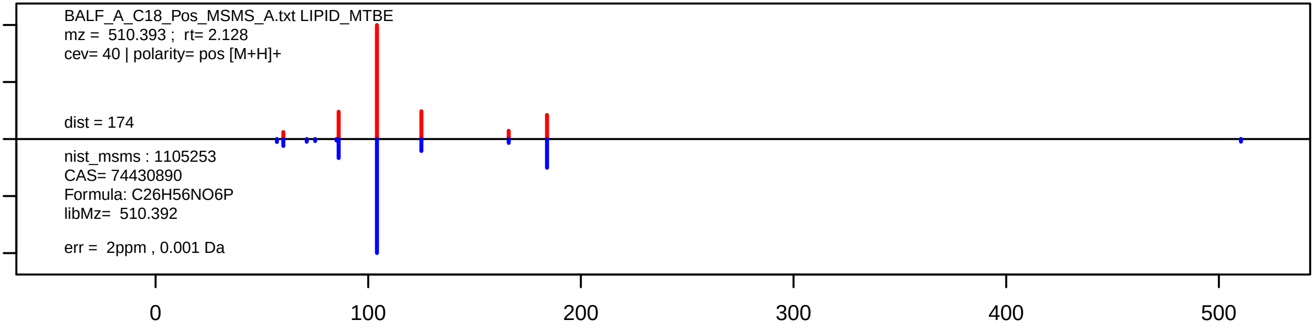

536 . 1−Octadecanoyl−2−octadecenoyl−sn−glycero−3−phosphocholine
Score=403 Dot=881 prob=99

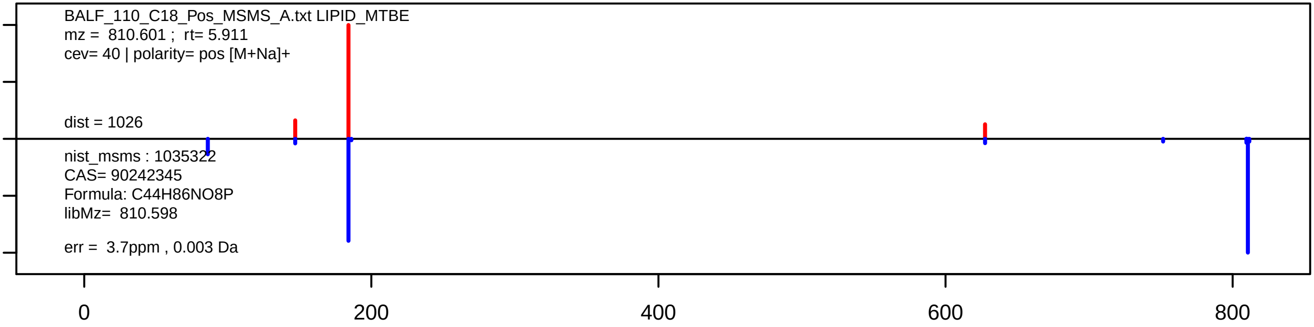

537 . 1−Oleoyl−2−palmitoyl−sn−glycero−3−phosphocholine
Score=617 Dot=985 prob=96.4

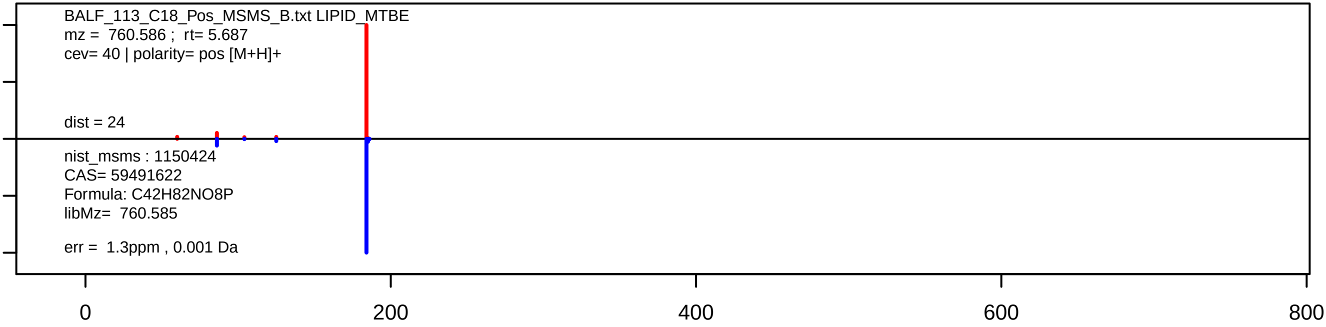

538 . 1−Oleoyl−sn−glycero−3−phosphocholine
Score=646 Dot=925 prob=98.4

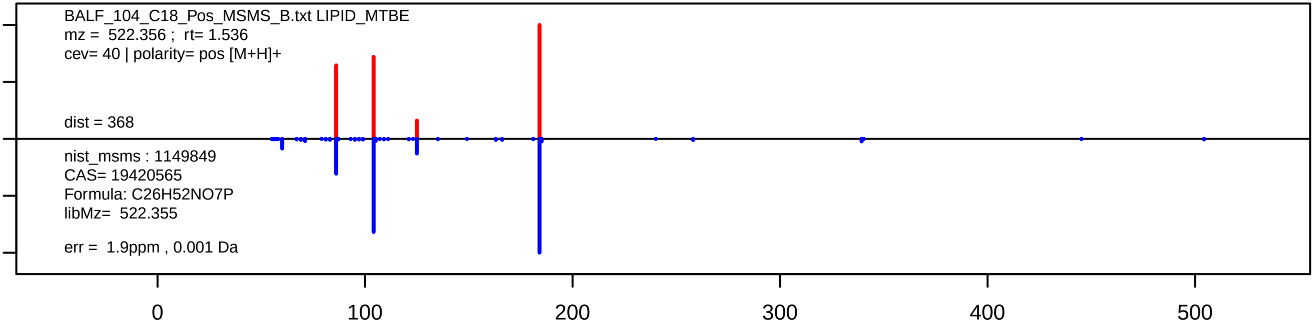

539 . 1−Palmitoyl−2−docosahexaenoyl−sn−glycero−3−phosphocholine
Score=300 Dot=856 prob=99

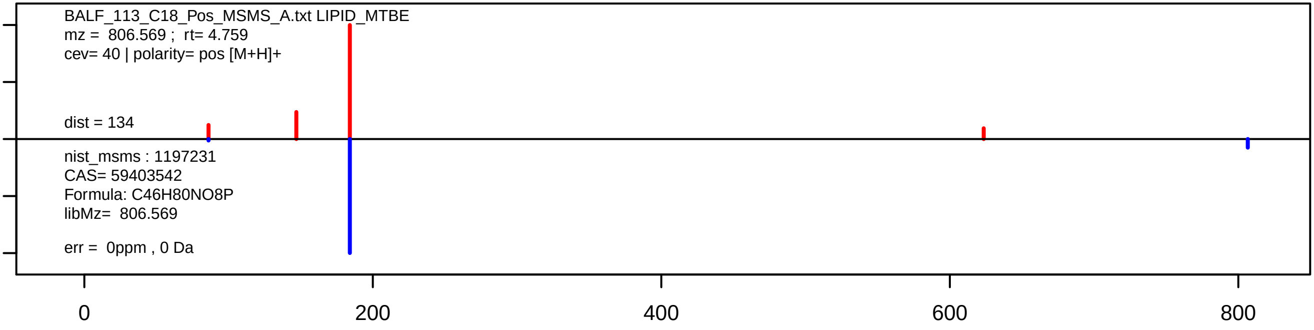

540 . 1−Palmitoyl−2−lauroyl−sn−glycero−3−phosphorylcholine
Score=310 Dot=956 prob=73.2

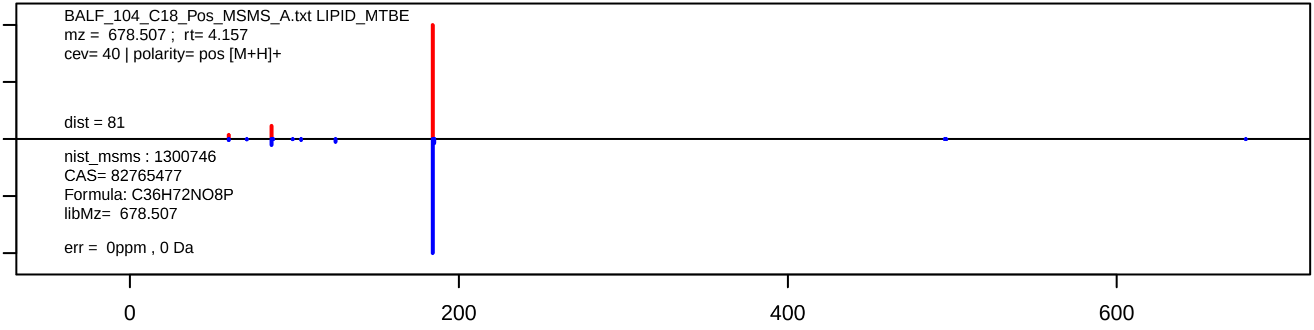

541 . 1−palmitoyl−2−linoleoyl−sn−glycero−3−phospho−(1'−rac−glycerol)
Score=347 Dot=957 prob=98.4

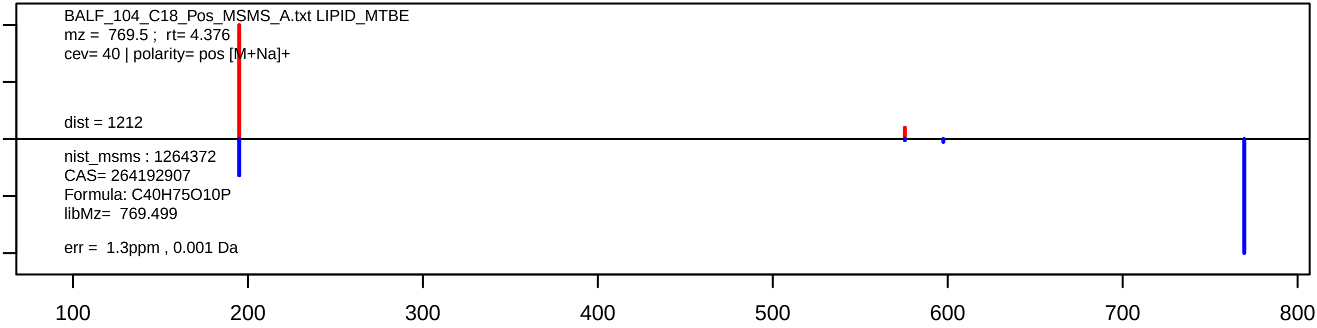

542 . 1−Palmitoyl−2−linoleoyl−sn−glycero−3−phosphocholine
Score=446 Dot=992 prob=62.3

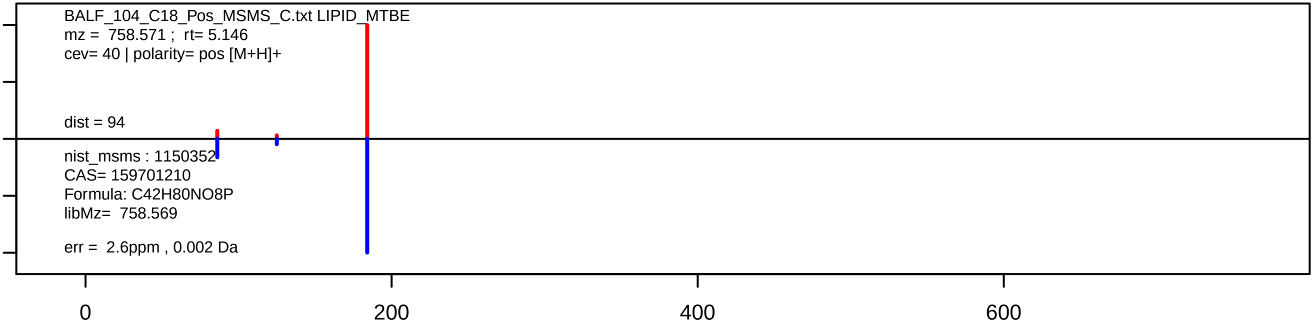

543 . 1−Palmitoyl−2−myristoyl−sn−glycero−3−phosphocholine
Score=695 Dot=913 prob=90.6

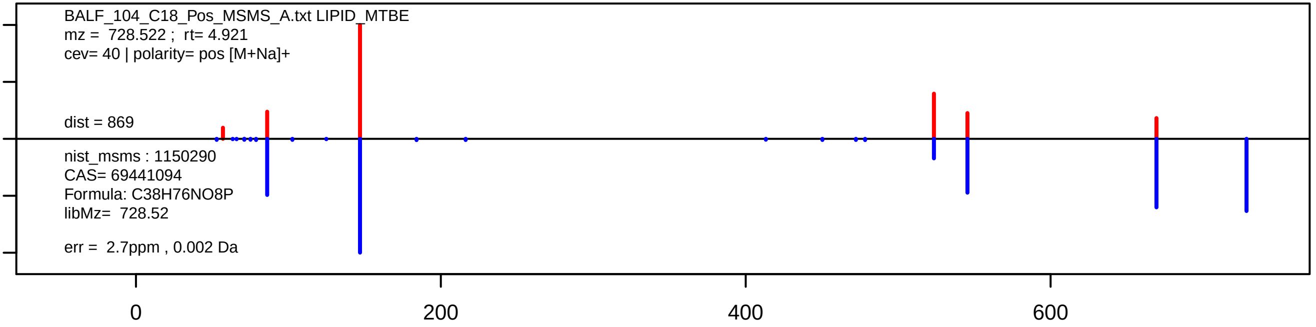

544 .   1−Palmitoyl−2−oleoyl−phosphatidylglycerol
Score=448 Dot=940 prob=100

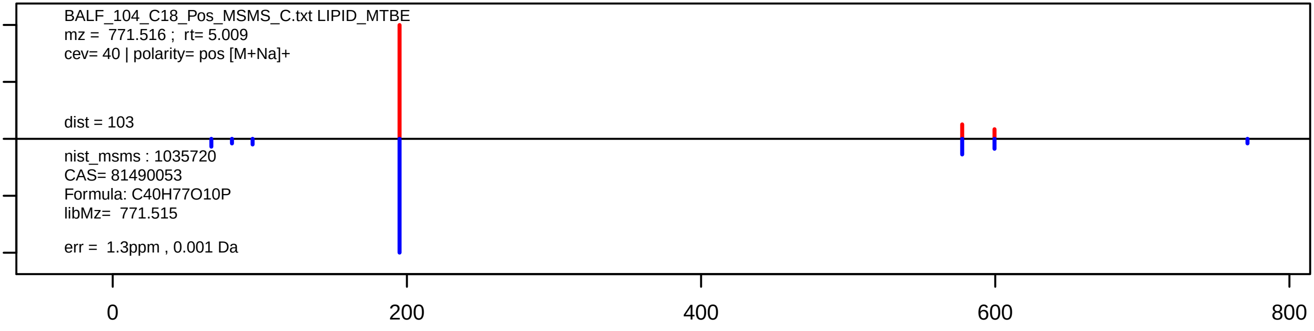

545 .   1−Palmitoyl−2−thiopalmitoyl phosphatidylcholine
Score=124 Dot=861 prob=95.4

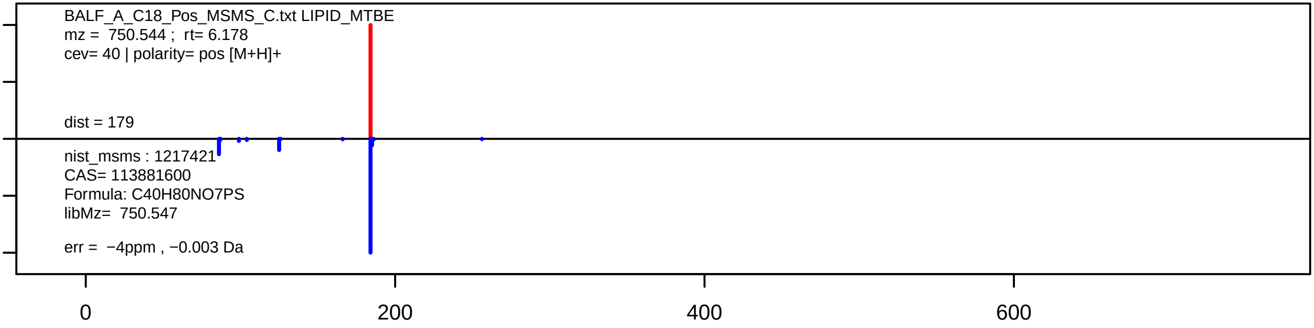

546 .   1−Palmitoyl−3−oleoyl−sn−glycero−2−phosphoethanolamine
Score=400 Dot=999 prob=9.4

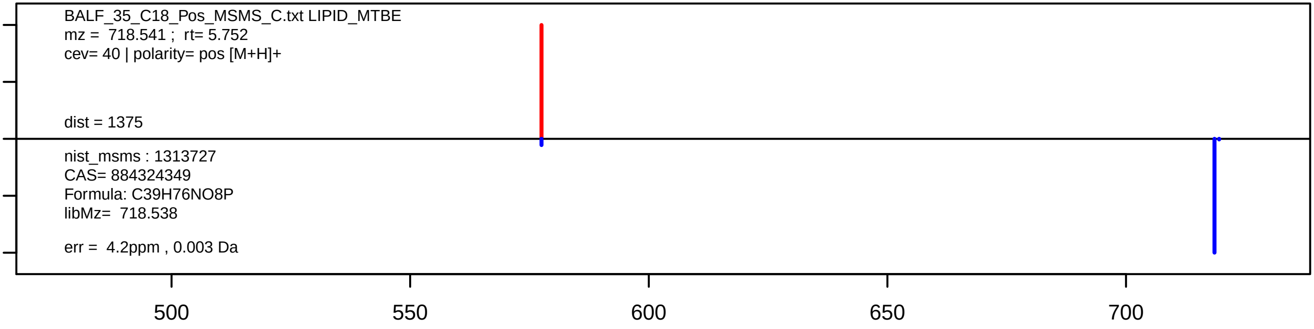

547 . 1−Palmitoyl−sn−glycero−3−phosphocholine
Score=514 Dot=857 prob=99

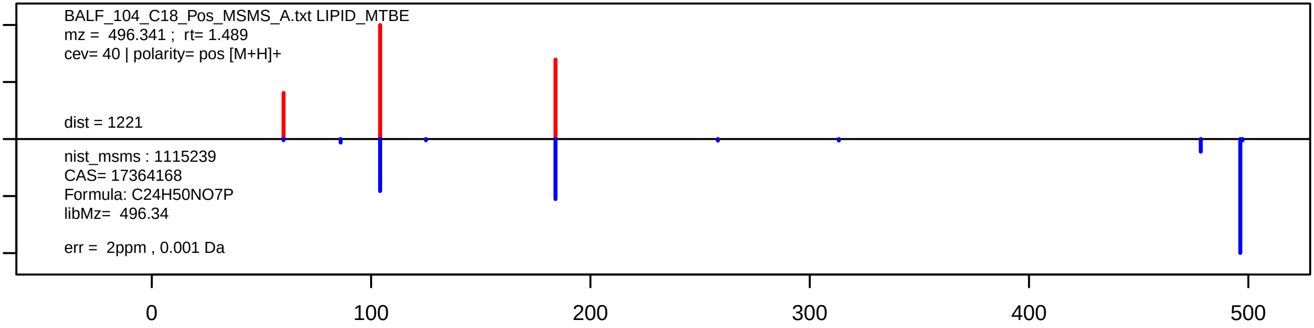

548 . 1−Pentadecanoyl−sn−glycero−3−phosphocholine
Score=856 Dot=953 prob=99

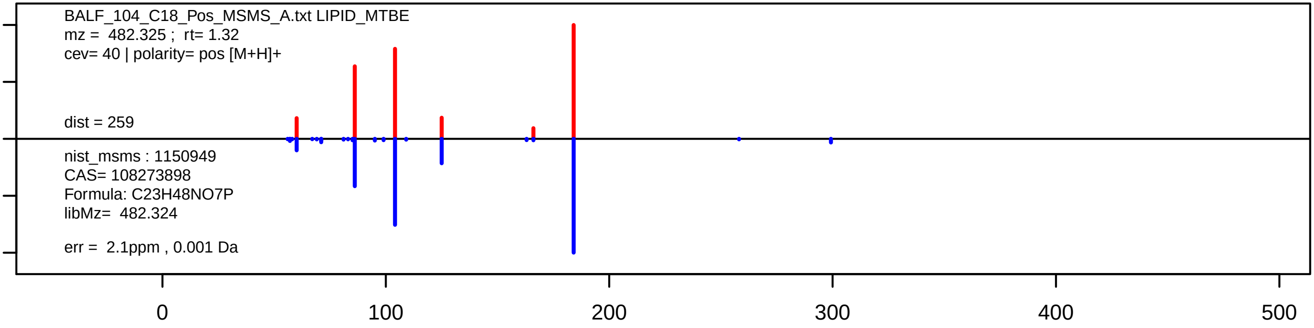

549 . 1−Propanol
Score=218 Dot=847 prob=95.2

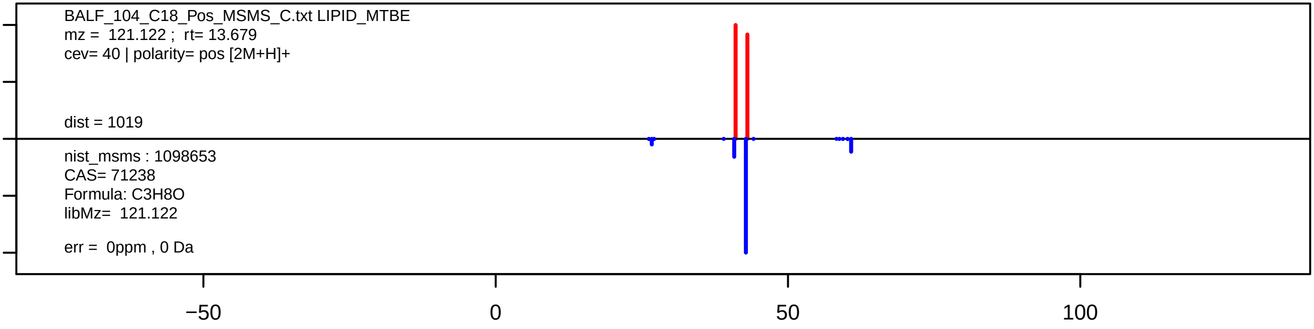

550 . 1−Stearoyl−2−arachidonyl−sn−glycero−3−phosphocholine
Score=246 Dot=956 prob=56

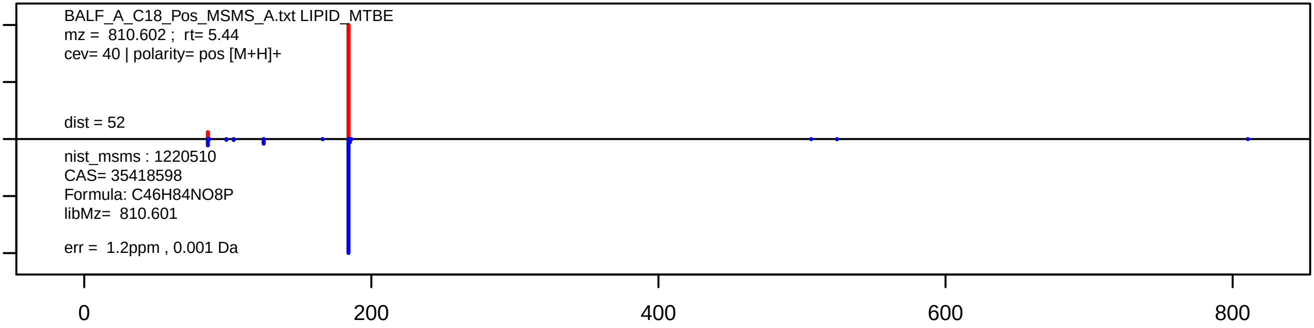

551 . 1−Stearoyl−2−hydroxy−sn−glycero−3−phosphocholine
Score=469 Dot=900 prob=98.9

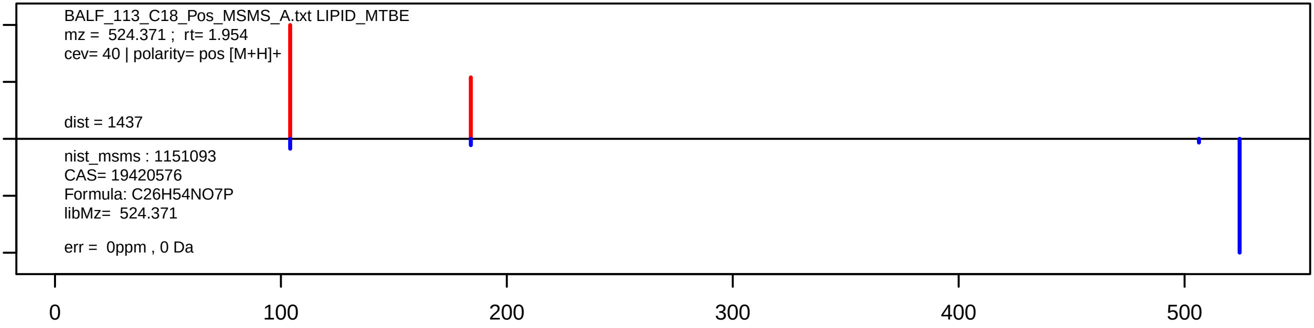

552 . 1−Stearoyl−2−hydroxy−sn−glycero−3−phosphoethanolamine
Score=708 Dot=853 prob=96.7

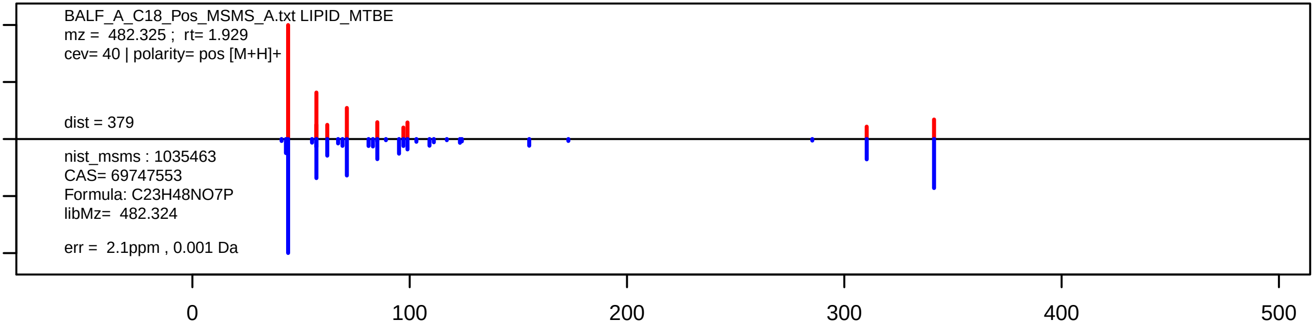

553 . 1−Stearoyl−2−linoleoyl−sn−glycero−3−phosphocholine
Score=388 Dot=967 prob=76.6

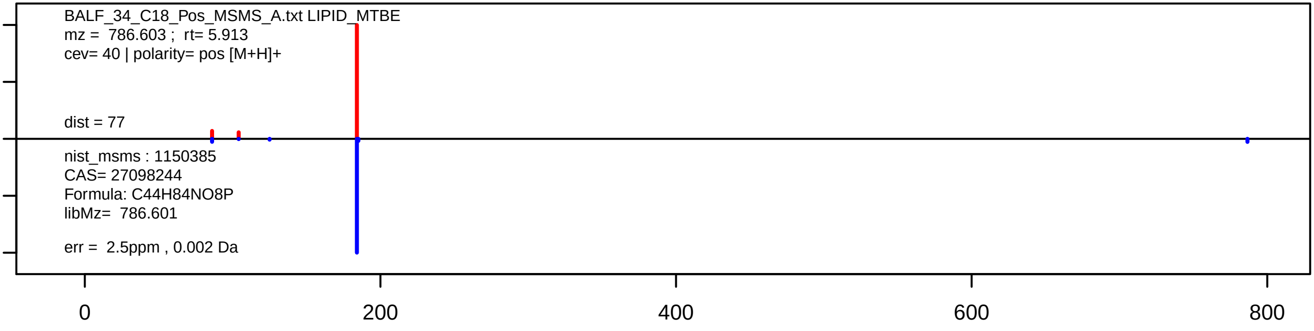

554 . 1−Stearoyl−2−linoleoyl−sn−glycerol
Score=396 Dot=804 prob=34.9

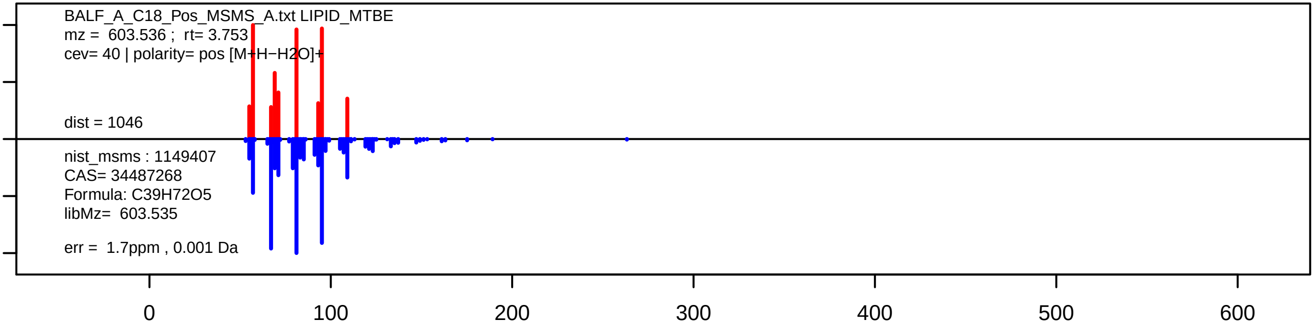

555 . 1−Stearoyl−2−oleoyl−sn−glycero−3−phosphoethanolamine
Score=400 Dot=999 prob=10.4

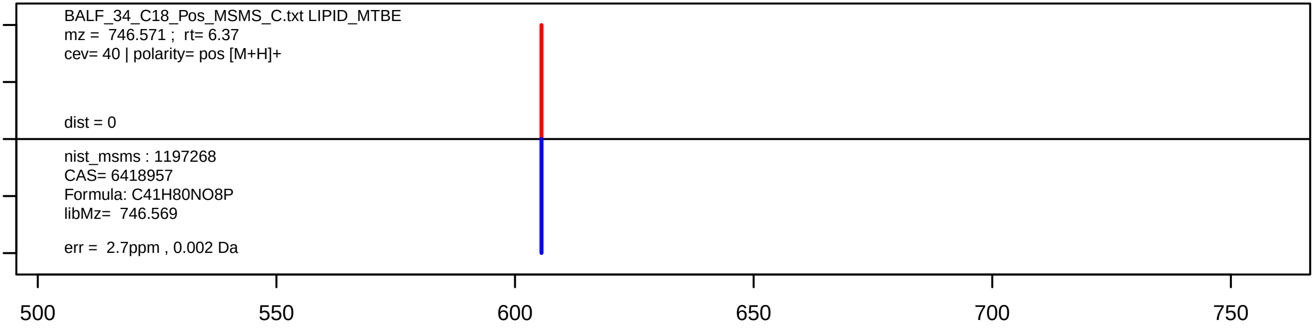

556 . 1,2−Benzenedicarboxylic acid
Score=511 Dot=976 prob=98.4

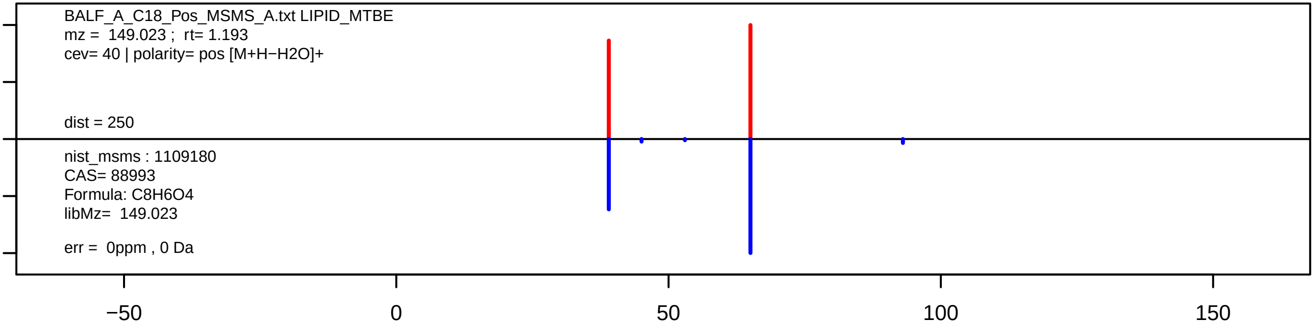

557 . 1,2−Diheptadecanoyl−sn−glycero−3−phosphocholine
Score=347 Dot=972 prob=97.4

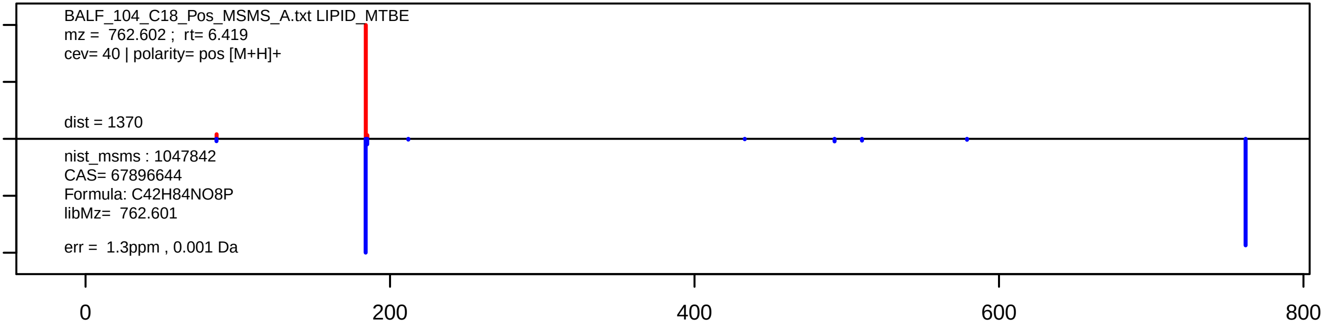

558 . 1,2−Dihexadecanoyl−sn−glycero−3−phosphocholine
Score=368 Dot=993 prob=92.4

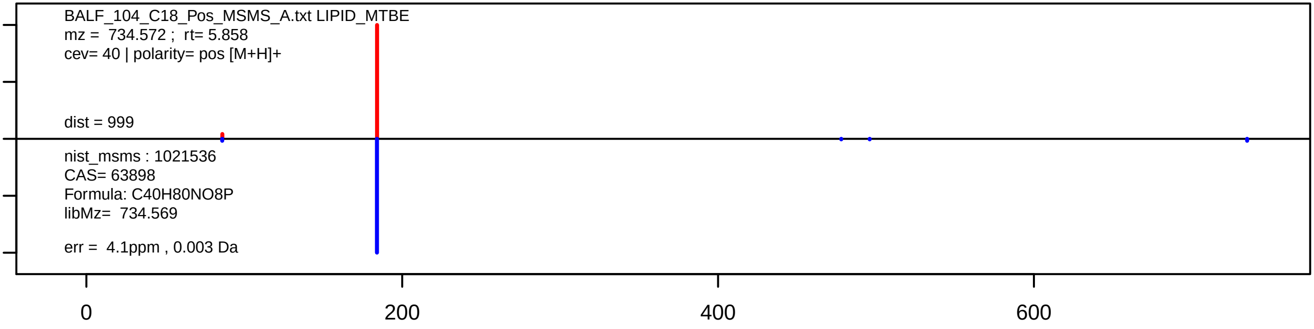

559 .   1,2−Dilinoleoyl−sn−glycero−3−phospho−(1'−rac−glycerol)
Score=232 Dot=807 prob=82.7

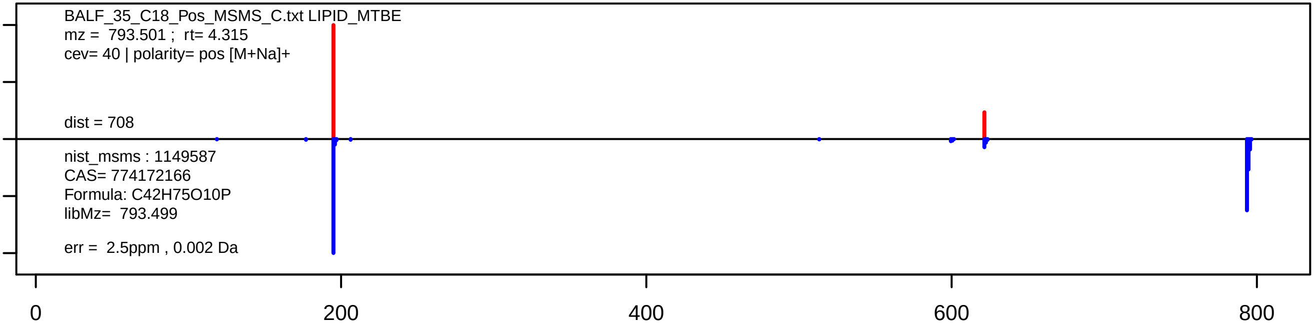

560 .   1,2−Dilinoleoyl−sn−glycero−3−phosphocholine
Score=589 Dot=945 prob=99

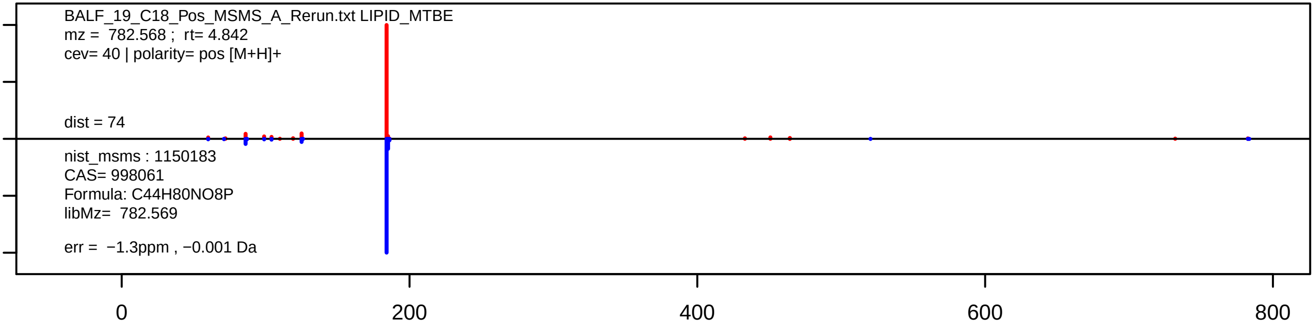

561 .   1,2−Dioleoyl−3−trimethylammoniumpropane cation
Score=803 Dot=927 prob=97.2

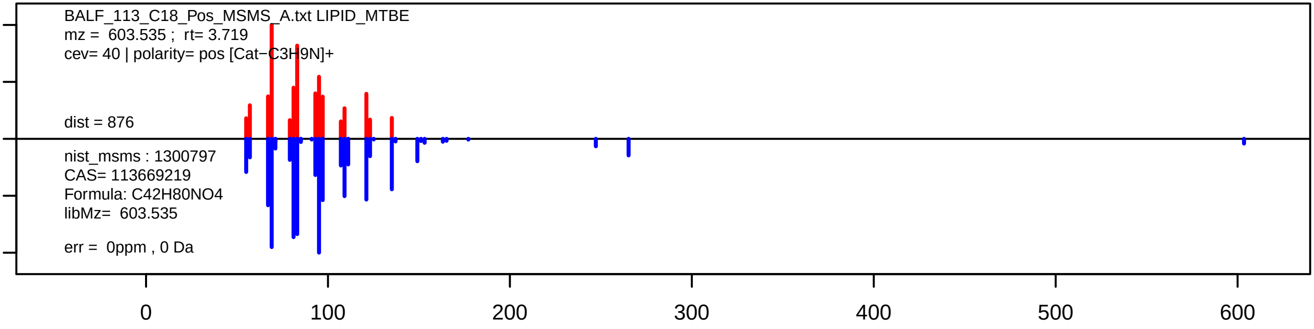

562 . 1,2−dioleoyl−sn−glycero−3−phosphatidylcholine
Score=400 Dot=999 prob=50

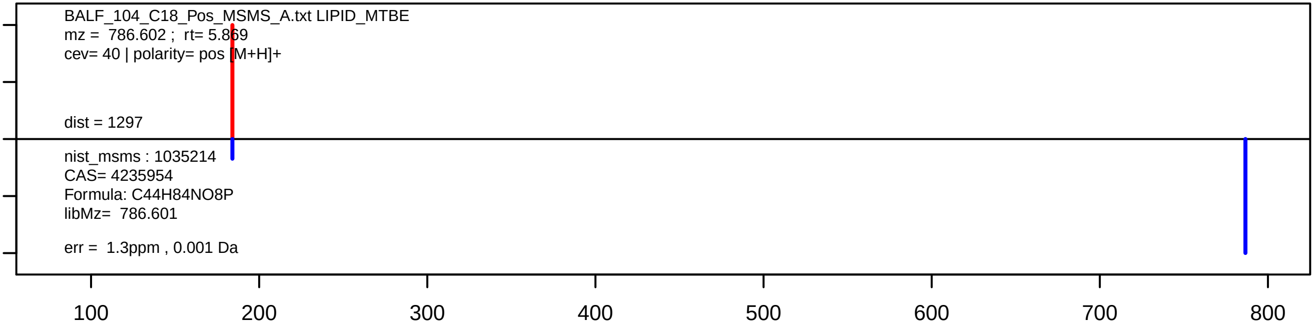

563 . 1,2−Dioleoyl−sn−glycero−3−phosphoethanolamine
Score=541 Dot=802 prob=95

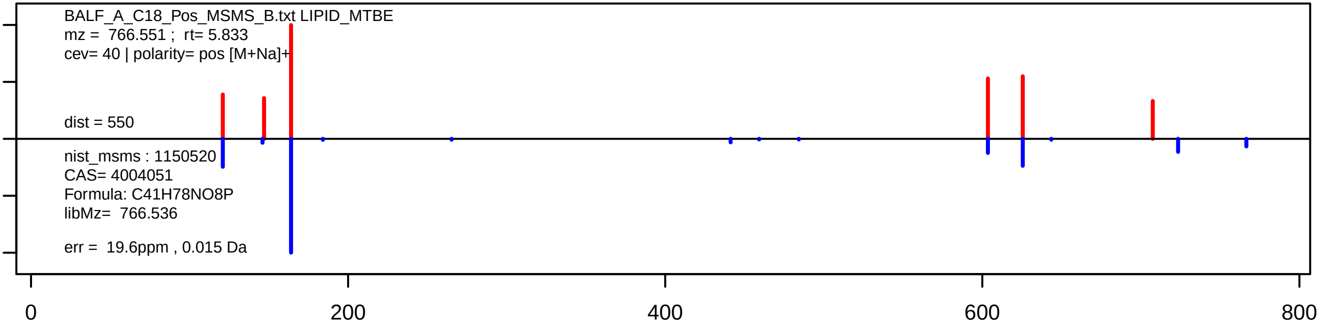

564 . 1,2−Dipalmitoleoyl−sn−glycero−3−phosphocholine
Score=808 Dot=883 prob=99

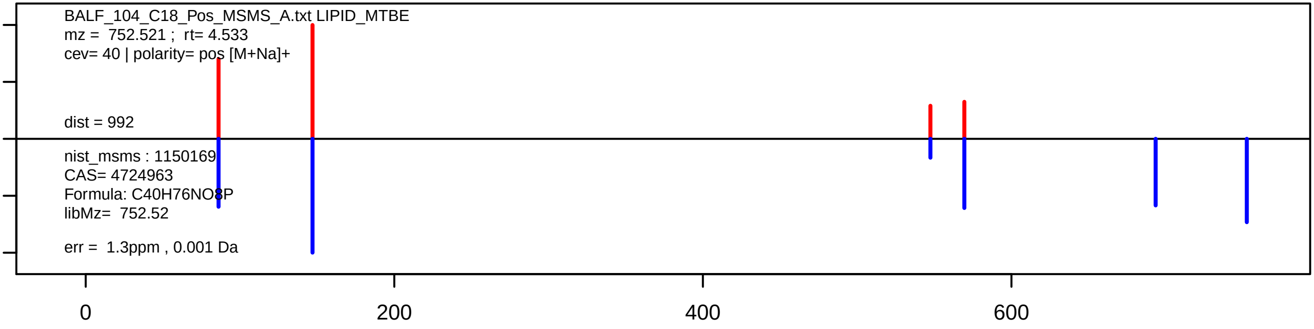

565 . 1,2−Dipalmitoyl−sn−glycero−3−phosphoethanolamine−N−methyl
Score=172 Dot=884 prob=40.4

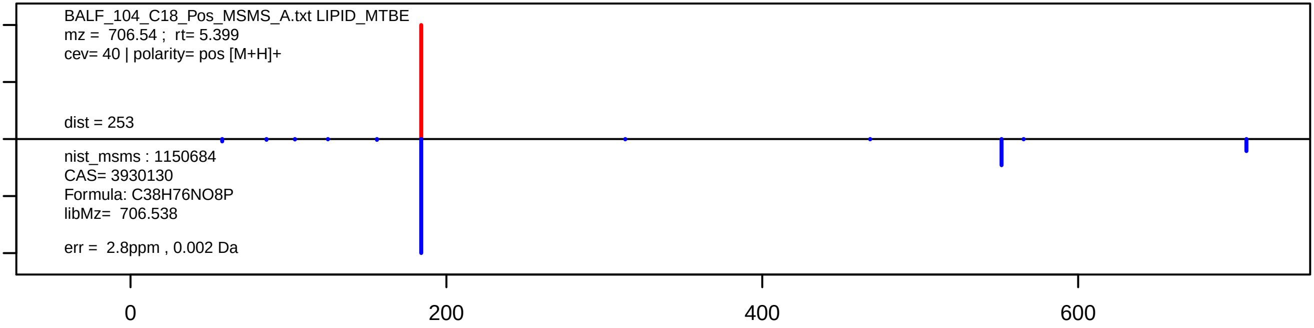

566 . 1,2−Dipalmitoyl−sn−glycero−O−ethyl−3−phosphatidylcholine cation
Score=793 Dot=981 prob=98.8

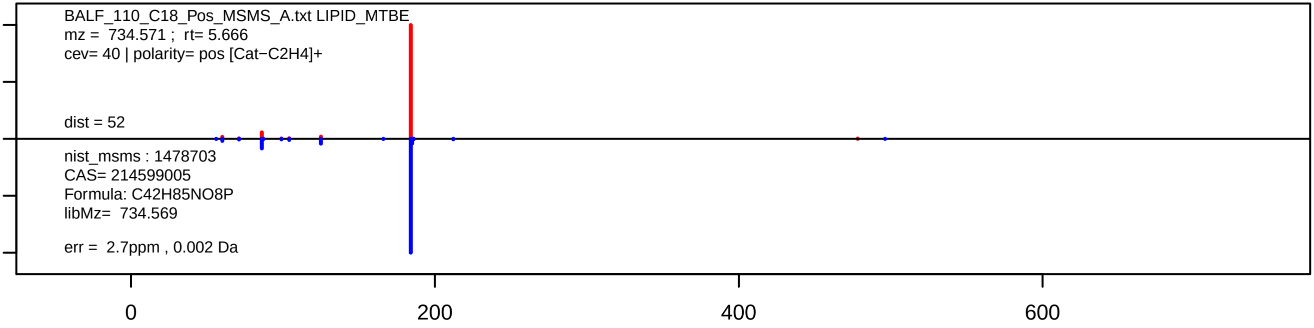

567 . 1,2−Dipentadecanoyl−sn−glycero−3−phosphocholine
Score=646 Dot=974 prob=88.2

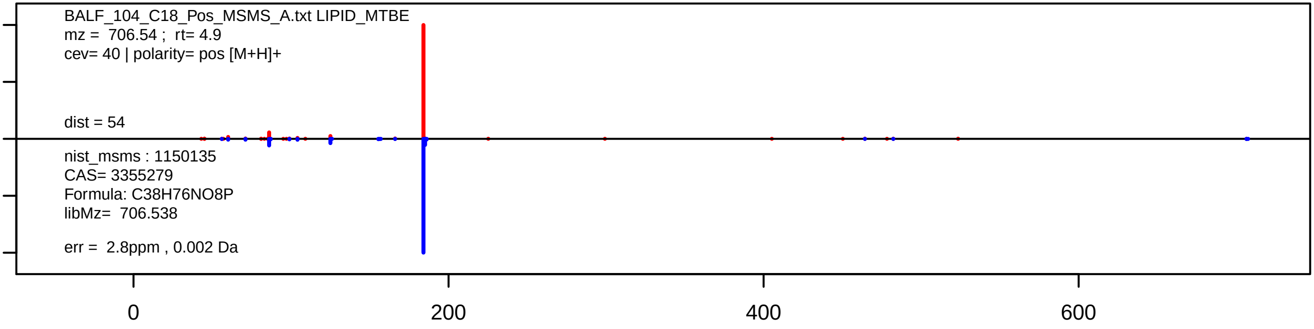

568 . 1,2−Ditetradecanoyl−sn−glycero−3−phosphocholine
Score=191 Dot=954 prob=60

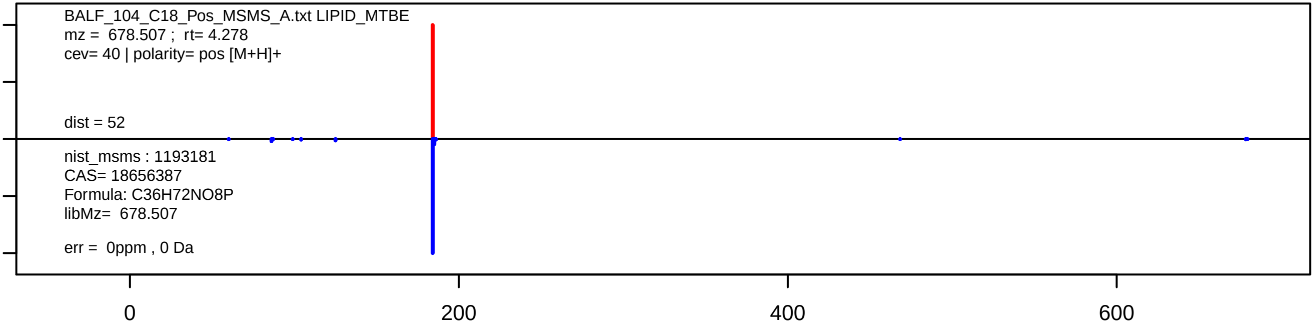

569 . 1,2,3−Trihexadecanoyl−sn−glycerol
Score=482 Dot=989 prob=98.9

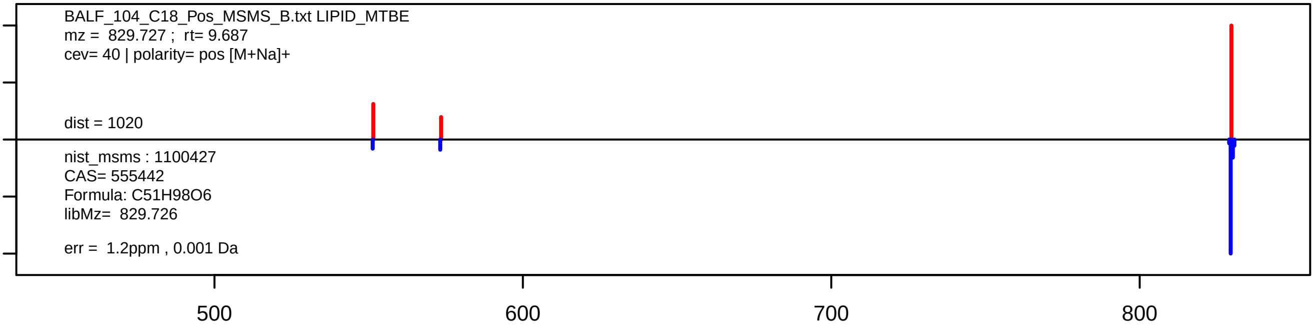

570 . 1,2,4−Triazin−5−amine, N−([1,1'−biphenyl]−4−ylmethyl)−6−phenyl−3−(2−pyridinyl)−
Score=562 Dot=874 prob=48

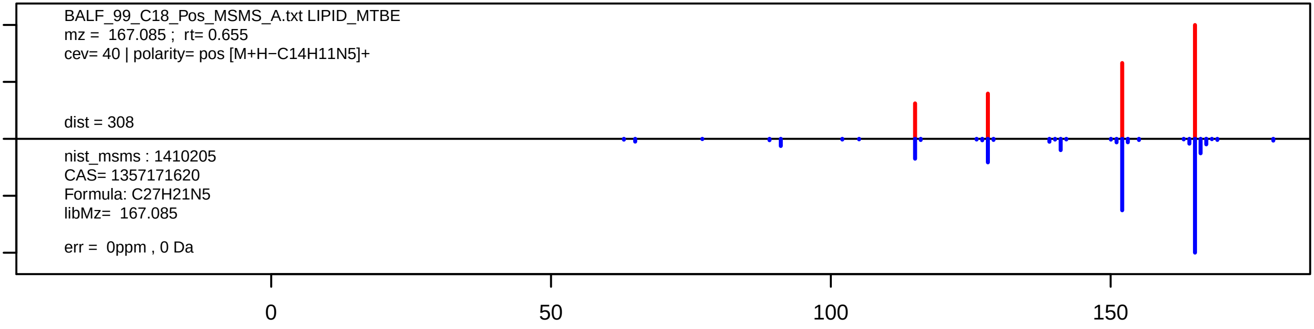

571 . 13−Hydroxy−9Z,11E−octadecadienoic acid, cholesteryl ester
Score=407 Dot=878 prob=65.8

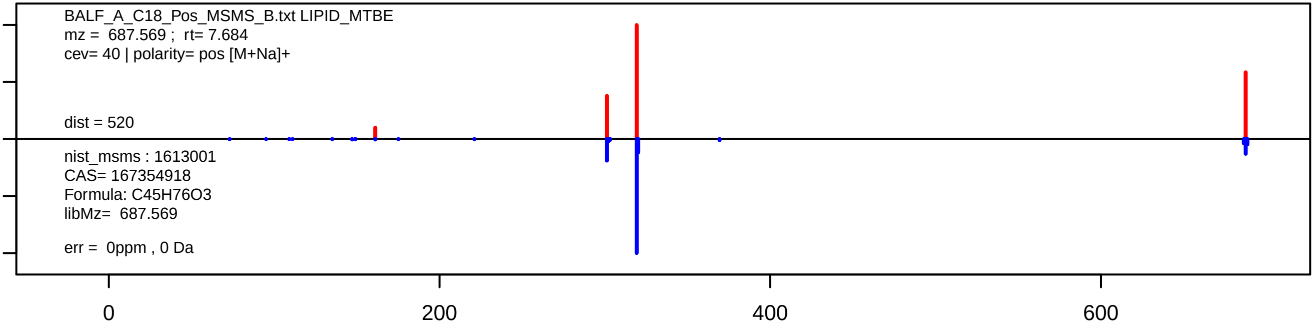

572 . 2−(2H−Benzotriazol−2−yl)−4,6−bis(1−methyl−1−phenylethyl)phenol
Score=926 Dot=964 prob=94.6

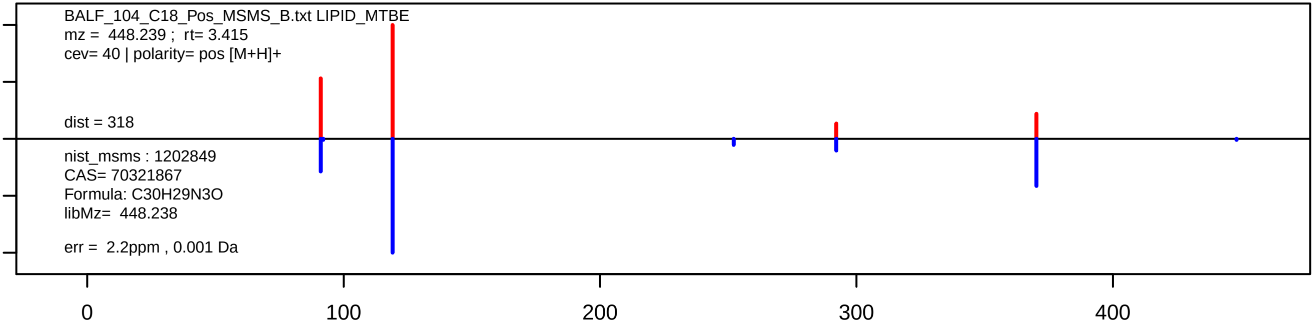

573 . 2−[(2,6−Dimethylphenyl)amino]−N,N,N−triethyl−2−oxoethanaminium cation
Score=559 Dot=999 prob=52.6

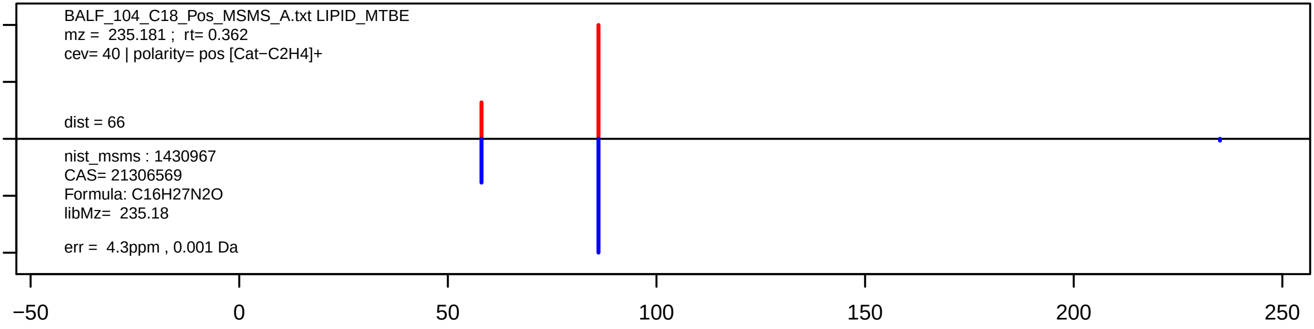

574 .   2−Amino−1−phenylbutane
Score=541 Dot=985 prob=28

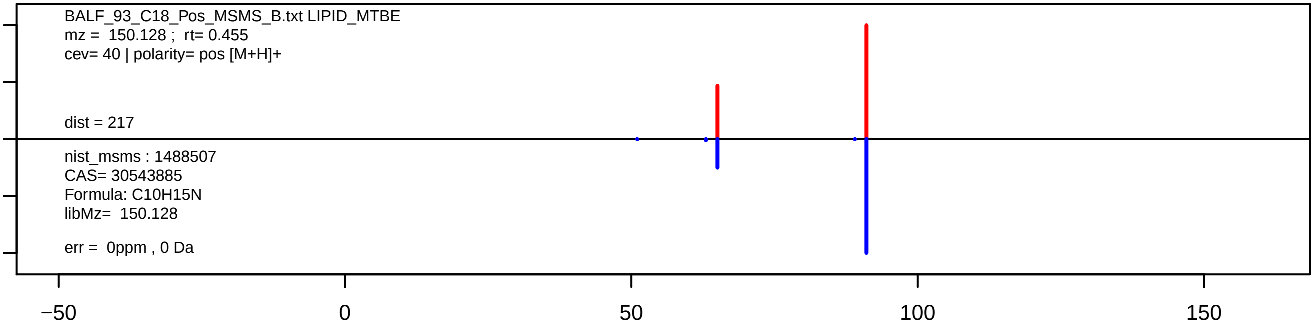

575 .   2−Ethylcyclohexanol
Score=370 Dot=909 prob=63.8

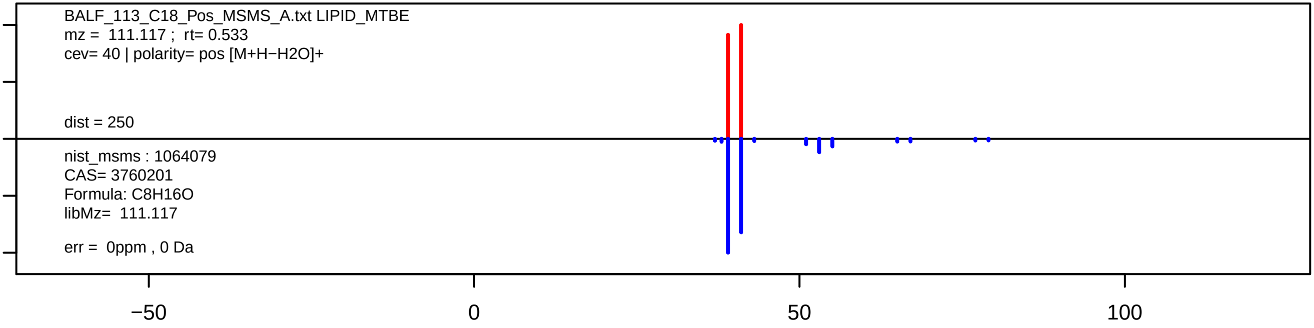

576 .   2−Hexadecanoylthio−1−ethylphosphorylcholine
Score=399 Dot=999 prob=97

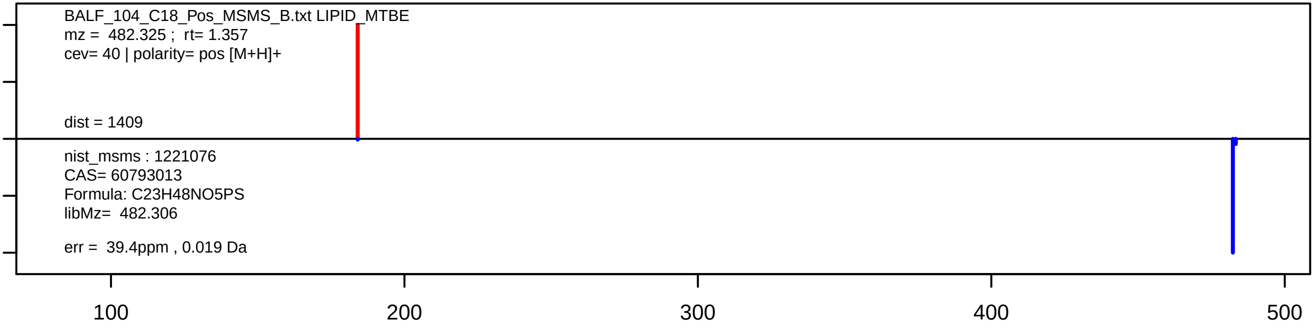

577 . 2−Oleoyl−1−palmitoyl−sn−glycero−3−phosphocholine
Score=422 Dot=984 prob=84.7

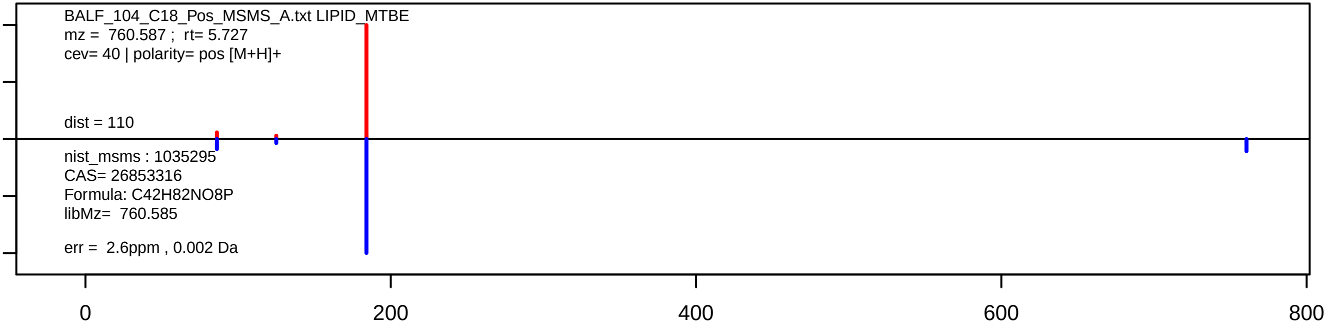

578 . 2−Oleoyl−1−stearoyl−sn−glycero−3−phosphoserine
Score=228 Dot=802 prob=48

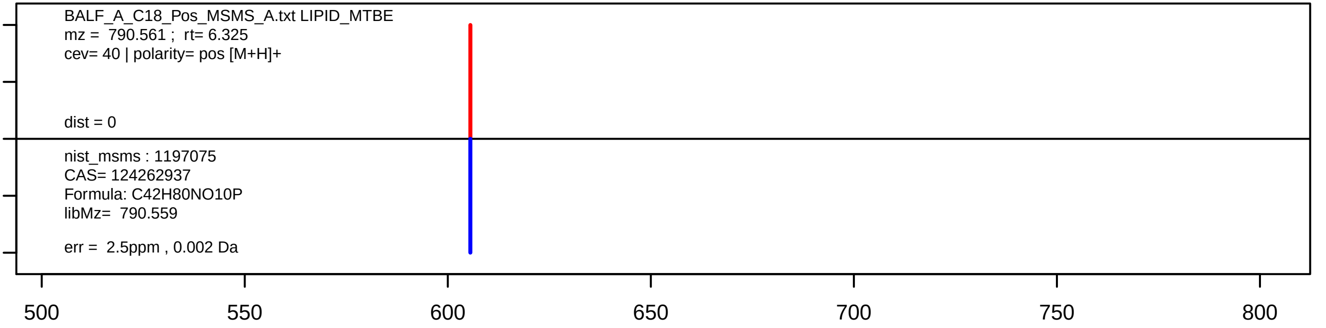

579 . 3−Aminopentanoic acid
Score=170 Dot=809 prob=43.9

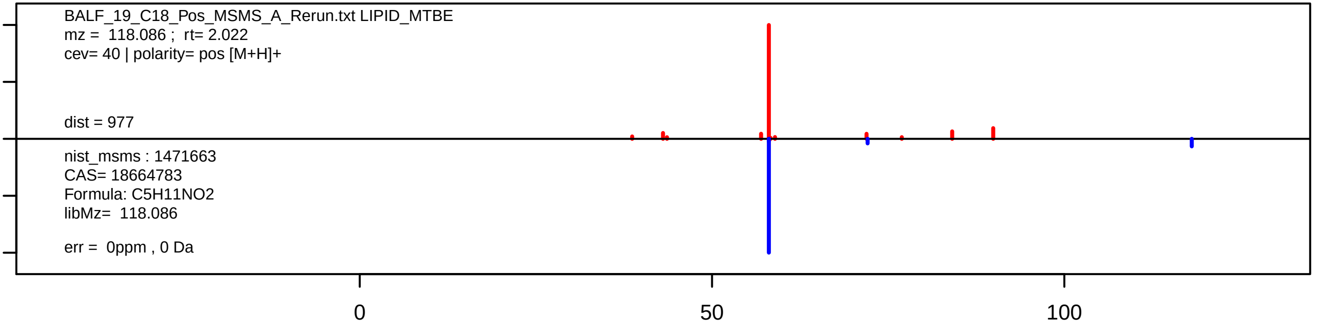

580 . 4−Methoxy−N,N−dimethyltryptamine
Score=280 Dot=847 prob=89.8

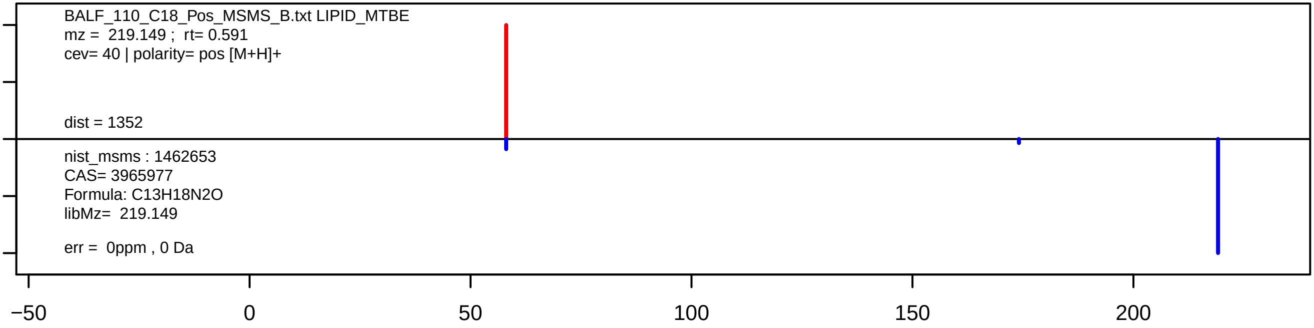

581 . 4−Pregnen−17.alpha., 20.alpha.−diol−3−one
Score=653 Dot=848 prob=73.6

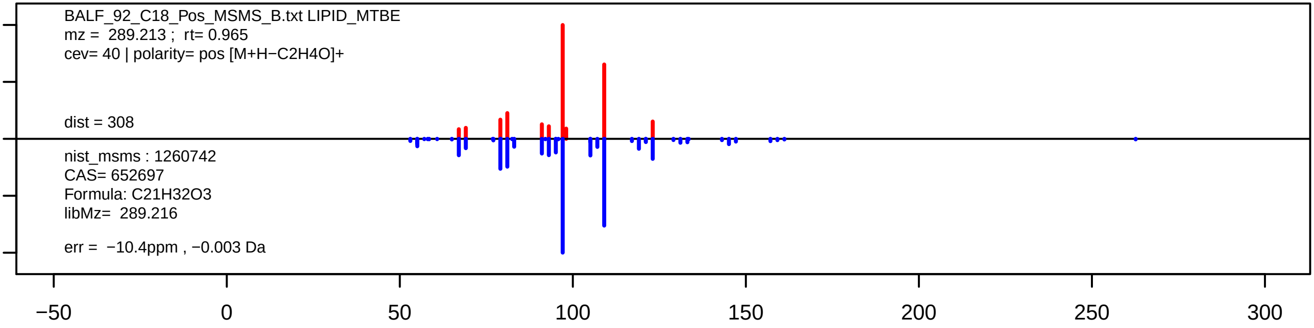

582 . 7−Oxocholesterol
Score=861 Dot=856 prob=97.8

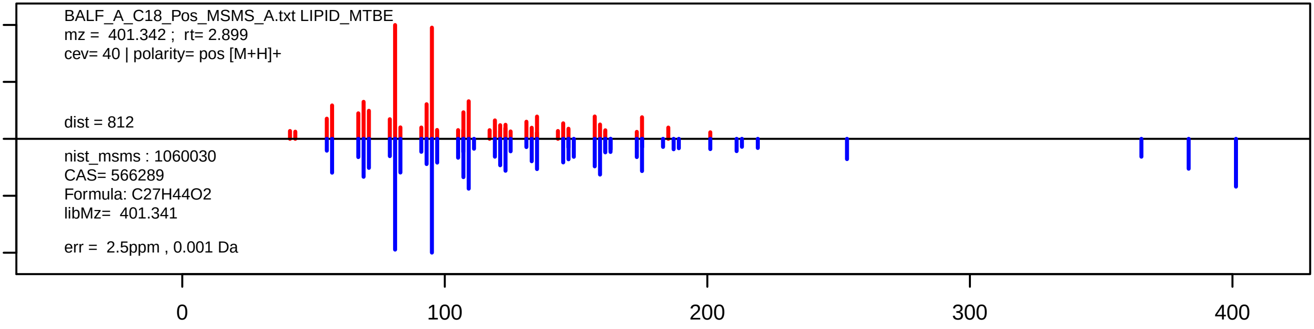

583 . 9−Hydroxy−10E,12Z−octadecadienoic acid, cholesteryl ester
Score=423 Dot=922 prob=87

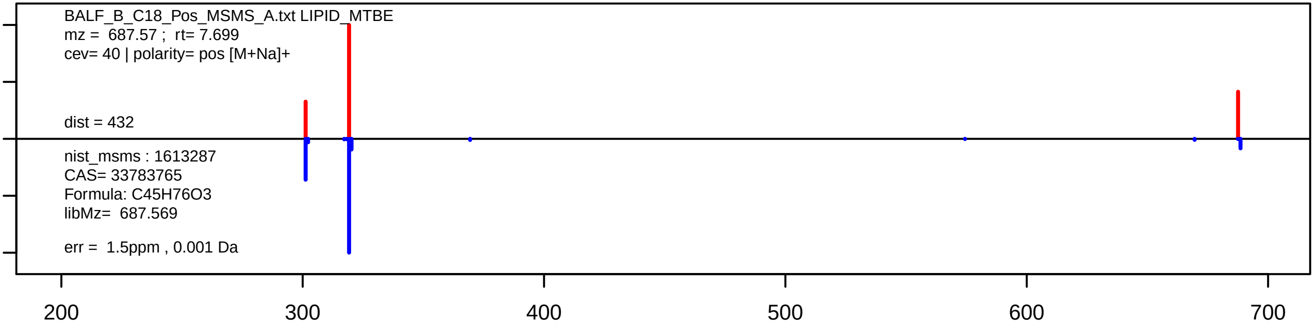

584 . Aminodiphenylmethane
Score=872 Dot=928 prob=27.6

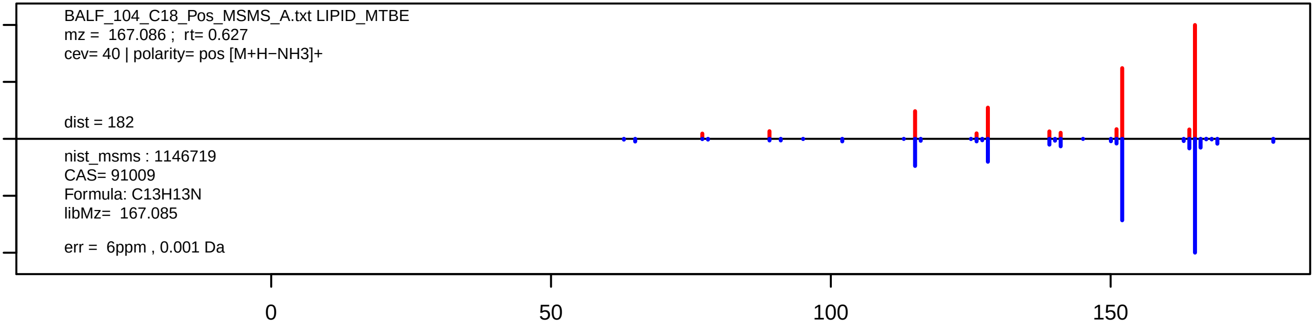

585 . Arachidonoylthiophosphorylcholine
Score=407 Dot=988 prob=99

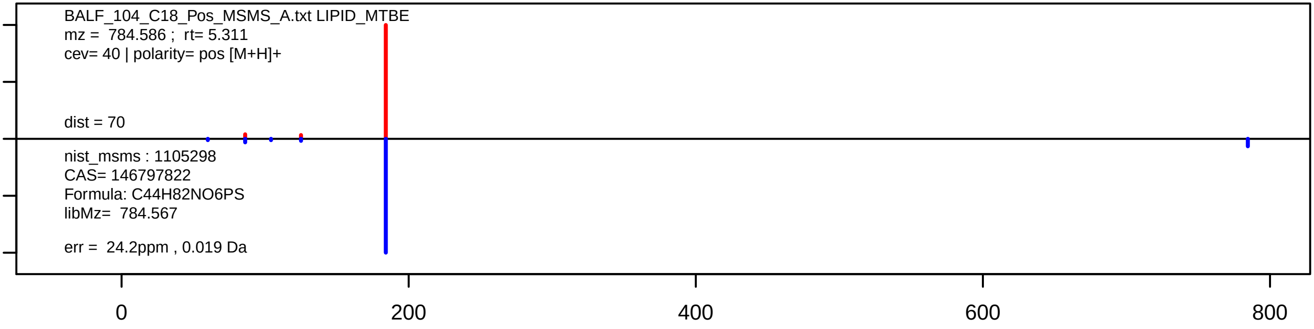

586 . Azithromycin
Score=716 Dot=863 prob=99

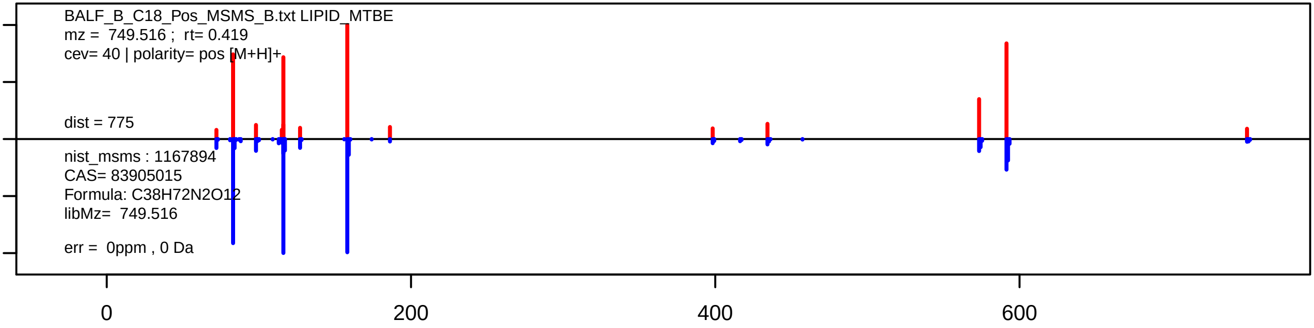

587 . Benzhydrol
Score=855 Dot=928 prob=39.7

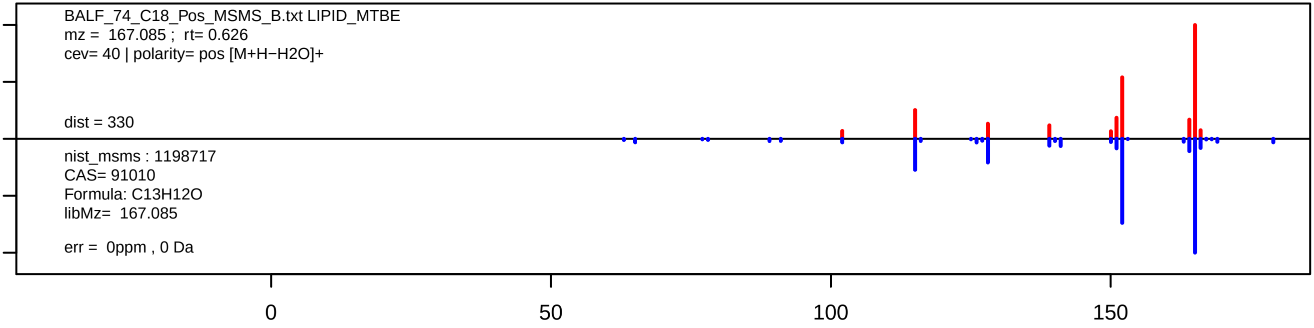

588 . Benzyl alcohol
Score=713 Dot=873 prob=98.6

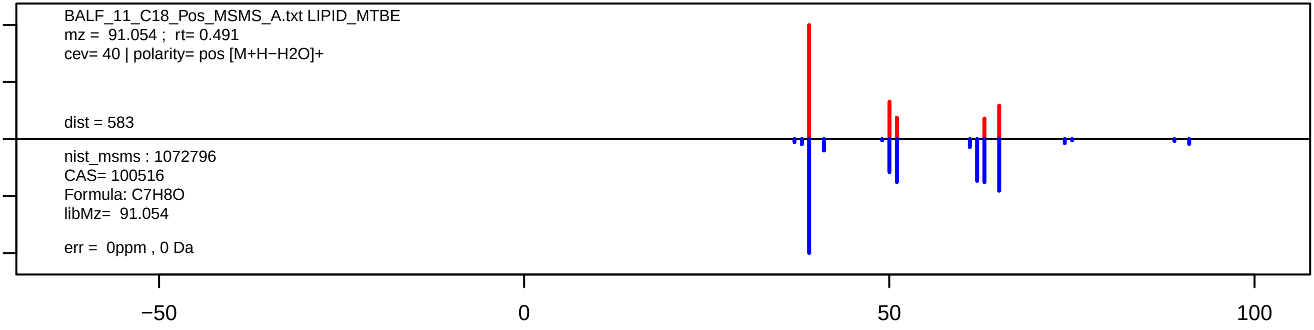

589 . Benzyldimethylstearylammonium cation
Score=475 Dot=945 prob=94.1

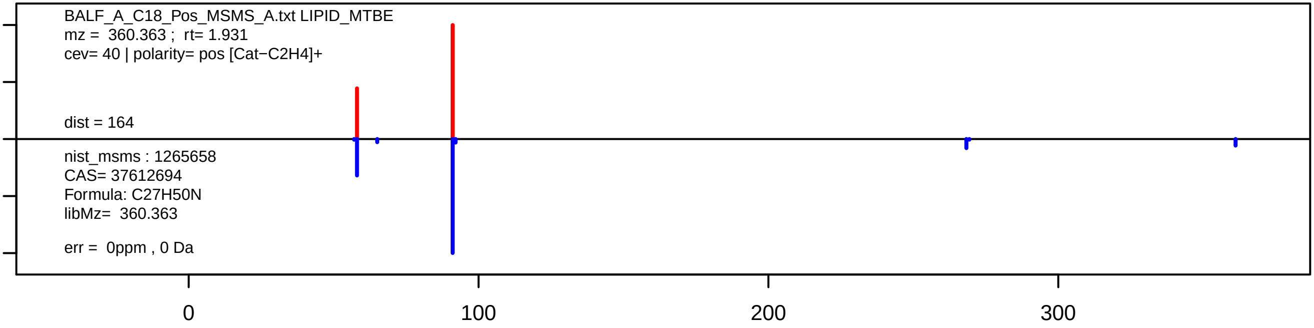

590 . Benzyldimethyltetradecylammonium cation
Score=476 Dot=948 prob=98.9

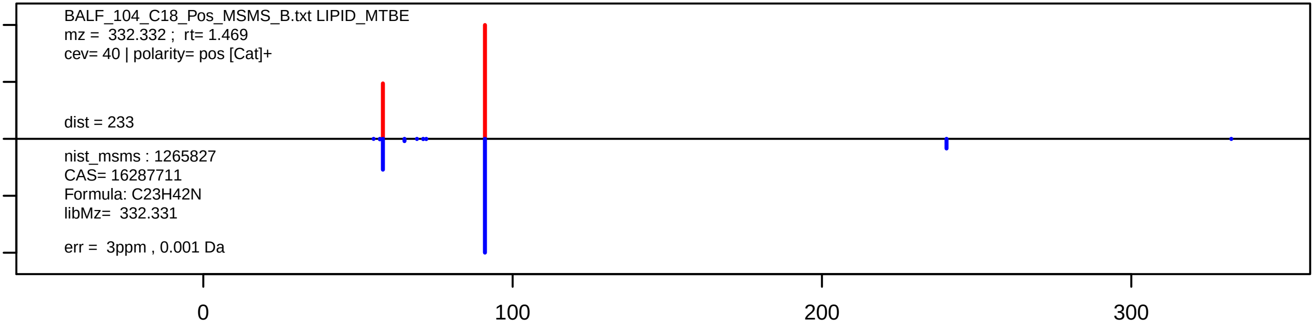

591 . Benzyldodecyldimethylammonium cation
Score=691 Dot=952 prob=98.8

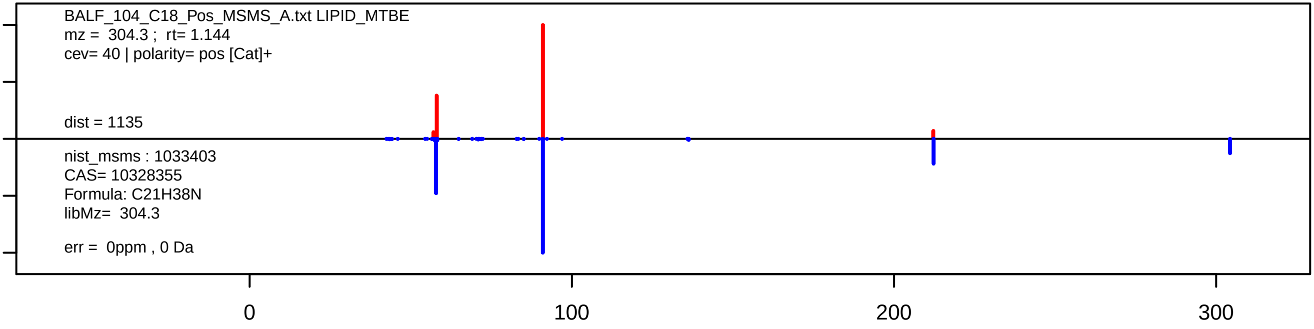

592 . Betaine
Score=341 Dot=883 prob=46.7

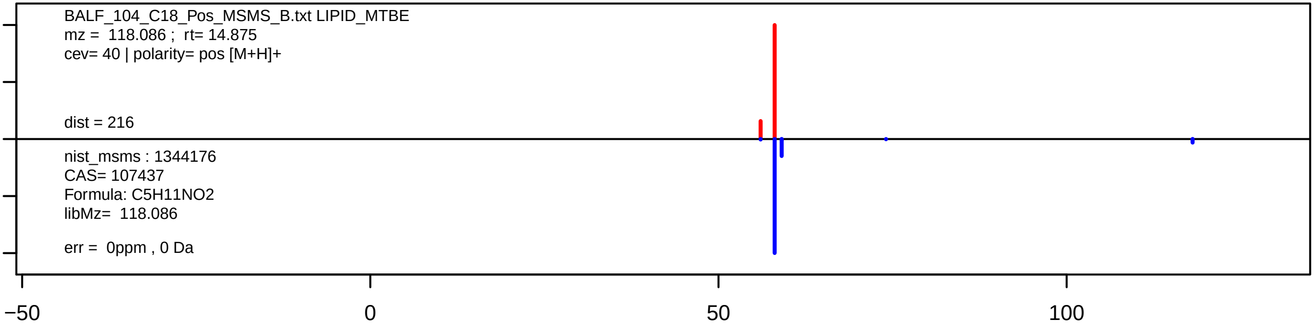

593 . Bis(2−ethylhexyl) adipate
Score=737 Dot=895 prob=98.9

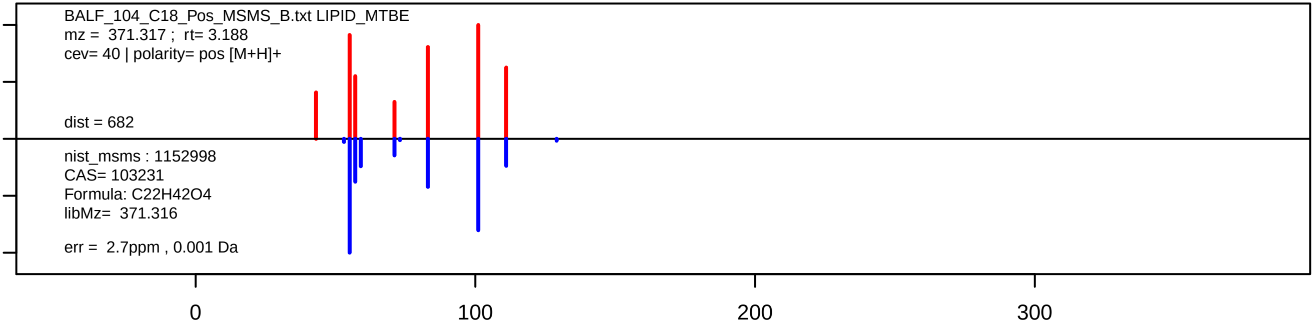

594 . Bupropion
Score=929 Dot=967 prob=98.6

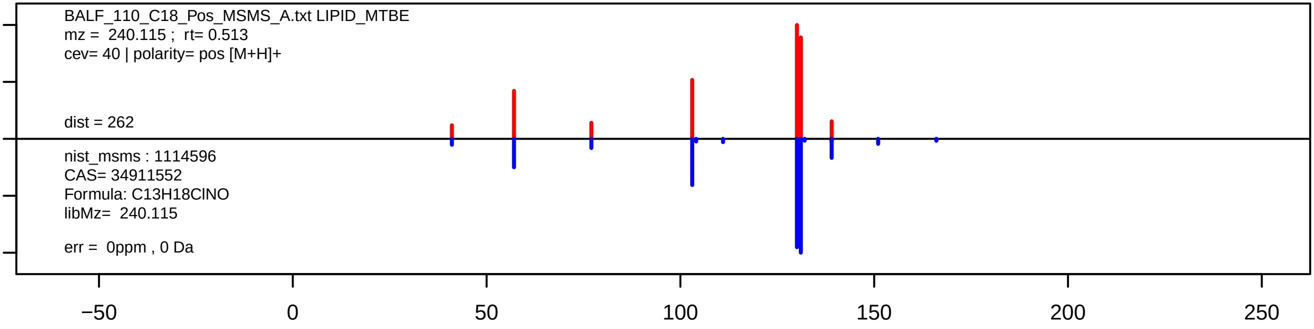

595 . Choline cation
Score=274 Dot=827 prob=28.4

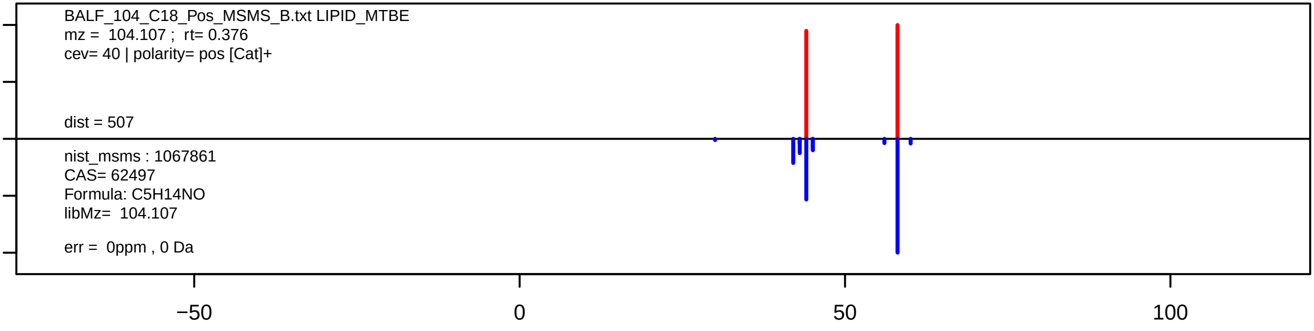

596 . Citalopram
Score=698 Dot=874 prob=96.6

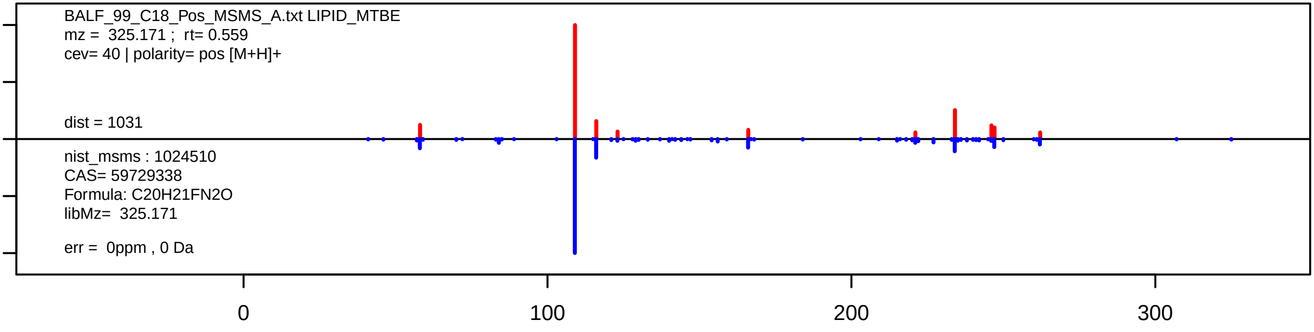

597 . Cyclizine
Score=887 Dot=949 prob=42.8

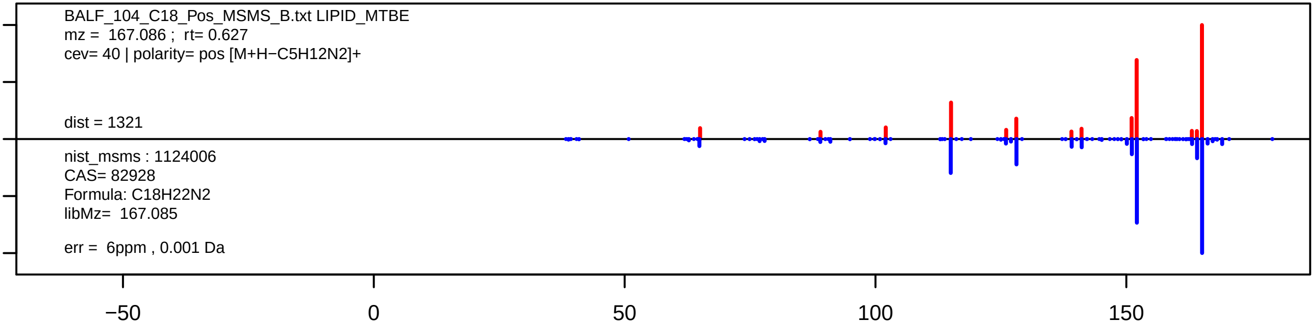

598 . D−erythro−N−stearoylsphingosine
Score=715 Dot=830 prob=98.9

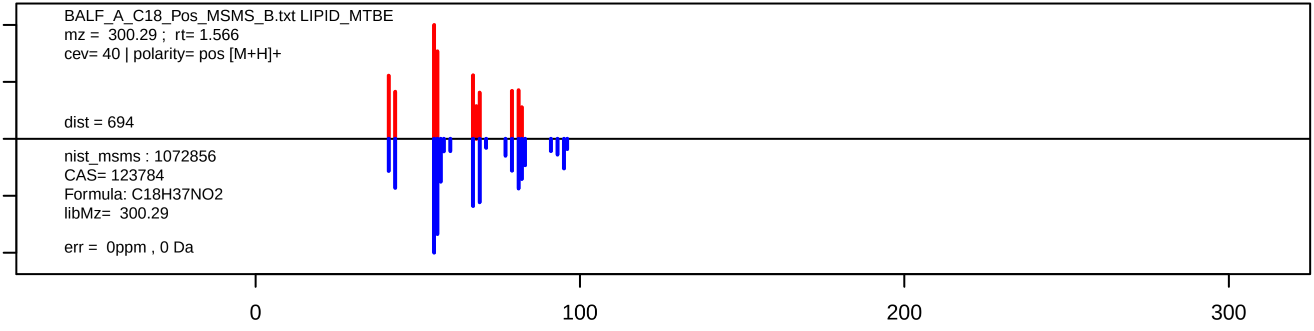

599 . D−erythro−Sphinganine
Score=533 Dot=807 prob=97.5

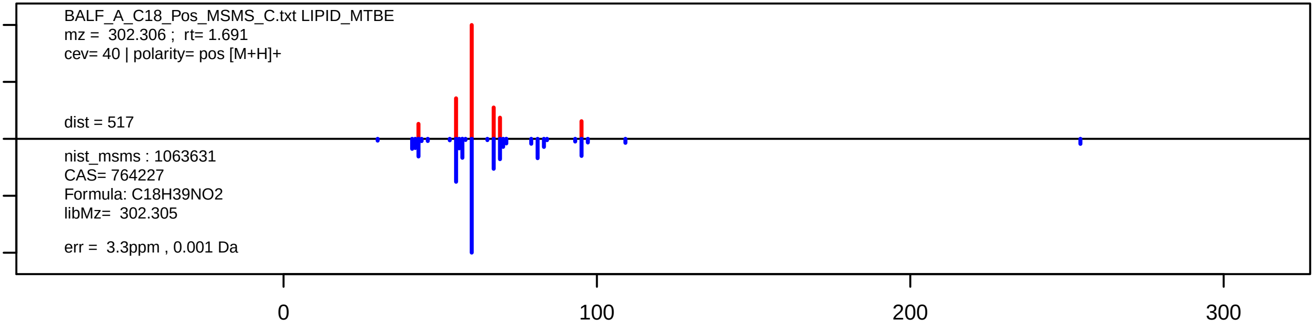

600 . Decamethylcyclopentasiloxane
Score=135 Dot=834 prob=94

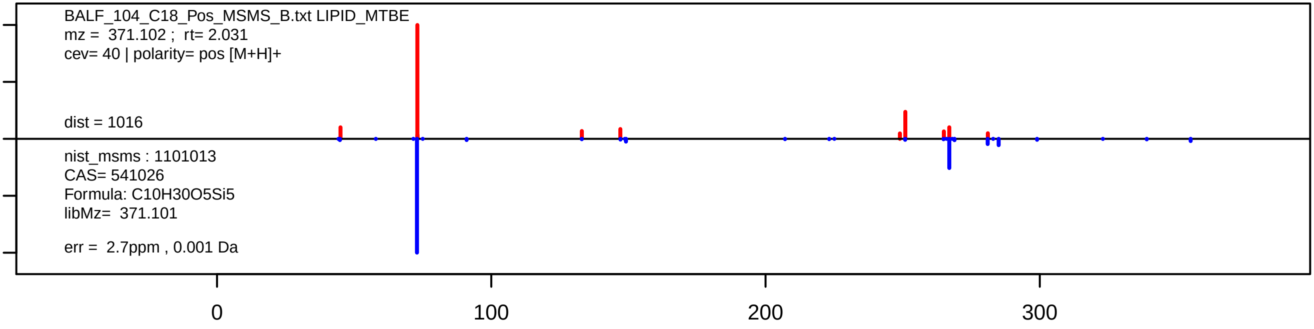

601 . Decanoyl−L−carnitine
Score=331 Dot=949 prob=98.6

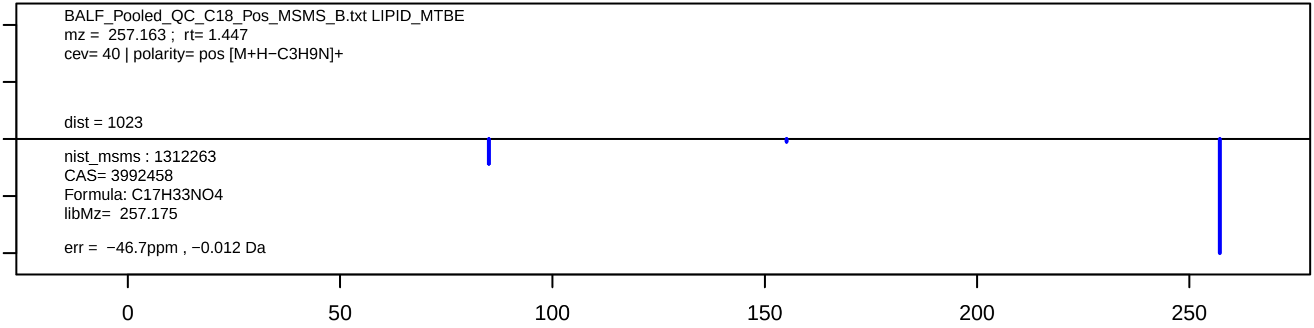

602 . Dibenzylamine
Score=458 Dot=957 prob=97

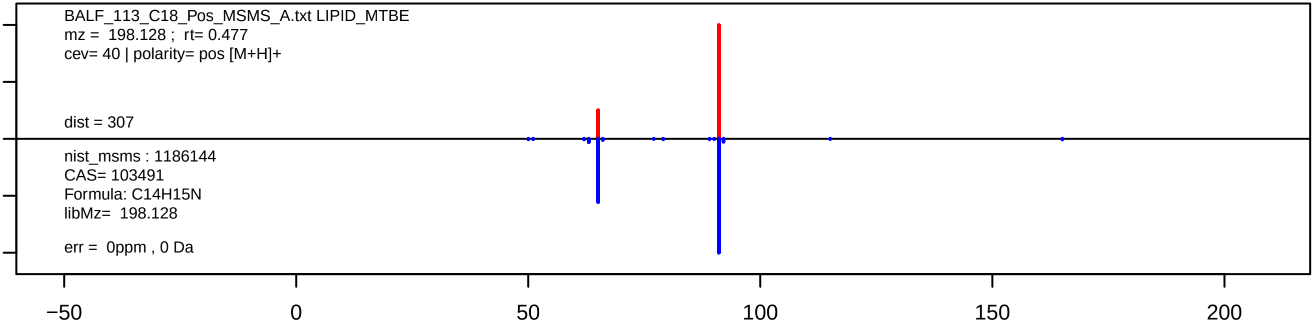

603 . Dibutyl phthalate
Score=282 Dot=979 prob=81

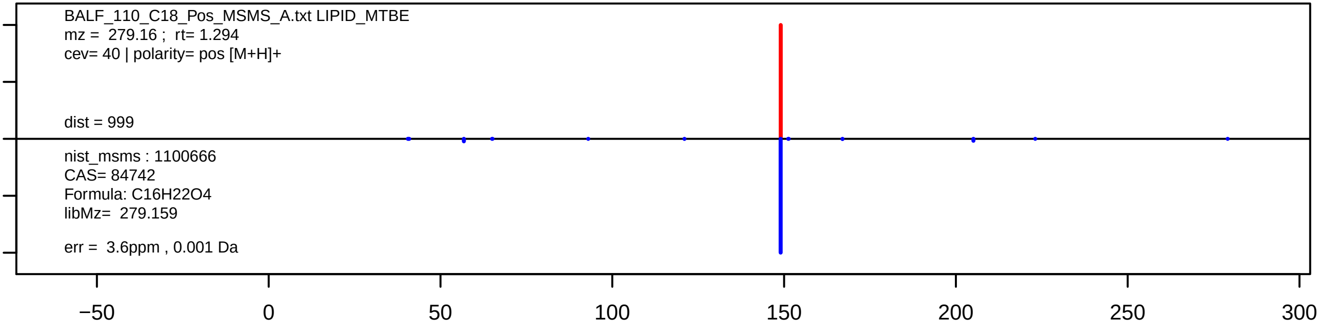

604 .   Dicyclohexylamine
Score=254 Dot=927 prob=92

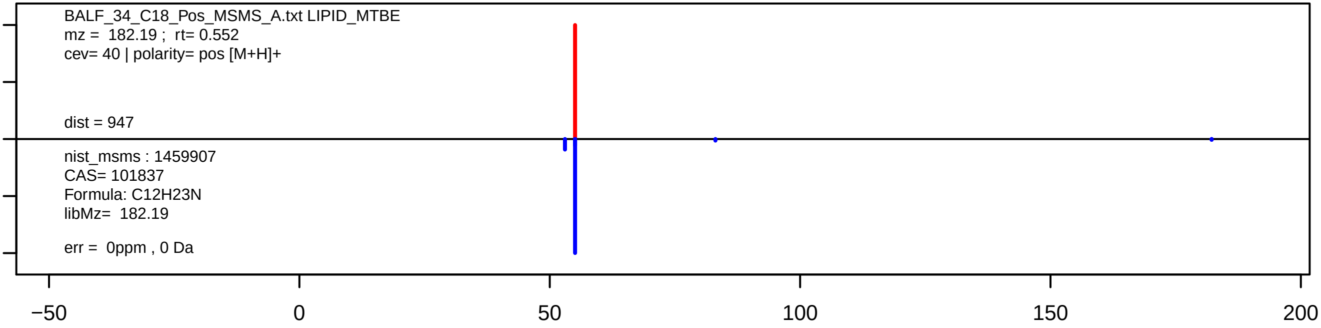

605 .   Dihexadecyldimethylammonium cation
Score=282 Dot=805 prob=98.5

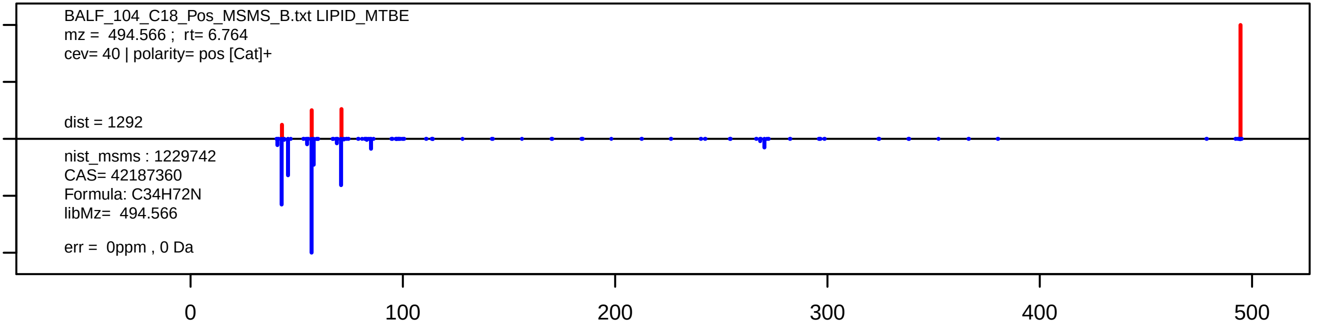

606 .   Diisooctyl phthalate
Score=302 Dot=985 prob=97.4

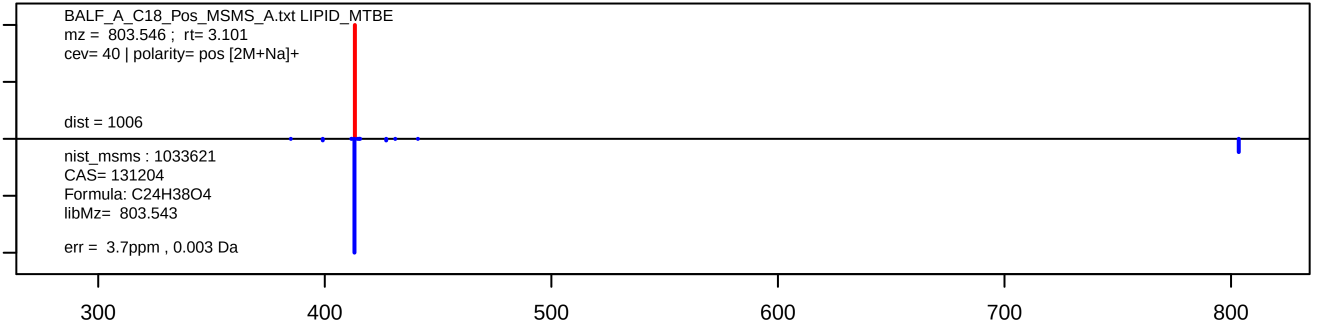

607 . Diltiazem
Score=400 Dot=999 prob=97.3

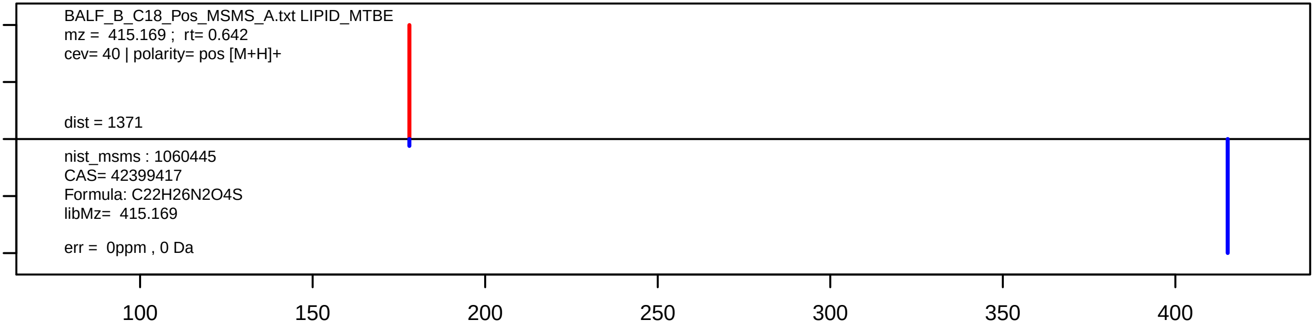

608 . Dimethyldioctadecylammonium cation
Score=195 Dot=886 prob=90.2

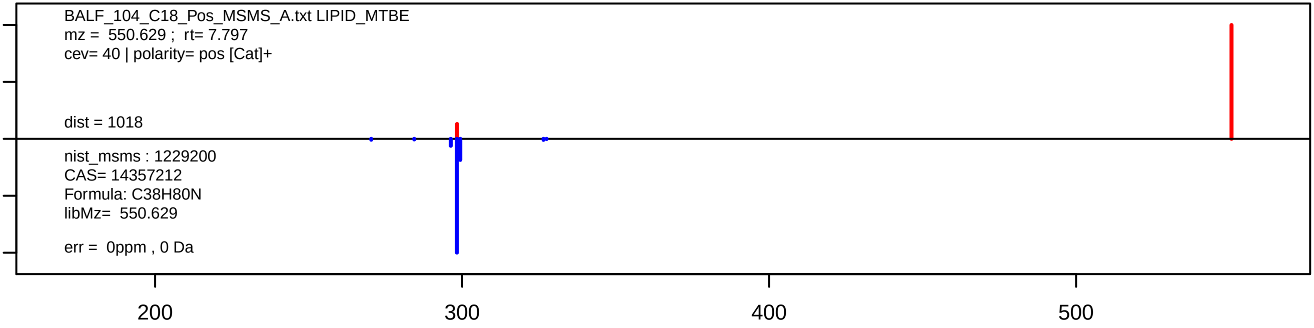

609 . Dioctyl phthalate
Score=241 Dot=906 prob=81.3

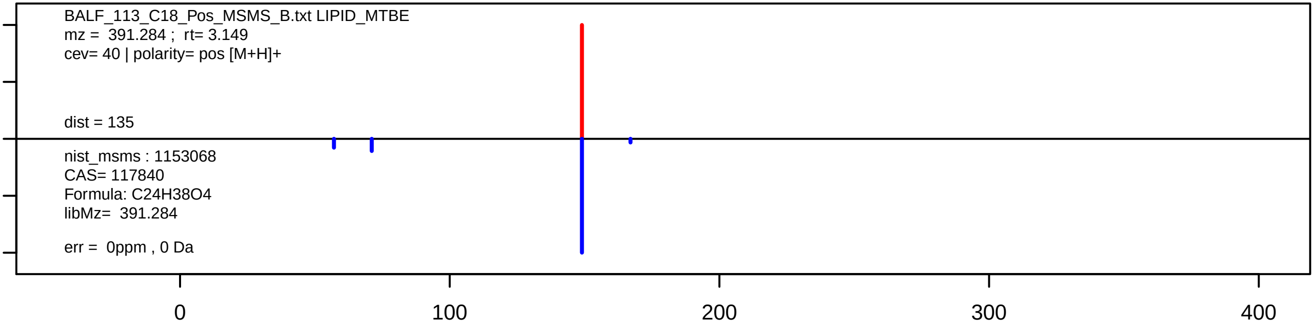

610 . Diphenhydramine
Score=960 Dot=973 prob=97.7

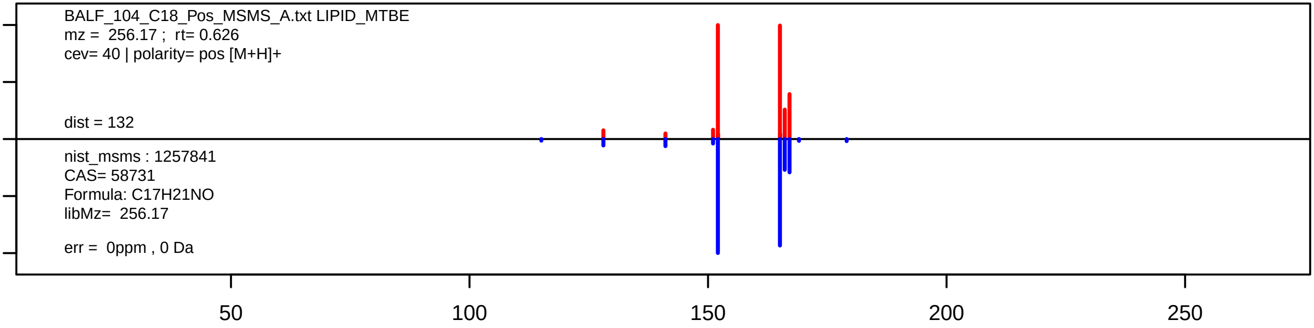

611 . Erucamide
Score=720 Dot=855 prob=91.7

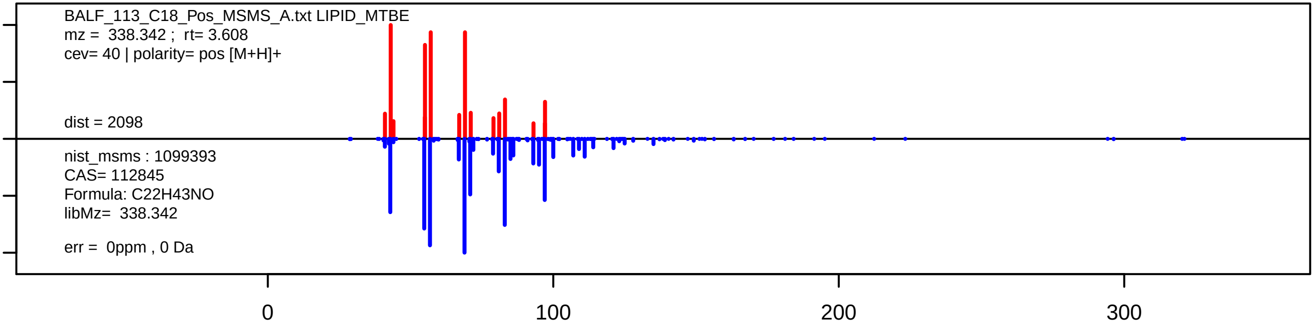

612 . Ethyl tramadol
Score=359 Dot=985 prob=98

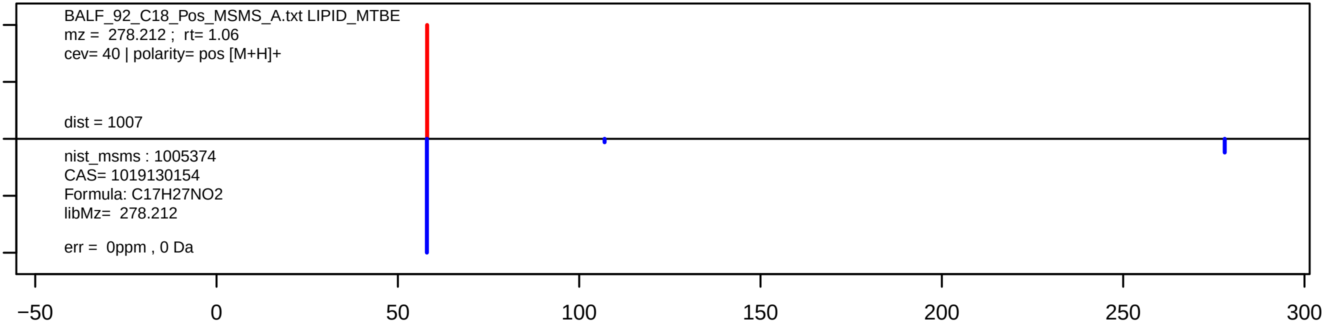

613 . Fluoxetine
Score=400 Dot=999 prob=98

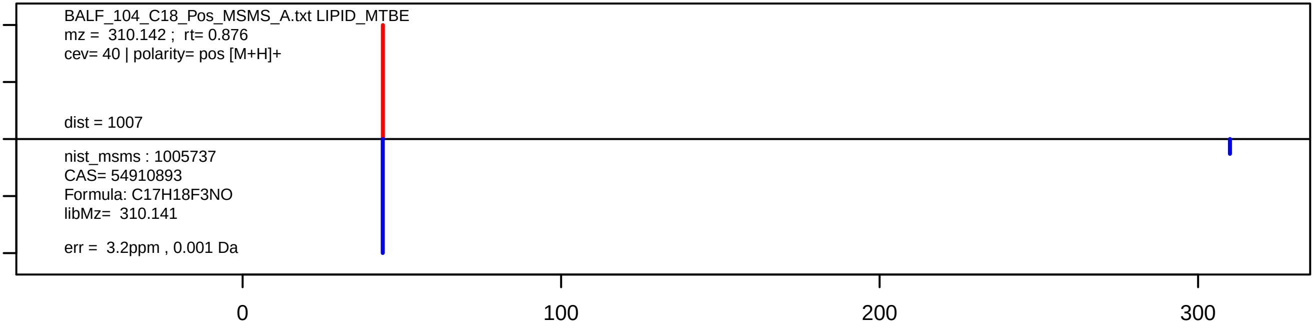

614 . Hemin cation
Score=186 Dot=927 prob=86.8

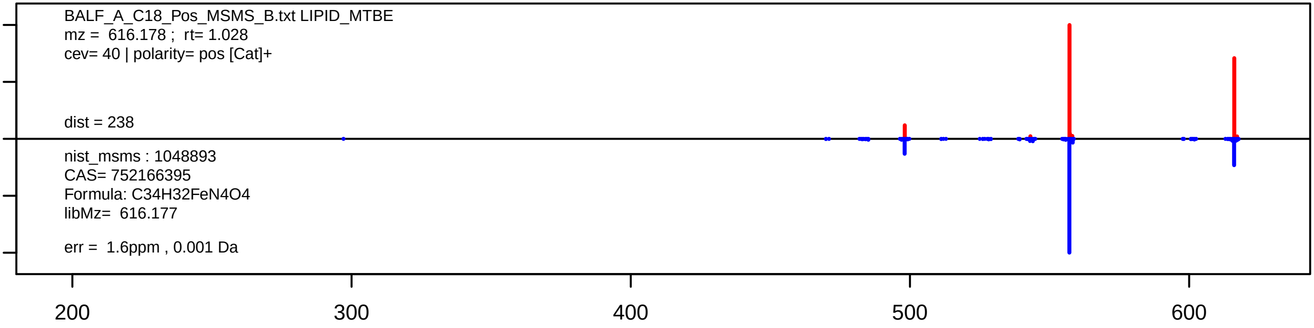

615 . Hexadecyltrimethylammonium cation
Score=214 Dot=837 prob=93

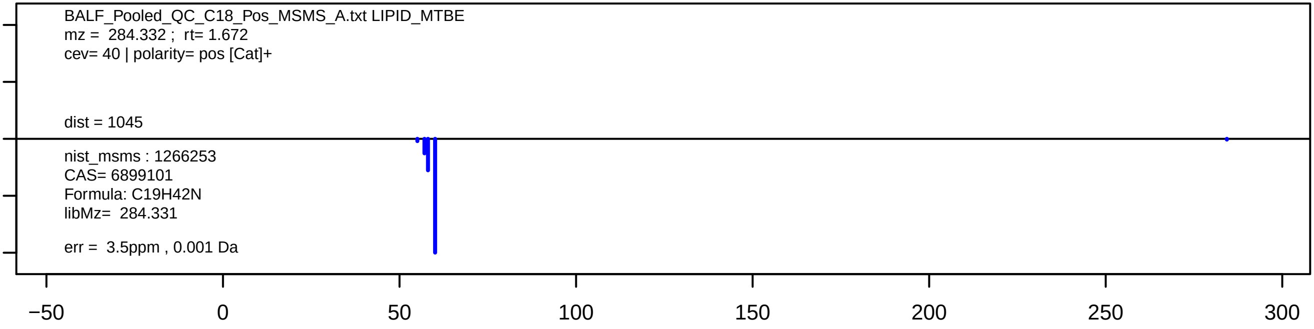

616 . Hydroxybupropion
Score=761 Dot=932 prob=97.6

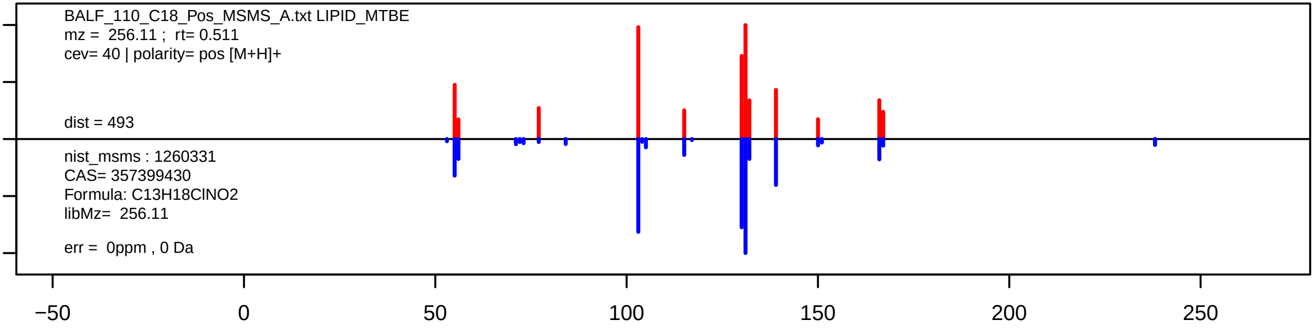

617 . Hydroxyzine
Score=360 Dot=888 prob=98.2

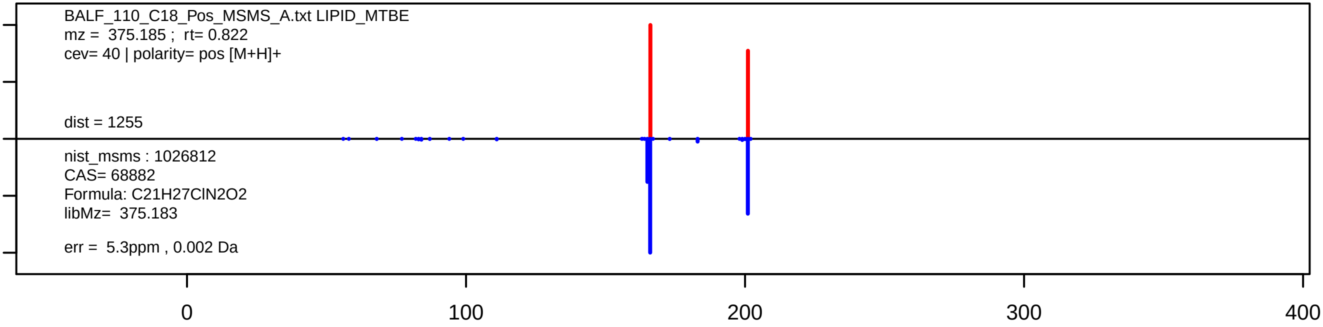

618 . Ipratropium cation
Score=319 Dot=817 prob=81.4

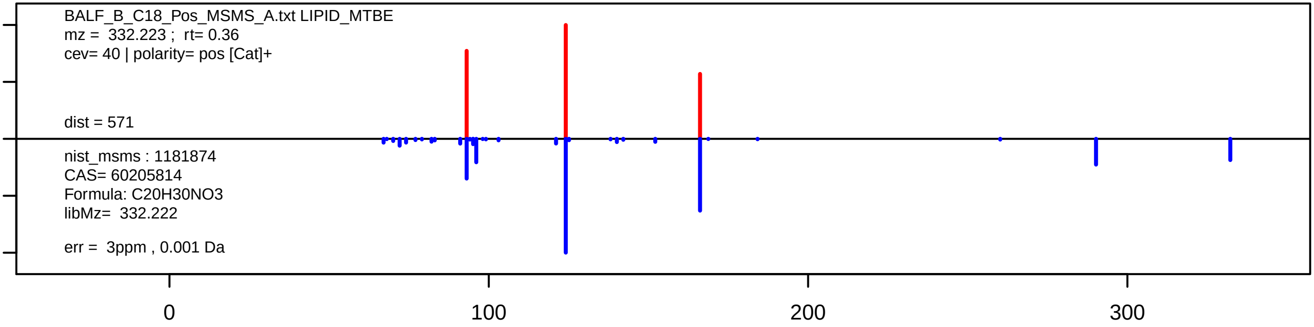

619 .   L−Leucine
Score=632 Dot=892 prob=98.9

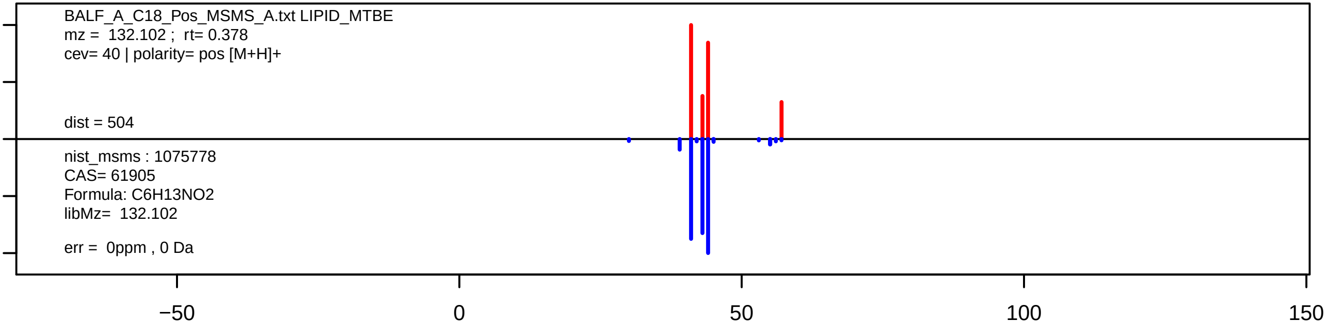

620 .   L−Propionylcarnitine
Score=112 Dot=886 prob=93

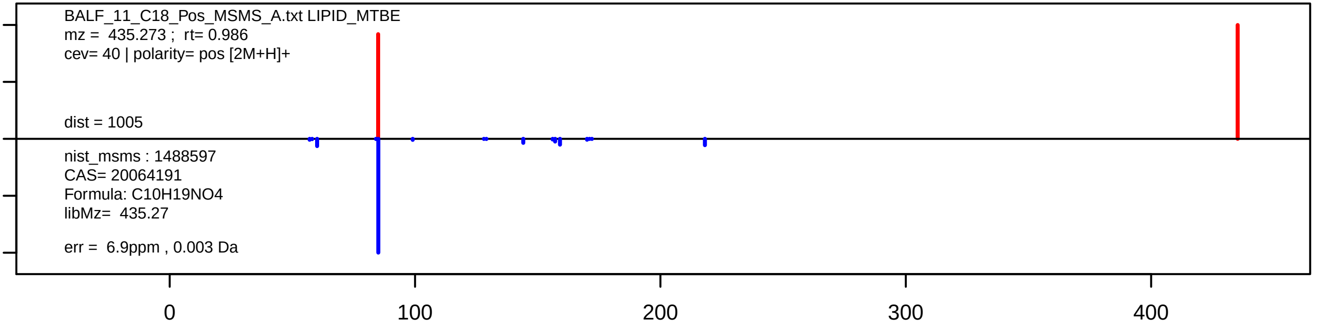

621 .   Lamotrigine
Score=693 Dot=844 prob=95.9

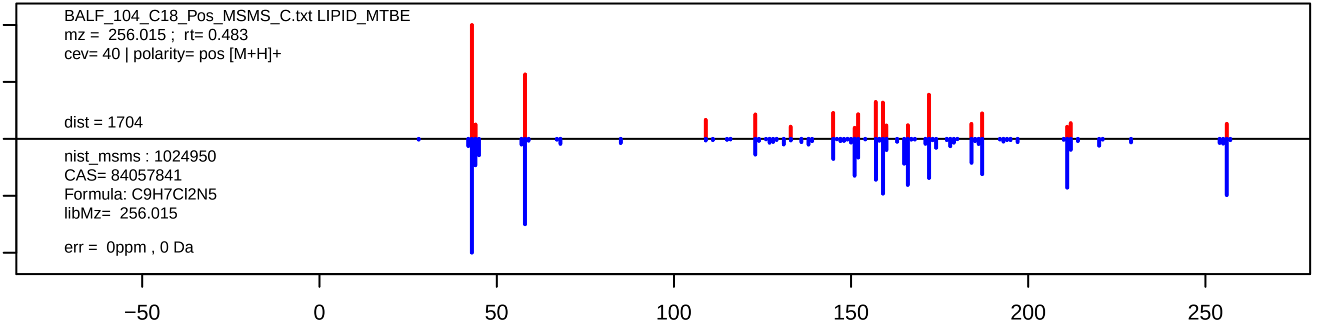

622 . Leu−Leu
Score=383 Dot=882 prob=72.2

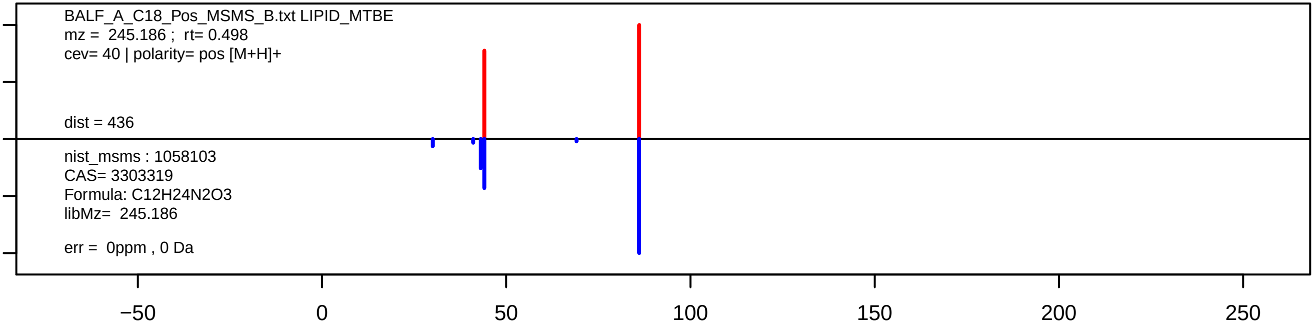

623 . Lidocaine
Score=814 Dot=992 prob=99

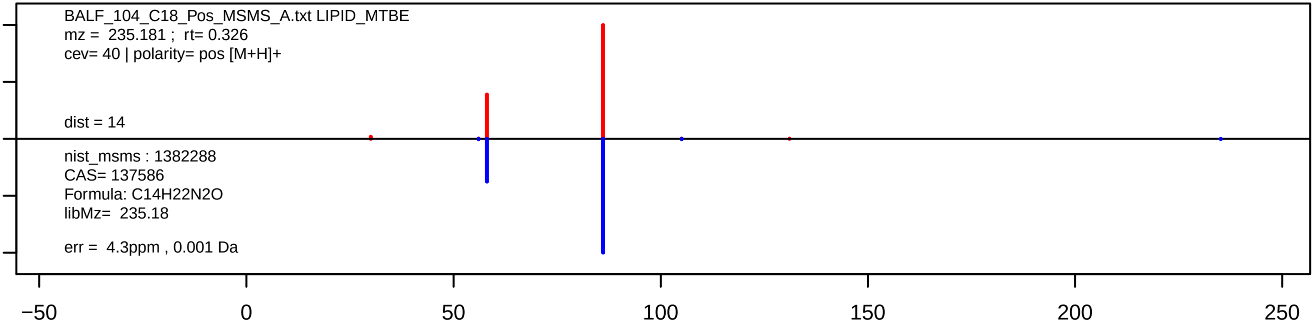

624 . Mefenorex
Score=536 Dot=983 prob=59

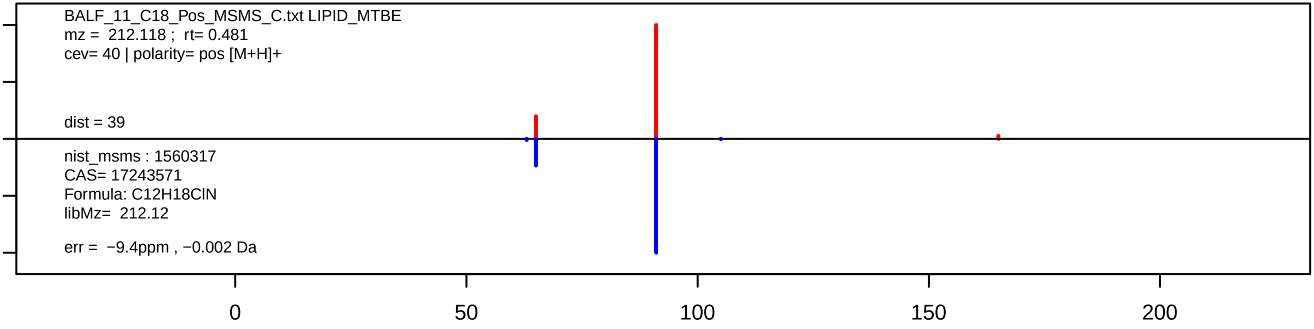

625 .   Metformin
Score=347 Dot=876 prob=98.3

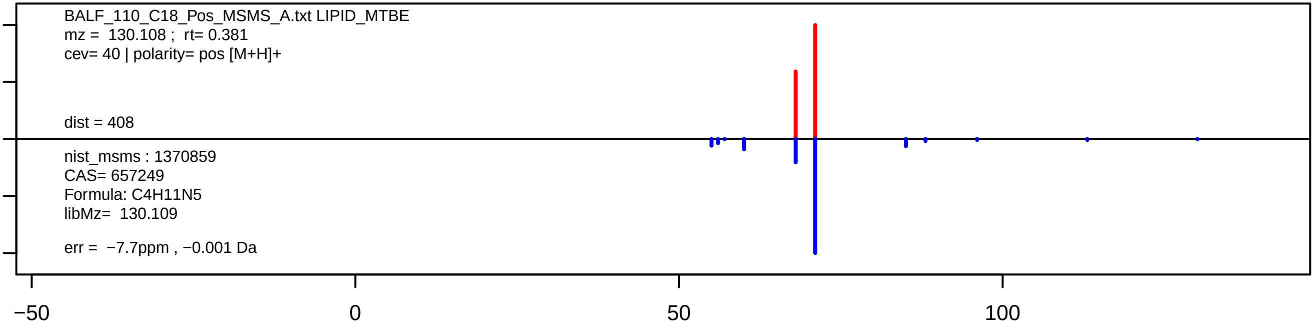

626 .   Methadone
Score=269 Dot=901 prob=94.3

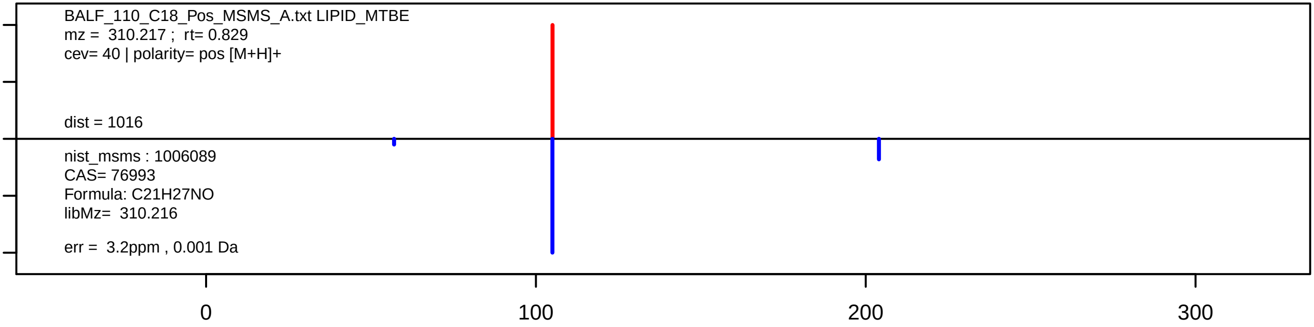

627 .   Mono−2−ethylhexyl phthalate
Score=211 Dot=841 prob=54.2

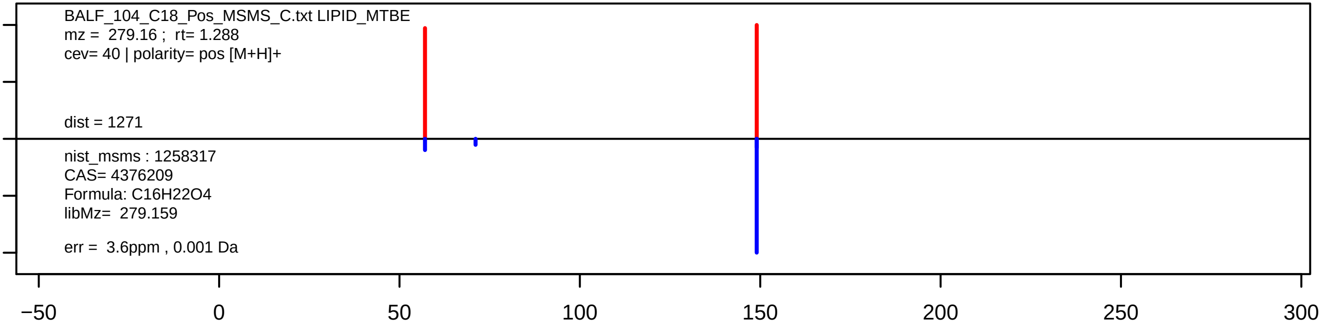

628 . Monoisobutyl phthalate
Score=274 Dot=980 prob=39.1

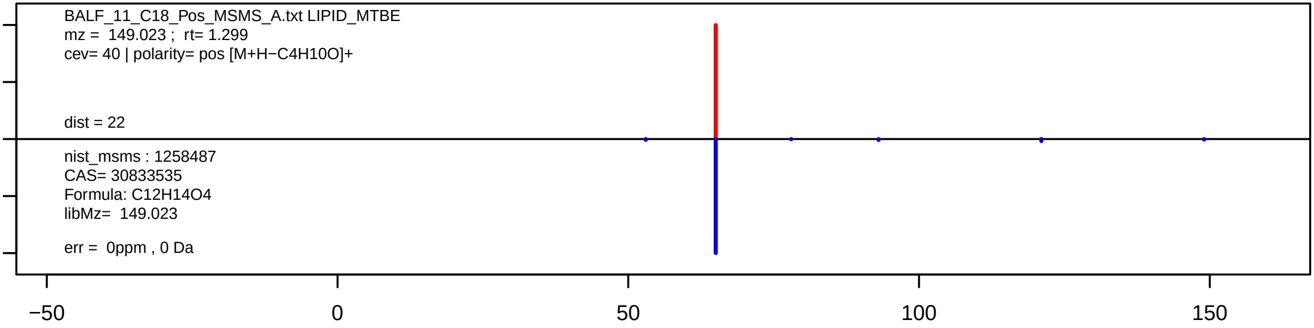

629 . N−(Octadecanoyl)sphing−4−enine−1−phosphocholine
Score=449 Dot=999 prob=97.2

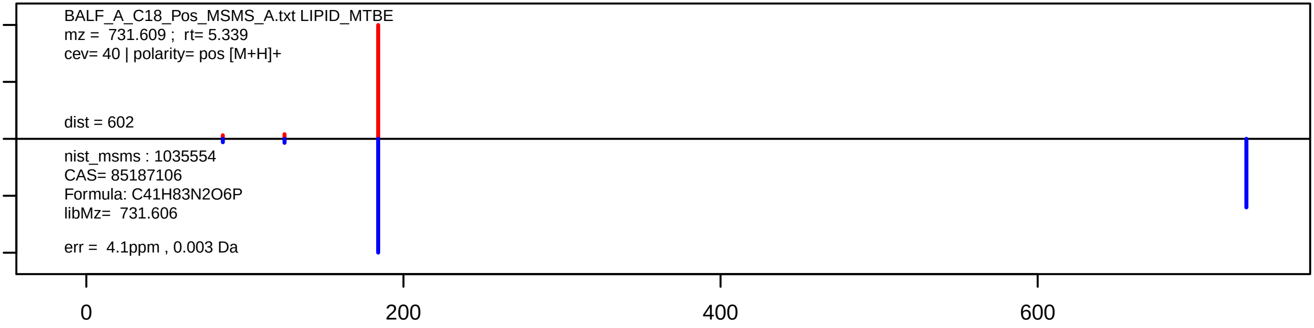

630 . N−Acetyl−D−lactosamine
Score=295 Dot=819 prob=47.7

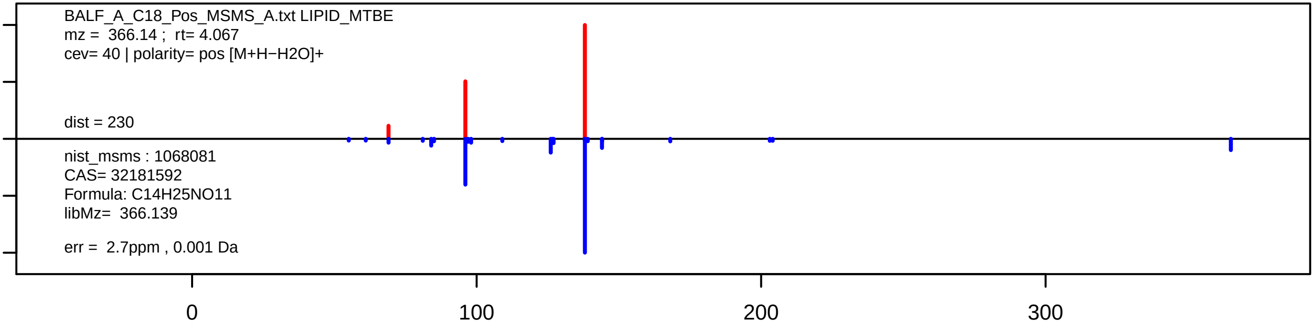

631 . N−Desmethyltramadol
Score=302 Dot=957 prob=98.5

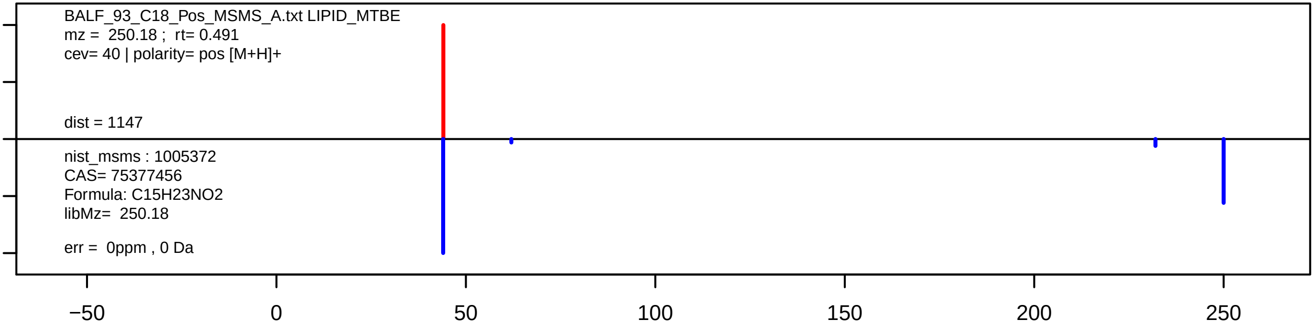

632 . N−Docosanoyl−4−sphingenyl−1−O−phosphorylcholine
Score=355 Dot=982 prob=91.7

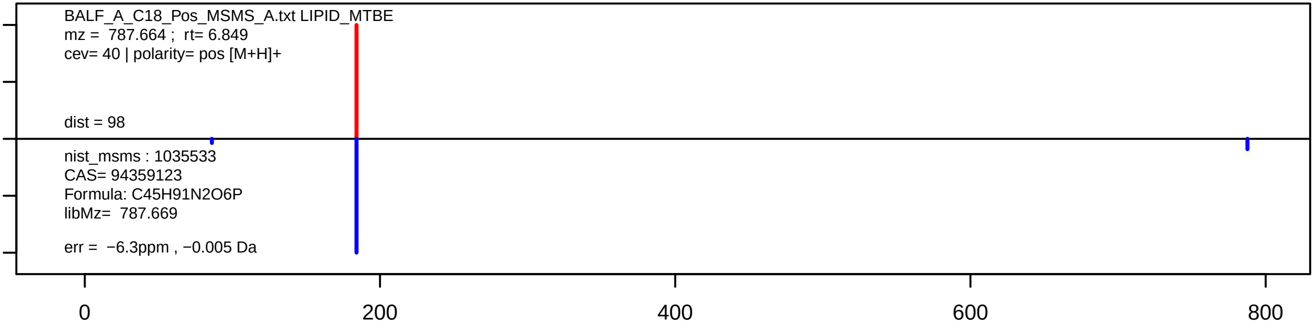

633 . N−Nervonoyl−D−erythro−sphingosylphosphorylcholine
Score=400 Dot=999 prob=100

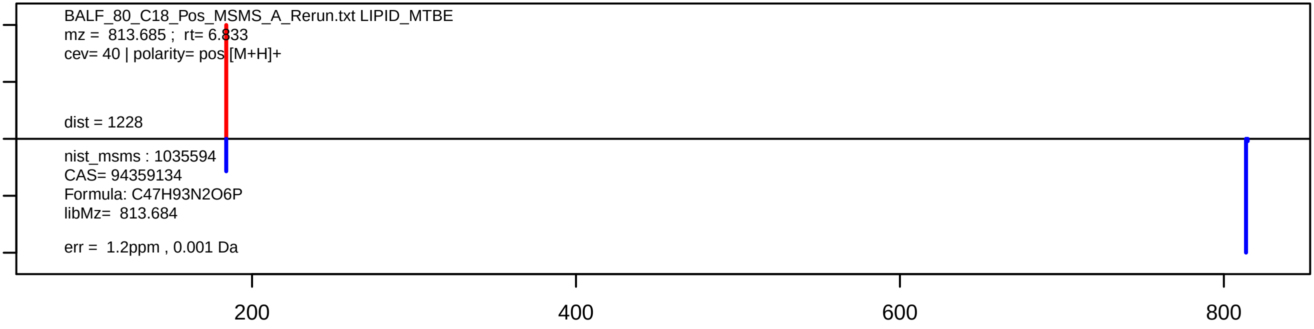

634 . n−Octadecylamine
Score=303 Dot=953 prob=96.7

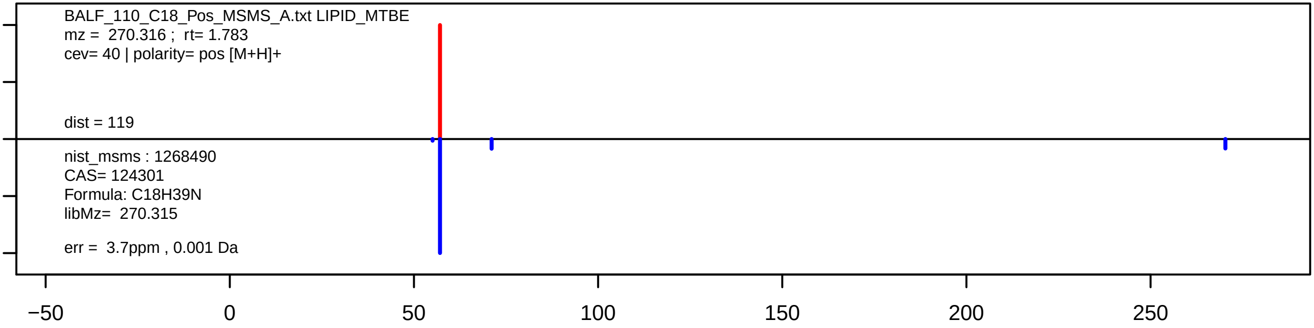

635 . N−Oleoyl−D−erythro−sphingosylphosphorylcholine
Score=404 Dot=971 prob=95.5

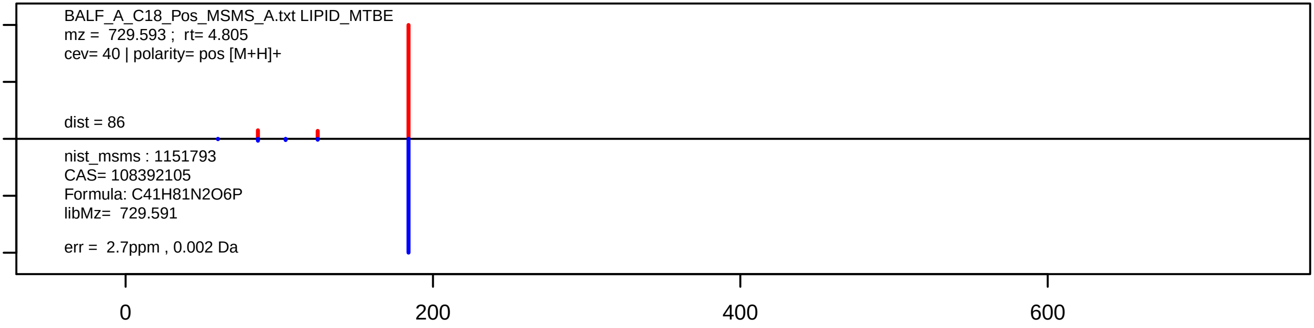

636 . N−Palmitoyl−D−sphingosine
Score=325 Dot=897 prob=99

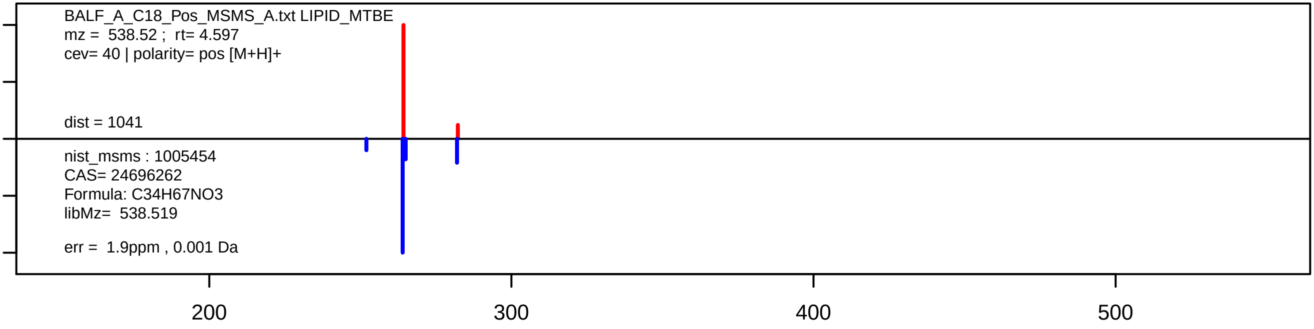

637 .   N−Tetracosanoyl−4−sphingenyl−1−O−phosphorylcholine
Score=194 Dot=906 prob=98

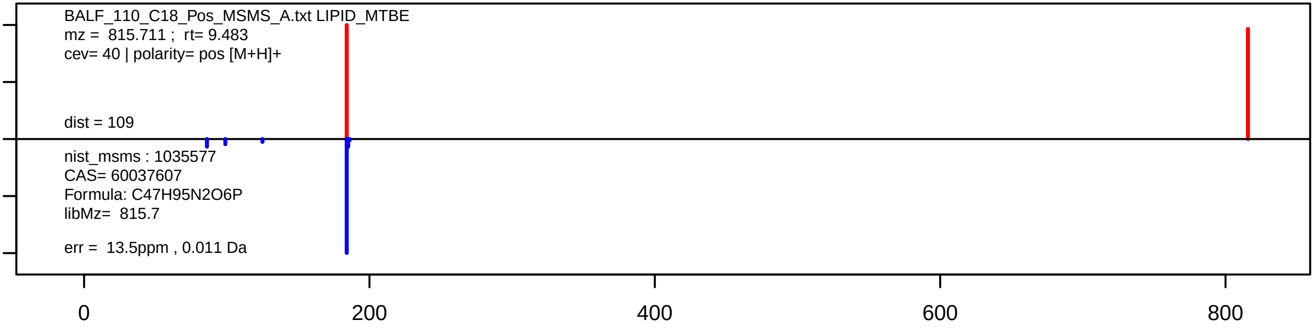

638 .   N−Tetracosenoyl−4−sphingenine
Score=455 Dot=901 prob=100

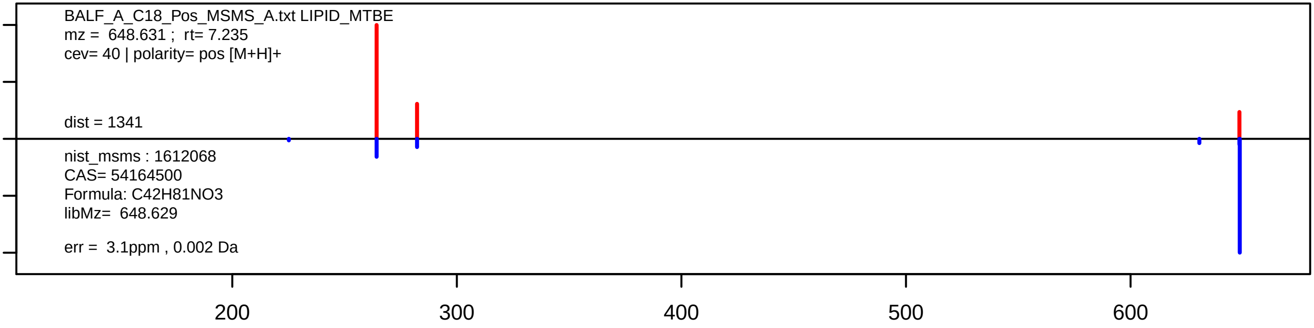

639 .   N,N−Dimethylbenzylamine
Score=577 Dot=945 prob=60.5

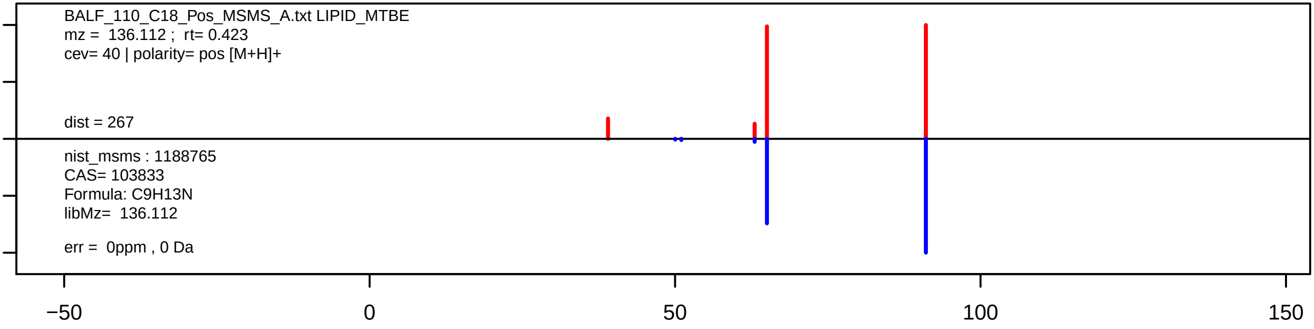

640 . Norquetiapine
Score=516 Dot=871 prob=96.5

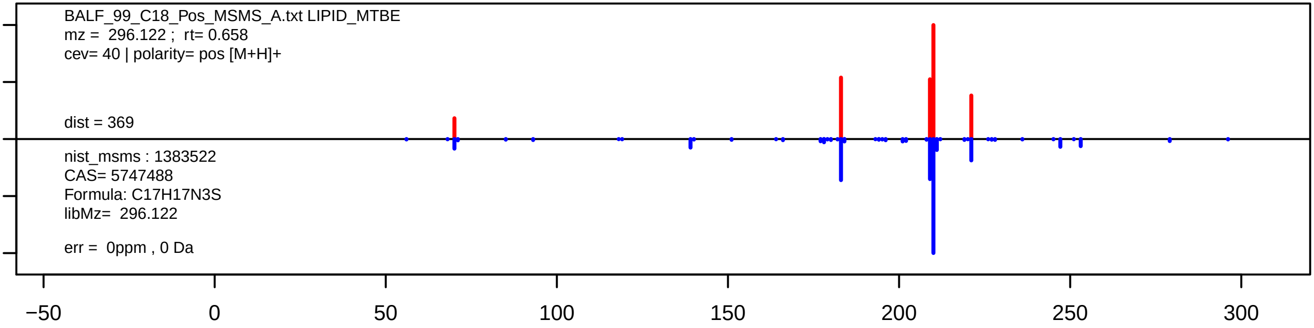

641 . Oleamide
Score=341 Dot=819 prob=89.7

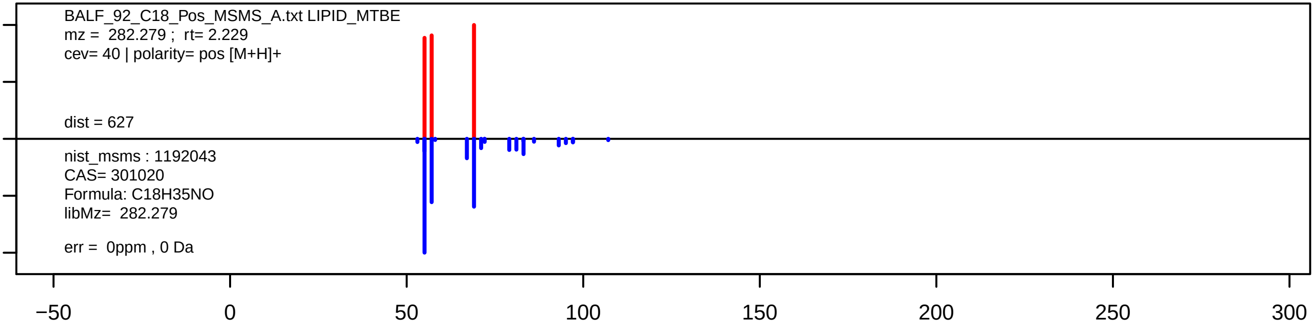

642 . Palmitoyl sphingomyelin
Score=381 Dot=973 prob=100

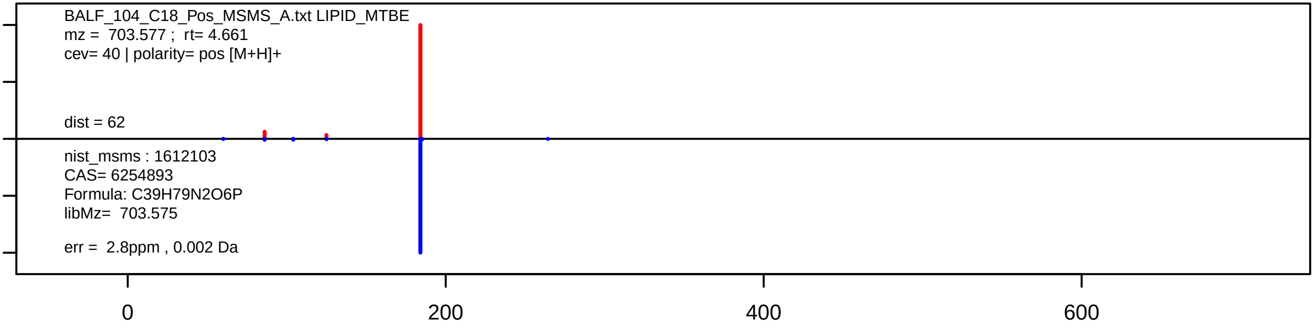

643 . Palmitoyl−L−carnitine
Score=453 Dot=893 prob=97.3

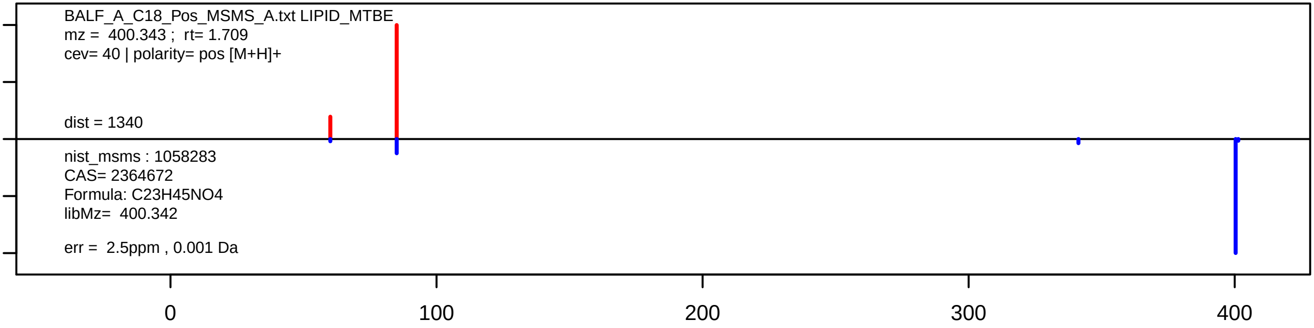

644 . Palmitoyleicosapentaenoyl phosphatidylcholine
Score=181 Dot=953 prob=100

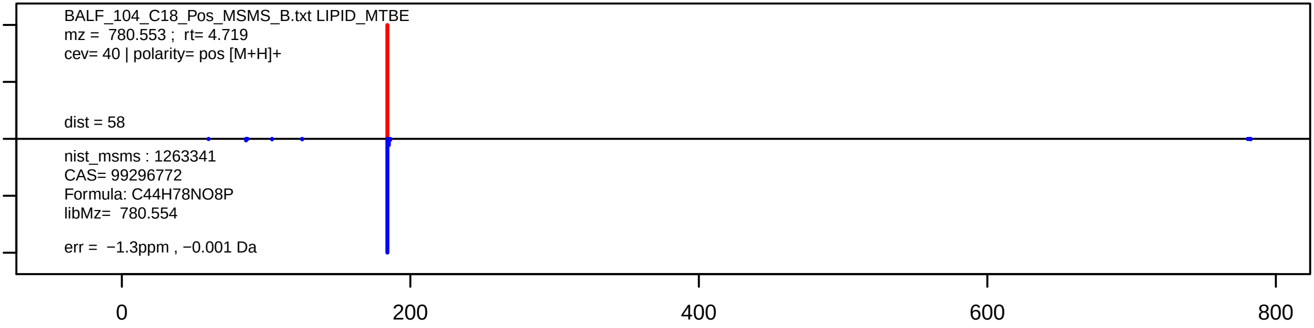

645 . PC 35:2; [M+Na]+; GPCho(9:0/26:2(5E,9Z))
Score=146 Dot=810 prob=1.9

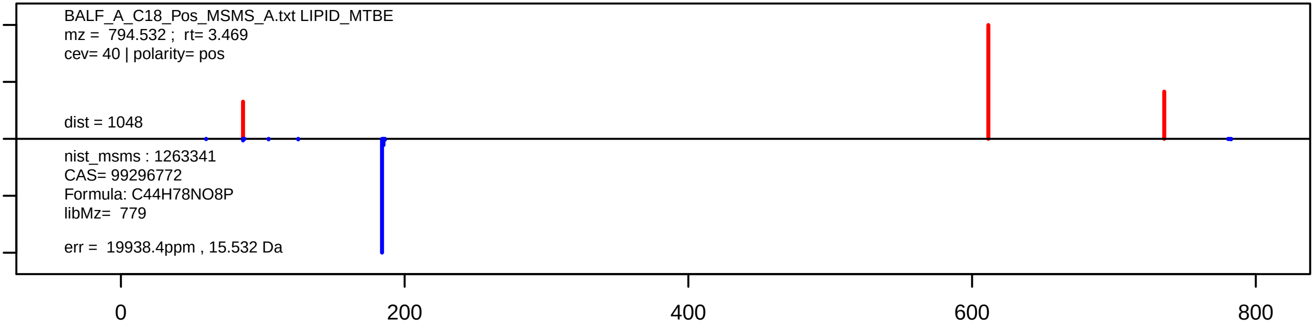

646 . PC 37:7; [M+Na]+; GPCho(15:1(9Z)/22:6(4Z,7Z,10Z,13Z,16Z,19Z))
Score=118 Dot=801 prob=24

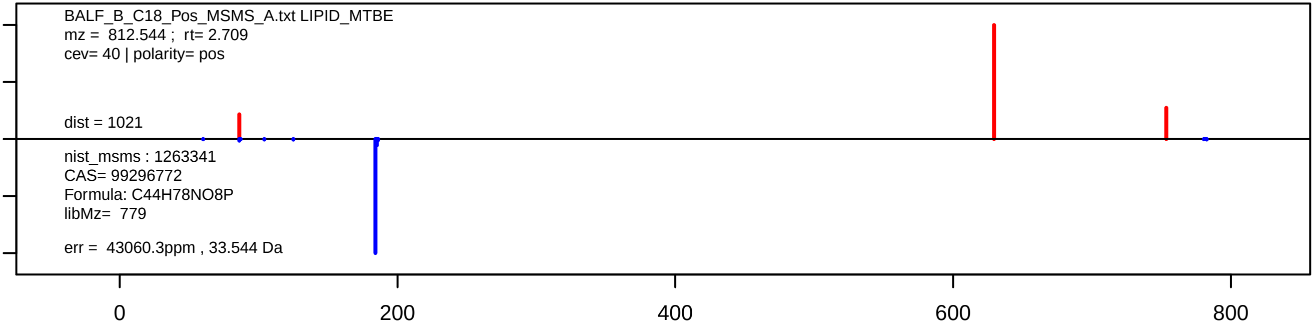

647 . PC 39:3; [M+Na]+; GPCho(17:1(9Z)/22:2(13Z,16Z))
Score=292 Dot=809 prob=8

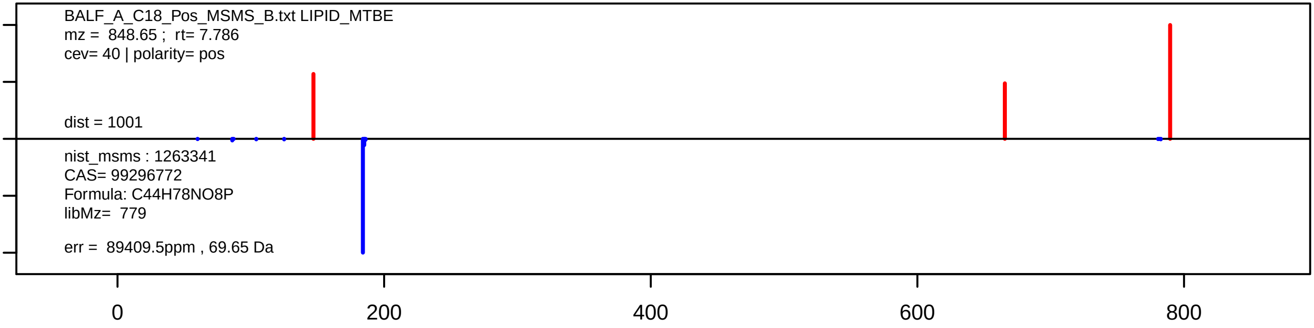

648 . PC 41:4; [M+Na]+; GPCho(17:0/24:4(5Z,8Z,11Z,14Z))
Score=477 Dot=952 prob=7.1

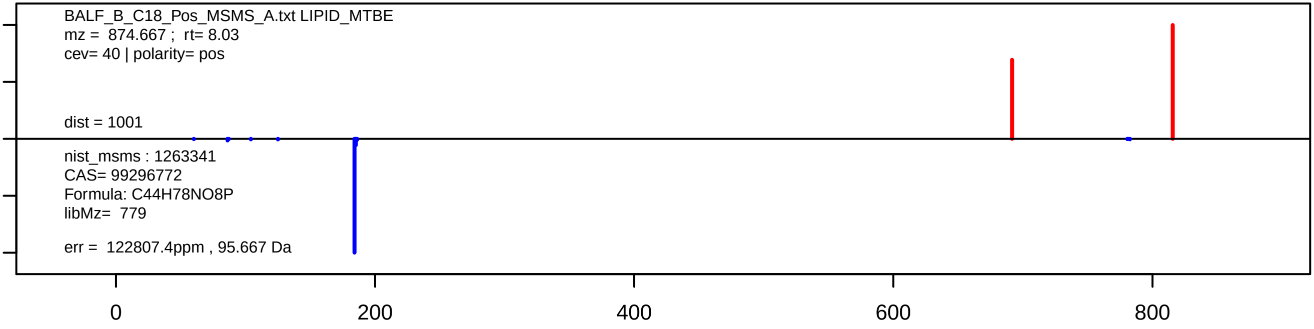

649 .   PC 41:5; [M+Na]+; GPCho(17:1(9Z)/24:4(5Z,8Z,11Z,14Z))
Score=293 Dot=808 prob=11.7

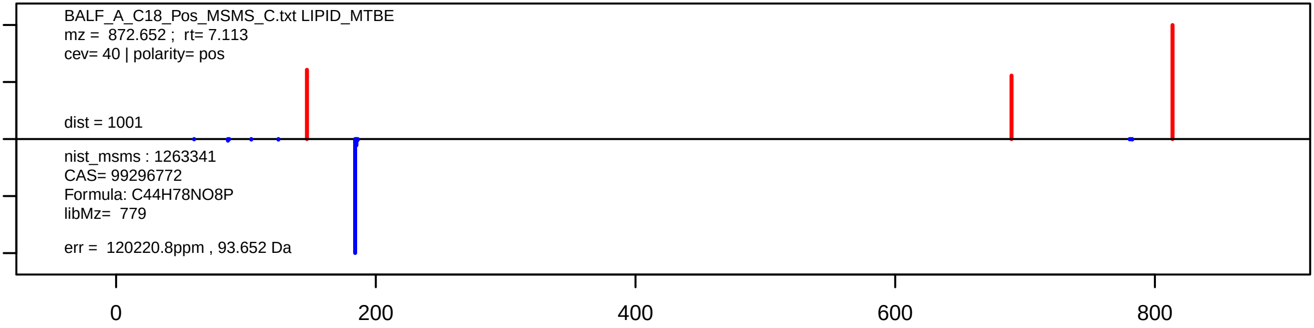

650 .   PC 43:5; [M+Na]+; GPCho(20:5(5Z,8Z,11Z,14Z,17Z)/23:0)
Score=283 Dot=812 prob=15.3

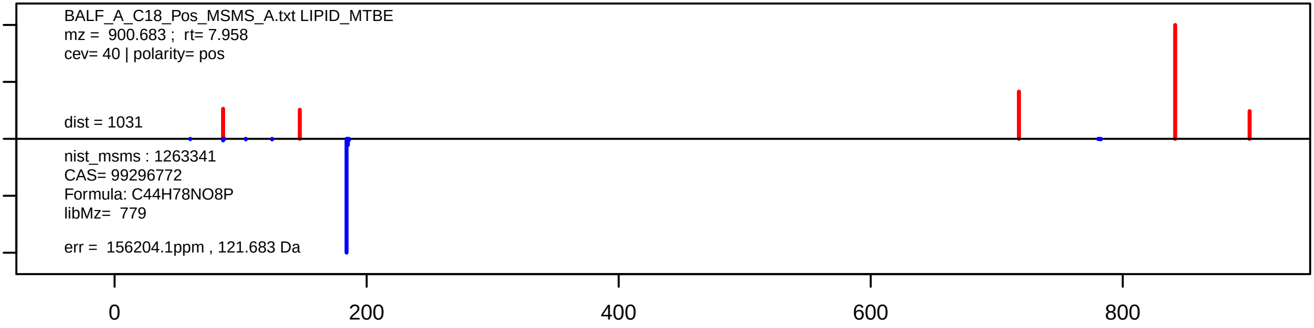

651 .   PE 34:0; [M+H]+; GPEtn(17:0/17:0)
Score=372 Dot=998 prob=11.6

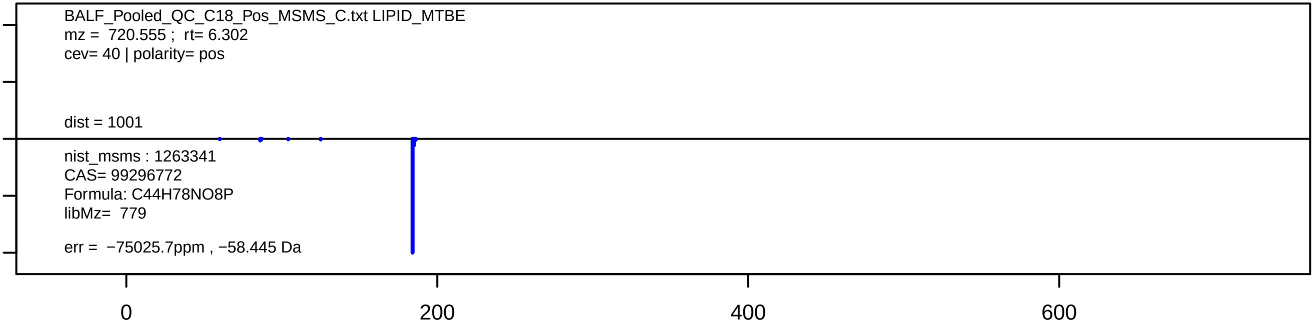

652 . PE 34:2; [M+H]+; GPEtn(17:1(9Z)/17:1(9Z))
Score=372 Dot=998 prob=3

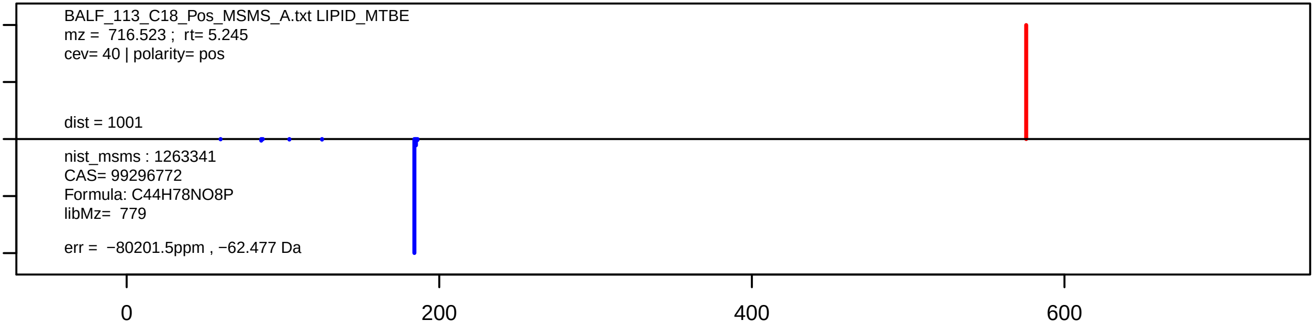

653 . plasmenyl−PC 34:0; [M+Na]+; PC(P−16:0/18:0)
Score=234 Dot=836 prob=25.7

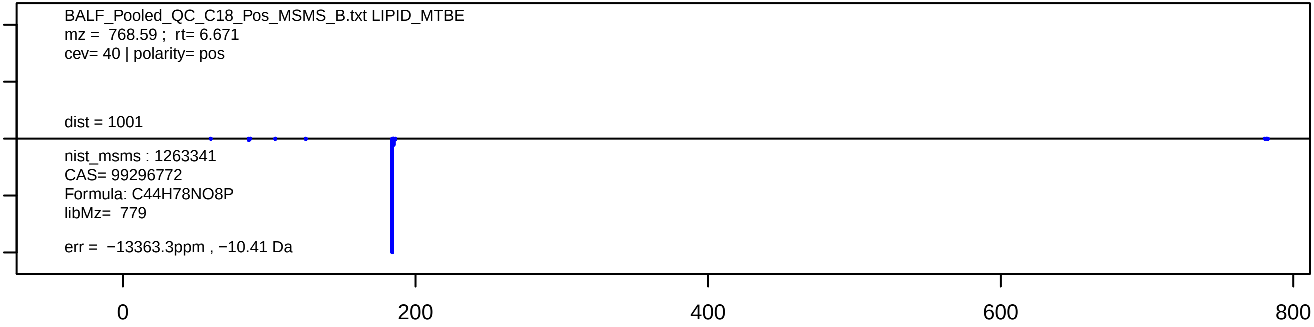

654 . plasmenyl−PC 40:1; [M+Na]+; PC(P−16:0/24:1(15Z))
Score=276 Dot=957 prob=14.5

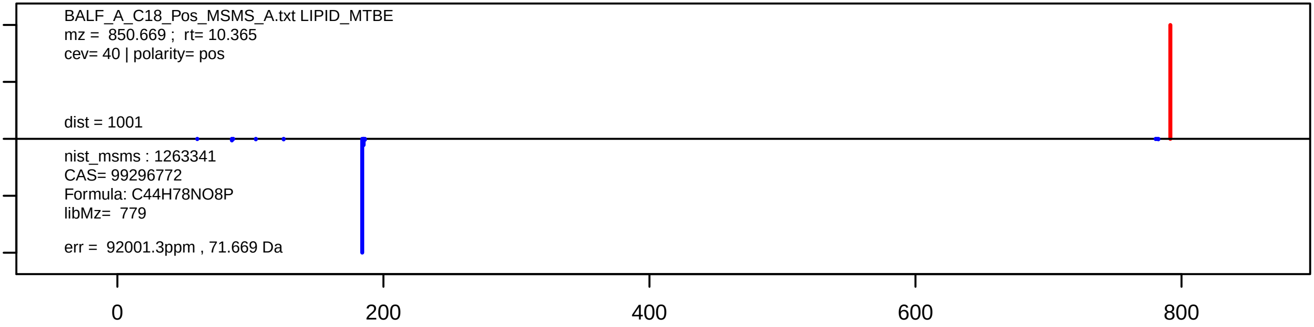

655 . plasmenyl−PC 42:2; [M+Na]+; PC(P−16:0/26:2(5E,9Z))
Score=329 Dot=811 prob=19.8

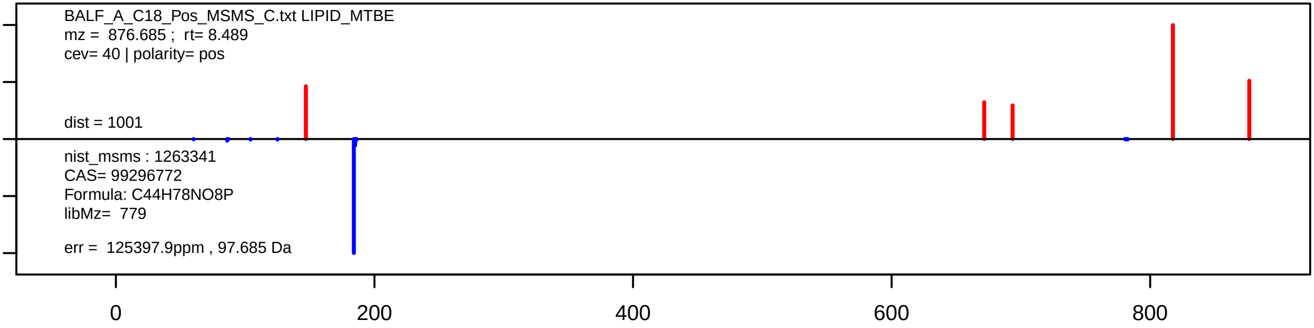

656 . plasmenyl−PC 44:4; [M+Na]+; PC(P−20:0/24:4(5Z,8Z,11Z,14Z))
Score=339 Dot=829 prob=64.8

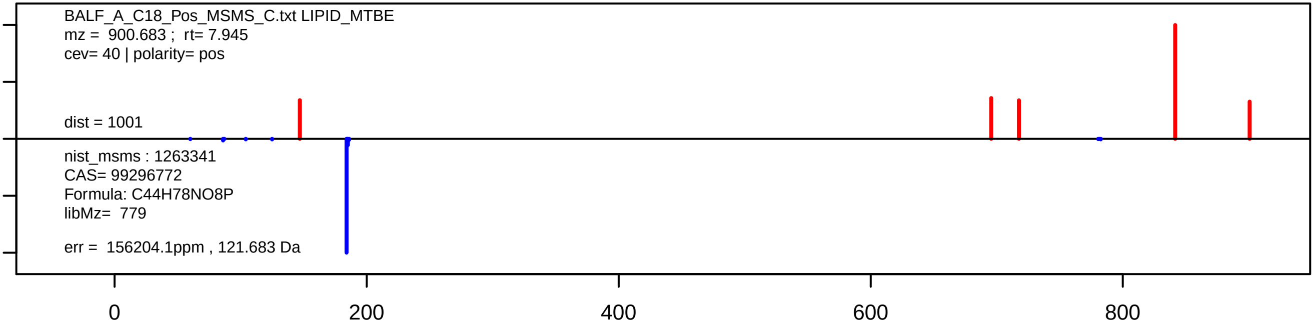

657 . rac erythro−Dihydrobupropion
Score=649 Dot=871 prob=62

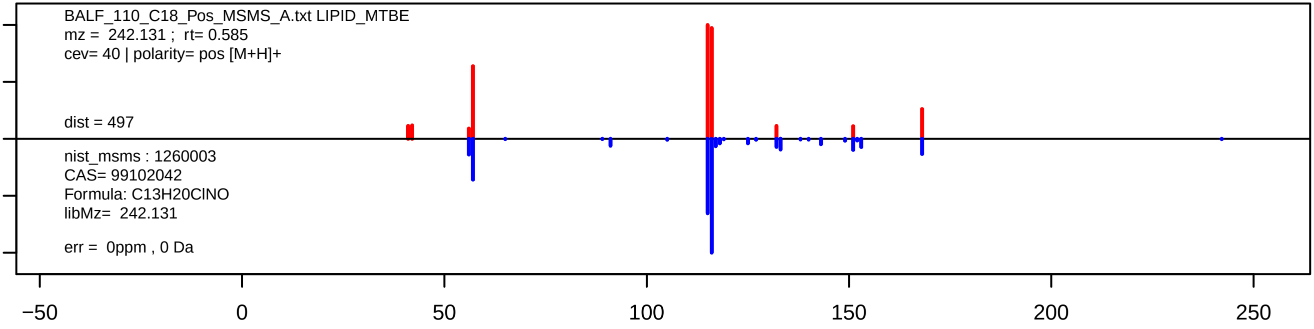

658 . rac−Cetirizine N−oxide
Score=629 Dot=967 prob=97.3

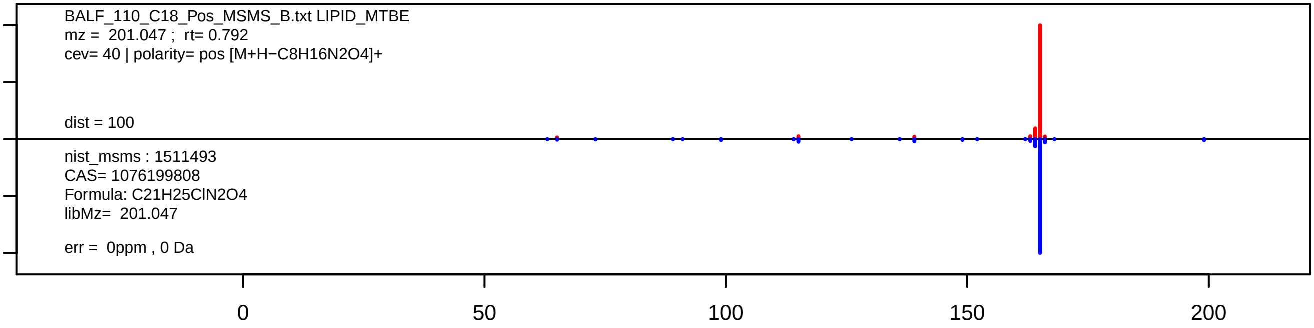

659 . Risperidone
Score=400 Dot=999 prob=96.4

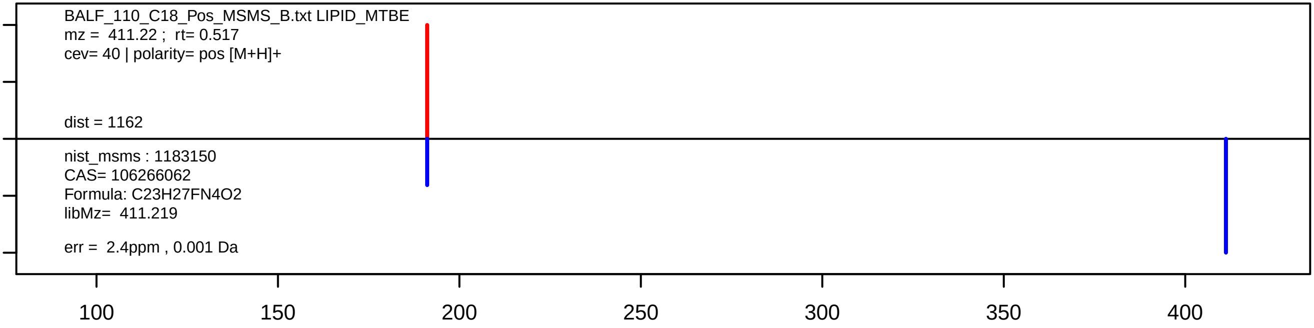

660 . SM 45:1; [M+Na]+; SM(d19:0/26:1(17Z))
Score=244 Dot=802 prob=50

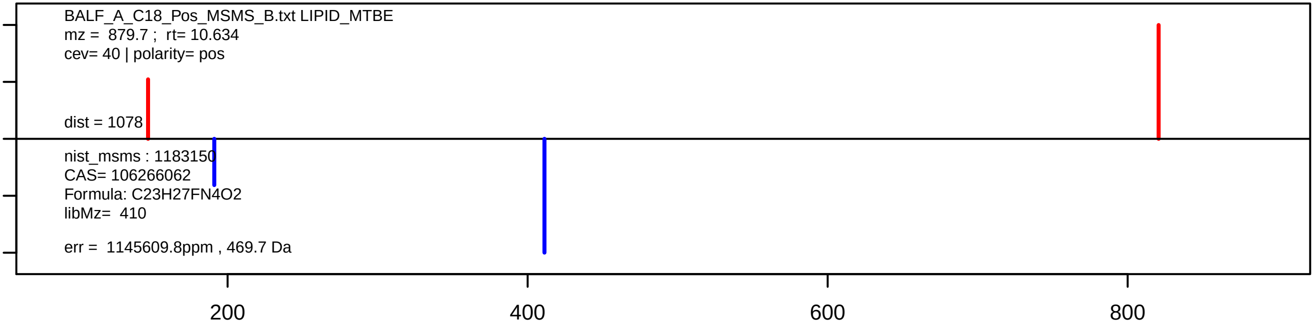

661 .   Stearoyl−L−carnitine
Score=246 Dot=875 prob=98.6

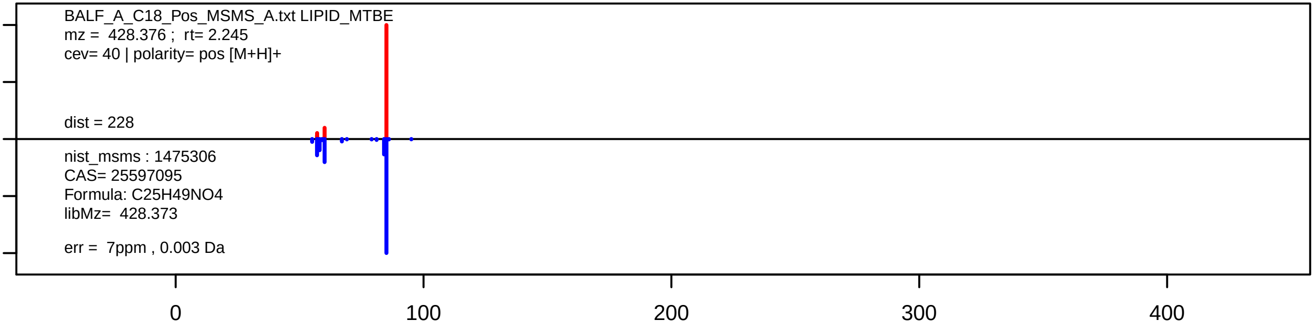

662 .   Testosterone
Score=402 Dot=805 prob=66.3

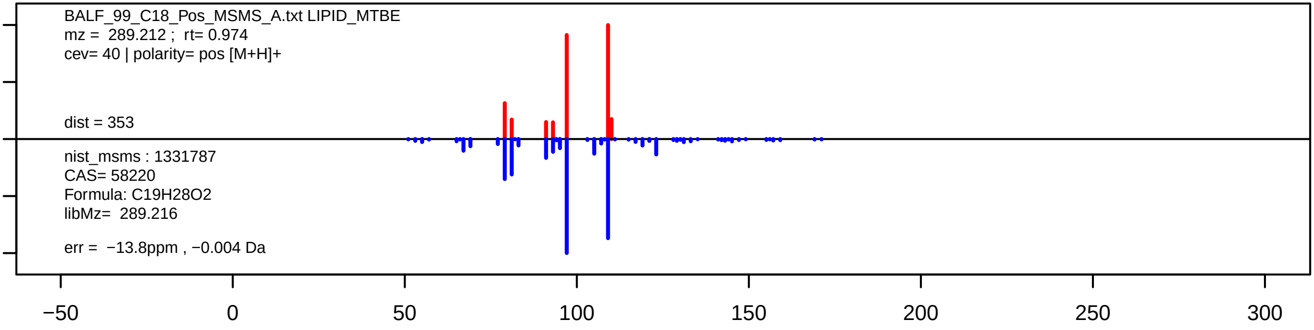

663 .   Tetra−n−dodecylammonium cation
Score=302 Dot=805 prob=95

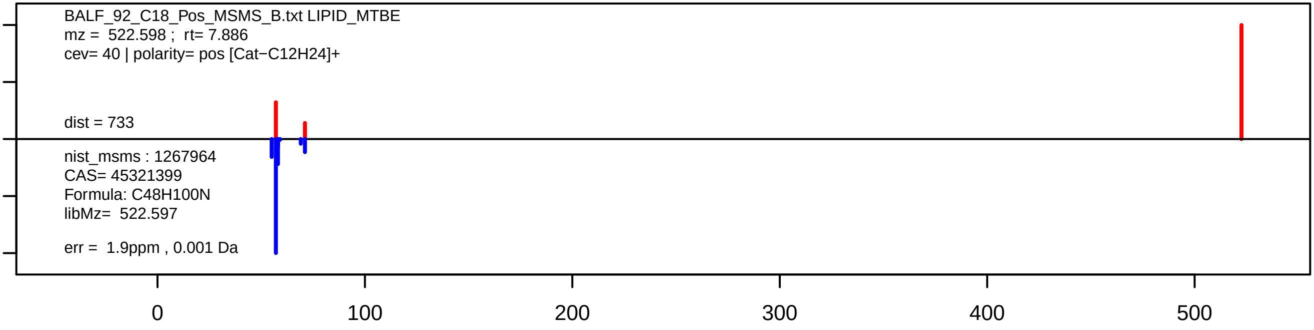

664 . Tetraheptylammonium cation
Score=156 Dot=888 prob=73.7

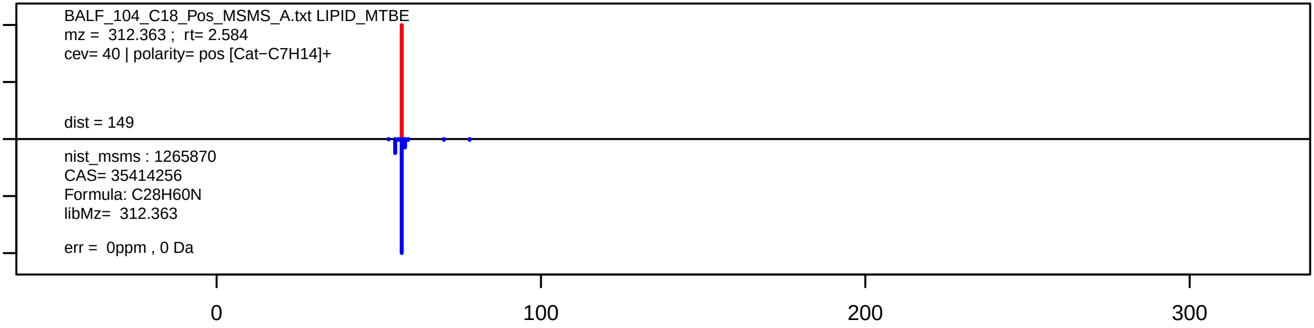

665 . TG 52:3; [M+Na]+; TG(16:0/18:1/18:2)
Score=386 Dot=815 prob=96.7

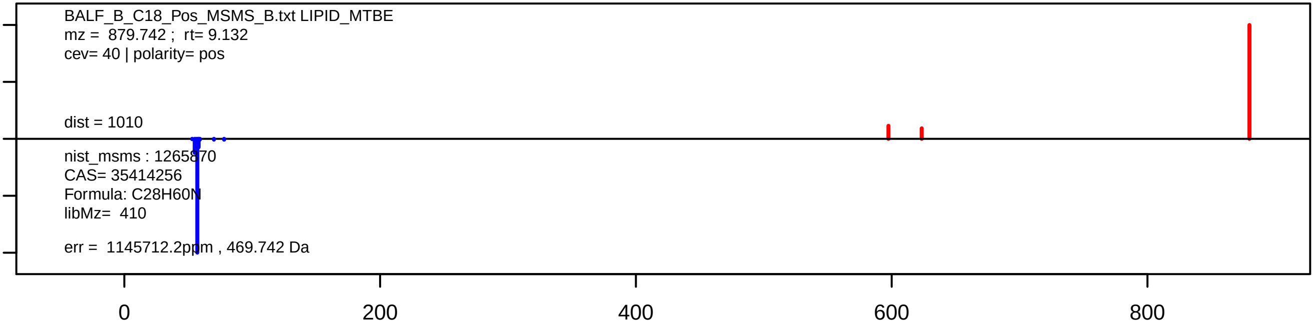

666 . TG 52:3; [M+NH4]+; TG(16:0/18:1/18:2)
Score=673 Dot=903 prob=96.1

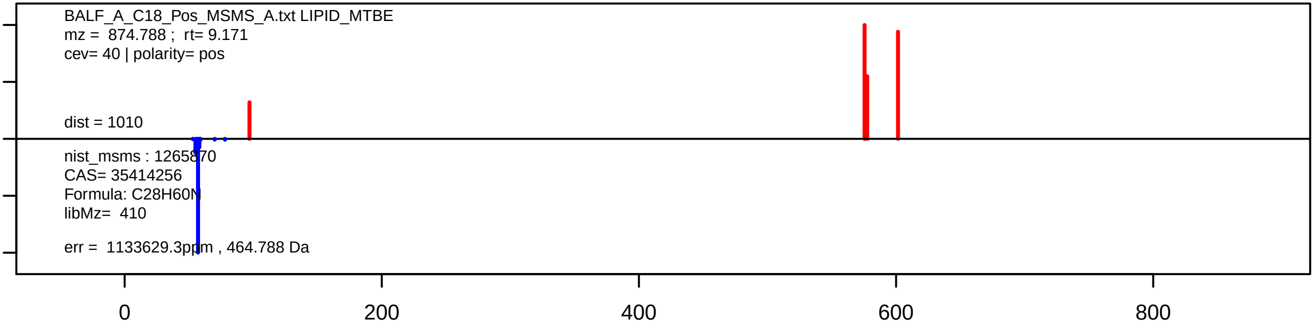

667 . TG 53:1; [M+Na]+; TG(16:0/18:1/19:0)
Score=373 Dot=813 prob=96.7

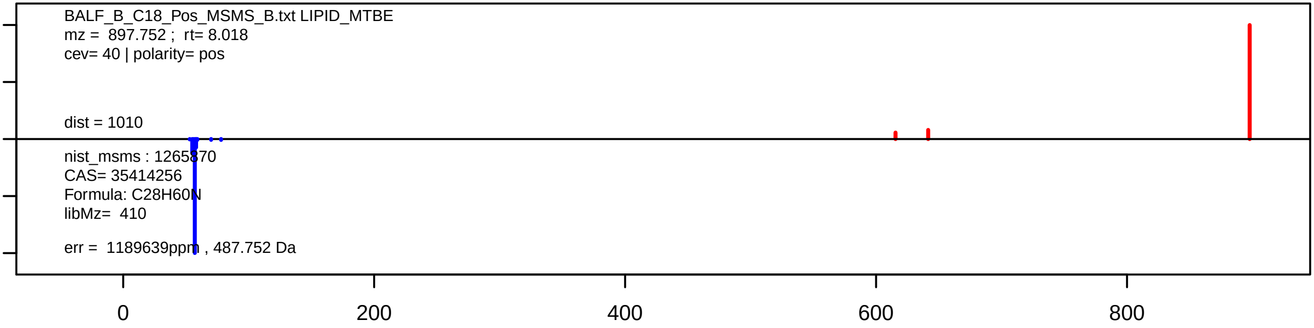

668 . TG 54:4; [M+Na]+; TG(18:1/18:1/18:2)
Score=421 Dot=845 prob=85.8

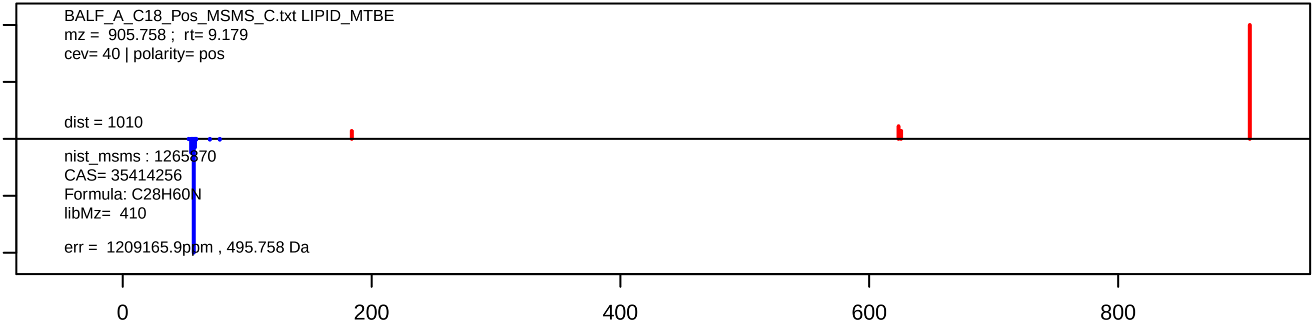

669 . Thioetheramidephosphatidylcholine
Score=305 Dot=881 prob=99

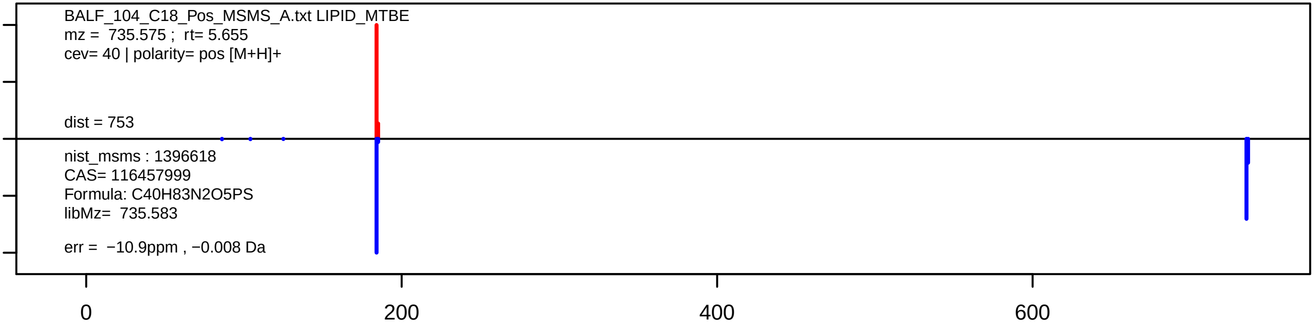

670 . threo−Dihydrobupropion
Score=873 Dot=936 prob=49.7

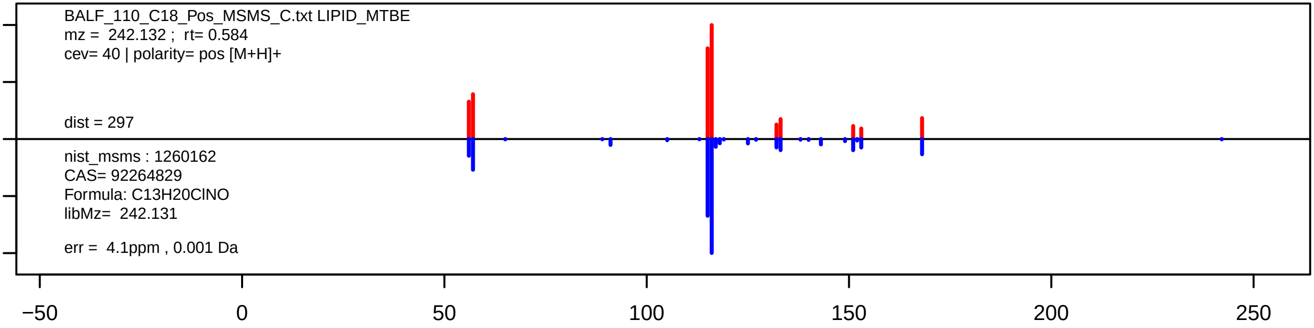

671 . Tramadol
Score=388 Dot=999 prob=98.6

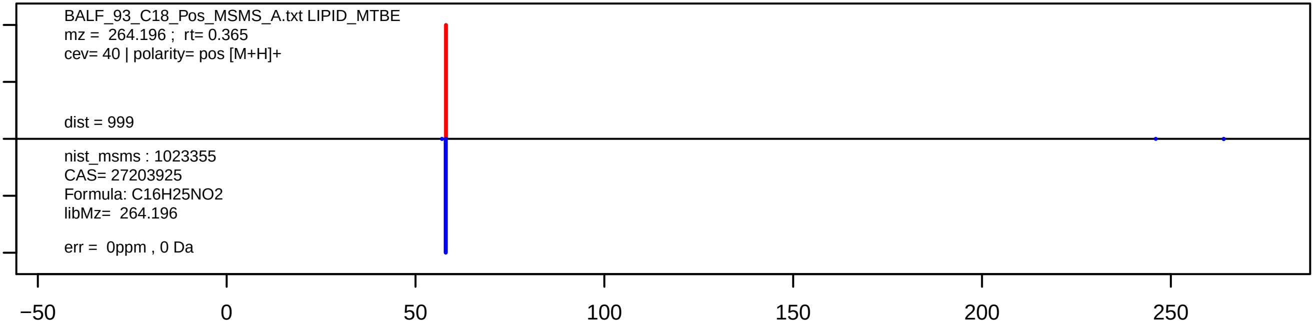

672 . Tributyl phosphate
Score=324 Dot=912 prob=77.3

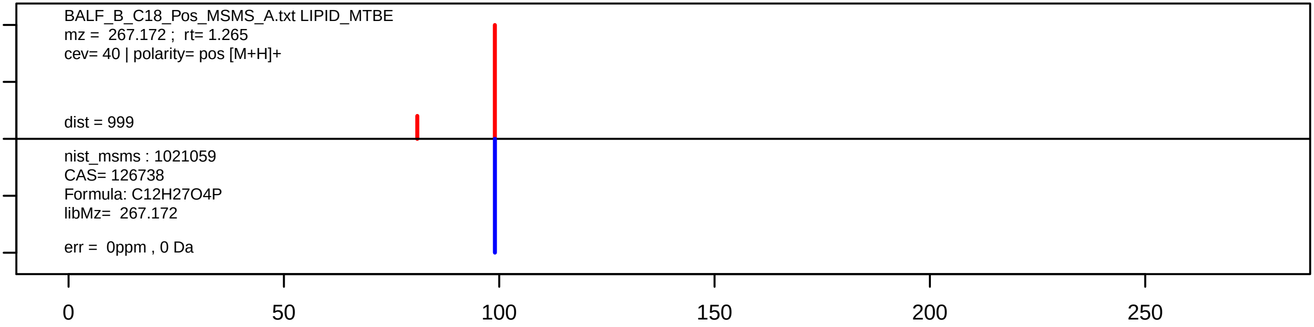

673 . Tridodecylmethylammonium cation
Score=222 Dot=816 prob=93.7

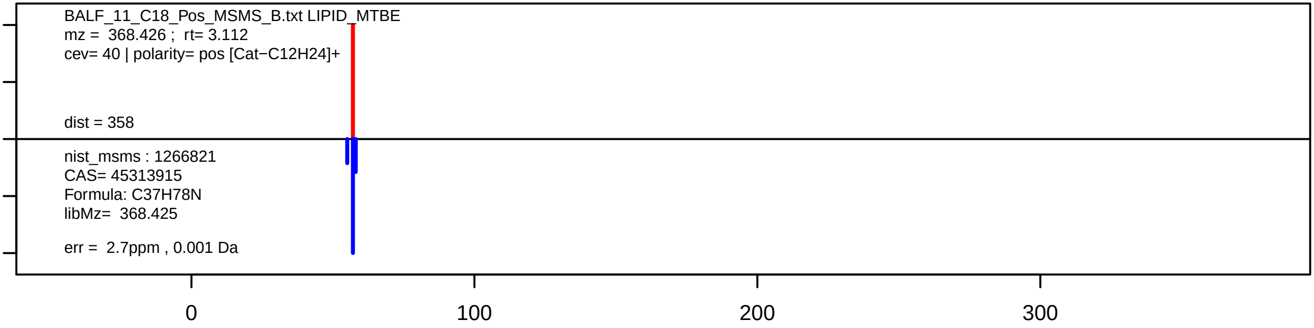

674 . Venlafaxine
Score=403 Dot=924 prob=94.2

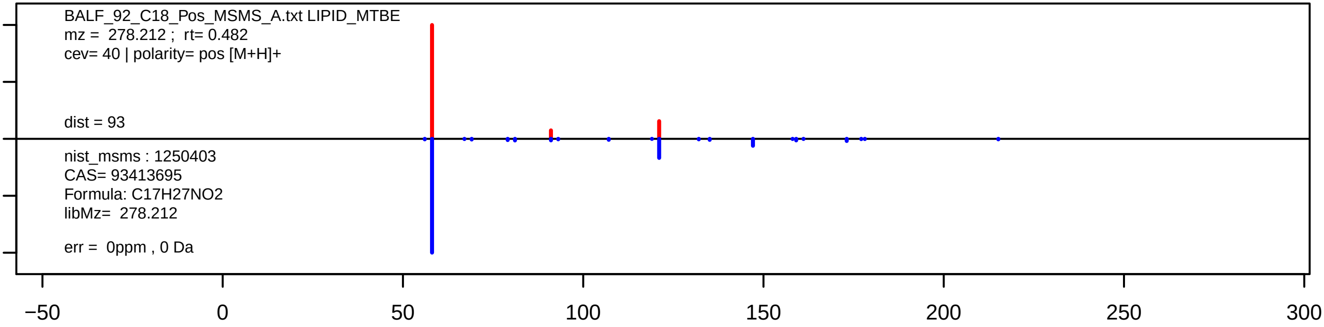

Supplement: Supplementary File 2 [file sdata201860-s3.pdf]
